# Supplementary material for: A Wolf in Another Wolf’s Clothing: Post-Genomic Regulation Dictates Venom Profiles of Medically-Important Cryptic Kraits in India
Source: Toxins (Basel). 2021 Jan 19;13(1):69. doi: 10.3390/toxins13010069 (PMC7832344; doi:10.3390/toxins13010069)
Supplement: Supplementary file 1 [file toxins-13-00069-s001.zip › toxins-1058980 supple for final.docx]

Supplementary Materials: A wolf in another wolf’s clothing: Post-genomic regulation dictates venom profiles of medically-important cryptic kraits in India

Kartik Sunagar, Suyog Khochare, R. R. Senji Laxme, Saurabh Attarde, Paulomi Dam, Vivek Suranse, Anil Khaire, Gerard Martin and Ashok Captain


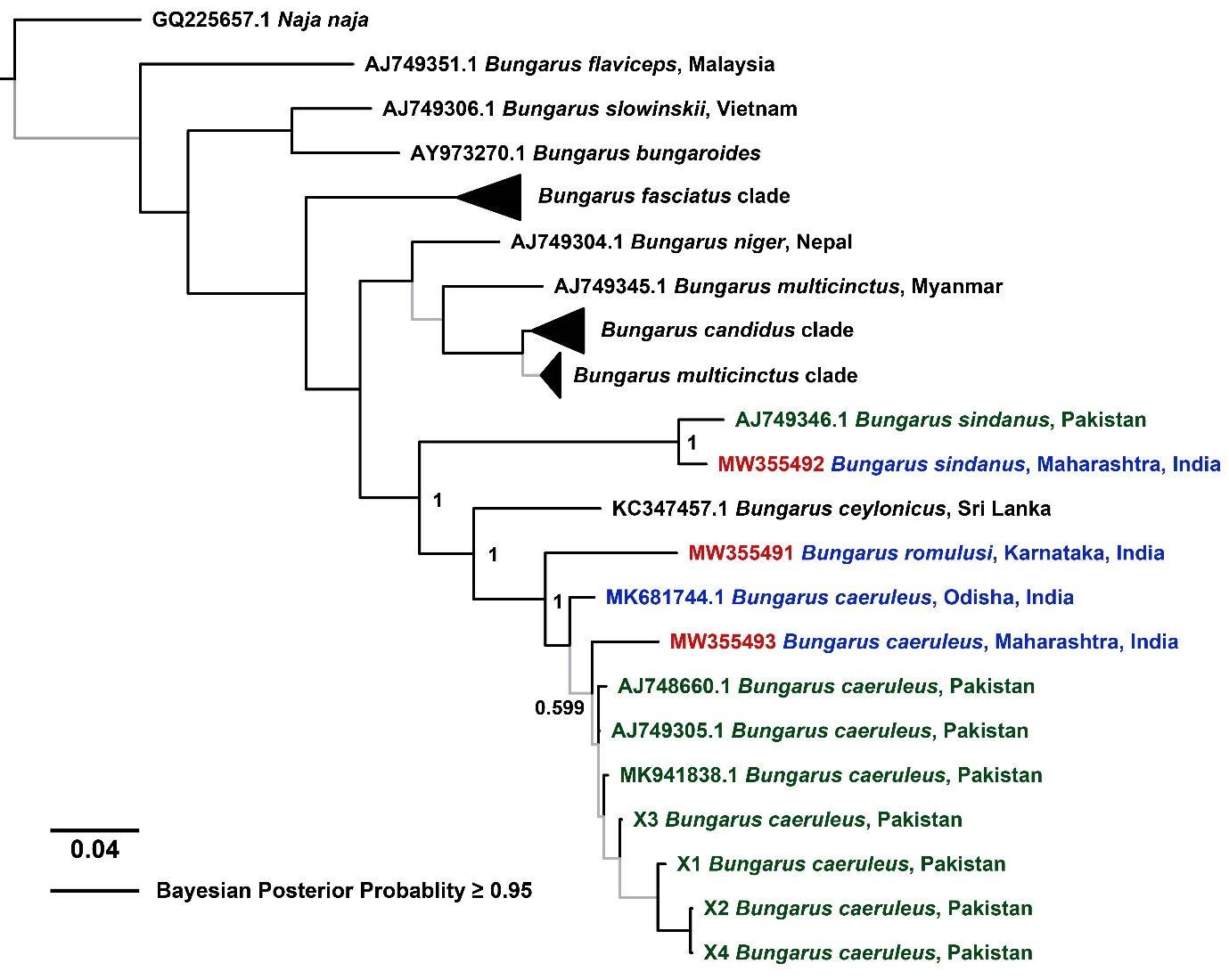


**Figure S1.** The Bayesian cytochrome *b* phylogeny of *Bungarus* species. The Bayesian cytochrome *b* phylogeny of *Bungarus* species in Asia is shown here. Terminal labels coloured in blue and green represent krait lineages from India and Pakistan, respectively, and the accession numbers of individuals sequenced in this study have been highlighted in red. Well-supported branches (BPP ≥ 0.95) are shown in thick black lines, and the node support for clades of interest are indicated. The scale for branch lengths (number of nucleotide substitutions per site) is also presented..


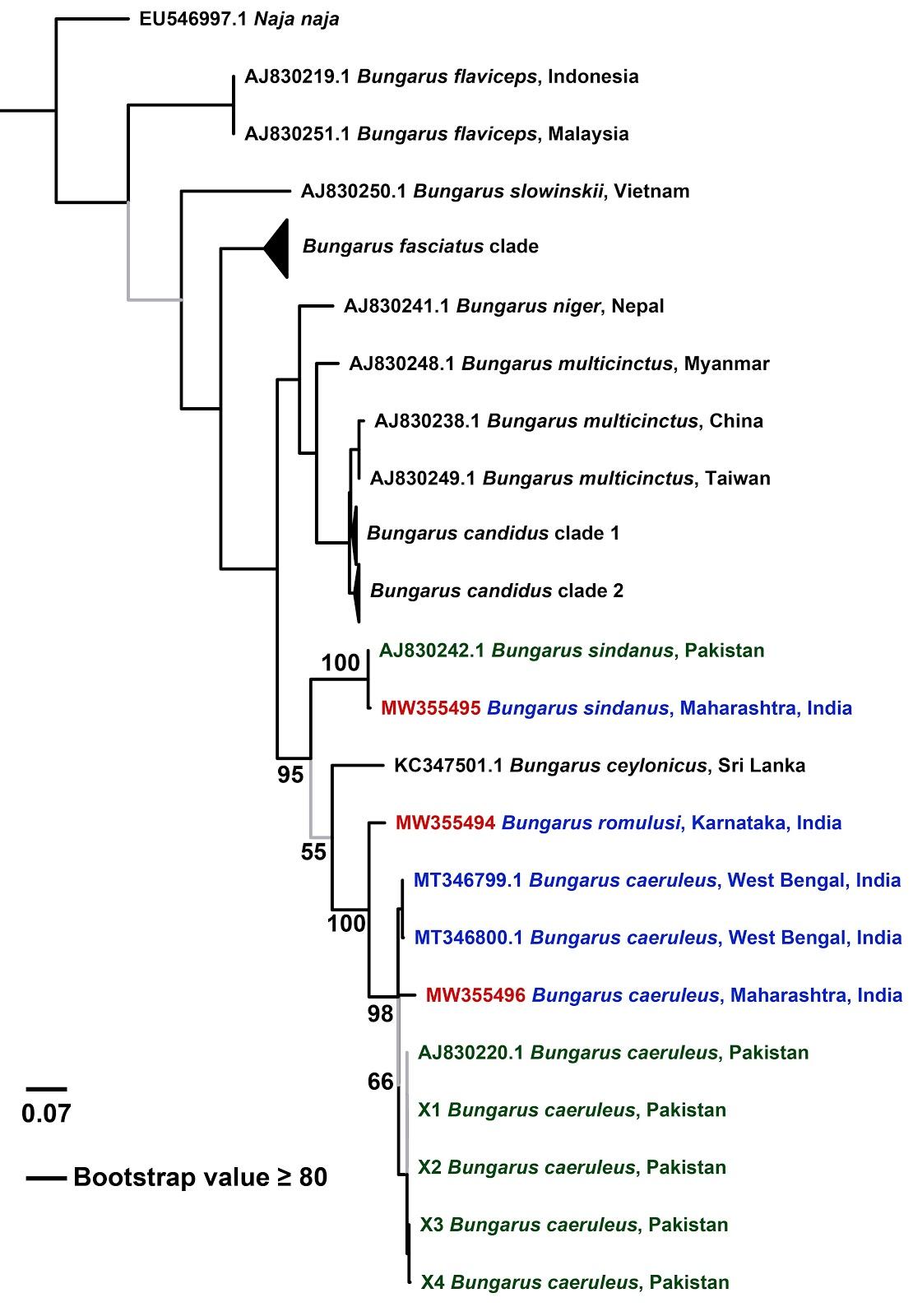


**Figure S2.** Maximum likelihood phylogeny (ND4 marker) for *Bungarus* species. This tree depicts the relationship between various species of kraits found in Southeast Asia. Terminal labels coloured in blue and green represent krait lineages from India and Pakistan, respectively, and the accession numbers of individuals sequenced in this study have been highlighted in red. Well-supported branches (BS ≥ 80) are shown in thick black lines, and the node support for clades of interest are indicated. The scale for branch lengths (number of nucleotide substitutions per site) is also presented..


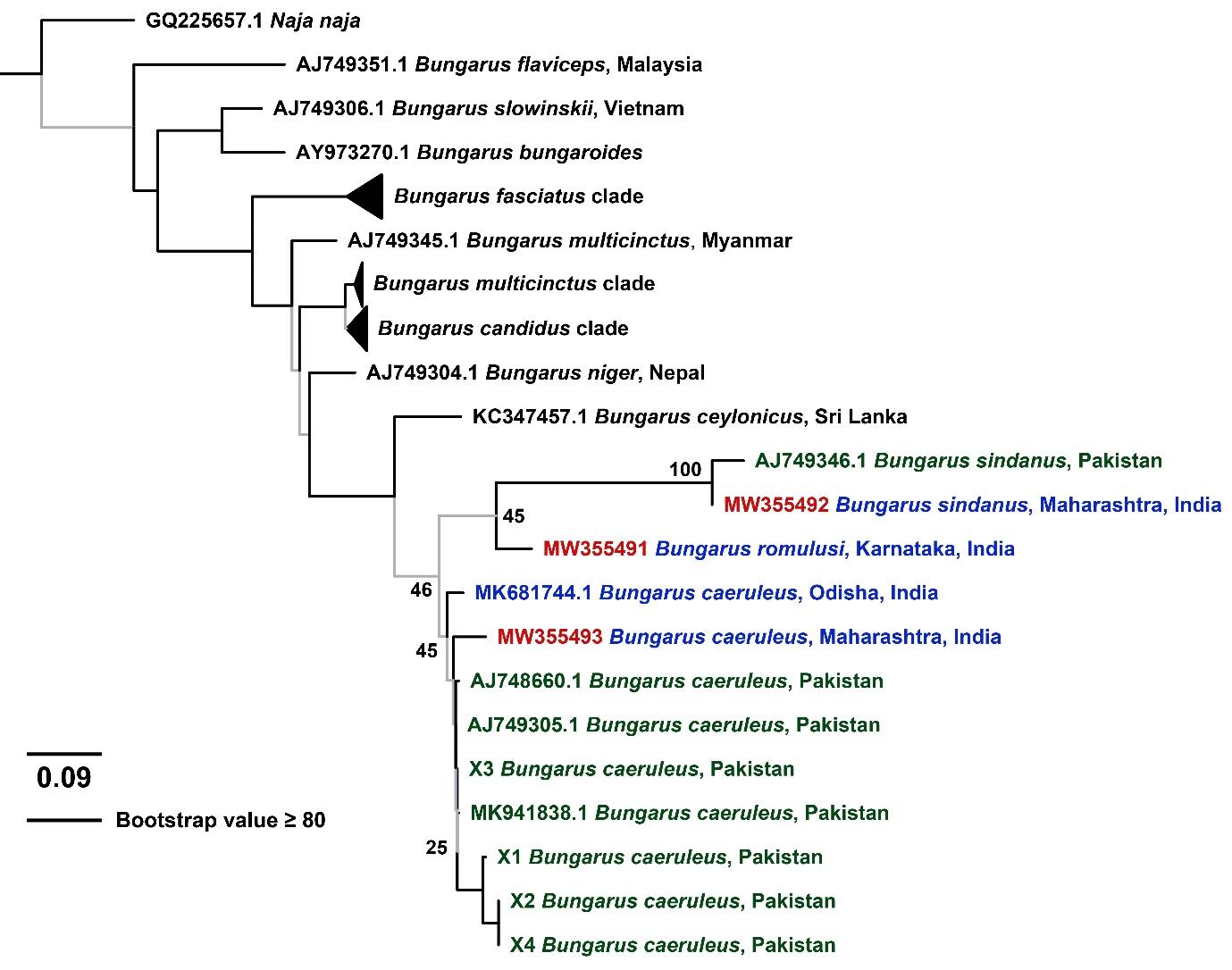


**Figure S3:** Maximum likelihood phylogeny (cyt *b* marker) for *Bungarus* species. Built using cytochrome *b* sequences, this ML tree depicts the relationship between various species of kraits found in Southeast Asia. Terminal labels coloured in blue and green represent krait lineages from India and Pakistan, respectively, and the accession numbers of individuals sequenced in this study have been highlighted in red. Well-supported branches (BS ≥ 80) are shown in thick black lines, and the node support for clades of interest are indicated. The scale for branch lengths (number of nucleotide substitutions per site) is also presented.


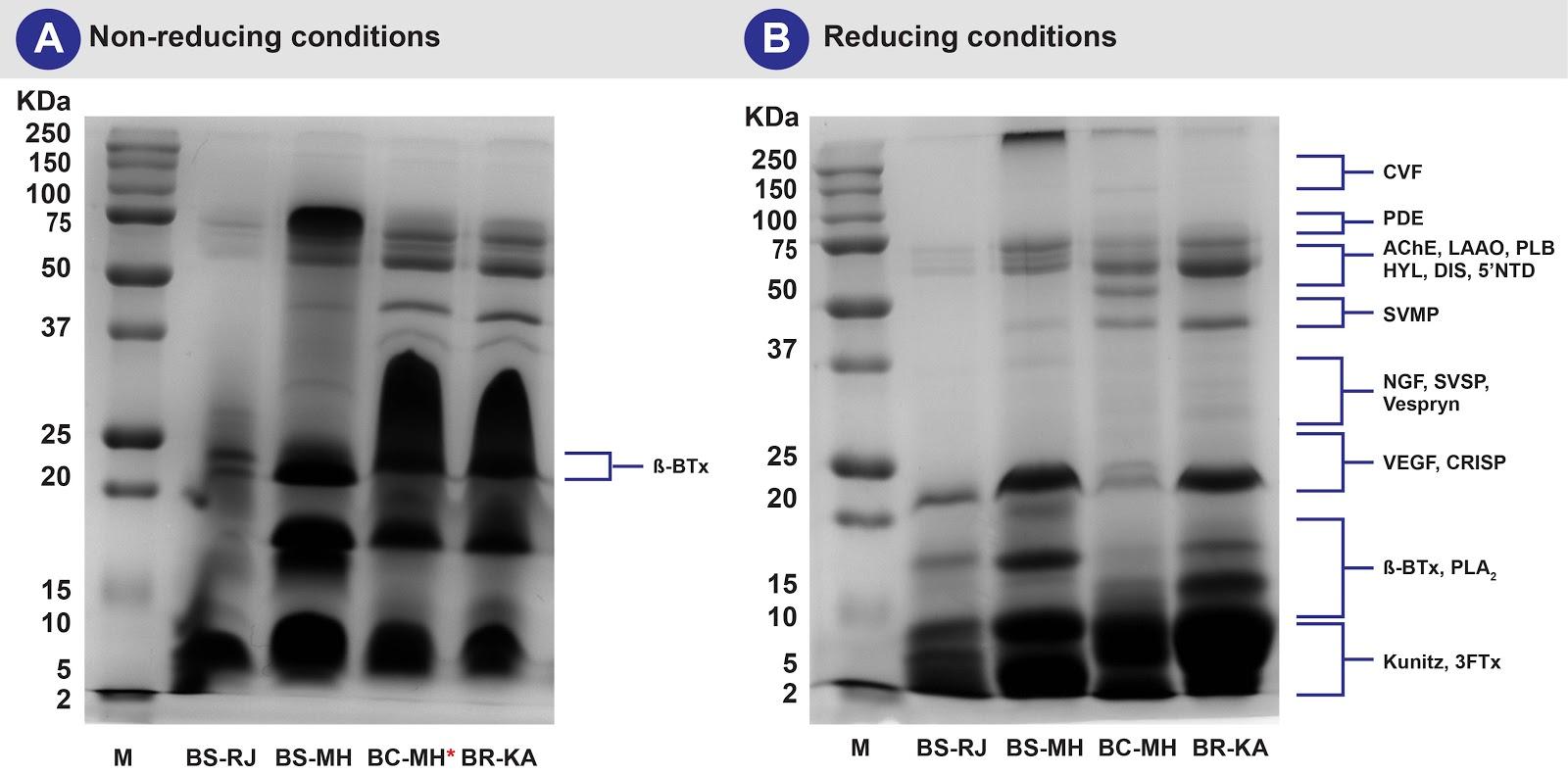


**Figure S4.** Electrophoretic separation of *Bungarus* venoms under non-reducing and reducing conditions. This figure depicts the SDS-PAGE profiles of *Bungarus* venoms under **(A)** non-reducing and **(B)** reducing conditions. Lanes 1 to 5 represent the marker (2 - 250 KDa), *B. sindanus* (Rajasthan and Maharashtra), *B. caeruleus* (Maharashtra) and *B. romulusi* (Karnataka).  *A pooled venom sample of *B. caeruleus* from Maharashtra has been used as a representative population .


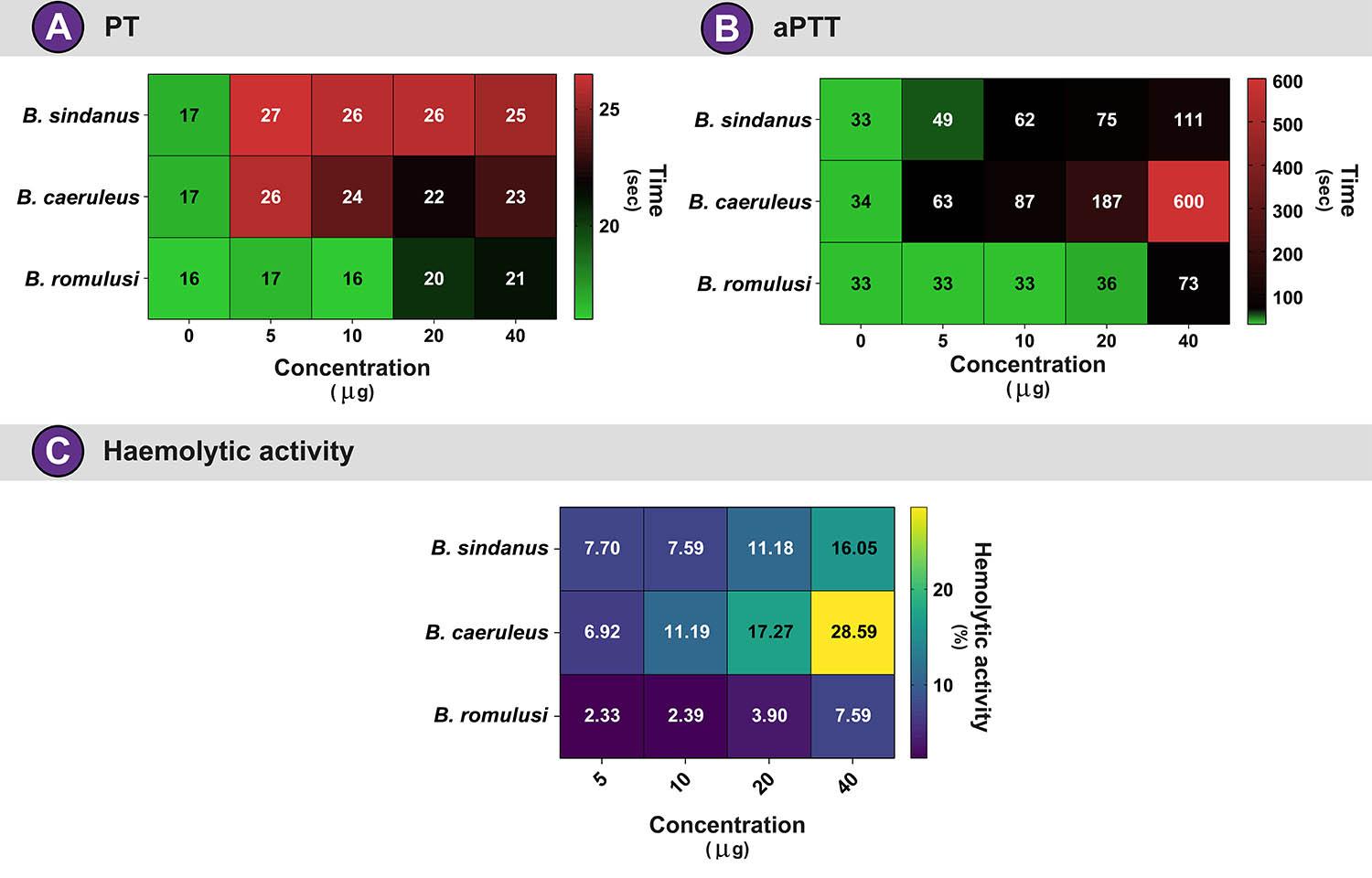


**Figure S5.** Pharmacological activities of *Bungarus* venoms from Western India. The effect of various concentrations (0, 5, 10, 20, 40 µg) of *Bungarus* venoms on the **(A)** extrinsic and **(B)** intrinsic pathways of blood coagulation, respectively, and **(C)** haemolysis are depicted in this figure as heatmaps. The time taken (in seconds) for the initial fibrin clot formation is represented by the numbers inside each square in panels A and B. The relative haemolytic activity (in %) calculated in relation to the activity of the positive control (0.5% Triton X) is represented by the numbers inside the squares in panel C.


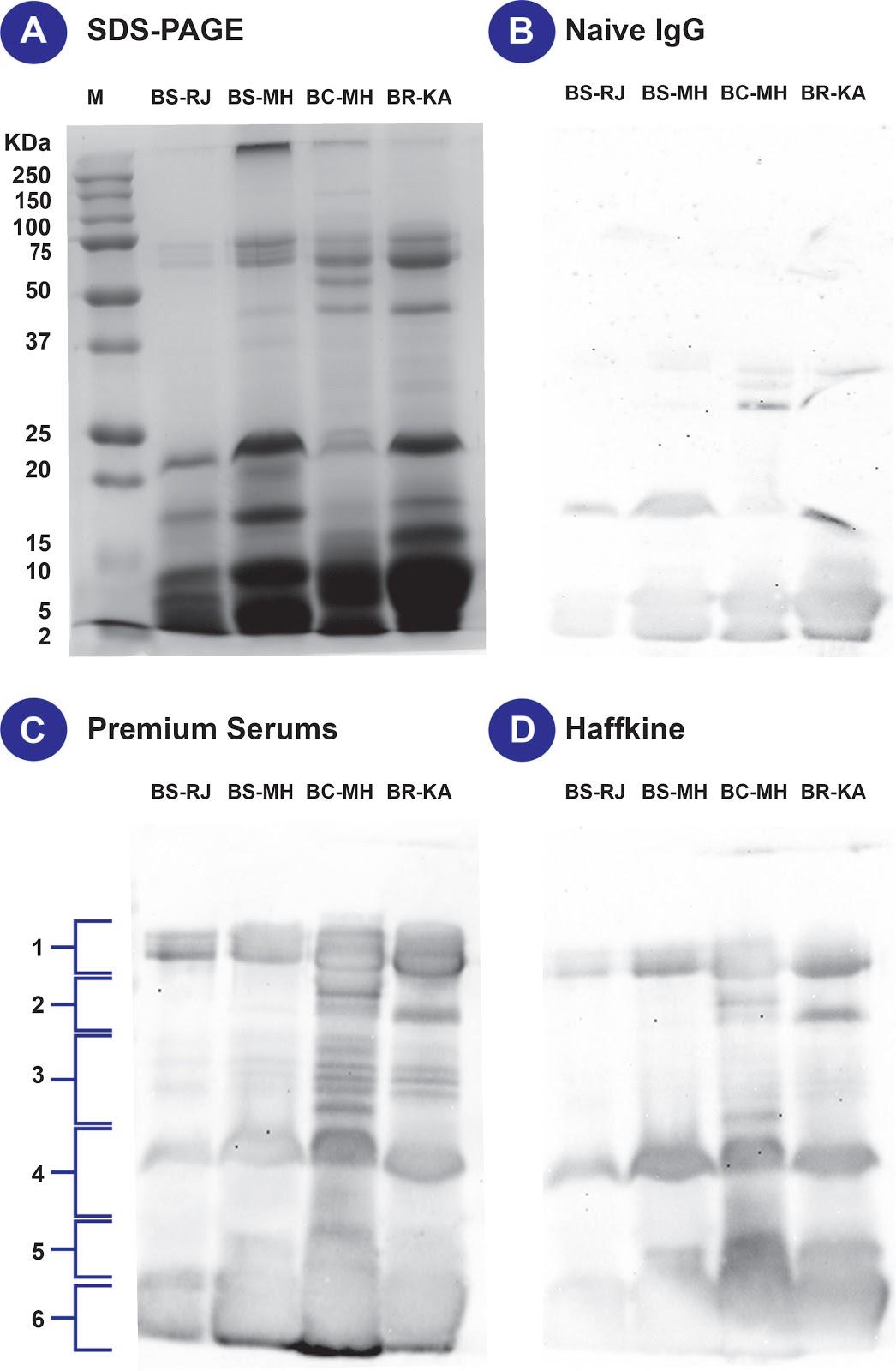


**Figure S6.** Western blotting of Indian polyvalent antivenoms against the cryptic *Bungarus* venoms. The recognition capability of commercial antivenoms against toxins (bands 1 - 6) in the venoms of *B. sindanus*, Rajasthan (BS-RJ); *B. sindanus,* Maharashtra (BS-MH); *B. caeruleus*, Maharashtra (BC-MH); and *B. romulusi*, Karnataka (BR-KA) is depicted here. Panels represent **(A)** SDS-PAGE, **(B)** Naive IgG, **(C)** Premium Serums and **(D)** Haffkine antivenoms. A standard protein marker (**M)** was run along with the venoms.

**Table S1.** Details of *Bungarus* venom samples investigated in this study.

| **Species** | **No. of**  **individuals** | **Protein concentration**  **(mg/ml)** | **Sampling location** |
| --- | --- | --- | --- |
| ***B. sindanus**** | 2 | 0.274 | Bikaner,  Rajasthan |
| ***B. sindanus*** | 1 | 0.128 | Pune,  Maharashtra |
| ***B. caeruleus*** | 1 | 0.099 | Pune,  Maharashtra |
| ***B. romulusi*** | 1 | 0.122 | Bannerghatta,  Karnataka |

*****This sample was analysed previously [15].

**Table S2.** A: Evolutionary sequence divergence estimated using the maximum composite likelihood (MCL) model for ND4 marker.

| **Accession no.**  **Species**  **Origin** | **AJ830220.1 *Bungarus caeruleus* Pakistan** | **AJ830242.1 *Bungarus sindanus* Pakistan** | ***Bungarus caeruleus* Pakistan 01** | ***Bungarus caeruleus* Pakistan 02** | ***Bungarus caeruleus* Pakistan 03** | ***Bungarus caeruleus* Pakistan 04** | **MT346799.1 *Bungarus caeruleus* West Bengal India** | **MT346800.1 *Bungarus caeruleus* West Bengal India** | **MW355496**  ***Bungarus caeruleus* Maharashtra India** | **MW355494**  ***Bungarus romulusi* Karnataka India** | **MW355495**  ***Bungarus sindanus* Maharashtra India** |
| --- | --- | --- | --- | --- | --- | --- | --- | --- | --- | --- | --- |
| AJ830220.1 *Bungarus caeruleus* Pakistan | 0 | 15.3 | 0 | 0 | 0.34 | 0.52 | 2.27 | 2.33 | 3.73 | 7.89 | 16.17 |
| AJ830242.1 *Bungarus sindanus* Pakistan |  | 0 | 15.97 | 15.97 | 15.98 | 15.77 | 15.63 | 15.32 | 16.66 | 14.37 | **0.31** |
| *Bungarus caeruleus* Pakistan 01 |  |  | 0 | 0 | 0.34 | 0.52 | 2.37 | 2.28 | 2.35 | 7.72 | 16.42 |
| *Bungarus caeruleus* Pakistan 02 |  |  |  | 0 | 0.34 | 0.52 | 2.37 | 2.28 | 2.35 | 7.72 | 16.42 |
| *Bungarus caeruleus* Pakistan 03 |  |  |  |  | 0 | 0.17 | 2.74 | 2.64 | 2.73 | 8.11 | 16.43 |
| *Bungarus caeruleus* Pakistan 04 |  |  |  |  |  | 0 | 2.93 | 2.82 | 2.91 | 8.30 | 16.21 |
| MT346799.1 *Bungarus caeruleus* West Bengal India |  |  |  |  |  |  | 0 | 0.15 | 2.69 | 7.09 | 16.28 |
| MT346800.1 *Bungarus caeruleus* West Bengal India |  |  |  |  |  |  |  | 0 | 3.25 | 6.96 | 16.02 |
| MW355496  *Bungarus caeruleus* Maharashtra India |  |  |  |  |  |  |  |  | 0 | **8.61** | 15.66 |
| MW355494  *Bungarus romulusi* Karnataka India |  |  |  |  |  |  |  |  |  | 0 | **14.87** |
| MW355495  *Bungarus sindanus* Maharashtra India |  |  |  |  |  |  |  |  |  |  | 0 |

This table shows the evolutionary distance between *Bungarus* species in Asia for ND4 marker calculated using maximum composite likelihood (MCL) model. Distances between species of interest have been highlighted in blue. Accession numbers of individuals sequenced in this study have been marked in red.

**Table S3.** Evolutionary sequence divergence estimated using the MCL model for cytochrome b marker.

| **Accession no.**  **Species**  **Origin** | **AJ749305.1 *Bungarus caeruleus* Pakistan** | **MK941838.1 *Bungarus caeruleus* Pakistan** | **MK681744.1 *Bungarus caeruleus* India** | ***Bungarus caeruleus* Pakistan 01** | ***Bungarus caeruleus* Pakistan 02** | ***Bungarus caeruleus* Pakistan 03** | ***Bungarus caeruleus* Pakistan 04** | **AJ749346.1 *Bungarus sindanus* Pakistan** | **MW355493**  ***Bungarus caeruleus* Maharashtra India** | **MW355491**  ***Bungarus romulusi* Karnataka India** | **MW355492**  ***Bungarus sindanus* Maharashtra India** |
| --- | --- | --- | --- | --- | --- | --- | --- | --- | --- | --- | --- |
| AJ749305.1 *Bungarus caeruleus* Pakistan | 0 | 0.29 | 2.86 | 1.38 | 4.23 | 0.16 | 4.23 | 28.75 | 3.93 | 10.07 | 20.66 |
| MK941838.1 *Bungarus caeruleus* Pakistan |  | 0 | 2.65 | 1.13 | 2.08 | 0.16 | 2.08 | 17.55 | 5.61 | 6.63 | 18.01 |
| MK681744.1 *Bungarus caeruleus* India |  |  | 0 | 2.86 | 2.86 | 2.88 | 2.86 | 18.74 | 3.42 | 5.94 | 19.56 |
| *Bungarus caeruleus* Pakistan 01 |  |  |  | 0 | 0.83 | 0 | 0.83 | 17.6 | 3.69 | 8.83 | 18.16 |
| *Bungarus caeruleus* Pakistan 02 |  |  |  |  | 0 | 0 | 0 | 29.72 | 6.55 | 12.04 | 23.03 |
| *Bungarus caeruleus* Pakistan 03 |  |  |  |  |  | 0 | 0 | 16.45 | 6.35 | 6.67 | 16.96 |
| *Bungarus caeruleus* Pakistan 04 |  |  |  |  |  |  | 0 | 29.72 | 6.55 | 12.04 | 23.03 |
| AJ749346.1 *Bungarus sindanus* Pakistan |  |  |  |  |  |  |  | 0 | 32.27 | 24.08 | **3.03** |
| MW355493  *Bungarus caeruleus* Maharashtra India |  |  |  |  |  |  |  |  | 0 | **14.26** | 24.24 |
| MW355491  *Bungarus romulusi* Karnataka India |  |  |  |  |  |  |  |  |  | 0 | **19.96** |
| MW355492  *Bungarus sindanus* Maharashtra India |  |  |  |  |  |  |  |  |  |  | 0 |

This table shows the evolutionary distance between *Bungarus* species in Asia for cytochrome b marker calculated using maximum composite likelihood (MCL) model. Distances between species of interest have been highlighted in blue. Accession numbers of individuals sequenced in this study have been marked in red..

**Table S4.** Evolutionary divergence estimated as p-distance for ND4 marker.

| **Accession no.**  **Species**  **Origin** | **AJ830221 *Bungarus caeruleus* Pakistan** | **AJ830242.1 *Bungarus sindanus* Pakistan** | ***Bungarus caeruleus* Pakistan 01** | ***Bungarus caeruleus* Pakistan 02** | ***Bungarus caeruleus* Pakistan 03** | ***Bungarus caeruleus* Pakistan 04** | **MT346799.1 *Bungarus caeruleus* West Bengal India** | **MT346801 *Bungarus caeruleus* West Bengal India** | **MW355496**  ***Bungarus caeruleus* Maharashtra India** | **MW355494**  ***Bungarus romulusi* Karnataka India** | **MW355495**  ***Bungarus sindanus* Maharashtra India** |
| --- | --- | --- | --- | --- | --- | --- | --- | --- | --- | --- | --- |
| AJ830221 *Bungarus caeruleus* Pakistan | 0 | 13.48 | 0 | 0 | 0.34 | 0.51 | 2.23 | 2.29 | 0.35 | 7.42 | 14.15 |
| AJ830242.1 *Bungarus sindanus* Pakistan |  | 0 | 13.98 | 13.98 | 13.98 | 13.81 | 13.71 | 13.45 | 14.39 | 12.82 | **0.31** |
| *Bungarus caeruleus* Pakistan 01 |  |  | 0 | 0 | 0.34 | 0.51 | 2.32 | 2.24 | 2.31 | **7.25** | 14.33 |
| *Bungarus caeruleus* Pakistan 02 |  |  |  | 0 | 0.34 | 0.51 | 2.32 | 2.24 | 2.31 | 7.25 | 14.33 |
| *Bungarus caeruleus* Pakistan 03 |  |  |  |  | 0 | 0.17 | 2.68 | 2.59 | 2.67 | 7.59 | 14.33 |
| *Bungarus caeruleus* Pakistan 04 |  |  |  |  |  | 0 | 2.86 | 2.76 | 2.85 | 7.77 | 14.16 |
| MT346799.1 *Bungarus caeruleus* West Bengal India |  |  |  |  |  |  | 0 | 0.15 | 2.62 | 6.69 | 14.21 |
| MT346801 *Bungarus caeruleus* West Bengal India |  |  |  |  |  |  |  | 0 | 3.14 | 6.57 | 13.99 |
| MW355496  *Bungarus caeruleus* Maharashtra India |  |  |  |  |  |  |  |  | 0 | **7.98** | 13.75 |
| MW355494  *Bungarus romulusi* Karnataka India |  |  |  |  |  |  |  |  |  | 0 | **13.20** |
| MW355495  *Bungarus sindanus* Maharashtra India |  |  |  |  |  |  |  |  |  |  | 0 |

This table shows the evolutionary distance between *Bungarus* species in Asia for ND4 marker estimated as p-distance. Distances between species of interest have been highlighted in blue. Accession numbers of individuals sequenced in this study have been marked in red.

**Table S5.**: Evolutionary divergence estimated as p-distance for cytochrome *b* marker.

| **Accession no.**  **Species**  **Origin** | **AJ749305.1 *Bungarus caeruleus* Pakistan** | **MK681744.1 *Bungarus caeruleus* India** | ***Bungarus caeruleus* Pakistan 01** | ***Bungarus caeruleus* Pakistan 02** | ***Bungarus caeruleus* Pakistan 03** | ***Bungarus caeruleus* Pakistan 04** | **AJ749346.1 *Bungarus sindanus* Pakistan** | **MW355493*Bungarus caeruleus* Maharashtra India** | **MW355491**  ***Bungarus romulusi* Karnataka India** | **MW355492**  ***Bungarus sindanus* Maharashtra India** |
| --- | --- | --- | --- | --- | --- | --- | --- | --- | --- | --- |
| AJ749305.1 *Bungarus caeruleus* Pakistan | 0 | 2.75 | 1.34 | 3.79 | 0.16 | 3.79 | 18.95 | 3.05 | 8.71 | 15.54 |
| MK681744.1 *Bungarus caeruleus* India |  | 0 | 2.75 | 2.75 | 2.77 | 2.75 | 14.53 | 3.28 | 5.51 | 15.03 |
| *Bungarus caeruleus* Pakistan 01 |  |  | 0 | 0.67 | 0 | 0.67 | 13.63 | 3.47 | 7.79 | 13.97 |
| *Bungarus caeruleus* Pakistan 02 |  |  |  | 0 | 0 | 0 | 18.92 | 5.73 | 9.98 | 16.29 |
| *Bungarus caeruleus* Pakistan 03 |  |  |  |  | 0 | 0 | 13.26 | 4.59 | 6.13 | 13.59 |
| *Bungarus caeruleus* Pakistan 04 |  |  |  |  |  | 0 | 18.92 | 5.73 | 9.98 | 16.29 |
| AJ749346.1 *Bungarus sindanus* Pakistan |  |  |  |  |  |  | 0 | 19.36 | 17.28 | **2.87** |
| MW355493  *Bungarus caeruleus* Maharashtra India |  |  |  |  |  |  |  | 0 | **10.92** | 16.38 |
| MW355491  *Bungarus romulusi* Karnataka India |  |  |  |  |  |  |  |  | 0 | **15.38** |
| MW355492  *Bungarus sindanus* Maharashtra India |  |  |  |  |  |  |  |  |  | 0 |

This table shows the evolutionary distance between *Bungarus* species in Asia for cytochrome b marker estimated as p-distance. Distances between species of interest have been highlighted in blue. Accession numbers of individuals sequenced in this study have been marked in red.

**Table S6, S7 and S8:** Venom proteomic compositions of cryptic *Bungarus* species. Peaks Studio X Plus was used to examine raw MS/MS spectra against the National Center for Biotechnology Information’s (NCBI) non-redundant (nr) database (Serpentes: 8570) for the identification of various toxin families present in the venom. The tables illustrates the key statistics of these searches, including the accession number, species name, -10lgP values, number of high confidence peptides, unique peptides, percent abundance of each toxin hit, average molecular mass (KDa), the toxin family of the matching NCBI entry.

**Table S6 .** The proteomic composition of *B. sindanus* venom.

| **Sr. No.** | **Accession** | **Species** | **-10lgP** | **#Peptides** | **#Unique** | **Relative Abundance of Toxin Hit (%)** | **Avg. Mass (KDa)** | **Family** |
| --- | --- | --- | --- | --- | --- | --- | --- | --- |
| **Phospholipase A2 (PLA_2_): 52.57%** | | | | | | | | |
| 1 | ABM88801 | *Hoplocephalus stephensii* | 300.23 | 19 | 14 | 27.550% | 11.66 | PLA2 |
| 2 | ACY68711 | *Parasuta nigriceps* | 421.05 | 38 | 37 | 14.775% | 17.26 | PLA2 |
| 3 | AAB24834 | *Bungarus fasciatus* | 338.21 | 24 | 23 | 8.711% | 16.66 | PLA2 |
| 4 | ABG75909 | *Bungarus fasciatus* | 310.13 | 14 | 7 | 1.175% | 9.80 | PLA2 |
| 5 | BAC77656.1 | *Bungarus flaviceps* | 96.75 | 1 | 1 | 0.114% | 15.88 | PLA2 |
| 6 | ADF50035.1 | *Bungarus flaviceps* | 96.75 | 1 | 1 | 0.114% | 15.85 | PLA2 |
| 7 | Q7T2Q4.1 | *Bungarus flaviceps flaviceps* | 96.75 | 1 | 1 | 0.114% | 15.88 | PLA2 |
| 8 | ACY68711.1 | *Parasuta nigriceps* | 87.44 | 1 | 1 | 0.003% | 15.46 | PLA2 |
| 9 | ABU68503.1 | *Trimorphodon biscutatus* | 125.75 | 3 | 1 | 0.001% | 16.62 | PLA2 |
| 10 | JAB52761.1 | *Micrurus fulvius* | 55.04 | 1 | 1 | 0.001% | 15.82 | PLA2 |
| 11 | JAS04986.1 | *Micrurus fulvius* | 55.04 | 1 | 1 | 0.001% | 15.92 | PLA2 |
| 12 | JAS04987.1 | *Micrurus fulvius* | 55.04 | 1 | 1 | 0.001% | 15.91 | PLA2 |
| 13 | JAS04982.1 | *Micrurus fulvius* | 55.04 | 1 | 1 | 0.001% | 15.92 | PLA2 |
| 14 | JAS04985.1 | *Micrurus fulvius* | 55.04 | 1 | 1 | 0.001% | 15.94 | PLA2 |
| 15 | JAI08996.1 | *Micrurus fulvius* | 55.04 | 1 | 1 | 0.001% | 15.94 | PLA2 |
| 16 | JAI08997.1 | *Micrurus fulvius* | 55.04 | 1 | 1 | 0.001% | 15.92 | PLA2 |
| 17 | JAI08998.1 | *Micrurus fulvius* | 55.04 | 1 | 1 | 0.001% | 15.91 | PLA2 |
| 18 | JAI08993.1 | *Micrurus fulvius* | 55.04 | 1 | 1 | 0.001% | 15.92 | PLA2 |
| 19 | JAB52775.1 | *Micrurus fulvius* | 55.04 | 1 | 1 | 0.001% | 15.92 | PLA2 |
| 20 | JAB52776.1 | *Micrurus fulvius* | 55.04 | 1 | 1 | 0.001% | 15.91 | PLA2 |
| 21 | JAS05103.1 | *Micrurus fulvius* | 55.04 | 1 | 1 | 0.001% | 15.81 | PLA2 |
| 22 | JAS05108.1 | *Micrurus fulvius* | 55.04 | 1 | 1 | 0.001% | 15.72 | PLA2 |
| 23 | JAS05109.1 | *Micrurus fulvius* | 55.04 | 1 | 1 | 0.001% | 15.87 | PLA2 |
| 24 | JAS05107.1 | *Micrurus fulvius* | 55.04 | 1 | 1 | 0.001% | 15.71 | PLA2 |
| 25 | JAS05106.1 | *Micrurus fulvius* | 55.04 | 1 | 1 | 0.001% | 15.77 | PLA2 |
| 26 | JAS05111.1 | *Micrurus fulvius* | 55.04 | 1 | 1 | 0.001% | 15.77 | PLA2 |
| 27 | JAS05101.1 | *Micrurus fulvius* | 55.04 | 1 | 1 | 0.001% | 15.74 | PLA2 |
| 28 | JAS05102.1 | *Micrurus fulvius* | 55.04 | 1 | 1 | 0.001% | 15.78 | PLA2 |
| 29 | JAS05104.1 | *Micrurus fulvius* | 55.04 | 1 | 1 | 0.001% | 15.78 | PLA2 |
| 30 | JAS05105.1 | *Micrurus fulvius* | 55.04 | 1 | 1 | 0.001% | 15.95 | PLA2 |
| 31 | JAS05110.1 | *Micrurus fulvius* | 55.04 | 1 | 1 | 0.001% | 15.77 | PLA2 |
| **Three-finger toxins (3FTx): 13.71%** | | | | | | | | |
| 32 | Q9YGI8.1 | *Bungarus multicinctus* | 200.58 | 5 | 5 | 1.891% | 9.52 | Type I (short) α-neurotoxin |
| 33 | CAA06887.1 | *Bungarus multicinctus multicinctus* | 200.58 | 5 | 5 | 1.891% | 9.52 | Type I (short) α-neurotoxin |
| 34 | P15816.1 | *Bungarus multicinctus* | 297.69 | 10 | 1 | 1.829% | 9.52 | Type II (long) κ-neurotoxin |
| 35 | CAA35774.1 | *Bungarus multicinctus* | 297.69 | 10 | 1 | 1.829% | 9.52 | Type II (long) κ-neurotoxin |
| 36 | CAA45882 | *Bungarus multicinctus* | 197.44 | 4 | 4 | 0.822% | 8.63 | Type I (short) α-neurotoxin |
| 37 | 1IJC | *Bungarus candidus* | 159.08 | 3 | 3 | 0.680% | 7.29 | Non-conventional neurotoxin |
| 38 | 1F94 | *Bungarus candidus* | 159.08 | 3 | 3 | 0.680% | 7.29 | Non-conventional neurotoxin |
| 39 | P81782.1 | *Bungarus candidus* | 159.08 | 3 | 3 | 0.680% | 7.29 | Non-conventional neurotoxin |
| 40 | CAA06885 | *Bungarus multicinctus multicinctus* | 203.81 | 4 | 4 | 0.621% | 10.63 | Type II (long) κ-neurotoxin |
| 41 | AAL30054.1 | *Bungarus candidus* | 219.26 | 8 | 6 | 0.507% | 9.58 | Type II (long) κ-neurotoxin |
| 42 | Q8AY56.1 | *Bungarus candidus* | 219.26 | 8 | 6 | 0.507% | 9.58 | Type II (long) κ-neurotoxin |
| 43 | Q9W729.1 | *Bungarus multicinctus* | 163.69 | 2 | 2 | 0.378% | 9.66 | Type II (long) κ-neurotoxin |
| 44 | CAB46659.1 | *Bungarus multicinctus* | 163.69 | 2 | 2 | 0.378% | 9.66 | Type II (long) κ-neurotoxin |
| 45 | CAA45882 | *Bungarus multicinctus* | 167.03 | 2 | 2 | 0.275% | 8.57 | Type I (short) α-neurotoxin |
| 46 | AAL30059 | *Bungarus candidus* | 157.51 | 3 | 3 | 0.212% | 9.75 | Non-conventional neurotoxin |
| 47 | CAM11302.1 | *Bungarus caeruleus* | 117.13 | 2 | 2 | 0.048% | 8.31 | Type II (long) α-neurotoxin |
| 48 | D2N116.1 | *Bungarus caeruleus* | 117.13 | 2 | 2 | 0.048% | 8.31 | Type II (long) α-neurotoxin |
| 49 | ACR78492.1 | *Drysdalia coronoides* | 54.81 | 1 | 1 | 0.017% | 9.61 | Type II (long) α-neurotoxin |
| 50 | ACR78484.1 | *Drysdalia coronoides* | 54.81 | 1 | 1 | 0.017% | 9.61 | Type II (long) α-neurotoxin |
| 51 | F8J2F2.1 | *Drysdalia coronoides* | 54.81 | 1 | 1 | 0.017% | 9.61 | Type II (long) α-neurotoxin |
| 52 | ACR78483.1 | *Drysdalia coronoides* | 54.81 | 1 | 1 | 0.017% | 9.66 | Type II (long) α-neurotoxin |
| 53 | ACR78488.1 | *Drysdalia coronoides* | 54.81 | 1 | 1 | 0.017% | 9.66 | Type II (long) α-neurotoxin |
| 54 | JAA74738.1 | *Denisonia devisi* | 54.81 | 1 | 1 | 0.017% | 10.11 | Type II (long) α-neurotoxin |
| 55 | ACR78486.1 | *Drysdalia coronoides* | 54.81 | 1 | 1 | 0.017% | 9.53 | Type II (long) α-neurotoxin |
| 56 | F8J2E6.1 | *Drysdalia coronoides* | 54.81 | 1 | 1 | 0.017% | 9.53 | Type II (long) α-neurotoxin |
| 57 | ACR78491.1 | *Drysdalia coronoides* | 54.81 | 1 | 1 | 0.017% | 9.50 | Type II (long) α-neurotoxin |
| 58 | JAA74652.1 | *Acanthophis wellsi* | 54.81 | 1 | 1 | 0.017% | 9.64 | Type II (long) α-neurotoxin |
| 59 | JAA74651.1 | *Acanthophis wellsi* | 54.81 | 1 | 1 | 0.017% | 9.64 | Type II (long) α-neurotoxin |
| 60 | JAA74655.1 | *Acanthophis wellsi* | 54.81 | 1 | 1 | 0.017% | 9.57 | Type II (long) α-neurotoxin |
| 61 | ABK63538.1 | *Tropidechis carinatus* | 54.81 | 1 | 1 | 0.017% | 10.14 | Type II (long) α-neurotoxin |
| 62 | A8HDK4.1 | *Tropidechis carinatus* | 54.81 | 1 | 1 | 0.017% | 10.14 | Type II (long) α-neurotoxin |
| 63 | ABX58163.1 | *Austrelaps labialis* | 54.81 | 1 | 1 | 0.017% | 9.59 | Type II (long) α-neurotoxin |
| 64 | ABX58162.1 | *Austrelaps labialis* | 54.81 | 1 | 1 | 0.017% | 9.57 | Type II (long) α-neurotoxin |
| 65 | ABX58156.1 | *Austrelaps labialis* | 54.81 | 1 | 1 | 0.017% | 9.59 | Type II (long) α-neurotoxin |
| 66 | ABX58159.1 | *Austrelaps labialis* | 54.81 | 1 | 1 | 0.017% | 9.57 | Type II (long) α-neurotoxin |
| 67 | ABX58160.1 | *Austrelaps labialis* | 54.81 | 1 | 1 | 0.017% | 9.65 | Type II (long) α-neurotoxin |
| 68 | ABX58161.1 | *Austrelaps labialis* | 54.81 | 1 | 1 | 0.017% | 9.62 | Type II (long) α-neurotoxin |
| 69 | ABX58158.1 | *Austrelaps labialis* | 54.81 | 1 | 1 | 0.017% | 9.57 | Type II (long) α-neurotoxin |
| 70 | JAA74768.1 | *Echiopsis curta* | 97.53 | 1 | 1 | 0.011% | 11.63 | Type II (long) α-neurotoxin |
| 71 | JAA74767.1 | *Echiopsis curta* | 97.53 | 1 | 1 | 0.011% | 11.63 | Type II (long) α-neurotoxin |
| 72 | P01387.1 | *Ophiophagus hannah* | 97.53 | 1 | 1 | 0.011% | 8.11 | Type II (long) α-neurotoxin |
| 73 | Q53B58.1 | *Ophiophagus hannah* | 97.53 | 1 | 1 | 0.011% | 10.21 | Type II (long) α-neurotoxin |
| 74 | AAT97250.1 | *Ophiophagus hannah* | 97.53 | 1 | 1 | 0.011% | 10.21 | Type II (long) α-neurotoxin |
| 75 | ETE58964 | *Ophiophagus hannah* | 164.12 | 2 | 2 | 0.009% | 9.28 | Type I (short) α-neurotoxin |
| 76 | P01397.2 | *Dendroaspis polylepis polylepis* | 54.53 | 1 | 1 | 0.002% | 7.95 | Type II (long) α-neurotoxin |
| 77 | P25667.1 | *Dendroaspis polylepis polylepis* | 54.53 | 1 | 1 | 0.002% | 7.94 | Type II (long) α-neurotoxin |
| 78 | 1MR6 | *Bungarus multicinctus* | 77.2 | 1 | 1 | 0.002% | 7.54 | Non-conventional neurotoxin |
| 79 | CAA06885.1 | *Bungarus multicinctus multicinctus* | 77.2 | 1 | 1 | 0.002% | 9.83 | Non-conventional neurotoxin |
| 80 | Q9YGJ0.1 | *Bungarus multicinctus* | 77.2 | 1 | 1 | 0.002% | 9.83 | Non-conventional neurotoxin |
| 81 | AAD41806.1 | *Bungarus multicinctus* | 77.2 | 1 | 1 | 0.002% | 9.83 | Non-conventional neurotoxin |
| 82 | CAA72941.1 | *Bungarus multicinctus* | 77.2 | 1 | 1 | 0.002% | 9.76 | Non-conventional neurotoxin |
| 83 | CAD01082.1 | *Bungarus multicinctus* | 77.2 | 1 | 1 | 0.002% | 9.83 | Non-conventional neurotoxin |
| 84 | O12963.1 | *Bungarus multicinctus* | 77.2 | 1 | 1 | 0.002% | 9.76 | Non-conventional neurotoxin |
| **β-bungarotoxin: 11.61%** | | | | | | | | |
| 85 | AAL87003 | *Bungarus caeruleus* | 361.32 | 24 | 10 | 10.925% | 15.49 | β-bungarotoxin |
| 86 | CAD24466 | *Bungarus multicinctus* | 343.33 | 21 | 3 | 0.683% | 14.90 | β-bungarotoxin |
| 87 | 0402253A | *Bungarus multicinctus* | 256.44 | 10 | 1 | 0.000% | 13.56 | β-bungarotoxin |
| 88 | 0412250A | *Bungarus multicinctus* | 256.44 | 10 | 1 | 0.000% | 13.49 | β-bungarotoxin |
| **Cysteine-rich secretory proteins (CRISP): 9.09%** | | | | | | | | |
| 89 | ACE73577 | *Bungarus candidus* | 429.3 | 34 | 10 | 4.522% | 26.26 | CRISP |
| 90 | ACE73577 | *Bungarus candidus* | 429.3 | 34 | 10 | 4.522% | 31.19 | CRISP |
| 91 | ACE73578.1 | *Macrovipera lebetina* | 377.32 | 19 | 1 | 0.042% | 26.44 | CRISP |
| 92 | ACY68722.1 | *Parasuta nigriceps* | 108.97 | 1 | 1 | 0.001% | 20.02 | CRISP |
| **Kunitz-type serine protease inhibitor (Kunitz): 9.02%** | | | | | | | | |
| 93 | CAP74383.1 | *Bungarus multicinctus* | 224.26 | 4 | 3 | 3.294% | 9.23 | Kunitz |
| 94 | B4ESA4.1 | *Bungarus multicinctus* | 224.26 | 4 | 3 | 3.294% | 9.23 | Kunitz |
| 95 | Q8AY42.2 | *Bungarus candidus* | 157.2 | 3 | 2 | 0.534% | 8.92 | Kunitz |
| 96 | Q8AY43.2 | *Bungarus candidus* | 157.2 | 3 | 2 | 0.534% | 9.08 | Kunitz |
| 97 | AAL30069.1 | *Bungarus candidus* | 157.2 | 3 | 2 | 0.534% | 9.39 | Kunitz |
| 98 | AAL30068.1 | *Bungarus candidus* | 157.2 | 3 | 2 | 0.534% | 9.61 | Kunitz |
| 99 | B4ESA3.1 | *Bungarus multicinctus* | 104.39 | 2 | 1 | 0.074% | 9.16 | Kunitz |
| 100 | Q8AY41.2 | *Bungarus candidus* | 104.39 | 2 | 1 | 0.074% | 9.13 | Kunitz |
| 101 | CAP74382.1 | *Bungarus multicinctus* | 104.39 | 2 | 1 | 0.074% | 9.16 | Kunitz |
| 102 | AAL30070.1 | *Bungarus candidus* | 104.39 | 2 | 1 | 0.074% | 9.66 | Kunitz |
| **Vascular endothelial growth factor (VEGF): 2.26%** | | | | | | | | |
| 103 | JAA74898 | *Pseudonaja modesta* | 174.15 | 6 | 6 | 0.453% | 22.46 | VEGF |
| 104 | JAA74898 | *Pseudonaja modesta* | 174.15 | 6 | 6 | 0.453% | 22.46 | VEGF |
| 105 | JAA74688 | *Cacophis squamulosus* | 174.15 | 6 | 6 | 0.453% | 24.61 | VEGF |
| 106 | XP_026551344 | *Pseudonaja textilis* | 174.15 | 6 | 6 | 0.453% | 25.39 | VEGF |
| 107 | XP_026551344 | *Pseudonaja textilis* | 174.15 | 6 | 6 | 0.453% | 25.39 | VEGF |
| **Acetylcholinesterase (AChE): 1.12%** | | | | | | | | |
| 108 | Q92035 | *Bungarus fasciatus* | 448.54 | 49 | 3 | 0.157% | 67.89 | AChE |
| 109 | Q92035 | *Bungarus fasciatus* | 448.54 | 49 | 3 | 0.157% | 68.17 | AChE |
| 110 | Q92035 | *Bungarus fasciatus* | 448.54 | 49 | 3 | 0.157% | 68.17 | AChE |
| 111 | Q92035 | *Bungarus fasciatus* | 448.54 | 49 | 3 | 0.157% | 67.34 | AChE |
| 112 | AAC59905 | *Bungarus fasciatus* | 452.57 | 52 | 6 | 0.123% | 64.77 | AChE |
| 113 | AAC59905 | *Bungarus fasciatus* | 452.57 | 52 | 6 | 0.123% | 69.05 | AChE |
| 114 | AAC59905 | *Bungarus fasciatus* | 452.57 | 52 | 6 | 0.123% | 74.79 | AChE |
| 115 | AAC59905 | *Bungarus fasciatus* | 452.57 | 52 | 6 | 0.123% | 77.95 | AChE |
| **Snake venom metalloproteinase (Disintegrin-like): 0.49%** | | | | | | | | |
| 116 | ABN72537.1 | *Bungarus multicinctus* | 304.6 | 21 | 3 | 0.143% | 68.99 | Disintegrin-like |
| 117 | A8QL49.1 | *Bungarus multicinctus* | 304.6 | 21 | 3 | 0.143% | 68.99 | Disintegrin-like |
| 118 | LAA20517 | *Micrurus lemniscatus carvalhoi* | 255.57 | 7 | 6 | 0.110% | 21.44 | Disintegrin-like |
| 119 | AEH95531 | *Drysdalia coronoides* | 227.52 | 7 | 1 | 0.066% | 25.77 | Disintegrin-like |
| 120 | ABN72537 | *Bungarus multicinctus* | 311.2 | 21 | 2 | 0.022% | 68.84 | Disintegrin-like |
| 121 | ABN72536 | *Bungarus fasciatus* | 301.36 | 12 | 2 | 0.009% | 31.50 | Disintegrin-like |
| **5’-nucleotidase: 0.04%** | | | | | | | | |
| 122 | JAB52815 | *Micrurus fulvius* | 281.36 | 13 | 3 | 0.010% | 64.59 | 5’-nucleotidase |
| 123 | JAB52815 | *Micrurus fulvius* | 281.36 | 13 | 3 | 0.010% | 64.59 | 5’-nucleotidase |
| 124 | JAB52815 | *Micrurus fulvius* | 281.36 | 13 | 3 | 0.010% | 64.59 | 5’-nucleotidase |
| 125 | JAB52815 | *Micrurus fulvius* | 281.36 | 13 | 3 | 0.010% | 64.59 | 5’-nucleotidase |
| 126 | AHJ80886.1 | *Macrovipera lebetina* | 165.14 | 2 | 1 | 0.003% | 45.03 | 5’-nucleotidase |
| **Peptidase: 0.03%** | | | | | | | | |
| 127 | XP_026571757.1 | *Pseudonaja textilis* | 223 | 5 | 5 | 0.027% | 37.55 | Peptidase C1 |
| 128 | XP_034296605.1 | *Pantherophis guttatus* | 209.17 | 6 | 1 | 0.002% | 106.08 | Peptidase M1 |
| 129 | XP_034296606.1 | *Pantherophis guttatus* | 209.17 | 6 | 1 | 0.002% | 106.08 | Peptidase M1 |
| 130 | XP_034296607.1 | *Pantherophis guttatus* | 209.17 | 6 | 1 | 0.002% | 98.37 | Peptidase M1 |
| **Nerve growth factor (NGF): 0.02%** | | | | | | | | |
| 131 | AAB25729.1 | *Bungarus multicinctus* | 104.44 | 1 | 1 | 0.008% | 27.51 | NGF |
| 132 | P34128.1 | *Bungarus multicinctus* | 104.44 | 1 | 1 | 0.008% | 27.51 | NGF |
| **Hyaluronidase: 0.02%** | | | | | | | | |
| 133 | XP_026524834 | *Notechis scutatus* | 287.68 | 16 | 5 | 0.013% | 54.56 | Hyaluronidase |
| 134 | XP_034277309.1 | *Pantherophis guttatus* | 182.52 | 6 | 1 | 0.003% | 54.30 | Hyaluronidase |
| **L-amino-acid oxidase (LAAO): 0.01%** | | | | | | | | |
| 135 | ABN72539 | *Bungarus multicinctus* | 336.02 | 23 | 2 | 0.004% | 62.87 | LAAO |
| 136 | ABN72539 | *Bungarus multicinctus* | 336.02 | 23 | 2 | 0.004% | 62.87 | LAAO |
| 137 | ABN72540 | *Bungarus fasciatus* | 330.74 | 22 | 1 | 0.001% | 62.83 | LAAO |
| 138 | ABN72540 | *Bungarus fasciatus* | 330.74 | 22 | 1 | 0.001% | 62.83 | LAAO |
| **Phospholipase B (PLB): 0.005%** | | | | | | | | |
| 139 | ACR78473 | *Drysdalia coronoides* | 192.46 | 6 | 6 | 0.005% | 64.07 | PLB |
| **Snake venom serine protease: 0.004%** | | | | | | | | |
| 140 | ABN72545 | *Bungarus multicinctus* | 161.71 | 4 | 2 | 0.002% | 31.78 | Serine Protease |
| 141 | ABN72545.1 | *Bungarus multicinctus* | 158.73 | 3 | 1 | 0.001% | 31.01 | Serine Protease |
| 142 | A8QL57.1 | *Bungarus multicinctus* | 158.73 | 3 | 1 | 0.001% | 31.01 | Serine Protease |
| **C-type lectin (CTL): 0.003%** | | | | | | | | |
| 143 | ABH05181.1 | *Bungarus multicinctus* | 128.06 | 1 | 1 | 0.002% | 18.27 | CTL |
| 144 | A1XXJ9.1 | *Bungarus multicinctus* | 128.06 | 1 | 1 | 0.002% | 18.27 | CTL |
| **Vespryn: 0.002%** | | | | | | | | |
| 145 | XP_026576508 | *Pseudonaja textilis* | 118.47 | 2 | 2 | 0.002% | 24.38 | Vespryn |
| **Phosphodiesterase (PDE): 0.002%** | | | | | | | | |
| 146 | XP_026521896.1 | *Notechis scutatus* | 199.72 | 4 | 1 | 0.001% | 96.90 | PDE |
| 147 | XP_026521895.1 | *Notechis scutatus* | 199.72 | 4 | 1 | 0.001% | 100.96 | PDE |

**Table S7.** The proteomic composition of *B. caeruleus* venom.

| **Sr. No.** | **Accession** | **Species** | **-10lgP** | **#Peptides** | **#Unique** | **Relative Abundance of Toxin Hit (%)** | **Avg. Mass (KDa)** | **Family** |
| --- | --- | --- | --- | --- | --- | --- | --- | --- |
| **Three-finger toxins (3FTx): 65.54%** | | | | | | | | |
| 1 | 2CTX | *Naja naja* | 158.07 | 3 | 2 | 9.260% | 7.83 | Type II (long) α-neurotoxin |
| 2 | 1LXH | *Naja kaouthia* | 158.07 | 3 | 2 | 9.260% | 7.83 | Type II (long) α-neurotoxin |
| 3 | 1LXG | *Naja kaouthia* | 158.07 | 3 | 2 | 9.260% | 7.83 | Type II (long) α-neurotoxin |
| 4 | P01391.1 | *Naja kaouthia* | 158.07 | 3 | 2 | 9.260% | 7.83 | Type II (long) α-neurotoxin |
| 5 | 4AEA | *Naja kaouthia* | 158.07 | 3 | 2 | 9.260% | 7.83 | Type II (long) α-neurotoxin |
| 6 | 1CTX | *Naja siamensis* | 158.07 | 3 | 2 | 9.260% | 7.83 | Type II (long) α-neurotoxin |
| 7 | 1YI5 | *Naja siamensis* | 158.07 | 3 | 2 | 9.260% | 7.83 | Type II (long) α-neurotoxin |
| 8 | P86538.2 | *Naja naja* | 185.04 | 3 | 3 | 0.134% | 6.71 | Cytotoxin |
| 9 | BAU24666.1 | *Naja naja* | 185.04 | 3 | 3 | 0.134% | 7.96 | Cytotoxin |
| 10 | LAB55960 | *Micrurus surinamensis* | 134.85 | 3 | 3 | 0.120% | 10.09 | Non-conventional neurotoxin |
| 11 | 1IJC | *Bungarus candidus* | 80.02 | 1 | 1 | 0.038% | 7.29 | Non-conventional neurotoxin |
| 12 | 1F94 | *Bungarus candidus* | 80.02 | 1 | 1 | 0.038% | 7.29 | Non-conventional neurotoxin |
| 13 | P81782.1 | *Bungarus candidus* | 80.02 | 1 | 1 | 0.038% | 7.29 | Non-conventional neurotoxin |
| 14 | 5MG9 | *Dendroaspis angusticeps* | 82.53 | 1 | 1 | 0.026% | 7.24 | Type I (short) aminergic toxin |
| 15 | P82463.1 | *Naja kaouthia* | 82.53 | 1 | 1 | 0.026% | 7.30 | Type C muscarinic toxin |
| 16 | Q9W717.1 | *Naja atra* | 89.64 | 1 | 1 | 0.025% | 9.70 | Type I (short) orphan neurotoxin |
| 17 | APB88857.1 | *Naja naja* | 89.64 | 1 | 1 | 0.025% | 9.70 | Type I (short) orphan neurotoxin |
| 18 | CAB45156.1 | *Naja naja* | 89.64 | 1 | 1 | 0.025% | 9.70 | Type I (short) orphan neurotoxin |
| 19 | 764177A | *Naja naja* | 118.46 | 2 | 1 | 0.016% | 7.71 | Non-conventional neurotoxin |
| 20 | P25669.1 | P25669.1 | 118.46 | 2 | 1 | 0.016% | 7.82 | Type II (long) α-neurotoxin |
| 21 | AAL30061.1 | *Bungarus candidus* | 51.91 | 1 | 1 | 0.004% | 9.76 | Non-conventional neurotoxin |
| 22 | Q8AY49.1 | *Bungarus candidus* | 51.91 | 1 | 1 | 0.004% | 9.76 | Non-conventional neurotoxin |
| 23 | Q8AY51.1 | *Bungarus candidus* | 51.91 | 1 | 1 | 0.004% | 9.71 | Non-conventional neurotoxin |
| 24 | AAL30059.1 | *Bungarus candidus* | 51.91 | 1 | 1 | 0.004% | 9.71 | Non-conventional neurotoxin |
| 25 | A2CKF7.1 | *Bungarus fasciatus* | 51.91 | 1 | 1 | 0.004% | 9.72 | Non-conventional neurotoxin |
| 26 | CAA11133.1 | *Naja atra* | 51.91 | 1 | 1 | 0.004% | 9.85 | Non-conventional neurotoxin |
| 27 | ADF50021.1 | *Bungarus flaviceps* | 51.91 | 1 | 1 | 0.004% | 9.74 | Non-conventional neurotoxin |
| 28 | ABI33871.1 | *Bungarus fasciatus* | 51.91 | 1 | 1 | 0.004% | 9.72 | Non-conventional neurotoxin |
| 29 | AAT38875.1 | *Bungarus candidus* | 51.91 | 1 | 1 | 0.004% | 9.72 | Non-conventional neurotoxin |
| 30 | AAL30060.1 | *Bungarus candidus* | 51.91 | 1 | 1 | 0.004% | 9.65 | Non-conventional neurotoxin |
| 31 | Q8AY50.1 | *Bungarus candidus* | 51.91 | 1 | 1 | 0.004% | 9.65 | Non-conventional neurotoxin |
| 32 | Q9YGI2.1 | *Naja atra* | 51.91 | 1 | 1 | 0.004% | 9.85 | Non-conventional neurotoxin |
| 33 | ADF50022.1 | *Bungarus flaviceps* | 51.91 | 1 | 1 | 0.004% | 9.76 | Non-conventional neurotoxin |
| 34 | ADF50020.1 | *Bungarus flaviceps* | 51.91 | 1 | 1 | 0.004% | 9.68 | Type I (short) α-neurotoxin |
| 35 | ADN67577.1 | *Naja atra* | 51.91 | 1 | 1 | 0.004% | 9.86 | Non-conventional neurotoxin |
| 36 | CAA11134.1 | *Naja atra* | 51.91 | 1 | 1 | 0.004% | 9.74 | Non-conventional neurotoxin |
| 37 | Q6IZ95.1 | *Bungarus candidus* | 51.91 | 1 | 1 | 0.004% | 9.72 | Non-conventional neurotoxin |
| 38 | Q9YGI1.1 | *Naja atra* | 51.91 | 1 | 1 | 0.004% | 9.74 | Non-conventional neurotoxin |
| **Phospholipase A2 (PLA_2_): 10.5%** | | | | | | | | |
| 39 | 1U4J | *Bungarus caeruleus* | 194.87 | 5 | 1 | 1.340% | 12.97 | PLA2 |
| 40 | 1G0Z | *Bungarus caeruleus* | 194.87 | 5 | 1 | 1.340% | 12.97 | PLA2 |
| 41 | Q6SLM1.1 | *Bungarus caeruleus* | 194.87 | 5 | 1 | 1.340% | 14.83 | PLA2 |
| 42 | AAR19228.1 | *Bungarus caeruleus* | 194.87 | 5 | 1 | 1.340% | 14.83 | PLA2 |
| 43 | 1PSH | *Naja naja* | 167.87 | 4 | 3 | 0.523% | 13.35 | PLA2 |
| 44 | 1A3F | *Naja naja* | 167.87 | 4 | 3 | 0.523% | 13.35 | PLA2 |
| 45 | 1A3D | *Naja naja* | 167.87 | 4 | 3 | 0.523% | 13.35 | PLA2 |
| 46 | P15445.1 | *Naja naja* | 167.87 | 4 | 3 | 0.523% | 13.35 | PLA2 |
| 47 | CAA45372.1 | *Naja naja* | 167.87 | 4 | 3 | 0.523% | 13.48 | PLA2 |
| 48 | 0702209A | *Bungarus multicinctus* | 120.69 | 2 | 2 | 0.082% | 12.83 | PLA2 |
| 49 | P00606.2 | *Bungarus multicinctus* | 120.69 | 2 | 2 | 0.082% | 15.59 | PLA2 |
| 50 | CAA37482.1 | *Bungarus multicinctus* | 120.69 | 2 | 2 | 0.082% | 15.59 | PLA2 |
| 51 | JAB52792.1 | *Micrurus fulvius* | 190.96 | 5 | 1 | 0.077% | 16.16 | PLA2 |
| 52 | AAZ29513.1 | *Micrurus fulvius* | 190.96 | 5 | 1 | 0.077% | 15.33 | PLA2 |
| 53 | JAB52790.1 | *Micrurus fulvius* | 190.96 | 5 | 1 | 0.077% | 15.98 | PLA2 |
| 54 | JAB52794.1 | *Micrurus fulvius* | 190.96 | 5 | 1 | 0.077% | 16.03 | PLA2 |
| 55 | JAB52809.1 | *Micrurus fulvius* | 190.96 | 5 | 1 | 0.077% | 16.08 | PLA2 |
| 56 | JAB52771.1 | *Micrurus fulvius* | 190.96 | 5 | 1 | 0.077% | 16.21 | PLA2 |
| 57 | JAS04998.1 | *Micrurus fulvius* | 190.96 | 5 | 1 | 0.077% | 16.16 | PLA2 |
| 58 | JAB52773.1 | *Micrurus fulvius* | 190.96 | 5 | 1 | 0.077% | 16.22 | PLA2 |
| 59 | JAB52772.1 | *Micrurus fulvius* | 190.96 | 5 | 1 | 0.077% | 16.19 | PLA2 |
| 60 | JAS04995.1 | *Micrurus fulvius* | 190.96 | 5 | 1 | 0.077% | 16.18 | PLA2 |
| 61 | JAB52770.1 | *Micrurus fulvius* | 190.96 | 5 | 1 | 0.077% | 16.16 | PLA2 |
| 62 | JAB52795.1 | *Micrurus fulvius* | 190.96 | 5 | 1 | 0.077% | 16.24 | PLA2 |
| 63 | JAB52774.1 | *Micrurus fulvius* | 190.96 | 5 | 1 | 0.077% | 16.18 | PLA2 |
| 64 | JAS04999.1 | *Micrurus fulvius* | 190.96 | 5 | 1 | 0.077% | 16.18 | PLA2 |
| 65 | JAI09010.1 | *Micrurus fulvius* | 190.96 | 5 | 1 | 0.077% | 16.18 | PLA2 |
| 66 | JAI09006.1 | *Micrurus fulvius* | 190.96 | 5 | 1 | 0.077% | 16.18 | PLA2 |
| 67 | JAI09004.1 | *Micrurus fulvius* | 190.96 | 5 | 1 | 0.077% | 16.30 | PLA2 |
| 68 | JAI09005.1 | *Micrurus fulvius* | 190.96 | 5 | 1 | 0.077% | 16.24 | PLA2 |
| 69 | JAI09009.1 | *Micrurus fulvius* | 190.96 | 5 | 1 | 0.077% | 16.16 | PLA2 |
| 70 | JAS04993.1 | *Micrurus fulvius* | 190.96 | 5 | 1 | 0.077% | 16.30 | PLA2 |
| 71 | JAB52804.1 | *Micrurus fulvius* | 190.96 | 5 | 1 | 0.077% | 16.29 | PLA2 |
| 72 | JAS04994.1 | *Micrurus fulvius* | 190.96 | 5 | 1 | 0.077% | 16.24 | PLA2 |
| 73 | JAB52764.1 | *Micrurus fulvius* | 190.96 | 5 | 1 | 0.077% | 16.16 | PLA2 |
| 74 | LAA98189.1 | *Micrurus lemniscatus lemniscatus* | 190.96 | 5 | 1 | 0.077% | 11.26 | PLA2 |
| 75 | JAS04997.1 | *Micrurus fulvius* | 190.96 | 5 | 1 | 0.077% | 16.16 | PLA2 |
| 76 | JAI09008.1 | *Micrurus fulvius* | 190.96 | 5 | 1 | 0.077% | 16.16 | PLA2 |
| 77 | ABU68503.1 | *Trimorphodon biscutatus* | 190.96 | 5 | 1 | 0.077% | 16.62 | PLA2 |
| 78 | ABU63164.1 | *Bungarus fasciatus* | 192.65 | 5 | 1 | 0.034% | 15.95 | PLA2 |
| 79 | ABU63166.1 | *Bungarus fasciatus* | 192.65 | 5 | 1 | 0.034% | 16.00 | PLA2 |
| 80 | AAO84769.1 | *Bungarus candidus* | 115.37 | 2 | 2 | 0.032% | 15.64 | PLA2 |
| 81 | Q802I1.1 | *Bungarus candidus* | 115.37 | 2 | 2 | 0.032% | 15.64 | PLA2 |
| 82 | AAL54920.1 | *Hydrophis hardwickii* | 60.95 | 1 | 1 | 0.028% | 16.11 | PLA2 |
| 83 | ABN54807.1 | *Hydrophis hardwickii* | 60.95 | 1 | 1 | 0.028% | 16.08 | PLA2 |
| **β-bungarotoxin: 7.1%** | | | | | | | | |
| 84 | Q8QFW3.1 | *Bungarus caeruleus* | 237.26 | 6 | 5 | 3.246% | 16.12 | β-bungarotoxin |
| 85 | AAL87004.1 | *Bungarus caeruleus* | 237.26 | 6 | 5 | 3.246% | 16.12 | β-bungarotoxin |
| 86 | Q8QFW4.1 | *Bungarus caeruleus* | 204.23 | 4 | 3 | 0.302% | 16.36 | β-bungarotoxin |
| 87 | AAL87003.1 | *Bungarus caeruleus* | 204.23 | 4 | 3 | 0.302% | 16.36 | β-bungarotoxin |
| **Acetylcholinesterase (AChE): 5.24%** | | | | | | | | |
| 88 | Q92035 | *Bungarus fasciatus* | 432.47 | 40 | 18 | 5.092% | 68.17 | AChE |
| 89 | S68801 | *Bungarus fasciatus* | 221.06 | 5 | 1 | 0.153% | 9.79 | AChE |
| **L-amino-acid oxidase (LAAO): 3.96%** | | | | | | | | |
| 90 | 5Z2G | *Naja atra* | 381.05 | 22 | 18 | 1.085% | 57.96 | LAAO |
| 91 | AVX27607.1 | *Naja atra* | 381.05 | 22 | 18 | 1.085% | 57.96 | LAAO |
| 92 | A8QL58.2 | *Naja atra* | 381.05 | 22 | 18 | 1.085% | 57.96 | LAAO |
| 93 | ABN72539.1 | *Bungarus multicinctus* | 325.23 | 17 | 5 | 0.235% | 58.81 | LAAO |
| 94 | A8QL51.1 | *Bungarus multicinctus* | 325.23 | 17 | 5 | 0.235% | 58.81 | LAAO |
| 95 | ABN72539 | *Bungarus multicinctus* | 308.62 | 15 | 2 | 0.065% | 58.92 | LAAO |
| 96 | ABN72539 | *Bungarus multicinctus* | 308.62 | 15 | 2 | 0.065% | 66.29 | LAAO |
| 97 | P0DI91.1 | *Naja oxiana* | 203.51 | 4 | 2 | 0.049% | 11.22 | LAAO |
| 98 | AXL95288.1 | *Spilotes sulphureus* | 222.07 | 6 | 1 | 0.016% | 58.59 | LAAO |
| 99 | AXL95287.1 | *Spilotes sulphureus* | 222.07 | 6 | 1 | 0.016% | 58.59 | LAAO |
| 100 | JAA74761.1 | *Echiopsis curta* | 241.21 | 9 | 1 | 0.009% | 59.21 | LAAO |
| 101 | LAA54621.1 | *Micrurus corallinus* | 228.7 | 7 | 1 | 0.008% | 58.75 | LAAO |
| 102 | JAC94991.1 | *Opheodrys aestivus* | 125.19 | 3 | 1 | 0.002% | 40.49 | LAAO |
| **Cobra venom factor (CVF): 3.14%** | | | | | | | | |
| 103 | 6I2X | *Naja kaouthia* | 387.98 | 23 | 15 | 0.449% | 184.52 | CVF |
| 104 | 3PRX | *Naja kaouthia* | 387.98 | 23 | 15 | 0.449% | 184.52 | CVF |
| 105 | I51018 | *Naja kaouthia* | 387.98 | 23 | 15 | 0.449% | 184.52 | CVF |
| 106 | 3PVM | *Naja kaouthia* | 387.98 | 23 | 15 | 0.449% | 184.52 | CVF |
| 107 | AAA68989.1 | *Naja kaouthia* | 387.98 | 23 | 15 | 0.449% | 184.52 | CVF |
| 108 | Q91132.1 | *Naja kaouthia* | 387.98 | 23 | 15 | 0.449% | 184.52 | CVF |
| 109 | CAI46845.1 | *Naja naja* | 387.98 | 23 | 15 | 0.449% | 184.52 | CVF |
| **Snake venom metalloproteinase (Disintegrin-like): 2.47%** | | | | | | | | |
| 110 | ABN72537 | *Bungarus multicinctus* | 273.37 | 8 | 5 | 0.648% | 68.85 | Disintegrin-like |
| 111 | ABN72537 | *Bungarus multicinctus* | 273.37 | 8 | 5 | 0.648% | 72.61 | Disintegrin-like |
| 112 | ABN72537.1 | *Bungarus multicinctus* | 294.11 | 13 | 7 | 0.229% | 68.99 | Disintegrin-like |
| 113 | A8QL49.1 | *Bungarus multicinctus* | 294.11 | 13 | 7 | 0.229% | 68.99 | Disintegrin-like |
| 114 | P82942.1 | *Naja kaouthia* | 262.78 | 11 | 7 | 0.172% | 44.49 | Disintegrin-like |
| 115 | Q9PVK7.1 | *Naja kaouthia* | 228.65 | 7 | 3 | 0.093% | 67.66 | Disintegrin-like |
| 116 | AAF00693.1 | *Naja naja* | 228.65 | 7 | 3 | 0.093% | 67.66 | Disintegrin-like |
| 117 | ABN72537 | *Bungarus multicinctus* | 254.45 | 8 | 2 | 0.062% | 50.60 | Disintegrin-like |
| 118 | XP_013922279.1 | *Thamnophis sirtalis* | 56.85 | 1 | 1 | 0.028% | 18.22 | Disintegrin-like |
| 119 | XP_032084681.1 | *Thamnophis elegans* | 56.85 | 1 | 1 | 0.028% | 69.29 | Disintegrin-like |
| 120 | ADD14036.1 | *Naja atra* | 244.4 | 7 | 2 | 0.022% | 44.75 | Disintegrin-like |
| 121 | D6PXE8.1 | *Naja atra* | 244.4 | 7 | 2 | 0.022% | 66.25 | Disintegrin-like |
| 122 | ADG02948.1 | *Naja atra* | 244.4 | 7 | 2 | 0.022% | 66.25 | Disintegrin-like |
| 123 | 3K7N | *Naja atra* | 244.4 | 7 | 2 | 0.022% | 44.19 | Disintegrin-like |
| 124 | ACN50005.1 | *Naja atra* | 244.4 | 7 | 2 | 0.022% | 66.29 | Disintegrin-like |
| 125 | D3TTC1.1 | *Naja atra* | 244.4 | 7 | 2 | 0.022% | 66.29 | Disintegrin-like |
| 126 | D5LMJ3.1 | *Naja atra* | 116.03 | 2 | 2 | 0.017% | 68.25 | Disintegrin-like |
| 127 | ADF43026.1 | *Naja atra* | 116.03 | 2 | 2 | 0.017% | 68.25 | Disintegrin-like |
| 128 | JAC96437.1 | *Hypsiglena sp. JMG-2014* | 79.39 | 1 | 1 | 0.004% | 68.09 | Disintegrin-like |
| 129 | JAS04919.1 | *Hypsiglena sp. JMG-2014* | 79.39 | 1 | 1 | 0.004% | 68.09 | Disintegrin-like |
| 130 | JAS04923.1 | *Hypsiglena sp. JMG-2014* | 79.39 | 1 | 1 | 0.004% | 68.47 | Disintegrin-like |
| 131 | JAC96439.1 | *Hypsiglena sp. JMG-2014* | 79.39 | 1 | 1 | 0.004% | 68.51 | Disintegrin-like |
| 132 | JAS04921.1 | *Hypsiglena sp. JMG-2014* | 79.39 | 1 | 1 | 0.004% | 68.51 | Disintegrin-like |
| 133 | JAS04922.1 | *Hypsiglena sp. JMG-2014* | 79.39 | 1 | 1 | 0.004% | 68.49 | Disintegrin-like |
| 134 | JAC96441.1 | *Hypsiglena sp. JMG-2014* | 79.39 | 1 | 1 | 0.004% | 68.47 | Disintegrin-like |
| 135 | JAC96440.1 | *Hypsiglena sp. JMG-2014* | 79.39 | 1 | 1 | 0.004% | 68.49 | Disintegrin-like |
| 136 | AXL96635.1 | *Ahaetulla prasina* | 148.44 | 4 | 1 | 0.003% | 68.84 | Disintegrin-like |
| 137 | AXL96667.1 | *Ahaetulla prasina* | 148.44 | 4 | 1 | 0.003% | 68.81 | Disintegrin-like |
| 138 | AXL96646.1 | *Ahaetulla prasina* | 148.44 | 4 | 1 | 0.003% | 68.78 | Disintegrin-like |
| 139 | AXL96640.1 | *Ahaetulla prasina* | 148.44 | 4 | 1 | 0.003% | 68.85 | Disintegrin-like |
| 140 | AXL96666.1 | *Ahaetulla prasina* | 148.44 | 4 | 1 | 0.003% | 68.87 | Disintegrin-like |
| 141 | AXL96626.1 | *Ahaetulla prasina* | 148.44 | 4 | 1 | 0.003% | 72.96 | Disintegrin-like |
| 142 | AXL96631.1 | *Ahaetulla prasina* | 148.44 | 4 | 1 | 0.003% | 67.77 | Disintegrin-like |
| 143 | AXL96650.1 | *Ahaetulla prasina* | 148.44 | 4 | 1 | 0.003% | 71.88 | Disintegrin-like |
| 144 | AXL96645.1 | *Ahaetulla prasina* | 148.44 | 4 | 1 | 0.003% | 71.45 | Disintegrin-like |
| 145 | AXL96653.1 | *Ahaetulla prasina* | 148.44 | 4 | 1 | 0.003% | 68.11 | Disintegrin-like |
| 146 | AXL96648.1 | *Ahaetulla prasina* | 148.44 | 4 | 1 | 0.003% | 68.24 | Disintegrin-like |
| 147 | AXL96655.1 | *Ahaetulla prasina* | 148.44 | 4 | 1 | 0.003% | 68.22 | Disintegrin-like |
| 148 | AXL96638.1 | *Ahaetulla prasina* | 148.44 | 4 | 1 | 0.003% | 68.15 | Disintegrin-like |
| 149 | AXL96643.1 | *Ahaetulla prasina* | 148.44 | 4 | 1 | 0.003% | 68.28 | Disintegrin-like |
| 150 | AXL96644.1 | *Ahaetulla prasina* | 148.44 | 4 | 1 | 0.003% | 68.22 | Disintegrin-like |
| 151 | AXL96633.1 | *Ahaetulla prasina* | 148.44 | 4 | 1 | 0.003% | 68.17 | Disintegrin-like |
| **Peptidase S1: 0.62%** | | | | | | | | |
| 152 | XP_034291085.1 | *Pantherophis guttatus* | 222.57 | 7 | 3 | 0.144% | 63.13 | Peptidase S1 |
| 153 | XP_034291087.1 | *Pantherophis guttatus* | 222.57 | 7 | 3 | 0.144% | 63.13 | Peptidase S1 |
| 154 | XP_034291086.1 | *Pantherophis guttatus* | 222.57 | 7 | 3 | 0.144% | 63.13 | Peptidase S1 |
| 155 | XP_026544671.1 | *Notechis scutatus* | 202.44 | 5 | 1 | 0.078% | 60.66 | Peptidase S1 |
| 156 | XP_026570965.1 | *Pseudonaja textilis* | 202.44 | 5 | 1 | 0.078% | 64.11 | Peptidase S1 |
| 157 | ETE66745.1 | *Ophiophagus hannah* | 144.06 | 2 | 1 | 0.026% | 24.25 | Peptidase S1 |
| **Vascular endothelial growth factor (VEGF): 0.35%** | | | | | | | | |
| 158 | XP_026551346 | *Pseudonaja textilis* | 126.27 | 2 | 2 | 0.056% | 16.57 | VEGF |
| 159 | XP_026551346 | *Pseudonaja textilis* | 126.27 | 2 | 2 | 0.056% | 16.57 | VEGF |
| 160 | XP_026551346 | *Pseudonaja textilis* | 126.27 | 2 | 2 | 0.056% | 16.57 | VEGF |
| 161 | XP_026551346 | *Pseudonaja textilis* | 126.27 | 2 | 2 | 0.056% | 17.34 | VEGF |
| 162 | JAA74898 | *Pseudonaja modesta* | 126.27 | 2 | 2 | 0.056% | 22.46 | VEGF |
| 163 | XP_026551344 | *Pseudonaja textilis* | 126.27 | 2 | 2 | 0.056% | 25.39 | VEGF |
| 164 | XP_007425787.1 | *Python bivittatus* | 50.53 | 1 | 1 | 0.001% | 42.64 | VEGF |
| 165 | XP_034298589.1 | *Pantherophis guttatus* | 50.53 | 1 | 1 | 0.001% | 42.84 | VEGF |
| 166 | JAC96561.1 | *Echis coloratus* | 50.53 | 1 | 1 | 0.001% | 47.65 | VEGF |
| 167 | JAI10614.1 | *Crotalus adamanteus* | 50.53 | 1 | 1 | 0.001% | 47.70 | VEGF |
| 168 | SMD28268.1 | *Crotalus durissus terrificus* | 50.53 | 1 | 1 | 0.001% | 47.61 | VEGF |
| 169 | JAC95037.1 | *Pantherophis guttatus* | 50.53 | 1 | 1 | 0.001% | 47.51 | VEGF |
| 170 | JAV48556.1 | *Agkistrodon contortrix contortrix* | 50.53 | 1 | 1 | 0.001% | 47.65 | VEGF |
| 171 | JAA95033.1 | *Crotalus horridus* | 50.53 | 1 | 1 | 0.001% | 47.64 | VEGF |
| 172 | XP_034298588.1 | *Pantherophis guttatus* | 50.53 | 1 | 1 | 0.001% | 47.51 | VEGF |
| 173 | XP_013912093.1 | *Thamnophis sirtalis* | 50.53 | 1 | 1 | 0.001% | 47.48 | VEGF |
| 174 | ASU45066.1 | *Daboia russelii* | 50.53 | 1 | 1 | 0.001% | 47.70 | VEGF |
| 175 | JAC94939.1 | *Python regius* | 50.53 | 1 | 1 | 0.001% | 47.54 | VEGF |
| 176 | XP_029139565.1 | *Protobothrops mucrosquamatus* | 50.53 | 1 | 1 | 0.001% | 47.71 | VEGF |
| 177 | JAC94973.1 | *Opheodrys aestivus* | 50.53 | 1 | 1 | 0.001% | 40.06 | VEGF |
| 178 | XP_034298590.1 | *Pantherophis guttatus* | 50.53 | 1 | 1 | 0.001% | 40.06 | VEGF |
| 179 | JAI08988.1 | *Micrurus fulvius* | 50.53 | 1 | 1 | 0.001% | 47.56 | VEGF |
| 180 | ETE60014.1 | *Ophiophagus hannah* | 50.53 | 1 | 1 | 0.001% | 47.56 | VEGF |
| 181 | JAB52939.1 | *Micrurus fulvius* | 50.53 | 1 | 1 | 0.001% | 47.56 | VEGF |
| 182 | LAB17921 | *Micrurus spixii* | 50.53 | 1 | 1 | 0.001% | 47.59 | VEGF |
| 183 | JAS04977.1 | *Micrurus fulvius* | 50.53 | 1 | 1 | 0.001% | 47.56 | VEGF |
| 184 | XP_026522979.1 | *Notechis scutatus* | 50.53 | 1 | 1 | 0.001% | 47.53 | VEGF |
| 185 | XP_026555459.1 | *Pseudonaja textilis* | 50.53 | 1 | 1 | 0.001% | 47.62 | VEGF |
| 186 | JAI09156.1 | *Micrurus fulvius* | 50.53 | 1 | 1 | 0.001% | 47.56 | VEGF |
| 187 | LAA17506.1 | *Micrurus lemniscatus carvalhoi* | 50.53 | 1 | 1 | 0.001% | 53.37 | VEGF |
| 188 | LAB17921.1 | *Micrurus spixii* | 50.53 | 1 | 1 | 0.001% | 53.46 | VEGF |
| 189 | XP_026555459 | *Pseudonaja textilis* | 50.53 | 1 | 1 | 0.001% | 32.63 | VEGF |
| 190 | LAB17920.1 | *Micrurus spixii* | 50.53 | 1 | 1 | 0.001% | 32.58 | VEGF |
| 191 | LAB17921 | *Micrurus spixii* | 50.53 | 1 | 1 | 0.001% | 40.13 | VEGF |
| **Cysteine-rich secretory proteins (CRISP): 0.32%** | | | | | | | | |
| 192 | ACE73577 | *Bungarus candidus* | 151.6 | 2 | 2 | 0.159% | 26.29 | CRISP |
| 193 | ACE73577 | *Bungarus candidus* | 151.6 | 2 | 2 | 0.159% | 26.29 | CRISP |
| **5’-nucleotidase: 0.19%** | | | | | | | | |
| 194 | JAS05143.1 | *Micrurus tener* | 290.68 | 14 | 4 | 0.021% | 63.01 | 5'-nucleotidase |
| 195 | JAI09048.1 | *Micrurus fulvius* | 290.68 | 14 | 4 | 0.021% | 63.03 | 5'-nucleotidase |
| 196 | JAS05036.1 | *Micrurus fulvius* | 290.68 | 14 | 4 | 0.021% | 62.98 | 5'-nucleotidase |
| 197 | JAI09049.1 | *Micrurus fulvius* | 290.68 | 14 | 4 | 0.021% | 63.03 | 5'-nucleotidase |
| 198 | JAS05038.1 | *Micrurus fulvius* | 290.68 | 14 | 4 | 0.021% | 63.03 | 5'-nucleotidase |
| 199 | JAS05037.1 | *Micrurus fulvius* | 290.68 | 14 | 4 | 0.021% | 63.03 | 5'-nucleotidase |
| 200 | JAI09047.1 | *Micrurus fulvius* | 290.68 | 14 | 4 | 0.021% | 62.98 | 5'-nucleotidase |
| 201 | JAB52815.1 | *Micrurus fulvius* | 290.68 | 14 | 4 | 0.021% | 62.98 | 5'-nucleotidase |
| 202 | XP_026581084 | *Pseudonaja textilis* | 143.89 | 3 | 1 | 0.010% | 17.09 | 5'-nucleotidase |
| 203 | AXL95273.1 | *Spilotes sulphureus* | 164.25 | 5 | 1 | 0.003% | 64.54 | 5'-nucleotidase |
| 204 | JAG67188.1 | *Boiga irregularis* | 164.25 | 5 | 1 | 0.003% | 64.76 | 5'-nucleotidase |
| 205 | B6EWW8.1 | *Gloydius brevicaudus* | 236.58 | 9 | 1 | 0.001% | 64.43 | 5'-nucleotidase |
| 206 | BAG82602.1 | *Gloydius brevicaudus* | 236.58 | 9 | 1 | 0.001% | 64.43 | 5'-nucleotidase |
| **Snake venom serine protease: 0.18%** | | | | | | | | |
| 207 | A8QL56.1 | *Ophiophagus hannah* | 144.06 | 2 | 1 | 0.026% | 28.66 | Serine Protease |
| 208 | ABN72544.1 | *Ophiophagus hannah* | 144.06 | 2 | 1 | 0.026% | 28.66 | Serine Protease |
| 209 | ABI74694.1 | *Philodryas olfersii* | 144.06 | 2 | 1 | 0.026% | 28.45 | Serine Protease |
| 210 | Q09GK1.1 | *Philodryas olfersii* | 144.06 | 2 | 1 | 0.026% | 28.45 | Serine Protease |
| 211 | AJB84504.1 | *Philodryas chamissonis* | 144.06 | 2 | 1 | 0.026% | 28.57 | Serine Protease |
| 212 | ABN72545.1 | *Bungarus multicinctus* | 184.12 | 4 | 3 | 0.024% | 31.01 | Serine Protease |
| 213 | A8QL57.1 | *Bungarus multicinctus* | 184.12 | 4 | 3 | 0.024% | 31.01 | Serine Protease |
| **Kunitz-type serine protease inhibitor (Kunitz): 0.16%** | | | | | | | | |
| 214 | 1702215A | *Naja naja* | 93.85 | 2 | 2 | 0.067% | 6.51 | Kunitz |
| 215 | P19859.1 | *Naja naja* | 93.85 | 2 | 2 | 0.067% | 6.51 | Kunitz |
| 216 | ABN72545 | *Bungarus multicinctus* | 184.12 | 4 | 3 | 0.024% | 31.55 | Kunitz |
| **Vespryn: 0.12%** | | | | | | | | |
| 217 | P82885.1 | *Naja kaouthia* | 153.4 | 3 | 3 | 0.117% | 12.04 | Vespryn |
| **Phosphodiesterase (PDE): 0.1%** | | | | | | | | |
| 218 | 5GZ4 | *Naja atra* | 241.28 | 8 | 8 | 0.034% | 94.62 | PDE |
| 219 | 5GZ5 | *Naja atra* | 241.28 | 8 | 8 | 0.034% | 94.62 | PDE |
| 220 | A0A2D0TC04.1 | *Naja atra* | 241.28 | 8 | 8 | 0.034% | 94.62 | PDE |
| **Hyaluronidase: 0.02%** | | | | | | | | |
| 221 | XP_013928511.1 | *Thamnophis sirtalis* | 141.83 | 4 | 1 | 0.010% | 52.12 | Hyaluronidase |
| 222 | XP_032077741.1 | *Thamnophis elegans* | 141.83 | 4 | 1 | 0.010% | 58.84 | Hyaluronidase |
| 223 | XP_026524834 | *Notechis scutatus* | 200.89 | 6 | 1 | 0.001% | 54.31 | Hyaluronidase |
| 224 | XP_026524834 | *Notechis scutatus* | 200.89 | 6 | 1 | 0.001% | 54.31 | Hyaluronidase |
| **Phospholipase B (PLB): 0.001%** | | | | | | | | |
| 225 | JAC94989.1 | *Opheodrys aestivus* | 86.74 | 2 | 1 | 0.001% | 63.91 | PLB |

**Table S8.** The proteomic composition of *B. romulusi* venom.

| **Sr. No.** | **Accession** | **Species** | **−10lgP** | **#Peptides** | **#Unique** | **Relative Abundance of Toxin Hit (%)** | **Avg. Mass (KDa)** | **Family** |
| --- | --- | --- | --- | --- | --- | --- | --- | --- |
| **Phospholipase A2 (PLA_2_): 42.34%** | | | | | | | | |
| 1 | 0702209A | *Bungarus multicinctus* | 331.88 | 41 | 15 | 9.055% | 11.39 | PLA2 |
| 2 | ADF50037 | *Bungarus flaviceps* | 322.68 | 30 | 4 | 5.453% | 19.18 | PLA2 |
| 3 | AAR19228 | *Bungarus caeruleus* | 312.93 | 27 | 19 | 3.512% | 13.52 | PLA2 |
| 4 | ADF50037 | *Bungarus flaviceps* | 221.41 | 9 | 6 | 3.347% | 19.32 | PLA2 |
| 5 | ADF50037 | *Bungarus flaviceps* | 221.41 | 9 | 6 | 3.347% | 19.36 | PLA2 |
| 6 | 1PO8 | *Bungarus caeruleus* | 199.58 | 9 | 5 | 3.009% | 13.20 | PLA2 |
| 7 | 1TC8 | *Bungarus caeruleus* | 199.58 | 9 | 5 | 3.009% | 13.20 | PLA2 |
| 8 | AAS20530.1 | *Bungarus caeruleus* | 199.58 | 9 | 5 | 3.009% | 15.06 | PLA2 |
| 9 | Q6SLM2.1 | *Bungarus caeruleus* | 199.58 | 9 | 5 | 3.009% | 15.06 | PLA2 |
| 10 | CAA37482 | *Bungarus multicinctus* | 188.15 | 7 | 6 | 2.602% | 10.07 | PLA2 |
| 11 | 0702209A | *Bungarus multicinctus* | 328.36 | 37 | 15 | 2.341% | 11.32 | PLA2 |
| 12 | CAA37482 | *Bungarus multicinctus* | 337.39 | 36 | 9 | 0.240% | 11.18 | PLA2 |
| 13 | Q9DF52.1 | *Bungarus caeruleus* | 71.14 | 2 | 1 | 0.147% | 15.77 | PLA2 |
| 14 | AAG13412.1 | *Bungarus caeruleus* | 71.14 | 2 | 1 | 0.147% | 15.77 | PLA2 |
| 15 | ABU63164.1 | *Bungarus fasciatus* | 226.51 | 8 | 2 | 0.047% | 15.95 | PLA2 |
| 16 | ABU63166.1 | *Bungarus fasciatus* | 226.51 | 8 | 2 | 0.047% | 16.00 | PLA2 |
| 17 | P0C551 | *Bungarus candidus* | 186.46 | 8 | 4 | 0.020% | 20.10 | PLA2 |
| **Acetylcholinesterase (AChE): 36.58%** | | | | | | | | |
| 18 | AAC59905 | *Bungarus fasciatus* | 391.12 | 60 | 45 | 7.316% | 63.95 | AChE |
| 19 | AAC59905 | *Bungarus fasciatus* | 391.12 | 60 | 45 | 7.316% | 64.77 | AChE |
| 20 | AAC59905 | *Bungarus fasciatus* | 391.12 | 60 | 45 | 7.316% | 64.77 | AChE |
| 21 | AAC59905 | *Bungarus fasciatus* | 391.12 | 60 | 45 | 7.316% | 64.77 | AChE |
| 22 | AAC59905 | *Bungarus fasciatus* | 391.12 | 60 | 45 | 7.316% | 64.77 | AChE |
| 23 | XP_025032622.1 | *Python bivittatus* | 115.41 | 3 | 1 | 0.001% | 67.50 | AChE |
| 24 | XP_025032620.1 | *Python bivittatus* | 115.41 | 3 | 1 | 0.001% | 67.50 | AChE |
| 25 | XP_025032621.1 | *Python bivittatus* | 115.41 | 3 | 1 | 0.001% | 67.50 | AChE |
| 26 | JAC94932.1 | *Python regius* | 115.41 | 3 | 1 | 0.001% | 69.04 | AChE |
| 27 | XP_025032619.1 | *Python bivittatus* | 115.41 | 3 | 1 | 0.001% | 72.05 | AChE |
| **Three-finger toxins (3FTx): 9.96%** | | | | | | | | |
| 28 | AAD41806 | *Bungarus multicinctus* | 274.4 | 16 | 16 | 8.604% | 9.90 | Type-X α-neurotoxin |
| 29 | ETE56933 | *Ophiophagus hannah* | 300.21 | 23 | 23 | 0.849% | 15.87 | Type-X α-neurotoxin |
| 30 | AAD41806 | *Bungarus multicinctus* | 211.53 | 9 | 9 | 0.345% | 9.83 | Type-X α-neurotoxin |
| 31 | AAL30059 | *Bungarus candidus* | 182.89 | 6 | 6 | 0.082% | 9.84 | Type II (long) α-neurotoxin |
| 32 | CAA06887 | *Bungarus multicinctus multicinctus* | 138.09 | 2 | 2 | 0.015% | 11.25 | Type I (short) α-neurotoxin |
| 33 | AAL30054.1 | *Bungarus candidus* | 67.87 | 1 | 1 | 0.011% | 9.58 | Type II (long) κ-neurotoxin |
| 34 | Q8AY56.1 | *Bungarus candidus* | 67.87 | 1 | 1 | 0.011% | 9.58 | Type II (long) κ-neurotoxin |
| 35 | AAC83997.1 | *Bungarus multicinctus* | 56.12 | 1 | 1 | 0.001% | 7.98 | Type II (long) α-neurotoxin |
| 36 | AAC83990.1 | *Bungarus multicinctus* | 56.12 | 1 | 1 | 0.001% | 8.02 | Type II (long) α-neurotoxin |
| 37 | P01385.1 | *Acanthophis antarcticus* | 56.12 | 1 | 1 | 0.001% | 8.14 | Type II (long) α-neurotoxin |
| 38 | 0512217A | *Notechis scutatus* | 56.12 | 1 | 1 | 0.001% | 8.06 | Type II (long) α-neurotoxin |
| 39 | AAC83992.1 | *Bungarus multicinctus* | 56.12 | 1 | 1 | 0.001% | 7.99 | Type II (long) α-neurotoxin |
| 40 | 1HAJ | *Bungarus multicinctus* | 56.12 | 1 | 1 | 0.001% | 7.99 | Type II (long) α-neurotoxin |
| 41 | 1IDH | *Bungarus multicinctus* | 56.12 | 1 | 1 | 0.001% | 7.99 | Type II (long) α-neurotoxin |
| 42 | 2BTX | *Bungarus multicinctus* | 56.12 | 1 | 1 | 0.001% | 7.99 | Type II (long) α-neurotoxin |
| 43 | 1IK8 | *Bungarus multicinctus* | 56.12 | 1 | 1 | 0.001% | 7.99 | Type II (long) α-neurotoxin |
| 44 | 1BXP | *Bungarus multicinctus* | 56.12 | 1 | 1 | 0.001% | 7.99 | Type II (long) α-neurotoxin |
| 45 | 1ABT | *Bungarus multicinctus* | 56.12 | 1 | 1 | 0.001% | 7.99 | Type II (long) α-neurotoxin |
| 46 | 1IKC | *Bungarus multicinctus* | 56.12 | 1 | 1 | 0.001% | 7.99 | Type II (long) α-neurotoxin |
| 47 | AAC83994.1 | *Bungarus multicinctus* | 56.12 | 1 | 1 | 0.001% | 7.99 | Type II (long) α-neurotoxin |
| 48 | AAC83991.1 | *Bungarus multicinctus* | 56.12 | 1 | 1 | 0.001% | 7.99 | Type II (long) α-neurotoxin |
| 49 | 1IDL | *Bungarus multicinctus* | 56.12 | 1 | 1 | 0.001% | 7.99 | Type II (long) α-neurotoxin |
| 50 | 1HOY | *Bungarus multicinctus* | 56.12 | 1 | 1 | 0.001% | 8.00 | Type II (long) α-neurotoxin |
| 51 | AAC83993.1 | *Bungarus multicinctus* | 56.12 | 1 | 1 | 0.001% | 7.99 | Type II (long) α-neurotoxin |
| 52 | 720920A | *Bungarus multicinctus* | 56.12 | 1 | 1 | 0.001% | 7.99 | Type II (long) α-neurotoxin |
| 53 | 2ABX | *Bungarus multicinctus* | 56.12 | 1 | 1 | 0.001% | 7.99 | Type II (long) α-neurotoxin |
| 54 | CAM11311.1 | *Bungarus candidus* | 56.12 | 1 | 1 | 0.001% | 8.26 | Type II (long) α-neurotoxin |
| 55 | ADN67593.1 | *Bungarus multicinctus* | 56.12 | 1 | 1 | 0.001% | 8.26 | Type II (long) α-neurotoxin |
| 56 | CAM11305.1 | *Bungarus candidus* | 56.12 | 1 | 1 | 0.001% | 8.26 | Type II (long) α-neurotoxin |
| 57 | CAM11309.1 | *Bungarus candidus* | 56.12 | 1 | 1 | 0.001% | 8.26 | Type II (long) α-neurotoxin |
| 58 | CAD92407.1 | *Bungarus candidus* | 56.12 | 1 | 1 | 0.001% | 8.26 | Type II (long) α-neurotoxin |
| 59 | CAM11312.1 | *Bungarus candidus* | 56.12 | 1 | 1 | 0.001% | 8.26 | Type II (long) α-neurotoxin |
| 60 | CAM11307.1 | *Bungarus multicinctus* | 56.12 | 1 | 1 | 0.001% | 8.26 | Type II (long) α-neurotoxin |
| 61 | CAM11310.1 | *Bungarus candidus* | 56.12 | 1 | 1 | 0.001% | 8.26 | Type II (long) α-neurotoxin |
| 62 | CAM11304.1 | *Bungarus candidus* | 56.12 | 1 | 1 | 0.001% | 8.26 | Type II (long) α-neurotoxin |
| 63 | CAM11302.1 | *Bungarus caeruleus* | 56.12 | 1 | 1 | 0.001% | 8.31 | Type II (long) α-neurotoxin |
| 64 | D2N116.1 | *Bungarus caeruleus* | 56.12 | 1 | 1 | 0.001% | 8.31 | Type II (long) α-neurotoxin |
| 65 | CAM11303.1 | *Bungarus candidus* | 56.12 | 1 | 1 | 0.001% | 8.23 | Type II (long) α-neurotoxin |
| 66 | CAM11306.1 | *Bungarus candidus* | 56.12 | 1 | 1 | 0.001% | 8.26 | Type II (long) α-neurotoxin |
| 67 | Q8UW29.1 | *Hydrophis hardwickii* | 56.12 | 1 | 1 | 0.001% | 10.22 | Type II (long) α-neurotoxin |
| 68 | AAL54892.1 | *Hydrophis hardwickii* | 56.12 | 1 | 1 | 0.001% | 10.22 | Type II (long) α-neurotoxin |
| 69 | A3FM53.1 | *Hydrophis hardwickii* | 56.12 | 1 | 1 | 0.001% | 10.41 | Type II (long) α-neurotoxin |
| 70 | ABN54806.1 | *Hydrophis hardwickii* | 56.12 | 1 | 1 | 0.001% | 10.41 | Type II (long) α-neurotoxin |
| 71 | JAA74770.1 | *Echiopsis curta* | 56.12 | 1 | 1 | 0.001% | 10.26 | Type II (long) α-neurotoxin |
| 72 | P01384.2 | *Notechis scutatus scutatus* | 56.12 | 1 | 1 | 0.001% | 10.29 | Type II (long) α-neurotoxin |
| 73 | ABK63537.1 | *Notechis scutatus* | 56.12 | 1 | 1 | 0.001% | 10.29 | Type II (long) α-neurotoxin |
| 74 | JAA74654.1 | *Acanthophis wellsi* | 56.12 | 1 | 1 | 0.001% | 10.39 | Type II (long) α-neurotoxin |
| 75 | AAC83981.1 | *Bungarus multicinctus* | 56.12 | 1 | 1 | 0.001% | 10.29 | Type II (long) α-neurotoxin |
| 76 | AAL30056.1 | *Bungarus candidus* | 56.12 | 1 | 1 | 0.001% | 10.29 | Type II (long) α-neurotoxin |
| **Snake venom metalloproteinase (Disintegrin-like): 6.38%** | | | | | | | | |
| 77 | ABN72537 | *Bungarus multicinctus* | 201.08 | 11 | 8 | 2.626% | 14.13 | Disintegrin-like |
| 78 | ABN72537 | *Bungarus multicinctus* | 201.08 | 11 | 8 | 2.626% | 17.02 | Disintegrin-like |
| 79 | ABN72537 | *Bungarus multicinctus* | 364.17 | 48 | 8 | 0.773% | 66.30 | Disintegrin-like |
| 80 | ABQ01136 | *Oxyuranus scutellatus* | 180.6 | 7 | 5 | 0.239% | 37.26 | Disintegrin-like |
| 81 | ABN72536 | *Bungarus fasciatus* | 179.26 | 8 | 8 | 0.068% | 17.07 | Disintegrin-like |
| 82 | ABQ01138 | *Notechis scutatus* | 239.39 | 15 | 13 | 0.043% | 21.92 | Disintegrin-like |
| **β-bungarotoxin: 3.85%** | | | | | | | | |
| 83 | Q8QFW3.1 | *Bungarus caeruleus* | 269.83 | 18 | 7 | 1.130% | 16.12 | β-bungarotoxin |
| 84 | AAL87004.1 | *Bungarus caeruleus* | 269.83 | 18 | 7 | 1.130% | 16.12 | β-bungarotoxin |
| 85 | Q8QFW4.1 | *Bungarus caeruleus* | 306.09 | 24 | 17 | 0.724% | 16.36 | β-bungarotoxin |
| 86 | AAL87003.1 | *Bungarus caeruleus* | 306.09 | 24 | 17 | 0.724% | 16.36 | β-bungarotoxin |
| 87 | ABU63164 | *Bungarus fasciatus* | 278.54 | 17 | 2 | 0.122% | 14.06 | β-bungarotoxin |
| 88 | ABG90492.1 | *Bungarus multicinctus* | 289.92 | 21 | 1 | 0.020% | 13.16 | β-bungarotoxin |
| **L-amino-acid oxidase (LAAO): 0.38%** | | | | | | | | |
| 89 | ABN72539 | *Bungarus multicinctus* | 222.97 | 13 | 3 | 0.116% | 47.27 | LAAO |
| 90 | ABN72539 | *Bungarus multicinctus* | 222.97 | 13 | 3 | 0.116% | 47.27 | LAAO |
| 91 | ABN72539 | *Bungarus multicinctus* | 222.97 | 13 | 3 | 0.116% | 47.27 | LAAO |
| 92 | ABN72539.1 | *Bungarus multicinctus* | 232.45 | 12 | 2 | 0.018% | 58.81 | LAAO |
| 93 | A8QL51.1 | *Bungarus multicinctus* | 232.45 | 12 | 2 | 0.018% | 58.81 | LAAO |
| **Vascular endothelial growth factor (VEGF): 0.26%** | | | | | | | | |
| 94 | XP_026551346 | *Pseudonaja textilis* | 178.85 | 9 | 9 | 0.041% | 16.47 | VEGF |
| 95 | XP_026551346 | *Pseudonaja textilis* | 178.85 | 9 | 9 | 0.041% | 16.47 | VEGF |
| 96 | XP_026551346 | *Pseudonaja textilis* | 178.85 | 9 | 9 | 0.041% | 16.47 | VEGF |
| 97 | XP_026551346 | *Pseudonaja textilis* | 178.85 | 9 | 9 | 0.041% | 17.24 | VEGF |
| 98 | JAA74898 | *Pseudonaja modesta* | 178.85 | 9 | 9 | 0.041% | 22.36 | VEGF |
| 99 | JAA74898 | *Pseudonaja modesta* | 178.85 | 9 | 9 | 0.041% | 22.36 | VEGF |
| 100 | XP_026555459 | *Pseudonaja textilis* | 137.59 | 5 | 5 | 0.004% | 32.63 | VEGF |
| 101 | LAB17921 | *Micrurus spixii* | 137.59 | 5 | 5 | 0.004% | 47.59 | VEGF |
| 102 | LAB17921 | *Micrurus spixii* | 137.59 | 5 | 5 | 0.004% | 47.59 | VEGF |
| **5’-nucleotidase: 0.11%** | | | | | | | | |
| 103 | JAB52815 | *Micrurus fulvius* | 343.67 | 35 | 28 | 0.105% | 64.64 | 5'-nucleotidase |
| **Peptidase S1: 0.07%** | | | | | | | | |
| 104 | XP_026555558.1 | *Pseudonaja textilis* | 189.93 | 9 | 9 | 0.063% | 61.35 | Peptidase S1 |
| 105 | ETE73401.1 | *Ophiophagus hannah* | 100.63 | 2 | 2 | 0.001% | 138.99 | Peptidase S1 |
| 106 | XP_026580326.1 | *Pseudonaja textilis* | 100.63 | 2 | 2 | 0.001% | 39.29 | Peptidase S1 |
| 107 | JAS05177.1 | *Micrurus tener* | 100.63 | 2 | 2 | 0.001% | 48.12 | Peptidase S1 |
| 108 | JAI09074.1 | *Micrurus fulvius* | 100.63 | 2 | 2 | 0.001% | 48.12 | Peptidase S1 |
| 109 | JAS05063.1 | *Micrurus fulvius* | 100.63 | 2 | 2 | 0.001% | 48.12 | Peptidase S1 |
| 110 | JAB54462.1 | *Micrurus fulvius* | 100.63 | 2 | 2 | 0.001% | 48.12 | Peptidase S1 |
| 111 | XP_026548111.1 | *Notechis scutatus* | 100.63 | 2 | 2 | 0.001% | 44.79 | Peptidase S1 |
| **Hyaluronidase: 0.04%** | | | | | | | | |
| 112 | XP_026524834 | *Notechis scutatus* | 334.73 | 32 | 13 | 0.043% | 54.36 | Hyaluronidase |
| **Cysteine-rich secretory proteins (CRISP): 0.02%** | | | | | | | | |
| 113 | ACE73577.1 | *Bungarus candidus* | 69.57 | 1 | 1 | 0.001% | 26.46 | CRISP |
| 114 | ACE73578.1 | *Bungarus candidus* | 69.57 | 1 | 1 | 0.001% | 26.44 | CRISP |
| 115 | ACE73577 | *Bungarus candidus* | 69.57 | 1 | 1 | 0.001% | 26.38 | CRISP |
| 116 | ACE73577 | *Bungarus candidus* | 69.57 | 1 | 1 | 0.001% | 26.38 | CRISP |
| 117 | ACE73577 | *Bungarus candidus* | 69.57 | 1 | 1 | 0.001% | 26.38 | CRISP |
| 118 | ACE73577 | *Bungarus candidus* | 69.57 | 1 | 1 | 0.001% | 26.38 | CRISP |
| 119 | ACE73577 | *Bungarus candidus* | 69.57 | 1 | 1 | 0.001% | 27.81 | CRISP |
| 120 | ACE73577 | *Bungarus candidus* | 69.57 | 1 | 1 | 0.001% | 31.26 | CRISP |
| 121 | P81993.1 | *Bungarus candidus* | 69.57 | 1 | 1 | 0.001% | 7.75 | CRISP |
| 122 | ACE73577 | *Bungarus candidus* | 69.57 | 1 | 1 | 0.001% | 19.40 | CRISP |
| 123 | ACN93671.1 | *Ophiophagus hannah* | 69.57 | 1 | 1 | 0.001% | 26.31 | CRISP |
| 124 | ACH73168.1 | *Naja kaouthia* | 69.57 | 1 | 1 | 0.001% | 26.22 | CRISP |
| 125 | AAP20603.1 | *Naja atra* | 69.57 | 1 | 1 | 0.001% | 26.25 | CRISP |
| 126 | Q7ZZN8.1 | *Naja atra* | 69.57 | 1 | 1 | 0.001% | 26.25 | CRISP |
| 127 | P84808.2 | *Naja kaouthia* | 69.57 | 1 | 1 | 0.001% | 26.22 | CRISP |
| 128 | AHZ08822.1 | *Micropechis ikaheca* | 69.57 | 1 | 1 | 0.001% | 26.22 | CRISP |
| 129 | XP_032069593.1 | *Thamnophis elegans* | 69.57 | 1 | 1 | 0.001% | 26.60 | CRISP |
| 130 | JAC94992.1 | *Opheodrys aestivus* | 69.57 | 1 | 1 | 0.001% | 26.75 | CRISP |
| **Phospholipase B (PLB): 0.01%** | | | | | | | | |
| 131 | ACR78473 | *Drysdalia coronoides* | 104.9 | 2 | 2 | 0.004% | 64.04 | PLB |
| 132 | ACR78473 | *Drysdalia coronoides* | 104.9 | 2 | 2 | 0.004% | 64.04 | PLB |

**Table S9**. Tissue transcriptome sequencing and assembly.

|  | **Reads** | | | | **Assembly** | |
| --- | --- | --- | --- | --- | --- | --- |
| **Tissue** | **Raw** | **Filtered** | **Expressed** | **Annotated** | **Transcripts** | **N50** |
| ***B. sindanus*** | | | | | | |
| Venom gland | 34,952,562 | 33,332,796 | 27,599,732 | 54,793 | 229,763 | 1,436 |
| Intestine | 12,058,000 | 10,740,803 | 9,153,810 |  |  |  |
| ***B. caeruleus*** | | | | | | |
| Venom gland | 51,842,239 | 46,397,238 | 39,446,271 | 98,441 | 308,570 | 1,701 |
| Intestine | 55,336,796 | 51,104,996 | 43,471,869 |  |  |  |
| ***B. romulusi*** | | | | | | |
| Venom gland | 24,343,212 | 21,256,495 | 16,583,652 | 58,007 | 225,522 | 1,863 |
| Intestine | 28,413,016 | 24,882,567 | 21,853,659 |  |  |  |

.

**Table 10*.*** *Bungarus sindanus*

| S. No. | **Isoform** | **VG** | **Int** | **M** | **Family** | **Subtype** | **Abundance** | **Accession** | **Scientific name** |
| --- | --- | --- | --- | --- | --- | --- | --- | --- | --- |
| 1 | TRINITY_DN526_c0_g1_i10 | 5667 | 0 | 5.2821 | 5'-nucleotidase |  | **0.23%** | JAB52815 | *Micrurus fulvius* |
| 2 | TRINITY_DN526_c0_g1_i36 | 4729 | 0 | 5.2821 | 5'-nucleotidase |  |  | JAB52815 | *Micrurus fulvius* |
| 3 | TRINITY_DN526_c0_g1_i28 | 2599 | 6 | 5.2821 | 5'-nucleotidase |  |  | XP_013918203 | *Thamnophis sirtalis* |
| 4 | TRINITY_DN526_c0_g1_i8 | 1990 | 0 | 5.2821 | 5'-nucleotidase |  |  | JAB52815 | *Micrurus fulvius* |
| 5 | TRINITY_DN526_c0_g1_i22 | 690 | 62 | 5.2821 | 5'-nucleotidase |  |  | JAB52815 | *Micrurus fulvius* |
| 6 | TRINITY_DN526_c0_g1_i9 | 356 | 11 | 5.2821 | 5'-nucleotidase |  |  | XP_026582322 | *Pseudonaja textilis* |
| 7 | TRINITY_DN1357_c0_g1_i9 | 575 | 22 | 2.2921 | 5'-nucleotidase |  |  | XP_026557747 | *Pseudonaja textilis* |
| 8 | TRINITY_DN1357_c0_g1_i15 | 194 | 0 | 2.2921 | 5'-nucleotidase |  |  | XP_026557747 | *Pseudonaja textilis* |
| 9 | TRINITY_DN3400_c0_g1_i4 | 614 | 3 | 3.9753 | 5'-nucleotidase |  |  | LAB37863 | *Micrurus spixii* |
| 10 | TRINITY_DN14672_c1_g2_i1 | 144 | 17 | 2.2259 | 5'-nucleotidase |  |  | XP_026539047 | *Notechis scutatus* |
| 11 | TRINITY_DN658_c0_g1_i17 | 25535 | 0 | 10.523 | Acetylcholinesterase |  | **0.95%** | AAC59905 | *Bungarus fasciatus* |
| 12 | TRINITY_DN658_c0_g1_i23 | 16198 | 0 | 10.523 | Acetylcholinesterase |  |  | AAC59905 | *Bungarus fasciatus* |
| 13 | TRINITY_DN658_c0_g1_i25 | 12626 | 0 | 10.523 | Acetylcholinesterase |  |  | Q92035 | *Bungarus fasciatus* |
| 14 | TRINITY_DN658_c0_g1_i26 | 10794 | 10 | 10.523 | Acetylcholinesterase |  |  | AAC59905 | *Bungarus fasciatus* |
| 15 | TRINITY_DN658_c0_g1_i11 | 3303 | 0 | 10.523 | Acetylcholinesterase |  |  | AAC59905 | *Bungarus fasciatus* |
| 16 | TRINITY_DN658_c0_g1_i20 | 3090 | 0 | 10.523 | Acetylcholinesterase |  |  | Q92035 | *Bungarus fasciatus* |
| 17 | TRINITY_DN658_c0_g1_i3 | 620 | 0 | 10.523 | Acetylcholinesterase |  |  | Q92035 | *Bungarus fasciatus* |
| 18 | TRINITY_DN658_c0_g1_i7 | 382 | 0 | 10.523 | Acetylcholinesterase |  |  | Q92035 | *Bungarus fasciatus* |
| 19 | TRINITY_DN658_c0_g1_i15 | 267 | 0 | 10.523 | Acetylcholinesterase |  |  | Q92035 | *Bungarus fasciatus* |
| 20 | TRINITY_DN658_c0_g1_i1 | 233 | 0 | 10.523 | Acetylcholinesterase |  |  | Q92035 | *Bungarus fasciatus* |
| 21 | TRINITY_DN95_c7_g3_i2 | 240 | 0 | 9.3464 | Acetylcholinesterase |  |  | XP_026576484 | *Pseudonaja textilis* |
| 22 | TRINITY_DN16_c0_g2_i2 | 188135 | 7 | 12.659 | CRISP |  | **6.30%** | ACE73577 | *Bungarus candidus* |
| 23 | TRINITY_DN16_c0_g2_i6 | 175526 | 7 | 12.659 | CRISP |  |  | ACE73577 | *Bungarus candidus* |
| 24 | TRINITY_DN16_c0_g2_i11 | 118278 | 22 | 12.659 | CRISP |  |  | ACE73577 | *Bungarus candidus* |
| 25 | TRINITY_DN16_c0_g2_i14 | 4528 | 0 | 12.659 | CRISP |  |  | ACE73577 | *Bungarus candidus* |
| 26 | TRINITY_DN16_c0_g2_i13 | 613 | 0 | 12.659 | CRISP |  |  | XP_026546646 | *Notechis scutatus* |
| 27 | TRINITY_DN16_c0_g2_i8 | 284 | 0 | 12.659 | CRISP |  |  | ETE62137 | *Ophiophagus hannah* |
| 28 | TRINITY_DN1102_c0_g1_i1 | 1485 | 72 | 3.5098 | CTL |  | **0.33%** | XP_026577709 | *Pseudonaja textilis* |
| 29 | TRINITY_DN2_c1_g2_i1 | 2138 | 1 | 7.1521 | CTL |  |  | ADF50042 | *Bungarus flaviceps* |
| 30 | TRINITY_DN2_c1_g2_i2 | 1198 | 12 | 7.1521 | CTL |  |  | ABP94128 | *Pseudechis porphyriacus* |
| 31 | TRINITY_DN111_c1_g2_i10 | 945 | 8 | 5.9946 | CTL |  |  | XP_026575421 | *Pseudonaja textilis* |
| 32 | TRINITY_DN111_c1_g2_i1 | 107 | 0 | 5.9946 | CTL |  |  | LAA90676 | *Micrurus lemniscatus lemniscatus* |
| 33 | TRINITY_DN286_c0_g1_i28 | 16976 | 0 | 11.125 | CTL |  |  | ACC67942 | *Oxyuranus microlepidotus* |
| 34 | TRINITY_DN286_c0_g1_i1 | 1735 | 0 | 11.125 | CTL |  |  | ABP94094 | *Oxyuranus scutellatus* |
| 35 | TRINITY_DN286_c0_g1_i9 | 866 | 0 | 11.125 | CTL |  |  | ABP94094 | *Oxyuranus scutellatus* |
| 36 | TRINITY_DN286_c0_g1_i7 | 135 | 0 | 11.125 | CTL |  |  | ABP94095 | *Oxyuranus scutellatus* |
| 37 | TRINITY_DN286_c0_g1_i11 | 113 | 4 | 11.125 | CTL |  |  | JAB52841 | *Micrurus fulvius* |
| 38 | TRINITY_DN12_c1_g1_i10 | 1326 | 19 | 3.8658 | Disintegrin-like |  | **1.13%** | XP_026527224 | *Notechis scutatus* |
| 39 | TRINITY_DN87082_c0_g1_i1 | 2594 | 88 | 4.025 | Disintegrin-like |  |  | XP_034289867 | *Pantherophis guttatus* |
| 40 | TRINITY_DN2791_c0_g1_i2 | 247 | 23 | 2.0976 | Disintegrin-like |  |  | ETE74105 | *Ophiophagus hannah* |
| 41 | TRINITY_DN2791_c0_g1_i1 | 187 | 33 | 2.0976 | Disintegrin-like |  |  | LAA74418 | *Micrurus lemniscatus lemniscatus* |
| 42 | TRINITY_DN10_c0_g1_i3 | 16256 | 0 | 10.764 | Disintegrin-like |  |  | LAA20517 | *Micrurus lemniscatus carvalhoi* |
| 43 | TRINITY_DN10_c0_g1_i16 | 16150 | 12 | 10.764 | Disintegrin-like |  |  | ABN72537 | *Bungarus multicinctus* |
| 44 | TRINITY_DN10_c0_g1_i10 | 12234 | 2 | 10.764 | Disintegrin-like |  |  | ABN72537 | *Bungarus multicinctus* |
| 45 | TRINITY_DN10_c0_g1_i11 | 8426 | 0 | 10.764 | Disintegrin-like |  |  | LAA20517 | *Micrurus lemniscatus carvalhoi* |
| 46 | TRINITY_DN10_c0_g1_i4 | 8296 | 0 | 10.764 | Disintegrin-like |  |  | LAA20517 | *Micrurus lemniscatus carvalhoi* |
| 47 | TRINITY_DN10_c0_g1_i7 | 6015 | 0 | 10.764 | Disintegrin-like |  |  | AEH95531 | *Drysdalia coronoides* |
| 48 | TRINITY_DN10_c0_g1_i5 | 1540 | 6 | 10.764 | Disintegrin-like |  |  | ABN72537 | *Bungarus multicinctus* |
| 49 | TRINITY_DN10_c0_g1_i6 | 1298 | 0 | 10.764 | Disintegrin-like |  |  | ABN72537 | *Bungarus multicinctus* |
| 50 | TRINITY_DN10_c0_g1_i8 | 1245 | 0 | 10.764 | Disintegrin-like |  |  | ABN72537 | *Bungarus multicinctus* |
| 51 | TRINITY_DN10_c0_g1_i18 | 803 | 0 | 10.764 | Disintegrin-like |  |  | ABN72536 | *Bungarus fasciatus* |
| 52 | TRINITY_DN10_c0_g1_i1 | 132 | 0 | 10.764 | Disintegrin-like |  |  | ABN72537 | *Bungarus multicinctus* |
| 53 | TRINITY_DN8820_c0_g1_i2 | 860 | 19 | 4.6437 | Disintegrin-like |  |  | ETE68273 | *Ophiophagus hannah* |
| 54 | TRINITY_DN8043_c0_g1_i1 | 542 | 17 | 4.1723 | Disintegrin-like |  |  | XP_034279507 | *Pantherophis guttatus* |
| 55 | TRINITY_DN9584_c0_g1_i2 | 328 | 0 | 6.0593 | Disintegrin-like |  |  | XP_034280049 | *Pantherophis guttatus* |
| 56 | TRINITY_DN9584_c0_g1_i6 | 237 | 0 | 6.0593 | Disintegrin-like |  |  | XP_026577550 | *Pseudonaja textilis* |
| 57 | TRINITY_DN9584_c0_g1_i7 | 147 | 1 | 6.0593 | Disintegrin-like |  |  | XP_034280049 | *Pantherophis guttatus* |
| 58 | TRINITY_DN1565_c0_g1_i2 | 810 | 5 | 6.4951 | Disintegrin-like |  |  | ABN72537 | *Bungarus multicinctus* |
| 59 | TRINITY_DN1565_c0_g1_i1 | 170 | 1 | 6.4951 | Disintegrin-like |  |  | ABN72537 | *Bungarus multicinctus* |
| 60 | TRINITY_DN389_c1_g1_i1 | 395 | 2 | 6.868 | Disintegrin-like |  |  | ABQ01138 | *Notechis scutatus* |
| 61 | TRINITY_DN2189_c0_g2_i3 | 2691 | 0 | 12.296 | Disintegrin-like |  |  | ABK63559 | *Demansia vestigiata* |
| 62 | TRINITY_DN10_c4_g1_i1 | 2526 | 0 | 12.087 | Disintegrin-like |  |  | JAA74899 | *Pseudonaja modesta* |
| 63 | TRINITY_DN2873_c0_g1_i1 | 792 | 0 | 10.414 | Disintegrin-like |  |  | XP_026536946 | *Notechis scutatus* |
| 64 | TRINITY_DN5452_c0_g1_i1 | 543 | 0 | 9.869 | Disintegrin-like |  |  | ABN72537 | *Bungarus multicinctus* |
| 65 | TRINITY_DN5452_c0_g3_i1 | 399 | 0 | 9.4244 | Disintegrin-like |  |  | ABN72537 | *Bungarus multicinctus* |
| 66 | TRINITY_DN389_c0_g1_i2 | 248 | 0 | 8.8231 | Disintegrin-like |  |  | XP_013922279 | *Thamnophis sirtalis* |
| 67 | TRINITY_DN1030_c0_g1_i5 | 334 | 11 | 2.8671 | Hyaluronidase |  | **0.14%** | ETE63480 | *Ophiophagus hannah* |
| 68 | TRINITY_DN3688_c2_g1_i4 | 9416 | 0 | 10.503 | Hyaluronidase |  |  | XP_026524834 | *Notechis scutatus* |
| 69 | TRINITY_DN3688_c2_g1_i7 | 473 | 0 | 10.503 | Hyaluronidase |  |  | XP_026524834 | *Notechis scutatus* |
| 70 | TRINITY_DN3688_c2_g1_i3 | 428 | 2 | 10.503 | Hyaluronidase |  |  | LAA86771 | *Micrurus lemniscatus lemniscatus* |
| 71 | TRINITY_DN3688_c2_g1_i6 | 182 | 2 | 10.503 | Hyaluronidase |  |  | XP_026524834 | *Notechis scutatus* |
| 72 | TRINITY_DN3_c0_g1_i38 | 819143 | 2 | 8.2948 | Kunitz |  | **11.89%** | LAA53789 | *Micrurus corallinus* |
| 73 | TRINITY_DN3_c0_g1_i35 | 429 | 0 | 8.2948 | Kunitz |  |  | LAA53789 | *Micrurus corallinus* |
| 74 | TRINITY_DN3_c0_g1_i37 | 192 | 0 | 8.2948 | Kunitz |  |  | LAA53789 | *Micrurus corallinus* |
| 75 | TRINITY_DN855_c0_g1_i43 | 1239 | 323 | 3.3088 | Kunitz |  |  | XP_026543549 | *Notechis scutatus* |
| 76 | TRINITY_DN855_c0_g1_i76 | 1128 | 0 | 3.3088 | Kunitz |  |  | XP_026543549 | *Notechis scutatus* |
| 77 | TRINITY_DN855_c0_g1_i62 | 855 | 260 | 3.3088 | Kunitz |  |  | XP_026543549 | *Notechis scutatus* |
| 78 | TRINITY_DN855_c0_g1_i89 | 629 | 334 | 3.3088 | Kunitz |  |  | XP_026543549 | *Notechis scutatus* |
| 79 | TRINITY_DN8916_c1_g1_i4 | 92109 | 1 | 10.885 | Kunitz |  |  | ACR78501 | *Drysdalia coronoides* |
| 80 | TRINITY_DN2341_c0_g1_i1 | 2869 | 13 | 6.9313 | Kunitz |  |  | XP_026579404 | *Pseudonaja textilis* |
| 81 | TRINITY_DN2341_c1_g1_i1 | 170 | 1 | 6.5528 | Kunitz |  |  | XP_026581272 | *Pseudonaja textilis* |
| 82 | TRINITY_DN2565_c0_g1_i1 | 398 | 0 | 9.4208 | Kunitz |  |  | XP_026545967 | *Notechis scutatus* |
| 83 | TRINITY_DN1055_c0_g1_i1 | 132 | 0 | 8.5786 | Kunitz |  |  | XP_026579465 | *Pseudonaja textilis* |
| 84 | TRINITY_DN8381_c0_g1_i5 | 104 | 0 | 8.6911 | Kunitz |  |  | XP_032074676 | *Thamnophis elegans* |
| 85 | TRINITY_DN420_c0_g1_i9 | 18068 | 0 | 7.7758 | **LAAO** |  | **0.40%** | ABN72539 | *Bungarus multicinctus* |
| 86 | TRINITY_DN420_c0_g1_i15 | 11506 | 0 | 7.7758 | **LAAO** |  |  | ABN72540 | *Bungarus fasciatus* |
| 87 | TRINITY_DN420_c0_g1_i14 | 688 | 5 | 7.7758 | **LAAO** |  |  | ABN72540 | *Bungarus fasciatus* |
| 88 | TRINITY_DN420_c0_g1_i8 | 605 | 0 | 7.7758 | **LAAO** |  |  | ABN72539 | *Bungarus multicinctus* |
| 89 | TRINITY_DN45_c1_g1_i2 | 120 | 0 | 9.9997 | Natriuretic peptide |  | **4.85%** | ADK12002 | *Ophiophagus hannah* |
| 90 | TRINITY_DN45_c1_g1_i5 | 195105 | 0 | 9.9997 | Natriuretic peptide |  |  | ADK12002 | *Ophiophagus hannah* |
| 91 | TRINITY_DN45_c1_g1_i4 | 171746 | 10 | 9.9997 | Natriuretic peptide |  |  | ADK12002 | *Ophiophagus hannah* |
| 92 | TRINITY_DN45_c1_g1_i6 | 7494 | 192 | 9.9997 | Natriuretic peptide |  |  | JAS05144 | *Micrurus tener* |
| 93 | TRINITY_DN295_c0_g1_i2 | 614 | 1 | 11.107 | Natriuretic peptide |  |  | ADK12002 | *Ophiophagus hannah* |
| 94 | TRINITY_DN205_c0_g1_i8 | 8309 | 2 | 9.6574 | **NGF** |  | **0.18%** | AAB25729 | *Bungarus multicinctus* |
| 95 | TRINITY_DN205_c0_g1_i15 | 1566 | 0 | 9.6574 | **NGF** |  |  | AAB25729 | *Bungarus multicinctus* |
| 96 | TRINITY_DN205_c0_g1_i11 | 1415 | 0 | 9.6574 | **NGF** |  |  | AAB25729 | *Bungarus multicinctus* |
| 97 | TRINITY_DN205_c0_g1_i3 | 1065 | 8 | 9.6574 | **NGF** |  |  | AAB25729 | *Bungarus multicinctus* |
| 98 | TRINITY_DN205_c0_g1_i7 | 627 | 0 | 9.6574 | **NGF** |  |  | AAB25729 | *Bungarus multicinctus* |
| 99 | TRINITY_DN205_c0_g1_i5 | 393 | 0 | 9.6574 | **NGF** |  |  | AAB25729 | *Bungarus multicinctus* |
| 100 | TRINITY_DN205_c0_g1_i13 | 388 | 0 | 9.6574 | **NGF** |  |  | AAB25729 | *Bungarus multicinctus* |
| 101 | TRINITY_DN205_c0_g1_i4 | 252 | 0 | 9.6574 | **NGF** |  |  | AAB25729 | *Bungarus multicinctus* |
| 102 | TRINITY_DN158_c0_g2_i21 | 416242 | 0 | 6.7164 | PLA2 |  | **23.33%** | ACY68711 | *Parasuta nigriceps* |
| 103 | TRINITY_DN158_c0_g2_i5 | 19472 | 0 | 6.7164 | PLA2 |  |  | LAA18991 | *Micrurus lemniscatus carvalhoi* |
| 104 | TRINITY_DN158_c0_g1_i7 | 523798 | 0 | 8.1888 | PLA2 |  |  | AAB24834 | *Bungarus fasciatus* |
| 105 | TRINITY_DN158_c0_g1_i18 | 337319 | 0 | 8.1888 | PLA2 |  |  | ABG75909 | *Bungarus fasciatus* |
| 106 | TRINITY_DN158_c0_g1_i27 | 11669 | 0 | 8.1888 | PLA2 |  |  | LAA39992 | *Micrurus corallinus* |
| 107 | TRINITY_DN158_c0_g1_i36 | 3947 | 0 | 8.1888 | PLA2 |  |  | AAB24834 | *Bungarus fasciatus* |
| 108 | TRINITY_DN66_c3_g1_i2 | 3595 | 0 | 2.7671 | PLA2 |  |  | XP_034286728 | *Pantherophis guttatus* |
| 109 | TRINITY_DN66_c3_g1_i1 | 337 | 319 | 2.7671 | PLA2 |  |  | XP_034286728 | *Pantherophis guttatus* |
| 110 | TRINITY_DN4685_c0_g1_i9 | 340 | 0 | 2.6072 | PLA2 |  |  | LAA68819 | *Micrurus lemniscatus lemniscatus* |
| 111 | TRINITY_DN4685_c0_g1_i20 | 208 | 0 | 2.6072 | PLA2 |  |  | LAA68819 | *Micrurus lemniscatus lemniscatus* |
| 112 | TRINITY_DN3_c4_g1_i16 | 1713 | 0 | 5.1741 | PLA2 |  |  | LAA68819 | *Micrurus lemniscatus lemniscatus* |
| 113 | TRINITY_DN3_c4_g1_i22 | 296 | 37 | 5.1741 | PLA2 |  |  | LAA68819 | *Micrurus lemniscatus lemniscatus* |
| 114 | TRINITY_DN241_c0_g1_i1 | 483423 | 4 | 16.026 | PLA2 |  |  | ABM88801 | *Hoplocephalus stephensii* |
| 115 | TRINITY_DN3025_c1_g2_i1 | 124 | 3 | 4.5127 | PLA2 |  |  | CAD24462 | *Bungarus multicinctus* |
| 116 | TRINITY_DN23151_c0_g1_i2 | 122 | 0 | 4.5243 | PLA2 |  |  | XP_026565791 | *Pseudonaja textilis* |
| 117 | TRINITY_DN5043_c1_g1_i8 | 1042 | 0 | 9.7582 | PLA2 |  |  | ADF50037 | *Bungarus flaviceps* |
| 118 | TRINITY_DN5043_c1_g1_i9 | 346 | 0 | 9.7582 | PLA2 |  |  | ADF50037 | *Bungarus flaviceps* |
| 119 | TRINITY_DN5043_c1_g1_i1 | 162 | 0 | 9.7582 | PLA2 |  |  | ADF50037 | *Bungarus flaviceps* |
| 120 | TRINITY_DN2464_c0_g1_i2 | 15349 | 74 | 5.1041 | PLB |  | **0.20%** | ACR78473 | *Drysdalia coronoides* |
| 121 | TRINITY_DN2464_c0_g1_i1 | 283 | 177 | 5.1041 | PLB |  |  | ACR78473 | *Drysdalia coronoides* |
| 122 | TRINITY_DN3093_c0_g1_i8 | 271 | 2 | 2.0355 | Serine Protease |  | **0.02%** | ABN72545 | *Bungarus multicinctus* |
| 123 | TRINITY_DN1_c16_g2_i4 | 1078 | 34 | 4.7938 | Serine Protease |  |  | XP_026562705 | *Pseudonaja textilis* |
| 124 | TRINITY_DN821_c0_g1_i7 | 57470 | 0 | 8.1587 | Type-I α-neurotoxin | **64.44%** | **29.06%** | CAA45882 | *Bungarus multicinctus* |
| 125 | TRINITY_DN821_c0_g1_i3 | 25406 | 0 | 8.1587 | Type-I α-neurotoxin |  |  | CAA45882 | *Bungarus multicinctus* |
| 126 | TRINITY_DN179_c0_g1_i11 | 85078 | 1 | 13.033 | Type-I α-neurotoxin |  |  | CAA06887 | *Bungarus multicinctus multicinctus* |
| 127 | TRINITY_DN179_c0_g1_i10 | 20468 | 0 | 13.033 | Type-I α-neurotoxin |  |  | CAA06887 | *Bungarus multicinctus multicinctus* |
| 128 | TRINITY_DN179_c0_g1_i3 | 19758 | 0 | 13.033 | Type-I α-neurotoxin |  |  | CAA06887 | *Bungarus multicinctus multicinctus* |
| 129 | TRINITY_DN4374_c0_g1_i2 | 165563 | 3 | 12.177 | Type-I α-neurotoxin |  |  | ETE58964 | *Ophiophagus hannah* |
| 130 | TRINITY_DN31129_c0_g1_i1 | 160874 | 29 | 11.095 | Type-II α-neurotoxin |  |  | AHZ08825 | *Micropechis ikaheca* |
| 131 | TRINITY_DN31129_c0_g1_i2 | 1544 | 7 | 11.095 | Type-II α-neurotoxin |  |  | CAJ77820 | *Bungarus candidus* |
| 132 | TRINITY_DN133_c4_g1_i5 | 896317 | 29 | 13.781 | Type-II α-neurotoxin |  |  | ETE56306 | *Ophiophagus hannah* |
| 133 | TRINITY_DN133_c4_g1_i1 | 3878 | 6 | 13.781 | Type-II α-neurotoxin |  |  | ETE56306 | *Ophiophagus hannah* |
| 134 | TRINITY_DN909_c0_g1_i13 | 3510 | 1 | 12.807 | Type-II α-neurotoxin |  |  | CAA10359 | *Bungarus multicinctus* |
| 135 | TRINITY_DN909_c0_g1_i38 | 2921 | 0 | 12.807 | Type-II α-neurotoxin |  |  | CAA10359 | *Bungarus multicinctus* |
| 136 | TRINITY_DN909_c0_g1_i40 | 1653 | 0 | 12.807 | Type-II α-neurotoxin |  |  | LAA40709 | *Micrurus corallinus* |
| 137 | TRINITY_DN10768_c0_g1_i3 | 824 | 0 | 17.948 | Type-II α-neurotoxin |  |  | ETE56205 | *Ophiophagus hannah* |
| 138 | TRINITY_DN909_c0_g1_i11 | 564 | 0 | 12.807 | Type-II α-neurotoxin |  |  | LAA40709 | *Micrurus corallinus* |
| 139 | TRINITY_DN909_c0_g1_i17 | 548 | 0 | 12.807 | Type-II α-neurotoxin |  |  | ABK63538 | *Tropidechis carinatus* |
| 140 | TRINITY_DN909_c0_g1_i1 | 199 | 0 | 12.807 | Type-II α-neurotoxin |  |  | LAA40709 | *Micrurus corallinus* |
| 141 | TRINITY_DN909_c0_g1_i37 | 177 | 0 | 12.807 | Type-II α-neurotoxin |  |  | ETE56521 | *Ophiophagus hannah* |
| 142 | TRINITY_DN2350_c0_g3_i1 | 854 | 0 | 10.522 | Type-II α-neurotoxin |  |  | LAA40709 | *Micrurus corallinus* |
| 143 | TRINITY_DN909_c1_g1_i2 | 389 | 0 | 9.9083 | Type-II α-neurotoxin |  |  | LAA40709 | *Micrurus corallinus* |
| 144 | TRINITY_DN3640_c0_g1_i5 | 147 | 0 | 11.252 | Type-II α-neurotoxin |  |  | ETE56205 | *Ophiophagus hannah* |
| 145 | TRINITY_DN3640_c0_g1_i10 | 120 | 0 | 11.252 | Type-II α-neurotoxin |  |  | LAA40709 | *Micrurus corallinus* |
| 146 | TRINITY_DN821_c0_g2_i2 | 187 | 0 | 12.673 | Unconventional 3FTx | **35.56%** |  | CAD56381 | *Bungarus multicinctus* |
| 147 | TRINITY_DN179_c0_g1_i4 | 173811 | 3 | 13.033 | Unconventional 3FTx |  |  | AAL30060 | *Bungarus candidus* |
| 148 | TRINITY_DN179_c0_g1_i37 | 163976 | 4 | 13.033 | Unconventional 3FTx |  |  | CAB53358 | *Bungarus multicinctus multicinctus* |
| 149 | TRINITY_DN179_c0_g1_i15 | 131062 | 0 | 13.033 | Unconventional 3FTx |  |  | LAA65595 | *Micrurus corallinus* |
| 150 | TRINITY_DN179_c0_g1_i31 | 95299 | 2 | 13.033 | Unconventional 3FTx |  |  | AAL30059 | *Bungarus candidus* |
| 151 | TRINITY_DN1177_c0_g1_i30 | 126997 | 5 | 15.099 | Unconventional 3FTx |  |  | CAA06885 | *Bungarus multicinctus multicinctus* |
| 152 | TRINITY_DN3640_c0_g2_i3 | 37781 | 1 | 11.648 | Unconventional 3FTx |  |  | CAB53358 | *Bungarus multicinctus multicinctus* |
| 153 | TRINITY_DN3640_c0_g2_i2 | 19948 | 7 | 11.648 | Unconventional 3FTx |  |  | CAB53358 | *Bungarus multicinctus multicinctus* |
| 154 | TRINITY_DN821_c0_g2_i1 | 47123 | 4 | 12.673 | Unconventional 3FTx |  |  | CAD56381 | *Bungarus multicinctus* |
| 155 | TRINITY_DN909_c0_g1_i25 | 2051 | 0 | 12.807 | Unconventional 3FTx |  |  | ETE56205 | *Ophiophagus hannah* |
| 156 | TRINITY_DN10768_c3_g1_i1 | 930 | 0 | 10.645 | Unconventional 3FTx |  |  | CAB53358 | *Bungarus multicinctus multicinctus* |
| 157 | TRINITY_DN21645_c1_g2_i1 | 104 | 0 | 7.4846 | Unconventional 3FTx |  |  | XP_034276006 | *Pantherophis guttatus* |
| 158 | TRINITY_DN596_c0_g1_i6 | 18639 | 0 | 7.6955 | VEGF |  | **0.51%** | JAA74898 | *Pseudonaja modesta* |
| 159 | TRINITY_DN596_c0_g1_i4 | 6532 | 16 | 7.6955 | VEGF |  |  | XP_026551346 | *Pseudonaja textilis* |
| 160 | TRINITY_DN596_c0_g1_i2 | 5719 | 30 | 7.6955 | VEGF |  |  | XP_026551346 | *Pseudonaja textilis* |
| 161 | TRINITY_DN596_c0_g1_i1 | 3064 | 0 | 7.6955 | VEGF |  |  | XP_026551344 | *Pseudonaja textilis* |
| 162 | TRINITY_DN596_c0_g1_i10 | 744 | 0 | 7.6955 | VEGF |  |  | XP_026551344 | *Pseudonaja textilis* |
| 163 | TRINITY_DN596_c0_g1_i5 | 667 | 0 | 7.6955 | VEGF |  |  | JAA74688 | *Cacophis squamulosus* |
| 164 | TRINITY_DN1555_c0_g1_i2 | 3490 | 40 | 5.0585 | VEGF |  |  | LAB17921 | *Micrurus spixii* |
| 165 | TRINITY_DN4014_c2_g2_i2 | 181 | 9 | 3.3181 | VEGF |  |  | XP_026542052 | *Notechis scutatus* |
| 166 | TRINITY_DN205_c0_g1_i9 | 607 | 0 | 9.6574 | VEGF |  |  | AAB25729 | *Bungarus multicinctus* |
| 167 | TRINITY_DN2536_c0_g1_i1 | 29714 | 4 | 12.002 | Vespryn |  | **0.38%** | XP_026576508 | *Pseudonaja textilis* |
| 168 | TRINITY_DN158_c0_g2_i12 | 513486 | 17 | 6.7164 | β-bungarotoxin |  | **20.09%** | BAD06267 | *Bungarus candidus* |
| 169 | TRINITY_DN158_c0_g1_i21 | 428465 | 178 | 8.1888 | β-bungarotoxin |  |  | CAD24462.1 | *Micrurus corallinus* |
| 170 | TRINITY_DN158_c0_g1_i11 | 128743 | 5 | 8.1888 | β-bungarotoxin |  |  | CAD24466 | *Bungarus multicinctus* |
| 171 | TRINITY_DN158_c0_g1_i31 | 31094 | 0 | 8.1888 | β-bungarotoxin |  |  | LAA39992 | *Micrurus corallinus* |
| 172 | TRINITY_DN158_c0_g1_i28 | 25784 | 0 | 8.1888 | β-bungarotoxin |  |  | LAA39992 | *Micrurus corallinus* |
| 173 | TRINITY_DN158_c0_g1_i9 | 4339 | 8 | 8.1888 | β-bungarotoxin |  |  | LAA39992 | *Micrurus corallinus* |
| 174 | TRINITY_DN9846_c0_g1_i3 | 57946 | 3 | 6.569 | β-bungarotoxin |  |  | JAA74785 | *Furina ornata* |
| 175 | TRINITY_DN4685_c0_g1_i23 | 284 | 0 | 2.6072 | β-bungarotoxin |  |  | ABG90492 | *Bungarus multicinctus* |
| 176 | TRINITY_DN4189_c0_g1_i6 | 229956 | 22 | 10.269 | β-bungarotoxin |  |  | AAL87003 | *Bungarus caeruleus* |
| 177 | TRINITY_DN374_c0_g1_i8 | 70499 | 1 | 11.932 | β-bungarotoxin |  |  | JAA74785 | *Furina ornata* |
| 178 | TRINITY_DN374_c0_g1_i17 | 55732 | 0 | 11.932 | β-bungarotoxin |  |  | JAA74785 | *Furina ornata* |
| 179 | TRINITY_DN374_c0_g1_i22 | 3123 | 0 | 11.932 | β-bungarotoxin |  |  | AAL30065 | *Bungarus candidus* |
| 180 | TRINITY_DN374_c0_g1_i10 | 241 | 12 | 11.932 | β-bungarotoxin |  |  | JAA74785 | *Furina ornata* |
| 181 | TRINITY_DN2983_c0_g1_i2 | 197 | 2 | 5.9443 | β-bungarotoxin |  |  | AAL30063 | *Bungarus candidus* |
| 182 | TRINITY_DN370_c0_g1_i7 | 3912 | 0 | 13.603 | β-bungarotoxin |  |  | CAA72809 | *Bungarus multicinctus* |

Each toxin family and the corresponding relative abundance is uniquely colour coded.

**Table S11.** Venom gland toxin transcripts from *Bungarus caeruleus.*

| **S. No.** | **Isoform** | | **VG** | **Int** | **M** | **Family** | **Subtype** | **Abundance** | **Accession** | **Scientific Name** |
| --- | --- | --- | --- | --- | --- | --- | --- | --- | --- | --- |
| 1 | TRINITY_DN770_c0_g1 | 5199 | | 8 | 6.12009511 | 5'-nucleotidase |  | **0.04%** | XP_026581084 | *Pseudonaja textilis* |
| 2 | TRINITY_DN4162_c0_g1 | 415 | | 27 | 2.338480665 | 5'-nucleotidase |  |  | XP_026539324 | *Notechis scutatus* |
| 3 | TRINITY_DN4162_c0_g1 | 238 | | 0 | 2.338480665 | 5'-nucleotidase |  |  | XP_026539324 | *Notechis scutatus* |
| 4 | TRINITY_DN4162_c0_g1 | 218 | | 125 | 2.338480665 | 5'-nucleotidase |  |  | XP_026539324 | *Notechis scutatus* |
| 5 | TRINITY_DN5084_c1_g2 | 204 | | 39 | 2.000383847 | 5'-nucleotidase |  |  | XP_026522489 | *Notechis scutatus* |
| 6 | TRINITY_DN4162_c0_g1 | 176 | | 18 | 2.338480665 | 5'-nucleotidase |  |  | XP_026539324 | *Notechis scutatus* |
| 7 | TRINITY_DN846_c0_g1 | 107953 | | 22 | 14.70189849 | Acetylcholinesterase |  | **1.28%** | AAC59905 | *Bungarus fasciatus* |
| 8 | TRINITY_DN846_c0_g1 | 68234 | | 3 | 14.70189849 | Acetylcholinesterase |  |  | AAC59905 | *Bungarus fasciatus* |
| 9 | TRINITY_DN846_c0_g1 | 31465 | | 0 | 14.70189849 | Acetylcholinesterase |  |  | Q92035 | *Bungarus fasciatus* |
| 10 | TRINITY_DN846_c0_g1 | 744 | | 0 | 14.70189849 | Acetylcholinesterase |  |  | Q92035 | *Bungarus fasciatus* |
| 11 | TRINITY_DN389_c1_g1 | 1202248 | | 48 | 17.10596803 | CRISP |  | **13.07%** | ACE73577 | *Bungarus candidus* |
| 12 | TRINITY_DN389_c1_g1 | 918054 | | 0 | 17.10596803 | CRISP |  |  | ACE73577 | *Bungarus candidus* |
| 13 | TRINITY_DN2703_c0_g1 | 10221 | | 669 | 4.223205459 | CRISP |  |  | JAB52844 | *Micrurus fulvius* |
| 14 | TRINITY_DN5802_c0_g1 | 743 | | 0 | 11.38622429 | CRISP |  |  | ACE73578 | *Bungarus candidus* |
| 15 | TRINITY_DN1897_c0_g1 | 676 | | 0 | 13.5002171 | CRISP |  |  | XP_015678374 | *Protobothrops mucrosquamatus* |
| 16 | TRINITY_DN11613_c0_g1 | 410 | | 0 | 10.51880993 | CRISP |  |  | AAP20603 | *Naja atra* |
| 17 | TRINITY_DN14181_c1_g1 | 237 | | 8 | 5.476371925 | CRISP |  |  | XP_026539804 | *Notechis scutatus* |
| 18 | TRINITY_DN2322_c0_g1 | 219 | | 0 | 2.755185109 | CRISP |  |  | LAB38794 | *Micrurus spixii* |
| 19 | TRINITY_DN140247_c0_g1 | 188 | | 0 | 9.393918685 | CRISP |  |  | XP_026568632 | *Pseudonaja textilis* |
| 20 | TRINITY_DN676_c1_g2 | 1541 | | 3 | 10.5291807 | CTL |  | **0.05%** | AAK43586 | *Bungarus multicinctus* |
| 21 | TRINITY_DN1049_c0_g1 | 1528 | | 20 | 8.369519998 | CTL |  |  | ABP94094 | *Oxyuranus scutellatus* |
| 22 | TRINITY_DN1049_c0_g1 | 1213 | | 0 | 8.369519998 | CTL |  |  | JAA74667 | *Brachyurophis roperi* |
| 23 | TRINITY_DN676_c1_g2 | 1020 | | 0 | 10.5291807 | CTL |  |  | ABH05181 | *Bungarus multicinctus* |
| 24 | TRINITY_DN676_c1_g2 | 629 | | 0 | 10.5291807 | CTL |  |  | AAK43586 | *Bungarus multicinctus* |
| 25 | TRINITY_DN676_c1_g2 | 496 | | 0 | 10.5291807 | CTL |  |  | AAK43586 | *Bungarus multicinctus* |
| 26 | TRINITY_DN1049_c0_g1 | 458 | | 7 | 8.369519998 | CTL |  |  | ACC67942 | *Oxyuranus microlepidotus* |
| 27 | TRINITY_DN4221_c0_g1 | 350 | | 44 | 4.666871011 | CTL |  |  | XP_026577709 | *Pseudonaja textilis* |
| 28 | TRINITY_DN1049_c0_g1 | 254 | | 0 | 8.369519998 | CTL |  |  | ABU68499 | *Leioheterodon madagascariensis* |
| 29 | TRINITY_DN1049_c0_g1 | 128 | | 0 | 8.369519998 | CTL |  |  | JAC95003 | *Pantherophis guttatus* |
| 30 | TRINITY_DN541_c0_g1 | 163938 | | 0 | 16.49630974 | Disintegrin-like |  | **2.52%** | ABQ01132 | *Tropidechis carinatus* |
| 31 | TRINITY_DN541_c0_g1 | 67618 | | 0 | 16.49630974 | Disintegrin-like |  |  | ABN72537 | *Bungarus multicinctus* |
| 32 | TRINITY_DN541_c0_g1 | 43227 | | 7 | 16.49630974 | Disintegrin-like |  |  | ABN72537 | *Bungarus multicinctus* |
| 33 | TRINITY_DN541_c0_g1 | 40431 | | 2 | 16.49630974 | Disintegrin-like |  |  | ABN72537 | *Bungarus multicinctus* |
| 34 | TRINITY_DN541_c0_g1 | 39001 | | 2 | 16.49630974 | Disintegrin-like |  |  | ABN72537 | *Bungarus multicinctus* |
| 35 | TRINITY_DN1050_c0_g1 | 18795 | | 3 | 12.89954547 | Disintegrin-like |  |  | ABN72537 | *Bungarus multicinctus* |
| 36 | TRINITY_DN541_c0_g1 | 8597 | | 0 | 16.49630974 | Disintegrin-like |  |  | LAB50889 | *Micrurus surinamensis* |
| 37 | TRINITY_DN541_c0_g1 | 8254 | | 0 | 16.49630974 | Disintegrin-like |  |  | ABN72537 | *Bungarus multicinctus* |
| 38 | TRINITY_DN17326_c0_g1 | 6890 | | 0 | 14.64265375 | Disintegrin-like |  |  | JAA74814 | *Hemiaspis signata* |
| 39 | TRINITY_DN541_c0_g1 | 3901 | | 0 | 16.49630974 | Disintegrin-like |  |  | ABN72537 | *Bungarus multicinctus* |
| 40 | TRINITY_DN995_c0_g1 | 2987 | | 0 | 7.945553716 | Disintegrin-like |  |  | ADI47635 | *Echis coloratus* |
| 41 | TRINITY_DN34742_c0_g1 | 2967 | | 0 | 13.47096164 | Disintegrin-like |  |  | XP_032084682 | *Thamnophis elegans* |
| 42 | TRINITY_DN541_c0_g1 | 1369 | | 2 | 16.49630974 | Disintegrin-like |  |  | ABN72537 | *Bungarus multicinctus* |
| 43 | TRINITY_DN5558_c0_g1 | 863 | | 7 | 7.021288627 | Disintegrin-like |  |  | XP_007430976 | *Python bivittatus* |
| 44 | TRINITY_DN36697_c0_g4 | 387 | | 0 | 5.357901342 | Disintegrin-like |  |  | JAA74866 | *Hoplocephalus bungaroides* |
| 45 | TRINITY_DN17326_c0_g1 | 231 | | 0 | 14.64265375 | Disintegrin-like |  |  | JAA74814 | *Hemiaspis signata* |
| 46 | TRINITY_DN6269_c0_g1 | 202 | | 98 | 2.849120917 | Disintegrin-like |  |  | XP_034287530 | *Pantherophis guttatus* |
| 47 | TRINITY_DN11657_c0_g1 | 201 | | 0 | 8.356915558 | Disintegrin-like |  |  | XP_034276822 | *Pantherophis guttatus* |
| 48 | TRINITY_DN1050_c0_g1 | 197 | | 0 | 12.89954547 | Disintegrin-like |  |  | LAB61872 | *Micrurus surinamensis* |
| 49 | TRINITY_DN5558_c0_g1 | 159 | | 8 | 7.021288627 | Disintegrin-like |  |  | XP_013912183 | *Thamnophis sirtalis* |
| 50 | TRINITY_DN995_c0_g1 | 142 | | 0 | 7.945553716 | Disintegrin-like |  |  | JAS05092 | *Micrurus tener* |
| 51 | TRINITY_DN995_c0_g1 | 112 | | 0 | 7.945553716 | Disintegrin-like |  |  | ABK63559 | *Demansia vestigiata* |
| 52 | TRINITY_DN6561_c0_g2 | 108 | | 54 | 2.822845138 | Disintegrin-like |  |  | XP_026538843 | *Notechis scutatus* |
| 53 | TRINITY_DN5558_c0_g1 | 108 | | 0 | 7.021288627 | Disintegrin-like |  |  | XP_013912183 | *Thamnophis sirtalis* |
| 54 | TRINITY_DN995_c0_g1 | 102 | | 0 | 7.945553716 | Disintegrin-like |  |  | ADI47635 | *Echis coloratus* |
| 55 | TRINITY_DN7688_c0_g1 | 6890 | | 0 | 2.192026844 | DPP |  | **0.06%** | XP_026530199 | *Notechis scutatus* |
| 56 | TRINITY_DN7688_c0_g1 | 2576 | | 0 | 2.192026844 | DPP |  |  | ABQ63098 | *Oxyuranus scutellatus* |
| 57 | TRINITY_DN7688_c0_g1 | 603 | | 90 | 2.192026844 | DPP |  |  | ABQ63103 | *Pseudechis australis* |
| 58 | TRINITY_DN7454_c0_g1 | 42758 | | 0 | 14.67713735 | Hyaluronidase |  | **0.40%** | XP_026524834 | *Notechis scutatus* |
| 59 | TRINITY_DN7454_c0_g1 | 19050 | | 0 | 14.67713735 | Hyaluronidase |  |  | XP_034277309 | *Pantherophis guttatus* |
| 60 | TRINITY_DN7454_c0_g1 | 1270 | | 0 | 14.67713735 | Hyaluronidase |  |  | XP_034277309 | *Pantherophis guttatus* |
| 61 | TRINITY_DN7454_c0_g1 | 1187 | | 0 | 14.67713735 | Hyaluronidase |  |  | XP_026524834 | *Notechis scutatus* |
| 62 | TRINITY_DN7454_c0_g1 | 551 | | 0 | 14.67713735 | Hyaluronidase |  |  | XP_026524834 | *Notechis scutatus* |
| 63 | TRINITY_DN7454_c0_g1 | 496 | | 0 | 14.67713735 | Hyaluronidase |  |  | LAA86771 | *Micrurus lemniscatus* |
| 64 | TRINITY_DN567_c0_g1 | 1282916 | | 0 | 13.81824703 | Kunitz |  | **8.94%** | ACR78501 | *Drysdalia coronoides* |
| 65 | TRINITY_DN9666_c0_g1 | 161168 | | 0 | 19.13753563 | Kunitz |  |  | XP_026579466 | *Pseudonaja textilis* |
| 66 | TRINITY_DN1301_c0_g1 | 6801 | | 0 | 2.848076319 | Kunitz |  |  | XP_026543549 | *Notechis scutatus* |
| 67 | TRINITY_DN389_c1_g2 | 3686 | | 0 | 13.69341901 | Kunitz |  |  | ABN72545 | *Bungarus multicinctus* |
| 68 | TRINITY_DN2477_c0_g1 | 2300 | | 1513 | 2.201361066 | Kunitz |  |  | XP_026520368 | *Notechis scutatus* |
| 69 | TRINITY_DN567_c0_g1 | 892 | | 0 | 13.81824703 | Kunitz |  |  | ACR78501 | *Drysdalia coronoides* |
| 70 | TRINITY_DN567_c0_g1 | 234 | | 0 | 13.81824703 | Kunitz |  |  | LAA53789 | *Micrurus corallinus* |
| 71 | TRINITY_DN567_c0_g1 | 208 | | 0 | 13.81824703 | Kunitz |  |  | LAA53789 | *Micrurus corallinus* |
| 72 | TRINITY_DN482_c0_g1 | 112710 | | 0 | 11.91885276 | **LAAO** |  | **1.72%** | ABN72539 | *Bungarus multicinctus* |
| 73 | TRINITY_DN482_c0_g1 | 51876 | | 0 | 11.91885276 | **LAAO** |  |  | ABN72540 | *Bungarus fasciatus* |
| 74 | TRINITY_DN482_c0_g1 | 36169 | | 0 | 11.91885276 | **LAAO** |  |  | ABN72539 | *Bungarus multicinctus* |
| 75 | TRINITY_DN482_c0_g1 | 31413 | | 0 | 11.91885276 | **LAAO** |  |  | ABN72539 | *Bungarus multicinctus* |
| 76 | TRINITY_DN482_c0_g1 | 25309 | | 0 | 11.91885276 | **LAAO** |  |  | ABN72539 | *Bungarus multicinctus* |
| 77 | TRINITY_DN482_c0_g1 | 15695 | | 0 | 11.91885276 | **LAAO** |  |  | ABN72539 | *Bungarus multicinctus* |
| 78 | TRINITY_DN482_c0_g1 | 2605 | | 6 | 11.91885276 | **LAAO** |  |  | ABN72540 | *Bungarus fasciatus* |
| 79 | TRINITY_DN482_c0_g1 | 1701 | | 0 | 11.91885276 | **LAAO** |  |  | ABN72539 | *Bungarus multicinctus* |
| 80 | TRINITY_DN8453_c0_g1 | 1018 | | 0 | 12.2197909 | **LAAO** |  |  | JAA74636 | *Acanthophis wellsi* |
| 81 | TRINITY_DN5468_c0_g1 | 585 | | 0 | 9.800504988 | **LAAO** |  |  | XP_026549343 | *Notechis scutatus* |
| 82 | TRINITY_DN5468_c0_g1 | 364 | | 0 | 9.800504988 | **LAAO** |  |  | XP_026549343 | *Notechis scutatus* |
| 83 | TRINITY_DN8453_c0_g1 | 315 | | 0 | 12.2197909 | **LAAO** |  |  | ABN72539 | *Bungarus multicinctus* |
| 84 | TRINITY_DN5468_c0_g1 | 168 | | 0 | 9.800504988 | **LAAO** |  |  | XP_026549343 | *Notechis scutatus* |
| 85 | TRINITY_DN7590_c3_g1 | 105 | | 0 | 8.553575351 | **LAAO** |  |  | ABN72540 | *Bungarus fasciatus* |
| 86 | TRINITY_DN450_c0_g1 | 441365 | | 0 | 11.86948642 | Natriuretic peptide |  | **3.59%** | ADK12002 | *Ophiophagus hannah* |
| 87 | TRINITY_DN450_c0_g1 | 123771 | | 0 | 11.86948642 | Natriuretic peptide |  |  | ADK12002 | *Ophiophagus hannah* |
| 88 | TRINITY_DN450_c0_g1 | 20717 | | 500 | 11.86948642 | Natriuretic peptide |  |  | JAS05144 | *Micrurus tener* |
| 89 | TRINITY_DN14642_c0_g1 | 122 | | 0 | 8.816609757 | Natriuretic peptide |  |  | ADK12002 | *Ophiophagus hannah* |
| 90 | TRINITY_DN676_c2_g1 | 52057 | | 10 | 7.763338989 | **NGF** |  | **0.35%** | AAB25729 | *Bungarus multicinctus* |
| 91 | TRINITY_DN676_c2_g1 | 2092 | | 828 | 7.763338989 | **NGF** |  |  | ABA60118 | *Pseudonaja textilis* |
| 92 | TRINITY_DN676_c2_g1 | 1839 | | 0 | 7.763338989 | **NGF** |  |  | AAB25729 | *Bungarus multicinctus* |
| 93 | TRINITY_DN676_c2_g1 | 611 | | 0 | 7.763338989 | **NGF** |  |  | AAB25729 | *Bungarus multicinctus* |
| 94 | TRINITY_DN676_c2_g1 | 296 | | 0 | 7.763338989 | **NGF** |  |  | AAB25729 | *Bungarus multicinctus* |
| 95 | TRINITY_DN2739_c1_g1 | 238 | | 9 | 4.460457436 | **NGF** |  |  | ETE72246 | *Ophiophagus hannah* |
| 96 | TRINITY_DN676_c2_g1 | 121 | | 0 | 7.763338989 | **NGF** |  |  | AAB25729 | *Bungarus multicinctus* |
| 97 | TRINITY_DN117_c4_g1 | 1317114 | | 0 | 13.23957567 | PLA2 |  | **24.71%** | ADF50037 | *Bungarus flaviceps* |
| 98 | TRINITY_DN117_c4_g1 | 1210811 | | 0 | 13.23957567 | PLA2 |  |  | ACY68711 | *Parasuta nigriceps* |
| 99 | TRINITY_DN117_c4_g1 | 821053 | | 0 | 13.23957567 | PLA2 |  |  | AAB24834 | *Bungarus fasciatus* |
| 100 | TRINITY_DN491_c1_g1 | 625587 | | 0 | 21.09418084 | PLA2 |  |  | BAC77655 | *Bungarus flaviceps* |
| 101 | TRINITY_DN117_c4_g1 | 31520 | | 0 | 13.23957567 | PLA2 |  |  | ADF50037 | *Bungarus flaviceps* |
| 102 | TRINITY_DN117_c4_g1 | 18769 | | 2 | 13.23957567 | PLA2 |  |  | ACY68711 | *Parasuta nigriceps* |
| 103 | TRINITY_DN117_c4_g1 | 5123 | | 0 | 13.23957567 | PLA2 |  |  | ACY68711 | *Parasuta nigriceps* |
| 104 | TRINITY_DN117_c4_g1 | 1819 | | 0 | 13.23957567 | PLA2 |  |  | ADF50037 | *Bungarus flaviceps* |
| 105 | TRINITY_DN117_c4_g1 | 721 | | 0 | 13.23957567 | PLA2 |  |  | ADF50037 | *Bungarus flaviceps* |
| 106 | TRINITY_DN117_c4_g1 | 473 | | 191 | 13.23957567 | PLA2 |  |  | AAB24834 | *Bungarus fasciatus* |
| 107 | TRINITY_DN7320_c0_g1 | 111 | | 0 | 3.207965507 | PLA2 |  |  | LAA68819 | *Micrurus lemniscatus* |
| 108 | TRINITY_DN117_c4_g1 | 106 | | 2 | 13.23957567 | PLA2 |  |  | ACY68711 | *Parasuta nigriceps* |
| 109 | TRINITY_DN839_c0_g1 | 22859 | | 0 | 8.996722072 | PLB |  | **0.38%** | ACR78473 | *Drysdalia coronoides* |
| 110 | TRINITY_DN839_c0_g1 | 19491 | | 29 | 8.996722072 | PLB |  |  | XP_026553321 | *Pseudonaja textilis* |
| 111 | TRINITY_DN839_c0_g1 | 16903 | | 289 | 8.996722072 | PLB |  |  | ACR78473 | *Drysdalia coronoides* |
| 112 | TRINITY_DN839_c0_g1 | 2787 | | 6 | 8.996722072 | PLB |  |  | ACR78473 | *Drysdalia coronoides* |
| 113 | TRINITY_DN839_c0_g1 | 310 | | 27 | 8.996722072 | PLB |  |  | ACR78473 | *Drysdalia coronoides* |
| 114 | TRINITY_DN839_c0_g1 | 152 | | 0 | 8.996722072 | PLB |  |  | ACR78473 | *Drysdalia coronoides* |
| 115 | TRINITY_DN3739_c0_g1 | 23304 | | 0 | 6.272974363 | Serine Protease |  | **0.14%** | JAB54462 | *Micrurus fulvius* |
| 116 | TRINITY_DN9079_c4_g1 | 100 | | 0 | 4.318947707 | Serine Protease |  |  | XP_026562705 | *Pseudonaja textilis* |
| 117 | TRINITY_DN7325_c0_g1 | 285459 | | 0 | 22.11784508 | Type-I α-neurotoxin | **61.82%** | **32.15%** | CAA45882 | *Bungarus multicinctus* |
| 118 | TRINITY_DN7325_c0_g1 | 262 | | 0 | 22.11784508 | Type-I α-neurotoxin |  |  | CAA45882 | *Bungarus multicinctus* |
| 119 | TRINITY_DN323_c0_g1 | 423609 | | 0 | 18.75215495 | Type-I α-neurotoxin |  |  | CAA06887 | *Bungarus multicinctus multicinctus* |
| 120 | TRINITY_DN323_c0_g1 | 94604 | | 0 | 18.75215495 | Type-I α-neurotoxin |  |  | CAA06887 | *Bungarus multicinctus multicinctus* |
| 121 | TRINITY_DN9940_c0_g1 | 39442 | | 0 | 16.07062565 | Type-I α-neurotoxin |  |  | ETE58964 | *Ophiophagus hannah* |
| 122 | TRINITY_DN323_c0_g1 | 17856 | | 0 | 18.75215495 | Type-I α-neurotoxin |  |  | CAA06887 | *Bungarus multicinctus multicinctus* |
| 123 | TRINITY_DN323_c0_g1 | 7722 | | 0 | 18.75215495 | Type-I α-neurotoxin |  |  | CAA06887 | *Bungarus multicinctus multicinctus* |
| 124 | TRINITY_DN1005_c0_g1 | 1085 | | 0 | 6.560991216 | Type-I α-neurotoxin |  |  | P82462 | *Ophiophagus hannah* |
| 125 | TRINITY_DN1005_c0_g1 | 691 | | 0 | 6.560991216 | Type-I α-neurotoxin |  |  | P82462 | *Ophiophagus hannah* |
| 126 | TRINITY_DN1005_c0_g1 | 258 | | 0 | 6.560991216 | Type-I α-neurotoxin |  |  | P82462 | *Ophiophagus hannah* |
| 127 | TRINITY_DN1005_c0_g1 | 140 | | 0 | 6.560991216 | Type-I α-neurotoxin |  |  | P82462 | *Ophiophagus hannah* |
| 128 | TRINITY_DN323_c0_g1 | 1018919 | | 0 | 18.75215495 | Type-II α-neurotoxin |  |  | ETE56306 | *Ophiophagus hannah* |
| 129 | TRINITY_DN1485_c0_g1 | 975145 | | 0 | 21.29056926 | Type-II α-neurotoxin |  |  | CAJ77819 | *Bungarus candidus* |
| 130 | TRINITY_DN1364_c0_g1 | 367500 | | 0 | 20.3818592 | Type-II α-neurotoxin |  |  | LAA95127 | *Micrurus lemniscatus lemniscatus* |
| 131 | TRINITY_DN1364_c0_g1 | 945 | | 0 | 20.3818592 | Type-II α-neurotoxin |  |  | LAA95127 | *Micrurus lemniscatus lemniscatus* |
| 132 | TRINITY_DN45054_c0_g1 | 527 | | 0 | 12.43272096 | Type-II α-neurotoxin |  |  | ABK63538 | *Tropidechis carinatus* |
| 133 | TRINITY_DN1364_c0_g1 | 423 | | 0 | 20.3818592 | Type-II α-neurotoxin |  |  | LAA95127 | *Micrurus lemniscatus lemniscatus* |
| 134 | TRINITY_DN1364_c0_g1 | 3910 | | 0 | 20.3818592 | Type-II α-neurotoxin |  |  | XP_026559448 | *Pseudonaja textilis* |
| 135 | TRINITY_DN1364_c0_g1 | 2291 | | 0 | 20.3818592 | Type-II α-neurotoxin |  |  | ETE56205 | *Ophiophagus hannah* |
| 136 | TRINITY_DN2007_c2_g1 | 891 | | 0 | 13.08488254 | Type-II α-neurotoxin |  |  | ETE56205 | *Ophiophagus hannah* |
| 137 | TRINITY_DN2007_c2_g1 | 581 | | 0 | 13.08488254 | Type-II α-neurotoxin |  |  | ETE56205 | *Ophiophagus hannah* |
| 138 | TRINITY_DN1364_c0_g2 | 478 | | 0 | 19.29174805 | Type-II α-neurotoxin |  |  | AAL30059 | *Bungarus candidus* |
| 139 | TRINITY_DN7325_c0_g1 | 330 | | 0 | 22.11784508 | Type-II α-neurotoxin |  |  | ETE56205 | *Ophiophagus hannah* |
| 140 | TRINITY_DN2007_c2_g1 | 301 | | 0 | 13.08488254 | Type-II α-neurotoxin |  |  | ETE56205 | *Ophiophagus hannah* |
| 141 | TRINITY_DN6859_c0_g1 | 236 | | 0 | 10.77596777 | Type-II α-neurotoxin |  |  | ETE56205 | *Ophiophagus hannah* |
| 142 | TRINITY_DN7325_c0_g1 | 224 | | 0 | 22.11784508 | Type-II α-neurotoxin |  |  | ETE56205 | *Ophiophagus hannah* |
| 143 | TRINITY_DN7325_c0_g1 | 148 | | 0 | 22.11784508 | Type-II α-neurotoxin |  |  | ETE56205 | *Ophiophagus hannah* |
| 144 | TRINITY_DN1485_c0_g1 | 825527 | | 0 | 21.29056926 | Unconventional 3FTx | **18.01%** |  | CAA06885 | *Bungarus multicinctus* |
| 145 | TRINITY_DN323_c0_g1 | 109907 | | 0 | 18.75215495 | Unconventional 3FTx |  |  | CAB53358 | *Bungarus multicinctus* |
| 146 | TRINITY_DN9940_c0_g1 | 9212 | | 4 | 16.07062565 | Unconventional 3FTx |  |  | LAB55960 | *Micrurus surinamensis* |
| 147 | TRINITY_DN1485_c2_g2 | 277 | | 0 | 10.10142468 | Unconventional 3FTx |  |  | XP_032078231 | *Thamnophis elegans* |
| 148 | TRINITY_DN24809_c0_g1 | 242 | | 0 | 9.75819307 | Unconventional 3FTx |  |  | CAD56381 | *Bungarus multicinctus* |
| 149 | TRINITY_DN1485_c0_g1 | 1015208 | | 0 | 21.29056926 | κ-bungarotoxin | **20.17%** |  | CAA35775 | *Bungarus multicinctus* |
| 150 | TRINITY_DN9940_c0_g2 | 25651 | | 0 | 16.48605728 | κ-bungarotoxin |  |  | AAL30054 | *Bungarus candidus* |
| 151 | TRINITY_DN1485_c0_g1 | 11382 | | 0 | 21.29056926 | κ-bungarotoxin |  |  | CAB46659 | *Bungarus multicinctus* |
| 152 | TRINITY_DN1485_c0_g1 | 4741 | | 0 | 21.29056926 | κ-bungarotoxin |  |  | CAB46659 | *Bungarus multicinctus* |
| 153 | TRINITY_DN1485_c0_g1 | 869 | | 0 | 21.29056926 | κ-bungarotoxin |  |  | CAB46659 | *Bungarus multicinctus* |
| 154 | TRINITY_DN2007_c2_g1 | 376 | | 0 | 13.08488254 | κ-bungarotoxin |  |  | CAA35775 | *Bungarus multicinctus* |
| 155 | TRINITY_DN2007_c2_g1 | 117 | | 0 | 13.08488254 | κ-bungarotoxin |  |  | CAA35775 | *Bungarus multicinctus* |
| 156 | TRINITY_DN66_c0_g1 | 122728 | | 16 | 9.076857398 | VEGF |  | **1.12%** | JAA74898 | *Pseudonaja modesta* |
| 157 | TRINITY_DN66_c0_g1 | 33393 | | 0 | 9.076857398 | VEGF |  |  | XP_026551346 | *Pseudonaja textilis* |
| 158 | TRINITY_DN66_c0_g1 | 12007 | | 11 | 9.076857398 | VEGF |  |  | XP_026551344 | *Pseudonaja textilis* |
| 159 | TRINITY_DN14480_c0_g1 | 6882 | | 56 | 6.555882396 | VEGF |  |  | LAB17921 | *Micrurus spixii* |
| 160 | TRINITY_DN66_c0_g1 | 3766 | | 0 | 9.076857398 | VEGF |  |  | XP_026551346 | *Pseudonaja textilis* |
| 161 | TRINITY_DN66_c0_g1 | 1699 | | 0 | 9.076857398 | VEGF |  |  | XP_026551346 | *Pseudonaja textilis* |
| 162 | TRINITY_DN66_c0_g1 | 1617 | | 0 | 9.076857398 | VEGF |  |  | XP_026551346 | *Pseudonaja textilis* |
| 163 | TRINITY_DN12448_c0_g1 | 256 | | 0 | 6.502128806 | VEGF |  |  | XP_026558913 | *Pseudonaja textilis* |
| 164 | TRINITY_DN14480_c0_g1 | 228 | | 0 | 6.555882396 | VEGF |  |  | LAB17921 | *Micrurus spixii* |
| 165 | TRINITY_DN12959_c0_g4 | 161 | | 14 | 5.198653474 | VEGF |  |  | LAA79011 | *Micrurus lemniscatus* |
| 166 | TRINITY_DN14480_c0_g1 | 143 | | 0 | 6.555882396 | VEGF |  |  | XP_026555459 | *Pseudonaja textilis* |
| 167 | TRINITY_DN12448_c0_g1 | 113 | | 13 | 6.502128806 | VEGF |  |  | XP_026558913 | *Pseudonaja textilis* |
| 168 | TRINITY_DN1325_c0_g1 | 110738 | | 0 | 18.62453962 | Vespryn |  | **0.68%** | XP_026576508 | *Pseudonaja textilis* |
| 169 | TRINITY_DN1076_c0_g2 | 2862 | | 0 | 13.32867607 | β-bungarotoxin |  | **8.80%** | AAL30067.1 | *Furina ornata* |
| 170 | TRINITY_DN768_c1_g1 | 710088 | | 0 | 9.915452031 | β-bungarotoxin |  |  | BAD06267 | *Bungarus candidus* |
| 171 | TRINITY_DN768_c1_g1 | 389230 | | 0 | 9.915452031 | β-bungarotoxin |  |  | BAD06267 | *Bungarus candidus* |
| 172 | TRINITY_DN768_c1_g1 | 200648 | | 492 | 9.915452031 | β-bungarotoxin |  |  | BAD06267 | *Bungarus candidus* |
| 173 | TRINITY_DN567_c0_g1 | 112704 | | 27 | 13.81824703 | β-bungarotoxin |  |  | AAL30065.1 | *Furina ornata* |
| 174 | TRINITY_DN11210_c1_g1 | 18577 | | 0 | 11.61691136 | β-bungarotoxin |  |  | CAA72809 | *Bungarus multicinctus* |
| 175 | TRINITY_DN4436_c0_g1 | 1861 | | 0 | 14.57255705 | β-bungarotoxin |  |  | CAB62503 | *Bungarus multicinctus* |

Each toxin family and the corresponding relative abundance is uniquely colour coded.

**Table S12.** Venom gland toxin transcripts from *Bungarus romulusi*.

| S. No. | **Isoform** | **VG** | **Int** | **M** | **Family** | **Subtype** | **Abundance** | **Accession** | **Scientific Name** |
| --- | --- | --- | --- | --- | --- | --- | --- | --- | --- |
| 1 | TRINITY_DN2049_c0_g2_i4 | 31860 | 304 | 5.235644987 | 5'-nucleotidase |  | **0.44%** | JAB52815 | *Micrurus fulvius* |
| 2 | TRINITY_DN19829_c0_g1_i3 | 135 | 23 | 5.235644987 | 5'-nucleotidase |  |  | XP_026522489 | *Notechis scutatus* |
| 3 | TRINITY_DN2049_c0_g2_i1 | 103 | 2 | 13.57174515 | 5'-nucleotidase |  |  | XP_026582322 | *Pseudonaja textilis* |
| 4 | TRINITY_DN359_c0_g1_i12 | 45074 | 0 | 13.57174515 | Acetylcholinesterase |  | **0.96%** | AAC59905 | *Bungarus fasciatus* |
| 5 | TRINITY_DN359_c0_g1_i3 | 20202 | 0 | 13.57174515 | Acetylcholinesterase |  |  | AAC59905 | *Bungarus fasciatus* |
| 6 | TRINITY_DN359_c0_g1_i9 | 2177 | 8 | 13.57174515 | Acetylcholinesterase |  |  | AAC59905 | *Bungarus fasciatus* |
| 7 | TRINITY_DN359_c0_g1_i7 | 1295 | 0 | 13.57174515 | Acetylcholinesterase |  |  | AAC59905 | *Bungarus fasciatus* |
| 8 | TRINITY_DN359_c0_g1_i1 | 1261 | 0 | 13.57174515 | Acetylcholinesterase |  |  | Q92035 | *Bungarus fasciatus* |
| 9 | TRINITY_DN359_c0_g1_i6 | 884 | 0 | 2.867876439 | Acetylcholinesterase |  |  | AAC59905 | *Bungarus fasciatus* |
| 10 | TRINITY_DN356_c3_g1_i2 | 42031 | 7828 | 3.367052312 | Calreticulin |  | **0.57%** | JAB54560 | *Micrurus fulvius* |
| 11 | TRINITY_DN49_c0_g1_i3 | 361934 | 10 | 3.367052312 | CRISP |  | **9.67%** | ACE73577 | *Bungarus candidus* |
| 12 | TRINITY_DN49_c0_g1_i4 | 344200 | 0 | 3.367052312 | CRISP |  |  | ACE73577 | *Bungarus candidus* |
| 13 | TRINITY_DN49_c0_g1_i6 | 5624 | 0 | 3.367052312 | CRISP |  |  | ACE73577 | *Bungarus candidus* |
| 14 | TRINITY_DN49_c0_g1_i7 | 567 | 0 | 3.367052312 | CRISP |  |  | ACE73577 | *Bungarus candidus* |
| 15 | TRINITY_DN49_c0_g1_i12 | 491 | 6 | 3.367052312 | CRISP |  |  | ACE73577 | *Bungarus candidus* |
| 16 | TRINITY_DN49_c0_g1_i1 | 236 | 0 | 10.74274075 | CRISP |  |  | ACE73577 | *Bungarus candidus* |
| 17 | TRINITY_DN755_c0_g2_i1 | 12517 | 10 | 15.13281705 | CTL |  | **0.39%** | XP_026579605 | *Pseudonaja textilis* |
| 18 | TRINITY_DN755_c0_g1_i2 | 10401 | 0 | 15.13281705 | CTL |  |  | ABP94094 | *Oxyuranus scutellatus* |
| 19 | TRINITY_DN755_c0_g1_i8 | 1665 | 0 | 15.13281705 | CTL |  |  | ABU68499 | *Leioheterodon madagascariensis* |
| 20 | TRINITY_DN755_c0_g1_i3 | 1340 | 0 | 15.13281705 | CTL |  |  | ABP94094 | *Oxyuranus scutellatus* |
| 21 | TRINITY_DN755_c0_g1_i13 | 1045 | 0 | 6.500581099 | CTL |  |  | JAC95003 | *Pantherophis guttatus* |
| 22 | TRINITY_DN1070_c0_g2_i2 | 450 | 7 | 9.111279121 | CTL |  |  | ADF50042 | *Bungarus flaviceps* |
| 23 | TRINITY_DN21017_c0_g1_i2 | 380 | 1 | 15.13281705 | CTL |  |  | XP_026527775 | *Notechis scutatus* |
| 24 | TRINITY_DN755_c0_g1_i11 | 271 | 0 | 15.13281705 | CTL |  |  | ABP94094 | *Oxyuranus scutellatus* |
| 25 | TRINITY_DN755_c0_g1_i5 | 260 | 0 | 2.575058162 | CTL |  |  | ABP94094 | *Oxyuranus scutellatus* |
| 26 | TRINITY_DN732_c0_g1_i8 | 181 | 74 | 8.410294102 | CTL |  |  | XP_026546618 | *Notechis scutatus* |
| 27 | TRINITY_DN1070_c0_g3_i2 | 144 | 0 | 15.13281705 | CTL |  |  | ABP94128 | *Pseudechis porphyriacus* |
| 28 | TRINITY_DN755_c0_g1_i7 | 132 | 0 | 10.61063388 | CTL |  |  | ABP94118 | *Notechis scutatus* |
| 29 | TRINITY_DN307_c0_g1_i10 | 96468 | 0 | 10.61063388 | Disintegrin-like |  | **5.60%** | ABN72537 | *Bungarus multicinctus* |
| 30 | TRINITY_DN307_c0_g1_i8 | 56832 | 0 | 16.94513734 | Disintegrin-like |  |  | ABN72537 | *Bungarus multicinctus* |
| 31 | TRINITY_DN863_c0_g1_i1 | 49210 | 0 | 10.61063388 | Disintegrin-like |  |  | ABN72537 | *Bungarus multicinctus* |
| 32 | TRINITY_DN307_c0_g1_i6 | 44473 | 0 | 10.61063388 | Disintegrin-like |  |  | ABN72537 | *Bungarus multicinctus* |
| 33 | TRINITY_DN307_c0_g1_i13 | 41782 | 0 | 10.61063388 | Disintegrin-like |  |  | ABN72537 | *Bungarus multicinctus* |
| 34 | TRINITY_DN307_c0_g1_i12 | 30232 | 0 | 10.61063388 | Disintegrin-like |  |  | ABQ01136 | *Oxyuranus scutellatus* |
| 35 | TRINITY_DN307_c0_g1_i9 | 27679 | 0 | 10.61063388 | Disintegrin-like |  |  | ABN72537 | *Bungarus multicinctus* |
| 36 | TRINITY_DN307_c0_g1_i5 | 26854 | 0 | 14.95171343 | Disintegrin-like |  |  | ABN72537 | *Bungarus multicinctus* |
| 37 | TRINITY_DN474_c0_g3_i1 | 10637 | 0 | 14.54386448 | Disintegrin-like |  |  | ABQ01138 | *Notechis scutatus* |
| 38 | TRINITY_DN2689_c0_g1_i2 | 8523 | 0 | 10.61063388 | Disintegrin-like |  |  | ABQ01138 | *Notechis scutatus* |
| 39 | TRINITY_DN307_c0_g1_i7 | 4275 | 287 | 16.94513734 | Disintegrin-like |  |  | ABQ01136 | *Oxyuranus scutellatus* |
| 40 | TRINITY_DN863_c0_g1_i3 | 4091 | 0 | 13.207235 | Disintegrin-like |  |  | ABN72536 | *Bungarus fasciatus* |
| 41 | TRINITY_DN1500_c0_g1_i1 | 4003 | 0 | 14.95171343 | Disintegrin-like |  |  | ABN72537 | *Bungarus multicinctus* |
| 42 | TRINITY_DN474_c0_g3_i2 | 2776 | 0 | 12.07484015 | Disintegrin-like |  |  | AAM51550 | *Naja mossambica* |
| 43 | TRINITY_DN16430_c0_g3_i1 | 1826 | 0 | 11.79591687 | Disintegrin-like |  |  | AXL96555 | *Borikenophis portoricensis* |
| 44 | TRINITY_DN16430_c0_g2_i1 | 1505 | 0 | 6.7682172 | Disintegrin-like |  |  | ABN72537 | *Bungarus multicinctus* |
| 45 | TRINITY_DN10280_c0_g1_i5 | 492 | 8 | 10.61063388 | Disintegrin-like |  |  | XP_026536946 | *Notechis scutatus* |
| 46 | TRINITY_DN307_c0_g1_i2 | 290 | 0 | 5.25304303 | Disintegrin-like |  |  | ABN72537 | *Bungarus multicinctus* |
| 47 | TRINITY_DN2668_c0_g1_i3 | 186 | 125 | 5.020752147 | Disintegrin-like |  |  | XP_013912183 | *Thamnophis sirtalis* |
| 48 | TRINITY_DN99187_c0_g1_i1 | 166 | 7 | 6.430347561 | Disintegrin-like |  |  | XP_029141633 | *Protobothrops mucrosquamatus* |
| 49 | TRINITY_DN863_c0_g3_i1 | 126 | 2 | 16.94513734 | Disintegrin-like |  |  | XP_013922279 | *Thamnophis sirtalis* |
| 50 | TRINITY_DN863_c0_g1_i2 | 107 | 0 | 7.940808819 | Disintegrin-like |  |  | ABN72537 | *Bungarus multicinctus* |
| 51 | TRINITY_DN9869_c0_g1_i1 | 104 | 0 | 17.41398768 | Disintegrin-like |  |  | XP_026527224 | *Notechis scutatus* |
| 52 | TRINITY_DN72_c0_g4_i7 | 72237 | 0 | 17.19726702 | Hyaluronidase |  | **0.98%** | XP_026524834 | *Notechis scutatus* |
| 53 | TRINITY_DN57_c15_g1_i1 | 37682 | 0 | 17.19726702 | Kunitz |  | **1.05%** | ACR78501 | *Drysdalia coronoides* |
| 54 | TRINITY_DN57_c15_g1_i2 | 24636 | 0 | 15.11285161 | Kunitz |  |  | ACR78501 | *Drysdalia coronoides* |
| 55 | TRINITY_DN7705_c0_g1_i1 | 14998 | 0 | 16.05485143 | Kunitz |  |  | ACR78501 | *Drysdalia coronoides* |
| 56 | TRINITY_DN95585_c0_g1_i1 | 165 | 153 | 13.78907201 | Kunitz |  |  | XP_026579404 | *Pseudonaja textilis* |
| 57 | TRINITY_DN830_c0_g1_i13 | 58549 | 2 | 13.78907201 | **LAAO** |  | **2.10%** | ABN72539 | *Bungarus multicinctus* |
| 58 | TRINITY_DN830_c0_g1_i12 | 48369 | 0 | 13.78907201 | **LAAO** |  |  | ABN72539 | *Bungarus multicinctus* |
| 59 | TRINITY_DN830_c0_g1_i3 | 33498 | 0 | 13.78907201 | **LAAO** |  |  | ABN72539 | *Bungarus multicinctus* |
| 60 | TRINITY_DN830_c0_g1_i22 | 6188 | 0 | 13.78907201 | **LAAO** |  |  | ABN72539 | *Bungarus multicinctus* |
| 61 | TRINITY_DN830_c0_g1_i7 | 2554 | 0 | 13.78907201 | **LAAO** |  |  | ABN72539 | *Bungarus multicinctus* |
| 62 | TRINITY_DN830_c0_g1_i6 | 2385 | 0 | 13.78907201 | **LAAO** |  |  | ABN72539 | *Bungarus multicinctus* |
| 63 | TRINITY_DN830_c0_g1_i14 | 1797 | 0 | 13.78907201 | **LAAO** |  |  | ABN72539 | *Bungarus multicinctus* |
| 64 | TRINITY_DN830_c0_g1_i19 | 1420 | 0 | 13.78907201 | **LAAO** |  |  | ABN72539 | *Bungarus multicinctus* |
| 65 | TRINITY_DN830_c0_g1_i17 | 194 | 1 | 13.78907201 | **LAAO** |  |  | ABN72539 | *Bungarus multicinctus* |
| 66 | TRINITY_DN830_c0_g1_i9 | 119 | 0 | 7.118403555 | **LAAO** |  |  | ABN72539 | *Bungarus multicinctus* |
| 67 | TRINITY_DN397_c1_g4_i1 | 3451 | 34 | 8.104433595 | Natriuretic peptide |  | **1.49%** | JAS05144 | *Micrurus tener* |
| 68 | TRINITY_DN57_c0_g1_i16 | 103484 | 0 | 8.104433595 | Natriuretic peptide |  |  | ADK12002 | *Ophiophagus hannah* |
| 69 | TRINITY_DN57_c0_g1_i7 | 2605 | 10 | 9.488296614 | Natriuretic peptide |  |  | ADK12002 | *Ophiophagus hannah* |
| 70 | TRINITY_DN8160_c1_g1_i1 | 304 | 0 | 12.42271112 | Natriuretic peptide |  |  | ADK12002 | *Ophiophagus hannah* |
| 71 | TRINITY_DN4045_c0_g3_i1 | 27478 | 7 | 6.136764092 | **NGF** |  | **0.38%** | AAB25729 | *Bungarus multicinctus* |
| 72 | TRINITY_DN1317_c1_g3_i1 | 257 | 5 | 19.70949826 | **NGF** |  |  | ETE59033 | *Ophiophagus hannah* |
| 73 | TRINITY_DN744_c0_g1_i2 | 1.00E+06 | 1 | 21.27715236 | PLA2 |  | **29.42%** | 0702209A | *Bungarus multicinctus* |
| 74 | TRINITY_DN134_c0_g1_i7 | 373525 | 1 | 19.70949826 | PLA2 |  |  | AAR19228 | *Bungarus caeruleus* |
| 75 | TRINITY_DN744_c0_g1_i7 | 295169 | 0 | 21.27715236 | PLA2 |  |  | 0702209A | *Bungarus multicinctus* |
| 76 | TRINITY_DN134_c0_g1_i3 | 219678 | 0 | 19.70949826 | PLA2 |  |  | ADF50037 | *Bungarus flaviceps* |
| 77 | TRINITY_DN744_c0_g1_i4 | 187297 | 0 | 21.27715236 | PLA2 |  |  | CAA37482 | *Bungarus multicinctus* |
| 78 | TRINITY_DN134_c0_g1_i13 | 28675 | 0 | 15.91273919 | PLA2 |  |  | P0C551 | *Bungarus candidus* |
| 79 | TRINITY_DN11387_c0_g2_i1 | 26111 | 0 | 21.27715236 | PLA2 |  |  | CAA37482 | *Bungarus multicinctus* |
| 80 | TRINITY_DN134_c0_g1_i12 | 13816 | 0 | 21.27715236 | PLA2 |  |  | ADF50037 | *Bungarus flaviceps* |
| 81 | TRINITY_DN134_c0_g1_i2 | 8977 | 0 | 8.865637485 | PLA2 |  |  | BAD06270 | *Bungarus candidus* |
| 82 | TRINITY_DN602_c0_g1_i3 | 4618 | 0 | 13.26377694 | PLA2 |  |  | BAD06270 | *Bungarus candidus* |
| 83 | TRINITY_DN42_c0_g2_i1 | 4163 | 0 | 12.82720889 | PLA2 |  |  | ETE56084 | *Ophiophagus hannah* |
| 84 | TRINITY_DN5510_c0_g1_i1 | 3076 | 0 | 12.60996645 | PLA2 |  |  | AAO84769 | *Bungarus candidus* |
| 85 | TRINITY_DN42_c5_g1_i2 | 2621 | 0 | 21.27715236 | PLA2 |  |  | LAA68819 | *Micrurus lemniscatus lemniscatus* |
| 86 | TRINITY_DN134_c0_g1_i4 | 431 | 0 | 10.37222606 | PLA2 |  |  | ADF50037 | *Bungarus flaviceps* |
| 87 | TRINITY_DN7462_c0_g2_i1 | 360 | 0 | 10.37222606 | PLA2 |  |  | LAA39990 | *Micrurus corallinus* |
| 88 | TRINITY_DN7630_c0_g1_i3 | 166 | 101 | 6.765950593 | PLA2 |  |  | XP_026554534 | *Pseudonaja textilis* |
| 89 | TRINITY_DN40744_c0_g1_i1 | 159 | 2 | 9.034784967 | PLA2 |  |  | ETE56084 | *Ophiophagus hannah* |
| 90 | TRINITY_DN3776_c0_g1_i2 | 141 | 0 | 11.57910548 | PLA2 |  |  | 0702209A | *Bungarus multicinctus* |
| 91 | TRINITY_DN462_c0_g1_i1 | 143 | 0 | 12.45306764 | PLB |  | **0.00%** | BAN82156 | *Ovophis okinavensis* |
| 92 | TRINITY_DN1122_c0_g2_i1 | 3945 | 0 | 12.45306764 | Serine protease |  | **0.06%** | ABN72545 | *Bungarus multicinctus* |
| 93 | TRINITY_DN1122_c0_g2_i4 | 119 | 1 | 13.29021765 | Serine protease |  |  | ETE72364 | *Ophiophagus hannah* |
| 94 | TRINITY_DN0_c1_g3_i2 | 4177 | 0 | 6.42194512 | Type-I α-neurotoxin | **86.67%** | **35.90%** | JAB52865 | *Micrurus fulvius* |
| 95 | TRINITY_DN608_c1_g1_i4 | 362 | 0 | 18.52072304 | Type-I α-neurotoxin |  |  | P82462 | *Naja atra* |
| 96 | TRINITY_DN203_c3_g1_i1 | 158749 | 0 | 7.88101313 | Type-I α-neurotoxin |  |  | CAA06887 | *Bungarus multicinctus multicinctus* |
| 97 | TRINITY_DN89_c5_g1_i2 | 734221 | 1 | 10.25102086 | Type-II α-neurotoxin |  |  | ETE56306 | *Ophiophagus hannah* |
| 98 | TRINITY_DN9485_c1_g1_i5 | 311337 | 0 | 17.84029405 | Type-II α-neurotoxin |  |  | ETE56306 | *Ophiophagus hannah* |
| 99 | TRINITY_DN0_c1_g1_i6 | 246022 | 2 | 10.25102086 | Type-II α-neurotoxin |  |  | CAJ77818 | *Bungarus candidus* |
| 100 | TRINITY_DN9485_c1_g1_i1 | 163857 | 0 | 7.88101313 | Type-II α-neurotoxin |  |  | ETE56306 | *Ophiophagus hannah* |
| 101 | TRINITY_DN89_c5_g1_i5 | 99045 | 5 | 17.84029405 | Type-II α-neurotoxin |  |  | AHZ08825 | *Micropechis ikaheca* |
| 102 | TRINITY_DN0_c1_g1_i4 | 35852 | 0 | 15.76405356 | Type-II α-neurotoxin |  |  | CAJ77818 | *Bungarus candidus* |
| 103 | TRINITY_DN8116_c0_g2_i2 | 23553 | 0 | 7.88101313 | Type-II α-neurotoxin |  |  | ETE56306 | *Ophiophagus hannah* |
| 104 | TRINITY_DN89_c5_g1_i8 | 11451 | 0 | 17.84029405 | Type-II α-neurotoxin |  |  | AHZ08825 | *Micropechis ikaheca* |
| 105 | TRINITY_DN0_c1_g1_i5 | 9023 | 0 | 10.25102086 | Type-II α-neurotoxin |  |  | CAJ77818 | *Bungarus candidus* |
| 106 | TRINITY_DN9485_c1_g1_i2 | 4474 | 0 | 17.84029405 | Type-II α-neurotoxin |  |  | ETE56306 | *Ophiophagus hannah* |
| 107 | TRINITY_DN0_c1_g1_i1 | 1897 | 0 | 18.09317133 | Type-II α-neurotoxin |  |  | CAJ77818 | *Bungarus candidus* |
| 108 | TRINITY_DN205_c0_g2_i4 | 939 | 0 | 8.955479842 | Type-II α-neurotoxin |  |  | AAB87417 | *Naja sputatrix* |
| 109 | TRINITY_DN0_c0_g1_i3 | 481357 | 0 | 14.64856614 | Type-II α-neurotoxin |  |  | AAL30059 | *Bungarus candidus* |
| 110 | TRINITY_DN525_c0_g1_i6 | 7708 | 0 | 8.955479842 | Type-II α-neurotoxin |  |  | ETE56205 | *Ophiophagus hannah* |
| 111 | TRINITY_DN0_c0_g1_i19 | 144778 | 0 | 17.85990133 | Unconventional 3FTx | **11.70%** |  | AAD41806 | *Bungarus multicinctus* |
| 112 | TRINITY_DN93_c0_g1_i3 | 96349 | 0 | 8.955479842 | Unconventional 3FTx |  |  | ETE56933 | *Ophiophagus hannah* |
| 113 | TRINITY_DN0_c0_g1_i4 | 66221 | 0 | 17.85990133 | Unconventional 3FTx |  |  | AAD41806 | *Bungarus multicinctus* |
| 114 | TRINITY_DN93_c0_g1_i2 | 2317 | 0 | 17.66841843 | Unconventional 3FTx |  |  | ETE56933 | *Ophiophagus hannah* |
| 115 | TRINITY_DN430_c0_g1_i3 | 43017 | 0 | 8.173542529 | κ-bungarotoxin | **1.63%** |  | CAB46659 | *Bungarus multicinctus* |
| 116 | TRINITY_DN397_c1_g1_i1 | 60867 | 227 | 8.173542529 | VEGF |  | **1.45%** | JAA74898 | *Pseudonaja modesta* |
| 117 | TRINITY_DN397_c1_g1_i7 | 13513 | 0 | 8.173542529 | VEGF |  |  | XP_026551346 | *Pseudonaja textilis* |
| 118 | TRINITY_DN397_c1_g1_i4 | 11872 | 142 | 8.173542529 | VEGF |  |  | XP_026551346 | *Pseudonaja textilis* |
| 119 | TRINITY_DN397_c1_g1_i5 | 8991 | 50 | 8.173542529 | VEGF |  |  | JAA74898 | *Pseudonaja modesta* |
| 120 | TRINITY_DN397_c1_g1_i2 | 5797 | 0 | 5.543356523 | VEGF |  |  | XP_026551346 | *Pseudonaja textilis* |
| 121 | TRINITY_DN4427_c0_g1_i1 | 3442 | 139 | 5.543356523 | VEGF |  |  | LAB17921 | *Micrurus spixii* |
| 122 | TRINITY_DN4427_c0_g1_i4 | 763 | 0 | 8.173542529 | VEGF |  |  | JAB52939 | *Micrurus fulvius* |
| 123 | TRINITY_DN397_c1_g1_i3 | 617 | 8 | 5.543356523 | VEGF |  |  | XP_026551346 | *Pseudonaja textilis* |
| 124 | TRINITY_DN1402_c0_g1_i2 | 267 | 0 | 2.050467242 | VEGF |  |  | XP_015682072 | *Protobothrops mucrosquamatus* |
| 125 | TRINITY_DN47628_c0_g1_i1 | 201 | 73 | 5.543356523 | VEGF |  |  | LAB47472 | *Micrurus surinamensis* |
| 126 | TRINITY_DN5400_c0_g1_i1 | 153 | 71 | 5.543356523 | VEGF |  |  | LAA87755 | *Micrurus lemniscatus lemniscatus* |
| 127 | TRINITY_DN4427_c0_g1_i3 | 111 | 0 | 21.27715236 | VEGF |  |  | LAB17921 | *Micrurus spixii* |
| 128 | TRINITY_DN134_c0_g1_i14 | 302713 | 0 | 21.27715236 | β-bungarotoxin |  | **9.55%** | CAA37482 | *Bungarus multicinctus* |
| 129 | TRINITY_DN134_c0_g1_i9 | 211831 | 0 | 21.27715236 | β-bungarotoxin |  |  | ABU63164 | *Bungarus fasciatus* |
| 130 | TRINITY_DN134_c0_g1_i8 | 107475 | 0 | 21.27715236 | β-bungarotoxin |  |  | AAL87003 | *Bungarus caeruleus* |
| 131 | TRINITY_DN134_c0_g1_i10 | 57814 | 0 | 16.05485143 | β-bungarotoxin |  |  | AAL87003 | *Bungarus caeruleus* |
| 132 | TRINITY_DN660_c0_g1_i3 | 18152 | 0 | 18.8570687 | β-bungarotoxin |  |  | ABG90493 | *Bungarus multicinctus* |
| 133 | TRINITY_DN485_c0_g1_i10 | 4243 | 0 | 18.8570687 | β-bungarotoxin |  |  | AAL30067 | *Bungarus candidus* |
| 134 | TRINITY_DN485_c0_g1_i9 | 1069 | 0 | 16.05485143 | β-bungarotoxin |  |  | AAL30067 | *Bungarus candidus* |
| 135 | TRINITY_DN660_c0_g1_i4 | 703 | 0 | 16.6518196 | β-bungarotoxin |  |  | ABG90493 | *Bungarus multicinctus* |
| 136 | TRINITY_DN5510_c0_g2_i2 | 358 | 0 | #N/A | β-bungarotoxin |  |  | BAD06267 | *Bungarus candidus* |

Each toxin family and the corresponding relative abundance is uniquely colour coded.

**Table S13.** Details of antivenom samples investigated in this study.

| **Manufacturer** | **Batch** | **Manufacture**  **(M) and**  **Expiry**  **(E) dates** | **Protein**  **Content**  **(mg/mL)** | **Marketed Neutralizing Efficacy**  **(mg/mL)** |
| --- | --- | --- | --- | --- |
| **Haffkine BioPharmaceutical** **Corporation Ltd.** | AS180611 | **M:** 06/2018  **E:** 11/2022 | 24.7 ± 0.5 | *N. naja*: 0.60  *B. caeruleus*: 0.45  *D. russelii*: 0.60  *E. carinatus*: 0.45 |
| **Premium Serums**  **& Vaccines Pvt.**  **Ltd.** | ASVS(I)  Lyo013 | **M:** 11/2018  **E:** 11/2022 | 26.2 ± 1.2 |  |

Details of the commercial Indian polyvalent antivenoms tested in this study are provided here. Batch numbers, manufacturing and expiry dates, protein concentrations, and marketed neutralising potencies are shown.

**Table S14.** Toxicity profiles of *Bungarus* species from Western India.

| **Sample ID** | **Venom Dose** (µg) | | | | | **Number of Survivors** | | | | | **LD_50_** (µg/mouse) | **LD_50_**  (mg/Kg) |
| --- | --- | --- | --- | --- | --- | --- | --- | --- | --- | --- | --- | --- |
| *B. sindanus* | 0.31 | 0.39 | 0.49 | 0.61 | 0.76 | 5 | 3 | 2 | 0 | 0 | 0.44  0.37–0.52 | 0.022  0.018–0.026 |
| *B. caeruleus* | 2.88 | 3.6 | 4.5 | 5.62 | 7.03 | 5 | 5 | 3 | 2 | 0 | 5.03  4.25–5.95 | 0.251  0.213–0.297 |
| *B. romulusi* | 1 | 1.28 | 1.6 | 2 | 2.5 | 2 | 1 | 0 | 0 | 0 | 0.90  0.734–1.10 | 0.045  0.037–0.055 |

This table contains the median lethal doses and survivorship data for venoms of *Bungarus* species.

**Table S15.** Neutralisation potencies of commercial Indian polyvalent antivenoms against *Bungarus* venoms.

| **Sample ID** | **Antivenom Used:** Indian Polyvalent Antivenom Manufactured by  Premium Serums and Vaccines Pvt. Ltd. (Batch No. ASVS-I Lyo.013) | | | | | | |
| --- | --- | --- | --- | --- | --- | --- | --- |
|  | **Amount of Antivenom Injected in the Venom-Antivenom Mixture** (µL) | | | | **ED_50_**  (µL) | **ED_50_** (µL antivenom/mg venom) | **Potency of Antivenom** (mg/mL) |
| *B. sindanus* | 14.63 | 9.76 | 6.51 | 4.34 | 11.62  8.60- 15.68 | 5,281.82  3909.09- 7127.27 | 0.152  0.11- 0.20 |
| *B. caeruleus* | 32.93 | 21.96 | 14.63 | 9.75 | 26.90  23.21- 31.16 | 1069.58  922.86- 1238.97 | 0.74  0.64- 0.86 |
| *B. romulusi* | 32.93 | 21.96 | 14.63 | 9.75 | 26.90  23.21- 31.16 | 5977.78  5157.78- 6924.44 | 0.13  0.11- 0.15 |

The neutralisation potencies of commercial Indian polyvalent antivenom against the venoms of *Bungarus* species are shown in the table above.
