# Supplementary material for: A Wolf in Another Wolf’s Clothing: Post-Genomic Regulation Dictates Venom Profiles of Medically-Important Cryptic Kraits in India
Source: Toxins (Basel). 2021 Jan 19;13(1):69. doi: 10.3390/toxins13010069 (PMC7832344; doi:10.3390/toxins13010069)
Supplement: Supplementary file 1 [file toxins-13-00069-s001.zip › File S3.zip!/B. caeruleus/B. caeruleus.html]

proteins


Summary

  

# 1. Notes

# 2. Result Statistics

**Figure 1.**
False discovery rate (FDR) curve. X axis is the number of peptide-spectrum matches (PSM) being kept. Y axis is the corresponding FDR.

  


  

**Figure 2.**
PSM score distribution.  **(a)**
Distribution of PEAKS peptide score;  **(b)**
Scatterplot of PEAKS peptide score versus precursor mass error.

**(a)**

**(b)**

**Table 1.**
Statistics of data.


|  |  |  |  |  |  |  |  |  |  |  |  |
| --- | --- | --- | --- | --- | --- | --- | --- | --- | --- | --- | --- |
|  | #Scans | | #Features | Identified | | | #Peptides | #Sequences | #Proteins^\*^ | | |
|  | MS1 | MS2 |  | #PSMs | #Scans | #Features |  |  | Groups | All | Top |
| Total | 15027 | 71603 | 60693 | 640 | 635 | 7481 | 324 | 323 | 106 | 1055 | 398 |
| BuCaMH04\_B15 | 15027 | 71603 | 60693 | 640 | 635 | 7481 | 324 | 323 | 106 | 1055 | 398 |

^\* proteins with significant peptides are used in counts.^

**Figure 3.**
Sample overlap for Proteins and Peptides (up to 8 samples).  **(a)**
All Proteins;  **(b)**
Top Proteins;  **(c)**
Peptides;

**(a)**

**Not applicable to only one sample**

**(b)**

**Not applicable to only one sample**

**(c)**

**Not applicable to only one sample**

**Figure 4.**
Distribution of peptide feature detection.  **(a)**
Feature m/z distribution;  **(b)**
Feature RT distribution.

**(a)**

**(b)**

**Figure 5.**
Distribution of identified peptide features.  **(a)**
Feature abundance distribution;  **(b)**
 *De novo*
sequencing validation.

**(a)**

**(b)**

**Table 2.**
Result filtration parameters.

|  |  |
| --- | --- |
| Peptide -10lgP | ≥50.5 |
| PTM Ascore | ≥0 |
| Protein -10lgP | ≥20 |
| Proteins unique peptides | ≥0 |
| De novo score(%) | ≥50% |


**Table 3.**
Statistics of filtered result.

|  |  |
| --- | --- |
| FDR (Peptide-Spectrum Matches) | 0.0% |
| FDR (Peptide Sequences) | 0.0% |
| FDR (Protein Group) | 8.3% |
| De Novo Only Spectra | 13577 |

**Table 4.**
PTM profile.

| Name | ∆Mass | Position | #PSM | -10lgP | Abundance | AScore || Carbamidomethyl | 57.02 | C | 178 | 123.10 | 2.41E7 | 1000.00 |

# 3. Experiment Control

**Figure 6.**
Precursor mass error of peptide-spectrum matches (PSM) in filtered result.  **(a)**
Distribution of precursor mass error in ppm;  **(b)**
Scatterplot of precursor m/z versus precursor mass error in ppm.

**(a)**

**(b)**

**Table 5.**
Number of identified peptides in each sample by the number of missed cleavages.

|  |  |  |  |  |  |
| --- | --- | --- | --- | --- | --- |
| Missed Cleavages | 0 | 1 | 2 | 3 | 4+ |
| BuCaMH04\_B15 | 294 | 28 | 2 | 0 | 0 |

# 4. Other Information

**Table 6.**
Search parameters.

Search Engine Name: PEAKS  
Parent Mass Error Tolerance: 10.0 ppm  
Fragment Mass Error Tolerance: 0.6 Da  
Precursor Mass Search Type: monoisotopic  
Enzyme: Trypsin  
Max Missed Cleavages: 2  
Digest Mode: Semispecific  
Fixed Modifications:   
  Carbamidomethylation: 57.02  
Variable Modifications:   
  Oxidation (M): 15.99  
Max Variable PTM Per Peptide: 3  
Database: NCBI\_Serpentes\_BuCaMH04  
Taxon: All  
Contaminant Database: cRAP\_contaminants  
Searched Entry: 410235  
FDR Estimation: Enabled  
Merge Options: no merge  
Precursor Options: corrected  
Charge Options: no correction  
Filter Charge: 2 - 8  
Process: true  
Associate chimera: yes

**Table 7.**
Instrument parameters.

Fractions: BuCaMH04.raw  
Ion Source: ESI(nano-spray)  
Fragmentation Mode: CID, CAD(y and b ions)  
MS Scan Mode: FT-ICR/Orbitrap  
MS/MS Scan Mode: FT-ICR/Orbitrap

  

Protein List

  

Protein Accession Contains:

Protein Description Contains:

Protein Sample Area >=

Protein PTM Contains:

| Protein Group | Protein ID | Accession | -10lgP | Coverage (%) | Coverage (%) BuCaMH04\_B15 | Area BuCaMH04\_B15 | #Peptides | #Unique | #Spec BuCaMH04\_B15 | PTM | Avg. Mass | Description |
| --- | --- | --- | --- | --- | --- | --- | --- | --- | --- | --- | --- | --- |
| 1 | 9 | T\_174 | 432.47 | 55 | 55 | 3.1981E9 | 40 | 18 | 103 | Y | 68173 | T\_174 |
| 3 | 1 | 6I2X | 387.98 | 17 | 17 | 2.817E8 | 23 | 15 | 42 | Y | 184517 | Chain B, Cobra venom factor |
| 3 | 2 | 3PRX | 387.98 | 17 | 17 | 2.817E8 | 23 | 15 | 42 | Y | 184517 | Chain D, Cobra Venom Factor |
| 3 | 3 | I51018 | 387.98 | 17 | 17 | 2.817E8 | 23 | 15 | 42 | Y | 184517 | cobra venom factor precursor - monocled cobra |
| 3 | 4 | 3PVM | 387.98 | 17 | 17 | 2.817E8 | 23 | 15 | 42 | Y | 184517 | Chain D, Cobra Venom Factor |
| 3 | 5 | AAA68989.1 | 387.98 | 17 | 17 | 2.817E8 | 23 | 15 | 42 | Y | 184517 | cobra venom factor precursor [Naja kaouthia] |
| 3 | 6 | Q91132.1 | 387.98 | 17 | 17 | 2.817E8 | 23 | 15 | 42 | Y | 184517 | RecName: Full=Cobra venom factor; Short=CVF; Short=CVFk; AltName: Full=Complement C3 homolog; Contains: RecName: Full=Cobra venom factor alpha chain; Contains: RecName: Full=Cobra venom factor gamma chain; Contains: RecName: Full=C... |
| 3 | 7 | CAI46845.1 | 387.98 | 17 | 17 | 2.817E8 | 23 | 15 | 42 | Y | 184517 | unnamed protein product [Naja naja] |
| 2 | 15 | 5Z2G | 381.05 | 34 | 34 | 6.8152E8 | 22 | 18 | 69 | Y | 57963 | Chain B, L-amino acid oxidase |
| 2 | 16 | AVX27607.1 | 381.05 | 34 | 34 | 6.8152E8 | 22 | 18 | 69 | Y | 57963 | L-amino acid oxidase, partial [Naja atra] |
| 2 | 17 | A8QL58.2 | 381.05 | 34 | 34 | 6.8152E8 | 22 | 18 | 69 | Y | 57963 | RecName: Full=L-amino-acid oxidase; Short=LAO; Short=NA-LAAO; Flags: Precursor |
| 6 | 27 | XP\_026581281.1 | 336.19 | 30 | 30 |  | 17 | 0 | 34 | Y | 67027 | acetylcholinesterase, partial [Pseudonaja textilis] |
| 10 | 30 | ABN72539.1 | 325.23 | 28 | 28 | 1.4793E8 | 17 | 5 | 35 | Y | 58811 | L-amino acid oxidase [Bungarus multicinctus] |
| 10 | 31 | A8QL51.1 | 325.23 | 28 | 28 | 1.4793E8 | 17 | 5 | 35 | Y | 58811 | RecName: Full=L-amino-acid oxidase; Short=Bm-LAAO; Short=LAO; Flags: Precursor |
| 9 | 33 | JAA74986.1 | 321.00 | 26 | 26 |  | 13 | 0 | 30 | Y | 63242 | ACN-Sut-1, partial [Suta fasciata] |
| 11 | 28 | T\_101 | 308.62 | 25 | 25 | 4.08E7 | 15 | 2 | 38 | Y | 58921 | T\_101 |
| 11 | 29 | T\_95 | 308.62 | 22 | 22 | 4.08E7 | 15 | 2 | 38 | Y | 66293 | T\_95 |
| 7 | 62 | AAY89680.1 | 296.07 | 18 | 18 |  | 11 | 0 | 42 | N | 59070 | L-amino acid oxidase precursor [Oxyuranus scutellatus] |
| 7 | 63 | Q4JHE3.1 | 296.07 | 18 | 18 |  | 11 | 0 | 42 | N | 59070 | RecName: Full=L-amino-acid oxidase; Short=LAAO; Short=LAO; Flags: Precursor |
| 4 | 44 | ABN72537.1 | 294.11 | 17 | 17 | 1.4363E8 | 13 | 7 | 31 | Y | 68988 | metalloproteinase [Bungarus multicinctus] |
| 4 | 45 | A8QL49.1 | 294.11 | 17 | 17 | 1.4363E8 | 13 | 7 | 31 | Y | 68988 | RecName: Full=Zinc metalloproteinase-disintegrin-like BmMP; AltName: Full=Snake venom metalloproteinase; Short=SVMP; Flags: Precursor |
| 15 | 19 | JAS05143.1 | 290.68 | 26 | 26 | 1.3453E7 | 14 | 4 | 21 | Y | 63012 | ecto-5'-nucleotidase [Micrurus tener] |
| 15 | 20 | JAI09048.1 | 290.68 | 26 | 26 | 1.3453E7 | 14 | 4 | 21 | Y | 63025 | Ecto-5'-nucleotidase 1b [Micrurus fulvius] |
| 15 | 21 | JAS05036.1 | 290.68 | 26 | 26 | 1.3453E7 | 14 | 4 | 21 | Y | 62983 | Ecto-5'-nucleotidase 1c [Micrurus fulvius] |
| 15 | 22 | JAI09049.1 | 290.68 | 26 | 26 | 1.3453E7 | 14 | 4 | 21 | Y | 63025 | Ecto-5'-nucleotidase 1a [Micrurus fulvius] |
| 15 | 23 | JAS05038.1 | 290.68 | 26 | 26 | 1.3453E7 | 14 | 4 | 21 | Y | 63025 | Ecto-5'-nucleotidase 1a [Micrurus fulvius] |
| 15 | 24 | JAS05037.1 | 290.68 | 26 | 26 | 1.3453E7 | 14 | 4 | 21 | Y | 63025 | Ecto-5'-nucleotidase 1b [Micrurus fulvius] |
| 15 | 25 | JAI09047.1 | 290.68 | 26 | 26 | 1.3453E7 | 14 | 4 | 21 | Y | 62983 | Ecto-5'-nucleotidase 1c [Micrurus fulvius] |
| 15 | 26 | JAB52815.1 | 290.68 | 26 | 26 | 1.3453E7 | 14 | 4 | 21 | Y | 62983 | Ecto-5'-nucleotidase 1 [Micrurus fulvius] |
| 21 | 91 | T\_109 | 273.37 | 17 | 17 | 4.0668E8 | 8 | 5 | 12 | Y | 68846 | T\_109 |
| 21 | 92 | T\_112 | 273.37 | 16 | 16 | 4.0668E8 | 8 | 5 | 12 | Y | 72609 | T\_112 |
| 5 | 61 | P82942.1 | 262.78 | 24 | 24 | 1.0759E8 | 11 | 7 | 34 | Y | 44493 | RecName: Full=Hemorrhagic metalloproteinase-disintegrin-like kaouthiagin; AltName: Full=Snake venom metalloproteinase; Short=SVMP |
| 17 | 51 | T\_113 | 254.45 | 22 | 22 | 3.9009E7 | 8 | 2 | 18 | Y | 50598 | T\_113 |
| 16 | 77 | ASU45022.1 | 250.37 | 10 | 10 |  | 8 | 0 | 16 | Y | 62791 | acetylcholinesterase, partial [Daboia russelii] |
| 18 | 60 | XP\_015668657.1 | 247.49 | 9 | 9 |  | 7 | 0 | 15 | Y | 65459 | acetylcholinesterase [Protobothrops mucrosquamatus] |
| 8 | 137 | ADD14036.1 | 244.40 | 13 | 13 | 1.3949E7 | 7 | 2 | 28 | Y | 44750 | metalloproteinase atrase B, partial [Naja atra] |
| 8 | 138 | D6PXE8.1 | 244.40 | 9 | 9 | 1.3949E7 | 7 | 2 | 28 | Y | 66246 | RecName: Full=Zinc metalloproteinase-disintegrin-like atrase-B; AltName: Full=Snake venom metalloproteinase; Short=SVMP; Flags: Precursor |
| 8 | 139 | ADG02948.1 | 244.40 | 9 | 9 | 1.3949E7 | 7 | 2 | 28 | Y | 66246 | metalloproteinase atrase B [Naja atra] |
| 8 | 140 | 3K7N | 244.40 | 13 | 13 | 1.3949E7 | 7 | 2 | 28 | Y | 44191 | Chain A, Structures Of Two Elapid Snake Venom Metalloproteases With Distinct Activities Highlight The Disulfide Patterns In The D Domain Of Adamalysin Family Proteins |
| 8 | 141 | ACN50005.1 | 244.40 | 9 | 9 | 1.3949E7 | 7 | 2 | 28 | Y | 66292 | K-like metalloprotease precursor, partial [Naja atra] |
| 8 | 142 | D3TTC1.1 | 244.40 | 9 | 9 | 1.3949E7 | 7 | 2 | 28 | Y | 66292 | RecName: Full=Zinc metalloproteinase-disintegrin-like kaouthiagin-like; Short=K-like; AltName: Full=Snake venom metalloproteinase; Short=SVMP; Flags: Precursor |
| 29 | 35 | 5GZ4 | 241.28 | 11 | 11 | 2.1314E7 | 8 | 8 | 10 | N | 94616 | Chain A, Snake Venom Phosphodiesterase (pde) |
| 29 | 36 | 5GZ5 | 241.28 | 11 | 11 | 2.1314E7 | 8 | 8 | 10 | N | 94616 | Chain A, Snake Venom Phosphodiesterase (pde) |
| 29 | 37 | A0A2D0TC04.1 | 241.28 | 11 | 11 | 2.1314E7 | 8 | 8 | 10 | N | 94616 | RecName: Full=Venom phosphodiesterase; Short=PDE |
| 12 | 131 | JAA74761.1 | 241.21 | 13 | 13 | 5.8499E6 | 9 | 1 | 36 | N | 59205 | LAO-Ech-1, partial [Echiopsis curta] |
| 13 | 177 | Q8QFW3.1 | 237.26 | 35 | 35 | 2.0404E9 | 6 | 5 | 20 | Y | 16120 | RecName: Full=Basic phospholipase A2 beta-bungarotoxin A2 chain; Short=Beta-BuTX A2 chain; Short=svPLA2; AltName: Full=Phosphatidylcholine 2-acylhydrolase; Flags: Precursor |
| 13 | 178 | AAL87004.1 | 237.26 | 35 | 35 | 2.0404E9 | 6 | 5 | 20 | Y | 16120 | beta-bungarotoxin A2 chain precursor [Bungarus caeruleus] |
| 34 | 75 | B6EWW8.1 | 236.58 | 16 | 16 | 4.1447E5 | 9 | 1 | 13 | Y | 64434 | RecName: Full=Snake venom 5'-nucleotidase; Short=5'-NT; AltName: Full=Ecto-5'-nucleotidase; Flags: Precursor |
| 34 | 76 | BAG82602.1 | 236.58 | 16 | 16 | 4.1447E5 | 9 | 1 | 13 | Y | 64434 | ecto-5'-nucleotidase [Gloydius brevicaudus] |
| 20 | 170 | XP\_032090372.1 | 233.42 | 7 | 7 |  | 7 | 0 | 14 | Y | 64554 | acetylcholinesterase isoform X2 [Thamnophis elegans] |
| 20 | 171 | XP\_032090371.1 | 233.42 | 7 | 7 |  | 7 | 0 | 14 | Y | 68064 | acetylcholinesterase isoform X1 [Thamnophis elegans] |
| 20 | 179 | XP\_013930899.1 | 233.42 | 7 | 7 |  | 7 | 0 | 14 | Y | 64629 | PREDICTED: acetylcholinesterase [Thamnophis sirtalis] |
| 33 | 249 | LAA54621.1 | 228.70 | 10 | 10 | 5.0012E6 | 7 | 1 | 14 | N | 58752 | hypothetical protein [Micrurus corallinus] |
| 19 | 83 | Q9PVK7.1 | 228.65 | 11 | 11 | 5.8631E7 | 7 | 3 | 15 | Y | 67662 | RecName: Full=Zinc metalloproteinase-disintegrin-like cobrin; AltName: Full=Snake venom metalloproteinase; Short=SVMP; Flags: Precursor |
| 19 | 84 | AAF00693.1 | 228.65 | 11 | 11 | 5.8631E7 | 7 | 3 | 15 | Y | 67662 | cobrin precursor [Naja naja] |
| 31 | 50 | ETE70041.1 | 226.11 | 14 | 14 | 2.5553E7 | 9 | 9 | 11 | N | 73994 | Sulfhydryl oxidase 1 [Ophiophagus hannah] |
| 46 | 114 | XP\_034291085.1 | 222.57 | 13 | 13 | 9.0579E7 | 7 | 3 | 9 | Y | 63129 | tissue-type plasminogen activator [Pantherophis guttatus] |
| 46 | 115 | XP\_034291087.1 | 222.57 | 13 | 13 | 9.0579E7 | 7 | 3 | 9 | Y | 63129 | tissue-type plasminogen activator [Pantherophis guttatus] |
| 46 | 116 | XP\_034291086.1 | 222.57 | 13 | 13 | 9.0579E7 | 7 | 3 | 9 | Y | 63129 | tissue-type plasminogen activator [Pantherophis guttatus] |
| 37 | 143 | AXL95288.1 | 222.07 | 13 | 13 | 9.7537E6 | 6 | 1 | 11 | N | 58594 | L-amino acid oxidase 2, partial [Spilotes sulphureus] |
| 37 | 144 | AXL95287.1 | 222.07 | 13 | 13 | 9.7537E6 | 6 | 1 | 11 | N | 58594 | L-amino acid oxidase 1, partial [Spilotes sulphureus] |
| 25 | 282 | S68801 | 221.06 | 43 | 43 | 9.6369E7 | 5 | 1 | 13 | N | 9793 | acetylcholinesterase (EC 3.1.1.7) - banded krait (fragments) |
| 30 | 59 | ABN72543.1 | 220.84 | 4 | 4 |  | 5 | 0 | 9 | N | 184353 | complement-depleting factor [Ophiophagus hannah] |
| 35 | 211 | JAC94932.1 | 210.70 | 5 | 5 |  | 7 | 0 | 10 | N | 69041 | Acetylcholinesterase transcript 2 [Python regius] |
| 35 | 214 | XP\_025032622.1 | 210.70 | 5 | 5 |  | 7 | 0 | 10 | N | 67497 | acetylcholinesterase isoform X2 [Python bivittatus] |
| 35 | 215 | XP\_025032620.1 | 210.70 | 5 | 5 |  | 7 | 0 | 10 | N | 67497 | acetylcholinesterase isoform X2 [Python bivittatus] |
| 35 | 216 | XP\_025032621.1 | 210.70 | 5 | 5 |  | 7 | 0 | 10 | N | 67497 | acetylcholinesterase isoform X2 [Python bivittatus] |
| 35 | 217 | XP\_025032619.1 | 210.70 | 5 | 5 |  | 7 | 0 | 10 | N | 72046 | acetylcholinesterase isoform X1 [Python bivittatus] |
| 27 | 54 | C3NJ | 209.13 | 3 | 3 |  | 5 | 0 | 8 | Y | 184926 | complement C3 precursor - monocled cobra |
| 27 | 55 | Q01833.1 | 209.13 | 3 | 3 |  | 5 | 0 | 8 | Y | 184926 | RecName: Full=Complement C3; Contains: RecName: Full=Complement C3 beta chain; Contains: RecName: Full=Complement C3 alpha chain; Contains: RecName: Full=C3a anaphylatoxin; Contains: RecName: Full=Complement C3b alpha' chain; Contai... |
| 27 | 56 | AAA49385.1 | 209.13 | 3 | 3 |  | 5 | 0 | 8 | Y | 184926 | complement component C3 [Naja naja] |
| 41 | 87 | AAZ81953.1 | 209.12 | 3 | 3 |  | 5 | 0 | 8 | Y | 184725 | venom factor 1 [Austrelaps superbus] |
| 41 | 88 | AAX86641.1 | 209.12 | 3 | 3 |  | 5 | 0 | 8 | Y | 184893 | venom factor [Austrelaps superbus] |
| 41 | 89 | Q0ZZJ6.1 | 209.12 | 3 | 3 |  | 5 | 0 | 8 | Y | 184725 | RecName: Full=A.superbus venom factor 1; Short=AVF-1; AltName: Full=CVF-like; AltName: Full=Complement C3 homolog; Contains: RecName: Full=AVF-1 alpha chain; Contains: RecName: Full=AVF-1 gamma chain; Contains: RecName: Full=AVF-1... |
| 41 | 90 | ABA28745.1 | 209.12 | 3 | 3 |  | 5 | 0 | 8 | Y | 184695 | complement protein C3-1 [Austrelaps superbus] |
| 39 | 309 | Q8QFW4.1 | 204.23 | 29 | 29 | 1.8951E8 | 4 | 3 | 9 | Y | 16358 | RecName: Full=Basic phospholipase A2 beta-bungarotoxin A1 chain; Short=Beta-BuTX A1 chain; Short=svPLA2; AltName: Full=Phosphatidylcholine 2-acylhydrolase; Flags: Precursor |
| 39 | 310 | AAL87003.1 | 204.23 | 29 | 29 | 1.8951E8 | 4 | 3 | 9 | Y | 16358 | beta-bungarotoxin A chain precursor [Bungarus caeruleus] |
| 44 | 544 | P0DI91.1 | 203.51 | 22 | 22 | 3.0762E7 | 4 | 2 | 8 | Y | 11216 | RecName: Full=L-amino-acid oxidase; Short=LAAO; Short=LAO |
| 57 | 125 | XP\_026544671.1 | 202.44 | 12 | 12 | 4.9155E7 | 5 | 1 | 7 | Y | 60656 | tissue-type plasminogen activator, partial [Notechis scutatus] |
| 57 | 126 | XP\_026570965.1 | 202.44 | 11 | 11 | 4.9155E7 | 5 | 1 | 7 | Y | 64106 | LOW QUALITY PROTEIN: tissue-type plasminogen activator [Pseudonaja textilis] |
| 26 | 57 | T\_156 | 200.89 | 17 | 17 | 5.5591E5 | 6 | 1 | 12 | Y | 54306 | T\_156 |
| 26 | 58 | T\_151 | 200.89 | 17 | 17 | 5.5591E5 | 6 | 1 | 12 | Y | 54306 | T\_151 |
| 50 | 158 | AXL96620.1 | 200.39 | 3 | 3 |  | 5 | 0 | 8 | N | 185522 | cobra venom factor 1, partial [Ahaetulla prasina] |
| 50 | 168 | JAC88987.1 | 200.39 | 3 | 3 |  | 5 | 0 | 8 | N | 185574 | Complement C3 [Pantherophis guttatus] |
| 50 | 169 | JAC95048.1 | 200.39 | 3 | 3 |  | 5 | 0 | 8 | N | 185574 | Complement C3 [Pantherophis guttatus] |
| 50 | 175 | XP\_034290336.1 | 200.39 | 3 | 3 |  | 5 | 0 | 8 | N | 185621 | complement C3 isoform X1 [Pantherophis guttatus] |
| 23 | 52 | XP\_026524834.1 | 199.53 | 14 | 14 |  | 6 | 0 | 12 | N | 54680 | hyaluronidase-2-like isoform X1 [Notechis scutatus] |
| 23 | 53 | XP\_026524835.1 | 199.53 | 14 | 14 |  | 6 | 0 | 12 | N | 54568 | hyaluronidase-2-like isoform X2 [Notechis scutatus] |
| 58 | 134 | LAB44384.1 | 196.48 | 11 | 11 | 2.4172E7 | 5 | 5 | 7 | N | 59763 | hypothetical protein [Micrurus spixii] |
| 58 | 135 | LAA86986.1 | 196.48 | 23 | 23 | 2.4172E7 | 5 | 5 | 7 | N | 28508 | hypothetical protein, partial [Micrurus lemniscatus lemniscatus] |
| 58 | 136 | LAB57337.1 | 196.48 | 21 | 21 | 2.4172E7 | 5 | 5 | 7 | N | 30451 | hypothetical protein, partial [Micrurus surinamensis] |
| 58 | 155 | XP\_026566518.1 | 196.48 | 11 | 11 | 2.4172E7 | 5 | 5 | 7 | N | 59709 | cochlin [Pseudonaja textilis] |
| 53 | 181 | XP\_034290338.1 | 194.98 | 2 | 2 |  | 4 | 0 | 7 | N | 186036 | complement C3 isoform X2 [Pantherophis guttatus] |
| 32 | 756 | 1U4J | 194.87 | 17 | 17 | 8.4167E8 | 5 | 1 | 10 | Y | 12970 | Chain B, Phospholipase A2 Isoform 2 |
| 32 | 757 | 1G0Z | 194.87 | 17 | 17 | 8.4167E8 | 5 | 1 | 10 | Y | 12970 | Chain B, Phospholipase A2 |
| 32 | 758 | Q6SLM1.1 | 194.87 | 15 | 15 | 8.4167E8 | 5 | 1 | 10 | Y | 14827 | RecName: Full=Basic phospholipase A2 2; Short=svPLA2; AltName: Full=Phosphatidylcholine 2-acylhydrolase; Flags: Precursor |
| 32 | 759 | AAR19228.1 | 194.87 | 15 | 15 | 8.4167E8 | 5 | 1 | 10 | Y | 14827 | phospholipase A2 isoform 2, partial [Bungarus caeruleus] |
| 49 | 902 | ABU63164.1 | 192.65 | 15 | 15 | 2.1599E7 | 5 | 1 | 8 | Y | 15950 | phospholipase A2 precursor BF-40 [Bungarus fasciatus] |
| 49 | 903 | ABU63166.1 | 192.65 | 15 | 15 | 2.1599E7 | 5 | 1 | 8 | Y | 16004 | phospholipase A2 precursor BF-42 [Bungarus fasciatus] |
| 45 | 764 | JAB52792.1 | 190.96 | 14 | 14 | 4.8615E7 | 5 | 1 | 9 | Y | 16159 | phospholipase A2 27 [Micrurus fulvius] |
| 45 | 919 | AAZ29513.1 | 190.96 | 15 | 15 | 4.8615E7 | 5 | 1 | 9 | Y | 15328 | phospholipase A2, partial [Micrurus fulvius] |
| 45 | 920 | JAB52790.1 | 190.96 | 14 | 14 | 4.8615E7 | 5 | 1 | 9 | Y | 15975 | phospholipase A2 29 [Micrurus fulvius] |
| 45 | 921 | JAB52794.1 | 190.96 | 14 | 14 | 4.8615E7 | 5 | 1 | 9 | Y | 16031 | phospholipase A2 25 [Micrurus fulvius] |
| 45 | 922 | JAB52809.1 | 190.96 | 14 | 14 | 4.8615E7 | 5 | 1 | 9 | Y | 16079 | phospholipase A2 14 [Micrurus fulvius] |
| 45 | 923 | JAB52771.1 | 190.96 | 14 | 14 | 4.8615E7 | 5 | 1 | 9 | Y | 16208 | phospholipase A2 4d [Micrurus fulvius] |
| 45 | 924 | JAS04998.1 | 190.96 | 14 | 14 | 4.8615E7 | 5 | 1 | 9 | Y | 16157 | Phospholipase A2 4b [Micrurus fulvius] |
| 45 | 925 | JAB52773.1 | 190.96 | 14 | 14 | 4.8615E7 | 5 | 1 | 9 | Y | 16217 | phospholipase A2 4b [Micrurus fulvius] |
| 45 | 926 | JAB52772.1 | 190.96 | 14 | 14 | 4.8615E7 | 5 | 1 | 9 | Y | 16191 | phospholipase A2 4c [Micrurus fulvius] |
| 45 | 927 | JAS04995.1 | 190.96 | 14 | 14 | 4.8615E7 | 5 | 1 | 9 | Y | 16176 | Phospholipase A2 4e [Micrurus fulvius] |
| 45 | 928 | JAB52770.1 | 190.96 | 14 | 14 | 4.8615E7 | 5 | 1 | 9 | Y | 16157 | phospholipase A2 4e [Micrurus fulvius] |
| 45 | 929 | JAB52795.1 | 190.96 | 14 | 14 | 4.8615E7 | 5 | 1 | 9 | Y | 16236 | phospholipase A2 24 [Micrurus fulvius] |
| 45 | 930 | JAB52774.1 | 190.96 | 14 | 14 | 4.8615E7 | 5 | 1 | 9 | Y | 16176 | phospholipase A2 4a [Micrurus fulvius] |
| 45 | 931 | JAS04999.1 | 190.96 | 14 | 14 | 4.8615E7 | 5 | 1 | 9 | Y | 16176 | Phospholipase A2 4a [Micrurus fulvius] |
| 45 | 932 | JAI09010.1 | 190.96 | 14 | 14 | 4.8615E7 | 5 | 1 | 9 | Y | 16176 | Phospholipase A2 4a [Micrurus fulvius] |
| 45 | 933 | JAI09006.1 | 190.96 | 14 | 14 | 4.8615E7 | 5 | 1 | 9 | Y | 16176 | Phospholipase A2 4e [Micrurus fulvius] |
| 45 | 934 | JAI09004.1 | 190.96 | 14 | 14 | 4.8615E7 | 5 | 1 | 9 | Y | 16300 | Phospholipase A2 5b [Micrurus fulvius] |
| 45 | 935 | JAI09005.1 | 190.96 | 14 | 14 | 4.8615E7 | 5 | 1 | 9 | Y | 16236 | Phospholipase A2 5a [Micrurus fulvius] |
| 45 | 936 | JAI09009.1 | 190.96 | 14 | 14 | 4.8615E7 | 5 | 1 | 9 | Y | 16157 | Phospholipase A2 4b [Micrurus fulvius] |
| 45 | 937 | JAS04993.1 | 190.96 | 14 | 14 | 4.8615E7 | 5 | 1 | 9 | Y | 16300 | Phospholipase A2 5b [Micrurus fulvius] |
| 45 | 938 | JAB52804.1 | 190.96 | 14 | 14 | 4.8615E7 | 5 | 1 | 9 | Y | 16290 | phospholipase A2 19 [Micrurus fulvius] |
| 45 | 939 | JAS04994.1 | 190.96 | 14 | 14 | 4.8615E7 | 5 | 1 | 9 | Y | 16236 | Phospholipase A2 5a [Micrurus fulvius] |
| 45 | 940 | JAB52764.1 | 190.96 | 14 | 14 | 4.8615E7 | 5 | 1 | 9 | Y | 16159 | phospholipase A2 6c [Micrurus fulvius] |
| 45 | 967 | LAA98189.1 | 190.96 | 20 | 20 | 4.8615E7 | 5 | 1 | 9 | Y | 11260 | hypothetical protein, partial [Micrurus lemniscatus lemniscatus] |
| 45 | 969 | JAS04997.1 | 190.96 | 14 | 14 | 4.8615E7 | 5 | 1 | 9 | Y | 16161 | Phospholipase A2 4c [Micrurus fulvius] |
| 45 | 970 | JAI09008.1 | 190.96 | 14 | 14 | 4.8615E7 | 5 | 1 | 9 | Y | 16161 | Phospholipase A2 4c [Micrurus fulvius] |
| 45 | 971 | ABU68503.1 | 190.96 | 13 | 13 | 4.8615E7 | 5 | 1 | 9 | Y | 16620 | PLA2(IB)-Tri1 [Trimorphodon biscutatus] |
| 14 | 191 | AXL96665.1 | 188.62 | 6 | 6 |  | 5 | 0 | 13 | Y | 68148 | metalloproteinase 37, partial [Ahaetulla prasina] |
| 14 | 192 | AXL96660.1 | 188.62 | 6 | 6 |  | 5 | 0 | 13 | Y | 68120 | metalloproteinase 31, partial [Ahaetulla prasina] |
| 14 | 193 | AXL96668.1 | 188.62 | 6 | 6 |  | 5 | 0 | 13 | Y | 68144 | metalloproteinase 17, partial [Ahaetulla prasina] |
| 14 | 194 | AXL96656.1 | 188.62 | 6 | 6 |  | 5 | 0 | 13 | Y | 68152 | metalloproteinase 22, partial [Ahaetulla prasina] |
| 14 | 195 | AXL96651.1 | 188.62 | 6 | 6 |  | 5 | 0 | 13 | Y | 68041 | metalloproteinase 35, partial [Ahaetulla prasina] |
| 48 | 762 | P86538.2 | 185.04 | 22 | 22 | 8.4022E7 | 3 | 3 | 8 | N | 6711 | RecName: Full=Cytotoxin 2a; Short=CTX2a; AltName: Full=Cytotoxin 2; Short=CTX2 |
| 48 | 763 | BAU24666.1 | 185.04 | 18 | 18 | 8.4022E7 | 3 | 3 | 8 | N | 7959 | cytotoxin 11, partial [Naja naja] |
| 51 | 261 | ABN72545.1 | 184.12 | 12 | 12 | 1.5206E7 | 4 | 3 | 9 | Y | 31010 | putative serine protease, partial [Bungarus multicinctus] |
| 51 | 262 | A8QL57.1 | 184.12 | 12 | 12 | 1.5206E7 | 4 | 3 | 9 | Y | 31010 | RecName: Full=Snake venom serine protease BmSP; Short=SVSP; Flags: Precursor |
| 51 | 284 | T\_82 | 184.12 | 11 | 11 | 1.5206E7 | 4 | 3 | 9 | Y | 31549 | T\_82 |
| 54 | 510 | XP\_034258146.1 | 174.62 | 13 | 13 | 1.7428E7 | 6 | 4 | 8 | Y | 28406 | glutathione peroxidase 3 [Pantherophis guttatus] |
| 47 | 270 | XP\_026555793.1 | 172.16 | 14 | 14 | 1.097E6 | 4 | 1 | 9 | N | 34167 | ADP-ribosyl cyclase/cyclic ADP-ribose hydrolase 1-like [Pseudonaja textilis] |
| 28 | 236 | 1PSH | 167.87 | 29 | 29 | 3.2859E8 | 4 | 3 | 8 | Y | 13346 | Chain C, Phospholipase A2 |
| 28 | 237 | 1A3F | 167.87 | 29 | 29 | 3.2859E8 | 4 | 3 | 8 | Y | 13346 | Chain C, Phospholipase A2 (Pla2) From Naja Naja Venom |
| 28 | 238 | 1A3D | 167.87 | 29 | 29 | 3.2859E8 | 4 | 3 | 8 | Y | 13346 | Chain A, Phospholipase A2 |
| 28 | 239 | P15445.1 | 167.87 | 29 | 29 | 3.2859E8 | 4 | 3 | 8 | Y | 13346 | RecName: Full=Acidic phospholipase A2 2; Short=PLA22; Short=svPLA2; AltName: Full=Phosphatidylcholine 2-acylhydrolase |
| 28 | 240 | CAA45372.1 | 167.87 | 29 | 29 | 3.2859E8 | 4 | 3 | 8 | Y | 13477 | phospholipase a2 [Naja naja] |
| 43 | 145 | AXL95273.1 | 164.25 | 11 | 11 | 1.7317E6 | 5 | 1 | 8 | Y | 64544 | 5' nucleotidase, partial [Spilotes sulphureus] |
| 43 | 151 | JAG67188.1 | 164.25 | 11 | 11 | 1.7317E6 | 5 | 1 | 8 | Y | 64759 | Snake venom 5'-nucleotidase [Boiga irregularis] |
| 38 | 494 | 2CTX | 158.07 | 41 | 41 | 5.8162E9 | 3 | 2 | 10 | Y | 7831 | Chain A, ALPHA-COBRATOXIN |
| 38 | 495 | 1LXH | 158.07 | 41 | 41 | 5.8162E9 | 3 | 2 | 10 | Y | 7831 | Chain A, LONG NEUROTOXIN 1 |
| 38 | 496 | 1LXG | 158.07 | 41 | 41 | 5.8162E9 | 3 | 2 | 10 | Y | 7831 | Chain A, Long neurotoxin 1 |
| 38 | 497 | P01391.1 | 158.07 | 41 | 41 | 5.8162E9 | 3 | 2 | 10 | Y | 7831 | RecName: Full=Alpha-cobratoxin; Short=Alpha-CbT; Short=alpha-CT; Short=alpha-Cbtx; AltName: Full=Alpha-elapitoxin-Nk2a; Short=Alpha-EPTX-Nk2a; AltName: Full=Long neurotoxin 1; AltName: Full=Siamensis 3 |
| 38 | 498 | 4AEA | 158.07 | 41 | 41 | 5.8162E9 | 3 | 2 | 10 | Y | 7831 | Chain B, Long Neurotoxin 1 |
| 38 | 499 | 1CTX | 158.07 | 41 | 41 | 5.8162E9 | 3 | 2 | 10 | Y | 7831 | Chain A, ALPHA-COBRATOXIN |
| 38 | 500 | 1YI5 | 158.07 | 41 | 41 | 5.8162E9 | 3 | 2 | 10 | Y | 7831 | Chain J, Long Neurotoxin 1 |
| 77 | 405 | XP\_013924291.1 | 153.60 | 5 | 5 |  | 4 | 0 | 5 | N | 53340 | PREDICTED: keratin, type II cytoskeletal cochleal-like [Thamnophis sirtalis] |
| 77 | 412 | XP\_032067160.1 | 153.60 | 5 | 5 |  | 4 | 0 | 5 | N | 53343 | keratin, type II cytoskeletal cochleal-like [Thamnophis elegans] |
| 77 | 502 | ETE64433.1 | 153.60 | 5 | 5 |  | 4 | 0 | 5 | N | 51876 | hypothetical protein L345\_09802, partial [Ophiophagus hannah] |
| 77 | 503 | XP\_026573164.1 | 153.60 | 5 | 5 |  | 4 | 0 | 5 | N | 53598 | keratin, type II cytoskeletal cochleal-like [Pseudonaja textilis] |
| 77 | 504 | JAG44289.1 | 153.60 | 5 | 5 |  | 4 | 0 | 5 | N | 53768 | Keratin, type II cytoskeletal cochleal-like [Crotalus horridus] |
| 77 | 505 | AFJ50410.1 | 153.60 | 5 | 5 |  | 4 | 0 | 5 | N | 53645 | Keratin, type II cytoskeletal cochleal-like [Crotalus adamanteus] |
| 78 | 501 | P82885.1 | 153.40 | 32 | 32 | 7.3297E7 | 3 | 3 | 4 | Y | 12038 | RecName: Full=Thaicobrin |
| 56 | 472 | T\_80 | 151.60 | 17 | 17 | 1.0015E8 | 2 | 2 | 5 | N | 26293 | T\_80 |
| 56 | 473 | T\_81 | 151.60 | 17 | 17 | 1.0015E8 | 2 | 2 | 5 | N | 26293 | T\_81 |
| 22 | 698 | AXL96635.1 | 148.44 | 2 | 2 | 1.8071E6 | 4 | 1 | 12 | N | 68842 | metalloproteinase 34, partial [Ahaetulla prasina] |
| 22 | 699 | AXL96667.1 | 148.44 | 2 | 2 | 1.8071E6 | 4 | 1 | 12 | N | 68806 | metalloproteinase 11, partial [Ahaetulla prasina] |
| 22 | 700 | AXL96646.1 | 148.44 | 2 | 2 | 1.8071E6 | 4 | 1 | 12 | N | 68782 | metalloproteinase 9, partial [Ahaetulla prasina] |
| 22 | 701 | AXL96640.1 | 148.44 | 2 | 2 | 1.8071E6 | 4 | 1 | 12 | N | 68845 | metalloproteinase 29, partial [Ahaetulla prasina] |
| 22 | 702 | AXL96666.1 | 148.44 | 2 | 2 | 1.8071E6 | 4 | 1 | 12 | N | 68871 | metalloproteinase 12, partial [Ahaetulla prasina] |
| 22 | 703 | AXL96626.1 | 148.44 | 2 | 2 | 1.8071E6 | 4 | 1 | 12 | N | 72956 | metalloproteinase 7, partial [Ahaetulla prasina] |
| 22 | 766 | AXL96631.1 | 148.44 | 2 | 2 | 1.8071E6 | 4 | 1 | 12 | N | 67772 | metalloproteinase 15, partial [Ahaetulla prasina] |
| 22 | 767 | AXL96650.1 | 148.44 | 2 | 2 | 1.8071E6 | 4 | 1 | 12 | N | 71879 | metalloproteinase 6, partial [Ahaetulla prasina] |
| 22 | 821 | AXL96645.1 | 148.44 | 2 | 2 | 1.8071E6 | 4 | 1 | 12 | N | 71446 | metalloproteinase 5, partial [Ahaetulla prasina] |
| 22 | 1058 | AXL96653.1 | 148.44 | 2 | 2 | 1.8071E6 | 4 | 1 | 12 | N | 68112 | metalloproteinase 24, partial [Ahaetulla prasina] |
| 22 | 1059 | AXL96648.1 | 148.44 | 2 | 2 | 1.8071E6 | 4 | 1 | 12 | N | 68242 | metalloproteinase 14, partial [Ahaetulla prasina] |
| 22 | 1060 | AXL96655.1 | 148.44 | 2 | 2 | 1.8071E6 | 4 | 1 | 12 | N | 68219 | metalloproteinase 2, partial [Ahaetulla prasina] |
| 22 | 1061 | AXL96638.1 | 148.44 | 2 | 2 | 1.8071E6 | 4 | 1 | 12 | N | 68153 | metalloproteinase 3, partial [Ahaetulla prasina] |
| 22 | 1062 | AXL96643.1 | 148.44 | 2 | 2 | 1.8071E6 | 4 | 1 | 12 | N | 68282 | metalloproteinase 25, partial [Ahaetulla prasina] |
| 22 | 1063 | AXL96644.1 | 148.44 | 2 | 2 | 1.8071E6 | 4 | 1 | 12 | N | 68220 | metalloproteinase 16, partial [Ahaetulla prasina] |
| 22 | 1064 | AXL96633.1 | 148.44 | 2 | 2 | 1.8071E6 | 4 | 1 | 12 | N | 68168 | metalloproteinase 20, partial [Ahaetulla prasina] |
| 52 | 506 | XP\_032064947.1 | 147.77 | 7 | 7 |  | 2 | 0 | 9 | N | 58410 | L-amino-acid oxidase [Thamnophis elegans] |
| 60 | 653 | A8QL56.1 | 144.06 | 6 | 6 | 1.6412E7 | 2 | 1 | 7 | Y | 28656 | RecName: Full=Alpha- and beta-fibrinogenase OhS1; AltName: Full=Snake venom serine protease; Short=SVSP; Flags: Precursor |
| 60 | 654 | ABN72544.1 | 144.06 | 6 | 6 | 1.6412E7 | 2 | 1 | 7 | Y | 28656 | thrombin-like serine protease [Ophiophagus hannah] |
| 60 | 862 | ABI74694.1 | 144.06 | 6 | 6 | 1.6412E7 | 2 | 1 | 7 | Y | 28447 | serine protease precursor [Philodryas olfersii] |
| 60 | 863 | Q09GK1.1 | 144.06 | 6 | 6 | 1.6412E7 | 2 | 1 | 7 | Y | 28447 | RecName: Full=Snake venom serine protease; Short=SVSP; Flags: Precursor |
| 60 | 964 | ETE66745.1 | 144.06 | 7 | 7 | 1.6412E7 | 2 | 1 | 7 | Y | 24246 | hypothetical protein L345\_07470, partial [Ophiophagus hannah] |
| 60 | 965 | AJB84504.1 | 144.06 | 6 | 6 | 1.6412E7 | 2 | 1 | 7 | Y | 28572 | serine protease precursor [Philodryas chamissonis] |
| 66 | 174 | ABN72542.1 | 144.03 | 2 | 2 |  | 2 | 0 | 3 | Y | 142836 | complement-depleting factor, partial [Bungarus fasciatus] |
| 68 | 395 | T\_164 | 143.89 | 21 | 21 | 6.4244E6 | 3 | 1 | 4 | Y | 17088 | T\_164 |
| 67 | 398 | XP\_032079507.1 | 142.60 | 9 | 9 |  | 3 | 0 | 5 | N | 34421 | ADP-ribosyl cyclase/cyclic ADP-ribose hydrolase 1 [Thamnophis elegans] |
| 67 | 433 | XP\_013915471.1 | 142.60 | 9 | 9 |  | 3 | 0 | 5 | N | 34359 | PREDICTED: ADP-ribosyl cyclase/cyclic ADP-ribose hydrolase 1 [Thamnophis sirtalis] |
| 67 | 434 | XP\_013915472.1 | 142.60 | 9 | 9 |  | 3 | 0 | 5 | N | 34359 | PREDICTED: ADP-ribosyl cyclase/cyclic ADP-ribose hydrolase 1 [Thamnophis sirtalis] |
| 67 | 483 | XP\_034264771.1 | 142.60 | 9 | 9 |  | 3 | 0 | 5 | N | 34352 | ADP-ribosyl cyclase/cyclic ADP-ribose hydrolase 1-like isoform X1 [Pantherophis guttatus] |
| 67 | 509 | XP\_034264772.1 | 142.60 | 10 | 10 |  | 3 | 0 | 5 | N | 28824 | ADP-ribosyl cyclase/cyclic ADP-ribose hydrolase 1-like isoform X2 [Pantherophis guttatus] |
| 24 | 332 | AAM51550.1 | 142.60 | 2 | 2 |  | 3 | 0 | 10 | N | 68176 | mocarhagin 1 [Naja mossambica] |
| 24 | 333 | Q10749.3 | 142.60 | 2 | 2 |  | 3 | 0 | 10 | N | 68176 | RecName: Full=Snake venom metalloproteinase-disintegrin-like mocarhagin; Short=MOC; Short=Mocarhagin-1; Short=SVMP; AltName: Full=Zinc metalloproteinase mocarhagin; Flags: Precursor |
| 42 | 152 | XP\_013928511.1 | 141.83 | 10 | 10 | 6.273E6 | 4 | 1 | 8 | N | 52115 | PREDICTED: hyaluronidase [Thamnophis sirtalis] |
| 42 | 154 | XP\_032077741.1 | 141.83 | 9 | 9 | 6.273E6 | 4 | 1 | 8 | N | 58836 | hyaluronidase-like [Thamnophis elegans] |
| 81 | 438 | XP\_026546198.1 | 136.50 | 10 | 10 | 3.664E7 | 2 | 2 | 4 | N | 31377 | endonuclease domain-containing 1 protein-like isoform X2 [Notechis scutatus] |
| 81 | 439 | XP\_026546199.1 | 136.50 | 10 | 10 | 3.664E7 | 2 | 2 | 4 | N | 31377 | endonuclease domain-containing 1 protein-like isoform X2 [Notechis scutatus] |
| 81 | 440 | XP\_026579205.1 | 136.50 | 10 | 10 | 3.664E7 | 2 | 2 | 4 | N | 31175 | endonuclease domain-containing 1 protein-like [Pseudonaja textilis] |
| 81 | 441 | XP\_026579207.1 | 136.50 | 10 | 10 | 3.664E7 | 2 | 2 | 4 | N | 31175 | endonuclease domain-containing 1 protein-like [Pseudonaja textilis] |
| 81 | 442 | XP\_026546197.1 | 136.50 | 10 | 10 | 3.664E7 | 2 | 2 | 4 | N | 31377 | endonuclease domain-containing 1 protein-like isoform X2 [Notechis scutatus] |
| 81 | 443 | XP\_026579206.1 | 136.50 | 10 | 10 | 3.664E7 | 2 | 2 | 4 | N | 31175 | endonuclease domain-containing 1 protein-like [Pseudonaja textilis] |
| 81 | 444 | XP\_026546196.1 | 136.50 | 10 | 10 | 3.664E7 | 2 | 2 | 4 | N | 33047 | endonuclease domain-containing 1 protein-like isoform X1 [Notechis scutatus] |
| 81 | 445 | JAB54304.1 | 136.50 | 10 | 10 | 3.664E7 | 2 | 2 | 4 | N | 31577 | endonuclease domain-containing 1 protein [Micrurus fulvius] |
| 74 | 450 | XP\_026541916.1 | 135.41 | 9 | 9 | 6.8113E6 | 3 | 1 | 5 | Y | 25191 | glutathione peroxidase 3 isoform X2 [Notechis scutatus] |
| 74 | 451 | XP\_026552407.1 | 135.41 | 9 | 9 | 6.8113E6 | 3 | 1 | 5 | Y | 25168 | glutathione peroxidase 3 isoform X2 [Pseudonaja textilis] |
| 74 | 452 | XP\_026552406.1 | 135.41 | 8 | 8 | 6.8113E6 | 3 | 1 | 5 | Y | 28619 | glutathione peroxidase 3 isoform X1 [Pseudonaja textilis] |
| 74 | 453 | XP\_026541908.1 | 135.41 | 8 | 8 | 6.8113E6 | 3 | 1 | 5 | Y | 28574 | glutathione peroxidase 3 isoform X1 [Notechis scutatus] |
| 74 | 528 | XP\_015679695.1 | 135.41 | 8 | 8 | 6.8113E6 | 3 | 1 | 5 | Y | 27808 | glutathione peroxidase 3 [Protobothrops mucrosquamatus] |
| 74 | 529 | ETE68810.1 | 135.41 | 8 | 8 | 6.8113E6 | 3 | 1 | 5 | Y | 29588 | Glutathione peroxidase 3, partial [Ophiophagus hannah] |
| 55 | 184 | XP\_034277309.1 | 135.03 | 4 | 4 |  | 2 | 0 | 4 | N | 54304 | hyaluronidase [Pantherophis guttatus] |
| 79 | 283 | T\_180 | 134.85 | 54 | 54 | 7.5503E7 | 3 | 3 | 4 | Y | 10090 | T\_180 |
| 63 | 537 | T\_133 | 126.27 | 10 | 10 | 3.5059E7 | 2 | 2 | 4 | N | 16573 | T\_133 |
| 63 | 538 | T\_131 | 126.27 | 10 | 10 | 3.5059E7 | 2 | 2 | 4 | N | 16573 | T\_131 |
| 63 | 539 | T\_132 | 126.27 | 10 | 10 | 3.5059E7 | 2 | 2 | 4 | N | 16573 | T\_132 |
| 63 | 540 | T\_128 | 126.27 | 9 | 9 | 3.5059E7 | 2 | 2 | 4 | N | 17343 | T\_128 |
| 63 | 541 | T\_129 | 126.27 | 7 | 7 | 3.5059E7 | 2 | 2 | 4 | N | 22464 | T\_129 |
| 63 | 542 | T\_130 | 126.27 | 6 | 6 | 3.5059E7 | 2 | 2 | 4 | N | 25392 | T\_130 |
| 64 | 514 | JAC94991.1 | 125.19 | 11 | 11 | 1.0884E6 | 3 | 1 | 6 | N | 40485 | L-amino acid oxidase, partial [Opheodrys aestivus] |
| 75 | 435 | XP\_015677037.1 | 123.58 | 7 | 7 |  | 2 | 0 | 5 | N | 34487 | ADP-ribosyl cyclase/cyclic ADP-ribose hydrolase 1 [Protobothrops mucrosquamatus] |
| 87 | 384 | XP\_026542555.1 | 122.77 | 4 | 4 | 6.5429E6 | 3 | 3 | 3 | N | 53135 | multiple inositol polyphosphate phosphatase 1 isoform X2 [Notechis scutatus] |
| 87 | 385 | XP\_026542553.1 | 122.77 | 4 | 4 | 6.5429E6 | 3 | 3 | 3 | N | 53549 | multiple inositol polyphosphate phosphatase 1 isoform X1 [Notechis scutatus] |
| 87 | 386 | XP\_026575692.1 | 122.77 | 4 | 4 | 6.5429E6 | 3 | 3 | 3 | N | 53443 | multiple inositol polyphosphate phosphatase 1 isoform X2 [Pseudonaja textilis] |
| 87 | 387 | XP\_026575691.1 | 122.77 | 4 | 4 | 6.5429E6 | 3 | 3 | 3 | N | 53858 | multiple inositol polyphosphate phosphatase 1 isoform X1 [Pseudonaja textilis] |
| 87 | 446 | XP\_029139434.1 | 122.77 | 4 | 4 | 6.5429E6 | 3 | 3 | 3 | N | 53980 | multiple inositol polyphosphate phosphatase 1 [Protobothrops mucrosquamatus] |
| 94 | 981 | 0702209A | 120.69 | 19 | 19 | 5.1572E7 | 2 | 2 | 2 | Y | 12832 | phospholipase A |
| 94 | 982 | P00606.2 | 120.69 | 15 | 15 | 5.1572E7 | 2 | 2 | 2 | Y | 15593 | RecName: Full=Acidic phospholipase A2; Short=svPLA2; AltName: Full=Phosphatidylcholine 2-acylhydrolase; Flags: Precursor |
| 94 | 983 | CAA37482.1 | 120.69 | 15 | 15 | 5.1572E7 | 2 | 2 | 2 | Y | 15593 | precursor polypeptide (AA-27 to 118) [Bungarus multicinctus] |
| 90 | 242 | XP\_026541801.1 | 119.34 | 15 | 15 | 7.7057E6 | 3 | 3 | 3 | N | 37577 | cathepsin B [Notechis scutatus] |
| 90 | 243 | XP\_026571757.1 | 119.34 | 15 | 15 | 7.7057E6 | 3 | 3 | 3 | N | 37548 | cathepsin B [Pseudonaja textilis] |
| 40 | 291 | P25668.1 | 119.06 | 28 | 28 | 0 | 2 | 1 | 4 | Y | 7847 | RecName: Full=Long neurotoxin 1; AltName: Full=Toxin A |
| 62 | 292 | 764177A | 118.46 | 29 | 29 | 9.9975E6 | 2 | 1 | 4 | Y | 7706 | toxin B |
| 62 | 293 | P25669.1 | 118.46 | 28 | 28 | 9.9975E6 | 2 | 1 | 4 | Y | 7821 | RecName: Full=Long neurotoxin 2; AltName: Full=Toxin B |
| 70 | 330 | D5LMJ3.1 | 116.03 | 2 | 2 | 1.0451E7 | 2 | 2 | 3 | N | 68254 | RecName: Full=Zinc metalloproteinase-disintegrin-like atrase-A; AltName: Full=Snake venom metalloproteinase; Short=SVMP; Flags: Precursor |
| 70 | 331 | ADF43026.1 | 116.03 | 2 | 2 | 1.0451E7 | 2 | 2 | 3 | N | 68254 | metalloproteinase atrase A [Naja atra] |
| 99 | 475 | AAO84769.1 | 115.37 | 16 | 16 | 2.0292E7 | 2 | 2 | 2 | Y | 15641 | phospholipase A2, partial [Bungarus candidus] |
| 99 | 476 | Q802I1.1 | 115.37 | 16 | 16 | 2.0292E7 | 2 | 2 | 2 | Y | 15641 | RecName: Full=Acidic phospholipase A2 Bc-PL; Short=svPLA2; AltName: Full=Phosphatidylcholine 2-acylhydrolase; Flags: Precursor |
| 73 | 207 | XP\_007420422.1 | 114.32 | 5 | 5 |  | 2 | 0 | 2 | N | 52310 | hyaluronidase-like [Python bivittatus] |
| 59 | 295 | XP\_026555558.1 | 109.48 | 3 | 3 | 7.8543E6 | 1 | 1 | 2 | Y | 61349 | hepatocyte growth factor activator [Pseudonaja textilis] |
| 61 | 414 | CAB62385.1 | 105.60 | 19 | 19 |  | 2 | 0 | 2 | Y | 15092 | beta-bungarotoxin A3 chain, partial [Bungarus multicinctus] |
| 61 | 415 | Q6YNQ0.1 | 105.60 | 19 | 19 |  | 2 | 0 | 2 | Y | 15092 | RecName: Full=Acidic phospholipase A2 beta-bungarotoxin A3 chain; Short=Beta-BuTX A3 chain; Short=svPLA2; AltName: Full=Phosphatidylcholine 2-acylhydrolase; Flags: Precursor |
| 61 | 416 | AAL30064.1 | 105.60 | 19 | 19 |  | 2 | 0 | 2 | Y | 15092 | beta bungaratoxin A3 chain, partial [Bungarus candidus] |
| 61 | 417 | P00619.2 | 105.60 | 18 | 18 |  | 2 | 0 | 2 | Y | 16218 | RecName: Full=Acidic phospholipase A2 beta-bungarotoxin A3 chain; Short=Beta-BuTX A3 chain; Short=svPLA2; AltName: Full=Phosphatidylcholine 2-acylhydrolase; Flags: Precursor |
| 61 | 418 | CAC08197.1 | 105.60 | 18 | 18 |  | 2 | 0 | 2 | Y | 16218 | beta-bungarotoxin A [Bungarus multicinctus multicinctus] |
| 61 | 421 | Q6YIJ3.1 | 105.60 | 19 | 19 |  | 2 | 0 | 2 | Y | 15063 | RecName: Full=Acidic phospholipase A2 beta-bungarotoxin A chain; Short=Beta-BuTX A chain; Short=svPLA2; AltName: Full=Phosphatidylcholine 2-acylhydrolase; Flags: Precursor |
| 61 | 422 | AAN16113.1 | 105.60 | 19 | 19 |  | 2 | 0 | 2 | Y | 15063 | beta bungaratoxin A chain, partial [Bungarus candidus] |
| 69 | 180 | ETE68500.1 | 104.09 | 0 | 0 |  | 0 | 0 | 0 | N | 91914 | Endoplasmic reticulum aminopeptidase 1, partial [Ophiophagus hannah] |
| 71 | 533 | P25672.1 | 97.08 | 14 | 14 |  | 1 | 0 | 3 | Y | 7889 | RecName: Full=Long neurotoxin 4; AltName: Full=Toxin D |
| 102 | 968 | ETE70042.1 | 95.37 | 14 | 14 | 1.2003E7 | 1 | 1 | 2 | N | 13780 | Sulfhydryl oxidase 1, partial [Ophiophagus hannah] |
| 95 | 436 | S59517 | 95.07 | 4 | 4 | 6.8007E5 | 2 | 2 | 2 | N | 69799 | serum albumin precursor - monocled cobra |
| 95 | 437 | CAA55333.1 | 95.07 | 4 | 4 | 6.8007E5 | 2 | 2 | 2 | N | 69799 | cobra serum albumin [Naja naja] |
| 80 | 257 | XP\_034296607.1 | 94.08 | 0 | 0 |  | 0 | 0 | 0 | N | 98369 | endoplasmic reticulum aminopeptidase 1 isoform X2 [Pantherophis guttatus] |
| 80 | 258 | XP\_034296605.1 | 94.08 | 0 | 0 |  | 0 | 0 | 0 | N | 106081 | endoplasmic reticulum aminopeptidase 1 isoform X1 [Pantherophis guttatus] |
| 80 | 259 | XP\_034296606.1 | 94.08 | 0 | 0 |  | 0 | 0 | 0 | N | 106081 | endoplasmic reticulum aminopeptidase 1 isoform X1 [Pantherophis guttatus] |
| 89 | 1067 | 1702215A | 93.85 | 39 | 39 | 4.1941E7 | 2 | 2 | 3 | Y | 6508 | chymotrypsin inhibitor |
| 89 | 1068 | P19859.1 | 93.85 | 39 | 39 | 4.1941E7 | 2 | 2 | 3 | Y | 6508 | RecName: Full=Kunitz-type serine protease inhibitor; AltName: Full=Venom chymotrypsin inhibitor |
| 84 | 222 | XP\_026553321.1 | 92.53 | 3 | 3 |  | 1 | 0 | 1 | N | 64313 | phospholipase B-like 1 [Pseudonaja textilis] |
| 84 | 223 | F8J2D3.1 | 92.53 | 3 | 3 |  | 1 | 0 | 1 | N | 64103 | RecName: Full=Phospholipase-B 81; Short=PLB; Flags: Precursor |
| 84 | 224 | ACR78473.1 | 92.53 | 3 | 3 |  | 1 | 0 | 1 | N | 64103 | putative phospholipase B 81b [Drysdalia coronoides] |
| 76 | 1106 | Q9W717.1 | 89.64 | 29 | 29 | 1.5388E7 | 1 | 1 | 1 | Y | 9695 | RecName: Full=Neurotoxin-like protein NTL2; Flags: Precursor |
| 76 | 1107 | APB88857.1 | 89.64 | 29 | 29 | 1.5388E7 | 1 | 1 | 1 | Y | 9695 | neurotoxin-like protein [Naja naja] |
| 76 | 1108 | CAB45156.1 | 89.64 | 29 | 29 | 1.5388E7 | 1 | 1 | 1 | Y | 9695 | neurotoxin-like protein [Naja atra] |
| 86 | 423 | JAC94989.1 | 86.74 | 5 | 5 | 6.9018E5 | 2 | 1 | 2 | N | 63907 | Phospholipase B [Opheodrys aestivus] |
| 97 | 858 | 5MG9 | 82.53 | 29 | 29 | 1.6197E7 | 1 | 1 | 1 | Y | 7244 | Chain A, Anctx1-w28r/i38s |
| 97 | 995 | P82463.1 | 82.53 | 29 | 29 | 1.6197E7 | 1 | 1 | 1 | Y | 7298 | RecName: Full=Muscarinic toxin-like protein 2; Short=MTLP-2 |
| 91 | 723 | XP\_015669101.1 | 81.42 | 3 | 3 | 6.8478E5 | 1 | 1 | 2 | N | 57981 | prosaposin [Protobothrops mucrosquamatus] |
| 91 | 724 | BAN82088.1 | 81.42 | 2 | 2 | 6.8478E5 | 1 | 1 | 2 | N | 58564 | hypothetical protein [Protobothrops flavoviridis] |
| 91 | 725 | XP\_032088229.1 | 81.42 | 3 | 3 | 6.8478E5 | 1 | 1 | 2 | N | 58050 | prosaposin-like isoform X2 [Thamnophis elegans] |
| 91 | 726 | XP\_032088228.1 | 81.42 | 2 | 2 | 6.8478E5 | 1 | 1 | 2 | N | 58435 | prosaposin-like isoform X1 [Thamnophis elegans] |
| 91 | 823 | AFJ51008.1 | 81.42 | 3 | 3 | 6.8478E5 | 1 | 1 | 2 | N | 58013 | Proactivator polypeptide-like [Crotalus adamanteus] |
| 91 | 824 | XP\_026528620.1 | 81.42 | 3 | 3 | 6.8478E5 | 1 | 1 | 2 | N | 58373 | prosaposin-like isoform X2 [Notechis scutatus] |
| 91 | 825 | XP\_026569354.1 | 81.42 | 3 | 3 | 6.8478E5 | 1 | 1 | 2 | N | 58289 | prosaposin isoform X2 [Pseudonaja textilis] |
| 91 | 826 | XP\_013923763.1 | 81.42 | 3 | 3 | 6.8478E5 | 1 | 1 | 2 | N | 58050 | PREDICTED: prosaposin isoform X2 [Thamnophis sirtalis] |
| 91 | 827 | XP\_013923762.1 | 81.42 | 2 | 2 | 6.8478E5 | 1 | 1 | 2 | N | 58435 | PREDICTED: prosaposin isoform X1 [Thamnophis sirtalis] |
| 91 | 828 | XP\_026528619.1 | 81.42 | 2 | 2 | 6.8478E5 | 1 | 1 | 2 | N | 58759 | prosaposin-like isoform X1 [Notechis scutatus] |
| 91 | 829 | XP\_026569353.1 | 81.42 | 2 | 2 | 6.8478E5 | 1 | 1 | 2 | N | 58674 | prosaposin isoform X1 [Pseudonaja textilis] |
| 91 | 830 | XP\_034291658.1 | 81.42 | 2 | 2 | 6.8478E5 | 1 | 1 | 2 | N | 58438 | prosaposin-like isoform X4 [Pantherophis guttatus] |
| 91 | 831 | XP\_034291656.1 | 81.42 | 2 | 2 | 6.8478E5 | 1 | 1 | 2 | N | 58696 | prosaposin-like isoform X2 [Pantherophis guttatus] |
| 91 | 832 | XP\_034291657.1 | 81.42 | 2 | 2 | 6.8478E5 | 1 | 1 | 2 | N | 58695 | prosaposin-like isoform X3 [Pantherophis guttatus] |
| 91 | 833 | XP\_034291655.1 | 81.42 | 2 | 2 | 6.8478E5 | 1 | 1 | 2 | N | 58824 | prosaposin-like isoform X1 [Pantherophis guttatus] |
| 91 | 864 | ETE61271.1 | 81.42 | 3 | 3 | 6.8478E5 | 1 | 1 | 2 | N | 54055 | Proactivator polypeptide [Ophiophagus hannah] |
| 91 | 909 | XP\_007441356.1 | 81.42 | 3 | 3 | 6.8478E5 | 1 | 1 | 2 | N | 42934 | prosaposin-like [Python bivittatus] |
| 92 | 352 | ETE64512.1 | 80.86 | 1 | 1 | 9.7335E5 | 1 | 1 | 1 | N | 105984 | Golgi apparatus protein 1, partial [Ophiophagus hannah] |
| 92 | 353 | XP\_026535930.1 | 80.86 | 1 | 1 | 9.7335E5 | 1 | 1 | 1 | N | 132789 | Golgi apparatus protein 1 [Notechis scutatus] |
| 92 | 354 | XP\_026570202.1 | 80.86 | 1 | 1 | 9.7335E5 | 1 | 1 | 1 | N | 133145 | Golgi apparatus protein 1 [Pseudonaja textilis] |
| 92 | 355 | LAB62693.1 | 80.86 | 1 | 1 | 9.7335E5 | 1 | 1 | 1 | N | 96178 | hypothetical protein [Micrurus surinamensis] |
| 92 | 356 | LAB62690.1 | 80.86 | 1 | 1 | 9.7335E5 | 1 | 1 | 1 | N | 122130 | hypothetical protein, partial [Micrurus surinamensis] |
| 92 | 467 | LAB18298.1 | 80.86 | 4 | 4 | 9.7335E5 | 1 | 1 | 1 | N | 30012 | hypothetical protein, partial [Micrurus spixii] |
| 92 | 468 | LAB18300.1 | 80.86 | 3 | 3 | 9.7335E5 | 1 | 1 | 1 | N | 39264 | hypothetical protein, partial [Micrurus spixii] |
| 96 | 798 | ETE56933.1 | 80.02 | 9 | 9 | 2.4038E7 | 1 | 1 | 2 | Y | 15656 | hypothetical protein L345\_17355, partial [Ophiophagus hannah] |
| 96 | 1003 | 1IJC | 80.02 | 19 | 19 | 2.4038E7 | 1 | 1 | 2 | Y | 7285 | Chain A, Solution Structure Of Bucandin, A Neurotoxin From The Venom Of The Malayan Krait |
| 96 | 1004 | 1F94 | 80.02 | 19 | 19 | 2.4038E7 | 1 | 1 | 2 | Y | 7285 | Chain A, Bucandin |
| 96 | 1005 | P81782.1 | 80.02 | 19 | 19 | 2.4038E7 | 1 | 1 | 2 | Y | 7285 | RecName: Full=Bucandin |
| 88 | 459 | JAC96437.1 | 79.39 | 2 | 2 | 2.2564E6 | 1 | 1 | 1 | N | 68086 | Metalloproteinase (type III) 2 [Hypsiglena sp. JMG-2014] |
| 88 | 460 | JAS04919.1 | 79.39 | 2 | 2 | 2.2564E6 | 1 | 1 | 1 | N | 68086 | Metalloproteinase type III 2 [Hypsiglena sp. JMG-2014] |
| 88 | 487 | JAS04923.1 | 79.39 | 2 | 2 | 2.2564E6 | 1 | 1 | 1 | N | 68472 | Metalloproteinase type III 14a [Hypsiglena sp. JMG-2014] |
| 88 | 488 | JAC96439.1 | 79.39 | 2 | 2 | 2.2564E6 | 1 | 1 | 1 | N | 68510 | Metalloproteinase (type III) 14c [Hypsiglena sp. JMG-2014] |
| 88 | 489 | JAS04921.1 | 79.39 | 2 | 2 | 2.2564E6 | 1 | 1 | 1 | N | 68510 | Metalloproteinase type III 14c [Hypsiglena sp. JMG-2014] |
| 88 | 490 | JAS04922.1 | 79.39 | 2 | 2 | 2.2564E6 | 1 | 1 | 1 | N | 68488 | Metalloproteinase type III 14b [Hypsiglena sp. JMG-2014] |
| 88 | 491 | JAC96441.1 | 79.39 | 2 | 2 | 2.2564E6 | 1 | 1 | 1 | N | 68472 | Metalloproteinase (type III) 14a [Hypsiglena sp. JMG-2014] |
| 88 | 492 | JAC96440.1 | 79.39 | 2 | 2 | 2.2564E6 | 1 | 1 | 1 | N | 68488 | Metalloproteinase (type III) 14b [Hypsiglena sp. JMG-2014] |
| 101 | 585 | P62377.1 | 72.90 | 15 | 15 | 0 | 1 | 1 | 1 | N | 7014 | RecName: Full=Cytotoxin-like basic protein; Short=CLBP; AltName: Full=Less-cytotoxic basic polypeptide; Short=LCBP |
| 101 | 586 | 1CVO | 72.90 | 15 | 15 | 0 | 1 | 1 | 1 | N | 7014 | Chain A, CARDIOTOXIN V |
| 101 | 587 | 1KXI | 72.90 | 15 | 15 | 0 | 1 | 1 | 1 | N | 7014 | Chain B, Structure Of Cytotoxin Homolog Precursor |
| 101 | 588 | Q91137.1 | 72.90 | 11 | 11 | 0 | 1 | 1 | 1 | N | 9309 | RecName: Full=Cytotoxin homolog 5; AltName: Full=Cardiotoxin V-like; Flags: Precursor |
| 101 | 589 | CAB50697.1 | 72.90 | 11 | 11 | 0 | 1 | 1 | 1 | N | 9333 | snake venom [Naja atra] |
| 101 | 590 | AAB36928.1 | 72.90 | 11 | 11 | 0 | 1 | 1 | 1 | N | 9305 | cardiotoxin 7'' [Naja atra] |
| 101 | 591 | Q91996.1 | 72.90 | 11 | 11 | 0 | 1 | 1 | 1 | N | 9305 | RecName: Full=Cardiotoxin 7''; AltName: Full=Cardiotoxin 7'; AltName: Full=Cardiotoxin VII; Flags: Precursor |
| 101 | 592 | AAB36929.1 | 72.90 | 11 | 11 | 0 | 1 | 1 | 1 | N | 9319 | cardiotoxin 7a [Naja atra] |
| 101 | 593 | CAA63979.1 | 72.90 | 11 | 11 | 0 | 1 | 1 | 1 | N | 9309 | cardiotoxin V-like protein [Naja atra] |
| 101 | 594 | P62375.1 | 72.90 | 11 | 11 | 0 | 1 | 1 | 1 | N | 9323 | RecName: Full=Cytotoxin A5; AltName: Full=CTX A5; Short=CT A5; AltName: Full=Cardiotoxin A5; AltName: Full=Cardiotoxin V; Short=CTX V; Short=CTXV; AltName: Full=Cardiotoxin-like basic polypeptide; Short=CLBP; Flags: Precursor |
| 101 | 595 | AAB03220.1 | 72.90 | 11 | 11 | 0 | 1 | 1 | 1 | N | 9319 | cardiotoxin 7a [Naja atra] |
| 101 | 596 | AAB03219.1 | 72.90 | 11 | 11 | 0 | 1 | 1 | 1 | N | 9305 | cardiotoxin 7'' [Naja atra] |
| 101 | 597 | Q9W716.1 | 72.90 | 11 | 11 | 0 | 1 | 1 | 1 | N | 9333 | RecName: Full=Cytotoxin homolog 5V; AltName: Full=Cardiotoxin-5V; Short=CTX5V; Flags: Precursor |
| 101 | 598 | AAB18387.1 | 72.90 | 11 | 11 | 0 | 1 | 1 | 1 | N | 9305 | cardiotoxin 7' [Naja atra] |
| 101 | 599 | CAA90964.1 | 72.90 | 11 | 11 | 0 | 1 | 1 | 1 | N | 9323 | cardiotoxin V [Naja naja] |
| 101 | 600 | Q91126.1 | 72.90 | 11 | 11 | 0 | 1 | 1 | 1 | N | 9319 | RecName: Full=Cardiotoxin 7a; Flags: Precursor |
| 101 | 601 | AAB01540.1 | 72.90 | 11 | 11 | 0 | 1 | 1 | 1 | N | 9305 | cardiotoxin VII [Naja atra] |
| 101 | 1110 | P0C547.1 | 72.90 | 43 | 43 | 0 | 1 | 1 | 1 | N | 2400 | RecName: Full=Cardiotoxin-like basic polypeptide ah; Short=CLBPah |
| 101 | 1111 | P62394.1 | 72.90 | 15 | 15 | 0 | 1 | 1 | 1 | N | 6842 | RecName: Full=Cytotoxin 11; AltName: Full=Toxin CM-12 |
| 101 | 1112 | P14541.1 | 72.90 | 15 | 15 | 0 | 1 | 1 | 1 | N | 6994 | RecName: Full=Cytotoxin homolog; AltName: Full=Cytotoxin-like basic protein; Short=CLBP |
| 101 | 1113 | P62390.1 | 72.90 | 15 | 15 | 0 | 1 | 1 | 1 | N | 6842 | RecName: Full=Cytotoxin 11; AltName: Full=Toxin CM-13a |
| 98 | 470 | JAA74859.1 | 71.97 | 3 | 3 |  | 1 | 0 | 1 | Y | 53521 | SVMP-Hop-46, partial [Hoplocephalus bungaroides] |
| 113 | 712 | XP\_026555881.1 | 71.91 | 2 | 2 | 2.3471E5 | 1 | 1 | 1 | N | 60632 | N-acetylglucosamine-6-sulfatase [Pseudonaja textilis] |
| 113 | 718 | XP\_034259005.1 | 71.91 | 2 | 2 | 2.3471E5 | 1 | 1 | 1 | N | 61079 | N-acetylglucosamine-6-sulfatase [Pantherophis guttatus] |
| 113 | 719 | ETE66000.1 | 71.91 | 3 | 3 | 2.3471E5 | 1 | 1 | 1 | N | 55155 | N-acetylglucosamine-6-sulfatase, partial [Ophiophagus hannah] |
| 113 | 720 | LAA18030.1 | 71.91 | 3 | 3 | 2.3471E5 | 1 | 1 | 1 | N | 56296 | hypothetical protein, partial [Micrurus lemniscatus carvalhoi] |
| 113 | 721 | LAA85609.1 | 71.91 | 2 | 2 | 2.3471E5 | 1 | 1 | 1 | N | 61053 | hypothetical protein [Micrurus lemniscatus lemniscatus] |
| 113 | 722 | LAA85607.1 | 71.91 | 2 | 2 | 2.3471E5 | 1 | 1 | 1 | N | 61053 | hypothetical protein [Micrurus lemniscatus lemniscatus] |
| 113 | 841 | XP\_026523569.1 | 71.91 | 2 | 2 | 2.3471E5 | 1 | 1 | 1 | N | 60676 | N-acetylglucosamine-6-sulfatase [Notechis scutatus] |
| 113 | 842 | XP\_032076778.1 | 71.91 | 2 | 2 | 2.3471E5 | 1 | 1 | 1 | N | 61159 | N-acetylglucosamine-6-sulfatase [Thamnophis elegans] |
| 113 | 843 | XP\_013929985.1 | 71.91 | 2 | 2 | 2.3471E5 | 1 | 1 | 1 | N | 61166 | PREDICTED: N-acetylglucosamine-6-sulfatase [Thamnophis sirtalis] |
| 85 | 768 | 1T37 | 71.14 | 10 | 10 |  | 1 | 0 | 1 | Y | 13162 | Chain A, Phospholipase A2 Isoform 3 |
| 85 | 769 | 1ZM6 | 71.14 | 10 | 10 |  | 1 | 0 | 1 | Y | 13162 | Chain A, Phospholipase A2 Isoform 3 |
| 116 | 604 | T\_79 | 71.01 | 3 | 3 | 2.6904E5 | 1 | 1 | 1 | N | 48214 | T\_79 |
| 128 | 1049 | XP\_034269327.1 | 70.41 | 4 | 4 | 2.5623E5 | 1 | 1 | 1 | N | 30735 | palmitoyl-protein thioesterase 1 isoform X2 [Pantherophis guttatus] |
| 128 | 1050 | XP\_013915921.1 | 70.41 | 4 | 4 | 2.5623E5 | 1 | 1 | 1 | N | 31606 | PREDICTED: palmitoyl-protein thioesterase 1 isoform X2 [Thamnophis sirtalis] |
| 128 | 1051 | XP\_015686279.2 | 70.41 | 4 | 4 | 2.5623E5 | 1 | 1 | 1 | N | 31521 | palmitoyl-protein thioesterase 1 [Protobothrops mucrosquamatus] |
| 128 | 1053 | XP\_026536429.1 | 70.41 | 3 | 3 | 2.5623E5 | 1 | 1 | 1 | N | 34292 | palmitoyl-protein thioesterase 1 [Notechis scutatus] |
| 128 | 1054 | XP\_026558426.1 | 70.41 | 3 | 3 | 2.5623E5 | 1 | 1 | 1 | N | 34281 | palmitoyl-protein thioesterase 1 [Pseudonaja textilis] |
| 128 | 1055 | XP\_007430564.1 | 70.41 | 3 | 3 | 2.5623E5 | 1 | 1 | 1 | N | 34760 | palmitoyl-protein thioesterase 1 [Python bivittatus] |
| 128 | 1065 | XP\_032084406.1 | 70.41 | 3 | 3 | 2.5623E5 | 1 | 1 | 1 | N | 35338 | palmitoyl-protein thioesterase 1 [Thamnophis elegans] |
| 128 | 1066 | XP\_034269326.1 | 70.41 | 3 | 3 | 2.5623E5 | 1 | 1 | 1 | N | 35574 | palmitoyl-protein thioesterase 1 isoform X1 [Pantherophis guttatus] |
| 117 | 637 | LAA21880.1 | 69.46 | 3 | 3 | 5.3487E5 | 1 | 1 | 1 | N | 55386 | hypothetical protein, partial [Micrurus lemniscatus carvalhoi] |
| 117 | 638 | LAA21878.1 | 69.46 | 2 | 2 | 5.3487E5 | 1 | 1 | 1 | N | 76625 | hypothetical protein [Micrurus lemniscatus carvalhoi] |
| 117 | 681 | XP\_026547769.1 | 69.46 | 2 | 2 | 5.3487E5 | 1 | 1 | 1 | N | 78354 | serotransferrin [Notechis scutatus] |
| 117 | 790 | XP\_026557455.1 | 69.46 | 2 | 2 | 5.3487E5 | 1 | 1 | 1 | N | 77840 | serotransferrin [Pseudonaja textilis] |
| 100 | 536 | XP\_013918203.1 | 65.62 | 0 | 0 |  | 0 | 0 | 0 | N | 33025 | PREDICTED: snake venom 5'-nucleotidase-like, partial [Thamnophis sirtalis] |
| 109 | 851 | AAL54920.1 | 60.95 | 18 | 18 | 1.7802E7 | 1 | 1 | 1 | Y | 16114 | PLA2 [Hydrophis hardwickii] |
| 109 | 1080 | ABN54807.1 | 60.95 | 18 | 18 | 1.7802E7 | 1 | 1 | 1 | Y | 16078 | phospholipase A2 73 [Hydrophis hardwickii] |
| 129 | 1086 | ETE72142.1 | 59.33 | 4 | 4 | 5.98E5 | 1 | 1 | 1 | N | 24461 | Ribonuclease T2 [Ophiophagus hannah] |
| 129 | 1087 | AFJ51166.1 | 59.33 | 3 | 3 | 5.98E5 | 1 | 1 | 1 | N | 30438 | Ribonuclease T2-like [Crotalus adamanteus] |
| 124 | 845 | XP\_013922279.1 | 56.85 | 7 | 7 | 1.7318E7 | 1 | 1 | 1 | Y | 18221 | PREDICTED: hemorrhagic metalloproteinase-disintegrin-like kaouthiagin, partial [Thamnophis sirtalis] |
| 124 | 1088 | XP\_032084681.1 | 56.85 | 2 | 2 | 1.7318E7 | 1 | 1 | 1 | Y | 69288 | zinc metalloproteinase-disintegrin-like MTP9 [Thamnophis elegans] |
| 112 | 694 | AAL30061.1 | 51.91 | 16 | 16 | 2.2026E6 | 1 | 1 | 1 | Y | 9758 | weak toxin 3 [Bungarus candidus] |
| 112 | 695 | Q8AY49.1 | 51.91 | 16 | 16 | 2.2026E6 | 1 | 1 | 1 | Y | 9758 | RecName: Full=Weak toxin 3; Flags: Precursor |
| 112 | 696 | Q8AY51.1 | 51.91 | 16 | 16 | 2.2026E6 | 1 | 1 | 1 | Y | 9713 | RecName: Full=Weak toxin 1; Flags: Precursor |
| 112 | 697 | AAL30059.1 | 51.91 | 16 | 16 | 2.2026E6 | 1 | 1 | 1 | Y | 9713 | weak toxin 1 [Bungarus candidus] |
| 112 | 1116 | A2CKF7.1 | 51.91 | 16 | 16 | 2.2026E6 | 1 | 1 | 1 | Y | 9723 | RecName: Full=Neurotoxin 3FTx-LT; Flags: Precursor |
| 112 | 1117 | CAA11133.1 | 51.91 | 16 | 16 | 2.2026E6 | 1 | 1 | 1 | Y | 9845 | unnamed protein product [Naja atra] |
| 112 | 1118 | ADF50021.1 | 51.91 | 16 | 16 | 2.2026E6 | 1 | 1 | 1 | Y | 9740 | non-conventional three finger toxin isoform 6 [Bungarus flaviceps] |
| 112 | 1119 | ABI33871.1 | 51.91 | 16 | 16 | 2.2026E6 | 1 | 1 | 1 | Y | 9723 | three finger toxin [Bungarus fasciatus] |
| 112 | 1120 | AAT38875.1 | 51.91 | 16 | 16 | 2.2026E6 | 1 | 1 | 1 | Y | 9723 | Ntx4 [Bungarus candidus] |
| 112 | 1121 | AAL30060.1 | 51.91 | 16 | 16 | 2.2026E6 | 1 | 1 | 1 | Y | 9652 | weak toxin 2 [Bungarus candidus] |
| 112 | 1122 | Q8AY50.1 | 51.91 | 16 | 16 | 2.2026E6 | 1 | 1 | 1 | Y | 9652 | RecName: Full=Weak toxin 2; Flags: Precursor |
| 112 | 1123 | Q9YGI2.1 | 51.91 | 16 | 16 | 2.2026E6 | 1 | 1 | 1 | Y | 9845 | RecName: Full=Probable weak neurotoxin NNAM1; Flags: Precursor |
| 112 | 1124 | ADF50022.1 | 51.91 | 16 | 16 | 2.2026E6 | 1 | 1 | 1 | Y | 9755 | non-conventional three finger toxin isoform 7 [Bungarus flaviceps] |
| 112 | 1125 | ADF50020.1 | 51.91 | 16 | 16 | 2.2026E6 | 1 | 1 | 1 | Y | 9679 | non-conventional three finger toxin isoform 5 [Bungarus flaviceps] |
| 112 | 1126 | ADN67577.1 | 51.91 | 16 | 16 | 2.2026E6 | 1 | 1 | 1 | Y | 9858 | three-finger toxin precursor [Naja atra] |
| 112 | 1127 | CAA11134.1 | 51.91 | 16 | 16 | 2.2026E6 | 1 | 1 | 1 | Y | 9743 | unnamed protein product [Naja atra] |
| 112 | 1128 | Q6IZ95.1 | 51.91 | 16 | 16 | 2.2026E6 | 1 | 1 | 1 | Y | 9723 | RecName: Full=Neurotoxin Ntx4; Flags: Precursor |
| 112 | 1129 | Q9YGI1.1 | 51.91 | 16 | 16 | 2.2026E6 | 1 | 1 | 1 | Y | 9743 | RecName: Full=Probable weak neurotoxin NNAM3; Flags: Precursor |
| 118 | 743 | XP\_007425787.1 | 50.53 | 3 | 3 | 3.2389E5 | 1 | 1 | 1 | Y | 42635 | vascular endothelial growth factor C, partial [Python bivittatus] |
| 118 | 744 | XP\_034298589.1 | 50.53 | 3 | 3 | 3.2389E5 | 1 | 1 | 1 | Y | 42840 | vascular endothelial growth factor C isoform X2 [Pantherophis guttatus] |
| 118 | 745 | JAC96561.1 | 50.53 | 3 | 3 | 3.2389E5 | 1 | 1 | 1 | Y | 47649 | Vascular endothelial growth factor C [Echis coloratus] |
| 118 | 746 | JAI10614.1 | 50.53 | 3 | 3 | 3.2389E5 | 1 | 1 | 1 | Y | 47697 | Vascular endothelial growth factor C-like protein [Crotalus adamanteus] |
| 118 | 747 | SMD28268.1 | 50.53 | 3 | 3 | 3.2389E5 | 1 | 1 | 1 | Y | 47611 | vascular endothelial growth factor CdtVEGF3 [Crotalus durissus terrificus] |
| 118 | 748 | JAC95037.1 | 50.53 | 3 | 3 | 3.2389E5 | 1 | 1 | 1 | Y | 47507 | Vascular endothelial growth factor C [Pantherophis guttatus] |
| 118 | 749 | JAV48556.1 | 50.53 | 3 | 3 | 3.2389E5 | 1 | 1 | 1 | Y | 47653 | Vascular endothelial growth factor C-like protein [Agkistrodon contortrix contortrix] |
| 118 | 750 | JAA95033.1 | 50.53 | 3 | 3 | 3.2389E5 | 1 | 1 | 1 | Y | 47639 | Vascular endothelial growth factor C-like protein [Crotalus horridus] |
| 118 | 751 | XP\_034298588.1 | 50.53 | 3 | 3 | 3.2389E5 | 1 | 1 | 1 | Y | 47507 | vascular endothelial growth factor C isoform X1 [Pantherophis guttatus] |
| 118 | 752 | XP\_013912093.1 | 50.53 | 3 | 3 | 3.2389E5 | 1 | 1 | 1 | Y | 47484 | PREDICTED: vascular endothelial growth factor C [Thamnophis sirtalis] |
| 118 | 753 | ASU45066.1 | 50.53 | 3 | 3 | 3.2389E5 | 1 | 1 | 1 | Y | 47699 | vascular endothelial growth factor C [Daboia russelii] |
| 118 | 754 | JAC94939.1 | 50.53 | 3 | 3 | 3.2389E5 | 1 | 1 | 1 | Y | 47538 | Vascular endothelial growth factor C [Python regius] |
| 118 | 755 | XP\_029139565.1 | 50.53 | 3 | 3 | 3.2389E5 | 1 | 1 | 1 | Y | 47709 | vascular endothelial growth factor C [Protobothrops mucrosquamatus] |
| 118 | 848 | JAC94973.1 | 50.53 | 4 | 4 | 3.2389E5 | 1 | 1 | 1 | Y | 40057 | Vascular endothelial growth factor C [Opheodrys aestivus] |
| 118 | 849 | XP\_034298590.1 | 50.53 | 4 | 4 | 3.2389E5 | 1 | 1 | 1 | Y | 40062 | vascular endothelial growth factor C isoform X3 [Pantherophis guttatus] |
| 118 | 942 | JAI08988.1 | 50.53 | 3 | 3 | 3.2389E5 | 1 | 1 | 1 | Y | 47564 | Vascular endothelial growth factor 2 [Micrurus fulvius] |
| 118 | 943 | ETE60014.1 | 50.53 | 3 | 3 | 3.2389E5 | 1 | 1 | 1 | Y | 47557 | Vascular endothelial growth factor C [Ophiophagus hannah] |
| 118 | 944 | JAB52939.1 | 50.53 | 3 | 3 | 3.2389E5 | 1 | 1 | 1 | Y | 47564 | vascular endothelial growth factor C [Micrurus fulvius] |
| 118 | 945 | T\_45 | 50.53 | 3 | 3 | 3.2389E5 | 1 | 1 | 1 | Y | 47592 | T\_45 |
| 118 | 946 | JAS04977.1 | 50.53 | 3 | 3 | 3.2389E5 | 1 | 1 | 1 | Y | 47564 | Vascular endothelial growth factor 2 [Micrurus fulvius] |
| 118 | 947 | XP\_026522979.1 | 50.53 | 3 | 3 | 3.2389E5 | 1 | 1 | 1 | Y | 47528 | vascular endothelial growth factor C [Notechis scutatus] |
| 118 | 948 | XP\_026555459.1 | 50.53 | 3 | 3 | 3.2389E5 | 1 | 1 | 1 | Y | 47615 | vascular endothelial growth factor C [Pseudonaja textilis] |
| 118 | 949 | JAI09156.1 | 50.53 | 3 | 3 | 3.2389E5 | 1 | 1 | 1 | Y | 47564 | Vascular endothelial growth factor C-like protein [Micrurus fulvius] |
| 118 | 950 | LAA17506.1 | 50.53 | 3 | 3 | 3.2389E5 | 1 | 1 | 1 | Y | 53369 | hypothetical protein, partial [Micrurus lemniscatus carvalhoi] |
| 118 | 951 | LAB17921.1 | 50.53 | 3 | 3 | 3.2389E5 | 1 | 1 | 1 | Y | 53456 | hypothetical protein, partial [Micrurus spixii] |
| 118 | 1140 | T\_47 | 50.53 | 4 | 4 | 3.2389E5 | 1 | 1 | 1 | Y | 32628 | T\_47 |
| 118 | 1141 | LAB17920.1 | 50.53 | 4 | 4 | 3.2389E5 | 1 | 1 | 1 | Y | 32584 | hypothetical protein [Micrurus spixii] |
| 118 | 1142 | T\_46 | 50.53 | 4 | 4 | 3.2389E5 | 1 | 1 | 1 | Y | 40133 | T\_46 |
| 130 | 1149 | ETE59007.1 | 50.29 | 0 | 0 |  | 0 | 0 | 0 | N | 9348 | hypothetical protein L345\_15265, partial [Ophiophagus hannah] |
| 115 | 549 | QIV64750.1 | 49.29 | 0 | 0 |  | 0 | 0 | 0 | N | 54040 | MAD-6 [Crotalus horridus] |
| 115 | 705 | AFJ49237.1 | 49.29 | 0 | 0 |  | 0 | 0 | 0 | N | 67994 | Snake venom metalloproteinase (type III) 2e [Crotalus adamanteus] |
| 115 | 706 | AAN39540.1 | 49.29 | 0 | 0 |  | 0 | 0 | 0 | N | 67652 | metalloprotease [Gloydius halys] |
| 115 | 707 | JC8056 | 49.29 | 0 | 0 |  | 0 | 0 | 0 | N | 67652 | halysase - Gloydius halys |
| 115 | 856 | QIV64763.1 | 49.29 | 0 | 0 |  | 0 | 0 | 0 | N | 68265 | MDC-8 [Crotalus durissus terrificus] |
| 115 | 892 | JAS05305.1 | 49.29 | 0 | 0 |  | 0 | 0 | 0 | N | 54402 | Metalloproteinase type II 2 [Sistrurus tergeminus] |
| 115 | 893 | JAS04678.1 | 49.29 | 0 | 0 |  | 0 | 0 | 0 | N | 67914 | Metalloproteinase type III 4c [Crotalus adamanteus] |
| 115 | 894 | AFJ49236.1 | 49.29 | 0 | 0 |  | 0 | 0 | 0 | N | 67850 | Snake venom metalloproteinase (type III) 2d [Crotalus adamanteus] |
| 115 | 895 | JAI10382.1 | 49.29 | 0 | 0 |  | 0 | 0 | 0 | N | 67914 | Metalloproteinase (type III) 4c [Crotalus adamanteus] |
| 115 | 896 | AFJ49235.1 | 49.29 | 0 | 0 |  | 0 | 0 | 0 | N | 67914 | Snake venom metalloproteinase (type III) 2c [Crotalus adamanteus] |
| 115 | 897 | AFJ49233.1 | 49.29 | 0 | 0 |  | 0 | 0 | 0 | N | 67944 | Snake venom metalloproteinase (type III) 2a [Crotalus adamanteus] |
| 115 | 898 | JAI10384.1 | 49.29 | 0 | 0 |  | 0 | 0 | 0 | N | 67944 | Metalloproteinase (type III) 4a [Crotalus adamanteus] |
| 115 | 899 | JAS04680.1 | 49.29 | 0 | 0 |  | 0 | 0 | 0 | N | 67944 | Metalloproteinase type III 4a [Crotalus adamanteus] |
| 115 | 957 | QIV64952.1 | 49.29 | 0 | 0 |  | 0 | 0 | 0 | N | 35986 | MDC-8a, partial [Crotalus atrox] |
| 115 | 958 | JAS05411.1 | 49.29 | 0 | 0 |  | 0 | 0 | 0 | N | 68316 | Metalloproteinase type III 7b [Sistrurus miliarius barbouri] |
| 115 | 959 | JAS05412.1 | 49.29 | 0 | 0 |  | 0 | 0 | 0 | N | 68472 | Metalloproteinase type III 7a [Sistrurus miliarius barbouri] |
| 115 | 1150 | JAS05304.1 | 49.29 | 0 | 0 |  | 0 | 0 | 0 | N | 54548 | Metalloproteinase type II 3 [Sistrurus tergeminus] |
| 115 | 1151 | QIV64951.1 | 49.29 | 0 | 0 |  | 0 | 0 | 0 | N | 54561 | MPF-1a [Crotalus atrox] |
| 115 | 1152 | JAS05442.1 | 49.29 | 0 | 0 |  | 0 | 0 | 0 | N | 54711 | Metalloproteinase type II 1 [Sistrurus miliarius barbouri] |
| 115 | 1153 | AJI77146.1 | 49.29 | 0 | 0 |  | 0 | 0 | 0 | N | 67670 | metalloprotease precursor [Gloydius intermedius] |
| 115 | 1154 | JAV48406.1 | 49.29 | 0 | 0 |  | 0 | 0 | 0 | N | 67959 | Cadam10\_SVMPIII-8 [Crotalus adamanteus] |
| 115 | 1155 | JAI10383.1 | 49.29 | 0 | 0 |  | 0 | 0 | 0 | N | 67997 | Metalloproteinase (type III) 4b [Crotalus adamanteus] |
| 115 | 1156 | JAI10380.1 | 49.29 | 0 | 0 |  | 0 | 0 | 0 | N | 67903 | Metalloproteinase (type III) 4e [Crotalus adamanteus] |
| 115 | 1157 | JAS04676.1 | 49.29 | 0 | 0 |  | 0 | 0 | 0 | N | 67903 | Metalloproteinase type III 4e [Crotalus adamanteus] |
| 115 | 1158 | JAI10381.1 | 49.29 | 0 | 0 |  | 0 | 0 | 0 | N | 67873 | Metalloproteinase (type III) 4d [Crotalus adamanteus] |
| 115 | 1159 | JAS04679.1 | 49.29 | 0 | 0 |  | 0 | 0 | 0 | N | 67997 | Metalloproteinase type III 4b [Crotalus adamanteus] |
| 115 | 1160 | AFJ49234.1 | 49.29 | 0 | 0 |  | 0 | 0 | 0 | N | 67997 | Snake venom metalloproteinase (type III) 2b [Crotalus adamanteus] |
| 115 | 1161 | AEJ31989.1 | 49.29 | 0 | 0 |  | 0 | 0 | 0 | N | 67873 | metalloproteinase 6 [Crotalus adamanteus] |
| 115 | 1162 | AEJ31987.1 | 49.29 | 0 | 0 |  | 0 | 0 | 0 | N | 68018 | metalloproteinase 4 [Crotalus adamanteus] |
| 115 | 1163 | JAS04677.1 | 49.29 | 0 | 0 |  | 0 | 0 | 0 | N | 67873 | Metalloproteinase type III 4d [Crotalus adamanteus] |
| 125 | 905 | XP\_015667429.1 | 49.18 | 0 | 0 |  | 0 | 0 | 0 | N | 82600 | ATP-dependent 6-phosphofructokinase, platelet type isoform X3 [Protobothrops mucrosquamatus] |
| 125 | 906 | XP\_015667428.1 | 49.18 | 0 | 0 |  | 0 | 0 | 0 | N | 82563 | ATP-dependent 6-phosphofructokinase, platelet type isoform X2 [Protobothrops mucrosquamatus] |
| 125 | 907 | XP\_015667426.1 | 49.18 | 0 | 0 |  | 0 | 0 | 0 | N | 82821 | ATP-dependent 6-phosphofructokinase, platelet type isoform X1 [Protobothrops mucrosquamatus] |
| 125 | 908 | JAI14540.1 | 49.18 | 0 | 0 |  | 0 | 0 | 0 | N | 85324 | 6-phosphofructokinase type C-like [Crotalus adamanteus] |
| 120 | 775 | AFJ49243.1 | 47.34 | 0 | 0 |  | 0 | 0 | 0 | N | 68434 | Snake venom metalloproteinase (type III) 5 [Crotalus adamanteus] |
| 120 | 776 | JAV48412.1 | 47.34 | 0 | 0 |  | 0 | 0 | 0 | N | 68421 | Cadam10\_SVMPIII-2 [Crotalus adamanteus] |
| 120 | 1166 | AEJ31991.1 | 47.34 | 0 | 0 |  | 0 | 0 | 0 | N | 54615 | metalloproteinase 8, partial [Crotalus adamanteus] |
| 120 | 1167 | QIV64930.1 | 47.34 | 0 | 0 |  | 0 | 0 | 0 | N | 68321 | MDC-3a [Crotalus atrox] |
| 93 | 952 | Q7T1K6.1 | 44.67 | 0 | 0 |  | 0 | 0 | 0 | N | 26882 | RecName: Full=Cysteine-rich venom protein natrin-1; AltName: Full=Cysteine-rich venom protein 1; AltName: Full=NA-CRVP1; AltName: Full=Protein G2a; Flags: Precursor |
| 93 | 953 | ACH73167.1 | 44.67 | 0 | 0 |  | 0 | 0 | 0 | N | 26846 | kaouthin-1 precursor [Naja kaouthia] |
| 93 | 954 | P84805.2 | 44.67 | 0 | 0 |  | 0 | 0 | 0 | N | 26846 | RecName: Full=Cysteine-rich venom protein kaouthin-1; AltName: Full=Cysteine-rich venom protein 25; Short=CRVP-25k; Flags: Precursor |
| 93 | 955 | AAP85301.1 | 44.67 | 0 | 0 |  | 0 | 0 | 0 | N | 26882 | natrin [Naja atra] |
| 93 | 1171 | 1XX5 | 44.67 | 0 | 0 |  | 0 | 0 | 0 | N | 24954 | Chain C, Natrin 1 |
| 93 | 1172 | 2GIZ | 44.67 | 0 | 0 |  | 0 | 0 | 0 | N | 24954 | Chain B, Structural And Functional Analysis Of Natrin, A Member Of Crisp-3 Family Blocks A Variety Of Ion Channels |
| 93 | 1173 | 3MZ8 | 44.67 | 0 | 0 |  | 0 | 0 | 0 | N | 24954 | Chain B, Crystal Structure Of Zinc-Bound Natrin From Naja Atra |
| 131 | 1174 | XP\_026556535.1 | 44.44 | 0 | 0 |  | 0 | 0 | 0 | N | 33288 | tRNA (cytosine(34)-C(5))-methyltransferase, mitochondrial isoform X3 [Pseudonaja textilis] |
| 132 | 1175 | XP\_026545733.1 | 43.32 | 0 | 0 |  | 0 | 0 | 0 | N | 148628 | regulating synaptic membrane exocytosis protein 2 isoform X11 [Notechis scutatus] |
| 132 | 1176 | XP\_026559309.1 | 43.32 | 0 | 0 |  | 0 | 0 | 0 | N | 179008 | regulating synaptic membrane exocytosis protein 2 isoform X2 [Pseudonaja textilis] |
| 132 | 1177 | XP\_034278049.1 | 43.32 | 0 | 0 |  | 0 | 0 | 0 | N | 181355 | regulating synaptic membrane exocytosis protein 2 isoform X6 [Pantherophis guttatus] |
| 133 | 1179 | XP\_013911472.1 | 42.81 | 0 | 0 |  | 0 | 0 | 0 | N | 74789 | PREDICTED: ubiquitin carboxyl-terminal hydrolase CYLD [Thamnophis sirtalis] |
| 121 | 1187 | P60311.1 | 41.77 | 0 | 0 |  | 0 | 0 | 0 | N | 6753 | RecName: Full=Cytotoxin KJC3 |
| 121 | 1188 | P0CH80.1 | 41.77 | 0 | 0 |  | 0 | 0 | 0 | N | 6807 | RecName: Full=Cytotoxin 1; Short=NK-CT1 |
| 121 | 1189 | AAC61318.1 | 41.77 | 0 | 0 |  | 0 | 0 | 0 | N | 7029 | cardiotoxin 6 precursor, partial [Naja sputatrix] |
| 121 | 1190 | O73858.1 | 41.77 | 0 | 0 |  | 0 | 0 | 0 | N | 7029 | RecName: Full=Cytotoxin 6; AltName: Full=Cardiotoxin-6; Short=CTX-6; Short=Ctx6; Flags: Precursor |
| 121 | 1191 | AAA49386.1 | 41.77 | 0 | 0 |  | 0 | 0 | 0 | N | 9098 | cardiotoxin [Naja sputatrix] |
| 83 | 1194 | P86382.1 | 39.29 | 0 | 0 |  | 0 | 0 | 0 | N | 6792 | RecName: Full=Cytotoxin 7; Short=CTX7 |
| 83 | 1195 | XP\_026521338.1 | 39.29 | 0 | 0 |  | 0 | 0 | 0 | N | 45014 | indian hedgehog protein [Notechis scutatus] |
| 83 | 1196 | XP\_026563870.1 | 39.29 | 0 | 0 |  | 0 | 0 | 0 | N | 45028 | indian hedgehog protein [Pseudonaja textilis] |
| 83 | 1197 | XP\_013918540.1 | 39.29 | 0 | 0 |  | 0 | 0 | 0 | N | 45093 | PREDICTED: indian hedgehog protein [Thamnophis sirtalis] |
| 83 | 1198 | XP\_015675982.1 | 39.29 | 0 | 0 |  | 0 | 0 | 0 | N | 45120 | indian hedgehog protein [Protobothrops mucrosquamatus] |
| 83 | 1199 | XP\_034289619.1 | 39.29 | 0 | 0 |  | 0 | 0 | 0 | N | 44990 | indian hedgehog protein [Pantherophis guttatus] |
| 83 | 1200 | XP\_032073432.1 | 39.29 | 0 | 0 |  | 0 | 0 | 0 | N | 45098 | indian hedgehog protein [Thamnophis elegans] |
| 83 | 1204 | P86539.1 | 39.29 | 0 | 0 |  | 0 | 0 | 0 | N | 3377 | RecName: Full=Cytotoxin 3a; Short=CTX3a |
| 83 | 1205 | 6RC7 | 39.29 | 0 | 0 |  | 0 | 0 | 0 | N | 6717 | Chain A, Cytotoxin 3 |
| 83 | 1206 | P01445.1 | 39.29 | 0 | 0 |  | 0 | 0 | 0 | N | 6745 | RecName: Full=Cytotoxin 2; Short=CX2; AltName: Full=Toxin CM-7A |
| 83 | 1207 | 1CDT | 39.29 | 0 | 0 |  | 0 | 0 | 0 | N | 6715 | Chain B, CARDIOTOXIN VII4 |
| 83 | 1208 | P01446.1 | 39.29 | 0 | 0 |  | 0 | 0 | 0 | N | 6717 | RecName: Full=Cytotoxin 3; Short=CX3; AltName: Full=Toxin CM-7 |
| 83 | 1209 | P24780.1 | 39.29 | 0 | 0 |  | 0 | 0 | 0 | N | 6745 | RecName: Full=Cytotoxin 3; AltName: Full=Cytotoxin IIa |
| 83 | 1210 | P01452.1 | 39.29 | 0 | 0 |  | 0 | 0 | 0 | N | 6715 | RecName: Full=Cytotoxin 4; AltName: Full=CTX M3; AltName: Full=Cardiotoxin V(II)4 |
| 83 | 1211 | P01447.1 | 39.29 | 0 | 0 |  | 0 | 0 | 0 | N | 6791 | RecName: Full=Cytotoxin 1; AltName: Full=Cobramine-A; AltName: Full=Cytotoxin I; AltName: Full=Cytotoxin XI; AltName: Full=Cytotoxin-like basic protein; Short=CLBP |
| 83 | 1212 | P24779.1 | 39.29 | 0 | 0 |  | 0 | 0 | 0 | N | 6654 | RecName: Full=Cytotoxin 5; AltName: Full=Cytotoxin II |
| 83 | 1213 | P86540.2 | 39.29 | 0 | 0 |  | 0 | 0 | 0 | N | 6793 | RecName: Full=Cytotoxin 8; Short=CTX8 |
| 83 | 1214 | AAB35381.1 | 39.29 | 0 | 0 |  | 0 | 0 | 0 | N | 6877 | cytotoxin-like basic protein, CLBP [Naja naja=Indian cobras, naja, venom, Peptide, 61 aa] |
| 83 | 1215 | BAU24669.1 | 39.29 | 0 | 0 |  | 0 | 0 | 0 | N | 7993 | cytotoxin 12, partial [Naja naja] |
| 83 | 1216 | BAU24676.1 | 39.29 | 0 | 0 |  | 0 | 0 | 0 | N | 7977 | cytotoxin 17, partial [Naja naja] |
| 83 | 1217 | BAU24671.1 | 39.29 | 0 | 0 |  | 0 | 0 | 0 | N | 7993 | cytotoxin 14, partial [Naja naja] |
| 83 | 1218 | BAU24670.1 | 39.29 | 0 | 0 |  | 0 | 0 | 0 | N | 7947 | cytotoxin 13, partial [Naja naja] |
| 83 | 1219 | BAU24673.1 | 39.29 | 0 | 0 |  | 0 | 0 | 0 | N | 7993 | cytotoxin 14, partial [Naja naja] |
| 83 | 1220 | BAU24667.1 | 39.29 | 0 | 0 |  | 0 | 0 | 0 | N | 7993 | cytotoxin 12, partial [Naja naja] |
| 83 | 1221 | BAU24668.1 | 39.29 | 0 | 0 |  | 0 | 0 | 0 | N | 7993 | cytotoxin 12, partial [Naja naja] |
| 83 | 1222 | BAU24672.1 | 39.29 | 0 | 0 |  | 0 | 0 | 0 | N | 7993 | cytotoxin 14, partial [Naja naja] |
| 83 | 1223 | BAU24675.1 | 39.29 | 0 | 0 |  | 0 | 0 | 0 | N | 7967 | cytotoxin 16, partial [Naja naja] |
| 83 | 1228 | XP\_007419884.1 | 39.29 | 0 | 0 |  | 0 | 0 | 0 | N | 45052 | indian hedgehog protein [Python bivittatus] |
| 134 | 1225 | XP\_025020898.1 | 38.79 | 0 | 0 |  | 0 | 0 | 0 | N | 265188 | golgin subfamily A member 4 isoform X3 [Python bivittatus] |
| 134 | 1226 | XP\_025020897.1 | 38.79 | 0 | 0 |  | 0 | 0 | 0 | N | 268628 | golgin subfamily A member 4 isoform X2 [Python bivittatus] |
| 134 | 1227 | XP\_025020896.1 | 38.79 | 0 | 0 |  | 0 | 0 | 0 | N | 268793 | golgin subfamily A member 4 isoform X1 [Python bivittatus] |
| 108 | 633 | XP\_013925438.1 | 37.88 | 0 | 0 |  | 0 | 0 | 0 | N | 292467 | PREDICTED: zinc finger protein 40 isoform X2 [Thamnophis sirtalis] |
| 108 | 634 | XP\_013925437.1 | 37.88 | 0 | 0 |  | 0 | 0 | 0 | N | 292596 | PREDICTED: zinc finger protein 40 isoform X1 [Thamnophis sirtalis] |
| 108 | 639 | XP\_032078666.1 | 37.88 | 0 | 0 |  | 0 | 0 | 0 | N | 292439 | zinc finger protein 40 isoform X2 [Thamnophis elegans] |
| 108 | 640 | XP\_032078662.1 | 37.88 | 0 | 0 |  | 0 | 0 | 0 | N | 292567 | zinc finger protein 40 isoform X1 [Thamnophis elegans] |
| 108 | 641 | XP\_032078664.1 | 37.88 | 0 | 0 |  | 0 | 0 | 0 | N | 292567 | zinc finger protein 40 isoform X1 [Thamnophis elegans] |
| 108 | 642 | XP\_032078665.1 | 37.88 | 0 | 0 |  | 0 | 0 | 0 | N | 292567 | zinc finger protein 40 isoform X1 [Thamnophis elegans] |
| 108 | 643 | XP\_032078663.1 | 37.88 | 0 | 0 |  | 0 | 0 | 0 | N | 292567 | zinc finger protein 40 isoform X1 [Thamnophis elegans] |
| 106 | 704 | XP\_026551948.1 | 36.44 | 0 | 0 |  | 0 | 0 | 0 | N | 252735 | LOW QUALITY PROTEIN: uncharacterized protein LOC113433547 [Pseudonaja textilis] |
| 106 | 740 | XP\_026541026.1 | 36.44 | 0 | 0 |  | 0 | 0 | 0 | N | 228018 | uncharacterized protein LOC113423713 [Notechis scutatus] |
| 106 | 1240 | ETE67277.1 | 36.44 | 0 | 0 |  | 0 | 0 | 0 | N | 74913 | hypothetical protein L345\_06937, partial [Ophiophagus hannah] |
| 104 | 689 | XP\_026535088.1 | 36.41 | 0 | 0 |  | 0 | 0 | 0 | N | 277959 | glutamate-rich protein 3 [Notechis scutatus] |
| 104 | 1168 | ETE71292.1 | 36.41 | 0 | 0 |  | 0 | 0 | 0 | N | 268452 | hypothetical protein L345\_02895, partial [Ophiophagus hannah] |
| 104 | 1170 | XP\_034292893.1 | 36.41 | 0 | 0 |  | 0 | 0 | 0 | N | 256438 | glutamate-rich protein 3 isoform X2 [Pantherophis guttatus] |
| 123 | 1241 | ETE72045.1 | 36.14 | 0 | 0 |  | 0 | 0 | 0 | N | 127525 | Peptidyl-glycine alpha-amidating monooxygenase, partial [Ophiophagus hannah] |
| total 500 proteins |  |  |  |  |  |  |  |  |  |  |  |  |
| --- | --- | --- | --- | --- | --- | --- | --- | --- | --- | --- | --- | --- |

  

T\_174

back to list

  

| Protein Coverage
| Supporting Peptides
|

Protein Coverage:

Supporting Peptides:

| Peptide | Uniq | -10lgP | Mass | Length | ppm | m/z | z | RT | Fraction | Scan | Source File | Area BuCaMH04\_B15 | #Feature | #Feature BuCaMH04\_B15 | Start | End | PTM | AScore | Found By |
| --- | --- | --- | --- | --- | --- | --- | --- | --- | --- | --- | --- | --- | --- | --- | --- | --- | --- | --- | --- |
| R.FLTYTQNVILVSLSYR.V | N | 114.43 | 1916.0408 | 16 | -0.1 | 959.0276 | 2 | 48.85 | 1 | 34834 | BuCaMH04.raw | 2.6107E7 | 2 | 2 | 165 | 180 |  |  | PEAKS DB |
| R.LALQWIQNNIHPFGGNPR.A | Y | 112.54 | 2074.0859 | 18 | 0.7 | 692.3698 | 3 | 35.35 | 1 | 22532 | BuCaMH04.raw | 3.2934E8 | 2 | 2 | 206 | 223 |  |  | PEAKS DB |
| K.NPQELIDEEWSVLPYK.S | Y | 110.40 | 1958.9625 | 16 | 0.7 | 980.4893 | 2 | 45.40 | 1 | 31557 | BuCaMH04.raw | 9.7311E7 | 1 | 1 | 301 | 316 |  |  | PEAKS DB |
| R.GLSLPVLDGHVTAFLGIPFAEPPVGR.M | Y | 109.13 | 2658.4534 | 26 | 1.0 | 887.1593 | 3 | 56.75 | 1 | 41697 | BuCaMH04.raw | 5.9935E8 | 4 | 4 | 48 | 73 |  |  | PEAKS DB |
| R.AILQSGGPNAPWATVTPAESR.R | N | 106.82 | 2122.0806 | 21 | 0.3 | 1062.0479 | 2 | 26.94 | 1 | 15006 | BuCaMH04.raw | 8.0499E8 | 2 | 2 | 253 | 273 |  |  | PEAKS DB |
| K.DEGSYFLIYGLPGFSK.D | N | 103.69 | 1791.8718 | 16 | 0.7 | 896.9438 | 2 | 56.99 | 1 | 42168 | BuCaMH04.raw | 1.8962E8 | 3 | 3 | 357 | 372 |  |  | PEAKS DB |
| R.SKNPQELIDEEWSVLPYK.S | Y | 101.78 | 2174.0896 | 18 | 0.2 | 1088.0522 | 2 | 37.46 | 1 | 24710 | BuCaMH04.raw | 7.3568E8 | 3 | 3 | 299 | 316 |  |  | PEAKS DB |
| R.AVTIFGESAGAASVGMHLLSTQSR.T | N | 101.28 | 2389.2061 | 24 | 1.2 | 797.4103 | 3 | 39.94 | 1 | 26891 | BuCaMH04.raw | 1.4086E8 | 1 | 1 | 224 | 247 |  |  | PEAKS DB |
| K.SGAWPTYTASQPQYVQLNTQPLATQPSLR.A | Y | 96.99 | 3202.6047 | 29 | 0.4 | 1068.5426 | 3 | 35.82 | 1 | 23244 | BuCaMH04.raw | 5.4891E8 | 2 | 2 | 520 | 548 |  |  | PEAKS DB |
| K.NREALDDIVGDHNVIC(+57.02)PVVQFANDYAK.R | Y | 90.61 | 3071.4771 | 27 | 0.2 | 768.8767 | 4 | 37.65 | 1 | 24807 | BuCaMH04.raw | 3.7376E7 | 1 | 1 | 418 | 444 | Carbamidomethylation | C16:Carbamidomethylation:1000.00 | PEAKS DB |
| E.LIDEEWSVLPYK.S | N | 89.30 | 1490.7656 | 12 | 0.9 | 746.3907 | 2 | 37.26 | 1 | 24455 | BuCaMH04.raw | 2.1123E7 | 1 | 1 | 305 | 316 |  |  | PEAKS DB |
| W.IYGGGFYSGAASLDVYDGR.F | N | 88.75 | 1966.9060 | 19 | 0.5 | 984.4608 | 2 | 34.51 | 1 | 21942 | BuCaMH04.raw | 6.0601E6 | 1 | 1 | 146 | 164 |  |  | PEAKS DB |
| R.MSVPHANDIATEAVVLQYTDWQDQDNR.E | Y | 86.67 | 3115.4304 | 27 | 0.8 | 1039.4849 | 3 | 40.24 | 1 | 27065 | BuCaMH04.raw | 1.1008E8 | 2 | 2 | 389 | 415 |  |  | PEAKS DB |
| R.FPFVPVIDGDFFPDTPEAMLSSGNFK.E | Y | 86.44 | 2873.3621 | 26 | 1.0 | 958.7956 | 3 | 65.46 | 1 | 49106 | BuCaMH04.raw | 8.2363E7 | 2 | 2 | 321 | 346 |  |  | PEAKS DB |
| R.EALDDIVGDHNVIC(+57.02)PVVQFANDYAK.R | Y | 84.11 | 2801.3330 | 25 | 0.7 | 934.7856 | 3 | 43.95 | 1 | 30231 | BuCaMH04.raw | 3.7478E8 | 3 | 3 | 420 | 444 | Carbamidomethylation | C14:Carbamidomethylation:1000.00 | PEAKS DB |
| N.PQELIDEEWSVLPYK.S | N | 79.86 | 1844.9196 | 15 | 0.6 | 923.4677 | 2 | 41.67 | 1 | 28438 | BuCaMH04.raw | 7.64E6 | 1 | 1 | 302 | 316 |  |  | PEAKS DB |
| R.VGAFGFLGLPGSPEAPGNMGLLDQR.L | Y | 78.95 | 2499.2581 | 25 | 0.4 | 834.0936 | 3 | 53.05 | 1 | 38405 | BuCaMH04.raw | 1.3053E7 | 1 | 1 | 181 | 205 |  |  | PEAKS DB |
| R.LALQWIQNNIHPF.G | N | 74.47 | 1592.8463 | 13 | 1.1 | 797.4313 | 2 | 47.45 | 1 | 33715 | BuCaMH04.raw | 1.0832E7 | 1 | 1 | 206 | 218 |  |  | PEAKS DB |
| R.LALQWIQNNIHP.F | N | 72.94 | 1445.7780 | 12 | 0.3 | 723.8965 | 2 | 38.64 | 1 | 25651 | BuCaMH04.raw | 1.8096E7 | 1 | 1 | 206 | 217 |  |  | PEAKS DB |
| L.DDIVGDHNVIC(+57.02)PVVQFANDYAK.R | N | 72.30 | 2488.1692 | 22 | 0.4 | 830.3973 | 3 | 39.09 | 1 | 25991 | BuCaMH04.raw | 1.3183E7 | 1 | 1 | 423 | 444 | Carbamidomethylation | C11:Carbamidomethylation:1000.00 | PEAKS DB |
| R.GLSLPVLDGHVTAF.L | Y | 71.71 | 1424.7664 | 14 | 0.4 | 713.3907 | 2 | 44.38 | 1 | 30914 | BuCaMH04.raw | 5.2039E7 | 1 | 1 | 48 | 61 |  |  | PEAKS DB |
| A.FLGIPFAEPPVGR.M | N | 70.62 | 1398.7659 | 13 | 0.9 | 700.3909 | 2 | 42.12 | 1 | 28605 | BuCaMH04.raw | 1.1387E8 | 1 | 1 | 61 | 73 |  |  | PEAKS DB |
| L.ALQWIQNNIHPFGGNPR.A | Y | 70.18 | 1961.0020 | 17 | 0.6 | 654.6750 | 3 | 26.16 | 1 | 14209 | BuCaMH04.raw | 8.6423E7 | 1 | 1 | 207 | 223 |  |  | PEAKS DB |
| R.GLSLPVLDGHVTA.F | Y | 69.50 | 1277.6979 | 13 | 1.2 | 639.8570 | 2 | 33.81 | 1 | 21445 | BuCaMH04.raw | 1.0577E8 | 1 | 1 | 48 | 60 |  |  | PEAKS DB |
| I.C(+57.02)AFWNHFLPK.L | N | 67.68 | 1318.6281 | 10 | 0.2 | 440.5500 | 3 | 27.24 | 1 | 15136 | BuCaMH04.raw | 4.6147E6 | 1 | 1 | 552 | 561 | Carbamidomethylation | C1:Carbamidomethylation:1000.00 | PEAKS DB |
| R.M(+15.99)SVPHANDIATEAVVLQYTDWQDQDNR.E | Y | 67.45 | 3131.4253 | 27 | -1.0 | 1044.8147 | 3 | 36.49 | 1 | 23816 | BuCaMH04.raw | 1.4623E7 | 1 | 1 | 389 | 415 | Oxidation (M) | M1:Oxidation (M):1000.00 | PEAKS DB |
| H.VTAFLGIPFAEPPVGR.M | Y | 64.75 | 1669.9191 | 16 | -0.1 | 835.9667 | 2 | 46.50 | 1 | 32659 | BuCaMH04.raw | 2.3079E6 | 1 | 1 | 58 | 73 |  |  | PEAKS DB |
| R.LALQWIQNNIHPFGGN.P | Y | 63.37 | 1820.9321 | 16 | 0.3 | 911.4736 | 2 | 44.66 | 1 | 31114 | BuCaMH04.raw | 3.7651E6 | 1 | 1 | 206 | 221 |  |  | PEAKS DB |
| A.LQWIQNNIHPFGGNPR.A | Y | 62.30 | 1889.9648 | 16 | -0.4 | 945.9893 | 2 | 24.00 | 1 | 12358 | BuCaMH04.raw | 6.7762E5 | 1 | 1 | 208 | 223 |  |  | PEAKS DB |
| F.LGIPFAEPPVGR.M | N | 62.25 | 1251.6975 | 12 | 0.5 | 626.8563 | 2 | 29.35 | 1 | 17318 | BuCaMH04.raw | 6.6683E7 | 1 | 1 | 62 | 73 |  |  | PEAKS DB |
| R.ASNLLWPPWMGVPH.G | N | 61.66 | 1603.7969 | 14 | 1.3 | 802.9067 | 2 | 53.12 | 1 | 38555 | BuCaMH04.raw | 1.3595E6 | 1 | 1 | 458 | 471 |  |  | PEAKS DB |
| K.QLGC(+57.02)HFNNDSELVSC(+57.02)LR.S | N | 61.24 | 2047.9204 | 17 | 1.0 | 683.6481 | 3 | 20.07 | 1 | 8865 | BuCaMH04.raw | 2.0907E7 | 1 | 1 | 282 | 298 | Carbamidomethylation | C4:Carbamidomethylation:1000.00;C15:Carbamidomethylation:1000.00 | PEAKS DB |
| R.AQIC(+57.02)AFWNHFLPK.L | N | 60.48 | 1630.8079 | 13 | 1.0 | 544.6104 | 3 | 36.94 | 1 | 23938 | BuCaMH04.raw | 1.8602E6 | 1 | 1 | 549 | 561 | Carbamidomethylation | C4:Carbamidomethylation:1000.00 | PEAKS DB |
| T.AFLGIPFAEPPVGR.M | N | 58.55 | 1469.8030 | 14 | 0.6 | 735.9092 | 2 | 43.95 | 1 | 30470 | BuCaMH04.raw | 5.0781E6 | 1 | 1 | 60 | 73 |  |  | PEAKS DB |
| N.DIATEAVVLQYTDWQDQDNR.E | Y | 58.52 | 2379.0979 | 20 | 0.3 | 1190.5566 | 2 | 46.51 | 1 | 32799 | BuCaMH04.raw | 2.4161E6 | 1 | 1 | 396 | 415 |  |  | PEAKS DB |
| L.IDEEWSVLPYK.S | N | 57.66 | 1377.6816 | 11 | 0.6 | 689.8485 | 2 | 33.06 | 1 | 20610 | BuCaMH04.raw | 2.6958E6 | 1 | 1 | 306 | 316 |  |  | PEAKS DB |
| A.ILQSGGPNAPWATVTPAESR.R | N | 56.43 | 2051.0435 | 20 | 2.0 | 1026.5310 | 2 | 24.16 | 1 | 12452 | BuCaMH04.raw | 1.9229E6 | 1 | 1 | 254 | 273 |  |  | PEAKS DB |
| T.IFGESAGAASVGMHLLSTQSR.T | N | 54.19 | 2118.0527 | 21 | 0.7 | 707.0253 | 3 | 27.10 | 1 | 15139 | BuCaMH04.raw | 7.8883E6 | 1 | 1 | 227 | 247 |  |  | PEAKS DB |
| Q.ELIDEEWSVLPYK.S | N | 51.47 | 1619.8082 | 13 | 0.7 | 810.9119 | 2 | 42.09 | 1 | 28660 | BuCaMH04.raw | 4.6799E5 | 1 | 1 | 304 | 316 |  |  | PEAKS DB |
| L.IYGLPGFSK.D | N | 51.29 | 980.5331 | 9 | 0.8 | 491.2742 | 2 | 27.30 | 1 | 15385 | BuCaMH04.raw | 4.7137E6 | 1 | 1 | 364 | 372 |  |  | PEAKS DB |
| A.VVLQYTDWQDQDNR.E | Y | 50.68 | 1778.8224 | 14 | 2.0 | 890.4203 | 2 | 23.84 | 1 | 12193 | BuCaMH04.raw | 1.8214E6 | 1 | 1 | 402 | 415 |  |  | PEAKS DB |
| total 41 peptides |  |  |  |  |  |  |  |  |  |  |  |  |  |  |  |  |  |  |  |
| --- | --- | --- | --- | --- | --- | --- | --- | --- | --- | --- | --- | --- | --- | --- | --- | --- | --- | --- | --- |

6I2X

back to list

  

| Protein Coverage
| Supporting Peptides
|

Protein Coverage:

Supporting Peptides:

| Peptide | Uniq | -10lgP | Mass | Length | ppm | m/z | z | RT | Fraction | Scan | Source File | Area BuCaMH04\_B15 | #Feature | #Feature BuCaMH04\_B15 | Start | End | PTM | AScore | Found By |
| --- | --- | --- | --- | --- | --- | --- | --- | --- | --- | --- | --- | --- | --- | --- | --- | --- | --- | --- | --- |
| R.IEEQDGNDIYVMDVLEVIK.Q | Y | 127.97 | 2221.0823 | 19 | -0.2 | 1111.5482 | 2 | 58.14 | 1 | 42915 | BuCaMH04.raw | 1.5532E7 | 2 | 2 | 1531 | 1549 |  |  | PEAKS DB |
| R.YLYGEEVEGVAFVLFGVK.I | N | 101.23 | 2018.0400 | 18 | 0.2 | 1010.0275 | 2 | 64.43 | 1 | 48252 | BuCaMH04.raw | 7.4395E6 | 2 | 2 | 251 | 268 |  |  | PEAKS DB |
| R.AVPFVIVPLEQGLHDVEIK.A | Y | 101.16 | 2102.1775 | 19 | 0.6 | 701.7335 | 3 | 44.28 | 1 | 30806 | BuCaMH04.raw | 7.6124E7 | 2 | 2 | 879 | 897 |  |  | PEAKS DB |
| K.VNDDYLIWGSR.S | Y | 99.55 | 1336.6411 | 11 | 0.6 | 669.3282 | 2 | 31.12 | 1 | 18953 | BuCaMH04.raw | 5.2426E7 | 1 | 1 | 1577 | 1587 |  |  | PEAKS DB |
| E.VEGVAFVLFGVK.I | N | 94.83 | 1263.7227 | 12 | 0.3 | 632.8688 | 2 | 46.03 | 1 | 32338 | BuCaMH04.raw | 2.7212E6 | 1 | 1 | 257 | 268 |  |  | PEAKS DB |
| K.FFYIDGNENFHVSITAR.Y | Y | 86.65 | 2028.9694 | 17 | 0.1 | 677.3304 | 3 | 33.21 | 1 | 20689 | BuCaMH04.raw | 6.862E6 | 1 | 1 | 234 | 250 |  |  | PEAKS DB |
| K.DLNLDITIELPDR.E | Y | 81.02 | 1525.7987 | 13 | 0.3 | 763.9069 | 2 | 45.23 | 1 | 31751 | BuCaMH04.raw | 2.5874E6 | 2 | 2 | 1272 | 1284 |  |  | PEAKS DB |
| R.INYENALLAR.T | Y | 80.85 | 1175.6299 | 10 | 1.4 | 588.8231 | 2 | 22.36 | 1 | 10916 | BuCaMH04.raw | 1.4785E7 | 1 | 1 | 1292 | 1301 |  |  | PEAKS DB |
| R.QNQYVVVQVTGPQVR.L | N | 77.22 | 1713.9163 | 15 | 0.6 | 572.3130 | 3 | 19.26 | 1 | 8230 | BuCaMH04.raw | 2.1525E7 | 2 | 2 | 100 | 114 |  |  | PEAKS DB |
| G.ALYTLITPAVLR.T | N | 77.10 | 1329.8020 | 12 | 0.6 | 665.9087 | 2 | 39.11 | 1 | 25893 | BuCaMH04.raw | 3.4301E7 | 1 | 1 | 23 | 34 |  |  | PEAKS DB |
| K.YFTYLILNK.G | N | 75.76 | 1173.6433 | 9 | 0.9 | 587.8295 | 2 | 33.34 | 1 | 20777 | BuCaMH04.raw | 1.8251E7 | 1 | 1 | 478 | 486 |  |  | PEAKS DB |
| R.VELLYNPAFC(+57.02)SASTK.G | Y | 75.53 | 1698.8286 | 15 | 0.8 | 850.4223 | 2 | 27.80 | 1 | 15715 | BuCaMH04.raw | 3.809E7 | 1 | 1 | 848 | 862 | Carbamidomethylation | C10:Carbamidomethylation:1000.00 | PEAKS DB |
| R.DDNEDGFIADSDIISR.S | Y | 74.02 | 1780.7751 | 16 | 1.2 | 891.3959 | 2 | 32.87 | 1 | 20586 | BuCaMH04.raw | 1.1975E7 | 1 | 1 | 733 | 748 |  |  | PEAKS DB |
| K.LILNIPLNAQSLPITVR.T | Y | 71.01 | 1874.1353 | 17 | 0.8 | 625.7195 | 3 | 45.50 | 1 | 31737 | BuCaMH04.raw | 1.3854E7 | 2 | 2 | 398 | 414 |  |  | PEAKS DB |
| H.GALYTLITPAVLR.T | N | 67.01 | 1386.8235 | 13 | 0.6 | 694.4194 | 2 | 39.60 | 1 | 26478 | BuCaMH04.raw | 7.2514E6 | 1 | 1 | 22 | 34 |  |  | PEAKS DB |
| K.ASVQEALWSDGVR.K | Y | 66.69 | 1416.6997 | 13 | 0.7 | 709.3576 | 2 | 24.61 | 1 | 12813 | BuCaMH04.raw | 1.6033E7 | 1 | 1 | 898 | 910 |  |  | PEAKS DB |
| K.YVLPSFEVR.L | N | 63.14 | 1108.5917 | 9 | 0.7 | 555.3035 | 2 | 28.54 | 1 | 16534 | BuCaMH04.raw | 1.3942E7 | 1 | 1 | 219 | 227 |  |  | PEAKS DB |
| K.IIIQGDPVAQIIEN.S | Y | 60.89 | 1521.8402 | 14 | 0.2 | 761.9276 | 2 | 42.93 | 1 | 29516 | BuCaMH04.raw | 2.2646E7 | 1 | 1 | 963 | 976 |  |  | PEAKS DB |
| K.ATMTILTFYNAQLQEK.A | Y | 60.55 | 1870.9498 | 16 | 1.2 | 936.4833 | 2 | 41.38 | 1 | 27984 | BuCaMH04.raw | 7.9697E6 | 1 | 1 | 1321 | 1336 |  |  | PEAKS DB |
| K.IIIQGDPVAQIIENSIDGSK.L | Y | 54.34 | 2109.1316 | 20 | 1.3 | 1055.5745 | 2 | 52.79 | 1 | 38314 | BuCaMH04.raw | 8.2472E5 | 1 | 1 | 963 | 982 |  |  | PEAKS DB |
| K.GIYTPGSPVLYR.V | N | 52.60 | 1321.7030 | 12 | 0.4 | 661.8590 | 2 | 22.35 | 1 | 11114 | BuCaMH04.raw | 2.9354E7 | 1 | 1 | 135 | 146 |  |  | PEAKS DB |
| R.VELLYNPAFC(+57.02)SASTKG.Q | Y | 52.38 | 1755.8501 | 16 | 0.6 | 878.9329 | 2 | 27.53 | 1 | 15433 | BuCaMH04.raw | 1.6571E6 | 1 | 1 | 848 | 863 | Carbamidomethylation | C10:Carbamidomethylation:1000.00 | PEAKS DB |
| R.DSITTWVVLAVSFTPTK.G | Y | 51.87 | 1863.9982 | 17 | 0.7 | 933.0070 | 2 | 67.31 | 1 | 50500 | BuCaMH04.raw | 3.3834E5 | 1 | 1 | 782 | 798 |  |  | PEAKS DB |
| total 23 peptides |  |  |  |  |  |  |  |  |  |  |  |  |  |  |  |  |  |  |  |
| --- | --- | --- | --- | --- | --- | --- | --- | --- | --- | --- | --- | --- | --- | --- | --- | --- | --- | --- | --- |

3PRX

back to list

  

| Protein Coverage
| Supporting Peptides
|

Protein Coverage:

Supporting Peptides:

| Peptide | Uniq | -10lgP | Mass | Length | ppm | m/z | z | RT | Fraction | Scan | Source File | Area BuCaMH04\_B15 | #Feature | #Feature BuCaMH04\_B15 | Start | End | PTM | AScore | Found By |
| --- | --- | --- | --- | --- | --- | --- | --- | --- | --- | --- | --- | --- | --- | --- | --- | --- | --- | --- | --- |
| R.IEEQDGNDIYVMDVLEVIK.Q | Y | 127.97 | 2221.0823 | 19 | -0.2 | 1111.5482 | 2 | 58.14 | 1 | 42915 | BuCaMH04.raw | 1.5532E7 | 2 | 2 | 1531 | 1549 |  |  | PEAKS DB |
| R.YLYGEEVEGVAFVLFGVK.I | N | 101.23 | 2018.0400 | 18 | 0.2 | 1010.0275 | 2 | 64.43 | 1 | 48252 | BuCaMH04.raw | 7.4395E6 | 2 | 2 | 251 | 268 |  |  | PEAKS DB |
| R.AVPFVIVPLEQGLHDVEIK.A | Y | 101.16 | 2102.1775 | 19 | 0.6 | 701.7335 | 3 | 44.28 | 1 | 30806 | BuCaMH04.raw | 7.6124E7 | 2 | 2 | 879 | 897 |  |  | PEAKS DB |
| K.VNDDYLIWGSR.S | Y | 99.55 | 1336.6411 | 11 | 0.6 | 669.3282 | 2 | 31.12 | 1 | 18953 | BuCaMH04.raw | 5.2426E7 | 1 | 1 | 1577 | 1587 |  |  | PEAKS DB |
| E.VEGVAFVLFGVK.I | N | 94.83 | 1263.7227 | 12 | 0.3 | 632.8688 | 2 | 46.03 | 1 | 32338 | BuCaMH04.raw | 2.7212E6 | 1 | 1 | 257 | 268 |  |  | PEAKS DB |
| K.FFYIDGNENFHVSITAR.Y | Y | 86.65 | 2028.9694 | 17 | 0.1 | 677.3304 | 3 | 33.21 | 1 | 20689 | BuCaMH04.raw | 6.862E6 | 1 | 1 | 234 | 250 |  |  | PEAKS DB |
| K.DLNLDITIELPDR.E | Y | 81.02 | 1525.7987 | 13 | 0.3 | 763.9069 | 2 | 45.23 | 1 | 31751 | BuCaMH04.raw | 2.5874E6 | 2 | 2 | 1272 | 1284 |  |  | PEAKS DB |
| R.INYENALLAR.T | Y | 80.85 | 1175.6299 | 10 | 1.4 | 588.8231 | 2 | 22.36 | 1 | 10916 | BuCaMH04.raw | 1.4785E7 | 1 | 1 | 1292 | 1301 |  |  | PEAKS DB |
| R.QNQYVVVQVTGPQVR.L | N | 77.22 | 1713.9163 | 15 | 0.6 | 572.3130 | 3 | 19.26 | 1 | 8230 | BuCaMH04.raw | 2.1525E7 | 2 | 2 | 100 | 114 |  |  | PEAKS DB |
| G.ALYTLITPAVLR.T | N | 77.10 | 1329.8020 | 12 | 0.6 | 665.9087 | 2 | 39.11 | 1 | 25893 | BuCaMH04.raw | 3.4301E7 | 1 | 1 | 23 | 34 |  |  | PEAKS DB |
| K.YFTYLILNK.G | N | 75.76 | 1173.6433 | 9 | 0.9 | 587.8295 | 2 | 33.34 | 1 | 20777 | BuCaMH04.raw | 1.8251E7 | 1 | 1 | 478 | 486 |  |  | PEAKS DB |
| R.VELLYNPAFC(+57.02)SASTK.G | Y | 75.53 | 1698.8286 | 15 | 0.8 | 850.4223 | 2 | 27.80 | 1 | 15715 | BuCaMH04.raw | 3.809E7 | 1 | 1 | 848 | 862 | Carbamidomethylation | C10:Carbamidomethylation:1000.00 | PEAKS DB |
| R.DDNEDGFIADSDIISR.S | Y | 74.02 | 1780.7751 | 16 | 1.2 | 891.3959 | 2 | 32.87 | 1 | 20586 | BuCaMH04.raw | 1.1975E7 | 1 | 1 | 733 | 748 |  |  | PEAKS DB |
| K.LILNIPLNAQSLPITVR.T | Y | 71.01 | 1874.1353 | 17 | 0.8 | 625.7195 | 3 | 45.50 | 1 | 31737 | BuCaMH04.raw | 1.3854E7 | 2 | 2 | 398 | 414 |  |  | PEAKS DB |
| H.GALYTLITPAVLR.T | N | 67.01 | 1386.8235 | 13 | 0.6 | 694.4194 | 2 | 39.60 | 1 | 26478 | BuCaMH04.raw | 7.2514E6 | 1 | 1 | 22 | 34 |  |  | PEAKS DB |
| K.ASVQEALWSDGVR.K | Y | 66.69 | 1416.6997 | 13 | 0.7 | 709.3576 | 2 | 24.61 | 1 | 12813 | BuCaMH04.raw | 1.6033E7 | 1 | 1 | 898 | 910 |  |  | PEAKS DB |
| K.YVLPSFEVR.L | N | 63.14 | 1108.5917 | 9 | 0.7 | 555.3035 | 2 | 28.54 | 1 | 16534 | BuCaMH04.raw | 1.3942E7 | 1 | 1 | 219 | 227 |  |  | PEAKS DB |
| K.IIIQGDPVAQIIEN.S | Y | 60.89 | 1521.8402 | 14 | 0.2 | 761.9276 | 2 | 42.93 | 1 | 29516 | BuCaMH04.raw | 2.2646E7 | 1 | 1 | 963 | 976 |  |  | PEAKS DB |
| K.ATMTILTFYNAQLQEK.A | Y | 60.55 | 1870.9498 | 16 | 1.2 | 936.4833 | 2 | 41.38 | 1 | 27984 | BuCaMH04.raw | 7.9697E6 | 1 | 1 | 1321 | 1336 |  |  | PEAKS DB |
| K.IIIQGDPVAQIIENSIDGSK.L | Y | 54.34 | 2109.1316 | 20 | 1.3 | 1055.5745 | 2 | 52.79 | 1 | 38314 | BuCaMH04.raw | 8.2472E5 | 1 | 1 | 963 | 982 |  |  | PEAKS DB |
| K.GIYTPGSPVLYR.V | N | 52.60 | 1321.7030 | 12 | 0.4 | 661.8590 | 2 | 22.35 | 1 | 11114 | BuCaMH04.raw | 2.9354E7 | 1 | 1 | 135 | 146 |  |  | PEAKS DB |
| R.VELLYNPAFC(+57.02)SASTKG.Q | Y | 52.38 | 1755.8501 | 16 | 0.6 | 878.9329 | 2 | 27.53 | 1 | 15433 | BuCaMH04.raw | 1.6571E6 | 1 | 1 | 848 | 863 | Carbamidomethylation | C10:Carbamidomethylation:1000.00 | PEAKS DB |
| R.DSITTWVVLAVSFTPTK.G | Y | 51.87 | 1863.9982 | 17 | 0.7 | 933.0070 | 2 | 67.31 | 1 | 50500 | BuCaMH04.raw | 3.3834E5 | 1 | 1 | 782 | 798 |  |  | PEAKS DB |
| total 23 peptides |  |  |  |  |  |  |  |  |  |  |  |  |  |  |  |  |  |  |  |
| --- | --- | --- | --- | --- | --- | --- | --- | --- | --- | --- | --- | --- | --- | --- | --- | --- | --- | --- | --- |

I51018

back to list

  

| Protein Coverage
| Supporting Peptides
|

Protein Coverage:

Supporting Peptides:

| Peptide | Uniq | -10lgP | Mass | Length | ppm | m/z | z | RT | Fraction | Scan | Source File | Area BuCaMH04\_B15 | #Feature | #Feature BuCaMH04\_B15 | Start | End | PTM | AScore | Found By |
| --- | --- | --- | --- | --- | --- | --- | --- | --- | --- | --- | --- | --- | --- | --- | --- | --- | --- | --- | --- |
| R.IEEQDGNDIYVMDVLEVIK.Q | Y | 127.97 | 2221.0823 | 19 | -0.2 | 1111.5482 | 2 | 58.14 | 1 | 42915 | BuCaMH04.raw | 1.5532E7 | 2 | 2 | 1531 | 1549 |  |  | PEAKS DB |
| R.YLYGEEVEGVAFVLFGVK.I | N | 101.23 | 2018.0400 | 18 | 0.2 | 1010.0275 | 2 | 64.43 | 1 | 48252 | BuCaMH04.raw | 7.4395E6 | 2 | 2 | 251 | 268 |  |  | PEAKS DB |
| R.AVPFVIVPLEQGLHDVEIK.A | Y | 101.16 | 2102.1775 | 19 | 0.6 | 701.7335 | 3 | 44.28 | 1 | 30806 | BuCaMH04.raw | 7.6124E7 | 2 | 2 | 879 | 897 |  |  | PEAKS DB |
| K.VNDDYLIWGSR.S | Y | 99.55 | 1336.6411 | 11 | 0.6 | 669.3282 | 2 | 31.12 | 1 | 18953 | BuCaMH04.raw | 5.2426E7 | 1 | 1 | 1577 | 1587 |  |  | PEAKS DB |
| E.VEGVAFVLFGVK.I | N | 94.83 | 1263.7227 | 12 | 0.3 | 632.8688 | 2 | 46.03 | 1 | 32338 | BuCaMH04.raw | 2.7212E6 | 1 | 1 | 257 | 268 |  |  | PEAKS DB |
| K.FFYIDGNENFHVSITAR.Y | Y | 86.65 | 2028.9694 | 17 | 0.1 | 677.3304 | 3 | 33.21 | 1 | 20689 | BuCaMH04.raw | 6.862E6 | 1 | 1 | 234 | 250 |  |  | PEAKS DB |
| K.DLNLDITIELPDR.E | Y | 81.02 | 1525.7987 | 13 | 0.3 | 763.9069 | 2 | 45.23 | 1 | 31751 | BuCaMH04.raw | 2.5874E6 | 2 | 2 | 1272 | 1284 |  |  | PEAKS DB |
| R.INYENALLAR.T | Y | 80.85 | 1175.6299 | 10 | 1.4 | 588.8231 | 2 | 22.36 | 1 | 10916 | BuCaMH04.raw | 1.4785E7 | 1 | 1 | 1292 | 1301 |  |  | PEAKS DB |
| R.QNQYVVVQVTGPQVR.L | N | 77.22 | 1713.9163 | 15 | 0.6 | 572.3130 | 3 | 19.26 | 1 | 8230 | BuCaMH04.raw | 2.1525E7 | 2 | 2 | 100 | 114 |  |  | PEAKS DB |
| G.ALYTLITPAVLR.T | N | 77.10 | 1329.8020 | 12 | 0.6 | 665.9087 | 2 | 39.11 | 1 | 25893 | BuCaMH04.raw | 3.4301E7 | 1 | 1 | 23 | 34 |  |  | PEAKS DB |
| K.YFTYLILNK.G | N | 75.76 | 1173.6433 | 9 | 0.9 | 587.8295 | 2 | 33.34 | 1 | 20777 | BuCaMH04.raw | 1.8251E7 | 1 | 1 | 478 | 486 |  |  | PEAKS DB |
| R.VELLYNPAFC(+57.02)SASTK.G | Y | 75.53 | 1698.8286 | 15 | 0.8 | 850.4223 | 2 | 27.80 | 1 | 15715 | BuCaMH04.raw | 3.809E7 | 1 | 1 | 848 | 862 | Carbamidomethylation | C10:Carbamidomethylation:1000.00 | PEAKS DB |
| R.DDNEDGFIADSDIISR.S | Y | 74.02 | 1780.7751 | 16 | 1.2 | 891.3959 | 2 | 32.87 | 1 | 20586 | BuCaMH04.raw | 1.1975E7 | 1 | 1 | 733 | 748 |  |  | PEAKS DB |
| K.LILNIPLNAQSLPITVR.T | Y | 71.01 | 1874.1353 | 17 | 0.8 | 625.7195 | 3 | 45.50 | 1 | 31737 | BuCaMH04.raw | 1.3854E7 | 2 | 2 | 398 | 414 |  |  | PEAKS DB |
| H.GALYTLITPAVLR.T | N | 67.01 | 1386.8235 | 13 | 0.6 | 694.4194 | 2 | 39.60 | 1 | 26478 | BuCaMH04.raw | 7.2514E6 | 1 | 1 | 22 | 34 |  |  | PEAKS DB |
| K.ASVQEALWSDGVR.K | Y | 66.69 | 1416.6997 | 13 | 0.7 | 709.3576 | 2 | 24.61 | 1 | 12813 | BuCaMH04.raw | 1.6033E7 | 1 | 1 | 898 | 910 |  |  | PEAKS DB |
| K.YVLPSFEVR.L | N | 63.14 | 1108.5917 | 9 | 0.7 | 555.3035 | 2 | 28.54 | 1 | 16534 | BuCaMH04.raw | 1.3942E7 | 1 | 1 | 219 | 227 |  |  | PEAKS DB |
| K.IIIQGDPVAQIIEN.S | Y | 60.89 | 1521.8402 | 14 | 0.2 | 761.9276 | 2 | 42.93 | 1 | 29516 | BuCaMH04.raw | 2.2646E7 | 1 | 1 | 963 | 976 |  |  | PEAKS DB |
| K.ATMTILTFYNAQLQEK.A | Y | 60.55 | 1870.9498 | 16 | 1.2 | 936.4833 | 2 | 41.38 | 1 | 27984 | BuCaMH04.raw | 7.9697E6 | 1 | 1 | 1321 | 1336 |  |  | PEAKS DB |
| K.IIIQGDPVAQIIENSIDGSK.L | Y | 54.34 | 2109.1316 | 20 | 1.3 | 1055.5745 | 2 | 52.79 | 1 | 38314 | BuCaMH04.raw | 8.2472E5 | 1 | 1 | 963 | 982 |  |  | PEAKS DB |
| K.GIYTPGSPVLYR.V | N | 52.60 | 1321.7030 | 12 | 0.4 | 661.8590 | 2 | 22.35 | 1 | 11114 | BuCaMH04.raw | 2.9354E7 | 1 | 1 | 135 | 146 |  |  | PEAKS DB |
| R.VELLYNPAFC(+57.02)SASTKG.Q | Y | 52.38 | 1755.8501 | 16 | 0.6 | 878.9329 | 2 | 27.53 | 1 | 15433 | BuCaMH04.raw | 1.6571E6 | 1 | 1 | 848 | 863 | Carbamidomethylation | C10:Carbamidomethylation:1000.00 | PEAKS DB |
| R.DSITTWVVLAVSFTPTK.G | Y | 51.87 | 1863.9982 | 17 | 0.7 | 933.0070 | 2 | 67.31 | 1 | 50500 | BuCaMH04.raw | 3.3834E5 | 1 | 1 | 782 | 798 |  |  | PEAKS DB |
| total 23 peptides |  |  |  |  |  |  |  |  |  |  |  |  |  |  |  |  |  |  |  |
| --- | --- | --- | --- | --- | --- | --- | --- | --- | --- | --- | --- | --- | --- | --- | --- | --- | --- | --- | --- |

3PVM

back to list

  

| Protein Coverage
| Supporting Peptides
|

Protein Coverage:

Supporting Peptides:

| Peptide | Uniq | -10lgP | Mass | Length | ppm | m/z | z | RT | Fraction | Scan | Source File | Area BuCaMH04\_B15 | #Feature | #Feature BuCaMH04\_B15 | Start | End | PTM | AScore | Found By |
| --- | --- | --- | --- | --- | --- | --- | --- | --- | --- | --- | --- | --- | --- | --- | --- | --- | --- | --- | --- |
| R.IEEQDGNDIYVMDVLEVIK.Q | Y | 127.97 | 2221.0823 | 19 | -0.2 | 1111.5482 | 2 | 58.14 | 1 | 42915 | BuCaMH04.raw | 1.5532E7 | 2 | 2 | 1531 | 1549 |  |  | PEAKS DB |
| R.YLYGEEVEGVAFVLFGVK.I | N | 101.23 | 2018.0400 | 18 | 0.2 | 1010.0275 | 2 | 64.43 | 1 | 48252 | BuCaMH04.raw | 7.4395E6 | 2 | 2 | 251 | 268 |  |  | PEAKS DB |
| R.AVPFVIVPLEQGLHDVEIK.A | Y | 101.16 | 2102.1775 | 19 | 0.6 | 701.7335 | 3 | 44.28 | 1 | 30806 | BuCaMH04.raw | 7.6124E7 | 2 | 2 | 879 | 897 |  |  | PEAKS DB |
| K.VNDDYLIWGSR.S | Y | 99.55 | 1336.6411 | 11 | 0.6 | 669.3282 | 2 | 31.12 | 1 | 18953 | BuCaMH04.raw | 5.2426E7 | 1 | 1 | 1577 | 1587 |  |  | PEAKS DB |
| E.VEGVAFVLFGVK.I | N | 94.83 | 1263.7227 | 12 | 0.3 | 632.8688 | 2 | 46.03 | 1 | 32338 | BuCaMH04.raw | 2.7212E6 | 1 | 1 | 257 | 268 |  |  | PEAKS DB |
| K.FFYIDGNENFHVSITAR.Y | Y | 86.65 | 2028.9694 | 17 | 0.1 | 677.3304 | 3 | 33.21 | 1 | 20689 | BuCaMH04.raw | 6.862E6 | 1 | 1 | 234 | 250 |  |  | PEAKS DB |
| K.DLNLDITIELPDR.E | Y | 81.02 | 1525.7987 | 13 | 0.3 | 763.9069 | 2 | 45.23 | 1 | 31751 | BuCaMH04.raw | 2.5874E6 | 2 | 2 | 1272 | 1284 |  |  | PEAKS DB |
| R.INYENALLAR.T | Y | 80.85 | 1175.6299 | 10 | 1.4 | 588.8231 | 2 | 22.36 | 1 | 10916 | BuCaMH04.raw | 1.4785E7 | 1 | 1 | 1292 | 1301 |  |  | PEAKS DB |
| R.QNQYVVVQVTGPQVR.L | N | 77.22 | 1713.9163 | 15 | 0.6 | 572.3130 | 3 | 19.26 | 1 | 8230 | BuCaMH04.raw | 2.1525E7 | 2 | 2 | 100 | 114 |  |  | PEAKS DB |
| G.ALYTLITPAVLR.T | N | 77.10 | 1329.8020 | 12 | 0.6 | 665.9087 | 2 | 39.11 | 1 | 25893 | BuCaMH04.raw | 3.4301E7 | 1 | 1 | 23 | 34 |  |  | PEAKS DB |
| K.YFTYLILNK.G | N | 75.76 | 1173.6433 | 9 | 0.9 | 587.8295 | 2 | 33.34 | 1 | 20777 | BuCaMH04.raw | 1.8251E7 | 1 | 1 | 478 | 486 |  |  | PEAKS DB |
| R.VELLYNPAFC(+57.02)SASTK.G | Y | 75.53 | 1698.8286 | 15 | 0.8 | 850.4223 | 2 | 27.80 | 1 | 15715 | BuCaMH04.raw | 3.809E7 | 1 | 1 | 848 | 862 | Carbamidomethylation | C10:Carbamidomethylation:1000.00 | PEAKS DB |
| R.DDNEDGFIADSDIISR.S | Y | 74.02 | 1780.7751 | 16 | 1.2 | 891.3959 | 2 | 32.87 | 1 | 20586 | BuCaMH04.raw | 1.1975E7 | 1 | 1 | 733 | 748 |  |  | PEAKS DB |
| K.LILNIPLNAQSLPITVR.T | Y | 71.01 | 1874.1353 | 17 | 0.8 | 625.7195 | 3 | 45.50 | 1 | 31737 | BuCaMH04.raw | 1.3854E7 | 2 | 2 | 398 | 414 |  |  | PEAKS DB |
| H.GALYTLITPAVLR.T | N | 67.01 | 1386.8235 | 13 | 0.6 | 694.4194 | 2 | 39.60 | 1 | 26478 | BuCaMH04.raw | 7.2514E6 | 1 | 1 | 22 | 34 |  |  | PEAKS DB |
| K.ASVQEALWSDGVR.K | Y | 66.69 | 1416.6997 | 13 | 0.7 | 709.3576 | 2 | 24.61 | 1 | 12813 | BuCaMH04.raw | 1.6033E7 | 1 | 1 | 898 | 910 |  |  | PEAKS DB |
| K.YVLPSFEVR.L | N | 63.14 | 1108.5917 | 9 | 0.7 | 555.3035 | 2 | 28.54 | 1 | 16534 | BuCaMH04.raw | 1.3942E7 | 1 | 1 | 219 | 227 |  |  | PEAKS DB |
| K.IIIQGDPVAQIIEN.S | Y | 60.89 | 1521.8402 | 14 | 0.2 | 761.9276 | 2 | 42.93 | 1 | 29516 | BuCaMH04.raw | 2.2646E7 | 1 | 1 | 963 | 976 |  |  | PEAKS DB |
| K.ATMTILTFYNAQLQEK.A | Y | 60.55 | 1870.9498 | 16 | 1.2 | 936.4833 | 2 | 41.38 | 1 | 27984 | BuCaMH04.raw | 7.9697E6 | 1 | 1 | 1321 | 1336 |  |  | PEAKS DB |
| K.IIIQGDPVAQIIENSIDGSK.L | Y | 54.34 | 2109.1316 | 20 | 1.3 | 1055.5745 | 2 | 52.79 | 1 | 38314 | BuCaMH04.raw | 8.2472E5 | 1 | 1 | 963 | 982 |  |  | PEAKS DB |
| K.GIYTPGSPVLYR.V | N | 52.60 | 1321.7030 | 12 | 0.4 | 661.8590 | 2 | 22.35 | 1 | 11114 | BuCaMH04.raw | 2.9354E7 | 1 | 1 | 135 | 146 |  |  | PEAKS DB |
| R.VELLYNPAFC(+57.02)SASTKG.Q | Y | 52.38 | 1755.8501 | 16 | 0.6 | 878.9329 | 2 | 27.53 | 1 | 15433 | BuCaMH04.raw | 1.6571E6 | 1 | 1 | 848 | 863 | Carbamidomethylation | C10:Carbamidomethylation:1000.00 | PEAKS DB |
| R.DSITTWVVLAVSFTPTK.G | Y | 51.87 | 1863.9982 | 17 | 0.7 | 933.0070 | 2 | 67.31 | 1 | 50500 | BuCaMH04.raw | 3.3834E5 | 1 | 1 | 782 | 798 |  |  | PEAKS DB |
| total 23 peptides |  |  |  |  |  |  |  |  |  |  |  |  |  |  |  |  |  |  |  |
| --- | --- | --- | --- | --- | --- | --- | --- | --- | --- | --- | --- | --- | --- | --- | --- | --- | --- | --- | --- |

AAA68989.1

back to list

  

| Protein Coverage
| Supporting Peptides
|

Protein Coverage:

Supporting Peptides:

| Peptide | Uniq | -10lgP | Mass | Length | ppm | m/z | z | RT | Fraction | Scan | Source File | Area BuCaMH04\_B15 | #Feature | #Feature BuCaMH04\_B15 | Start | End | PTM | AScore | Found By |
| --- | --- | --- | --- | --- | --- | --- | --- | --- | --- | --- | --- | --- | --- | --- | --- | --- | --- | --- | --- |
| R.IEEQDGNDIYVMDVLEVIK.Q | Y | 127.97 | 2221.0823 | 19 | -0.2 | 1111.5482 | 2 | 58.14 | 1 | 42915 | BuCaMH04.raw | 1.5532E7 | 2 | 2 | 1531 | 1549 |  |  | PEAKS DB |
| R.YLYGEEVEGVAFVLFGVK.I | N | 101.23 | 2018.0400 | 18 | 0.2 | 1010.0275 | 2 | 64.43 | 1 | 48252 | BuCaMH04.raw | 7.4395E6 | 2 | 2 | 251 | 268 |  |  | PEAKS DB |
| R.AVPFVIVPLEQGLHDVEIK.A | Y | 101.16 | 2102.1775 | 19 | 0.6 | 701.7335 | 3 | 44.28 | 1 | 30806 | BuCaMH04.raw | 7.6124E7 | 2 | 2 | 879 | 897 |  |  | PEAKS DB |
| K.VNDDYLIWGSR.S | Y | 99.55 | 1336.6411 | 11 | 0.6 | 669.3282 | 2 | 31.12 | 1 | 18953 | BuCaMH04.raw | 5.2426E7 | 1 | 1 | 1577 | 1587 |  |  | PEAKS DB |
| E.VEGVAFVLFGVK.I | N | 94.83 | 1263.7227 | 12 | 0.3 | 632.8688 | 2 | 46.03 | 1 | 32338 | BuCaMH04.raw | 2.7212E6 | 1 | 1 | 257 | 268 |  |  | PEAKS DB |
| K.FFYIDGNENFHVSITAR.Y | Y | 86.65 | 2028.9694 | 17 | 0.1 | 677.3304 | 3 | 33.21 | 1 | 20689 | BuCaMH04.raw | 6.862E6 | 1 | 1 | 234 | 250 |  |  | PEAKS DB |
| K.DLNLDITIELPDR.E | Y | 81.02 | 1525.7987 | 13 | 0.3 | 763.9069 | 2 | 45.23 | 1 | 31751 | BuCaMH04.raw | 2.5874E6 | 2 | 2 | 1272 | 1284 |  |  | PEAKS DB |
| R.INYENALLAR.T | Y | 80.85 | 1175.6299 | 10 | 1.4 | 588.8231 | 2 | 22.36 | 1 | 10916 | BuCaMH04.raw | 1.4785E7 | 1 | 1 | 1292 | 1301 |  |  | PEAKS DB |
| R.QNQYVVVQVTGPQVR.L | N | 77.22 | 1713.9163 | 15 | 0.6 | 572.3130 | 3 | 19.26 | 1 | 8230 | BuCaMH04.raw | 2.1525E7 | 2 | 2 | 100 | 114 |  |  | PEAKS DB |
| G.ALYTLITPAVLR.T | N | 77.10 | 1329.8020 | 12 | 0.6 | 665.9087 | 2 | 39.11 | 1 | 25893 | BuCaMH04.raw | 3.4301E7 | 1 | 1 | 23 | 34 |  |  | PEAKS DB |
| K.YFTYLILNK.G | N | 75.76 | 1173.6433 | 9 | 0.9 | 587.8295 | 2 | 33.34 | 1 | 20777 | BuCaMH04.raw | 1.8251E7 | 1 | 1 | 478 | 486 |  |  | PEAKS DB |
| R.VELLYNPAFC(+57.02)SASTK.G | Y | 75.53 | 1698.8286 | 15 | 0.8 | 850.4223 | 2 | 27.80 | 1 | 15715 | BuCaMH04.raw | 3.809E7 | 1 | 1 | 848 | 862 | Carbamidomethylation | C10:Carbamidomethylation:1000.00 | PEAKS DB |
| R.DDNEDGFIADSDIISR.S | Y | 74.02 | 1780.7751 | 16 | 1.2 | 891.3959 | 2 | 32.87 | 1 | 20586 | BuCaMH04.raw | 1.1975E7 | 1 | 1 | 733 | 748 |  |  | PEAKS DB |
| K.LILNIPLNAQSLPITVR.T | Y | 71.01 | 1874.1353 | 17 | 0.8 | 625.7195 | 3 | 45.50 | 1 | 31737 | BuCaMH04.raw | 1.3854E7 | 2 | 2 | 398 | 414 |  |  | PEAKS DB |
| H.GALYTLITPAVLR.T | N | 67.01 | 1386.8235 | 13 | 0.6 | 694.4194 | 2 | 39.60 | 1 | 26478 | BuCaMH04.raw | 7.2514E6 | 1 | 1 | 22 | 34 |  |  | PEAKS DB |
| K.ASVQEALWSDGVR.K | Y | 66.69 | 1416.6997 | 13 | 0.7 | 709.3576 | 2 | 24.61 | 1 | 12813 | BuCaMH04.raw | 1.6033E7 | 1 | 1 | 898 | 910 |  |  | PEAKS DB |
| K.YVLPSFEVR.L | N | 63.14 | 1108.5917 | 9 | 0.7 | 555.3035 | 2 | 28.54 | 1 | 16534 | BuCaMH04.raw | 1.3942E7 | 1 | 1 | 219 | 227 |  |  | PEAKS DB |
| K.IIIQGDPVAQIIEN.S | Y | 60.89 | 1521.8402 | 14 | 0.2 | 761.9276 | 2 | 42.93 | 1 | 29516 | BuCaMH04.raw | 2.2646E7 | 1 | 1 | 963 | 976 |  |  | PEAKS DB |
| K.ATMTILTFYNAQLQEK.A | Y | 60.55 | 1870.9498 | 16 | 1.2 | 936.4833 | 2 | 41.38 | 1 | 27984 | BuCaMH04.raw | 7.9697E6 | 1 | 1 | 1321 | 1336 |  |  | PEAKS DB |
| K.IIIQGDPVAQIIENSIDGSK.L | Y | 54.34 | 2109.1316 | 20 | 1.3 | 1055.5745 | 2 | 52.79 | 1 | 38314 | BuCaMH04.raw | 8.2472E5 | 1 | 1 | 963 | 982 |  |  | PEAKS DB |
| K.GIYTPGSPVLYR.V | N | 52.60 | 1321.7030 | 12 | 0.4 | 661.8590 | 2 | 22.35 | 1 | 11114 | BuCaMH04.raw | 2.9354E7 | 1 | 1 | 135 | 146 |  |  | PEAKS DB |
| R.VELLYNPAFC(+57.02)SASTKG.Q | Y | 52.38 | 1755.8501 | 16 | 0.6 | 878.9329 | 2 | 27.53 | 1 | 15433 | BuCaMH04.raw | 1.6571E6 | 1 | 1 | 848 | 863 | Carbamidomethylation | C10:Carbamidomethylation:1000.00 | PEAKS DB |
| R.DSITTWVVLAVSFTPTK.G | Y | 51.87 | 1863.9982 | 17 | 0.7 | 933.0070 | 2 | 67.31 | 1 | 50500 | BuCaMH04.raw | 3.3834E5 | 1 | 1 | 782 | 798 |  |  | PEAKS DB |
| total 23 peptides |  |  |  |  |  |  |  |  |  |  |  |  |  |  |  |  |  |  |  |
| --- | --- | --- | --- | --- | --- | --- | --- | --- | --- | --- | --- | --- | --- | --- | --- | --- | --- | --- | --- |

Q91132.1

back to list

  

| Protein Coverage
| Supporting Peptides
|

Protein Coverage:

Supporting Peptides:

| Peptide | Uniq | -10lgP | Mass | Length | ppm | m/z | z | RT | Fraction | Scan | Source File | Area BuCaMH04\_B15 | #Feature | #Feature BuCaMH04\_B15 | Start | End | PTM | AScore | Found By |
| --- | --- | --- | --- | --- | --- | --- | --- | --- | --- | --- | --- | --- | --- | --- | --- | --- | --- | --- | --- |
| R.IEEQDGNDIYVMDVLEVIK.Q | Y | 127.97 | 2221.0823 | 19 | -0.2 | 1111.5482 | 2 | 58.14 | 1 | 42915 | BuCaMH04.raw | 1.5532E7 | 2 | 2 | 1531 | 1549 |  |  | PEAKS DB |
| R.YLYGEEVEGVAFVLFGVK.I | N | 101.23 | 2018.0400 | 18 | 0.2 | 1010.0275 | 2 | 64.43 | 1 | 48252 | BuCaMH04.raw | 7.4395E6 | 2 | 2 | 251 | 268 |  |  | PEAKS DB |
| R.AVPFVIVPLEQGLHDVEIK.A | Y | 101.16 | 2102.1775 | 19 | 0.6 | 701.7335 | 3 | 44.28 | 1 | 30806 | BuCaMH04.raw | 7.6124E7 | 2 | 2 | 879 | 897 |  |  | PEAKS DB |
| K.VNDDYLIWGSR.S | Y | 99.55 | 1336.6411 | 11 | 0.6 | 669.3282 | 2 | 31.12 | 1 | 18953 | BuCaMH04.raw | 5.2426E7 | 1 | 1 | 1577 | 1587 |  |  | PEAKS DB |
| E.VEGVAFVLFGVK.I | N | 94.83 | 1263.7227 | 12 | 0.3 | 632.8688 | 2 | 46.03 | 1 | 32338 | BuCaMH04.raw | 2.7212E6 | 1 | 1 | 257 | 268 |  |  | PEAKS DB |
| K.FFYIDGNENFHVSITAR.Y | Y | 86.65 | 2028.9694 | 17 | 0.1 | 677.3304 | 3 | 33.21 | 1 | 20689 | BuCaMH04.raw | 6.862E6 | 1 | 1 | 234 | 250 |  |  | PEAKS DB |
| K.DLNLDITIELPDR.E | Y | 81.02 | 1525.7987 | 13 | 0.3 | 763.9069 | 2 | 45.23 | 1 | 31751 | BuCaMH04.raw | 2.5874E6 | 2 | 2 | 1272 | 1284 |  |  | PEAKS DB |
| R.INYENALLAR.T | Y | 80.85 | 1175.6299 | 10 | 1.4 | 588.8231 | 2 | 22.36 | 1 | 10916 | BuCaMH04.raw | 1.4785E7 | 1 | 1 | 1292 | 1301 |  |  | PEAKS DB |
| R.QNQYVVVQVTGPQVR.L | N | 77.22 | 1713.9163 | 15 | 0.6 | 572.3130 | 3 | 19.26 | 1 | 8230 | BuCaMH04.raw | 2.1525E7 | 2 | 2 | 100 | 114 |  |  | PEAKS DB |
| G.ALYTLITPAVLR.T | N | 77.10 | 1329.8020 | 12 | 0.6 | 665.9087 | 2 | 39.11 | 1 | 25893 | BuCaMH04.raw | 3.4301E7 | 1 | 1 | 23 | 34 |  |  | PEAKS DB |
| K.YFTYLILNK.G | N | 75.76 | 1173.6433 | 9 | 0.9 | 587.8295 | 2 | 33.34 | 1 | 20777 | BuCaMH04.raw | 1.8251E7 | 1 | 1 | 478 | 486 |  |  | PEAKS DB |
| R.VELLYNPAFC(+57.02)SASTK.G | Y | 75.53 | 1698.8286 | 15 | 0.8 | 850.4223 | 2 | 27.80 | 1 | 15715 | BuCaMH04.raw | 3.809E7 | 1 | 1 | 848 | 862 | Carbamidomethylation | C10:Carbamidomethylation:1000.00 | PEAKS DB |
| R.DDNEDGFIADSDIISR.S | Y | 74.02 | 1780.7751 | 16 | 1.2 | 891.3959 | 2 | 32.87 | 1 | 20586 | BuCaMH04.raw | 1.1975E7 | 1 | 1 | 733 | 748 |  |  | PEAKS DB |
| K.LILNIPLNAQSLPITVR.T | Y | 71.01 | 1874.1353 | 17 | 0.8 | 625.7195 | 3 | 45.50 | 1 | 31737 | BuCaMH04.raw | 1.3854E7 | 2 | 2 | 398 | 414 |  |  | PEAKS DB |
| H.GALYTLITPAVLR.T | N | 67.01 | 1386.8235 | 13 | 0.6 | 694.4194 | 2 | 39.60 | 1 | 26478 | BuCaMH04.raw | 7.2514E6 | 1 | 1 | 22 | 34 |  |  | PEAKS DB |
| K.ASVQEALWSDGVR.K | Y | 66.69 | 1416.6997 | 13 | 0.7 | 709.3576 | 2 | 24.61 | 1 | 12813 | BuCaMH04.raw | 1.6033E7 | 1 | 1 | 898 | 910 |  |  | PEAKS DB |
| K.YVLPSFEVR.L | N | 63.14 | 1108.5917 | 9 | 0.7 | 555.3035 | 2 | 28.54 | 1 | 16534 | BuCaMH04.raw | 1.3942E7 | 1 | 1 | 219 | 227 |  |  | PEAKS DB |
| K.IIIQGDPVAQIIEN.S | Y | 60.89 | 1521.8402 | 14 | 0.2 | 761.9276 | 2 | 42.93 | 1 | 29516 | BuCaMH04.raw | 2.2646E7 | 1 | 1 | 963 | 976 |  |  | PEAKS DB |
| K.ATMTILTFYNAQLQEK.A | Y | 60.55 | 1870.9498 | 16 | 1.2 | 936.4833 | 2 | 41.38 | 1 | 27984 | BuCaMH04.raw | 7.9697E6 | 1 | 1 | 1321 | 1336 |  |  | PEAKS DB |
| K.IIIQGDPVAQIIENSIDGSK.L | Y | 54.34 | 2109.1316 | 20 | 1.3 | 1055.5745 | 2 | 52.79 | 1 | 38314 | BuCaMH04.raw | 8.2472E5 | 1 | 1 | 963 | 982 |  |  | PEAKS DB |
| K.GIYTPGSPVLYR.V | N | 52.60 | 1321.7030 | 12 | 0.4 | 661.8590 | 2 | 22.35 | 1 | 11114 | BuCaMH04.raw | 2.9354E7 | 1 | 1 | 135 | 146 |  |  | PEAKS DB |
| R.VELLYNPAFC(+57.02)SASTKG.Q | Y | 52.38 | 1755.8501 | 16 | 0.6 | 878.9329 | 2 | 27.53 | 1 | 15433 | BuCaMH04.raw | 1.6571E6 | 1 | 1 | 848 | 863 | Carbamidomethylation | C10:Carbamidomethylation:1000.00 | PEAKS DB |
| R.DSITTWVVLAVSFTPTK.G | Y | 51.87 | 1863.9982 | 17 | 0.7 | 933.0070 | 2 | 67.31 | 1 | 50500 | BuCaMH04.raw | 3.3834E5 | 1 | 1 | 782 | 798 |  |  | PEAKS DB |
| total 23 peptides |  |  |  |  |  |  |  |  |  |  |  |  |  |  |  |  |  |  |  |
| --- | --- | --- | --- | --- | --- | --- | --- | --- | --- | --- | --- | --- | --- | --- | --- | --- | --- | --- | --- |

CAI46845.1

back to list

  

| Protein Coverage
| Supporting Peptides
|

Protein Coverage:

Supporting Peptides:

| Peptide | Uniq | -10lgP | Mass | Length | ppm | m/z | z | RT | Fraction | Scan | Source File | Area BuCaMH04\_B15 | #Feature | #Feature BuCaMH04\_B15 | Start | End | PTM | AScore | Found By |
| --- | --- | --- | --- | --- | --- | --- | --- | --- | --- | --- | --- | --- | --- | --- | --- | --- | --- | --- | --- |
| R.IEEQDGNDIYVMDVLEVIK.Q | Y | 127.97 | 2221.0823 | 19 | -0.2 | 1111.5482 | 2 | 58.14 | 1 | 42915 | BuCaMH04.raw | 1.5532E7 | 2 | 2 | 1531 | 1549 |  |  | PEAKS DB |
| R.YLYGEEVEGVAFVLFGVK.I | N | 101.23 | 2018.0400 | 18 | 0.2 | 1010.0275 | 2 | 64.43 | 1 | 48252 | BuCaMH04.raw | 7.4395E6 | 2 | 2 | 251 | 268 |  |  | PEAKS DB |
| R.AVPFVIVPLEQGLHDVEIK.A | Y | 101.16 | 2102.1775 | 19 | 0.6 | 701.7335 | 3 | 44.28 | 1 | 30806 | BuCaMH04.raw | 7.6124E7 | 2 | 2 | 879 | 897 |  |  | PEAKS DB |
| K.VNDDYLIWGSR.S | Y | 99.55 | 1336.6411 | 11 | 0.6 | 669.3282 | 2 | 31.12 | 1 | 18953 | BuCaMH04.raw | 5.2426E7 | 1 | 1 | 1577 | 1587 |  |  | PEAKS DB |
| E.VEGVAFVLFGVK.I | N | 94.83 | 1263.7227 | 12 | 0.3 | 632.8688 | 2 | 46.03 | 1 | 32338 | BuCaMH04.raw | 2.7212E6 | 1 | 1 | 257 | 268 |  |  | PEAKS DB |
| K.FFYIDGNENFHVSITAR.Y | Y | 86.65 | 2028.9694 | 17 | 0.1 | 677.3304 | 3 | 33.21 | 1 | 20689 | BuCaMH04.raw | 6.862E6 | 1 | 1 | 234 | 250 |  |  | PEAKS DB |
| K.DLNLDITIELPDR.E | Y | 81.02 | 1525.7987 | 13 | 0.3 | 763.9069 | 2 | 45.23 | 1 | 31751 | BuCaMH04.raw | 2.5874E6 | 2 | 2 | 1272 | 1284 |  |  | PEAKS DB |
| R.INYENALLAR.T | Y | 80.85 | 1175.6299 | 10 | 1.4 | 588.8231 | 2 | 22.36 | 1 | 10916 | BuCaMH04.raw | 1.4785E7 | 1 | 1 | 1292 | 1301 |  |  | PEAKS DB |
| R.QNQYVVVQVTGPQVR.L | N | 77.22 | 1713.9163 | 15 | 0.6 | 572.3130 | 3 | 19.26 | 1 | 8230 | BuCaMH04.raw | 2.1525E7 | 2 | 2 | 100 | 114 |  |  | PEAKS DB |
| G.ALYTLITPAVLR.T | N | 77.10 | 1329.8020 | 12 | 0.6 | 665.9087 | 2 | 39.11 | 1 | 25893 | BuCaMH04.raw | 3.4301E7 | 1 | 1 | 23 | 34 |  |  | PEAKS DB |
| K.YFTYLILNK.G | N | 75.76 | 1173.6433 | 9 | 0.9 | 587.8295 | 2 | 33.34 | 1 | 20777 | BuCaMH04.raw | 1.8251E7 | 1 | 1 | 478 | 486 |  |  | PEAKS DB |
| R.VELLYNPAFC(+57.02)SASTK.G | Y | 75.53 | 1698.8286 | 15 | 0.8 | 850.4223 | 2 | 27.80 | 1 | 15715 | BuCaMH04.raw | 3.809E7 | 1 | 1 | 848 | 862 | Carbamidomethylation | C10:Carbamidomethylation:1000.00 | PEAKS DB |
| R.DDNEDGFIADSDIISR.S | Y | 74.02 | 1780.7751 | 16 | 1.2 | 891.3959 | 2 | 32.87 | 1 | 20586 | BuCaMH04.raw | 1.1975E7 | 1 | 1 | 733 | 748 |  |  | PEAKS DB |
| K.LILNIPLNAQSLPITVR.T | Y | 71.01 | 1874.1353 | 17 | 0.8 | 625.7195 | 3 | 45.50 | 1 | 31737 | BuCaMH04.raw | 1.3854E7 | 2 | 2 | 398 | 414 |  |  | PEAKS DB |
| H.GALYTLITPAVLR.T | N | 67.01 | 1386.8235 | 13 | 0.6 | 694.4194 | 2 | 39.60 | 1 | 26478 | BuCaMH04.raw | 7.2514E6 | 1 | 1 | 22 | 34 |  |  | PEAKS DB |
| K.ASVQEALWSDGVR.K | Y | 66.69 | 1416.6997 | 13 | 0.7 | 709.3576 | 2 | 24.61 | 1 | 12813 | BuCaMH04.raw | 1.6033E7 | 1 | 1 | 898 | 910 |  |  | PEAKS DB |
| K.YVLPSFEVR.L | N | 63.14 | 1108.5917 | 9 | 0.7 | 555.3035 | 2 | 28.54 | 1 | 16534 | BuCaMH04.raw | 1.3942E7 | 1 | 1 | 219 | 227 |  |  | PEAKS DB |
| K.IIIQGDPVAQIIEN.S | Y | 60.89 | 1521.8402 | 14 | 0.2 | 761.9276 | 2 | 42.93 | 1 | 29516 | BuCaMH04.raw | 2.2646E7 | 1 | 1 | 963 | 976 |  |  | PEAKS DB |
| K.ATMTILTFYNAQLQEK.A | Y | 60.55 | 1870.9498 | 16 | 1.2 | 936.4833 | 2 | 41.38 | 1 | 27984 | BuCaMH04.raw | 7.9697E6 | 1 | 1 | 1321 | 1336 |  |  | PEAKS DB |
| K.IIIQGDPVAQIIENSIDGSK.L | Y | 54.34 | 2109.1316 | 20 | 1.3 | 1055.5745 | 2 | 52.79 | 1 | 38314 | BuCaMH04.raw | 8.2472E5 | 1 | 1 | 963 | 982 |  |  | PEAKS DB |
| K.GIYTPGSPVLYR.V | N | 52.60 | 1321.7030 | 12 | 0.4 | 661.8590 | 2 | 22.35 | 1 | 11114 | BuCaMH04.raw | 2.9354E7 | 1 | 1 | 135 | 146 |  |  | PEAKS DB |
| R.VELLYNPAFC(+57.02)SASTKG.Q | Y | 52.38 | 1755.8501 | 16 | 0.6 | 878.9329 | 2 | 27.53 | 1 | 15433 | BuCaMH04.raw | 1.6571E6 | 1 | 1 | 848 | 863 | Carbamidomethylation | C10:Carbamidomethylation:1000.00 | PEAKS DB |
| R.DSITTWVVLAVSFTPTK.G | Y | 51.87 | 1863.9982 | 17 | 0.7 | 933.0070 | 2 | 67.31 | 1 | 50500 | BuCaMH04.raw | 3.3834E5 | 1 | 1 | 782 | 798 |  |  | PEAKS DB |
| total 23 peptides |  |  |  |  |  |  |  |  |  |  |  |  |  |  |  |  |  |  |  |
| --- | --- | --- | --- | --- | --- | --- | --- | --- | --- | --- | --- | --- | --- | --- | --- | --- | --- | --- | --- |

5Z2G

back to list

  

| Protein Coverage
| Supporting Peptides
|

Protein Coverage:

Supporting Peptides:

| Peptide | Uniq | -10lgP | Mass | Length | ppm | m/z | z | RT | Fraction | Scan | Source File | Area BuCaMH04\_B15 | #Feature | #Feature BuCaMH04\_B15 | Start | End | PTM | AScore | Found By |
| --- | --- | --- | --- | --- | --- | --- | --- | --- | --- | --- | --- | --- | --- | --- | --- | --- | --- | --- | --- |
| K.TC(+57.02)ADIVINDLSLIHDLPK.R | Y | 123.10 | 2036.0612 | 18 | -0.1 | 1019.0377 | 2 | 43.44 | 1 | 29941 | BuCaMH04.raw | 2.6763E8 | 3 | 3 | 405 | 422 | Carbamidomethylation | C2:Carbamidomethylation:1000.00 | PEAKS DB |
| K.LNEFFQENENAWYYINNIR.K | Y | 107.89 | 2476.1448 | 19 | -0.3 | 1239.0793 | 2 | 51.15 | 1 | 36690 | BuCaMH04.raw | 5.8054E7 | 2 | 2 | 128 | 146 |  |  | PEAKS DB |
| K.HVVVVGAGMAGLSAAYVLAGAGHK.V | N | 106.63 | 2234.1992 | 24 | 0.7 | 745.7409 | 3 | 30.19 | 1 | 18119 | BuCaMH04.raw | 2.655E7 | 1 | 1 | 54 | 77 |  |  | PEAKS DB |
| K.TFVTADYVIVC(+57.02)STSR.A | Y | 96.20 | 1717.8345 | 15 | 0.1 | 859.9246 | 2 | 28.83 | 1 | 16853 | BuCaMH04.raw | 1.3866E8 | 2 | 2 | 300 | 314 | Carbamidomethylation | C11:Carbamidomethylation:1000.00 | PEAKS DB |
| K.YTMGSITSFTPYQFQDYFESAAAPVGR.I | Y | 94.90 | 3033.3855 | 27 | 0.5 | 1012.1362 | 3 | 55.78 | 1 | 40894 | BuCaMH04.raw | 1.2675E7 | 1 | 1 | 441 | 467 |  |  | PEAKS DB |
| Y.VLADDSDFFQALDTK.T | Y | 93.09 | 1683.7991 | 15 | 0.7 | 842.9074 | 2 | 40.29 | 1 | 26943 | BuCaMH04.raw | 1.1718E8 | 1 | 1 | 390 | 404 |  |  | PEAKS DB |
| M.AYVLADDSDFFQALDTK.T | Y | 92.42 | 1917.8995 | 17 | -0.3 | 959.9568 | 2 | 46.01 | 1 | 32305 | BuCaMH04.raw | 5.6345E6 | 1 | 1 | 388 | 404 |  |  | PEAKS DB |
| S.FTPYQFQDYFESAAAPVGR.I | Y | 91.29 | 2193.0166 | 19 | 0.6 | 1097.5162 | 2 | 45.01 | 1 | 31430 | BuCaMH04.raw | 1.6354E7 | 2 | 2 | 449 | 467 |  |  | PEAKS DB |
| R.IYFEPPLPPK.K | N | 84.04 | 1199.6589 | 10 | 0.3 | 600.8369 | 2 | 25.08 | 1 | 14052 | BuCaMH04.raw | 8.9718E7 | 2 | 2 | 319 | 328 |  |  | PEAKS DB |
| R.FHGWIDSTIMTGLR.A | Y | 79.15 | 1632.8082 | 14 | 0.9 | 545.2772 | 3 | 34.16 | 1 | 21564 | BuCaMH04.raw | 5.0059E6 | 1 | 1 | 478 | 491 |  |  | PEAKS DB |
| F.TPYQFQDYFESAAAPVGR.I | Y | 79.08 | 2045.9482 | 18 | 0.7 | 1023.9821 | 2 | 37.99 | 1 | 25075 | BuCaMH04.raw | 4.1508E6 | 1 | 1 | 450 | 467 |  |  | PEAKS DB |
| A.DIVINDLSLIHDLPK.R | Y | 73.01 | 1703.9458 | 15 | -0.1 | 568.9891 | 3 | 41.18 | 1 | 27840 | BuCaMH04.raw | 5.8144E6 | 2 | 2 | 408 | 422 |  |  | PEAKS DB |
| Y.QFQDYFESAAAPVGR.I | Y | 72.55 | 1684.7844 | 15 | 0.6 | 843.4000 | 2 | 28.27 | 1 | 16233 | BuCaMH04.raw | 2.4355E7 | 2 | 2 | 453 | 467 |  |  | PEAKS DB |
| Q.FQDYFESAAAPVGR.I | Y | 71.07 | 1556.7258 | 14 | 1.0 | 779.3710 | 2 | 24.89 | 1 | 13101 | BuCaMH04.raw | 1.7333E6 | 1 | 1 | 454 | 467 |  |  | PEAKS DB |
| E.FFQENENAWYYINNIR.K | Y | 65.16 | 2119.9751 | 16 | -0.6 | 1060.9941 | 2 | 43.48 | 1 | 29962 | BuCaMH04.raw | 8.5243E5 | 1 | 1 | 131 | 146 |  |  | PEAKS DB |
| F.HGWIDSTIMTGLR.A | Y | 64.58 | 1485.7399 | 13 | 0.9 | 743.8779 | 2 | 28.60 | 1 | 16655 | BuCaMH04.raw | 9.0318E6 | 2 | 2 | 479 | 491 |  |  | PEAKS DB |
| S.ITSFTPYQFQDYFESAAAPVGR.I | Y | 64.21 | 2494.1804 | 22 | 0.5 | 1248.0981 | 2 | 49.09 | 1 | 35082 | BuCaMH04.raw | 6.3034E6 | 1 | 1 | 446 | 467 |  |  | PEAKS DB |
| H.GWIDSTIMTGLR.A | Y | 58.10 | 1348.6809 | 12 | 0.3 | 675.3480 | 2 | 41.65 | 1 | 28245 | BuCaMH04.raw | 7.7826E5 | 1 | 1 | 480 | 491 |  |  | PEAKS DB |
| A.YVLADDSDFFQALDTK.T | Y | 57.53 | 1846.8624 | 16 | 0.1 | 924.4385 | 2 | 44.71 | 1 | 31151 | BuCaMH04.raw | 2.4199E6 | 1 | 1 | 389 | 404 |  |  | PEAKS DB |
| A.DDSDFFQALDTK.T | Y | 57.09 | 1400.6095 | 12 | 0.3 | 701.3123 | 2 | 38.59 | 1 | 25553 | BuCaMH04.raw | 4.8806E6 | 1 | 1 | 393 | 404 |  |  | PEAKS DB |
| K.RFDEIVGGFDQLPIS.M | N | 52.08 | 1691.8518 | 15 | 0.2 | 846.9333 | 2 | 43.43 | 1 | 29880 | BuCaMH04.raw | 8.7751E5 | 1 | 1 | 251 | 265 |  |  | PEAKS DB |
| R.EGWYVNMGPMR.L | N | 52.03 | 1338.5850 | 11 | 0.3 | 670.3000 | 2 | 30.40 | 1 | 18128 | BuCaMH04.raw | 1.6917E6 | 1 | 1 | 99 | 109 |  |  | PEAKS DB |
| total 22 peptides |  |  |  |  |  |  |  |  |  |  |  |  |  |  |  |  |  |  |  |
| --- | --- | --- | --- | --- | --- | --- | --- | --- | --- | --- | --- | --- | --- | --- | --- | --- | --- | --- | --- |

AVX27607.1

back to list

  

| Protein Coverage
| Supporting Peptides
|

Protein Coverage:

Supporting Peptides:

| Peptide | Uniq | -10lgP | Mass | Length | ppm | m/z | z | RT | Fraction | Scan | Source File | Area BuCaMH04\_B15 | #Feature | #Feature BuCaMH04\_B15 | Start | End | PTM | AScore | Found By |
| --- | --- | --- | --- | --- | --- | --- | --- | --- | --- | --- | --- | --- | --- | --- | --- | --- | --- | --- | --- |
| K.TC(+57.02)ADIVINDLSLIHDLPK.R | Y | 123.10 | 2036.0612 | 18 | -0.1 | 1019.0377 | 2 | 43.44 | 1 | 29941 | BuCaMH04.raw | 2.6763E8 | 3 | 3 | 405 | 422 | Carbamidomethylation | C2:Carbamidomethylation:1000.00 | PEAKS DB |
| K.LNEFFQENENAWYYINNIR.K | Y | 107.89 | 2476.1448 | 19 | -0.3 | 1239.0793 | 2 | 51.15 | 1 | 36690 | BuCaMH04.raw | 5.8054E7 | 2 | 2 | 128 | 146 |  |  | PEAKS DB |
| K.HVVVVGAGMAGLSAAYVLAGAGHK.V | N | 106.63 | 2234.1992 | 24 | 0.7 | 745.7409 | 3 | 30.19 | 1 | 18119 | BuCaMH04.raw | 2.655E7 | 1 | 1 | 54 | 77 |  |  | PEAKS DB |
| K.TFVTADYVIVC(+57.02)STSR.A | Y | 96.20 | 1717.8345 | 15 | 0.1 | 859.9246 | 2 | 28.83 | 1 | 16853 | BuCaMH04.raw | 1.3866E8 | 2 | 2 | 300 | 314 | Carbamidomethylation | C11:Carbamidomethylation:1000.00 | PEAKS DB |
| K.YTMGSITSFTPYQFQDYFESAAAPVGR.I | Y | 94.90 | 3033.3855 | 27 | 0.5 | 1012.1362 | 3 | 55.78 | 1 | 40894 | BuCaMH04.raw | 1.2675E7 | 1 | 1 | 441 | 467 |  |  | PEAKS DB |
| Y.VLADDSDFFQALDTK.T | Y | 93.09 | 1683.7991 | 15 | 0.7 | 842.9074 | 2 | 40.29 | 1 | 26943 | BuCaMH04.raw | 1.1718E8 | 1 | 1 | 390 | 404 |  |  | PEAKS DB |
| M.AYVLADDSDFFQALDTK.T | Y | 92.42 | 1917.8995 | 17 | -0.3 | 959.9568 | 2 | 46.01 | 1 | 32305 | BuCaMH04.raw | 5.6345E6 | 1 | 1 | 388 | 404 |  |  | PEAKS DB |
| S.FTPYQFQDYFESAAAPVGR.I | Y | 91.29 | 2193.0166 | 19 | 0.6 | 1097.5162 | 2 | 45.01 | 1 | 31430 | BuCaMH04.raw | 1.6354E7 | 2 | 2 | 449 | 467 |  |  | PEAKS DB |
| R.IYFEPPLPPK.K | N | 84.04 | 1199.6589 | 10 | 0.3 | 600.8369 | 2 | 25.08 | 1 | 14052 | BuCaMH04.raw | 8.9718E7 | 2 | 2 | 319 | 328 |  |  | PEAKS DB |
| R.FHGWIDSTIMTGLR.A | Y | 79.15 | 1632.8082 | 14 | 0.9 | 545.2772 | 3 | 34.16 | 1 | 21564 | BuCaMH04.raw | 5.0059E6 | 1 | 1 | 478 | 491 |  |  | PEAKS DB |
| F.TPYQFQDYFESAAAPVGR.I | Y | 79.08 | 2045.9482 | 18 | 0.7 | 1023.9821 | 2 | 37.99 | 1 | 25075 | BuCaMH04.raw | 4.1508E6 | 1 | 1 | 450 | 467 |  |  | PEAKS DB |
| A.DIVINDLSLIHDLPK.R | Y | 73.01 | 1703.9458 | 15 | -0.1 | 568.9891 | 3 | 41.18 | 1 | 27840 | BuCaMH04.raw | 5.8144E6 | 2 | 2 | 408 | 422 |  |  | PEAKS DB |
| Y.QFQDYFESAAAPVGR.I | Y | 72.55 | 1684.7844 | 15 | 0.6 | 843.4000 | 2 | 28.27 | 1 | 16233 | BuCaMH04.raw | 2.4355E7 | 2 | 2 | 453 | 467 |  |  | PEAKS DB |
| Q.FQDYFESAAAPVGR.I | Y | 71.07 | 1556.7258 | 14 | 1.0 | 779.3710 | 2 | 24.89 | 1 | 13101 | BuCaMH04.raw | 1.7333E6 | 1 | 1 | 454 | 467 |  |  | PEAKS DB |
| E.FFQENENAWYYINNIR.K | Y | 65.16 | 2119.9751 | 16 | -0.6 | 1060.9941 | 2 | 43.48 | 1 | 29962 | BuCaMH04.raw | 8.5243E5 | 1 | 1 | 131 | 146 |  |  | PEAKS DB |
| F.HGWIDSTIMTGLR.A | Y | 64.58 | 1485.7399 | 13 | 0.9 | 743.8779 | 2 | 28.60 | 1 | 16655 | BuCaMH04.raw | 9.0318E6 | 2 | 2 | 479 | 491 |  |  | PEAKS DB |
| S.ITSFTPYQFQDYFESAAAPVGR.I | Y | 64.21 | 2494.1804 | 22 | 0.5 | 1248.0981 | 2 | 49.09 | 1 | 35082 | BuCaMH04.raw | 6.3034E6 | 1 | 1 | 446 | 467 |  |  | PEAKS DB |
| H.GWIDSTIMTGLR.A | Y | 58.10 | 1348.6809 | 12 | 0.3 | 675.3480 | 2 | 41.65 | 1 | 28245 | BuCaMH04.raw | 7.7826E5 | 1 | 1 | 480 | 491 |  |  | PEAKS DB |
| A.YVLADDSDFFQALDTK.T | Y | 57.53 | 1846.8624 | 16 | 0.1 | 924.4385 | 2 | 44.71 | 1 | 31151 | BuCaMH04.raw | 2.4199E6 | 1 | 1 | 389 | 404 |  |  | PEAKS DB |
| A.DDSDFFQALDTK.T | Y | 57.09 | 1400.6095 | 12 | 0.3 | 701.3123 | 2 | 38.59 | 1 | 25553 | BuCaMH04.raw | 4.8806E6 | 1 | 1 | 393 | 404 |  |  | PEAKS DB |
| K.RFDEIVGGFDQLPIS.M | N | 52.08 | 1691.8518 | 15 | 0.2 | 846.9333 | 2 | 43.43 | 1 | 29880 | BuCaMH04.raw | 8.7751E5 | 1 | 1 | 251 | 265 |  |  | PEAKS DB |
| R.EGWYVNMGPMR.L | N | 52.03 | 1338.5850 | 11 | 0.3 | 670.3000 | 2 | 30.40 | 1 | 18128 | BuCaMH04.raw | 1.6917E6 | 1 | 1 | 99 | 109 |  |  | PEAKS DB |
| total 22 peptides |  |  |  |  |  |  |  |  |  |  |  |  |  |  |  |  |  |  |  |
| --- | --- | --- | --- | --- | --- | --- | --- | --- | --- | --- | --- | --- | --- | --- | --- | --- | --- | --- | --- |

A8QL58.2

back to list

  

| Protein Coverage
| Supporting Peptides
|

Protein Coverage:

Supporting Peptides:

| Peptide | Uniq | -10lgP | Mass | Length | ppm | m/z | z | RT | Fraction | Scan | Source File | Area BuCaMH04\_B15 | #Feature | #Feature BuCaMH04\_B15 | Start | End | PTM | AScore | Found By |
| --- | --- | --- | --- | --- | --- | --- | --- | --- | --- | --- | --- | --- | --- | --- | --- | --- | --- | --- | --- |
| K.TC(+57.02)ADIVINDLSLIHDLPK.R | Y | 123.10 | 2036.0612 | 18 | -0.1 | 1019.0377 | 2 | 43.44 | 1 | 29941 | BuCaMH04.raw | 2.6763E8 | 3 | 3 | 405 | 422 | Carbamidomethylation | C2:Carbamidomethylation:1000.00 | PEAKS DB |
| K.LNEFFQENENAWYYINNIR.K | Y | 107.89 | 2476.1448 | 19 | -0.3 | 1239.0793 | 2 | 51.15 | 1 | 36690 | BuCaMH04.raw | 5.8054E7 | 2 | 2 | 128 | 146 |  |  | PEAKS DB |
| K.HVVVVGAGMAGLSAAYVLAGAGHK.V | N | 106.63 | 2234.1992 | 24 | 0.7 | 745.7409 | 3 | 30.19 | 1 | 18119 | BuCaMH04.raw | 2.655E7 | 1 | 1 | 54 | 77 |  |  | PEAKS DB |
| K.TFVTADYVIVC(+57.02)STSR.A | Y | 96.20 | 1717.8345 | 15 | 0.1 | 859.9246 | 2 | 28.83 | 1 | 16853 | BuCaMH04.raw | 1.3866E8 | 2 | 2 | 300 | 314 | Carbamidomethylation | C11:Carbamidomethylation:1000.00 | PEAKS DB |
| K.YTMGSITSFTPYQFQDYFESAAAPVGR.I | Y | 94.90 | 3033.3855 | 27 | 0.5 | 1012.1362 | 3 | 55.78 | 1 | 40894 | BuCaMH04.raw | 1.2675E7 | 1 | 1 | 441 | 467 |  |  | PEAKS DB |
| Y.VLADDSDFFQALDTK.T | Y | 93.09 | 1683.7991 | 15 | 0.7 | 842.9074 | 2 | 40.29 | 1 | 26943 | BuCaMH04.raw | 1.1718E8 | 1 | 1 | 390 | 404 |  |  | PEAKS DB |
| M.AYVLADDSDFFQALDTK.T | Y | 92.42 | 1917.8995 | 17 | -0.3 | 959.9568 | 2 | 46.01 | 1 | 32305 | BuCaMH04.raw | 5.6345E6 | 1 | 1 | 388 | 404 |  |  | PEAKS DB |
| S.FTPYQFQDYFESAAAPVGR.I | Y | 91.29 | 2193.0166 | 19 | 0.6 | 1097.5162 | 2 | 45.01 | 1 | 31430 | BuCaMH04.raw | 1.6354E7 | 2 | 2 | 449 | 467 |  |  | PEAKS DB |
| R.IYFEPPLPPK.K | N | 84.04 | 1199.6589 | 10 | 0.3 | 600.8369 | 2 | 25.08 | 1 | 14052 | BuCaMH04.raw | 8.9718E7 | 2 | 2 | 319 | 328 |  |  | PEAKS DB |
| R.FHGWIDSTIMTGLR.A | Y | 79.15 | 1632.8082 | 14 | 0.9 | 545.2772 | 3 | 34.16 | 1 | 21564 | BuCaMH04.raw | 5.0059E6 | 1 | 1 | 478 | 491 |  |  | PEAKS DB |
| F.TPYQFQDYFESAAAPVGR.I | Y | 79.08 | 2045.9482 | 18 | 0.7 | 1023.9821 | 2 | 37.99 | 1 | 25075 | BuCaMH04.raw | 4.1508E6 | 1 | 1 | 450 | 467 |  |  | PEAKS DB |
| A.DIVINDLSLIHDLPK.R | Y | 73.01 | 1703.9458 | 15 | -0.1 | 568.9891 | 3 | 41.18 | 1 | 27840 | BuCaMH04.raw | 5.8144E6 | 2 | 2 | 408 | 422 |  |  | PEAKS DB |
| Y.QFQDYFESAAAPVGR.I | Y | 72.55 | 1684.7844 | 15 | 0.6 | 843.4000 | 2 | 28.27 | 1 | 16233 | BuCaMH04.raw | 2.4355E7 | 2 | 2 | 453 | 467 |  |  | PEAKS DB |
| Q.FQDYFESAAAPVGR.I | Y | 71.07 | 1556.7258 | 14 | 1.0 | 779.3710 | 2 | 24.89 | 1 | 13101 | BuCaMH04.raw | 1.7333E6 | 1 | 1 | 454 | 467 |  |  | PEAKS DB |
| E.FFQENENAWYYINNIR.K | Y | 65.16 | 2119.9751 | 16 | -0.6 | 1060.9941 | 2 | 43.48 | 1 | 29962 | BuCaMH04.raw | 8.5243E5 | 1 | 1 | 131 | 146 |  |  | PEAKS DB |
| F.HGWIDSTIMTGLR.A | Y | 64.58 | 1485.7399 | 13 | 0.9 | 743.8779 | 2 | 28.60 | 1 | 16655 | BuCaMH04.raw | 9.0318E6 | 2 | 2 | 479 | 491 |  |  | PEAKS DB |
| S.ITSFTPYQFQDYFESAAAPVGR.I | Y | 64.21 | 2494.1804 | 22 | 0.5 | 1248.0981 | 2 | 49.09 | 1 | 35082 | BuCaMH04.raw | 6.3034E6 | 1 | 1 | 446 | 467 |  |  | PEAKS DB |
| H.GWIDSTIMTGLR.A | Y | 58.10 | 1348.6809 | 12 | 0.3 | 675.3480 | 2 | 41.65 | 1 | 28245 | BuCaMH04.raw | 7.7826E5 | 1 | 1 | 480 | 491 |  |  | PEAKS DB |
| A.YVLADDSDFFQALDTK.T | Y | 57.53 | 1846.8624 | 16 | 0.1 | 924.4385 | 2 | 44.71 | 1 | 31151 | BuCaMH04.raw | 2.4199E6 | 1 | 1 | 389 | 404 |  |  | PEAKS DB |
| A.DDSDFFQALDTK.T | Y | 57.09 | 1400.6095 | 12 | 0.3 | 701.3123 | 2 | 38.59 | 1 | 25553 | BuCaMH04.raw | 4.8806E6 | 1 | 1 | 393 | 404 |  |  | PEAKS DB |
| K.RFDEIVGGFDQLPIS.M | N | 52.08 | 1691.8518 | 15 | 0.2 | 846.9333 | 2 | 43.43 | 1 | 29880 | BuCaMH04.raw | 8.7751E5 | 1 | 1 | 251 | 265 |  |  | PEAKS DB |
| R.EGWYVNMGPMR.L | N | 52.03 | 1338.5850 | 11 | 0.3 | 670.3000 | 2 | 30.40 | 1 | 18128 | BuCaMH04.raw | 1.6917E6 | 1 | 1 | 99 | 109 |  |  | PEAKS DB |
| total 22 peptides |  |  |  |  |  |  |  |  |  |  |  |  |  |  |  |  |  |  |  |
| --- | --- | --- | --- | --- | --- | --- | --- | --- | --- | --- | --- | --- | --- | --- | --- | --- | --- | --- | --- |

XP\_026581281.1

back to list

  

| Protein Coverage
| Supporting Peptides
|

Protein Coverage:

Supporting Peptides:

| Peptide | Uniq | -10lgP | Mass | Length | ppm | m/z | z | RT | Fraction | Scan | Source File | Area BuCaMH04\_B15 | #Feature | #Feature BuCaMH04\_B15 | Start | End | PTM | AScore | Found By |
| --- | --- | --- | --- | --- | --- | --- | --- | --- | --- | --- | --- | --- | --- | --- | --- | --- | --- | --- | --- |
| R.FLTYTQNVILVSLSYR.V | N | 114.43 | 1916.0408 | 16 | -0.1 | 959.0276 | 2 | 48.85 | 1 | 34834 | BuCaMH04.raw | 2.6107E7 | 2 | 2 | 165 | 180 |  |  | PEAKS DB |
| R.AILQSGGPNAPWATVTPAESR.R | N | 106.82 | 2122.0806 | 21 | 0.3 | 1062.0479 | 2 | 26.94 | 1 | 15006 | BuCaMH04.raw | 8.0499E8 | 2 | 2 | 253 | 273 |  |  | PEAKS DB |
| K.DEGSYFLIYGLPGFSK.D | N | 103.69 | 1791.8718 | 16 | 0.7 | 896.9438 | 2 | 56.99 | 1 | 42168 | BuCaMH04.raw | 1.8962E8 | 3 | 3 | 357 | 372 |  |  | PEAKS DB |
| S.AVTIFGESAGAASVGMHLLSTQSR.G | N | 101.28 | 2389.2061 | 24 | 1.2 | 797.4103 | 3 | 39.94 | 1 | 26891 | BuCaMH04.raw | 1.4086E8 | 1 | 1 | 224 | 247 |  |  | PEAKS DB |
| E.LIDEEWSVLPYK.S | N | 89.30 | 1490.7656 | 12 | 0.9 | 746.3907 | 2 | 37.26 | 1 | 24455 | BuCaMH04.raw | 2.1123E7 | 1 | 1 | 305 | 316 |  |  | PEAKS DB |
| W.IYGGGFYSGAASLDVYDGR.F | N | 88.75 | 1966.9060 | 19 | 0.5 | 984.4608 | 2 | 34.51 | 1 | 21942 | BuCaMH04.raw | 6.0601E6 | 1 | 1 | 146 | 164 |  |  | PEAKS DB |
| S.PQELIDEEWSVLPYK.S | N | 79.86 | 1844.9196 | 15 | 0.6 | 923.4677 | 2 | 41.67 | 1 | 28438 | BuCaMH04.raw | 7.64E6 | 1 | 1 | 302 | 316 |  |  | PEAKS DB |
| I.C(+57.02)AFWNHFLPK.L | N | 67.68 | 1318.6281 | 10 | 0.2 | 440.5500 | 3 | 27.24 | 1 | 15136 | BuCaMH04.raw | 4.6147E6 | 1 | 1 | 552 | 561 | Carbamidomethylation | C1:Carbamidomethylation:1000.00 | PEAKS DB |
| R.ASNLLWPPWMGVPH.G | N | 61.66 | 1603.7969 | 14 | 1.3 | 802.9067 | 2 | 53.12 | 1 | 38555 | BuCaMH04.raw | 1.3595E6 | 1 | 1 | 458 | 471 |  |  | PEAKS DB |
| K.QLGC(+57.02)HFNNDSELVSC(+57.02)LR.S | N | 61.24 | 2047.9204 | 17 | 1.0 | 683.6481 | 3 | 20.07 | 1 | 8865 | BuCaMH04.raw | 2.0907E7 | 1 | 1 | 282 | 298 | Carbamidomethylation | C4:Carbamidomethylation:1000.00;C15:Carbamidomethylation:1000.00 | PEAKS DB |
| R.GLSLPVLDGHVSAFLGVPFAEPPVGR.M | N | 61.21 | 2630.4219 | 26 | 0.8 | 877.8153 | 3 | 52.84 | 1 | 38141 | BuCaMH04.raw | 1.2925E7 | 1 | 1 | 48 | 73 |  |  | PEAKS DB |
| R.AQIC(+57.02)AFWNHFLPK.L | N | 60.48 | 1630.8079 | 13 | 1.0 | 544.6104 | 3 | 36.94 | 1 | 23938 | BuCaMH04.raw | 1.8602E6 | 1 | 1 | 549 | 561 | Carbamidomethylation | C4:Carbamidomethylation:1000.00 | PEAKS DB |
| L.IDEEWSVLPYK.S | N | 57.66 | 1377.6816 | 11 | 0.6 | 689.8485 | 2 | 33.06 | 1 | 20610 | BuCaMH04.raw | 2.6958E6 | 1 | 1 | 306 | 316 |  |  | PEAKS DB |
| A.ILQSGGPNAPWATVTPAESR.R | N | 56.43 | 2051.0435 | 20 | 2.0 | 1026.5310 | 2 | 24.16 | 1 | 12452 | BuCaMH04.raw | 1.9229E6 | 1 | 1 | 254 | 273 |  |  | PEAKS DB |
| T.IFGESAGAASVGMHLLSTQSR.G | N | 54.19 | 2118.0527 | 21 | 0.7 | 707.0253 | 3 | 27.10 | 1 | 15139 | BuCaMH04.raw | 7.8883E6 | 1 | 1 | 227 | 247 |  |  | PEAKS DB |
| Q.ELIDEEWSVLPYK.S | N | 51.47 | 1619.8082 | 13 | 0.7 | 810.9119 | 2 | 42.09 | 1 | 28660 | BuCaMH04.raw | 4.6799E5 | 1 | 1 | 304 | 316 |  |  | PEAKS DB |
| L.IYGLPGFSK.D | N | 51.29 | 980.5331 | 9 | 0.8 | 491.2742 | 2 | 27.30 | 1 | 15385 | BuCaMH04.raw | 4.7137E6 | 1 | 1 | 364 | 372 |  |  | PEAKS DB |
| total 17 peptides |  |  |  |  |  |  |  |  |  |  |  |  |  |  |  |  |  |  |  |
| --- | --- | --- | --- | --- | --- | --- | --- | --- | --- | --- | --- | --- | --- | --- | --- | --- | --- | --- | --- |

ABN72539.1

back to list

  

| Protein Coverage
| Supporting Peptides
|

Protein Coverage:

Supporting Peptides:

| Peptide | Uniq | -10lgP | Mass | Length | ppm | m/z | z | RT | Fraction | Scan | Source File | Area BuCaMH04\_B15 | #Feature | #Feature BuCaMH04\_B15 | Start | End | PTM | AScore | Found By |
| --- | --- | --- | --- | --- | --- | --- | --- | --- | --- | --- | --- | --- | --- | --- | --- | --- | --- | --- | --- |
| K.TLSYVTADYVIVC(+57.02)ATSR.A | N | 99.22 | 1917.9506 | 17 | 0.5 | 959.9830 | 2 | 37.85 | 1 | 25014 | BuCaMH04.raw | 4.4152E7 | 2 | 2 | 300 | 316 | Carbamidomethylation | C13:Carbamidomethylation:1000.00 | PEAKS DB |
| K.LNEFFQENENAWYFIR.N | N | 98.32 | 2118.9800 | 16 | 0.4 | 1060.4977 | 2 | 49.95 | 1 | 35860 | BuCaMH04.raw | 1.7944E7 | 1 | 1 | 128 | 143 |  |  | PEAKS DB |
| K.TSADIVINDLSLIHQLPK.N | N | 98.11 | 1976.0942 | 18 | 0.9 | 659.7059 | 3 | 39.96 | 1 | 27043 | BuCaMH04.raw | 9.5523E7 | 2 | 2 | 407 | 424 |  |  | PEAKS DB |
| L.TSFTPYQFQDYSETVAAPVGR.I | Y | 94.35 | 2363.1069 | 21 | 0.0 | 1182.5608 | 2 | 34.46 | 1 | 21913 | BuCaMH04.raw | 2.9292E6 | 1 | 1 | 450 | 470 |  |  | PEAKS DB |
| R.EADYEEFLEIAR.N | N | 92.74 | 1483.6830 | 12 | 1.0 | 742.8495 | 2 | 40.39 | 1 | 27344 | BuCaMH04.raw | 8.6584E7 | 1 | 1 | 32 | 43 |  |  | PEAKS DB |
| Y.VLADDSDFFQALDIK.T | N | 90.69 | 1695.8354 | 15 | 0.3 | 848.9252 | 2 | 47.62 | 1 | 33725 | BuCaMH04.raw | 3.6219E7 | 1 | 1 | 392 | 406 |  |  | PEAKS DB |
| R.FDEISGGFDQLPK.S | Y | 88.02 | 1451.6932 | 13 | 0.8 | 726.8545 | 2 | 27.17 | 1 | 15138 | BuCaMH04.raw | 8.861E7 | 1 | 1 | 252 | 264 |  |  | PEAKS DB |
| L.NEFFQENENAWYFIR.N | N | 85.39 | 2005.8959 | 15 | 0.0 | 1003.9552 | 2 | 47.08 | 1 | 33265 | BuCaMH04.raw | 1.6334E7 | 1 | 1 | 129 | 143 |  |  | PEAKS DB |
| K.RFDEISGGFDQLPK.S | Y | 84.94 | 1607.7943 | 14 | -0.1 | 536.9387 | 3 | 22.80 | 1 | 11254 | BuCaMH04.raw | 4.8498E7 | 2 | 2 | 251 | 264 |  |  | PEAKS DB |
| E.ADYEEFLEIAR.N | N | 74.83 | 1354.6404 | 11 | 0.3 | 678.3276 | 2 | 38.08 | 1 | 25194 | BuCaMH04.raw | 3.5897E7 | 1 | 1 | 33 | 43 |  |  | PEAKS DB |
| A.DIVINDLSLIHQLPK.N | N | 69.37 | 1716.9774 | 15 | 0.4 | 859.4963 | 2 | 37.46 | 1 | 24584 | BuCaMH04.raw | 4.3779E7 | 2 | 2 | 410 | 424 |  |  | PEAKS DB |
| A.DYEEFLEIAR.N | N | 65.68 | 1283.6033 | 10 | 0.6 | 642.8093 | 2 | 40.14 | 1 | 26895 | BuCaMH04.raw | 2.3334E6 | 1 | 1 | 34 | 43 |  |  | PEAKS DB |
| A.DDSDFFQALDIK.T | N | 62.96 | 1412.6459 | 12 | -0.6 | 707.3298 | 2 | 47.61 | 1 | 33856 | BuCaMH04.raw | 1.8992E6 | 1 | 1 | 395 | 406 |  |  | PEAKS DB |
| D.YEEFLEIAR.N | N | 59.77 | 1168.5764 | 9 | 0.4 | 585.2957 | 2 | 26.94 | 1 | 14950 | BuCaMH04.raw | 2.8615E6 | 1 | 1 | 35 | 43 |  |  | PEAKS DB |
| S.FTPYQFQDYSETVAAPVGR.I | Y | 52.88 | 2175.0273 | 19 | -1.4 | 1088.5194 | 2 | 33.14 | 1 | 20696 | BuCaMH04.raw | 8.2205E5 | 1 | 1 | 452 | 470 |  |  | PEAKS DB |
| K.HVVVVGAGMAGLSAAYVLEK.A | Y | 52.79 | 1970.0659 | 20 | 0.9 | 657.6965 | 3 | 30.92 | 1 | 18572 | BuCaMH04.raw | 7.0763E6 | 1 | 1 | 54 | 73 |  |  | PEAKS DB |
| K.EGWYVNMGPMR.L | N | 52.03 | 1338.5850 | 11 | 0.3 | 670.3000 | 2 | 30.40 | 1 | 18128 | BuCaMH04.raw | 1.6917E6 | 1 | 1 | 99 | 109 |  |  | PEAKS DB |
| total 17 peptides |  |  |  |  |  |  |  |  |  |  |  |  |  |  |  |  |  |  |  |
| --- | --- | --- | --- | --- | --- | --- | --- | --- | --- | --- | --- | --- | --- | --- | --- | --- | --- | --- | --- |

A8QL51.1

back to list

  

| Protein Coverage
| Supporting Peptides
|

Protein Coverage:

Supporting Peptides:

| Peptide | Uniq | -10lgP | Mass | Length | ppm | m/z | z | RT | Fraction | Scan | Source File | Area BuCaMH04\_B15 | #Feature | #Feature BuCaMH04\_B15 | Start | End | PTM | AScore | Found By |
| --- | --- | --- | --- | --- | --- | --- | --- | --- | --- | --- | --- | --- | --- | --- | --- | --- | --- | --- | --- |
| K.TLSYVTADYVIVC(+57.02)ATSR.A | N | 99.22 | 1917.9506 | 17 | 0.5 | 959.9830 | 2 | 37.85 | 1 | 25014 | BuCaMH04.raw | 4.4152E7 | 2 | 2 | 300 | 316 | Carbamidomethylation | C13:Carbamidomethylation:1000.00 | PEAKS DB |
| K.LNEFFQENENAWYFIR.N | N | 98.32 | 2118.9800 | 16 | 0.4 | 1060.4977 | 2 | 49.95 | 1 | 35860 | BuCaMH04.raw | 1.7944E7 | 1 | 1 | 128 | 143 |  |  | PEAKS DB |
| K.TSADIVINDLSLIHQLPK.N | N | 98.11 | 1976.0942 | 18 | 0.9 | 659.7059 | 3 | 39.96 | 1 | 27043 | BuCaMH04.raw | 9.5523E7 | 2 | 2 | 407 | 424 |  |  | PEAKS DB |
| L.TSFTPYQFQDYSETVAAPVGR.I | Y | 94.35 | 2363.1069 | 21 | 0.0 | 1182.5608 | 2 | 34.46 | 1 | 21913 | BuCaMH04.raw | 2.9292E6 | 1 | 1 | 450 | 470 |  |  | PEAKS DB |
| R.EADYEEFLEIAR.N | N | 92.74 | 1483.6830 | 12 | 1.0 | 742.8495 | 2 | 40.39 | 1 | 27344 | BuCaMH04.raw | 8.6584E7 | 1 | 1 | 32 | 43 |  |  | PEAKS DB |
| Y.VLADDSDFFQALDIK.T | N | 90.69 | 1695.8354 | 15 | 0.3 | 848.9252 | 2 | 47.62 | 1 | 33725 | BuCaMH04.raw | 3.6219E7 | 1 | 1 | 392 | 406 |  |  | PEAKS DB |
| R.FDEISGGFDQLPK.S | Y | 88.02 | 1451.6932 | 13 | 0.8 | 726.8545 | 2 | 27.17 | 1 | 15138 | BuCaMH04.raw | 8.861E7 | 1 | 1 | 252 | 264 |  |  | PEAKS DB |
| L.NEFFQENENAWYFIR.N | N | 85.39 | 2005.8959 | 15 | 0.0 | 1003.9552 | 2 | 47.08 | 1 | 33265 | BuCaMH04.raw | 1.6334E7 | 1 | 1 | 129 | 143 |  |  | PEAKS DB |
| K.RFDEISGGFDQLPK.S | Y | 84.94 | 1607.7943 | 14 | -0.1 | 536.9387 | 3 | 22.80 | 1 | 11254 | BuCaMH04.raw | 4.8498E7 | 2 | 2 | 251 | 264 |  |  | PEAKS DB |
| E.ADYEEFLEIAR.N | N | 74.83 | 1354.6404 | 11 | 0.3 | 678.3276 | 2 | 38.08 | 1 | 25194 | BuCaMH04.raw | 3.5897E7 | 1 | 1 | 33 | 43 |  |  | PEAKS DB |
| A.DIVINDLSLIHQLPK.N | N | 69.37 | 1716.9774 | 15 | 0.4 | 859.4963 | 2 | 37.46 | 1 | 24584 | BuCaMH04.raw | 4.3779E7 | 2 | 2 | 410 | 424 |  |  | PEAKS DB |
| A.DYEEFLEIAR.N | N | 65.68 | 1283.6033 | 10 | 0.6 | 642.8093 | 2 | 40.14 | 1 | 26895 | BuCaMH04.raw | 2.3334E6 | 1 | 1 | 34 | 43 |  |  | PEAKS DB |
| A.DDSDFFQALDIK.T | N | 62.96 | 1412.6459 | 12 | -0.6 | 707.3298 | 2 | 47.61 | 1 | 33856 | BuCaMH04.raw | 1.8992E6 | 1 | 1 | 395 | 406 |  |  | PEAKS DB |
| D.YEEFLEIAR.N | N | 59.77 | 1168.5764 | 9 | 0.4 | 585.2957 | 2 | 26.94 | 1 | 14950 | BuCaMH04.raw | 2.8615E6 | 1 | 1 | 35 | 43 |  |  | PEAKS DB |
| S.FTPYQFQDYSETVAAPVGR.I | Y | 52.88 | 2175.0273 | 19 | -1.4 | 1088.5194 | 2 | 33.14 | 1 | 20696 | BuCaMH04.raw | 8.2205E5 | 1 | 1 | 452 | 470 |  |  | PEAKS DB |
| K.HVVVVGAGMAGLSAAYVLEK.A | Y | 52.79 | 1970.0659 | 20 | 0.9 | 657.6965 | 3 | 30.92 | 1 | 18572 | BuCaMH04.raw | 7.0763E6 | 1 | 1 | 54 | 73 |  |  | PEAKS DB |
| K.EGWYVNMGPMR.L | N | 52.03 | 1338.5850 | 11 | 0.3 | 670.3000 | 2 | 30.40 | 1 | 18128 | BuCaMH04.raw | 1.6917E6 | 1 | 1 | 99 | 109 |  |  | PEAKS DB |
| total 17 peptides |  |  |  |  |  |  |  |  |  |  |  |  |  |  |  |  |  |  |  |
| --- | --- | --- | --- | --- | --- | --- | --- | --- | --- | --- | --- | --- | --- | --- | --- | --- | --- | --- | --- |

JAA74986.1

back to list

  

| Protein Coverage
| Supporting Peptides
|

Protein Coverage:

Supporting Peptides:

| Peptide | Uniq | -10lgP | Mass | Length | ppm | m/z | z | RT | Fraction | Scan | Source File | Area BuCaMH04\_B15 | #Feature | #Feature BuCaMH04\_B15 | Start | End | PTM | AScore | Found By |
| --- | --- | --- | --- | --- | --- | --- | --- | --- | --- | --- | --- | --- | --- | --- | --- | --- | --- | --- | --- |
| R.FLTYTQNVILVSLSYR.V | N | 114.43 | 1916.0408 | 16 | -0.1 | 959.0276 | 2 | 48.85 | 1 | 34834 | BuCaMH04.raw | 2.6107E7 | 2 | 2 | 165 | 180 |  |  | PEAKS DB |
| R.AILQSGGPNAPWATVTPAESR.R | N | 106.82 | 2122.0806 | 21 | 0.3 | 1062.0479 | 2 | 26.94 | 1 | 15006 | BuCaMH04.raw | 8.0499E8 | 2 | 2 | 253 | 273 |  |  | PEAKS DB |
| K.DEGSYFLIYGLPGFSK.D | N | 103.69 | 1791.8718 | 16 | 0.7 | 896.9438 | 2 | 56.99 | 1 | 42168 | BuCaMH04.raw | 1.8962E8 | 3 | 3 | 357 | 372 |  |  | PEAKS DB |
| E.LIDEEWSVLPYK.S | N | 89.30 | 1490.7656 | 12 | 0.9 | 746.3907 | 2 | 37.26 | 1 | 24455 | BuCaMH04.raw | 2.1123E7 | 1 | 1 | 305 | 316 |  |  | PEAKS DB |
| W.IYGGGFYSGAASLDVYDGR.F | N | 88.75 | 1966.9060 | 19 | 0.5 | 984.4608 | 2 | 34.51 | 1 | 21942 | BuCaMH04.raw | 6.0601E6 | 1 | 1 | 146 | 164 |  |  | PEAKS DB |
| T.PQELIDEEWSVLPYK.S | N | 79.86 | 1844.9196 | 15 | 0.6 | 923.4677 | 2 | 41.67 | 1 | 28438 | BuCaMH04.raw | 7.64E6 | 1 | 1 | 302 | 316 |  |  | PEAKS DB |
| M.DDIVGDHNVIC(+57.02)PVVQFANDYAK.R | N | 72.30 | 2488.1692 | 22 | 0.4 | 830.3973 | 3 | 39.09 | 1 | 25991 | BuCaMH04.raw | 1.3183E7 | 1 | 1 | 423 | 444 | Carbamidomethylation | C11:Carbamidomethylation:1000.00 | PEAKS DB |
| R.ASNLLWPPWMGVPH.G | N | 61.66 | 1603.7969 | 14 | 1.3 | 802.9067 | 2 | 53.12 | 1 | 38555 | BuCaMH04.raw | 1.3595E6 | 1 | 1 | 458 | 471 |  |  | PEAKS DB |
| R.GLSLPVLDGHVSAFLGVPFAEPPVGR.M | N | 61.21 | 2630.4219 | 26 | 0.8 | 877.8153 | 3 | 52.84 | 1 | 38141 | BuCaMH04.raw | 1.2925E7 | 1 | 1 | 48 | 73 |  |  | PEAKS DB |
| L.IDEEWSVLPYK.S | N | 57.66 | 1377.6816 | 11 | 0.6 | 689.8485 | 2 | 33.06 | 1 | 20610 | BuCaMH04.raw | 2.6958E6 | 1 | 1 | 306 | 316 |  |  | PEAKS DB |
| A.ILQSGGPNAPWATVTPAESR.R | N | 56.43 | 2051.0435 | 20 | 2.0 | 1026.5310 | 2 | 24.16 | 1 | 12452 | BuCaMH04.raw | 1.9229E6 | 1 | 1 | 254 | 273 |  |  | PEAKS DB |
| Q.ELIDEEWSVLPYK.S | N | 51.47 | 1619.8082 | 13 | 0.7 | 810.9119 | 2 | 42.09 | 1 | 28660 | BuCaMH04.raw | 4.6799E5 | 1 | 1 | 304 | 316 |  |  | PEAKS DB |
| L.IYGLPGFSK.D | N | 51.29 | 980.5331 | 9 | 0.8 | 491.2742 | 2 | 27.30 | 1 | 15385 | BuCaMH04.raw | 4.7137E6 | 1 | 1 | 364 | 372 |  |  | PEAKS DB |
| total 13 peptides |  |  |  |  |  |  |  |  |  |  |  |  |  |  |  |  |  |  |  |
| --- | --- | --- | --- | --- | --- | --- | --- | --- | --- | --- | --- | --- | --- | --- | --- | --- | --- | --- | --- |

T\_101

back to list

  

| Protein Coverage
| Supporting Peptides
|

Protein Coverage:

Supporting Peptides:

| Peptide | Uniq | -10lgP | Mass | Length | ppm | m/z | z | RT | Fraction | Scan | Source File | Area BuCaMH04\_B15 | #Feature | #Feature BuCaMH04\_B15 | Start | End | PTM | AScore | Found By |
| --- | --- | --- | --- | --- | --- | --- | --- | --- | --- | --- | --- | --- | --- | --- | --- | --- | --- | --- | --- |
| K.TLSYVTADYVIVC(+57.02)ATSR.A | N | 99.22 | 1917.9506 | 17 | 0.5 | 959.9830 | 2 | 37.85 | 1 | 25014 | BuCaMH04.raw | 4.4152E7 | 2 | 2 | 300 | 316 | Carbamidomethylation | C13:Carbamidomethylation:1000.00 | PEAKS DB |
| K.LNEFFQENENAWYFIR.N | N | 98.32 | 2118.9800 | 16 | 0.4 | 1060.4977 | 2 | 49.95 | 1 | 35860 | BuCaMH04.raw | 1.7944E7 | 1 | 1 | 128 | 143 |  |  | PEAKS DB |
| K.TSADIVINDLSLIHQLPK.N | N | 98.11 | 1976.0942 | 18 | 0.9 | 659.7059 | 3 | 39.96 | 1 | 27043 | BuCaMH04.raw | 9.5523E7 | 2 | 2 | 407 | 424 |  |  | PEAKS DB |
| R.EADYEEFLEIAR.N | N | 92.74 | 1483.6830 | 12 | 1.0 | 742.8495 | 2 | 40.39 | 1 | 27344 | BuCaMH04.raw | 8.6584E7 | 1 | 1 | 32 | 43 |  |  | PEAKS DB |
| Y.VLADDSDFFQALDIK.T | N | 90.69 | 1695.8354 | 15 | 0.3 | 848.9252 | 2 | 47.62 | 1 | 33725 | BuCaMH04.raw | 3.6219E7 | 1 | 1 | 392 | 406 |  |  | PEAKS DB |
| L.NEFFQENENAWYFIR.N | N | 85.39 | 2005.8959 | 15 | 0.0 | 1003.9552 | 2 | 47.08 | 1 | 33265 | BuCaMH04.raw | 1.6334E7 | 1 | 1 | 129 | 143 |  |  | PEAKS DB |
| R.ISFEPPLPPK.K | N | 82.29 | 1123.6277 | 10 | 0.5 | 562.8214 | 2 | 22.87 | 1 | 11505 | BuCaMH04.raw | 1.674E7 | 4 | 4 | 321 | 330 |  |  | PEAKS DB |
| E.ADYEEFLEIAR.N | N | 74.83 | 1354.6404 | 11 | 0.3 | 678.3276 | 2 | 38.08 | 1 | 25194 | BuCaMH04.raw | 3.5897E7 | 1 | 1 | 33 | 43 |  |  | PEAKS DB |
| K.NEIQDLC(+57.02)YPSLIK.K | Y | 74.52 | 1591.7915 | 13 | 1.4 | 796.9042 | 2 | 31.13 | 1 | 19005 | BuCaMH04.raw | 3.9483E7 | 1 | 1 | 425 | 437 | Carbamidomethylation | C7:Carbamidomethylation:1000.00 | PEAKS DB |
| A.DIVINDLSLIHQLPK.N | N | 69.37 | 1716.9774 | 15 | 0.4 | 859.4963 | 2 | 37.46 | 1 | 24584 | BuCaMH04.raw | 4.3779E7 | 2 | 2 | 410 | 424 |  |  | PEAKS DB |
| A.DYEEFLEIAR.N | N | 65.68 | 1283.6033 | 10 | 0.6 | 642.8093 | 2 | 40.14 | 1 | 26895 | BuCaMH04.raw | 2.3334E6 | 1 | 1 | 34 | 43 |  |  | PEAKS DB |
| A.DDSDFFQALDIK.T | N | 62.96 | 1412.6459 | 12 | -0.6 | 707.3298 | 2 | 47.61 | 1 | 33856 | BuCaMH04.raw | 1.8992E6 | 1 | 1 | 395 | 406 |  |  | PEAKS DB |
| D.YEEFLEIAR.N | N | 59.77 | 1168.5764 | 9 | 0.4 | 585.2957 | 2 | 26.94 | 1 | 14950 | BuCaMH04.raw | 2.8615E6 | 1 | 1 | 35 | 43 |  |  | PEAKS DB |
| Y.QFQDYSETFAAPVGR.I | Y | 57.11 | 1714.7950 | 15 | -2.7 | 858.4025 | 2 | 24.51 | 1 | 12799 | BuCaMH04.raw | 1.3171E6 | 1 | 1 | 456 | 470 |  |  | PEAKS DB |
| K.EGWYVNMGPMR.L | N | 52.03 | 1338.5850 | 11 | 0.3 | 670.3000 | 2 | 30.40 | 1 | 18128 | BuCaMH04.raw | 1.6917E6 | 1 | 1 | 99 | 109 |  |  | PEAKS DB |
| total 15 peptides |  |  |  |  |  |  |  |  |  |  |  |  |  |  |  |  |  |  |  |
| --- | --- | --- | --- | --- | --- | --- | --- | --- | --- | --- | --- | --- | --- | --- | --- | --- | --- | --- | --- |

T\_95

back to list

  

| Protein Coverage
| Supporting Peptides
|

Protein Coverage:

Supporting Peptides:

| Peptide | Uniq | -10lgP | Mass | Length | ppm | m/z | z | RT | Fraction | Scan | Source File | Area BuCaMH04\_B15 | #Feature | #Feature BuCaMH04\_B15 | Start | End | PTM | AScore | Found By |
| --- | --- | --- | --- | --- | --- | --- | --- | --- | --- | --- | --- | --- | --- | --- | --- | --- | --- | --- | --- |
| K.TLSYVTADYVIVC(+57.02)ATSR.A | N | 99.22 | 1917.9506 | 17 | 0.5 | 959.9830 | 2 | 37.85 | 1 | 25014 | BuCaMH04.raw | 4.4152E7 | 2 | 2 | 367 | 383 | Carbamidomethylation | C13:Carbamidomethylation:1000.00 | PEAKS DB |
| K.LNEFFQENENAWYFIR.N | N | 98.32 | 2118.9800 | 16 | 0.4 | 1060.4977 | 2 | 49.95 | 1 | 35860 | BuCaMH04.raw | 1.7944E7 | 1 | 1 | 195 | 210 |  |  | PEAKS DB |
| K.TSADIVINDLSLIHQLPK.N | N | 98.11 | 1976.0942 | 18 | 0.9 | 659.7059 | 3 | 39.96 | 1 | 27043 | BuCaMH04.raw | 9.5523E7 | 2 | 2 | 474 | 491 |  |  | PEAKS DB |
| R.EADYEEFLEIAR.N | N | 92.74 | 1483.6830 | 12 | 1.0 | 742.8495 | 2 | 40.39 | 1 | 27344 | BuCaMH04.raw | 8.6584E7 | 1 | 1 | 99 | 110 |  |  | PEAKS DB |
| Y.VLADDSDFFQALDIK.T | N | 90.69 | 1695.8354 | 15 | 0.3 | 848.9252 | 2 | 47.62 | 1 | 33725 | BuCaMH04.raw | 3.6219E7 | 1 | 1 | 459 | 473 |  |  | PEAKS DB |
| L.NEFFQENENAWYFIR.N | N | 85.39 | 2005.8959 | 15 | 0.0 | 1003.9552 | 2 | 47.08 | 1 | 33265 | BuCaMH04.raw | 1.6334E7 | 1 | 1 | 196 | 210 |  |  | PEAKS DB |
| R.ISFEPPLPPK.K | N | 82.29 | 1123.6277 | 10 | 0.5 | 562.8214 | 2 | 22.87 | 1 | 11505 | BuCaMH04.raw | 1.674E7 | 4 | 4 | 388 | 397 |  |  | PEAKS DB |
| E.ADYEEFLEIAR.N | N | 74.83 | 1354.6404 | 11 | 0.3 | 678.3276 | 2 | 38.08 | 1 | 25194 | BuCaMH04.raw | 3.5897E7 | 1 | 1 | 100 | 110 |  |  | PEAKS DB |
| K.NEIQDLC(+57.02)YPSLIK.K | Y | 74.52 | 1591.7915 | 13 | 1.4 | 796.9042 | 2 | 31.13 | 1 | 19005 | BuCaMH04.raw | 3.9483E7 | 1 | 1 | 492 | 504 | Carbamidomethylation | C7:Carbamidomethylation:1000.00 | PEAKS DB |
| A.DIVINDLSLIHQLPK.N | N | 69.37 | 1716.9774 | 15 | 0.4 | 859.4963 | 2 | 37.46 | 1 | 24584 | BuCaMH04.raw | 4.3779E7 | 2 | 2 | 477 | 491 |  |  | PEAKS DB |
| A.DYEEFLEIAR.N | N | 65.68 | 1283.6033 | 10 | 0.6 | 642.8093 | 2 | 40.14 | 1 | 26895 | BuCaMH04.raw | 2.3334E6 | 1 | 1 | 101 | 110 |  |  | PEAKS DB |
| A.DDSDFFQALDIK.T | N | 62.96 | 1412.6459 | 12 | -0.6 | 707.3298 | 2 | 47.61 | 1 | 33856 | BuCaMH04.raw | 1.8992E6 | 1 | 1 | 462 | 473 |  |  | PEAKS DB |
| D.YEEFLEIAR.N | N | 59.77 | 1168.5764 | 9 | 0.4 | 585.2957 | 2 | 26.94 | 1 | 14950 | BuCaMH04.raw | 2.8615E6 | 1 | 1 | 102 | 110 |  |  | PEAKS DB |
| Y.QFQDYSETFAAPVGR.I | Y | 57.11 | 1714.7950 | 15 | -2.7 | 858.4025 | 2 | 24.51 | 1 | 12799 | BuCaMH04.raw | 1.3171E6 | 1 | 1 | 523 | 537 |  |  | PEAKS DB |
| K.EGWYVNMGPMR.L | N | 52.03 | 1338.5850 | 11 | 0.3 | 670.3000 | 2 | 30.40 | 1 | 18128 | BuCaMH04.raw | 1.6917E6 | 1 | 1 | 166 | 176 |  |  | PEAKS DB |
| total 15 peptides |  |  |  |  |  |  |  |  |  |  |  |  |  |  |  |  |  |  |  |
| --- | --- | --- | --- | --- | --- | --- | --- | --- | --- | --- | --- | --- | --- | --- | --- | --- | --- | --- | --- |

AAY89680.1

back to list

  

| Protein Coverage
| Supporting Peptides
|

Protein Coverage:

Supporting Peptides:

| Peptide | Uniq | -10lgP | Mass | Length | ppm | m/z | z | RT | Fraction | Scan | Source File | Area BuCaMH04\_B15 | #Feature | #Feature BuCaMH04\_B15 | Start | End | PTM | AScore | Found By |
| --- | --- | --- | --- | --- | --- | --- | --- | --- | --- | --- | --- | --- | --- | --- | --- | --- | --- | --- | --- |
| K.HVVVVGAGMAGLSAAYVLAGAGHK.V | N | 106.63 | 2234.1992 | 24 | 0.7 | 745.7409 | 3 | 30.19 | 1 | 18119 | BuCaMH04.raw | 2.655E7 | 1 | 1 | 54 | 77 |  |  | PEAKS DB |
| K.LNEFFQENENAWYFIR.N | N | 98.32 | 2118.9800 | 16 | 0.4 | 1060.4977 | 2 | 49.95 | 1 | 35860 | BuCaMH04.raw | 1.7944E7 | 1 | 1 | 128 | 143 |  |  | PEAKS DB |
| K.TSADIVINDLSLIHQLPK.K | N | 98.11 | 1976.0942 | 18 | 0.9 | 659.7059 | 3 | 39.96 | 1 | 27043 | BuCaMH04.raw | 9.5523E7 | 2 | 2 | 407 | 424 |  |  | PEAKS DB |
| R.EADYEEFLEIAR.N | N | 92.74 | 1483.6830 | 12 | 1.0 | 742.8495 | 2 | 40.39 | 1 | 27344 | BuCaMH04.raw | 8.6584E7 | 1 | 1 | 32 | 43 |  |  | PEAKS DB |
| L.NEFFQENENAWYFIR.N | N | 85.39 | 2005.8959 | 15 | 0.0 | 1003.9552 | 2 | 47.08 | 1 | 33265 | BuCaMH04.raw | 1.6334E7 | 1 | 1 | 129 | 143 |  |  | PEAKS DB |
| R.IYFEPPLPPK.K | N | 84.04 | 1199.6589 | 10 | 0.3 | 600.8369 | 2 | 25.08 | 1 | 14052 | BuCaMH04.raw | 8.9718E7 | 2 | 2 | 321 | 330 |  |  | PEAKS DB |
| E.ADYEEFLEIAR.N | N | 74.83 | 1354.6404 | 11 | 0.3 | 678.3276 | 2 | 38.08 | 1 | 25194 | BuCaMH04.raw | 3.5897E7 | 1 | 1 | 33 | 43 |  |  | PEAKS DB |
| A.DIVINDLSLIHQLPK.K | N | 69.37 | 1716.9774 | 15 | 0.4 | 859.4963 | 2 | 37.46 | 1 | 24584 | BuCaMH04.raw | 4.3779E7 | 2 | 2 | 410 | 424 |  |  | PEAKS DB |
| A.DYEEFLEIAR.N | N | 65.68 | 1283.6033 | 10 | 0.6 | 642.8093 | 2 | 40.14 | 1 | 26895 | BuCaMH04.raw | 2.3334E6 | 1 | 1 | 34 | 43 |  |  | PEAKS DB |
| D.YEEFLEIAR.N | N | 59.77 | 1168.5764 | 9 | 0.4 | 585.2957 | 2 | 26.94 | 1 | 14950 | BuCaMH04.raw | 2.8615E6 | 1 | 1 | 35 | 43 |  |  | PEAKS DB |
| K.RFDEIVGGFDQLPIS.M | N | 52.08 | 1691.8518 | 15 | 0.2 | 846.9333 | 2 | 43.43 | 1 | 29880 | BuCaMH04.raw | 8.7751E5 | 1 | 1 | 251 | 265 |  |  | PEAKS DB |
| total 11 peptides |  |  |  |  |  |  |  |  |  |  |  |  |  |  |  |  |  |  |  |
| --- | --- | --- | --- | --- | --- | --- | --- | --- | --- | --- | --- | --- | --- | --- | --- | --- | --- | --- | --- |

Q4JHE3.1

back to list

  

| Protein Coverage
| Supporting Peptides
|

Protein Coverage:

Supporting Peptides:

| Peptide | Uniq | -10lgP | Mass | Length | ppm | m/z | z | RT | Fraction | Scan | Source File | Area BuCaMH04\_B15 | #Feature | #Feature BuCaMH04\_B15 | Start | End | PTM | AScore | Found By |
| --- | --- | --- | --- | --- | --- | --- | --- | --- | --- | --- | --- | --- | --- | --- | --- | --- | --- | --- | --- |
| K.HVVVVGAGMAGLSAAYVLAGAGHK.V | N | 106.63 | 2234.1992 | 24 | 0.7 | 745.7409 | 3 | 30.19 | 1 | 18119 | BuCaMH04.raw | 2.655E7 | 1 | 1 | 54 | 77 |  |  | PEAKS DB |
| K.LNEFFQENENAWYFIR.N | N | 98.32 | 2118.9800 | 16 | 0.4 | 1060.4977 | 2 | 49.95 | 1 | 35860 | BuCaMH04.raw | 1.7944E7 | 1 | 1 | 128 | 143 |  |  | PEAKS DB |
| K.TSADIVINDLSLIHQLPK.K | N | 98.11 | 1976.0942 | 18 | 0.9 | 659.7059 | 3 | 39.96 | 1 | 27043 | BuCaMH04.raw | 9.5523E7 | 2 | 2 | 407 | 424 |  |  | PEAKS DB |
| R.EADYEEFLEIAR.N | N | 92.74 | 1483.6830 | 12 | 1.0 | 742.8495 | 2 | 40.39 | 1 | 27344 | BuCaMH04.raw | 8.6584E7 | 1 | 1 | 32 | 43 |  |  | PEAKS DB |
| L.NEFFQENENAWYFIR.N | N | 85.39 | 2005.8959 | 15 | 0.0 | 1003.9552 | 2 | 47.08 | 1 | 33265 | BuCaMH04.raw | 1.6334E7 | 1 | 1 | 129 | 143 |  |  | PEAKS DB |
| R.IYFEPPLPPK.K | N | 84.04 | 1199.6589 | 10 | 0.3 | 600.8369 | 2 | 25.08 | 1 | 14052 | BuCaMH04.raw | 8.9718E7 | 2 | 2 | 321 | 330 |  |  | PEAKS DB |
| E.ADYEEFLEIAR.N | N | 74.83 | 1354.6404 | 11 | 0.3 | 678.3276 | 2 | 38.08 | 1 | 25194 | BuCaMH04.raw | 3.5897E7 | 1 | 1 | 33 | 43 |  |  | PEAKS DB |
| A.DIVINDLSLIHQLPK.K | N | 69.37 | 1716.9774 | 15 | 0.4 | 859.4963 | 2 | 37.46 | 1 | 24584 | BuCaMH04.raw | 4.3779E7 | 2 | 2 | 410 | 424 |  |  | PEAKS DB |
| A.DYEEFLEIAR.N | N | 65.68 | 1283.6033 | 10 | 0.6 | 642.8093 | 2 | 40.14 | 1 | 26895 | BuCaMH04.raw | 2.3334E6 | 1 | 1 | 34 | 43 |  |  | PEAKS DB |
| D.YEEFLEIAR.N | N | 59.77 | 1168.5764 | 9 | 0.4 | 585.2957 | 2 | 26.94 | 1 | 14950 | BuCaMH04.raw | 2.8615E6 | 1 | 1 | 35 | 43 |  |  | PEAKS DB |
| K.RFDEIVGGFDQLPIS.M | N | 52.08 | 1691.8518 | 15 | 0.2 | 846.9333 | 2 | 43.43 | 1 | 29880 | BuCaMH04.raw | 8.7751E5 | 1 | 1 | 251 | 265 |  |  | PEAKS DB |
| total 11 peptides |  |  |  |  |  |  |  |  |  |  |  |  |  |  |  |  |  |  |  |
| --- | --- | --- | --- | --- | --- | --- | --- | --- | --- | --- | --- | --- | --- | --- | --- | --- | --- | --- | --- |

ABN72537.1

back to list

  

| Protein Coverage
| Supporting Peptides
|

Protein Coverage:

Supporting Peptides:

| Peptide | Uniq | -10lgP | Mass | Length | ppm | m/z | z | RT | Fraction | Scan | Source File | Area BuCaMH04\_B15 | #Feature | #Feature BuCaMH04\_B15 | Start | End | PTM | AScore | Found By |
| --- | --- | --- | --- | --- | --- | --- | --- | --- | --- | --- | --- | --- | --- | --- | --- | --- | --- | --- | --- |
| F.NGNTVGLAALGSLC(+57.02)SVK.Y | N | 96.93 | 1659.8613 | 17 | 0.9 | 830.9387 | 2 | 42.94 | 1 | 29615 | BuCaMH04.raw | 1.323E7 | 1 | 1 | 303 | 319 | Carbamidomethylation | C14:Carbamidomethylation:1000.00 | PEAKS DB |
| R.VYELVNILNTILR.R | Y | 94.54 | 1558.9082 | 13 | 0.5 | 780.4618 | 2 | 65.14 | 1 | 49070 | BuCaMH04.raw | 1.1235E8 | 2 | 2 | 232 | 244 |  |  | PEAKS DB |
| K.C(+57.02)PTLTNQC(+57.02)IALMGPNVK.V | N | 82.56 | 1915.9318 | 17 | 1.0 | 958.9741 | 2 | 24.20 | 1 | 12489 | BuCaMH04.raw | 1.1923E7 | 1 | 1 | 511 | 527 | Carbamidomethylation | C1:Carbamidomethylation:1000.00;C8:Carbamidomethylation:1000.00 | PEAKS DB |
| N.YFVEVGEEC(+57.02)DC(+57.02)GSPR.D | N | 82.16 | 1802.7240 | 15 | 0.5 | 902.3698 | 2 | 18.36 | 1 | 7501 | BuCaMH04.raw | 1.0067E8 | 1 | 1 | 415 | 429 | Carbamidomethylation | C9:Carbamidomethylation:1000.00;C11:Carbamidomethylation:1000.00 | PEAKS DB |
| K.YSVAVIQDYSK.R | N | 80.81 | 1271.6398 | 11 | 0.4 | 636.8274 | 2 | 19.99 | 1 | 8958 | BuCaMH04.raw | 9.4287E7 | 1 | 1 | 320 | 330 |  |  | PEAKS DB |
| C.LILPDDPNYGMVETGTK.C | N | 80.12 | 1861.9131 | 17 | 0.2 | 931.9640 | 2 | 27.95 | 1 | 15970 | BuCaMH04.raw | 3.1118E6 | 1 | 1 | 579 | 595 |  |  | PEAKS DB |
| V.YELVNILNTILR.R | Y | 75.02 | 1459.8398 | 12 | 0.4 | 730.9275 | 2 | 59.51 | 1 | 44161 | BuCaMH04.raw | 1.372E7 | 1 | 1 | 233 | 244 |  |  | PEAKS DB |
| N.GNTVGLAALGSLC(+57.02)SVK.Y | N | 72.59 | 1545.8185 | 16 | 0.0 | 773.9165 | 2 | 30.49 | 1 | 18460 | BuCaMH04.raw | 5.7108E8 | 3 | 3 | 304 | 319 | Carbamidomethylation | C13:Carbamidomethylation:1000.00 | PEAKS DB |
| R.RLNFHIALIGLEIWSK.R | Y | 71.69 | 1909.0938 | 16 | 1.0 | 478.2812 | 4 | 43.04 | 1 | 29535 | BuCaMH04.raw | 4.2204E5 | 1 | 1 | 245 | 260 |  |  | PEAKS DB |
| H.IALIGLEIWSK.R | Y | 66.79 | 1241.7383 | 11 | 0.9 | 621.8770 | 2 | 46.50 | 1 | 32759 | BuCaMH04.raw | 1.8284E6 | 1 | 1 | 250 | 260 |  |  | PEAKS DB |
| R.RVYELVNILNTILR.R | Y | 63.68 | 1715.0094 | 14 | 0.7 | 572.6775 | 3 | 52.48 | 1 | 38081 | BuCaMH04.raw | 1.3218E7 | 1 | 1 | 231 | 244 |  |  | PEAKS DB |
| R.LNFHIALIGLEIWSK.R | Y | 58.99 | 1752.9927 | 15 | -0.1 | 585.3381 | 3 | 51.34 | 1 | 36932 | BuCaMH04.raw | 6.5213E5 | 1 | 1 | 246 | 260 |  |  | PEAKS DB |
| R.VYELVNILNTILRR.L | Y | 52.50 | 1715.0094 | 14 | 0.7 | 572.6775 | 3 | 56.95 | 1 | 41802 | BuCaMH04.raw | 1.4339E6 | 1 | 1 | 232 | 245 |  |  | PEAKS DB |
| total 13 peptides |  |  |  |  |  |  |  |  |  |  |  |  |  |  |  |  |  |  |  |
| --- | --- | --- | --- | --- | --- | --- | --- | --- | --- | --- | --- | --- | --- | --- | --- | --- | --- | --- | --- |

A8QL49.1

back to list

  

| Protein Coverage
| Supporting Peptides
|

Protein Coverage:

Supporting Peptides:

| Peptide | Uniq | -10lgP | Mass | Length | ppm | m/z | z | RT | Fraction | Scan | Source File | Area BuCaMH04\_B15 | #Feature | #Feature BuCaMH04\_B15 | Start | End | PTM | AScore | Found By |
| --- | --- | --- | --- | --- | --- | --- | --- | --- | --- | --- | --- | --- | --- | --- | --- | --- | --- | --- | --- |
| F.NGNTVGLAALGSLC(+57.02)SVK.Y | N | 96.93 | 1659.8613 | 17 | 0.9 | 830.9387 | 2 | 42.94 | 1 | 29615 | BuCaMH04.raw | 1.323E7 | 1 | 1 | 303 | 319 | Carbamidomethylation | C14:Carbamidomethylation:1000.00 | PEAKS DB |
| R.VYELVNILNTILR.R | Y | 94.54 | 1558.9082 | 13 | 0.5 | 780.4618 | 2 | 65.14 | 1 | 49070 | BuCaMH04.raw | 1.1235E8 | 2 | 2 | 232 | 244 |  |  | PEAKS DB |
| K.C(+57.02)PTLTNQC(+57.02)IALMGPNVK.V | N | 82.56 | 1915.9318 | 17 | 1.0 | 958.9741 | 2 | 24.20 | 1 | 12489 | BuCaMH04.raw | 1.1923E7 | 1 | 1 | 511 | 527 | Carbamidomethylation | C1:Carbamidomethylation:1000.00;C8:Carbamidomethylation:1000.00 | PEAKS DB |
| N.YFVEVGEEC(+57.02)DC(+57.02)GSPR.D | N | 82.16 | 1802.7240 | 15 | 0.5 | 902.3698 | 2 | 18.36 | 1 | 7501 | BuCaMH04.raw | 1.0067E8 | 1 | 1 | 415 | 429 | Carbamidomethylation | C9:Carbamidomethylation:1000.00;C11:Carbamidomethylation:1000.00 | PEAKS DB |
| K.YSVAVIQDYSK.R | N | 80.81 | 1271.6398 | 11 | 0.4 | 636.8274 | 2 | 19.99 | 1 | 8958 | BuCaMH04.raw | 9.4287E7 | 1 | 1 | 320 | 330 |  |  | PEAKS DB |
| C.LILPDDPNYGMVETGTK.C | N | 80.12 | 1861.9131 | 17 | 0.2 | 931.9640 | 2 | 27.95 | 1 | 15970 | BuCaMH04.raw | 3.1118E6 | 1 | 1 | 579 | 595 |  |  | PEAKS DB |
| V.YELVNILNTILR.R | Y | 75.02 | 1459.8398 | 12 | 0.4 | 730.9275 | 2 | 59.51 | 1 | 44161 | BuCaMH04.raw | 1.372E7 | 1 | 1 | 233 | 244 |  |  | PEAKS DB |
| N.GNTVGLAALGSLC(+57.02)SVK.Y | N | 72.59 | 1545.8185 | 16 | 0.0 | 773.9165 | 2 | 30.49 | 1 | 18460 | BuCaMH04.raw | 5.7108E8 | 3 | 3 | 304 | 319 | Carbamidomethylation | C13:Carbamidomethylation:1000.00 | PEAKS DB |
| R.RLNFHIALIGLEIWSK.R | Y | 71.69 | 1909.0938 | 16 | 1.0 | 478.2812 | 4 | 43.04 | 1 | 29535 | BuCaMH04.raw | 4.2204E5 | 1 | 1 | 245 | 260 |  |  | PEAKS DB |
| H.IALIGLEIWSK.R | Y | 66.79 | 1241.7383 | 11 | 0.9 | 621.8770 | 2 | 46.50 | 1 | 32759 | BuCaMH04.raw | 1.8284E6 | 1 | 1 | 250 | 260 |  |  | PEAKS DB |
| R.RVYELVNILNTILR.R | Y | 63.68 | 1715.0094 | 14 | 0.7 | 572.6775 | 3 | 52.48 | 1 | 38081 | BuCaMH04.raw | 1.3218E7 | 1 | 1 | 231 | 244 |  |  | PEAKS DB |
| R.LNFHIALIGLEIWSK.R | Y | 58.99 | 1752.9927 | 15 | -0.1 | 585.3381 | 3 | 51.34 | 1 | 36932 | BuCaMH04.raw | 6.5213E5 | 1 | 1 | 246 | 260 |  |  | PEAKS DB |
| R.VYELVNILNTILRR.L | Y | 52.50 | 1715.0094 | 14 | 0.7 | 572.6775 | 3 | 56.95 | 1 | 41802 | BuCaMH04.raw | 1.4339E6 | 1 | 1 | 232 | 245 |  |  | PEAKS DB |
| total 13 peptides |  |  |  |  |  |  |  |  |  |  |  |  |  |  |  |  |  |  |  |
| --- | --- | --- | --- | --- | --- | --- | --- | --- | --- | --- | --- | --- | --- | --- | --- | --- | --- | --- | --- |

JAS05143.1

back to list

  

| Protein Coverage
| Supporting Peptides
|

Protein Coverage:

Supporting Peptides:

| Peptide | Uniq | -10lgP | Mass | Length | ppm | m/z | z | RT | Fraction | Scan | Source File | Area BuCaMH04\_B15 | #Feature | #Feature BuCaMH04\_B15 | Start | End | PTM | AScore | Found By |
| --- | --- | --- | --- | --- | --- | --- | --- | --- | --- | --- | --- | --- | --- | --- | --- | --- | --- | --- | --- |
| K.YLGYLNVIFDDK.G | N | 102.03 | 1458.7394 | 12 | 0.6 | 730.3774 | 2 | 47.23 | 1 | 33232 | BuCaMH04.raw | 1.4989E7 | 1 | 1 | 300 | 311 |  |  | PEAKS DB |
| K.VLLPSFLAAGGDGYYMLK.G | Y | 94.29 | 1913.9961 | 18 | 0.9 | 958.0062 | 2 | 51.12 | 1 | 36679 | BuCaMH04.raw | 4.3518E6 | 1 | 1 | 510 | 527 |  |  | PEAKS DB |
| R.NVLLLDAGDQYQGTVWFNYFK.G | Y | 93.88 | 2490.2219 | 21 | 0.3 | 1246.1187 | 2 | 58.59 | 1 | 43390 | BuCaMH04.raw | 5.0627E6 | 1 | 1 | 91 | 111 |  |  | PEAKS DB |
| K.YLGYLNVIFDDKGK.V | Y | 87.87 | 1643.8558 | 14 | -0.4 | 548.9590 | 3 | 36.56 | 1 | 23752 | BuCaMH04.raw | 1.7237E6 | 1 | 1 | 300 | 313 |  |  | PEAKS DB |
| R.VVSLNVLC(+57.02)TEC(+57.02)R.V | N | 85.35 | 1448.7115 | 12 | 0.3 | 725.3633 | 2 | 23.30 | 1 | 11847 | BuCaMH04.raw | 3.0811E8 | 1 | 1 | 484 | 495 | Carbamidomethylation | C8:Carbamidomethylation:1000.00;C11:Carbamidomethylation:1000.00 | PEAKS DB |
| A.VLPFGGTFDLLQIK.G | N | 75.40 | 1546.8759 | 14 | 0.8 | 774.4458 | 2 | 52.72 | 1 | 38151 | BuCaMH04.raw | 4.902E6 | 1 | 1 | 429 | 442 |  |  | PEAKS DB |
| K.ETPVLSNPGPYLEFRDEVEELQK.Q | N | 72.40 | 2688.3281 | 23 | 0.9 | 897.1174 | 3 | 41.06 | 1 | 27972 | BuCaMH04.raw | 3.1625E7 | 1 | 1 | 194 | 216 |  |  | PEAKS DB |
| R.FHEC(+57.02)NLGNLIC(+57.02)DAVVYNNLR.H | N | 71.60 | 2420.1365 | 20 | 0.6 | 807.7199 | 3 | 41.26 | 1 | 27842 | BuCaMH04.raw | 2.9367E7 | 1 | 1 | 369 | 388 | Carbamidomethylation | C4:Carbamidomethylation:1000.00;C11:Carbamidomethylation:1000.00 | PEAKS DB |
| Y.LGYLNVIFDDK.G | N | 69.50 | 1295.6761 | 11 | 0.8 | 648.8459 | 2 | 41.12 | 1 | 27795 | BuCaMH04.raw | 7.8437E5 | 1 | 1 | 301 | 311 |  |  | PEAKS DB |
| K.ETPVLSNPGPYLEFR.D | N | 67.68 | 1717.8674 | 15 | 1.3 | 859.9421 | 2 | 33.85 | 1 | 21396 | BuCaMH04.raw | 6.7956E6 | 1 | 1 | 194 | 208 |  |  | PEAKS DB |
| V.LPFGGTFDLLQIK.G | N | 65.66 | 1447.8075 | 13 | 0.5 | 724.9114 | 2 | 49.97 | 1 | 35712 | BuCaMH04.raw | 4.3937E5 | 1 | 1 | 430 | 442 |  |  | PEAKS DB |
| S.SGDLDISIVSDYIK.R | N | 62.14 | 1523.7719 | 14 | 0.9 | 762.8939 | 2 | 41.97 | 1 | 28608 | BuCaMH04.raw | 6.6435E5 | 1 | 1 | 535 | 548 |  |  | PEAKS DB |
| L.LPSFLAAGGDGYYMLK.G | Y | 55.12 | 1701.8436 | 16 | 0.3 | 851.9293 | 2 | 42.68 | 1 | 29092 | BuCaMH04.raw | 2.3151E6 | 1 | 1 | 512 | 527 |  |  | PEAKS DB |
| R.QVPVVQAYAFGK.Y | N | 54.70 | 1305.7081 | 12 | -0.4 | 653.8611 | 2 | 22.79 | 1 | 11255 | BuCaMH04.raw | 5.2789E6 | 1 | 1 | 288 | 299 |  |  | PEAKS DB |
| total 14 peptides |  |  |  |  |  |  |  |  |  |  |  |  |  |  |  |  |  |  |  |
| --- | --- | --- | --- | --- | --- | --- | --- | --- | --- | --- | --- | --- | --- | --- | --- | --- | --- | --- | --- |

JAI09048.1

back to list

  

| Protein Coverage
| Supporting Peptides
|

Protein Coverage:

Supporting Peptides:

| Peptide | Uniq | -10lgP | Mass | Length | ppm | m/z | z | RT | Fraction | Scan | Source File | Area BuCaMH04\_B15 | #Feature | #Feature BuCaMH04\_B15 | Start | End | PTM | AScore | Found By |
| --- | --- | --- | --- | --- | --- | --- | --- | --- | --- | --- | --- | --- | --- | --- | --- | --- | --- | --- | --- |
| K.YLGYLNVIFDDK.G | N | 102.03 | 1458.7394 | 12 | 0.6 | 730.3774 | 2 | 47.23 | 1 | 33232 | BuCaMH04.raw | 1.4989E7 | 1 | 1 | 300 | 311 |  |  | PEAKS DB |
| K.VLLPSFLAAGGDGYYMLK.G | Y | 94.29 | 1913.9961 | 18 | 0.9 | 958.0062 | 2 | 51.12 | 1 | 36679 | BuCaMH04.raw | 4.3518E6 | 1 | 1 | 510 | 527 |  |  | PEAKS DB |
| R.NVLLLDAGDQYQGTVWFNYFK.G | Y | 93.88 | 2490.2219 | 21 | 0.3 | 1246.1187 | 2 | 58.59 | 1 | 43390 | BuCaMH04.raw | 5.0627E6 | 1 | 1 | 91 | 111 |  |  | PEAKS DB |
| K.YLGYLNVIFDDKGK.V | Y | 87.87 | 1643.8558 | 14 | -0.4 | 548.9590 | 3 | 36.56 | 1 | 23752 | BuCaMH04.raw | 1.7237E6 | 1 | 1 | 300 | 313 |  |  | PEAKS DB |
| R.VVSLNVLC(+57.02)TEC(+57.02)R.V | N | 85.35 | 1448.7115 | 12 | 0.3 | 725.3633 | 2 | 23.30 | 1 | 11847 | BuCaMH04.raw | 3.0811E8 | 1 | 1 | 484 | 495 | Carbamidomethylation | C8:Carbamidomethylation:1000.00;C11:Carbamidomethylation:1000.00 | PEAKS DB |
| A.VLPFGGTFDLLQIK.G | N | 75.40 | 1546.8759 | 14 | 0.8 | 774.4458 | 2 | 52.72 | 1 | 38151 | BuCaMH04.raw | 4.902E6 | 1 | 1 | 429 | 442 |  |  | PEAKS DB |
| K.ETPVLSNPGPYLEFRDEVEELQK.Q | N | 72.40 | 2688.3281 | 23 | 0.9 | 897.1174 | 3 | 41.06 | 1 | 27972 | BuCaMH04.raw | 3.1625E7 | 1 | 1 | 194 | 216 |  |  | PEAKS DB |
| R.FHEC(+57.02)NLGNLIC(+57.02)DAVVYNNLR.H | N | 71.60 | 2420.1365 | 20 | 0.6 | 807.7199 | 3 | 41.26 | 1 | 27842 | BuCaMH04.raw | 2.9367E7 | 1 | 1 | 369 | 388 | Carbamidomethylation | C4:Carbamidomethylation:1000.00;C11:Carbamidomethylation:1000.00 | PEAKS DB |
| Y.LGYLNVIFDDK.G | N | 69.50 | 1295.6761 | 11 | 0.8 | 648.8459 | 2 | 41.12 | 1 | 27795 | BuCaMH04.raw | 7.8437E5 | 1 | 1 | 301 | 311 |  |  | PEAKS DB |
| K.ETPVLSNPGPYLEFR.D | N | 67.68 | 1717.8674 | 15 | 1.3 | 859.9421 | 2 | 33.85 | 1 | 21396 | BuCaMH04.raw | 6.7956E6 | 1 | 1 | 194 | 208 |  |  | PEAKS DB |
| V.LPFGGTFDLLQIK.G | N | 65.66 | 1447.8075 | 13 | 0.5 | 724.9114 | 2 | 49.97 | 1 | 35712 | BuCaMH04.raw | 4.3937E5 | 1 | 1 | 430 | 442 |  |  | PEAKS DB |
| S.SGDLDISIVSDYIK.R | N | 62.14 | 1523.7719 | 14 | 0.9 | 762.8939 | 2 | 41.97 | 1 | 28608 | BuCaMH04.raw | 6.6435E5 | 1 | 1 | 535 | 548 |  |  | PEAKS DB |
| L.LPSFLAAGGDGYYMLK.G | Y | 55.12 | 1701.8436 | 16 | 0.3 | 851.9293 | 2 | 42.68 | 1 | 29092 | BuCaMH04.raw | 2.3151E6 | 1 | 1 | 512 | 527 |  |  | PEAKS DB |
| R.QVPVVQAYAFGK.Y | N | 54.70 | 1305.7081 | 12 | -0.4 | 653.8611 | 2 | 22.79 | 1 | 11255 | BuCaMH04.raw | 5.2789E6 | 1 | 1 | 288 | 299 |  |  | PEAKS DB |
| total 14 peptides |  |  |  |  |  |  |  |  |  |  |  |  |  |  |  |  |  |  |  |
| --- | --- | --- | --- | --- | --- | --- | --- | --- | --- | --- | --- | --- | --- | --- | --- | --- | --- | --- | --- |

JAS05036.1

back to list

  

| Protein Coverage
| Supporting Peptides
|

Protein Coverage:

Supporting Peptides:

| Peptide | Uniq | -10lgP | Mass | Length | ppm | m/z | z | RT | Fraction | Scan | Source File | Area BuCaMH04\_B15 | #Feature | #Feature BuCaMH04\_B15 | Start | End | PTM | AScore | Found By |
| --- | --- | --- | --- | --- | --- | --- | --- | --- | --- | --- | --- | --- | --- | --- | --- | --- | --- | --- | --- |
| K.YLGYLNVIFDDK.G | N | 102.03 | 1458.7394 | 12 | 0.6 | 730.3774 | 2 | 47.23 | 1 | 33232 | BuCaMH04.raw | 1.4989E7 | 1 | 1 | 300 | 311 |  |  | PEAKS DB |
| K.VLLPSFLAAGGDGYYMLK.G | Y | 94.29 | 1913.9961 | 18 | 0.9 | 958.0062 | 2 | 51.12 | 1 | 36679 | BuCaMH04.raw | 4.3518E6 | 1 | 1 | 510 | 527 |  |  | PEAKS DB |
| R.NVLLLDAGDQYQGTVWFNYFK.G | Y | 93.88 | 2490.2219 | 21 | 0.3 | 1246.1187 | 2 | 58.59 | 1 | 43390 | BuCaMH04.raw | 5.0627E6 | 1 | 1 | 91 | 111 |  |  | PEAKS DB |
| K.YLGYLNVIFDDKGK.V | Y | 87.87 | 1643.8558 | 14 | -0.4 | 548.9590 | 3 | 36.56 | 1 | 23752 | BuCaMH04.raw | 1.7237E6 | 1 | 1 | 300 | 313 |  |  | PEAKS DB |
| R.VVSLNVLC(+57.02)TEC(+57.02)R.V | N | 85.35 | 1448.7115 | 12 | 0.3 | 725.3633 | 2 | 23.30 | 1 | 11847 | BuCaMH04.raw | 3.0811E8 | 1 | 1 | 484 | 495 | Carbamidomethylation | C8:Carbamidomethylation:1000.00;C11:Carbamidomethylation:1000.00 | PEAKS DB |
| A.VLPFGGTFDLLQIK.G | N | 75.40 | 1546.8759 | 14 | 0.8 | 774.4458 | 2 | 52.72 | 1 | 38151 | BuCaMH04.raw | 4.902E6 | 1 | 1 | 429 | 442 |  |  | PEAKS DB |
| K.ETPVLSNPGPYLEFRDEVEELQK.Q | N | 72.40 | 2688.3281 | 23 | 0.9 | 897.1174 | 3 | 41.06 | 1 | 27972 | BuCaMH04.raw | 3.1625E7 | 1 | 1 | 194 | 216 |  |  | PEAKS DB |
| R.FHEC(+57.02)NLGNLIC(+57.02)DAVVYNNLR.H | N | 71.60 | 2420.1365 | 20 | 0.6 | 807.7199 | 3 | 41.26 | 1 | 27842 | BuCaMH04.raw | 2.9367E7 | 1 | 1 | 369 | 388 | Carbamidomethylation | C4:Carbamidomethylation:1000.00;C11:Carbamidomethylation:1000.00 | PEAKS DB |
| Y.LGYLNVIFDDK.G | N | 69.50 | 1295.6761 | 11 | 0.8 | 648.8459 | 2 | 41.12 | 1 | 27795 | BuCaMH04.raw | 7.8437E5 | 1 | 1 | 301 | 311 |  |  | PEAKS DB |
| K.ETPVLSNPGPYLEFR.D | N | 67.68 | 1717.8674 | 15 | 1.3 | 859.9421 | 2 | 33.85 | 1 | 21396 | BuCaMH04.raw | 6.7956E6 | 1 | 1 | 194 | 208 |  |  | PEAKS DB |
| V.LPFGGTFDLLQIK.G | N | 65.66 | 1447.8075 | 13 | 0.5 | 724.9114 | 2 | 49.97 | 1 | 35712 | BuCaMH04.raw | 4.3937E5 | 1 | 1 | 430 | 442 |  |  | PEAKS DB |
| S.SGDLDISIVSDYIK.R | N | 62.14 | 1523.7719 | 14 | 0.9 | 762.8939 | 2 | 41.97 | 1 | 28608 | BuCaMH04.raw | 6.6435E5 | 1 | 1 | 535 | 548 |  |  | PEAKS DB |
| L.LPSFLAAGGDGYYMLK.G | Y | 55.12 | 1701.8436 | 16 | 0.3 | 851.9293 | 2 | 42.68 | 1 | 29092 | BuCaMH04.raw | 2.3151E6 | 1 | 1 | 512 | 527 |  |  | PEAKS DB |
| R.QVPVVQAYAFGK.Y | N | 54.70 | 1305.7081 | 12 | -0.4 | 653.8611 | 2 | 22.79 | 1 | 11255 | BuCaMH04.raw | 5.2789E6 | 1 | 1 | 288 | 299 |  |  | PEAKS DB |
| total 14 peptides |  |  |  |  |  |  |  |  |  |  |  |  |  |  |  |  |  |  |  |
| --- | --- | --- | --- | --- | --- | --- | --- | --- | --- | --- | --- | --- | --- | --- | --- | --- | --- | --- | --- |

JAI09049.1

back to list

  

| Protein Coverage
| Supporting Peptides
|

Protein Coverage:

Supporting Peptides:

| Peptide | Uniq | -10lgP | Mass | Length | ppm | m/z | z | RT | Fraction | Scan | Source File | Area BuCaMH04\_B15 | #Feature | #Feature BuCaMH04\_B15 | Start | End | PTM | AScore | Found By |
| --- | --- | --- | --- | --- | --- | --- | --- | --- | --- | --- | --- | --- | --- | --- | --- | --- | --- | --- | --- |
| K.YLGYLNVIFDDK.G | N | 102.03 | 1458.7394 | 12 | 0.6 | 730.3774 | 2 | 47.23 | 1 | 33232 | BuCaMH04.raw | 1.4989E7 | 1 | 1 | 300 | 311 |  |  | PEAKS DB |
| K.VLLPSFLAAGGDGYYMLK.G | Y | 94.29 | 1913.9961 | 18 | 0.9 | 958.0062 | 2 | 51.12 | 1 | 36679 | BuCaMH04.raw | 4.3518E6 | 1 | 1 | 510 | 527 |  |  | PEAKS DB |
| R.NVLLLDAGDQYQGTVWFNYFK.G | Y | 93.88 | 2490.2219 | 21 | 0.3 | 1246.1187 | 2 | 58.59 | 1 | 43390 | BuCaMH04.raw | 5.0627E6 | 1 | 1 | 91 | 111 |  |  | PEAKS DB |
| K.YLGYLNVIFDDKGK.V | Y | 87.87 | 1643.8558 | 14 | -0.4 | 548.9590 | 3 | 36.56 | 1 | 23752 | BuCaMH04.raw | 1.7237E6 | 1 | 1 | 300 | 313 |  |  | PEAKS DB |
| R.VVSLNVLC(+57.02)TEC(+57.02)R.V | N | 85.35 | 1448.7115 | 12 | 0.3 | 725.3633 | 2 | 23.30 | 1 | 11847 | BuCaMH04.raw | 3.0811E8 | 1 | 1 | 484 | 495 | Carbamidomethylation | C8:Carbamidomethylation:1000.00;C11:Carbamidomethylation:1000.00 | PEAKS DB |
| A.VLPFGGTFDLLQIK.G | N | 75.40 | 1546.8759 | 14 | 0.8 | 774.4458 | 2 | 52.72 | 1 | 38151 | BuCaMH04.raw | 4.902E6 | 1 | 1 | 429 | 442 |  |  | PEAKS DB |
| K.ETPVLSNPGPYLEFRDEVEELQK.Q | N | 72.40 | 2688.3281 | 23 | 0.9 | 897.1174 | 3 | 41.06 | 1 | 27972 | BuCaMH04.raw | 3.1625E7 | 1 | 1 | 194 | 216 |  |  | PEAKS DB |
| R.FHEC(+57.02)NLGNLIC(+57.02)DAVVYNNLR.H | N | 71.60 | 2420.1365 | 20 | 0.6 | 807.7199 | 3 | 41.26 | 1 | 27842 | BuCaMH04.raw | 2.9367E7 | 1 | 1 | 369 | 388 | Carbamidomethylation | C4:Carbamidomethylation:1000.00;C11:Carbamidomethylation:1000.00 | PEAKS DB |
| Y.LGYLNVIFDDK.G | N | 69.50 | 1295.6761 | 11 | 0.8 | 648.8459 | 2 | 41.12 | 1 | 27795 | BuCaMH04.raw | 7.8437E5 | 1 | 1 | 301 | 311 |  |  | PEAKS DB |
| K.ETPVLSNPGPYLEFR.D | N | 67.68 | 1717.8674 | 15 | 1.3 | 859.9421 | 2 | 33.85 | 1 | 21396 | BuCaMH04.raw | 6.7956E6 | 1 | 1 | 194 | 208 |  |  | PEAKS DB |
| V.LPFGGTFDLLQIK.G | N | 65.66 | 1447.8075 | 13 | 0.5 | 724.9114 | 2 | 49.97 | 1 | 35712 | BuCaMH04.raw | 4.3937E5 | 1 | 1 | 430 | 442 |  |  | PEAKS DB |
| S.SGDLDISIVSDYIK.R | N | 62.14 | 1523.7719 | 14 | 0.9 | 762.8939 | 2 | 41.97 | 1 | 28608 | BuCaMH04.raw | 6.6435E5 | 1 | 1 | 535 | 548 |  |  | PEAKS DB |
| L.LPSFLAAGGDGYYMLK.G | Y | 55.12 | 1701.8436 | 16 | 0.3 | 851.9293 | 2 | 42.68 | 1 | 29092 | BuCaMH04.raw | 2.3151E6 | 1 | 1 | 512 | 527 |  |  | PEAKS DB |
| R.QVPVVQAYAFGK.Y | N | 54.70 | 1305.7081 | 12 | -0.4 | 653.8611 | 2 | 22.79 | 1 | 11255 | BuCaMH04.raw | 5.2789E6 | 1 | 1 | 288 | 299 |  |  | PEAKS DB |
| total 14 peptides |  |  |  |  |  |  |  |  |  |  |  |  |  |  |  |  |  |  |  |
| --- | --- | --- | --- | --- | --- | --- | --- | --- | --- | --- | --- | --- | --- | --- | --- | --- | --- | --- | --- |

JAS05038.1

back to list

  

| Protein Coverage
| Supporting Peptides
|

Protein Coverage:

Supporting Peptides:

| Peptide | Uniq | -10lgP | Mass | Length | ppm | m/z | z | RT | Fraction | Scan | Source File | Area BuCaMH04\_B15 | #Feature | #Feature BuCaMH04\_B15 | Start | End | PTM | AScore | Found By |
| --- | --- | --- | --- | --- | --- | --- | --- | --- | --- | --- | --- | --- | --- | --- | --- | --- | --- | --- | --- |
| K.YLGYLNVIFDDK.G | N | 102.03 | 1458.7394 | 12 | 0.6 | 730.3774 | 2 | 47.23 | 1 | 33232 | BuCaMH04.raw | 1.4989E7 | 1 | 1 | 300 | 311 |  |  | PEAKS DB |
| K.VLLPSFLAAGGDGYYMLK.G | Y | 94.29 | 1913.9961 | 18 | 0.9 | 958.0062 | 2 | 51.12 | 1 | 36679 | BuCaMH04.raw | 4.3518E6 | 1 | 1 | 510 | 527 |  |  | PEAKS DB |
| R.NVLLLDAGDQYQGTVWFNYFK.G | Y | 93.88 | 2490.2219 | 21 | 0.3 | 1246.1187 | 2 | 58.59 | 1 | 43390 | BuCaMH04.raw | 5.0627E6 | 1 | 1 | 91 | 111 |  |  | PEAKS DB |
| K.YLGYLNVIFDDKGK.V | Y | 87.87 | 1643.8558 | 14 | -0.4 | 548.9590 | 3 | 36.56 | 1 | 23752 | BuCaMH04.raw | 1.7237E6 | 1 | 1 | 300 | 313 |  |  | PEAKS DB |
| R.VVSLNVLC(+57.02)TEC(+57.02)R.V | N | 85.35 | 1448.7115 | 12 | 0.3 | 725.3633 | 2 | 23.30 | 1 | 11847 | BuCaMH04.raw | 3.0811E8 | 1 | 1 | 484 | 495 | Carbamidomethylation | C8:Carbamidomethylation:1000.00;C11:Carbamidomethylation:1000.00 | PEAKS DB |
| A.VLPFGGTFDLLQIK.G | N | 75.40 | 1546.8759 | 14 | 0.8 | 774.4458 | 2 | 52.72 | 1 | 38151 | BuCaMH04.raw | 4.902E6 | 1 | 1 | 429 | 442 |  |  | PEAKS DB |
| K.ETPVLSNPGPYLEFRDEVEELQK.Q | N | 72.40 | 2688.3281 | 23 | 0.9 | 897.1174 | 3 | 41.06 | 1 | 27972 | BuCaMH04.raw | 3.1625E7 | 1 | 1 | 194 | 216 |  |  | PEAKS DB |
| R.FHEC(+57.02)NLGNLIC(+57.02)DAVVYNNLR.H | N | 71.60 | 2420.1365 | 20 | 0.6 | 807.7199 | 3 | 41.26 | 1 | 27842 | BuCaMH04.raw | 2.9367E7 | 1 | 1 | 369 | 388 | Carbamidomethylation | C4:Carbamidomethylation:1000.00;C11:Carbamidomethylation:1000.00 | PEAKS DB |
| Y.LGYLNVIFDDK.G | N | 69.50 | 1295.6761 | 11 | 0.8 | 648.8459 | 2 | 41.12 | 1 | 27795 | BuCaMH04.raw | 7.8437E5 | 1 | 1 | 301 | 311 |  |  | PEAKS DB |
| K.ETPVLSNPGPYLEFR.D | N | 67.68 | 1717.8674 | 15 | 1.3 | 859.9421 | 2 | 33.85 | 1 | 21396 | BuCaMH04.raw | 6.7956E6 | 1 | 1 | 194 | 208 |  |  | PEAKS DB |
| V.LPFGGTFDLLQIK.G | N | 65.66 | 1447.8075 | 13 | 0.5 | 724.9114 | 2 | 49.97 | 1 | 35712 | BuCaMH04.raw | 4.3937E5 | 1 | 1 | 430 | 442 |  |  | PEAKS DB |
| S.SGDLDISIVSDYIK.R | N | 62.14 | 1523.7719 | 14 | 0.9 | 762.8939 | 2 | 41.97 | 1 | 28608 | BuCaMH04.raw | 6.6435E5 | 1 | 1 | 535 | 548 |  |  | PEAKS DB |
| L.LPSFLAAGGDGYYMLK.G | Y | 55.12 | 1701.8436 | 16 | 0.3 | 851.9293 | 2 | 42.68 | 1 | 29092 | BuCaMH04.raw | 2.3151E6 | 1 | 1 | 512 | 527 |  |  | PEAKS DB |
| R.QVPVVQAYAFGK.Y | N | 54.70 | 1305.7081 | 12 | -0.4 | 653.8611 | 2 | 22.79 | 1 | 11255 | BuCaMH04.raw | 5.2789E6 | 1 | 1 | 288 | 299 |  |  | PEAKS DB |
| total 14 peptides |  |  |  |  |  |  |  |  |  |  |  |  |  |  |  |  |  |  |  |
| --- | --- | --- | --- | --- | --- | --- | --- | --- | --- | --- | --- | --- | --- | --- | --- | --- | --- | --- | --- |

JAS05037.1

back to list

  

| Protein Coverage
| Supporting Peptides
|

Protein Coverage:

Supporting Peptides:

| Peptide | Uniq | -10lgP | Mass | Length | ppm | m/z | z | RT | Fraction | Scan | Source File | Area BuCaMH04\_B15 | #Feature | #Feature BuCaMH04\_B15 | Start | End | PTM | AScore | Found By |
| --- | --- | --- | --- | --- | --- | --- | --- | --- | --- | --- | --- | --- | --- | --- | --- | --- | --- | --- | --- |
| K.YLGYLNVIFDDK.G | N | 102.03 | 1458.7394 | 12 | 0.6 | 730.3774 | 2 | 47.23 | 1 | 33232 | BuCaMH04.raw | 1.4989E7 | 1 | 1 | 300 | 311 |  |  | PEAKS DB |
| K.VLLPSFLAAGGDGYYMLK.G | Y | 94.29 | 1913.9961 | 18 | 0.9 | 958.0062 | 2 | 51.12 | 1 | 36679 | BuCaMH04.raw | 4.3518E6 | 1 | 1 | 510 | 527 |  |  | PEAKS DB |
| R.NVLLLDAGDQYQGTVWFNYFK.G | Y | 93.88 | 2490.2219 | 21 | 0.3 | 1246.1187 | 2 | 58.59 | 1 | 43390 | BuCaMH04.raw | 5.0627E6 | 1 | 1 | 91 | 111 |  |  | PEAKS DB |
| K.YLGYLNVIFDDKGK.V | Y | 87.87 | 1643.8558 | 14 | -0.4 | 548.9590 | 3 | 36.56 | 1 | 23752 | BuCaMH04.raw | 1.7237E6 | 1 | 1 | 300 | 313 |  |  | PEAKS DB |
| R.VVSLNVLC(+57.02)TEC(+57.02)R.V | N | 85.35 | 1448.7115 | 12 | 0.3 | 725.3633 | 2 | 23.30 | 1 | 11847 | BuCaMH04.raw | 3.0811E8 | 1 | 1 | 484 | 495 | Carbamidomethylation | C8:Carbamidomethylation:1000.00;C11:Carbamidomethylation:1000.00 | PEAKS DB |
| A.VLPFGGTFDLLQIK.G | N | 75.40 | 1546.8759 | 14 | 0.8 | 774.4458 | 2 | 52.72 | 1 | 38151 | BuCaMH04.raw | 4.902E6 | 1 | 1 | 429 | 442 |  |  | PEAKS DB |
| K.ETPVLSNPGPYLEFRDEVEELQK.Q | N | 72.40 | 2688.3281 | 23 | 0.9 | 897.1174 | 3 | 41.06 | 1 | 27972 | BuCaMH04.raw | 3.1625E7 | 1 | 1 | 194 | 216 |  |  | PEAKS DB |
| R.FHEC(+57.02)NLGNLIC(+57.02)DAVVYNNLR.H | N | 71.60 | 2420.1365 | 20 | 0.6 | 807.7199 | 3 | 41.26 | 1 | 27842 | BuCaMH04.raw | 2.9367E7 | 1 | 1 | 369 | 388 | Carbamidomethylation | C4:Carbamidomethylation:1000.00;C11:Carbamidomethylation:1000.00 | PEAKS DB |
| Y.LGYLNVIFDDK.G | N | 69.50 | 1295.6761 | 11 | 0.8 | 648.8459 | 2 | 41.12 | 1 | 27795 | BuCaMH04.raw | 7.8437E5 | 1 | 1 | 301 | 311 |  |  | PEAKS DB |
| K.ETPVLSNPGPYLEFR.D | N | 67.68 | 1717.8674 | 15 | 1.3 | 859.9421 | 2 | 33.85 | 1 | 21396 | BuCaMH04.raw | 6.7956E6 | 1 | 1 | 194 | 208 |  |  | PEAKS DB |
| V.LPFGGTFDLLQIK.G | N | 65.66 | 1447.8075 | 13 | 0.5 | 724.9114 | 2 | 49.97 | 1 | 35712 | BuCaMH04.raw | 4.3937E5 | 1 | 1 | 430 | 442 |  |  | PEAKS DB |
| S.SGDLDISIVSDYIK.R | N | 62.14 | 1523.7719 | 14 | 0.9 | 762.8939 | 2 | 41.97 | 1 | 28608 | BuCaMH04.raw | 6.6435E5 | 1 | 1 | 535 | 548 |  |  | PEAKS DB |
| L.LPSFLAAGGDGYYMLK.G | Y | 55.12 | 1701.8436 | 16 | 0.3 | 851.9293 | 2 | 42.68 | 1 | 29092 | BuCaMH04.raw | 2.3151E6 | 1 | 1 | 512 | 527 |  |  | PEAKS DB |
| R.QVPVVQAYAFGK.Y | N | 54.70 | 1305.7081 | 12 | -0.4 | 653.8611 | 2 | 22.79 | 1 | 11255 | BuCaMH04.raw | 5.2789E6 | 1 | 1 | 288 | 299 |  |  | PEAKS DB |
| total 14 peptides |  |  |  |  |  |  |  |  |  |  |  |  |  |  |  |  |  |  |  |
| --- | --- | --- | --- | --- | --- | --- | --- | --- | --- | --- | --- | --- | --- | --- | --- | --- | --- | --- | --- |

JAI09047.1

back to list

  

| Protein Coverage
| Supporting Peptides
|

Protein Coverage:

Supporting Peptides:

| Peptide | Uniq | -10lgP | Mass | Length | ppm | m/z | z | RT | Fraction | Scan | Source File | Area BuCaMH04\_B15 | #Feature | #Feature BuCaMH04\_B15 | Start | End | PTM | AScore | Found By |
| --- | --- | --- | --- | --- | --- | --- | --- | --- | --- | --- | --- | --- | --- | --- | --- | --- | --- | --- | --- |
| K.YLGYLNVIFDDK.G | N | 102.03 | 1458.7394 | 12 | 0.6 | 730.3774 | 2 | 47.23 | 1 | 33232 | BuCaMH04.raw | 1.4989E7 | 1 | 1 | 300 | 311 |  |  | PEAKS DB |
| K.VLLPSFLAAGGDGYYMLK.G | Y | 94.29 | 1913.9961 | 18 | 0.9 | 958.0062 | 2 | 51.12 | 1 | 36679 | BuCaMH04.raw | 4.3518E6 | 1 | 1 | 510 | 527 |  |  | PEAKS DB |
| R.NVLLLDAGDQYQGTVWFNYFK.G | Y | 93.88 | 2490.2219 | 21 | 0.3 | 1246.1187 | 2 | 58.59 | 1 | 43390 | BuCaMH04.raw | 5.0627E6 | 1 | 1 | 91 | 111 |  |  | PEAKS DB |
| K.YLGYLNVIFDDKGK.V | Y | 87.87 | 1643.8558 | 14 | -0.4 | 548.9590 | 3 | 36.56 | 1 | 23752 | BuCaMH04.raw | 1.7237E6 | 1 | 1 | 300 | 313 |  |  | PEAKS DB |
| R.VVSLNVLC(+57.02)TEC(+57.02)R.V | N | 85.35 | 1448.7115 | 12 | 0.3 | 725.3633 | 2 | 23.30 | 1 | 11847 | BuCaMH04.raw | 3.0811E8 | 1 | 1 | 484 | 495 | Carbamidomethylation | C8:Carbamidomethylation:1000.00;C11:Carbamidomethylation:1000.00 | PEAKS DB |
| A.VLPFGGTFDLLQIK.G | N | 75.40 | 1546.8759 | 14 | 0.8 | 774.4458 | 2 | 52.72 | 1 | 38151 | BuCaMH04.raw | 4.902E6 | 1 | 1 | 429 | 442 |  |  | PEAKS DB |
| K.ETPVLSNPGPYLEFRDEVEELQK.Q | N | 72.40 | 2688.3281 | 23 | 0.9 | 897.1174 | 3 | 41.06 | 1 | 27972 | BuCaMH04.raw | 3.1625E7 | 1 | 1 | 194 | 216 |  |  | PEAKS DB |
| R.FHEC(+57.02)NLGNLIC(+57.02)DAVVYNNLR.H | N | 71.60 | 2420.1365 | 20 | 0.6 | 807.7199 | 3 | 41.26 | 1 | 27842 | BuCaMH04.raw | 2.9367E7 | 1 | 1 | 369 | 388 | Carbamidomethylation | C4:Carbamidomethylation:1000.00;C11:Carbamidomethylation:1000.00 | PEAKS DB |
| Y.LGYLNVIFDDK.G | N | 69.50 | 1295.6761 | 11 | 0.8 | 648.8459 | 2 | 41.12 | 1 | 27795 | BuCaMH04.raw | 7.8437E5 | 1 | 1 | 301 | 311 |  |  | PEAKS DB |
| K.ETPVLSNPGPYLEFR.D | N | 67.68 | 1717.8674 | 15 | 1.3 | 859.9421 | 2 | 33.85 | 1 | 21396 | BuCaMH04.raw | 6.7956E6 | 1 | 1 | 194 | 208 |  |  | PEAKS DB |
| V.LPFGGTFDLLQIK.G | N | 65.66 | 1447.8075 | 13 | 0.5 | 724.9114 | 2 | 49.97 | 1 | 35712 | BuCaMH04.raw | 4.3937E5 | 1 | 1 | 430 | 442 |  |  | PEAKS DB |
| S.SGDLDISIVSDYIK.R | N | 62.14 | 1523.7719 | 14 | 0.9 | 762.8939 | 2 | 41.97 | 1 | 28608 | BuCaMH04.raw | 6.6435E5 | 1 | 1 | 535 | 548 |  |  | PEAKS DB |
| L.LPSFLAAGGDGYYMLK.G | Y | 55.12 | 1701.8436 | 16 | 0.3 | 851.9293 | 2 | 42.68 | 1 | 29092 | BuCaMH04.raw | 2.3151E6 | 1 | 1 | 512 | 527 |  |  | PEAKS DB |
| R.QVPVVQAYAFGK.Y | N | 54.70 | 1305.7081 | 12 | -0.4 | 653.8611 | 2 | 22.79 | 1 | 11255 | BuCaMH04.raw | 5.2789E6 | 1 | 1 | 288 | 299 |  |  | PEAKS DB |
| total 14 peptides |  |  |  |  |  |  |  |  |  |  |  |  |  |  |  |  |  |  |  |
| --- | --- | --- | --- | --- | --- | --- | --- | --- | --- | --- | --- | --- | --- | --- | --- | --- | --- | --- | --- |

JAB52815.1

back to list

  

| Protein Coverage
| Supporting Peptides
|

Protein Coverage:

Supporting Peptides:

| Peptide | Uniq | -10lgP | Mass | Length | ppm | m/z | z | RT | Fraction | Scan | Source File | Area BuCaMH04\_B15 | #Feature | #Feature BuCaMH04\_B15 | Start | End | PTM | AScore | Found By |
| --- | --- | --- | --- | --- | --- | --- | --- | --- | --- | --- | --- | --- | --- | --- | --- | --- | --- | --- | --- |
| K.YLGYLNVIFDDK.G | N | 102.03 | 1458.7394 | 12 | 0.6 | 730.3774 | 2 | 47.23 | 1 | 33232 | BuCaMH04.raw | 1.4989E7 | 1 | 1 | 300 | 311 |  |  | PEAKS DB |
| K.VLLPSFLAAGGDGYYMLK.G | Y | 94.29 | 1913.9961 | 18 | 0.9 | 958.0062 | 2 | 51.12 | 1 | 36679 | BuCaMH04.raw | 4.3518E6 | 1 | 1 | 510 | 527 |  |  | PEAKS DB |
| R.NVLLLDAGDQYQGTVWFNYFK.G | Y | 93.88 | 2490.2219 | 21 | 0.3 | 1246.1187 | 2 | 58.59 | 1 | 43390 | BuCaMH04.raw | 5.0627E6 | 1 | 1 | 91 | 111 |  |  | PEAKS DB |
| K.YLGYLNVIFDDKGK.V | Y | 87.87 | 1643.8558 | 14 | -0.4 | 548.9590 | 3 | 36.56 | 1 | 23752 | BuCaMH04.raw | 1.7237E6 | 1 | 1 | 300 | 313 |  |  | PEAKS DB |
| R.VVSLNVLC(+57.02)TEC(+57.02)R.V | N | 85.35 | 1448.7115 | 12 | 0.3 | 725.3633 | 2 | 23.30 | 1 | 11847 | BuCaMH04.raw | 3.0811E8 | 1 | 1 | 484 | 495 | Carbamidomethylation | C8:Carbamidomethylation:1000.00;C11:Carbamidomethylation:1000.00 | PEAKS DB |
| A.VLPFGGTFDLLQIK.G | N | 75.40 | 1546.8759 | 14 | 0.8 | 774.4458 | 2 | 52.72 | 1 | 38151 | BuCaMH04.raw | 4.902E6 | 1 | 1 | 429 | 442 |  |  | PEAKS DB |
| K.ETPVLSNPGPYLEFRDEVEELQK.Q | N | 72.40 | 2688.3281 | 23 | 0.9 | 897.1174 | 3 | 41.06 | 1 | 27972 | BuCaMH04.raw | 3.1625E7 | 1 | 1 | 194 | 216 |  |  | PEAKS DB |
| R.FHEC(+57.02)NLGNLIC(+57.02)DAVVYNNLR.H | N | 71.60 | 2420.1365 | 20 | 0.6 | 807.7199 | 3 | 41.26 | 1 | 27842 | BuCaMH04.raw | 2.9367E7 | 1 | 1 | 369 | 388 | Carbamidomethylation | C4:Carbamidomethylation:1000.00;C11:Carbamidomethylation:1000.00 | PEAKS DB |
| Y.LGYLNVIFDDK.G | N | 69.50 | 1295.6761 | 11 | 0.8 | 648.8459 | 2 | 41.12 | 1 | 27795 | BuCaMH04.raw | 7.8437E5 | 1 | 1 | 301 | 311 |  |  | PEAKS DB |
| K.ETPVLSNPGPYLEFR.D | N | 67.68 | 1717.8674 | 15 | 1.3 | 859.9421 | 2 | 33.85 | 1 | 21396 | BuCaMH04.raw | 6.7956E6 | 1 | 1 | 194 | 208 |  |  | PEAKS DB |
| V.LPFGGTFDLLQIK.G | N | 65.66 | 1447.8075 | 13 | 0.5 | 724.9114 | 2 | 49.97 | 1 | 35712 | BuCaMH04.raw | 4.3937E5 | 1 | 1 | 430 | 442 |  |  | PEAKS DB |
| S.SGDLDISIVSDYIK.R | N | 62.14 | 1523.7719 | 14 | 0.9 | 762.8939 | 2 | 41.97 | 1 | 28608 | BuCaMH04.raw | 6.6435E5 | 1 | 1 | 535 | 548 |  |  | PEAKS DB |
| L.LPSFLAAGGDGYYMLK.G | Y | 55.12 | 1701.8436 | 16 | 0.3 | 851.9293 | 2 | 42.68 | 1 | 29092 | BuCaMH04.raw | 2.3151E6 | 1 | 1 | 512 | 527 |  |  | PEAKS DB |
| R.QVPVVQAYAFGK.Y | N | 54.70 | 1305.7081 | 12 | -0.4 | 653.8611 | 2 | 22.79 | 1 | 11255 | BuCaMH04.raw | 5.2789E6 | 1 | 1 | 288 | 299 |  |  | PEAKS DB |
| total 14 peptides |  |  |  |  |  |  |  |  |  |  |  |  |  |  |  |  |  |  |  |
| --- | --- | --- | --- | --- | --- | --- | --- | --- | --- | --- | --- | --- | --- | --- | --- | --- | --- | --- | --- |

T\_109

back to list

  

| Protein Coverage
| Supporting Peptides
|

Protein Coverage:

Supporting Peptides:

| Peptide | Uniq | -10lgP | Mass | Length | ppm | m/z | z | RT | Fraction | Scan | Source File | Area BuCaMH04\_B15 | #Feature | #Feature BuCaMH04\_B15 | Start | End | PTM | AScore | Found By |
| --- | --- | --- | --- | --- | --- | --- | --- | --- | --- | --- | --- | --- | --- | --- | --- | --- | --- | --- | --- |
| K.RDEPLYEFSFC(+57.02)SVQEHR.R | Y | 99.81 | 2197.9851 | 17 | -0.1 | 733.6689 | 3 | 23.72 | 1 | 12007 | BuCaMH04.raw | 1.5787E8 | 1 | 1 | 369 | 385 | Carbamidomethylation | C11:Carbamidomethylation:1000.00 | PEAKS DB |
| R.DEPLYEFSFC(+57.02)SVQEHR.R | Y | 91.20 | 2041.8839 | 16 | 0.3 | 1021.9496 | 2 | 33.37 | 1 | 20997 | BuCaMH04.raw | 3.3779E7 | 2 | 2 | 370 | 385 | Carbamidomethylation | C10:Carbamidomethylation:1000.00 | PEAKS DB |
| R.DRPQC(+57.02)ILNKPLSTNIVAPPVC(+57.02)GNYFVEVGEEC(+57.02)DC(+57.02)GSPR.D | Y | 90.74 | 4347.0181 | 38 | 0.6 | 1087.7625 | 4 | 34.28 | 1 | 21702 | BuCaMH04.raw | 2.1349E8 | 1 | 1 | 391 | 428 | Carbamidomethylation | C5:Carbamidomethylation:1000.00;C21:Carbamidomethylation:1000.00;C32:Carbamidomethylation:1000.00;C34:Carbamidomethylation:1000.00 | PEAKS DB |
| R.VYELVNILNMIFR.R | Y | 86.50 | 1622.8854 | 13 | 0.7 | 812.4506 | 2 | 75.30 | 1 | 57144 | BuCaMH04.raw | 3.6997E5 | 1 | 1 | 232 | 244 |  |  | PEAKS DB |
| K.C(+57.02)PTLTNQC(+57.02)IALMGPNVK.A | N | 82.56 | 1915.9318 | 17 | 1.0 | 958.9741 | 2 | 24.20 | 1 | 12489 | BuCaMH04.raw | 1.1923E7 | 1 | 1 | 510 | 526 | Carbamidomethylation | C1:Carbamidomethylation:1000.00;C8:Carbamidomethylation:1000.00 | PEAKS DB |
| N.YFVEVGEEC(+57.02)DC(+57.02)GSPR.D | N | 82.16 | 1802.7240 | 15 | 0.5 | 902.3698 | 2 | 18.36 | 1 | 7501 | BuCaMH04.raw | 1.0067E8 | 1 | 1 | 414 | 428 | Carbamidomethylation | C9:Carbamidomethylation:1000.00;C11:Carbamidomethylation:1000.00 | PEAKS DB |
| C.LILPDDPNYGMVETGTK.C | N | 80.12 | 1861.9131 | 17 | 0.2 | 931.9640 | 2 | 27.95 | 1 | 15970 | BuCaMH04.raw | 3.1118E6 | 1 | 1 | 578 | 594 |  |  | PEAKS DB |
| V.APPVC(+57.02)GNYFVEVGEEC(+57.02)DC(+57.02)GSPR.D | Y | 64.33 | 2498.0300 | 22 | 0.3 | 1250.0227 | 2 | 26.72 | 1 | 14695 | BuCaMH04.raw | 1.1803E6 | 1 | 1 | 407 | 428 | Carbamidomethylation | C5:Carbamidomethylation:1000.00;C16:Carbamidomethylation:1000.00;C18:Carbamidomethylation:1000.00 | PEAKS DB |
| total 8 peptides |  |  |  |  |  |  |  |  |  |  |  |  |  |  |  |  |  |  |  |
| --- | --- | --- | --- | --- | --- | --- | --- | --- | --- | --- | --- | --- | --- | --- | --- | --- | --- | --- | --- |

T\_112

back to list

  

| Protein Coverage
| Supporting Peptides
|

Protein Coverage:

Supporting Peptides:

| Peptide | Uniq | -10lgP | Mass | Length | ppm | m/z | z | RT | Fraction | Scan | Source File | Area BuCaMH04\_B15 | #Feature | #Feature BuCaMH04\_B15 | Start | End | PTM | AScore | Found By |
| --- | --- | --- | --- | --- | --- | --- | --- | --- | --- | --- | --- | --- | --- | --- | --- | --- | --- | --- | --- |
| K.RDEPLYEFSFC(+57.02)SVQEHR.R | Y | 99.81 | 2197.9851 | 17 | -0.1 | 733.6689 | 3 | 23.72 | 1 | 12007 | BuCaMH04.raw | 1.5787E8 | 1 | 1 | 402 | 418 | Carbamidomethylation | C11:Carbamidomethylation:1000.00 | PEAKS DB |
| R.DEPLYEFSFC(+57.02)SVQEHR.R | Y | 91.20 | 2041.8839 | 16 | 0.3 | 1021.9496 | 2 | 33.37 | 1 | 20997 | BuCaMH04.raw | 3.3779E7 | 2 | 2 | 403 | 418 | Carbamidomethylation | C10:Carbamidomethylation:1000.00 | PEAKS DB |
| R.DRPQC(+57.02)ILNKPLSTNIVAPPVC(+57.02)GNYFVEVGEEC(+57.02)DC(+57.02)GSPR.D | Y | 90.74 | 4347.0181 | 38 | 0.6 | 1087.7625 | 4 | 34.28 | 1 | 21702 | BuCaMH04.raw | 2.1349E8 | 1 | 1 | 424 | 461 | Carbamidomethylation | C5:Carbamidomethylation:1000.00;C21:Carbamidomethylation:1000.00;C32:Carbamidomethylation:1000.00;C34:Carbamidomethylation:1000.00 | PEAKS DB |
| R.VYELVNILNMIFR.R | Y | 86.50 | 1622.8854 | 13 | 0.7 | 812.4506 | 2 | 75.30 | 1 | 57144 | BuCaMH04.raw | 3.6997E5 | 1 | 1 | 265 | 277 |  |  | PEAKS DB |
| K.C(+57.02)PTLTNQC(+57.02)IALMGPNVK.A | N | 82.56 | 1915.9318 | 17 | 1.0 | 958.9741 | 2 | 24.20 | 1 | 12489 | BuCaMH04.raw | 1.1923E7 | 1 | 1 | 543 | 559 | Carbamidomethylation | C1:Carbamidomethylation:1000.00;C8:Carbamidomethylation:1000.00 | PEAKS DB |
| N.YFVEVGEEC(+57.02)DC(+57.02)GSPR.D | N | 82.16 | 1802.7240 | 15 | 0.5 | 902.3698 | 2 | 18.36 | 1 | 7501 | BuCaMH04.raw | 1.0067E8 | 1 | 1 | 447 | 461 | Carbamidomethylation | C9:Carbamidomethylation:1000.00;C11:Carbamidomethylation:1000.00 | PEAKS DB |
| C.LILPDDPNYGMVETGTK.C | N | 80.12 | 1861.9131 | 17 | 0.2 | 931.9640 | 2 | 27.95 | 1 | 15970 | BuCaMH04.raw | 3.1118E6 | 1 | 1 | 611 | 627 |  |  | PEAKS DB |
| V.APPVC(+57.02)GNYFVEVGEEC(+57.02)DC(+57.02)GSPR.D | Y | 64.33 | 2498.0300 | 22 | 0.3 | 1250.0227 | 2 | 26.72 | 1 | 14695 | BuCaMH04.raw | 1.1803E6 | 1 | 1 | 440 | 461 | Carbamidomethylation | C5:Carbamidomethylation:1000.00;C16:Carbamidomethylation:1000.00;C18:Carbamidomethylation:1000.00 | PEAKS DB |
| total 8 peptides |  |  |  |  |  |  |  |  |  |  |  |  |  |  |  |  |  |  |  |
| --- | --- | --- | --- | --- | --- | --- | --- | --- | --- | --- | --- | --- | --- | --- | --- | --- | --- | --- | --- |

P82942.1

back to list

  

| Protein Coverage
| Supporting Peptides
|

Protein Coverage:

Supporting Peptides:

| Peptide | Uniq | -10lgP | Mass | Length | ppm | m/z | z | RT | Fraction | Scan | Source File | Area BuCaMH04\_B15 | #Feature | #Feature BuCaMH04\_B15 | Start | End | PTM | AScore | Found By |
| --- | --- | --- | --- | --- | --- | --- | --- | --- | --- | --- | --- | --- | --- | --- | --- | --- | --- | --- | --- |
| R.TAPAFQFSSC(+57.02)SIR.D | N | 103.50 | 1470.6925 | 13 | 0.6 | 736.3540 | 2 | 22.56 | 1 | 11205 | BuCaMH04.raw | 3.1767E8 | 1 | 1 | 178 | 190 | Carbamidomethylation | C10:Carbamidomethylation:1000.00 | PEAKS DB |
| K.YIEFYVIVDNR.M | N | 97.02 | 1429.7241 | 11 | 1.0 | 715.8701 | 2 | 39.02 | 1 | 25913 | BuCaMH04.raw | 2.6888E7 | 10 | 10 | 15 | 25 |  |  | PEAKS DB |
| K.HDC(+57.02)DLPELC(+57.02)TGQSAEC(+57.02)PTDSLQR.N | Y | 94.92 | 2688.1213 | 23 | 0.1 | 897.0479 | 3 | 18.90 | 1 | 7858 | BuCaMH04.raw | 4.6386E7 | 1 | 1 | 262 | 284 | Carbamidomethylation | C3:Carbamidomethylation:1000.00;C9:Carbamidomethylation:1000.00;C16:Carbamidomethylation:1000.00 | PEAKS DB |
| K.C(+57.02)PTLTNQC(+57.02)IALLGPHFTVSPK.G | Y | 72.88 | 2353.1921 | 21 | 0.8 | 785.4053 | 3 | 30.26 | 1 | 18109 | BuCaMH04.raw | 2.2752E7 | 1 | 1 | 301 | 321 | Carbamidomethylation | C1:Carbamidomethylation:1000.00;C8:Carbamidomethylation:1000.00 | PEAKS DB |
| Y.IEFYVIVDNR.M | N | 65.61 | 1266.6608 | 10 | 0.2 | 634.3378 | 2 | 32.73 | 1 | 21088 | BuCaMH04.raw | 4.3601E7 | 1 | 1 | 16 | 25 |  |  | PEAKS DB |
| I.EFYVIVDNR.M | N | 62.40 | 1153.5768 | 9 | 0.4 | 577.7959 | 2 | 23.35 | 1 | 11810 | BuCaMH04.raw | 3.851E6 | 1 | 1 | 17 | 25 |  |  | PEAKS DB |
| R.TAPAFQFSSC(+57.02)SIRDYQEYLLR.D | Y | 60.50 | 2551.2166 | 21 | 0.3 | 851.4131 | 3 | 40.81 | 1 | 27524 | BuCaMH04.raw | 3.1753E6 | 1 | 1 | 178 | 198 | Carbamidomethylation | C10:Carbamidomethylation:1000.00 | PEAKS DB |
| K.IHIALIGLEIWSNEDKFEVKP.A | Y | 58.11 | 2450.3208 | 21 | 0.2 | 613.5876 | 4 | 44.08 | 1 | 30512 | BuCaMH04.raw | 4.5999E6 | 2 | 2 | 57 | 77 |  |  | PEAKS DB |
| K.IHIALIGLEIWSNEDKFEVK.P | Y | 58.08 | 2353.2681 | 20 | 0.1 | 785.4301 | 3 | 43.44 | 1 | 29951 | BuCaMH04.raw | 1.1719E6 | 1 | 1 | 57 | 76 |  |  | PEAKS DB |
| K.IHIALIGLEIWSNEDK.F | Y | 57.35 | 1849.9938 | 16 | 0.6 | 617.6722 | 3 | 44.14 | 1 | 30556 | BuCaMH04.raw | 3.5497E6 | 1 | 1 | 57 | 72 |  |  | PEAKS DB |
| R.DYQEYLLR.D | Y | 56.42 | 1098.5345 | 8 | -0.1 | 550.2745 | 2 | 25.51 | 1 | 13573 | BuCaMH04.raw | 2.5952E7 | 1 | 1 | 191 | 198 |  |  | PEAKS DB |
| total 11 peptides |  |  |  |  |  |  |  |  |  |  |  |  |  |  |  |  |  |  |  |
| --- | --- | --- | --- | --- | --- | --- | --- | --- | --- | --- | --- | --- | --- | --- | --- | --- | --- | --- | --- |

T\_113

back to list

  

| Protein Coverage
| Supporting Peptides
|

Protein Coverage:

Supporting Peptides:

| Peptide | Uniq | -10lgP | Mass | Length | ppm | m/z | z | RT | Fraction | Scan | Source File | Area BuCaMH04\_B15 | #Feature | #Feature BuCaMH04\_B15 | Start | End | PTM | AScore | Found By |
| --- | --- | --- | --- | --- | --- | --- | --- | --- | --- | --- | --- | --- | --- | --- | --- | --- | --- | --- | --- |
| F.NGNTVGLAALGSLC(+57.02)SVK.Y | N | 96.93 | 1659.8613 | 17 | 0.9 | 830.9387 | 2 | 42.94 | 1 | 29615 | BuCaMH04.raw | 1.323E7 | 1 | 1 | 140 | 156 | Carbamidomethylation | C14:Carbamidomethylation:1000.00 | PEAKS DB |
| K.YIELYMAVDNR.M | Y | 91.04 | 1385.6649 | 11 | 1.0 | 693.8405 | 2 | 29.37 | 1 | 17347 | BuCaMH04.raw | 3.7674E7 | 1 | 1 | 43 | 53 |  |  | PEAKS DB |
| K.C(+57.02)PTLTNQC(+57.02)IALMGPNVK.A | N | 82.56 | 1915.9318 | 17 | 1.0 | 958.9741 | 2 | 24.20 | 1 | 12489 | BuCaMH04.raw | 1.1923E7 | 1 | 1 | 348 | 364 | Carbamidomethylation | C1:Carbamidomethylation:1000.00;C8:Carbamidomethylation:1000.00 | PEAKS DB |
| N.YFVEVGEEC(+57.02)DC(+57.02)GSPR.D | N | 82.16 | 1802.7240 | 15 | 0.5 | 902.3698 | 2 | 18.36 | 1 | 7501 | BuCaMH04.raw | 1.0067E8 | 1 | 1 | 252 | 266 | Carbamidomethylation | C9:Carbamidomethylation:1000.00;C11:Carbamidomethylation:1000.00 | PEAKS DB |
| K.YSVAVIQDYSK.R | N | 80.81 | 1271.6398 | 11 | 0.4 | 636.8274 | 2 | 19.99 | 1 | 8958 | BuCaMH04.raw | 9.4287E7 | 1 | 1 | 157 | 167 |  |  | PEAKS DB |
| C.LILPDDPNYGMVETGTK.C | N | 80.12 | 1861.9131 | 17 | 0.2 | 931.9640 | 2 | 27.95 | 1 | 15970 | BuCaMH04.raw | 3.1118E6 | 1 | 1 | 416 | 432 |  |  | PEAKS DB |
| N.GNTVGLAALGSLC(+57.02)SVK.Y | N | 72.59 | 1545.8185 | 16 | 0.0 | 773.9165 | 2 | 30.49 | 1 | 18460 | BuCaMH04.raw | 5.7108E8 | 3 | 3 | 141 | 156 | Carbamidomethylation | C13:Carbamidomethylation:1000.00 | PEAKS DB |
| K.VYGLVNHLNMIYK.T | Y | 52.25 | 1562.8279 | 13 | 0.3 | 521.9501 | 3 | 23.86 | 1 | 12172 | BuCaMH04.raw | 1.3352E6 | 1 | 1 | 69 | 81 |  |  | PEAKS DB |
| total 8 peptides |  |  |  |  |  |  |  |  |  |  |  |  |  |  |  |  |  |  |  |
| --- | --- | --- | --- | --- | --- | --- | --- | --- | --- | --- | --- | --- | --- | --- | --- | --- | --- | --- | --- |

ASU45022.1

back to list

  

| Protein Coverage
| Supporting Peptides
|

Protein Coverage:

Supporting Peptides:

| Peptide | Uniq | -10lgP | Mass | Length | ppm | m/z | z | RT | Fraction | Scan | Source File | Area BuCaMH04\_B15 | #Feature | #Feature BuCaMH04\_B15 | Start | End | PTM | AScore | Found By |
| --- | --- | --- | --- | --- | --- | --- | --- | --- | --- | --- | --- | --- | --- | --- | --- | --- | --- | --- | --- |
| R.FLTYTQNVILVSLSYR.V | N | 114.43 | 1916.0408 | 16 | -0.1 | 959.0276 | 2 | 48.85 | 1 | 34834 | BuCaMH04.raw | 2.6107E7 | 2 | 2 | 157 | 172 |  |  | PEAKS DB |
| Q.LIDEEWSVLPYK.S | N | 89.30 | 1490.7656 | 12 | 0.9 | 746.3907 | 2 | 37.26 | 1 | 24455 | BuCaMH04.raw | 2.1123E7 | 1 | 1 | 297 | 308 |  |  | PEAKS DB |
| A.FLGIPFAEPPVGR.L | N | 70.62 | 1398.7659 | 13 | 0.9 | 700.3909 | 2 | 42.12 | 1 | 28605 | BuCaMH04.raw | 1.1387E8 | 1 | 1 | 53 | 65 |  |  | PEAKS DB |
| I.C(+57.02)AFWNHFLPK.L | N | 67.68 | 1318.6281 | 10 | 0.2 | 440.5500 | 3 | 27.24 | 1 | 15136 | BuCaMH04.raw | 4.6147E6 | 1 | 1 | 544 | 553 | Carbamidomethylation | C1:Carbamidomethylation:1000.00 | PEAKS DB |
| F.LGIPFAEPPVGR.L | N | 62.25 | 1251.6975 | 12 | 0.5 | 626.8563 | 2 | 29.35 | 1 | 17318 | BuCaMH04.raw | 6.6683E7 | 1 | 1 | 54 | 65 |  |  | PEAKS DB |
| R.AQIC(+57.02)AFWNHFLPK.L | N | 60.48 | 1630.8079 | 13 | 1.0 | 544.6104 | 3 | 36.94 | 1 | 23938 | BuCaMH04.raw | 1.8602E6 | 1 | 1 | 541 | 553 | Carbamidomethylation | C4:Carbamidomethylation:1000.00 | PEAKS DB |
| S.AFLGIPFAEPPVGR.L | N | 58.55 | 1469.8030 | 14 | 0.6 | 735.9092 | 2 | 43.95 | 1 | 30470 | BuCaMH04.raw | 5.0781E6 | 1 | 1 | 52 | 65 |  |  | PEAKS DB |
| L.IDEEWSVLPYK.S | N | 57.66 | 1377.6816 | 11 | 0.6 | 689.8485 | 2 | 33.06 | 1 | 20610 | BuCaMH04.raw | 2.6958E6 | 1 | 1 | 298 | 308 |  |  | PEAKS DB |
| total 8 peptides |  |  |  |  |  |  |  |  |  |  |  |  |  |  |  |  |  |  |  |
| --- | --- | --- | --- | --- | --- | --- | --- | --- | --- | --- | --- | --- | --- | --- | --- | --- | --- | --- | --- |

XP\_015668657.1

back to list

  

| Protein Coverage
| Supporting Peptides
|

Protein Coverage:

Supporting Peptides:

| Peptide | Uniq | -10lgP | Mass | Length | ppm | m/z | z | RT | Fraction | Scan | Source File | Area BuCaMH04\_B15 | #Feature | #Feature BuCaMH04\_B15 | Start | End | PTM | AScore | Found By |
| --- | --- | --- | --- | --- | --- | --- | --- | --- | --- | --- | --- | --- | --- | --- | --- | --- | --- | --- | --- |
| R.FLTYTQNVILVSLSYR.V | N | 114.43 | 1916.0408 | 16 | -0.1 | 959.0276 | 2 | 48.85 | 1 | 34834 | BuCaMH04.raw | 2.6107E7 | 2 | 2 | 168 | 183 |  |  | PEAKS DB |
| Q.LIDEEWSVLPYK.S | N | 89.30 | 1490.7656 | 12 | 0.9 | 746.3907 | 2 | 37.26 | 1 | 24455 | BuCaMH04.raw | 2.1123E7 | 1 | 1 | 308 | 319 |  |  | PEAKS DB |
| A.FLGIPFAEPPVGR.L | N | 70.62 | 1398.7659 | 13 | 0.9 | 700.3909 | 2 | 42.12 | 1 | 28605 | BuCaMH04.raw | 1.1387E8 | 1 | 1 | 64 | 76 |  |  | PEAKS DB |
| I.C(+57.02)AFWNHFLPK.L | N | 67.68 | 1318.6281 | 10 | 0.2 | 440.5500 | 3 | 27.24 | 1 | 15136 | BuCaMH04.raw | 4.6147E6 | 1 | 1 | 555 | 564 | Carbamidomethylation | C1:Carbamidomethylation:1000.00 | PEAKS DB |
| F.LGIPFAEPPVGR.L | N | 62.25 | 1251.6975 | 12 | 0.5 | 626.8563 | 2 | 29.35 | 1 | 17318 | BuCaMH04.raw | 6.6683E7 | 1 | 1 | 65 | 76 |  |  | PEAKS DB |
| S.AFLGIPFAEPPVGR.L | N | 58.55 | 1469.8030 | 14 | 0.6 | 735.9092 | 2 | 43.95 | 1 | 30470 | BuCaMH04.raw | 5.0781E6 | 1 | 1 | 63 | 76 |  |  | PEAKS DB |
| L.IDEEWSVLPYK.S | N | 57.66 | 1377.6816 | 11 | 0.6 | 689.8485 | 2 | 33.06 | 1 | 20610 | BuCaMH04.raw | 2.6958E6 | 1 | 1 | 309 | 319 |  |  | PEAKS DB |
| total 7 peptides |  |  |  |  |  |  |  |  |  |  |  |  |  |  |  |  |  |  |  |
| --- | --- | --- | --- | --- | --- | --- | --- | --- | --- | --- | --- | --- | --- | --- | --- | --- | --- | --- | --- |

ADD14036.1

back to list

  

| Protein Coverage
| Supporting Peptides
|

Protein Coverage:

Supporting Peptides:

| Peptide | Uniq | -10lgP | Mass | Length | ppm | m/z | z | RT | Fraction | Scan | Source File | Area BuCaMH04\_B15 | #Feature | #Feature BuCaMH04\_B15 | Start | End | PTM | AScore | Found By |
| --- | --- | --- | --- | --- | --- | --- | --- | --- | --- | --- | --- | --- | --- | --- | --- | --- | --- | --- | --- |
| R.TAPAFQFSSC(+57.02)SIR.E | N | 103.50 | 1470.6925 | 13 | 0.6 | 736.3540 | 2 | 22.56 | 1 | 11205 | BuCaMH04.raw | 3.1767E8 | 1 | 1 | 179 | 191 | Carbamidomethylation | C10:Carbamidomethylation:1000.00 | PEAKS DB |
| K.YIEFYVIVDNR.M | N | 97.02 | 1429.7241 | 11 | 1.0 | 715.8701 | 2 | 39.02 | 1 | 25913 | BuCaMH04.raw | 2.6888E7 | 10 | 10 | 15 | 25 |  |  | PEAKS DB |
| R.NDNAQLLTGIDFNGNTVGR.A | Y | 84.17 | 2017.9817 | 19 | 0.0 | 1009.9981 | 2 | 32.28 | 1 | 19864 | BuCaMH04.raw | 1.2605E7 | 1 | 1 | 100 | 118 |  |  | PEAKS DB |
| K.RNDNAQLLTGIDFNGNTVGR.A | Y | 75.40 | 2174.0828 | 20 | -1.0 | 725.7008 | 3 | 24.27 | 1 | 12540 | BuCaMH04.raw | 1.3437E6 | 1 | 1 | 99 | 118 |  |  | PEAKS DB |
| Y.IEFYVIVDNR.M | N | 65.61 | 1266.6608 | 10 | 0.2 | 634.3378 | 2 | 32.73 | 1 | 21088 | BuCaMH04.raw | 4.3601E7 | 1 | 1 | 16 | 25 |  |  | PEAKS DB |
| I.EFYVIVDNR.M | N | 62.40 | 1153.5768 | 9 | 0.4 | 577.7959 | 2 | 23.35 | 1 | 11810 | BuCaMH04.raw | 3.851E6 | 1 | 1 | 17 | 25 |  |  | PEAKS DB |
| L.IGLEIWSNK.D | N | 50.68 | 1058.5760 | 9 | 0.6 | 530.2956 | 2 | 27.50 | 1 | 15397 | BuCaMH04.raw | 8.2315E6 | 1 | 1 | 62 | 70 |  |  | PEAKS DB |
| total 7 peptides |  |  |  |  |  |  |  |  |  |  |  |  |  |  |  |  |  |  |  |
| --- | --- | --- | --- | --- | --- | --- | --- | --- | --- | --- | --- | --- | --- | --- | --- | --- | --- | --- | --- |

D6PXE8.1

back to list

  

| Protein Coverage
| Supporting Peptides
|

Protein Coverage:

Supporting Peptides:

| Peptide | Uniq | -10lgP | Mass | Length | ppm | m/z | z | RT | Fraction | Scan | Source File | Area BuCaMH04\_B15 | #Feature | #Feature BuCaMH04\_B15 | Start | End | PTM | AScore | Found By |
| --- | --- | --- | --- | --- | --- | --- | --- | --- | --- | --- | --- | --- | --- | --- | --- | --- | --- | --- | --- |
| R.TAPAFQFSSC(+57.02)SIR.E | N | 103.50 | 1470.6925 | 13 | 0.6 | 736.3540 | 2 | 22.56 | 1 | 11205 | BuCaMH04.raw | 3.1767E8 | 1 | 1 | 370 | 382 | Carbamidomethylation | C10:Carbamidomethylation:1000.00 | PEAKS DB |
| K.YIEFYVIVDNR.M | N | 97.02 | 1429.7241 | 11 | 1.0 | 715.8701 | 2 | 39.02 | 1 | 25913 | BuCaMH04.raw | 2.6888E7 | 10 | 10 | 206 | 216 |  |  | PEAKS DB |
| R.NDNAQLLTGIDFNGNTVGR.A | Y | 84.17 | 2017.9817 | 19 | 0.0 | 1009.9981 | 2 | 32.28 | 1 | 19864 | BuCaMH04.raw | 1.2605E7 | 1 | 1 | 291 | 309 |  |  | PEAKS DB |
| K.RNDNAQLLTGIDFNGNTVGR.A | Y | 75.40 | 2174.0828 | 20 | -1.0 | 725.7008 | 3 | 24.27 | 1 | 12540 | BuCaMH04.raw | 1.3437E6 | 1 | 1 | 290 | 309 |  |  | PEAKS DB |
| Y.IEFYVIVDNR.M | N | 65.61 | 1266.6608 | 10 | 0.2 | 634.3378 | 2 | 32.73 | 1 | 21088 | BuCaMH04.raw | 4.3601E7 | 1 | 1 | 207 | 216 |  |  | PEAKS DB |
| I.EFYVIVDNR.M | N | 62.40 | 1153.5768 | 9 | 0.4 | 577.7959 | 2 | 23.35 | 1 | 11810 | BuCaMH04.raw | 3.851E6 | 1 | 1 | 208 | 216 |  |  | PEAKS DB |
| L.IGLEIWSNK.D | N | 50.68 | 1058.5760 | 9 | 0.6 | 530.2956 | 2 | 27.50 | 1 | 15397 | BuCaMH04.raw | 8.2315E6 | 1 | 1 | 253 | 261 |  |  | PEAKS DB |
| total 7 peptides |  |  |  |  |  |  |  |  |  |  |  |  |  |  |  |  |  |  |  |
| --- | --- | --- | --- | --- | --- | --- | --- | --- | --- | --- | --- | --- | --- | --- | --- | --- | --- | --- | --- |

ADG02948.1

back to list

  

| Protein Coverage
| Supporting Peptides
|

Protein Coverage:

Supporting Peptides:

| Peptide | Uniq | -10lgP | Mass | Length | ppm | m/z | z | RT | Fraction | Scan | Source File | Area BuCaMH04\_B15 | #Feature | #Feature BuCaMH04\_B15 | Start | End | PTM | AScore | Found By |
| --- | --- | --- | --- | --- | --- | --- | --- | --- | --- | --- | --- | --- | --- | --- | --- | --- | --- | --- | --- |
| R.TAPAFQFSSC(+57.02)SIR.E | N | 103.50 | 1470.6925 | 13 | 0.6 | 736.3540 | 2 | 22.56 | 1 | 11205 | BuCaMH04.raw | 3.1767E8 | 1 | 1 | 370 | 382 | Carbamidomethylation | C10:Carbamidomethylation:1000.00 | PEAKS DB |
| K.YIEFYVIVDNR.M | N | 97.02 | 1429.7241 | 11 | 1.0 | 715.8701 | 2 | 39.02 | 1 | 25913 | BuCaMH04.raw | 2.6888E7 | 10 | 10 | 206 | 216 |  |  | PEAKS DB |
| R.NDNAQLLTGIDFNGNTVGR.A | Y | 84.17 | 2017.9817 | 19 | 0.0 | 1009.9981 | 2 | 32.28 | 1 | 19864 | BuCaMH04.raw | 1.2605E7 | 1 | 1 | 291 | 309 |  |  | PEAKS DB |
| K.RNDNAQLLTGIDFNGNTVGR.A | Y | 75.40 | 2174.0828 | 20 | -1.0 | 725.7008 | 3 | 24.27 | 1 | 12540 | BuCaMH04.raw | 1.3437E6 | 1 | 1 | 290 | 309 |  |  | PEAKS DB |
| Y.IEFYVIVDNR.M | N | 65.61 | 1266.6608 | 10 | 0.2 | 634.3378 | 2 | 32.73 | 1 | 21088 | BuCaMH04.raw | 4.3601E7 | 1 | 1 | 207 | 216 |  |  | PEAKS DB |
| I.EFYVIVDNR.M | N | 62.40 | 1153.5768 | 9 | 0.4 | 577.7959 | 2 | 23.35 | 1 | 11810 | BuCaMH04.raw | 3.851E6 | 1 | 1 | 208 | 216 |  |  | PEAKS DB |
| L.IGLEIWSNK.D | N | 50.68 | 1058.5760 | 9 | 0.6 | 530.2956 | 2 | 27.50 | 1 | 15397 | BuCaMH04.raw | 8.2315E6 | 1 | 1 | 253 | 261 |  |  | PEAKS DB |
| total 7 peptides |  |  |  |  |  |  |  |  |  |  |  |  |  |  |  |  |  |  |  |
| --- | --- | --- | --- | --- | --- | --- | --- | --- | --- | --- | --- | --- | --- | --- | --- | --- | --- | --- | --- |

3K7N

back to list

  

| Protein Coverage
| Supporting Peptides
|

Protein Coverage:

Supporting Peptides:

| Peptide | Uniq | -10lgP | Mass | Length | ppm | m/z | z | RT | Fraction | Scan | Source File | Area BuCaMH04\_B15 | #Feature | #Feature BuCaMH04\_B15 | Start | End | PTM | AScore | Found By |
| --- | --- | --- | --- | --- | --- | --- | --- | --- | --- | --- | --- | --- | --- | --- | --- | --- | --- | --- | --- |
| R.TAPAFQFSSC(+57.02)SIR.E | N | 103.50 | 1470.6925 | 13 | 0.6 | 736.3540 | 2 | 22.56 | 1 | 11205 | BuCaMH04.raw | 3.1767E8 | 1 | 1 | 174 | 186 | Carbamidomethylation | C10:Carbamidomethylation:1000.00 | PEAKS DB |
| K.YIEFYVIVDNR.M | N | 97.02 | 1429.7241 | 11 | 1.0 | 715.8701 | 2 | 39.02 | 1 | 25913 | BuCaMH04.raw | 2.6888E7 | 10 | 10 | 10 | 20 |  |  | PEAKS DB |
| R.NDNAQLLTGIDFNGNTVGR.A | Y | 84.17 | 2017.9817 | 19 | 0.0 | 1009.9981 | 2 | 32.28 | 1 | 19864 | BuCaMH04.raw | 1.2605E7 | 1 | 1 | 95 | 113 |  |  | PEAKS DB |
| K.RNDNAQLLTGIDFNGNTVGR.A | Y | 75.40 | 2174.0828 | 20 | -1.0 | 725.7008 | 3 | 24.27 | 1 | 12540 | BuCaMH04.raw | 1.3437E6 | 1 | 1 | 94 | 113 |  |  | PEAKS DB |
| Y.IEFYVIVDNR.M | N | 65.61 | 1266.6608 | 10 | 0.2 | 634.3378 | 2 | 32.73 | 1 | 21088 | BuCaMH04.raw | 4.3601E7 | 1 | 1 | 11 | 20 |  |  | PEAKS DB |
| I.EFYVIVDNR.M | N | 62.40 | 1153.5768 | 9 | 0.4 | 577.7959 | 2 | 23.35 | 1 | 11810 | BuCaMH04.raw | 3.851E6 | 1 | 1 | 12 | 20 |  |  | PEAKS DB |
| L.IGLEIWSNK.D | N | 50.68 | 1058.5760 | 9 | 0.6 | 530.2956 | 2 | 27.50 | 1 | 15397 | BuCaMH04.raw | 8.2315E6 | 1 | 1 | 57 | 65 |  |  | PEAKS DB |
| total 7 peptides |  |  |  |  |  |  |  |  |  |  |  |  |  |  |  |  |  |  |  |
| --- | --- | --- | --- | --- | --- | --- | --- | --- | --- | --- | --- | --- | --- | --- | --- | --- | --- | --- | --- |

ACN50005.1

back to list

  

| Protein Coverage
| Supporting Peptides
|

Protein Coverage:

Supporting Peptides:

| Peptide | Uniq | -10lgP | Mass | Length | ppm | m/z | z | RT | Fraction | Scan | Source File | Area BuCaMH04\_B15 | #Feature | #Feature BuCaMH04\_B15 | Start | End | PTM | AScore | Found By |
| --- | --- | --- | --- | --- | --- | --- | --- | --- | --- | --- | --- | --- | --- | --- | --- | --- | --- | --- | --- |
| R.TAPAFQFSSC(+57.02)SIR.E | N | 103.50 | 1470.6925 | 13 | 0.6 | 736.3540 | 2 | 22.56 | 1 | 11205 | BuCaMH04.raw | 3.1767E8 | 1 | 1 | 370 | 382 | Carbamidomethylation | C10:Carbamidomethylation:1000.00 | PEAKS DB |
| K.YIEFYVIVDNR.M | N | 97.02 | 1429.7241 | 11 | 1.0 | 715.8701 | 2 | 39.02 | 1 | 25913 | BuCaMH04.raw | 2.6888E7 | 10 | 10 | 206 | 216 |  |  | PEAKS DB |
| R.NDNAQLLTGIDFNGNTVGR.A | Y | 84.17 | 2017.9817 | 19 | 0.0 | 1009.9981 | 2 | 32.28 | 1 | 19864 | BuCaMH04.raw | 1.2605E7 | 1 | 1 | 291 | 309 |  |  | PEAKS DB |
| K.RNDNAQLLTGIDFNGNTVGR.A | Y | 75.40 | 2174.0828 | 20 | -1.0 | 725.7008 | 3 | 24.27 | 1 | 12540 | BuCaMH04.raw | 1.3437E6 | 1 | 1 | 290 | 309 |  |  | PEAKS DB |
| Y.IEFYVIVDNR.M | N | 65.61 | 1266.6608 | 10 | 0.2 | 634.3378 | 2 | 32.73 | 1 | 21088 | BuCaMH04.raw | 4.3601E7 | 1 | 1 | 207 | 216 |  |  | PEAKS DB |
| I.EFYVIVDNR.M | N | 62.40 | 1153.5768 | 9 | 0.4 | 577.7959 | 2 | 23.35 | 1 | 11810 | BuCaMH04.raw | 3.851E6 | 1 | 1 | 208 | 216 |  |  | PEAKS DB |
| L.IGLEIWSNK.D | N | 50.68 | 1058.5760 | 9 | 0.6 | 530.2956 | 2 | 27.50 | 1 | 15397 | BuCaMH04.raw | 8.2315E6 | 1 | 1 | 253 | 261 |  |  | PEAKS DB |
| total 7 peptides |  |  |  |  |  |  |  |  |  |  |  |  |  |  |  |  |  |  |  |
| --- | --- | --- | --- | --- | --- | --- | --- | --- | --- | --- | --- | --- | --- | --- | --- | --- | --- | --- | --- |

D3TTC1.1

back to list

  

| Protein Coverage
| Supporting Peptides
|

Protein Coverage:

Supporting Peptides:

| Peptide | Uniq | -10lgP | Mass | Length | ppm | m/z | z | RT | Fraction | Scan | Source File | Area BuCaMH04\_B15 | #Feature | #Feature BuCaMH04\_B15 | Start | End | PTM | AScore | Found By |
| --- | --- | --- | --- | --- | --- | --- | --- | --- | --- | --- | --- | --- | --- | --- | --- | --- | --- | --- | --- |
| R.TAPAFQFSSC(+57.02)SIR.E | N | 103.50 | 1470.6925 | 13 | 0.6 | 736.3540 | 2 | 22.56 | 1 | 11205 | BuCaMH04.raw | 3.1767E8 | 1 | 1 | 370 | 382 | Carbamidomethylation | C10:Carbamidomethylation:1000.00 | PEAKS DB |
| K.YIEFYVIVDNR.M | N | 97.02 | 1429.7241 | 11 | 1.0 | 715.8701 | 2 | 39.02 | 1 | 25913 | BuCaMH04.raw | 2.6888E7 | 10 | 10 | 206 | 216 |  |  | PEAKS DB |
| R.NDNAQLLTGIDFNGNTVGR.A | Y | 84.17 | 2017.9817 | 19 | 0.0 | 1009.9981 | 2 | 32.28 | 1 | 19864 | BuCaMH04.raw | 1.2605E7 | 1 | 1 | 291 | 309 |  |  | PEAKS DB |
| K.RNDNAQLLTGIDFNGNTVGR.A | Y | 75.40 | 2174.0828 | 20 | -1.0 | 725.7008 | 3 | 24.27 | 1 | 12540 | BuCaMH04.raw | 1.3437E6 | 1 | 1 | 290 | 309 |  |  | PEAKS DB |
| Y.IEFYVIVDNR.M | N | 65.61 | 1266.6608 | 10 | 0.2 | 634.3378 | 2 | 32.73 | 1 | 21088 | BuCaMH04.raw | 4.3601E7 | 1 | 1 | 207 | 216 |  |  | PEAKS DB |
| I.EFYVIVDNR.M | N | 62.40 | 1153.5768 | 9 | 0.4 | 577.7959 | 2 | 23.35 | 1 | 11810 | BuCaMH04.raw | 3.851E6 | 1 | 1 | 208 | 216 |  |  | PEAKS DB |
| L.IGLEIWSNK.D | N | 50.68 | 1058.5760 | 9 | 0.6 | 530.2956 | 2 | 27.50 | 1 | 15397 | BuCaMH04.raw | 8.2315E6 | 1 | 1 | 253 | 261 |  |  | PEAKS DB |
| total 7 peptides |  |  |  |  |  |  |  |  |  |  |  |  |  |  |  |  |  |  |  |
| --- | --- | --- | --- | --- | --- | --- | --- | --- | --- | --- | --- | --- | --- | --- | --- | --- | --- | --- | --- |

5GZ4

back to list

  

| Protein Coverage
| Supporting Peptides
|

Protein Coverage:

Supporting Peptides:

| Peptide | Uniq | -10lgP | Mass | Length | ppm | m/z | z | RT | Fraction | Scan | Source File | Area BuCaMH04\_B15 | #Feature | #Feature BuCaMH04\_B15 | Start | End | PTM | AScore | Found By |
| --- | --- | --- | --- | --- | --- | --- | --- | --- | --- | --- | --- | --- | --- | --- | --- | --- | --- | --- | --- |
| R.DVELLTGLDFYSALKQPLSETLR.L | Y | 106.53 | 2607.3794 | 23 | 1.0 | 870.1346 | 3 | 55.21 | 1 | 40430 | BuCaMH04.raw | 1.5639E6 | 1 | 1 | 795 | 817 |  |  | PEAKS DB |
| K.TFLPIFINSVN | Y | 83.65 | 1263.6863 | 11 | 0.8 | 632.8509 | 2 | 57.34 | 1 | 42335 | BuCaMH04.raw | 1.9991E6 | 1 | 1 | 820 | 830 |  |  | PEAKS DB |
| R.VRDVELLTGLDFYSALK.Q | Y | 76.29 | 1938.0461 | 17 | 0.8 | 647.0232 | 3 | 42.84 | 1 | 29290 | BuCaMH04.raw | 3.868E6 | 1 | 1 | 793 | 809 |  |  | PEAKS DB |
| R.VRDVELLTGLDFYSALKQPLSETLR.L | Y | 76.17 | 2862.5491 | 25 | 0.6 | 716.6450 | 4 | 45.23 | 1 | 31750 | BuCaMH04.raw | 2.8862E6 | 1 | 1 | 793 | 817 |  |  | PEAKS DB |
| K.SMEAIFLAHGPGFK.E | Y | 63.13 | 1503.7544 | 14 | 0.1 | 502.2588 | 3 | 27.04 | 1 | 15068 | BuCaMH04.raw | 1.2312E6 | 2 | 2 | 447 | 460 |  |  | PEAKS DB |
| R.NAAWWGGQPIWHTASYQGLK.A | Y | 57.32 | 2270.1021 | 20 | 1.0 | 757.7087 | 3 | 34.06 | 1 | 21492 | BuCaMH04.raw | 2.1579E6 | 1 | 1 | 203 | 222 |  |  | PEAKS DB |
| R.LWNYFHSTLLPK.Y | Y | 51.18 | 1517.8031 | 12 | 0.6 | 506.9420 | 3 | 29.95 | 1 | 17747 | BuCaMH04.raw | 1.9945E6 | 1 | 1 | 677 | 688 |  |  | PEAKS DB |
| K.DFYTFDSEAIVK.K | Y | 50.66 | 1433.6714 | 12 | 1.3 | 717.8439 | 2 | 35.49 | 1 | 22924 | BuCaMH04.raw | 5.6133E6 | 1 | 1 | 370 | 381 |  |  | PEAKS DB |
| total 8 peptides |  |  |  |  |  |  |  |  |  |  |  |  |  |  |  |  |  |  |  |
| --- | --- | --- | --- | --- | --- | --- | --- | --- | --- | --- | --- | --- | --- | --- | --- | --- | --- | --- | --- |

5GZ5

back to list

  

| Protein Coverage
| Supporting Peptides
|

Protein Coverage:

Supporting Peptides:

| Peptide | Uniq | -10lgP | Mass | Length | ppm | m/z | z | RT | Fraction | Scan | Source File | Area BuCaMH04\_B15 | #Feature | #Feature BuCaMH04\_B15 | Start | End | PTM | AScore | Found By |
| --- | --- | --- | --- | --- | --- | --- | --- | --- | --- | --- | --- | --- | --- | --- | --- | --- | --- | --- | --- |
| R.DVELLTGLDFYSALKQPLSETLR.L | Y | 106.53 | 2607.3794 | 23 | 1.0 | 870.1346 | 3 | 55.21 | 1 | 40430 | BuCaMH04.raw | 1.5639E6 | 1 | 1 | 795 | 817 |  |  | PEAKS DB |
| K.TFLPIFINSVN | Y | 83.65 | 1263.6863 | 11 | 0.8 | 632.8509 | 2 | 57.34 | 1 | 42335 | BuCaMH04.raw | 1.9991E6 | 1 | 1 | 820 | 830 |  |  | PEAKS DB |
| R.VRDVELLTGLDFYSALK.Q | Y | 76.29 | 1938.0461 | 17 | 0.8 | 647.0232 | 3 | 42.84 | 1 | 29290 | BuCaMH04.raw | 3.868E6 | 1 | 1 | 793 | 809 |  |  | PEAKS DB |
| R.VRDVELLTGLDFYSALKQPLSETLR.L | Y | 76.17 | 2862.5491 | 25 | 0.6 | 716.6450 | 4 | 45.23 | 1 | 31750 | BuCaMH04.raw | 2.8862E6 | 1 | 1 | 793 | 817 |  |  | PEAKS DB |
| K.SMEAIFLAHGPGFK.E | Y | 63.13 | 1503.7544 | 14 | 0.1 | 502.2588 | 3 | 27.04 | 1 | 15068 | BuCaMH04.raw | 1.2312E6 | 2 | 2 | 447 | 460 |  |  | PEAKS DB |
| R.NAAWWGGQPIWHTASYQGLK.A | Y | 57.32 | 2270.1021 | 20 | 1.0 | 757.7087 | 3 | 34.06 | 1 | 21492 | BuCaMH04.raw | 2.1579E6 | 1 | 1 | 203 | 222 |  |  | PEAKS DB |
| R.LWNYFHSTLLPK.Y | Y | 51.18 | 1517.8031 | 12 | 0.6 | 506.9420 | 3 | 29.95 | 1 | 17747 | BuCaMH04.raw | 1.9945E6 | 1 | 1 | 677 | 688 |  |  | PEAKS DB |
| K.DFYTFDSEAIVK.K | Y | 50.66 | 1433.6714 | 12 | 1.3 | 717.8439 | 2 | 35.49 | 1 | 22924 | BuCaMH04.raw | 5.6133E6 | 1 | 1 | 370 | 381 |  |  | PEAKS DB |
| total 8 peptides |  |  |  |  |  |  |  |  |  |  |  |  |  |  |  |  |  |  |  |
| --- | --- | --- | --- | --- | --- | --- | --- | --- | --- | --- | --- | --- | --- | --- | --- | --- | --- | --- | --- |

A0A2D0TC04.1

back to list

  

| Protein Coverage
| Supporting Peptides
|

Protein Coverage:

Supporting Peptides:

| Peptide | Uniq | -10lgP | Mass | Length | ppm | m/z | z | RT | Fraction | Scan | Source File | Area BuCaMH04\_B15 | #Feature | #Feature BuCaMH04\_B15 | Start | End | PTM | AScore | Found By |
| --- | --- | --- | --- | --- | --- | --- | --- | --- | --- | --- | --- | --- | --- | --- | --- | --- | --- | --- | --- |
| R.DVELLTGLDFYSALKQPLSETLR.L | Y | 106.53 | 2607.3794 | 23 | 1.0 | 870.1346 | 3 | 55.21 | 1 | 40430 | BuCaMH04.raw | 1.5639E6 | 1 | 1 | 795 | 817 |  |  | PEAKS DB |
| K.TFLPIFINSVN | Y | 83.65 | 1263.6863 | 11 | 0.8 | 632.8509 | 2 | 57.34 | 1 | 42335 | BuCaMH04.raw | 1.9991E6 | 1 | 1 | 820 | 830 |  |  | PEAKS DB |
| R.VRDVELLTGLDFYSALK.Q | Y | 76.29 | 1938.0461 | 17 | 0.8 | 647.0232 | 3 | 42.84 | 1 | 29290 | BuCaMH04.raw | 3.868E6 | 1 | 1 | 793 | 809 |  |  | PEAKS DB |
| R.VRDVELLTGLDFYSALKQPLSETLR.L | Y | 76.17 | 2862.5491 | 25 | 0.6 | 716.6450 | 4 | 45.23 | 1 | 31750 | BuCaMH04.raw | 2.8862E6 | 1 | 1 | 793 | 817 |  |  | PEAKS DB |
| K.SMEAIFLAHGPGFK.E | Y | 63.13 | 1503.7544 | 14 | 0.1 | 502.2588 | 3 | 27.04 | 1 | 15068 | BuCaMH04.raw | 1.2312E6 | 2 | 2 | 447 | 460 |  |  | PEAKS DB |
| R.NAAWWGGQPIWHTASYQGLK.A | Y | 57.32 | 2270.1021 | 20 | 1.0 | 757.7087 | 3 | 34.06 | 1 | 21492 | BuCaMH04.raw | 2.1579E6 | 1 | 1 | 203 | 222 |  |  | PEAKS DB |
| R.LWNYFHSTLLPK.Y | Y | 51.18 | 1517.8031 | 12 | 0.6 | 506.9420 | 3 | 29.95 | 1 | 17747 | BuCaMH04.raw | 1.9945E6 | 1 | 1 | 677 | 688 |  |  | PEAKS DB |
| K.DFYTFDSEAIVK.K | Y | 50.66 | 1433.6714 | 12 | 1.3 | 717.8439 | 2 | 35.49 | 1 | 22924 | BuCaMH04.raw | 5.6133E6 | 1 | 1 | 370 | 381 |  |  | PEAKS DB |
| total 8 peptides |  |  |  |  |  |  |  |  |  |  |  |  |  |  |  |  |  |  |  |
| --- | --- | --- | --- | --- | --- | --- | --- | --- | --- | --- | --- | --- | --- | --- | --- | --- | --- | --- | --- |

JAA74761.1

back to list

  

| Protein Coverage
| Supporting Peptides
|

Protein Coverage:

Supporting Peptides:

| Peptide | Uniq | -10lgP | Mass | Length | ppm | m/z | z | RT | Fraction | Scan | Source File | Area BuCaMH04\_B15 | #Feature | #Feature BuCaMH04\_B15 | Start | End | PTM | AScore | Found By |
| --- | --- | --- | --- | --- | --- | --- | --- | --- | --- | --- | --- | --- | --- | --- | --- | --- | --- | --- | --- |
| K.TSADIVINDLSLIHQLPK.E | N | 98.11 | 1976.0942 | 18 | 0.9 | 659.7059 | 3 | 39.96 | 1 | 27043 | BuCaMH04.raw | 9.5523E7 | 2 | 2 | 383 | 400 |  |  | PEAKS DB |
| Q.EADYEEFLEIAR.N | N | 92.74 | 1483.6830 | 12 | 1.0 | 742.8495 | 2 | 40.39 | 1 | 27344 | BuCaMH04.raw | 8.6584E7 | 1 | 1 | 8 | 19 |  |  | PEAKS DB |
| R.IYFEPPLPPK.K | N | 84.04 | 1199.6589 | 10 | 0.3 | 600.8369 | 2 | 25.08 | 1 | 14052 | BuCaMH04.raw | 8.9718E7 | 2 | 2 | 297 | 306 |  |  | PEAKS DB |
| E.ADYEEFLEIAR.N | N | 74.83 | 1354.6404 | 11 | 0.3 | 678.3276 | 2 | 38.08 | 1 | 25194 | BuCaMH04.raw | 3.5897E7 | 1 | 1 | 9 | 19 |  |  | PEAKS DB |
| A.DIVINDLSLIHQLPK.E | N | 69.37 | 1716.9774 | 15 | 0.4 | 859.4963 | 2 | 37.46 | 1 | 24584 | BuCaMH04.raw | 4.3779E7 | 2 | 2 | 386 | 400 |  |  | PEAKS DB |
| A.DYEEFLEIAR.N | N | 65.68 | 1283.6033 | 10 | 0.6 | 642.8093 | 2 | 40.14 | 1 | 26895 | BuCaMH04.raw | 2.3334E6 | 1 | 1 | 10 | 19 |  |  | PEAKS DB |
| D.YEEFLEIAR.N | N | 59.77 | 1168.5764 | 9 | 0.4 | 585.2957 | 2 | 26.94 | 1 | 14950 | BuCaMH04.raw | 2.8615E6 | 1 | 1 | 11 | 19 |  |  | PEAKS DB |
| K.RFDEIVGGFDQLPISMYQAIAKMVHLNAQ.V | Y | 55.01 | 3290.6580 | 29 | -5.5 | 823.6672 | 4 | 61.37 | 1 | 45564 | BuCaMH04.raw | 5.8499E6 | 1 | 1 | 227 | 255 |  |  | PEAKS DB |
| K.RFDEIVGGFDQLPIS.M | N | 52.08 | 1691.8518 | 15 | 0.2 | 846.9333 | 2 | 43.43 | 1 | 29880 | BuCaMH04.raw | 8.7751E5 | 1 | 1 | 227 | 241 |  |  | PEAKS DB |
| total 9 peptides |  |  |  |  |  |  |  |  |  |  |  |  |  |  |  |  |  |  |  |
| --- | --- | --- | --- | --- | --- | --- | --- | --- | --- | --- | --- | --- | --- | --- | --- | --- | --- | --- | --- |

Q8QFW3.1

back to list

  

| Protein Coverage
| Supporting Peptides
|

Protein Coverage:

Supporting Peptides:

| Peptide | Uniq | -10lgP | Mass | Length | ppm | m/z | z | RT | Fraction | Scan | Source File | Area BuCaMH04\_B15 | #Feature | #Feature BuCaMH04\_B15 | Start | End | PTM | AScore | Found By |
| --- | --- | --- | --- | --- | --- | --- | --- | --- | --- | --- | --- | --- | --- | --- | --- | --- | --- | --- | --- |
| K.TWGHYADYGC(+57.02)YC(+57.02)GAGGSGTPVDALDR.C | Y | 106.20 | 2805.1548 | 26 | 0.6 | 936.0594 | 3 | 24.57 | 1 | 12915 | BuCaMH04.raw | 1.7123E9 | 2 | 2 | 45 | 70 | Carbamidomethylation | C10:Carbamidomethylation:1000.00;C12:Carbamidomethylation:1000.00 | PEAKS DB |
| R.TAALC(+57.02)FGDSEYIGAHK.N | Y | 97.90 | 1738.7985 | 16 | -0.3 | 580.6066 | 3 | 19.02 | 1 | 8048 | BuCaMH04.raw | 2.3488E8 | 3 | 3 | 123 | 138 | Carbamidomethylation | C5:Carbamidomethylation:1000.00 | PEAKS DB |
| H.YADYGC(+57.02)YC(+57.02)GAGGSGTPVDALDR.C | Y | 89.81 | 2323.9473 | 22 | 1.1 | 1162.9822 | 2 | 23.57 | 1 | 11836 | BuCaMH04.raw | 5.0818E7 | 1 | 1 | 49 | 70 | Carbamidomethylation | C6:Carbamidomethylation:1000.00;C8:Carbamidomethylation:1000.00 | PEAKS DB |
| A.DYGC(+57.02)YC(+57.02)GAGGSGTPVDALDR.C | Y | 70.43 | 2089.8469 | 20 | -0.1 | 1045.9307 | 2 | 22.99 | 1 | 11380 | BuCaMH04.raw | 3.808E7 | 1 | 1 | 51 | 70 | Carbamidomethylation | C4:Carbamidomethylation:1000.00;C6:Carbamidomethylation:1000.00 | PEAKS DB |
| L.NLINFMEMIR.Y | N | 64.70 | 1279.6417 | 10 | -0.1 | 640.8281 | 2 | 50.79 | 1 | 36537 | BuCaMH04.raw | 7.4404E7 | 1 | 1 | 28 | 37 |  |  | PEAKS DB |
| A.LC(+57.02)FGDSEYIGAHK.N | Y | 58.84 | 1495.6765 | 13 | 0.5 | 499.5664 | 3 | 15.39 | 1 | 4991 | BuCaMH04.raw | 4.3168E6 | 1 | 1 | 126 | 138 | Carbamidomethylation | C2:Carbamidomethylation:1000.00 | PEAKS DB |
| total 6 peptides |  |  |  |  |  |  |  |  |  |  |  |  |  |  |  |  |  |  |  |
| --- | --- | --- | --- | --- | --- | --- | --- | --- | --- | --- | --- | --- | --- | --- | --- | --- | --- | --- | --- |

AAL87004.1

back to list

  

| Protein Coverage
| Supporting Peptides
|

Protein Coverage:

Supporting Peptides:

| Peptide | Uniq | -10lgP | Mass | Length | ppm | m/z | z | RT | Fraction | Scan | Source File | Area BuCaMH04\_B15 | #Feature | #Feature BuCaMH04\_B15 | Start | End | PTM | AScore | Found By |
| --- | --- | --- | --- | --- | --- | --- | --- | --- | --- | --- | --- | --- | --- | --- | --- | --- | --- | --- | --- |
| K.TWGHYADYGC(+57.02)YC(+57.02)GAGGSGTPVDALDR.C | Y | 106.20 | 2805.1548 | 26 | 0.6 | 936.0594 | 3 | 24.57 | 1 | 12915 | BuCaMH04.raw | 1.7123E9 | 2 | 2 | 45 | 70 | Carbamidomethylation | C10:Carbamidomethylation:1000.00;C12:Carbamidomethylation:1000.00 | PEAKS DB |
| R.TAALC(+57.02)FGDSEYIGAHK.N | Y | 97.90 | 1738.7985 | 16 | -0.3 | 580.6066 | 3 | 19.02 | 1 | 8048 | BuCaMH04.raw | 2.3488E8 | 3 | 3 | 123 | 138 | Carbamidomethylation | C5:Carbamidomethylation:1000.00 | PEAKS DB |
| H.YADYGC(+57.02)YC(+57.02)GAGGSGTPVDALDR.C | Y | 89.81 | 2323.9473 | 22 | 1.1 | 1162.9822 | 2 | 23.57 | 1 | 11836 | BuCaMH04.raw | 5.0818E7 | 1 | 1 | 49 | 70 | Carbamidomethylation | C6:Carbamidomethylation:1000.00;C8:Carbamidomethylation:1000.00 | PEAKS DB |
| A.DYGC(+57.02)YC(+57.02)GAGGSGTPVDALDR.C | Y | 70.43 | 2089.8469 | 20 | -0.1 | 1045.9307 | 2 | 22.99 | 1 | 11380 | BuCaMH04.raw | 3.808E7 | 1 | 1 | 51 | 70 | Carbamidomethylation | C4:Carbamidomethylation:1000.00;C6:Carbamidomethylation:1000.00 | PEAKS DB |
| L.NLINFMEMIR.Y | N | 64.70 | 1279.6417 | 10 | -0.1 | 640.8281 | 2 | 50.79 | 1 | 36537 | BuCaMH04.raw | 7.4404E7 | 1 | 1 | 28 | 37 |  |  | PEAKS DB |
| A.LC(+57.02)FGDSEYIGAHK.N | Y | 58.84 | 1495.6765 | 13 | 0.5 | 499.5664 | 3 | 15.39 | 1 | 4991 | BuCaMH04.raw | 4.3168E6 | 1 | 1 | 126 | 138 | Carbamidomethylation | C2:Carbamidomethylation:1000.00 | PEAKS DB |
| total 6 peptides |  |  |  |  |  |  |  |  |  |  |  |  |  |  |  |  |  |  |  |
| --- | --- | --- | --- | --- | --- | --- | --- | --- | --- | --- | --- | --- | --- | --- | --- | --- | --- | --- | --- |

B6EWW8.1

back to list

  

| Protein Coverage
| Supporting Peptides
|

Protein Coverage:

Supporting Peptides:

| Peptide | Uniq | -10lgP | Mass | Length | ppm | m/z | z | RT | Fraction | Scan | Source File | Area BuCaMH04\_B15 | #Feature | #Feature BuCaMH04\_B15 | Start | End | PTM | AScore | Found By |
| --- | --- | --- | --- | --- | --- | --- | --- | --- | --- | --- | --- | --- | --- | --- | --- | --- | --- | --- | --- |
| K.YLGYLNVIFDDK.G | N | 102.03 | 1458.7394 | 12 | 0.6 | 730.3774 | 2 | 47.23 | 1 | 33232 | BuCaMH04.raw | 1.4989E7 | 1 | 1 | 300 | 311 |  |  | PEAKS DB |
| R.VVSLNVLC(+57.02)TEC(+57.02)R.V | N | 85.35 | 1448.7115 | 12 | 0.3 | 725.3633 | 2 | 23.30 | 1 | 11847 | BuCaMH04.raw | 3.0811E8 | 1 | 1 | 484 | 495 | Carbamidomethylation | C8:Carbamidomethylation:1000.00;C11:Carbamidomethylation:1000.00 | PEAKS DB |
| A.VLPFGGTFDLLQIK.G | N | 75.40 | 1546.8759 | 14 | 0.8 | 774.4458 | 2 | 52.72 | 1 | 38151 | BuCaMH04.raw | 4.902E6 | 1 | 1 | 429 | 442 |  |  | PEAKS DB |
| K.ETPVLSNPGPYLEFRDEVEELQK.H | N | 72.40 | 2688.3281 | 23 | 0.9 | 897.1174 | 3 | 41.06 | 1 | 27972 | BuCaMH04.raw | 3.1625E7 | 1 | 1 | 194 | 216 |  |  | PEAKS DB |
| Y.LGYLNVIFDDK.G | N | 69.50 | 1295.6761 | 11 | 0.8 | 648.8459 | 2 | 41.12 | 1 | 27795 | BuCaMH04.raw | 7.8437E5 | 1 | 1 | 301 | 311 |  |  | PEAKS DB |
| K.ETPVLSNPGPYLEFR.D | N | 67.68 | 1717.8674 | 15 | 1.3 | 859.9421 | 2 | 33.85 | 1 | 21396 | BuCaMH04.raw | 6.7956E6 | 1 | 1 | 194 | 208 |  |  | PEAKS DB |
| V.LPFGGTFDLLQIK.G | N | 65.66 | 1447.8075 | 13 | 0.5 | 724.9114 | 2 | 49.97 | 1 | 35712 | BuCaMH04.raw | 4.3937E5 | 1 | 1 | 430 | 442 |  |  | PEAKS DB |
| R.FHEC(+57.02)NLGNLIC(+57.02)DAVIYNNVR.H | Y | 63.93 | 2420.1365 | 20 | -0.3 | 1211.0752 | 2 | 41.26 | 1 | 27931 | BuCaMH04.raw | 4.1447E5 | 1 | 1 | 369 | 388 | Carbamidomethylation | C4:Carbamidomethylation:1000.00;C11:Carbamidomethylation:1000.00 | PEAKS DB |
| R.QVPVVQAYAFGK.Y | N | 54.70 | 1305.7081 | 12 | -0.4 | 653.8611 | 2 | 22.79 | 1 | 11255 | BuCaMH04.raw | 5.2789E6 | 1 | 1 | 288 | 299 |  |  | PEAKS DB |
| total 9 peptides |  |  |  |  |  |  |  |  |  |  |  |  |  |  |  |  |  |  |  |
| --- | --- | --- | --- | --- | --- | --- | --- | --- | --- | --- | --- | --- | --- | --- | --- | --- | --- | --- | --- |

BAG82602.1

back to list

  

| Protein Coverage
| Supporting Peptides
|

Protein Coverage:

Supporting Peptides:

| Peptide | Uniq | -10lgP | Mass | Length | ppm | m/z | z | RT | Fraction | Scan | Source File | Area BuCaMH04\_B15 | #Feature | #Feature BuCaMH04\_B15 | Start | End | PTM | AScore | Found By |
| --- | --- | --- | --- | --- | --- | --- | --- | --- | --- | --- | --- | --- | --- | --- | --- | --- | --- | --- | --- |
| K.YLGYLNVIFDDK.G | N | 102.03 | 1458.7394 | 12 | 0.6 | 730.3774 | 2 | 47.23 | 1 | 33232 | BuCaMH04.raw | 1.4989E7 | 1 | 1 | 300 | 311 |  |  | PEAKS DB |
| R.VVSLNVLC(+57.02)TEC(+57.02)R.V | N | 85.35 | 1448.7115 | 12 | 0.3 | 725.3633 | 2 | 23.30 | 1 | 11847 | BuCaMH04.raw | 3.0811E8 | 1 | 1 | 484 | 495 | Carbamidomethylation | C8:Carbamidomethylation:1000.00;C11:Carbamidomethylation:1000.00 | PEAKS DB |
| A.VLPFGGTFDLLQIK.G | N | 75.40 | 1546.8759 | 14 | 0.8 | 774.4458 | 2 | 52.72 | 1 | 38151 | BuCaMH04.raw | 4.902E6 | 1 | 1 | 429 | 442 |  |  | PEAKS DB |
| K.ETPVLSNPGPYLEFRDEVEELQK.H | N | 72.40 | 2688.3281 | 23 | 0.9 | 897.1174 | 3 | 41.06 | 1 | 27972 | BuCaMH04.raw | 3.1625E7 | 1 | 1 | 194 | 216 |  |  | PEAKS DB |
| Y.LGYLNVIFDDK.G | N | 69.50 | 1295.6761 | 11 | 0.8 | 648.8459 | 2 | 41.12 | 1 | 27795 | BuCaMH04.raw | 7.8437E5 | 1 | 1 | 301 | 311 |  |  | PEAKS DB |
| K.ETPVLSNPGPYLEFR.D | N | 67.68 | 1717.8674 | 15 | 1.3 | 859.9421 | 2 | 33.85 | 1 | 21396 | BuCaMH04.raw | 6.7956E6 | 1 | 1 | 194 | 208 |  |  | PEAKS DB |
| V.LPFGGTFDLLQIK.G | N | 65.66 | 1447.8075 | 13 | 0.5 | 724.9114 | 2 | 49.97 | 1 | 35712 | BuCaMH04.raw | 4.3937E5 | 1 | 1 | 430 | 442 |  |  | PEAKS DB |
| R.FHEC(+57.02)NLGNLIC(+57.02)DAVIYNNVR.H | Y | 63.93 | 2420.1365 | 20 | -0.3 | 1211.0752 | 2 | 41.26 | 1 | 27931 | BuCaMH04.raw | 4.1447E5 | 1 | 1 | 369 | 388 | Carbamidomethylation | C4:Carbamidomethylation:1000.00;C11:Carbamidomethylation:1000.00 | PEAKS DB |
| R.QVPVVQAYAFGK.Y | N | 54.70 | 1305.7081 | 12 | -0.4 | 653.8611 | 2 | 22.79 | 1 | 11255 | BuCaMH04.raw | 5.2789E6 | 1 | 1 | 288 | 299 |  |  | PEAKS DB |
| total 9 peptides |  |  |  |  |  |  |  |  |  |  |  |  |  |  |  |  |  |  |  |
| --- | --- | --- | --- | --- | --- | --- | --- | --- | --- | --- | --- | --- | --- | --- | --- | --- | --- | --- | --- |

XP\_032090372.1

back to list

  

| Protein Coverage
| Supporting Peptides
|

Protein Coverage:

Supporting Peptides:

| Peptide | Uniq | -10lgP | Mass | Length | ppm | m/z | z | RT | Fraction | Scan | Source File | Area BuCaMH04\_B15 | #Feature | #Feature BuCaMH04\_B15 | Start | End | PTM | AScore | Found By |
| --- | --- | --- | --- | --- | --- | --- | --- | --- | --- | --- | --- | --- | --- | --- | --- | --- | --- | --- | --- |
| K.DEGSYFLIYGLPGFSK.D | N | 103.69 | 1791.8718 | 16 | 0.7 | 896.9438 | 2 | 56.99 | 1 | 42168 | BuCaMH04.raw | 1.8962E8 | 3 | 3 | 355 | 370 |  |  | PEAKS DB |
| A.FLGIPFAEPPVGR.M | N | 70.62 | 1398.7659 | 13 | 0.9 | 700.3909 | 2 | 42.12 | 1 | 28605 | BuCaMH04.raw | 1.1387E8 | 1 | 1 | 59 | 71 |  |  | PEAKS DB |
| I.C(+57.02)AFWNHFLPK.L | N | 67.68 | 1318.6281 | 10 | 0.2 | 440.5500 | 3 | 27.24 | 1 | 15136 | BuCaMH04.raw | 4.6147E6 | 1 | 1 | 550 | 559 | Carbamidomethylation | C1:Carbamidomethylation:1000.00 | PEAKS DB |
| F.LGIPFAEPPVGR.M | N | 62.25 | 1251.6975 | 12 | 0.5 | 626.8563 | 2 | 29.35 | 1 | 17318 | BuCaMH04.raw | 6.6683E7 | 1 | 1 | 60 | 71 |  |  | PEAKS DB |
| R.AQIC(+57.02)AFWNHFLPK.L | N | 60.48 | 1630.8079 | 13 | 1.0 | 544.6104 | 3 | 36.94 | 1 | 23938 | BuCaMH04.raw | 1.8602E6 | 1 | 1 | 547 | 559 | Carbamidomethylation | C4:Carbamidomethylation:1000.00 | PEAKS DB |
| S.AFLGIPFAEPPVGR.M | N | 58.55 | 1469.8030 | 14 | 0.6 | 735.9092 | 2 | 43.95 | 1 | 30470 | BuCaMH04.raw | 5.0781E6 | 1 | 1 | 58 | 71 |  |  | PEAKS DB |
| L.IYGLPGFSK.D | N | 51.29 | 980.5331 | 9 | 0.8 | 491.2742 | 2 | 27.30 | 1 | 15385 | BuCaMH04.raw | 4.7137E6 | 1 | 1 | 362 | 370 |  |  | PEAKS DB |
| total 7 peptides |  |  |  |  |  |  |  |  |  |  |  |  |  |  |  |  |  |  |  |
| --- | --- | --- | --- | --- | --- | --- | --- | --- | --- | --- | --- | --- | --- | --- | --- | --- | --- | --- | --- |

XP\_032090371.1

back to list

  

| Protein Coverage
| Supporting Peptides
|

Protein Coverage:

Supporting Peptides:

| Peptide | Uniq | -10lgP | Mass | Length | ppm | m/z | z | RT | Fraction | Scan | Source File | Area BuCaMH04\_B15 | #Feature | #Feature BuCaMH04\_B15 | Start | End | PTM | AScore | Found By |
| --- | --- | --- | --- | --- | --- | --- | --- | --- | --- | --- | --- | --- | --- | --- | --- | --- | --- | --- | --- |
| K.DEGSYFLIYGLPGFSK.D | N | 103.69 | 1791.8718 | 16 | 0.7 | 896.9438 | 2 | 56.99 | 1 | 42168 | BuCaMH04.raw | 1.8962E8 | 3 | 3 | 355 | 370 |  |  | PEAKS DB |
| A.FLGIPFAEPPVGR.M | N | 70.62 | 1398.7659 | 13 | 0.9 | 700.3909 | 2 | 42.12 | 1 | 28605 | BuCaMH04.raw | 1.1387E8 | 1 | 1 | 59 | 71 |  |  | PEAKS DB |
| I.C(+57.02)AFWNHFLPK.L | N | 67.68 | 1318.6281 | 10 | 0.2 | 440.5500 | 3 | 27.24 | 1 | 15136 | BuCaMH04.raw | 4.6147E6 | 1 | 1 | 550 | 559 | Carbamidomethylation | C1:Carbamidomethylation:1000.00 | PEAKS DB |
| F.LGIPFAEPPVGR.M | N | 62.25 | 1251.6975 | 12 | 0.5 | 626.8563 | 2 | 29.35 | 1 | 17318 | BuCaMH04.raw | 6.6683E7 | 1 | 1 | 60 | 71 |  |  | PEAKS DB |
| R.AQIC(+57.02)AFWNHFLPK.L | N | 60.48 | 1630.8079 | 13 | 1.0 | 544.6104 | 3 | 36.94 | 1 | 23938 | BuCaMH04.raw | 1.8602E6 | 1 | 1 | 547 | 559 | Carbamidomethylation | C4:Carbamidomethylation:1000.00 | PEAKS DB |
| S.AFLGIPFAEPPVGR.M | N | 58.55 | 1469.8030 | 14 | 0.6 | 735.9092 | 2 | 43.95 | 1 | 30470 | BuCaMH04.raw | 5.0781E6 | 1 | 1 | 58 | 71 |  |  | PEAKS DB |
| L.IYGLPGFSK.D | N | 51.29 | 980.5331 | 9 | 0.8 | 491.2742 | 2 | 27.30 | 1 | 15385 | BuCaMH04.raw | 4.7137E6 | 1 | 1 | 362 | 370 |  |  | PEAKS DB |
| total 7 peptides |  |  |  |  |  |  |  |  |  |  |  |  |  |  |  |  |  |  |  |
| --- | --- | --- | --- | --- | --- | --- | --- | --- | --- | --- | --- | --- | --- | --- | --- | --- | --- | --- | --- |

XP\_013930899.1

back to list

  

| Protein Coverage
| Supporting Peptides
|

Protein Coverage:

Supporting Peptides:

| Peptide | Uniq | -10lgP | Mass | Length | ppm | m/z | z | RT | Fraction | Scan | Source File | Area BuCaMH04\_B15 | #Feature | #Feature BuCaMH04\_B15 | Start | End | PTM | AScore | Found By |
| --- | --- | --- | --- | --- | --- | --- | --- | --- | --- | --- | --- | --- | --- | --- | --- | --- | --- | --- | --- |
| K.DEGSYFLIYGLPGFSK.D | N | 103.69 | 1791.8718 | 16 | 0.7 | 896.9438 | 2 | 56.99 | 1 | 42168 | BuCaMH04.raw | 1.8962E8 | 3 | 3 | 355 | 370 |  |  | PEAKS DB |
| A.FLGIPFAEPPVGR.M | N | 70.62 | 1398.7659 | 13 | 0.9 | 700.3909 | 2 | 42.12 | 1 | 28605 | BuCaMH04.raw | 1.1387E8 | 1 | 1 | 59 | 71 |  |  | PEAKS DB |
| I.C(+57.02)AFWNHFLPK.L | N | 67.68 | 1318.6281 | 10 | 0.2 | 440.5500 | 3 | 27.24 | 1 | 15136 | BuCaMH04.raw | 4.6147E6 | 1 | 1 | 550 | 559 | Carbamidomethylation | C1:Carbamidomethylation:1000.00 | PEAKS DB |
| F.LGIPFAEPPVGR.M | N | 62.25 | 1251.6975 | 12 | 0.5 | 626.8563 | 2 | 29.35 | 1 | 17318 | BuCaMH04.raw | 6.6683E7 | 1 | 1 | 60 | 71 |  |  | PEAKS DB |
| R.AQIC(+57.02)AFWNHFLPK.L | N | 60.48 | 1630.8079 | 13 | 1.0 | 544.6104 | 3 | 36.94 | 1 | 23938 | BuCaMH04.raw | 1.8602E6 | 1 | 1 | 547 | 559 | Carbamidomethylation | C4:Carbamidomethylation:1000.00 | PEAKS DB |
| S.AFLGIPFAEPPVGR.M | N | 58.55 | 1469.8030 | 14 | 0.6 | 735.9092 | 2 | 43.95 | 1 | 30470 | BuCaMH04.raw | 5.0781E6 | 1 | 1 | 58 | 71 |  |  | PEAKS DB |
| L.IYGLPGFSK.D | N | 51.29 | 980.5331 | 9 | 0.8 | 491.2742 | 2 | 27.30 | 1 | 15385 | BuCaMH04.raw | 4.7137E6 | 1 | 1 | 362 | 370 |  |  | PEAKS DB |
| total 7 peptides |  |  |  |  |  |  |  |  |  |  |  |  |  |  |  |  |  |  |  |
| --- | --- | --- | --- | --- | --- | --- | --- | --- | --- | --- | --- | --- | --- | --- | --- | --- | --- | --- | --- |

LAA54621.1

back to list

  

| Protein Coverage
| Supporting Peptides
|

Protein Coverage:

Supporting Peptides:

| Peptide | Uniq | -10lgP | Mass | Length | ppm | m/z | z | RT | Fraction | Scan | Source File | Area BuCaMH04\_B15 | #Feature | #Feature BuCaMH04\_B15 | Start | End | PTM | AScore | Found By |
| --- | --- | --- | --- | --- | --- | --- | --- | --- | --- | --- | --- | --- | --- | --- | --- | --- | --- | --- | --- |
| K.HVVVVGAGMAGLSAAYVLAGAGHK.V | N | 106.63 | 2234.1992 | 24 | 0.7 | 745.7409 | 3 | 30.19 | 1 | 18119 | BuCaMH04.raw | 2.655E7 | 1 | 1 | 54 | 77 |  |  | PEAKS DB |
| R.EADYEEFLEIAR.N | N | 92.74 | 1483.6830 | 12 | 1.0 | 742.8495 | 2 | 40.39 | 1 | 27344 | BuCaMH04.raw | 8.6584E7 | 1 | 1 | 32 | 43 |  |  | PEAKS DB |
| E.ADYEEFLEIAR.N | N | 74.83 | 1354.6404 | 11 | 0.3 | 678.3276 | 2 | 38.08 | 1 | 25194 | BuCaMH04.raw | 3.5897E7 | 1 | 1 | 33 | 43 |  |  | PEAKS DB |
| S.GDIVINDLSLIHQLPK.E | Y | 69.57 | 1773.9988 | 16 | 0.9 | 888.0074 | 2 | 40.14 | 1 | 27045 | BuCaMH04.raw | 5.0012E6 | 1 | 1 | 409 | 424 |  |  | PEAKS DB |
| G.DIVINDLSLIHQLPK.E | N | 69.37 | 1716.9774 | 15 | 0.4 | 859.4963 | 2 | 37.46 | 1 | 24584 | BuCaMH04.raw | 4.3779E7 | 2 | 2 | 410 | 424 |  |  | PEAKS DB |
| A.DYEEFLEIAR.N | N | 65.68 | 1283.6033 | 10 | 0.6 | 642.8093 | 2 | 40.14 | 1 | 26895 | BuCaMH04.raw | 2.3334E6 | 1 | 1 | 34 | 43 |  |  | PEAKS DB |
| D.YEEFLEIAR.N | N | 59.77 | 1168.5764 | 9 | 0.4 | 585.2957 | 2 | 26.94 | 1 | 14950 | BuCaMH04.raw | 2.8615E6 | 1 | 1 | 35 | 43 |  |  | PEAKS DB |
| total 7 peptides |  |  |  |  |  |  |  |  |  |  |  |  |  |  |  |  |  |  |  |
| --- | --- | --- | --- | --- | --- | --- | --- | --- | --- | --- | --- | --- | --- | --- | --- | --- | --- | --- | --- |

Q9PVK7.1

back to list

  

| Protein Coverage
| Supporting Peptides
|

Protein Coverage:

Supporting Peptides:

| Peptide | Uniq | -10lgP | Mass | Length | ppm | m/z | z | RT | Fraction | Scan | Source File | Area BuCaMH04\_B15 | #Feature | #Feature BuCaMH04\_B15 | Start | End | PTM | AScore | Found By |
| --- | --- | --- | --- | --- | --- | --- | --- | --- | --- | --- | --- | --- | --- | --- | --- | --- | --- | --- | --- |
| K.DDC(+57.02)DLPELC(+57.02)TGQSAEC(+57.02)PTDVFQR.N | Y | 107.66 | 2712.1101 | 23 | 0.0 | 1357.0624 | 2 | 34.56 | 1 | 21920 | BuCaMH04.raw | 5.0363E7 | 2 | 2 | 459 | 481 | Carbamidomethylation | C3:Carbamidomethylation:1000.00;C9:Carbamidomethylation:1000.00;C16:Carbamidomethylation:1000.00 | PEAKS DB |
| A.LIGLEIWSNINEINVQSDVK.A | Y | 80.37 | 2283.2109 | 20 | 0.4 | 1142.6132 | 2 | 51.67 | 1 | 37383 | BuCaMH04.raw | 6.1952E6 | 1 | 1 | 240 | 259 |  |  | PEAKS DB |
| K.ATLDLFGEWR.E | N | 72.05 | 1206.6033 | 10 | 0.3 | 604.3091 | 2 | 42.94 | 1 | 29582 | BuCaMH04.raw | 1.2834E7 | 1 | 1 | 260 | 269 |  |  | PEAKS DB |
| L.IGLEIWSNINEINVQSDVK.A | Y | 68.26 | 2170.1270 | 19 | 0.3 | 1086.0710 | 2 | 44.24 | 1 | 30615 | BuCaMH04.raw | 2.0722E6 | 1 | 1 | 241 | 259 |  |  | PEAKS DB |
| A.TLDLFGEWR.E | N | 65.70 | 1135.5662 | 9 | 0.5 | 568.7906 | 2 | 42.31 | 1 | 29257 | BuCaMH04.raw | 4.2198E8 | 1 | 1 | 261 | 269 |  |  | PEAKS DB |
| T.LDLFGEWR.E | N | 57.32 | 1034.5186 | 8 | 0.6 | 518.2668 | 2 | 41.26 | 1 | 27863 | BuCaMH04.raw | 4.6562E7 | 1 | 1 | 262 | 269 |  |  | PEAKS DB |
| K.C(+57.02)PIMTNQC(+57.02)IALR.G | N | 52.31 | 1475.7047 | 12 | -0.2 | 738.8595 | 2 | 38.34 | 1 | 25350 | BuCaMH04.raw | 4.1161E5 | 1 | 1 | 497 | 508 | Carbamidomethylation | C1:Carbamidomethylation:1000.00;C8:Carbamidomethylation:1000.00 | PEAKS DB |
| total 7 peptides |  |  |  |  |  |  |  |  |  |  |  |  |  |  |  |  |  |  |  |
| --- | --- | --- | --- | --- | --- | --- | --- | --- | --- | --- | --- | --- | --- | --- | --- | --- | --- | --- | --- |

AAF00693.1

back to list

  

| Protein Coverage
| Supporting Peptides
|

Protein Coverage:

Supporting Peptides:

| Peptide | Uniq | -10lgP | Mass | Length | ppm | m/z | z | RT | Fraction | Scan | Source File | Area BuCaMH04\_B15 | #Feature | #Feature BuCaMH04\_B15 | Start | End | PTM | AScore | Found By |
| --- | --- | --- | --- | --- | --- | --- | --- | --- | --- | --- | --- | --- | --- | --- | --- | --- | --- | --- | --- |
| K.DDC(+57.02)DLPELC(+57.02)TGQSAEC(+57.02)PTDVFQR.N | Y | 107.66 | 2712.1101 | 23 | 0.0 | 1357.0624 | 2 | 34.56 | 1 | 21920 | BuCaMH04.raw | 5.0363E7 | 2 | 2 | 459 | 481 | Carbamidomethylation | C3:Carbamidomethylation:1000.00;C9:Carbamidomethylation:1000.00;C16:Carbamidomethylation:1000.00 | PEAKS DB |
| A.LIGLEIWSNINEINVQSDVK.A | Y | 80.37 | 2283.2109 | 20 | 0.4 | 1142.6132 | 2 | 51.67 | 1 | 37383 | BuCaMH04.raw | 6.1952E6 | 1 | 1 | 240 | 259 |  |  | PEAKS DB |
| K.ATLDLFGEWR.E | N | 72.05 | 1206.6033 | 10 | 0.3 | 604.3091 | 2 | 42.94 | 1 | 29582 | BuCaMH04.raw | 1.2834E7 | 1 | 1 | 260 | 269 |  |  | PEAKS DB |
| L.IGLEIWSNINEINVQSDVK.A | Y | 68.26 | 2170.1270 | 19 | 0.3 | 1086.0710 | 2 | 44.24 | 1 | 30615 | BuCaMH04.raw | 2.0722E6 | 1 | 1 | 241 | 259 |  |  | PEAKS DB |
| A.TLDLFGEWR.E | N | 65.70 | 1135.5662 | 9 | 0.5 | 568.7906 | 2 | 42.31 | 1 | 29257 | BuCaMH04.raw | 4.2198E8 | 1 | 1 | 261 | 269 |  |  | PEAKS DB |
| T.LDLFGEWR.E | N | 57.32 | 1034.5186 | 8 | 0.6 | 518.2668 | 2 | 41.26 | 1 | 27863 | BuCaMH04.raw | 4.6562E7 | 1 | 1 | 262 | 269 |  |  | PEAKS DB |
| K.C(+57.02)PIMTNQC(+57.02)IALR.G | N | 52.31 | 1475.7047 | 12 | -0.2 | 738.8595 | 2 | 38.34 | 1 | 25350 | BuCaMH04.raw | 4.1161E5 | 1 | 1 | 497 | 508 | Carbamidomethylation | C1:Carbamidomethylation:1000.00;C8:Carbamidomethylation:1000.00 | PEAKS DB |
| total 7 peptides |  |  |  |  |  |  |  |  |  |  |  |  |  |  |  |  |  |  |  |
| --- | --- | --- | --- | --- | --- | --- | --- | --- | --- | --- | --- | --- | --- | --- | --- | --- | --- | --- | --- |

ETE70041.1

back to list

  

| Protein Coverage
| Supporting Peptides
|

Protein Coverage:

Supporting Peptides:

| Peptide | Uniq | -10lgP | Mass | Length | ppm | m/z | z | RT | Fraction | Scan | Source File | Area BuCaMH04\_B15 | #Feature | #Feature BuCaMH04\_B15 | Start | End | PTM | AScore | Found By |
| --- | --- | --- | --- | --- | --- | --- | --- | --- | --- | --- | --- | --- | --- | --- | --- | --- | --- | --- | --- |
| K.DFVSVLVQHFAGR.L | Y | 87.29 | 1473.7728 | 13 | 0.7 | 737.8942 | 2 | 50.89 | 1 | 36389 | BuCaMH04.raw | 8.868E5 | 1 | 1 | 209 | 221 |  |  | PEAKS DB |
| K.TIVLQSEWEEALK.N | Y | 84.69 | 1544.8086 | 13 | 0.5 | 773.4120 | 2 | 35.62 | 1 | 23020 | BuCaMH04.raw | 7.9149E6 | 1 | 1 | 239 | 251 |  |  | PEAKS DB |
| R.EVALDMSQFENIAVR.R | Y | 81.07 | 1720.8453 | 15 | 0.6 | 861.4304 | 2 | 40.04 | 1 | 26830 | BuCaMH04.raw | 1.3658E6 | 1 | 1 | 78 | 92 |  |  | PEAKS DB |
| K.NRDEAVLWLWSR.H | Y | 69.89 | 1543.7896 | 12 | 0.6 | 515.6041 | 3 | 35.25 | 1 | 22700 | BuCaMH04.raw | 3.2958E6 | 1 | 1 | 343 | 354 |  |  | PEAKS DB |
| R.LSALKDFVSVLVQHFAGR.L | Y | 64.40 | 1986.1050 | 18 | 0.9 | 663.0428 | 3 | 53.15 | 1 | 38491 | BuCaMH04.raw | 8.2803E5 | 1 | 1 | 204 | 221 |  |  | PEAKS DB |
| R.SIPGLEDWR.S | Y | 62.84 | 1071.5349 | 9 | 0.3 | 536.7749 | 2 | 27.93 | 1 | 15918 | BuCaMH04.raw | 8.5997E6 | 1 | 1 | 124 | 132 |  |  | PEAKS DB |
| R.DEAVLWLWSR.H | Y | 52.61 | 1273.6455 | 10 | 0.1 | 637.8301 | 2 | 50.92 | 1 | 36486 | BuCaMH04.raw | 6.2011E5 | 1 | 1 | 345 | 354 |  |  | PEAKS DB |
| R.IEAAVFPNLEGER.L | Y | 52.04 | 1443.7357 | 13 | 2.7 | 722.8771 | 2 | 26.27 | 1 | 14338 | BuCaMH04.raw | 2.0422E6 | 1 | 1 | 191 | 203 |  |  | PEAKS DB |
| N.FLYNLDLWLRPK.T | Y | 50.59 | 1576.8766 | 12 | -0.1 | 526.6328 | 3 | 42.62 | 1 | 29200 | BuCaMH04.raw | 0 | 0 | 0 | 227 | 238 |  |  | PEAKS DB |
| total 9 peptides |  |  |  |  |  |  |  |  |  |  |  |  |  |  |  |  |  |  |  |
| --- | --- | --- | --- | --- | --- | --- | --- | --- | --- | --- | --- | --- | --- | --- | --- | --- | --- | --- | --- |

XP\_034291085.1

back to list

  

| Protein Coverage
| Supporting Peptides
|

Protein Coverage:

Supporting Peptides:

| Peptide | Uniq | -10lgP | Mass | Length | ppm | m/z | z | RT | Fraction | Scan | Source File | Area BuCaMH04\_B15 | #Feature | #Feature BuCaMH04\_B15 | Start | End | PTM | AScore | Found By |
| --- | --- | --- | --- | --- | --- | --- | --- | --- | --- | --- | --- | --- | --- | --- | --- | --- | --- | --- | --- |
| K.ALYSPNLFIC(+57.02)FC(+57.02)PPGFSGK.F | N | 103.15 | 2174.0330 | 19 | 0.0 | 1088.0238 | 2 | 52.04 | 1 | 37562 | BuCaMH04.raw | 4.0781E6 | 1 | 1 | 101 | 119 | Carbamidomethylation | C10:Carbamidomethylation:1000.00;C12:Carbamidomethylation:1000.00 | PEAKS DB |
| K.LIGIISWGVGC(+57.02)GK.K | Y | 81.06 | 1358.7380 | 13 | 0.7 | 680.3768 | 2 | 38.46 | 1 | 25335 | BuCaMH04.raw | 1.7572E7 | 1 | 1 | 522 | 534 | Carbamidomethylation | C11:Carbamidomethylation:1000.00 | PEAKS DB |
| S.YIESHPWQAGIFVK.N | N | 74.84 | 1673.8566 | 14 | 0.8 | 558.9599 | 3 | 23.41 | 1 | 11817 | BuCaMH04.raw | 2.6952E6 | 1 | 1 | 320 | 333 |  |  | PEAKS DB |
| L.IGIISWGVGC(+57.02)GK.K | Y | 74.02 | 1245.6539 | 12 | 0.2 | 623.8344 | 2 | 30.99 | 1 | 18855 | BuCaMH04.raw | 1.1095E7 | 1 | 1 | 523 | 534 | Carbamidomethylation | C10:Carbamidomethylation:1000.00 | PEAKS DB |
| K.SSVTEQIFQVER.Y | Y | 69.15 | 1421.7151 | 12 | 0.4 | 711.8651 | 2 | 23.78 | 1 | 12070 | BuCaMH04.raw | 6.1912E7 | 1 | 1 | 381 | 392 |  |  | PEAKS DB |
| K.GGAYSYIESHPWQAGIFVK.N | N | 52.03 | 2109.0320 | 19 | -0.2 | 704.0178 | 3 | 33.22 | 1 | 20705 | BuCaMH04.raw | 2.0132E7 | 1 | 1 | 315 | 333 |  |  | PEAKS DB |
| R.TLDNDIALLK.L | N | 51.46 | 1114.6234 | 10 | 0.1 | 558.3190 | 2 | 25.03 | 1 | 13396 | BuCaMH04.raw | 5.4174E7 | 1 | 1 | 403 | 412 |  |  | PEAKS DB |
| total 7 peptides |  |  |  |  |  |  |  |  |  |  |  |  |  |  |  |  |  |  |  |
| --- | --- | --- | --- | --- | --- | --- | --- | --- | --- | --- | --- | --- | --- | --- | --- | --- | --- | --- | --- |

XP\_034291087.1

back to list

  

| Protein Coverage
| Supporting Peptides
|

Protein Coverage:

Supporting Peptides:

| Peptide | Uniq | -10lgP | Mass | Length | ppm | m/z | z | RT | Fraction | Scan | Source File | Area BuCaMH04\_B15 | #Feature | #Feature BuCaMH04\_B15 | Start | End | PTM | AScore | Found By |
| --- | --- | --- | --- | --- | --- | --- | --- | --- | --- | --- | --- | --- | --- | --- | --- | --- | --- | --- | --- |
| K.ALYSPNLFIC(+57.02)FC(+57.02)PPGFSGK.F | N | 103.15 | 2174.0330 | 19 | 0.0 | 1088.0238 | 2 | 52.04 | 1 | 37562 | BuCaMH04.raw | 4.0781E6 | 1 | 1 | 101 | 119 | Carbamidomethylation | C10:Carbamidomethylation:1000.00;C12:Carbamidomethylation:1000.00 | PEAKS DB |
| K.LIGIISWGVGC(+57.02)GK.K | Y | 81.06 | 1358.7380 | 13 | 0.7 | 680.3768 | 2 | 38.46 | 1 | 25335 | BuCaMH04.raw | 1.7572E7 | 1 | 1 | 522 | 534 | Carbamidomethylation | C11:Carbamidomethylation:1000.00 | PEAKS DB |
| S.YIESHPWQAGIFVK.N | N | 74.84 | 1673.8566 | 14 | 0.8 | 558.9599 | 3 | 23.41 | 1 | 11817 | BuCaMH04.raw | 2.6952E6 | 1 | 1 | 320 | 333 |  |  | PEAKS DB |
| L.IGIISWGVGC(+57.02)GK.K | Y | 74.02 | 1245.6539 | 12 | 0.2 | 623.8344 | 2 | 30.99 | 1 | 18855 | BuCaMH04.raw | 1.1095E7 | 1 | 1 | 523 | 534 | Carbamidomethylation | C10:Carbamidomethylation:1000.00 | PEAKS DB |
| K.SSVTEQIFQVER.Y | Y | 69.15 | 1421.7151 | 12 | 0.4 | 711.8651 | 2 | 23.78 | 1 | 12070 | BuCaMH04.raw | 6.1912E7 | 1 | 1 | 381 | 392 |  |  | PEAKS DB |
| K.GGAYSYIESHPWQAGIFVK.N | N | 52.03 | 2109.0320 | 19 | -0.2 | 704.0178 | 3 | 33.22 | 1 | 20705 | BuCaMH04.raw | 2.0132E7 | 1 | 1 | 315 | 333 |  |  | PEAKS DB |
| R.TLDNDIALLK.L | N | 51.46 | 1114.6234 | 10 | 0.1 | 558.3190 | 2 | 25.03 | 1 | 13396 | BuCaMH04.raw | 5.4174E7 | 1 | 1 | 403 | 412 |  |  | PEAKS DB |
| total 7 peptides |  |  |  |  |  |  |  |  |  |  |  |  |  |  |  |  |  |  |  |
| --- | --- | --- | --- | --- | --- | --- | --- | --- | --- | --- | --- | --- | --- | --- | --- | --- | --- | --- | --- |

XP\_034291086.1

back to list

  

| Protein Coverage
| Supporting Peptides
|

Protein Coverage:

Supporting Peptides:

| Peptide | Uniq | -10lgP | Mass | Length | ppm | m/z | z | RT | Fraction | Scan | Source File | Area BuCaMH04\_B15 | #Feature | #Feature BuCaMH04\_B15 | Start | End | PTM | AScore | Found By |
| --- | --- | --- | --- | --- | --- | --- | --- | --- | --- | --- | --- | --- | --- | --- | --- | --- | --- | --- | --- |
| K.ALYSPNLFIC(+57.02)FC(+57.02)PPGFSGK.F | N | 103.15 | 2174.0330 | 19 | 0.0 | 1088.0238 | 2 | 52.04 | 1 | 37562 | BuCaMH04.raw | 4.0781E6 | 1 | 1 | 101 | 119 | Carbamidomethylation | C10:Carbamidomethylation:1000.00;C12:Carbamidomethylation:1000.00 | PEAKS DB |
| K.LIGIISWGVGC(+57.02)GK.K | Y | 81.06 | 1358.7380 | 13 | 0.7 | 680.3768 | 2 | 38.46 | 1 | 25335 | BuCaMH04.raw | 1.7572E7 | 1 | 1 | 522 | 534 | Carbamidomethylation | C11:Carbamidomethylation:1000.00 | PEAKS DB |
| S.YIESHPWQAGIFVK.N | N | 74.84 | 1673.8566 | 14 | 0.8 | 558.9599 | 3 | 23.41 | 1 | 11817 | BuCaMH04.raw | 2.6952E6 | 1 | 1 | 320 | 333 |  |  | PEAKS DB |
| L.IGIISWGVGC(+57.02)GK.K | Y | 74.02 | 1245.6539 | 12 | 0.2 | 623.8344 | 2 | 30.99 | 1 | 18855 | BuCaMH04.raw | 1.1095E7 | 1 | 1 | 523 | 534 | Carbamidomethylation | C10:Carbamidomethylation:1000.00 | PEAKS DB |
| K.SSVTEQIFQVER.Y | Y | 69.15 | 1421.7151 | 12 | 0.4 | 711.8651 | 2 | 23.78 | 1 | 12070 | BuCaMH04.raw | 6.1912E7 | 1 | 1 | 381 | 392 |  |  | PEAKS DB |
| K.GGAYSYIESHPWQAGIFVK.N | N | 52.03 | 2109.0320 | 19 | -0.2 | 704.0178 | 3 | 33.22 | 1 | 20705 | BuCaMH04.raw | 2.0132E7 | 1 | 1 | 315 | 333 |  |  | PEAKS DB |
| R.TLDNDIALLK.L | N | 51.46 | 1114.6234 | 10 | 0.1 | 558.3190 | 2 | 25.03 | 1 | 13396 | BuCaMH04.raw | 5.4174E7 | 1 | 1 | 403 | 412 |  |  | PEAKS DB |
| total 7 peptides |  |  |  |  |  |  |  |  |  |  |  |  |  |  |  |  |  |  |  |
| --- | --- | --- | --- | --- | --- | --- | --- | --- | --- | --- | --- | --- | --- | --- | --- | --- | --- | --- | --- |

AXL95288.1

back to list

  

| Protein Coverage
| Supporting Peptides
|

Protein Coverage:

Supporting Peptides:

| Peptide | Uniq | -10lgP | Mass | Length | ppm | m/z | z | RT | Fraction | Scan | Source File | Area BuCaMH04\_B15 | #Feature | #Feature BuCaMH04\_B15 | Start | End | PTM | AScore | Found By |
| --- | --- | --- | --- | --- | --- | --- | --- | --- | --- | --- | --- | --- | --- | --- | --- | --- | --- | --- | --- |
| K.HVVVVGAGMAGLSAAYVLAGAGHK.V | N | 106.63 | 2234.1992 | 24 | 0.7 | 745.7409 | 3 | 30.19 | 1 | 18119 | BuCaMH04.raw | 2.655E7 | 1 | 1 | 53 | 76 |  |  | PEAKS DB |
| K.STADIVINDLSLIHQLPK.K | Y | 99.16 | 1976.0942 | 18 | 0.8 | 989.0552 | 2 | 39.96 | 1 | 26902 | BuCaMH04.raw | 9.7537E6 | 1 | 1 | 406 | 423 |  |  | PEAKS DB |
| A.DIVINDLSLIHQLPK.K | N | 69.37 | 1716.9774 | 15 | 0.4 | 859.4963 | 2 | 37.46 | 1 | 24584 | BuCaMH04.raw | 4.3779E7 | 2 | 2 | 409 | 423 |  |  | PEAKS DB |
| D.DYEEFLEIAR.N | N | 65.68 | 1283.6033 | 10 | 0.6 | 642.8093 | 2 | 40.14 | 1 | 26895 | BuCaMH04.raw | 2.3334E6 | 1 | 1 | 33 | 42 |  |  | PEAKS DB |
| D.YEEFLEIAR.N | N | 59.77 | 1168.5764 | 9 | 0.4 | 585.2957 | 2 | 26.94 | 1 | 14950 | BuCaMH04.raw | 2.8615E6 | 1 | 1 | 34 | 42 |  |  | PEAKS DB |
| K.RFDEIVGGFDQLPIS.M | N | 52.08 | 1691.8518 | 15 | 0.2 | 846.9333 | 2 | 43.43 | 1 | 29880 | BuCaMH04.raw | 8.7751E5 | 1 | 1 | 250 | 264 |  |  | PEAKS DB |
| total 6 peptides |  |  |  |  |  |  |  |  |  |  |  |  |  |  |  |  |  |  |  |
| --- | --- | --- | --- | --- | --- | --- | --- | --- | --- | --- | --- | --- | --- | --- | --- | --- | --- | --- | --- |

AXL95287.1

back to list

  

| Protein Coverage
| Supporting Peptides
|

Protein Coverage:

Supporting Peptides:

| Peptide | Uniq | -10lgP | Mass | Length | ppm | m/z | z | RT | Fraction | Scan | Source File | Area BuCaMH04\_B15 | #Feature | #Feature BuCaMH04\_B15 | Start | End | PTM | AScore | Found By |
| --- | --- | --- | --- | --- | --- | --- | --- | --- | --- | --- | --- | --- | --- | --- | --- | --- | --- | --- | --- |
| K.HVVVVGAGMAGLSAAYVLAGAGHK.V | N | 106.63 | 2234.1992 | 24 | 0.7 | 745.7409 | 3 | 30.19 | 1 | 18119 | BuCaMH04.raw | 2.655E7 | 1 | 1 | 53 | 76 |  |  | PEAKS DB |
| K.STADIVINDLSLIHQLPK.K | Y | 99.16 | 1976.0942 | 18 | 0.8 | 989.0552 | 2 | 39.96 | 1 | 26902 | BuCaMH04.raw | 9.7537E6 | 1 | 1 | 406 | 423 |  |  | PEAKS DB |
| A.DIVINDLSLIHQLPK.K | N | 69.37 | 1716.9774 | 15 | 0.4 | 859.4963 | 2 | 37.46 | 1 | 24584 | BuCaMH04.raw | 4.3779E7 | 2 | 2 | 409 | 423 |  |  | PEAKS DB |
| D.DYEEFLEIAR.N | N | 65.68 | 1283.6033 | 10 | 0.6 | 642.8093 | 2 | 40.14 | 1 | 26895 | BuCaMH04.raw | 2.3334E6 | 1 | 1 | 33 | 42 |  |  | PEAKS DB |
| D.YEEFLEIAR.N | N | 59.77 | 1168.5764 | 9 | 0.4 | 585.2957 | 2 | 26.94 | 1 | 14950 | BuCaMH04.raw | 2.8615E6 | 1 | 1 | 34 | 42 |  |  | PEAKS DB |
| K.RFDEIVGGFDQLPIS.M | N | 52.08 | 1691.8518 | 15 | 0.2 | 846.9333 | 2 | 43.43 | 1 | 29880 | BuCaMH04.raw | 8.7751E5 | 1 | 1 | 250 | 264 |  |  | PEAKS DB |
| total 6 peptides |  |  |  |  |  |  |  |  |  |  |  |  |  |  |  |  |  |  |  |
| --- | --- | --- | --- | --- | --- | --- | --- | --- | --- | --- | --- | --- | --- | --- | --- | --- | --- | --- | --- |

S68801

back to list

  

| Protein Coverage
| Supporting Peptides
|

Protein Coverage:

Supporting Peptides:

| Peptide | Uniq | -10lgP | Mass | Length | ppm | m/z | z | RT | Fraction | Scan | Source File | Area BuCaMH04\_B15 | #Feature | #Feature BuCaMH04\_B15 | Start | End | PTM | AScore | Found By |
| --- | --- | --- | --- | --- | --- | --- | --- | --- | --- | --- | --- | --- | --- | --- | --- | --- | --- | --- | --- |
| K.DEGSYFLIYGLPGFSK.Y | N | 103.69 | 1791.8718 | 16 | 0.7 | 896.9438 | 2 | 56.99 | 1 | 42168 | BuCaMH04.raw | 1.8962E8 | 3 | 3 | 66 | 81 |  |  | PEAKS DB |
| LALQWIQNNIHPF.G | N | 74.47 | 1592.8463 | 13 | 1.1 | 797.4313 | 2 | 47.45 | 1 | 33715 | BuCaMH04.raw | 1.0832E7 | 1 | 1 | 1 | 13 |  |  | PEAKS DB |
| LALQWIQNNIHP.F | N | 72.94 | 1445.7780 | 12 | 0.3 | 723.8965 | 2 | 38.64 | 1 | 25651 | BuCaMH04.raw | 1.8096E7 | 1 | 1 | 1 | 12 |  |  | PEAKS DB |
| L.ETQLLLGVVK.D | Y | 63.74 | 1098.6648 | 10 | 0.1 | 550.3397 | 2 | 25.03 | 1 | 13123 | BuCaMH04.raw | 9.6369E7 | 1 | 1 | 56 | 65 |  |  | PEAKS DB |
| L.IYGLPGFSK.Y | N | 51.29 | 980.5331 | 9 | 0.8 | 491.2742 | 2 | 27.30 | 1 | 15385 | BuCaMH04.raw | 4.7137E6 | 1 | 1 | 73 | 81 |  |  | PEAKS DB |
| total 5 peptides |  |  |  |  |  |  |  |  |  |  |  |  |  |  |  |  |  |  |  |
| --- | --- | --- | --- | --- | --- | --- | --- | --- | --- | --- | --- | --- | --- | --- | --- | --- | --- | --- | --- |

ABN72543.1

back to list

  

| Protein Coverage
| Supporting Peptides
|

Protein Coverage:

Supporting Peptides:

| Peptide | Uniq | -10lgP | Mass | Length | ppm | m/z | z | RT | Fraction | Scan | Source File | Area BuCaMH04\_B15 | #Feature | #Feature BuCaMH04\_B15 | Start | End | PTM | AScore | Found By |
| --- | --- | --- | --- | --- | --- | --- | --- | --- | --- | --- | --- | --- | --- | --- | --- | --- | --- | --- | --- |
| R.YLYGEEVEGVAFVLFGVK.I | N | 101.23 | 2018.0400 | 18 | 0.2 | 1010.0275 | 2 | 64.43 | 1 | 48252 | BuCaMH04.raw | 7.4395E6 | 2 | 2 | 251 | 268 |  |  | PEAKS DB |
| E.VEGVAFVLFGVK.I | N | 94.83 | 1263.7227 | 12 | 0.3 | 632.8688 | 2 | 46.03 | 1 | 32338 | BuCaMH04.raw | 2.7212E6 | 1 | 1 | 257 | 268 |  |  | PEAKS DB |
| R.QNQYVVVQVTGPQVR.L | N | 77.22 | 1713.9163 | 15 | 0.6 | 572.3130 | 3 | 19.26 | 1 | 8230 | BuCaMH04.raw | 2.1525E7 | 2 | 2 | 100 | 114 |  |  | PEAKS DB |
| R.TDTEEQILVEAHGDNTPK.Q | N | 69.53 | 1995.9385 | 18 | 0.8 | 998.9773 | 2 | 13.46 | 1 | 3248 | BuCaMH04.raw | 7.9907E5 | 1 | 1 | 35 | 52 |  |  | PEAKS DB |
| K.GIYTPGSPVLYR.V | N | 52.60 | 1321.7030 | 12 | 0.4 | 661.8590 | 2 | 22.35 | 1 | 11114 | BuCaMH04.raw | 2.9354E7 | 1 | 1 | 135 | 146 |  |  | PEAKS DB |
| total 5 peptides |  |  |  |  |  |  |  |  |  |  |  |  |  |  |  |  |  |  |  |
| --- | --- | --- | --- | --- | --- | --- | --- | --- | --- | --- | --- | --- | --- | --- | --- | --- | --- | --- | --- |

JAC94932.1

back to list

  

| Protein Coverage
| Supporting Peptides
|

Protein Coverage:

Supporting Peptides:

| Peptide | Uniq | -10lgP | Mass | Length | ppm | m/z | z | RT | Fraction | Scan | Source File | Area BuCaMH04\_B15 | #Feature | #Feature BuCaMH04\_B15 | Start | End | PTM | AScore | Found By |
| --- | --- | --- | --- | --- | --- | --- | --- | --- | --- | --- | --- | --- | --- | --- | --- | --- | --- | --- | --- |
| E.LIDEEWSVLPYK.S | N | 89.30 | 1490.7656 | 12 | 0.9 | 746.3907 | 2 | 37.26 | 1 | 24455 | BuCaMH04.raw | 2.1123E7 | 1 | 1 | 308 | 319 |  |  | PEAKS DB |
| P.PQELIDEEWSVLPYK.S | N | 79.86 | 1844.9196 | 15 | 0.6 | 923.4677 | 2 | 41.67 | 1 | 28438 | BuCaMH04.raw | 7.64E6 | 1 | 1 | 305 | 319 |  |  | PEAKS DB |
| A.FLGIPFAEPPVGR.L | N | 70.62 | 1398.7659 | 13 | 0.9 | 700.3909 | 2 | 42.12 | 1 | 28605 | BuCaMH04.raw | 1.1387E8 | 1 | 1 | 64 | 76 |  |  | PEAKS DB |
| F.LGIPFAEPPVGR.L | N | 62.25 | 1251.6975 | 12 | 0.5 | 626.8563 | 2 | 29.35 | 1 | 17318 | BuCaMH04.raw | 6.6683E7 | 1 | 1 | 65 | 76 |  |  | PEAKS DB |
| S.AFLGIPFAEPPVGR.L | N | 58.55 | 1469.8030 | 14 | 0.6 | 735.9092 | 2 | 43.95 | 1 | 30470 | BuCaMH04.raw | 5.0781E6 | 1 | 1 | 63 | 76 |  |  | PEAKS DB |
| L.IDEEWSVLPYK.S | N | 57.66 | 1377.6816 | 11 | 0.6 | 689.8485 | 2 | 33.06 | 1 | 20610 | BuCaMH04.raw | 2.6958E6 | 1 | 1 | 309 | 319 |  |  | PEAKS DB |
| Q.ELIDEEWSVLPYK.S | N | 51.47 | 1619.8082 | 13 | 0.7 | 810.9119 | 2 | 42.09 | 1 | 28660 | BuCaMH04.raw | 4.6799E5 | 1 | 1 | 307 | 319 |  |  | PEAKS DB |
| total 7 peptides |  |  |  |  |  |  |  |  |  |  |  |  |  |  |  |  |  |  |  |
| --- | --- | --- | --- | --- | --- | --- | --- | --- | --- | --- | --- | --- | --- | --- | --- | --- | --- | --- | --- |

XP\_025032622.1

back to list

  

| Protein Coverage
| Supporting Peptides
|

Protein Coverage:

Supporting Peptides:

| Peptide | Uniq | -10lgP | Mass | Length | ppm | m/z | z | RT | Fraction | Scan | Source File | Area BuCaMH04\_B15 | #Feature | #Feature BuCaMH04\_B15 | Start | End | PTM | AScore | Found By |
| --- | --- | --- | --- | --- | --- | --- | --- | --- | --- | --- | --- | --- | --- | --- | --- | --- | --- | --- | --- |
| E.LIDEEWSVLPYK.S | N | 89.30 | 1490.7656 | 12 | 0.9 | 746.3907 | 2 | 37.26 | 1 | 24455 | BuCaMH04.raw | 2.1123E7 | 1 | 1 | 300 | 311 |  |  | PEAKS DB |
| T.PQELIDEEWSVLPYK.S | N | 79.86 | 1844.9196 | 15 | 0.6 | 923.4677 | 2 | 41.67 | 1 | 28438 | BuCaMH04.raw | 7.64E6 | 1 | 1 | 297 | 311 |  |  | PEAKS DB |
| A.FLGIPFAEPPVGR.R | N | 70.62 | 1398.7659 | 13 | 0.9 | 700.3909 | 2 | 42.12 | 1 | 28605 | BuCaMH04.raw | 1.1387E8 | 1 | 1 | 56 | 68 |  |  | PEAKS DB |
| F.LGIPFAEPPVGR.R | N | 62.25 | 1251.6975 | 12 | 0.5 | 626.8563 | 2 | 29.35 | 1 | 17318 | BuCaMH04.raw | 6.6683E7 | 1 | 1 | 57 | 68 |  |  | PEAKS DB |
| S.AFLGIPFAEPPVGR.R | N | 58.55 | 1469.8030 | 14 | 0.6 | 735.9092 | 2 | 43.95 | 1 | 30470 | BuCaMH04.raw | 5.0781E6 | 1 | 1 | 55 | 68 |  |  | PEAKS DB |
| L.IDEEWSVLPYK.S | N | 57.66 | 1377.6816 | 11 | 0.6 | 689.8485 | 2 | 33.06 | 1 | 20610 | BuCaMH04.raw | 2.6958E6 | 1 | 1 | 301 | 311 |  |  | PEAKS DB |
| Q.ELIDEEWSVLPYK.S | N | 51.47 | 1619.8082 | 13 | 0.7 | 810.9119 | 2 | 42.09 | 1 | 28660 | BuCaMH04.raw | 4.6799E5 | 1 | 1 | 299 | 311 |  |  | PEAKS DB |
| total 7 peptides |  |  |  |  |  |  |  |  |  |  |  |  |  |  |  |  |  |  |  |
| --- | --- | --- | --- | --- | --- | --- | --- | --- | --- | --- | --- | --- | --- | --- | --- | --- | --- | --- | --- |

XP\_025032620.1

back to list

  

| Protein Coverage
| Supporting Peptides
|

Protein Coverage:

Supporting Peptides:

| Peptide | Uniq | -10lgP | Mass | Length | ppm | m/z | z | RT | Fraction | Scan | Source File | Area BuCaMH04\_B15 | #Feature | #Feature BuCaMH04\_B15 | Start | End | PTM | AScore | Found By |
| --- | --- | --- | --- | --- | --- | --- | --- | --- | --- | --- | --- | --- | --- | --- | --- | --- | --- | --- | --- |
| E.LIDEEWSVLPYK.S | N | 89.30 | 1490.7656 | 12 | 0.9 | 746.3907 | 2 | 37.26 | 1 | 24455 | BuCaMH04.raw | 2.1123E7 | 1 | 1 | 300 | 311 |  |  | PEAKS DB |
| T.PQELIDEEWSVLPYK.S | N | 79.86 | 1844.9196 | 15 | 0.6 | 923.4677 | 2 | 41.67 | 1 | 28438 | BuCaMH04.raw | 7.64E6 | 1 | 1 | 297 | 311 |  |  | PEAKS DB |
| A.FLGIPFAEPPVGR.R | N | 70.62 | 1398.7659 | 13 | 0.9 | 700.3909 | 2 | 42.12 | 1 | 28605 | BuCaMH04.raw | 1.1387E8 | 1 | 1 | 56 | 68 |  |  | PEAKS DB |
| F.LGIPFAEPPVGR.R | N | 62.25 | 1251.6975 | 12 | 0.5 | 626.8563 | 2 | 29.35 | 1 | 17318 | BuCaMH04.raw | 6.6683E7 | 1 | 1 | 57 | 68 |  |  | PEAKS DB |
| S.AFLGIPFAEPPVGR.R | N | 58.55 | 1469.8030 | 14 | 0.6 | 735.9092 | 2 | 43.95 | 1 | 30470 | BuCaMH04.raw | 5.0781E6 | 1 | 1 | 55 | 68 |  |  | PEAKS DB |
| L.IDEEWSVLPYK.S | N | 57.66 | 1377.6816 | 11 | 0.6 | 689.8485 | 2 | 33.06 | 1 | 20610 | BuCaMH04.raw | 2.6958E6 | 1 | 1 | 301 | 311 |  |  | PEAKS DB |
| Q.ELIDEEWSVLPYK.S | N | 51.47 | 1619.8082 | 13 | 0.7 | 810.9119 | 2 | 42.09 | 1 | 28660 | BuCaMH04.raw | 4.6799E5 | 1 | 1 | 299 | 311 |  |  | PEAKS DB |
| total 7 peptides |  |  |  |  |  |  |  |  |  |  |  |  |  |  |  |  |  |  |  |
| --- | --- | --- | --- | --- | --- | --- | --- | --- | --- | --- | --- | --- | --- | --- | --- | --- | --- | --- | --- |

XP\_025032621.1

back to list

  

| Protein Coverage
| Supporting Peptides
|

Protein Coverage:

Supporting Peptides:

| Peptide | Uniq | -10lgP | Mass | Length | ppm | m/z | z | RT | Fraction | Scan | Source File | Area BuCaMH04\_B15 | #Feature | #Feature BuCaMH04\_B15 | Start | End | PTM | AScore | Found By |
| --- | --- | --- | --- | --- | --- | --- | --- | --- | --- | --- | --- | --- | --- | --- | --- | --- | --- | --- | --- |
| E.LIDEEWSVLPYK.S | N | 89.30 | 1490.7656 | 12 | 0.9 | 746.3907 | 2 | 37.26 | 1 | 24455 | BuCaMH04.raw | 2.1123E7 | 1 | 1 | 300 | 311 |  |  | PEAKS DB |
| T.PQELIDEEWSVLPYK.S | N | 79.86 | 1844.9196 | 15 | 0.6 | 923.4677 | 2 | 41.67 | 1 | 28438 | BuCaMH04.raw | 7.64E6 | 1 | 1 | 297 | 311 |  |  | PEAKS DB |
| A.FLGIPFAEPPVGR.R | N | 70.62 | 1398.7659 | 13 | 0.9 | 700.3909 | 2 | 42.12 | 1 | 28605 | BuCaMH04.raw | 1.1387E8 | 1 | 1 | 56 | 68 |  |  | PEAKS DB |
| F.LGIPFAEPPVGR.R | N | 62.25 | 1251.6975 | 12 | 0.5 | 626.8563 | 2 | 29.35 | 1 | 17318 | BuCaMH04.raw | 6.6683E7 | 1 | 1 | 57 | 68 |  |  | PEAKS DB |
| S.AFLGIPFAEPPVGR.R | N | 58.55 | 1469.8030 | 14 | 0.6 | 735.9092 | 2 | 43.95 | 1 | 30470 | BuCaMH04.raw | 5.0781E6 | 1 | 1 | 55 | 68 |  |  | PEAKS DB |
| L.IDEEWSVLPYK.S | N | 57.66 | 1377.6816 | 11 | 0.6 | 689.8485 | 2 | 33.06 | 1 | 20610 | BuCaMH04.raw | 2.6958E6 | 1 | 1 | 301 | 311 |  |  | PEAKS DB |
| Q.ELIDEEWSVLPYK.S | N | 51.47 | 1619.8082 | 13 | 0.7 | 810.9119 | 2 | 42.09 | 1 | 28660 | BuCaMH04.raw | 4.6799E5 | 1 | 1 | 299 | 311 |  |  | PEAKS DB |
| total 7 peptides |  |  |  |  |  |  |  |  |  |  |  |  |  |  |  |  |  |  |  |
| --- | --- | --- | --- | --- | --- | --- | --- | --- | --- | --- | --- | --- | --- | --- | --- | --- | --- | --- | --- |

XP\_025032619.1

back to list

  

| Protein Coverage
| Supporting Peptides
|

Protein Coverage:

Supporting Peptides:

| Peptide | Uniq | -10lgP | Mass | Length | ppm | m/z | z | RT | Fraction | Scan | Source File | Area BuCaMH04\_B15 | #Feature | #Feature BuCaMH04\_B15 | Start | End | PTM | AScore | Found By |
| --- | --- | --- | --- | --- | --- | --- | --- | --- | --- | --- | --- | --- | --- | --- | --- | --- | --- | --- | --- |
| E.LIDEEWSVLPYK.S | N | 89.30 | 1490.7656 | 12 | 0.9 | 746.3907 | 2 | 37.26 | 1 | 24455 | BuCaMH04.raw | 2.1123E7 | 1 | 1 | 340 | 351 |  |  | PEAKS DB |
| T.PQELIDEEWSVLPYK.S | N | 79.86 | 1844.9196 | 15 | 0.6 | 923.4677 | 2 | 41.67 | 1 | 28438 | BuCaMH04.raw | 7.64E6 | 1 | 1 | 337 | 351 |  |  | PEAKS DB |
| A.FLGIPFAEPPVGR.R | N | 70.62 | 1398.7659 | 13 | 0.9 | 700.3909 | 2 | 42.12 | 1 | 28605 | BuCaMH04.raw | 1.1387E8 | 1 | 1 | 96 | 108 |  |  | PEAKS DB |
| F.LGIPFAEPPVGR.R | N | 62.25 | 1251.6975 | 12 | 0.5 | 626.8563 | 2 | 29.35 | 1 | 17318 | BuCaMH04.raw | 6.6683E7 | 1 | 1 | 97 | 108 |  |  | PEAKS DB |
| S.AFLGIPFAEPPVGR.R | N | 58.55 | 1469.8030 | 14 | 0.6 | 735.9092 | 2 | 43.95 | 1 | 30470 | BuCaMH04.raw | 5.0781E6 | 1 | 1 | 95 | 108 |  |  | PEAKS DB |
| L.IDEEWSVLPYK.S | N | 57.66 | 1377.6816 | 11 | 0.6 | 689.8485 | 2 | 33.06 | 1 | 20610 | BuCaMH04.raw | 2.6958E6 | 1 | 1 | 341 | 351 |  |  | PEAKS DB |
| Q.ELIDEEWSVLPYK.S | N | 51.47 | 1619.8082 | 13 | 0.7 | 810.9119 | 2 | 42.09 | 1 | 28660 | BuCaMH04.raw | 4.6799E5 | 1 | 1 | 339 | 351 |  |  | PEAKS DB |
| total 7 peptides |  |  |  |  |  |  |  |  |  |  |  |  |  |  |  |  |  |  |  |
| --- | --- | --- | --- | --- | --- | --- | --- | --- | --- | --- | --- | --- | --- | --- | --- | --- | --- | --- | --- |

C3NJ

back to list

  

| Protein Coverage
| Supporting Peptides
|

Protein Coverage:

Supporting Peptides:

| Peptide | Uniq | -10lgP | Mass | Length | ppm | m/z | z | RT | Fraction | Scan | Source File | Area BuCaMH04\_B15 | #Feature | #Feature BuCaMH04\_B15 | Start | End | PTM | AScore | Found By |
| --- | --- | --- | --- | --- | --- | --- | --- | --- | --- | --- | --- | --- | --- | --- | --- | --- | --- | --- | --- |
| R.VELIYNPAFC(+57.02)SASTEGQR.Y | N | 80.36 | 2040.9574 | 18 | 0.6 | 1021.4866 | 2 | 29.17 | 1 | 17056 | BuCaMH04.raw | 9.7967E6 | 1 | 1 | 854 | 871 | Carbamidomethylation | C10:Carbamidomethylation:1000.00 | PEAKS DB |
| G.ALYTLITPAVLR.T | N | 77.10 | 1329.8020 | 12 | 0.6 | 665.9087 | 2 | 39.11 | 1 | 25893 | BuCaMH04.raw | 3.4301E7 | 1 | 1 | 23 | 34 |  |  | PEAKS DB |
| K.YFTYLILNK.G | N | 75.76 | 1173.6433 | 9 | 0.9 | 587.8295 | 2 | 33.34 | 1 | 20777 | BuCaMH04.raw | 1.8251E7 | 1 | 1 | 480 | 488 |  |  | PEAKS DB |
| H.GALYTLITPAVLR.T | N | 67.01 | 1386.8235 | 13 | 0.6 | 694.4194 | 2 | 39.60 | 1 | 26478 | BuCaMH04.raw | 7.2514E6 | 1 | 1 | 22 | 34 |  |  | PEAKS DB |
| E.YVLPSFEVR.L | N | 63.14 | 1108.5917 | 9 | 0.7 | 555.3035 | 2 | 28.54 | 1 | 16534 | BuCaMH04.raw | 1.3942E7 | 1 | 1 | 221 | 229 |  |  | PEAKS DB |
| total 5 peptides |  |  |  |  |  |  |  |  |  |  |  |  |  |  |  |  |  |  |  |
| --- | --- | --- | --- | --- | --- | --- | --- | --- | --- | --- | --- | --- | --- | --- | --- | --- | --- | --- | --- |

Q01833.1

back to list

  

| Protein Coverage
| Supporting Peptides
|

Protein Coverage:

Supporting Peptides:

| Peptide | Uniq | -10lgP | Mass | Length | ppm | m/z | z | RT | Fraction | Scan | Source File | Area BuCaMH04\_B15 | #Feature | #Feature BuCaMH04\_B15 | Start | End | PTM | AScore | Found By |
| --- | --- | --- | --- | --- | --- | --- | --- | --- | --- | --- | --- | --- | --- | --- | --- | --- | --- | --- | --- |
| R.VELIYNPAFC(+57.02)SASTEGQR.Y | N | 80.36 | 2040.9574 | 18 | 0.6 | 1021.4866 | 2 | 29.17 | 1 | 17056 | BuCaMH04.raw | 9.7967E6 | 1 | 1 | 854 | 871 | Carbamidomethylation | C10:Carbamidomethylation:1000.00 | PEAKS DB |
| G.ALYTLITPAVLR.T | N | 77.10 | 1329.8020 | 12 | 0.6 | 665.9087 | 2 | 39.11 | 1 | 25893 | BuCaMH04.raw | 3.4301E7 | 1 | 1 | 23 | 34 |  |  | PEAKS DB |
| K.YFTYLILNK.G | N | 75.76 | 1173.6433 | 9 | 0.9 | 587.8295 | 2 | 33.34 | 1 | 20777 | BuCaMH04.raw | 1.8251E7 | 1 | 1 | 480 | 488 |  |  | PEAKS DB |
| H.GALYTLITPAVLR.T | N | 67.01 | 1386.8235 | 13 | 0.6 | 694.4194 | 2 | 39.60 | 1 | 26478 | BuCaMH04.raw | 7.2514E6 | 1 | 1 | 22 | 34 |  |  | PEAKS DB |
| E.YVLPSFEVR.L | N | 63.14 | 1108.5917 | 9 | 0.7 | 555.3035 | 2 | 28.54 | 1 | 16534 | BuCaMH04.raw | 1.3942E7 | 1 | 1 | 221 | 229 |  |  | PEAKS DB |
| total 5 peptides |  |  |  |  |  |  |  |  |  |  |  |  |  |  |  |  |  |  |  |
| --- | --- | --- | --- | --- | --- | --- | --- | --- | --- | --- | --- | --- | --- | --- | --- | --- | --- | --- | --- |

AAA49385.1

back to list

  

| Protein Coverage
| Supporting Peptides
|

Protein Coverage:

Supporting Peptides:

| Peptide | Uniq | -10lgP | Mass | Length | ppm | m/z | z | RT | Fraction | Scan | Source File | Area BuCaMH04\_B15 | #Feature | #Feature BuCaMH04\_B15 | Start | End | PTM | AScore | Found By |
| --- | --- | --- | --- | --- | --- | --- | --- | --- | --- | --- | --- | --- | --- | --- | --- | --- | --- | --- | --- |
| R.VELIYNPAFC(+57.02)SASTEGQR.Y | N | 80.36 | 2040.9574 | 18 | 0.6 | 1021.4866 | 2 | 29.17 | 1 | 17056 | BuCaMH04.raw | 9.7967E6 | 1 | 1 | 854 | 871 | Carbamidomethylation | C10:Carbamidomethylation:1000.00 | PEAKS DB |
| G.ALYTLITPAVLR.T | N | 77.10 | 1329.8020 | 12 | 0.6 | 665.9087 | 2 | 39.11 | 1 | 25893 | BuCaMH04.raw | 3.4301E7 | 1 | 1 | 23 | 34 |  |  | PEAKS DB |
| K.YFTYLILNK.G | N | 75.76 | 1173.6433 | 9 | 0.9 | 587.8295 | 2 | 33.34 | 1 | 20777 | BuCaMH04.raw | 1.8251E7 | 1 | 1 | 480 | 488 |  |  | PEAKS DB |
| H.GALYTLITPAVLR.T | N | 67.01 | 1386.8235 | 13 | 0.6 | 694.4194 | 2 | 39.60 | 1 | 26478 | BuCaMH04.raw | 7.2514E6 | 1 | 1 | 22 | 34 |  |  | PEAKS DB |
| E.YVLPSFEVR.L | N | 63.14 | 1108.5917 | 9 | 0.7 | 555.3035 | 2 | 28.54 | 1 | 16534 | BuCaMH04.raw | 1.3942E7 | 1 | 1 | 221 | 229 |  |  | PEAKS DB |
| total 5 peptides |  |  |  |  |  |  |  |  |  |  |  |  |  |  |  |  |  |  |  |
| --- | --- | --- | --- | --- | --- | --- | --- | --- | --- | --- | --- | --- | --- | --- | --- | --- | --- | --- | --- |

AAZ81953.1

back to list

  

| Protein Coverage
| Supporting Peptides
|

Protein Coverage:

Supporting Peptides:

| Peptide | Uniq | -10lgP | Mass | Length | ppm | m/z | z | RT | Fraction | Scan | Source File | Area BuCaMH04\_B15 | #Feature | #Feature BuCaMH04\_B15 | Start | End | PTM | AScore | Found By |
| --- | --- | --- | --- | --- | --- | --- | --- | --- | --- | --- | --- | --- | --- | --- | --- | --- | --- | --- | --- |
| K.KVEGVAFVLFGVK.I | N | 96.20 | 1391.8176 | 13 | 0.9 | 464.9469 | 3 | 34.34 | 1 | 21741 | BuCaMH04.raw | 3.3382E6 | 2 | 2 | 259 | 271 |  |  | PEAKS DB |
| K.VEGVAFVLFGVK.I | N | 94.83 | 1263.7227 | 12 | 0.3 | 632.8688 | 2 | 46.03 | 1 | 32338 | BuCaMH04.raw | 2.7212E6 | 1 | 1 | 260 | 271 |  |  | PEAKS DB |
| R.VELLYNPAFC(+57.02)SASTEGQR.Y | N | 80.36 | 2040.9574 | 18 | 0.6 | 1021.4866 | 2 | 29.17 | 1 | 17056 | BuCaMH04.raw | 9.7967E6 | 1 | 1 | 855 | 872 | Carbamidomethylation | C10:Carbamidomethylation:1000.00 | PEAKS DB |
| K.YFTYLILTK.G | N | 80.29 | 1160.6481 | 9 | 1.2 | 581.3320 | 2 | 35.60 | 1 | 23009 | BuCaMH04.raw | 5.5623E6 | 1 | 1 | 481 | 489 |  |  | PEAKS DB |
| E.YVLPSFEVR.L | N | 63.14 | 1108.5917 | 9 | 0.7 | 555.3035 | 2 | 28.54 | 1 | 16534 | BuCaMH04.raw | 1.3942E7 | 1 | 1 | 222 | 230 |  |  | PEAKS DB |
| total 5 peptides |  |  |  |  |  |  |  |  |  |  |  |  |  |  |  |  |  |  |  |
| --- | --- | --- | --- | --- | --- | --- | --- | --- | --- | --- | --- | --- | --- | --- | --- | --- | --- | --- | --- |

AAX86641.1

back to list

  

| Protein Coverage
| Supporting Peptides
|

Protein Coverage:

Supporting Peptides:

| Peptide | Uniq | -10lgP | Mass | Length | ppm | m/z | z | RT | Fraction | Scan | Source File | Area BuCaMH04\_B15 | #Feature | #Feature BuCaMH04\_B15 | Start | End | PTM | AScore | Found By |
| --- | --- | --- | --- | --- | --- | --- | --- | --- | --- | --- | --- | --- | --- | --- | --- | --- | --- | --- | --- |
| K.KVEGVAFVLFGVK.I | N | 96.20 | 1391.8176 | 13 | 0.9 | 464.9469 | 3 | 34.34 | 1 | 21741 | BuCaMH04.raw | 3.3382E6 | 2 | 2 | 259 | 271 |  |  | PEAKS DB |
| K.VEGVAFVLFGVK.I | N | 94.83 | 1263.7227 | 12 | 0.3 | 632.8688 | 2 | 46.03 | 1 | 32338 | BuCaMH04.raw | 2.7212E6 | 1 | 1 | 260 | 271 |  |  | PEAKS DB |
| R.VELLYNPAFC(+57.02)SASTEGQR.Y | N | 80.36 | 2040.9574 | 18 | 0.6 | 1021.4866 | 2 | 29.17 | 1 | 17056 | BuCaMH04.raw | 9.7967E6 | 1 | 1 | 855 | 872 | Carbamidomethylation | C10:Carbamidomethylation:1000.00 | PEAKS DB |
| K.YFTYLILTK.G | N | 80.29 | 1160.6481 | 9 | 1.2 | 581.3320 | 2 | 35.60 | 1 | 23009 | BuCaMH04.raw | 5.5623E6 | 1 | 1 | 481 | 489 |  |  | PEAKS DB |
| E.YVLPSFEVR.L | N | 63.14 | 1108.5917 | 9 | 0.7 | 555.3035 | 2 | 28.54 | 1 | 16534 | BuCaMH04.raw | 1.3942E7 | 1 | 1 | 222 | 230 |  |  | PEAKS DB |
| total 5 peptides |  |  |  |  |  |  |  |  |  |  |  |  |  |  |  |  |  |  |  |
| --- | --- | --- | --- | --- | --- | --- | --- | --- | --- | --- | --- | --- | --- | --- | --- | --- | --- | --- | --- |

Q0ZZJ6.1

back to list

  

| Protein Coverage
| Supporting Peptides
|

Protein Coverage:

Supporting Peptides:

| Peptide | Uniq | -10lgP | Mass | Length | ppm | m/z | z | RT | Fraction | Scan | Source File | Area BuCaMH04\_B15 | #Feature | #Feature BuCaMH04\_B15 | Start | End | PTM | AScore | Found By |
| --- | --- | --- | --- | --- | --- | --- | --- | --- | --- | --- | --- | --- | --- | --- | --- | --- | --- | --- | --- |
| K.KVEGVAFVLFGVK.I | N | 96.20 | 1391.8176 | 13 | 0.9 | 464.9469 | 3 | 34.34 | 1 | 21741 | BuCaMH04.raw | 3.3382E6 | 2 | 2 | 259 | 271 |  |  | PEAKS DB |
| K.VEGVAFVLFGVK.I | N | 94.83 | 1263.7227 | 12 | 0.3 | 632.8688 | 2 | 46.03 | 1 | 32338 | BuCaMH04.raw | 2.7212E6 | 1 | 1 | 260 | 271 |  |  | PEAKS DB |
| R.VELLYNPAFC(+57.02)SASTEGQR.Y | N | 80.36 | 2040.9574 | 18 | 0.6 | 1021.4866 | 2 | 29.17 | 1 | 17056 | BuCaMH04.raw | 9.7967E6 | 1 | 1 | 855 | 872 | Carbamidomethylation | C10:Carbamidomethylation:1000.00 | PEAKS DB |
| K.YFTYLILTK.G | N | 80.29 | 1160.6481 | 9 | 1.2 | 581.3320 | 2 | 35.60 | 1 | 23009 | BuCaMH04.raw | 5.5623E6 | 1 | 1 | 481 | 489 |  |  | PEAKS DB |
| E.YVLPSFEVR.L | N | 63.14 | 1108.5917 | 9 | 0.7 | 555.3035 | 2 | 28.54 | 1 | 16534 | BuCaMH04.raw | 1.3942E7 | 1 | 1 | 222 | 230 |  |  | PEAKS DB |
| total 5 peptides |  |  |  |  |  |  |  |  |  |  |  |  |  |  |  |  |  |  |  |
| --- | --- | --- | --- | --- | --- | --- | --- | --- | --- | --- | --- | --- | --- | --- | --- | --- | --- | --- | --- |

ABA28745.1

back to list

  

| Protein Coverage
| Supporting Peptides
|

Protein Coverage:

Supporting Peptides:

| Peptide | Uniq | -10lgP | Mass | Length | ppm | m/z | z | RT | Fraction | Scan | Source File | Area BuCaMH04\_B15 | #Feature | #Feature BuCaMH04\_B15 | Start | End | PTM | AScore | Found By |
| --- | --- | --- | --- | --- | --- | --- | --- | --- | --- | --- | --- | --- | --- | --- | --- | --- | --- | --- | --- |
| K.KVEGVAFVLFGVK.I | N | 96.20 | 1391.8176 | 13 | 0.9 | 464.9469 | 3 | 34.34 | 1 | 21741 | BuCaMH04.raw | 3.3382E6 | 2 | 2 | 259 | 271 |  |  | PEAKS DB |
| K.VEGVAFVLFGVK.I | N | 94.83 | 1263.7227 | 12 | 0.3 | 632.8688 | 2 | 46.03 | 1 | 32338 | BuCaMH04.raw | 2.7212E6 | 1 | 1 | 260 | 271 |  |  | PEAKS DB |
| R.VELLYNPAFC(+57.02)SASTEGQR.Y | N | 80.36 | 2040.9574 | 18 | 0.6 | 1021.4866 | 2 | 29.17 | 1 | 17056 | BuCaMH04.raw | 9.7967E6 | 1 | 1 | 855 | 872 | Carbamidomethylation | C10:Carbamidomethylation:1000.00 | PEAKS DB |
| K.YFTYLILTK.G | N | 80.29 | 1160.6481 | 9 | 1.2 | 581.3320 | 2 | 35.60 | 1 | 23009 | BuCaMH04.raw | 5.5623E6 | 1 | 1 | 481 | 489 |  |  | PEAKS DB |
| E.YVLPSFEVR.L | N | 63.14 | 1108.5917 | 9 | 0.7 | 555.3035 | 2 | 28.54 | 1 | 16534 | BuCaMH04.raw | 1.3942E7 | 1 | 1 | 222 | 230 |  |  | PEAKS DB |
| total 5 peptides |  |  |  |  |  |  |  |  |  |  |  |  |  |  |  |  |  |  |  |
| --- | --- | --- | --- | --- | --- | --- | --- | --- | --- | --- | --- | --- | --- | --- | --- | --- | --- | --- | --- |

Q8QFW4.1

back to list

  

| Protein Coverage
| Supporting Peptides
|

Protein Coverage:

Supporting Peptides:

| Peptide | Uniq | -10lgP | Mass | Length | ppm | m/z | z | RT | Fraction | Scan | Source File | Area BuCaMH04\_B15 | #Feature | #Feature BuCaMH04\_B15 | Start | End | PTM | AScore | Found By |
| --- | --- | --- | --- | --- | --- | --- | --- | --- | --- | --- | --- | --- | --- | --- | --- | --- | --- | --- | --- |
| K.TWGEYTNYGC(+57.02)YC(+57.02)GAGGSGTPIDALDR.C | Y | 111.42 | 2840.1807 | 26 | 0.3 | 1421.0980 | 2 | 35.22 | 1 | 22626 | BuCaMH04.raw | 1.8481E8 | 2 | 2 | 45 | 70 | Carbamidomethylation | C10:Carbamidomethylation:1000.00;C12:Carbamidomethylation:1000.00 | PEAKS DB |
| R.TAALC(+57.02)FGDSEYIEGHKR.I | Y | 76.28 | 1952.9050 | 17 | -0.4 | 489.2333 | 4 | 13.31 | 1 | 3111 | BuCaMH04.raw | 1.231E6 | 1 | 1 | 123 | 139 | Carbamidomethylation | C5:Carbamidomethylation:1000.00 | PEAKS DB |
| R.TAALC(+57.02)FGDSEYIEGHK.R | N | 73.24 | 1796.8040 | 16 | -1.5 | 599.9410 | 3 | 19.83 | 1 | 8688 | BuCaMH04.raw | 3.584E7 | 1 | 1 | 123 | 138 | Carbamidomethylation | C5:Carbamidomethylation:1000.00 | PEAKS DB |
| Y.GC(+57.02)YC(+57.02)GAGGSGTPIDALDR.C | Y | 60.52 | 1825.7723 | 18 | 0.7 | 913.8941 | 2 | 19.49 | 1 | 8482 | BuCaMH04.raw | 3.4694E6 | 1 | 1 | 53 | 70 | Carbamidomethylation | C2:Carbamidomethylation:1000.00;C4:Carbamidomethylation:1000.00 | PEAKS DB |
| total 4 peptides |  |  |  |  |  |  |  |  |  |  |  |  |  |  |  |  |  |  |  |
| --- | --- | --- | --- | --- | --- | --- | --- | --- | --- | --- | --- | --- | --- | --- | --- | --- | --- | --- | --- |

AAL87003.1

back to list

  

| Protein Coverage
| Supporting Peptides
|

Protein Coverage:

Supporting Peptides:

| Peptide | Uniq | -10lgP | Mass | Length | ppm | m/z | z | RT | Fraction | Scan | Source File | Area BuCaMH04\_B15 | #Feature | #Feature BuCaMH04\_B15 | Start | End | PTM | AScore | Found By |
| --- | --- | --- | --- | --- | --- | --- | --- | --- | --- | --- | --- | --- | --- | --- | --- | --- | --- | --- | --- |
| K.TWGEYTNYGC(+57.02)YC(+57.02)GAGGSGTPIDALDR.C | Y | 111.42 | 2840.1807 | 26 | 0.3 | 1421.0980 | 2 | 35.22 | 1 | 22626 | BuCaMH04.raw | 1.8481E8 | 2 | 2 | 45 | 70 | Carbamidomethylation | C10:Carbamidomethylation:1000.00;C12:Carbamidomethylation:1000.00 | PEAKS DB |
| R.TAALC(+57.02)FGDSEYIEGHKR.I | Y | 76.28 | 1952.9050 | 17 | -0.4 | 489.2333 | 4 | 13.31 | 1 | 3111 | BuCaMH04.raw | 1.231E6 | 1 | 1 | 123 | 139 | Carbamidomethylation | C5:Carbamidomethylation:1000.00 | PEAKS DB |
| R.TAALC(+57.02)FGDSEYIEGHK.R | N | 73.24 | 1796.8040 | 16 | -1.5 | 599.9410 | 3 | 19.83 | 1 | 8688 | BuCaMH04.raw | 3.584E7 | 1 | 1 | 123 | 138 | Carbamidomethylation | C5:Carbamidomethylation:1000.00 | PEAKS DB |
| Y.GC(+57.02)YC(+57.02)GAGGSGTPIDALDR.C | Y | 60.52 | 1825.7723 | 18 | 0.7 | 913.8941 | 2 | 19.49 | 1 | 8482 | BuCaMH04.raw | 3.4694E6 | 1 | 1 | 53 | 70 | Carbamidomethylation | C2:Carbamidomethylation:1000.00;C4:Carbamidomethylation:1000.00 | PEAKS DB |
| total 4 peptides |  |  |  |  |  |  |  |  |  |  |  |  |  |  |  |  |  |  |  |
| --- | --- | --- | --- | --- | --- | --- | --- | --- | --- | --- | --- | --- | --- | --- | --- | --- | --- | --- | --- |

P0DI91.1

back to list

  

| Protein Coverage
| Supporting Peptides
|

Protein Coverage:

Supporting Peptides:

| Peptide | Uniq | -10lgP | Mass | Length | ppm | m/z | z | RT | Fraction | Scan | Source File | Area BuCaMH04\_B15 | #Feature | #Feature BuCaMH04\_B15 | Start | End | PTM | AScore | Found By |
| --- | --- | --- | --- | --- | --- | --- | --- | --- | --- | --- | --- | --- | --- | --- | --- | --- | --- | --- | --- |
| R.SPLEEC(+57.02)FQQNDYEEFLEIAR.N | Y | 111.04 | 2516.1165 | 20 | 0.1 | 1259.0657 | 2 | 48.30 | 1 | 34338 | BuCaMH04.raw | 1.8416E7 | 2 | 2 | 5 | 24 | Carbamidomethylation | C6:Carbamidomethylation:1000.00 | PEAKS DB |
| R.RSPLEEC(+57.02)FQQNDYEEFLEIAR.N | Y | 95.84 | 2672.2175 | 21 | 0.6 | 891.7470 | 3 | 41.45 | 1 | 28083 | BuCaMH04.raw | 1.2347E7 | 1 | 1 | 4 | 24 | Carbamidomethylation | C7:Carbamidomethylation:1000.00 | PEAKS DB |
| N.DYEEFLEIAR.N | N | 65.68 | 1283.6033 | 10 | 0.6 | 642.8093 | 2 | 40.14 | 1 | 26895 | BuCaMH04.raw | 2.3334E6 | 1 | 1 | 15 | 24 |  |  | PEAKS DB |
| D.YEEFLEIAR.N | N | 59.77 | 1168.5764 | 9 | 0.4 | 585.2957 | 2 | 26.94 | 1 | 14950 | BuCaMH04.raw | 2.8615E6 | 1 | 1 | 16 | 24 |  |  | PEAKS DB |
| total 4 peptides |  |  |  |  |  |  |  |  |  |  |  |  |  |  |  |  |  |  |  |
| --- | --- | --- | --- | --- | --- | --- | --- | --- | --- | --- | --- | --- | --- | --- | --- | --- | --- | --- | --- |

XP\_026544671.1

back to list

  

| Protein Coverage
| Supporting Peptides
|

Protein Coverage:

Supporting Peptides:

| Peptide | Uniq | -10lgP | Mass | Length | ppm | m/z | z | RT | Fraction | Scan | Source File | Area BuCaMH04\_B15 | #Feature | #Feature BuCaMH04\_B15 | Start | End | PTM | AScore | Found By |
| --- | --- | --- | --- | --- | --- | --- | --- | --- | --- | --- | --- | --- | --- | --- | --- | --- | --- | --- | --- |
| K.ALYSPNLFIC(+57.02)FC(+57.02)PPGFSGK.F | N | 103.15 | 2174.0330 | 19 | 0.0 | 1088.0238 | 2 | 52.04 | 1 | 37562 | BuCaMH04.raw | 4.0781E6 | 1 | 1 | 77 | 95 | Carbamidomethylation | C10:Carbamidomethylation:1000.00;C12:Carbamidomethylation:1000.00 | PEAKS DB |
| Q.LPDWTEC(+57.02)EVSGYGK.H | Y | 89.73 | 1639.7188 | 14 | 1.0 | 820.8675 | 2 | 25.16 | 1 | 13223 | BuCaMH04.raw | 4.9155E7 | 1 | 1 | 416 | 429 | Carbamidomethylation | C7:Carbamidomethylation:1000.00 | PEAKS DB |
| S.YIESHPWQAGIFVK.N | N | 74.84 | 1673.8566 | 14 | 0.8 | 558.9599 | 3 | 23.41 | 1 | 11817 | BuCaMH04.raw | 2.6952E6 | 1 | 1 | 297 | 310 |  |  | PEAKS DB |
| K.GGAYSYIESHPWQAGIFVK.N | N | 52.03 | 2109.0320 | 19 | -0.2 | 704.0178 | 3 | 33.22 | 1 | 20705 | BuCaMH04.raw | 2.0132E7 | 1 | 1 | 292 | 310 |  |  | PEAKS DB |
| R.TLDNDIALLK.L | N | 51.46 | 1114.6234 | 10 | 0.1 | 558.3190 | 2 | 25.03 | 1 | 13396 | BuCaMH04.raw | 5.4174E7 | 1 | 1 | 380 | 389 |  |  | PEAKS DB |
| total 5 peptides |  |  |  |  |  |  |  |  |  |  |  |  |  |  |  |  |  |  |  |
| --- | --- | --- | --- | --- | --- | --- | --- | --- | --- | --- | --- | --- | --- | --- | --- | --- | --- | --- | --- |

XP\_026570965.1

back to list

  

| Protein Coverage
| Supporting Peptides
|

Protein Coverage:

Supporting Peptides:

| Peptide | Uniq | -10lgP | Mass | Length | ppm | m/z | z | RT | Fraction | Scan | Source File | Area BuCaMH04\_B15 | #Feature | #Feature BuCaMH04\_B15 | Start | End | PTM | AScore | Found By |
| --- | --- | --- | --- | --- | --- | --- | --- | --- | --- | --- | --- | --- | --- | --- | --- | --- | --- | --- | --- |
| K.ALYSPNLFIC(+57.02)FC(+57.02)PPGFSGK.F | N | 103.15 | 2174.0330 | 19 | 0.0 | 1088.0238 | 2 | 52.04 | 1 | 37562 | BuCaMH04.raw | 4.0781E6 | 1 | 1 | 99 | 117 | Carbamidomethylation | C10:Carbamidomethylation:1000.00;C12:Carbamidomethylation:1000.00 | PEAKS DB |
| Q.LPDWTEC(+57.02)EVSGYGK.H | Y | 89.73 | 1639.7188 | 14 | 1.0 | 820.8675 | 2 | 25.16 | 1 | 13223 | BuCaMH04.raw | 4.9155E7 | 1 | 1 | 450 | 463 | Carbamidomethylation | C7:Carbamidomethylation:1000.00 | PEAKS DB |
| S.YIESHPWQAGIFVK.N | N | 74.84 | 1673.8566 | 14 | 0.8 | 558.9599 | 3 | 23.41 | 1 | 11817 | BuCaMH04.raw | 2.6952E6 | 1 | 1 | 331 | 344 |  |  | PEAKS DB |
| K.GGAYSYIESHPWQAGIFVK.N | N | 52.03 | 2109.0320 | 19 | -0.2 | 704.0178 | 3 | 33.22 | 1 | 20705 | BuCaMH04.raw | 2.0132E7 | 1 | 1 | 326 | 344 |  |  | PEAKS DB |
| R.TLDNDIALLK.L | N | 51.46 | 1114.6234 | 10 | 0.1 | 558.3190 | 2 | 25.03 | 1 | 13396 | BuCaMH04.raw | 5.4174E7 | 1 | 1 | 414 | 423 |  |  | PEAKS DB |
| total 5 peptides |  |  |  |  |  |  |  |  |  |  |  |  |  |  |  |  |  |  |  |
| --- | --- | --- | --- | --- | --- | --- | --- | --- | --- | --- | --- | --- | --- | --- | --- | --- | --- | --- | --- |

T\_156

back to list

  

| Protein Coverage
| Supporting Peptides
|

Protein Coverage:

Supporting Peptides:

| Peptide | Uniq | -10lgP | Mass | Length | ppm | m/z | z | RT | Fraction | Scan | Source File | Area BuCaMH04\_B15 | #Feature | #Feature BuCaMH04\_B15 | Start | End | PTM | AScore | Found By |
| --- | --- | --- | --- | --- | --- | --- | --- | --- | --- | --- | --- | --- | --- | --- | --- | --- | --- | --- | --- |
| R.NDQLLWLWR.D | N | 80.31 | 1242.6509 | 9 | 0.3 | 622.3329 | 2 | 47.58 | 1 | 33471 | BuCaMH04.raw | 1.0577E8 | 1 | 1 | 251 | 259 |  |  | PEAKS DB |
| K.TFHGLGVIDWENWR.P | N | 78.32 | 1728.8372 | 14 | 0.1 | 865.4259 | 2 | 39.69 | 1 | 26511 | BuCaMH04.raw | 5.0487E7 | 2 | 2 | 141 | 154 |  |  | PEAKS DB |
| R.DTLLLAENMRPGGYWG.Y | N | 73.71 | 1791.8613 | 16 | 1.1 | 896.9389 | 2 | 44.16 | 1 | 30560 | BuCaMH04.raw | 2.3022E6 | 1 | 1 | 205 | 220 |  |  | PEAKS DB |
| K.TFHGLGVIDWENWRPQWDR.N | N | 70.85 | 2411.1560 | 19 | 0.6 | 603.7966 | 4 | 41.36 | 1 | 28114 | BuCaMH04.raw | 1.9611E7 | 1 | 1 | 141 | 159 |  |  | PEAKS DB |
| K.APMYPNEPFLVFWNAPTTQC(+57.02)QMR.Y | Y | 65.96 | 2797.2815 | 23 | -0.8 | 933.4337 | 3 | 53.14 | 1 | 38556 | BuCaMH04.raw | 5.5591E5 | 1 | 1 | 44 | 66 | Carbamidomethylation | C20:Carbamidomethylation:1000.00 | PEAKS DB |
| K.HSDSNAFLHLFP.D | N | 59.60 | 1383.6571 | 12 | 0.1 | 462.2264 | 3 | 33.71 | 1 | 21163 | BuCaMH04.raw | 1.1569E6 | 1 | 1 | 396 | 407 |  |  | PEAKS DB |
| total 6 peptides |  |  |  |  |  |  |  |  |  |  |  |  |  |  |  |  |  |  |  |
| --- | --- | --- | --- | --- | --- | --- | --- | --- | --- | --- | --- | --- | --- | --- | --- | --- | --- | --- | --- |

T\_151

back to list

  

| Protein Coverage
| Supporting Peptides
|

Protein Coverage:

Supporting Peptides:

| Peptide | Uniq | -10lgP | Mass | Length | ppm | m/z | z | RT | Fraction | Scan | Source File | Area BuCaMH04\_B15 | #Feature | #Feature BuCaMH04\_B15 | Start | End | PTM | AScore | Found By |
| --- | --- | --- | --- | --- | --- | --- | --- | --- | --- | --- | --- | --- | --- | --- | --- | --- | --- | --- | --- |
| R.NDQLLWLWR.D | N | 80.31 | 1242.6509 | 9 | 0.3 | 622.3329 | 2 | 47.58 | 1 | 33471 | BuCaMH04.raw | 1.0577E8 | 1 | 1 | 251 | 259 |  |  | PEAKS DB |
| K.TFHGLGVIDWENWR.P | N | 78.32 | 1728.8372 | 14 | 0.1 | 865.4259 | 2 | 39.69 | 1 | 26511 | BuCaMH04.raw | 5.0487E7 | 2 | 2 | 141 | 154 |  |  | PEAKS DB |
| R.DTLLLAENMRPGGYWG.Y | N | 73.71 | 1791.8613 | 16 | 1.1 | 896.9389 | 2 | 44.16 | 1 | 30560 | BuCaMH04.raw | 2.3022E6 | 1 | 1 | 205 | 220 |  |  | PEAKS DB |
| K.TFHGLGVIDWENWRPQWDR.N | N | 70.85 | 2411.1560 | 19 | 0.6 | 603.7966 | 4 | 41.36 | 1 | 28114 | BuCaMH04.raw | 1.9611E7 | 1 | 1 | 141 | 159 |  |  | PEAKS DB |
| K.APMYPNEPFLVFWNAPTTQC(+57.02)QMR.Y | Y | 65.96 | 2797.2815 | 23 | -0.8 | 933.4337 | 3 | 53.14 | 1 | 38556 | BuCaMH04.raw | 5.5591E5 | 1 | 1 | 44 | 66 | Carbamidomethylation | C20:Carbamidomethylation:1000.00 | PEAKS DB |
| K.HSDSNAFLHLFP.D | N | 59.60 | 1383.6571 | 12 | 0.1 | 462.2264 | 3 | 33.71 | 1 | 21163 | BuCaMH04.raw | 1.1569E6 | 1 | 1 | 396 | 407 |  |  | PEAKS DB |
| total 6 peptides |  |  |  |  |  |  |  |  |  |  |  |  |  |  |  |  |  |  |  |
| --- | --- | --- | --- | --- | --- | --- | --- | --- | --- | --- | --- | --- | --- | --- | --- | --- | --- | --- | --- |

AXL96620.1

back to list

  

| Protein Coverage
| Supporting Peptides
|

Protein Coverage:

Supporting Peptides:

| Peptide | Uniq | -10lgP | Mass | Length | ppm | m/z | z | RT | Fraction | Scan | Source File | Area BuCaMH04\_B15 | #Feature | #Feature BuCaMH04\_B15 | Start | End | PTM | AScore | Found By |
| --- | --- | --- | --- | --- | --- | --- | --- | --- | --- | --- | --- | --- | --- | --- | --- | --- | --- | --- | --- |
| K.KVEGVAFVLFGVK.I | N | 96.20 | 1391.8176 | 13 | 0.9 | 464.9469 | 3 | 34.34 | 1 | 21741 | BuCaMH04.raw | 3.3382E6 | 2 | 2 | 261 | 273 |  |  | PEAKS DB |
| K.VEGVAFVLFGVK.I | N | 94.83 | 1263.7227 | 12 | 0.3 | 632.8688 | 2 | 46.03 | 1 | 32338 | BuCaMH04.raw | 2.7212E6 | 1 | 1 | 262 | 273 |  |  | PEAKS DB |
| Q.YFTYLILTK.G | N | 80.29 | 1160.6481 | 9 | 1.2 | 581.3320 | 2 | 35.60 | 1 | 23009 | BuCaMH04.raw | 5.5623E6 | 1 | 1 | 483 | 491 |  |  | PEAKS DB |
| R.TDTEEQILVEAHGDNTPK.Q | N | 69.53 | 1995.9385 | 18 | 0.8 | 998.9773 | 2 | 13.46 | 1 | 3248 | BuCaMH04.raw | 7.9907E5 | 1 | 1 | 35 | 52 |  |  | PEAKS DB |
| E.YVLPSFEVR.L | N | 63.14 | 1108.5917 | 9 | 0.7 | 555.3035 | 2 | 28.54 | 1 | 16534 | BuCaMH04.raw | 1.3942E7 | 1 | 1 | 224 | 232 |  |  | PEAKS DB |
| total 5 peptides |  |  |  |  |  |  |  |  |  |  |  |  |  |  |  |  |  |  |  |
| --- | --- | --- | --- | --- | --- | --- | --- | --- | --- | --- | --- | --- | --- | --- | --- | --- | --- | --- | --- |

JAC88987.1

back to list

  

| Protein Coverage
| Supporting Peptides
|

Protein Coverage:

Supporting Peptides:

| Peptide | Uniq | -10lgP | Mass | Length | ppm | m/z | z | RT | Fraction | Scan | Source File | Area BuCaMH04\_B15 | #Feature | #Feature BuCaMH04\_B15 | Start | End | PTM | AScore | Found By |
| --- | --- | --- | --- | --- | --- | --- | --- | --- | --- | --- | --- | --- | --- | --- | --- | --- | --- | --- | --- |
| K.KVEGVAFVLFGVK.I | N | 96.20 | 1391.8176 | 13 | 0.9 | 464.9469 | 3 | 34.34 | 1 | 21741 | BuCaMH04.raw | 3.3382E6 | 2 | 2 | 261 | 273 |  |  | PEAKS DB |
| K.VEGVAFVLFGVK.I | N | 94.83 | 1263.7227 | 12 | 0.3 | 632.8688 | 2 | 46.03 | 1 | 32338 | BuCaMH04.raw | 2.7212E6 | 1 | 1 | 262 | 273 |  |  | PEAKS DB |
| Q.YFTYLILTK.G | N | 80.29 | 1160.6481 | 9 | 1.2 | 581.3320 | 2 | 35.60 | 1 | 23009 | BuCaMH04.raw | 5.5623E6 | 1 | 1 | 483 | 491 |  |  | PEAKS DB |
| R.TDTEEQILVEAHGDNTPK.Q | N | 69.53 | 1995.9385 | 18 | 0.8 | 998.9773 | 2 | 13.46 | 1 | 3248 | BuCaMH04.raw | 7.9907E5 | 1 | 1 | 35 | 52 |  |  | PEAKS DB |
| E.YVLPSFEVR.L | N | 63.14 | 1108.5917 | 9 | 0.7 | 555.3035 | 2 | 28.54 | 1 | 16534 | BuCaMH04.raw | 1.3942E7 | 1 | 1 | 224 | 232 |  |  | PEAKS DB |
| total 5 peptides |  |  |  |  |  |  |  |  |  |  |  |  |  |  |  |  |  |  |  |
| --- | --- | --- | --- | --- | --- | --- | --- | --- | --- | --- | --- | --- | --- | --- | --- | --- | --- | --- | --- |

JAC95048.1

back to list

  

| Protein Coverage
| Supporting Peptides
|

Protein Coverage:

Supporting Peptides:

| Peptide | Uniq | -10lgP | Mass | Length | ppm | m/z | z | RT | Fraction | Scan | Source File | Area BuCaMH04\_B15 | #Feature | #Feature BuCaMH04\_B15 | Start | End | PTM | AScore | Found By |
| --- | --- | --- | --- | --- | --- | --- | --- | --- | --- | --- | --- | --- | --- | --- | --- | --- | --- | --- | --- |
| K.KVEGVAFVLFGVK.I | N | 96.20 | 1391.8176 | 13 | 0.9 | 464.9469 | 3 | 34.34 | 1 | 21741 | BuCaMH04.raw | 3.3382E6 | 2 | 2 | 261 | 273 |  |  | PEAKS DB |
| K.VEGVAFVLFGVK.I | N | 94.83 | 1263.7227 | 12 | 0.3 | 632.8688 | 2 | 46.03 | 1 | 32338 | BuCaMH04.raw | 2.7212E6 | 1 | 1 | 262 | 273 |  |  | PEAKS DB |
| Q.YFTYLILTK.G | N | 80.29 | 1160.6481 | 9 | 1.2 | 581.3320 | 2 | 35.60 | 1 | 23009 | BuCaMH04.raw | 5.5623E6 | 1 | 1 | 483 | 491 |  |  | PEAKS DB |
| R.TDTEEQILVEAHGDNTPK.Q | N | 69.53 | 1995.9385 | 18 | 0.8 | 998.9773 | 2 | 13.46 | 1 | 3248 | BuCaMH04.raw | 7.9907E5 | 1 | 1 | 35 | 52 |  |  | PEAKS DB |
| E.YVLPSFEVR.L | N | 63.14 | 1108.5917 | 9 | 0.7 | 555.3035 | 2 | 28.54 | 1 | 16534 | BuCaMH04.raw | 1.3942E7 | 1 | 1 | 224 | 232 |  |  | PEAKS DB |
| total 5 peptides |  |  |  |  |  |  |  |  |  |  |  |  |  |  |  |  |  |  |  |
| --- | --- | --- | --- | --- | --- | --- | --- | --- | --- | --- | --- | --- | --- | --- | --- | --- | --- | --- | --- |

XP\_034290336.1

back to list

  

| Protein Coverage
| Supporting Peptides
|

Protein Coverage:

Supporting Peptides:

| Peptide | Uniq | -10lgP | Mass | Length | ppm | m/z | z | RT | Fraction | Scan | Source File | Area BuCaMH04\_B15 | #Feature | #Feature BuCaMH04\_B15 | Start | End | PTM | AScore | Found By |
| --- | --- | --- | --- | --- | --- | --- | --- | --- | --- | --- | --- | --- | --- | --- | --- | --- | --- | --- | --- |
| K.KVEGVAFVLFGVK.I | N | 96.20 | 1391.8176 | 13 | 0.9 | 464.9469 | 3 | 34.34 | 1 | 21741 | BuCaMH04.raw | 3.3382E6 | 2 | 2 | 261 | 273 |  |  | PEAKS DB |
| K.VEGVAFVLFGVK.I | N | 94.83 | 1263.7227 | 12 | 0.3 | 632.8688 | 2 | 46.03 | 1 | 32338 | BuCaMH04.raw | 2.7212E6 | 1 | 1 | 262 | 273 |  |  | PEAKS DB |
| Q.YFTYLILTK.G | N | 80.29 | 1160.6481 | 9 | 1.2 | 581.3320 | 2 | 35.60 | 1 | 23009 | BuCaMH04.raw | 5.5623E6 | 1 | 1 | 483 | 491 |  |  | PEAKS DB |
| R.TDTEEQILVEAHGDNTPK.Q | N | 69.53 | 1995.9385 | 18 | 0.8 | 998.9773 | 2 | 13.46 | 1 | 3248 | BuCaMH04.raw | 7.9907E5 | 1 | 1 | 35 | 52 |  |  | PEAKS DB |
| E.YVLPSFEVR.L | N | 63.14 | 1108.5917 | 9 | 0.7 | 555.3035 | 2 | 28.54 | 1 | 16534 | BuCaMH04.raw | 1.3942E7 | 1 | 1 | 224 | 232 |  |  | PEAKS DB |
| total 5 peptides |  |  |  |  |  |  |  |  |  |  |  |  |  |  |  |  |  |  |  |
| --- | --- | --- | --- | --- | --- | --- | --- | --- | --- | --- | --- | --- | --- | --- | --- | --- | --- | --- | --- |

XP\_026524834.1

back to list

  

| Protein Coverage
| Supporting Peptides
|

Protein Coverage:

Supporting Peptides:

| Peptide | Uniq | -10lgP | Mass | Length | ppm | m/z | z | RT | Fraction | Scan | Source File | Area BuCaMH04\_B15 | #Feature | #Feature BuCaMH04\_B15 | Start | End | PTM | AScore | Found By |
| --- | --- | --- | --- | --- | --- | --- | --- | --- | --- | --- | --- | --- | --- | --- | --- | --- | --- | --- | --- |
| R.NDQLLWLWR.D | N | 80.31 | 1242.6509 | 9 | 0.3 | 622.3329 | 2 | 47.58 | 1 | 33471 | BuCaMH04.raw | 1.0577E8 | 1 | 1 | 253 | 261 |  |  | PEAKS DB |
| K.TFHGLGVIDWENWR.P | N | 78.32 | 1728.8372 | 14 | 0.1 | 865.4259 | 2 | 39.69 | 1 | 26511 | BuCaMH04.raw | 5.0487E7 | 2 | 2 | 143 | 156 |  |  | PEAKS DB |
| R.DTLLLAENMRPGGYWG.Y | N | 73.71 | 1791.8613 | 16 | 1.1 | 896.9389 | 2 | 44.16 | 1 | 30560 | BuCaMH04.raw | 2.3022E6 | 1 | 1 | 207 | 222 |  |  | PEAKS DB |
| K.TFHGLGVIDWENWRPQWDR.N | N | 70.85 | 2411.1560 | 19 | 0.6 | 603.7966 | 4 | 41.36 | 1 | 28114 | BuCaMH04.raw | 1.9611E7 | 1 | 1 | 143 | 161 |  |  | PEAKS DB |
| K.HSDSNAFLHLFP.E | N | 59.60 | 1383.6571 | 12 | 0.1 | 462.2264 | 3 | 33.71 | 1 | 21163 | BuCaMH04.raw | 1.1569E6 | 1 | 1 | 398 | 409 |  |  | PEAKS DB |
| K.DYALPVFVYAR.P | N | 59.05 | 1312.6815 | 11 | 0.3 | 657.3482 | 2 | 43.46 | 1 | 30072 | BuCaMH04.raw | 5.4049E6 | 1 | 1 | 303 | 313 |  |  | PEAKS DB |
| total 6 peptides |  |  |  |  |  |  |  |  |  |  |  |  |  |  |  |  |  |  |  |
| --- | --- | --- | --- | --- | --- | --- | --- | --- | --- | --- | --- | --- | --- | --- | --- | --- | --- | --- | --- |

XP\_026524835.1

back to list

  

| Protein Coverage
| Supporting Peptides
|

Protein Coverage:

Supporting Peptides:

| Peptide | Uniq | -10lgP | Mass | Length | ppm | m/z | z | RT | Fraction | Scan | Source File | Area BuCaMH04\_B15 | #Feature | #Feature BuCaMH04\_B15 | Start | End | PTM | AScore | Found By |
| --- | --- | --- | --- | --- | --- | --- | --- | --- | --- | --- | --- | --- | --- | --- | --- | --- | --- | --- | --- |
| R.NDQLLWLWR.D | N | 80.31 | 1242.6509 | 9 | 0.3 | 622.3329 | 2 | 47.58 | 1 | 33471 | BuCaMH04.raw | 1.0577E8 | 1 | 1 | 253 | 261 |  |  | PEAKS DB |
| K.TFHGLGVIDWENWR.P | N | 78.32 | 1728.8372 | 14 | 0.1 | 865.4259 | 2 | 39.69 | 1 | 26511 | BuCaMH04.raw | 5.0487E7 | 2 | 2 | 143 | 156 |  |  | PEAKS DB |
| R.DTLLLAENMRPGGYWG.Y | N | 73.71 | 1791.8613 | 16 | 1.1 | 896.9389 | 2 | 44.16 | 1 | 30560 | BuCaMH04.raw | 2.3022E6 | 1 | 1 | 207 | 222 |  |  | PEAKS DB |
| K.TFHGLGVIDWENWRPQWDR.N | N | 70.85 | 2411.1560 | 19 | 0.6 | 603.7966 | 4 | 41.36 | 1 | 28114 | BuCaMH04.raw | 1.9611E7 | 1 | 1 | 143 | 161 |  |  | PEAKS DB |
| K.HSDSNAFLHLFP.E | N | 59.60 | 1383.6571 | 12 | 0.1 | 462.2264 | 3 | 33.71 | 1 | 21163 | BuCaMH04.raw | 1.1569E6 | 1 | 1 | 398 | 409 |  |  | PEAKS DB |
| K.DYALPVFVYAR.P | N | 59.05 | 1312.6815 | 11 | 0.3 | 657.3482 | 2 | 43.46 | 1 | 30072 | BuCaMH04.raw | 5.4049E6 | 1 | 1 | 303 | 313 |  |  | PEAKS DB |
| total 6 peptides |  |  |  |  |  |  |  |  |  |  |  |  |  |  |  |  |  |  |  |
| --- | --- | --- | --- | --- | --- | --- | --- | --- | --- | --- | --- | --- | --- | --- | --- | --- | --- | --- | --- |

LAB44384.1

back to list

  

| Protein Coverage
| Supporting Peptides
|

Protein Coverage:

Supporting Peptides:

| Peptide | Uniq | -10lgP | Mass | Length | ppm | m/z | z | RT | Fraction | Scan | Source File | Area BuCaMH04\_B15 | #Feature | #Feature BuCaMH04\_B15 | Start | End | PTM | AScore | Found By |
| --- | --- | --- | --- | --- | --- | --- | --- | --- | --- | --- | --- | --- | --- | --- | --- | --- | --- | --- | --- |
| K.NFLVLLTDGQSYDDIR.G | Y | 93.34 | 1867.9315 | 16 | 0.4 | 934.9734 | 2 | 47.84 | 1 | 33812 | BuCaMH04.raw | 7.9745E6 | 1 | 1 | 464 | 479 |  |  | PEAKS DB |
| K.EFTGLEQIVPNIVR.A | Y | 90.56 | 1613.8777 | 14 | 0.9 | 807.9468 | 2 | 42.57 | 1 | 29138 | BuCaMH04.raw | 8.2477E6 | 1 | 1 | 522 | 535 |  |  | PEAKS DB |
| R.IEFSFTDYK.T | Y | 71.60 | 1148.5389 | 9 | 1.6 | 575.2776 | 2 | 29.37 | 1 | 17166 | BuCaMH04.raw | 4.3527E6 | 1 | 1 | 413 | 421 |  |  | PEAKS DB |
| K.AGINIFSVGIAWAPLEDLR.D | Y | 59.53 | 2041.0996 | 19 | -0.2 | 1021.5569 | 2 | 63.63 | 1 | 47676 | BuCaMH04.raw | 1.4863E6 | 1 | 1 | 488 | 506 |  |  | PEAKS DB |
| F.SVGIAWAPLEDLR.D | Y | 56.33 | 1425.7616 | 13 | 0.7 | 713.8885 | 2 | 42.57 | 1 | 29084 | BuCaMH04.raw | 2.1106E6 | 1 | 1 | 494 | 506 |  |  | PEAKS DB |
| total 5 peptides |  |  |  |  |  |  |  |  |  |  |  |  |  |  |  |  |  |  |  |
| --- | --- | --- | --- | --- | --- | --- | --- | --- | --- | --- | --- | --- | --- | --- | --- | --- | --- | --- | --- |

LAA86986.1

back to list

  

| Protein Coverage
| Supporting Peptides
|

Protein Coverage:

Supporting Peptides:

| Peptide | Uniq | -10lgP | Mass | Length | ppm | m/z | z | RT | Fraction | Scan | Source File | Area BuCaMH04\_B15 | #Feature | #Feature BuCaMH04\_B15 | Start | End | PTM | AScore | Found By |
| --- | --- | --- | --- | --- | --- | --- | --- | --- | --- | --- | --- | --- | --- | --- | --- | --- | --- | --- | --- |
| K.NFLVLLTDGQSYDDIR.G | Y | 93.34 | 1867.9315 | 16 | 0.4 | 934.9734 | 2 | 47.84 | 1 | 33812 | BuCaMH04.raw | 7.9745E6 | 1 | 1 | 175 | 190 |  |  | PEAKS DB |
| K.EFTGLEQIVPNIVR.A | Y | 90.56 | 1613.8777 | 14 | 0.9 | 807.9468 | 2 | 42.57 | 1 | 29138 | BuCaMH04.raw | 8.2477E6 | 1 | 1 | 233 | 246 |  |  | PEAKS DB |
| R.IEFSFTDYK.T | Y | 71.60 | 1148.5389 | 9 | 1.6 | 575.2776 | 2 | 29.37 | 1 | 17166 | BuCaMH04.raw | 4.3527E6 | 1 | 1 | 124 | 132 |  |  | PEAKS DB |
| K.AGINIFSVGIAWAPLEDLR.D | Y | 59.53 | 2041.0996 | 19 | -0.2 | 1021.5569 | 2 | 63.63 | 1 | 47676 | BuCaMH04.raw | 1.4863E6 | 1 | 1 | 199 | 217 |  |  | PEAKS DB |
| F.SVGIAWAPLEDLR.D | Y | 56.33 | 1425.7616 | 13 | 0.7 | 713.8885 | 2 | 42.57 | 1 | 29084 | BuCaMH04.raw | 2.1106E6 | 1 | 1 | 205 | 217 |  |  | PEAKS DB |
| total 5 peptides |  |  |  |  |  |  |  |  |  |  |  |  |  |  |  |  |  |  |  |
| --- | --- | --- | --- | --- | --- | --- | --- | --- | --- | --- | --- | --- | --- | --- | --- | --- | --- | --- | --- |

LAB57337.1

back to list

  

| Protein Coverage
| Supporting Peptides
|

Protein Coverage:

Supporting Peptides:

| Peptide | Uniq | -10lgP | Mass | Length | ppm | m/z | z | RT | Fraction | Scan | Source File | Area BuCaMH04\_B15 | #Feature | #Feature BuCaMH04\_B15 | Start | End | PTM | AScore | Found By |
| --- | --- | --- | --- | --- | --- | --- | --- | --- | --- | --- | --- | --- | --- | --- | --- | --- | --- | --- | --- |
| K.NFLVLLTDGQSYDDIR.G | Y | 93.34 | 1867.9315 | 16 | 0.4 | 934.9734 | 2 | 47.84 | 1 | 33812 | BuCaMH04.raw | 7.9745E6 | 1 | 1 | 193 | 208 |  |  | PEAKS DB |
| K.EFTGLEQIVPNIVR.A | Y | 90.56 | 1613.8777 | 14 | 0.9 | 807.9468 | 2 | 42.57 | 1 | 29138 | BuCaMH04.raw | 8.2477E6 | 1 | 1 | 251 | 264 |  |  | PEAKS DB |
| R.IEFSFTDYK.T | Y | 71.60 | 1148.5389 | 9 | 1.6 | 575.2776 | 2 | 29.37 | 1 | 17166 | BuCaMH04.raw | 4.3527E6 | 1 | 1 | 142 | 150 |  |  | PEAKS DB |
| K.AGINIFSVGIAWAPLEDLR.D | Y | 59.53 | 2041.0996 | 19 | -0.2 | 1021.5569 | 2 | 63.63 | 1 | 47676 | BuCaMH04.raw | 1.4863E6 | 1 | 1 | 217 | 235 |  |  | PEAKS DB |
| F.SVGIAWAPLEDLR.D | Y | 56.33 | 1425.7616 | 13 | 0.7 | 713.8885 | 2 | 42.57 | 1 | 29084 | BuCaMH04.raw | 2.1106E6 | 1 | 1 | 223 | 235 |  |  | PEAKS DB |
| total 5 peptides |  |  |  |  |  |  |  |  |  |  |  |  |  |  |  |  |  |  |  |
| --- | --- | --- | --- | --- | --- | --- | --- | --- | --- | --- | --- | --- | --- | --- | --- | --- | --- | --- | --- |

XP\_026566518.1

back to list

  

| Protein Coverage
| Supporting Peptides
|

Protein Coverage:

Supporting Peptides:

| Peptide | Uniq | -10lgP | Mass | Length | ppm | m/z | z | RT | Fraction | Scan | Source File | Area BuCaMH04\_B15 | #Feature | #Feature BuCaMH04\_B15 | Start | End | PTM | AScore | Found By |
| --- | --- | --- | --- | --- | --- | --- | --- | --- | --- | --- | --- | --- | --- | --- | --- | --- | --- | --- | --- |
| K.NFLVLLTDGQSYDDIR.G | Y | 93.34 | 1867.9315 | 16 | 0.4 | 934.9734 | 2 | 47.84 | 1 | 33812 | BuCaMH04.raw | 7.9745E6 | 1 | 1 | 464 | 479 |  |  | PEAKS DB |
| K.EFTGLEQIVPNIVR.A | Y | 90.56 | 1613.8777 | 14 | 0.9 | 807.9468 | 2 | 42.57 | 1 | 29138 | BuCaMH04.raw | 8.2477E6 | 1 | 1 | 522 | 535 |  |  | PEAKS DB |
| R.IEFSFTDYK.T | Y | 71.60 | 1148.5389 | 9 | 1.6 | 575.2776 | 2 | 29.37 | 1 | 17166 | BuCaMH04.raw | 4.3527E6 | 1 | 1 | 413 | 421 |  |  | PEAKS DB |
| K.AGINIFSVGIAWAPLEDLR.D | Y | 59.53 | 2041.0996 | 19 | -0.2 | 1021.5569 | 2 | 63.63 | 1 | 47676 | BuCaMH04.raw | 1.4863E6 | 1 | 1 | 488 | 506 |  |  | PEAKS DB |
| F.SVGIAWAPLEDLR.D | Y | 56.33 | 1425.7616 | 13 | 0.7 | 713.8885 | 2 | 42.57 | 1 | 29084 | BuCaMH04.raw | 2.1106E6 | 1 | 1 | 494 | 506 |  |  | PEAKS DB |
| total 5 peptides |  |  |  |  |  |  |  |  |  |  |  |  |  |  |  |  |  |  |  |
| --- | --- | --- | --- | --- | --- | --- | --- | --- | --- | --- | --- | --- | --- | --- | --- | --- | --- | --- | --- |

XP\_034290338.1

back to list

  

| Protein Coverage
| Supporting Peptides
|

Protein Coverage:

Supporting Peptides:

| Peptide | Uniq | -10lgP | Mass | Length | ppm | m/z | z | RT | Fraction | Scan | Source File | Area BuCaMH04\_B15 | #Feature | #Feature BuCaMH04\_B15 | Start | End | PTM | AScore | Found By |
| --- | --- | --- | --- | --- | --- | --- | --- | --- | --- | --- | --- | --- | --- | --- | --- | --- | --- | --- | --- |
| K.KVEGVAFVLFGVK.I | N | 96.20 | 1391.8176 | 13 | 0.9 | 464.9469 | 3 | 34.34 | 1 | 21741 | BuCaMH04.raw | 3.3382E6 | 2 | 2 | 259 | 271 |  |  | PEAKS DB |
| K.VEGVAFVLFGVK.I | N | 94.83 | 1263.7227 | 12 | 0.3 | 632.8688 | 2 | 46.03 | 1 | 32338 | BuCaMH04.raw | 2.7212E6 | 1 | 1 | 260 | 271 |  |  | PEAKS DB |
| Q.YFTYLILTK.G | N | 80.29 | 1160.6481 | 9 | 1.2 | 581.3320 | 2 | 35.60 | 1 | 23009 | BuCaMH04.raw | 5.5623E6 | 1 | 1 | 481 | 489 |  |  | PEAKS DB |
| R.TDTEEQILVEAHGDNTPK.Q | N | 69.53 | 1995.9385 | 18 | 0.8 | 998.9773 | 2 | 13.46 | 1 | 3248 | BuCaMH04.raw | 7.9907E5 | 1 | 1 | 35 | 52 |  |  | PEAKS DB |
| total 4 peptides |  |  |  |  |  |  |  |  |  |  |  |  |  |  |  |  |  |  |  |
| --- | --- | --- | --- | --- | --- | --- | --- | --- | --- | --- | --- | --- | --- | --- | --- | --- | --- | --- | --- |

1U4J

back to list

  

| Protein Coverage
| Supporting Peptides
|

Protein Coverage:

Supporting Peptides:

| Peptide | Uniq | -10lgP | Mass | Length | ppm | m/z | z | RT | Fraction | Scan | Source File | Area BuCaMH04\_B15 | #Feature | #Feature BuCaMH04\_B15 | Start | End | PTM | AScore | Found By |
| --- | --- | --- | --- | --- | --- | --- | --- | --- | --- | --- | --- | --- | --- | --- | --- | --- | --- | --- | --- |
| G.YGC(+57.02)YC(+57.02)GYGGSGTPVDELDR.C | N | 96.11 | 2124.8516 | 19 | 0.0 | 1063.4331 | 2 | 22.61 | 1 | 11043 | BuCaMH04.raw | 4.7004E9 | 2 | 2 | 23 | 41 | Carbamidomethylation | C3:Carbamidomethylation:1000.00;C5:Carbamidomethylation:1000.00 | PEAKS DB |
| I.GYGC(+57.02)YC(+57.02)GYGGSGTPVDELDR.C | Y | 82.73 | 2181.8730 | 20 | 0.2 | 1091.9440 | 2 | 22.96 | 1 | 11568 | BuCaMH04.raw | 8.4167E8 | 3 | 3 | 22 | 41 | Carbamidomethylation | C4:Carbamidomethylation:1000.00;C6:Carbamidomethylation:1000.00 | PEAKS DB |
| Y.GC(+57.02)YC(+57.02)GYGGSGTPVDELDR.C | N | 69.63 | 1961.7883 | 18 | -0.1 | 981.9014 | 2 | 20.01 | 1 | 8793 | BuCaMH04.raw | 4.946E7 | 1 | 1 | 24 | 41 | Carbamidomethylation | C2:Carbamidomethylation:1000.00;C4:Carbamidomethylation:1000.00 | PEAKS DB |
| C.YC(+57.02)GYGGSGTPVDELDR.C | N | 63.69 | 1744.7362 | 16 | 0.1 | 873.3755 | 2 | 19.14 | 1 | 8251 | BuCaMH04.raw | 1.6782E7 | 1 | 1 | 26 | 41 | Carbamidomethylation | C2:Carbamidomethylation:1000.00 | PEAKS DB |
| G.C(+57.02)YC(+57.02)GYGGSGTPVDELDR.C | N | 51.59 | 1904.7668 | 17 | 0.9 | 953.3916 | 2 | 19.93 | 1 | 8813 | BuCaMH04.raw | 3.883E6 | 1 | 1 | 25 | 41 | Carbamidomethylation | C1:Carbamidomethylation:1000.00;C3:Carbamidomethylation:1000.00 | PEAKS DB |
| total 5 peptides |  |  |  |  |  |  |  |  |  |  |  |  |  |  |  |  |  |  |  |
| --- | --- | --- | --- | --- | --- | --- | --- | --- | --- | --- | --- | --- | --- | --- | --- | --- | --- | --- | --- |

1G0Z

back to list

  

| Protein Coverage
| Supporting Peptides
|

Protein Coverage:

Supporting Peptides:

| Peptide | Uniq | -10lgP | Mass | Length | ppm | m/z | z | RT | Fraction | Scan | Source File | Area BuCaMH04\_B15 | #Feature | #Feature BuCaMH04\_B15 | Start | End | PTM | AScore | Found By |
| --- | --- | --- | --- | --- | --- | --- | --- | --- | --- | --- | --- | --- | --- | --- | --- | --- | --- | --- | --- |
| G.YGC(+57.02)YC(+57.02)GYGGSGTPVDELDR.C | N | 96.11 | 2124.8516 | 19 | 0.0 | 1063.4331 | 2 | 22.61 | 1 | 11043 | BuCaMH04.raw | 4.7004E9 | 2 | 2 | 23 | 41 | Carbamidomethylation | C3:Carbamidomethylation:1000.00;C5:Carbamidomethylation:1000.00 | PEAKS DB |
| I.GYGC(+57.02)YC(+57.02)GYGGSGTPVDELDR.C | Y | 82.73 | 2181.8730 | 20 | 0.2 | 1091.9440 | 2 | 22.96 | 1 | 11568 | BuCaMH04.raw | 8.4167E8 | 3 | 3 | 22 | 41 | Carbamidomethylation | C4:Carbamidomethylation:1000.00;C6:Carbamidomethylation:1000.00 | PEAKS DB |
| Y.GC(+57.02)YC(+57.02)GYGGSGTPVDELDR.C | N | 69.63 | 1961.7883 | 18 | -0.1 | 981.9014 | 2 | 20.01 | 1 | 8793 | BuCaMH04.raw | 4.946E7 | 1 | 1 | 24 | 41 | Carbamidomethylation | C2:Carbamidomethylation:1000.00;C4:Carbamidomethylation:1000.00 | PEAKS DB |
| C.YC(+57.02)GYGGSGTPVDELDR.C | N | 63.69 | 1744.7362 | 16 | 0.1 | 873.3755 | 2 | 19.14 | 1 | 8251 | BuCaMH04.raw | 1.6782E7 | 1 | 1 | 26 | 41 | Carbamidomethylation | C2:Carbamidomethylation:1000.00 | PEAKS DB |
| G.C(+57.02)YC(+57.02)GYGGSGTPVDELDR.C | N | 51.59 | 1904.7668 | 17 | 0.9 | 953.3916 | 2 | 19.93 | 1 | 8813 | BuCaMH04.raw | 3.883E6 | 1 | 1 | 25 | 41 | Carbamidomethylation | C1:Carbamidomethylation:1000.00;C3:Carbamidomethylation:1000.00 | PEAKS DB |
| total 5 peptides |  |  |  |  |  |  |  |  |  |  |  |  |  |  |  |  |  |  |  |
| --- | --- | --- | --- | --- | --- | --- | --- | --- | --- | --- | --- | --- | --- | --- | --- | --- | --- | --- | --- |

Q6SLM1.1

back to list

  

| Protein Coverage
| Supporting Peptides
|

Protein Coverage:

Supporting Peptides:

| Peptide | Uniq | -10lgP | Mass | Length | ppm | m/z | z | RT | Fraction | Scan | Source File | Area BuCaMH04\_B15 | #Feature | #Feature BuCaMH04\_B15 | Start | End | PTM | AScore | Found By |
| --- | --- | --- | --- | --- | --- | --- | --- | --- | --- | --- | --- | --- | --- | --- | --- | --- | --- | --- | --- |
| G.YGC(+57.02)YC(+57.02)GYGGSGTPVDELDR.C | N | 96.11 | 2124.8516 | 19 | 0.0 | 1063.4331 | 2 | 22.61 | 1 | 11043 | BuCaMH04.raw | 4.7004E9 | 2 | 2 | 42 | 60 | Carbamidomethylation | C3:Carbamidomethylation:1000.00;C5:Carbamidomethylation:1000.00 | PEAKS DB |
| I.GYGC(+57.02)YC(+57.02)GYGGSGTPVDELDR.C | Y | 82.73 | 2181.8730 | 20 | 0.2 | 1091.9440 | 2 | 22.96 | 1 | 11568 | BuCaMH04.raw | 8.4167E8 | 3 | 3 | 41 | 60 | Carbamidomethylation | C4:Carbamidomethylation:1000.00;C6:Carbamidomethylation:1000.00 | PEAKS DB |
| Y.GC(+57.02)YC(+57.02)GYGGSGTPVDELDR.C | N | 69.63 | 1961.7883 | 18 | -0.1 | 981.9014 | 2 | 20.01 | 1 | 8793 | BuCaMH04.raw | 4.946E7 | 1 | 1 | 43 | 60 | Carbamidomethylation | C2:Carbamidomethylation:1000.00;C4:Carbamidomethylation:1000.00 | PEAKS DB |
| C.YC(+57.02)GYGGSGTPVDELDR.C | N | 63.69 | 1744.7362 | 16 | 0.1 | 873.3755 | 2 | 19.14 | 1 | 8251 | BuCaMH04.raw | 1.6782E7 | 1 | 1 | 45 | 60 | Carbamidomethylation | C2:Carbamidomethylation:1000.00 | PEAKS DB |
| G.C(+57.02)YC(+57.02)GYGGSGTPVDELDR.C | N | 51.59 | 1904.7668 | 17 | 0.9 | 953.3916 | 2 | 19.93 | 1 | 8813 | BuCaMH04.raw | 3.883E6 | 1 | 1 | 44 | 60 | Carbamidomethylation | C1:Carbamidomethylation:1000.00;C3:Carbamidomethylation:1000.00 | PEAKS DB |
| total 5 peptides |  |  |  |  |  |  |  |  |  |  |  |  |  |  |  |  |  |  |  |
| --- | --- | --- | --- | --- | --- | --- | --- | --- | --- | --- | --- | --- | --- | --- | --- | --- | --- | --- | --- |

AAR19228.1

back to list

  

| Protein Coverage
| Supporting Peptides
|

Protein Coverage:

Supporting Peptides:

| Peptide | Uniq | -10lgP | Mass | Length | ppm | m/z | z | RT | Fraction | Scan | Source File | Area BuCaMH04\_B15 | #Feature | #Feature BuCaMH04\_B15 | Start | End | PTM | AScore | Found By |
| --- | --- | --- | --- | --- | --- | --- | --- | --- | --- | --- | --- | --- | --- | --- | --- | --- | --- | --- | --- |
| G.YGC(+57.02)YC(+57.02)GYGGSGTPVDELDR.C | N | 96.11 | 2124.8516 | 19 | 0.0 | 1063.4331 | 2 | 22.61 | 1 | 11043 | BuCaMH04.raw | 4.7004E9 | 2 | 2 | 42 | 60 | Carbamidomethylation | C3:Carbamidomethylation:1000.00;C5:Carbamidomethylation:1000.00 | PEAKS DB |
| I.GYGC(+57.02)YC(+57.02)GYGGSGTPVDELDR.C | Y | 82.73 | 2181.8730 | 20 | 0.2 | 1091.9440 | 2 | 22.96 | 1 | 11568 | BuCaMH04.raw | 8.4167E8 | 3 | 3 | 41 | 60 | Carbamidomethylation | C4:Carbamidomethylation:1000.00;C6:Carbamidomethylation:1000.00 | PEAKS DB |
| Y.GC(+57.02)YC(+57.02)GYGGSGTPVDELDR.C | N | 69.63 | 1961.7883 | 18 | -0.1 | 981.9014 | 2 | 20.01 | 1 | 8793 | BuCaMH04.raw | 4.946E7 | 1 | 1 | 43 | 60 | Carbamidomethylation | C2:Carbamidomethylation:1000.00;C4:Carbamidomethylation:1000.00 | PEAKS DB |
| C.YC(+57.02)GYGGSGTPVDELDR.C | N | 63.69 | 1744.7362 | 16 | 0.1 | 873.3755 | 2 | 19.14 | 1 | 8251 | BuCaMH04.raw | 1.6782E7 | 1 | 1 | 45 | 60 | Carbamidomethylation | C2:Carbamidomethylation:1000.00 | PEAKS DB |
| G.C(+57.02)YC(+57.02)GYGGSGTPVDELDR.C | N | 51.59 | 1904.7668 | 17 | 0.9 | 953.3916 | 2 | 19.93 | 1 | 8813 | BuCaMH04.raw | 3.883E6 | 1 | 1 | 44 | 60 | Carbamidomethylation | C1:Carbamidomethylation:1000.00;C3:Carbamidomethylation:1000.00 | PEAKS DB |
| total 5 peptides |  |  |  |  |  |  |  |  |  |  |  |  |  |  |  |  |  |  |  |
| --- | --- | --- | --- | --- | --- | --- | --- | --- | --- | --- | --- | --- | --- | --- | --- | --- | --- | --- | --- |

ABU63164.1

back to list

  

| Protein Coverage
| Supporting Peptides
|

Protein Coverage:

Supporting Peptides:

| Peptide | Uniq | -10lgP | Mass | Length | ppm | m/z | z | RT | Fraction | Scan | Source File | Area BuCaMH04\_B15 | #Feature | #Feature BuCaMH04\_B15 | Start | End | PTM | AScore | Found By |
| --- | --- | --- | --- | --- | --- | --- | --- | --- | --- | --- | --- | --- | --- | --- | --- | --- | --- | --- | --- |
| S.YGC(+57.02)YC(+57.02)GYGGSGTPVDELDR.C | N | 96.11 | 2124.8516 | 19 | 0.0 | 1063.4331 | 2 | 22.61 | 1 | 11043 | BuCaMH04.raw | 4.7004E9 | 2 | 2 | 50 | 68 | Carbamidomethylation | C3:Carbamidomethylation:1000.00;C5:Carbamidomethylation:1000.00 | PEAKS DB |
| Y.VSYGC(+57.02)YC(+57.02)GYGGSGTPVDELDR.C | Y | 78.30 | 2310.9521 | 21 | -0.2 | 1156.4832 | 2 | 24.38 | 1 | 12610 | BuCaMH04.raw | 2.1599E7 | 1 | 1 | 48 | 68 | Carbamidomethylation | C5:Carbamidomethylation:1000.00;C7:Carbamidomethylation:1000.00 | PEAKS DB |
| Y.GC(+57.02)YC(+57.02)GYGGSGTPVDELDR.C | N | 69.63 | 1961.7883 | 18 | -0.1 | 981.9014 | 2 | 20.01 | 1 | 8793 | BuCaMH04.raw | 4.946E7 | 1 | 1 | 51 | 68 | Carbamidomethylation | C2:Carbamidomethylation:1000.00;C4:Carbamidomethylation:1000.00 | PEAKS DB |
| C.YC(+57.02)GYGGSGTPVDELDR.C | N | 63.69 | 1744.7362 | 16 | 0.1 | 873.3755 | 2 | 19.14 | 1 | 8251 | BuCaMH04.raw | 1.6782E7 | 1 | 1 | 53 | 68 | Carbamidomethylation | C2:Carbamidomethylation:1000.00 | PEAKS DB |
| G.C(+57.02)YC(+57.02)GYGGSGTPVDELDR.C | N | 51.59 | 1904.7668 | 17 | 0.9 | 953.3916 | 2 | 19.93 | 1 | 8813 | BuCaMH04.raw | 3.883E6 | 1 | 1 | 52 | 68 | Carbamidomethylation | C1:Carbamidomethylation:1000.00;C3:Carbamidomethylation:1000.00 | PEAKS DB |
| total 5 peptides |  |  |  |  |  |  |  |  |  |  |  |  |  |  |  |  |  |  |  |
| --- | --- | --- | --- | --- | --- | --- | --- | --- | --- | --- | --- | --- | --- | --- | --- | --- | --- | --- | --- |

ABU63166.1

back to list

  

| Protein Coverage
| Supporting Peptides
|

Protein Coverage:

Supporting Peptides:

| Peptide | Uniq | -10lgP | Mass | Length | ppm | m/z | z | RT | Fraction | Scan | Source File | Area BuCaMH04\_B15 | #Feature | #Feature BuCaMH04\_B15 | Start | End | PTM | AScore | Found By |
| --- | --- | --- | --- | --- | --- | --- | --- | --- | --- | --- | --- | --- | --- | --- | --- | --- | --- | --- | --- |
| S.YGC(+57.02)YC(+57.02)GYGGSGTPVDELDR.C | N | 96.11 | 2124.8516 | 19 | 0.0 | 1063.4331 | 2 | 22.61 | 1 | 11043 | BuCaMH04.raw | 4.7004E9 | 2 | 2 | 50 | 68 | Carbamidomethylation | C3:Carbamidomethylation:1000.00;C5:Carbamidomethylation:1000.00 | PEAKS DB |
| Y.VSYGC(+57.02)YC(+57.02)GYGGSGTPVDELDR.C | Y | 78.30 | 2310.9521 | 21 | -0.2 | 1156.4832 | 2 | 24.38 | 1 | 12610 | BuCaMH04.raw | 2.1599E7 | 1 | 1 | 48 | 68 | Carbamidomethylation | C5:Carbamidomethylation:1000.00;C7:Carbamidomethylation:1000.00 | PEAKS DB |
| Y.GC(+57.02)YC(+57.02)GYGGSGTPVDELDR.C | N | 69.63 | 1961.7883 | 18 | -0.1 | 981.9014 | 2 | 20.01 | 1 | 8793 | BuCaMH04.raw | 4.946E7 | 1 | 1 | 51 | 68 | Carbamidomethylation | C2:Carbamidomethylation:1000.00;C4:Carbamidomethylation:1000.00 | PEAKS DB |
| C.YC(+57.02)GYGGSGTPVDELDR.C | N | 63.69 | 1744.7362 | 16 | 0.1 | 873.3755 | 2 | 19.14 | 1 | 8251 | BuCaMH04.raw | 1.6782E7 | 1 | 1 | 53 | 68 | Carbamidomethylation | C2:Carbamidomethylation:1000.00 | PEAKS DB |
| G.C(+57.02)YC(+57.02)GYGGSGTPVDELDR.C | N | 51.59 | 1904.7668 | 17 | 0.9 | 953.3916 | 2 | 19.93 | 1 | 8813 | BuCaMH04.raw | 3.883E6 | 1 | 1 | 52 | 68 | Carbamidomethylation | C1:Carbamidomethylation:1000.00;C3:Carbamidomethylation:1000.00 | PEAKS DB |
| total 5 peptides |  |  |  |  |  |  |  |  |  |  |  |  |  |  |  |  |  |  |  |
| --- | --- | --- | --- | --- | --- | --- | --- | --- | --- | --- | --- | --- | --- | --- | --- | --- | --- | --- | --- |

JAB52792.1

back to list

  

| Protein Coverage
| Supporting Peptides
|

Protein Coverage:

Supporting Peptides:

| Peptide | Uniq | -10lgP | Mass | Length | ppm | m/z | z | RT | Fraction | Scan | Source File | Area BuCaMH04\_B15 | #Feature | #Feature BuCaMH04\_B15 | Start | End | PTM | AScore | Found By |
| --- | --- | --- | --- | --- | --- | --- | --- | --- | --- | --- | --- | --- | --- | --- | --- | --- | --- | --- | --- |
| N.YGC(+57.02)YC(+57.02)GYGGSGTPVDELDR.C | N | 96.11 | 2124.8516 | 19 | 0.0 | 1063.4331 | 2 | 22.61 | 1 | 11043 | BuCaMH04.raw | 4.7004E9 | 2 | 2 | 50 | 68 | Carbamidomethylation | C3:Carbamidomethylation:1000.00;C5:Carbamidomethylation:1000.00 | PEAKS DB |
| T.NYGC(+57.02)YC(+57.02)GYGGSGTPVDELDR.C | Y | 74.91 | 2238.8945 | 20 | -0.8 | 1120.4536 | 2 | 22.65 | 1 | 11172 | BuCaMH04.raw | 4.8615E7 | 2 | 2 | 49 | 68 | Carbamidomethylation | C4:Carbamidomethylation:1000.00;C6:Carbamidomethylation:1000.00 | PEAKS DB |
| Y.GC(+57.02)YC(+57.02)GYGGSGTPVDELDR.C | N | 69.63 | 1961.7883 | 18 | -0.1 | 981.9014 | 2 | 20.01 | 1 | 8793 | BuCaMH04.raw | 4.946E7 | 1 | 1 | 51 | 68 | Carbamidomethylation | C2:Carbamidomethylation:1000.00;C4:Carbamidomethylation:1000.00 | PEAKS DB |
| C.YC(+57.02)GYGGSGTPVDELDR.C | N | 63.69 | 1744.7362 | 16 | 0.1 | 873.3755 | 2 | 19.14 | 1 | 8251 | BuCaMH04.raw | 1.6782E7 | 1 | 1 | 53 | 68 | Carbamidomethylation | C2:Carbamidomethylation:1000.00 | PEAKS DB |
| G.C(+57.02)YC(+57.02)GYGGSGTPVDELDR.C | N | 51.59 | 1904.7668 | 17 | 0.9 | 953.3916 | 2 | 19.93 | 1 | 8813 | BuCaMH04.raw | 3.883E6 | 1 | 1 | 52 | 68 | Carbamidomethylation | C1:Carbamidomethylation:1000.00;C3:Carbamidomethylation:1000.00 | PEAKS DB |
| total 5 peptides |  |  |  |  |  |  |  |  |  |  |  |  |  |  |  |  |  |  |  |
| --- | --- | --- | --- | --- | --- | --- | --- | --- | --- | --- | --- | --- | --- | --- | --- | --- | --- | --- | --- |

AAZ29513.1

back to list

  

| Protein Coverage
| Supporting Peptides
|

Protein Coverage:

Supporting Peptides:

| Peptide | Uniq | -10lgP | Mass | Length | ppm | m/z | z | RT | Fraction | Scan | Source File | Area BuCaMH04\_B15 | #Feature | #Feature BuCaMH04\_B15 | Start | End | PTM | AScore | Found By |
| --- | --- | --- | --- | --- | --- | --- | --- | --- | --- | --- | --- | --- | --- | --- | --- | --- | --- | --- | --- |
| N.YGC(+57.02)YC(+57.02)GYGGSGTPVDELDR.C | N | 96.11 | 2124.8516 | 19 | 0.0 | 1063.4331 | 2 | 22.61 | 1 | 11043 | BuCaMH04.raw | 4.7004E9 | 2 | 2 | 42 | 60 | Carbamidomethylation | C3:Carbamidomethylation:1000.00;C5:Carbamidomethylation:1000.00 | PEAKS DB |
| T.NYGC(+57.02)YC(+57.02)GYGGSGTPVDELDR.C | Y | 74.91 | 2238.8945 | 20 | -0.8 | 1120.4536 | 2 | 22.65 | 1 | 11172 | BuCaMH04.raw | 4.8615E7 | 2 | 2 | 41 | 60 | Carbamidomethylation | C4:Carbamidomethylation:1000.00;C6:Carbamidomethylation:1000.00 | PEAKS DB |
| Y.GC(+57.02)YC(+57.02)GYGGSGTPVDELDR.C | N | 69.63 | 1961.7883 | 18 | -0.1 | 981.9014 | 2 | 20.01 | 1 | 8793 | BuCaMH04.raw | 4.946E7 | 1 | 1 | 43 | 60 | Carbamidomethylation | C2:Carbamidomethylation:1000.00;C4:Carbamidomethylation:1000.00 | PEAKS DB |
| C.YC(+57.02)GYGGSGTPVDELDR.C | N | 63.69 | 1744.7362 | 16 | 0.1 | 873.3755 | 2 | 19.14 | 1 | 8251 | BuCaMH04.raw | 1.6782E7 | 1 | 1 | 45 | 60 | Carbamidomethylation | C2:Carbamidomethylation:1000.00 | PEAKS DB |
| G.C(+57.02)YC(+57.02)GYGGSGTPVDELDR.C | N | 51.59 | 1904.7668 | 17 | 0.9 | 953.3916 | 2 | 19.93 | 1 | 8813 | BuCaMH04.raw | 3.883E6 | 1 | 1 | 44 | 60 | Carbamidomethylation | C1:Carbamidomethylation:1000.00;C3:Carbamidomethylation:1000.00 | PEAKS DB |
| total 5 peptides |  |  |  |  |  |  |  |  |  |  |  |  |  |  |  |  |  |  |  |
| --- | --- | --- | --- | --- | --- | --- | --- | --- | --- | --- | --- | --- | --- | --- | --- | --- | --- | --- | --- |

JAB52790.1

back to list

  

| Protein Coverage
| Supporting Peptides
|

Protein Coverage:

Supporting Peptides:

| Peptide | Uniq | -10lgP | Mass | Length | ppm | m/z | z | RT | Fraction | Scan | Source File | Area BuCaMH04\_B15 | #Feature | #Feature BuCaMH04\_B15 | Start | End | PTM | AScore | Found By |
| --- | --- | --- | --- | --- | --- | --- | --- | --- | --- | --- | --- | --- | --- | --- | --- | --- | --- | --- | --- |
| N.YGC(+57.02)YC(+57.02)GYGGSGTPVDELDR.C | N | 96.11 | 2124.8516 | 19 | 0.0 | 1063.4331 | 2 | 22.61 | 1 | 11043 | BuCaMH04.raw | 4.7004E9 | 2 | 2 | 50 | 68 | Carbamidomethylation | C3:Carbamidomethylation:1000.00;C5:Carbamidomethylation:1000.00 | PEAKS DB |
| T.NYGC(+57.02)YC(+57.02)GYGGSGTPVDELDR.C | Y | 74.91 | 2238.8945 | 20 | -0.8 | 1120.4536 | 2 | 22.65 | 1 | 11172 | BuCaMH04.raw | 4.8615E7 | 2 | 2 | 49 | 68 | Carbamidomethylation | C4:Carbamidomethylation:1000.00;C6:Carbamidomethylation:1000.00 | PEAKS DB |
| Y.GC(+57.02)YC(+57.02)GYGGSGTPVDELDR.C | N | 69.63 | 1961.7883 | 18 | -0.1 | 981.9014 | 2 | 20.01 | 1 | 8793 | BuCaMH04.raw | 4.946E7 | 1 | 1 | 51 | 68 | Carbamidomethylation | C2:Carbamidomethylation:1000.00;C4:Carbamidomethylation:1000.00 | PEAKS DB |
| C.YC(+57.02)GYGGSGTPVDELDR.C | N | 63.69 | 1744.7362 | 16 | 0.1 | 873.3755 | 2 | 19.14 | 1 | 8251 | BuCaMH04.raw | 1.6782E7 | 1 | 1 | 53 | 68 | Carbamidomethylation | C2:Carbamidomethylation:1000.00 | PEAKS DB |
| G.C(+57.02)YC(+57.02)GYGGSGTPVDELDR.C | N | 51.59 | 1904.7668 | 17 | 0.9 | 953.3916 | 2 | 19.93 | 1 | 8813 | BuCaMH04.raw | 3.883E6 | 1 | 1 | 52 | 68 | Carbamidomethylation | C1:Carbamidomethylation:1000.00;C3:Carbamidomethylation:1000.00 | PEAKS DB |
| total 5 peptides |  |  |  |  |  |  |  |  |  |  |  |  |  |  |  |  |  |  |  |
| --- | --- | --- | --- | --- | --- | --- | --- | --- | --- | --- | --- | --- | --- | --- | --- | --- | --- | --- | --- |

JAB52794.1

back to list

  

| Protein Coverage
| Supporting Peptides
|

Protein Coverage:

Supporting Peptides:

| Peptide | Uniq | -10lgP | Mass | Length | ppm | m/z | z | RT | Fraction | Scan | Source File | Area BuCaMH04\_B15 | #Feature | #Feature BuCaMH04\_B15 | Start | End | PTM | AScore | Found By |
| --- | --- | --- | --- | --- | --- | --- | --- | --- | --- | --- | --- | --- | --- | --- | --- | --- | --- | --- | --- |
| N.YGC(+57.02)YC(+57.02)GYGGSGTPVDELDR.C | N | 96.11 | 2124.8516 | 19 | 0.0 | 1063.4331 | 2 | 22.61 | 1 | 11043 | BuCaMH04.raw | 4.7004E9 | 2 | 2 | 48 | 66 | Carbamidomethylation | C3:Carbamidomethylation:1000.00;C5:Carbamidomethylation:1000.00 | PEAKS DB |
| T.NYGC(+57.02)YC(+57.02)GYGGSGTPVDELDR.C | Y | 74.91 | 2238.8945 | 20 | -0.8 | 1120.4536 | 2 | 22.65 | 1 | 11172 | BuCaMH04.raw | 4.8615E7 | 2 | 2 | 47 | 66 | Carbamidomethylation | C4:Carbamidomethylation:1000.00;C6:Carbamidomethylation:1000.00 | PEAKS DB |
| Y.GC(+57.02)YC(+57.02)GYGGSGTPVDELDR.C | N | 69.63 | 1961.7883 | 18 | -0.1 | 981.9014 | 2 | 20.01 | 1 | 8793 | BuCaMH04.raw | 4.946E7 | 1 | 1 | 49 | 66 | Carbamidomethylation | C2:Carbamidomethylation:1000.00;C4:Carbamidomethylation:1000.00 | PEAKS DB |
| C.YC(+57.02)GYGGSGTPVDELDR.C | N | 63.69 | 1744.7362 | 16 | 0.1 | 873.3755 | 2 | 19.14 | 1 | 8251 | BuCaMH04.raw | 1.6782E7 | 1 | 1 | 51 | 66 | Carbamidomethylation | C2:Carbamidomethylation:1000.00 | PEAKS DB |
| G.C(+57.02)YC(+57.02)GYGGSGTPVDELDR.C | N | 51.59 | 1904.7668 | 17 | 0.9 | 953.3916 | 2 | 19.93 | 1 | 8813 | BuCaMH04.raw | 3.883E6 | 1 | 1 | 50 | 66 | Carbamidomethylation | C1:Carbamidomethylation:1000.00;C3:Carbamidomethylation:1000.00 | PEAKS DB |
| total 5 peptides |  |  |  |  |  |  |  |  |  |  |  |  |  |  |  |  |  |  |  |
| --- | --- | --- | --- | --- | --- | --- | --- | --- | --- | --- | --- | --- | --- | --- | --- | --- | --- | --- | --- |

JAB52809.1

back to list

  

| Protein Coverage
| Supporting Peptides
|

Protein Coverage:

Supporting Peptides:

| Peptide | Uniq | -10lgP | Mass | Length | ppm | m/z | z | RT | Fraction | Scan | Source File | Area BuCaMH04\_B15 | #Feature | #Feature BuCaMH04\_B15 | Start | End | PTM | AScore | Found By |
| --- | --- | --- | --- | --- | --- | --- | --- | --- | --- | --- | --- | --- | --- | --- | --- | --- | --- | --- | --- |
| N.YGC(+57.02)YC(+57.02)GYGGSGTPVDELDR.C | N | 96.11 | 2124.8516 | 19 | 0.0 | 1063.4331 | 2 | 22.61 | 1 | 11043 | BuCaMH04.raw | 4.7004E9 | 2 | 2 | 48 | 66 | Carbamidomethylation | C3:Carbamidomethylation:1000.00;C5:Carbamidomethylation:1000.00 | PEAKS DB |
| T.NYGC(+57.02)YC(+57.02)GYGGSGTPVDELDR.C | Y | 74.91 | 2238.8945 | 20 | -0.8 | 1120.4536 | 2 | 22.65 | 1 | 11172 | BuCaMH04.raw | 4.8615E7 | 2 | 2 | 47 | 66 | Carbamidomethylation | C4:Carbamidomethylation:1000.00;C6:Carbamidomethylation:1000.00 | PEAKS DB |
| Y.GC(+57.02)YC(+57.02)GYGGSGTPVDELDR.C | N | 69.63 | 1961.7883 | 18 | -0.1 | 981.9014 | 2 | 20.01 | 1 | 8793 | BuCaMH04.raw | 4.946E7 | 1 | 1 | 49 | 66 | Carbamidomethylation | C2:Carbamidomethylation:1000.00;C4:Carbamidomethylation:1000.00 | PEAKS DB |
| C.YC(+57.02)GYGGSGTPVDELDR.C | N | 63.69 | 1744.7362 | 16 | 0.1 | 873.3755 | 2 | 19.14 | 1 | 8251 | BuCaMH04.raw | 1.6782E7 | 1 | 1 | 51 | 66 | Carbamidomethylation | C2:Carbamidomethylation:1000.00 | PEAKS DB |
| G.C(+57.02)YC(+57.02)GYGGSGTPVDELDR.C | N | 51.59 | 1904.7668 | 17 | 0.9 | 953.3916 | 2 | 19.93 | 1 | 8813 | BuCaMH04.raw | 3.883E6 | 1 | 1 | 50 | 66 | Carbamidomethylation | C1:Carbamidomethylation:1000.00;C3:Carbamidomethylation:1000.00 | PEAKS DB |
| total 5 peptides |  |  |  |  |  |  |  |  |  |  |  |  |  |  |  |  |  |  |  |
| --- | --- | --- | --- | --- | --- | --- | --- | --- | --- | --- | --- | --- | --- | --- | --- | --- | --- | --- | --- |

JAB52771.1

back to list

  

| Protein Coverage
| Supporting Peptides
|

Protein Coverage:

Supporting Peptides:

| Peptide | Uniq | -10lgP | Mass | Length | ppm | m/z | z | RT | Fraction | Scan | Source File | Area BuCaMH04\_B15 | #Feature | #Feature BuCaMH04\_B15 | Start | End | PTM | AScore | Found By |
| --- | --- | --- | --- | --- | --- | --- | --- | --- | --- | --- | --- | --- | --- | --- | --- | --- | --- | --- | --- |
| N.YGC(+57.02)YC(+57.02)GYGGSGTPVDELDR.C | N | 96.11 | 2124.8516 | 19 | 0.0 | 1063.4331 | 2 | 22.61 | 1 | 11043 | BuCaMH04.raw | 4.7004E9 | 2 | 2 | 50 | 68 | Carbamidomethylation | C3:Carbamidomethylation:1000.00;C5:Carbamidomethylation:1000.00 | PEAKS DB |
| T.NYGC(+57.02)YC(+57.02)GYGGSGTPVDELDR.C | Y | 74.91 | 2238.8945 | 20 | -0.8 | 1120.4536 | 2 | 22.65 | 1 | 11172 | BuCaMH04.raw | 4.8615E7 | 2 | 2 | 49 | 68 | Carbamidomethylation | C4:Carbamidomethylation:1000.00;C6:Carbamidomethylation:1000.00 | PEAKS DB |
| Y.GC(+57.02)YC(+57.02)GYGGSGTPVDELDR.C | N | 69.63 | 1961.7883 | 18 | -0.1 | 981.9014 | 2 | 20.01 | 1 | 8793 | BuCaMH04.raw | 4.946E7 | 1 | 1 | 51 | 68 | Carbamidomethylation | C2:Carbamidomethylation:1000.00;C4:Carbamidomethylation:1000.00 | PEAKS DB |
| C.YC(+57.02)GYGGSGTPVDELDR.C | N | 63.69 | 1744.7362 | 16 | 0.1 | 873.3755 | 2 | 19.14 | 1 | 8251 | BuCaMH04.raw | 1.6782E7 | 1 | 1 | 53 | 68 | Carbamidomethylation | C2:Carbamidomethylation:1000.00 | PEAKS DB |
| G.C(+57.02)YC(+57.02)GYGGSGTPVDELDR.C | N | 51.59 | 1904.7668 | 17 | 0.9 | 953.3916 | 2 | 19.93 | 1 | 8813 | BuCaMH04.raw | 3.883E6 | 1 | 1 | 52 | 68 | Carbamidomethylation | C1:Carbamidomethylation:1000.00;C3:Carbamidomethylation:1000.00 | PEAKS DB |
| total 5 peptides |  |  |  |  |  |  |  |  |  |  |  |  |  |  |  |  |  |  |  |
| --- | --- | --- | --- | --- | --- | --- | --- | --- | --- | --- | --- | --- | --- | --- | --- | --- | --- | --- | --- |

JAS04998.1

back to list

  

| Protein Coverage
| Supporting Peptides
|

Protein Coverage:

Supporting Peptides:

| Peptide | Uniq | -10lgP | Mass | Length | ppm | m/z | z | RT | Fraction | Scan | Source File | Area BuCaMH04\_B15 | #Feature | #Feature BuCaMH04\_B15 | Start | End | PTM | AScore | Found By |
| --- | --- | --- | --- | --- | --- | --- | --- | --- | --- | --- | --- | --- | --- | --- | --- | --- | --- | --- | --- |
| N.YGC(+57.02)YC(+57.02)GYGGSGTPVDELDR.C | N | 96.11 | 2124.8516 | 19 | 0.0 | 1063.4331 | 2 | 22.61 | 1 | 11043 | BuCaMH04.raw | 4.7004E9 | 2 | 2 | 50 | 68 | Carbamidomethylation | C3:Carbamidomethylation:1000.00;C5:Carbamidomethylation:1000.00 | PEAKS DB |
| T.NYGC(+57.02)YC(+57.02)GYGGSGTPVDELDR.C | Y | 74.91 | 2238.8945 | 20 | -0.8 | 1120.4536 | 2 | 22.65 | 1 | 11172 | BuCaMH04.raw | 4.8615E7 | 2 | 2 | 49 | 68 | Carbamidomethylation | C4:Carbamidomethylation:1000.00;C6:Carbamidomethylation:1000.00 | PEAKS DB |
| Y.GC(+57.02)YC(+57.02)GYGGSGTPVDELDR.C | N | 69.63 | 1961.7883 | 18 | -0.1 | 981.9014 | 2 | 20.01 | 1 | 8793 | BuCaMH04.raw | 4.946E7 | 1 | 1 | 51 | 68 | Carbamidomethylation | C2:Carbamidomethylation:1000.00;C4:Carbamidomethylation:1000.00 | PEAKS DB |
| C.YC(+57.02)GYGGSGTPVDELDR.C | N | 63.69 | 1744.7362 | 16 | 0.1 | 873.3755 | 2 | 19.14 | 1 | 8251 | BuCaMH04.raw | 1.6782E7 | 1 | 1 | 53 | 68 | Carbamidomethylation | C2:Carbamidomethylation:1000.00 | PEAKS DB |
| G.C(+57.02)YC(+57.02)GYGGSGTPVDELDR.C | N | 51.59 | 1904.7668 | 17 | 0.9 | 953.3916 | 2 | 19.93 | 1 | 8813 | BuCaMH04.raw | 3.883E6 | 1 | 1 | 52 | 68 | Carbamidomethylation | C1:Carbamidomethylation:1000.00;C3:Carbamidomethylation:1000.00 | PEAKS DB |
| total 5 peptides |  |  |  |  |  |  |  |  |  |  |  |  |  |  |  |  |  |  |  |
| --- | --- | --- | --- | --- | --- | --- | --- | --- | --- | --- | --- | --- | --- | --- | --- | --- | --- | --- | --- |

JAB52773.1

back to list

  

| Protein Coverage
| Supporting Peptides
|

Protein Coverage:

Supporting Peptides:

| Peptide | Uniq | -10lgP | Mass | Length | ppm | m/z | z | RT | Fraction | Scan | Source File | Area BuCaMH04\_B15 | #Feature | #Feature BuCaMH04\_B15 | Start | End | PTM | AScore | Found By |
| --- | --- | --- | --- | --- | --- | --- | --- | --- | --- | --- | --- | --- | --- | --- | --- | --- | --- | --- | --- |
| N.YGC(+57.02)YC(+57.02)GYGGSGTPVDELDR.C | N | 96.11 | 2124.8516 | 19 | 0.0 | 1063.4331 | 2 | 22.61 | 1 | 11043 | BuCaMH04.raw | 4.7004E9 | 2 | 2 | 50 | 68 | Carbamidomethylation | C3:Carbamidomethylation:1000.00;C5:Carbamidomethylation:1000.00 | PEAKS DB |
| T.NYGC(+57.02)YC(+57.02)GYGGSGTPVDELDR.C | Y | 74.91 | 2238.8945 | 20 | -0.8 | 1120.4536 | 2 | 22.65 | 1 | 11172 | BuCaMH04.raw | 4.8615E7 | 2 | 2 | 49 | 68 | Carbamidomethylation | C4:Carbamidomethylation:1000.00;C6:Carbamidomethylation:1000.00 | PEAKS DB |
| Y.GC(+57.02)YC(+57.02)GYGGSGTPVDELDR.C | N | 69.63 | 1961.7883 | 18 | -0.1 | 981.9014 | 2 | 20.01 | 1 | 8793 | BuCaMH04.raw | 4.946E7 | 1 | 1 | 51 | 68 | Carbamidomethylation | C2:Carbamidomethylation:1000.00;C4:Carbamidomethylation:1000.00 | PEAKS DB |
| C.YC(+57.02)GYGGSGTPVDELDR.C | N | 63.69 | 1744.7362 | 16 | 0.1 | 873.3755 | 2 | 19.14 | 1 | 8251 | BuCaMH04.raw | 1.6782E7 | 1 | 1 | 53 | 68 | Carbamidomethylation | C2:Carbamidomethylation:1000.00 | PEAKS DB |
| G.C(+57.02)YC(+57.02)GYGGSGTPVDELDR.C | N | 51.59 | 1904.7668 | 17 | 0.9 | 953.3916 | 2 | 19.93 | 1 | 8813 | BuCaMH04.raw | 3.883E6 | 1 | 1 | 52 | 68 | Carbamidomethylation | C1:Carbamidomethylation:1000.00;C3:Carbamidomethylation:1000.00 | PEAKS DB |
| total 5 peptides |  |  |  |  |  |  |  |  |  |  |  |  |  |  |  |  |  |  |  |
| --- | --- | --- | --- | --- | --- | --- | --- | --- | --- | --- | --- | --- | --- | --- | --- | --- | --- | --- | --- |

JAB52772.1

back to list

  

| Protein Coverage
| Supporting Peptides
|

Protein Coverage:

Supporting Peptides:

| Peptide | Uniq | -10lgP | Mass | Length | ppm | m/z | z | RT | Fraction | Scan | Source File | Area BuCaMH04\_B15 | #Feature | #Feature BuCaMH04\_B15 | Start | End | PTM | AScore | Found By |
| --- | --- | --- | --- | --- | --- | --- | --- | --- | --- | --- | --- | --- | --- | --- | --- | --- | --- | --- | --- |
| N.YGC(+57.02)YC(+57.02)GYGGSGTPVDELDR.C | N | 96.11 | 2124.8516 | 19 | 0.0 | 1063.4331 | 2 | 22.61 | 1 | 11043 | BuCaMH04.raw | 4.7004E9 | 2 | 2 | 50 | 68 | Carbamidomethylation | C3:Carbamidomethylation:1000.00;C5:Carbamidomethylation:1000.00 | PEAKS DB |
| T.NYGC(+57.02)YC(+57.02)GYGGSGTPVDELDR.C | Y | 74.91 | 2238.8945 | 20 | -0.8 | 1120.4536 | 2 | 22.65 | 1 | 11172 | BuCaMH04.raw | 4.8615E7 | 2 | 2 | 49 | 68 | Carbamidomethylation | C4:Carbamidomethylation:1000.00;C6:Carbamidomethylation:1000.00 | PEAKS DB |
| Y.GC(+57.02)YC(+57.02)GYGGSGTPVDELDR.C | N | 69.63 | 1961.7883 | 18 | -0.1 | 981.9014 | 2 | 20.01 | 1 | 8793 | BuCaMH04.raw | 4.946E7 | 1 | 1 | 51 | 68 | Carbamidomethylation | C2:Carbamidomethylation:1000.00;C4:Carbamidomethylation:1000.00 | PEAKS DB |
| C.YC(+57.02)GYGGSGTPVDELDR.C | N | 63.69 | 1744.7362 | 16 | 0.1 | 873.3755 | 2 | 19.14 | 1 | 8251 | BuCaMH04.raw | 1.6782E7 | 1 | 1 | 53 | 68 | Carbamidomethylation | C2:Carbamidomethylation:1000.00 | PEAKS DB |
| G.C(+57.02)YC(+57.02)GYGGSGTPVDELDR.C | N | 51.59 | 1904.7668 | 17 | 0.9 | 953.3916 | 2 | 19.93 | 1 | 8813 | BuCaMH04.raw | 3.883E6 | 1 | 1 | 52 | 68 | Carbamidomethylation | C1:Carbamidomethylation:1000.00;C3:Carbamidomethylation:1000.00 | PEAKS DB |
| total 5 peptides |  |  |  |  |  |  |  |  |  |  |  |  |  |  |  |  |  |  |  |
| --- | --- | --- | --- | --- | --- | --- | --- | --- | --- | --- | --- | --- | --- | --- | --- | --- | --- | --- | --- |

JAS04995.1

back to list

  

| Protein Coverage
| Supporting Peptides
|

Protein Coverage:

Supporting Peptides:

| Peptide | Uniq | -10lgP | Mass | Length | ppm | m/z | z | RT | Fraction | Scan | Source File | Area BuCaMH04\_B15 | #Feature | #Feature BuCaMH04\_B15 | Start | End | PTM | AScore | Found By |
| --- | --- | --- | --- | --- | --- | --- | --- | --- | --- | --- | --- | --- | --- | --- | --- | --- | --- | --- | --- |
| N.YGC(+57.02)YC(+57.02)GYGGSGTPVDELDR.C | N | 96.11 | 2124.8516 | 19 | 0.0 | 1063.4331 | 2 | 22.61 | 1 | 11043 | BuCaMH04.raw | 4.7004E9 | 2 | 2 | 50 | 68 | Carbamidomethylation | C3:Carbamidomethylation:1000.00;C5:Carbamidomethylation:1000.00 | PEAKS DB |
| T.NYGC(+57.02)YC(+57.02)GYGGSGTPVDELDR.C | Y | 74.91 | 2238.8945 | 20 | -0.8 | 1120.4536 | 2 | 22.65 | 1 | 11172 | BuCaMH04.raw | 4.8615E7 | 2 | 2 | 49 | 68 | Carbamidomethylation | C4:Carbamidomethylation:1000.00;C6:Carbamidomethylation:1000.00 | PEAKS DB |
| Y.GC(+57.02)YC(+57.02)GYGGSGTPVDELDR.C | N | 69.63 | 1961.7883 | 18 | -0.1 | 981.9014 | 2 | 20.01 | 1 | 8793 | BuCaMH04.raw | 4.946E7 | 1 | 1 | 51 | 68 | Carbamidomethylation | C2:Carbamidomethylation:1000.00;C4:Carbamidomethylation:1000.00 | PEAKS DB |
| C.YC(+57.02)GYGGSGTPVDELDR.C | N | 63.69 | 1744.7362 | 16 | 0.1 | 873.3755 | 2 | 19.14 | 1 | 8251 | BuCaMH04.raw | 1.6782E7 | 1 | 1 | 53 | 68 | Carbamidomethylation | C2:Carbamidomethylation:1000.00 | PEAKS DB |
| G.C(+57.02)YC(+57.02)GYGGSGTPVDELDR.C | N | 51.59 | 1904.7668 | 17 | 0.9 | 953.3916 | 2 | 19.93 | 1 | 8813 | BuCaMH04.raw | 3.883E6 | 1 | 1 | 52 | 68 | Carbamidomethylation | C1:Carbamidomethylation:1000.00;C3:Carbamidomethylation:1000.00 | PEAKS DB |
| total 5 peptides |  |  |  |  |  |  |  |  |  |  |  |  |  |  |  |  |  |  |  |
| --- | --- | --- | --- | --- | --- | --- | --- | --- | --- | --- | --- | --- | --- | --- | --- | --- | --- | --- | --- |

JAB52770.1

back to list

  

| Protein Coverage
| Supporting Peptides
|

Protein Coverage:

Supporting Peptides:

| Peptide | Uniq | -10lgP | Mass | Length | ppm | m/z | z | RT | Fraction | Scan | Source File | Area BuCaMH04\_B15 | #Feature | #Feature BuCaMH04\_B15 | Start | End | PTM | AScore | Found By |
| --- | --- | --- | --- | --- | --- | --- | --- | --- | --- | --- | --- | --- | --- | --- | --- | --- | --- | --- | --- |
| N.YGC(+57.02)YC(+57.02)GYGGSGTPVDELDR.C | N | 96.11 | 2124.8516 | 19 | 0.0 | 1063.4331 | 2 | 22.61 | 1 | 11043 | BuCaMH04.raw | 4.7004E9 | 2 | 2 | 50 | 68 | Carbamidomethylation | C3:Carbamidomethylation:1000.00;C5:Carbamidomethylation:1000.00 | PEAKS DB |
| T.NYGC(+57.02)YC(+57.02)GYGGSGTPVDELDR.C | Y | 74.91 | 2238.8945 | 20 | -0.8 | 1120.4536 | 2 | 22.65 | 1 | 11172 | BuCaMH04.raw | 4.8615E7 | 2 | 2 | 49 | 68 | Carbamidomethylation | C4:Carbamidomethylation:1000.00;C6:Carbamidomethylation:1000.00 | PEAKS DB |
| Y.GC(+57.02)YC(+57.02)GYGGSGTPVDELDR.C | N | 69.63 | 1961.7883 | 18 | -0.1 | 981.9014 | 2 | 20.01 | 1 | 8793 | BuCaMH04.raw | 4.946E7 | 1 | 1 | 51 | 68 | Carbamidomethylation | C2:Carbamidomethylation:1000.00;C4:Carbamidomethylation:1000.00 | PEAKS DB |
| C.YC(+57.02)GYGGSGTPVDELDR.C | N | 63.69 | 1744.7362 | 16 | 0.1 | 873.3755 | 2 | 19.14 | 1 | 8251 | BuCaMH04.raw | 1.6782E7 | 1 | 1 | 53 | 68 | Carbamidomethylation | C2:Carbamidomethylation:1000.00 | PEAKS DB |
| G.C(+57.02)YC(+57.02)GYGGSGTPVDELDR.C | N | 51.59 | 1904.7668 | 17 | 0.9 | 953.3916 | 2 | 19.93 | 1 | 8813 | BuCaMH04.raw | 3.883E6 | 1 | 1 | 52 | 68 | Carbamidomethylation | C1:Carbamidomethylation:1000.00;C3:Carbamidomethylation:1000.00 | PEAKS DB |
| total 5 peptides |  |  |  |  |  |  |  |  |  |  |  |  |  |  |  |  |  |  |  |
| --- | --- | --- | --- | --- | --- | --- | --- | --- | --- | --- | --- | --- | --- | --- | --- | --- | --- | --- | --- |

JAB52795.1

back to list

  

| Protein Coverage
| Supporting Peptides
|

Protein Coverage:

Supporting Peptides:

| Peptide | Uniq | -10lgP | Mass | Length | ppm | m/z | z | RT | Fraction | Scan | Source File | Area BuCaMH04\_B15 | #Feature | #Feature BuCaMH04\_B15 | Start | End | PTM | AScore | Found By |
| --- | --- | --- | --- | --- | --- | --- | --- | --- | --- | --- | --- | --- | --- | --- | --- | --- | --- | --- | --- |
| N.YGC(+57.02)YC(+57.02)GYGGSGTPVDELDR.C | N | 96.11 | 2124.8516 | 19 | 0.0 | 1063.4331 | 2 | 22.61 | 1 | 11043 | BuCaMH04.raw | 4.7004E9 | 2 | 2 | 50 | 68 | Carbamidomethylation | C3:Carbamidomethylation:1000.00;C5:Carbamidomethylation:1000.00 | PEAKS DB |
| T.NYGC(+57.02)YC(+57.02)GYGGSGTPVDELDR.C | Y | 74.91 | 2238.8945 | 20 | -0.8 | 1120.4536 | 2 | 22.65 | 1 | 11172 | BuCaMH04.raw | 4.8615E7 | 2 | 2 | 49 | 68 | Carbamidomethylation | C4:Carbamidomethylation:1000.00;C6:Carbamidomethylation:1000.00 | PEAKS DB |
| Y.GC(+57.02)YC(+57.02)GYGGSGTPVDELDR.C | N | 69.63 | 1961.7883 | 18 | -0.1 | 981.9014 | 2 | 20.01 | 1 | 8793 | BuCaMH04.raw | 4.946E7 | 1 | 1 | 51 | 68 | Carbamidomethylation | C2:Carbamidomethylation:1000.00;C4:Carbamidomethylation:1000.00 | PEAKS DB |
| C.YC(+57.02)GYGGSGTPVDELDR.C | N | 63.69 | 1744.7362 | 16 | 0.1 | 873.3755 | 2 | 19.14 | 1 | 8251 | BuCaMH04.raw | 1.6782E7 | 1 | 1 | 53 | 68 | Carbamidomethylation | C2:Carbamidomethylation:1000.00 | PEAKS DB |
| G.C(+57.02)YC(+57.02)GYGGSGTPVDELDR.C | N | 51.59 | 1904.7668 | 17 | 0.9 | 953.3916 | 2 | 19.93 | 1 | 8813 | BuCaMH04.raw | 3.883E6 | 1 | 1 | 52 | 68 | Carbamidomethylation | C1:Carbamidomethylation:1000.00;C3:Carbamidomethylation:1000.00 | PEAKS DB |
| total 5 peptides |  |  |  |  |  |  |  |  |  |  |  |  |  |  |  |  |  |  |  |
| --- | --- | --- | --- | --- | --- | --- | --- | --- | --- | --- | --- | --- | --- | --- | --- | --- | --- | --- | --- |

JAB52774.1

back to list

  

| Protein Coverage
| Supporting Peptides
|

Protein Coverage:

Supporting Peptides:

| Peptide | Uniq | -10lgP | Mass | Length | ppm | m/z | z | RT | Fraction | Scan | Source File | Area BuCaMH04\_B15 | #Feature | #Feature BuCaMH04\_B15 | Start | End | PTM | AScore | Found By |
| --- | --- | --- | --- | --- | --- | --- | --- | --- | --- | --- | --- | --- | --- | --- | --- | --- | --- | --- | --- |
| N.YGC(+57.02)YC(+57.02)GYGGSGTPVDELDR.C | N | 96.11 | 2124.8516 | 19 | 0.0 | 1063.4331 | 2 | 22.61 | 1 | 11043 | BuCaMH04.raw | 4.7004E9 | 2 | 2 | 50 | 68 | Carbamidomethylation | C3:Carbamidomethylation:1000.00;C5:Carbamidomethylation:1000.00 | PEAKS DB |
| T.NYGC(+57.02)YC(+57.02)GYGGSGTPVDELDR.C | Y | 74.91 | 2238.8945 | 20 | -0.8 | 1120.4536 | 2 | 22.65 | 1 | 11172 | BuCaMH04.raw | 4.8615E7 | 2 | 2 | 49 | 68 | Carbamidomethylation | C4:Carbamidomethylation:1000.00;C6:Carbamidomethylation:1000.00 | PEAKS DB |
| Y.GC(+57.02)YC(+57.02)GYGGSGTPVDELDR.C | N | 69.63 | 1961.7883 | 18 | -0.1 | 981.9014 | 2 | 20.01 | 1 | 8793 | BuCaMH04.raw | 4.946E7 | 1 | 1 | 51 | 68 | Carbamidomethylation | C2:Carbamidomethylation:1000.00;C4:Carbamidomethylation:1000.00 | PEAKS DB |
| C.YC(+57.02)GYGGSGTPVDELDR.C | N | 63.69 | 1744.7362 | 16 | 0.1 | 873.3755 | 2 | 19.14 | 1 | 8251 | BuCaMH04.raw | 1.6782E7 | 1 | 1 | 53 | 68 | Carbamidomethylation | C2:Carbamidomethylation:1000.00 | PEAKS DB |
| G.C(+57.02)YC(+57.02)GYGGSGTPVDELDR.C | N | 51.59 | 1904.7668 | 17 | 0.9 | 953.3916 | 2 | 19.93 | 1 | 8813 | BuCaMH04.raw | 3.883E6 | 1 | 1 | 52 | 68 | Carbamidomethylation | C1:Carbamidomethylation:1000.00;C3:Carbamidomethylation:1000.00 | PEAKS DB |
| total 5 peptides |  |  |  |  |  |  |  |  |  |  |  |  |  |  |  |  |  |  |  |
| --- | --- | --- | --- | --- | --- | --- | --- | --- | --- | --- | --- | --- | --- | --- | --- | --- | --- | --- | --- |

JAS04999.1

back to list

  

| Protein Coverage
| Supporting Peptides
|

Protein Coverage:

Supporting Peptides:

| Peptide | Uniq | -10lgP | Mass | Length | ppm | m/z | z | RT | Fraction | Scan | Source File | Area BuCaMH04\_B15 | #Feature | #Feature BuCaMH04\_B15 | Start | End | PTM | AScore | Found By |
| --- | --- | --- | --- | --- | --- | --- | --- | --- | --- | --- | --- | --- | --- | --- | --- | --- | --- | --- | --- |
| N.YGC(+57.02)YC(+57.02)GYGGSGTPVDELDR.C | N | 96.11 | 2124.8516 | 19 | 0.0 | 1063.4331 | 2 | 22.61 | 1 | 11043 | BuCaMH04.raw | 4.7004E9 | 2 | 2 | 50 | 68 | Carbamidomethylation | C3:Carbamidomethylation:1000.00;C5:Carbamidomethylation:1000.00 | PEAKS DB |
| T.NYGC(+57.02)YC(+57.02)GYGGSGTPVDELDR.C | Y | 74.91 | 2238.8945 | 20 | -0.8 | 1120.4536 | 2 | 22.65 | 1 | 11172 | BuCaMH04.raw | 4.8615E7 | 2 | 2 | 49 | 68 | Carbamidomethylation | C4:Carbamidomethylation:1000.00;C6:Carbamidomethylation:1000.00 | PEAKS DB |
| Y.GC(+57.02)YC(+57.02)GYGGSGTPVDELDR.C | N | 69.63 | 1961.7883 | 18 | -0.1 | 981.9014 | 2 | 20.01 | 1 | 8793 | BuCaMH04.raw | 4.946E7 | 1 | 1 | 51 | 68 | Carbamidomethylation | C2:Carbamidomethylation:1000.00;C4:Carbamidomethylation:1000.00 | PEAKS DB |
| C.YC(+57.02)GYGGSGTPVDELDR.C | N | 63.69 | 1744.7362 | 16 | 0.1 | 873.3755 | 2 | 19.14 | 1 | 8251 | BuCaMH04.raw | 1.6782E7 | 1 | 1 | 53 | 68 | Carbamidomethylation | C2:Carbamidomethylation:1000.00 | PEAKS DB |
| G.C(+57.02)YC(+57.02)GYGGSGTPVDELDR.C | N | 51.59 | 1904.7668 | 17 | 0.9 | 953.3916 | 2 | 19.93 | 1 | 8813 | BuCaMH04.raw | 3.883E6 | 1 | 1 | 52 | 68 | Carbamidomethylation | C1:Carbamidomethylation:1000.00;C3:Carbamidomethylation:1000.00 | PEAKS DB |
| total 5 peptides |  |  |  |  |  |  |  |  |  |  |  |  |  |  |  |  |  |  |  |
| --- | --- | --- | --- | --- | --- | --- | --- | --- | --- | --- | --- | --- | --- | --- | --- | --- | --- | --- | --- |

JAI09010.1

back to list

  

| Protein Coverage
| Supporting Peptides
|

Protein Coverage:

Supporting Peptides:

| Peptide | Uniq | -10lgP | Mass | Length | ppm | m/z | z | RT | Fraction | Scan | Source File | Area BuCaMH04\_B15 | #Feature | #Feature BuCaMH04\_B15 | Start | End | PTM | AScore | Found By |
| --- | --- | --- | --- | --- | --- | --- | --- | --- | --- | --- | --- | --- | --- | --- | --- | --- | --- | --- | --- |
| N.YGC(+57.02)YC(+57.02)GYGGSGTPVDELDR.C | N | 96.11 | 2124.8516 | 19 | 0.0 | 1063.4331 | 2 | 22.61 | 1 | 11043 | BuCaMH04.raw | 4.7004E9 | 2 | 2 | 50 | 68 | Carbamidomethylation | C3:Carbamidomethylation:1000.00;C5:Carbamidomethylation:1000.00 | PEAKS DB |
| T.NYGC(+57.02)YC(+57.02)GYGGSGTPVDELDR.C | Y | 74.91 | 2238.8945 | 20 | -0.8 | 1120.4536 | 2 | 22.65 | 1 | 11172 | BuCaMH04.raw | 4.8615E7 | 2 | 2 | 49 | 68 | Carbamidomethylation | C4:Carbamidomethylation:1000.00;C6:Carbamidomethylation:1000.00 | PEAKS DB |
| Y.GC(+57.02)YC(+57.02)GYGGSGTPVDELDR.C | N | 69.63 | 1961.7883 | 18 | -0.1 | 981.9014 | 2 | 20.01 | 1 | 8793 | BuCaMH04.raw | 4.946E7 | 1 | 1 | 51 | 68 | Carbamidomethylation | C2:Carbamidomethylation:1000.00;C4:Carbamidomethylation:1000.00 | PEAKS DB |
| C.YC(+57.02)GYGGSGTPVDELDR.C | N | 63.69 | 1744.7362 | 16 | 0.1 | 873.3755 | 2 | 19.14 | 1 | 8251 | BuCaMH04.raw | 1.6782E7 | 1 | 1 | 53 | 68 | Carbamidomethylation | C2:Carbamidomethylation:1000.00 | PEAKS DB |
| G.C(+57.02)YC(+57.02)GYGGSGTPVDELDR.C | N | 51.59 | 1904.7668 | 17 | 0.9 | 953.3916 | 2 | 19.93 | 1 | 8813 | BuCaMH04.raw | 3.883E6 | 1 | 1 | 52 | 68 | Carbamidomethylation | C1:Carbamidomethylation:1000.00;C3:Carbamidomethylation:1000.00 | PEAKS DB |
| total 5 peptides |  |  |  |  |  |  |  |  |  |  |  |  |  |  |  |  |  |  |  |
| --- | --- | --- | --- | --- | --- | --- | --- | --- | --- | --- | --- | --- | --- | --- | --- | --- | --- | --- | --- |

JAI09006.1

back to list

  

| Protein Coverage
| Supporting Peptides
|

Protein Coverage:

Supporting Peptides:

| Peptide | Uniq | -10lgP | Mass | Length | ppm | m/z | z | RT | Fraction | Scan | Source File | Area BuCaMH04\_B15 | #Feature | #Feature BuCaMH04\_B15 | Start | End | PTM | AScore | Found By |
| --- | --- | --- | --- | --- | --- | --- | --- | --- | --- | --- | --- | --- | --- | --- | --- | --- | --- | --- | --- |
| N.YGC(+57.02)YC(+57.02)GYGGSGTPVDELDR.C | N | 96.11 | 2124.8516 | 19 | 0.0 | 1063.4331 | 2 | 22.61 | 1 | 11043 | BuCaMH04.raw | 4.7004E9 | 2 | 2 | 50 | 68 | Carbamidomethylation | C3:Carbamidomethylation:1000.00;C5:Carbamidomethylation:1000.00 | PEAKS DB |
| T.NYGC(+57.02)YC(+57.02)GYGGSGTPVDELDR.C | Y | 74.91 | 2238.8945 | 20 | -0.8 | 1120.4536 | 2 | 22.65 | 1 | 11172 | BuCaMH04.raw | 4.8615E7 | 2 | 2 | 49 | 68 | Carbamidomethylation | C4:Carbamidomethylation:1000.00;C6:Carbamidomethylation:1000.00 | PEAKS DB |
| Y.GC(+57.02)YC(+57.02)GYGGSGTPVDELDR.C | N | 69.63 | 1961.7883 | 18 | -0.1 | 981.9014 | 2 | 20.01 | 1 | 8793 | BuCaMH04.raw | 4.946E7 | 1 | 1 | 51 | 68 | Carbamidomethylation | C2:Carbamidomethylation:1000.00;C4:Carbamidomethylation:1000.00 | PEAKS DB |
| C.YC(+57.02)GYGGSGTPVDELDR.C | N | 63.69 | 1744.7362 | 16 | 0.1 | 873.3755 | 2 | 19.14 | 1 | 8251 | BuCaMH04.raw | 1.6782E7 | 1 | 1 | 53 | 68 | Carbamidomethylation | C2:Carbamidomethylation:1000.00 | PEAKS DB |
| G.C(+57.02)YC(+57.02)GYGGSGTPVDELDR.C | N | 51.59 | 1904.7668 | 17 | 0.9 | 953.3916 | 2 | 19.93 | 1 | 8813 | BuCaMH04.raw | 3.883E6 | 1 | 1 | 52 | 68 | Carbamidomethylation | C1:Carbamidomethylation:1000.00;C3:Carbamidomethylation:1000.00 | PEAKS DB |
| total 5 peptides |  |  |  |  |  |  |  |  |  |  |  |  |  |  |  |  |  |  |  |
| --- | --- | --- | --- | --- | --- | --- | --- | --- | --- | --- | --- | --- | --- | --- | --- | --- | --- | --- | --- |

JAI09004.1

back to list

  

| Protein Coverage
| Supporting Peptides
|

Protein Coverage:

Supporting Peptides:

| Peptide | Uniq | -10lgP | Mass | Length | ppm | m/z | z | RT | Fraction | Scan | Source File | Area BuCaMH04\_B15 | #Feature | #Feature BuCaMH04\_B15 | Start | End | PTM | AScore | Found By |
| --- | --- | --- | --- | --- | --- | --- | --- | --- | --- | --- | --- | --- | --- | --- | --- | --- | --- | --- | --- |
| N.YGC(+57.02)YC(+57.02)GYGGSGTPVDELDR.C | N | 96.11 | 2124.8516 | 19 | 0.0 | 1063.4331 | 2 | 22.61 | 1 | 11043 | BuCaMH04.raw | 4.7004E9 | 2 | 2 | 50 | 68 | Carbamidomethylation | C3:Carbamidomethylation:1000.00;C5:Carbamidomethylation:1000.00 | PEAKS DB |
| T.NYGC(+57.02)YC(+57.02)GYGGSGTPVDELDR.C | Y | 74.91 | 2238.8945 | 20 | -0.8 | 1120.4536 | 2 | 22.65 | 1 | 11172 | BuCaMH04.raw | 4.8615E7 | 2 | 2 | 49 | 68 | Carbamidomethylation | C4:Carbamidomethylation:1000.00;C6:Carbamidomethylation:1000.00 | PEAKS DB |
| Y.GC(+57.02)YC(+57.02)GYGGSGTPVDELDR.C | N | 69.63 | 1961.7883 | 18 | -0.1 | 981.9014 | 2 | 20.01 | 1 | 8793 | BuCaMH04.raw | 4.946E7 | 1 | 1 | 51 | 68 | Carbamidomethylation | C2:Carbamidomethylation:1000.00;C4:Carbamidomethylation:1000.00 | PEAKS DB |
| C.YC(+57.02)GYGGSGTPVDELDR.C | N | 63.69 | 1744.7362 | 16 | 0.1 | 873.3755 | 2 | 19.14 | 1 | 8251 | BuCaMH04.raw | 1.6782E7 | 1 | 1 | 53 | 68 | Carbamidomethylation | C2:Carbamidomethylation:1000.00 | PEAKS DB |
| G.C(+57.02)YC(+57.02)GYGGSGTPVDELDR.C | N | 51.59 | 1904.7668 | 17 | 0.9 | 953.3916 | 2 | 19.93 | 1 | 8813 | BuCaMH04.raw | 3.883E6 | 1 | 1 | 52 | 68 | Carbamidomethylation | C1:Carbamidomethylation:1000.00;C3:Carbamidomethylation:1000.00 | PEAKS DB |
| total 5 peptides |  |  |  |  |  |  |  |  |  |  |  |  |  |  |  |  |  |  |  |
| --- | --- | --- | --- | --- | --- | --- | --- | --- | --- | --- | --- | --- | --- | --- | --- | --- | --- | --- | --- |

JAI09005.1

back to list

  

| Protein Coverage
| Supporting Peptides
|

Protein Coverage:

Supporting Peptides:

| Peptide | Uniq | -10lgP | Mass | Length | ppm | m/z | z | RT | Fraction | Scan | Source File | Area BuCaMH04\_B15 | #Feature | #Feature BuCaMH04\_B15 | Start | End | PTM | AScore | Found By |
| --- | --- | --- | --- | --- | --- | --- | --- | --- | --- | --- | --- | --- | --- | --- | --- | --- | --- | --- | --- |
| N.YGC(+57.02)YC(+57.02)GYGGSGTPVDELDR.C | N | 96.11 | 2124.8516 | 19 | 0.0 | 1063.4331 | 2 | 22.61 | 1 | 11043 | BuCaMH04.raw | 4.7004E9 | 2 | 2 | 50 | 68 | Carbamidomethylation | C3:Carbamidomethylation:1000.00;C5:Carbamidomethylation:1000.00 | PEAKS DB |
| T.NYGC(+57.02)YC(+57.02)GYGGSGTPVDELDR.C | Y | 74.91 | 2238.8945 | 20 | -0.8 | 1120.4536 | 2 | 22.65 | 1 | 11172 | BuCaMH04.raw | 4.8615E7 | 2 | 2 | 49 | 68 | Carbamidomethylation | C4:Carbamidomethylation:1000.00;C6:Carbamidomethylation:1000.00 | PEAKS DB |
| Y.GC(+57.02)YC(+57.02)GYGGSGTPVDELDR.C | N | 69.63 | 1961.7883 | 18 | -0.1 | 981.9014 | 2 | 20.01 | 1 | 8793 | BuCaMH04.raw | 4.946E7 | 1 | 1 | 51 | 68 | Carbamidomethylation | C2:Carbamidomethylation:1000.00;C4:Carbamidomethylation:1000.00 | PEAKS DB |
| C.YC(+57.02)GYGGSGTPVDELDR.C | N | 63.69 | 1744.7362 | 16 | 0.1 | 873.3755 | 2 | 19.14 | 1 | 8251 | BuCaMH04.raw | 1.6782E7 | 1 | 1 | 53 | 68 | Carbamidomethylation | C2:Carbamidomethylation:1000.00 | PEAKS DB |
| G.C(+57.02)YC(+57.02)GYGGSGTPVDELDR.C | N | 51.59 | 1904.7668 | 17 | 0.9 | 953.3916 | 2 | 19.93 | 1 | 8813 | BuCaMH04.raw | 3.883E6 | 1 | 1 | 52 | 68 | Carbamidomethylation | C1:Carbamidomethylation:1000.00;C3:Carbamidomethylation:1000.00 | PEAKS DB |
| total 5 peptides |  |  |  |  |  |  |  |  |  |  |  |  |  |  |  |  |  |  |  |
| --- | --- | --- | --- | --- | --- | --- | --- | --- | --- | --- | --- | --- | --- | --- | --- | --- | --- | --- | --- |

JAI09009.1

back to list

  

| Protein Coverage
| Supporting Peptides
|

Protein Coverage:

Supporting Peptides:

| Peptide | Uniq | -10lgP | Mass | Length | ppm | m/z | z | RT | Fraction | Scan | Source File | Area BuCaMH04\_B15 | #Feature | #Feature BuCaMH04\_B15 | Start | End | PTM | AScore | Found By |
| --- | --- | --- | --- | --- | --- | --- | --- | --- | --- | --- | --- | --- | --- | --- | --- | --- | --- | --- | --- |
| N.YGC(+57.02)YC(+57.02)GYGGSGTPVDELDR.C | N | 96.11 | 2124.8516 | 19 | 0.0 | 1063.4331 | 2 | 22.61 | 1 | 11043 | BuCaMH04.raw | 4.7004E9 | 2 | 2 | 50 | 68 | Carbamidomethylation | C3:Carbamidomethylation:1000.00;C5:Carbamidomethylation:1000.00 | PEAKS DB |
| T.NYGC(+57.02)YC(+57.02)GYGGSGTPVDELDR.C | Y | 74.91 | 2238.8945 | 20 | -0.8 | 1120.4536 | 2 | 22.65 | 1 | 11172 | BuCaMH04.raw | 4.8615E7 | 2 | 2 | 49 | 68 | Carbamidomethylation | C4:Carbamidomethylation:1000.00;C6:Carbamidomethylation:1000.00 | PEAKS DB |
| Y.GC(+57.02)YC(+57.02)GYGGSGTPVDELDR.C | N | 69.63 | 1961.7883 | 18 | -0.1 | 981.9014 | 2 | 20.01 | 1 | 8793 | BuCaMH04.raw | 4.946E7 | 1 | 1 | 51 | 68 | Carbamidomethylation | C2:Carbamidomethylation:1000.00;C4:Carbamidomethylation:1000.00 | PEAKS DB |
| C.YC(+57.02)GYGGSGTPVDELDR.C | N | 63.69 | 1744.7362 | 16 | 0.1 | 873.3755 | 2 | 19.14 | 1 | 8251 | BuCaMH04.raw | 1.6782E7 | 1 | 1 | 53 | 68 | Carbamidomethylation | C2:Carbamidomethylation:1000.00 | PEAKS DB |
| G.C(+57.02)YC(+57.02)GYGGSGTPVDELDR.C | N | 51.59 | 1904.7668 | 17 | 0.9 | 953.3916 | 2 | 19.93 | 1 | 8813 | BuCaMH04.raw | 3.883E6 | 1 | 1 | 52 | 68 | Carbamidomethylation | C1:Carbamidomethylation:1000.00;C3:Carbamidomethylation:1000.00 | PEAKS DB |
| total 5 peptides |  |  |  |  |  |  |  |  |  |  |  |  |  |  |  |  |  |  |  |
| --- | --- | --- | --- | --- | --- | --- | --- | --- | --- | --- | --- | --- | --- | --- | --- | --- | --- | --- | --- |

JAS04993.1

back to list

  

| Protein Coverage
| Supporting Peptides
|

Protein Coverage:

Supporting Peptides:

| Peptide | Uniq | -10lgP | Mass | Length | ppm | m/z | z | RT | Fraction | Scan | Source File | Area BuCaMH04\_B15 | #Feature | #Feature BuCaMH04\_B15 | Start | End | PTM | AScore | Found By |
| --- | --- | --- | --- | --- | --- | --- | --- | --- | --- | --- | --- | --- | --- | --- | --- | --- | --- | --- | --- |
| N.YGC(+57.02)YC(+57.02)GYGGSGTPVDELDR.C | N | 96.11 | 2124.8516 | 19 | 0.0 | 1063.4331 | 2 | 22.61 | 1 | 11043 | BuCaMH04.raw | 4.7004E9 | 2 | 2 | 50 | 68 | Carbamidomethylation | C3:Carbamidomethylation:1000.00;C5:Carbamidomethylation:1000.00 | PEAKS DB |
| T.NYGC(+57.02)YC(+57.02)GYGGSGTPVDELDR.C | Y | 74.91 | 2238.8945 | 20 | -0.8 | 1120.4536 | 2 | 22.65 | 1 | 11172 | BuCaMH04.raw | 4.8615E7 | 2 | 2 | 49 | 68 | Carbamidomethylation | C4:Carbamidomethylation:1000.00;C6:Carbamidomethylation:1000.00 | PEAKS DB |
| Y.GC(+57.02)YC(+57.02)GYGGSGTPVDELDR.C | N | 69.63 | 1961.7883 | 18 | -0.1 | 981.9014 | 2 | 20.01 | 1 | 8793 | BuCaMH04.raw | 4.946E7 | 1 | 1 | 51 | 68 | Carbamidomethylation | C2:Carbamidomethylation:1000.00;C4:Carbamidomethylation:1000.00 | PEAKS DB |
| C.YC(+57.02)GYGGSGTPVDELDR.C | N | 63.69 | 1744.7362 | 16 | 0.1 | 873.3755 | 2 | 19.14 | 1 | 8251 | BuCaMH04.raw | 1.6782E7 | 1 | 1 | 53 | 68 | Carbamidomethylation | C2:Carbamidomethylation:1000.00 | PEAKS DB |
| G.C(+57.02)YC(+57.02)GYGGSGTPVDELDR.C | N | 51.59 | 1904.7668 | 17 | 0.9 | 953.3916 | 2 | 19.93 | 1 | 8813 | BuCaMH04.raw | 3.883E6 | 1 | 1 | 52 | 68 | Carbamidomethylation | C1:Carbamidomethylation:1000.00;C3:Carbamidomethylation:1000.00 | PEAKS DB |
| total 5 peptides |  |  |  |  |  |  |  |  |  |  |  |  |  |  |  |  |  |  |  |
| --- | --- | --- | --- | --- | --- | --- | --- | --- | --- | --- | --- | --- | --- | --- | --- | --- | --- | --- | --- |

JAB52804.1

back to list

  

| Protein Coverage
| Supporting Peptides
|

Protein Coverage:

Supporting Peptides:

| Peptide | Uniq | -10lgP | Mass | Length | ppm | m/z | z | RT | Fraction | Scan | Source File | Area BuCaMH04\_B15 | #Feature | #Feature BuCaMH04\_B15 | Start | End | PTM | AScore | Found By |
| --- | --- | --- | --- | --- | --- | --- | --- | --- | --- | --- | --- | --- | --- | --- | --- | --- | --- | --- | --- |
| N.YGC(+57.02)YC(+57.02)GYGGSGTPVDELDR.C | N | 96.11 | 2124.8516 | 19 | 0.0 | 1063.4331 | 2 | 22.61 | 1 | 11043 | BuCaMH04.raw | 4.7004E9 | 2 | 2 | 50 | 68 | Carbamidomethylation | C3:Carbamidomethylation:1000.00;C5:Carbamidomethylation:1000.00 | PEAKS DB |
| T.NYGC(+57.02)YC(+57.02)GYGGSGTPVDELDR.C | Y | 74.91 | 2238.8945 | 20 | -0.8 | 1120.4536 | 2 | 22.65 | 1 | 11172 | BuCaMH04.raw | 4.8615E7 | 2 | 2 | 49 | 68 | Carbamidomethylation | C4:Carbamidomethylation:1000.00;C6:Carbamidomethylation:1000.00 | PEAKS DB |
| Y.GC(+57.02)YC(+57.02)GYGGSGTPVDELDR.C | N | 69.63 | 1961.7883 | 18 | -0.1 | 981.9014 | 2 | 20.01 | 1 | 8793 | BuCaMH04.raw | 4.946E7 | 1 | 1 | 51 | 68 | Carbamidomethylation | C2:Carbamidomethylation:1000.00;C4:Carbamidomethylation:1000.00 | PEAKS DB |
| C.YC(+57.02)GYGGSGTPVDELDR.C | N | 63.69 | 1744.7362 | 16 | 0.1 | 873.3755 | 2 | 19.14 | 1 | 8251 | BuCaMH04.raw | 1.6782E7 | 1 | 1 | 53 | 68 | Carbamidomethylation | C2:Carbamidomethylation:1000.00 | PEAKS DB |
| G.C(+57.02)YC(+57.02)GYGGSGTPVDELDR.C | N | 51.59 | 1904.7668 | 17 | 0.9 | 953.3916 | 2 | 19.93 | 1 | 8813 | BuCaMH04.raw | 3.883E6 | 1 | 1 | 52 | 68 | Carbamidomethylation | C1:Carbamidomethylation:1000.00;C3:Carbamidomethylation:1000.00 | PEAKS DB |
| total 5 peptides |  |  |  |  |  |  |  |  |  |  |  |  |  |  |  |  |  |  |  |
| --- | --- | --- | --- | --- | --- | --- | --- | --- | --- | --- | --- | --- | --- | --- | --- | --- | --- | --- | --- |

JAS04994.1

back to list

  

| Protein Coverage
| Supporting Peptides
|

Protein Coverage:

Supporting Peptides:

| Peptide | Uniq | -10lgP | Mass | Length | ppm | m/z | z | RT | Fraction | Scan | Source File | Area BuCaMH04\_B15 | #Feature | #Feature BuCaMH04\_B15 | Start | End | PTM | AScore | Found By |
| --- | --- | --- | --- | --- | --- | --- | --- | --- | --- | --- | --- | --- | --- | --- | --- | --- | --- | --- | --- |
| N.YGC(+57.02)YC(+57.02)GYGGSGTPVDELDR.C | N | 96.11 | 2124.8516 | 19 | 0.0 | 1063.4331 | 2 | 22.61 | 1 | 11043 | BuCaMH04.raw | 4.7004E9 | 2 | 2 | 50 | 68 | Carbamidomethylation | C3:Carbamidomethylation:1000.00;C5:Carbamidomethylation:1000.00 | PEAKS DB |
| T.NYGC(+57.02)YC(+57.02)GYGGSGTPVDELDR.C | Y | 74.91 | 2238.8945 | 20 | -0.8 | 1120.4536 | 2 | 22.65 | 1 | 11172 | BuCaMH04.raw | 4.8615E7 | 2 | 2 | 49 | 68 | Carbamidomethylation | C4:Carbamidomethylation:1000.00;C6:Carbamidomethylation:1000.00 | PEAKS DB |
| Y.GC(+57.02)YC(+57.02)GYGGSGTPVDELDR.C | N | 69.63 | 1961.7883 | 18 | -0.1 | 981.9014 | 2 | 20.01 | 1 | 8793 | BuCaMH04.raw | 4.946E7 | 1 | 1 | 51 | 68 | Carbamidomethylation | C2:Carbamidomethylation:1000.00;C4:Carbamidomethylation:1000.00 | PEAKS DB |
| C.YC(+57.02)GYGGSGTPVDELDR.C | N | 63.69 | 1744.7362 | 16 | 0.1 | 873.3755 | 2 | 19.14 | 1 | 8251 | BuCaMH04.raw | 1.6782E7 | 1 | 1 | 53 | 68 | Carbamidomethylation | C2:Carbamidomethylation:1000.00 | PEAKS DB |
| G.C(+57.02)YC(+57.02)GYGGSGTPVDELDR.C | N | 51.59 | 1904.7668 | 17 | 0.9 | 953.3916 | 2 | 19.93 | 1 | 8813 | BuCaMH04.raw | 3.883E6 | 1 | 1 | 52 | 68 | Carbamidomethylation | C1:Carbamidomethylation:1000.00;C3:Carbamidomethylation:1000.00 | PEAKS DB |
| total 5 peptides |  |  |  |  |  |  |  |  |  |  |  |  |  |  |  |  |  |  |  |
| --- | --- | --- | --- | --- | --- | --- | --- | --- | --- | --- | --- | --- | --- | --- | --- | --- | --- | --- | --- |

JAB52764.1

back to list

  

| Protein Coverage
| Supporting Peptides
|

Protein Coverage:

Supporting Peptides:

| Peptide | Uniq | -10lgP | Mass | Length | ppm | m/z | z | RT | Fraction | Scan | Source File | Area BuCaMH04\_B15 | #Feature | #Feature BuCaMH04\_B15 | Start | End | PTM | AScore | Found By |
| --- | --- | --- | --- | --- | --- | --- | --- | --- | --- | --- | --- | --- | --- | --- | --- | --- | --- | --- | --- |
| N.YGC(+57.02)YC(+57.02)GYGGSGTPVDELDR.C | N | 96.11 | 2124.8516 | 19 | 0.0 | 1063.4331 | 2 | 22.61 | 1 | 11043 | BuCaMH04.raw | 4.7004E9 | 2 | 2 | 50 | 68 | Carbamidomethylation | C3:Carbamidomethylation:1000.00;C5:Carbamidomethylation:1000.00 | PEAKS DB |
| T.NYGC(+57.02)YC(+57.02)GYGGSGTPVDELDR.C | Y | 74.91 | 2238.8945 | 20 | -0.8 | 1120.4536 | 2 | 22.65 | 1 | 11172 | BuCaMH04.raw | 4.8615E7 | 2 | 2 | 49 | 68 | Carbamidomethylation | C4:Carbamidomethylation:1000.00;C6:Carbamidomethylation:1000.00 | PEAKS DB |
| Y.GC(+57.02)YC(+57.02)GYGGSGTPVDELDR.C | N | 69.63 | 1961.7883 | 18 | -0.1 | 981.9014 | 2 | 20.01 | 1 | 8793 | BuCaMH04.raw | 4.946E7 | 1 | 1 | 51 | 68 | Carbamidomethylation | C2:Carbamidomethylation:1000.00;C4:Carbamidomethylation:1000.00 | PEAKS DB |
| C.YC(+57.02)GYGGSGTPVDELDR.C | N | 63.69 | 1744.7362 | 16 | 0.1 | 873.3755 | 2 | 19.14 | 1 | 8251 | BuCaMH04.raw | 1.6782E7 | 1 | 1 | 53 | 68 | Carbamidomethylation | C2:Carbamidomethylation:1000.00 | PEAKS DB |
| G.C(+57.02)YC(+57.02)GYGGSGTPVDELDR.C | N | 51.59 | 1904.7668 | 17 | 0.9 | 953.3916 | 2 | 19.93 | 1 | 8813 | BuCaMH04.raw | 3.883E6 | 1 | 1 | 52 | 68 | Carbamidomethylation | C1:Carbamidomethylation:1000.00;C3:Carbamidomethylation:1000.00 | PEAKS DB |
| total 5 peptides |  |  |  |  |  |  |  |  |  |  |  |  |  |  |  |  |  |  |  |
| --- | --- | --- | --- | --- | --- | --- | --- | --- | --- | --- | --- | --- | --- | --- | --- | --- | --- | --- | --- |

LAA98189.1

back to list

  

| Protein Coverage
| Supporting Peptides
|

Protein Coverage:

Supporting Peptides:

| Peptide | Uniq | -10lgP | Mass | Length | ppm | m/z | z | RT | Fraction | Scan | Source File | Area BuCaMH04\_B15 | #Feature | #Feature BuCaMH04\_B15 | Start | End | PTM | AScore | Found By |
| --- | --- | --- | --- | --- | --- | --- | --- | --- | --- | --- | --- | --- | --- | --- | --- | --- | --- | --- | --- |
| N.YGC(+57.02)YC(+57.02)GYGGSGTPVDELDR.C | N | 96.11 | 2124.8516 | 19 | 0.0 | 1063.4331 | 2 | 22.61 | 1 | 11043 | BuCaMH04.raw | 4.7004E9 | 2 | 2 | 64 | 82 | Carbamidomethylation | C3:Carbamidomethylation:1000.00;C5:Carbamidomethylation:1000.00 | PEAKS DB |
| R.NYGC(+57.02)YC(+57.02)GYGGSGTPVDELDR.C | Y | 74.91 | 2238.8945 | 20 | -0.8 | 1120.4536 | 2 | 22.65 | 1 | 11172 | BuCaMH04.raw | 4.8615E7 | 2 | 2 | 63 | 82 | Carbamidomethylation | C4:Carbamidomethylation:1000.00;C6:Carbamidomethylation:1000.00 | PEAKS DB |
| Y.GC(+57.02)YC(+57.02)GYGGSGTPVDELDR.C | N | 69.63 | 1961.7883 | 18 | -0.1 | 981.9014 | 2 | 20.01 | 1 | 8793 | BuCaMH04.raw | 4.946E7 | 1 | 1 | 65 | 82 | Carbamidomethylation | C2:Carbamidomethylation:1000.00;C4:Carbamidomethylation:1000.00 | PEAKS DB |
| C.YC(+57.02)GYGGSGTPVDELDR.C | N | 63.69 | 1744.7362 | 16 | 0.1 | 873.3755 | 2 | 19.14 | 1 | 8251 | BuCaMH04.raw | 1.6782E7 | 1 | 1 | 67 | 82 | Carbamidomethylation | C2:Carbamidomethylation:1000.00 | PEAKS DB |
| G.C(+57.02)YC(+57.02)GYGGSGTPVDELDR.C | N | 51.59 | 1904.7668 | 17 | 0.9 | 953.3916 | 2 | 19.93 | 1 | 8813 | BuCaMH04.raw | 3.883E6 | 1 | 1 | 66 | 82 | Carbamidomethylation | C1:Carbamidomethylation:1000.00;C3:Carbamidomethylation:1000.00 | PEAKS DB |
| total 5 peptides |  |  |  |  |  |  |  |  |  |  |  |  |  |  |  |  |  |  |  |
| --- | --- | --- | --- | --- | --- | --- | --- | --- | --- | --- | --- | --- | --- | --- | --- | --- | --- | --- | --- |

JAS04997.1

back to list

  

| Protein Coverage
| Supporting Peptides
|

Protein Coverage:

Supporting Peptides:

| Peptide | Uniq | -10lgP | Mass | Length | ppm | m/z | z | RT | Fraction | Scan | Source File | Area BuCaMH04\_B15 | #Feature | #Feature BuCaMH04\_B15 | Start | End | PTM | AScore | Found By |
| --- | --- | --- | --- | --- | --- | --- | --- | --- | --- | --- | --- | --- | --- | --- | --- | --- | --- | --- | --- |
| N.YGC(+57.02)YC(+57.02)GYGGSGTPVDELDR.C | N | 96.11 | 2124.8516 | 19 | 0.0 | 1063.4331 | 2 | 22.61 | 1 | 11043 | BuCaMH04.raw | 4.7004E9 | 2 | 2 | 50 | 68 | Carbamidomethylation | C3:Carbamidomethylation:1000.00;C5:Carbamidomethylation:1000.00 | PEAKS DB |
| T.NYGC(+57.02)YC(+57.02)GYGGSGTPVDELDR.C | Y | 74.91 | 2238.8945 | 20 | -0.8 | 1120.4536 | 2 | 22.65 | 1 | 11172 | BuCaMH04.raw | 4.8615E7 | 2 | 2 | 49 | 68 | Carbamidomethylation | C4:Carbamidomethylation:1000.00;C6:Carbamidomethylation:1000.00 | PEAKS DB |
| Y.GC(+57.02)YC(+57.02)GYGGSGTPVDELDR.C | N | 69.63 | 1961.7883 | 18 | -0.1 | 981.9014 | 2 | 20.01 | 1 | 8793 | BuCaMH04.raw | 4.946E7 | 1 | 1 | 51 | 68 | Carbamidomethylation | C2:Carbamidomethylation:1000.00;C4:Carbamidomethylation:1000.00 | PEAKS DB |
| C.YC(+57.02)GYGGSGTPVDELDR.C | N | 63.69 | 1744.7362 | 16 | 0.1 | 873.3755 | 2 | 19.14 | 1 | 8251 | BuCaMH04.raw | 1.6782E7 | 1 | 1 | 53 | 68 | Carbamidomethylation | C2:Carbamidomethylation:1000.00 | PEAKS DB |
| G.C(+57.02)YC(+57.02)GYGGSGTPVDELDR.C | N | 51.59 | 1904.7668 | 17 | 0.9 | 953.3916 | 2 | 19.93 | 1 | 8813 | BuCaMH04.raw | 3.883E6 | 1 | 1 | 52 | 68 | Carbamidomethylation | C1:Carbamidomethylation:1000.00;C3:Carbamidomethylation:1000.00 | PEAKS DB |
| total 5 peptides |  |  |  |  |  |  |  |  |  |  |  |  |  |  |  |  |  |  |  |
| --- | --- | --- | --- | --- | --- | --- | --- | --- | --- | --- | --- | --- | --- | --- | --- | --- | --- | --- | --- |

JAI09008.1

back to list

  

| Protein Coverage
| Supporting Peptides
|

Protein Coverage:

Supporting Peptides:

| Peptide | Uniq | -10lgP | Mass | Length | ppm | m/z | z | RT | Fraction | Scan | Source File | Area BuCaMH04\_B15 | #Feature | #Feature BuCaMH04\_B15 | Start | End | PTM | AScore | Found By |
| --- | --- | --- | --- | --- | --- | --- | --- | --- | --- | --- | --- | --- | --- | --- | --- | --- | --- | --- | --- |
| N.YGC(+57.02)YC(+57.02)GYGGSGTPVDELDR.C | N | 96.11 | 2124.8516 | 19 | 0.0 | 1063.4331 | 2 | 22.61 | 1 | 11043 | BuCaMH04.raw | 4.7004E9 | 2 | 2 | 50 | 68 | Carbamidomethylation | C3:Carbamidomethylation:1000.00;C5:Carbamidomethylation:1000.00 | PEAKS DB |
| T.NYGC(+57.02)YC(+57.02)GYGGSGTPVDELDR.C | Y | 74.91 | 2238.8945 | 20 | -0.8 | 1120.4536 | 2 | 22.65 | 1 | 11172 | BuCaMH04.raw | 4.8615E7 | 2 | 2 | 49 | 68 | Carbamidomethylation | C4:Carbamidomethylation:1000.00;C6:Carbamidomethylation:1000.00 | PEAKS DB |
| Y.GC(+57.02)YC(+57.02)GYGGSGTPVDELDR.C | N | 69.63 | 1961.7883 | 18 | -0.1 | 981.9014 | 2 | 20.01 | 1 | 8793 | BuCaMH04.raw | 4.946E7 | 1 | 1 | 51 | 68 | Carbamidomethylation | C2:Carbamidomethylation:1000.00;C4:Carbamidomethylation:1000.00 | PEAKS DB |
| C.YC(+57.02)GYGGSGTPVDELDR.C | N | 63.69 | 1744.7362 | 16 | 0.1 | 873.3755 | 2 | 19.14 | 1 | 8251 | BuCaMH04.raw | 1.6782E7 | 1 | 1 | 53 | 68 | Carbamidomethylation | C2:Carbamidomethylation:1000.00 | PEAKS DB |
| G.C(+57.02)YC(+57.02)GYGGSGTPVDELDR.C | N | 51.59 | 1904.7668 | 17 | 0.9 | 953.3916 | 2 | 19.93 | 1 | 8813 | BuCaMH04.raw | 3.883E6 | 1 | 1 | 52 | 68 | Carbamidomethylation | C1:Carbamidomethylation:1000.00;C3:Carbamidomethylation:1000.00 | PEAKS DB |
| total 5 peptides |  |  |  |  |  |  |  |  |  |  |  |  |  |  |  |  |  |  |  |
| --- | --- | --- | --- | --- | --- | --- | --- | --- | --- | --- | --- | --- | --- | --- | --- | --- | --- | --- | --- |

ABU68503.1

back to list

  

| Protein Coverage
| Supporting Peptides
|

Protein Coverage:

Supporting Peptides:

| Peptide | Uniq | -10lgP | Mass | Length | ppm | m/z | z | RT | Fraction | Scan | Source File | Area BuCaMH04\_B15 | #Feature | #Feature BuCaMH04\_B15 | Start | End | PTM | AScore | Found By |
| --- | --- | --- | --- | --- | --- | --- | --- | --- | --- | --- | --- | --- | --- | --- | --- | --- | --- | --- | --- |
| N.YGC(+57.02)YC(+57.02)GYGGSGTPVDELDR.C | N | 96.11 | 2124.8516 | 19 | 0.0 | 1063.4331 | 2 | 22.61 | 1 | 11043 | BuCaMH04.raw | 4.7004E9 | 2 | 2 | 52 | 70 | Carbamidomethylation | C3:Carbamidomethylation:1000.00;C5:Carbamidomethylation:1000.00 | PEAKS DB |
| G.NYGC(+57.02)YC(+57.02)GYGGSGTPVDELDR.C | Y | 74.91 | 2238.8945 | 20 | -0.8 | 1120.4536 | 2 | 22.65 | 1 | 11172 | BuCaMH04.raw | 4.8615E7 | 2 | 2 | 51 | 70 | Carbamidomethylation | C4:Carbamidomethylation:1000.00;C6:Carbamidomethylation:1000.00 | PEAKS DB |
| Y.GC(+57.02)YC(+57.02)GYGGSGTPVDELDR.C | N | 69.63 | 1961.7883 | 18 | -0.1 | 981.9014 | 2 | 20.01 | 1 | 8793 | BuCaMH04.raw | 4.946E7 | 1 | 1 | 53 | 70 | Carbamidomethylation | C2:Carbamidomethylation:1000.00;C4:Carbamidomethylation:1000.00 | PEAKS DB |
| C.YC(+57.02)GYGGSGTPVDELDR.C | N | 63.69 | 1744.7362 | 16 | 0.1 | 873.3755 | 2 | 19.14 | 1 | 8251 | BuCaMH04.raw | 1.6782E7 | 1 | 1 | 55 | 70 | Carbamidomethylation | C2:Carbamidomethylation:1000.00 | PEAKS DB |
| G.C(+57.02)YC(+57.02)GYGGSGTPVDELDR.C | N | 51.59 | 1904.7668 | 17 | 0.9 | 953.3916 | 2 | 19.93 | 1 | 8813 | BuCaMH04.raw | 3.883E6 | 1 | 1 | 54 | 70 | Carbamidomethylation | C1:Carbamidomethylation:1000.00;C3:Carbamidomethylation:1000.00 | PEAKS DB |
| total 5 peptides |  |  |  |  |  |  |  |  |  |  |  |  |  |  |  |  |  |  |  |
| --- | --- | --- | --- | --- | --- | --- | --- | --- | --- | --- | --- | --- | --- | --- | --- | --- | --- | --- | --- |

AXL96665.1

back to list

  

| Protein Coverage
| Supporting Peptides
|

Protein Coverage:

Supporting Peptides:

| Peptide | Uniq | -10lgP | Mass | Length | ppm | m/z | z | RT | Fraction | Scan | Source File | Area BuCaMH04\_B15 | #Feature | #Feature BuCaMH04\_B15 | Start | End | PTM | AScore | Found By |
| --- | --- | --- | --- | --- | --- | --- | --- | --- | --- | --- | --- | --- | --- | --- | --- | --- | --- | --- | --- |
| N.YFVEVGEEC(+57.02)DC(+57.02)GSPR.N | N | 82.16 | 1802.7240 | 15 | 0.5 | 902.3698 | 2 | 18.36 | 1 | 7501 | BuCaMH04.raw | 1.0067E8 | 1 | 1 | 414 | 428 | Carbamidomethylation | C9:Carbamidomethylation:1000.00;C11:Carbamidomethylation:1000.00 | PEAKS DB |
| N.VTLDLFGEWR.E | N | 73.86 | 1234.6346 | 10 | 0.6 | 618.3250 | 2 | 45.55 | 1 | 31857 | BuCaMH04.raw | 9.3115E6 | 1 | 1 | 271 | 280 |  |  | PEAKS DB |
| V.TLDLFGEWR.E | N | 65.70 | 1135.5662 | 9 | 0.5 | 568.7906 | 2 | 42.31 | 1 | 29257 | BuCaMH04.raw | 4.2198E8 | 1 | 1 | 272 | 280 |  |  | PEAKS DB |
| T.LDLFGEWR.E | N | 57.32 | 1034.5186 | 8 | 0.6 | 518.2668 | 2 | 41.26 | 1 | 27863 | BuCaMH04.raw | 4.6562E7 | 1 | 1 | 273 | 280 |  |  | PEAKS DB |
| L.IGLEIWSNK.D | N | 50.68 | 1058.5760 | 9 | 0.6 | 530.2956 | 2 | 27.50 | 1 | 15397 | BuCaMH04.raw | 8.2315E6 | 1 | 1 | 252 | 260 |  |  | PEAKS DB |
| total 5 peptides |  |  |  |  |  |  |  |  |  |  |  |  |  |  |  |  |  |  |  |
| --- | --- | --- | --- | --- | --- | --- | --- | --- | --- | --- | --- | --- | --- | --- | --- | --- | --- | --- | --- |

AXL96660.1

back to list

  

| Protein Coverage
| Supporting Peptides
|

Protein Coverage:

Supporting Peptides:

| Peptide | Uniq | -10lgP | Mass | Length | ppm | m/z | z | RT | Fraction | Scan | Source File | Area BuCaMH04\_B15 | #Feature | #Feature BuCaMH04\_B15 | Start | End | PTM | AScore | Found By |
| --- | --- | --- | --- | --- | --- | --- | --- | --- | --- | --- | --- | --- | --- | --- | --- | --- | --- | --- | --- |
| N.YFVEVGEEC(+57.02)DC(+57.02)GSPR.N | N | 82.16 | 1802.7240 | 15 | 0.5 | 902.3698 | 2 | 18.36 | 1 | 7501 | BuCaMH04.raw | 1.0067E8 | 1 | 1 | 414 | 428 | Carbamidomethylation | C9:Carbamidomethylation:1000.00;C11:Carbamidomethylation:1000.00 | PEAKS DB |
| N.VTLDLFGEWR.E | N | 73.86 | 1234.6346 | 10 | 0.6 | 618.3250 | 2 | 45.55 | 1 | 31857 | BuCaMH04.raw | 9.3115E6 | 1 | 1 | 271 | 280 |  |  | PEAKS DB |
| V.TLDLFGEWR.E | N | 65.70 | 1135.5662 | 9 | 0.5 | 568.7906 | 2 | 42.31 | 1 | 29257 | BuCaMH04.raw | 4.2198E8 | 1 | 1 | 272 | 280 |  |  | PEAKS DB |
| T.LDLFGEWR.E | N | 57.32 | 1034.5186 | 8 | 0.6 | 518.2668 | 2 | 41.26 | 1 | 27863 | BuCaMH04.raw | 4.6562E7 | 1 | 1 | 273 | 280 |  |  | PEAKS DB |
| L.IGLEIWSNK.D | N | 50.68 | 1058.5760 | 9 | 0.6 | 530.2956 | 2 | 27.50 | 1 | 15397 | BuCaMH04.raw | 8.2315E6 | 1 | 1 | 252 | 260 |  |  | PEAKS DB |
| total 5 peptides |  |  |  |  |  |  |  |  |  |  |  |  |  |  |  |  |  |  |  |
| --- | --- | --- | --- | --- | --- | --- | --- | --- | --- | --- | --- | --- | --- | --- | --- | --- | --- | --- | --- |

AXL96668.1

back to list

  

| Protein Coverage
| Supporting Peptides
|

Protein Coverage:

Supporting Peptides:

| Peptide | Uniq | -10lgP | Mass | Length | ppm | m/z | z | RT | Fraction | Scan | Source File | Area BuCaMH04\_B15 | #Feature | #Feature BuCaMH04\_B15 | Start | End | PTM | AScore | Found By |
| --- | --- | --- | --- | --- | --- | --- | --- | --- | --- | --- | --- | --- | --- | --- | --- | --- | --- | --- | --- |
| N.YFVEVGEEC(+57.02)DC(+57.02)GSPR.N | N | 82.16 | 1802.7240 | 15 | 0.5 | 902.3698 | 2 | 18.36 | 1 | 7501 | BuCaMH04.raw | 1.0067E8 | 1 | 1 | 414 | 428 | Carbamidomethylation | C9:Carbamidomethylation:1000.00;C11:Carbamidomethylation:1000.00 | PEAKS DB |
| N.VTLDLFGEWR.E | N | 73.86 | 1234.6346 | 10 | 0.6 | 618.3250 | 2 | 45.55 | 1 | 31857 | BuCaMH04.raw | 9.3115E6 | 1 | 1 | 271 | 280 |  |  | PEAKS DB |
| V.TLDLFGEWR.E | N | 65.70 | 1135.5662 | 9 | 0.5 | 568.7906 | 2 | 42.31 | 1 | 29257 | BuCaMH04.raw | 4.2198E8 | 1 | 1 | 272 | 280 |  |  | PEAKS DB |
| T.LDLFGEWR.E | N | 57.32 | 1034.5186 | 8 | 0.6 | 518.2668 | 2 | 41.26 | 1 | 27863 | BuCaMH04.raw | 4.6562E7 | 1 | 1 | 273 | 280 |  |  | PEAKS DB |
| L.IGLEIWSNK.D | N | 50.68 | 1058.5760 | 9 | 0.6 | 530.2956 | 2 | 27.50 | 1 | 15397 | BuCaMH04.raw | 8.2315E6 | 1 | 1 | 252 | 260 |  |  | PEAKS DB |
| total 5 peptides |  |  |  |  |  |  |  |  |  |  |  |  |  |  |  |  |  |  |  |
| --- | --- | --- | --- | --- | --- | --- | --- | --- | --- | --- | --- | --- | --- | --- | --- | --- | --- | --- | --- |

AXL96656.1

back to list

  

| Protein Coverage
| Supporting Peptides
|

Protein Coverage:

Supporting Peptides:

| Peptide | Uniq | -10lgP | Mass | Length | ppm | m/z | z | RT | Fraction | Scan | Source File | Area BuCaMH04\_B15 | #Feature | #Feature BuCaMH04\_B15 | Start | End | PTM | AScore | Found By |
| --- | --- | --- | --- | --- | --- | --- | --- | --- | --- | --- | --- | --- | --- | --- | --- | --- | --- | --- | --- |
| N.YFVEVGEEC(+57.02)DC(+57.02)GSPR.N | N | 82.16 | 1802.7240 | 15 | 0.5 | 902.3698 | 2 | 18.36 | 1 | 7501 | BuCaMH04.raw | 1.0067E8 | 1 | 1 | 414 | 428 | Carbamidomethylation | C9:Carbamidomethylation:1000.00;C11:Carbamidomethylation:1000.00 | PEAKS DB |
| N.VTLDLFGEWR.E | N | 73.86 | 1234.6346 | 10 | 0.6 | 618.3250 | 2 | 45.55 | 1 | 31857 | BuCaMH04.raw | 9.3115E6 | 1 | 1 | 271 | 280 |  |  | PEAKS DB |
| V.TLDLFGEWR.E | N | 65.70 | 1135.5662 | 9 | 0.5 | 568.7906 | 2 | 42.31 | 1 | 29257 | BuCaMH04.raw | 4.2198E8 | 1 | 1 | 272 | 280 |  |  | PEAKS DB |
| T.LDLFGEWR.E | N | 57.32 | 1034.5186 | 8 | 0.6 | 518.2668 | 2 | 41.26 | 1 | 27863 | BuCaMH04.raw | 4.6562E7 | 1 | 1 | 273 | 280 |  |  | PEAKS DB |
| L.IGLEIWSNK.D | N | 50.68 | 1058.5760 | 9 | 0.6 | 530.2956 | 2 | 27.50 | 1 | 15397 | BuCaMH04.raw | 8.2315E6 | 1 | 1 | 252 | 260 |  |  | PEAKS DB |
| total 5 peptides |  |  |  |  |  |  |  |  |  |  |  |  |  |  |  |  |  |  |  |
| --- | --- | --- | --- | --- | --- | --- | --- | --- | --- | --- | --- | --- | --- | --- | --- | --- | --- | --- | --- |

AXL96651.1

back to list

  

| Protein Coverage
| Supporting Peptides
|

Protein Coverage:

Supporting Peptides:

| Peptide | Uniq | -10lgP | Mass | Length | ppm | m/z | z | RT | Fraction | Scan | Source File | Area BuCaMH04\_B15 | #Feature | #Feature BuCaMH04\_B15 | Start | End | PTM | AScore | Found By |
| --- | --- | --- | --- | --- | --- | --- | --- | --- | --- | --- | --- | --- | --- | --- | --- | --- | --- | --- | --- |
| N.YFVEVGEEC(+57.02)DC(+57.02)GSPR.N | N | 82.16 | 1802.7240 | 15 | 0.5 | 902.3698 | 2 | 18.36 | 1 | 7501 | BuCaMH04.raw | 1.0067E8 | 1 | 1 | 414 | 428 | Carbamidomethylation | C9:Carbamidomethylation:1000.00;C11:Carbamidomethylation:1000.00 | PEAKS DB |
| N.VTLDLFGEWR.E | N | 73.86 | 1234.6346 | 10 | 0.6 | 618.3250 | 2 | 45.55 | 1 | 31857 | BuCaMH04.raw | 9.3115E6 | 1 | 1 | 271 | 280 |  |  | PEAKS DB |
| V.TLDLFGEWR.E | N | 65.70 | 1135.5662 | 9 | 0.5 | 568.7906 | 2 | 42.31 | 1 | 29257 | BuCaMH04.raw | 4.2198E8 | 1 | 1 | 272 | 280 |  |  | PEAKS DB |
| T.LDLFGEWR.E | N | 57.32 | 1034.5186 | 8 | 0.6 | 518.2668 | 2 | 41.26 | 1 | 27863 | BuCaMH04.raw | 4.6562E7 | 1 | 1 | 273 | 280 |  |  | PEAKS DB |
| L.IGLEIWSNK.D | N | 50.68 | 1058.5760 | 9 | 0.6 | 530.2956 | 2 | 27.50 | 1 | 15397 | BuCaMH04.raw | 8.2315E6 | 1 | 1 | 252 | 260 |  |  | PEAKS DB |
| total 5 peptides |  |  |  |  |  |  |  |  |  |  |  |  |  |  |  |  |  |  |  |
| --- | --- | --- | --- | --- | --- | --- | --- | --- | --- | --- | --- | --- | --- | --- | --- | --- | --- | --- | --- |

P86538.2

back to list

  

| Protein Coverage
| Supporting Peptides
|

Protein Coverage:

Supporting Peptides:

| Peptide | Uniq | -10lgP | Mass | Length | ppm | m/z | z | RT | Fraction | Scan | Source File | Area BuCaMH04\_B15 | #Feature | #Feature BuCaMH04\_B15 | Start | End | PTM | AScore | Found By |
| --- | --- | --- | --- | --- | --- | --- | --- | --- | --- | --- | --- | --- | --- | --- | --- | --- | --- | --- | --- |
| K.MFMVSDLTIPVKR.G | Y | 99.29 | 1535.8203 | 13 | 0.0 | 512.9474 | 3 | 30.48 | 1 | 18302 | BuCaMH04.raw | 5.8296E7 | 2 | 2 | 24 | 36 |  |  | PEAKS DB |
| K.MFMVSDLTIPVK.R | Y | 92.45 | 1379.7192 | 12 | 1.2 | 690.8677 | 2 | 40.57 | 1 | 27377 | BuCaMH04.raw | 2.2041E7 | 1 | 1 | 24 | 35 |  |  | PEAKS DB |
| M.FMVSDLTIPVK.R | Y | 56.22 | 1248.6787 | 11 | 0.5 | 625.3469 | 2 | 33.36 | 1 | 20861 | BuCaMH04.raw | 3.6841E6 | 1 | 1 | 25 | 35 |  |  | PEAKS DB |
| total 3 peptides |  |  |  |  |  |  |  |  |  |  |  |  |  |  |  |  |  |  |  |
| --- | --- | --- | --- | --- | --- | --- | --- | --- | --- | --- | --- | --- | --- | --- | --- | --- | --- | --- | --- |

BAU24666.1

back to list

  

| Protein Coverage
| Supporting Peptides
|

Protein Coverage:

Supporting Peptides:

| Peptide | Uniq | -10lgP | Mass | Length | ppm | m/z | z | RT | Fraction | Scan | Source File | Area BuCaMH04\_B15 | #Feature | #Feature BuCaMH04\_B15 | Start | End | PTM | AScore | Found By |
| --- | --- | --- | --- | --- | --- | --- | --- | --- | --- | --- | --- | --- | --- | --- | --- | --- | --- | --- | --- |
| K.MFMVSDLTIPVKR.G | Y | 99.29 | 1535.8203 | 13 | 0.0 | 512.9474 | 3 | 30.48 | 1 | 18302 | BuCaMH04.raw | 5.8296E7 | 2 | 2 | 36 | 48 |  |  | PEAKS DB |
| K.MFMVSDLTIPVK.R | Y | 92.45 | 1379.7192 | 12 | 1.2 | 690.8677 | 2 | 40.57 | 1 | 27377 | BuCaMH04.raw | 2.2041E7 | 1 | 1 | 36 | 47 |  |  | PEAKS DB |
| M.FMVSDLTIPVK.R | Y | 56.22 | 1248.6787 | 11 | 0.5 | 625.3469 | 2 | 33.36 | 1 | 20861 | BuCaMH04.raw | 3.6841E6 | 1 | 1 | 37 | 47 |  |  | PEAKS DB |
| total 3 peptides |  |  |  |  |  |  |  |  |  |  |  |  |  |  |  |  |  |  |  |
| --- | --- | --- | --- | --- | --- | --- | --- | --- | --- | --- | --- | --- | --- | --- | --- | --- | --- | --- | --- |

ABN72545.1

back to list

  

| Protein Coverage
| Supporting Peptides
|

Protein Coverage:

Supporting Peptides:

| Peptide | Uniq | -10lgP | Mass | Length | ppm | m/z | z | RT | Fraction | Scan | Source File | Area BuCaMH04\_B15 | #Feature | #Feature BuCaMH04\_B15 | Start | End | PTM | AScore | Found By |
| --- | --- | --- | --- | --- | --- | --- | --- | --- | --- | --- | --- | --- | --- | --- | --- | --- | --- | --- | --- |
| R.FPC(+57.02)AQLLEPGVYTK.V | N | 103.82 | 1621.8174 | 14 | 0.9 | 811.9167 | 2 | 30.60 | 1 | 18503 | BuCaMH04.raw | 7.1547E7 | 1 | 1 | 254 | 267 | Carbamidomethylation | C3:Carbamidomethylation:1000.00 | PEAKS DB |
| K.VFDYIDWIK.G | Y | 90.52 | 1197.6069 | 9 | 0.7 | 599.8112 | 2 | 45.47 | 1 | 31759 | BuCaMH04.raw | 1.3832E7 | 1 | 1 | 268 | 276 |  |  | PEAKS DB |
| V.FDYIDWIK.G | Y | 61.97 | 1098.5386 | 8 | 1.0 | 550.2771 | 2 | 42.91 | 1 | 29381 | BuCaMH04.raw | 1.3215E6 | 1 | 1 | 269 | 276 |  |  | PEAKS DB |
| G.QIQGIVSWGR.F | Y | 57.51 | 1142.6196 | 10 | -1.0 | 572.3165 | 2 | 44.30 | 1 | 30715 | BuCaMH04.raw | 5.2197E4 | 1 | 1 | 244 | 253 |  |  | PEAKS DB |
| total 4 peptides |  |  |  |  |  |  |  |  |  |  |  |  |  |  |  |  |  |  |  |
| --- | --- | --- | --- | --- | --- | --- | --- | --- | --- | --- | --- | --- | --- | --- | --- | --- | --- | --- | --- |

A8QL57.1

back to list

  

| Protein Coverage
| Supporting Peptides
|

Protein Coverage:

Supporting Peptides:

| Peptide | Uniq | -10lgP | Mass | Length | ppm | m/z | z | RT | Fraction | Scan | Source File | Area BuCaMH04\_B15 | #Feature | #Feature BuCaMH04\_B15 | Start | End | PTM | AScore | Found By |
| --- | --- | --- | --- | --- | --- | --- | --- | --- | --- | --- | --- | --- | --- | --- | --- | --- | --- | --- | --- |
| R.FPC(+57.02)AQLLEPGVYTK.V | N | 103.82 | 1621.8174 | 14 | 0.9 | 811.9167 | 2 | 30.60 | 1 | 18503 | BuCaMH04.raw | 7.1547E7 | 1 | 1 | 254 | 267 | Carbamidomethylation | C3:Carbamidomethylation:1000.00 | PEAKS DB |
| K.VFDYIDWIK.G | Y | 90.52 | 1197.6069 | 9 | 0.7 | 599.8112 | 2 | 45.47 | 1 | 31759 | BuCaMH04.raw | 1.3832E7 | 1 | 1 | 268 | 276 |  |  | PEAKS DB |
| V.FDYIDWIK.G | Y | 61.97 | 1098.5386 | 8 | 1.0 | 550.2771 | 2 | 42.91 | 1 | 29381 | BuCaMH04.raw | 1.3215E6 | 1 | 1 | 269 | 276 |  |  | PEAKS DB |
| G.QIQGIVSWGR.F | Y | 57.51 | 1142.6196 | 10 | -1.0 | 572.3165 | 2 | 44.30 | 1 | 30715 | BuCaMH04.raw | 5.2197E4 | 1 | 1 | 244 | 253 |  |  | PEAKS DB |
| total 4 peptides |  |  |  |  |  |  |  |  |  |  |  |  |  |  |  |  |  |  |  |
| --- | --- | --- | --- | --- | --- | --- | --- | --- | --- | --- | --- | --- | --- | --- | --- | --- | --- | --- | --- |

T\_82

back to list

  

| Protein Coverage
| Supporting Peptides
|

Protein Coverage:

Supporting Peptides:

| Peptide | Uniq | -10lgP | Mass | Length | ppm | m/z | z | RT | Fraction | Scan | Source File | Area BuCaMH04\_B15 | #Feature | #Feature BuCaMH04\_B15 | Start | End | PTM | AScore | Found By |
| --- | --- | --- | --- | --- | --- | --- | --- | --- | --- | --- | --- | --- | --- | --- | --- | --- | --- | --- | --- |
| R.FPC(+57.02)AQLLEPGVYTK.V | N | 103.82 | 1621.8174 | 14 | 0.9 | 811.9167 | 2 | 30.60 | 1 | 18503 | BuCaMH04.raw | 7.1547E7 | 1 | 1 | 254 | 267 | Carbamidomethylation | C3:Carbamidomethylation:1000.00 | PEAKS DB |
| K.VFDYIDWIK.G | Y | 90.52 | 1197.6069 | 9 | 0.7 | 599.8112 | 2 | 45.47 | 1 | 31759 | BuCaMH04.raw | 1.3832E7 | 1 | 1 | 268 | 276 |  |  | PEAKS DB |
| V.FDYIDWIK.G | Y | 61.97 | 1098.5386 | 8 | 1.0 | 550.2771 | 2 | 42.91 | 1 | 29381 | BuCaMH04.raw | 1.3215E6 | 1 | 1 | 269 | 276 |  |  | PEAKS DB |
| G.QIQGIVSWGR.F | Y | 57.51 | 1142.6196 | 10 | -1.0 | 572.3165 | 2 | 44.30 | 1 | 30715 | BuCaMH04.raw | 5.2197E4 | 1 | 1 | 244 | 253 |  |  | PEAKS DB |
| total 4 peptides |  |  |  |  |  |  |  |  |  |  |  |  |  |  |  |  |  |  |  |
| --- | --- | --- | --- | --- | --- | --- | --- | --- | --- | --- | --- | --- | --- | --- | --- | --- | --- | --- | --- |

XP\_034258146.1

back to list

  

| Protein Coverage
| Supporting Peptides
|

Protein Coverage:

Supporting Peptides:

| Peptide | Uniq | -10lgP | Mass | Length | ppm | m/z | z | RT | Fraction | Scan | Source File | Area BuCaMH04\_B15 | #Feature | #Feature BuCaMH04\_B15 | Start | End | PTM | AScore | Found By |
| --- | --- | --- | --- | --- | --- | --- | --- | --- | --- | --- | --- | --- | --- | --- | --- | --- | --- | --- | --- |
| R.LVILGFPC(+57.02)NQFGK.Q | N | 87.60 | 1491.7908 | 13 | 0.3 | 746.9029 | 2 | 39.13 | 1 | 26133 | BuCaMH04.raw | 3.6053E7 | 1 | 1 | 124 | 136 | Carbamidomethylation | C8:Carbamidomethylation:1000.00 | PEAKS DB |
| G.SAHDYGALSITGDYIAFR.N | Y | 62.18 | 1955.9377 | 18 | -0.1 | 652.9865 | 3 | 33.44 | 1 | 20972 | BuCaMH04.raw | 0 | 0 | 0 | 70 | 87 |  |  | PEAKS DB |
| R.LVILGFPC(+57.02)NQ.F | N | 61.53 | 1159.6060 | 10 | -0.1 | 580.8102 | 2 | 45.31 | 1 | 31387 | BuCaMH04.raw | 5.0904E6 | 1 | 1 | 124 | 133 | Carbamidomethylation | C8:Carbamidomethylation:1000.00 | PEAKS DB |
| Q.GSAHDYGALSITGDYIAFR.N | Y | 60.11 | 2012.9591 | 19 | 0.9 | 671.9943 | 3 | 32.92 | 1 | 20655 | BuCaMH04.raw | 1.5081E7 | 1 | 1 | 69 | 87 |  |  | PEAKS DB |
| H.DYGALSITGDYIAFR.N | Y | 58.35 | 1660.8096 | 15 | 0.9 | 831.4128 | 2 | 46.01 | 1 | 32371 | BuCaMH04.raw | 6.5675E5 | 1 | 1 | 73 | 87 |  |  | PEAKS DB |
| L.SITGDYIAFR.N | Y | 52.31 | 1141.5768 | 10 | 0.5 | 571.7960 | 2 | 25.05 | 1 | 13234 | BuCaMH04.raw | 1.6904E6 | 1 | 1 | 78 | 87 |  |  | PEAKS DB |
| total 6 peptides |  |  |  |  |  |  |  |  |  |  |  |  |  |  |  |  |  |  |  |
| --- | --- | --- | --- | --- | --- | --- | --- | --- | --- | --- | --- | --- | --- | --- | --- | --- | --- | --- | --- |

XP\_026555793.1

back to list

  

| Protein Coverage
| Supporting Peptides
|

Protein Coverage:

Supporting Peptides:

| Peptide | Uniq | -10lgP | Mass | Length | ppm | m/z | z | RT | Fraction | Scan | Source File | Area BuCaMH04\_B15 | #Feature | #Feature BuCaMH04\_B15 | Start | End | PTM | AScore | Found By |
| --- | --- | --- | --- | --- | --- | --- | --- | --- | --- | --- | --- | --- | --- | --- | --- | --- | --- | --- | --- |
| R.GTTEHLLEIVLGR.C | N | 85.53 | 1436.7987 | 13 | 0.8 | 479.9406 | 3 | 36.09 | 1 | 23451 | BuCaMH04.raw | 2.1732E7 | 2 | 2 | 56 | 68 |  |  | PEAKS DB |
| K.SSIFGSVEIVNLNPEK.V | N | 82.75 | 1731.9043 | 16 | 1.0 | 866.9603 | 2 | 39.29 | 1 | 26305 | BuCaMH04.raw | 2.9489E7 | 1 | 1 | 222 | 237 |  |  | PEAKS DB |
| R.KSSIFGSVEIVNLNPEK.V | N | 65.40 | 1859.9993 | 17 | 0.7 | 621.0074 | 3 | 27.30 | 1 | 15442 | BuCaMH04.raw | 8.8246E6 | 1 | 1 | 221 | 237 |  |  | PEAKS DB |
| M.QIWLMHDIGGPQR.E | Y | 64.96 | 1549.7823 | 13 | 0.8 | 517.6018 | 3 | 23.73 | 1 | 12129 | BuCaMH04.raw | 1.097E6 | 1 | 1 | 242 | 254 |  |  | PEAKS DB |
| total 4 peptides |  |  |  |  |  |  |  |  |  |  |  |  |  |  |  |  |  |  |  |
| --- | --- | --- | --- | --- | --- | --- | --- | --- | --- | --- | --- | --- | --- | --- | --- | --- | --- | --- | --- |

1PSH

back to list

  

| Protein Coverage
| Supporting Peptides
|

Protein Coverage:

Supporting Peptides:

| Peptide | Uniq | -10lgP | Mass | Length | ppm | m/z | z | RT | Fraction | Scan | Source File | Area BuCaMH04\_B15 | #Feature | #Feature BuCaMH04\_B15 | Start | End | PTM | AScore | Found By |
| --- | --- | --- | --- | --- | --- | --- | --- | --- | --- | --- | --- | --- | --- | --- | --- | --- | --- | --- | --- |
| K.ISGC(+57.02)WPYFK.T | Y | 80.10 | 1156.5375 | 9 | 0.9 | 579.2766 | 2 | 29.35 | 1 | 17392 | BuCaMH04.raw | 2.2609E8 | 1 | 1 | 57 | 65 | Carbamidomethylation | C4:Carbamidomethylation:1000.00 | PEAKS DB |
| R.SWWDFADYGC(+57.02)YC(+57.02)GR.G | Y | 71.56 | 1841.6926 | 14 | 0.3 | 921.8539 | 2 | 49.95 | 1 | 35609 | BuCaMH04.raw | 3.943E6 | 1 | 1 | 17 | 30 | Carbamidomethylation | C10:Carbamidomethylation:1000.00;C12:Carbamidomethylation:1000.00 | PEAKS DB |
| S.WWDFADYGC(+57.02)YC(+57.02)GR.G | Y | 65.11 | 1754.6605 | 13 | 0.7 | 878.3381 | 2 | 44.02 | 1 | 30567 | BuCaMH04.raw | 9.8555E7 | 1 | 1 | 18 | 30 | Carbamidomethylation | C9:Carbamidomethylation:1000.00;C11:Carbamidomethylation:1000.00 | PEAKS DB |
| R.LAAIC(+57.02)FAGAPYN.D | N | 52.26 | 1266.6067 | 12 | -0.4 | 634.3104 | 2 | 35.93 | 1 | 23316 | BuCaMH04.raw | 9.6858E6 | 1 | 1 | 95 | 106 | Carbamidomethylation | C5:Carbamidomethylation:1000.00 | PEAKS DB |
| total 4 peptides |  |  |  |  |  |  |  |  |  |  |  |  |  |  |  |  |  |  |  |
| --- | --- | --- | --- | --- | --- | --- | --- | --- | --- | --- | --- | --- | --- | --- | --- | --- | --- | --- | --- |

1A3F

back to list

  

| Protein Coverage
| Supporting Peptides
|

Protein Coverage:

Supporting Peptides:

| Peptide | Uniq | -10lgP | Mass | Length | ppm | m/z | z | RT | Fraction | Scan | Source File | Area BuCaMH04\_B15 | #Feature | #Feature BuCaMH04\_B15 | Start | End | PTM | AScore | Found By |
| --- | --- | --- | --- | --- | --- | --- | --- | --- | --- | --- | --- | --- | --- | --- | --- | --- | --- | --- | --- |
| K.ISGC(+57.02)WPYFK.T | Y | 80.10 | 1156.5375 | 9 | 0.9 | 579.2766 | 2 | 29.35 | 1 | 17392 | BuCaMH04.raw | 2.2609E8 | 1 | 1 | 57 | 65 | Carbamidomethylation | C4:Carbamidomethylation:1000.00 | PEAKS DB |
| R.SWWDFADYGC(+57.02)YC(+57.02)GR.G | Y | 71.56 | 1841.6926 | 14 | 0.3 | 921.8539 | 2 | 49.95 | 1 | 35609 | BuCaMH04.raw | 3.943E6 | 1 | 1 | 17 | 30 | Carbamidomethylation | C10:Carbamidomethylation:1000.00;C12:Carbamidomethylation:1000.00 | PEAKS DB |
| S.WWDFADYGC(+57.02)YC(+57.02)GR.G | Y | 65.11 | 1754.6605 | 13 | 0.7 | 878.3381 | 2 | 44.02 | 1 | 30567 | BuCaMH04.raw | 9.8555E7 | 1 | 1 | 18 | 30 | Carbamidomethylation | C9:Carbamidomethylation:1000.00;C11:Carbamidomethylation:1000.00 | PEAKS DB |
| R.LAAIC(+57.02)FAGAPYN.D | N | 52.26 | 1266.6067 | 12 | -0.4 | 634.3104 | 2 | 35.93 | 1 | 23316 | BuCaMH04.raw | 9.6858E6 | 1 | 1 | 95 | 106 | Carbamidomethylation | C5:Carbamidomethylation:1000.00 | PEAKS DB |
| total 4 peptides |  |  |  |  |  |  |  |  |  |  |  |  |  |  |  |  |  |  |  |
| --- | --- | --- | --- | --- | --- | --- | --- | --- | --- | --- | --- | --- | --- | --- | --- | --- | --- | --- | --- |

1A3D

back to list

  

| Protein Coverage
| Supporting Peptides
|

Protein Coverage:

Supporting Peptides:

| Peptide | Uniq | -10lgP | Mass | Length | ppm | m/z | z | RT | Fraction | Scan | Source File | Area BuCaMH04\_B15 | #Feature | #Feature BuCaMH04\_B15 | Start | End | PTM | AScore | Found By |
| --- | --- | --- | --- | --- | --- | --- | --- | --- | --- | --- | --- | --- | --- | --- | --- | --- | --- | --- | --- |
| K.ISGC(+57.02)WPYFK.T | Y | 80.10 | 1156.5375 | 9 | 0.9 | 579.2766 | 2 | 29.35 | 1 | 17392 | BuCaMH04.raw | 2.2609E8 | 1 | 1 | 57 | 65 | Carbamidomethylation | C4:Carbamidomethylation:1000.00 | PEAKS DB |
| R.SWWDFADYGC(+57.02)YC(+57.02)GR.G | Y | 71.56 | 1841.6926 | 14 | 0.3 | 921.8539 | 2 | 49.95 | 1 | 35609 | BuCaMH04.raw | 3.943E6 | 1 | 1 | 17 | 30 | Carbamidomethylation | C10:Carbamidomethylation:1000.00;C12:Carbamidomethylation:1000.00 | PEAKS DB |
| S.WWDFADYGC(+57.02)YC(+57.02)GR.G | Y | 65.11 | 1754.6605 | 13 | 0.7 | 878.3381 | 2 | 44.02 | 1 | 30567 | BuCaMH04.raw | 9.8555E7 | 1 | 1 | 18 | 30 | Carbamidomethylation | C9:Carbamidomethylation:1000.00;C11:Carbamidomethylation:1000.00 | PEAKS DB |
| R.LAAIC(+57.02)FAGAPYN.D | N | 52.26 | 1266.6067 | 12 | -0.4 | 634.3104 | 2 | 35.93 | 1 | 23316 | BuCaMH04.raw | 9.6858E6 | 1 | 1 | 95 | 106 | Carbamidomethylation | C5:Carbamidomethylation:1000.00 | PEAKS DB |
| total 4 peptides |  |  |  |  |  |  |  |  |  |  |  |  |  |  |  |  |  |  |  |
| --- | --- | --- | --- | --- | --- | --- | --- | --- | --- | --- | --- | --- | --- | --- | --- | --- | --- | --- | --- |

P15445.1

back to list

  

| Protein Coverage
| Supporting Peptides
|

Protein Coverage:

Supporting Peptides:

| Peptide | Uniq | -10lgP | Mass | Length | ppm | m/z | z | RT | Fraction | Scan | Source File | Area BuCaMH04\_B15 | #Feature | #Feature BuCaMH04\_B15 | Start | End | PTM | AScore | Found By |
| --- | --- | --- | --- | --- | --- | --- | --- | --- | --- | --- | --- | --- | --- | --- | --- | --- | --- | --- | --- |
| K.ISGC(+57.02)WPYFK.T | Y | 80.10 | 1156.5375 | 9 | 0.9 | 579.2766 | 2 | 29.35 | 1 | 17392 | BuCaMH04.raw | 2.2609E8 | 1 | 1 | 57 | 65 | Carbamidomethylation | C4:Carbamidomethylation:1000.00 | PEAKS DB |
| R.SWWDFADYGC(+57.02)YC(+57.02)GR.G | Y | 71.56 | 1841.6926 | 14 | 0.3 | 921.8539 | 2 | 49.95 | 1 | 35609 | BuCaMH04.raw | 3.943E6 | 1 | 1 | 17 | 30 | Carbamidomethylation | C10:Carbamidomethylation:1000.00;C12:Carbamidomethylation:1000.00 | PEAKS DB |
| S.WWDFADYGC(+57.02)YC(+57.02)GR.G | Y | 65.11 | 1754.6605 | 13 | 0.7 | 878.3381 | 2 | 44.02 | 1 | 30567 | BuCaMH04.raw | 9.8555E7 | 1 | 1 | 18 | 30 | Carbamidomethylation | C9:Carbamidomethylation:1000.00;C11:Carbamidomethylation:1000.00 | PEAKS DB |
| R.LAAIC(+57.02)FAGAPYN.D | N | 52.26 | 1266.6067 | 12 | -0.4 | 634.3104 | 2 | 35.93 | 1 | 23316 | BuCaMH04.raw | 9.6858E6 | 1 | 1 | 95 | 106 | Carbamidomethylation | C5:Carbamidomethylation:1000.00 | PEAKS DB |
| total 4 peptides |  |  |  |  |  |  |  |  |  |  |  |  |  |  |  |  |  |  |  |
| --- | --- | --- | --- | --- | --- | --- | --- | --- | --- | --- | --- | --- | --- | --- | --- | --- | --- | --- | --- |

CAA45372.1

back to list

  

| Protein Coverage
| Supporting Peptides
|

Protein Coverage:

Supporting Peptides:

| Peptide | Uniq | -10lgP | Mass | Length | ppm | m/z | z | RT | Fraction | Scan | Source File | Area BuCaMH04\_B15 | #Feature | #Feature BuCaMH04\_B15 | Start | End | PTM | AScore | Found By |
| --- | --- | --- | --- | --- | --- | --- | --- | --- | --- | --- | --- | --- | --- | --- | --- | --- | --- | --- | --- |
| K.ISGC(+57.02)WPYFK.T | Y | 80.10 | 1156.5375 | 9 | 0.9 | 579.2766 | 2 | 29.35 | 1 | 17392 | BuCaMH04.raw | 2.2609E8 | 1 | 1 | 58 | 66 | Carbamidomethylation | C4:Carbamidomethylation:1000.00 | PEAKS DB |
| R.SWWDFADYGC(+57.02)YC(+57.02)GR.G | Y | 71.56 | 1841.6926 | 14 | 0.3 | 921.8539 | 2 | 49.95 | 1 | 35609 | BuCaMH04.raw | 3.943E6 | 1 | 1 | 18 | 31 | Carbamidomethylation | C10:Carbamidomethylation:1000.00;C12:Carbamidomethylation:1000.00 | PEAKS DB |
| S.WWDFADYGC(+57.02)YC(+57.02)GR.G | Y | 65.11 | 1754.6605 | 13 | 0.7 | 878.3381 | 2 | 44.02 | 1 | 30567 | BuCaMH04.raw | 9.8555E7 | 1 | 1 | 19 | 31 | Carbamidomethylation | C9:Carbamidomethylation:1000.00;C11:Carbamidomethylation:1000.00 | PEAKS DB |
| R.LAAIC(+57.02)FAGAPYN.D | N | 52.26 | 1266.6067 | 12 | -0.4 | 634.3104 | 2 | 35.93 | 1 | 23316 | BuCaMH04.raw | 9.6858E6 | 1 | 1 | 96 | 107 | Carbamidomethylation | C5:Carbamidomethylation:1000.00 | PEAKS DB |
| total 4 peptides |  |  |  |  |  |  |  |  |  |  |  |  |  |  |  |  |  |  |  |
| --- | --- | --- | --- | --- | --- | --- | --- | --- | --- | --- | --- | --- | --- | --- | --- | --- | --- | --- | --- |

AXL95273.1

back to list

  

| Protein Coverage
| Supporting Peptides
|

Protein Coverage:

Supporting Peptides:

| Peptide | Uniq | -10lgP | Mass | Length | ppm | m/z | z | RT | Fraction | Scan | Source File | Area BuCaMH04\_B15 | #Feature | #Feature BuCaMH04\_B15 | Start | End | PTM | AScore | Found By |
| --- | --- | --- | --- | --- | --- | --- | --- | --- | --- | --- | --- | --- | --- | --- | --- | --- | --- | --- | --- |
| K.ETPVLSNPGPYLEFRDEVEELQK.H | N | 72.40 | 2688.3281 | 23 | 0.9 | 897.1174 | 3 | 41.06 | 1 | 27972 | BuCaMH04.raw | 3.1625E7 | 1 | 1 | 194 | 216 |  |  | PEAKS DB |
| R.FHEC(+57.02)NLGNLIC(+57.02)DAVVYNNLR.H | N | 71.60 | 2420.1365 | 20 | 0.6 | 807.7199 | 3 | 41.26 | 1 | 27842 | BuCaMH04.raw | 2.9367E7 | 1 | 1 | 369 | 388 | Carbamidomethylation | C4:Carbamidomethylation:1000.00;C11:Carbamidomethylation:1000.00 | PEAKS DB |
| K.ETPVLSNPGPYLEFR.D | N | 67.68 | 1717.8674 | 15 | 1.3 | 859.9421 | 2 | 33.85 | 1 | 21396 | BuCaMH04.raw | 6.7956E6 | 1 | 1 | 194 | 208 |  |  | PEAKS DB |
| R.EVVQFMNSLR.Y | Y | 59.13 | 1221.6176 | 10 | 2.0 | 611.8173 | 2 | 26.10 | 1 | 14189 | BuCaMH04.raw | 1.7317E6 | 1 | 1 | 114 | 123 |  |  | PEAKS DB |
| C.QVPVVQAYAFGK.Y | N | 54.70 | 1305.7081 | 12 | -0.4 | 653.8611 | 2 | 22.79 | 1 | 11255 | BuCaMH04.raw | 5.2789E6 | 1 | 1 | 288 | 299 |  |  | PEAKS DB |
| total 5 peptides |  |  |  |  |  |  |  |  |  |  |  |  |  |  |  |  |  |  |  |
| --- | --- | --- | --- | --- | --- | --- | --- | --- | --- | --- | --- | --- | --- | --- | --- | --- | --- | --- | --- |

JAG67188.1

back to list

  

| Protein Coverage
| Supporting Peptides
|

Protein Coverage:

Supporting Peptides:

| Peptide | Uniq | -10lgP | Mass | Length | ppm | m/z | z | RT | Fraction | Scan | Source File | Area BuCaMH04\_B15 | #Feature | #Feature BuCaMH04\_B15 | Start | End | PTM | AScore | Found By |
| --- | --- | --- | --- | --- | --- | --- | --- | --- | --- | --- | --- | --- | --- | --- | --- | --- | --- | --- | --- |
| K.ETPVLSNPGPYLEFRDEVEELQK.H | N | 72.40 | 2688.3281 | 23 | 0.9 | 897.1174 | 3 | 41.06 | 1 | 27972 | BuCaMH04.raw | 3.1625E7 | 1 | 1 | 194 | 216 |  |  | PEAKS DB |
| R.FHEC(+57.02)NLGNLIC(+57.02)DAVVYNNLR.H | N | 71.60 | 2420.1365 | 20 | 0.6 | 807.7199 | 3 | 41.26 | 1 | 27842 | BuCaMH04.raw | 2.9367E7 | 1 | 1 | 369 | 388 | Carbamidomethylation | C4:Carbamidomethylation:1000.00;C11:Carbamidomethylation:1000.00 | PEAKS DB |
| K.ETPVLSNPGPYLEFR.D | N | 67.68 | 1717.8674 | 15 | 1.3 | 859.9421 | 2 | 33.85 | 1 | 21396 | BuCaMH04.raw | 6.7956E6 | 1 | 1 | 194 | 208 |  |  | PEAKS DB |
| R.EVVQFMNSLR.Y | Y | 59.13 | 1221.6176 | 10 | 2.0 | 611.8173 | 2 | 26.10 | 1 | 14189 | BuCaMH04.raw | 1.7317E6 | 1 | 1 | 114 | 123 |  |  | PEAKS DB |
| R.QVPVVQAYAFGK.Y | N | 54.70 | 1305.7081 | 12 | -0.4 | 653.8611 | 2 | 22.79 | 1 | 11255 | BuCaMH04.raw | 5.2789E6 | 1 | 1 | 288 | 299 |  |  | PEAKS DB |
| total 5 peptides |  |  |  |  |  |  |  |  |  |  |  |  |  |  |  |  |  |  |  |
| --- | --- | --- | --- | --- | --- | --- | --- | --- | --- | --- | --- | --- | --- | --- | --- | --- | --- | --- | --- |

2CTX

back to list

  

| Protein Coverage
| Supporting Peptides
|

Protein Coverage:

Supporting Peptides:

| Peptide | Uniq | -10lgP | Mass | Length | ppm | m/z | z | RT | Fraction | Scan | Source File | Area BuCaMH04\_B15 | #Feature | #Feature BuCaMH04\_B15 | Start | End | PTM | AScore | Found By |
| --- | --- | --- | --- | --- | --- | --- | --- | --- | --- | --- | --- | --- | --- | --- | --- | --- | --- | --- | --- |
| K.TGVDIQC(+57.02)C(+57.02)STDNC(+57.02)NPFPTR.K | Y | 101.34 | 2240.9248 | 19 | -0.3 | 1121.4694 | 2 | 20.06 | 1 | 9098 | BuCaMH04.raw | 5.8117E9 | 2 | 2 | 50 | 68 | Carbamidomethylation | C7:Carbamidomethylation:1000.00;C8:Carbamidomethylation:1000.00;C13:Carbamidomethylation:1000.00 | PEAKS DB |
| R.C(+57.02)FITPDITSK.D | N | 75.14 | 1180.5798 | 10 | 0.3 | 591.2974 | 2 | 43.30 | 1 | 30061 | BuCaMH04.raw | 1.3723E8 | 2 | 2 | 3 | 12 | Carbamidomethylation | C1:Carbamidomethylation:1000.00 | PEAKS DB |
| G.VDIQC(+57.02)C(+57.02)STDNC(+57.02)NPFPTR.K | Y | 57.46 | 2082.8557 | 17 | 0.2 | 1042.4353 | 2 | 17.82 | 1 | 6985 | BuCaMH04.raw | 4.5197E6 | 1 | 1 | 52 | 68 | Carbamidomethylation | C5:Carbamidomethylation:1000.00;C6:Carbamidomethylation:1000.00;C11:Carbamidomethylation:1000.00 | PEAKS DB |
| total 3 peptides |  |  |  |  |  |  |  |  |  |  |  |  |  |  |  |  |  |  |  |
| --- | --- | --- | --- | --- | --- | --- | --- | --- | --- | --- | --- | --- | --- | --- | --- | --- | --- | --- | --- |

1LXH

back to list

  

| Protein Coverage
| Supporting Peptides
|

Protein Coverage:

Supporting Peptides:

| Peptide | Uniq | -10lgP | Mass | Length | ppm | m/z | z | RT | Fraction | Scan | Source File | Area BuCaMH04\_B15 | #Feature | #Feature BuCaMH04\_B15 | Start | End | PTM | AScore | Found By |
| --- | --- | --- | --- | --- | --- | --- | --- | --- | --- | --- | --- | --- | --- | --- | --- | --- | --- | --- | --- |
| K.TGVDIQC(+57.02)C(+57.02)STDNC(+57.02)NPFPTR.K | Y | 101.34 | 2240.9248 | 19 | -0.3 | 1121.4694 | 2 | 20.06 | 1 | 9098 | BuCaMH04.raw | 5.8117E9 | 2 | 2 | 50 | 68 | Carbamidomethylation | C7:Carbamidomethylation:1000.00;C8:Carbamidomethylation:1000.00;C13:Carbamidomethylation:1000.00 | PEAKS DB |
| R.C(+57.02)FITPDITSK.D | N | 75.14 | 1180.5798 | 10 | 0.3 | 591.2974 | 2 | 43.30 | 1 | 30061 | BuCaMH04.raw | 1.3723E8 | 2 | 2 | 3 | 12 | Carbamidomethylation | C1:Carbamidomethylation:1000.00 | PEAKS DB |
| G.VDIQC(+57.02)C(+57.02)STDNC(+57.02)NPFPTR.K | Y | 57.46 | 2082.8557 | 17 | 0.2 | 1042.4353 | 2 | 17.82 | 1 | 6985 | BuCaMH04.raw | 4.5197E6 | 1 | 1 | 52 | 68 | Carbamidomethylation | C5:Carbamidomethylation:1000.00;C6:Carbamidomethylation:1000.00;C11:Carbamidomethylation:1000.00 | PEAKS DB |
| total 3 peptides |  |  |  |  |  |  |  |  |  |  |  |  |  |  |  |  |  |  |  |
| --- | --- | --- | --- | --- | --- | --- | --- | --- | --- | --- | --- | --- | --- | --- | --- | --- | --- | --- | --- |

1LXG

back to list

  

| Protein Coverage
| Supporting Peptides
|

Protein Coverage:

Supporting Peptides:

| Peptide | Uniq | -10lgP | Mass | Length | ppm | m/z | z | RT | Fraction | Scan | Source File | Area BuCaMH04\_B15 | #Feature | #Feature BuCaMH04\_B15 | Start | End | PTM | AScore | Found By |
| --- | --- | --- | --- | --- | --- | --- | --- | --- | --- | --- | --- | --- | --- | --- | --- | --- | --- | --- | --- |
| K.TGVDIQC(+57.02)C(+57.02)STDNC(+57.02)NPFPTR.K | Y | 101.34 | 2240.9248 | 19 | -0.3 | 1121.4694 | 2 | 20.06 | 1 | 9098 | BuCaMH04.raw | 5.8117E9 | 2 | 2 | 50 | 68 | Carbamidomethylation | C7:Carbamidomethylation:1000.00;C8:Carbamidomethylation:1000.00;C13:Carbamidomethylation:1000.00 | PEAKS DB |
| R.C(+57.02)FITPDITSK.D | N | 75.14 | 1180.5798 | 10 | 0.3 | 591.2974 | 2 | 43.30 | 1 | 30061 | BuCaMH04.raw | 1.3723E8 | 2 | 2 | 3 | 12 | Carbamidomethylation | C1:Carbamidomethylation:1000.00 | PEAKS DB |
| G.VDIQC(+57.02)C(+57.02)STDNC(+57.02)NPFPTR.K | Y | 57.46 | 2082.8557 | 17 | 0.2 | 1042.4353 | 2 | 17.82 | 1 | 6985 | BuCaMH04.raw | 4.5197E6 | 1 | 1 | 52 | 68 | Carbamidomethylation | C5:Carbamidomethylation:1000.00;C6:Carbamidomethylation:1000.00;C11:Carbamidomethylation:1000.00 | PEAKS DB |
| total 3 peptides |  |  |  |  |  |  |  |  |  |  |  |  |  |  |  |  |  |  |  |
| --- | --- | --- | --- | --- | --- | --- | --- | --- | --- | --- | --- | --- | --- | --- | --- | --- | --- | --- | --- |

P01391.1

back to list

  

| Protein Coverage
| Supporting Peptides
|

Protein Coverage:

Supporting Peptides:

| Peptide | Uniq | -10lgP | Mass | Length | ppm | m/z | z | RT | Fraction | Scan | Source File | Area BuCaMH04\_B15 | #Feature | #Feature BuCaMH04\_B15 | Start | End | PTM | AScore | Found By |
| --- | --- | --- | --- | --- | --- | --- | --- | --- | --- | --- | --- | --- | --- | --- | --- | --- | --- | --- | --- |
| K.TGVDIQC(+57.02)C(+57.02)STDNC(+57.02)NPFPTR.K | Y | 101.34 | 2240.9248 | 19 | -0.3 | 1121.4694 | 2 | 20.06 | 1 | 9098 | BuCaMH04.raw | 5.8117E9 | 2 | 2 | 50 | 68 | Carbamidomethylation | C7:Carbamidomethylation:1000.00;C8:Carbamidomethylation:1000.00;C13:Carbamidomethylation:1000.00 | PEAKS DB |
| R.C(+57.02)FITPDITSK.D | N | 75.14 | 1180.5798 | 10 | 0.3 | 591.2974 | 2 | 43.30 | 1 | 30061 | BuCaMH04.raw | 1.3723E8 | 2 | 2 | 3 | 12 | Carbamidomethylation | C1:Carbamidomethylation:1000.00 | PEAKS DB |
| G.VDIQC(+57.02)C(+57.02)STDNC(+57.02)NPFPTR.K | Y | 57.46 | 2082.8557 | 17 | 0.2 | 1042.4353 | 2 | 17.82 | 1 | 6985 | BuCaMH04.raw | 4.5197E6 | 1 | 1 | 52 | 68 | Carbamidomethylation | C5:Carbamidomethylation:1000.00;C6:Carbamidomethylation:1000.00;C11:Carbamidomethylation:1000.00 | PEAKS DB |
| total 3 peptides |  |  |  |  |  |  |  |  |  |  |  |  |  |  |  |  |  |  |  |
| --- | --- | --- | --- | --- | --- | --- | --- | --- | --- | --- | --- | --- | --- | --- | --- | --- | --- | --- | --- |

4AEA

back to list

  

| Protein Coverage
| Supporting Peptides
|

Protein Coverage:

Supporting Peptides:

| Peptide | Uniq | -10lgP | Mass | Length | ppm | m/z | z | RT | Fraction | Scan | Source File | Area BuCaMH04\_B15 | #Feature | #Feature BuCaMH04\_B15 | Start | End | PTM | AScore | Found By |
| --- | --- | --- | --- | --- | --- | --- | --- | --- | --- | --- | --- | --- | --- | --- | --- | --- | --- | --- | --- |
| K.TGVDIQC(+57.02)C(+57.02)STDNC(+57.02)NPFPTR.K | Y | 101.34 | 2240.9248 | 19 | -0.3 | 1121.4694 | 2 | 20.06 | 1 | 9098 | BuCaMH04.raw | 5.8117E9 | 2 | 2 | 50 | 68 | Carbamidomethylation | C7:Carbamidomethylation:1000.00;C8:Carbamidomethylation:1000.00;C13:Carbamidomethylation:1000.00 | PEAKS DB |
| R.C(+57.02)FITPDITSK.D | N | 75.14 | 1180.5798 | 10 | 0.3 | 591.2974 | 2 | 43.30 | 1 | 30061 | BuCaMH04.raw | 1.3723E8 | 2 | 2 | 3 | 12 | Carbamidomethylation | C1:Carbamidomethylation:1000.00 | PEAKS DB |
| G.VDIQC(+57.02)C(+57.02)STDNC(+57.02)NPFPTR.K | Y | 57.46 | 2082.8557 | 17 | 0.2 | 1042.4353 | 2 | 17.82 | 1 | 6985 | BuCaMH04.raw | 4.5197E6 | 1 | 1 | 52 | 68 | Carbamidomethylation | C5:Carbamidomethylation:1000.00;C6:Carbamidomethylation:1000.00;C11:Carbamidomethylation:1000.00 | PEAKS DB |
| total 3 peptides |  |  |  |  |  |  |  |  |  |  |  |  |  |  |  |  |  |  |  |
| --- | --- | --- | --- | --- | --- | --- | --- | --- | --- | --- | --- | --- | --- | --- | --- | --- | --- | --- | --- |

1CTX

back to list

  

| Protein Coverage
| Supporting Peptides
|

Protein Coverage:

Supporting Peptides:

| Peptide | Uniq | -10lgP | Mass | Length | ppm | m/z | z | RT | Fraction | Scan | Source File | Area BuCaMH04\_B15 | #Feature | #Feature BuCaMH04\_B15 | Start | End | PTM | AScore | Found By |
| --- | --- | --- | --- | --- | --- | --- | --- | --- | --- | --- | --- | --- | --- | --- | --- | --- | --- | --- | --- |
| K.TGVDIQC(+57.02)C(+57.02)STDNC(+57.02)NPFPTR.K | Y | 101.34 | 2240.9248 | 19 | -0.3 | 1121.4694 | 2 | 20.06 | 1 | 9098 | BuCaMH04.raw | 5.8117E9 | 2 | 2 | 50 | 68 | Carbamidomethylation | C7:Carbamidomethylation:1000.00;C8:Carbamidomethylation:1000.00;C13:Carbamidomethylation:1000.00 | PEAKS DB |
| R.C(+57.02)FITPDITSK.D | N | 75.14 | 1180.5798 | 10 | 0.3 | 591.2974 | 2 | 43.30 | 1 | 30061 | BuCaMH04.raw | 1.3723E8 | 2 | 2 | 3 | 12 | Carbamidomethylation | C1:Carbamidomethylation:1000.00 | PEAKS DB |
| G.VDIQC(+57.02)C(+57.02)STDNC(+57.02)NPFPTR.K | Y | 57.46 | 2082.8557 | 17 | 0.2 | 1042.4353 | 2 | 17.82 | 1 | 6985 | BuCaMH04.raw | 4.5197E6 | 1 | 1 | 52 | 68 | Carbamidomethylation | C5:Carbamidomethylation:1000.00;C6:Carbamidomethylation:1000.00;C11:Carbamidomethylation:1000.00 | PEAKS DB |
| total 3 peptides |  |  |  |  |  |  |  |  |  |  |  |  |  |  |  |  |  |  |  |
| --- | --- | --- | --- | --- | --- | --- | --- | --- | --- | --- | --- | --- | --- | --- | --- | --- | --- | --- | --- |

1YI5

back to list

  

| Protein Coverage
| Supporting Peptides
|

Protein Coverage:

Supporting Peptides:

| Peptide | Uniq | -10lgP | Mass | Length | ppm | m/z | z | RT | Fraction | Scan | Source File | Area BuCaMH04\_B15 | #Feature | #Feature BuCaMH04\_B15 | Start | End | PTM | AScore | Found By |
| --- | --- | --- | --- | --- | --- | --- | --- | --- | --- | --- | --- | --- | --- | --- | --- | --- | --- | --- | --- |
| K.TGVDIQC(+57.02)C(+57.02)STDNC(+57.02)NPFPTR.K | Y | 101.34 | 2240.9248 | 19 | -0.3 | 1121.4694 | 2 | 20.06 | 1 | 9098 | BuCaMH04.raw | 5.8117E9 | 2 | 2 | 50 | 68 | Carbamidomethylation | C7:Carbamidomethylation:1000.00;C8:Carbamidomethylation:1000.00;C13:Carbamidomethylation:1000.00 | PEAKS DB |
| R.C(+57.02)FITPDITSK.D | N | 75.14 | 1180.5798 | 10 | 0.3 | 591.2974 | 2 | 43.30 | 1 | 30061 | BuCaMH04.raw | 1.3723E8 | 2 | 2 | 3 | 12 | Carbamidomethylation | C1:Carbamidomethylation:1000.00 | PEAKS DB |
| G.VDIQC(+57.02)C(+57.02)STDNC(+57.02)NPFPTR.K | Y | 57.46 | 2082.8557 | 17 | 0.2 | 1042.4353 | 2 | 17.82 | 1 | 6985 | BuCaMH04.raw | 4.5197E6 | 1 | 1 | 52 | 68 | Carbamidomethylation | C5:Carbamidomethylation:1000.00;C6:Carbamidomethylation:1000.00;C11:Carbamidomethylation:1000.00 | PEAKS DB |
| total 3 peptides |  |  |  |  |  |  |  |  |  |  |  |  |  |  |  |  |  |  |  |
| --- | --- | --- | --- | --- | --- | --- | --- | --- | --- | --- | --- | --- | --- | --- | --- | --- | --- | --- | --- |

XP\_013924291.1

back to list

  

| Protein Coverage
| Supporting Peptides
|

Protein Coverage:

Supporting Peptides:

| Peptide | Uniq | -10lgP | Mass | Length | ppm | m/z | z | RT | Fraction | Scan | Source File | Area BuCaMH04\_B15 | #Feature | #Feature BuCaMH04\_B15 | Start | End | PTM | AScore | Found By |
| --- | --- | --- | --- | --- | --- | --- | --- | --- | --- | --- | --- | --- | --- | --- | --- | --- | --- | --- | --- |
| K.LALDIEIATYR.K | N | 82.54 | 1276.7026 | 11 | 0.6 | 639.3590 | 2 | 34.70 | 1 | 22142 | BuCaMH04.raw | 2.0032E6 | 1 | 1 | 387 | 397 |  |  | PEAKS DB |
| R.NLDLDSIIAEVK.A | N | 79.10 | 1328.7188 | 12 | 0.7 | 665.3671 | 2 | 45.17 | 1 | 31484 | BuCaMH04.raw | 4.9909E6 | 1 | 1 | 258 | 269 |  |  | PEAKS DB |
| L.ALDIEIATYR.K | N | 54.65 | 1163.6187 | 10 | 0.3 | 582.8168 | 2 | 26.29 | 1 | 14374 | BuCaMH04.raw | 3.853E6 | 1 | 1 | 388 | 397 |  |  | PEAKS DB |
| A.LDIEIATYR.K | N | 53.15 | 1092.5815 | 9 | 0.0 | 547.2980 | 2 | 25.30 | 1 | 13477 | BuCaMH04.raw | 9.0958E5 | 1 | 1 | 389 | 397 |  |  | PEAKS DB |
| total 4 peptides |  |  |  |  |  |  |  |  |  |  |  |  |  |  |  |  |  |  |  |
| --- | --- | --- | --- | --- | --- | --- | --- | --- | --- | --- | --- | --- | --- | --- | --- | --- | --- | --- | --- |

XP\_032067160.1

back to list

  

| Protein Coverage
| Supporting Peptides
|

Protein Coverage:

Supporting Peptides:

| Peptide | Uniq | -10lgP | Mass | Length | ppm | m/z | z | RT | Fraction | Scan | Source File | Area BuCaMH04\_B15 | #Feature | #Feature BuCaMH04\_B15 | Start | End | PTM | AScore | Found By |
| --- | --- | --- | --- | --- | --- | --- | --- | --- | --- | --- | --- | --- | --- | --- | --- | --- | --- | --- | --- |
| K.LALDIEIATYR.K | N | 82.54 | 1276.7026 | 11 | 0.6 | 639.3590 | 2 | 34.70 | 1 | 22142 | BuCaMH04.raw | 2.0032E6 | 1 | 1 | 387 | 397 |  |  | PEAKS DB |
| R.NLDLDSIIAEVK.A | N | 79.10 | 1328.7188 | 12 | 0.7 | 665.3671 | 2 | 45.17 | 1 | 31484 | BuCaMH04.raw | 4.9909E6 | 1 | 1 | 258 | 269 |  |  | PEAKS DB |
| L.ALDIEIATYR.K | N | 54.65 | 1163.6187 | 10 | 0.3 | 582.8168 | 2 | 26.29 | 1 | 14374 | BuCaMH04.raw | 3.853E6 | 1 | 1 | 388 | 397 |  |  | PEAKS DB |
| A.LDIEIATYR.K | N | 53.15 | 1092.5815 | 9 | 0.0 | 547.2980 | 2 | 25.30 | 1 | 13477 | BuCaMH04.raw | 9.0958E5 | 1 | 1 | 389 | 397 |  |  | PEAKS DB |
| total 4 peptides |  |  |  |  |  |  |  |  |  |  |  |  |  |  |  |  |  |  |  |
| --- | --- | --- | --- | --- | --- | --- | --- | --- | --- | --- | --- | --- | --- | --- | --- | --- | --- | --- | --- |

ETE64433.1

back to list

  

| Protein Coverage
| Supporting Peptides
|

Protein Coverage:

Supporting Peptides:

| Peptide | Uniq | -10lgP | Mass | Length | ppm | m/z | z | RT | Fraction | Scan | Source File | Area BuCaMH04\_B15 | #Feature | #Feature BuCaMH04\_B15 | Start | End | PTM | AScore | Found By |
| --- | --- | --- | --- | --- | --- | --- | --- | --- | --- | --- | --- | --- | --- | --- | --- | --- | --- | --- | --- |
| K.LALDIEIATYR.K | N | 82.54 | 1276.7026 | 11 | 0.6 | 639.3590 | 2 | 34.70 | 1 | 22142 | BuCaMH04.raw | 2.0032E6 | 1 | 1 | 395 | 405 |  |  | PEAKS DB |
| R.NLDLDSIIAEVK.A | N | 79.10 | 1328.7188 | 12 | 0.7 | 665.3671 | 2 | 45.17 | 1 | 31484 | BuCaMH04.raw | 4.9909E6 | 1 | 1 | 266 | 277 |  |  | PEAKS DB |
| L.ALDIEIATYR.K | N | 54.65 | 1163.6187 | 10 | 0.3 | 582.8168 | 2 | 26.29 | 1 | 14374 | BuCaMH04.raw | 3.853E6 | 1 | 1 | 396 | 405 |  |  | PEAKS DB |
| A.LDIEIATYR.K | N | 53.15 | 1092.5815 | 9 | 0.0 | 547.2980 | 2 | 25.30 | 1 | 13477 | BuCaMH04.raw | 9.0958E5 | 1 | 1 | 397 | 405 |  |  | PEAKS DB |
| total 4 peptides |  |  |  |  |  |  |  |  |  |  |  |  |  |  |  |  |  |  |  |
| --- | --- | --- | --- | --- | --- | --- | --- | --- | --- | --- | --- | --- | --- | --- | --- | --- | --- | --- | --- |

XP\_026573164.1

back to list

  

| Protein Coverage
| Supporting Peptides
|

Protein Coverage:

Supporting Peptides:

| Peptide | Uniq | -10lgP | Mass | Length | ppm | m/z | z | RT | Fraction | Scan | Source File | Area BuCaMH04\_B15 | #Feature | #Feature BuCaMH04\_B15 | Start | End | PTM | AScore | Found By |
| --- | --- | --- | --- | --- | --- | --- | --- | --- | --- | --- | --- | --- | --- | --- | --- | --- | --- | --- | --- |
| K.LALDIEIATYR.K | N | 82.54 | 1276.7026 | 11 | 0.6 | 639.3590 | 2 | 34.70 | 1 | 22142 | BuCaMH04.raw | 2.0032E6 | 1 | 1 | 395 | 405 |  |  | PEAKS DB |
| R.NLDLDSIIAEVK.A | N | 79.10 | 1328.7188 | 12 | 0.7 | 665.3671 | 2 | 45.17 | 1 | 31484 | BuCaMH04.raw | 4.9909E6 | 1 | 1 | 266 | 277 |  |  | PEAKS DB |
| L.ALDIEIATYR.K | N | 54.65 | 1163.6187 | 10 | 0.3 | 582.8168 | 2 | 26.29 | 1 | 14374 | BuCaMH04.raw | 3.853E6 | 1 | 1 | 396 | 405 |  |  | PEAKS DB |
| A.LDIEIATYR.K | N | 53.15 | 1092.5815 | 9 | 0.0 | 547.2980 | 2 | 25.30 | 1 | 13477 | BuCaMH04.raw | 9.0958E5 | 1 | 1 | 397 | 405 |  |  | PEAKS DB |
| total 4 peptides |  |  |  |  |  |  |  |  |  |  |  |  |  |  |  |  |  |  |  |
| --- | --- | --- | --- | --- | --- | --- | --- | --- | --- | --- | --- | --- | --- | --- | --- | --- | --- | --- | --- |

JAG44289.1

back to list

  

| Protein Coverage
| Supporting Peptides
|

Protein Coverage:

Supporting Peptides:

| Peptide | Uniq | -10lgP | Mass | Length | ppm | m/z | z | RT | Fraction | Scan | Source File | Area BuCaMH04\_B15 | #Feature | #Feature BuCaMH04\_B15 | Start | End | PTM | AScore | Found By |
| --- | --- | --- | --- | --- | --- | --- | --- | --- | --- | --- | --- | --- | --- | --- | --- | --- | --- | --- | --- |
| K.LALDIEIATYR.K | N | 82.54 | 1276.7026 | 11 | 0.6 | 639.3590 | 2 | 34.70 | 1 | 22142 | BuCaMH04.raw | 2.0032E6 | 1 | 1 | 395 | 405 |  |  | PEAKS DB |
| R.NLDLDSIIAEVK.A | N | 79.10 | 1328.7188 | 12 | 0.7 | 665.3671 | 2 | 45.17 | 1 | 31484 | BuCaMH04.raw | 4.9909E6 | 1 | 1 | 266 | 277 |  |  | PEAKS DB |
| L.ALDIEIATYR.K | N | 54.65 | 1163.6187 | 10 | 0.3 | 582.8168 | 2 | 26.29 | 1 | 14374 | BuCaMH04.raw | 3.853E6 | 1 | 1 | 396 | 405 |  |  | PEAKS DB |
| A.LDIEIATYR.K | N | 53.15 | 1092.5815 | 9 | 0.0 | 547.2980 | 2 | 25.30 | 1 | 13477 | BuCaMH04.raw | 9.0958E5 | 1 | 1 | 397 | 405 |  |  | PEAKS DB |
| total 4 peptides |  |  |  |  |  |  |  |  |  |  |  |  |  |  |  |  |  |  |  |
| --- | --- | --- | --- | --- | --- | --- | --- | --- | --- | --- | --- | --- | --- | --- | --- | --- | --- | --- | --- |

AFJ50410.1

back to list

  

| Protein Coverage
| Supporting Peptides
|

Protein Coverage:

Supporting Peptides:

| Peptide | Uniq | -10lgP | Mass | Length | ppm | m/z | z | RT | Fraction | Scan | Source File | Area BuCaMH04\_B15 | #Feature | #Feature BuCaMH04\_B15 | Start | End | PTM | AScore | Found By |
| --- | --- | --- | --- | --- | --- | --- | --- | --- | --- | --- | --- | --- | --- | --- | --- | --- | --- | --- | --- |
| K.LALDIEIATYR.K | N | 82.54 | 1276.7026 | 11 | 0.6 | 639.3590 | 2 | 34.70 | 1 | 22142 | BuCaMH04.raw | 2.0032E6 | 1 | 1 | 395 | 405 |  |  | PEAKS DB |
| R.NLDLDSIIAEVK.A | N | 79.10 | 1328.7188 | 12 | 0.7 | 665.3671 | 2 | 45.17 | 1 | 31484 | BuCaMH04.raw | 4.9909E6 | 1 | 1 | 266 | 277 |  |  | PEAKS DB |
| L.ALDIEIATYR.K | N | 54.65 | 1163.6187 | 10 | 0.3 | 582.8168 | 2 | 26.29 | 1 | 14374 | BuCaMH04.raw | 3.853E6 | 1 | 1 | 396 | 405 |  |  | PEAKS DB |
| A.LDIEIATYR.K | N | 53.15 | 1092.5815 | 9 | 0.0 | 547.2980 | 2 | 25.30 | 1 | 13477 | BuCaMH04.raw | 9.0958E5 | 1 | 1 | 397 | 405 |  |  | PEAKS DB |
| total 4 peptides |  |  |  |  |  |  |  |  |  |  |  |  |  |  |  |  |  |  |  |
| --- | --- | --- | --- | --- | --- | --- | --- | --- | --- | --- | --- | --- | --- | --- | --- | --- | --- | --- | --- |

P82885.1

back to list

  

| Protein Coverage
| Supporting Peptides
|

Protein Coverage:

Supporting Peptides:

| Peptide | Uniq | -10lgP | Mass | Length | ppm | m/z | z | RT | Fraction | Scan | Source File | Area BuCaMH04\_B15 | #Feature | #Feature BuCaMH04\_B15 | Start | End | PTM | AScore | Found By |
| --- | --- | --- | --- | --- | --- | --- | --- | --- | --- | --- | --- | --- | --- | --- | --- | --- | --- | --- | --- |
| K.ADVTFDSNTAFESLVVSPDKK.T | Y | 88.03 | 2269.1113 | 21 | 1.0 | 757.3785 | 3 | 32.33 | 1 | 19842 | BuCaMH04.raw | 5.123E7 | 1 | 1 | 9 | 29 |  |  | PEAKS DB |
| R.FDGSPC(+57.02)VLGSPGFR.S | Y | 72.41 | 1494.6925 | 14 | 0.3 | 748.3538 | 2 | 24.05 | 1 | 12286 | BuCaMH04.raw | 2.0811E7 | 1 | 1 | 47 | 60 | Carbamidomethylation | C6:Carbamidomethylation:1000.00 | PEAKS DB |
| K.ADVTFDSNTAFESLVVSPDK.K | Y | 55.95 | 2141.0164 | 20 | 1.3 | 1071.5168 | 2 | 40.73 | 1 | 27496 | BuCaMH04.raw | 1.2567E6 | 1 | 1 | 9 | 28 |  |  | PEAKS DB |
| total 3 peptides |  |  |  |  |  |  |  |  |  |  |  |  |  |  |  |  |  |  |  |
| --- | --- | --- | --- | --- | --- | --- | --- | --- | --- | --- | --- | --- | --- | --- | --- | --- | --- | --- | --- |

T\_80

back to list

  

| Protein Coverage
| Supporting Peptides
|

Protein Coverage:

Supporting Peptides:

| Peptide | Uniq | -10lgP | Mass | Length | ppm | m/z | z | RT | Fraction | Scan | Source File | Area BuCaMH04\_B15 | #Feature | #Feature BuCaMH04\_B15 | Start | End | PTM | AScore | Found By |
| --- | --- | --- | --- | --- | --- | --- | --- | --- | --- | --- | --- | --- | --- | --- | --- | --- | --- | --- | --- |
| K.NFVYGVGANPPDSVIGHYTQIVWYK.S | Y | 95.17 | 2823.4021 | 25 | 0.6 | 942.1418 | 3 | 42.52 | 1 | 29395 | BuCaMH04.raw | 8.1396E7 | 1 | 1 | 116 | 140 |  |  | PEAKS DB |
| R.NMLQMEWNSNAAQNAK.R | Y | 84.14 | 1848.8247 | 16 | 1.3 | 925.4208 | 2 | 24.29 | 1 | 12542 | BuCaMH04.raw | 1.8757E7 | 1 | 1 | 52 | 67 |  |  | PEAKS DB |
| total 2 peptides |  |  |  |  |  |  |  |  |  |  |  |  |  |  |  |  |  |  |  |
| --- | --- | --- | --- | --- | --- | --- | --- | --- | --- | --- | --- | --- | --- | --- | --- | --- | --- | --- | --- |

T\_81

back to list

  

| Protein Coverage
| Supporting Peptides
|

Protein Coverage:

Supporting Peptides:

| Peptide | Uniq | -10lgP | Mass | Length | ppm | m/z | z | RT | Fraction | Scan | Source File | Area BuCaMH04\_B15 | #Feature | #Feature BuCaMH04\_B15 | Start | End | PTM | AScore | Found By |
| --- | --- | --- | --- | --- | --- | --- | --- | --- | --- | --- | --- | --- | --- | --- | --- | --- | --- | --- | --- |
| K.NFVYGVGANPPDSVIGHYTQIVWYK.S | Y | 95.17 | 2823.4021 | 25 | 0.6 | 942.1418 | 3 | 42.52 | 1 | 29395 | BuCaMH04.raw | 8.1396E7 | 1 | 1 | 116 | 140 |  |  | PEAKS DB |
| R.NMLQMEWNSNAAQNAK.R | Y | 84.14 | 1848.8247 | 16 | 1.3 | 925.4208 | 2 | 24.29 | 1 | 12542 | BuCaMH04.raw | 1.8757E7 | 1 | 1 | 52 | 67 |  |  | PEAKS DB |
| total 2 peptides |  |  |  |  |  |  |  |  |  |  |  |  |  |  |  |  |  |  |  |
| --- | --- | --- | --- | --- | --- | --- | --- | --- | --- | --- | --- | --- | --- | --- | --- | --- | --- | --- | --- |

AXL96635.1

back to list

  

| Protein Coverage
| Supporting Peptides
|

Protein Coverage:

Supporting Peptides:

| Peptide | Uniq | -10lgP | Mass | Length | ppm | m/z | z | RT | Fraction | Scan | Source File | Area BuCaMH04\_B15 | #Feature | #Feature BuCaMH04\_B15 | Start | End | PTM | AScore | Found By |
| --- | --- | --- | --- | --- | --- | --- | --- | --- | --- | --- | --- | --- | --- | --- | --- | --- | --- | --- | --- |
| G.VTLDLFGEWR.E | N | 73.86 | 1234.6346 | 10 | 0.6 | 618.3250 | 2 | 45.55 | 1 | 31857 | BuCaMH04.raw | 9.3115E6 | 1 | 1 | 272 | 281 |  |  | PEAKS DB |
| V.TLDLFGEWR.E | N | 65.70 | 1135.5662 | 9 | 0.5 | 568.7906 | 2 | 42.31 | 1 | 29257 | BuCaMH04.raw | 4.2198E8 | 1 | 1 | 273 | 281 |  |  | PEAKS DB |
| T.LDLFGEWR.E | N | 57.32 | 1034.5186 | 8 | 0.6 | 518.2668 | 2 | 41.26 | 1 | 27863 | BuCaMH04.raw | 4.6562E7 | 1 | 1 | 274 | 281 |  |  | PEAKS DB |
| S.GVTLDLFGEWR.E | Y | 56.48 | 1291.6560 | 11 | 0.1 | 646.8353 | 2 | 46.32 | 1 | 32629 | BuCaMH04.raw | 1.8071E6 | 1 | 1 | 271 | 281 |  |  | PEAKS DB |
| total 4 peptides |  |  |  |  |  |  |  |  |  |  |  |  |  |  |  |  |  |  |  |
| --- | --- | --- | --- | --- | --- | --- | --- | --- | --- | --- | --- | --- | --- | --- | --- | --- | --- | --- | --- |

AXL96667.1

back to list

  

| Protein Coverage
| Supporting Peptides
|

Protein Coverage:

Supporting Peptides:

| Peptide | Uniq | -10lgP | Mass | Length | ppm | m/z | z | RT | Fraction | Scan | Source File | Area BuCaMH04\_B15 | #Feature | #Feature BuCaMH04\_B15 | Start | End | PTM | AScore | Found By |
| --- | --- | --- | --- | --- | --- | --- | --- | --- | --- | --- | --- | --- | --- | --- | --- | --- | --- | --- | --- |
| G.VTLDLFGEWR.E | N | 73.86 | 1234.6346 | 10 | 0.6 | 618.3250 | 2 | 45.55 | 1 | 31857 | BuCaMH04.raw | 9.3115E6 | 1 | 1 | 272 | 281 |  |  | PEAKS DB |
| V.TLDLFGEWR.E | N | 65.70 | 1135.5662 | 9 | 0.5 | 568.7906 | 2 | 42.31 | 1 | 29257 | BuCaMH04.raw | 4.2198E8 | 1 | 1 | 273 | 281 |  |  | PEAKS DB |
| T.LDLFGEWR.E | N | 57.32 | 1034.5186 | 8 | 0.6 | 518.2668 | 2 | 41.26 | 1 | 27863 | BuCaMH04.raw | 4.6562E7 | 1 | 1 | 274 | 281 |  |  | PEAKS DB |
| S.GVTLDLFGEWR.E | Y | 56.48 | 1291.6560 | 11 | 0.1 | 646.8353 | 2 | 46.32 | 1 | 32629 | BuCaMH04.raw | 1.8071E6 | 1 | 1 | 271 | 281 |  |  | PEAKS DB |
| total 4 peptides |  |  |  |  |  |  |  |  |  |  |  |  |  |  |  |  |  |  |  |
| --- | --- | --- | --- | --- | --- | --- | --- | --- | --- | --- | --- | --- | --- | --- | --- | --- | --- | --- | --- |

AXL96646.1

back to list

  

| Protein Coverage
| Supporting Peptides
|

Protein Coverage:

Supporting Peptides:

| Peptide | Uniq | -10lgP | Mass | Length | ppm | m/z | z | RT | Fraction | Scan | Source File | Area BuCaMH04\_B15 | #Feature | #Feature BuCaMH04\_B15 | Start | End | PTM | AScore | Found By |
| --- | --- | --- | --- | --- | --- | --- | --- | --- | --- | --- | --- | --- | --- | --- | --- | --- | --- | --- | --- |
| G.VTLDLFGEWR.E | N | 73.86 | 1234.6346 | 10 | 0.6 | 618.3250 | 2 | 45.55 | 1 | 31857 | BuCaMH04.raw | 9.3115E6 | 1 | 1 | 272 | 281 |  |  | PEAKS DB |
| V.TLDLFGEWR.E | N | 65.70 | 1135.5662 | 9 | 0.5 | 568.7906 | 2 | 42.31 | 1 | 29257 | BuCaMH04.raw | 4.2198E8 | 1 | 1 | 273 | 281 |  |  | PEAKS DB |
| T.LDLFGEWR.E | N | 57.32 | 1034.5186 | 8 | 0.6 | 518.2668 | 2 | 41.26 | 1 | 27863 | BuCaMH04.raw | 4.6562E7 | 1 | 1 | 274 | 281 |  |  | PEAKS DB |
| S.GVTLDLFGEWR.E | Y | 56.48 | 1291.6560 | 11 | 0.1 | 646.8353 | 2 | 46.32 | 1 | 32629 | BuCaMH04.raw | 1.8071E6 | 1 | 1 | 271 | 281 |  |  | PEAKS DB |
| total 4 peptides |  |  |  |  |  |  |  |  |  |  |  |  |  |  |  |  |  |  |  |
| --- | --- | --- | --- | --- | --- | --- | --- | --- | --- | --- | --- | --- | --- | --- | --- | --- | --- | --- | --- |

AXL96640.1

back to list

  

| Protein Coverage
| Supporting Peptides
|

Protein Coverage:

Supporting Peptides:

| Peptide | Uniq | -10lgP | Mass | Length | ppm | m/z | z | RT | Fraction | Scan | Source File | Area BuCaMH04\_B15 | #Feature | #Feature BuCaMH04\_B15 | Start | End | PTM | AScore | Found By |
| --- | --- | --- | --- | --- | --- | --- | --- | --- | --- | --- | --- | --- | --- | --- | --- | --- | --- | --- | --- |
| G.VTLDLFGEWR.E | N | 73.86 | 1234.6346 | 10 | 0.6 | 618.3250 | 2 | 45.55 | 1 | 31857 | BuCaMH04.raw | 9.3115E6 | 1 | 1 | 272 | 281 |  |  | PEAKS DB |
| V.TLDLFGEWR.E | N | 65.70 | 1135.5662 | 9 | 0.5 | 568.7906 | 2 | 42.31 | 1 | 29257 | BuCaMH04.raw | 4.2198E8 | 1 | 1 | 273 | 281 |  |  | PEAKS DB |
| T.LDLFGEWR.E | N | 57.32 | 1034.5186 | 8 | 0.6 | 518.2668 | 2 | 41.26 | 1 | 27863 | BuCaMH04.raw | 4.6562E7 | 1 | 1 | 274 | 281 |  |  | PEAKS DB |
| S.GVTLDLFGEWR.E | Y | 56.48 | 1291.6560 | 11 | 0.1 | 646.8353 | 2 | 46.32 | 1 | 32629 | BuCaMH04.raw | 1.8071E6 | 1 | 1 | 271 | 281 |  |  | PEAKS DB |
| total 4 peptides |  |  |  |  |  |  |  |  |  |  |  |  |  |  |  |  |  |  |  |
| --- | --- | --- | --- | --- | --- | --- | --- | --- | --- | --- | --- | --- | --- | --- | --- | --- | --- | --- | --- |

AXL96666.1

back to list

  

| Protein Coverage
| Supporting Peptides
|

Protein Coverage:

Supporting Peptides:

| Peptide | Uniq | -10lgP | Mass | Length | ppm | m/z | z | RT | Fraction | Scan | Source File | Area BuCaMH04\_B15 | #Feature | #Feature BuCaMH04\_B15 | Start | End | PTM | AScore | Found By |
| --- | --- | --- | --- | --- | --- | --- | --- | --- | --- | --- | --- | --- | --- | --- | --- | --- | --- | --- | --- |
| G.VTLDLFGEWR.E | N | 73.86 | 1234.6346 | 10 | 0.6 | 618.3250 | 2 | 45.55 | 1 | 31857 | BuCaMH04.raw | 9.3115E6 | 1 | 1 | 272 | 281 |  |  | PEAKS DB |
| V.TLDLFGEWR.E | N | 65.70 | 1135.5662 | 9 | 0.5 | 568.7906 | 2 | 42.31 | 1 | 29257 | BuCaMH04.raw | 4.2198E8 | 1 | 1 | 273 | 281 |  |  | PEAKS DB |
| T.LDLFGEWR.E | N | 57.32 | 1034.5186 | 8 | 0.6 | 518.2668 | 2 | 41.26 | 1 | 27863 | BuCaMH04.raw | 4.6562E7 | 1 | 1 | 274 | 281 |  |  | PEAKS DB |
| S.GVTLDLFGEWR.E | Y | 56.48 | 1291.6560 | 11 | 0.1 | 646.8353 | 2 | 46.32 | 1 | 32629 | BuCaMH04.raw | 1.8071E6 | 1 | 1 | 271 | 281 |  |  | PEAKS DB |
| total 4 peptides |  |  |  |  |  |  |  |  |  |  |  |  |  |  |  |  |  |  |  |
| --- | --- | --- | --- | --- | --- | --- | --- | --- | --- | --- | --- | --- | --- | --- | --- | --- | --- | --- | --- |

AXL96626.1

back to list

  

| Protein Coverage
| Supporting Peptides
|

Protein Coverage:

Supporting Peptides:

| Peptide | Uniq | -10lgP | Mass | Length | ppm | m/z | z | RT | Fraction | Scan | Source File | Area BuCaMH04\_B15 | #Feature | #Feature BuCaMH04\_B15 | Start | End | PTM | AScore | Found By |
| --- | --- | --- | --- | --- | --- | --- | --- | --- | --- | --- | --- | --- | --- | --- | --- | --- | --- | --- | --- |
| G.VTLDLFGEWR.E | N | 73.86 | 1234.6346 | 10 | 0.6 | 618.3250 | 2 | 45.55 | 1 | 31857 | BuCaMH04.raw | 9.3115E6 | 1 | 1 | 309 | 318 |  |  | PEAKS DB |
| V.TLDLFGEWR.E | N | 65.70 | 1135.5662 | 9 | 0.5 | 568.7906 | 2 | 42.31 | 1 | 29257 | BuCaMH04.raw | 4.2198E8 | 1 | 1 | 310 | 318 |  |  | PEAKS DB |
| T.LDLFGEWR.E | N | 57.32 | 1034.5186 | 8 | 0.6 | 518.2668 | 2 | 41.26 | 1 | 27863 | BuCaMH04.raw | 4.6562E7 | 1 | 1 | 311 | 318 |  |  | PEAKS DB |
| S.GVTLDLFGEWR.E | Y | 56.48 | 1291.6560 | 11 | 0.1 | 646.8353 | 2 | 46.32 | 1 | 32629 | BuCaMH04.raw | 1.8071E6 | 1 | 1 | 308 | 318 |  |  | PEAKS DB |
| total 4 peptides |  |  |  |  |  |  |  |  |  |  |  |  |  |  |  |  |  |  |  |
| --- | --- | --- | --- | --- | --- | --- | --- | --- | --- | --- | --- | --- | --- | --- | --- | --- | --- | --- | --- |

AXL96631.1

back to list

  

| Protein Coverage
| Supporting Peptides
|

Protein Coverage:

Supporting Peptides:

| Peptide | Uniq | -10lgP | Mass | Length | ppm | m/z | z | RT | Fraction | Scan | Source File | Area BuCaMH04\_B15 | #Feature | #Feature BuCaMH04\_B15 | Start | End | PTM | AScore | Found By |
| --- | --- | --- | --- | --- | --- | --- | --- | --- | --- | --- | --- | --- | --- | --- | --- | --- | --- | --- | --- |
| G.VTLDLFGEWR.E | N | 73.86 | 1234.6346 | 10 | 0.6 | 618.3250 | 2 | 45.55 | 1 | 31857 | BuCaMH04.raw | 9.3115E6 | 1 | 1 | 272 | 281 |  |  | PEAKS DB |
| V.TLDLFGEWR.E | N | 65.70 | 1135.5662 | 9 | 0.5 | 568.7906 | 2 | 42.31 | 1 | 29257 | BuCaMH04.raw | 4.2198E8 | 1 | 1 | 273 | 281 |  |  | PEAKS DB |
| T.LDLFGEWR.E | N | 57.32 | 1034.5186 | 8 | 0.6 | 518.2668 | 2 | 41.26 | 1 | 27863 | BuCaMH04.raw | 4.6562E7 | 1 | 1 | 274 | 281 |  |  | PEAKS DB |
| S.GVTLDLFGEWR.E | Y | 56.48 | 1291.6560 | 11 | 0.1 | 646.8353 | 2 | 46.32 | 1 | 32629 | BuCaMH04.raw | 1.8071E6 | 1 | 1 | 271 | 281 |  |  | PEAKS DB |
| total 4 peptides |  |  |  |  |  |  |  |  |  |  |  |  |  |  |  |  |  |  |  |
| --- | --- | --- | --- | --- | --- | --- | --- | --- | --- | --- | --- | --- | --- | --- | --- | --- | --- | --- | --- |

AXL96650.1

back to list

  

| Protein Coverage
| Supporting Peptides
|

Protein Coverage:

Supporting Peptides:

| Peptide | Uniq | -10lgP | Mass | Length | ppm | m/z | z | RT | Fraction | Scan | Source File | Area BuCaMH04\_B15 | #Feature | #Feature BuCaMH04\_B15 | Start | End | PTM | AScore | Found By |
| --- | --- | --- | --- | --- | --- | --- | --- | --- | --- | --- | --- | --- | --- | --- | --- | --- | --- | --- | --- |
| G.VTLDLFGEWR.E | N | 73.86 | 1234.6346 | 10 | 0.6 | 618.3250 | 2 | 45.55 | 1 | 31857 | BuCaMH04.raw | 9.3115E6 | 1 | 1 | 309 | 318 |  |  | PEAKS DB |
| V.TLDLFGEWR.E | N | 65.70 | 1135.5662 | 9 | 0.5 | 568.7906 | 2 | 42.31 | 1 | 29257 | BuCaMH04.raw | 4.2198E8 | 1 | 1 | 310 | 318 |  |  | PEAKS DB |
| T.LDLFGEWR.E | N | 57.32 | 1034.5186 | 8 | 0.6 | 518.2668 | 2 | 41.26 | 1 | 27863 | BuCaMH04.raw | 4.6562E7 | 1 | 1 | 311 | 318 |  |  | PEAKS DB |
| S.GVTLDLFGEWR.E | Y | 56.48 | 1291.6560 | 11 | 0.1 | 646.8353 | 2 | 46.32 | 1 | 32629 | BuCaMH04.raw | 1.8071E6 | 1 | 1 | 308 | 318 |  |  | PEAKS DB |
| total 4 peptides |  |  |  |  |  |  |  |  |  |  |  |  |  |  |  |  |  |  |  |
| --- | --- | --- | --- | --- | --- | --- | --- | --- | --- | --- | --- | --- | --- | --- | --- | --- | --- | --- | --- |

AXL96645.1

back to list

  

| Protein Coverage
| Supporting Peptides
|

Protein Coverage:

Supporting Peptides:

| Peptide | Uniq | -10lgP | Mass | Length | ppm | m/z | z | RT | Fraction | Scan | Source File | Area BuCaMH04\_B15 | #Feature | #Feature BuCaMH04\_B15 | Start | End | PTM | AScore | Found By |
| --- | --- | --- | --- | --- | --- | --- | --- | --- | --- | --- | --- | --- | --- | --- | --- | --- | --- | --- | --- |
| G.VTLDLFGEWR.E | N | 73.86 | 1234.6346 | 10 | 0.6 | 618.3250 | 2 | 45.55 | 1 | 31857 | BuCaMH04.raw | 9.3115E6 | 1 | 1 | 302 | 311 |  |  | PEAKS DB |
| V.TLDLFGEWR.E | N | 65.70 | 1135.5662 | 9 | 0.5 | 568.7906 | 2 | 42.31 | 1 | 29257 | BuCaMH04.raw | 4.2198E8 | 1 | 1 | 303 | 311 |  |  | PEAKS DB |
| T.LDLFGEWR.E | N | 57.32 | 1034.5186 | 8 | 0.6 | 518.2668 | 2 | 41.26 | 1 | 27863 | BuCaMH04.raw | 4.6562E7 | 1 | 1 | 304 | 311 |  |  | PEAKS DB |
| S.GVTLDLFGEWR.E | Y | 56.48 | 1291.6560 | 11 | 0.1 | 646.8353 | 2 | 46.32 | 1 | 32629 | BuCaMH04.raw | 1.8071E6 | 1 | 1 | 301 | 311 |  |  | PEAKS DB |
| total 4 peptides |  |  |  |  |  |  |  |  |  |  |  |  |  |  |  |  |  |  |  |
| --- | --- | --- | --- | --- | --- | --- | --- | --- | --- | --- | --- | --- | --- | --- | --- | --- | --- | --- | --- |

AXL96653.1

back to list

  

| Protein Coverage
| Supporting Peptides
|

Protein Coverage:

Supporting Peptides:

| Peptide | Uniq | -10lgP | Mass | Length | ppm | m/z | z | RT | Fraction | Scan | Source File | Area BuCaMH04\_B15 | #Feature | #Feature BuCaMH04\_B15 | Start | End | PTM | AScore | Found By |
| --- | --- | --- | --- | --- | --- | --- | --- | --- | --- | --- | --- | --- | --- | --- | --- | --- | --- | --- | --- |
| G.VTLDLFGEWR.E | N | 73.86 | 1234.6346 | 10 | 0.6 | 618.3250 | 2 | 45.55 | 1 | 31857 | BuCaMH04.raw | 9.3115E6 | 1 | 1 | 272 | 281 |  |  | PEAKS DB |
| V.TLDLFGEWR.E | N | 65.70 | 1135.5662 | 9 | 0.5 | 568.7906 | 2 | 42.31 | 1 | 29257 | BuCaMH04.raw | 4.2198E8 | 1 | 1 | 273 | 281 |  |  | PEAKS DB |
| T.LDLFGEWR.E | N | 57.32 | 1034.5186 | 8 | 0.6 | 518.2668 | 2 | 41.26 | 1 | 27863 | BuCaMH04.raw | 4.6562E7 | 1 | 1 | 274 | 281 |  |  | PEAKS DB |
| S.GVTLDLFGEWR.E | Y | 56.48 | 1291.6560 | 11 | 0.1 | 646.8353 | 2 | 46.32 | 1 | 32629 | BuCaMH04.raw | 1.8071E6 | 1 | 1 | 271 | 281 |  |  | PEAKS DB |
| total 4 peptides |  |  |  |  |  |  |  |  |  |  |  |  |  |  |  |  |  |  |  |
| --- | --- | --- | --- | --- | --- | --- | --- | --- | --- | --- | --- | --- | --- | --- | --- | --- | --- | --- | --- |

AXL96648.1

back to list

  

| Protein Coverage
| Supporting Peptides
|

Protein Coverage:

Supporting Peptides:

| Peptide | Uniq | -10lgP | Mass | Length | ppm | m/z | z | RT | Fraction | Scan | Source File | Area BuCaMH04\_B15 | #Feature | #Feature BuCaMH04\_B15 | Start | End | PTM | AScore | Found By |
| --- | --- | --- | --- | --- | --- | --- | --- | --- | --- | --- | --- | --- | --- | --- | --- | --- | --- | --- | --- |
| G.VTLDLFGEWR.E | N | 73.86 | 1234.6346 | 10 | 0.6 | 618.3250 | 2 | 45.55 | 1 | 31857 | BuCaMH04.raw | 9.3115E6 | 1 | 1 | 272 | 281 |  |  | PEAKS DB |
| V.TLDLFGEWR.E | N | 65.70 | 1135.5662 | 9 | 0.5 | 568.7906 | 2 | 42.31 | 1 | 29257 | BuCaMH04.raw | 4.2198E8 | 1 | 1 | 273 | 281 |  |  | PEAKS DB |
| T.LDLFGEWR.E | N | 57.32 | 1034.5186 | 8 | 0.6 | 518.2668 | 2 | 41.26 | 1 | 27863 | BuCaMH04.raw | 4.6562E7 | 1 | 1 | 274 | 281 |  |  | PEAKS DB |
| S.GVTLDLFGEWR.E | Y | 56.48 | 1291.6560 | 11 | 0.1 | 646.8353 | 2 | 46.32 | 1 | 32629 | BuCaMH04.raw | 1.8071E6 | 1 | 1 | 271 | 281 |  |  | PEAKS DB |
| total 4 peptides |  |  |  |  |  |  |  |  |  |  |  |  |  |  |  |  |  |  |  |
| --- | --- | --- | --- | --- | --- | --- | --- | --- | --- | --- | --- | --- | --- | --- | --- | --- | --- | --- | --- |

AXL96655.1

back to list

  

| Protein Coverage
| Supporting Peptides
|

Protein Coverage:

Supporting Peptides:

| Peptide | Uniq | -10lgP | Mass | Length | ppm | m/z | z | RT | Fraction | Scan | Source File | Area BuCaMH04\_B15 | #Feature | #Feature BuCaMH04\_B15 | Start | End | PTM | AScore | Found By |
| --- | --- | --- | --- | --- | --- | --- | --- | --- | --- | --- | --- | --- | --- | --- | --- | --- | --- | --- | --- |
| G.VTLDLFGEWR.E | N | 73.86 | 1234.6346 | 10 | 0.6 | 618.3250 | 2 | 45.55 | 1 | 31857 | BuCaMH04.raw | 9.3115E6 | 1 | 1 | 272 | 281 |  |  | PEAKS DB |
| V.TLDLFGEWR.E | N | 65.70 | 1135.5662 | 9 | 0.5 | 568.7906 | 2 | 42.31 | 1 | 29257 | BuCaMH04.raw | 4.2198E8 | 1 | 1 | 273 | 281 |  |  | PEAKS DB |
| T.LDLFGEWR.E | N | 57.32 | 1034.5186 | 8 | 0.6 | 518.2668 | 2 | 41.26 | 1 | 27863 | BuCaMH04.raw | 4.6562E7 | 1 | 1 | 274 | 281 |  |  | PEAKS DB |
| S.GVTLDLFGEWR.E | Y | 56.48 | 1291.6560 | 11 | 0.1 | 646.8353 | 2 | 46.32 | 1 | 32629 | BuCaMH04.raw | 1.8071E6 | 1 | 1 | 271 | 281 |  |  | PEAKS DB |
| total 4 peptides |  |  |  |  |  |  |  |  |  |  |  |  |  |  |  |  |  |  |  |
| --- | --- | --- | --- | --- | --- | --- | --- | --- | --- | --- | --- | --- | --- | --- | --- | --- | --- | --- | --- |

AXL96638.1

back to list

  

| Protein Coverage
| Supporting Peptides
|

Protein Coverage:

Supporting Peptides:

| Peptide | Uniq | -10lgP | Mass | Length | ppm | m/z | z | RT | Fraction | Scan | Source File | Area BuCaMH04\_B15 | #Feature | #Feature BuCaMH04\_B15 | Start | End | PTM | AScore | Found By |
| --- | --- | --- | --- | --- | --- | --- | --- | --- | --- | --- | --- | --- | --- | --- | --- | --- | --- | --- | --- |
| G.VTLDLFGEWR.E | N | 73.86 | 1234.6346 | 10 | 0.6 | 618.3250 | 2 | 45.55 | 1 | 31857 | BuCaMH04.raw | 9.3115E6 | 1 | 1 | 272 | 281 |  |  | PEAKS DB |
| V.TLDLFGEWR.E | N | 65.70 | 1135.5662 | 9 | 0.5 | 568.7906 | 2 | 42.31 | 1 | 29257 | BuCaMH04.raw | 4.2198E8 | 1 | 1 | 273 | 281 |  |  | PEAKS DB |
| T.LDLFGEWR.E | N | 57.32 | 1034.5186 | 8 | 0.6 | 518.2668 | 2 | 41.26 | 1 | 27863 | BuCaMH04.raw | 4.6562E7 | 1 | 1 | 274 | 281 |  |  | PEAKS DB |
| S.GVTLDLFGEWR.E | Y | 56.48 | 1291.6560 | 11 | 0.1 | 646.8353 | 2 | 46.32 | 1 | 32629 | BuCaMH04.raw | 1.8071E6 | 1 | 1 | 271 | 281 |  |  | PEAKS DB |
| total 4 peptides |  |  |  |  |  |  |  |  |  |  |  |  |  |  |  |  |  |  |  |
| --- | --- | --- | --- | --- | --- | --- | --- | --- | --- | --- | --- | --- | --- | --- | --- | --- | --- | --- | --- |

AXL96643.1

back to list

  

| Protein Coverage
| Supporting Peptides
|

Protein Coverage:

Supporting Peptides:

| Peptide | Uniq | -10lgP | Mass | Length | ppm | m/z | z | RT | Fraction | Scan | Source File | Area BuCaMH04\_B15 | #Feature | #Feature BuCaMH04\_B15 | Start | End | PTM | AScore | Found By |
| --- | --- | --- | --- | --- | --- | --- | --- | --- | --- | --- | --- | --- | --- | --- | --- | --- | --- | --- | --- |
| G.VTLDLFGEWR.E | N | 73.86 | 1234.6346 | 10 | 0.6 | 618.3250 | 2 | 45.55 | 1 | 31857 | BuCaMH04.raw | 9.3115E6 | 1 | 1 | 272 | 281 |  |  | PEAKS DB |
| V.TLDLFGEWR.E | N | 65.70 | 1135.5662 | 9 | 0.5 | 568.7906 | 2 | 42.31 | 1 | 29257 | BuCaMH04.raw | 4.2198E8 | 1 | 1 | 273 | 281 |  |  | PEAKS DB |
| T.LDLFGEWR.E | N | 57.32 | 1034.5186 | 8 | 0.6 | 518.2668 | 2 | 41.26 | 1 | 27863 | BuCaMH04.raw | 4.6562E7 | 1 | 1 | 274 | 281 |  |  | PEAKS DB |
| S.GVTLDLFGEWR.E | Y | 56.48 | 1291.6560 | 11 | 0.1 | 646.8353 | 2 | 46.32 | 1 | 32629 | BuCaMH04.raw | 1.8071E6 | 1 | 1 | 271 | 281 |  |  | PEAKS DB |
| total 4 peptides |  |  |  |  |  |  |  |  |  |  |  |  |  |  |  |  |  |  |  |
| --- | --- | --- | --- | --- | --- | --- | --- | --- | --- | --- | --- | --- | --- | --- | --- | --- | --- | --- | --- |

AXL96644.1

back to list

  

| Protein Coverage
| Supporting Peptides
|

Protein Coverage:

Supporting Peptides:

| Peptide | Uniq | -10lgP | Mass | Length | ppm | m/z | z | RT | Fraction | Scan | Source File | Area BuCaMH04\_B15 | #Feature | #Feature BuCaMH04\_B15 | Start | End | PTM | AScore | Found By |
| --- | --- | --- | --- | --- | --- | --- | --- | --- | --- | --- | --- | --- | --- | --- | --- | --- | --- | --- | --- |
| G.VTLDLFGEWR.E | N | 73.86 | 1234.6346 | 10 | 0.6 | 618.3250 | 2 | 45.55 | 1 | 31857 | BuCaMH04.raw | 9.3115E6 | 1 | 1 | 272 | 281 |  |  | PEAKS DB |
| V.TLDLFGEWR.E | N | 65.70 | 1135.5662 | 9 | 0.5 | 568.7906 | 2 | 42.31 | 1 | 29257 | BuCaMH04.raw | 4.2198E8 | 1 | 1 | 273 | 281 |  |  | PEAKS DB |
| T.LDLFGEWR.E | N | 57.32 | 1034.5186 | 8 | 0.6 | 518.2668 | 2 | 41.26 | 1 | 27863 | BuCaMH04.raw | 4.6562E7 | 1 | 1 | 274 | 281 |  |  | PEAKS DB |
| S.GVTLDLFGEWR.E | Y | 56.48 | 1291.6560 | 11 | 0.1 | 646.8353 | 2 | 46.32 | 1 | 32629 | BuCaMH04.raw | 1.8071E6 | 1 | 1 | 271 | 281 |  |  | PEAKS DB |
| total 4 peptides |  |  |  |  |  |  |  |  |  |  |  |  |  |  |  |  |  |  |  |
| --- | --- | --- | --- | --- | --- | --- | --- | --- | --- | --- | --- | --- | --- | --- | --- | --- | --- | --- | --- |

AXL96633.1

back to list

  

| Protein Coverage
| Supporting Peptides
|

Protein Coverage:

Supporting Peptides:

| Peptide | Uniq | -10lgP | Mass | Length | ppm | m/z | z | RT | Fraction | Scan | Source File | Area BuCaMH04\_B15 | #Feature | #Feature BuCaMH04\_B15 | Start | End | PTM | AScore | Found By |
| --- | --- | --- | --- | --- | --- | --- | --- | --- | --- | --- | --- | --- | --- | --- | --- | --- | --- | --- | --- |
| G.VTLDLFGEWR.E | N | 73.86 | 1234.6346 | 10 | 0.6 | 618.3250 | 2 | 45.55 | 1 | 31857 | BuCaMH04.raw | 9.3115E6 | 1 | 1 | 272 | 281 |  |  | PEAKS DB |
| V.TLDLFGEWR.E | N | 65.70 | 1135.5662 | 9 | 0.5 | 568.7906 | 2 | 42.31 | 1 | 29257 | BuCaMH04.raw | 4.2198E8 | 1 | 1 | 273 | 281 |  |  | PEAKS DB |
| T.LDLFGEWR.E | N | 57.32 | 1034.5186 | 8 | 0.6 | 518.2668 | 2 | 41.26 | 1 | 27863 | BuCaMH04.raw | 4.6562E7 | 1 | 1 | 274 | 281 |  |  | PEAKS DB |
| S.GVTLDLFGEWR.E | Y | 56.48 | 1291.6560 | 11 | 0.1 | 646.8353 | 2 | 46.32 | 1 | 32629 | BuCaMH04.raw | 1.8071E6 | 1 | 1 | 271 | 281 |  |  | PEAKS DB |
| total 4 peptides |  |  |  |  |  |  |  |  |  |  |  |  |  |  |  |  |  |  |  |
| --- | --- | --- | --- | --- | --- | --- | --- | --- | --- | --- | --- | --- | --- | --- | --- | --- | --- | --- | --- |

XP\_032064947.1

back to list

  

| Protein Coverage
| Supporting Peptides
|

Protein Coverage:

Supporting Peptides:

| Peptide | Uniq | -10lgP | Mass | Length | ppm | m/z | z | RT | Fraction | Scan | Source File | Area BuCaMH04\_B15 | #Feature | #Feature BuCaMH04\_B15 | Start | End | PTM | AScore | Found By |
| --- | --- | --- | --- | --- | --- | --- | --- | --- | --- | --- | --- | --- | --- | --- | --- | --- | --- | --- | --- |
| K.HVVVVGAGMAGLSAAYVLAGAGHK.V | N | 106.63 | 2234.1992 | 24 | 0.7 | 745.7409 | 3 | 30.19 | 1 | 18119 | BuCaMH04.raw | 2.655E7 | 1 | 1 | 53 | 76 |  |  | PEAKS DB |
| R.ISFEPPLPPK.K | N | 82.29 | 1123.6277 | 10 | 0.5 | 562.8214 | 2 | 22.87 | 1 | 11505 | BuCaMH04.raw | 1.674E7 | 4 | 4 | 320 | 329 |  |  | PEAKS DB |
| total 2 peptides |  |  |  |  |  |  |  |  |  |  |  |  |  |  |  |  |  |  |  |
| --- | --- | --- | --- | --- | --- | --- | --- | --- | --- | --- | --- | --- | --- | --- | --- | --- | --- | --- | --- |

A8QL56.1

back to list

  

| Protein Coverage
| Supporting Peptides
|

Protein Coverage:

Supporting Peptides:

| Peptide | Uniq | -10lgP | Mass | Length | ppm | m/z | z | RT | Fraction | Scan | Source File | Area BuCaMH04\_B15 | #Feature | #Feature BuCaMH04\_B15 | Start | End | PTM | AScore | Found By |
| --- | --- | --- | --- | --- | --- | --- | --- | --- | --- | --- | --- | --- | --- | --- | --- | --- | --- | --- | --- |
| G.FPC(+57.02)AQLLEPGVYTK.V | N | 103.82 | 1621.8174 | 14 | 0.9 | 811.9167 | 2 | 30.60 | 1 | 18503 | BuCaMH04.raw | 7.1547E7 | 1 | 1 | 222 | 235 | Carbamidomethylation | C3:Carbamidomethylation:1000.00 | PEAKS DB |
| G.GFPC(+57.02)AQLLEPGVYTK.V | Y | 80.47 | 1678.8389 | 15 | 0.1 | 840.4268 | 2 | 32.23 | 1 | 19810 | BuCaMH04.raw | 1.6412E7 | 1 | 1 | 221 | 235 | Carbamidomethylation | C4:Carbamidomethylation:1000.00 | PEAKS DB |
| total 2 peptides |  |  |  |  |  |  |  |  |  |  |  |  |  |  |  |  |  |  |  |
| --- | --- | --- | --- | --- | --- | --- | --- | --- | --- | --- | --- | --- | --- | --- | --- | --- | --- | --- | --- |

ABN72544.1

back to list

  

| Protein Coverage
| Supporting Peptides
|

Protein Coverage:

Supporting Peptides:

| Peptide | Uniq | -10lgP | Mass | Length | ppm | m/z | z | RT | Fraction | Scan | Source File | Area BuCaMH04\_B15 | #Feature | #Feature BuCaMH04\_B15 | Start | End | PTM | AScore | Found By |
| --- | --- | --- | --- | --- | --- | --- | --- | --- | --- | --- | --- | --- | --- | --- | --- | --- | --- | --- | --- |
| G.FPC(+57.02)AQLLEPGVYTK.V | N | 103.82 | 1621.8174 | 14 | 0.9 | 811.9167 | 2 | 30.60 | 1 | 18503 | BuCaMH04.raw | 7.1547E7 | 1 | 1 | 222 | 235 | Carbamidomethylation | C3:Carbamidomethylation:1000.00 | PEAKS DB |
| G.GFPC(+57.02)AQLLEPGVYTK.V | Y | 80.47 | 1678.8389 | 15 | 0.1 | 840.4268 | 2 | 32.23 | 1 | 19810 | BuCaMH04.raw | 1.6412E7 | 1 | 1 | 221 | 235 | Carbamidomethylation | C4:Carbamidomethylation:1000.00 | PEAKS DB |
| total 2 peptides |  |  |  |  |  |  |  |  |  |  |  |  |  |  |  |  |  |  |  |
| --- | --- | --- | --- | --- | --- | --- | --- | --- | --- | --- | --- | --- | --- | --- | --- | --- | --- | --- | --- |

ABI74694.1

back to list

  

| Protein Coverage
| Supporting Peptides
|

Protein Coverage:

Supporting Peptides:

| Peptide | Uniq | -10lgP | Mass | Length | ppm | m/z | z | RT | Fraction | Scan | Source File | Area BuCaMH04\_B15 | #Feature | #Feature BuCaMH04\_B15 | Start | End | PTM | AScore | Found By |
| --- | --- | --- | --- | --- | --- | --- | --- | --- | --- | --- | --- | --- | --- | --- | --- | --- | --- | --- | --- |
| G.FPC(+57.02)AQILEPGVYTK.V | N | 103.82 | 1621.8174 | 14 | 0.9 | 811.9167 | 2 | 30.60 | 1 | 18503 | BuCaMH04.raw | 7.1547E7 | 1 | 1 | 223 | 236 | Carbamidomethylation | C3:Carbamidomethylation:1000.00 | PEAKS DB |
| G.GFPC(+57.02)AQILEPGVYTK.V | Y | 80.47 | 1678.8389 | 15 | 0.1 | 840.4268 | 2 | 32.23 | 1 | 19810 | BuCaMH04.raw | 1.6412E7 | 1 | 1 | 222 | 236 | Carbamidomethylation | C4:Carbamidomethylation:1000.00 | PEAKS DB |
| total 2 peptides |  |  |  |  |  |  |  |  |  |  |  |  |  |  |  |  |  |  |  |
| --- | --- | --- | --- | --- | --- | --- | --- | --- | --- | --- | --- | --- | --- | --- | --- | --- | --- | --- | --- |

Q09GK1.1

back to list

  

| Protein Coverage
| Supporting Peptides
|

Protein Coverage:

Supporting Peptides:

| Peptide | Uniq | -10lgP | Mass | Length | ppm | m/z | z | RT | Fraction | Scan | Source File | Area BuCaMH04\_B15 | #Feature | #Feature BuCaMH04\_B15 | Start | End | PTM | AScore | Found By |
| --- | --- | --- | --- | --- | --- | --- | --- | --- | --- | --- | --- | --- | --- | --- | --- | --- | --- | --- | --- |
| G.FPC(+57.02)AQILEPGVYTK.V | N | 103.82 | 1621.8174 | 14 | 0.9 | 811.9167 | 2 | 30.60 | 1 | 18503 | BuCaMH04.raw | 7.1547E7 | 1 | 1 | 223 | 236 | Carbamidomethylation | C3:Carbamidomethylation:1000.00 | PEAKS DB |
| G.GFPC(+57.02)AQILEPGVYTK.V | Y | 80.47 | 1678.8389 | 15 | 0.1 | 840.4268 | 2 | 32.23 | 1 | 19810 | BuCaMH04.raw | 1.6412E7 | 1 | 1 | 222 | 236 | Carbamidomethylation | C4:Carbamidomethylation:1000.00 | PEAKS DB |
| total 2 peptides |  |  |  |  |  |  |  |  |  |  |  |  |  |  |  |  |  |  |  |
| --- | --- | --- | --- | --- | --- | --- | --- | --- | --- | --- | --- | --- | --- | --- | --- | --- | --- | --- | --- |

ETE66745.1

back to list

  

| Protein Coverage
| Supporting Peptides
|

Protein Coverage:

Supporting Peptides:

| Peptide | Uniq | -10lgP | Mass | Length | ppm | m/z | z | RT | Fraction | Scan | Source File | Area BuCaMH04\_B15 | #Feature | #Feature BuCaMH04\_B15 | Start | End | PTM | AScore | Found By |
| --- | --- | --- | --- | --- | --- | --- | --- | --- | --- | --- | --- | --- | --- | --- | --- | --- | --- | --- | --- |
| G.FPC(+57.02)AQLLEPGVYTK.V | N | 103.82 | 1621.8174 | 14 | 0.9 | 811.9167 | 2 | 30.60 | 1 | 18503 | BuCaMH04.raw | 7.1547E7 | 1 | 1 | 185 | 198 | Carbamidomethylation | C3:Carbamidomethylation:1000.00 | PEAKS DB |
| G.GFPC(+57.02)AQLLEPGVYTK.V | Y | 80.47 | 1678.8389 | 15 | 0.1 | 840.4268 | 2 | 32.23 | 1 | 19810 | BuCaMH04.raw | 1.6412E7 | 1 | 1 | 184 | 198 | Carbamidomethylation | C4:Carbamidomethylation:1000.00 | PEAKS DB |
| total 2 peptides |  |  |  |  |  |  |  |  |  |  |  |  |  |  |  |  |  |  |  |
| --- | --- | --- | --- | --- | --- | --- | --- | --- | --- | --- | --- | --- | --- | --- | --- | --- | --- | --- | --- |

AJB84504.1

back to list

  

| Protein Coverage
| Supporting Peptides
|

Protein Coverage:

Supporting Peptides:

| Peptide | Uniq | -10lgP | Mass | Length | ppm | m/z | z | RT | Fraction | Scan | Source File | Area BuCaMH04\_B15 | #Feature | #Feature BuCaMH04\_B15 | Start | End | PTM | AScore | Found By |
| --- | --- | --- | --- | --- | --- | --- | --- | --- | --- | --- | --- | --- | --- | --- | --- | --- | --- | --- | --- |
| G.FPC(+57.02)AQILEPGVYTK.V | N | 103.82 | 1621.8174 | 14 | 0.9 | 811.9167 | 2 | 30.60 | 1 | 18503 | BuCaMH04.raw | 7.1547E7 | 1 | 1 | 223 | 236 | Carbamidomethylation | C3:Carbamidomethylation:1000.00 | PEAKS DB |
| G.GFPC(+57.02)AQILEPGVYTK.V | Y | 80.47 | 1678.8389 | 15 | 0.1 | 840.4268 | 2 | 32.23 | 1 | 19810 | BuCaMH04.raw | 1.6412E7 | 1 | 1 | 222 | 236 | Carbamidomethylation | C4:Carbamidomethylation:1000.00 | PEAKS DB |
| total 2 peptides |  |  |  |  |  |  |  |  |  |  |  |  |  |  |  |  |  |  |  |
| --- | --- | --- | --- | --- | --- | --- | --- | --- | --- | --- | --- | --- | --- | --- | --- | --- | --- | --- | --- |

ABN72542.1

back to list

  

| Protein Coverage
| Supporting Peptides
|

Protein Coverage:

Supporting Peptides:

| Peptide | Uniq | -10lgP | Mass | Length | ppm | m/z | z | RT | Fraction | Scan | Source File | Area BuCaMH04\_B15 | #Feature | #Feature BuCaMH04\_B15 | Start | End | PTM | AScore | Found By |
| --- | --- | --- | --- | --- | --- | --- | --- | --- | --- | --- | --- | --- | --- | --- | --- | --- | --- | --- | --- |
| R.VELLYNPAFC(+57.02)SASTEGQR.Y | N | 80.36 | 2040.9574 | 18 | 0.6 | 1021.4866 | 2 | 29.17 | 1 | 17056 | BuCaMH04.raw | 9.7967E6 | 1 | 1 | 477 | 494 | Carbamidomethylation | C10:Carbamidomethylation:1000.00 | PEAKS DB |
| K.YFTYLILTK.G | N | 80.29 | 1160.6481 | 9 | 1.2 | 581.3320 | 2 | 35.60 | 1 | 23009 | BuCaMH04.raw | 5.5623E6 | 1 | 1 | 103 | 111 |  |  | PEAKS DB |
| total 2 peptides |  |  |  |  |  |  |  |  |  |  |  |  |  |  |  |  |  |  |  |
| --- | --- | --- | --- | --- | --- | --- | --- | --- | --- | --- | --- | --- | --- | --- | --- | --- | --- | --- | --- |

T\_164

back to list

  

| Protein Coverage
| Supporting Peptides
|

Protein Coverage:

Supporting Peptides:

| Peptide | Uniq | -10lgP | Mass | Length | ppm | m/z | z | RT | Fraction | Scan | Source File | Area BuCaMH04\_B15 | #Feature | #Feature BuCaMH04\_B15 | Start | End | PTM | AScore | Found By |
| --- | --- | --- | --- | --- | --- | --- | --- | --- | --- | --- | --- | --- | --- | --- | --- | --- | --- | --- | --- |
| R.VVSLNVLC(+57.02)TEC(+57.02)R.V | N | 85.35 | 1448.7115 | 12 | 0.3 | 725.3633 | 2 | 23.30 | 1 | 11847 | BuCaMH04.raw | 3.0811E8 | 1 | 1 | 66 | 77 | Carbamidomethylation | C8:Carbamidomethylation:1000.00;C11:Carbamidomethylation:1000.00 | PEAKS DB |
| R.GDSSNHNSGDLDISIVSDYIK.R | Y | 75.65 | 2235.0291 | 21 | -0.2 | 1118.5216 | 2 | 33.77 | 1 | 21211 | BuCaMH04.raw | 6.4244E6 | 1 | 1 | 110 | 130 |  |  | PEAKS DB |
| N.SGDLDISIVSDYIK.R | N | 62.14 | 1523.7719 | 14 | 0.9 | 762.8939 | 2 | 41.97 | 1 | 28608 | BuCaMH04.raw | 6.6435E5 | 1 | 1 | 117 | 130 |  |  | PEAKS DB |
| total 3 peptides |  |  |  |  |  |  |  |  |  |  |  |  |  |  |  |  |  |  |  |
| --- | --- | --- | --- | --- | --- | --- | --- | --- | --- | --- | --- | --- | --- | --- | --- | --- | --- | --- | --- |

XP\_032079507.1

back to list

  

| Protein Coverage
| Supporting Peptides
|

Protein Coverage:

Supporting Peptides:

| Peptide | Uniq | -10lgP | Mass | Length | ppm | m/z | z | RT | Fraction | Scan | Source File | Area BuCaMH04\_B15 | #Feature | #Feature BuCaMH04\_B15 | Start | End | PTM | AScore | Found By |
| --- | --- | --- | --- | --- | --- | --- | --- | --- | --- | --- | --- | --- | --- | --- | --- | --- | --- | --- | --- |
| K.SSIFGSVEIVNLNPEK.V | N | 82.75 | 1731.9043 | 16 | 1.0 | 866.9603 | 2 | 39.29 | 1 | 26305 | BuCaMH04.raw | 2.9489E7 | 1 | 1 | 222 | 237 |  |  | PEAKS DB |
| K.LFEQAFLYK.D | N | 76.10 | 1157.6121 | 9 | 0.5 | 579.8136 | 2 | 30.84 | 1 | 18607 | BuCaMH04.raw | 1.6044E7 | 1 | 1 | 91 | 99 |  |  | PEAKS DB |
| R.KSSIFGSVEIVNLNPEK.V | N | 65.40 | 1859.9993 | 17 | 0.7 | 621.0074 | 3 | 27.30 | 1 | 15442 | BuCaMH04.raw | 8.8246E6 | 1 | 1 | 221 | 237 |  |  | PEAKS DB |
| total 3 peptides |  |  |  |  |  |  |  |  |  |  |  |  |  |  |  |  |  |  |  |
| --- | --- | --- | --- | --- | --- | --- | --- | --- | --- | --- | --- | --- | --- | --- | --- | --- | --- | --- | --- |

XP\_013915471.1

back to list

  

| Protein Coverage
| Supporting Peptides
|

Protein Coverage:

Supporting Peptides:

| Peptide | Uniq | -10lgP | Mass | Length | ppm | m/z | z | RT | Fraction | Scan | Source File | Area BuCaMH04\_B15 | #Feature | #Feature BuCaMH04\_B15 | Start | End | PTM | AScore | Found By |
| --- | --- | --- | --- | --- | --- | --- | --- | --- | --- | --- | --- | --- | --- | --- | --- | --- | --- | --- | --- |
| K.SSIFGSVEIVNLNPEK.V | N | 82.75 | 1731.9043 | 16 | 1.0 | 866.9603 | 2 | 39.29 | 1 | 26305 | BuCaMH04.raw | 2.9489E7 | 1 | 1 | 222 | 237 |  |  | PEAKS DB |
| K.LFEQAFLYK.D | N | 76.10 | 1157.6121 | 9 | 0.5 | 579.8136 | 2 | 30.84 | 1 | 18607 | BuCaMH04.raw | 1.6044E7 | 1 | 1 | 91 | 99 |  |  | PEAKS DB |
| R.KSSIFGSVEIVNLNPEK.V | N | 65.40 | 1859.9993 | 17 | 0.7 | 621.0074 | 3 | 27.30 | 1 | 15442 | BuCaMH04.raw | 8.8246E6 | 1 | 1 | 221 | 237 |  |  | PEAKS DB |
| total 3 peptides |  |  |  |  |  |  |  |  |  |  |  |  |  |  |  |  |  |  |  |
| --- | --- | --- | --- | --- | --- | --- | --- | --- | --- | --- | --- | --- | --- | --- | --- | --- | --- | --- | --- |

XP\_013915472.1

back to list

  

| Protein Coverage
| Supporting Peptides
|

Protein Coverage:

Supporting Peptides:

| Peptide | Uniq | -10lgP | Mass | Length | ppm | m/z | z | RT | Fraction | Scan | Source File | Area BuCaMH04\_B15 | #Feature | #Feature BuCaMH04\_B15 | Start | End | PTM | AScore | Found By |
| --- | --- | --- | --- | --- | --- | --- | --- | --- | --- | --- | --- | --- | --- | --- | --- | --- | --- | --- | --- |
| K.SSIFGSVEIVNLNPEK.V | N | 82.75 | 1731.9043 | 16 | 1.0 | 866.9603 | 2 | 39.29 | 1 | 26305 | BuCaMH04.raw | 2.9489E7 | 1 | 1 | 222 | 237 |  |  | PEAKS DB |
| K.LFEQAFLYK.D | N | 76.10 | 1157.6121 | 9 | 0.5 | 579.8136 | 2 | 30.84 | 1 | 18607 | BuCaMH04.raw | 1.6044E7 | 1 | 1 | 91 | 99 |  |  | PEAKS DB |
| R.KSSIFGSVEIVNLNPEK.V | N | 65.40 | 1859.9993 | 17 | 0.7 | 621.0074 | 3 | 27.30 | 1 | 15442 | BuCaMH04.raw | 8.8246E6 | 1 | 1 | 221 | 237 |  |  | PEAKS DB |
| total 3 peptides |  |  |  |  |  |  |  |  |  |  |  |  |  |  |  |  |  |  |  |
| --- | --- | --- | --- | --- | --- | --- | --- | --- | --- | --- | --- | --- | --- | --- | --- | --- | --- | --- | --- |

XP\_034264771.1

back to list

  

| Protein Coverage
| Supporting Peptides
|

Protein Coverage:

Supporting Peptides:

| Peptide | Uniq | -10lgP | Mass | Length | ppm | m/z | z | RT | Fraction | Scan | Source File | Area BuCaMH04\_B15 | #Feature | #Feature BuCaMH04\_B15 | Start | End | PTM | AScore | Found By |
| --- | --- | --- | --- | --- | --- | --- | --- | --- | --- | --- | --- | --- | --- | --- | --- | --- | --- | --- | --- |
| K.SSIFGSVEIVNLNPEK.V | N | 82.75 | 1731.9043 | 16 | 1.0 | 866.9603 | 2 | 39.29 | 1 | 26305 | BuCaMH04.raw | 2.9489E7 | 1 | 1 | 222 | 237 |  |  | PEAKS DB |
| K.LFEQAFLYK.D | N | 76.10 | 1157.6121 | 9 | 0.5 | 579.8136 | 2 | 30.84 | 1 | 18607 | BuCaMH04.raw | 1.6044E7 | 1 | 1 | 91 | 99 |  |  | PEAKS DB |
| R.KSSIFGSVEIVNLNPEK.V | N | 65.40 | 1859.9993 | 17 | 0.7 | 621.0074 | 3 | 27.30 | 1 | 15442 | BuCaMH04.raw | 8.8246E6 | 1 | 1 | 221 | 237 |  |  | PEAKS DB |
| total 3 peptides |  |  |  |  |  |  |  |  |  |  |  |  |  |  |  |  |  |  |  |
| --- | --- | --- | --- | --- | --- | --- | --- | --- | --- | --- | --- | --- | --- | --- | --- | --- | --- | --- | --- |

XP\_034264772.1

back to list

  

| Protein Coverage
| Supporting Peptides
|

Protein Coverage:

Supporting Peptides:

| Peptide | Uniq | -10lgP | Mass | Length | ppm | m/z | z | RT | Fraction | Scan | Source File | Area BuCaMH04\_B15 | #Feature | #Feature BuCaMH04\_B15 | Start | End | PTM | AScore | Found By |
| --- | --- | --- | --- | --- | --- | --- | --- | --- | --- | --- | --- | --- | --- | --- | --- | --- | --- | --- | --- |
| K.SSIFGSVEIVNLNPEK.V | N | 82.75 | 1731.9043 | 16 | 1.0 | 866.9603 | 2 | 39.29 | 1 | 26305 | BuCaMH04.raw | 2.9489E7 | 1 | 1 | 222 | 237 |  |  | PEAKS DB |
| K.LFEQAFLYK.D | N | 76.10 | 1157.6121 | 9 | 0.5 | 579.8136 | 2 | 30.84 | 1 | 18607 | BuCaMH04.raw | 1.6044E7 | 1 | 1 | 91 | 99 |  |  | PEAKS DB |
| R.KSSIFGSVEIVNLNPEK.V | N | 65.40 | 1859.9993 | 17 | 0.7 | 621.0074 | 3 | 27.30 | 1 | 15442 | BuCaMH04.raw | 8.8246E6 | 1 | 1 | 221 | 237 |  |  | PEAKS DB |
| total 3 peptides |  |  |  |  |  |  |  |  |  |  |  |  |  |  |  |  |  |  |  |
| --- | --- | --- | --- | --- | --- | --- | --- | --- | --- | --- | --- | --- | --- | --- | --- | --- | --- | --- | --- |

AAM51550.1

back to list

  

| Protein Coverage
| Supporting Peptides
|

Protein Coverage:

Supporting Peptides:

| Peptide | Uniq | -10lgP | Mass | Length | ppm | m/z | z | RT | Fraction | Scan | Source File | Area BuCaMH04\_B15 | #Feature | #Feature BuCaMH04\_B15 | Start | End | PTM | AScore | Found By |
| --- | --- | --- | --- | --- | --- | --- | --- | --- | --- | --- | --- | --- | --- | --- | --- | --- | --- | --- | --- |
| Q.ATLDLFGEWR.E | N | 72.05 | 1206.6033 | 10 | 0.3 | 604.3091 | 2 | 42.94 | 1 | 29582 | BuCaMH04.raw | 1.2834E7 | 1 | 1 | 272 | 281 |  |  | PEAKS DB |
| A.TLDLFGEWR.E | N | 65.70 | 1135.5662 | 9 | 0.5 | 568.7906 | 2 | 42.31 | 1 | 29257 | BuCaMH04.raw | 4.2198E8 | 1 | 1 | 273 | 281 |  |  | PEAKS DB |
| T.LDLFGEWR.E | N | 57.32 | 1034.5186 | 8 | 0.6 | 518.2668 | 2 | 41.26 | 1 | 27863 | BuCaMH04.raw | 4.6562E7 | 1 | 1 | 274 | 281 |  |  | PEAKS DB |
| total 3 peptides |  |  |  |  |  |  |  |  |  |  |  |  |  |  |  |  |  |  |  |
| --- | --- | --- | --- | --- | --- | --- | --- | --- | --- | --- | --- | --- | --- | --- | --- | --- | --- | --- | --- |

Q10749.3

back to list

  

| Protein Coverage
| Supporting Peptides
|

Protein Coverage:

Supporting Peptides:

| Peptide | Uniq | -10lgP | Mass | Length | ppm | m/z | z | RT | Fraction | Scan | Source File | Area BuCaMH04\_B15 | #Feature | #Feature BuCaMH04\_B15 | Start | End | PTM | AScore | Found By |
| --- | --- | --- | --- | --- | --- | --- | --- | --- | --- | --- | --- | --- | --- | --- | --- | --- | --- | --- | --- |
| Q.ATLDLFGEWR.E | N | 72.05 | 1206.6033 | 10 | 0.3 | 604.3091 | 2 | 42.94 | 1 | 29582 | BuCaMH04.raw | 1.2834E7 | 1 | 1 | 272 | 281 |  |  | PEAKS DB |
| A.TLDLFGEWR.E | N | 65.70 | 1135.5662 | 9 | 0.5 | 568.7906 | 2 | 42.31 | 1 | 29257 | BuCaMH04.raw | 4.2198E8 | 1 | 1 | 273 | 281 |  |  | PEAKS DB |
| T.LDLFGEWR.E | N | 57.32 | 1034.5186 | 8 | 0.6 | 518.2668 | 2 | 41.26 | 1 | 27863 | BuCaMH04.raw | 4.6562E7 | 1 | 1 | 274 | 281 |  |  | PEAKS DB |
| total 3 peptides |  |  |  |  |  |  |  |  |  |  |  |  |  |  |  |  |  |  |  |
| --- | --- | --- | --- | --- | --- | --- | --- | --- | --- | --- | --- | --- | --- | --- | --- | --- | --- | --- | --- |

XP\_013928511.1

back to list

  

| Protein Coverage
| Supporting Peptides
|

Protein Coverage:

Supporting Peptides:

| Peptide | Uniq | -10lgP | Mass | Length | ppm | m/z | z | RT | Fraction | Scan | Source File | Area BuCaMH04\_B15 | #Feature | #Feature BuCaMH04\_B15 | Start | End | PTM | AScore | Found By |
| --- | --- | --- | --- | --- | --- | --- | --- | --- | --- | --- | --- | --- | --- | --- | --- | --- | --- | --- | --- |
| K.TFHGLGVIDWENWR.P | N | 78.32 | 1728.8372 | 14 | 0.1 | 865.4259 | 2 | 39.69 | 1 | 26511 | BuCaMH04.raw | 5.0487E7 | 2 | 2 | 125 | 138 |  |  | PEAKS DB |
| K.HSDSNAFLHLFP.E | N | 59.60 | 1383.6571 | 12 | 0.1 | 462.2264 | 3 | 33.71 | 1 | 21163 | BuCaMH04.raw | 1.1569E6 | 1 | 1 | 380 | 391 |  |  | PEAKS DB |
| K.DYALPVFVYAR.P | N | 59.05 | 1312.6815 | 11 | 0.3 | 657.3482 | 2 | 43.46 | 1 | 30072 | BuCaMH04.raw | 5.4049E6 | 1 | 1 | 285 | 295 |  |  | PEAKS DB |
| R.NDELLWLWR.D | Y | 56.09 | 1243.6349 | 9 | 0.6 | 622.8251 | 2 | 54.03 | 1 | 39434 | BuCaMH04.raw | 6.273E6 | 2 | 2 | 235 | 243 |  |  | PEAKS DB |
| total 4 peptides |  |  |  |  |  |  |  |  |  |  |  |  |  |  |  |  |  |  |  |
| --- | --- | --- | --- | --- | --- | --- | --- | --- | --- | --- | --- | --- | --- | --- | --- | --- | --- | --- | --- |

XP\_032077741.1

back to list

  

| Protein Coverage
| Supporting Peptides
|

Protein Coverage:

Supporting Peptides:

| Peptide | Uniq | -10lgP | Mass | Length | ppm | m/z | z | RT | Fraction | Scan | Source File | Area BuCaMH04\_B15 | #Feature | #Feature BuCaMH04\_B15 | Start | End | PTM | AScore | Found By |
| --- | --- | --- | --- | --- | --- | --- | --- | --- | --- | --- | --- | --- | --- | --- | --- | --- | --- | --- | --- |
| K.TFHGLGVIDWENWR.P | N | 78.32 | 1728.8372 | 14 | 0.1 | 865.4259 | 2 | 39.69 | 1 | 26511 | BuCaMH04.raw | 5.0487E7 | 2 | 2 | 125 | 138 |  |  | PEAKS DB |
| K.HSDSNAFLHLFP.E | N | 59.60 | 1383.6571 | 12 | 0.1 | 462.2264 | 3 | 33.71 | 1 | 21163 | BuCaMH04.raw | 1.1569E6 | 1 | 1 | 380 | 391 |  |  | PEAKS DB |
| K.DYALPVFVYAR.P | N | 59.05 | 1312.6815 | 11 | 0.3 | 657.3482 | 2 | 43.46 | 1 | 30072 | BuCaMH04.raw | 5.4049E6 | 1 | 1 | 285 | 295 |  |  | PEAKS DB |
| R.NDELLWLWR.D | Y | 56.09 | 1243.6349 | 9 | 0.6 | 622.8251 | 2 | 54.03 | 1 | 39434 | BuCaMH04.raw | 6.273E6 | 2 | 2 | 235 | 243 |  |  | PEAKS DB |
| total 4 peptides |  |  |  |  |  |  |  |  |  |  |  |  |  |  |  |  |  |  |  |
| --- | --- | --- | --- | --- | --- | --- | --- | --- | --- | --- | --- | --- | --- | --- | --- | --- | --- | --- | --- |

XP\_026546198.1

back to list

  

| Protein Coverage
| Supporting Peptides
|

Protein Coverage:

Supporting Peptides:

| Peptide | Uniq | -10lgP | Mass | Length | ppm | m/z | z | RT | Fraction | Scan | Source File | Area BuCaMH04\_B15 | #Feature | #Feature BuCaMH04\_B15 | Start | End | PTM | AScore | Found By |
| --- | --- | --- | --- | --- | --- | --- | --- | --- | --- | --- | --- | --- | --- | --- | --- | --- | --- | --- | --- |
| R.IPVYSAYVYNPGK.A | Y | 97.00 | 1469.7554 | 13 | -0.4 | 735.8846 | 2 | 23.66 | 1 | 11932 | BuCaMH04.raw | 1.3027E7 | 1 | 1 | 70 | 82 |  |  | PEAKS DB |
| K.SSTFTLTNIVPQFIK.L | Y | 79.00 | 1694.9243 | 15 | 0.5 | 848.4698 | 2 | 48.05 | 1 | 34118 | BuCaMH04.raw | 2.3613E7 | 1 | 1 | 154 | 168 |  |  | PEAKS DB |
| total 2 peptides |  |  |  |  |  |  |  |  |  |  |  |  |  |  |  |  |  |  |  |
| --- | --- | --- | --- | --- | --- | --- | --- | --- | --- | --- | --- | --- | --- | --- | --- | --- | --- | --- | --- |

XP\_026546199.1

back to list

  

| Protein Coverage
| Supporting Peptides
|

Protein Coverage:

Supporting Peptides:

| Peptide | Uniq | -10lgP | Mass | Length | ppm | m/z | z | RT | Fraction | Scan | Source File | Area BuCaMH04\_B15 | #Feature | #Feature BuCaMH04\_B15 | Start | End | PTM | AScore | Found By |
| --- | --- | --- | --- | --- | --- | --- | --- | --- | --- | --- | --- | --- | --- | --- | --- | --- | --- | --- | --- |
| R.IPVYSAYVYNPGK.A | Y | 97.00 | 1469.7554 | 13 | -0.4 | 735.8846 | 2 | 23.66 | 1 | 11932 | BuCaMH04.raw | 1.3027E7 | 1 | 1 | 70 | 82 |  |  | PEAKS DB |
| K.SSTFTLTNIVPQFIK.L | Y | 79.00 | 1694.9243 | 15 | 0.5 | 848.4698 | 2 | 48.05 | 1 | 34118 | BuCaMH04.raw | 2.3613E7 | 1 | 1 | 154 | 168 |  |  | PEAKS DB |
| total 2 peptides |  |  |  |  |  |  |  |  |  |  |  |  |  |  |  |  |  |  |  |
| --- | --- | --- | --- | --- | --- | --- | --- | --- | --- | --- | --- | --- | --- | --- | --- | --- | --- | --- | --- |

XP\_026579205.1

back to list

  

| Protein Coverage
| Supporting Peptides
|

Protein Coverage:

Supporting Peptides:

| Peptide | Uniq | -10lgP | Mass | Length | ppm | m/z | z | RT | Fraction | Scan | Source File | Area BuCaMH04\_B15 | #Feature | #Feature BuCaMH04\_B15 | Start | End | PTM | AScore | Found By |
| --- | --- | --- | --- | --- | --- | --- | --- | --- | --- | --- | --- | --- | --- | --- | --- | --- | --- | --- | --- |
| R.IPVYSAYVYNPGK.A | Y | 97.00 | 1469.7554 | 13 | -0.4 | 735.8846 | 2 | 23.66 | 1 | 11932 | BuCaMH04.raw | 1.3027E7 | 1 | 1 | 70 | 82 |  |  | PEAKS DB |
| K.SSTFTLTNIVPQFIK.L | Y | 79.00 | 1694.9243 | 15 | 0.5 | 848.4698 | 2 | 48.05 | 1 | 34118 | BuCaMH04.raw | 2.3613E7 | 1 | 1 | 154 | 168 |  |  | PEAKS DB |
| total 2 peptides |  |  |  |  |  |  |  |  |  |  |  |  |  |  |  |  |  |  |  |
| --- | --- | --- | --- | --- | --- | --- | --- | --- | --- | --- | --- | --- | --- | --- | --- | --- | --- | --- | --- |

XP\_026579207.1

back to list

  

| Protein Coverage
| Supporting Peptides
|

Protein Coverage:

Supporting Peptides:

| Peptide | Uniq | -10lgP | Mass | Length | ppm | m/z | z | RT | Fraction | Scan | Source File | Area BuCaMH04\_B15 | #Feature | #Feature BuCaMH04\_B15 | Start | End | PTM | AScore | Found By |
| --- | --- | --- | --- | --- | --- | --- | --- | --- | --- | --- | --- | --- | --- | --- | --- | --- | --- | --- | --- |
| R.IPVYSAYVYNPGK.A | Y | 97.00 | 1469.7554 | 13 | -0.4 | 735.8846 | 2 | 23.66 | 1 | 11932 | BuCaMH04.raw | 1.3027E7 | 1 | 1 | 70 | 82 |  |  | PEAKS DB |
| K.SSTFTLTNIVPQFIK.L | Y | 79.00 | 1694.9243 | 15 | 0.5 | 848.4698 | 2 | 48.05 | 1 | 34118 | BuCaMH04.raw | 2.3613E7 | 1 | 1 | 154 | 168 |  |  | PEAKS DB |
| total 2 peptides |  |  |  |  |  |  |  |  |  |  |  |  |  |  |  |  |  |  |  |
| --- | --- | --- | --- | --- | --- | --- | --- | --- | --- | --- | --- | --- | --- | --- | --- | --- | --- | --- | --- |

XP\_026546197.1

back to list

  

| Protein Coverage
| Supporting Peptides
|

Protein Coverage:

Supporting Peptides:

| Peptide | Uniq | -10lgP | Mass | Length | ppm | m/z | z | RT | Fraction | Scan | Source File | Area BuCaMH04\_B15 | #Feature | #Feature BuCaMH04\_B15 | Start | End | PTM | AScore | Found By |
| --- | --- | --- | --- | --- | --- | --- | --- | --- | --- | --- | --- | --- | --- | --- | --- | --- | --- | --- | --- |
| R.IPVYSAYVYNPGK.A | Y | 97.00 | 1469.7554 | 13 | -0.4 | 735.8846 | 2 | 23.66 | 1 | 11932 | BuCaMH04.raw | 1.3027E7 | 1 | 1 | 70 | 82 |  |  | PEAKS DB |
| K.SSTFTLTNIVPQFIK.L | Y | 79.00 | 1694.9243 | 15 | 0.5 | 848.4698 | 2 | 48.05 | 1 | 34118 | BuCaMH04.raw | 2.3613E7 | 1 | 1 | 154 | 168 |  |  | PEAKS DB |
| total 2 peptides |  |  |  |  |  |  |  |  |  |  |  |  |  |  |  |  |  |  |  |
| --- | --- | --- | --- | --- | --- | --- | --- | --- | --- | --- | --- | --- | --- | --- | --- | --- | --- | --- | --- |

XP\_026579206.1

back to list

  

| Protein Coverage
| Supporting Peptides
|

Protein Coverage:

Supporting Peptides:

| Peptide | Uniq | -10lgP | Mass | Length | ppm | m/z | z | RT | Fraction | Scan | Source File | Area BuCaMH04\_B15 | #Feature | #Feature BuCaMH04\_B15 | Start | End | PTM | AScore | Found By |
| --- | --- | --- | --- | --- | --- | --- | --- | --- | --- | --- | --- | --- | --- | --- | --- | --- | --- | --- | --- |
| R.IPVYSAYVYNPGK.A | Y | 97.00 | 1469.7554 | 13 | -0.4 | 735.8846 | 2 | 23.66 | 1 | 11932 | BuCaMH04.raw | 1.3027E7 | 1 | 1 | 70 | 82 |  |  | PEAKS DB |
| K.SSTFTLTNIVPQFIK.L | Y | 79.00 | 1694.9243 | 15 | 0.5 | 848.4698 | 2 | 48.05 | 1 | 34118 | BuCaMH04.raw | 2.3613E7 | 1 | 1 | 154 | 168 |  |  | PEAKS DB |
| total 2 peptides |  |  |  |  |  |  |  |  |  |  |  |  |  |  |  |  |  |  |  |
| --- | --- | --- | --- | --- | --- | --- | --- | --- | --- | --- | --- | --- | --- | --- | --- | --- | --- | --- | --- |

XP\_026546196.1

back to list

  

| Protein Coverage
| Supporting Peptides
|

Protein Coverage:

Supporting Peptides:

| Peptide | Uniq | -10lgP | Mass | Length | ppm | m/z | z | RT | Fraction | Scan | Source File | Area BuCaMH04\_B15 | #Feature | #Feature BuCaMH04\_B15 | Start | End | PTM | AScore | Found By |
| --- | --- | --- | --- | --- | --- | --- | --- | --- | --- | --- | --- | --- | --- | --- | --- | --- | --- | --- | --- |
| R.IPVYSAYVYNPGK.A | Y | 97.00 | 1469.7554 | 13 | -0.4 | 735.8846 | 2 | 23.66 | 1 | 11932 | BuCaMH04.raw | 1.3027E7 | 1 | 1 | 84 | 96 |  |  | PEAKS DB |
| K.SSTFTLTNIVPQFIK.L | Y | 79.00 | 1694.9243 | 15 | 0.5 | 848.4698 | 2 | 48.05 | 1 | 34118 | BuCaMH04.raw | 2.3613E7 | 1 | 1 | 168 | 182 |  |  | PEAKS DB |
| total 2 peptides |  |  |  |  |  |  |  |  |  |  |  |  |  |  |  |  |  |  |  |
| --- | --- | --- | --- | --- | --- | --- | --- | --- | --- | --- | --- | --- | --- | --- | --- | --- | --- | --- | --- |

JAB54304.1

back to list

  

| Protein Coverage
| Supporting Peptides
|

Protein Coverage:

Supporting Peptides:

| Peptide | Uniq | -10lgP | Mass | Length | ppm | m/z | z | RT | Fraction | Scan | Source File | Area BuCaMH04\_B15 | #Feature | #Feature BuCaMH04\_B15 | Start | End | PTM | AScore | Found By |
| --- | --- | --- | --- | --- | --- | --- | --- | --- | --- | --- | --- | --- | --- | --- | --- | --- | --- | --- | --- |
| H.IPVYSAYVYNPGK.A | Y | 97.00 | 1469.7554 | 13 | -0.4 | 735.8846 | 2 | 23.66 | 1 | 11932 | BuCaMH04.raw | 1.3027E7 | 1 | 1 | 70 | 82 |  |  | PEAKS DB |
| K.SSTFTLTNIVPQFIK.L | Y | 79.00 | 1694.9243 | 15 | 0.5 | 848.4698 | 2 | 48.05 | 1 | 34118 | BuCaMH04.raw | 2.3613E7 | 1 | 1 | 154 | 168 |  |  | PEAKS DB |
| total 2 peptides |  |  |  |  |  |  |  |  |  |  |  |  |  |  |  |  |  |  |  |
| --- | --- | --- | --- | --- | --- | --- | --- | --- | --- | --- | --- | --- | --- | --- | --- | --- | --- | --- | --- |

XP\_026541916.1

back to list

  

| Protein Coverage
| Supporting Peptides
|

Protein Coverage:

Supporting Peptides:

| Peptide | Uniq | -10lgP | Mass | Length | ppm | m/z | z | RT | Fraction | Scan | Source File | Area BuCaMH04\_B15 | #Feature | #Feature BuCaMH04\_B15 | Start | End | PTM | AScore | Found By |
| --- | --- | --- | --- | --- | --- | --- | --- | --- | --- | --- | --- | --- | --- | --- | --- | --- | --- | --- | --- |
| R.LVILGFPC(+57.02)NQFGK.Q | N | 87.60 | 1491.7908 | 13 | 0.3 | 746.9029 | 2 | 39.13 | 1 | 26133 | BuCaMH04.raw | 3.6053E7 | 1 | 1 | 93 | 105 | Carbamidomethylation | C8:Carbamidomethylation:1000.00 | PEAKS DB |
| R.LVILGFPC(+57.02)NQ.F | N | 61.53 | 1159.6060 | 10 | -0.1 | 580.8102 | 2 | 45.31 | 1 | 31387 | BuCaMH04.raw | 5.0904E6 | 1 | 1 | 93 | 102 | Carbamidomethylation | C8:Carbamidomethylation:1000.00 | PEAKS DB |
| R.LFWSPLK.I | Y | 51.15 | 889.5062 | 7 | 0.4 | 445.7605 | 2 | 32.29 | 1 | 19854 | BuCaMH04.raw | 6.8113E6 | 1 | 1 | 168 | 174 |  |  | PEAKS DB |
| total 3 peptides |  |  |  |  |  |  |  |  |  |  |  |  |  |  |  |  |  |  |  |
| --- | --- | --- | --- | --- | --- | --- | --- | --- | --- | --- | --- | --- | --- | --- | --- | --- | --- | --- | --- |

XP\_026552407.1

back to list

  

| Protein Coverage
| Supporting Peptides
|

Protein Coverage:

Supporting Peptides:

| Peptide | Uniq | -10lgP | Mass | Length | ppm | m/z | z | RT | Fraction | Scan | Source File | Area BuCaMH04\_B15 | #Feature | #Feature BuCaMH04\_B15 | Start | End | PTM | AScore | Found By |
| --- | --- | --- | --- | --- | --- | --- | --- | --- | --- | --- | --- | --- | --- | --- | --- | --- | --- | --- | --- |
| R.LVILGFPC(+57.02)NQFGK.Q | N | 87.60 | 1491.7908 | 13 | 0.3 | 746.9029 | 2 | 39.13 | 1 | 26133 | BuCaMH04.raw | 3.6053E7 | 1 | 1 | 93 | 105 | Carbamidomethylation | C8:Carbamidomethylation:1000.00 | PEAKS DB |
| R.LVILGFPC(+57.02)NQ.F | N | 61.53 | 1159.6060 | 10 | -0.1 | 580.8102 | 2 | 45.31 | 1 | 31387 | BuCaMH04.raw | 5.0904E6 | 1 | 1 | 93 | 102 | Carbamidomethylation | C8:Carbamidomethylation:1000.00 | PEAKS DB |
| R.LFWSPLK.I | Y | 51.15 | 889.5062 | 7 | 0.4 | 445.7605 | 2 | 32.29 | 1 | 19854 | BuCaMH04.raw | 6.8113E6 | 1 | 1 | 168 | 174 |  |  | PEAKS DB |
| total 3 peptides |  |  |  |  |  |  |  |  |  |  |  |  |  |  |  |  |  |  |  |
| --- | --- | --- | --- | --- | --- | --- | --- | --- | --- | --- | --- | --- | --- | --- | --- | --- | --- | --- | --- |

XP\_026552406.1

back to list

  

| Protein Coverage
| Supporting Peptides
|

Protein Coverage:

Supporting Peptides:

| Peptide | Uniq | -10lgP | Mass | Length | ppm | m/z | z | RT | Fraction | Scan | Source File | Area BuCaMH04\_B15 | #Feature | #Feature BuCaMH04\_B15 | Start | End | PTM | AScore | Found By |
| --- | --- | --- | --- | --- | --- | --- | --- | --- | --- | --- | --- | --- | --- | --- | --- | --- | --- | --- | --- |
| R.LVILGFPC(+57.02)NQFGK.Q | N | 87.60 | 1491.7908 | 13 | 0.3 | 746.9029 | 2 | 39.13 | 1 | 26133 | BuCaMH04.raw | 3.6053E7 | 1 | 1 | 124 | 136 | Carbamidomethylation | C8:Carbamidomethylation:1000.00 | PEAKS DB |
| R.LVILGFPC(+57.02)NQ.F | N | 61.53 | 1159.6060 | 10 | -0.1 | 580.8102 | 2 | 45.31 | 1 | 31387 | BuCaMH04.raw | 5.0904E6 | 1 | 1 | 124 | 133 | Carbamidomethylation | C8:Carbamidomethylation:1000.00 | PEAKS DB |
| R.LFWSPLK.I | Y | 51.15 | 889.5062 | 7 | 0.4 | 445.7605 | 2 | 32.29 | 1 | 19854 | BuCaMH04.raw | 6.8113E6 | 1 | 1 | 199 | 205 |  |  | PEAKS DB |
| total 3 peptides |  |  |  |  |  |  |  |  |  |  |  |  |  |  |  |  |  |  |  |
| --- | --- | --- | --- | --- | --- | --- | --- | --- | --- | --- | --- | --- | --- | --- | --- | --- | --- | --- | --- |

XP\_026541908.1

back to list

  

| Protein Coverage
| Supporting Peptides
|

Protein Coverage:

Supporting Peptides:

| Peptide | Uniq | -10lgP | Mass | Length | ppm | m/z | z | RT | Fraction | Scan | Source File | Area BuCaMH04\_B15 | #Feature | #Feature BuCaMH04\_B15 | Start | End | PTM | AScore | Found By |
| --- | --- | --- | --- | --- | --- | --- | --- | --- | --- | --- | --- | --- | --- | --- | --- | --- | --- | --- | --- |
| R.LVILGFPC(+57.02)NQFGK.Q | N | 87.60 | 1491.7908 | 13 | 0.3 | 746.9029 | 2 | 39.13 | 1 | 26133 | BuCaMH04.raw | 3.6053E7 | 1 | 1 | 124 | 136 | Carbamidomethylation | C8:Carbamidomethylation:1000.00 | PEAKS DB |
| R.LVILGFPC(+57.02)NQ.F | N | 61.53 | 1159.6060 | 10 | -0.1 | 580.8102 | 2 | 45.31 | 1 | 31387 | BuCaMH04.raw | 5.0904E6 | 1 | 1 | 124 | 133 | Carbamidomethylation | C8:Carbamidomethylation:1000.00 | PEAKS DB |
| R.LFWSPLK.I | Y | 51.15 | 889.5062 | 7 | 0.4 | 445.7605 | 2 | 32.29 | 1 | 19854 | BuCaMH04.raw | 6.8113E6 | 1 | 1 | 199 | 205 |  |  | PEAKS DB |
| total 3 peptides |  |  |  |  |  |  |  |  |  |  |  |  |  |  |  |  |  |  |  |
| --- | --- | --- | --- | --- | --- | --- | --- | --- | --- | --- | --- | --- | --- | --- | --- | --- | --- | --- | --- |

XP\_015679695.1

back to list

  

| Protein Coverage
| Supporting Peptides
|

Protein Coverage:

Supporting Peptides:

| Peptide | Uniq | -10lgP | Mass | Length | ppm | m/z | z | RT | Fraction | Scan | Source File | Area BuCaMH04\_B15 | #Feature | #Feature BuCaMH04\_B15 | Start | End | PTM | AScore | Found By |
| --- | --- | --- | --- | --- | --- | --- | --- | --- | --- | --- | --- | --- | --- | --- | --- | --- | --- | --- | --- |
| R.LVILGFPC(+57.02)NQFGK.Q | N | 87.60 | 1491.7908 | 13 | 0.3 | 746.9029 | 2 | 39.13 | 1 | 26133 | BuCaMH04.raw | 3.6053E7 | 1 | 1 | 121 | 133 | Carbamidomethylation | C8:Carbamidomethylation:1000.00 | PEAKS DB |
| R.LVILGFPC(+57.02)NQ.F | N | 61.53 | 1159.6060 | 10 | -0.1 | 580.8102 | 2 | 45.31 | 1 | 31387 | BuCaMH04.raw | 5.0904E6 | 1 | 1 | 121 | 130 | Carbamidomethylation | C8:Carbamidomethylation:1000.00 | PEAKS DB |
| K.LFWSPLK.T | Y | 51.15 | 889.5062 | 7 | 0.4 | 445.7605 | 2 | 32.29 | 1 | 19854 | BuCaMH04.raw | 6.8113E6 | 1 | 1 | 196 | 202 |  |  | PEAKS DB |
| total 3 peptides |  |  |  |  |  |  |  |  |  |  |  |  |  |  |  |  |  |  |  |
| --- | --- | --- | --- | --- | --- | --- | --- | --- | --- | --- | --- | --- | --- | --- | --- | --- | --- | --- | --- |

ETE68810.1

back to list

  

| Protein Coverage
| Supporting Peptides
|

Protein Coverage:

Supporting Peptides:

| Peptide | Uniq | -10lgP | Mass | Length | ppm | m/z | z | RT | Fraction | Scan | Source File | Area BuCaMH04\_B15 | #Feature | #Feature BuCaMH04\_B15 | Start | End | PTM | AScore | Found By |
| --- | --- | --- | --- | --- | --- | --- | --- | --- | --- | --- | --- | --- | --- | --- | --- | --- | --- | --- | --- |
| R.LVILGFPC(+57.02)NQFGK.Q | N | 87.60 | 1491.7908 | 13 | 0.3 | 746.9029 | 2 | 39.13 | 1 | 26133 | BuCaMH04.raw | 3.6053E7 | 1 | 1 | 137 | 149 | Carbamidomethylation | C8:Carbamidomethylation:1000.00 | PEAKS DB |
| R.LVILGFPC(+57.02)NQ.F | N | 61.53 | 1159.6060 | 10 | -0.1 | 580.8102 | 2 | 45.31 | 1 | 31387 | BuCaMH04.raw | 5.0904E6 | 1 | 1 | 137 | 146 | Carbamidomethylation | C8:Carbamidomethylation:1000.00 | PEAKS DB |
| K.LFWSPLK.I | Y | 51.15 | 889.5062 | 7 | 0.4 | 445.7605 | 2 | 32.29 | 1 | 19854 | BuCaMH04.raw | 6.8113E6 | 1 | 1 | 212 | 218 |  |  | PEAKS DB |
| total 3 peptides |  |  |  |  |  |  |  |  |  |  |  |  |  |  |  |  |  |  |  |
| --- | --- | --- | --- | --- | --- | --- | --- | --- | --- | --- | --- | --- | --- | --- | --- | --- | --- | --- | --- |

XP\_034277309.1

back to list

  

| Protein Coverage
| Supporting Peptides
|

Protein Coverage:

Supporting Peptides:

| Peptide | Uniq | -10lgP | Mass | Length | ppm | m/z | z | RT | Fraction | Scan | Source File | Area BuCaMH04\_B15 | #Feature | #Feature BuCaMH04\_B15 | Start | End | PTM | AScore | Found By |
| --- | --- | --- | --- | --- | --- | --- | --- | --- | --- | --- | --- | --- | --- | --- | --- | --- | --- | --- | --- |
| R.NDQLLWLWR.D | N | 80.31 | 1242.6509 | 9 | 0.3 | 622.3329 | 2 | 47.58 | 1 | 33471 | BuCaMH04.raw | 1.0577E8 | 1 | 1 | 251 | 259 |  |  | PEAKS DB |
| K.DYALPVFVYAR.P | N | 59.05 | 1312.6815 | 11 | 0.3 | 657.3482 | 2 | 43.46 | 1 | 30072 | BuCaMH04.raw | 5.4049E6 | 1 | 1 | 301 | 311 |  |  | PEAKS DB |
| total 2 peptides |  |  |  |  |  |  |  |  |  |  |  |  |  |  |  |  |  |  |  |
| --- | --- | --- | --- | --- | --- | --- | --- | --- | --- | --- | --- | --- | --- | --- | --- | --- | --- | --- | --- |

T\_180

back to list

  

| Protein Coverage
| Supporting Peptides
|

Protein Coverage:

Supporting Peptides:

| Peptide | Uniq | -10lgP | Mass | Length | ppm | m/z | z | RT | Fraction | Scan | Source File | Area BuCaMH04\_B15 | #Feature | #Feature BuCaMH04\_B15 | Start | End | PTM | AScore | Found By |
| --- | --- | --- | --- | --- | --- | --- | --- | --- | --- | --- | --- | --- | --- | --- | --- | --- | --- | --- | --- |
| R.WYPGSSISAGC(+57.02)AVTC(+57.02)PK.K | Y | 79.18 | 1839.8284 | 17 | 0.6 | 920.9220 | 2 | 21.84 | 1 | 10605 | BuCaMH04.raw | 6.9785E7 | 1 | 1 | 58 | 74 | Carbamidomethylation | C11:Carbamidomethylation:1000.00;C15:Carbamidomethylation:1000.00 | PEAKS DB |
| K.LPLEVVEC(+57.02)C(+57.02)TTDR.C | Y | 73.81 | 1590.7382 | 13 | 1.8 | 796.3778 | 2 | 21.46 | 1 | 10140 | BuCaMH04.raw | 4.9851E5 | 1 | 1 | 76 | 88 | Carbamidomethylation | C8:Carbamidomethylation:1000.00;C9:Carbamidomethylation:1000.00 | PEAKS DB |
| K.SVVFETSETC(+57.02)LYGQNVC(+57.02)FK.R | Y | 56.28 | 2267.0239 | 19 | 1.4 | 756.6830 | 3 | 32.16 | 1 | 19724 | BuCaMH04.raw | 5.2189E6 | 1 | 1 | 38 | 56 | Carbamidomethylation | C10:Carbamidomethylation:1000.00;C17:Carbamidomethylation:1000.00 | PEAKS DB |
| total 3 peptides |  |  |  |  |  |  |  |  |  |  |  |  |  |  |  |  |  |  |  |
| --- | --- | --- | --- | --- | --- | --- | --- | --- | --- | --- | --- | --- | --- | --- | --- | --- | --- | --- | --- |

T\_133

back to list

  

| Protein Coverage
| Supporting Peptides
|

Protein Coverage:

Supporting Peptides:

| Peptide | Uniq | -10lgP | Mass | Length | ppm | m/z | z | RT | Fraction | Scan | Source File | Area BuCaMH04\_B15 | #Feature | #Feature BuCaMH04\_B15 | Start | End | PTM | AScore | Found By |
| --- | --- | --- | --- | --- | --- | --- | --- | --- | --- | --- | --- | --- | --- | --- | --- | --- | --- | --- | --- |
| Q.LDEVIPFMTVFER.S | Y | 77.47 | 1594.8065 | 13 | 0.1 | 798.4106 | 2 | 55.38 | 1 | 40495 | BuCaMH04.raw | 3.4288E7 | 1 | 1 | 37 | 49 |  |  | PEAKS DB |
| K.QLDEVIPFMTVFER.S | Y | 51.15 | 1722.8650 | 14 | 1.0 | 862.4407 | 2 | 56.93 | 1 | 41733 | BuCaMH04.raw | 7.7108E5 | 1 | 1 | 36 | 49 |  |  | PEAKS DB |
| total 2 peptides |  |  |  |  |  |  |  |  |  |  |  |  |  |  |  |  |  |  |  |
| --- | --- | --- | --- | --- | --- | --- | --- | --- | --- | --- | --- | --- | --- | --- | --- | --- | --- | --- | --- |

T\_131

back to list

  

| Protein Coverage
| Supporting Peptides
|

Protein Coverage:

Supporting Peptides:

| Peptide | Uniq | -10lgP | Mass | Length | ppm | m/z | z | RT | Fraction | Scan | Source File | Area BuCaMH04\_B15 | #Feature | #Feature BuCaMH04\_B15 | Start | End | PTM | AScore | Found By |
| --- | --- | --- | --- | --- | --- | --- | --- | --- | --- | --- | --- | --- | --- | --- | --- | --- | --- | --- | --- |
| Q.LDEVIPFMTVFER.S | Y | 77.47 | 1594.8065 | 13 | 0.1 | 798.4106 | 2 | 55.38 | 1 | 40495 | BuCaMH04.raw | 3.4288E7 | 1 | 1 | 37 | 49 |  |  | PEAKS DB |
| K.QLDEVIPFMTVFER.S | Y | 51.15 | 1722.8650 | 14 | 1.0 | 862.4407 | 2 | 56.93 | 1 | 41733 | BuCaMH04.raw | 7.7108E5 | 1 | 1 | 36 | 49 |  |  | PEAKS DB |
| total 2 peptides |  |  |  |  |  |  |  |  |  |  |  |  |  |  |  |  |  |  |  |
| --- | --- | --- | --- | --- | --- | --- | --- | --- | --- | --- | --- | --- | --- | --- | --- | --- | --- | --- | --- |

T\_132

back to list

  

| Protein Coverage
| Supporting Peptides
|

Protein Coverage:

Supporting Peptides:

| Peptide | Uniq | -10lgP | Mass | Length | ppm | m/z | z | RT | Fraction | Scan | Source File | Area BuCaMH04\_B15 | #Feature | #Feature BuCaMH04\_B15 | Start | End | PTM | AScore | Found By |
| --- | --- | --- | --- | --- | --- | --- | --- | --- | --- | --- | --- | --- | --- | --- | --- | --- | --- | --- | --- |
| Q.LDEVIPFMTVFER.S | Y | 77.47 | 1594.8065 | 13 | 0.1 | 798.4106 | 2 | 55.38 | 1 | 40495 | BuCaMH04.raw | 3.4288E7 | 1 | 1 | 37 | 49 |  |  | PEAKS DB |
| K.QLDEVIPFMTVFER.S | Y | 51.15 | 1722.8650 | 14 | 1.0 | 862.4407 | 2 | 56.93 | 1 | 41733 | BuCaMH04.raw | 7.7108E5 | 1 | 1 | 36 | 49 |  |  | PEAKS DB |
| total 2 peptides |  |  |  |  |  |  |  |  |  |  |  |  |  |  |  |  |  |  |  |
| --- | --- | --- | --- | --- | --- | --- | --- | --- | --- | --- | --- | --- | --- | --- | --- | --- | --- | --- | --- |

T\_128

back to list

  

| Protein Coverage
| Supporting Peptides
|

Protein Coverage:

Supporting Peptides:

| Peptide | Uniq | -10lgP | Mass | Length | ppm | m/z | z | RT | Fraction | Scan | Source File | Area BuCaMH04\_B15 | #Feature | #Feature BuCaMH04\_B15 | Start | End | PTM | AScore | Found By |
| --- | --- | --- | --- | --- | --- | --- | --- | --- | --- | --- | --- | --- | --- | --- | --- | --- | --- | --- | --- |
| Q.LDEVIPFMTVFER.S | Y | 77.47 | 1594.8065 | 13 | 0.1 | 798.4106 | 2 | 55.38 | 1 | 40495 | BuCaMH04.raw | 3.4288E7 | 1 | 1 | 37 | 49 |  |  | PEAKS DB |
| K.QLDEVIPFMTVFER.S | Y | 51.15 | 1722.8650 | 14 | 1.0 | 862.4407 | 2 | 56.93 | 1 | 41733 | BuCaMH04.raw | 7.7108E5 | 1 | 1 | 36 | 49 |  |  | PEAKS DB |
| total 2 peptides |  |  |  |  |  |  |  |  |  |  |  |  |  |  |  |  |  |  |  |
| --- | --- | --- | --- | --- | --- | --- | --- | --- | --- | --- | --- | --- | --- | --- | --- | --- | --- | --- | --- |

T\_129

back to list

  

| Protein Coverage
| Supporting Peptides
|

Protein Coverage:

Supporting Peptides:

| Peptide | Uniq | -10lgP | Mass | Length | ppm | m/z | z | RT | Fraction | Scan | Source File | Area BuCaMH04\_B15 | #Feature | #Feature BuCaMH04\_B15 | Start | End | PTM | AScore | Found By |
| --- | --- | --- | --- | --- | --- | --- | --- | --- | --- | --- | --- | --- | --- | --- | --- | --- | --- | --- | --- |
| Q.LDEVIPFMTVFER.S | Y | 77.47 | 1594.8065 | 13 | 0.1 | 798.4106 | 2 | 55.38 | 1 | 40495 | BuCaMH04.raw | 3.4288E7 | 1 | 1 | 37 | 49 |  |  | PEAKS DB |
| K.QLDEVIPFMTVFER.S | Y | 51.15 | 1722.8650 | 14 | 1.0 | 862.4407 | 2 | 56.93 | 1 | 41733 | BuCaMH04.raw | 7.7108E5 | 1 | 1 | 36 | 49 |  |  | PEAKS DB |
| total 2 peptides |  |  |  |  |  |  |  |  |  |  |  |  |  |  |  |  |  |  |  |
| --- | --- | --- | --- | --- | --- | --- | --- | --- | --- | --- | --- | --- | --- | --- | --- | --- | --- | --- | --- |

T\_130

back to list

  

| Protein Coverage
| Supporting Peptides
|

Protein Coverage:

Supporting Peptides:

| Peptide | Uniq | -10lgP | Mass | Length | ppm | m/z | z | RT | Fraction | Scan | Source File | Area BuCaMH04\_B15 | #Feature | #Feature BuCaMH04\_B15 | Start | End | PTM | AScore | Found By |
| --- | --- | --- | --- | --- | --- | --- | --- | --- | --- | --- | --- | --- | --- | --- | --- | --- | --- | --- | --- |
| Q.LDEVIPFMTVFER.S | Y | 77.47 | 1594.8065 | 13 | 0.1 | 798.4106 | 2 | 55.38 | 1 | 40495 | BuCaMH04.raw | 3.4288E7 | 1 | 1 | 37 | 49 |  |  | PEAKS DB |
| K.QLDEVIPFMTVFER.S | Y | 51.15 | 1722.8650 | 14 | 1.0 | 862.4407 | 2 | 56.93 | 1 | 41733 | BuCaMH04.raw | 7.7108E5 | 1 | 1 | 36 | 49 |  |  | PEAKS DB |
| total 2 peptides |  |  |  |  |  |  |  |  |  |  |  |  |  |  |  |  |  |  |  |
| --- | --- | --- | --- | --- | --- | --- | --- | --- | --- | --- | --- | --- | --- | --- | --- | --- | --- | --- | --- |

JAC94991.1

back to list

  

| Protein Coverage
| Supporting Peptides
|

Protein Coverage:

Supporting Peptides:

| Peptide | Uniq | -10lgP | Mass | Length | ppm | m/z | z | RT | Fraction | Scan | Source File | Area BuCaMH04\_B15 | #Feature | #Feature BuCaMH04\_B15 | Start | End | PTM | AScore | Found By |
| --- | --- | --- | --- | --- | --- | --- | --- | --- | --- | --- | --- | --- | --- | --- | --- | --- | --- | --- | --- |
| A.DIVINDLSLIHQLPK.E | N | 69.37 | 1716.9774 | 15 | 0.4 | 859.4963 | 2 | 37.46 | 1 | 24584 | BuCaMH04.raw | 4.3779E7 | 2 | 2 | 252 | 266 |  |  | PEAKS DB |
| K.RFDEIVGGFDQLPISMYQAIAETVH.L | Y | 57.02 | 2835.3901 | 25 | 0.5 | 946.1378 | 3 | 61.96 | 1 | 46272 | BuCaMH04.raw | 1.0884E6 | 1 | 1 | 93 | 117 |  |  | PEAKS DB |
| K.RFDEIVGGFDQLPIS.M | N | 52.08 | 1691.8518 | 15 | 0.2 | 846.9333 | 2 | 43.43 | 1 | 29880 | BuCaMH04.raw | 8.7751E5 | 1 | 1 | 93 | 107 |  |  | PEAKS DB |
| total 3 peptides |  |  |  |  |  |  |  |  |  |  |  |  |  |  |  |  |  |  |  |
| --- | --- | --- | --- | --- | --- | --- | --- | --- | --- | --- | --- | --- | --- | --- | --- | --- | --- | --- | --- |

XP\_015677037.1

back to list

  

| Protein Coverage
| Supporting Peptides
|

Protein Coverage:

Supporting Peptides:

| Peptide | Uniq | -10lgP | Mass | Length | ppm | m/z | z | RT | Fraction | Scan | Source File | Area BuCaMH04\_B15 | #Feature | #Feature BuCaMH04\_B15 | Start | End | PTM | AScore | Found By |
| --- | --- | --- | --- | --- | --- | --- | --- | --- | --- | --- | --- | --- | --- | --- | --- | --- | --- | --- | --- |
| R.GTTEHLLEIVLGR.C | N | 85.53 | 1436.7987 | 13 | 0.8 | 479.9406 | 3 | 36.09 | 1 | 23451 | BuCaMH04.raw | 2.1732E7 | 2 | 2 | 56 | 68 |  |  | PEAKS DB |
| K.LFEQAFLYK.D | N | 76.10 | 1157.6121 | 9 | 0.5 | 579.8136 | 2 | 30.84 | 1 | 18607 | BuCaMH04.raw | 1.6044E7 | 1 | 1 | 91 | 99 |  |  | PEAKS DB |
| total 2 peptides |  |  |  |  |  |  |  |  |  |  |  |  |  |  |  |  |  |  |  |
| --- | --- | --- | --- | --- | --- | --- | --- | --- | --- | --- | --- | --- | --- | --- | --- | --- | --- | --- | --- |

XP\_026542555.1

back to list

  

| Protein Coverage
| Supporting Peptides
|

Protein Coverage:

Supporting Peptides:

| Peptide | Uniq | -10lgP | Mass | Length | ppm | m/z | z | RT | Fraction | Scan | Source File | Area BuCaMH04\_B15 | #Feature | #Feature BuCaMH04\_B15 | Start | End | PTM | AScore | Found By |
| --- | --- | --- | --- | --- | --- | --- | --- | --- | --- | --- | --- | --- | --- | --- | --- | --- | --- | --- | --- |
| R.ATLLSPFFGTK.S | Y | 74.21 | 1180.6492 | 11 | 1.1 | 591.3325 | 2 | 39.06 | 1 | 25971 | BuCaMH04.raw | 1.4097E6 | 1 | 1 | 31 | 41 |  |  | PEAKS DB |
| A.TLLSPFFGTK.S | Y | 59.47 | 1109.6121 | 10 | 1.0 | 555.8138 | 2 | 37.16 | 1 | 24246 | BuCaMH04.raw | 8.7599E5 | 1 | 1 | 32 | 41 |  |  | PEAKS DB |
| K.VLEYLNDLK.Q | Y | 56.45 | 1105.6019 | 9 | 1.0 | 553.8088 | 2 | 25.14 | 1 | 13298 | BuCaMH04.raw | 4.2573E6 | 1 | 1 | 293 | 301 |  |  | PEAKS DB |
| total 3 peptides |  |  |  |  |  |  |  |  |  |  |  |  |  |  |  |  |  |  |  |
| --- | --- | --- | --- | --- | --- | --- | --- | --- | --- | --- | --- | --- | --- | --- | --- | --- | --- | --- | --- |

XP\_026542553.1

back to list

  

| Protein Coverage
| Supporting Peptides
|

Protein Coverage:

Supporting Peptides:

| Peptide | Uniq | -10lgP | Mass | Length | ppm | m/z | z | RT | Fraction | Scan | Source File | Area BuCaMH04\_B15 | #Feature | #Feature BuCaMH04\_B15 | Start | End | PTM | AScore | Found By |
| --- | --- | --- | --- | --- | --- | --- | --- | --- | --- | --- | --- | --- | --- | --- | --- | --- | --- | --- | --- |
| R.ATLLSPFFGTK.S | Y | 74.21 | 1180.6492 | 11 | 1.1 | 591.3325 | 2 | 39.06 | 1 | 25971 | BuCaMH04.raw | 1.4097E6 | 1 | 1 | 31 | 41 |  |  | PEAKS DB |
| A.TLLSPFFGTK.S | Y | 59.47 | 1109.6121 | 10 | 1.0 | 555.8138 | 2 | 37.16 | 1 | 24246 | BuCaMH04.raw | 8.7599E5 | 1 | 1 | 32 | 41 |  |  | PEAKS DB |
| K.VLEYLNDLK.Q | Y | 56.45 | 1105.6019 | 9 | 1.0 | 553.8088 | 2 | 25.14 | 1 | 13298 | BuCaMH04.raw | 4.2573E6 | 1 | 1 | 297 | 305 |  |  | PEAKS DB |
| total 3 peptides |  |  |  |  |  |  |  |  |  |  |  |  |  |  |  |  |  |  |  |
| --- | --- | --- | --- | --- | --- | --- | --- | --- | --- | --- | --- | --- | --- | --- | --- | --- | --- | --- | --- |

XP\_026575692.1

back to list

  

| Protein Coverage
| Supporting Peptides
|

Protein Coverage:

Supporting Peptides:

| Peptide | Uniq | -10lgP | Mass | Length | ppm | m/z | z | RT | Fraction | Scan | Source File | Area BuCaMH04\_B15 | #Feature | #Feature BuCaMH04\_B15 | Start | End | PTM | AScore | Found By |
| --- | --- | --- | --- | --- | --- | --- | --- | --- | --- | --- | --- | --- | --- | --- | --- | --- | --- | --- | --- |
| R.ATLLSPFFGTK.S | Y | 74.21 | 1180.6492 | 11 | 1.1 | 591.3325 | 2 | 39.06 | 1 | 25971 | BuCaMH04.raw | 1.4097E6 | 1 | 1 | 33 | 43 |  |  | PEAKS DB |
| A.TLLSPFFGTK.S | Y | 59.47 | 1109.6121 | 10 | 1.0 | 555.8138 | 2 | 37.16 | 1 | 24246 | BuCaMH04.raw | 8.7599E5 | 1 | 1 | 34 | 43 |  |  | PEAKS DB |
| K.VLEYLNDLK.Q | Y | 56.45 | 1105.6019 | 9 | 1.0 | 553.8088 | 2 | 25.14 | 1 | 13298 | BuCaMH04.raw | 4.2573E6 | 1 | 1 | 296 | 304 |  |  | PEAKS DB |
| total 3 peptides |  |  |  |  |  |  |  |  |  |  |  |  |  |  |  |  |  |  |  |
| --- | --- | --- | --- | --- | --- | --- | --- | --- | --- | --- | --- | --- | --- | --- | --- | --- | --- | --- | --- |

XP\_026575691.1

back to list

  

| Protein Coverage
| Supporting Peptides
|

Protein Coverage:

Supporting Peptides:

| Peptide | Uniq | -10lgP | Mass | Length | ppm | m/z | z | RT | Fraction | Scan | Source File | Area BuCaMH04\_B15 | #Feature | #Feature BuCaMH04\_B15 | Start | End | PTM | AScore | Found By |
| --- | --- | --- | --- | --- | --- | --- | --- | --- | --- | --- | --- | --- | --- | --- | --- | --- | --- | --- | --- |
| R.ATLLSPFFGTK.S | Y | 74.21 | 1180.6492 | 11 | 1.1 | 591.3325 | 2 | 39.06 | 1 | 25971 | BuCaMH04.raw | 1.4097E6 | 1 | 1 | 33 | 43 |  |  | PEAKS DB |
| A.TLLSPFFGTK.S | Y | 59.47 | 1109.6121 | 10 | 1.0 | 555.8138 | 2 | 37.16 | 1 | 24246 | BuCaMH04.raw | 8.7599E5 | 1 | 1 | 34 | 43 |  |  | PEAKS DB |
| K.VLEYLNDLK.Q | Y | 56.45 | 1105.6019 | 9 | 1.0 | 553.8088 | 2 | 25.14 | 1 | 13298 | BuCaMH04.raw | 4.2573E6 | 1 | 1 | 300 | 308 |  |  | PEAKS DB |
| total 3 peptides |  |  |  |  |  |  |  |  |  |  |  |  |  |  |  |  |  |  |  |
| --- | --- | --- | --- | --- | --- | --- | --- | --- | --- | --- | --- | --- | --- | --- | --- | --- | --- | --- | --- |

XP\_029139434.1

back to list

  

| Protein Coverage
| Supporting Peptides
|

Protein Coverage:

Supporting Peptides:

| Peptide | Uniq | -10lgP | Mass | Length | ppm | m/z | z | RT | Fraction | Scan | Source File | Area BuCaMH04\_B15 | #Feature | #Feature BuCaMH04\_B15 | Start | End | PTM | AScore | Found By |
| --- | --- | --- | --- | --- | --- | --- | --- | --- | --- | --- | --- | --- | --- | --- | --- | --- | --- | --- | --- |
| R.ATLLSPFFGTK.S | Y | 74.21 | 1180.6492 | 11 | 1.1 | 591.3325 | 2 | 39.06 | 1 | 25971 | BuCaMH04.raw | 1.4097E6 | 1 | 1 | 35 | 45 |  |  | PEAKS DB |
| A.TLLSPFFGTK.S | Y | 59.47 | 1109.6121 | 10 | 1.0 | 555.8138 | 2 | 37.16 | 1 | 24246 | BuCaMH04.raw | 8.7599E5 | 1 | 1 | 36 | 45 |  |  | PEAKS DB |
| K.VLEYLNDLK.Q | Y | 56.45 | 1105.6019 | 9 | 1.0 | 553.8088 | 2 | 25.14 | 1 | 13298 | BuCaMH04.raw | 4.2573E6 | 1 | 1 | 299 | 307 |  |  | PEAKS DB |
| total 3 peptides |  |  |  |  |  |  |  |  |  |  |  |  |  |  |  |  |  |  |  |
| --- | --- | --- | --- | --- | --- | --- | --- | --- | --- | --- | --- | --- | --- | --- | --- | --- | --- | --- | --- |

0702209A

back to list

  

| Protein Coverage
| Supporting Peptides
|

Protein Coverage:

Supporting Peptides:

| Peptide | Uniq | -10lgP | Mass | Length | ppm | m/z | z | RT | Fraction | Scan | Source File | Area BuCaMH04\_B15 | #Feature | #Feature BuCaMH04\_B15 | Start | End | PTM | AScore | Found By |
| --- | --- | --- | --- | --- | --- | --- | --- | --- | --- | --- | --- | --- | --- | --- | --- | --- | --- | --- | --- |
| Y.VNYGC(+57.02)YC(+57.02)GAGGSGTPVDELDR.C | Y | 84.31 | 2245.9368 | 21 | 0.6 | 1123.9763 | 2 | 21.56 | 1 | 10223 | BuCaMH04.raw | 3.9434E7 | 1 | 1 | 21 | 41 | Carbamidomethylation | C5:Carbamidomethylation:1000.00;C7:Carbamidomethylation:1000.00 | PEAKS DB |
| G.YVNYGC(+57.02)YC(+57.02)GAGGSGTPVDELDR.C | Y | 72.76 | 2409.0000 | 22 | 2.0 | 1205.5096 | 2 | 23.52 | 1 | 11852 | BuCaMH04.raw | 1.2139E7 | 1 | 1 | 20 | 41 | Carbamidomethylation | C6:Carbamidomethylation:1000.00;C8:Carbamidomethylation:1000.00 | PEAKS DB |
| total 2 peptides |  |  |  |  |  |  |  |  |  |  |  |  |  |  |  |  |  |  |  |
| --- | --- | --- | --- | --- | --- | --- | --- | --- | --- | --- | --- | --- | --- | --- | --- | --- | --- | --- | --- |

P00606.2

back to list

  

| Protein Coverage
| Supporting Peptides
|

Protein Coverage:

Supporting Peptides:

| Peptide | Uniq | -10lgP | Mass | Length | ppm | m/z | z | RT | Fraction | Scan | Source File | Area BuCaMH04\_B15 | #Feature | #Feature BuCaMH04\_B15 | Start | End | PTM | AScore | Found By |
| --- | --- | --- | --- | --- | --- | --- | --- | --- | --- | --- | --- | --- | --- | --- | --- | --- | --- | --- | --- |
| Y.VNYGC(+57.02)YC(+57.02)GAGGSGTPVDELDR.C | Y | 84.31 | 2245.9368 | 21 | 0.6 | 1123.9763 | 2 | 21.56 | 1 | 10223 | BuCaMH04.raw | 3.9434E7 | 1 | 1 | 48 | 68 | Carbamidomethylation | C5:Carbamidomethylation:1000.00;C7:Carbamidomethylation:1000.00 | PEAKS DB |
| G.YVNYGC(+57.02)YC(+57.02)GAGGSGTPVDELDR.C | Y | 72.76 | 2409.0000 | 22 | 2.0 | 1205.5096 | 2 | 23.52 | 1 | 11852 | BuCaMH04.raw | 1.2139E7 | 1 | 1 | 47 | 68 | Carbamidomethylation | C6:Carbamidomethylation:1000.00;C8:Carbamidomethylation:1000.00 | PEAKS DB |
| total 2 peptides |  |  |  |  |  |  |  |  |  |  |  |  |  |  |  |  |  |  |  |
| --- | --- | --- | --- | --- | --- | --- | --- | --- | --- | --- | --- | --- | --- | --- | --- | --- | --- | --- | --- |

CAA37482.1

back to list

  

| Protein Coverage
| Supporting Peptides
|

Protein Coverage:

Supporting Peptides:

| Peptide | Uniq | -10lgP | Mass | Length | ppm | m/z | z | RT | Fraction | Scan | Source File | Area BuCaMH04\_B15 | #Feature | #Feature BuCaMH04\_B15 | Start | End | PTM | AScore | Found By |
| --- | --- | --- | --- | --- | --- | --- | --- | --- | --- | --- | --- | --- | --- | --- | --- | --- | --- | --- | --- |
| Y.VNYGC(+57.02)YC(+57.02)GAGGSGTPVDELDR.C | Y | 84.31 | 2245.9368 | 21 | 0.6 | 1123.9763 | 2 | 21.56 | 1 | 10223 | BuCaMH04.raw | 3.9434E7 | 1 | 1 | 48 | 68 | Carbamidomethylation | C5:Carbamidomethylation:1000.00;C7:Carbamidomethylation:1000.00 | PEAKS DB |
| G.YVNYGC(+57.02)YC(+57.02)GAGGSGTPVDELDR.C | Y | 72.76 | 2409.0000 | 22 | 2.0 | 1205.5096 | 2 | 23.52 | 1 | 11852 | BuCaMH04.raw | 1.2139E7 | 1 | 1 | 47 | 68 | Carbamidomethylation | C6:Carbamidomethylation:1000.00;C8:Carbamidomethylation:1000.00 | PEAKS DB |
| total 2 peptides |  |  |  |  |  |  |  |  |  |  |  |  |  |  |  |  |  |  |  |
| --- | --- | --- | --- | --- | --- | --- | --- | --- | --- | --- | --- | --- | --- | --- | --- | --- | --- | --- | --- |

XP\_026541801.1

back to list

  

| Protein Coverage
| Supporting Peptides
|

Protein Coverage:

Supporting Peptides:

| Peptide | Uniq | -10lgP | Mass | Length | ppm | m/z | z | RT | Fraction | Scan | Source File | Area BuCaMH04\_B15 | #Feature | #Feature BuCaMH04\_B15 | Start | End | PTM | AScore | Found By |
| --- | --- | --- | --- | --- | --- | --- | --- | --- | --- | --- | --- | --- | --- | --- | --- | --- | --- | --- | --- |
| R.FEFAGDLVLPQNFDSR.Q | Y | 75.33 | 1853.8948 | 16 | 1.0 | 927.9556 | 2 | 44.19 | 1 | 30574 | BuCaMH04.raw | 5.2548E6 | 1 | 1 | 72 | 87 |  |  | PEAKS DB |
| R.NVPYFPPLSPDLVNYINK.L | Y | 53.72 | 2089.0884 | 18 | 1.4 | 1045.5530 | 2 | 49.56 | 1 | 35481 | BuCaMH04.raw | 1.6126E6 | 1 | 1 | 19 | 36 |  |  | PEAKS DB |
| K.NGPVEGAFTVYSDFLMYK.S | Y | 51.45 | 2036.9553 | 18 | 1.5 | 1019.4865 | 2 | 54.17 | 1 | 39394 | BuCaMH04.raw | 8.3839E5 | 1 | 1 | 247 | 264 |  |  | PEAKS DB |
| total 3 peptides |  |  |  |  |  |  |  |  |  |  |  |  |  |  |  |  |  |  |  |
| --- | --- | --- | --- | --- | --- | --- | --- | --- | --- | --- | --- | --- | --- | --- | --- | --- | --- | --- | --- |

XP\_026571757.1

back to list

  

| Protein Coverage
| Supporting Peptides
|

Protein Coverage:

Supporting Peptides:

| Peptide | Uniq | -10lgP | Mass | Length | ppm | m/z | z | RT | Fraction | Scan | Source File | Area BuCaMH04\_B15 | #Feature | #Feature BuCaMH04\_B15 | Start | End | PTM | AScore | Found By |
| --- | --- | --- | --- | --- | --- | --- | --- | --- | --- | --- | --- | --- | --- | --- | --- | --- | --- | --- | --- |
| R.FEFAGDLVLPQNFDSR.Q | Y | 75.33 | 1853.8948 | 16 | 1.0 | 927.9556 | 2 | 44.19 | 1 | 30574 | BuCaMH04.raw | 5.2548E6 | 1 | 1 | 72 | 87 |  |  | PEAKS DB |
| R.NVPYFPPLSPDLVNYINK.L | Y | 53.72 | 2089.0884 | 18 | 1.4 | 1045.5530 | 2 | 49.56 | 1 | 35481 | BuCaMH04.raw | 1.6126E6 | 1 | 1 | 19 | 36 |  |  | PEAKS DB |
| K.NGPVEGAFTVYSDFLMYK.S | Y | 51.45 | 2036.9553 | 18 | 1.5 | 1019.4865 | 2 | 54.17 | 1 | 39394 | BuCaMH04.raw | 8.3839E5 | 1 | 1 | 247 | 264 |  |  | PEAKS DB |
| total 3 peptides |  |  |  |  |  |  |  |  |  |  |  |  |  |  |  |  |  |  |  |
| --- | --- | --- | --- | --- | --- | --- | --- | --- | --- | --- | --- | --- | --- | --- | --- | --- | --- | --- | --- |

P25668.1

back to list

  

| Protein Coverage
| Supporting Peptides
|

Protein Coverage:

Supporting Peptides:

| Peptide | Uniq | -10lgP | Mass | Length | ppm | m/z | z | RT | Fraction | Scan | Source File | Area BuCaMH04\_B15 | #Feature | #Feature BuCaMH04\_B15 | Start | End | PTM | AScore | Found By |
| --- | --- | --- | --- | --- | --- | --- | --- | --- | --- | --- | --- | --- | --- | --- | --- | --- | --- | --- | --- |
| R.C(+57.02)FITPDITSK.D | N | 75.14 | 1180.5798 | 10 | 0.3 | 591.2974 | 2 | 43.30 | 1 | 30061 | BuCaMH04.raw | 1.3723E8 | 2 | 2 | 3 | 12 | Carbamidomethylation | C1:Carbamidomethylation:1000.00 | PEAKS DB |
| K.TWC(+57.02)DGFC(+57.02)SIR.G | Y | 55.24 | 1300.5328 | 10 | 0.8 | 651.2742 | 2 | 24.59 | 1 | 12843 | BuCaMH04.raw | 0 | 0 | 0 | 24 | 33 | Carbamidomethylation | C3:Carbamidomethylation:1000.00;C7:Carbamidomethylation:1000.00 | PEAKS DB |
| total 2 peptides |  |  |  |  |  |  |  |  |  |  |  |  |  |  |  |  |  |  |  |
| --- | --- | --- | --- | --- | --- | --- | --- | --- | --- | --- | --- | --- | --- | --- | --- | --- | --- | --- | --- |

764177A

back to list

  

| Protein Coverage
| Supporting Peptides
|

Protein Coverage:

Supporting Peptides:

| Peptide | Uniq | -10lgP | Mass | Length | ppm | m/z | z | RT | Fraction | Scan | Source File | Area BuCaMH04\_B15 | #Feature | #Feature BuCaMH04\_B15 | Start | End | PTM | AScore | Found By |
| --- | --- | --- | --- | --- | --- | --- | --- | --- | --- | --- | --- | --- | --- | --- | --- | --- | --- | --- | --- |
| R.C(+57.02)FITPDITSK.D | N | 75.14 | 1180.5798 | 10 | 0.3 | 591.2974 | 2 | 43.30 | 1 | 30061 | BuCaMH04.raw | 1.3723E8 | 2 | 2 | 3 | 12 | Carbamidomethylation | C1:Carbamidomethylation:1000.00 | PEAKS DB |
| K.TWC(+57.02)DGFC(+57.02)SSR.G | Y | 54.05 | 1274.4808 | 10 | 0.3 | 638.2479 | 2 | 16.85 | 1 | 6268 | BuCaMH04.raw | 9.9975E6 | 1 | 1 | 24 | 33 | Carbamidomethylation | C3:Carbamidomethylation:1000.00;C7:Carbamidomethylation:1000.00 | PEAKS DB |
| total 2 peptides |  |  |  |  |  |  |  |  |  |  |  |  |  |  |  |  |  |  |  |
| --- | --- | --- | --- | --- | --- | --- | --- | --- | --- | --- | --- | --- | --- | --- | --- | --- | --- | --- | --- |

P25669.1

back to list

  

| Protein Coverage
| Supporting Peptides
|

Protein Coverage:

Supporting Peptides:

| Peptide | Uniq | -10lgP | Mass | Length | ppm | m/z | z | RT | Fraction | Scan | Source File | Area BuCaMH04\_B15 | #Feature | #Feature BuCaMH04\_B15 | Start | End | PTM | AScore | Found By |
| --- | --- | --- | --- | --- | --- | --- | --- | --- | --- | --- | --- | --- | --- | --- | --- | --- | --- | --- | --- |
| R.C(+57.02)FITPDITSK.D | N | 75.14 | 1180.5798 | 10 | 0.3 | 591.2974 | 2 | 43.30 | 1 | 30061 | BuCaMH04.raw | 1.3723E8 | 2 | 2 | 3 | 12 | Carbamidomethylation | C1:Carbamidomethylation:1000.00 | PEAKS DB |
| K.TWC(+57.02)DGFC(+57.02)SSR.G | Y | 54.05 | 1274.4808 | 10 | 0.3 | 638.2479 | 2 | 16.85 | 1 | 6268 | BuCaMH04.raw | 9.9975E6 | 1 | 1 | 24 | 33 | Carbamidomethylation | C3:Carbamidomethylation:1000.00;C7:Carbamidomethylation:1000.00 | PEAKS DB |
| total 2 peptides |  |  |  |  |  |  |  |  |  |  |  |  |  |  |  |  |  |  |  |
| --- | --- | --- | --- | --- | --- | --- | --- | --- | --- | --- | --- | --- | --- | --- | --- | --- | --- | --- | --- |

D5LMJ3.1

back to list

  

| Protein Coverage
| Supporting Peptides
|

Protein Coverage:

Supporting Peptides:

| Peptide | Uniq | -10lgP | Mass | Length | ppm | m/z | z | RT | Fraction | Scan | Source File | Area BuCaMH04\_B15 | #Feature | #Feature BuCaMH04\_B15 | Start | End | PTM | AScore | Found By |
| --- | --- | --- | --- | --- | --- | --- | --- | --- | --- | --- | --- | --- | --- | --- | --- | --- | --- | --- | --- |
| K.YIEFYLVVDNK.M | Y | 69.21 | 1401.7180 | 11 | 0.2 | 701.8665 | 2 | 38.64 | 1 | 25956 | BuCaMH04.raw | 6.7811E6 | 1 | 1 | 206 | 216 |  |  | PEAKS DB |
| Y.IEFYLVVDNK.M | Y | 61.45 | 1238.6547 | 10 | -0.3 | 620.3344 | 2 | 32.85 | 1 | 20604 | BuCaMH04.raw | 3.6696E6 | 1 | 1 | 207 | 216 |  |  | PEAKS DB |
| total 2 peptides |  |  |  |  |  |  |  |  |  |  |  |  |  |  |  |  |  |  |  |
| --- | --- | --- | --- | --- | --- | --- | --- | --- | --- | --- | --- | --- | --- | --- | --- | --- | --- | --- | --- |

ADF43026.1

back to list

  

| Protein Coverage
| Supporting Peptides
|

Protein Coverage:

Supporting Peptides:

| Peptide | Uniq | -10lgP | Mass | Length | ppm | m/z | z | RT | Fraction | Scan | Source File | Area BuCaMH04\_B15 | #Feature | #Feature BuCaMH04\_B15 | Start | End | PTM | AScore | Found By |
| --- | --- | --- | --- | --- | --- | --- | --- | --- | --- | --- | --- | --- | --- | --- | --- | --- | --- | --- | --- |
| K.YIEFYLVVDNK.M | Y | 69.21 | 1401.7180 | 11 | 0.2 | 701.8665 | 2 | 38.64 | 1 | 25956 | BuCaMH04.raw | 6.7811E6 | 1 | 1 | 206 | 216 |  |  | PEAKS DB |
| Y.IEFYLVVDNK.M | Y | 61.45 | 1238.6547 | 10 | -0.3 | 620.3344 | 2 | 32.85 | 1 | 20604 | BuCaMH04.raw | 3.6696E6 | 1 | 1 | 207 | 216 |  |  | PEAKS DB |
| total 2 peptides |  |  |  |  |  |  |  |  |  |  |  |  |  |  |  |  |  |  |  |
| --- | --- | --- | --- | --- | --- | --- | --- | --- | --- | --- | --- | --- | --- | --- | --- | --- | --- | --- | --- |

AAO84769.1

back to list

  

| Protein Coverage
| Supporting Peptides
|

Protein Coverage:

Supporting Peptides:

| Peptide | Uniq | -10lgP | Mass | Length | ppm | m/z | z | RT | Fraction | Scan | Source File | Area BuCaMH04\_B15 | #Feature | #Feature BuCaMH04\_B15 | Start | End | PTM | AScore | Found By |
| --- | --- | --- | --- | --- | --- | --- | --- | --- | --- | --- | --- | --- | --- | --- | --- | --- | --- | --- | --- |
| R.TAALC(+57.02)FAEAPYK.R | Y | 80.68 | 1340.6434 | 12 | -1.7 | 671.3278 | 2 | 21.96 | 1 | 10609 | BuCaMH04.raw | 7.4805E5 | 1 | 1 | 118 | 129 | Carbamidomethylation | C5:Carbamidomethylation:1000.00 | PEAKS DB |
| K.C(+57.02)SSLLNVPYVK.Q | Y | 69.39 | 1278.6642 | 11 | 0.7 | 640.3398 | 2 | 24.31 | 1 | 12564 | BuCaMH04.raw | 1.9544E7 | 1 | 1 | 78 | 88 | Carbamidomethylation | C1:Carbamidomethylation:1000.00 | PEAKS DB |
| total 2 peptides |  |  |  |  |  |  |  |  |  |  |  |  |  |  |  |  |  |  |  |
| --- | --- | --- | --- | --- | --- | --- | --- | --- | --- | --- | --- | --- | --- | --- | --- | --- | --- | --- | --- |

Q802I1.1

back to list

  

| Protein Coverage
| Supporting Peptides
|

Protein Coverage:

Supporting Peptides:

| Peptide | Uniq | -10lgP | Mass | Length | ppm | m/z | z | RT | Fraction | Scan | Source File | Area BuCaMH04\_B15 | #Feature | #Feature BuCaMH04\_B15 | Start | End | PTM | AScore | Found By |
| --- | --- | --- | --- | --- | --- | --- | --- | --- | --- | --- | --- | --- | --- | --- | --- | --- | --- | --- | --- |
| R.TAALC(+57.02)FAEAPYK.R | Y | 80.68 | 1340.6434 | 12 | -1.7 | 671.3278 | 2 | 21.96 | 1 | 10609 | BuCaMH04.raw | 7.4805E5 | 1 | 1 | 118 | 129 | Carbamidomethylation | C5:Carbamidomethylation:1000.00 | PEAKS DB |
| K.C(+57.02)SSLLNVPYVK.Q | Y | 69.39 | 1278.6642 | 11 | 0.7 | 640.3398 | 2 | 24.31 | 1 | 12564 | BuCaMH04.raw | 1.9544E7 | 1 | 1 | 78 | 88 | Carbamidomethylation | C1:Carbamidomethylation:1000.00 | PEAKS DB |
| total 2 peptides |  |  |  |  |  |  |  |  |  |  |  |  |  |  |  |  |  |  |  |
| --- | --- | --- | --- | --- | --- | --- | --- | --- | --- | --- | --- | --- | --- | --- | --- | --- | --- | --- | --- |

XP\_007420422.1

back to list

  

| Protein Coverage
| Supporting Peptides
|

Protein Coverage:

Supporting Peptides:

| Peptide | Uniq | -10lgP | Mass | Length | ppm | m/z | z | RT | Fraction | Scan | Source File | Area BuCaMH04\_B15 | #Feature | #Feature BuCaMH04\_B15 | Start | End | PTM | AScore | Found By |
| --- | --- | --- | --- | --- | --- | --- | --- | --- | --- | --- | --- | --- | --- | --- | --- | --- | --- | --- | --- |
| K.HSDSNAFLHLFP.E | N | 59.60 | 1383.6571 | 12 | 0.1 | 462.2264 | 3 | 33.71 | 1 | 21163 | BuCaMH04.raw | 1.1569E6 | 1 | 1 | 380 | 391 |  |  | PEAKS DB |
| K.DYALPVFVYAR.P | N | 59.05 | 1312.6815 | 11 | 0.3 | 657.3482 | 2 | 43.46 | 1 | 30072 | BuCaMH04.raw | 5.4049E6 | 1 | 1 | 285 | 295 |  |  | PEAKS DB |
| total 2 peptides |  |  |  |  |  |  |  |  |  |  |  |  |  |  |  |  |  |  |  |
| --- | --- | --- | --- | --- | --- | --- | --- | --- | --- | --- | --- | --- | --- | --- | --- | --- | --- | --- | --- |

XP\_026555558.1

back to list

  

| Protein Coverage
| Supporting Peptides
|

Protein Coverage:

Supporting Peptides:

| Peptide | Uniq | -10lgP | Mass | Length | ppm | m/z | z | RT | Fraction | Scan | Source File | Area BuCaMH04\_B15 | #Feature | #Feature BuCaMH04\_B15 | Start | End | PTM | AScore | Found By |
| --- | --- | --- | --- | --- | --- | --- | --- | --- | --- | --- | --- | --- | --- | --- | --- | --- | --- | --- | --- |
| R.SPDEDEQPWC(+57.02)YFIK.D | Y | 62.65 | 1812.7665 | 14 | -0.7 | 907.3899 | 2 | 43.52 | 1 | 29717 | BuCaMH04.raw | 7.8543E6 | 1 | 1 | 235 | 248 | Carbamidomethylation | C10:Carbamidomethylation:1000.00 | PEAKS DB |
| total 1 peptides |  |  |  |  |  |  |  |  |  |  |  |  |  |  |  |  |  |  |  |
| --- | --- | --- | --- | --- | --- | --- | --- | --- | --- | --- | --- | --- | --- | --- | --- | --- | --- | --- | --- |

CAB62385.1

back to list

  

| Protein Coverage
| Supporting Peptides
|

Protein Coverage:

Supporting Peptides:

| Peptide | Uniq | -10lgP | Mass | Length | ppm | m/z | z | RT | Fraction | Scan | Source File | Area BuCaMH04\_B15 | #Feature | #Feature BuCaMH04\_B15 | Start | End | PTM | AScore | Found By |
| --- | --- | --- | --- | --- | --- | --- | --- | --- | --- | --- | --- | --- | --- | --- | --- | --- | --- | --- | --- |
| R.TAALC(+57.02)FGDSEYIEGHK.N | N | 73.24 | 1796.8040 | 16 | -1.5 | 599.9410 | 3 | 19.83 | 1 | 8688 | BuCaMH04.raw | 3.584E7 | 1 | 1 | 113 | 128 | Carbamidomethylation | C5:Carbamidomethylation:1000.00 | PEAKS DB |
| L.NLINFMEMIR.Y | N | 64.70 | 1279.6417 | 10 | -0.1 | 640.8281 | 2 | 50.79 | 1 | 36537 | BuCaMH04.raw | 7.4404E7 | 1 | 1 | 18 | 27 |  |  | PEAKS DB |
| total 2 peptides |  |  |  |  |  |  |  |  |  |  |  |  |  |  |  |  |  |  |  |
| --- | --- | --- | --- | --- | --- | --- | --- | --- | --- | --- | --- | --- | --- | --- | --- | --- | --- | --- | --- |

Q6YNQ0.1

back to list

  

| Protein Coverage
| Supporting Peptides
|

Protein Coverage:

Supporting Peptides:

| Peptide | Uniq | -10lgP | Mass | Length | ppm | m/z | z | RT | Fraction | Scan | Source File | Area BuCaMH04\_B15 | #Feature | #Feature BuCaMH04\_B15 | Start | End | PTM | AScore | Found By |
| --- | --- | --- | --- | --- | --- | --- | --- | --- | --- | --- | --- | --- | --- | --- | --- | --- | --- | --- | --- |
| R.TAALC(+57.02)FGDSEYIEGHK.N | N | 73.24 | 1796.8040 | 16 | -1.5 | 599.9410 | 3 | 19.83 | 1 | 8688 | BuCaMH04.raw | 3.584E7 | 1 | 1 | 113 | 128 | Carbamidomethylation | C5:Carbamidomethylation:1000.00 | PEAKS DB |
| L.NLINFMEMIR.Y | N | 64.70 | 1279.6417 | 10 | -0.1 | 640.8281 | 2 | 50.79 | 1 | 36537 | BuCaMH04.raw | 7.4404E7 | 1 | 1 | 18 | 27 |  |  | PEAKS DB |
| total 2 peptides |  |  |  |  |  |  |  |  |  |  |  |  |  |  |  |  |  |  |  |
| --- | --- | --- | --- | --- | --- | --- | --- | --- | --- | --- | --- | --- | --- | --- | --- | --- | --- | --- | --- |

AAL30064.1

back to list

  

| Protein Coverage
| Supporting Peptides
|

Protein Coverage:

Supporting Peptides:

| Peptide | Uniq | -10lgP | Mass | Length | ppm | m/z | z | RT | Fraction | Scan | Source File | Area BuCaMH04\_B15 | #Feature | #Feature BuCaMH04\_B15 | Start | End | PTM | AScore | Found By |
| --- | --- | --- | --- | --- | --- | --- | --- | --- | --- | --- | --- | --- | --- | --- | --- | --- | --- | --- | --- |
| R.TAALC(+57.02)FGDSEYIEGHK.N | N | 73.24 | 1796.8040 | 16 | -1.5 | 599.9410 | 3 | 19.83 | 1 | 8688 | BuCaMH04.raw | 3.584E7 | 1 | 1 | 113 | 128 | Carbamidomethylation | C5:Carbamidomethylation:1000.00 | PEAKS DB |
| L.NLINFMEMIR.Y | N | 64.70 | 1279.6417 | 10 | -0.1 | 640.8281 | 2 | 50.79 | 1 | 36537 | BuCaMH04.raw | 7.4404E7 | 1 | 1 | 18 | 27 |  |  | PEAKS DB |
| total 2 peptides |  |  |  |  |  |  |  |  |  |  |  |  |  |  |  |  |  |  |  |
| --- | --- | --- | --- | --- | --- | --- | --- | --- | --- | --- | --- | --- | --- | --- | --- | --- | --- | --- | --- |

P00619.2

back to list

  

| Protein Coverage
| Supporting Peptides
|

Protein Coverage:

Supporting Peptides:

| Peptide | Uniq | -10lgP | Mass | Length | ppm | m/z | z | RT | Fraction | Scan | Source File | Area BuCaMH04\_B15 | #Feature | #Feature BuCaMH04\_B15 | Start | End | PTM | AScore | Found By |
| --- | --- | --- | --- | --- | --- | --- | --- | --- | --- | --- | --- | --- | --- | --- | --- | --- | --- | --- | --- |
| R.TAALC(+57.02)FGDSEYIEGHK.N | N | 73.24 | 1796.8040 | 16 | -1.5 | 599.9410 | 3 | 19.83 | 1 | 8688 | BuCaMH04.raw | 3.584E7 | 1 | 1 | 123 | 138 | Carbamidomethylation | C5:Carbamidomethylation:1000.00 | PEAKS DB |
| L.NLINFMEMIR.Y | N | 64.70 | 1279.6417 | 10 | -0.1 | 640.8281 | 2 | 50.79 | 1 | 36537 | BuCaMH04.raw | 7.4404E7 | 1 | 1 | 28 | 37 |  |  | PEAKS DB |
| total 2 peptides |  |  |  |  |  |  |  |  |  |  |  |  |  |  |  |  |  |  |  |
| --- | --- | --- | --- | --- | --- | --- | --- | --- | --- | --- | --- | --- | --- | --- | --- | --- | --- | --- | --- |

CAC08197.1

back to list

  

| Protein Coverage
| Supporting Peptides
|

Protein Coverage:

Supporting Peptides:

| Peptide | Uniq | -10lgP | Mass | Length | ppm | m/z | z | RT | Fraction | Scan | Source File | Area BuCaMH04\_B15 | #Feature | #Feature BuCaMH04\_B15 | Start | End | PTM | AScore | Found By |
| --- | --- | --- | --- | --- | --- | --- | --- | --- | --- | --- | --- | --- | --- | --- | --- | --- | --- | --- | --- |
| R.TAALC(+57.02)FGDSEYIEGHK.N | N | 73.24 | 1796.8040 | 16 | -1.5 | 599.9410 | 3 | 19.83 | 1 | 8688 | BuCaMH04.raw | 3.584E7 | 1 | 1 | 123 | 138 | Carbamidomethylation | C5:Carbamidomethylation:1000.00 | PEAKS DB |
| L.NLINFMEMIR.Y | N | 64.70 | 1279.6417 | 10 | -0.1 | 640.8281 | 2 | 50.79 | 1 | 36537 | BuCaMH04.raw | 7.4404E7 | 1 | 1 | 28 | 37 |  |  | PEAKS DB |
| total 2 peptides |  |  |  |  |  |  |  |  |  |  |  |  |  |  |  |  |  |  |  |
| --- | --- | --- | --- | --- | --- | --- | --- | --- | --- | --- | --- | --- | --- | --- | --- | --- | --- | --- | --- |

Q6YIJ3.1

back to list

  

| Protein Coverage
| Supporting Peptides
|

Protein Coverage:

Supporting Peptides:

| Peptide | Uniq | -10lgP | Mass | Length | ppm | m/z | z | RT | Fraction | Scan | Source File | Area BuCaMH04\_B15 | #Feature | #Feature BuCaMH04\_B15 | Start | End | PTM | AScore | Found By |
| --- | --- | --- | --- | --- | --- | --- | --- | --- | --- | --- | --- | --- | --- | --- | --- | --- | --- | --- | --- |
| R.TAALC(+57.02)FGDSEYIEGHK.N | N | 73.24 | 1796.8040 | 16 | -1.5 | 599.9410 | 3 | 19.83 | 1 | 8688 | BuCaMH04.raw | 3.584E7 | 1 | 1 | 113 | 128 | Carbamidomethylation | C5:Carbamidomethylation:1000.00 | PEAKS DB |
| L.NLINFMEMIR.Y | N | 64.70 | 1279.6417 | 10 | -0.1 | 640.8281 | 2 | 50.79 | 1 | 36537 | BuCaMH04.raw | 7.4404E7 | 1 | 1 | 18 | 27 |  |  | PEAKS DB |
| total 2 peptides |  |  |  |  |  |  |  |  |  |  |  |  |  |  |  |  |  |  |  |
| --- | --- | --- | --- | --- | --- | --- | --- | --- | --- | --- | --- | --- | --- | --- | --- | --- | --- | --- | --- |

AAN16113.1

back to list

  

| Protein Coverage
| Supporting Peptides
|

Protein Coverage:

Supporting Peptides:

| Peptide | Uniq | -10lgP | Mass | Length | ppm | m/z | z | RT | Fraction | Scan | Source File | Area BuCaMH04\_B15 | #Feature | #Feature BuCaMH04\_B15 | Start | End | PTM | AScore | Found By |
| --- | --- | --- | --- | --- | --- | --- | --- | --- | --- | --- | --- | --- | --- | --- | --- | --- | --- | --- | --- |
| R.TAALC(+57.02)FGDSEYIEGHK.N | N | 73.24 | 1796.8040 | 16 | -1.5 | 599.9410 | 3 | 19.83 | 1 | 8688 | BuCaMH04.raw | 3.584E7 | 1 | 1 | 113 | 128 | Carbamidomethylation | C5:Carbamidomethylation:1000.00 | PEAKS DB |
| L.NLINFMEMIR.Y | N | 64.70 | 1279.6417 | 10 | -0.1 | 640.8281 | 2 | 50.79 | 1 | 36537 | BuCaMH04.raw | 7.4404E7 | 1 | 1 | 18 | 27 |  |  | PEAKS DB |
| total 2 peptides |  |  |  |  |  |  |  |  |  |  |  |  |  |  |  |  |  |  |  |
| --- | --- | --- | --- | --- | --- | --- | --- | --- | --- | --- | --- | --- | --- | --- | --- | --- | --- | --- | --- |

ETE68500.1

back to list

  

| Protein Coverage
| Supporting Peptides
|

Protein Coverage:

Supporting Peptides:

| Peptide | Uniq | -10lgP | Mass | Length | ppm | m/z | z | RT | Fraction | Scan | Source File | Area BuCaMH04\_B15 | #Feature | #Feature BuCaMH04\_B15 | Start | End | PTM | AScore | Found By |
| --- | --- | --- | --- | --- | --- | --- | --- | --- | --- | --- | --- | --- | --- | --- | --- | --- | --- | --- | --- |
| total 0 peptides |  |  |  |  |  |  |  |  |  |  |  |  |  |  |  |  |  |  |  |
| --- | --- | --- | --- | --- | --- | --- | --- | --- | --- | --- | --- | --- | --- | --- | --- | --- | --- | --- | --- |

P25672.1

back to list

  

| Protein Coverage
| Supporting Peptides
|

Protein Coverage:

Supporting Peptides:

| Peptide | Uniq | -10lgP | Mass | Length | ppm | m/z | z | RT | Fraction | Scan | Source File | Area BuCaMH04\_B15 | #Feature | #Feature BuCaMH04\_B15 | Start | End | PTM | AScore | Found By |
| --- | --- | --- | --- | --- | --- | --- | --- | --- | --- | --- | --- | --- | --- | --- | --- | --- | --- | --- | --- |
| R.C(+57.02)FITPDITSK.D | N | 75.14 | 1180.5798 | 10 | 0.3 | 591.2974 | 2 | 43.30 | 1 | 30061 | BuCaMH04.raw | 1.3723E8 | 2 | 2 | 3 | 12 | Carbamidomethylation | C1:Carbamidomethylation:1000.00 | PEAKS DB |
| total 1 peptides |  |  |  |  |  |  |  |  |  |  |  |  |  |  |  |  |  |  |  |
| --- | --- | --- | --- | --- | --- | --- | --- | --- | --- | --- | --- | --- | --- | --- | --- | --- | --- | --- | --- |

ETE70042.1

back to list

  

| Protein Coverage
| Supporting Peptides
|

Protein Coverage:

Supporting Peptides:

| Peptide | Uniq | -10lgP | Mass | Length | ppm | m/z | z | RT | Fraction | Scan | Source File | Area BuCaMH04\_B15 | #Feature | #Feature BuCaMH04\_B15 | Start | End | PTM | AScore | Found By |
| --- | --- | --- | --- | --- | --- | --- | --- | --- | --- | --- | --- | --- | --- | --- | --- | --- | --- | --- | --- |
| L.GLYSPDDPLVELDTNTLER.R | Y | 95.37 | 2146.0430 | 19 | 0.3 | 1074.0291 | 2 | 41.48 | 1 | 28280 | BuCaMH04.raw | 1.2003E7 | 1 | 1 | 25 | 43 |  |  | PEAKS DB |
| total 1 peptides |  |  |  |  |  |  |  |  |  |  |  |  |  |  |  |  |  |  |  |
| --- | --- | --- | --- | --- | --- | --- | --- | --- | --- | --- | --- | --- | --- | --- | --- | --- | --- | --- | --- |

S59517

back to list

  

| Protein Coverage
| Supporting Peptides
|

Protein Coverage:

Supporting Peptides:

| Peptide | Uniq | -10lgP | Mass | Length | ppm | m/z | z | RT | Fraction | Scan | Source File | Area BuCaMH04\_B15 | #Feature | #Feature BuCaMH04\_B15 | Start | End | PTM | AScore | Found By |
| --- | --- | --- | --- | --- | --- | --- | --- | --- | --- | --- | --- | --- | --- | --- | --- | --- | --- | --- | --- |
| R.DSVLAQYIFELSR.R | Y | 65.72 | 1539.7932 | 13 | 0.1 | 770.9040 | 2 | 56.75 | 1 | 41632 | BuCaMH04.raw | 1.1252E5 | 1 | 1 | 160 | 172 |  |  | PEAKS DB |
| K.ESFLFTLTR.N | Y | 58.69 | 1112.5865 | 9 | 0.9 | 557.3010 | 2 | 37.50 | 1 | 24683 | BuCaMH04.raw | 5.6755E5 | 1 | 1 | 356 | 364 |  |  | PEAKS DB |
| total 2 peptides |  |  |  |  |  |  |  |  |  |  |  |  |  |  |  |  |  |  |  |
| --- | --- | --- | --- | --- | --- | --- | --- | --- | --- | --- | --- | --- | --- | --- | --- | --- | --- | --- | --- |

CAA55333.1

back to list

  

| Protein Coverage
| Supporting Peptides
|

Protein Coverage:

Supporting Peptides:

| Peptide | Uniq | -10lgP | Mass | Length | ppm | m/z | z | RT | Fraction | Scan | Source File | Area BuCaMH04\_B15 | #Feature | #Feature BuCaMH04\_B15 | Start | End | PTM | AScore | Found By |
| --- | --- | --- | --- | --- | --- | --- | --- | --- | --- | --- | --- | --- | --- | --- | --- | --- | --- | --- | --- |
| R.DSVLAQYIFELSR.R | Y | 65.72 | 1539.7932 | 13 | 0.1 | 770.9040 | 2 | 56.75 | 1 | 41632 | BuCaMH04.raw | 1.1252E5 | 1 | 1 | 160 | 172 |  |  | PEAKS DB |
| K.ESFLFTLTR.N | Y | 58.69 | 1112.5865 | 9 | 0.9 | 557.3010 | 2 | 37.50 | 1 | 24683 | BuCaMH04.raw | 5.6755E5 | 1 | 1 | 356 | 364 |  |  | PEAKS DB |
| total 2 peptides |  |  |  |  |  |  |  |  |  |  |  |  |  |  |  |  |  |  |  |
| --- | --- | --- | --- | --- | --- | --- | --- | --- | --- | --- | --- | --- | --- | --- | --- | --- | --- | --- | --- |

XP\_034296607.1

back to list

  

| Protein Coverage
| Supporting Peptides
|

Protein Coverage:

Supporting Peptides:

| Peptide | Uniq | -10lgP | Mass | Length | ppm | m/z | z | RT | Fraction | Scan | Source File | Area BuCaMH04\_B15 | #Feature | #Feature BuCaMH04\_B15 | Start | End | PTM | AScore | Found By |
| --- | --- | --- | --- | --- | --- | --- | --- | --- | --- | --- | --- | --- | --- | --- | --- | --- | --- | --- | --- |
| total 0 peptides |  |  |  |  |  |  |  |  |  |  |  |  |  |  |  |  |  |  |  |
| --- | --- | --- | --- | --- | --- | --- | --- | --- | --- | --- | --- | --- | --- | --- | --- | --- | --- | --- | --- |

XP\_034296605.1

back to list

  

| Protein Coverage
| Supporting Peptides
|

Protein Coverage:

Supporting Peptides:

| Peptide | Uniq | -10lgP | Mass | Length | ppm | m/z | z | RT | Fraction | Scan | Source File | Area BuCaMH04\_B15 | #Feature | #Feature BuCaMH04\_B15 | Start | End | PTM | AScore | Found By |
| --- | --- | --- | --- | --- | --- | --- | --- | --- | --- | --- | --- | --- | --- | --- | --- | --- | --- | --- | --- |
| total 0 peptides |  |  |  |  |  |  |  |  |  |  |  |  |  |  |  |  |  |  |  |
| --- | --- | --- | --- | --- | --- | --- | --- | --- | --- | --- | --- | --- | --- | --- | --- | --- | --- | --- | --- |

XP\_034296606.1

back to list

  

| Protein Coverage
| Supporting Peptides
|

Protein Coverage:

Supporting Peptides:

| Peptide | Uniq | -10lgP | Mass | Length | ppm | m/z | z | RT | Fraction | Scan | Source File | Area BuCaMH04\_B15 | #Feature | #Feature BuCaMH04\_B15 | Start | End | PTM | AScore | Found By |
| --- | --- | --- | --- | --- | --- | --- | --- | --- | --- | --- | --- | --- | --- | --- | --- | --- | --- | --- | --- |
| total 0 peptides |  |  |  |  |  |  |  |  |  |  |  |  |  |  |  |  |  |  |  |
| --- | --- | --- | --- | --- | --- | --- | --- | --- | --- | --- | --- | --- | --- | --- | --- | --- | --- | --- | --- |

1702215A

back to list

  

| Protein Coverage
| Supporting Peptides
|

Protein Coverage:

Supporting Peptides:

| Peptide | Uniq | -10lgP | Mass | Length | ppm | m/z | z | RT | Fraction | Scan | Source File | Area BuCaMH04\_B15 | #Feature | #Feature BuCaMH04\_B15 | Start | End | PTM | AScore | Found By |
| --- | --- | --- | --- | --- | --- | --- | --- | --- | --- | --- | --- | --- | --- | --- | --- | --- | --- | --- | --- |
| R.FC(+57.02)ELAPSAGSC(+57.02)FGFVSSYYYNR.Y | Y | 67.19 | 2581.1042 | 22 | -0.1 | 1291.5593 | 2 | 42.72 | 1 | 29235 | BuCaMH04.raw | 7.6924E6 | 2 | 2 | 4 | 25 | Carbamidomethylation | C2:Carbamidomethylation:1000.00;C11:Carbamidomethylation:1000.00 | PEAKS DB |
| R.FC(+57.02)ELAPSAGSC(+57.02)FG.F | Y | 53.31 | 1401.5693 | 13 | 0.4 | 701.7922 | 2 | 30.03 | 1 | 17809 | BuCaMH04.raw | 3.4249E7 | 1 | 1 | 4 | 16 | Carbamidomethylation | C2:Carbamidomethylation:1000.00;C11:Carbamidomethylation:1000.00 | PEAKS DB |
| total 2 peptides |  |  |  |  |  |  |  |  |  |  |  |  |  |  |  |  |  |  |  |
| --- | --- | --- | --- | --- | --- | --- | --- | --- | --- | --- | --- | --- | --- | --- | --- | --- | --- | --- | --- |

P19859.1

back to list

  

| Protein Coverage
| Supporting Peptides
|

Protein Coverage:

Supporting Peptides:

| Peptide | Uniq | -10lgP | Mass | Length | ppm | m/z | z | RT | Fraction | Scan | Source File | Area BuCaMH04\_B15 | #Feature | #Feature BuCaMH04\_B15 | Start | End | PTM | AScore | Found By |
| --- | --- | --- | --- | --- | --- | --- | --- | --- | --- | --- | --- | --- | --- | --- | --- | --- | --- | --- | --- |
| R.FC(+57.02)ELAPSAGSC(+57.02)FGFVSSYYYNR.Y | Y | 67.19 | 2581.1042 | 22 | -0.1 | 1291.5593 | 2 | 42.72 | 1 | 29235 | BuCaMH04.raw | 7.6924E6 | 2 | 2 | 4 | 25 | Carbamidomethylation | C2:Carbamidomethylation:1000.00;C11:Carbamidomethylation:1000.00 | PEAKS DB |
| R.FC(+57.02)ELAPSAGSC(+57.02)FG.F | Y | 53.31 | 1401.5693 | 13 | 0.4 | 701.7922 | 2 | 30.03 | 1 | 17809 | BuCaMH04.raw | 3.4249E7 | 1 | 1 | 4 | 16 | Carbamidomethylation | C2:Carbamidomethylation:1000.00;C11:Carbamidomethylation:1000.00 | PEAKS DB |
| total 2 peptides |  |  |  |  |  |  |  |  |  |  |  |  |  |  |  |  |  |  |  |
| --- | --- | --- | --- | --- | --- | --- | --- | --- | --- | --- | --- | --- | --- | --- | --- | --- | --- | --- | --- |

XP\_026553321.1

back to list

  

| Protein Coverage
| Supporting Peptides
|

Protein Coverage:

Supporting Peptides:

| Peptide | Uniq | -10lgP | Mass | Length | ppm | m/z | z | RT | Fraction | Scan | Source File | Area BuCaMH04\_B15 | #Feature | #Feature BuCaMH04\_B15 | Start | End | PTM | AScore | Found By |
| --- | --- | --- | --- | --- | --- | --- | --- | --- | --- | --- | --- | --- | --- | --- | --- | --- | --- | --- | --- |
| A.DLHYATVYWLEAEK.S | N | 53.99 | 1736.8409 | 14 | 0.4 | 579.9545 | 3 | 35.21 | 1 | 22530 | BuCaMH04.raw | 9.7317E5 | 1 | 1 | 37 | 50 |  |  | PEAKS DB |
| total 1 peptides |  |  |  |  |  |  |  |  |  |  |  |  |  |  |  |  |  |  |  |
| --- | --- | --- | --- | --- | --- | --- | --- | --- | --- | --- | --- | --- | --- | --- | --- | --- | --- | --- | --- |

F8J2D3.1

back to list

  

| Protein Coverage
| Supporting Peptides
|

Protein Coverage:

Supporting Peptides:

| Peptide | Uniq | -10lgP | Mass | Length | ppm | m/z | z | RT | Fraction | Scan | Source File | Area BuCaMH04\_B15 | #Feature | #Feature BuCaMH04\_B15 | Start | End | PTM | AScore | Found By |
| --- | --- | --- | --- | --- | --- | --- | --- | --- | --- | --- | --- | --- | --- | --- | --- | --- | --- | --- | --- |
| A.DLHYATVYWLEAEK.S | N | 53.99 | 1736.8409 | 14 | 0.4 | 579.9545 | 3 | 35.21 | 1 | 22530 | BuCaMH04.raw | 9.7317E5 | 1 | 1 | 37 | 50 |  |  | PEAKS DB |
| total 1 peptides |  |  |  |  |  |  |  |  |  |  |  |  |  |  |  |  |  |  |  |
| --- | --- | --- | --- | --- | --- | --- | --- | --- | --- | --- | --- | --- | --- | --- | --- | --- | --- | --- | --- |

ACR78473.1

back to list

  

| Protein Coverage
| Supporting Peptides
|

Protein Coverage:

Supporting Peptides:

| Peptide | Uniq | -10lgP | Mass | Length | ppm | m/z | z | RT | Fraction | Scan | Source File | Area BuCaMH04\_B15 | #Feature | #Feature BuCaMH04\_B15 | Start | End | PTM | AScore | Found By |
| --- | --- | --- | --- | --- | --- | --- | --- | --- | --- | --- | --- | --- | --- | --- | --- | --- | --- | --- | --- |
| A.DLHYATVYWLEAEK.S | N | 53.99 | 1736.8409 | 14 | 0.4 | 579.9545 | 3 | 35.21 | 1 | 22530 | BuCaMH04.raw | 9.7317E5 | 1 | 1 | 37 | 50 |  |  | PEAKS DB |
| total 1 peptides |  |  |  |  |  |  |  |  |  |  |  |  |  |  |  |  |  |  |  |
| --- | --- | --- | --- | --- | --- | --- | --- | --- | --- | --- | --- | --- | --- | --- | --- | --- | --- | --- | --- |

Q9W717.1

back to list

  

| Protein Coverage
| Supporting Peptides
|

Protein Coverage:

Supporting Peptides:

| Peptide | Uniq | -10lgP | Mass | Length | ppm | m/z | z | RT | Fraction | Scan | Source File | Area BuCaMH04\_B15 | #Feature | #Feature BuCaMH04\_B15 | Start | End | PTM | AScore | Found By |
| --- | --- | --- | --- | --- | --- | --- | --- | --- | --- | --- | --- | --- | --- | --- | --- | --- | --- | --- | --- |
| R.LC(+57.02)LSDYSIFSETIEIC(+57.02)PDGHNFC(+57.02)FK.K | Y | 53.74 | 3051.3452 | 25 | 0.7 | 1018.1230 | 3 | 49.97 | 1 | 35684 | BuCaMH04.raw | 1.5388E7 | 1 | 1 | 23 | 47 | Carbamidomethylation | C2:Carbamidomethylation:1000.00;C16:Carbamidomethylation:1000.00;C23:Carbamidomethylation:1000.00 | PEAKS DB |
| total 1 peptides |  |  |  |  |  |  |  |  |  |  |  |  |  |  |  |  |  |  |  |
| --- | --- | --- | --- | --- | --- | --- | --- | --- | --- | --- | --- | --- | --- | --- | --- | --- | --- | --- | --- |

APB88857.1

back to list

  

| Protein Coverage
| Supporting Peptides
|

Protein Coverage:

Supporting Peptides:

| Peptide | Uniq | -10lgP | Mass | Length | ppm | m/z | z | RT | Fraction | Scan | Source File | Area BuCaMH04\_B15 | #Feature | #Feature BuCaMH04\_B15 | Start | End | PTM | AScore | Found By |
| --- | --- | --- | --- | --- | --- | --- | --- | --- | --- | --- | --- | --- | --- | --- | --- | --- | --- | --- | --- |
| R.LC(+57.02)LSDYSIFSETIEIC(+57.02)PDGHNFC(+57.02)FK.K | Y | 53.74 | 3051.3452 | 25 | 0.7 | 1018.1230 | 3 | 49.97 | 1 | 35684 | BuCaMH04.raw | 1.5388E7 | 1 | 1 | 23 | 47 | Carbamidomethylation | C2:Carbamidomethylation:1000.00;C16:Carbamidomethylation:1000.00;C23:Carbamidomethylation:1000.00 | PEAKS DB |
| total 1 peptides |  |  |  |  |  |  |  |  |  |  |  |  |  |  |  |  |  |  |  |
| --- | --- | --- | --- | --- | --- | --- | --- | --- | --- | --- | --- | --- | --- | --- | --- | --- | --- | --- | --- |

CAB45156.1

back to list

  

| Protein Coverage
| Supporting Peptides
|

Protein Coverage:

Supporting Peptides:

| Peptide | Uniq | -10lgP | Mass | Length | ppm | m/z | z | RT | Fraction | Scan | Source File | Area BuCaMH04\_B15 | #Feature | #Feature BuCaMH04\_B15 | Start | End | PTM | AScore | Found By |
| --- | --- | --- | --- | --- | --- | --- | --- | --- | --- | --- | --- | --- | --- | --- | --- | --- | --- | --- | --- |
| R.LC(+57.02)LSDYSIFSETIEIC(+57.02)PDGHNFC(+57.02)FK.K | Y | 53.74 | 3051.3452 | 25 | 0.7 | 1018.1230 | 3 | 49.97 | 1 | 35684 | BuCaMH04.raw | 1.5388E7 | 1 | 1 | 23 | 47 | Carbamidomethylation | C2:Carbamidomethylation:1000.00;C16:Carbamidomethylation:1000.00;C23:Carbamidomethylation:1000.00 | PEAKS DB |
| total 1 peptides |  |  |  |  |  |  |  |  |  |  |  |  |  |  |  |  |  |  |  |
| --- | --- | --- | --- | --- | --- | --- | --- | --- | --- | --- | --- | --- | --- | --- | --- | --- | --- | --- | --- |

JAC94989.1

back to list

  

| Protein Coverage
| Supporting Peptides
|

Protein Coverage:

Supporting Peptides:

| Peptide | Uniq | -10lgP | Mass | Length | ppm | m/z | z | RT | Fraction | Scan | Source File | Area BuCaMH04\_B15 | #Feature | #Feature BuCaMH04\_B15 | Start | End | PTM | AScore | Found By |
| --- | --- | --- | --- | --- | --- | --- | --- | --- | --- | --- | --- | --- | --- | --- | --- | --- | --- | --- | --- |
| K.QVVPESLLAWER.V | Y | 59.74 | 1425.7616 | 12 | 0.8 | 713.8887 | 2 | 36.18 | 1 | 23350 | BuCaMH04.raw | 6.9018E5 | 1 | 1 | 319 | 330 |  |  | PEAKS DB |
| A.DLHYATVYWLEAEK.T | N | 53.99 | 1736.8409 | 14 | 0.4 | 579.9545 | 3 | 35.21 | 1 | 22530 | BuCaMH04.raw | 9.7317E5 | 1 | 1 | 37 | 50 |  |  | PEAKS DB |
| total 2 peptides |  |  |  |  |  |  |  |  |  |  |  |  |  |  |  |  |  |  |  |
| --- | --- | --- | --- | --- | --- | --- | --- | --- | --- | --- | --- | --- | --- | --- | --- | --- | --- | --- | --- |

5MG9

back to list

  

| Protein Coverage
| Supporting Peptides
|

Protein Coverage:

Supporting Peptides:

| Peptide | Uniq | -10lgP | Mass | Length | ppm | m/z | z | RT | Fraction | Scan | Source File | Area BuCaMH04\_B15 | #Feature | #Feature BuCaMH04\_B15 | Start | End | PTM | AScore | Found By |
| --- | --- | --- | --- | --- | --- | --- | --- | --- | --- | --- | --- | --- | --- | --- | --- | --- | --- | --- | --- |
| K.SIFGVTTEDC(+57.02)PDGQNLC(+57.02)FK.R | Y | 82.53 | 2186.9612 | 19 | 0.4 | 1094.4883 | 2 | 33.40 | 1 | 20847 | BuCaMH04.raw | 1.6197E7 | 1 | 1 | 8 | 26 | Carbamidomethylation | C10:Carbamidomethylation:1000.00;C17:Carbamidomethylation:1000.00 | PEAKS DB |
| total 1 peptides |  |  |  |  |  |  |  |  |  |  |  |  |  |  |  |  |  |  |  |
| --- | --- | --- | --- | --- | --- | --- | --- | --- | --- | --- | --- | --- | --- | --- | --- | --- | --- | --- | --- |

P82463.1

back to list

  

| Protein Coverage
| Supporting Peptides
|

Protein Coverage:

Supporting Peptides:

| Peptide | Uniq | -10lgP | Mass | Length | ppm | m/z | z | RT | Fraction | Scan | Source File | Area BuCaMH04\_B15 | #Feature | #Feature BuCaMH04\_B15 | Start | End | PTM | AScore | Found By |
| --- | --- | --- | --- | --- | --- | --- | --- | --- | --- | --- | --- | --- | --- | --- | --- | --- | --- | --- | --- |
| K.SIFGVTTEDC(+57.02)PDGQNLC(+57.02)FK.R | Y | 82.53 | 2186.9612 | 19 | 0.4 | 1094.4883 | 2 | 33.40 | 1 | 20847 | BuCaMH04.raw | 1.6197E7 | 1 | 1 | 8 | 26 | Carbamidomethylation | C10:Carbamidomethylation:1000.00;C17:Carbamidomethylation:1000.00 | PEAKS DB |
| total 1 peptides |  |  |  |  |  |  |  |  |  |  |  |  |  |  |  |  |  |  |  |
| --- | --- | --- | --- | --- | --- | --- | --- | --- | --- | --- | --- | --- | --- | --- | --- | --- | --- | --- | --- |

XP\_015669101.1

back to list

  

| Protein Coverage
| Supporting Peptides
|

Protein Coverage:

Supporting Peptides:

| Peptide | Uniq | -10lgP | Mass | Length | ppm | m/z | z | RT | Fraction | Scan | Source File | Area BuCaMH04\_B15 | #Feature | #Feature BuCaMH04\_B15 | Start | End | PTM | AScore | Found By |
| --- | --- | --- | --- | --- | --- | --- | --- | --- | --- | --- | --- | --- | --- | --- | --- | --- | --- | --- | --- |
| K.SILDMLLEATSPK.M | Y | 81.42 | 1416.7534 | 13 | 1.1 | 709.3848 | 2 | 61.90 | 1 | 45965 | BuCaMH04.raw | 6.8478E5 | 1 | 1 | 362 | 374 |  |  | PEAKS DB |
| total 1 peptides |  |  |  |  |  |  |  |  |  |  |  |  |  |  |  |  |  |  |  |
| --- | --- | --- | --- | --- | --- | --- | --- | --- | --- | --- | --- | --- | --- | --- | --- | --- | --- | --- | --- |

BAN82088.1

back to list

  

| Protein Coverage
| Supporting Peptides
|

Protein Coverage:

Supporting Peptides:

| Peptide | Uniq | -10lgP | Mass | Length | ppm | m/z | z | RT | Fraction | Scan | Source File | Area BuCaMH04\_B15 | #Feature | #Feature BuCaMH04\_B15 | Start | End | PTM | AScore | Found By |
| --- | --- | --- | --- | --- | --- | --- | --- | --- | --- | --- | --- | --- | --- | --- | --- | --- | --- | --- | --- |
| K.SILDMLLEATSPK.M | Y | 81.42 | 1416.7534 | 13 | 1.1 | 709.3848 | 2 | 61.90 | 1 | 45965 | BuCaMH04.raw | 6.8478E5 | 1 | 1 | 365 | 377 |  |  | PEAKS DB |
| total 1 peptides |  |  |  |  |  |  |  |  |  |  |  |  |  |  |  |  |  |  |  |
| --- | --- | --- | --- | --- | --- | --- | --- | --- | --- | --- | --- | --- | --- | --- | --- | --- | --- | --- | --- |

XP\_032088229.1

back to list

  

| Protein Coverage
| Supporting Peptides
|

Protein Coverage:

Supporting Peptides:

| Peptide | Uniq | -10lgP | Mass | Length | ppm | m/z | z | RT | Fraction | Scan | Source File | Area BuCaMH04\_B15 | #Feature | #Feature BuCaMH04\_B15 | Start | End | PTM | AScore | Found By |
| --- | --- | --- | --- | --- | --- | --- | --- | --- | --- | --- | --- | --- | --- | --- | --- | --- | --- | --- | --- |
| K.SILDMLLEATSPK.M | Y | 81.42 | 1416.7534 | 13 | 1.1 | 709.3848 | 2 | 61.90 | 1 | 45965 | BuCaMH04.raw | 6.8478E5 | 1 | 1 | 362 | 374 |  |  | PEAKS DB |
| total 1 peptides |  |  |  |  |  |  |  |  |  |  |  |  |  |  |  |  |  |  |  |
| --- | --- | --- | --- | --- | --- | --- | --- | --- | --- | --- | --- | --- | --- | --- | --- | --- | --- | --- | --- |

XP\_032088228.1

back to list

  

| Protein Coverage
| Supporting Peptides
|

Protein Coverage:

Supporting Peptides:

| Peptide | Uniq | -10lgP | Mass | Length | ppm | m/z | z | RT | Fraction | Scan | Source File | Area BuCaMH04\_B15 | #Feature | #Feature BuCaMH04\_B15 | Start | End | PTM | AScore | Found By |
| --- | --- | --- | --- | --- | --- | --- | --- | --- | --- | --- | --- | --- | --- | --- | --- | --- | --- | --- | --- |
| K.SILDMLLEATSPK.M | Y | 81.42 | 1416.7534 | 13 | 1.1 | 709.3848 | 2 | 61.90 | 1 | 45965 | BuCaMH04.raw | 6.8478E5 | 1 | 1 | 365 | 377 |  |  | PEAKS DB |
| total 1 peptides |  |  |  |  |  |  |  |  |  |  |  |  |  |  |  |  |  |  |  |
| --- | --- | --- | --- | --- | --- | --- | --- | --- | --- | --- | --- | --- | --- | --- | --- | --- | --- | --- | --- |

AFJ51008.1

back to list

  

| Protein Coverage
| Supporting Peptides
|

Protein Coverage:

Supporting Peptides:

| Peptide | Uniq | -10lgP | Mass | Length | ppm | m/z | z | RT | Fraction | Scan | Source File | Area BuCaMH04\_B15 | #Feature | #Feature BuCaMH04\_B15 | Start | End | PTM | AScore | Found By |
| --- | --- | --- | --- | --- | --- | --- | --- | --- | --- | --- | --- | --- | --- | --- | --- | --- | --- | --- | --- |
| K.SILDMLLEATSPK.M | Y | 81.42 | 1416.7534 | 13 | 1.1 | 709.3848 | 2 | 61.90 | 1 | 45965 | BuCaMH04.raw | 6.8478E5 | 1 | 1 | 362 | 374 |  |  | PEAKS DB |
| total 1 peptides |  |  |  |  |  |  |  |  |  |  |  |  |  |  |  |  |  |  |  |
| --- | --- | --- | --- | --- | --- | --- | --- | --- | --- | --- | --- | --- | --- | --- | --- | --- | --- | --- | --- |

XP\_026528620.1

back to list

  

| Protein Coverage
| Supporting Peptides
|

Protein Coverage:

Supporting Peptides:

| Peptide | Uniq | -10lgP | Mass | Length | ppm | m/z | z | RT | Fraction | Scan | Source File | Area BuCaMH04\_B15 | #Feature | #Feature BuCaMH04\_B15 | Start | End | PTM | AScore | Found By |
| --- | --- | --- | --- | --- | --- | --- | --- | --- | --- | --- | --- | --- | --- | --- | --- | --- | --- | --- | --- |
| K.SILDMLLEATSPK.M | Y | 81.42 | 1416.7534 | 13 | 1.1 | 709.3848 | 2 | 61.90 | 1 | 45965 | BuCaMH04.raw | 6.8478E5 | 1 | 1 | 362 | 374 |  |  | PEAKS DB |
| total 1 peptides |  |  |  |  |  |  |  |  |  |  |  |  |  |  |  |  |  |  |  |
| --- | --- | --- | --- | --- | --- | --- | --- | --- | --- | --- | --- | --- | --- | --- | --- | --- | --- | --- | --- |

XP\_026569354.1

back to list

  

| Protein Coverage
| Supporting Peptides
|

Protein Coverage:

Supporting Peptides:

| Peptide | Uniq | -10lgP | Mass | Length | ppm | m/z | z | RT | Fraction | Scan | Source File | Area BuCaMH04\_B15 | #Feature | #Feature BuCaMH04\_B15 | Start | End | PTM | AScore | Found By |
| --- | --- | --- | --- | --- | --- | --- | --- | --- | --- | --- | --- | --- | --- | --- | --- | --- | --- | --- | --- |
| K.SILDMLLEATSPK.M | Y | 81.42 | 1416.7534 | 13 | 1.1 | 709.3848 | 2 | 61.90 | 1 | 45965 | BuCaMH04.raw | 6.8478E5 | 1 | 1 | 362 | 374 |  |  | PEAKS DB |
| total 1 peptides |  |  |  |  |  |  |  |  |  |  |  |  |  |  |  |  |  |  |  |
| --- | --- | --- | --- | --- | --- | --- | --- | --- | --- | --- | --- | --- | --- | --- | --- | --- | --- | --- | --- |

XP\_013923763.1

back to list

  

| Protein Coverage
| Supporting Peptides
|

Protein Coverage:

Supporting Peptides:

| Peptide | Uniq | -10lgP | Mass | Length | ppm | m/z | z | RT | Fraction | Scan | Source File | Area BuCaMH04\_B15 | #Feature | #Feature BuCaMH04\_B15 | Start | End | PTM | AScore | Found By |
| --- | --- | --- | --- | --- | --- | --- | --- | --- | --- | --- | --- | --- | --- | --- | --- | --- | --- | --- | --- |
| K.SILDMLLEATSPK.M | Y | 81.42 | 1416.7534 | 13 | 1.1 | 709.3848 | 2 | 61.90 | 1 | 45965 | BuCaMH04.raw | 6.8478E5 | 1 | 1 | 362 | 374 |  |  | PEAKS DB |
| total 1 peptides |  |  |  |  |  |  |  |  |  |  |  |  |  |  |  |  |  |  |  |
| --- | --- | --- | --- | --- | --- | --- | --- | --- | --- | --- | --- | --- | --- | --- | --- | --- | --- | --- | --- |

XP\_013923762.1

back to list

  

| Protein Coverage
| Supporting Peptides
|

Protein Coverage:

Supporting Peptides:

| Peptide | Uniq | -10lgP | Mass | Length | ppm | m/z | z | RT | Fraction | Scan | Source File | Area BuCaMH04\_B15 | #Feature | #Feature BuCaMH04\_B15 | Start | End | PTM | AScore | Found By |
| --- | --- | --- | --- | --- | --- | --- | --- | --- | --- | --- | --- | --- | --- | --- | --- | --- | --- | --- | --- |
| K.SILDMLLEATSPK.M | Y | 81.42 | 1416.7534 | 13 | 1.1 | 709.3848 | 2 | 61.90 | 1 | 45965 | BuCaMH04.raw | 6.8478E5 | 1 | 1 | 365 | 377 |  |  | PEAKS DB |
| total 1 peptides |  |  |  |  |  |  |  |  |  |  |  |  |  |  |  |  |  |  |  |
| --- | --- | --- | --- | --- | --- | --- | --- | --- | --- | --- | --- | --- | --- | --- | --- | --- | --- | --- | --- |

XP\_026528619.1

back to list

  

| Protein Coverage
| Supporting Peptides
|

Protein Coverage:

Supporting Peptides:

| Peptide | Uniq | -10lgP | Mass | Length | ppm | m/z | z | RT | Fraction | Scan | Source File | Area BuCaMH04\_B15 | #Feature | #Feature BuCaMH04\_B15 | Start | End | PTM | AScore | Found By |
| --- | --- | --- | --- | --- | --- | --- | --- | --- | --- | --- | --- | --- | --- | --- | --- | --- | --- | --- | --- |
| K.SILDMLLEATSPK.M | Y | 81.42 | 1416.7534 | 13 | 1.1 | 709.3848 | 2 | 61.90 | 1 | 45965 | BuCaMH04.raw | 6.8478E5 | 1 | 1 | 365 | 377 |  |  | PEAKS DB |
| total 1 peptides |  |  |  |  |  |  |  |  |  |  |  |  |  |  |  |  |  |  |  |
| --- | --- | --- | --- | --- | --- | --- | --- | --- | --- | --- | --- | --- | --- | --- | --- | --- | --- | --- | --- |

XP\_026569353.1

back to list

  

| Protein Coverage
| Supporting Peptides
|

Protein Coverage:

Supporting Peptides:

| Peptide | Uniq | -10lgP | Mass | Length | ppm | m/z | z | RT | Fraction | Scan | Source File | Area BuCaMH04\_B15 | #Feature | #Feature BuCaMH04\_B15 | Start | End | PTM | AScore | Found By |
| --- | --- | --- | --- | --- | --- | --- | --- | --- | --- | --- | --- | --- | --- | --- | --- | --- | --- | --- | --- |
| K.SILDMLLEATSPK.M | Y | 81.42 | 1416.7534 | 13 | 1.1 | 709.3848 | 2 | 61.90 | 1 | 45965 | BuCaMH04.raw | 6.8478E5 | 1 | 1 | 365 | 377 |  |  | PEAKS DB |
| total 1 peptides |  |  |  |  |  |  |  |  |  |  |  |  |  |  |  |  |  |  |  |
| --- | --- | --- | --- | --- | --- | --- | --- | --- | --- | --- | --- | --- | --- | --- | --- | --- | --- | --- | --- |

XP\_034291658.1

back to list

  

| Protein Coverage
| Supporting Peptides
|

Protein Coverage:

Supporting Peptides:

| Peptide | Uniq | -10lgP | Mass | Length | ppm | m/z | z | RT | Fraction | Scan | Source File | Area BuCaMH04\_B15 | #Feature | #Feature BuCaMH04\_B15 | Start | End | PTM | AScore | Found By |
| --- | --- | --- | --- | --- | --- | --- | --- | --- | --- | --- | --- | --- | --- | --- | --- | --- | --- | --- | --- |
| K.SILDMLLEATSPK.M | Y | 81.42 | 1416.7534 | 13 | 1.1 | 709.3848 | 2 | 61.90 | 1 | 45965 | BuCaMH04.raw | 6.8478E5 | 1 | 1 | 365 | 377 |  |  | PEAKS DB |
| total 1 peptides |  |  |  |  |  |  |  |  |  |  |  |  |  |  |  |  |  |  |  |
| --- | --- | --- | --- | --- | --- | --- | --- | --- | --- | --- | --- | --- | --- | --- | --- | --- | --- | --- | --- |

XP\_034291656.1

back to list

  

| Protein Coverage
| Supporting Peptides
|

Protein Coverage:

Supporting Peptides:

| Peptide | Uniq | -10lgP | Mass | Length | ppm | m/z | z | RT | Fraction | Scan | Source File | Area BuCaMH04\_B15 | #Feature | #Feature BuCaMH04\_B15 | Start | End | PTM | AScore | Found By |
| --- | --- | --- | --- | --- | --- | --- | --- | --- | --- | --- | --- | --- | --- | --- | --- | --- | --- | --- | --- |
| K.SILDMLLEATSPK.M | Y | 81.42 | 1416.7534 | 13 | 1.1 | 709.3848 | 2 | 61.90 | 1 | 45965 | BuCaMH04.raw | 6.8478E5 | 1 | 1 | 367 | 379 |  |  | PEAKS DB |
| total 1 peptides |  |  |  |  |  |  |  |  |  |  |  |  |  |  |  |  |  |  |  |
| --- | --- | --- | --- | --- | --- | --- | --- | --- | --- | --- | --- | --- | --- | --- | --- | --- | --- | --- | --- |

XP\_034291657.1

back to list

  

| Protein Coverage
| Supporting Peptides
|

Protein Coverage:

Supporting Peptides:

| Peptide | Uniq | -10lgP | Mass | Length | ppm | m/z | z | RT | Fraction | Scan | Source File | Area BuCaMH04\_B15 | #Feature | #Feature BuCaMH04\_B15 | Start | End | PTM | AScore | Found By |
| --- | --- | --- | --- | --- | --- | --- | --- | --- | --- | --- | --- | --- | --- | --- | --- | --- | --- | --- | --- |
| K.SILDMLLEATSPK.M | Y | 81.42 | 1416.7534 | 13 | 1.1 | 709.3848 | 2 | 61.90 | 1 | 45965 | BuCaMH04.raw | 6.8478E5 | 1 | 1 | 368 | 380 |  |  | PEAKS DB |
| total 1 peptides |  |  |  |  |  |  |  |  |  |  |  |  |  |  |  |  |  |  |  |
| --- | --- | --- | --- | --- | --- | --- | --- | --- | --- | --- | --- | --- | --- | --- | --- | --- | --- | --- | --- |

XP\_034291655.1

back to list

  

| Protein Coverage
| Supporting Peptides
|

Protein Coverage:

Supporting Peptides:

| Peptide | Uniq | -10lgP | Mass | Length | ppm | m/z | z | RT | Fraction | Scan | Source File | Area BuCaMH04\_B15 | #Feature | #Feature BuCaMH04\_B15 | Start | End | PTM | AScore | Found By |
| --- | --- | --- | --- | --- | --- | --- | --- | --- | --- | --- | --- | --- | --- | --- | --- | --- | --- | --- | --- |
| K.SILDMLLEATSPK.M | Y | 81.42 | 1416.7534 | 13 | 1.1 | 709.3848 | 2 | 61.90 | 1 | 45965 | BuCaMH04.raw | 6.8478E5 | 1 | 1 | 368 | 380 |  |  | PEAKS DB |
| total 1 peptides |  |  |  |  |  |  |  |  |  |  |  |  |  |  |  |  |  |  |  |
| --- | --- | --- | --- | --- | --- | --- | --- | --- | --- | --- | --- | --- | --- | --- | --- | --- | --- | --- | --- |

ETE61271.1

back to list

  

| Protein Coverage
| Supporting Peptides
|

Protein Coverage:

Supporting Peptides:

| Peptide | Uniq | -10lgP | Mass | Length | ppm | m/z | z | RT | Fraction | Scan | Source File | Area BuCaMH04\_B15 | #Feature | #Feature BuCaMH04\_B15 | Start | End | PTM | AScore | Found By |
| --- | --- | --- | --- | --- | --- | --- | --- | --- | --- | --- | --- | --- | --- | --- | --- | --- | --- | --- | --- |
| K.SILDMLLEATSPK.M | Y | 81.42 | 1416.7534 | 13 | 1.1 | 709.3848 | 2 | 61.90 | 1 | 45965 | BuCaMH04.raw | 6.8478E5 | 1 | 1 | 366 | 378 |  |  | PEAKS DB |
| total 1 peptides |  |  |  |  |  |  |  |  |  |  |  |  |  |  |  |  |  |  |  |
| --- | --- | --- | --- | --- | --- | --- | --- | --- | --- | --- | --- | --- | --- | --- | --- | --- | --- | --- | --- |

XP\_007441356.1

back to list

  

| Protein Coverage
| Supporting Peptides
|

Protein Coverage:

Supporting Peptides:

| Peptide | Uniq | -10lgP | Mass | Length | ppm | m/z | z | RT | Fraction | Scan | Source File | Area BuCaMH04\_B15 | #Feature | #Feature BuCaMH04\_B15 | Start | End | PTM | AScore | Found By |
| --- | --- | --- | --- | --- | --- | --- | --- | --- | --- | --- | --- | --- | --- | --- | --- | --- | --- | --- | --- |
| K.SIIDMLLEATSPK.M | Y | 81.42 | 1416.7534 | 13 | 1.1 | 709.3848 | 2 | 61.90 | 1 | 45965 | BuCaMH04.raw | 6.8478E5 | 1 | 1 | 227 | 239 |  |  | PEAKS DB |
| total 1 peptides |  |  |  |  |  |  |  |  |  |  |  |  |  |  |  |  |  |  |  |
| --- | --- | --- | --- | --- | --- | --- | --- | --- | --- | --- | --- | --- | --- | --- | --- | --- | --- | --- | --- |

ETE64512.1

back to list

  

| Protein Coverage
| Supporting Peptides
|

Protein Coverage:

Supporting Peptides:

| Peptide | Uniq | -10lgP | Mass | Length | ppm | m/z | z | RT | Fraction | Scan | Source File | Area BuCaMH04\_B15 | #Feature | #Feature BuCaMH04\_B15 | Start | End | PTM | AScore | Found By |
| --- | --- | --- | --- | --- | --- | --- | --- | --- | --- | --- | --- | --- | --- | --- | --- | --- | --- | --- | --- |
| R.LLELQYFISR.D | Y | 56.34 | 1280.7129 | 10 | 0.6 | 641.3641 | 2 | 42.61 | 1 | 29121 | BuCaMH04.raw | 9.7335E5 | 1 | 1 | 400 | 409 |  |  | PEAKS DB |
| total 1 peptides |  |  |  |  |  |  |  |  |  |  |  |  |  |  |  |  |  |  |  |
| --- | --- | --- | --- | --- | --- | --- | --- | --- | --- | --- | --- | --- | --- | --- | --- | --- | --- | --- | --- |

XP\_026535930.1

back to list

  

| Protein Coverage
| Supporting Peptides
|

Protein Coverage:

Supporting Peptides:

| Peptide | Uniq | -10lgP | Mass | Length | ppm | m/z | z | RT | Fraction | Scan | Source File | Area BuCaMH04\_B15 | #Feature | #Feature BuCaMH04\_B15 | Start | End | PTM | AScore | Found By |
| --- | --- | --- | --- | --- | --- | --- | --- | --- | --- | --- | --- | --- | --- | --- | --- | --- | --- | --- | --- |
| R.LLELQYFISR.D | Y | 56.34 | 1280.7129 | 10 | 0.6 | 641.3641 | 2 | 42.61 | 1 | 29121 | BuCaMH04.raw | 9.7335E5 | 1 | 1 | 528 | 537 |  |  | PEAKS DB |
| total 1 peptides |  |  |  |  |  |  |  |  |  |  |  |  |  |  |  |  |  |  |  |
| --- | --- | --- | --- | --- | --- | --- | --- | --- | --- | --- | --- | --- | --- | --- | --- | --- | --- | --- | --- |

XP\_026570202.1

back to list

  

| Protein Coverage
| Supporting Peptides
|

Protein Coverage:

Supporting Peptides:

| Peptide | Uniq | -10lgP | Mass | Length | ppm | m/z | z | RT | Fraction | Scan | Source File | Area BuCaMH04\_B15 | #Feature | #Feature BuCaMH04\_B15 | Start | End | PTM | AScore | Found By |
| --- | --- | --- | --- | --- | --- | --- | --- | --- | --- | --- | --- | --- | --- | --- | --- | --- | --- | --- | --- |
| R.LLELQYFISR.D | Y | 56.34 | 1280.7129 | 10 | 0.6 | 641.3641 | 2 | 42.61 | 1 | 29121 | BuCaMH04.raw | 9.7335E5 | 1 | 1 | 532 | 541 |  |  | PEAKS DB |
| total 1 peptides |  |  |  |  |  |  |  |  |  |  |  |  |  |  |  |  |  |  |  |
| --- | --- | --- | --- | --- | --- | --- | --- | --- | --- | --- | --- | --- | --- | --- | --- | --- | --- | --- | --- |

LAB62693.1

back to list

  

| Protein Coverage
| Supporting Peptides
|

Protein Coverage:

Supporting Peptides:

| Peptide | Uniq | -10lgP | Mass | Length | ppm | m/z | z | RT | Fraction | Scan | Source File | Area BuCaMH04\_B15 | #Feature | #Feature BuCaMH04\_B15 | Start | End | PTM | AScore | Found By |
| --- | --- | --- | --- | --- | --- | --- | --- | --- | --- | --- | --- | --- | --- | --- | --- | --- | --- | --- | --- |
| R.LLELQYFISR.D | Y | 56.34 | 1280.7129 | 10 | 0.6 | 641.3641 | 2 | 42.61 | 1 | 29121 | BuCaMH04.raw | 9.7335E5 | 1 | 1 | 200 | 209 |  |  | PEAKS DB |
| total 1 peptides |  |  |  |  |  |  |  |  |  |  |  |  |  |  |  |  |  |  |  |
| --- | --- | --- | --- | --- | --- | --- | --- | --- | --- | --- | --- | --- | --- | --- | --- | --- | --- | --- | --- |

LAB62690.1

back to list

  

| Protein Coverage
| Supporting Peptides
|

Protein Coverage:

Supporting Peptides:

| Peptide | Uniq | -10lgP | Mass | Length | ppm | m/z | z | RT | Fraction | Scan | Source File | Area BuCaMH04\_B15 | #Feature | #Feature BuCaMH04\_B15 | Start | End | PTM | AScore | Found By |
| --- | --- | --- | --- | --- | --- | --- | --- | --- | --- | --- | --- | --- | --- | --- | --- | --- | --- | --- | --- |
| R.LLELQYFISR.D | Y | 56.34 | 1280.7129 | 10 | 0.6 | 641.3641 | 2 | 42.61 | 1 | 29121 | BuCaMH04.raw | 9.7335E5 | 1 | 1 | 427 | 436 |  |  | PEAKS DB |
| total 1 peptides |  |  |  |  |  |  |  |  |  |  |  |  |  |  |  |  |  |  |  |
| --- | --- | --- | --- | --- | --- | --- | --- | --- | --- | --- | --- | --- | --- | --- | --- | --- | --- | --- | --- |

LAB18298.1

back to list

  

| Protein Coverage
| Supporting Peptides
|

Protein Coverage:

Supporting Peptides:

| Peptide | Uniq | -10lgP | Mass | Length | ppm | m/z | z | RT | Fraction | Scan | Source File | Area BuCaMH04\_B15 | #Feature | #Feature BuCaMH04\_B15 | Start | End | PTM | AScore | Found By |
| --- | --- | --- | --- | --- | --- | --- | --- | --- | --- | --- | --- | --- | --- | --- | --- | --- | --- | --- | --- |
| R.LLELQYFISR.D | Y | 56.34 | 1280.7129 | 10 | 0.6 | 641.3641 | 2 | 42.61 | 1 | 29121 | BuCaMH04.raw | 9.7335E5 | 1 | 1 | 139 | 148 |  |  | PEAKS DB |
| total 1 peptides |  |  |  |  |  |  |  |  |  |  |  |  |  |  |  |  |  |  |  |
| --- | --- | --- | --- | --- | --- | --- | --- | --- | --- | --- | --- | --- | --- | --- | --- | --- | --- | --- | --- |

LAB18300.1

back to list

  

| Protein Coverage
| Supporting Peptides
|

Protein Coverage:

Supporting Peptides:

| Peptide | Uniq | -10lgP | Mass | Length | ppm | m/z | z | RT | Fraction | Scan | Source File | Area BuCaMH04\_B15 | #Feature | #Feature BuCaMH04\_B15 | Start | End | PTM | AScore | Found By |
| --- | --- | --- | --- | --- | --- | --- | --- | --- | --- | --- | --- | --- | --- | --- | --- | --- | --- | --- | --- |
| R.LLELQYFISR.D | Y | 56.34 | 1280.7129 | 10 | 0.6 | 641.3641 | 2 | 42.61 | 1 | 29121 | BuCaMH04.raw | 9.7335E5 | 1 | 1 | 218 | 227 |  |  | PEAKS DB |
| total 1 peptides |  |  |  |  |  |  |  |  |  |  |  |  |  |  |  |  |  |  |  |
| --- | --- | --- | --- | --- | --- | --- | --- | --- | --- | --- | --- | --- | --- | --- | --- | --- | --- | --- | --- |

ETE56933.1

back to list

  

| Protein Coverage
| Supporting Peptides
|

Protein Coverage:

Supporting Peptides:

| Peptide | Uniq | -10lgP | Mass | Length | ppm | m/z | z | RT | Fraction | Scan | Source File | Area BuCaMH04\_B15 | #Feature | #Feature BuCaMH04\_B15 | Start | End | PTM | AScore | Found By |
| --- | --- | --- | --- | --- | --- | --- | --- | --- | --- | --- | --- | --- | --- | --- | --- | --- | --- | --- | --- |
| K.ITC(+57.02)SAEETFC(+57.02)YK.W | Y | 80.02 | 1507.6323 | 12 | 0.3 | 754.8237 | 2 | 14.03 | 1 | 3660 | BuCaMH04.raw | 2.4038E7 | 1 | 1 | 90 | 101 | Carbamidomethylation | C3:Carbamidomethylation:1000.00;C10:Carbamidomethylation:1000.00 | PEAKS DB |
| total 1 peptides |  |  |  |  |  |  |  |  |  |  |  |  |  |  |  |  |  |  |  |
| --- | --- | --- | --- | --- | --- | --- | --- | --- | --- | --- | --- | --- | --- | --- | --- | --- | --- | --- | --- |

1IJC

back to list

  

| Protein Coverage
| Supporting Peptides
|

Protein Coverage:

Supporting Peptides:

| Peptide | Uniq | -10lgP | Mass | Length | ppm | m/z | z | RT | Fraction | Scan | Source File | Area BuCaMH04\_B15 | #Feature | #Feature BuCaMH04\_B15 | Start | End | PTM | AScore | Found By |
| --- | --- | --- | --- | --- | --- | --- | --- | --- | --- | --- | --- | --- | --- | --- | --- | --- | --- | --- | --- |
| K.ITC(+57.02)SAEETFC(+57.02)YK.W | Y | 80.02 | 1507.6323 | 12 | 0.3 | 754.8237 | 2 | 14.03 | 1 | 3660 | BuCaMH04.raw | 2.4038E7 | 1 | 1 | 15 | 26 | Carbamidomethylation | C3:Carbamidomethylation:1000.00;C10:Carbamidomethylation:1000.00 | PEAKS DB |
| total 1 peptides |  |  |  |  |  |  |  |  |  |  |  |  |  |  |  |  |  |  |  |
| --- | --- | --- | --- | --- | --- | --- | --- | --- | --- | --- | --- | --- | --- | --- | --- | --- | --- | --- | --- |

1F94

back to list

  

| Protein Coverage
| Supporting Peptides
|

Protein Coverage:

Supporting Peptides:

| Peptide | Uniq | -10lgP | Mass | Length | ppm | m/z | z | RT | Fraction | Scan | Source File | Area BuCaMH04\_B15 | #Feature | #Feature BuCaMH04\_B15 | Start | End | PTM | AScore | Found By |
| --- | --- | --- | --- | --- | --- | --- | --- | --- | --- | --- | --- | --- | --- | --- | --- | --- | --- | --- | --- |
| K.ITC(+57.02)SAEETFC(+57.02)YK.W | Y | 80.02 | 1507.6323 | 12 | 0.3 | 754.8237 | 2 | 14.03 | 1 | 3660 | BuCaMH04.raw | 2.4038E7 | 1 | 1 | 15 | 26 | Carbamidomethylation | C3:Carbamidomethylation:1000.00;C10:Carbamidomethylation:1000.00 | PEAKS DB |
| total 1 peptides |  |  |  |  |  |  |  |  |  |  |  |  |  |  |  |  |  |  |  |
| --- | --- | --- | --- | --- | --- | --- | --- | --- | --- | --- | --- | --- | --- | --- | --- | --- | --- | --- | --- |

P81782.1

back to list

  

| Protein Coverage
| Supporting Peptides
|

Protein Coverage:

Supporting Peptides:

| Peptide | Uniq | -10lgP | Mass | Length | ppm | m/z | z | RT | Fraction | Scan | Source File | Area BuCaMH04\_B15 | #Feature | #Feature BuCaMH04\_B15 | Start | End | PTM | AScore | Found By |
| --- | --- | --- | --- | --- | --- | --- | --- | --- | --- | --- | --- | --- | --- | --- | --- | --- | --- | --- | --- |
| K.ITC(+57.02)SAEETFC(+57.02)YK.W | Y | 80.02 | 1507.6323 | 12 | 0.3 | 754.8237 | 2 | 14.03 | 1 | 3660 | BuCaMH04.raw | 2.4038E7 | 1 | 1 | 15 | 26 | Carbamidomethylation | C3:Carbamidomethylation:1000.00;C10:Carbamidomethylation:1000.00 | PEAKS DB |
| total 1 peptides |  |  |  |  |  |  |  |  |  |  |  |  |  |  |  |  |  |  |  |
| --- | --- | --- | --- | --- | --- | --- | --- | --- | --- | --- | --- | --- | --- | --- | --- | --- | --- | --- | --- |

JAC96437.1

back to list

  

| Protein Coverage
| Supporting Peptides
|

Protein Coverage:

Supporting Peptides:

| Peptide | Uniq | -10lgP | Mass | Length | ppm | m/z | z | RT | Fraction | Scan | Source File | Area BuCaMH04\_B15 | #Feature | #Feature BuCaMH04\_B15 | Start | End | PTM | AScore | Found By |
| --- | --- | --- | --- | --- | --- | --- | --- | --- | --- | --- | --- | --- | --- | --- | --- | --- | --- | --- | --- |
| A.LIGLEIWSDQDK.I | Y | 56.59 | 1415.7296 | 12 | 1.4 | 708.8731 | 2 | 37.79 | 1 | 24890 | BuCaMH04.raw | 2.2564E6 | 1 | 1 | 253 | 264 |  |  | PEAKS DB |
| total 1 peptides |  |  |  |  |  |  |  |  |  |  |  |  |  |  |  |  |  |  |  |
| --- | --- | --- | --- | --- | --- | --- | --- | --- | --- | --- | --- | --- | --- | --- | --- | --- | --- | --- | --- |

JAS04919.1

back to list

  

| Protein Coverage
| Supporting Peptides
|

Protein Coverage:

Supporting Peptides:

| Peptide | Uniq | -10lgP | Mass | Length | ppm | m/z | z | RT | Fraction | Scan | Source File | Area BuCaMH04\_B15 | #Feature | #Feature BuCaMH04\_B15 | Start | End | PTM | AScore | Found By |
| --- | --- | --- | --- | --- | --- | --- | --- | --- | --- | --- | --- | --- | --- | --- | --- | --- | --- | --- | --- |
| A.LIGLEIWSDQDK.I | Y | 56.59 | 1415.7296 | 12 | 1.4 | 708.8731 | 2 | 37.79 | 1 | 24890 | BuCaMH04.raw | 2.2564E6 | 1 | 1 | 253 | 264 |  |  | PEAKS DB |
| total 1 peptides |  |  |  |  |  |  |  |  |  |  |  |  |  |  |  |  |  |  |  |
| --- | --- | --- | --- | --- | --- | --- | --- | --- | --- | --- | --- | --- | --- | --- | --- | --- | --- | --- | --- |

JAS04923.1

back to list

  

| Protein Coverage
| Supporting Peptides
|

Protein Coverage:

Supporting Peptides:

| Peptide | Uniq | -10lgP | Mass | Length | ppm | m/z | z | RT | Fraction | Scan | Source File | Area BuCaMH04\_B15 | #Feature | #Feature BuCaMH04\_B15 | Start | End | PTM | AScore | Found By |
| --- | --- | --- | --- | --- | --- | --- | --- | --- | --- | --- | --- | --- | --- | --- | --- | --- | --- | --- | --- |
| A.LIGLEIWSDQDK.I | Y | 56.59 | 1415.7296 | 12 | 1.4 | 708.8731 | 2 | 37.79 | 1 | 24890 | BuCaMH04.raw | 2.2564E6 | 1 | 1 | 253 | 264 |  |  | PEAKS DB |
| total 1 peptides |  |  |  |  |  |  |  |  |  |  |  |  |  |  |  |  |  |  |  |
| --- | --- | --- | --- | --- | --- | --- | --- | --- | --- | --- | --- | --- | --- | --- | --- | --- | --- | --- | --- |

JAC96439.1

back to list

  

| Protein Coverage
| Supporting Peptides
|

Protein Coverage:

Supporting Peptides:

| Peptide | Uniq | -10lgP | Mass | Length | ppm | m/z | z | RT | Fraction | Scan | Source File | Area BuCaMH04\_B15 | #Feature | #Feature BuCaMH04\_B15 | Start | End | PTM | AScore | Found By |
| --- | --- | --- | --- | --- | --- | --- | --- | --- | --- | --- | --- | --- | --- | --- | --- | --- | --- | --- | --- |
| A.LIGLEIWSDQDK.I | Y | 56.59 | 1415.7296 | 12 | 1.4 | 708.8731 | 2 | 37.79 | 1 | 24890 | BuCaMH04.raw | 2.2564E6 | 1 | 1 | 253 | 264 |  |  | PEAKS DB |
| total 1 peptides |  |  |  |  |  |  |  |  |  |  |  |  |  |  |  |  |  |  |  |
| --- | --- | --- | --- | --- | --- | --- | --- | --- | --- | --- | --- | --- | --- | --- | --- | --- | --- | --- | --- |

JAS04921.1

back to list

  

| Protein Coverage
| Supporting Peptides
|

Protein Coverage:

Supporting Peptides:

| Peptide | Uniq | -10lgP | Mass | Length | ppm | m/z | z | RT | Fraction | Scan | Source File | Area BuCaMH04\_B15 | #Feature | #Feature BuCaMH04\_B15 | Start | End | PTM | AScore | Found By |
| --- | --- | --- | --- | --- | --- | --- | --- | --- | --- | --- | --- | --- | --- | --- | --- | --- | --- | --- | --- |
| A.LIGLEIWSDQDK.I | Y | 56.59 | 1415.7296 | 12 | 1.4 | 708.8731 | 2 | 37.79 | 1 | 24890 | BuCaMH04.raw | 2.2564E6 | 1 | 1 | 253 | 264 |  |  | PEAKS DB |
| total 1 peptides |  |  |  |  |  |  |  |  |  |  |  |  |  |  |  |  |  |  |  |
| --- | --- | --- | --- | --- | --- | --- | --- | --- | --- | --- | --- | --- | --- | --- | --- | --- | --- | --- | --- |

JAS04922.1

back to list

  

| Protein Coverage
| Supporting Peptides
|

Protein Coverage:

Supporting Peptides:

| Peptide | Uniq | -10lgP | Mass | Length | ppm | m/z | z | RT | Fraction | Scan | Source File | Area BuCaMH04\_B15 | #Feature | #Feature BuCaMH04\_B15 | Start | End | PTM | AScore | Found By |
| --- | --- | --- | --- | --- | --- | --- | --- | --- | --- | --- | --- | --- | --- | --- | --- | --- | --- | --- | --- |
| A.LIGLEIWSDQDK.I | Y | 56.59 | 1415.7296 | 12 | 1.4 | 708.8731 | 2 | 37.79 | 1 | 24890 | BuCaMH04.raw | 2.2564E6 | 1 | 1 | 253 | 264 |  |  | PEAKS DB |
| total 1 peptides |  |  |  |  |  |  |  |  |  |  |  |  |  |  |  |  |  |  |  |
| --- | --- | --- | --- | --- | --- | --- | --- | --- | --- | --- | --- | --- | --- | --- | --- | --- | --- | --- | --- |

JAC96441.1

back to list

  

| Protein Coverage
| Supporting Peptides
|

Protein Coverage:

Supporting Peptides:

| Peptide | Uniq | -10lgP | Mass | Length | ppm | m/z | z | RT | Fraction | Scan | Source File | Area BuCaMH04\_B15 | #Feature | #Feature BuCaMH04\_B15 | Start | End | PTM | AScore | Found By |
| --- | --- | --- | --- | --- | --- | --- | --- | --- | --- | --- | --- | --- | --- | --- | --- | --- | --- | --- | --- |
| A.LIGLEIWSDQDK.I | Y | 56.59 | 1415.7296 | 12 | 1.4 | 708.8731 | 2 | 37.79 | 1 | 24890 | BuCaMH04.raw | 2.2564E6 | 1 | 1 | 253 | 264 |  |  | PEAKS DB |
| total 1 peptides |  |  |  |  |  |  |  |  |  |  |  |  |  |  |  |  |  |  |  |
| --- | --- | --- | --- | --- | --- | --- | --- | --- | --- | --- | --- | --- | --- | --- | --- | --- | --- | --- | --- |

JAC96440.1

back to list

  

| Protein Coverage
| Supporting Peptides
|

Protein Coverage:

Supporting Peptides:

| Peptide | Uniq | -10lgP | Mass | Length | ppm | m/z | z | RT | Fraction | Scan | Source File | Area BuCaMH04\_B15 | #Feature | #Feature BuCaMH04\_B15 | Start | End | PTM | AScore | Found By |
| --- | --- | --- | --- | --- | --- | --- | --- | --- | --- | --- | --- | --- | --- | --- | --- | --- | --- | --- | --- |
| A.LIGLEIWSDQDK.I | Y | 56.59 | 1415.7296 | 12 | 1.4 | 708.8731 | 2 | 37.79 | 1 | 24890 | BuCaMH04.raw | 2.2564E6 | 1 | 1 | 253 | 264 |  |  | PEAKS DB |
| total 1 peptides |  |  |  |  |  |  |  |  |  |  |  |  |  |  |  |  |  |  |  |
| --- | --- | --- | --- | --- | --- | --- | --- | --- | --- | --- | --- | --- | --- | --- | --- | --- | --- | --- | --- |

P62377.1

back to list

  

| Protein Coverage
| Supporting Peptides
|

Protein Coverage:

Supporting Peptides:

| Peptide | Uniq | -10lgP | Mass | Length | ppm | m/z | z | RT | Fraction | Scan | Source File | Area BuCaMH04\_B15 | #Feature | #Feature BuCaMH04\_B15 | Start | End | PTM | AScore | Found By |
| --- | --- | --- | --- | --- | --- | --- | --- | --- | --- | --- | --- | --- | --- | --- | --- | --- | --- | --- | --- |
| H.NTQLPFIYK.T | Y | 52.80 | 1122.6073 | 9 | 1.7 | 562.3119 | 2 | 24.67 | 1 | 12905 | BuCaMH04.raw | 0 | 0 | 0 | 5 | 13 |  |  | PEAKS DB |
| total 1 peptides |  |  |  |  |  |  |  |  |  |  |  |  |  |  |  |  |  |  |  |
| --- | --- | --- | --- | --- | --- | --- | --- | --- | --- | --- | --- | --- | --- | --- | --- | --- | --- | --- | --- |

1CVO

back to list

  

| Protein Coverage
| Supporting Peptides
|

Protein Coverage:

Supporting Peptides:

| Peptide | Uniq | -10lgP | Mass | Length | ppm | m/z | z | RT | Fraction | Scan | Source File | Area BuCaMH04\_B15 | #Feature | #Feature BuCaMH04\_B15 | Start | End | PTM | AScore | Found By |
| --- | --- | --- | --- | --- | --- | --- | --- | --- | --- | --- | --- | --- | --- | --- | --- | --- | --- | --- | --- |
| H.NTQLPFIYK.T | Y | 52.80 | 1122.6073 | 9 | 1.7 | 562.3119 | 2 | 24.67 | 1 | 12905 | BuCaMH04.raw | 0 | 0 | 0 | 5 | 13 |  |  | PEAKS DB |
| total 1 peptides |  |  |  |  |  |  |  |  |  |  |  |  |  |  |  |  |  |  |  |
| --- | --- | --- | --- | --- | --- | --- | --- | --- | --- | --- | --- | --- | --- | --- | --- | --- | --- | --- | --- |

1KXI

back to list

  

| Protein Coverage
| Supporting Peptides
|

Protein Coverage:

Supporting Peptides:

| Peptide | Uniq | -10lgP | Mass | Length | ppm | m/z | z | RT | Fraction | Scan | Source File | Area BuCaMH04\_B15 | #Feature | #Feature BuCaMH04\_B15 | Start | End | PTM | AScore | Found By |
| --- | --- | --- | --- | --- | --- | --- | --- | --- | --- | --- | --- | --- | --- | --- | --- | --- | --- | --- | --- |
| H.NTQLPFIYK.T | Y | 52.80 | 1122.6073 | 9 | 1.7 | 562.3119 | 2 | 24.67 | 1 | 12905 | BuCaMH04.raw | 0 | 0 | 0 | 5 | 13 |  |  | PEAKS DB |
| total 1 peptides |  |  |  |  |  |  |  |  |  |  |  |  |  |  |  |  |  |  |  |
| --- | --- | --- | --- | --- | --- | --- | --- | --- | --- | --- | --- | --- | --- | --- | --- | --- | --- | --- | --- |

Q91137.1

back to list

  

| Protein Coverage
| Supporting Peptides
|

Protein Coverage:

Supporting Peptides:

| Peptide | Uniq | -10lgP | Mass | Length | ppm | m/z | z | RT | Fraction | Scan | Source File | Area BuCaMH04\_B15 | #Feature | #Feature BuCaMH04\_B15 | Start | End | PTM | AScore | Found By |
| --- | --- | --- | --- | --- | --- | --- | --- | --- | --- | --- | --- | --- | --- | --- | --- | --- | --- | --- | --- |
| H.NTQLPFIYK.T | Y | 52.80 | 1122.6073 | 9 | 1.7 | 562.3119 | 2 | 24.67 | 1 | 12905 | BuCaMH04.raw | 0 | 0 | 0 | 26 | 34 |  |  | PEAKS DB |
| total 1 peptides |  |  |  |  |  |  |  |  |  |  |  |  |  |  |  |  |  |  |  |
| --- | --- | --- | --- | --- | --- | --- | --- | --- | --- | --- | --- | --- | --- | --- | --- | --- | --- | --- | --- |

CAB50697.1

back to list

  

| Protein Coverage
| Supporting Peptides
|

Protein Coverage:

Supporting Peptides:

| Peptide | Uniq | -10lgP | Mass | Length | ppm | m/z | z | RT | Fraction | Scan | Source File | Area BuCaMH04\_B15 | #Feature | #Feature BuCaMH04\_B15 | Start | End | PTM | AScore | Found By |
| --- | --- | --- | --- | --- | --- | --- | --- | --- | --- | --- | --- | --- | --- | --- | --- | --- | --- | --- | --- |
| H.NTQLPFIYK.T | Y | 52.80 | 1122.6073 | 9 | 1.7 | 562.3119 | 2 | 24.67 | 1 | 12905 | BuCaMH04.raw | 0 | 0 | 0 | 26 | 34 |  |  | PEAKS DB |
| total 1 peptides |  |  |  |  |  |  |  |  |  |  |  |  |  |  |  |  |  |  |  |
| --- | --- | --- | --- | --- | --- | --- | --- | --- | --- | --- | --- | --- | --- | --- | --- | --- | --- | --- | --- |

AAB36928.1

back to list

  

| Protein Coverage
| Supporting Peptides
|

Protein Coverage:

Supporting Peptides:

| Peptide | Uniq | -10lgP | Mass | Length | ppm | m/z | z | RT | Fraction | Scan | Source File | Area BuCaMH04\_B15 | #Feature | #Feature BuCaMH04\_B15 | Start | End | PTM | AScore | Found By |
| --- | --- | --- | --- | --- | --- | --- | --- | --- | --- | --- | --- | --- | --- | --- | --- | --- | --- | --- | --- |
| H.NTQLPFIYK.T | Y | 52.80 | 1122.6073 | 9 | 1.7 | 562.3119 | 2 | 24.67 | 1 | 12905 | BuCaMH04.raw | 0 | 0 | 0 | 26 | 34 |  |  | PEAKS DB |
| total 1 peptides |  |  |  |  |  |  |  |  |  |  |  |  |  |  |  |  |  |  |  |
| --- | --- | --- | --- | --- | --- | --- | --- | --- | --- | --- | --- | --- | --- | --- | --- | --- | --- | --- | --- |

Q91996.1

back to list

  

| Protein Coverage
| Supporting Peptides
|

Protein Coverage:

Supporting Peptides:

| Peptide | Uniq | -10lgP | Mass | Length | ppm | m/z | z | RT | Fraction | Scan | Source File | Area BuCaMH04\_B15 | #Feature | #Feature BuCaMH04\_B15 | Start | End | PTM | AScore | Found By |
| --- | --- | --- | --- | --- | --- | --- | --- | --- | --- | --- | --- | --- | --- | --- | --- | --- | --- | --- | --- |
| H.NTQLPFIYK.T | Y | 52.80 | 1122.6073 | 9 | 1.7 | 562.3119 | 2 | 24.67 | 1 | 12905 | BuCaMH04.raw | 0 | 0 | 0 | 26 | 34 |  |  | PEAKS DB |
| total 1 peptides |  |  |  |  |  |  |  |  |  |  |  |  |  |  |  |  |  |  |  |
| --- | --- | --- | --- | --- | --- | --- | --- | --- | --- | --- | --- | --- | --- | --- | --- | --- | --- | --- | --- |

AAB36929.1

back to list

  

| Protein Coverage
| Supporting Peptides
|

Protein Coverage:

Supporting Peptides:

| Peptide | Uniq | -10lgP | Mass | Length | ppm | m/z | z | RT | Fraction | Scan | Source File | Area BuCaMH04\_B15 | #Feature | #Feature BuCaMH04\_B15 | Start | End | PTM | AScore | Found By |
| --- | --- | --- | --- | --- | --- | --- | --- | --- | --- | --- | --- | --- | --- | --- | --- | --- | --- | --- | --- |
| H.NTQLPFIYK.T | Y | 52.80 | 1122.6073 | 9 | 1.7 | 562.3119 | 2 | 24.67 | 1 | 12905 | BuCaMH04.raw | 0 | 0 | 0 | 26 | 34 |  |  | PEAKS DB |
| total 1 peptides |  |  |  |  |  |  |  |  |  |  |  |  |  |  |  |  |  |  |  |
| --- | --- | --- | --- | --- | --- | --- | --- | --- | --- | --- | --- | --- | --- | --- | --- | --- | --- | --- | --- |

CAA63979.1

back to list

  

| Protein Coverage
| Supporting Peptides
|

Protein Coverage:

Supporting Peptides:

| Peptide | Uniq | -10lgP | Mass | Length | ppm | m/z | z | RT | Fraction | Scan | Source File | Area BuCaMH04\_B15 | #Feature | #Feature BuCaMH04\_B15 | Start | End | PTM | AScore | Found By |
| --- | --- | --- | --- | --- | --- | --- | --- | --- | --- | --- | --- | --- | --- | --- | --- | --- | --- | --- | --- |
| H.NTQLPFIYK.T | Y | 52.80 | 1122.6073 | 9 | 1.7 | 562.3119 | 2 | 24.67 | 1 | 12905 | BuCaMH04.raw | 0 | 0 | 0 | 26 | 34 |  |  | PEAKS DB |
| total 1 peptides |  |  |  |  |  |  |  |  |  |  |  |  |  |  |  |  |  |  |  |
| --- | --- | --- | --- | --- | --- | --- | --- | --- | --- | --- | --- | --- | --- | --- | --- | --- | --- | --- | --- |

P62375.1

back to list

  

| Protein Coverage
| Supporting Peptides
|

Protein Coverage:

Supporting Peptides:

| Peptide | Uniq | -10lgP | Mass | Length | ppm | m/z | z | RT | Fraction | Scan | Source File | Area BuCaMH04\_B15 | #Feature | #Feature BuCaMH04\_B15 | Start | End | PTM | AScore | Found By |
| --- | --- | --- | --- | --- | --- | --- | --- | --- | --- | --- | --- | --- | --- | --- | --- | --- | --- | --- | --- |
| H.NTQLPFIYK.T | Y | 52.80 | 1122.6073 | 9 | 1.7 | 562.3119 | 2 | 24.67 | 1 | 12905 | BuCaMH04.raw | 0 | 0 | 0 | 26 | 34 |  |  | PEAKS DB |
| total 1 peptides |  |  |  |  |  |  |  |  |  |  |  |  |  |  |  |  |  |  |  |
| --- | --- | --- | --- | --- | --- | --- | --- | --- | --- | --- | --- | --- | --- | --- | --- | --- | --- | --- | --- |

AAB03220.1

back to list

  

| Protein Coverage
| Supporting Peptides
|

Protein Coverage:

Supporting Peptides:

| Peptide | Uniq | -10lgP | Mass | Length | ppm | m/z | z | RT | Fraction | Scan | Source File | Area BuCaMH04\_B15 | #Feature | #Feature BuCaMH04\_B15 | Start | End | PTM | AScore | Found By |
| --- | --- | --- | --- | --- | --- | --- | --- | --- | --- | --- | --- | --- | --- | --- | --- | --- | --- | --- | --- |
| H.NTQLPFIYK.T | Y | 52.80 | 1122.6073 | 9 | 1.7 | 562.3119 | 2 | 24.67 | 1 | 12905 | BuCaMH04.raw | 0 | 0 | 0 | 26 | 34 |  |  | PEAKS DB |
| total 1 peptides |  |  |  |  |  |  |  |  |  |  |  |  |  |  |  |  |  |  |  |
| --- | --- | --- | --- | --- | --- | --- | --- | --- | --- | --- | --- | --- | --- | --- | --- | --- | --- | --- | --- |

AAB03219.1

back to list

  

| Protein Coverage
| Supporting Peptides
|

Protein Coverage:

Supporting Peptides:

| Peptide | Uniq | -10lgP | Mass | Length | ppm | m/z | z | RT | Fraction | Scan | Source File | Area BuCaMH04\_B15 | #Feature | #Feature BuCaMH04\_B15 | Start | End | PTM | AScore | Found By |
| --- | --- | --- | --- | --- | --- | --- | --- | --- | --- | --- | --- | --- | --- | --- | --- | --- | --- | --- | --- |
| H.NTQLPFIYK.T | Y | 52.80 | 1122.6073 | 9 | 1.7 | 562.3119 | 2 | 24.67 | 1 | 12905 | BuCaMH04.raw | 0 | 0 | 0 | 26 | 34 |  |  | PEAKS DB |
| total 1 peptides |  |  |  |  |  |  |  |  |  |  |  |  |  |  |  |  |  |  |  |
| --- | --- | --- | --- | --- | --- | --- | --- | --- | --- | --- | --- | --- | --- | --- | --- | --- | --- | --- | --- |

Q9W716.1

back to list

  

| Protein Coverage
| Supporting Peptides
|

Protein Coverage:

Supporting Peptides:

| Peptide | Uniq | -10lgP | Mass | Length | ppm | m/z | z | RT | Fraction | Scan | Source File | Area BuCaMH04\_B15 | #Feature | #Feature BuCaMH04\_B15 | Start | End | PTM | AScore | Found By |
| --- | --- | --- | --- | --- | --- | --- | --- | --- | --- | --- | --- | --- | --- | --- | --- | --- | --- | --- | --- |
| H.NTQLPFIYK.T | Y | 52.80 | 1122.6073 | 9 | 1.7 | 562.3119 | 2 | 24.67 | 1 | 12905 | BuCaMH04.raw | 0 | 0 | 0 | 26 | 34 |  |  | PEAKS DB |
| total 1 peptides |  |  |  |  |  |  |  |  |  |  |  |  |  |  |  |  |  |  |  |
| --- | --- | --- | --- | --- | --- | --- | --- | --- | --- | --- | --- | --- | --- | --- | --- | --- | --- | --- | --- |

AAB18387.1

back to list

  

| Protein Coverage
| Supporting Peptides
|

Protein Coverage:

Supporting Peptides:

| Peptide | Uniq | -10lgP | Mass | Length | ppm | m/z | z | RT | Fraction | Scan | Source File | Area BuCaMH04\_B15 | #Feature | #Feature BuCaMH04\_B15 | Start | End | PTM | AScore | Found By |
| --- | --- | --- | --- | --- | --- | --- | --- | --- | --- | --- | --- | --- | --- | --- | --- | --- | --- | --- | --- |
| H.NTQLPFIYK.T | Y | 52.80 | 1122.6073 | 9 | 1.7 | 562.3119 | 2 | 24.67 | 1 | 12905 | BuCaMH04.raw | 0 | 0 | 0 | 26 | 34 |  |  | PEAKS DB |
| total 1 peptides |  |  |  |  |  |  |  |  |  |  |  |  |  |  |  |  |  |  |  |
| --- | --- | --- | --- | --- | --- | --- | --- | --- | --- | --- | --- | --- | --- | --- | --- | --- | --- | --- | --- |

CAA90964.1

back to list

  

| Protein Coverage
| Supporting Peptides
|

Protein Coverage:

Supporting Peptides:

| Peptide | Uniq | -10lgP | Mass | Length | ppm | m/z | z | RT | Fraction | Scan | Source File | Area BuCaMH04\_B15 | #Feature | #Feature BuCaMH04\_B15 | Start | End | PTM | AScore | Found By |
| --- | --- | --- | --- | --- | --- | --- | --- | --- | --- | --- | --- | --- | --- | --- | --- | --- | --- | --- | --- |
| H.NTQLPFIYK.T | Y | 52.80 | 1122.6073 | 9 | 1.7 | 562.3119 | 2 | 24.67 | 1 | 12905 | BuCaMH04.raw | 0 | 0 | 0 | 26 | 34 |  |  | PEAKS DB |
| total 1 peptides |  |  |  |  |  |  |  |  |  |  |  |  |  |  |  |  |  |  |  |
| --- | --- | --- | --- | --- | --- | --- | --- | --- | --- | --- | --- | --- | --- | --- | --- | --- | --- | --- | --- |

Q91126.1

back to list

  

| Protein Coverage
| Supporting Peptides
|

Protein Coverage:

Supporting Peptides:

| Peptide | Uniq | -10lgP | Mass | Length | ppm | m/z | z | RT | Fraction | Scan | Source File | Area BuCaMH04\_B15 | #Feature | #Feature BuCaMH04\_B15 | Start | End | PTM | AScore | Found By |
| --- | --- | --- | --- | --- | --- | --- | --- | --- | --- | --- | --- | --- | --- | --- | --- | --- | --- | --- | --- |
| H.NTQLPFIYK.T | Y | 52.80 | 1122.6073 | 9 | 1.7 | 562.3119 | 2 | 24.67 | 1 | 12905 | BuCaMH04.raw | 0 | 0 | 0 | 26 | 34 |  |  | PEAKS DB |
| total 1 peptides |  |  |  |  |  |  |  |  |  |  |  |  |  |  |  |  |  |  |  |
| --- | --- | --- | --- | --- | --- | --- | --- | --- | --- | --- | --- | --- | --- | --- | --- | --- | --- | --- | --- |

AAB01540.1

back to list

  

| Protein Coverage
| Supporting Peptides
|

Protein Coverage:

Supporting Peptides:

| Peptide | Uniq | -10lgP | Mass | Length | ppm | m/z | z | RT | Fraction | Scan | Source File | Area BuCaMH04\_B15 | #Feature | #Feature BuCaMH04\_B15 | Start | End | PTM | AScore | Found By |
| --- | --- | --- | --- | --- | --- | --- | --- | --- | --- | --- | --- | --- | --- | --- | --- | --- | --- | --- | --- |
| H.NTQLPFIYK.T | Y | 52.80 | 1122.6073 | 9 | 1.7 | 562.3119 | 2 | 24.67 | 1 | 12905 | BuCaMH04.raw | 0 | 0 | 0 | 26 | 34 |  |  | PEAKS DB |
| total 1 peptides |  |  |  |  |  |  |  |  |  |  |  |  |  |  |  |  |  |  |  |
| --- | --- | --- | --- | --- | --- | --- | --- | --- | --- | --- | --- | --- | --- | --- | --- | --- | --- | --- | --- |

P0C547.1

back to list

  

| Protein Coverage
| Supporting Peptides
|

Protein Coverage:

Supporting Peptides:

| Peptide | Uniq | -10lgP | Mass | Length | ppm | m/z | z | RT | Fraction | Scan | Source File | Area BuCaMH04\_B15 | #Feature | #Feature BuCaMH04\_B15 | Start | End | PTM | AScore | Found By |
| --- | --- | --- | --- | --- | --- | --- | --- | --- | --- | --- | --- | --- | --- | --- | --- | --- | --- | --- | --- |
| H.NTQLPFIYK.T | Y | 52.80 | 1122.6073 | 9 | 1.7 | 562.3119 | 2 | 24.67 | 1 | 12905 | BuCaMH04.raw | 0 | 0 | 0 | 5 | 13 |  |  | PEAKS DB |
| total 1 peptides |  |  |  |  |  |  |  |  |  |  |  |  |  |  |  |  |  |  |  |
| --- | --- | --- | --- | --- | --- | --- | --- | --- | --- | --- | --- | --- | --- | --- | --- | --- | --- | --- | --- |

P62394.1

back to list

  

| Protein Coverage
| Supporting Peptides
|

Protein Coverage:

Supporting Peptides:

| Peptide | Uniq | -10lgP | Mass | Length | ppm | m/z | z | RT | Fraction | Scan | Source File | Area BuCaMH04\_B15 | #Feature | #Feature BuCaMH04\_B15 | Start | End | PTM | AScore | Found By |
| --- | --- | --- | --- | --- | --- | --- | --- | --- | --- | --- | --- | --- | --- | --- | --- | --- | --- | --- | --- |
| H.NTQLPFIYK.T | Y | 52.80 | 1122.6073 | 9 | 1.7 | 562.3119 | 2 | 24.67 | 1 | 12905 | BuCaMH04.raw | 0 | 0 | 0 | 5 | 13 |  |  | PEAKS DB |
| total 1 peptides |  |  |  |  |  |  |  |  |  |  |  |  |  |  |  |  |  |  |  |
| --- | --- | --- | --- | --- | --- | --- | --- | --- | --- | --- | --- | --- | --- | --- | --- | --- | --- | --- | --- |

P14541.1

back to list

  

| Protein Coverage
| Supporting Peptides
|

Protein Coverage:

Supporting Peptides:

| Peptide | Uniq | -10lgP | Mass | Length | ppm | m/z | z | RT | Fraction | Scan | Source File | Area BuCaMH04\_B15 | #Feature | #Feature BuCaMH04\_B15 | Start | End | PTM | AScore | Found By |
| --- | --- | --- | --- | --- | --- | --- | --- | --- | --- | --- | --- | --- | --- | --- | --- | --- | --- | --- | --- |
| H.NTQLPFIYK.T | Y | 52.80 | 1122.6073 | 9 | 1.7 | 562.3119 | 2 | 24.67 | 1 | 12905 | BuCaMH04.raw | 0 | 0 | 0 | 5 | 13 |  |  | PEAKS DB |
| total 1 peptides |  |  |  |  |  |  |  |  |  |  |  |  |  |  |  |  |  |  |  |
| --- | --- | --- | --- | --- | --- | --- | --- | --- | --- | --- | --- | --- | --- | --- | --- | --- | --- | --- | --- |

P62390.1

back to list

  

| Protein Coverage
| Supporting Peptides
|

Protein Coverage:

Supporting Peptides:

| Peptide | Uniq | -10lgP | Mass | Length | ppm | m/z | z | RT | Fraction | Scan | Source File | Area BuCaMH04\_B15 | #Feature | #Feature BuCaMH04\_B15 | Start | End | PTM | AScore | Found By |
| --- | --- | --- | --- | --- | --- | --- | --- | --- | --- | --- | --- | --- | --- | --- | --- | --- | --- | --- | --- |
| H.NTQLPFIYK.T | Y | 52.80 | 1122.6073 | 9 | 1.7 | 562.3119 | 2 | 24.67 | 1 | 12905 | BuCaMH04.raw | 0 | 0 | 0 | 5 | 13 |  |  | PEAKS DB |
| total 1 peptides |  |  |  |  |  |  |  |  |  |  |  |  |  |  |  |  |  |  |  |
| --- | --- | --- | --- | --- | --- | --- | --- | --- | --- | --- | --- | --- | --- | --- | --- | --- | --- | --- | --- |

JAA74859.1

back to list

  

| Protein Coverage
| Supporting Peptides
|

Protein Coverage:

Supporting Peptides:

| Peptide | Uniq | -10lgP | Mass | Length | ppm | m/z | z | RT | Fraction | Scan | Source File | Area BuCaMH04\_B15 | #Feature | #Feature BuCaMH04\_B15 | Start | End | PTM | AScore | Found By |
| --- | --- | --- | --- | --- | --- | --- | --- | --- | --- | --- | --- | --- | --- | --- | --- | --- | --- | --- | --- |
| K.C(+57.02)PIMTNQC(+57.02)IALR.G | N | 52.31 | 1475.7047 | 12 | -0.2 | 738.8595 | 2 | 38.34 | 1 | 25350 | BuCaMH04.raw | 4.1161E5 | 1 | 1 | 419 | 430 | Carbamidomethylation | C1:Carbamidomethylation:1000.00;C8:Carbamidomethylation:1000.00 | PEAKS DB |
| total 1 peptides |  |  |  |  |  |  |  |  |  |  |  |  |  |  |  |  |  |  |  |
| --- | --- | --- | --- | --- | --- | --- | --- | --- | --- | --- | --- | --- | --- | --- | --- | --- | --- | --- | --- |

XP\_026555881.1

back to list

  

| Protein Coverage
| Supporting Peptides
|

Protein Coverage:

Supporting Peptides:

| Peptide | Uniq | -10lgP | Mass | Length | ppm | m/z | z | RT | Fraction | Scan | Source File | Area BuCaMH04\_B15 | #Feature | #Feature BuCaMH04\_B15 | Start | End | PTM | AScore | Found By |
| --- | --- | --- | --- | --- | --- | --- | --- | --- | --- | --- | --- | --- | --- | --- | --- | --- | --- | --- | --- |
| R.WQTLLSVDDLIEK.L | Y | 71.91 | 1558.8242 | 13 | 1.0 | 780.4202 | 2 | 44.28 | 1 | 30668 | BuCaMH04.raw | 2.3471E5 | 1 | 1 | 281 | 293 |  |  | PEAKS DB |
| total 1 peptides |  |  |  |  |  |  |  |  |  |  |  |  |  |  |  |  |  |  |  |
| --- | --- | --- | --- | --- | --- | --- | --- | --- | --- | --- | --- | --- | --- | --- | --- | --- | --- | --- | --- |

XP\_034259005.1

back to list

  

| Protein Coverage
| Supporting Peptides
|

Protein Coverage:

Supporting Peptides:

| Peptide | Uniq | -10lgP | Mass | Length | ppm | m/z | z | RT | Fraction | Scan | Source File | Area BuCaMH04\_B15 | #Feature | #Feature BuCaMH04\_B15 | Start | End | PTM | AScore | Found By |
| --- | --- | --- | --- | --- | --- | --- | --- | --- | --- | --- | --- | --- | --- | --- | --- | --- | --- | --- | --- |
| R.WQTLLSVDDLIEK.L | Y | 71.91 | 1558.8242 | 13 | 1.0 | 780.4202 | 2 | 44.28 | 1 | 30668 | BuCaMH04.raw | 2.3471E5 | 1 | 1 | 285 | 297 |  |  | PEAKS DB |
| total 1 peptides |  |  |  |  |  |  |  |  |  |  |  |  |  |  |  |  |  |  |  |
| --- | --- | --- | --- | --- | --- | --- | --- | --- | --- | --- | --- | --- | --- | --- | --- | --- | --- | --- | --- |

ETE66000.1

back to list

  

| Protein Coverage
| Supporting Peptides
|

Protein Coverage:

Supporting Peptides:

| Peptide | Uniq | -10lgP | Mass | Length | ppm | m/z | z | RT | Fraction | Scan | Source File | Area BuCaMH04\_B15 | #Feature | #Feature BuCaMH04\_B15 | Start | End | PTM | AScore | Found By |
| --- | --- | --- | --- | --- | --- | --- | --- | --- | --- | --- | --- | --- | --- | --- | --- | --- | --- | --- | --- |
| R.WQTLLSVDDLIEK.L | Y | 71.91 | 1558.8242 | 13 | 1.0 | 780.4202 | 2 | 44.28 | 1 | 30668 | BuCaMH04.raw | 2.3471E5 | 1 | 1 | 275 | 287 |  |  | PEAKS DB |
| total 1 peptides |  |  |  |  |  |  |  |  |  |  |  |  |  |  |  |  |  |  |  |
| --- | --- | --- | --- | --- | --- | --- | --- | --- | --- | --- | --- | --- | --- | --- | --- | --- | --- | --- | --- |

LAA18030.1

back to list

  

| Protein Coverage
| Supporting Peptides
|

Protein Coverage:

Supporting Peptides:

| Peptide | Uniq | -10lgP | Mass | Length | ppm | m/z | z | RT | Fraction | Scan | Source File | Area BuCaMH04\_B15 | #Feature | #Feature BuCaMH04\_B15 | Start | End | PTM | AScore | Found By |
| --- | --- | --- | --- | --- | --- | --- | --- | --- | --- | --- | --- | --- | --- | --- | --- | --- | --- | --- | --- |
| R.WQTLLSVDDLIEK.L | Y | 71.91 | 1558.8242 | 13 | 1.0 | 780.4202 | 2 | 44.28 | 1 | 30668 | BuCaMH04.raw | 2.3471E5 | 1 | 1 | 312 | 324 |  |  | PEAKS DB |
| total 1 peptides |  |  |  |  |  |  |  |  |  |  |  |  |  |  |  |  |  |  |  |
| --- | --- | --- | --- | --- | --- | --- | --- | --- | --- | --- | --- | --- | --- | --- | --- | --- | --- | --- | --- |

LAA85609.1

back to list

  

| Protein Coverage
| Supporting Peptides
|

Protein Coverage:

Supporting Peptides:

| Peptide | Uniq | -10lgP | Mass | Length | ppm | m/z | z | RT | Fraction | Scan | Source File | Area BuCaMH04\_B15 | #Feature | #Feature BuCaMH04\_B15 | Start | End | PTM | AScore | Found By |
| --- | --- | --- | --- | --- | --- | --- | --- | --- | --- | --- | --- | --- | --- | --- | --- | --- | --- | --- | --- |
| R.WQTLLSVDDLIEK.L | Y | 71.91 | 1558.8242 | 13 | 1.0 | 780.4202 | 2 | 44.28 | 1 | 30668 | BuCaMH04.raw | 2.3471E5 | 1 | 1 | 285 | 297 |  |  | PEAKS DB |
| total 1 peptides |  |  |  |  |  |  |  |  |  |  |  |  |  |  |  |  |  |  |  |
| --- | --- | --- | --- | --- | --- | --- | --- | --- | --- | --- | --- | --- | --- | --- | --- | --- | --- | --- | --- |

LAA85607.1

back to list

  

| Protein Coverage
| Supporting Peptides
|

Protein Coverage:

Supporting Peptides:

| Peptide | Uniq | -10lgP | Mass | Length | ppm | m/z | z | RT | Fraction | Scan | Source File | Area BuCaMH04\_B15 | #Feature | #Feature BuCaMH04\_B15 | Start | End | PTM | AScore | Found By |
| --- | --- | --- | --- | --- | --- | --- | --- | --- | --- | --- | --- | --- | --- | --- | --- | --- | --- | --- | --- |
| R.WQTLLSVDDLIEK.L | Y | 71.91 | 1558.8242 | 13 | 1.0 | 780.4202 | 2 | 44.28 | 1 | 30668 | BuCaMH04.raw | 2.3471E5 | 1 | 1 | 285 | 297 |  |  | PEAKS DB |
| total 1 peptides |  |  |  |  |  |  |  |  |  |  |  |  |  |  |  |  |  |  |  |
| --- | --- | --- | --- | --- | --- | --- | --- | --- | --- | --- | --- | --- | --- | --- | --- | --- | --- | --- | --- |

XP\_026523569.1

back to list

  

| Protein Coverage
| Supporting Peptides
|

Protein Coverage:

Supporting Peptides:

| Peptide | Uniq | -10lgP | Mass | Length | ppm | m/z | z | RT | Fraction | Scan | Source File | Area BuCaMH04\_B15 | #Feature | #Feature BuCaMH04\_B15 | Start | End | PTM | AScore | Found By |
| --- | --- | --- | --- | --- | --- | --- | --- | --- | --- | --- | --- | --- | --- | --- | --- | --- | --- | --- | --- |
| R.WQTLLSVDDLIEK.L | Y | 71.91 | 1558.8242 | 13 | 1.0 | 780.4202 | 2 | 44.28 | 1 | 30668 | BuCaMH04.raw | 2.3471E5 | 1 | 1 | 281 | 293 |  |  | PEAKS DB |
| total 1 peptides |  |  |  |  |  |  |  |  |  |  |  |  |  |  |  |  |  |  |  |
| --- | --- | --- | --- | --- | --- | --- | --- | --- | --- | --- | --- | --- | --- | --- | --- | --- | --- | --- | --- |

XP\_032076778.1

back to list

  

| Protein Coverage
| Supporting Peptides
|

Protein Coverage:

Supporting Peptides:

| Peptide | Uniq | -10lgP | Mass | Length | ppm | m/z | z | RT | Fraction | Scan | Source File | Area BuCaMH04\_B15 | #Feature | #Feature BuCaMH04\_B15 | Start | End | PTM | AScore | Found By |
| --- | --- | --- | --- | --- | --- | --- | --- | --- | --- | --- | --- | --- | --- | --- | --- | --- | --- | --- | --- |
| R.WQTLLSVDDLIEK.L | Y | 71.91 | 1558.8242 | 13 | 1.0 | 780.4202 | 2 | 44.28 | 1 | 30668 | BuCaMH04.raw | 2.3471E5 | 1 | 1 | 285 | 297 |  |  | PEAKS DB |
| total 1 peptides |  |  |  |  |  |  |  |  |  |  |  |  |  |  |  |  |  |  |  |
| --- | --- | --- | --- | --- | --- | --- | --- | --- | --- | --- | --- | --- | --- | --- | --- | --- | --- | --- | --- |

XP\_013929985.1

back to list

  

| Protein Coverage
| Supporting Peptides
|

Protein Coverage:

Supporting Peptides:

| Peptide | Uniq | -10lgP | Mass | Length | ppm | m/z | z | RT | Fraction | Scan | Source File | Area BuCaMH04\_B15 | #Feature | #Feature BuCaMH04\_B15 | Start | End | PTM | AScore | Found By |
| --- | --- | --- | --- | --- | --- | --- | --- | --- | --- | --- | --- | --- | --- | --- | --- | --- | --- | --- | --- |
| R.WQTLLSVDDLIEK.L | Y | 71.91 | 1558.8242 | 13 | 1.0 | 780.4202 | 2 | 44.28 | 1 | 30668 | BuCaMH04.raw | 2.3471E5 | 1 | 1 | 285 | 297 |  |  | PEAKS DB |
| total 1 peptides |  |  |  |  |  |  |  |  |  |  |  |  |  |  |  |  |  |  |  |
| --- | --- | --- | --- | --- | --- | --- | --- | --- | --- | --- | --- | --- | --- | --- | --- | --- | --- | --- | --- |

1T37

back to list

  

| Protein Coverage
| Supporting Peptides
|

Protein Coverage:

Supporting Peptides:

| Peptide | Uniq | -10lgP | Mass | Length | ppm | m/z | z | RT | Fraction | Scan | Source File | Area BuCaMH04\_B15 | #Feature | #Feature BuCaMH04\_B15 | Start | End | PTM | AScore | Found By |
| --- | --- | --- | --- | --- | --- | --- | --- | --- | --- | --- | --- | --- | --- | --- | --- | --- | --- | --- | --- |
| R.LAAIC(+57.02)FAGAPYN.D | N | 52.26 | 1266.6067 | 12 | -0.4 | 634.3104 | 2 | 35.93 | 1 | 23316 | BuCaMH04.raw | 9.6858E6 | 1 | 1 | 95 | 106 | Carbamidomethylation | C5:Carbamidomethylation:1000.00 | PEAKS DB |
| total 1 peptides |  |  |  |  |  |  |  |  |  |  |  |  |  |  |  |  |  |  |  |
| --- | --- | --- | --- | --- | --- | --- | --- | --- | --- | --- | --- | --- | --- | --- | --- | --- | --- | --- | --- |

1ZM6

back to list

  

| Protein Coverage
| Supporting Peptides
|

Protein Coverage:

Supporting Peptides:

| Peptide | Uniq | -10lgP | Mass | Length | ppm | m/z | z | RT | Fraction | Scan | Source File | Area BuCaMH04\_B15 | #Feature | #Feature BuCaMH04\_B15 | Start | End | PTM | AScore | Found By |
| --- | --- | --- | --- | --- | --- | --- | --- | --- | --- | --- | --- | --- | --- | --- | --- | --- | --- | --- | --- |
| R.LAAIC(+57.02)FAGAPYN.D | N | 52.26 | 1266.6067 | 12 | -0.4 | 634.3104 | 2 | 35.93 | 1 | 23316 | BuCaMH04.raw | 9.6858E6 | 1 | 1 | 95 | 106 | Carbamidomethylation | C5:Carbamidomethylation:1000.00 | PEAKS DB |
| total 1 peptides |  |  |  |  |  |  |  |  |  |  |  |  |  |  |  |  |  |  |  |
| --- | --- | --- | --- | --- | --- | --- | --- | --- | --- | --- | --- | --- | --- | --- | --- | --- | --- | --- | --- |

T\_79

back to list

  

| Protein Coverage
| Supporting Peptides
|

Protein Coverage:

Supporting Peptides:

| Peptide | Uniq | -10lgP | Mass | Length | ppm | m/z | z | RT | Fraction | Scan | Source File | Area BuCaMH04\_B15 | #Feature | #Feature BuCaMH04\_B15 | Start | End | PTM | AScore | Found By |
| --- | --- | --- | --- | --- | --- | --- | --- | --- | --- | --- | --- | --- | --- | --- | --- | --- | --- | --- | --- |
| K.YSFQTIDNDIALLR.L | Y | 71.01 | 1667.8518 | 14 | 1.6 | 834.9345 | 2 | 44.05 | 1 | 30486 | BuCaMH04.raw | 2.6904E5 | 1 | 1 | 271 | 284 |  |  | PEAKS DB |
| total 1 peptides |  |  |  |  |  |  |  |  |  |  |  |  |  |  |  |  |  |  |  |
| --- | --- | --- | --- | --- | --- | --- | --- | --- | --- | --- | --- | --- | --- | --- | --- | --- | --- | --- | --- |

XP\_034269327.1

back to list

  

| Protein Coverage
| Supporting Peptides
|

Protein Coverage:

Supporting Peptides:

| Peptide | Uniq | -10lgP | Mass | Length | ppm | m/z | z | RT | Fraction | Scan | Source File | Area BuCaMH04\_B15 | #Feature | #Feature BuCaMH04\_B15 | Start | End | PTM | AScore | Found By |
| --- | --- | --- | --- | --- | --- | --- | --- | --- | --- | --- | --- | --- | --- | --- | --- | --- | --- | --- | --- |
| K.IPGIYVLSLK.I | Y | 70.41 | 1101.6797 | 10 | 1.6 | 551.8480 | 2 | 34.90 | 1 | 22318 | BuCaMH04.raw | 2.5623E5 | 1 | 1 | 12 | 21 |  |  | PEAKS DB |
| total 1 peptides |  |  |  |  |  |  |  |  |  |  |  |  |  |  |  |  |  |  |  |
| --- | --- | --- | --- | --- | --- | --- | --- | --- | --- | --- | --- | --- | --- | --- | --- | --- | --- | --- | --- |

XP\_013915921.1

back to list

  

| Protein Coverage
| Supporting Peptides
|

Protein Coverage:

Supporting Peptides:

| Peptide | Uniq | -10lgP | Mass | Length | ppm | m/z | z | RT | Fraction | Scan | Source File | Area BuCaMH04\_B15 | #Feature | #Feature BuCaMH04\_B15 | Start | End | PTM | AScore | Found By |
| --- | --- | --- | --- | --- | --- | --- | --- | --- | --- | --- | --- | --- | --- | --- | --- | --- | --- | --- | --- |
| K.IPGIYVLSLK.I | Y | 70.41 | 1101.6797 | 10 | 1.6 | 551.8480 | 2 | 34.90 | 1 | 22318 | BuCaMH04.raw | 2.5623E5 | 1 | 1 | 22 | 31 |  |  | PEAKS DB |
| total 1 peptides |  |  |  |  |  |  |  |  |  |  |  |  |  |  |  |  |  |  |  |
| --- | --- | --- | --- | --- | --- | --- | --- | --- | --- | --- | --- | --- | --- | --- | --- | --- | --- | --- | --- |

XP\_015686279.2

back to list

  

| Protein Coverage
| Supporting Peptides
|

Protein Coverage:

Supporting Peptides:

| Peptide | Uniq | -10lgP | Mass | Length | ppm | m/z | z | RT | Fraction | Scan | Source File | Area BuCaMH04\_B15 | #Feature | #Feature BuCaMH04\_B15 | Start | End | PTM | AScore | Found By |
| --- | --- | --- | --- | --- | --- | --- | --- | --- | --- | --- | --- | --- | --- | --- | --- | --- | --- | --- | --- |
| K.IPGIYVLSLK.I | Y | 70.41 | 1101.6797 | 10 | 1.6 | 551.8480 | 2 | 34.90 | 1 | 22318 | BuCaMH04.raw | 2.5623E5 | 1 | 1 | 22 | 31 |  |  | PEAKS DB |
| total 1 peptides |  |  |  |  |  |  |  |  |  |  |  |  |  |  |  |  |  |  |  |
| --- | --- | --- | --- | --- | --- | --- | --- | --- | --- | --- | --- | --- | --- | --- | --- | --- | --- | --- | --- |

XP\_026536429.1

back to list

  

| Protein Coverage
| Supporting Peptides
|

Protein Coverage:

Supporting Peptides:

| Peptide | Uniq | -10lgP | Mass | Length | ppm | m/z | z | RT | Fraction | Scan | Source File | Area BuCaMH04\_B15 | #Feature | #Feature BuCaMH04\_B15 | Start | End | PTM | AScore | Found By |
| --- | --- | --- | --- | --- | --- | --- | --- | --- | --- | --- | --- | --- | --- | --- | --- | --- | --- | --- | --- |
| K.IPGIYVLSLK.I | Y | 70.41 | 1101.6797 | 10 | 1.6 | 551.8480 | 2 | 34.90 | 1 | 22318 | BuCaMH04.raw | 2.5623E5 | 1 | 1 | 60 | 69 |  |  | PEAKS DB |
| total 1 peptides |  |  |  |  |  |  |  |  |  |  |  |  |  |  |  |  |  |  |  |
| --- | --- | --- | --- | --- | --- | --- | --- | --- | --- | --- | --- | --- | --- | --- | --- | --- | --- | --- | --- |

XP\_026558426.1

back to list

  

| Protein Coverage
| Supporting Peptides
|

Protein Coverage:

Supporting Peptides:

| Peptide | Uniq | -10lgP | Mass | Length | ppm | m/z | z | RT | Fraction | Scan | Source File | Area BuCaMH04\_B15 | #Feature | #Feature BuCaMH04\_B15 | Start | End | PTM | AScore | Found By |
| --- | --- | --- | --- | --- | --- | --- | --- | --- | --- | --- | --- | --- | --- | --- | --- | --- | --- | --- | --- |
| K.IPGIYVLSLK.I | Y | 70.41 | 1101.6797 | 10 | 1.6 | 551.8480 | 2 | 34.90 | 1 | 22318 | BuCaMH04.raw | 2.5623E5 | 1 | 1 | 60 | 69 |  |  | PEAKS DB |
| total 1 peptides |  |  |  |  |  |  |  |  |  |  |  |  |  |  |  |  |  |  |  |
| --- | --- | --- | --- | --- | --- | --- | --- | --- | --- | --- | --- | --- | --- | --- | --- | --- | --- | --- | --- |

XP\_007430564.1

back to list

  

| Protein Coverage
| Supporting Peptides
|

Protein Coverage:

Supporting Peptides:

| Peptide | Uniq | -10lgP | Mass | Length | ppm | m/z | z | RT | Fraction | Scan | Source File | Area BuCaMH04\_B15 | #Feature | #Feature BuCaMH04\_B15 | Start | End | PTM | AScore | Found By |
| --- | --- | --- | --- | --- | --- | --- | --- | --- | --- | --- | --- | --- | --- | --- | --- | --- | --- | --- | --- |
| K.IPGIYVLSLK.I | Y | 70.41 | 1101.6797 | 10 | 1.6 | 551.8480 | 2 | 34.90 | 1 | 22318 | BuCaMH04.raw | 2.5623E5 | 1 | 1 | 63 | 72 |  |  | PEAKS DB |
| total 1 peptides |  |  |  |  |  |  |  |  |  |  |  |  |  |  |  |  |  |  |  |
| --- | --- | --- | --- | --- | --- | --- | --- | --- | --- | --- | --- | --- | --- | --- | --- | --- | --- | --- | --- |

XP\_032084406.1

back to list

  

| Protein Coverage
| Supporting Peptides
|

Protein Coverage:

Supporting Peptides:

| Peptide | Uniq | -10lgP | Mass | Length | ppm | m/z | z | RT | Fraction | Scan | Source File | Area BuCaMH04\_B15 | #Feature | #Feature BuCaMH04\_B15 | Start | End | PTM | AScore | Found By |
| --- | --- | --- | --- | --- | --- | --- | --- | --- | --- | --- | --- | --- | --- | --- | --- | --- | --- | --- | --- |
| K.IPGIYVLSLK.I | Y | 70.41 | 1101.6797 | 10 | 1.6 | 551.8480 | 2 | 34.90 | 1 | 22318 | BuCaMH04.raw | 2.5623E5 | 1 | 1 | 59 | 68 |  |  | PEAKS DB |
| total 1 peptides |  |  |  |  |  |  |  |  |  |  |  |  |  |  |  |  |  |  |  |
| --- | --- | --- | --- | --- | --- | --- | --- | --- | --- | --- | --- | --- | --- | --- | --- | --- | --- | --- | --- |

XP\_034269326.1

back to list

  

| Protein Coverage
| Supporting Peptides
|

Protein Coverage:

Supporting Peptides:

| Peptide | Uniq | -10lgP | Mass | Length | ppm | m/z | z | RT | Fraction | Scan | Source File | Area BuCaMH04\_B15 | #Feature | #Feature BuCaMH04\_B15 | Start | End | PTM | AScore | Found By |
| --- | --- | --- | --- | --- | --- | --- | --- | --- | --- | --- | --- | --- | --- | --- | --- | --- | --- | --- | --- |
| K.IPGIYVLSLK.I | Y | 70.41 | 1101.6797 | 10 | 1.6 | 551.8480 | 2 | 34.90 | 1 | 22318 | BuCaMH04.raw | 2.5623E5 | 1 | 1 | 60 | 69 |  |  | PEAKS DB |
| total 1 peptides |  |  |  |  |  |  |  |  |  |  |  |  |  |  |  |  |  |  |  |
| --- | --- | --- | --- | --- | --- | --- | --- | --- | --- | --- | --- | --- | --- | --- | --- | --- | --- | --- | --- |

LAA21880.1

back to list

  

| Protein Coverage
| Supporting Peptides
|

Protein Coverage:

Supporting Peptides:

| Peptide | Uniq | -10lgP | Mass | Length | ppm | m/z | z | RT | Fraction | Scan | Source File | Area BuCaMH04\_B15 | #Feature | #Feature BuCaMH04\_B15 | Start | End | PTM | AScore | Found By |
| --- | --- | --- | --- | --- | --- | --- | --- | --- | --- | --- | --- | --- | --- | --- | --- | --- | --- | --- | --- |
| R.EADAITLDGGHIFTAGK.C | Y | 69.46 | 1714.8525 | 17 | 0.5 | 572.6251 | 3 | 22.85 | 1 | 11378 | BuCaMH04.raw | 5.3487E5 | 1 | 1 | 393 | 409 |  |  | PEAKS DB |
| total 1 peptides |  |  |  |  |  |  |  |  |  |  |  |  |  |  |  |  |  |  |  |
| --- | --- | --- | --- | --- | --- | --- | --- | --- | --- | --- | --- | --- | --- | --- | --- | --- | --- | --- | --- |

LAA21878.1

back to list

  

| Protein Coverage
| Supporting Peptides
|

Protein Coverage:

Supporting Peptides:

| Peptide | Uniq | -10lgP | Mass | Length | ppm | m/z | z | RT | Fraction | Scan | Source File | Area BuCaMH04\_B15 | #Feature | #Feature BuCaMH04\_B15 | Start | End | PTM | AScore | Found By |
| --- | --- | --- | --- | --- | --- | --- | --- | --- | --- | --- | --- | --- | --- | --- | --- | --- | --- | --- | --- |
| R.EADAITLDGGHIFTAGK.C | Y | 69.46 | 1714.8525 | 17 | 0.5 | 572.6251 | 3 | 22.85 | 1 | 11378 | BuCaMH04.raw | 5.3487E5 | 1 | 1 | 393 | 409 |  |  | PEAKS DB |
| total 1 peptides |  |  |  |  |  |  |  |  |  |  |  |  |  |  |  |  |  |  |  |
| --- | --- | --- | --- | --- | --- | --- | --- | --- | --- | --- | --- | --- | --- | --- | --- | --- | --- | --- | --- |

XP\_026547769.1

back to list

  

| Protein Coverage
| Supporting Peptides
|

Protein Coverage:

Supporting Peptides:

| Peptide | Uniq | -10lgP | Mass | Length | ppm | m/z | z | RT | Fraction | Scan | Source File | Area BuCaMH04\_B15 | #Feature | #Feature BuCaMH04\_B15 | Start | End | PTM | AScore | Found By |
| --- | --- | --- | --- | --- | --- | --- | --- | --- | --- | --- | --- | --- | --- | --- | --- | --- | --- | --- | --- |
| R.EADAITLDGGHIFTAGK.C | Y | 69.46 | 1714.8525 | 17 | 0.5 | 572.6251 | 3 | 22.85 | 1 | 11378 | BuCaMH04.raw | 5.3487E5 | 1 | 1 | 405 | 421 |  |  | PEAKS DB |
| total 1 peptides |  |  |  |  |  |  |  |  |  |  |  |  |  |  |  |  |  |  |  |
| --- | --- | --- | --- | --- | --- | --- | --- | --- | --- | --- | --- | --- | --- | --- | --- | --- | --- | --- | --- |

XP\_026557455.1

back to list

  

| Protein Coverage
| Supporting Peptides
|

Protein Coverage:

Supporting Peptides:

| Peptide | Uniq | -10lgP | Mass | Length | ppm | m/z | z | RT | Fraction | Scan | Source File | Area BuCaMH04\_B15 | #Feature | #Feature BuCaMH04\_B15 | Start | End | PTM | AScore | Found By |
| --- | --- | --- | --- | --- | --- | --- | --- | --- | --- | --- | --- | --- | --- | --- | --- | --- | --- | --- | --- |
| R.EADAITLDGGHIFTAGK.C | Y | 69.46 | 1714.8525 | 17 | 0.5 | 572.6251 | 3 | 22.85 | 1 | 11378 | BuCaMH04.raw | 5.3487E5 | 1 | 1 | 402 | 418 |  |  | PEAKS DB |
| total 1 peptides |  |  |  |  |  |  |  |  |  |  |  |  |  |  |  |  |  |  |  |
| --- | --- | --- | --- | --- | --- | --- | --- | --- | --- | --- | --- | --- | --- | --- | --- | --- | --- | --- | --- |

XP\_013918203.1

back to list

  

| Protein Coverage
| Supporting Peptides
|

Protein Coverage:

Supporting Peptides:

| Peptide | Uniq | -10lgP | Mass | Length | ppm | m/z | z | RT | Fraction | Scan | Source File | Area BuCaMH04\_B15 | #Feature | #Feature BuCaMH04\_B15 | Start | End | PTM | AScore | Found By |
| --- | --- | --- | --- | --- | --- | --- | --- | --- | --- | --- | --- | --- | --- | --- | --- | --- | --- | --- | --- |
| total 0 peptides |  |  |  |  |  |  |  |  |  |  |  |  |  |  |  |  |  |  |  |
| --- | --- | --- | --- | --- | --- | --- | --- | --- | --- | --- | --- | --- | --- | --- | --- | --- | --- | --- | --- |

AAL54920.1

back to list

  

| Protein Coverage
| Supporting Peptides
|

Protein Coverage:

Supporting Peptides:

| Peptide | Uniq | -10lgP | Mass | Length | ppm | m/z | z | RT | Fraction | Scan | Source File | Area BuCaMH04\_B15 | #Feature | #Feature BuCaMH04\_B15 | Start | End | PTM | AScore | Found By |
| --- | --- | --- | --- | --- | --- | --- | --- | --- | --- | --- | --- | --- | --- | --- | --- | --- | --- | --- | --- |
| R.RSSLDYADYGC(+57.02)YC(+57.02)GAGGSGTPVDELDR.C | Y | 60.95 | 2940.2290 | 27 | 0.4 | 1471.1223 | 2 | 41.32 | 1 | 27990 | BuCaMH04.raw | 1.7802E7 | 1 | 1 | 44 | 70 | Carbamidomethylation | C11:Carbamidomethylation:1000.00;C13:Carbamidomethylation:1000.00 | PEAKS DB |
| total 1 peptides |  |  |  |  |  |  |  |  |  |  |  |  |  |  |  |  |  |  |  |
| --- | --- | --- | --- | --- | --- | --- | --- | --- | --- | --- | --- | --- | --- | --- | --- | --- | --- | --- | --- |

ABN54807.1

back to list

  

| Protein Coverage
| Supporting Peptides
|

Protein Coverage:

Supporting Peptides:

| Peptide | Uniq | -10lgP | Mass | Length | ppm | m/z | z | RT | Fraction | Scan | Source File | Area BuCaMH04\_B15 | #Feature | #Feature BuCaMH04\_B15 | Start | End | PTM | AScore | Found By |
| --- | --- | --- | --- | --- | --- | --- | --- | --- | --- | --- | --- | --- | --- | --- | --- | --- | --- | --- | --- |
| R.RSSLDYADYGC(+57.02)YC(+57.02)GAGGSGTPVDELDR.C | Y | 60.95 | 2940.2290 | 27 | 0.4 | 1471.1223 | 2 | 41.32 | 1 | 27990 | BuCaMH04.raw | 1.7802E7 | 1 | 1 | 44 | 70 | Carbamidomethylation | C11:Carbamidomethylation:1000.00;C13:Carbamidomethylation:1000.00 | PEAKS DB |
| total 1 peptides |  |  |  |  |  |  |  |  |  |  |  |  |  |  |  |  |  |  |  |
| --- | --- | --- | --- | --- | --- | --- | --- | --- | --- | --- | --- | --- | --- | --- | --- | --- | --- | --- | --- |

ETE72142.1

back to list

  

| Protein Coverage
| Supporting Peptides
|

Protein Coverage:

Supporting Peptides:

| Peptide | Uniq | -10lgP | Mass | Length | ppm | m/z | z | RT | Fraction | Scan | Source File | Area BuCaMH04\_B15 | #Feature | #Feature BuCaMH04\_B15 | Start | End | PTM | AScore | Found By |
| --- | --- | --- | --- | --- | --- | --- | --- | --- | --- | --- | --- | --- | --- | --- | --- | --- | --- | --- | --- |
| K.IDLNSFLLK.V | Y | 59.33 | 1061.6121 | 9 | 0.6 | 531.8136 | 2 | 41.31 | 1 | 27955 | BuCaMH04.raw | 5.98E5 | 1 | 1 | 94 | 102 |  |  | PEAKS DB |
| total 1 peptides |  |  |  |  |  |  |  |  |  |  |  |  |  |  |  |  |  |  |  |
| --- | --- | --- | --- | --- | --- | --- | --- | --- | --- | --- | --- | --- | --- | --- | --- | --- | --- | --- | --- |

AFJ51166.1

back to list

  

| Protein Coverage
| Supporting Peptides
|

Protein Coverage:

Supporting Peptides:

| Peptide | Uniq | -10lgP | Mass | Length | ppm | m/z | z | RT | Fraction | Scan | Source File | Area BuCaMH04\_B15 | #Feature | #Feature BuCaMH04\_B15 | Start | End | PTM | AScore | Found By |
| --- | --- | --- | --- | --- | --- | --- | --- | --- | --- | --- | --- | --- | --- | --- | --- | --- | --- | --- | --- |
| K.IDLNSFLLK.V | Y | 59.33 | 1061.6121 | 9 | 0.6 | 531.8136 | 2 | 41.31 | 1 | 27955 | BuCaMH04.raw | 5.98E5 | 1 | 1 | 144 | 152 |  |  | PEAKS DB |
| total 1 peptides |  |  |  |  |  |  |  |  |  |  |  |  |  |  |  |  |  |  |  |
| --- | --- | --- | --- | --- | --- | --- | --- | --- | --- | --- | --- | --- | --- | --- | --- | --- | --- | --- | --- |

XP\_013922279.1

back to list

  

| Protein Coverage
| Supporting Peptides
|

Protein Coverage:

Supporting Peptides:

| Peptide | Uniq | -10lgP | Mass | Length | ppm | m/z | z | RT | Fraction | Scan | Source File | Area BuCaMH04\_B15 | #Feature | #Feature BuCaMH04\_B15 | Start | End | PTM | AScore | Found By |
| --- | --- | --- | --- | --- | --- | --- | --- | --- | --- | --- | --- | --- | --- | --- | --- | --- | --- | --- | --- |
| R.DDC(+57.02)DFPELC(+57.02)TGR.S | Y | 56.85 | 1483.5708 | 12 | 0.7 | 742.7932 | 2 | 24.15 | 1 | 12367 | BuCaMH04.raw | 1.7318E7 | 1 | 1 | 25 | 36 | Carbamidomethylation | C3:Carbamidomethylation:1000.00;C9:Carbamidomethylation:1000.00 | PEAKS DB |
| total 1 peptides |  |  |  |  |  |  |  |  |  |  |  |  |  |  |  |  |  |  |  |
| --- | --- | --- | --- | --- | --- | --- | --- | --- | --- | --- | --- | --- | --- | --- | --- | --- | --- | --- | --- |

XP\_032084681.1

back to list

  

| Protein Coverage
| Supporting Peptides
|

Protein Coverage:

Supporting Peptides:

| Peptide | Uniq | -10lgP | Mass | Length | ppm | m/z | z | RT | Fraction | Scan | Source File | Area BuCaMH04\_B15 | #Feature | #Feature BuCaMH04\_B15 | Start | End | PTM | AScore | Found By |
| --- | --- | --- | --- | --- | --- | --- | --- | --- | --- | --- | --- | --- | --- | --- | --- | --- | --- | --- | --- |
| R.DDC(+57.02)DFPELC(+57.02)TGR.S | Y | 56.85 | 1483.5708 | 12 | 0.7 | 742.7932 | 2 | 24.15 | 1 | 12367 | BuCaMH04.raw | 1.7318E7 | 1 | 1 | 473 | 484 | Carbamidomethylation | C3:Carbamidomethylation:1000.00;C9:Carbamidomethylation:1000.00 | PEAKS DB |
| total 1 peptides |  |  |  |  |  |  |  |  |  |  |  |  |  |  |  |  |  |  |  |
| --- | --- | --- | --- | --- | --- | --- | --- | --- | --- | --- | --- | --- | --- | --- | --- | --- | --- | --- | --- |

AAL30061.1

back to list

  

| Protein Coverage
| Supporting Peptides
|

Protein Coverage:

Supporting Peptides:

| Peptide | Uniq | -10lgP | Mass | Length | ppm | m/z | z | RT | Fraction | Scan | Source File | Area BuCaMH04\_B15 | #Feature | #Feature BuCaMH04\_B15 | Start | End | PTM | AScore | Found By |
| --- | --- | --- | --- | --- | --- | --- | --- | --- | --- | --- | --- | --- | --- | --- | --- | --- | --- | --- | --- |
| L.DLGYTLTC(+57.02)LIC(+57.02)PEK.D | Y | 51.91 | 1681.8055 | 14 | 0.6 | 841.9106 | 2 | 47.45 | 1 | 33361 | BuCaMH04.raw | 2.2026E6 | 1 | 1 | 17 | 30 | Carbamidomethylation | C8:Carbamidomethylation:1000.00;C11:Carbamidomethylation:1000.00 | PEAKS DB |
| total 1 peptides |  |  |  |  |  |  |  |  |  |  |  |  |  |  |  |  |  |  |  |
| --- | --- | --- | --- | --- | --- | --- | --- | --- | --- | --- | --- | --- | --- | --- | --- | --- | --- | --- | --- |

Q8AY49.1

back to list

  

| Protein Coverage
| Supporting Peptides
|

Protein Coverage:

Supporting Peptides:

| Peptide | Uniq | -10lgP | Mass | Length | ppm | m/z | z | RT | Fraction | Scan | Source File | Area BuCaMH04\_B15 | #Feature | #Feature BuCaMH04\_B15 | Start | End | PTM | AScore | Found By |
| --- | --- | --- | --- | --- | --- | --- | --- | --- | --- | --- | --- | --- | --- | --- | --- | --- | --- | --- | --- |
| L.DLGYTLTC(+57.02)LIC(+57.02)PEK.D | Y | 51.91 | 1681.8055 | 14 | 0.6 | 841.9106 | 2 | 47.45 | 1 | 33361 | BuCaMH04.raw | 2.2026E6 | 1 | 1 | 17 | 30 | Carbamidomethylation | C8:Carbamidomethylation:1000.00;C11:Carbamidomethylation:1000.00 | PEAKS DB |
| total 1 peptides |  |  |  |  |  |  |  |  |  |  |  |  |  |  |  |  |  |  |  |
| --- | --- | --- | --- | --- | --- | --- | --- | --- | --- | --- | --- | --- | --- | --- | --- | --- | --- | --- | --- |

Q8AY51.1

back to list

  

| Protein Coverage
| Supporting Peptides
|

Protein Coverage:

Supporting Peptides:

| Peptide | Uniq | -10lgP | Mass | Length | ppm | m/z | z | RT | Fraction | Scan | Source File | Area BuCaMH04\_B15 | #Feature | #Feature BuCaMH04\_B15 | Start | End | PTM | AScore | Found By |
| --- | --- | --- | --- | --- | --- | --- | --- | --- | --- | --- | --- | --- | --- | --- | --- | --- | --- | --- | --- |
| L.DLGYTLTC(+57.02)LIC(+57.02)PEK.D | Y | 51.91 | 1681.8055 | 14 | 0.6 | 841.9106 | 2 | 47.45 | 1 | 33361 | BuCaMH04.raw | 2.2026E6 | 1 | 1 | 17 | 30 | Carbamidomethylation | C8:Carbamidomethylation:1000.00;C11:Carbamidomethylation:1000.00 | PEAKS DB |
| total 1 peptides |  |  |  |  |  |  |  |  |  |  |  |  |  |  |  |  |  |  |  |
| --- | --- | --- | --- | --- | --- | --- | --- | --- | --- | --- | --- | --- | --- | --- | --- | --- | --- | --- | --- |

AAL30059.1

back to list

  

| Protein Coverage
| Supporting Peptides
|

Protein Coverage:

Supporting Peptides:

| Peptide | Uniq | -10lgP | Mass | Length | ppm | m/z | z | RT | Fraction | Scan | Source File | Area BuCaMH04\_B15 | #Feature | #Feature BuCaMH04\_B15 | Start | End | PTM | AScore | Found By |
| --- | --- | --- | --- | --- | --- | --- | --- | --- | --- | --- | --- | --- | --- | --- | --- | --- | --- | --- | --- |
| L.DLGYTLTC(+57.02)LIC(+57.02)PEK.D | Y | 51.91 | 1681.8055 | 14 | 0.6 | 841.9106 | 2 | 47.45 | 1 | 33361 | BuCaMH04.raw | 2.2026E6 | 1 | 1 | 17 | 30 | Carbamidomethylation | C8:Carbamidomethylation:1000.00;C11:Carbamidomethylation:1000.00 | PEAKS DB |
| total 1 peptides |  |  |  |  |  |  |  |  |  |  |  |  |  |  |  |  |  |  |  |
| --- | --- | --- | --- | --- | --- | --- | --- | --- | --- | --- | --- | --- | --- | --- | --- | --- | --- | --- | --- |

A2CKF7.1

back to list

  

| Protein Coverage
| Supporting Peptides
|

Protein Coverage:

Supporting Peptides:

| Peptide | Uniq | -10lgP | Mass | Length | ppm | m/z | z | RT | Fraction | Scan | Source File | Area BuCaMH04\_B15 | #Feature | #Feature BuCaMH04\_B15 | Start | End | PTM | AScore | Found By |
| --- | --- | --- | --- | --- | --- | --- | --- | --- | --- | --- | --- | --- | --- | --- | --- | --- | --- | --- | --- |
| L.DLGYTLTC(+57.02)LIC(+57.02)PEK.Y | Y | 51.91 | 1681.8055 | 14 | 0.6 | 841.9106 | 2 | 47.45 | 1 | 33361 | BuCaMH04.raw | 2.2026E6 | 1 | 1 | 17 | 30 | Carbamidomethylation | C8:Carbamidomethylation:1000.00;C11:Carbamidomethylation:1000.00 | PEAKS DB |
| total 1 peptides |  |  |  |  |  |  |  |  |  |  |  |  |  |  |  |  |  |  |  |
| --- | --- | --- | --- | --- | --- | --- | --- | --- | --- | --- | --- | --- | --- | --- | --- | --- | --- | --- | --- |

CAA11133.1

back to list

  

| Protein Coverage
| Supporting Peptides
|

Protein Coverage:

Supporting Peptides:

| Peptide | Uniq | -10lgP | Mass | Length | ppm | m/z | z | RT | Fraction | Scan | Source File | Area BuCaMH04\_B15 | #Feature | #Feature BuCaMH04\_B15 | Start | End | PTM | AScore | Found By |
| --- | --- | --- | --- | --- | --- | --- | --- | --- | --- | --- | --- | --- | --- | --- | --- | --- | --- | --- | --- |
| L.DLGYTLTC(+57.02)LIC(+57.02)PEK.Y | Y | 51.91 | 1681.8055 | 14 | 0.6 | 841.9106 | 2 | 47.45 | 1 | 33361 | BuCaMH04.raw | 2.2026E6 | 1 | 1 | 17 | 30 | Carbamidomethylation | C8:Carbamidomethylation:1000.00;C11:Carbamidomethylation:1000.00 | PEAKS DB |
| total 1 peptides |  |  |  |  |  |  |  |  |  |  |  |  |  |  |  |  |  |  |  |
| --- | --- | --- | --- | --- | --- | --- | --- | --- | --- | --- | --- | --- | --- | --- | --- | --- | --- | --- | --- |

ADF50021.1

back to list

  

| Protein Coverage
| Supporting Peptides
|

Protein Coverage:

Supporting Peptides:

| Peptide | Uniq | -10lgP | Mass | Length | ppm | m/z | z | RT | Fraction | Scan | Source File | Area BuCaMH04\_B15 | #Feature | #Feature BuCaMH04\_B15 | Start | End | PTM | AScore | Found By |
| --- | --- | --- | --- | --- | --- | --- | --- | --- | --- | --- | --- | --- | --- | --- | --- | --- | --- | --- | --- |
| L.DLGYTLTC(+57.02)LIC(+57.02)PEK.Y | Y | 51.91 | 1681.8055 | 14 | 0.6 | 841.9106 | 2 | 47.45 | 1 | 33361 | BuCaMH04.raw | 2.2026E6 | 1 | 1 | 17 | 30 | Carbamidomethylation | C8:Carbamidomethylation:1000.00;C11:Carbamidomethylation:1000.00 | PEAKS DB |
| total 1 peptides |  |  |  |  |  |  |  |  |  |  |  |  |  |  |  |  |  |  |  |
| --- | --- | --- | --- | --- | --- | --- | --- | --- | --- | --- | --- | --- | --- | --- | --- | --- | --- | --- | --- |

ABI33871.1

back to list

  

| Protein Coverage
| Supporting Peptides
|

Protein Coverage:

Supporting Peptides:

| Peptide | Uniq | -10lgP | Mass | Length | ppm | m/z | z | RT | Fraction | Scan | Source File | Area BuCaMH04\_B15 | #Feature | #Feature BuCaMH04\_B15 | Start | End | PTM | AScore | Found By |
| --- | --- | --- | --- | --- | --- | --- | --- | --- | --- | --- | --- | --- | --- | --- | --- | --- | --- | --- | --- |
| L.DLGYTLTC(+57.02)LIC(+57.02)PEK.Y | Y | 51.91 | 1681.8055 | 14 | 0.6 | 841.9106 | 2 | 47.45 | 1 | 33361 | BuCaMH04.raw | 2.2026E6 | 1 | 1 | 17 | 30 | Carbamidomethylation | C8:Carbamidomethylation:1000.00;C11:Carbamidomethylation:1000.00 | PEAKS DB |
| total 1 peptides |  |  |  |  |  |  |  |  |  |  |  |  |  |  |  |  |  |  |  |
| --- | --- | --- | --- | --- | --- | --- | --- | --- | --- | --- | --- | --- | --- | --- | --- | --- | --- | --- | --- |

AAT38875.1

back to list

  

| Protein Coverage
| Supporting Peptides
|

Protein Coverage:

Supporting Peptides:

| Peptide | Uniq | -10lgP | Mass | Length | ppm | m/z | z | RT | Fraction | Scan | Source File | Area BuCaMH04\_B15 | #Feature | #Feature BuCaMH04\_B15 | Start | End | PTM | AScore | Found By |
| --- | --- | --- | --- | --- | --- | --- | --- | --- | --- | --- | --- | --- | --- | --- | --- | --- | --- | --- | --- |
| L.DLGYTLTC(+57.02)LIC(+57.02)PEK.Y | Y | 51.91 | 1681.8055 | 14 | 0.6 | 841.9106 | 2 | 47.45 | 1 | 33361 | BuCaMH04.raw | 2.2026E6 | 1 | 1 | 17 | 30 | Carbamidomethylation | C8:Carbamidomethylation:1000.00;C11:Carbamidomethylation:1000.00 | PEAKS DB |
| total 1 peptides |  |  |  |  |  |  |  |  |  |  |  |  |  |  |  |  |  |  |  |
| --- | --- | --- | --- | --- | --- | --- | --- | --- | --- | --- | --- | --- | --- | --- | --- | --- | --- | --- | --- |

AAL30060.1

back to list

  

| Protein Coverage
| Supporting Peptides
|

Protein Coverage:

Supporting Peptides:

| Peptide | Uniq | -10lgP | Mass | Length | ppm | m/z | z | RT | Fraction | Scan | Source File | Area BuCaMH04\_B15 | #Feature | #Feature BuCaMH04\_B15 | Start | End | PTM | AScore | Found By |
| --- | --- | --- | --- | --- | --- | --- | --- | --- | --- | --- | --- | --- | --- | --- | --- | --- | --- | --- | --- |
| L.DLGYTLTC(+57.02)LIC(+57.02)PEK.D | Y | 51.91 | 1681.8055 | 14 | 0.6 | 841.9106 | 2 | 47.45 | 1 | 33361 | BuCaMH04.raw | 2.2026E6 | 1 | 1 | 17 | 30 | Carbamidomethylation | C8:Carbamidomethylation:1000.00;C11:Carbamidomethylation:1000.00 | PEAKS DB |
| total 1 peptides |  |  |  |  |  |  |  |  |  |  |  |  |  |  |  |  |  |  |  |
| --- | --- | --- | --- | --- | --- | --- | --- | --- | --- | --- | --- | --- | --- | --- | --- | --- | --- | --- | --- |

Q8AY50.1

back to list

  

| Protein Coverage
| Supporting Peptides
|

Protein Coverage:

Supporting Peptides:

| Peptide | Uniq | -10lgP | Mass | Length | ppm | m/z | z | RT | Fraction | Scan | Source File | Area BuCaMH04\_B15 | #Feature | #Feature BuCaMH04\_B15 | Start | End | PTM | AScore | Found By |
| --- | --- | --- | --- | --- | --- | --- | --- | --- | --- | --- | --- | --- | --- | --- | --- | --- | --- | --- | --- |
| L.DLGYTLTC(+57.02)LIC(+57.02)PEK.D | Y | 51.91 | 1681.8055 | 14 | 0.6 | 841.9106 | 2 | 47.45 | 1 | 33361 | BuCaMH04.raw | 2.2026E6 | 1 | 1 | 17 | 30 | Carbamidomethylation | C8:Carbamidomethylation:1000.00;C11:Carbamidomethylation:1000.00 | PEAKS DB |
| total 1 peptides |  |  |  |  |  |  |  |  |  |  |  |  |  |  |  |  |  |  |  |
| --- | --- | --- | --- | --- | --- | --- | --- | --- | --- | --- | --- | --- | --- | --- | --- | --- | --- | --- | --- |

Q9YGI2.1

back to list

  

| Protein Coverage
| Supporting Peptides
|

Protein Coverage:

Supporting Peptides:

| Peptide | Uniq | -10lgP | Mass | Length | ppm | m/z | z | RT | Fraction | Scan | Source File | Area BuCaMH04\_B15 | #Feature | #Feature BuCaMH04\_B15 | Start | End | PTM | AScore | Found By |
| --- | --- | --- | --- | --- | --- | --- | --- | --- | --- | --- | --- | --- | --- | --- | --- | --- | --- | --- | --- |
| L.DLGYTLTC(+57.02)LIC(+57.02)PEK.Y | Y | 51.91 | 1681.8055 | 14 | 0.6 | 841.9106 | 2 | 47.45 | 1 | 33361 | BuCaMH04.raw | 2.2026E6 | 1 | 1 | 17 | 30 | Carbamidomethylation | C8:Carbamidomethylation:1000.00;C11:Carbamidomethylation:1000.00 | PEAKS DB |
| total 1 peptides |  |  |  |  |  |  |  |  |  |  |  |  |  |  |  |  |  |  |  |
| --- | --- | --- | --- | --- | --- | --- | --- | --- | --- | --- | --- | --- | --- | --- | --- | --- | --- | --- | --- |

ADF50022.1

back to list

  

| Protein Coverage
| Supporting Peptides
|

Protein Coverage:

Supporting Peptides:

| Peptide | Uniq | -10lgP | Mass | Length | ppm | m/z | z | RT | Fraction | Scan | Source File | Area BuCaMH04\_B15 | #Feature | #Feature BuCaMH04\_B15 | Start | End | PTM | AScore | Found By |
| --- | --- | --- | --- | --- | --- | --- | --- | --- | --- | --- | --- | --- | --- | --- | --- | --- | --- | --- | --- |
| L.DLGYTLTC(+57.02)LIC(+57.02)PEK.Y | Y | 51.91 | 1681.8055 | 14 | 0.6 | 841.9106 | 2 | 47.45 | 1 | 33361 | BuCaMH04.raw | 2.2026E6 | 1 | 1 | 17 | 30 | Carbamidomethylation | C8:Carbamidomethylation:1000.00;C11:Carbamidomethylation:1000.00 | PEAKS DB |
| total 1 peptides |  |  |  |  |  |  |  |  |  |  |  |  |  |  |  |  |  |  |  |
| --- | --- | --- | --- | --- | --- | --- | --- | --- | --- | --- | --- | --- | --- | --- | --- | --- | --- | --- | --- |

ADF50020.1

back to list

  

| Protein Coverage
| Supporting Peptides
|

Protein Coverage:

Supporting Peptides:

| Peptide | Uniq | -10lgP | Mass | Length | ppm | m/z | z | RT | Fraction | Scan | Source File | Area BuCaMH04\_B15 | #Feature | #Feature BuCaMH04\_B15 | Start | End | PTM | AScore | Found By |
| --- | --- | --- | --- | --- | --- | --- | --- | --- | --- | --- | --- | --- | --- | --- | --- | --- | --- | --- | --- |
| L.DLGYTLTC(+57.02)LIC(+57.02)PEK.Y | Y | 51.91 | 1681.8055 | 14 | 0.6 | 841.9106 | 2 | 47.45 | 1 | 33361 | BuCaMH04.raw | 2.2026E6 | 1 | 1 | 17 | 30 | Carbamidomethylation | C8:Carbamidomethylation:1000.00;C11:Carbamidomethylation:1000.00 | PEAKS DB |
| total 1 peptides |  |  |  |  |  |  |  |  |  |  |  |  |  |  |  |  |  |  |  |
| --- | --- | --- | --- | --- | --- | --- | --- | --- | --- | --- | --- | --- | --- | --- | --- | --- | --- | --- | --- |

ADN67577.1

back to list

  

| Protein Coverage
| Supporting Peptides
|

Protein Coverage:

Supporting Peptides:

| Peptide | Uniq | -10lgP | Mass | Length | ppm | m/z | z | RT | Fraction | Scan | Source File | Area BuCaMH04\_B15 | #Feature | #Feature BuCaMH04\_B15 | Start | End | PTM | AScore | Found By |
| --- | --- | --- | --- | --- | --- | --- | --- | --- | --- | --- | --- | --- | --- | --- | --- | --- | --- | --- | --- |
| L.DLGYTLTC(+57.02)LIC(+57.02)PEK.Y | Y | 51.91 | 1681.8055 | 14 | 0.6 | 841.9106 | 2 | 47.45 | 1 | 33361 | BuCaMH04.raw | 2.2026E6 | 1 | 1 | 17 | 30 | Carbamidomethylation | C8:Carbamidomethylation:1000.00;C11:Carbamidomethylation:1000.00 | PEAKS DB |
| total 1 peptides |  |  |  |  |  |  |  |  |  |  |  |  |  |  |  |  |  |  |  |
| --- | --- | --- | --- | --- | --- | --- | --- | --- | --- | --- | --- | --- | --- | --- | --- | --- | --- | --- | --- |

CAA11134.1

back to list

  

| Protein Coverage
| Supporting Peptides
|

Protein Coverage:

Supporting Peptides:

| Peptide | Uniq | -10lgP | Mass | Length | ppm | m/z | z | RT | Fraction | Scan | Source File | Area BuCaMH04\_B15 | #Feature | #Feature BuCaMH04\_B15 | Start | End | PTM | AScore | Found By |
| --- | --- | --- | --- | --- | --- | --- | --- | --- | --- | --- | --- | --- | --- | --- | --- | --- | --- | --- | --- |
| L.DLGYTLTC(+57.02)LIC(+57.02)PEK.Y | Y | 51.91 | 1681.8055 | 14 | 0.6 | 841.9106 | 2 | 47.45 | 1 | 33361 | BuCaMH04.raw | 2.2026E6 | 1 | 1 | 17 | 30 | Carbamidomethylation | C8:Carbamidomethylation:1000.00;C11:Carbamidomethylation:1000.00 | PEAKS DB |
| total 1 peptides |  |  |  |  |  |  |  |  |  |  |  |  |  |  |  |  |  |  |  |
| --- | --- | --- | --- | --- | --- | --- | --- | --- | --- | --- | --- | --- | --- | --- | --- | --- | --- | --- | --- |

Q6IZ95.1

back to list

  

| Protein Coverage
| Supporting Peptides
|

Protein Coverage:

Supporting Peptides:

| Peptide | Uniq | -10lgP | Mass | Length | ppm | m/z | z | RT | Fraction | Scan | Source File | Area BuCaMH04\_B15 | #Feature | #Feature BuCaMH04\_B15 | Start | End | PTM | AScore | Found By |
| --- | --- | --- | --- | --- | --- | --- | --- | --- | --- | --- | --- | --- | --- | --- | --- | --- | --- | --- | --- |
| L.DLGYTLTC(+57.02)LIC(+57.02)PEK.Y | Y | 51.91 | 1681.8055 | 14 | 0.6 | 841.9106 | 2 | 47.45 | 1 | 33361 | BuCaMH04.raw | 2.2026E6 | 1 | 1 | 17 | 30 | Carbamidomethylation | C8:Carbamidomethylation:1000.00;C11:Carbamidomethylation:1000.00 | PEAKS DB |
| total 1 peptides |  |  |  |  |  |  |  |  |  |  |  |  |  |  |  |  |  |  |  |
| --- | --- | --- | --- | --- | --- | --- | --- | --- | --- | --- | --- | --- | --- | --- | --- | --- | --- | --- | --- |

Q9YGI1.1

back to list

  

| Protein Coverage
| Supporting Peptides
|

Protein Coverage:

Supporting Peptides:

| Peptide | Uniq | -10lgP | Mass | Length | ppm | m/z | z | RT | Fraction | Scan | Source File | Area BuCaMH04\_B15 | #Feature | #Feature BuCaMH04\_B15 | Start | End | PTM | AScore | Found By |
| --- | --- | --- | --- | --- | --- | --- | --- | --- | --- | --- | --- | --- | --- | --- | --- | --- | --- | --- | --- |
| L.DLGYTLTC(+57.02)LIC(+57.02)PEK.Y | Y | 51.91 | 1681.8055 | 14 | 0.6 | 841.9106 | 2 | 47.45 | 1 | 33361 | BuCaMH04.raw | 2.2026E6 | 1 | 1 | 17 | 30 | Carbamidomethylation | C8:Carbamidomethylation:1000.00;C11:Carbamidomethylation:1000.00 | PEAKS DB |
| total 1 peptides |  |  |  |  |  |  |  |  |  |  |  |  |  |  |  |  |  |  |  |
| --- | --- | --- | --- | --- | --- | --- | --- | --- | --- | --- | --- | --- | --- | --- | --- | --- | --- | --- | --- |

XP\_007425787.1

back to list

  

| Protein Coverage
| Supporting Peptides
|

Protein Coverage:

Supporting Peptides:

| Peptide | Uniq | -10lgP | Mass | Length | ppm | m/z | z | RT | Fraction | Scan | Source File | Area BuCaMH04\_B15 | #Feature | #Feature BuCaMH04\_B15 | Start | End | PTM | AScore | Found By |
| --- | --- | --- | --- | --- | --- | --- | --- | --- | --- | --- | --- | --- | --- | --- | --- | --- | --- | --- | --- |
| R.C(+57.02)DSGFYYSEEVC(+57.02)R.C | Y | 50.53 | 1670.6340 | 13 | 0.8 | 836.3250 | 2 | 33.63 | 1 | 21186 | BuCaMH04.raw | 3.2389E5 | 1 | 1 | 350 | 362 | Carbamidomethylation | C1:Carbamidomethylation:1000.00;C12:Carbamidomethylation:1000.00 | PEAKS DB |
| total 1 peptides |  |  |  |  |  |  |  |  |  |  |  |  |  |  |  |  |  |  |  |
| --- | --- | --- | --- | --- | --- | --- | --- | --- | --- | --- | --- | --- | --- | --- | --- | --- | --- | --- | --- |

XP\_034298589.1

back to list

  

| Protein Coverage
| Supporting Peptides
|

Protein Coverage:

Supporting Peptides:

| Peptide | Uniq | -10lgP | Mass | Length | ppm | m/z | z | RT | Fraction | Scan | Source File | Area BuCaMH04\_B15 | #Feature | #Feature BuCaMH04\_B15 | Start | End | PTM | AScore | Found By |
| --- | --- | --- | --- | --- | --- | --- | --- | --- | --- | --- | --- | --- | --- | --- | --- | --- | --- | --- | --- |
| R.C(+57.02)DSGFYYSEEVC(+57.02)R.C | Y | 50.53 | 1670.6340 | 13 | 0.8 | 836.3250 | 2 | 33.63 | 1 | 21186 | BuCaMH04.raw | 3.2389E5 | 1 | 1 | 353 | 365 | Carbamidomethylation | C1:Carbamidomethylation:1000.00;C12:Carbamidomethylation:1000.00 | PEAKS DB |
| total 1 peptides |  |  |  |  |  |  |  |  |  |  |  |  |  |  |  |  |  |  |  |
| --- | --- | --- | --- | --- | --- | --- | --- | --- | --- | --- | --- | --- | --- | --- | --- | --- | --- | --- | --- |

JAC96561.1

back to list

  

| Protein Coverage
| Supporting Peptides
|

Protein Coverage:

Supporting Peptides:

| Peptide | Uniq | -10lgP | Mass | Length | ppm | m/z | z | RT | Fraction | Scan | Source File | Area BuCaMH04\_B15 | #Feature | #Feature BuCaMH04\_B15 | Start | End | PTM | AScore | Found By |
| --- | --- | --- | --- | --- | --- | --- | --- | --- | --- | --- | --- | --- | --- | --- | --- | --- | --- | --- | --- |
| R.C(+57.02)DSGFYYSEEVC(+57.02)R.C | Y | 50.53 | 1670.6340 | 13 | 0.8 | 836.3250 | 2 | 33.63 | 1 | 21186 | BuCaMH04.raw | 3.2389E5 | 1 | 1 | 397 | 409 | Carbamidomethylation | C1:Carbamidomethylation:1000.00;C12:Carbamidomethylation:1000.00 | PEAKS DB |
| total 1 peptides |  |  |  |  |  |  |  |  |  |  |  |  |  |  |  |  |  |  |  |
| --- | --- | --- | --- | --- | --- | --- | --- | --- | --- | --- | --- | --- | --- | --- | --- | --- | --- | --- | --- |

JAI10614.1

back to list

  

| Protein Coverage
| Supporting Peptides
|

Protein Coverage:

Supporting Peptides:

| Peptide | Uniq | -10lgP | Mass | Length | ppm | m/z | z | RT | Fraction | Scan | Source File | Area BuCaMH04\_B15 | #Feature | #Feature BuCaMH04\_B15 | Start | End | PTM | AScore | Found By |
| --- | --- | --- | --- | --- | --- | --- | --- | --- | --- | --- | --- | --- | --- | --- | --- | --- | --- | --- | --- |
| R.C(+57.02)DSGFYYSEEVC(+57.02)R.C | Y | 50.53 | 1670.6340 | 13 | 0.8 | 836.3250 | 2 | 33.63 | 1 | 21186 | BuCaMH04.raw | 3.2389E5 | 1 | 1 | 397 | 409 | Carbamidomethylation | C1:Carbamidomethylation:1000.00;C12:Carbamidomethylation:1000.00 | PEAKS DB |
| total 1 peptides |  |  |  |  |  |  |  |  |  |  |  |  |  |  |  |  |  |  |  |
| --- | --- | --- | --- | --- | --- | --- | --- | --- | --- | --- | --- | --- | --- | --- | --- | --- | --- | --- | --- |

SMD28268.1

back to list

  

| Protein Coverage
| Supporting Peptides
|

Protein Coverage:

Supporting Peptides:

| Peptide | Uniq | -10lgP | Mass | Length | ppm | m/z | z | RT | Fraction | Scan | Source File | Area BuCaMH04\_B15 | #Feature | #Feature BuCaMH04\_B15 | Start | End | PTM | AScore | Found By |
| --- | --- | --- | --- | --- | --- | --- | --- | --- | --- | --- | --- | --- | --- | --- | --- | --- | --- | --- | --- |
| R.C(+57.02)DSGFYYSEEVC(+57.02)R.C | Y | 50.53 | 1670.6340 | 13 | 0.8 | 836.3250 | 2 | 33.63 | 1 | 21186 | BuCaMH04.raw | 3.2389E5 | 1 | 1 | 397 | 409 | Carbamidomethylation | C1:Carbamidomethylation:1000.00;C12:Carbamidomethylation:1000.00 | PEAKS DB |
| total 1 peptides |  |  |  |  |  |  |  |  |  |  |  |  |  |  |  |  |  |  |  |
| --- | --- | --- | --- | --- | --- | --- | --- | --- | --- | --- | --- | --- | --- | --- | --- | --- | --- | --- | --- |

JAC95037.1

back to list

  

| Protein Coverage
| Supporting Peptides
|

Protein Coverage:

Supporting Peptides:

| Peptide | Uniq | -10lgP | Mass | Length | ppm | m/z | z | RT | Fraction | Scan | Source File | Area BuCaMH04\_B15 | #Feature | #Feature BuCaMH04\_B15 | Start | End | PTM | AScore | Found By |
| --- | --- | --- | --- | --- | --- | --- | --- | --- | --- | --- | --- | --- | --- | --- | --- | --- | --- | --- | --- |
| R.C(+57.02)DSGFYYSEEVC(+57.02)R.C | Y | 50.53 | 1670.6340 | 13 | 0.8 | 836.3250 | 2 | 33.63 | 1 | 21186 | BuCaMH04.raw | 3.2389E5 | 1 | 1 | 397 | 409 | Carbamidomethylation | C1:Carbamidomethylation:1000.00;C12:Carbamidomethylation:1000.00 | PEAKS DB |
| total 1 peptides |  |  |  |  |  |  |  |  |  |  |  |  |  |  |  |  |  |  |  |
| --- | --- | --- | --- | --- | --- | --- | --- | --- | --- | --- | --- | --- | --- | --- | --- | --- | --- | --- | --- |

JAV48556.1

back to list

  

| Protein Coverage
| Supporting Peptides
|

Protein Coverage:

Supporting Peptides:

| Peptide | Uniq | -10lgP | Mass | Length | ppm | m/z | z | RT | Fraction | Scan | Source File | Area BuCaMH04\_B15 | #Feature | #Feature BuCaMH04\_B15 | Start | End | PTM | AScore | Found By |
| --- | --- | --- | --- | --- | --- | --- | --- | --- | --- | --- | --- | --- | --- | --- | --- | --- | --- | --- | --- |
| R.C(+57.02)DSGFYYSEEVC(+57.02)R.C | Y | 50.53 | 1670.6340 | 13 | 0.8 | 836.3250 | 2 | 33.63 | 1 | 21186 | BuCaMH04.raw | 3.2389E5 | 1 | 1 | 397 | 409 | Carbamidomethylation | C1:Carbamidomethylation:1000.00;C12:Carbamidomethylation:1000.00 | PEAKS DB |
| total 1 peptides |  |  |  |  |  |  |  |  |  |  |  |  |  |  |  |  |  |  |  |
| --- | --- | --- | --- | --- | --- | --- | --- | --- | --- | --- | --- | --- | --- | --- | --- | --- | --- | --- | --- |

JAA95033.1

back to list

  

| Protein Coverage
| Supporting Peptides
|

Protein Coverage:

Supporting Peptides:

| Peptide | Uniq | -10lgP | Mass | Length | ppm | m/z | z | RT | Fraction | Scan | Source File | Area BuCaMH04\_B15 | #Feature | #Feature BuCaMH04\_B15 | Start | End | PTM | AScore | Found By |
| --- | --- | --- | --- | --- | --- | --- | --- | --- | --- | --- | --- | --- | --- | --- | --- | --- | --- | --- | --- |
| R.C(+57.02)DSGFYYSEEVC(+57.02)R.C | Y | 50.53 | 1670.6340 | 13 | 0.8 | 836.3250 | 2 | 33.63 | 1 | 21186 | BuCaMH04.raw | 3.2389E5 | 1 | 1 | 397 | 409 | Carbamidomethylation | C1:Carbamidomethylation:1000.00;C12:Carbamidomethylation:1000.00 | PEAKS DB |
| total 1 peptides |  |  |  |  |  |  |  |  |  |  |  |  |  |  |  |  |  |  |  |
| --- | --- | --- | --- | --- | --- | --- | --- | --- | --- | --- | --- | --- | --- | --- | --- | --- | --- | --- | --- |

XP\_034298588.1

back to list

  

| Protein Coverage
| Supporting Peptides
|

Protein Coverage:

Supporting Peptides:

| Peptide | Uniq | -10lgP | Mass | Length | ppm | m/z | z | RT | Fraction | Scan | Source File | Area BuCaMH04\_B15 | #Feature | #Feature BuCaMH04\_B15 | Start | End | PTM | AScore | Found By |
| --- | --- | --- | --- | --- | --- | --- | --- | --- | --- | --- | --- | --- | --- | --- | --- | --- | --- | --- | --- |
| R.C(+57.02)DSGFYYSEEVC(+57.02)R.C | Y | 50.53 | 1670.6340 | 13 | 0.8 | 836.3250 | 2 | 33.63 | 1 | 21186 | BuCaMH04.raw | 3.2389E5 | 1 | 1 | 397 | 409 | Carbamidomethylation | C1:Carbamidomethylation:1000.00;C12:Carbamidomethylation:1000.00 | PEAKS DB |
| total 1 peptides |  |  |  |  |  |  |  |  |  |  |  |  |  |  |  |  |  |  |  |
| --- | --- | --- | --- | --- | --- | --- | --- | --- | --- | --- | --- | --- | --- | --- | --- | --- | --- | --- | --- |

XP\_013912093.1

back to list

  

| Protein Coverage
| Supporting Peptides
|

Protein Coverage:

Supporting Peptides:

| Peptide | Uniq | -10lgP | Mass | Length | ppm | m/z | z | RT | Fraction | Scan | Source File | Area BuCaMH04\_B15 | #Feature | #Feature BuCaMH04\_B15 | Start | End | PTM | AScore | Found By |
| --- | --- | --- | --- | --- | --- | --- | --- | --- | --- | --- | --- | --- | --- | --- | --- | --- | --- | --- | --- |
| R.C(+57.02)DSGFYYSEEVC(+57.02)R.C | Y | 50.53 | 1670.6340 | 13 | 0.8 | 836.3250 | 2 | 33.63 | 1 | 21186 | BuCaMH04.raw | 3.2389E5 | 1 | 1 | 397 | 409 | Carbamidomethylation | C1:Carbamidomethylation:1000.00;C12:Carbamidomethylation:1000.00 | PEAKS DB |
| total 1 peptides |  |  |  |  |  |  |  |  |  |  |  |  |  |  |  |  |  |  |  |
| --- | --- | --- | --- | --- | --- | --- | --- | --- | --- | --- | --- | --- | --- | --- | --- | --- | --- | --- | --- |

ASU45066.1

back to list

  

| Protein Coverage
| Supporting Peptides
|

Protein Coverage:

Supporting Peptides:

| Peptide | Uniq | -10lgP | Mass | Length | ppm | m/z | z | RT | Fraction | Scan | Source File | Area BuCaMH04\_B15 | #Feature | #Feature BuCaMH04\_B15 | Start | End | PTM | AScore | Found By |
| --- | --- | --- | --- | --- | --- | --- | --- | --- | --- | --- | --- | --- | --- | --- | --- | --- | --- | --- | --- |
| R.C(+57.02)DSGFYYSEEVC(+57.02)R.C | Y | 50.53 | 1670.6340 | 13 | 0.8 | 836.3250 | 2 | 33.63 | 1 | 21186 | BuCaMH04.raw | 3.2389E5 | 1 | 1 | 397 | 409 | Carbamidomethylation | C1:Carbamidomethylation:1000.00;C12:Carbamidomethylation:1000.00 | PEAKS DB |
| total 1 peptides |  |  |  |  |  |  |  |  |  |  |  |  |  |  |  |  |  |  |  |
| --- | --- | --- | --- | --- | --- | --- | --- | --- | --- | --- | --- | --- | --- | --- | --- | --- | --- | --- | --- |

JAC94939.1

back to list

  

| Protein Coverage
| Supporting Peptides
|

Protein Coverage:

Supporting Peptides:

| Peptide | Uniq | -10lgP | Mass | Length | ppm | m/z | z | RT | Fraction | Scan | Source File | Area BuCaMH04\_B15 | #Feature | #Feature BuCaMH04\_B15 | Start | End | PTM | AScore | Found By |
| --- | --- | --- | --- | --- | --- | --- | --- | --- | --- | --- | --- | --- | --- | --- | --- | --- | --- | --- | --- |
| R.C(+57.02)DSGFYYSEEVC(+57.02)R.C | Y | 50.53 | 1670.6340 | 13 | 0.8 | 836.3250 | 2 | 33.63 | 1 | 21186 | BuCaMH04.raw | 3.2389E5 | 1 | 1 | 398 | 410 | Carbamidomethylation | C1:Carbamidomethylation:1000.00;C12:Carbamidomethylation:1000.00 | PEAKS DB |
| total 1 peptides |  |  |  |  |  |  |  |  |  |  |  |  |  |  |  |  |  |  |  |
| --- | --- | --- | --- | --- | --- | --- | --- | --- | --- | --- | --- | --- | --- | --- | --- | --- | --- | --- | --- |

XP\_029139565.1

back to list

  

| Protein Coverage
| Supporting Peptides
|

Protein Coverage:

Supporting Peptides:

| Peptide | Uniq | -10lgP | Mass | Length | ppm | m/z | z | RT | Fraction | Scan | Source File | Area BuCaMH04\_B15 | #Feature | #Feature BuCaMH04\_B15 | Start | End | PTM | AScore | Found By |
| --- | --- | --- | --- | --- | --- | --- | --- | --- | --- | --- | --- | --- | --- | --- | --- | --- | --- | --- | --- |
| R.C(+57.02)DSGFYYSEEVC(+57.02)R.C | Y | 50.53 | 1670.6340 | 13 | 0.8 | 836.3250 | 2 | 33.63 | 1 | 21186 | BuCaMH04.raw | 3.2389E5 | 1 | 1 | 398 | 410 | Carbamidomethylation | C1:Carbamidomethylation:1000.00;C12:Carbamidomethylation:1000.00 | PEAKS DB |
| total 1 peptides |  |  |  |  |  |  |  |  |  |  |  |  |  |  |  |  |  |  |  |
| --- | --- | --- | --- | --- | --- | --- | --- | --- | --- | --- | --- | --- | --- | --- | --- | --- | --- | --- | --- |

JAC94973.1

back to list

  

| Protein Coverage
| Supporting Peptides
|

Protein Coverage:

Supporting Peptides:

| Peptide | Uniq | -10lgP | Mass | Length | ppm | m/z | z | RT | Fraction | Scan | Source File | Area BuCaMH04\_B15 | #Feature | #Feature BuCaMH04\_B15 | Start | End | PTM | AScore | Found By |
| --- | --- | --- | --- | --- | --- | --- | --- | --- | --- | --- | --- | --- | --- | --- | --- | --- | --- | --- | --- |
| R.C(+57.02)DSGFYYSEEVC(+57.02)R.C | Y | 50.53 | 1670.6340 | 13 | 0.8 | 836.3250 | 2 | 33.63 | 1 | 21186 | BuCaMH04.raw | 3.2389E5 | 1 | 1 | 328 | 340 | Carbamidomethylation | C1:Carbamidomethylation:1000.00;C12:Carbamidomethylation:1000.00 | PEAKS DB |
| total 1 peptides |  |  |  |  |  |  |  |  |  |  |  |  |  |  |  |  |  |  |  |
| --- | --- | --- | --- | --- | --- | --- | --- | --- | --- | --- | --- | --- | --- | --- | --- | --- | --- | --- | --- |

XP\_034298590.1

back to list

  

| Protein Coverage
| Supporting Peptides
|

Protein Coverage:

Supporting Peptides:

| Peptide | Uniq | -10lgP | Mass | Length | ppm | m/z | z | RT | Fraction | Scan | Source File | Area BuCaMH04\_B15 | #Feature | #Feature BuCaMH04\_B15 | Start | End | PTM | AScore | Found By |
| --- | --- | --- | --- | --- | --- | --- | --- | --- | --- | --- | --- | --- | --- | --- | --- | --- | --- | --- | --- |
| R.C(+57.02)DSGFYYSEEVC(+57.02)R.C | Y | 50.53 | 1670.6340 | 13 | 0.8 | 836.3250 | 2 | 33.63 | 1 | 21186 | BuCaMH04.raw | 3.2389E5 | 1 | 1 | 328 | 340 | Carbamidomethylation | C1:Carbamidomethylation:1000.00;C12:Carbamidomethylation:1000.00 | PEAKS DB |
| total 1 peptides |  |  |  |  |  |  |  |  |  |  |  |  |  |  |  |  |  |  |  |
| --- | --- | --- | --- | --- | --- | --- | --- | --- | --- | --- | --- | --- | --- | --- | --- | --- | --- | --- | --- |

JAI08988.1

back to list

  

| Protein Coverage
| Supporting Peptides
|

Protein Coverage:

Supporting Peptides:

| Peptide | Uniq | -10lgP | Mass | Length | ppm | m/z | z | RT | Fraction | Scan | Source File | Area BuCaMH04\_B15 | #Feature | #Feature BuCaMH04\_B15 | Start | End | PTM | AScore | Found By |
| --- | --- | --- | --- | --- | --- | --- | --- | --- | --- | --- | --- | --- | --- | --- | --- | --- | --- | --- | --- |
| R.C(+57.02)DSGFYYSEEVC(+57.02)R.C | Y | 50.53 | 1670.6340 | 13 | 0.8 | 836.3250 | 2 | 33.63 | 1 | 21186 | BuCaMH04.raw | 3.2389E5 | 1 | 1 | 397 | 409 | Carbamidomethylation | C1:Carbamidomethylation:1000.00;C12:Carbamidomethylation:1000.00 | PEAKS DB |
| total 1 peptides |  |  |  |  |  |  |  |  |  |  |  |  |  |  |  |  |  |  |  |
| --- | --- | --- | --- | --- | --- | --- | --- | --- | --- | --- | --- | --- | --- | --- | --- | --- | --- | --- | --- |

ETE60014.1

back to list

  

| Protein Coverage
| Supporting Peptides
|

Protein Coverage:

Supporting Peptides:

| Peptide | Uniq | -10lgP | Mass | Length | ppm | m/z | z | RT | Fraction | Scan | Source File | Area BuCaMH04\_B15 | #Feature | #Feature BuCaMH04\_B15 | Start | End | PTM | AScore | Found By |
| --- | --- | --- | --- | --- | --- | --- | --- | --- | --- | --- | --- | --- | --- | --- | --- | --- | --- | --- | --- |
| R.C(+57.02)DSGFYYSEEVC(+57.02)R.C | Y | 50.53 | 1670.6340 | 13 | 0.8 | 836.3250 | 2 | 33.63 | 1 | 21186 | BuCaMH04.raw | 3.2389E5 | 1 | 1 | 397 | 409 | Carbamidomethylation | C1:Carbamidomethylation:1000.00;C12:Carbamidomethylation:1000.00 | PEAKS DB |
| total 1 peptides |  |  |  |  |  |  |  |  |  |  |  |  |  |  |  |  |  |  |  |
| --- | --- | --- | --- | --- | --- | --- | --- | --- | --- | --- | --- | --- | --- | --- | --- | --- | --- | --- | --- |

JAB52939.1

back to list

  

| Protein Coverage
| Supporting Peptides
|

Protein Coverage:

Supporting Peptides:

| Peptide | Uniq | -10lgP | Mass | Length | ppm | m/z | z | RT | Fraction | Scan | Source File | Area BuCaMH04\_B15 | #Feature | #Feature BuCaMH04\_B15 | Start | End | PTM | AScore | Found By |
| --- | --- | --- | --- | --- | --- | --- | --- | --- | --- | --- | --- | --- | --- | --- | --- | --- | --- | --- | --- |
| R.C(+57.02)DSGFYYSEEVC(+57.02)R.C | Y | 50.53 | 1670.6340 | 13 | 0.8 | 836.3250 | 2 | 33.63 | 1 | 21186 | BuCaMH04.raw | 3.2389E5 | 1 | 1 | 397 | 409 | Carbamidomethylation | C1:Carbamidomethylation:1000.00;C12:Carbamidomethylation:1000.00 | PEAKS DB |
| total 1 peptides |  |  |  |  |  |  |  |  |  |  |  |  |  |  |  |  |  |  |  |
| --- | --- | --- | --- | --- | --- | --- | --- | --- | --- | --- | --- | --- | --- | --- | --- | --- | --- | --- | --- |

T\_45

back to list

  

| Protein Coverage
| Supporting Peptides
|

Protein Coverage:

Supporting Peptides:

| Peptide | Uniq | -10lgP | Mass | Length | ppm | m/z | z | RT | Fraction | Scan | Source File | Area BuCaMH04\_B15 | #Feature | #Feature BuCaMH04\_B15 | Start | End | PTM | AScore | Found By |
| --- | --- | --- | --- | --- | --- | --- | --- | --- | --- | --- | --- | --- | --- | --- | --- | --- | --- | --- | --- |
| R.C(+57.02)DSGFYYSEEVC(+57.02)R.C | Y | 50.53 | 1670.6340 | 13 | 0.8 | 836.3250 | 2 | 33.63 | 1 | 21186 | BuCaMH04.raw | 3.2389E5 | 1 | 1 | 397 | 409 | Carbamidomethylation | C1:Carbamidomethylation:1000.00;C12:Carbamidomethylation:1000.00 | PEAKS DB |
| total 1 peptides |  |  |  |  |  |  |  |  |  |  |  |  |  |  |  |  |  |  |  |
| --- | --- | --- | --- | --- | --- | --- | --- | --- | --- | --- | --- | --- | --- | --- | --- | --- | --- | --- | --- |

JAS04977.1

back to list

  

| Protein Coverage
| Supporting Peptides
|

Protein Coverage:

Supporting Peptides:

| Peptide | Uniq | -10lgP | Mass | Length | ppm | m/z | z | RT | Fraction | Scan | Source File | Area BuCaMH04\_B15 | #Feature | #Feature BuCaMH04\_B15 | Start | End | PTM | AScore | Found By |
| --- | --- | --- | --- | --- | --- | --- | --- | --- | --- | --- | --- | --- | --- | --- | --- | --- | --- | --- | --- |
| R.C(+57.02)DSGFYYSEEVC(+57.02)R.C | Y | 50.53 | 1670.6340 | 13 | 0.8 | 836.3250 | 2 | 33.63 | 1 | 21186 | BuCaMH04.raw | 3.2389E5 | 1 | 1 | 397 | 409 | Carbamidomethylation | C1:Carbamidomethylation:1000.00;C12:Carbamidomethylation:1000.00 | PEAKS DB |
| total 1 peptides |  |  |  |  |  |  |  |  |  |  |  |  |  |  |  |  |  |  |  |
| --- | --- | --- | --- | --- | --- | --- | --- | --- | --- | --- | --- | --- | --- | --- | --- | --- | --- | --- | --- |

XP\_026522979.1

back to list

  

| Protein Coverage
| Supporting Peptides
|

Protein Coverage:

Supporting Peptides:

| Peptide | Uniq | -10lgP | Mass | Length | ppm | m/z | z | RT | Fraction | Scan | Source File | Area BuCaMH04\_B15 | #Feature | #Feature BuCaMH04\_B15 | Start | End | PTM | AScore | Found By |
| --- | --- | --- | --- | --- | --- | --- | --- | --- | --- | --- | --- | --- | --- | --- | --- | --- | --- | --- | --- |
| R.C(+57.02)DSGFYYSEEVC(+57.02)R.C | Y | 50.53 | 1670.6340 | 13 | 0.8 | 836.3250 | 2 | 33.63 | 1 | 21186 | BuCaMH04.raw | 3.2389E5 | 1 | 1 | 397 | 409 | Carbamidomethylation | C1:Carbamidomethylation:1000.00;C12:Carbamidomethylation:1000.00 | PEAKS DB |
| total 1 peptides |  |  |  |  |  |  |  |  |  |  |  |  |  |  |  |  |  |  |  |
| --- | --- | --- | --- | --- | --- | --- | --- | --- | --- | --- | --- | --- | --- | --- | --- | --- | --- | --- | --- |

XP\_026555459.1

back to list

  

| Protein Coverage
| Supporting Peptides
|

Protein Coverage:

Supporting Peptides:

| Peptide | Uniq | -10lgP | Mass | Length | ppm | m/z | z | RT | Fraction | Scan | Source File | Area BuCaMH04\_B15 | #Feature | #Feature BuCaMH04\_B15 | Start | End | PTM | AScore | Found By |
| --- | --- | --- | --- | --- | --- | --- | --- | --- | --- | --- | --- | --- | --- | --- | --- | --- | --- | --- | --- |
| R.C(+57.02)DSGFYYSEEVC(+57.02)R.C | Y | 50.53 | 1670.6340 | 13 | 0.8 | 836.3250 | 2 | 33.63 | 1 | 21186 | BuCaMH04.raw | 3.2389E5 | 1 | 1 | 397 | 409 | Carbamidomethylation | C1:Carbamidomethylation:1000.00;C12:Carbamidomethylation:1000.00 | PEAKS DB |
| total 1 peptides |  |  |  |  |  |  |  |  |  |  |  |  |  |  |  |  |  |  |  |
| --- | --- | --- | --- | --- | --- | --- | --- | --- | --- | --- | --- | --- | --- | --- | --- | --- | --- | --- | --- |

JAI09156.1

back to list

  

| Protein Coverage
| Supporting Peptides
|

Protein Coverage:

Supporting Peptides:

| Peptide | Uniq | -10lgP | Mass | Length | ppm | m/z | z | RT | Fraction | Scan | Source File | Area BuCaMH04\_B15 | #Feature | #Feature BuCaMH04\_B15 | Start | End | PTM | AScore | Found By |
| --- | --- | --- | --- | --- | --- | --- | --- | --- | --- | --- | --- | --- | --- | --- | --- | --- | --- | --- | --- |
| R.C(+57.02)DSGFYYSEEVC(+57.02)R.C | Y | 50.53 | 1670.6340 | 13 | 0.8 | 836.3250 | 2 | 33.63 | 1 | 21186 | BuCaMH04.raw | 3.2389E5 | 1 | 1 | 397 | 409 | Carbamidomethylation | C1:Carbamidomethylation:1000.00;C12:Carbamidomethylation:1000.00 | PEAKS DB |
| total 1 peptides |  |  |  |  |  |  |  |  |  |  |  |  |  |  |  |  |  |  |  |
| --- | --- | --- | --- | --- | --- | --- | --- | --- | --- | --- | --- | --- | --- | --- | --- | --- | --- | --- | --- |

LAA17506.1

back to list

  

| Protein Coverage
| Supporting Peptides
|

Protein Coverage:

Supporting Peptides:

| Peptide | Uniq | -10lgP | Mass | Length | ppm | m/z | z | RT | Fraction | Scan | Source File | Area BuCaMH04\_B15 | #Feature | #Feature BuCaMH04\_B15 | Start | End | PTM | AScore | Found By |
| --- | --- | --- | --- | --- | --- | --- | --- | --- | --- | --- | --- | --- | --- | --- | --- | --- | --- | --- | --- |
| R.C(+57.02)DSGFYYSEEVC(+57.02)R.C | Y | 50.53 | 1670.6340 | 13 | 0.8 | 836.3250 | 2 | 33.63 | 1 | 21186 | BuCaMH04.raw | 3.2389E5 | 1 | 1 | 450 | 462 | Carbamidomethylation | C1:Carbamidomethylation:1000.00;C12:Carbamidomethylation:1000.00 | PEAKS DB |
| total 1 peptides |  |  |  |  |  |  |  |  |  |  |  |  |  |  |  |  |  |  |  |
| --- | --- | --- | --- | --- | --- | --- | --- | --- | --- | --- | --- | --- | --- | --- | --- | --- | --- | --- | --- |

LAB17921.1

back to list

  

| Protein Coverage
| Supporting Peptides
|

Protein Coverage:

Supporting Peptides:

| Peptide | Uniq | -10lgP | Mass | Length | ppm | m/z | z | RT | Fraction | Scan | Source File | Area BuCaMH04\_B15 | #Feature | #Feature BuCaMH04\_B15 | Start | End | PTM | AScore | Found By |
| --- | --- | --- | --- | --- | --- | --- | --- | --- | --- | --- | --- | --- | --- | --- | --- | --- | --- | --- | --- |
| R.C(+57.02)DSGFYYSEEVC(+57.02)R.C | Y | 50.53 | 1670.6340 | 13 | 0.8 | 836.3250 | 2 | 33.63 | 1 | 21186 | BuCaMH04.raw | 3.2389E5 | 1 | 1 | 454 | 466 | Carbamidomethylation | C1:Carbamidomethylation:1000.00;C12:Carbamidomethylation:1000.00 | PEAKS DB |
| total 1 peptides |  |  |  |  |  |  |  |  |  |  |  |  |  |  |  |  |  |  |  |
| --- | --- | --- | --- | --- | --- | --- | --- | --- | --- | --- | --- | --- | --- | --- | --- | --- | --- | --- | --- |

T\_47

back to list

  

| Protein Coverage
| Supporting Peptides
|

Protein Coverage:

Supporting Peptides:

| Peptide | Uniq | -10lgP | Mass | Length | ppm | m/z | z | RT | Fraction | Scan | Source File | Area BuCaMH04\_B15 | #Feature | #Feature BuCaMH04\_B15 | Start | End | PTM | AScore | Found By |
| --- | --- | --- | --- | --- | --- | --- | --- | --- | --- | --- | --- | --- | --- | --- | --- | --- | --- | --- | --- |
| R.C(+57.02)DSGFYYSEEVC(+57.02)R.C | Y | 50.53 | 1670.6340 | 13 | 0.8 | 836.3250 | 2 | 33.63 | 1 | 21186 | BuCaMH04.raw | 3.2389E5 | 1 | 1 | 265 | 277 | Carbamidomethylation | C1:Carbamidomethylation:1000.00;C12:Carbamidomethylation:1000.00 | PEAKS DB |
| total 1 peptides |  |  |  |  |  |  |  |  |  |  |  |  |  |  |  |  |  |  |  |
| --- | --- | --- | --- | --- | --- | --- | --- | --- | --- | --- | --- | --- | --- | --- | --- | --- | --- | --- | --- |

LAB17920.1

back to list

  

| Protein Coverage
| Supporting Peptides
|

Protein Coverage:

Supporting Peptides:

| Peptide | Uniq | -10lgP | Mass | Length | ppm | m/z | z | RT | Fraction | Scan | Source File | Area BuCaMH04\_B15 | #Feature | #Feature BuCaMH04\_B15 | Start | End | PTM | AScore | Found By |
| --- | --- | --- | --- | --- | --- | --- | --- | --- | --- | --- | --- | --- | --- | --- | --- | --- | --- | --- | --- |
| R.C(+57.02)DSGFYYSEEVC(+57.02)R.C | Y | 50.53 | 1670.6340 | 13 | 0.8 | 836.3250 | 2 | 33.63 | 1 | 21186 | BuCaMH04.raw | 3.2389E5 | 1 | 1 | 265 | 277 | Carbamidomethylation | C1:Carbamidomethylation:1000.00;C12:Carbamidomethylation:1000.00 | PEAKS DB |
| total 1 peptides |  |  |  |  |  |  |  |  |  |  |  |  |  |  |  |  |  |  |  |
| --- | --- | --- | --- | --- | --- | --- | --- | --- | --- | --- | --- | --- | --- | --- | --- | --- | --- | --- | --- |

T\_46

back to list

  

| Protein Coverage
| Supporting Peptides
|

Protein Coverage:

Supporting Peptides:

| Peptide | Uniq | -10lgP | Mass | Length | ppm | m/z | z | RT | Fraction | Scan | Source File | Area BuCaMH04\_B15 | #Feature | #Feature BuCaMH04\_B15 | Start | End | PTM | AScore | Found By |
| --- | --- | --- | --- | --- | --- | --- | --- | --- | --- | --- | --- | --- | --- | --- | --- | --- | --- | --- | --- |
| R.C(+57.02)DSGFYYSEEVC(+57.02)R.C | Y | 50.53 | 1670.6340 | 13 | 0.8 | 836.3250 | 2 | 33.63 | 1 | 21186 | BuCaMH04.raw | 3.2389E5 | 1 | 1 | 328 | 340 | Carbamidomethylation | C1:Carbamidomethylation:1000.00;C12:Carbamidomethylation:1000.00 | PEAKS DB |
| total 1 peptides |  |  |  |  |  |  |  |  |  |  |  |  |  |  |  |  |  |  |  |
| --- | --- | --- | --- | --- | --- | --- | --- | --- | --- | --- | --- | --- | --- | --- | --- | --- | --- | --- | --- |

ETE59007.1

back to list

  

| Protein Coverage
| Supporting Peptides
|

Protein Coverage:

Supporting Peptides:

| Peptide | Uniq | -10lgP | Mass | Length | ppm | m/z | z | RT | Fraction | Scan | Source File | Area BuCaMH04\_B15 | #Feature | #Feature BuCaMH04\_B15 | Start | End | PTM | AScore | Found By |
| --- | --- | --- | --- | --- | --- | --- | --- | --- | --- | --- | --- | --- | --- | --- | --- | --- | --- | --- | --- |
| total 0 peptides |  |  |  |  |  |  |  |  |  |  |  |  |  |  |  |  |  |  |  |
| --- | --- | --- | --- | --- | --- | --- | --- | --- | --- | --- | --- | --- | --- | --- | --- | --- | --- | --- | --- |

QIV64750.1

back to list

  

| Protein Coverage
| Supporting Peptides
|

Protein Coverage:

Supporting Peptides:

| Peptide | Uniq | -10lgP | Mass | Length | ppm | m/z | z | RT | Fraction | Scan | Source File | Area BuCaMH04\_B15 | #Feature | #Feature BuCaMH04\_B15 | Start | End | PTM | AScore | Found By |
| --- | --- | --- | --- | --- | --- | --- | --- | --- | --- | --- | --- | --- | --- | --- | --- | --- | --- | --- | --- |
| total 0 peptides |  |  |  |  |  |  |  |  |  |  |  |  |  |  |  |  |  |  |  |
| --- | --- | --- | --- | --- | --- | --- | --- | --- | --- | --- | --- | --- | --- | --- | --- | --- | --- | --- | --- |

AFJ49237.1

back to list

  

| Protein Coverage
| Supporting Peptides
|

Protein Coverage:

Supporting Peptides:

| Peptide | Uniq | -10lgP | Mass | Length | ppm | m/z | z | RT | Fraction | Scan | Source File | Area BuCaMH04\_B15 | #Feature | #Feature BuCaMH04\_B15 | Start | End | PTM | AScore | Found By |
| --- | --- | --- | --- | --- | --- | --- | --- | --- | --- | --- | --- | --- | --- | --- | --- | --- | --- | --- | --- |
| total 0 peptides |  |  |  |  |  |  |  |  |  |  |  |  |  |  |  |  |  |  |  |
| --- | --- | --- | --- | --- | --- | --- | --- | --- | --- | --- | --- | --- | --- | --- | --- | --- | --- | --- | --- |

AAN39540.1

back to list

  

| Protein Coverage
| Supporting Peptides
|

Protein Coverage:

Supporting Peptides:

| Peptide | Uniq | -10lgP | Mass | Length | ppm | m/z | z | RT | Fraction | Scan | Source File | Area BuCaMH04\_B15 | #Feature | #Feature BuCaMH04\_B15 | Start | End | PTM | AScore | Found By |
| --- | --- | --- | --- | --- | --- | --- | --- | --- | --- | --- | --- | --- | --- | --- | --- | --- | --- | --- | --- |
| total 0 peptides |  |  |  |  |  |  |  |  |  |  |  |  |  |  |  |  |  |  |  |
| --- | --- | --- | --- | --- | --- | --- | --- | --- | --- | --- | --- | --- | --- | --- | --- | --- | --- | --- | --- |

JC8056

back to list

  

| Protein Coverage
| Supporting Peptides
|

Protein Coverage:

Supporting Peptides:

| Peptide | Uniq | -10lgP | Mass | Length | ppm | m/z | z | RT | Fraction | Scan | Source File | Area BuCaMH04\_B15 | #Feature | #Feature BuCaMH04\_B15 | Start | End | PTM | AScore | Found By |
| --- | --- | --- | --- | --- | --- | --- | --- | --- | --- | --- | --- | --- | --- | --- | --- | --- | --- | --- | --- |
| total 0 peptides |  |  |  |  |  |  |  |  |  |  |  |  |  |  |  |  |  |  |  |
| --- | --- | --- | --- | --- | --- | --- | --- | --- | --- | --- | --- | --- | --- | --- | --- | --- | --- | --- | --- |

QIV64763.1

back to list

  

| Protein Coverage
| Supporting Peptides
|

Protein Coverage:

Supporting Peptides:

| Peptide | Uniq | -10lgP | Mass | Length | ppm | m/z | z | RT | Fraction | Scan | Source File | Area BuCaMH04\_B15 | #Feature | #Feature BuCaMH04\_B15 | Start | End | PTM | AScore | Found By |
| --- | --- | --- | --- | --- | --- | --- | --- | --- | --- | --- | --- | --- | --- | --- | --- | --- | --- | --- | --- |
| total 0 peptides |  |  |  |  |  |  |  |  |  |  |  |  |  |  |  |  |  |  |  |
| --- | --- | --- | --- | --- | --- | --- | --- | --- | --- | --- | --- | --- | --- | --- | --- | --- | --- | --- | --- |

JAS05305.1

back to list

  

| Protein Coverage
| Supporting Peptides
|

Protein Coverage:

Supporting Peptides:

| Peptide | Uniq | -10lgP | Mass | Length | ppm | m/z | z | RT | Fraction | Scan | Source File | Area BuCaMH04\_B15 | #Feature | #Feature BuCaMH04\_B15 | Start | End | PTM | AScore | Found By |
| --- | --- | --- | --- | --- | --- | --- | --- | --- | --- | --- | --- | --- | --- | --- | --- | --- | --- | --- | --- |
| total 0 peptides |  |  |  |  |  |  |  |  |  |  |  |  |  |  |  |  |  |  |  |
| --- | --- | --- | --- | --- | --- | --- | --- | --- | --- | --- | --- | --- | --- | --- | --- | --- | --- | --- | --- |

JAS04678.1

back to list

  

| Protein Coverage
| Supporting Peptides
|

Protein Coverage:

Supporting Peptides:

| Peptide | Uniq | -10lgP | Mass | Length | ppm | m/z | z | RT | Fraction | Scan | Source File | Area BuCaMH04\_B15 | #Feature | #Feature BuCaMH04\_B15 | Start | End | PTM | AScore | Found By |
| --- | --- | --- | --- | --- | --- | --- | --- | --- | --- | --- | --- | --- | --- | --- | --- | --- | --- | --- | --- |
| total 0 peptides |  |  |  |  |  |  |  |  |  |  |  |  |  |  |  |  |  |  |  |
| --- | --- | --- | --- | --- | --- | --- | --- | --- | --- | --- | --- | --- | --- | --- | --- | --- | --- | --- | --- |

AFJ49236.1

back to list

  

| Protein Coverage
| Supporting Peptides
|

Protein Coverage:

Supporting Peptides:

| Peptide | Uniq | -10lgP | Mass | Length | ppm | m/z | z | RT | Fraction | Scan | Source File | Area BuCaMH04\_B15 | #Feature | #Feature BuCaMH04\_B15 | Start | End | PTM | AScore | Found By |
| --- | --- | --- | --- | --- | --- | --- | --- | --- | --- | --- | --- | --- | --- | --- | --- | --- | --- | --- | --- |
| total 0 peptides |  |  |  |  |  |  |  |  |  |  |  |  |  |  |  |  |  |  |  |
| --- | --- | --- | --- | --- | --- | --- | --- | --- | --- | --- | --- | --- | --- | --- | --- | --- | --- | --- | --- |

JAI10382.1

back to list

  

| Protein Coverage
| Supporting Peptides
|

Protein Coverage:

Supporting Peptides:

| Peptide | Uniq | -10lgP | Mass | Length | ppm | m/z | z | RT | Fraction | Scan | Source File | Area BuCaMH04\_B15 | #Feature | #Feature BuCaMH04\_B15 | Start | End | PTM | AScore | Found By |
| --- | --- | --- | --- | --- | --- | --- | --- | --- | --- | --- | --- | --- | --- | --- | --- | --- | --- | --- | --- |
| total 0 peptides |  |  |  |  |  |  |  |  |  |  |  |  |  |  |  |  |  |  |  |
| --- | --- | --- | --- | --- | --- | --- | --- | --- | --- | --- | --- | --- | --- | --- | --- | --- | --- | --- | --- |

AFJ49235.1

back to list

  

| Protein Coverage
| Supporting Peptides
|

Protein Coverage:

Supporting Peptides:

| Peptide | Uniq | -10lgP | Mass | Length | ppm | m/z | z | RT | Fraction | Scan | Source File | Area BuCaMH04\_B15 | #Feature | #Feature BuCaMH04\_B15 | Start | End | PTM | AScore | Found By |
| --- | --- | --- | --- | --- | --- | --- | --- | --- | --- | --- | --- | --- | --- | --- | --- | --- | --- | --- | --- |
| total 0 peptides |  |  |  |  |  |  |  |  |  |  |  |  |  |  |  |  |  |  |  |
| --- | --- | --- | --- | --- | --- | --- | --- | --- | --- | --- | --- | --- | --- | --- | --- | --- | --- | --- | --- |

AFJ49233.1

back to list

  

| Protein Coverage
| Supporting Peptides
|

Protein Coverage:

Supporting Peptides:

| Peptide | Uniq | -10lgP | Mass | Length | ppm | m/z | z | RT | Fraction | Scan | Source File | Area BuCaMH04\_B15 | #Feature | #Feature BuCaMH04\_B15 | Start | End | PTM | AScore | Found By |
| --- | --- | --- | --- | --- | --- | --- | --- | --- | --- | --- | --- | --- | --- | --- | --- | --- | --- | --- | --- |
| total 0 peptides |  |  |  |  |  |  |  |  |  |  |  |  |  |  |  |  |  |  |  |
| --- | --- | --- | --- | --- | --- | --- | --- | --- | --- | --- | --- | --- | --- | --- | --- | --- | --- | --- | --- |

JAI10384.1

back to list

  

| Protein Coverage
| Supporting Peptides
|

Protein Coverage:

Supporting Peptides:

| Peptide | Uniq | -10lgP | Mass | Length | ppm | m/z | z | RT | Fraction | Scan | Source File | Area BuCaMH04\_B15 | #Feature | #Feature BuCaMH04\_B15 | Start | End | PTM | AScore | Found By |
| --- | --- | --- | --- | --- | --- | --- | --- | --- | --- | --- | --- | --- | --- | --- | --- | --- | --- | --- | --- |
| total 0 peptides |  |  |  |  |  |  |  |  |  |  |  |  |  |  |  |  |  |  |  |
| --- | --- | --- | --- | --- | --- | --- | --- | --- | --- | --- | --- | --- | --- | --- | --- | --- | --- | --- | --- |

JAS04680.1

back to list

  

| Protein Coverage
| Supporting Peptides
|

Protein Coverage:

Supporting Peptides:

| Peptide | Uniq | -10lgP | Mass | Length | ppm | m/z | z | RT | Fraction | Scan | Source File | Area BuCaMH04\_B15 | #Feature | #Feature BuCaMH04\_B15 | Start | End | PTM | AScore | Found By |
| --- | --- | --- | --- | --- | --- | --- | --- | --- | --- | --- | --- | --- | --- | --- | --- | --- | --- | --- | --- |
| total 0 peptides |  |  |  |  |  |  |  |  |  |  |  |  |  |  |  |  |  |  |  |
| --- | --- | --- | --- | --- | --- | --- | --- | --- | --- | --- | --- | --- | --- | --- | --- | --- | --- | --- | --- |

QIV64952.1

back to list

  

| Protein Coverage
| Supporting Peptides
|

Protein Coverage:

Supporting Peptides:

| Peptide | Uniq | -10lgP | Mass | Length | ppm | m/z | z | RT | Fraction | Scan | Source File | Area BuCaMH04\_B15 | #Feature | #Feature BuCaMH04\_B15 | Start | End | PTM | AScore | Found By |
| --- | --- | --- | --- | --- | --- | --- | --- | --- | --- | --- | --- | --- | --- | --- | --- | --- | --- | --- | --- |
| total 0 peptides |  |  |  |  |  |  |  |  |  |  |  |  |  |  |  |  |  |  |  |
| --- | --- | --- | --- | --- | --- | --- | --- | --- | --- | --- | --- | --- | --- | --- | --- | --- | --- | --- | --- |

JAS05411.1

back to list

  

| Protein Coverage
| Supporting Peptides
|

Protein Coverage:

Supporting Peptides:

| Peptide | Uniq | -10lgP | Mass | Length | ppm | m/z | z | RT | Fraction | Scan | Source File | Area BuCaMH04\_B15 | #Feature | #Feature BuCaMH04\_B15 | Start | End | PTM | AScore | Found By |
| --- | --- | --- | --- | --- | --- | --- | --- | --- | --- | --- | --- | --- | --- | --- | --- | --- | --- | --- | --- |
| total 0 peptides |  |  |  |  |  |  |  |  |  |  |  |  |  |  |  |  |  |  |  |
| --- | --- | --- | --- | --- | --- | --- | --- | --- | --- | --- | --- | --- | --- | --- | --- | --- | --- | --- | --- |

JAS05412.1

back to list

  

| Protein Coverage
| Supporting Peptides
|

Protein Coverage:

Supporting Peptides:

| Peptide | Uniq | -10lgP | Mass | Length | ppm | m/z | z | RT | Fraction | Scan | Source File | Area BuCaMH04\_B15 | #Feature | #Feature BuCaMH04\_B15 | Start | End | PTM | AScore | Found By |
| --- | --- | --- | --- | --- | --- | --- | --- | --- | --- | --- | --- | --- | --- | --- | --- | --- | --- | --- | --- |
| total 0 peptides |  |  |  |  |  |  |  |  |  |  |  |  |  |  |  |  |  |  |  |
| --- | --- | --- | --- | --- | --- | --- | --- | --- | --- | --- | --- | --- | --- | --- | --- | --- | --- | --- | --- |

JAS05304.1

back to list

  

| Protein Coverage
| Supporting Peptides
|

Protein Coverage:

Supporting Peptides:

| Peptide | Uniq | -10lgP | Mass | Length | ppm | m/z | z | RT | Fraction | Scan | Source File | Area BuCaMH04\_B15 | #Feature | #Feature BuCaMH04\_B15 | Start | End | PTM | AScore | Found By |
| --- | --- | --- | --- | --- | --- | --- | --- | --- | --- | --- | --- | --- | --- | --- | --- | --- | --- | --- | --- |
| total 0 peptides |  |  |  |  |  |  |  |  |  |  |  |  |  |  |  |  |  |  |  |
| --- | --- | --- | --- | --- | --- | --- | --- | --- | --- | --- | --- | --- | --- | --- | --- | --- | --- | --- | --- |

QIV64951.1

back to list

  

| Protein Coverage
| Supporting Peptides
|

Protein Coverage:

Supporting Peptides:

| Peptide | Uniq | -10lgP | Mass | Length | ppm | m/z | z | RT | Fraction | Scan | Source File | Area BuCaMH04\_B15 | #Feature | #Feature BuCaMH04\_B15 | Start | End | PTM | AScore | Found By |
| --- | --- | --- | --- | --- | --- | --- | --- | --- | --- | --- | --- | --- | --- | --- | --- | --- | --- | --- | --- |
| total 0 peptides |  |  |  |  |  |  |  |  |  |  |  |  |  |  |  |  |  |  |  |
| --- | --- | --- | --- | --- | --- | --- | --- | --- | --- | --- | --- | --- | --- | --- | --- | --- | --- | --- | --- |

JAS05442.1

back to list

  

| Protein Coverage
| Supporting Peptides
|

Protein Coverage:

Supporting Peptides:

| Peptide | Uniq | -10lgP | Mass | Length | ppm | m/z | z | RT | Fraction | Scan | Source File | Area BuCaMH04\_B15 | #Feature | #Feature BuCaMH04\_B15 | Start | End | PTM | AScore | Found By |
| --- | --- | --- | --- | --- | --- | --- | --- | --- | --- | --- | --- | --- | --- | --- | --- | --- | --- | --- | --- |
| total 0 peptides |  |  |  |  |  |  |  |  |  |  |  |  |  |  |  |  |  |  |  |
| --- | --- | --- | --- | --- | --- | --- | --- | --- | --- | --- | --- | --- | --- | --- | --- | --- | --- | --- | --- |

AJI77146.1

back to list

  

| Protein Coverage
| Supporting Peptides
|

Protein Coverage:

Supporting Peptides:

| Peptide | Uniq | -10lgP | Mass | Length | ppm | m/z | z | RT | Fraction | Scan | Source File | Area BuCaMH04\_B15 | #Feature | #Feature BuCaMH04\_B15 | Start | End | PTM | AScore | Found By |
| --- | --- | --- | --- | --- | --- | --- | --- | --- | --- | --- | --- | --- | --- | --- | --- | --- | --- | --- | --- |
| total 0 peptides |  |  |  |  |  |  |  |  |  |  |  |  |  |  |  |  |  |  |  |
| --- | --- | --- | --- | --- | --- | --- | --- | --- | --- | --- | --- | --- | --- | --- | --- | --- | --- | --- | --- |

JAV48406.1

back to list

  

| Protein Coverage
| Supporting Peptides
|

Protein Coverage:

Supporting Peptides:

| Peptide | Uniq | -10lgP | Mass | Length | ppm | m/z | z | RT | Fraction | Scan | Source File | Area BuCaMH04\_B15 | #Feature | #Feature BuCaMH04\_B15 | Start | End | PTM | AScore | Found By |
| --- | --- | --- | --- | --- | --- | --- | --- | --- | --- | --- | --- | --- | --- | --- | --- | --- | --- | --- | --- |
| total 0 peptides |  |  |  |  |  |  |  |  |  |  |  |  |  |  |  |  |  |  |  |
| --- | --- | --- | --- | --- | --- | --- | --- | --- | --- | --- | --- | --- | --- | --- | --- | --- | --- | --- | --- |

JAI10383.1

back to list

  

| Protein Coverage
| Supporting Peptides
|

Protein Coverage:

Supporting Peptides:

| Peptide | Uniq | -10lgP | Mass | Length | ppm | m/z | z | RT | Fraction | Scan | Source File | Area BuCaMH04\_B15 | #Feature | #Feature BuCaMH04\_B15 | Start | End | PTM | AScore | Found By |
| --- | --- | --- | --- | --- | --- | --- | --- | --- | --- | --- | --- | --- | --- | --- | --- | --- | --- | --- | --- |
| total 0 peptides |  |  |  |  |  |  |  |  |  |  |  |  |  |  |  |  |  |  |  |
| --- | --- | --- | --- | --- | --- | --- | --- | --- | --- | --- | --- | --- | --- | --- | --- | --- | --- | --- | --- |

JAI10380.1

back to list

  

| Protein Coverage
| Supporting Peptides
|

Protein Coverage:

Supporting Peptides:

| Peptide | Uniq | -10lgP | Mass | Length | ppm | m/z | z | RT | Fraction | Scan | Source File | Area BuCaMH04\_B15 | #Feature | #Feature BuCaMH04\_B15 | Start | End | PTM | AScore | Found By |
| --- | --- | --- | --- | --- | --- | --- | --- | --- | --- | --- | --- | --- | --- | --- | --- | --- | --- | --- | --- |
| total 0 peptides |  |  |  |  |  |  |  |  |  |  |  |  |  |  |  |  |  |  |  |
| --- | --- | --- | --- | --- | --- | --- | --- | --- | --- | --- | --- | --- | --- | --- | --- | --- | --- | --- | --- |

JAS04676.1

back to list

  

| Protein Coverage
| Supporting Peptides
|

Protein Coverage:

Supporting Peptides:

| Peptide | Uniq | -10lgP | Mass | Length | ppm | m/z | z | RT | Fraction | Scan | Source File | Area BuCaMH04\_B15 | #Feature | #Feature BuCaMH04\_B15 | Start | End | PTM | AScore | Found By |
| --- | --- | --- | --- | --- | --- | --- | --- | --- | --- | --- | --- | --- | --- | --- | --- | --- | --- | --- | --- |
| total 0 peptides |  |  |  |  |  |  |  |  |  |  |  |  |  |  |  |  |  |  |  |
| --- | --- | --- | --- | --- | --- | --- | --- | --- | --- | --- | --- | --- | --- | --- | --- | --- | --- | --- | --- |

JAI10381.1

back to list

  

| Protein Coverage
| Supporting Peptides
|

Protein Coverage:

Supporting Peptides:

| Peptide | Uniq | -10lgP | Mass | Length | ppm | m/z | z | RT | Fraction | Scan | Source File | Area BuCaMH04\_B15 | #Feature | #Feature BuCaMH04\_B15 | Start | End | PTM | AScore | Found By |
| --- | --- | --- | --- | --- | --- | --- | --- | --- | --- | --- | --- | --- | --- | --- | --- | --- | --- | --- | --- |
| total 0 peptides |  |  |  |  |  |  |  |  |  |  |  |  |  |  |  |  |  |  |  |
| --- | --- | --- | --- | --- | --- | --- | --- | --- | --- | --- | --- | --- | --- | --- | --- | --- | --- | --- | --- |

JAS04679.1

back to list

  

| Protein Coverage
| Supporting Peptides
|

Protein Coverage:

Supporting Peptides:

| Peptide | Uniq | -10lgP | Mass | Length | ppm | m/z | z | RT | Fraction | Scan | Source File | Area BuCaMH04\_B15 | #Feature | #Feature BuCaMH04\_B15 | Start | End | PTM | AScore | Found By |
| --- | --- | --- | --- | --- | --- | --- | --- | --- | --- | --- | --- | --- | --- | --- | --- | --- | --- | --- | --- |
| total 0 peptides |  |  |  |  |  |  |  |  |  |  |  |  |  |  |  |  |  |  |  |
| --- | --- | --- | --- | --- | --- | --- | --- | --- | --- | --- | --- | --- | --- | --- | --- | --- | --- | --- | --- |

AFJ49234.1

back to list

  

| Protein Coverage
| Supporting Peptides
|

Protein Coverage:

Supporting Peptides:

| Peptide | Uniq | -10lgP | Mass | Length | ppm | m/z | z | RT | Fraction | Scan | Source File | Area BuCaMH04\_B15 | #Feature | #Feature BuCaMH04\_B15 | Start | End | PTM | AScore | Found By |
| --- | --- | --- | --- | --- | --- | --- | --- | --- | --- | --- | --- | --- | --- | --- | --- | --- | --- | --- | --- |
| total 0 peptides |  |  |  |  |  |  |  |  |  |  |  |  |  |  |  |  |  |  |  |
| --- | --- | --- | --- | --- | --- | --- | --- | --- | --- | --- | --- | --- | --- | --- | --- | --- | --- | --- | --- |

AEJ31989.1

back to list

  

| Protein Coverage
| Supporting Peptides
|

Protein Coverage:

Supporting Peptides:

| Peptide | Uniq | -10lgP | Mass | Length | ppm | m/z | z | RT | Fraction | Scan | Source File | Area BuCaMH04\_B15 | #Feature | #Feature BuCaMH04\_B15 | Start | End | PTM | AScore | Found By |
| --- | --- | --- | --- | --- | --- | --- | --- | --- | --- | --- | --- | --- | --- | --- | --- | --- | --- | --- | --- |
| total 0 peptides |  |  |  |  |  |  |  |  |  |  |  |  |  |  |  |  |  |  |  |
| --- | --- | --- | --- | --- | --- | --- | --- | --- | --- | --- | --- | --- | --- | --- | --- | --- | --- | --- | --- |

AEJ31987.1

back to list

  

| Protein Coverage
| Supporting Peptides
|

Protein Coverage:

Supporting Peptides:

| Peptide | Uniq | -10lgP | Mass | Length | ppm | m/z | z | RT | Fraction | Scan | Source File | Area BuCaMH04\_B15 | #Feature | #Feature BuCaMH04\_B15 | Start | End | PTM | AScore | Found By |
| --- | --- | --- | --- | --- | --- | --- | --- | --- | --- | --- | --- | --- | --- | --- | --- | --- | --- | --- | --- |
| total 0 peptides |  |  |  |  |  |  |  |  |  |  |  |  |  |  |  |  |  |  |  |
| --- | --- | --- | --- | --- | --- | --- | --- | --- | --- | --- | --- | --- | --- | --- | --- | --- | --- | --- | --- |

JAS04677.1

back to list

  

| Protein Coverage
| Supporting Peptides
|

Protein Coverage:

Supporting Peptides:

| Peptide | Uniq | -10lgP | Mass | Length | ppm | m/z | z | RT | Fraction | Scan | Source File | Area BuCaMH04\_B15 | #Feature | #Feature BuCaMH04\_B15 | Start | End | PTM | AScore | Found By |
| --- | --- | --- | --- | --- | --- | --- | --- | --- | --- | --- | --- | --- | --- | --- | --- | --- | --- | --- | --- |
| total 0 peptides |  |  |  |  |  |  |  |  |  |  |  |  |  |  |  |  |  |  |  |
| --- | --- | --- | --- | --- | --- | --- | --- | --- | --- | --- | --- | --- | --- | --- | --- | --- | --- | --- | --- |

XP\_015667429.1

back to list

  

| Protein Coverage
| Supporting Peptides
|

Protein Coverage:

Supporting Peptides:

| Peptide | Uniq | -10lgP | Mass | Length | ppm | m/z | z | RT | Fraction | Scan | Source File | Area BuCaMH04\_B15 | #Feature | #Feature BuCaMH04\_B15 | Start | End | PTM | AScore | Found By |
| --- | --- | --- | --- | --- | --- | --- | --- | --- | --- | --- | --- | --- | --- | --- | --- | --- | --- | --- | --- |
| total 0 peptides |  |  |  |  |  |  |  |  |  |  |  |  |  |  |  |  |  |  |  |
| --- | --- | --- | --- | --- | --- | --- | --- | --- | --- | --- | --- | --- | --- | --- | --- | --- | --- | --- | --- |

XP\_015667428.1

back to list

  

| Protein Coverage
| Supporting Peptides
|

Protein Coverage:

Supporting Peptides:

| Peptide | Uniq | -10lgP | Mass | Length | ppm | m/z | z | RT | Fraction | Scan | Source File | Area BuCaMH04\_B15 | #Feature | #Feature BuCaMH04\_B15 | Start | End | PTM | AScore | Found By |
| --- | --- | --- | --- | --- | --- | --- | --- | --- | --- | --- | --- | --- | --- | --- | --- | --- | --- | --- | --- |
| total 0 peptides |  |  |  |  |  |  |  |  |  |  |  |  |  |  |  |  |  |  |  |
| --- | --- | --- | --- | --- | --- | --- | --- | --- | --- | --- | --- | --- | --- | --- | --- | --- | --- | --- | --- |

XP\_015667426.1

back to list

  

| Protein Coverage
| Supporting Peptides
|

Protein Coverage:

Supporting Peptides:

| Peptide | Uniq | -10lgP | Mass | Length | ppm | m/z | z | RT | Fraction | Scan | Source File | Area BuCaMH04\_B15 | #Feature | #Feature BuCaMH04\_B15 | Start | End | PTM | AScore | Found By |
| --- | --- | --- | --- | --- | --- | --- | --- | --- | --- | --- | --- | --- | --- | --- | --- | --- | --- | --- | --- |
| total 0 peptides |  |  |  |  |  |  |  |  |  |  |  |  |  |  |  |  |  |  |  |
| --- | --- | --- | --- | --- | --- | --- | --- | --- | --- | --- | --- | --- | --- | --- | --- | --- | --- | --- | --- |

JAI14540.1

back to list

  

| Protein Coverage
| Supporting Peptides
|

Protein Coverage:

Supporting Peptides:

| Peptide | Uniq | -10lgP | Mass | Length | ppm | m/z | z | RT | Fraction | Scan | Source File | Area BuCaMH04\_B15 | #Feature | #Feature BuCaMH04\_B15 | Start | End | PTM | AScore | Found By |
| --- | --- | --- | --- | --- | --- | --- | --- | --- | --- | --- | --- | --- | --- | --- | --- | --- | --- | --- | --- |
| total 0 peptides |  |  |  |  |  |  |  |  |  |  |  |  |  |  |  |  |  |  |  |
| --- | --- | --- | --- | --- | --- | --- | --- | --- | --- | --- | --- | --- | --- | --- | --- | --- | --- | --- | --- |

AFJ49243.1

back to list

  

| Protein Coverage
| Supporting Peptides
|

Protein Coverage:

Supporting Peptides:

| Peptide | Uniq | -10lgP | Mass | Length | ppm | m/z | z | RT | Fraction | Scan | Source File | Area BuCaMH04\_B15 | #Feature | #Feature BuCaMH04\_B15 | Start | End | PTM | AScore | Found By |
| --- | --- | --- | --- | --- | --- | --- | --- | --- | --- | --- | --- | --- | --- | --- | --- | --- | --- | --- | --- |
| total 0 peptides |  |  |  |  |  |  |  |  |  |  |  |  |  |  |  |  |  |  |  |
| --- | --- | --- | --- | --- | --- | --- | --- | --- | --- | --- | --- | --- | --- | --- | --- | --- | --- | --- | --- |

JAV48412.1

back to list

  

| Protein Coverage
| Supporting Peptides
|

Protein Coverage:

Supporting Peptides:

| Peptide | Uniq | -10lgP | Mass | Length | ppm | m/z | z | RT | Fraction | Scan | Source File | Area BuCaMH04\_B15 | #Feature | #Feature BuCaMH04\_B15 | Start | End | PTM | AScore | Found By |
| --- | --- | --- | --- | --- | --- | --- | --- | --- | --- | --- | --- | --- | --- | --- | --- | --- | --- | --- | --- |
| total 0 peptides |  |  |  |  |  |  |  |  |  |  |  |  |  |  |  |  |  |  |  |
| --- | --- | --- | --- | --- | --- | --- | --- | --- | --- | --- | --- | --- | --- | --- | --- | --- | --- | --- | --- |

AEJ31991.1

back to list

  

| Protein Coverage
| Supporting Peptides
|

Protein Coverage:

Supporting Peptides:

| Peptide | Uniq | -10lgP | Mass | Length | ppm | m/z | z | RT | Fraction | Scan | Source File | Area BuCaMH04\_B15 | #Feature | #Feature BuCaMH04\_B15 | Start | End | PTM | AScore | Found By |
| --- | --- | --- | --- | --- | --- | --- | --- | --- | --- | --- | --- | --- | --- | --- | --- | --- | --- | --- | --- |
| total 0 peptides |  |  |  |  |  |  |  |  |  |  |  |  |  |  |  |  |  |  |  |
| --- | --- | --- | --- | --- | --- | --- | --- | --- | --- | --- | --- | --- | --- | --- | --- | --- | --- | --- | --- |

QIV64930.1

back to list

  

| Protein Coverage
| Supporting Peptides
|

Protein Coverage:

Supporting Peptides:

| Peptide | Uniq | -10lgP | Mass | Length | ppm | m/z | z | RT | Fraction | Scan | Source File | Area BuCaMH04\_B15 | #Feature | #Feature BuCaMH04\_B15 | Start | End | PTM | AScore | Found By |
| --- | --- | --- | --- | --- | --- | --- | --- | --- | --- | --- | --- | --- | --- | --- | --- | --- | --- | --- | --- |
| total 0 peptides |  |  |  |  |  |  |  |  |  |  |  |  |  |  |  |  |  |  |  |
| --- | --- | --- | --- | --- | --- | --- | --- | --- | --- | --- | --- | --- | --- | --- | --- | --- | --- | --- | --- |

Q7T1K6.1

back to list

  

| Protein Coverage
| Supporting Peptides
|

Protein Coverage:

Supporting Peptides:

| Peptide | Uniq | -10lgP | Mass | Length | ppm | m/z | z | RT | Fraction | Scan | Source File | Area BuCaMH04\_B15 | #Feature | #Feature BuCaMH04\_B15 | Start | End | PTM | AScore | Found By |
| --- | --- | --- | --- | --- | --- | --- | --- | --- | --- | --- | --- | --- | --- | --- | --- | --- | --- | --- | --- |
| total 0 peptides |  |  |  |  |  |  |  |  |  |  |  |  |  |  |  |  |  |  |  |
| --- | --- | --- | --- | --- | --- | --- | --- | --- | --- | --- | --- | --- | --- | --- | --- | --- | --- | --- | --- |

ACH73167.1

back to list

  

| Protein Coverage
| Supporting Peptides
|

Protein Coverage:

Supporting Peptides:

| Peptide | Uniq | -10lgP | Mass | Length | ppm | m/z | z | RT | Fraction | Scan | Source File | Area BuCaMH04\_B15 | #Feature | #Feature BuCaMH04\_B15 | Start | End | PTM | AScore | Found By |
| --- | --- | --- | --- | --- | --- | --- | --- | --- | --- | --- | --- | --- | --- | --- | --- | --- | --- | --- | --- |
| total 0 peptides |  |  |  |  |  |  |  |  |  |  |  |  |  |  |  |  |  |  |  |
| --- | --- | --- | --- | --- | --- | --- | --- | --- | --- | --- | --- | --- | --- | --- | --- | --- | --- | --- | --- |

P84805.2

back to list

  

| Protein Coverage
| Supporting Peptides
|

Protein Coverage:

Supporting Peptides:

| Peptide | Uniq | -10lgP | Mass | Length | ppm | m/z | z | RT | Fraction | Scan | Source File | Area BuCaMH04\_B15 | #Feature | #Feature BuCaMH04\_B15 | Start | End | PTM | AScore | Found By |
| --- | --- | --- | --- | --- | --- | --- | --- | --- | --- | --- | --- | --- | --- | --- | --- | --- | --- | --- | --- |
| total 0 peptides |  |  |  |  |  |  |  |  |  |  |  |  |  |  |  |  |  |  |  |
| --- | --- | --- | --- | --- | --- | --- | --- | --- | --- | --- | --- | --- | --- | --- | --- | --- | --- | --- | --- |

AAP85301.1

back to list

  

| Protein Coverage
| Supporting Peptides
|

Protein Coverage:

Supporting Peptides:

| Peptide | Uniq | -10lgP | Mass | Length | ppm | m/z | z | RT | Fraction | Scan | Source File | Area BuCaMH04\_B15 | #Feature | #Feature BuCaMH04\_B15 | Start | End | PTM | AScore | Found By |
| --- | --- | --- | --- | --- | --- | --- | --- | --- | --- | --- | --- | --- | --- | --- | --- | --- | --- | --- | --- |
| total 0 peptides |  |  |  |  |  |  |  |  |  |  |  |  |  |  |  |  |  |  |  |
| --- | --- | --- | --- | --- | --- | --- | --- | --- | --- | --- | --- | --- | --- | --- | --- | --- | --- | --- | --- |

1XX5

back to list

  

| Protein Coverage
| Supporting Peptides
|

Protein Coverage:

Supporting Peptides:

| Peptide | Uniq | -10lgP | Mass | Length | ppm | m/z | z | RT | Fraction | Scan | Source File | Area BuCaMH04\_B15 | #Feature | #Feature BuCaMH04\_B15 | Start | End | PTM | AScore | Found By |
| --- | --- | --- | --- | --- | --- | --- | --- | --- | --- | --- | --- | --- | --- | --- | --- | --- | --- | --- | --- |
| total 0 peptides |  |  |  |  |  |  |  |  |  |  |  |  |  |  |  |  |  |  |  |
| --- | --- | --- | --- | --- | --- | --- | --- | --- | --- | --- | --- | --- | --- | --- | --- | --- | --- | --- | --- |

2GIZ

back to list

  

| Protein Coverage
| Supporting Peptides
|

Protein Coverage:

Supporting Peptides:

| Peptide | Uniq | -10lgP | Mass | Length | ppm | m/z | z | RT | Fraction | Scan | Source File | Area BuCaMH04\_B15 | #Feature | #Feature BuCaMH04\_B15 | Start | End | PTM | AScore | Found By |
| --- | --- | --- | --- | --- | --- | --- | --- | --- | --- | --- | --- | --- | --- | --- | --- | --- | --- | --- | --- |
| total 0 peptides |  |  |  |  |  |  |  |  |  |  |  |  |  |  |  |  |  |  |  |
| --- | --- | --- | --- | --- | --- | --- | --- | --- | --- | --- | --- | --- | --- | --- | --- | --- | --- | --- | --- |

3MZ8

back to list

  

| Protein Coverage
| Supporting Peptides
|

Protein Coverage:

Supporting Peptides:

| Peptide | Uniq | -10lgP | Mass | Length | ppm | m/z | z | RT | Fraction | Scan | Source File | Area BuCaMH04\_B15 | #Feature | #Feature BuCaMH04\_B15 | Start | End | PTM | AScore | Found By |
| --- | --- | --- | --- | --- | --- | --- | --- | --- | --- | --- | --- | --- | --- | --- | --- | --- | --- | --- | --- |
| total 0 peptides |  |  |  |  |  |  |  |  |  |  |  |  |  |  |  |  |  |  |  |
| --- | --- | --- | --- | --- | --- | --- | --- | --- | --- | --- | --- | --- | --- | --- | --- | --- | --- | --- | --- |

XP\_026556535.1

back to list

  

| Protein Coverage
| Supporting Peptides
|

Protein Coverage:

Supporting Peptides:

| Peptide | Uniq | -10lgP | Mass | Length | ppm | m/z | z | RT | Fraction | Scan | Source File | Area BuCaMH04\_B15 | #Feature | #Feature BuCaMH04\_B15 | Start | End | PTM | AScore | Found By |
| --- | --- | --- | --- | --- | --- | --- | --- | --- | --- | --- | --- | --- | --- | --- | --- | --- | --- | --- | --- |
| total 0 peptides |  |  |  |  |  |  |  |  |  |  |  |  |  |  |  |  |  |  |  |
| --- | --- | --- | --- | --- | --- | --- | --- | --- | --- | --- | --- | --- | --- | --- | --- | --- | --- | --- | --- |

XP\_026545733.1

back to list

  

| Protein Coverage
| Supporting Peptides
|

Protein Coverage:

Supporting Peptides:

| Peptide | Uniq | -10lgP | Mass | Length | ppm | m/z | z | RT | Fraction | Scan | Source File | Area BuCaMH04\_B15 | #Feature | #Feature BuCaMH04\_B15 | Start | End | PTM | AScore | Found By |
| --- | --- | --- | --- | --- | --- | --- | --- | --- | --- | --- | --- | --- | --- | --- | --- | --- | --- | --- | --- |
| total 0 peptides |  |  |  |  |  |  |  |  |  |  |  |  |  |  |  |  |  |  |  |
| --- | --- | --- | --- | --- | --- | --- | --- | --- | --- | --- | --- | --- | --- | --- | --- | --- | --- | --- | --- |

XP\_026559309.1

back to list

  

| Protein Coverage
| Supporting Peptides
|

Protein Coverage:

Supporting Peptides:

| Peptide | Uniq | -10lgP | Mass | Length | ppm | m/z | z | RT | Fraction | Scan | Source File | Area BuCaMH04\_B15 | #Feature | #Feature BuCaMH04\_B15 | Start | End | PTM | AScore | Found By |
| --- | --- | --- | --- | --- | --- | --- | --- | --- | --- | --- | --- | --- | --- | --- | --- | --- | --- | --- | --- |
| total 0 peptides |  |  |  |  |  |  |  |  |  |  |  |  |  |  |  |  |  |  |  |
| --- | --- | --- | --- | --- | --- | --- | --- | --- | --- | --- | --- | --- | --- | --- | --- | --- | --- | --- | --- |

XP\_034278049.1

back to list

  

| Protein Coverage
| Supporting Peptides
|

Protein Coverage:

Supporting Peptides:

| Peptide | Uniq | -10lgP | Mass | Length | ppm | m/z | z | RT | Fraction | Scan | Source File | Area BuCaMH04\_B15 | #Feature | #Feature BuCaMH04\_B15 | Start | End | PTM | AScore | Found By |
| --- | --- | --- | --- | --- | --- | --- | --- | --- | --- | --- | --- | --- | --- | --- | --- | --- | --- | --- | --- |
| total 0 peptides |  |  |  |  |  |  |  |  |  |  |  |  |  |  |  |  |  |  |  |
| --- | --- | --- | --- | --- | --- | --- | --- | --- | --- | --- | --- | --- | --- | --- | --- | --- | --- | --- | --- |

XP\_013911472.1

back to list

  

| Protein Coverage
| Supporting Peptides
|

Protein Coverage:

Supporting Peptides:

| Peptide | Uniq | -10lgP | Mass | Length | ppm | m/z | z | RT | Fraction | Scan | Source File | Area BuCaMH04\_B15 | #Feature | #Feature BuCaMH04\_B15 | Start | End | PTM | AScore | Found By |
| --- | --- | --- | --- | --- | --- | --- | --- | --- | --- | --- | --- | --- | --- | --- | --- | --- | --- | --- | --- |
| total 0 peptides |  |  |  |  |  |  |  |  |  |  |  |  |  |  |  |  |  |  |  |
| --- | --- | --- | --- | --- | --- | --- | --- | --- | --- | --- | --- | --- | --- | --- | --- | --- | --- | --- | --- |

P60311.1

back to list

  

| Protein Coverage
| Supporting Peptides
|

Protein Coverage:

Supporting Peptides:

| Peptide | Uniq | -10lgP | Mass | Length | ppm | m/z | z | RT | Fraction | Scan | Source File | Area BuCaMH04\_B15 | #Feature | #Feature BuCaMH04\_B15 | Start | End | PTM | AScore | Found By |
| --- | --- | --- | --- | --- | --- | --- | --- | --- | --- | --- | --- | --- | --- | --- | --- | --- | --- | --- | --- |
| total 0 peptides |  |  |  |  |  |  |  |  |  |  |  |  |  |  |  |  |  |  |  |
| --- | --- | --- | --- | --- | --- | --- | --- | --- | --- | --- | --- | --- | --- | --- | --- | --- | --- | --- | --- |

P0CH80.1

back to list

  

| Protein Coverage
| Supporting Peptides
|

Protein Coverage:

Supporting Peptides:

| Peptide | Uniq | -10lgP | Mass | Length | ppm | m/z | z | RT | Fraction | Scan | Source File | Area BuCaMH04\_B15 | #Feature | #Feature BuCaMH04\_B15 | Start | End | PTM | AScore | Found By |
| --- | --- | --- | --- | --- | --- | --- | --- | --- | --- | --- | --- | --- | --- | --- | --- | --- | --- | --- | --- |
| total 0 peptides |  |  |  |  |  |  |  |  |  |  |  |  |  |  |  |  |  |  |  |
| --- | --- | --- | --- | --- | --- | --- | --- | --- | --- | --- | --- | --- | --- | --- | --- | --- | --- | --- | --- |

AAC61318.1

back to list

  

| Protein Coverage
| Supporting Peptides
|

Protein Coverage:

Supporting Peptides:

| Peptide | Uniq | -10lgP | Mass | Length | ppm | m/z | z | RT | Fraction | Scan | Source File | Area BuCaMH04\_B15 | #Feature | #Feature BuCaMH04\_B15 | Start | End | PTM | AScore | Found By |
| --- | --- | --- | --- | --- | --- | --- | --- | --- | --- | --- | --- | --- | --- | --- | --- | --- | --- | --- | --- |
| total 0 peptides |  |  |  |  |  |  |  |  |  |  |  |  |  |  |  |  |  |  |  |
| --- | --- | --- | --- | --- | --- | --- | --- | --- | --- | --- | --- | --- | --- | --- | --- | --- | --- | --- | --- |

O73858.1

back to list

  

| Protein Coverage
| Supporting Peptides
|

Protein Coverage:

Supporting Peptides:

| Peptide | Uniq | -10lgP | Mass | Length | ppm | m/z | z | RT | Fraction | Scan | Source File | Area BuCaMH04\_B15 | #Feature | #Feature BuCaMH04\_B15 | Start | End | PTM | AScore | Found By |
| --- | --- | --- | --- | --- | --- | --- | --- | --- | --- | --- | --- | --- | --- | --- | --- | --- | --- | --- | --- |
| total 0 peptides |  |  |  |  |  |  |  |  |  |  |  |  |  |  |  |  |  |  |  |
| --- | --- | --- | --- | --- | --- | --- | --- | --- | --- | --- | --- | --- | --- | --- | --- | --- | --- | --- | --- |

AAA49386.1

back to list

  

| Protein Coverage
| Supporting Peptides
|

Protein Coverage:

Supporting Peptides:

| Peptide | Uniq | -10lgP | Mass | Length | ppm | m/z | z | RT | Fraction | Scan | Source File | Area BuCaMH04\_B15 | #Feature | #Feature BuCaMH04\_B15 | Start | End | PTM | AScore | Found By |
| --- | --- | --- | --- | --- | --- | --- | --- | --- | --- | --- | --- | --- | --- | --- | --- | --- | --- | --- | --- |
| total 0 peptides |  |  |  |  |  |  |  |  |  |  |  |  |  |  |  |  |  |  |  |
| --- | --- | --- | --- | --- | --- | --- | --- | --- | --- | --- | --- | --- | --- | --- | --- | --- | --- | --- | --- |

P86382.1

back to list

  

| Protein Coverage
| Supporting Peptides
|

Protein Coverage:

Supporting Peptides:

| Peptide | Uniq | -10lgP | Mass | Length | ppm | m/z | z | RT | Fraction | Scan | Source File | Area BuCaMH04\_B15 | #Feature | #Feature BuCaMH04\_B15 | Start | End | PTM | AScore | Found By |
| --- | --- | --- | --- | --- | --- | --- | --- | --- | --- | --- | --- | --- | --- | --- | --- | --- | --- | --- | --- |
| total 0 peptides |  |  |  |  |  |  |  |  |  |  |  |  |  |  |  |  |  |  |  |
| --- | --- | --- | --- | --- | --- | --- | --- | --- | --- | --- | --- | --- | --- | --- | --- | --- | --- | --- | --- |

XP\_026521338.1

back to list

  

| Protein Coverage
| Supporting Peptides
|

Protein Coverage:

Supporting Peptides:

| Peptide | Uniq | -10lgP | Mass | Length | ppm | m/z | z | RT | Fraction | Scan | Source File | Area BuCaMH04\_B15 | #Feature | #Feature BuCaMH04\_B15 | Start | End | PTM | AScore | Found By |
| --- | --- | --- | --- | --- | --- | --- | --- | --- | --- | --- | --- | --- | --- | --- | --- | --- | --- | --- | --- |
| total 0 peptides |  |  |  |  |  |  |  |  |  |  |  |  |  |  |  |  |  |  |  |
| --- | --- | --- | --- | --- | --- | --- | --- | --- | --- | --- | --- | --- | --- | --- | --- | --- | --- | --- | --- |

XP\_026563870.1

back to list

  

| Protein Coverage
| Supporting Peptides
|

Protein Coverage:

Supporting Peptides:

| Peptide | Uniq | -10lgP | Mass | Length | ppm | m/z | z | RT | Fraction | Scan | Source File | Area BuCaMH04\_B15 | #Feature | #Feature BuCaMH04\_B15 | Start | End | PTM | AScore | Found By |
| --- | --- | --- | --- | --- | --- | --- | --- | --- | --- | --- | --- | --- | --- | --- | --- | --- | --- | --- | --- |
| total 0 peptides |  |  |  |  |  |  |  |  |  |  |  |  |  |  |  |  |  |  |  |
| --- | --- | --- | --- | --- | --- | --- | --- | --- | --- | --- | --- | --- | --- | --- | --- | --- | --- | --- | --- |

XP\_013918540.1

back to list

  

| Protein Coverage
| Supporting Peptides
|

Protein Coverage:

Supporting Peptides:

| Peptide | Uniq | -10lgP | Mass | Length | ppm | m/z | z | RT | Fraction | Scan | Source File | Area BuCaMH04\_B15 | #Feature | #Feature BuCaMH04\_B15 | Start | End | PTM | AScore | Found By |
| --- | --- | --- | --- | --- | --- | --- | --- | --- | --- | --- | --- | --- | --- | --- | --- | --- | --- | --- | --- |
| total 0 peptides |  |  |  |  |  |  |  |  |  |  |  |  |  |  |  |  |  |  |  |
| --- | --- | --- | --- | --- | --- | --- | --- | --- | --- | --- | --- | --- | --- | --- | --- | --- | --- | --- | --- |

XP\_015675982.1

back to list

  

| Protein Coverage
| Supporting Peptides
|

Protein Coverage:

Supporting Peptides:

| Peptide | Uniq | -10lgP | Mass | Length | ppm | m/z | z | RT | Fraction | Scan | Source File | Area BuCaMH04\_B15 | #Feature | #Feature BuCaMH04\_B15 | Start | End | PTM | AScore | Found By |
| --- | --- | --- | --- | --- | --- | --- | --- | --- | --- | --- | --- | --- | --- | --- | --- | --- | --- | --- | --- |
| total 0 peptides |  |  |  |  |  |  |  |  |  |  |  |  |  |  |  |  |  |  |  |
| --- | --- | --- | --- | --- | --- | --- | --- | --- | --- | --- | --- | --- | --- | --- | --- | --- | --- | --- | --- |

XP\_034289619.1

back to list

  

| Protein Coverage
| Supporting Peptides
|

Protein Coverage:

Supporting Peptides:

| Peptide | Uniq | -10lgP | Mass | Length | ppm | m/z | z | RT | Fraction | Scan | Source File | Area BuCaMH04\_B15 | #Feature | #Feature BuCaMH04\_B15 | Start | End | PTM | AScore | Found By |
| --- | --- | --- | --- | --- | --- | --- | --- | --- | --- | --- | --- | --- | --- | --- | --- | --- | --- | --- | --- |
| total 0 peptides |  |  |  |  |  |  |  |  |  |  |  |  |  |  |  |  |  |  |  |
| --- | --- | --- | --- | --- | --- | --- | --- | --- | --- | --- | --- | --- | --- | --- | --- | --- | --- | --- | --- |

XP\_032073432.1

back to list

  

| Protein Coverage
| Supporting Peptides
|

Protein Coverage:

Supporting Peptides:

| Peptide | Uniq | -10lgP | Mass | Length | ppm | m/z | z | RT | Fraction | Scan | Source File | Area BuCaMH04\_B15 | #Feature | #Feature BuCaMH04\_B15 | Start | End | PTM | AScore | Found By |
| --- | --- | --- | --- | --- | --- | --- | --- | --- | --- | --- | --- | --- | --- | --- | --- | --- | --- | --- | --- |
| total 0 peptides |  |  |  |  |  |  |  |  |  |  |  |  |  |  |  |  |  |  |  |
| --- | --- | --- | --- | --- | --- | --- | --- | --- | --- | --- | --- | --- | --- | --- | --- | --- | --- | --- | --- |

P86539.1

back to list

  

| Protein Coverage
| Supporting Peptides
|

Protein Coverage:

Supporting Peptides:

| Peptide | Uniq | -10lgP | Mass | Length | ppm | m/z | z | RT | Fraction | Scan | Source File | Area BuCaMH04\_B15 | #Feature | #Feature BuCaMH04\_B15 | Start | End | PTM | AScore | Found By |
| --- | --- | --- | --- | --- | --- | --- | --- | --- | --- | --- | --- | --- | --- | --- | --- | --- | --- | --- | --- |
| total 0 peptides |  |  |  |  |  |  |  |  |  |  |  |  |  |  |  |  |  |  |  |
| --- | --- | --- | --- | --- | --- | --- | --- | --- | --- | --- | --- | --- | --- | --- | --- | --- | --- | --- | --- |

6RC7

back to list

  

| Protein Coverage
| Supporting Peptides
|

Protein Coverage:

Supporting Peptides:

| Peptide | Uniq | -10lgP | Mass | Length | ppm | m/z | z | RT | Fraction | Scan | Source File | Area BuCaMH04\_B15 | #Feature | #Feature BuCaMH04\_B15 | Start | End | PTM | AScore | Found By |
| --- | --- | --- | --- | --- | --- | --- | --- | --- | --- | --- | --- | --- | --- | --- | --- | --- | --- | --- | --- |
| total 0 peptides |  |  |  |  |  |  |  |  |  |  |  |  |  |  |  |  |  |  |  |
| --- | --- | --- | --- | --- | --- | --- | --- | --- | --- | --- | --- | --- | --- | --- | --- | --- | --- | --- | --- |

P01445.1

back to list

  

| Protein Coverage
| Supporting Peptides
|

Protein Coverage:

Supporting Peptides:

| Peptide | Uniq | -10lgP | Mass | Length | ppm | m/z | z | RT | Fraction | Scan | Source File | Area BuCaMH04\_B15 | #Feature | #Feature BuCaMH04\_B15 | Start | End | PTM | AScore | Found By |
| --- | --- | --- | --- | --- | --- | --- | --- | --- | --- | --- | --- | --- | --- | --- | --- | --- | --- | --- | --- |
| total 0 peptides |  |  |  |  |  |  |  |  |  |  |  |  |  |  |  |  |  |  |  |
| --- | --- | --- | --- | --- | --- | --- | --- | --- | --- | --- | --- | --- | --- | --- | --- | --- | --- | --- | --- |

1CDT

back to list

  

| Protein Coverage
| Supporting Peptides
|

Protein Coverage:

Supporting Peptides:

| Peptide | Uniq | -10lgP | Mass | Length | ppm | m/z | z | RT | Fraction | Scan | Source File | Area BuCaMH04\_B15 | #Feature | #Feature BuCaMH04\_B15 | Start | End | PTM | AScore | Found By |
| --- | --- | --- | --- | --- | --- | --- | --- | --- | --- | --- | --- | --- | --- | --- | --- | --- | --- | --- | --- |
| total 0 peptides |  |  |  |  |  |  |  |  |  |  |  |  |  |  |  |  |  |  |  |
| --- | --- | --- | --- | --- | --- | --- | --- | --- | --- | --- | --- | --- | --- | --- | --- | --- | --- | --- | --- |

P01446.1

back to list

  

| Protein Coverage
| Supporting Peptides
|

Protein Coverage:

Supporting Peptides:

| Peptide | Uniq | -10lgP | Mass | Length | ppm | m/z | z | RT | Fraction | Scan | Source File | Area BuCaMH04\_B15 | #Feature | #Feature BuCaMH04\_B15 | Start | End | PTM | AScore | Found By |
| --- | --- | --- | --- | --- | --- | --- | --- | --- | --- | --- | --- | --- | --- | --- | --- | --- | --- | --- | --- |
| total 0 peptides |  |  |  |  |  |  |  |  |  |  |  |  |  |  |  |  |  |  |  |
| --- | --- | --- | --- | --- | --- | --- | --- | --- | --- | --- | --- | --- | --- | --- | --- | --- | --- | --- | --- |

P24780.1

back to list

  

| Protein Coverage
| Supporting Peptides
|

Protein Coverage:

Supporting Peptides:

| Peptide | Uniq | -10lgP | Mass | Length | ppm | m/z | z | RT | Fraction | Scan | Source File | Area BuCaMH04\_B15 | #Feature | #Feature BuCaMH04\_B15 | Start | End | PTM | AScore | Found By |
| --- | --- | --- | --- | --- | --- | --- | --- | --- | --- | --- | --- | --- | --- | --- | --- | --- | --- | --- | --- |
| total 0 peptides |  |  |  |  |  |  |  |  |  |  |  |  |  |  |  |  |  |  |  |
| --- | --- | --- | --- | --- | --- | --- | --- | --- | --- | --- | --- | --- | --- | --- | --- | --- | --- | --- | --- |

P01452.1

back to list

  

| Protein Coverage
| Supporting Peptides
|

Protein Coverage:

Supporting Peptides:

| Peptide | Uniq | -10lgP | Mass | Length | ppm | m/z | z | RT | Fraction | Scan | Source File | Area BuCaMH04\_B15 | #Feature | #Feature BuCaMH04\_B15 | Start | End | PTM | AScore | Found By |
| --- | --- | --- | --- | --- | --- | --- | --- | --- | --- | --- | --- | --- | --- | --- | --- | --- | --- | --- | --- |
| total 0 peptides |  |  |  |  |  |  |  |  |  |  |  |  |  |  |  |  |  |  |  |
| --- | --- | --- | --- | --- | --- | --- | --- | --- | --- | --- | --- | --- | --- | --- | --- | --- | --- | --- | --- |

P01447.1

back to list

  

| Protein Coverage
| Supporting Peptides
|

Protein Coverage:

Supporting Peptides:

| Peptide | Uniq | -10lgP | Mass | Length | ppm | m/z | z | RT | Fraction | Scan | Source File | Area BuCaMH04\_B15 | #Feature | #Feature BuCaMH04\_B15 | Start | End | PTM | AScore | Found By |
| --- | --- | --- | --- | --- | --- | --- | --- | --- | --- | --- | --- | --- | --- | --- | --- | --- | --- | --- | --- |
| total 0 peptides |  |  |  |  |  |  |  |  |  |  |  |  |  |  |  |  |  |  |  |
| --- | --- | --- | --- | --- | --- | --- | --- | --- | --- | --- | --- | --- | --- | --- | --- | --- | --- | --- | --- |

P24779.1

back to list

  

| Protein Coverage
| Supporting Peptides
|

Protein Coverage:

Supporting Peptides:

| Peptide | Uniq | -10lgP | Mass | Length | ppm | m/z | z | RT | Fraction | Scan | Source File | Area BuCaMH04\_B15 | #Feature | #Feature BuCaMH04\_B15 | Start | End | PTM | AScore | Found By |
| --- | --- | --- | --- | --- | --- | --- | --- | --- | --- | --- | --- | --- | --- | --- | --- | --- | --- | --- | --- |
| total 0 peptides |  |  |  |  |  |  |  |  |  |  |  |  |  |  |  |  |  |  |  |
| --- | --- | --- | --- | --- | --- | --- | --- | --- | --- | --- | --- | --- | --- | --- | --- | --- | --- | --- | --- |

P86540.2

back to list

  

| Protein Coverage
| Supporting Peptides
|

Protein Coverage:

Supporting Peptides:

| Peptide | Uniq | -10lgP | Mass | Length | ppm | m/z | z | RT | Fraction | Scan | Source File | Area BuCaMH04\_B15 | #Feature | #Feature BuCaMH04\_B15 | Start | End | PTM | AScore | Found By |
| --- | --- | --- | --- | --- | --- | --- | --- | --- | --- | --- | --- | --- | --- | --- | --- | --- | --- | --- | --- |
| total 0 peptides |  |  |  |  |  |  |  |  |  |  |  |  |  |  |  |  |  |  |  |
| --- | --- | --- | --- | --- | --- | --- | --- | --- | --- | --- | --- | --- | --- | --- | --- | --- | --- | --- | --- |

AAB35381.1

back to list

  

| Protein Coverage
| Supporting Peptides
|

Protein Coverage:

Supporting Peptides:

| Peptide | Uniq | -10lgP | Mass | Length | ppm | m/z | z | RT | Fraction | Scan | Source File | Area BuCaMH04\_B15 | #Feature | #Feature BuCaMH04\_B15 | Start | End | PTM | AScore | Found By |
| --- | --- | --- | --- | --- | --- | --- | --- | --- | --- | --- | --- | --- | --- | --- | --- | --- | --- | --- | --- |
| total 0 peptides |  |  |  |  |  |  |  |  |  |  |  |  |  |  |  |  |  |  |  |
| --- | --- | --- | --- | --- | --- | --- | --- | --- | --- | --- | --- | --- | --- | --- | --- | --- | --- | --- | --- |

BAU24669.1

back to list

  

| Protein Coverage
| Supporting Peptides
|

Protein Coverage:

Supporting Peptides:

| Peptide | Uniq | -10lgP | Mass | Length | ppm | m/z | z | RT | Fraction | Scan | Source File | Area BuCaMH04\_B15 | #Feature | #Feature BuCaMH04\_B15 | Start | End | PTM | AScore | Found By |
| --- | --- | --- | --- | --- | --- | --- | --- | --- | --- | --- | --- | --- | --- | --- | --- | --- | --- | --- | --- |
| total 0 peptides |  |  |  |  |  |  |  |  |  |  |  |  |  |  |  |  |  |  |  |
| --- | --- | --- | --- | --- | --- | --- | --- | --- | --- | --- | --- | --- | --- | --- | --- | --- | --- | --- | --- |

BAU24676.1

back to list

  

| Protein Coverage
| Supporting Peptides
|

Protein Coverage:

Supporting Peptides:

| Peptide | Uniq | -10lgP | Mass | Length | ppm | m/z | z | RT | Fraction | Scan | Source File | Area BuCaMH04\_B15 | #Feature | #Feature BuCaMH04\_B15 | Start | End | PTM | AScore | Found By |
| --- | --- | --- | --- | --- | --- | --- | --- | --- | --- | --- | --- | --- | --- | --- | --- | --- | --- | --- | --- |
| total 0 peptides |  |  |  |  |  |  |  |  |  |  |  |  |  |  |  |  |  |  |  |
| --- | --- | --- | --- | --- | --- | --- | --- | --- | --- | --- | --- | --- | --- | --- | --- | --- | --- | --- | --- |

BAU24671.1

back to list

  

| Protein Coverage
| Supporting Peptides
|

Protein Coverage:

Supporting Peptides:

| Peptide | Uniq | -10lgP | Mass | Length | ppm | m/z | z | RT | Fraction | Scan | Source File | Area BuCaMH04\_B15 | #Feature | #Feature BuCaMH04\_B15 | Start | End | PTM | AScore | Found By |
| --- | --- | --- | --- | --- | --- | --- | --- | --- | --- | --- | --- | --- | --- | --- | --- | --- | --- | --- | --- |
| total 0 peptides |  |  |  |  |  |  |  |  |  |  |  |  |  |  |  |  |  |  |  |
| --- | --- | --- | --- | --- | --- | --- | --- | --- | --- | --- | --- | --- | --- | --- | --- | --- | --- | --- | --- |

BAU24670.1

back to list

  

| Protein Coverage
| Supporting Peptides
|

Protein Coverage:

Supporting Peptides:

| Peptide | Uniq | -10lgP | Mass | Length | ppm | m/z | z | RT | Fraction | Scan | Source File | Area BuCaMH04\_B15 | #Feature | #Feature BuCaMH04\_B15 | Start | End | PTM | AScore | Found By |
| --- | --- | --- | --- | --- | --- | --- | --- | --- | --- | --- | --- | --- | --- | --- | --- | --- | --- | --- | --- |
| total 0 peptides |  |  |  |  |  |  |  |  |  |  |  |  |  |  |  |  |  |  |  |
| --- | --- | --- | --- | --- | --- | --- | --- | --- | --- | --- | --- | --- | --- | --- | --- | --- | --- | --- | --- |

BAU24673.1

back to list

  

| Protein Coverage
| Supporting Peptides
|

Protein Coverage:

Supporting Peptides:

| Peptide | Uniq | -10lgP | Mass | Length | ppm | m/z | z | RT | Fraction | Scan | Source File | Area BuCaMH04\_B15 | #Feature | #Feature BuCaMH04\_B15 | Start | End | PTM | AScore | Found By |
| --- | --- | --- | --- | --- | --- | --- | --- | --- | --- | --- | --- | --- | --- | --- | --- | --- | --- | --- | --- |
| total 0 peptides |  |  |  |  |  |  |  |  |  |  |  |  |  |  |  |  |  |  |  |
| --- | --- | --- | --- | --- | --- | --- | --- | --- | --- | --- | --- | --- | --- | --- | --- | --- | --- | --- | --- |

BAU24667.1

back to list

  

| Protein Coverage
| Supporting Peptides
|

Protein Coverage:

Supporting Peptides:

| Peptide | Uniq | -10lgP | Mass | Length | ppm | m/z | z | RT | Fraction | Scan | Source File | Area BuCaMH04\_B15 | #Feature | #Feature BuCaMH04\_B15 | Start | End | PTM | AScore | Found By |
| --- | --- | --- | --- | --- | --- | --- | --- | --- | --- | --- | --- | --- | --- | --- | --- | --- | --- | --- | --- |
| total 0 peptides |  |  |  |  |  |  |  |  |  |  |  |  |  |  |  |  |  |  |  |
| --- | --- | --- | --- | --- | --- | --- | --- | --- | --- | --- | --- | --- | --- | --- | --- | --- | --- | --- | --- |

BAU24668.1

back to list

  

| Protein Coverage
| Supporting Peptides
|

Protein Coverage:

Supporting Peptides:

| Peptide | Uniq | -10lgP | Mass | Length | ppm | m/z | z | RT | Fraction | Scan | Source File | Area BuCaMH04\_B15 | #Feature | #Feature BuCaMH04\_B15 | Start | End | PTM | AScore | Found By |
| --- | --- | --- | --- | --- | --- | --- | --- | --- | --- | --- | --- | --- | --- | --- | --- | --- | --- | --- | --- |
| total 0 peptides |  |  |  |  |  |  |  |  |  |  |  |  |  |  |  |  |  |  |  |
| --- | --- | --- | --- | --- | --- | --- | --- | --- | --- | --- | --- | --- | --- | --- | --- | --- | --- | --- | --- |

BAU24672.1

back to list

  

| Protein Coverage
| Supporting Peptides
|

Protein Coverage:

Supporting Peptides:

| Peptide | Uniq | -10lgP | Mass | Length | ppm | m/z | z | RT | Fraction | Scan | Source File | Area BuCaMH04\_B15 | #Feature | #Feature BuCaMH04\_B15 | Start | End | PTM | AScore | Found By |
| --- | --- | --- | --- | --- | --- | --- | --- | --- | --- | --- | --- | --- | --- | --- | --- | --- | --- | --- | --- |
| total 0 peptides |  |  |  |  |  |  |  |  |  |  |  |  |  |  |  |  |  |  |  |
| --- | --- | --- | --- | --- | --- | --- | --- | --- | --- | --- | --- | --- | --- | --- | --- | --- | --- | --- | --- |

BAU24675.1

back to list

  

| Protein Coverage
| Supporting Peptides
|

Protein Coverage:

Supporting Peptides:

| Peptide | Uniq | -10lgP | Mass | Length | ppm | m/z | z | RT | Fraction | Scan | Source File | Area BuCaMH04\_B15 | #Feature | #Feature BuCaMH04\_B15 | Start | End | PTM | AScore | Found By |
| --- | --- | --- | --- | --- | --- | --- | --- | --- | --- | --- | --- | --- | --- | --- | --- | --- | --- | --- | --- |
| total 0 peptides |  |  |  |  |  |  |  |  |  |  |  |  |  |  |  |  |  |  |  |
| --- | --- | --- | --- | --- | --- | --- | --- | --- | --- | --- | --- | --- | --- | --- | --- | --- | --- | --- | --- |

XP\_007419884.1

back to list

  

| Protein Coverage
| Supporting Peptides
|

Protein Coverage:

Supporting Peptides:

| Peptide | Uniq | -10lgP | Mass | Length | ppm | m/z | z | RT | Fraction | Scan | Source File | Area BuCaMH04\_B15 | #Feature | #Feature BuCaMH04\_B15 | Start | End | PTM | AScore | Found By |
| --- | --- | --- | --- | --- | --- | --- | --- | --- | --- | --- | --- | --- | --- | --- | --- | --- | --- | --- | --- |
| total 0 peptides |  |  |  |  |  |  |  |  |  |  |  |  |  |  |  |  |  |  |  |
| --- | --- | --- | --- | --- | --- | --- | --- | --- | --- | --- | --- | --- | --- | --- | --- | --- | --- | --- | --- |

XP\_025020898.1

back to list

  

| Protein Coverage
| Supporting Peptides
|

Protein Coverage:

Supporting Peptides:

| Peptide | Uniq | -10lgP | Mass | Length | ppm | m/z | z | RT | Fraction | Scan | Source File | Area BuCaMH04\_B15 | #Feature | #Feature BuCaMH04\_B15 | Start | End | PTM | AScore | Found By |
| --- | --- | --- | --- | --- | --- | --- | --- | --- | --- | --- | --- | --- | --- | --- | --- | --- | --- | --- | --- |
| total 0 peptides |  |  |  |  |  |  |  |  |  |  |  |  |  |  |  |  |  |  |  |
| --- | --- | --- | --- | --- | --- | --- | --- | --- | --- | --- | --- | --- | --- | --- | --- | --- | --- | --- | --- |

XP\_025020897.1

back to list

  

| Protein Coverage
| Supporting Peptides
|

Protein Coverage:

Supporting Peptides:

| Peptide | Uniq | -10lgP | Mass | Length | ppm | m/z | z | RT | Fraction | Scan | Source File | Area BuCaMH04\_B15 | #Feature | #Feature BuCaMH04\_B15 | Start | End | PTM | AScore | Found By |
| --- | --- | --- | --- | --- | --- | --- | --- | --- | --- | --- | --- | --- | --- | --- | --- | --- | --- | --- | --- |
| total 0 peptides |  |  |  |  |  |  |  |  |  |  |  |  |  |  |  |  |  |  |  |
| --- | --- | --- | --- | --- | --- | --- | --- | --- | --- | --- | --- | --- | --- | --- | --- | --- | --- | --- | --- |

XP\_025020896.1

back to list

  

| Protein Coverage
| Supporting Peptides
|

Protein Coverage:

Supporting Peptides:

| Peptide | Uniq | -10lgP | Mass | Length | ppm | m/z | z | RT | Fraction | Scan | Source File | Area BuCaMH04\_B15 | #Feature | #Feature BuCaMH04\_B15 | Start | End | PTM | AScore | Found By |
| --- | --- | --- | --- | --- | --- | --- | --- | --- | --- | --- | --- | --- | --- | --- | --- | --- | --- | --- | --- |
| total 0 peptides |  |  |  |  |  |  |  |  |  |  |  |  |  |  |  |  |  |  |  |
| --- | --- | --- | --- | --- | --- | --- | --- | --- | --- | --- | --- | --- | --- | --- | --- | --- | --- | --- | --- |

XP\_013925438.1

back to list

  

| Protein Coverage
| Supporting Peptides
|

Protein Coverage:

Supporting Peptides:

| Peptide | Uniq | -10lgP | Mass | Length | ppm | m/z | z | RT | Fraction | Scan | Source File | Area BuCaMH04\_B15 | #Feature | #Feature BuCaMH04\_B15 | Start | End | PTM | AScore | Found By |
| --- | --- | --- | --- | --- | --- | --- | --- | --- | --- | --- | --- | --- | --- | --- | --- | --- | --- | --- | --- |
| total 0 peptides |  |  |  |  |  |  |  |  |  |  |  |  |  |  |  |  |  |  |  |
| --- | --- | --- | --- | --- | --- | --- | --- | --- | --- | --- | --- | --- | --- | --- | --- | --- | --- | --- | --- |

XP\_013925437.1

back to list

  

| Protein Coverage
| Supporting Peptides
|

Protein Coverage:

Supporting Peptides:

| Peptide | Uniq | -10lgP | Mass | Length | ppm | m/z | z | RT | Fraction | Scan | Source File | Area BuCaMH04\_B15 | #Feature | #Feature BuCaMH04\_B15 | Start | End | PTM | AScore | Found By |
| --- | --- | --- | --- | --- | --- | --- | --- | --- | --- | --- | --- | --- | --- | --- | --- | --- | --- | --- | --- |
| total 0 peptides |  |  |  |  |  |  |  |  |  |  |  |  |  |  |  |  |  |  |  |
| --- | --- | --- | --- | --- | --- | --- | --- | --- | --- | --- | --- | --- | --- | --- | --- | --- | --- | --- | --- |

XP\_032078666.1

back to list

  

| Protein Coverage
| Supporting Peptides
|

Protein Coverage:

Supporting Peptides:

| Peptide | Uniq | -10lgP | Mass | Length | ppm | m/z | z | RT | Fraction | Scan | Source File | Area BuCaMH04\_B15 | #Feature | #Feature BuCaMH04\_B15 | Start | End | PTM | AScore | Found By |
| --- | --- | --- | --- | --- | --- | --- | --- | --- | --- | --- | --- | --- | --- | --- | --- | --- | --- | --- | --- |
| total 0 peptides |  |  |  |  |  |  |  |  |  |  |  |  |  |  |  |  |  |  |  |
| --- | --- | --- | --- | --- | --- | --- | --- | --- | --- | --- | --- | --- | --- | --- | --- | --- | --- | --- | --- |

XP\_032078662.1

back to list

  

| Protein Coverage
| Supporting Peptides
|

Protein Coverage:

Supporting Peptides:

| Peptide | Uniq | -10lgP | Mass | Length | ppm | m/z | z | RT | Fraction | Scan | Source File | Area BuCaMH04\_B15 | #Feature | #Feature BuCaMH04\_B15 | Start | End | PTM | AScore | Found By |
| --- | --- | --- | --- | --- | --- | --- | --- | --- | --- | --- | --- | --- | --- | --- | --- | --- | --- | --- | --- |
| total 0 peptides |  |  |  |  |  |  |  |  |  |  |  |  |  |  |  |  |  |  |  |
| --- | --- | --- | --- | --- | --- | --- | --- | --- | --- | --- | --- | --- | --- | --- | --- | --- | --- | --- | --- |

XP\_032078664.1

back to list

  

| Protein Coverage
| Supporting Peptides
|

Protein Coverage:

Supporting Peptides:

| Peptide | Uniq | -10lgP | Mass | Length | ppm | m/z | z | RT | Fraction | Scan | Source File | Area BuCaMH04\_B15 | #Feature | #Feature BuCaMH04\_B15 | Start | End | PTM | AScore | Found By |
| --- | --- | --- | --- | --- | --- | --- | --- | --- | --- | --- | --- | --- | --- | --- | --- | --- | --- | --- | --- |
| total 0 peptides |  |  |  |  |  |  |  |  |  |  |  |  |  |  |  |  |  |  |  |
| --- | --- | --- | --- | --- | --- | --- | --- | --- | --- | --- | --- | --- | --- | --- | --- | --- | --- | --- | --- |

XP\_032078665.1

back to list

  

| Protein Coverage
| Supporting Peptides
|

Protein Coverage:

Supporting Peptides:

| Peptide | Uniq | -10lgP | Mass | Length | ppm | m/z | z | RT | Fraction | Scan | Source File | Area BuCaMH04\_B15 | #Feature | #Feature BuCaMH04\_B15 | Start | End | PTM | AScore | Found By |
| --- | --- | --- | --- | --- | --- | --- | --- | --- | --- | --- | --- | --- | --- | --- | --- | --- | --- | --- | --- |
| total 0 peptides |  |  |  |  |  |  |  |  |  |  |  |  |  |  |  |  |  |  |  |
| --- | --- | --- | --- | --- | --- | --- | --- | --- | --- | --- | --- | --- | --- | --- | --- | --- | --- | --- | --- |

XP\_032078663.1

back to list

  

| Protein Coverage
| Supporting Peptides
|

Protein Coverage:

Supporting Peptides:

| Peptide | Uniq | -10lgP | Mass | Length | ppm | m/z | z | RT | Fraction | Scan | Source File | Area BuCaMH04\_B15 | #Feature | #Feature BuCaMH04\_B15 | Start | End | PTM | AScore | Found By |
| --- | --- | --- | --- | --- | --- | --- | --- | --- | --- | --- | --- | --- | --- | --- | --- | --- | --- | --- | --- |
| total 0 peptides |  |  |  |  |  |  |  |  |  |  |  |  |  |  |  |  |  |  |  |
| --- | --- | --- | --- | --- | --- | --- | --- | --- | --- | --- | --- | --- | --- | --- | --- | --- | --- | --- | --- |

XP\_026551948.1

back to list

  

| Protein Coverage
| Supporting Peptides
|

Protein Coverage:

Supporting Peptides:

| Peptide | Uniq | -10lgP | Mass | Length | ppm | m/z | z | RT | Fraction | Scan | Source File | Area BuCaMH04\_B15 | #Feature | #Feature BuCaMH04\_B15 | Start | End | PTM | AScore | Found By |
| --- | --- | --- | --- | --- | --- | --- | --- | --- | --- | --- | --- | --- | --- | --- | --- | --- | --- | --- | --- |
| total 0 peptides |  |  |  |  |  |  |  |  |  |  |  |  |  |  |  |  |  |  |  |
| --- | --- | --- | --- | --- | --- | --- | --- | --- | --- | --- | --- | --- | --- | --- | --- | --- | --- | --- | --- |

XP\_026541026.1

back to list

  

| Protein Coverage
| Supporting Peptides
|

Protein Coverage:

Supporting Peptides:

| Peptide | Uniq | -10lgP | Mass | Length | ppm | m/z | z | RT | Fraction | Scan | Source File | Area BuCaMH04\_B15 | #Feature | #Feature BuCaMH04\_B15 | Start | End | PTM | AScore | Found By |
| --- | --- | --- | --- | --- | --- | --- | --- | --- | --- | --- | --- | --- | --- | --- | --- | --- | --- | --- | --- |
| total 0 peptides |  |  |  |  |  |  |  |  |  |  |  |  |  |  |  |  |  |  |  |
| --- | --- | --- | --- | --- | --- | --- | --- | --- | --- | --- | --- | --- | --- | --- | --- | --- | --- | --- | --- |

ETE67277.1

back to list

  

| Protein Coverage
| Supporting Peptides
|

Protein Coverage:

Supporting Peptides:

| Peptide | Uniq | -10lgP | Mass | Length | ppm | m/z | z | RT | Fraction | Scan | Source File | Area BuCaMH04\_B15 | #Feature | #Feature BuCaMH04\_B15 | Start | End | PTM | AScore | Found By |
| --- | --- | --- | --- | --- | --- | --- | --- | --- | --- | --- | --- | --- | --- | --- | --- | --- | --- | --- | --- |
| total 0 peptides |  |  |  |  |  |  |  |  |  |  |  |  |  |  |  |  |  |  |  |
| --- | --- | --- | --- | --- | --- | --- | --- | --- | --- | --- | --- | --- | --- | --- | --- | --- | --- | --- | --- |

XP\_026535088.1

back to list

  

| Protein Coverage
| Supporting Peptides
|

Protein Coverage:

Supporting Peptides:

| Peptide | Uniq | -10lgP | Mass | Length | ppm | m/z | z | RT | Fraction | Scan | Source File | Area BuCaMH04\_B15 | #Feature | #Feature BuCaMH04\_B15 | Start | End | PTM | AScore | Found By |
| --- | --- | --- | --- | --- | --- | --- | --- | --- | --- | --- | --- | --- | --- | --- | --- | --- | --- | --- | --- |
| total 0 peptides |  |  |  |  |  |  |  |  |  |  |  |  |  |  |  |  |  |  |  |
| --- | --- | --- | --- | --- | --- | --- | --- | --- | --- | --- | --- | --- | --- | --- | --- | --- | --- | --- | --- |

ETE71292.1

back to list

  

| Protein Coverage
| Supporting Peptides
|

Protein Coverage:

Supporting Peptides:

| Peptide | Uniq | -10lgP | Mass | Length | ppm | m/z | z | RT | Fraction | Scan | Source File | Area BuCaMH04\_B15 | #Feature | #Feature BuCaMH04\_B15 | Start | End | PTM | AScore | Found By |
| --- | --- | --- | --- | --- | --- | --- | --- | --- | --- | --- | --- | --- | --- | --- | --- | --- | --- | --- | --- |
| total 0 peptides |  |  |  |  |  |  |  |  |  |  |  |  |  |  |  |  |  |  |  |
| --- | --- | --- | --- | --- | --- | --- | --- | --- | --- | --- | --- | --- | --- | --- | --- | --- | --- | --- | --- |

XP\_034292893.1

back to list

  

| Protein Coverage
| Supporting Peptides
|

Protein Coverage:

Supporting Peptides:

| Peptide | Uniq | -10lgP | Mass | Length | ppm | m/z | z | RT | Fraction | Scan | Source File | Area BuCaMH04\_B15 | #Feature | #Feature BuCaMH04\_B15 | Start | End | PTM | AScore | Found By |
| --- | --- | --- | --- | --- | --- | --- | --- | --- | --- | --- | --- | --- | --- | --- | --- | --- | --- | --- | --- |
| total 0 peptides |  |  |  |  |  |  |  |  |  |  |  |  |  |  |  |  |  |  |  |
| --- | --- | --- | --- | --- | --- | --- | --- | --- | --- | --- | --- | --- | --- | --- | --- | --- | --- | --- | --- |

ETE72045.1

back to list

  

| Protein Coverage
| Supporting Peptides
|

Protein Coverage:

Supporting Peptides:

| Peptide | Uniq | -10lgP | Mass | Length | ppm | m/z | z | RT | Fraction | Scan | Source File | Area BuCaMH04\_B15 | #Feature | #Feature BuCaMH04\_B15 | Start | End | PTM | AScore | Found By |
| --- | --- | --- | --- | --- | --- | --- | --- | --- | --- | --- | --- | --- | --- | --- | --- | --- | --- | --- | --- |
| total 0 peptides |  |  |  |  |  |  |  |  |  |  |  |  |  |  |  |  |  |  |  |
| --- | --- | --- | --- | --- | --- | --- | --- | --- | --- | --- | --- | --- | --- | --- | --- | --- | --- | --- | --- |

Peptide List

  
  

---

Prepared with PEAKS ™ (bioinfor.com)
