# Supplementary material for: A Wolf in Another Wolf’s Clothing: Post-Genomic Regulation Dictates Venom Profiles of Medically-Important Cryptic Kraits in India
Source: Toxins (Basel). 2021 Jan 19;13(1):69. doi: 10.3390/toxins13010069 (PMC7832344; doi:10.3390/toxins13010069)
Supplement: Supplementary file 1 [file toxins-13-00069-s001.zip › File S3.zip!/B. romulusi/B. romulusi.html]

proteins


Summary

  

# 1. Notes

# 2. Result Statistics

**Figure 1.**
False discovery rate (FDR) curve. X axis is the number of peptide-spectrum matches (PSM) being kept. Y axis is the corresponding FDR.

  


  

**Figure 2.**
PSM score distribution.  **(a)**
Distribution of PEAKS peptide score;  **(b)**
Scatterplot of PEAKS peptide score versus precursor mass error.

**(a)**

**(b)**

**Table 1.**
Statistics of data.


|  |  |  |  |  |  |  |  |  |  |  |  |
| --- | --- | --- | --- | --- | --- | --- | --- | --- | --- | --- | --- |
|  | #Scans | | #Features | Identified | | | #Peptides | #Sequences | #Proteins^\*^ | | |
|  | MS1 | MS2 |  | #PSMs | #Scans | #Features |  |  | Groups | All | Top |
| Total | 12449 | 80019 | 113530 | 4185 | 3776 | 14221 | 625 | 601 | 82 | 590 | 347 |
| BRom | 12449 | 80019 | 113530 | 4185 | 3776 | 14221 | 625 | 601 | 82 | 590 | 347 |

^\* proteins with significant peptides are used in counts.^

**Figure 3.**
Sample overlap for Proteins and Peptides (up to 8 samples).  **(a)**
All Proteins;  **(b)**
Top Proteins;  **(c)**
Peptides;

**(a)**

**Not applicable to only one sample**

**(b)**

**Not applicable to only one sample**

**(c)**

**Not applicable to only one sample**

**Figure 4.**
Distribution of peptide feature detection.  **(a)**
Feature m/z distribution;  **(b)**
Feature RT distribution.

**(a)**

**(b)**

**Figure 5.**
Distribution of identified peptide features.  **(a)**
Feature abundance distribution;  **(b)**
 *De novo*
sequencing validation.

**(a)**

**(b)**

**Table 2.**
Result filtration parameters.

|  |  |
| --- | --- |
| Peptide -10lgP | ≥39.2 |
| PTM Ascore | ≥0 |
| Protein -10lgP | ≥20 |
| Proteins unique peptides | ≥1 |
| De novo score(%) | ≥50% |


**Table 3.**
Statistics of filtered result.

|  |  |
| --- | --- |
| FDR (Peptide-Spectrum Matches) | 0.1% |
| FDR (Peptide Sequences) | 0.6% |
| FDR (Protein Group) | 4.9% |
| De Novo Only Spectra | 29088 |

**Table 4.**
PTM profile.

| Name | ∆Mass | Position | #PSM | -10lgP | Abundance | AScore || Carbamidomethyl | 57.02 | C | 3171 | 97.96 | 1.64E8 | 1000.00 |
| Oxidation | 15.99 | M | 151 | 82.39 | 1.29E8 | 1000.00 |

# 3. Experiment Control

**Figure 6.**
Precursor mass error of peptide-spectrum matches (PSM) in filtered result.  **(a)**
Distribution of precursor mass error in ppm;  **(b)**
Scatterplot of precursor m/z versus precursor mass error in ppm.

**(a)**

**(b)**

**Table 5.**
Number of identified peptides in each sample by the number of missed cleavages.

|  |  |  |  |  |  |
| --- | --- | --- | --- | --- | --- |
| Missed Cleavages | 0 | 1 | 2 | 3 | 4+ |
| BRom | 231 | 233 | 161 | 0 | 0 |

# 4. Other Information

**Table 6.**
Search parameters.

Search Engine Name: PEAKS  
Parent Mass Error Tolerance: 10.0 ppm  
Fragment Mass Error Tolerance: 0.6 Da  
Precursor Mass Search Type: monoisotopic  
Enzyme: Trypsin  
Max Missed Cleavages: 2  
Digest Mode: Semispecific  
Fixed Modifications:   
  Carbamidomethylation: 57.02  
Variable Modifications:   
  Oxidation (M): 15.99  
Max Variable PTM Per Peptide: 3  
Database: NCBI\_Serpentes\_BRom  
Taxon: All  
Contaminant Database: cRAP\_contaminants  
Searched Entry: 410203  
FDR Estimation: Enabled  
Merge Options: no merge  
Precursor Options: corrected  
Charge Options: no correction  
Filter Charge: 2 - 8  
Process: true  
Associate chimera: yes

**Table 7.**
Instrument parameters.

Fractions: 01122020\_RID\_2209\_BuCaKA02.raw  
Ion Source: ESI(nano-spray)  
Fragmentation Mode: CID, CAD(y and b ions)  
MS Scan Mode: FT-ICR/Orbitrap  
MS/MS Scan Mode: FT-ICR/Orbitrap

  

Protein List

  

Protein Accession Contains:

Protein Description Contains:

Protein Sample Area >=

Protein PTM Contains:

| Protein Group | Protein ID | Accession | -10lgP | Coverage (%) | Coverage (%) BRom | Area BRom | #Peptides | #Unique | #Spec BRom | PTM | Avg. Mass | Description |
| --- | --- | --- | --- | --- | --- | --- | --- | --- | --- | --- | --- | --- |
| 6 | 2 | T\_61 | 391.12 | 47 | 47 | 8.7342E10 | 60 | 45 | 258 | Y | 63952 | T\_61 |
| 6 | 3 | T\_55 | 391.12 | 46 | 46 | 8.7342E10 | 60 | 45 | 258 | Y | 64771 | T\_55 |
| 6 | 4 | T\_59 | 391.12 | 46 | 46 | 8.7342E10 | 60 | 45 | 258 | Y | 64771 | T\_59 |
| 6 | 5 | T\_58 | 391.12 | 46 | 46 | 8.7342E10 | 60 | 45 | 258 | Y | 64771 | T\_58 |
| 6 | 6 | T\_60 | 391.12 | 46 | 46 | 8.7342E10 | 60 | 45 | 258 | Y | 64771 | T\_60 |
| 10 | 12 | T\_46 | 364.17 | 32 | 32 | 9.2239E9 | 48 | 8 | 220 | Y | 66303 | T\_46 |
| 20 | 1 | T\_35 | 343.67 | 53 | 53 | 1.259E9 | 35 | 28 | 70 | Y | 64644 | T\_35 |
| 16 | 30 | T\_114 | 337.39 | 100 | 100 | 2.868E9 | 36 | 9 | 109 | Y | 11178 | T\_114 |
| 18 | 13 | T\_111 | 334.73 | 45 | 45 | 5.192E8 | 32 | 13 | 72 | Y | 54357 | T\_111 |
| 1 | 31 | T\_113 | 331.88 | 81 | 81 | 1.081E11 | 41 | 15 | 934 | Y | 11386 | T\_113 |
| 9 | 35 | T\_115 | 328.36 | 81 | 81 | 2.795E10 | 37 | 15 | 186 | Y | 11316 | T\_115 |
| 3 | 45 | T\_18 | 322.68 | 50 | 50 | 6.5104E10 | 30 | 4 | 398 | Y | 19178 | T\_18 |
| 13 | 48 | T\_24 | 312.93 | 67 | 67 | 4.1932E10 | 27 | 19 | 154 | Y | 13522 | T\_24 |
| 14 | 49 | Q8QFW4.1 | 306.09 | 68 | 68 | 8.6489E9 | 24 | 17 | 173 | Y | 16358 | RecName: Full=Basic phospholipase A2 beta-bungarotoxin A1 chain; Short=Beta-BuTX A1 chain; Short=svPLA2; AltName: Full=Phosphatidylcholine 2-acylhydrolase; Flags: Precursor |
| 14 | 50 | AAL87003.1 | 306.09 | 68 | 68 | 8.6489E9 | 24 | 17 | 173 | Y | 16358 | beta-bungarotoxin A chain precursor [Bungarus caeruleus] |
| 12 | 72 | T\_148 | 300.21 | 38 | 38 | 1.0136E10 | 23 | 23 | 195 | Y | 15865 | T\_148 |
| 4 | 71 | ABG90492.1 | 289.92 | 52 | 52 | 2.4407E8 | 21 | 1 | 298 | Y | 13164 | beta-bungarotoxin A8 chain, partial [Bungarus multicinctus] |
| 21 | 88 | T\_26 | 278.54 | 44 | 44 | 1.4514E9 | 17 | 2 | 56 | Y | 14060 | T\_26 |
| 8 | 130 | T\_1 | 274.40 | 65 | 65 | 1.0272E11 | 16 | 16 | 253 | Y | 9903 | T\_1 |
| 19 | 43 | Q8QFW3.1 | 269.83 | 51 | 51 | 1.3486E10 | 18 | 7 | 70 | Y | 16120 | RecName: Full=Basic phospholipase A2 beta-bungarotoxin A2 chain; Short=Beta-BuTX A2 chain; Short=svPLA2; AltName: Full=Phosphatidylcholine 2-acylhydrolase; Flags: Precursor |
| 19 | 44 | AAL87004.1 | 269.83 | 51 | 51 | 1.3486E10 | 18 | 7 | 70 | Y | 16120 | beta-bungarotoxin A2 chain precursor [Bungarus caeruleus] |
| 25 | 52 | JAA74736.1 | 262.34 | 21 | 21 | 4.0747E6 | 11 | 1 | 39 | Y | 60425 | ACN-Den-1, partial [Denisonia devisi] |
| 36 | 59 | T\_84 | 239.39 | 36 | 36 | 5.1117E8 | 15 | 13 | 24 | N | 21916 | T\_84 |
| 26 | 61 | ABN72539.1 | 232.45 | 24 | 24 | 2.1472E8 | 12 | 2 | 38 | Y | 58811 | L-amino acid oxidase [Bungarus multicinctus] |
| 26 | 62 | A8QL51.1 | 232.45 | 24 | 24 | 2.1472E8 | 12 | 2 | 38 | Y | 58811 | RecName: Full=L-amino-acid oxidase; Short=Bm-LAAO; Short=LAO; Flags: Precursor |
| 39 | 800 | ABU63164.1 | 226.51 | 15 | 15 | 5.6303E8 | 8 | 2 | 19 | Y | 15950 | phospholipase A2 precursor BF-40 [Bungarus fasciatus] |
| 39 | 802 | ABU63166.1 | 226.51 | 15 | 15 | 5.6303E8 | 8 | 2 | 19 | Y | 16004 | phospholipase A2 precursor BF-42 [Bungarus fasciatus] |
| 31 | 36 | T\_134 | 222.97 | 34 | 34 | 1.3852E9 | 13 | 3 | 31 | Y | 47274 | T\_134 |
| 31 | 37 | T\_137 | 222.97 | 34 | 34 | 1.3852E9 | 13 | 3 | 31 | Y | 47274 | T\_137 |
| 31 | 38 | T\_130 | 222.97 | 34 | 34 | 1.3852E9 | 13 | 3 | 31 | Y | 47274 | T\_130 |
| 27 | 46 | T\_22 | 221.41 | 51 | 51 | 3.9964E10 | 9 | 6 | 25 | Y | 19316 | T\_22 |
| 27 | 47 | T\_23 | 221.41 | 51 | 51 | 3.9964E10 | 9 | 6 | 25 | Y | 19364 | T\_23 |
| 32 | 213 | XP\_026570824.1 | 220.81 | 37 | 37 | 1.0003E9 | 7 | 7 | 29 | N | 15616 | hemoglobin subunit alpha [Pseudonaja textilis] |
| 33 | 184 | T\_3 | 211.53 | 71 | 71 | 4.116E9 | 9 | 9 | 22 | Y | 9828 | T\_3 |
| 34 | 135 | XP\_026548868.1 | 207.69 | 43 | 43 | 2.9266E8 | 13 | 6 | 26 | Y | 16270 | hemoglobin subunit beta-1 [Notechis scutatus] |
| 22 | 239 | T\_141 | 201.08 | 35 | 35 | 3.1355E10 | 11 | 8 | 47 | Y | 14129 | T\_141 |
| 22 | 240 | T\_140 | 201.08 | 29 | 29 | 3.1355E10 | 11 | 8 | 47 | Y | 17021 | T\_140 |
| 17 | 804 | 1PO8 | 199.58 | 19 | 19 | 3.5918E10 | 9 | 5 | 75 | Y | 13201 | Chain A, Phospholipase A2 |
| 17 | 805 | 1TC8 | 199.58 | 19 | 19 | 3.5918E10 | 9 | 5 | 75 | Y | 13201 | Chain A, Phospholipase A2 Isoform 1 |
| 17 | 806 | AAS20530.1 | 199.58 | 16 | 16 | 3.5918E10 | 9 | 5 | 75 | Y | 15058 | phospholipase A2 isoform 1, partial [Bungarus caeruleus] |
| 17 | 807 | Q6SLM2.1 | 199.58 | 16 | 16 | 3.5918E10 | 9 | 5 | 75 | Y | 15058 | RecName: Full=Acidic phospholipase A2 1; Short=svPLA2; AltName: Full=Phosphatidylcholine 2-acylhydrolase; Flags: Precursor |
| 41 | 231 | XP\_026555558.1 | 189.93 | 10 | 10 | 7.5733E8 | 9 | 9 | 13 | Y | 61349 | hepatocyte growth factor activator [Pseudonaja textilis] |
| 2 | 394 | T\_15 | 188.15 | 39 | 39 | 3.1068E10 | 7 | 6 | 499 | Y | 10070 | T\_15 |
| 40 | 87 | T\_19 | 186.46 | 43 | 43 | 2.4096E8 | 8 | 4 | 13 | Y | 20104 | T\_19 |
| 49 | 247 | XP\_034286707.1 | 185.07 | 7 | 7 | 0 | 8 | 1 | 12 | Y | 64689 | acetylcholinesterase isoform X3 [Pantherophis guttatus] |
| 49 | 248 | XP\_034286706.1 | 185.07 | 7 | 7 | 0 | 8 | 1 | 12 | Y | 68560 | acetylcholinesterase isoform X2 [Pantherophis guttatus] |
| 49 | 249 | XP\_034286705.1 | 185.07 | 7 | 7 | 0 | 8 | 1 | 12 | Y | 68691 | acetylcholinesterase isoform X1 [Pantherophis guttatus] |
| 46 | 379 | T\_2 | 182.89 | 38 | 38 | 9.7681E8 | 6 | 6 | 15 | Y | 9840 | T\_2 |
| 44 | 69 | T\_21 | 182.71 | 47 | 47 | 0 | 8 | 1 | 11 | Y | 20086 | T\_21 |
| 38 | 86 | LAA65154.1 | 182.69 | 47 | 47 | 0 | 8 | 1 | 16 | Y | 12644 | hypothetical protein [Micrurus corallinus] |
| 42 | 81 | T\_43 | 180.60 | 33 | 33 | 2.855E9 | 7 | 5 | 17 | Y | 37263 | T\_43 |
| 37 | 85 | T\_142 | 179.26 | 48 | 48 | 8.1278E8 | 8 | 8 | 16 | Y | 17070 | T\_142 |
| 35 | 372 | T\_64 | 178.85 | 36 | 36 | 4.8443E8 | 9 | 9 | 20 | Y | 16469 | T\_64 |
| 35 | 373 | T\_65 | 178.85 | 36 | 36 | 4.8443E8 | 9 | 9 | 20 | Y | 16469 | T\_65 |
| 35 | 374 | T\_68 | 178.85 | 36 | 36 | 4.8443E8 | 9 | 9 | 20 | Y | 16469 | T\_68 |
| 35 | 375 | T\_66 | 178.85 | 34 | 34 | 4.8443E8 | 9 | 9 | 20 | Y | 17239 | T\_66 |
| 35 | 376 | T\_63 | 178.85 | 27 | 27 | 4.8443E8 | 9 | 9 | 20 | Y | 22360 | T\_63 |
| 35 | 377 | T\_67 | 178.85 | 27 | 27 | 4.8443E8 | 9 | 9 | 20 | Y | 22360 | T\_67 |
| 59 | 131 | ETE71045.1 | 168.06 | 62 | 62 | 2.1386E7 | 6 | 6 | 6 | Y | 11903 | hypothetical protein L345\_03149, partial [Ophiophagus hannah] |
| 63 | 102 | XP\_026559591.1 | 163.42 | 25 | 25 | 5.184E7 | 5 | 5 | 5 | Y | 38486 | annexin A1 [Pseudonaja textilis] |
| 63 | 103 | XP\_026559592.1 | 163.42 | 25 | 25 | 5.184E7 | 5 | 5 | 5 | Y | 38486 | annexin A1 [Pseudonaja textilis] |
| 53 | 158 | 0412250A | 142.26 | 32 | 32 | 0 | 4 | 1 | 8 | Y | 13489 | toxin A,beta1 bungaro |
| 53 | 168 | 0402253A | 142.26 | 32 | 32 | 0 | 4 | 1 | 8 | Y | 13561 | toxin RCM A,beta1 bungaro |
| 58 | 148 | T\_105 | 140.27 | 44 | 44 | 3.8676E6 | 4 | 1 | 5 | Y | 11193 | T\_105 |
| 55 | 359 | T\_33 | 138.09 | 29 | 29 | 1.8382E8 | 2 | 2 | 8 | Y | 11247 | T\_33 |
| 56 | 113 | T\_79 | 137.59 | 27 | 27 | 5.2864E7 | 5 | 5 | 7 | Y | 32628 | T\_79 |
| 56 | 114 | T\_75 | 137.59 | 19 | 19 | 5.2864E7 | 5 | 5 | 7 | Y | 47592 | T\_75 |
| 56 | 115 | T\_76 | 137.59 | 19 | 19 | 5.2864E7 | 5 | 5 | 7 | Y | 47592 | T\_76 |
| 54 | 303 | XP\_026542555.1 | 135.98 | 6 | 6 | 8.5801E7 | 4 | 4 | 9 | Y | 53135 | multiple inositol polyphosphate phosphatase 1 isoform X2 [Notechis scutatus] |
| 54 | 304 | XP\_026542553.1 | 135.98 | 6 | 6 | 8.5801E7 | 4 | 4 | 9 | Y | 53549 | multiple inositol polyphosphate phosphatase 1 isoform X1 [Notechis scutatus] |
| 64 | 672 | ETE60521.1 | 134.32 | 6 | 6 | 2.1746E7 | 4 | 4 | 5 | Y | 48759 | hypothetical protein L345\_13731, partial [Ophiophagus hannah] |
| 64 | 896 | XP\_026580631.1 | 134.32 | 23 | 23 | 2.1746E7 | 4 | 4 | 5 | Y | 11919 | protein S100-A11-like [Pseudonaja textilis] |
| 64 | 897 | XP\_026580634.1 | 134.32 | 23 | 23 | 2.1746E7 | 4 | 4 | 5 | Y | 11919 | protein S100-A11-like [Pseudonaja textilis] |
| 64 | 898 | JAB53385.1 | 134.32 | 23 | 23 | 2.1746E7 | 4 | 4 | 5 | Y | 11921 | protein S100-A11 [Micrurus fulvius] |
| 64 | 899 | XP\_026580630.1 | 134.32 | 23 | 23 | 2.1746E7 | 4 | 4 | 5 | Y | 11919 | protein S100-A11-like [Pseudonaja textilis] |
| 64 | 900 | XP\_026580635.1 | 134.32 | 23 | 23 | 2.1746E7 | 4 | 4 | 5 | Y | 11919 | protein S100-A11-like [Pseudonaja textilis] |
| 61 | 211 | XP\_026555793.1 | 134.12 | 19 | 19 | 2.321E8 | 3 | 3 | 6 | Y | 34167 | ADP-ribosyl cyclase/cyclic ADP-ribose hydrolase 1-like [Pseudonaja textilis] |
| 69 | 317 | XP\_032087784.1 | 118.39 | 8 | 8 | 1.5375E6 | 3 | 1 | 3 | N | 54267 | multiple inositol polyphosphate phosphatase 1 [Thamnophis elegans] |
| 69 | 455 | XP\_013925207.1 | 118.39 | 11 | 11 | 1.5375E6 | 3 | 1 | 3 | N | 39926 | PREDICTED: multiple inositol polyphosphate phosphatase 1 [Thamnophis sirtalis] |
| 70 | 545 | XP\_025032622.1 | 115.41 | 3 | 3 | 6.7052E6 | 3 | 1 | 4 | N | 67497 | acetylcholinesterase isoform X2 [Python bivittatus] |
| 70 | 546 | XP\_025032620.1 | 115.41 | 3 | 3 | 6.7052E6 | 3 | 1 | 4 | N | 67497 | acetylcholinesterase isoform X2 [Python bivittatus] |
| 70 | 547 | XP\_025032621.1 | 115.41 | 3 | 3 | 6.7052E6 | 3 | 1 | 4 | N | 67497 | acetylcholinesterase isoform X2 [Python bivittatus] |
| 70 | 548 | JAC94932.1 | 115.41 | 3 | 3 | 6.7052E6 | 3 | 1 | 4 | N | 69041 | Acetylcholinesterase transcript 2 [Python regius] |
| 70 | 549 | XP\_025032619.1 | 115.41 | 3 | 3 | 6.7052E6 | 3 | 1 | 4 | N | 72046 | acetylcholinesterase isoform X1 [Python bivittatus] |
| 80 | 302 | ETE64512.1 | 109.22 | 3 | 3 | 9.6843E6 | 2 | 2 | 2 | Y | 105984 | Golgi apparatus protein 1, partial [Ophiophagus hannah] |
| 80 | 305 | XP\_015667558.1 | 109.22 | 3 | 3 | 9.6843E6 | 2 | 2 | 2 | Y | 118841 | Golgi apparatus protein 1, partial [Protobothrops mucrosquamatus] |
| 80 | 309 | XP\_032086218.1 | 109.22 | 3 | 3 | 9.6843E6 | 2 | 2 | 2 | Y | 131375 | Golgi apparatus protein 1 [Thamnophis elegans] |
| 80 | 310 | XP\_034281438.1 | 109.22 | 3 | 3 | 9.6843E6 | 2 | 2 | 2 | Y | 131761 | Golgi apparatus protein 1 [Pantherophis guttatus] |
| 80 | 313 | LAB62693.1 | 109.22 | 4 | 4 | 9.6843E6 | 2 | 2 | 2 | Y | 96178 | hypothetical protein [Micrurus surinamensis] |
| 80 | 314 | LAB62690.1 | 109.22 | 3 | 3 | 9.6843E6 | 2 | 2 | 2 | Y | 122130 | hypothetical protein, partial [Micrurus surinamensis] |
| 80 | 315 | XP\_026570202.1 | 109.22 | 3 | 3 | 9.6843E6 | 2 | 2 | 2 | Y | 133145 | Golgi apparatus protein 1 [Pseudonaja textilis] |
| 80 | 316 | XP\_026535930.1 | 109.22 | 3 | 3 | 9.6843E6 | 2 | 2 | 2 | Y | 132789 | Golgi apparatus protein 1 [Notechis scutatus] |
| 80 | 337 | XP\_025027126.1 | 109.22 | 10 | 10 | 9.6843E6 | 2 | 2 | 2 | Y | 35196 | Golgi apparatus protein 1 [Python bivittatus] |
| 80 | 362 | LAB18302.1 | 109.22 | 28 | 28 | 9.6843E6 | 2 | 2 | 2 | Y | 13184 | hypothetical protein, partial [Micrurus spixii] |
| 80 | 363 | XP\_015686495.1 | 109.22 | 19 | 19 | 9.6843E6 | 2 | 2 | 2 | Y | 18984 | Golgi apparatus protein 1-like, partial [Protobothrops mucrosquamatus] |
| 80 | 364 | LAB18300.1 | 109.22 | 9 | 9 | 9.6843E6 | 2 | 2 | 2 | Y | 39264 | hypothetical protein, partial [Micrurus spixii] |
| 85 | 378 | XP\_026547933.1 | 108.91 | 18 | 18 | 1.5097E7 | 2 | 2 | 2 | N | 17232 | fucolectin-like, partial [Notechis scutatus] |
| 85 | 390 | XP\_026578807.1 | 108.91 | 4 | 4 | 1.5097E7 | 2 | 2 | 2 | N | 88287 | LOW QUALITY PROTEIN: uncharacterized protein LOC113451644 [Pseudonaja textilis] |
| 76 | 339 | LAB57810.1 | 106.24 | 8 | 8 | 1.6383E7 | 2 | 2 | 3 | N | 34061 | hypothetical protein, partial [Micrurus surinamensis] |
| 76 | 504 | P80512.1 | 106.24 | 7 | 7 | 1.6383E7 | 2 | 2 | 3 | N | 39955 | RecName: Full=Alcohol dehydrogenase 1 |
| 76 | 505 | XP\_026528465.1 | 106.24 | 7 | 7 | 1.6383E7 | 2 | 2 | 3 | N | 39895 | alcohol dehydrogenase 1C [Notechis scutatus] |
| 76 | 506 | LAA59635.1 | 106.24 | 7 | 7 | 1.6383E7 | 2 | 2 | 3 | N | 39954 | hypothetical protein [Micrurus corallinus] |
| 76 | 507 | XP\_026554078.1 | 106.24 | 7 | 7 | 1.6383E7 | 2 | 2 | 3 | N | 39896 | alcohol dehydrogenase 1 [Pseudonaja textilis] |
| 60 | 418 | T\_81 | 104.90 | 5 | 5 | 4.3601E7 | 2 | 2 | 5 | N | 64044 | T\_81 |
| 60 | 419 | T\_83 | 104.90 | 5 | 5 | 4.3601E7 | 2 | 2 | 5 | N | 64044 | T\_83 |
| 66 | 495 | XP\_026545897.1 | 103.33 | 14 | 14 | 8.3688E7 | 2 | 2 | 4 | Y | 16186 | hemoglobin subunit beta [Notechis scutatus] |
| 66 | 916 | JAG67762.1 | 103.33 | 14 | 14 | 8.3688E7 | 2 | 2 | 4 | Y | 16362 | Hemoglobin subunit beta-2 [Boiga irregularis] |
| 66 | 917 | JAG67761.1 | 103.33 | 14 | 14 | 8.3688E7 | 2 | 2 | 4 | Y | 16362 | Hemoglobin subunit rho [Boiga irregularis] |
| 71 | 268 | ETE73401.1 | 100.63 | 3 | 3 | 1.4993E7 | 2 | 2 | 3 | Y | 138992 | Coagulation factor X isoform 1, partial [Ophiophagus hannah] |
| 71 | 294 | XP\_026580326.1 | 100.63 | 11 | 11 | 1.4993E7 | 2 | 2 | 3 | Y | 39293 | coagulation factor VII [Pseudonaja textilis] |
| 71 | 318 | JAS05177.1 | 100.63 | 9 | 9 | 1.4993E7 | 2 | 2 | 3 | Y | 48122 | coagulation factor VII [Micrurus tener] |
| 71 | 319 | JAI09074.1 | 100.63 | 9 | 9 | 1.4993E7 | 2 | 2 | 3 | Y | 48122 | coagulation factor VII [Micrurus fulvius] |
| 71 | 320 | JAS05063.1 | 100.63 | 9 | 9 | 1.4993E7 | 2 | 2 | 3 | Y | 48122 | coagulation factor VII [Micrurus fulvius] |
| 71 | 321 | JAB54462.1 | 100.63 | 9 | 9 | 1.4993E7 | 2 | 2 | 3 | Y | 48122 | coagulation factor 7 [Micrurus fulvius] |
| 71 | 345 | XP\_026548111.1 | 100.63 | 10 | 10 | 1.4993E7 | 2 | 2 | 3 | Y | 44790 | coagulation factor VII, partial [Notechis scutatus] |
| 50 | 837 | AAB20783.1 | 96.71 | 36 | 36 | 3.4561E6 | 2 | 1 | 11 | Y | 13014 | notechis 11'2=non-toxic phospholipase A2 [Notechis scutatus=Australian tiger snakes, ssp. scutatus, venom, Peptide, 118 aa] |
| 50 | 841 | P08873.1 | 96.71 | 30 | 30 | 3.4561E6 | 2 | 1 | 11 | Y | 15903 | RecName: Full=Basic phospholipase A2 notechis 11'2; Short=svPLA2; AltName: Full=Phosphatidylcholine 2-acylhydrolase; Flags: Precursor |
| 50 | 842 | CAA31125.1 | 96.71 | 30 | 30 | 3.4561E6 | 2 | 1 | 11 | Y | 15903 | PLA2 preprotein (AA-27 to 118) [Notechis scutatus] |
| 78 | 420 | AFJ49950.1 | 84.90 | 12 | 12 | 1.8158E7 | 2 | 2 | 2 | N | 50201 | Elongation factor 1-alpha 1-like [Crotalus adamanteus] |
| 78 | 421 | JAA97186.1 | 84.90 | 12 | 12 | 1.8158E7 | 2 | 2 | 2 | N | 50201 | Elongation factor 1-alpha 1 [Crotalus horridus] |
| 78 | 422 | JAI13488.1 | 84.90 | 12 | 12 | 1.8158E7 | 2 | 2 | 2 | N | 50201 | elongation factor 1-alpha 1 [Crotalus adamanteus] |
| 78 | 423 | JAC95701.1 | 84.90 | 12 | 12 | 1.8158E7 | 2 | 2 | 2 | N | 50205 | elongation factor-1 alpha [Hypsiglena sp. JMG-2014] |
| 78 | 424 | JAC95700.1 | 84.90 | 12 | 12 | 1.8158E7 | 2 | 2 | 2 | N | 50205 | Elongation factor 1-alpha 1 [Hypsiglena sp. JMG-2014] |
| 78 | 425 | JAI10049.1 | 84.90 | 12 | 12 | 1.8158E7 | 2 | 2 | 2 | N | 50125 | elongation factor 1-alpha 1 [Micrurus fulvius] |
| 78 | 426 | JAG46923.1 | 84.90 | 12 | 12 | 1.8158E7 | 2 | 2 | 2 | N | 50201 | Elongation factor 1-alpha 1 [Crotalus horridus] |
| 78 | 427 | XP\_015672964.1 | 84.90 | 12 | 12 | 1.8158E7 | 2 | 2 | 2 | N | 50155 | elongation factor 1-alpha 1 [Protobothrops mucrosquamatus] |
| 78 | 428 | JAG44960.1 | 84.90 | 12 | 12 | 1.8158E7 | 2 | 2 | 2 | N | 50201 | elongation factor 1-alpha 1 [Crotalus horridus] |
| 78 | 429 | JAV50678.1 | 84.90 | 12 | 12 | 1.8158E7 | 2 | 2 | 2 | N | 50201 | Elongation factor 1-alpha 1 [Agkistrodon contortrix contortrix] |
| 78 | 430 | JAB54325.1 | 84.90 | 12 | 12 | 1.8158E7 | 2 | 2 | 2 | N | 50125 | Elongation factor 1-alpha 1 [Micrurus fulvius] |
| 151 | 817 | XP\_015682720.1 | 76.91 | 11 | 11 | 8.7682E6 | 1 | 1 | 1 | N | 16531 | hemoglobin subunit beta-2 [Protobothrops mucrosquamatus] |
| 99 | 492 | XP\_026542227.1 | 75.36 | 4 | 4 | 5.3043E5 | 1 | 1 | 1 | N | 51879 | 6-phosphogluconate dehydrogenase, decarboxylating [Notechis scutatus] |
| 99 | 663 | JAI12109.1 | 75.36 | 4 | 4 | 5.3043E5 | 1 | 1 | 1 | N | 53182 | 6-phosphogluconate dehydrogenase, decarboxylating [Crotalus adamanteus] |
| 99 | 664 | JAG46036.1 | 75.36 | 4 | 4 | 5.3043E5 | 1 | 1 | 1 | N | 53165 | 6-phosphogluconate dehydrogenase, decarboxylating [Crotalus horridus] |
| 99 | 665 | XP\_032087395.1 | 75.36 | 4 | 4 | 5.3043E5 | 1 | 1 | 1 | N | 53028 | 6-phosphogluconate dehydrogenase, decarboxylating [Thamnophis elegans] |
| 99 | 666 | XP\_015676923.1 | 75.36 | 4 | 4 | 5.3043E5 | 1 | 1 | 1 | N | 53093 | 6-phosphogluconate dehydrogenase, decarboxylating [Protobothrops mucrosquamatus] |
| 99 | 667 | AFJ50914.1 | 75.36 | 4 | 4 | 5.3043E5 | 1 | 1 | 1 | N | 53182 | 6-phosphogluconate dehydrogenase [Crotalus adamanteus] |
| 99 | 668 | JAB53691.1 | 75.36 | 4 | 4 | 5.3043E5 | 1 | 1 | 1 | N | 53044 | 6-phosphogluconate dehydrogenase [Micrurus fulvius] |
| 99 | 669 | JAV51398.1 | 75.36 | 4 | 4 | 5.3043E5 | 1 | 1 | 1 | N | 53093 | 6-phosphogluconate dehydrogenase, decarboxylating [Agkistrodon contortrix contortrix] |
| 99 | 670 | XP\_034294750.1 | 75.36 | 4 | 4 | 5.3043E5 | 1 | 1 | 1 | N | 53151 | 6-phosphogluconate dehydrogenase, decarboxylating [Pantherophis guttatus] |
| 99 | 671 | LAA63155.1 | 75.36 | 4 | 4 | 5.3043E5 | 1 | 1 | 1 | N | 53179 | hypothetical protein, partial [Micrurus corallinus] |
| 99 | 683 | JAG68880.1 | 75.36 | 4 | 4 | 5.3043E5 | 1 | 1 | 1 | N | 53122 | 6-phosphogluconate dehydrogenase [Boiga irregularis] |
| 99 | 684 | LAB46151.1 | 75.36 | 3 | 3 | 5.3043E5 | 1 | 1 | 1 | N | 56657 | hypothetical protein, partial [Micrurus surinamensis] |
| 48 | 437 | Q9DF52.1 | 71.14 | 33 | 33 | 1.7513E9 | 2 | 1 | 7 | Y | 15765 | RecName: Full=Basic phospholipase A2 KPA2; Short=svPLA2; AltName: Full=Phosphatidylcholine 2-acylhydrolase; Flags: Precursor |
| 48 | 438 | AAG13412.1 | 71.14 | 33 | 33 | 1.7513E9 | 2 | 1 | 7 | Y | 15765 | phospholipase A2 [Bungarus caeruleus] |
| 139 | 686 | P15816.1 | 70.87 | 18 | 18 | 1.7586E6 | 1 | 1 | 1 | Y | 9517 | RecName: Full=Kappa-2-bungarotoxin; AltName: Full=Kappa-neurotoxin CB1; Flags: Precursor |
| 139 | 687 | CAA35774.1 | 70.87 | 18 | 18 | 1.7586E6 | 1 | 1 | 1 | Y | 9517 | unnamed protein product [Bungarus multicinctus] |
| 139 | 876 | O12962.1 | 70.87 | 18 | 18 | 1.7586E6 | 1 | 1 | 1 | Y | 9474 | RecName: Full=Kappa-5-bungarotoxin; Flags: Precursor |
| 139 | 877 | CAA72940.1 | 70.87 | 18 | 18 | 1.7586E6 | 1 | 1 | 1 | Y | 9474 | kappa5 bungarotoxin [Bungarus multicinctus] |
| 137 | 602 | ACE73577.1 | 69.57 | 7 | 7 | 1.3601E7 | 1 | 1 | 1 | N | 26458 | cysteine-rich seceretory protein Bc-CRPb [Bungarus candidus] |
| 137 | 603 | ACE73578.1 | 69.57 | 7 | 7 | 1.3601E7 | 1 | 1 | 1 | N | 26443 | cysteine-rich seceretory protein Bc-CRPa [Bungarus candidus] |
| 137 | 604 | T\_89 | 69.57 | 7 | 7 | 1.3601E7 | 1 | 1 | 1 | N | 26379 | T\_89 |
| 137 | 605 | T\_93 | 69.57 | 7 | 7 | 1.3601E7 | 1 | 1 | 1 | N | 26379 | T\_93 |
| 137 | 606 | T\_92 | 69.57 | 7 | 7 | 1.3601E7 | 1 | 1 | 1 | N | 26379 | T\_92 |
| 137 | 607 | T\_95 | 69.57 | 7 | 7 | 1.3601E7 | 1 | 1 | 1 | N | 26379 | T\_95 |
| 137 | 608 | T\_91 | 69.57 | 7 | 7 | 1.3601E7 | 1 | 1 | 1 | N | 27807 | T\_91 |
| 137 | 609 | T\_94 | 69.57 | 6 | 6 | 1.3601E7 | 1 | 1 | 1 | N | 31259 | T\_94 |
| 137 | 885 | P81993.1 | 69.57 | 25 | 25 | 1.3601E7 | 1 | 1 | 1 | N | 7752 | RecName: Full=Cysteine-rich venom protein bucarin |
| 137 | 886 | T\_96 | 69.57 | 10 | 10 | 1.3601E7 | 1 | 1 | 1 | N | 19403 | T\_96 |
| 137 | 887 | ACN93671.1 | 69.57 | 7 | 7 | 1.3601E7 | 1 | 1 | 1 | N | 26305 | opharin precursor [Ophiophagus hannah] |
| 137 | 888 | ACH73168.1 | 69.57 | 7 | 7 | 1.3601E7 | 1 | 1 | 1 | N | 26216 | kaouthin-2 precursor [Naja kaouthia] |
| 137 | 889 | AAP20603.1 | 69.57 | 7 | 7 | 1.3601E7 | 1 | 1 | 1 | N | 26246 | cysteine-rich venom protein [Naja atra] |
| 137 | 890 | Q7ZZN8.1 | 69.57 | 7 | 7 | 1.3601E7 | 1 | 1 | 1 | N | 26246 | RecName: Full=Cysteine-rich venom protein natrin-2; AltName: Full=Cysteine-rich venom protein 2; AltName: Full=NA-CRVP2; AltName: Full=Protein G2b; Flags: Precursor |
| 137 | 891 | P84808.2 | 69.57 | 7 | 7 | 1.3601E7 | 1 | 1 | 1 | N | 26216 | RecName: Full=Cysteine-rich venom protein kaouthin-2; AltName: Full=Cysteine-rich venom protein 23; Short=CRVP-23k; Flags: Precursor |
| 137 | 892 | AHZ08822.1 | 69.57 | 7 | 7 | 1.3601E7 | 1 | 1 | 1 | N | 26216 | cysteine-rich secretory protein [Micropechis ikaheca] |
| 137 | 893 | XP\_032069593.1 | 69.57 | 7 | 7 | 1.3601E7 | 1 | 1 | 1 | N | 26603 | cysteine-rich venom protein kaouthin-2 [Thamnophis elegans] |
| 137 | 894 | JAC94992.1 | 69.57 | 7 | 7 | 1.3601E7 | 1 | 1 | 1 | N | 26749 | Cysteine-rich secretory protein A [Opheodrys aestivus] |
| 137 | 895 | ETE62137.1 | 69.57 | 7 | 7 | 1.3601E7 | 1 | 1 | 1 | N | 27268 | hypothetical protein L345\_12110, partial [Ophiophagus hannah] |
| 143 | 774 | XP\_034292109.1 | 69.56 | 7 | 7 | 1.5314E7 | 1 | 1 | 1 | Y | 33533 | neural proliferation differentiation and control protein 1 [Pantherophis guttatus] |
| 77 | 716 | Q9PRL9.1 | 68.46 | 9 | 9 | 8.8287E6 | 1 | 1 | 3 | N | 15448 | RecName: Full=Hemoglobin subunit alpha-1; AltName: Full=Alpha-1-globin; AltName: Full=Hemoglobin alpha-1 chain; AltName: Full=Hemoglobin alpha-I chain |
| 77 | 717 | AAB34003.1 | 68.46 | 9 | 9 | 8.8287E6 | 1 | 1 | 3 | N | 15448 | hemoglobin alpha I chain [Naja naja=cobra snakes, naja, blood, Peptide, 141 aa] |
| 128 | 748 | XP\_034287104.1 | 68.09 | 5 | 5 | 8.9964E5 | 1 | 1 | 1 | N | 32547 | annexin A3 [Pantherophis guttatus] |
| 128 | 755 | LAB37887.1 | 68.09 | 5 | 5 | 8.9964E5 | 1 | 1 | 1 | N | 35845 | hypothetical protein [Micrurus spixii] |
| 128 | 765 | JAG68721.1 | 68.09 | 5 | 5 | 8.9964E5 | 1 | 1 | 1 | N | 35839 | annexin A3 isoform 3 [Boiga irregularis] |
| 128 | 911 | XP\_026532239.1 | 68.09 | 5 | 5 | 8.9964E5 | 1 | 1 | 1 | N | 35736 | annexin A3 [Notechis scutatus] |
| 128 | 912 | XP\_013926160.1 | 68.09 | 5 | 5 | 8.9964E5 | 1 | 1 | 1 | N | 35733 | PREDICTED: annexin A4 [Thamnophis sirtalis] |
| 128 | 913 | XP\_026574715.1 | 68.09 | 5 | 5 | 8.9964E5 | 1 | 1 | 1 | N | 35888 | annexin A3 [Pseudonaja textilis] |
| 128 | 914 | XP\_032080318.1 | 68.09 | 5 | 5 | 8.9964E5 | 1 | 1 | 1 | N | 35795 | annexin A3 [Thamnophis elegans] |
| 128 | 915 | JAC96259.1 | 68.09 | 5 | 5 | 8.9964E5 | 1 | 1 | 1 | N | 35689 | annexin A3 [Hypsiglena sp. JMG-2014] |
| 112 | 776 | AAL30054.1 | 67.87 | 28 | 28 | 1.3114E8 | 1 | 1 | 1 | Y | 9580 | kappa 1a bungaratoxin [Bungarus candidus] |
| 112 | 777 | Q8AY56.1 | 67.87 | 28 | 28 | 1.3114E8 | 1 | 1 | 1 | Y | 9580 | RecName: Full=Kappa 1a-bungarotoxin; Flags: Precursor |
| 140 | 702 | LAA33342.1 | 67.24 | 14 | 14 | 0 | 1 | 1 | 1 | Y | 18057 | hypothetical protein, partial [Micrurus lemniscatus carvalhoi] |
| 140 | 919 | LAB47895.1 | 67.24 | 10 | 10 | 0 | 1 | 1 | 1 | Y | 25556 | hypothetical protein, partial [Micrurus surinamensis] |
| 152 | 920 | T\_74 | 66.13 | 18 | 18 | 1.8424E6 | 1 | 1 | 1 | Y | 10142 | T\_74 |
| 121 | 554 | XP\_026528620.1 | 61.92 | 3 | 3 | 2.5269E5 | 1 | 1 | 1 | N | 58373 | prosaposin-like isoform X2 [Notechis scutatus] |
| 121 | 555 | XP\_026569354.1 | 61.92 | 3 | 3 | 2.5269E5 | 1 | 1 | 1 | N | 58289 | prosaposin isoform X2 [Pseudonaja textilis] |
| 121 | 556 | XP\_026528619.1 | 61.92 | 2 | 2 | 2.5269E5 | 1 | 1 | 1 | N | 58759 | prosaposin-like isoform X1 [Notechis scutatus] |
| 121 | 557 | XP\_026569353.1 | 61.92 | 2 | 2 | 2.5269E5 | 1 | 1 | 1 | N | 58674 | prosaposin isoform X1 [Pseudonaja textilis] |
| 121 | 558 | XP\_015669101.1 | 61.92 | 3 | 3 | 2.5269E5 | 1 | 1 | 1 | N | 57981 | prosaposin [Protobothrops mucrosquamatus] |
| 121 | 559 | AFJ51008.1 | 61.92 | 3 | 3 | 2.5269E5 | 1 | 1 | 1 | N | 58013 | Proactivator polypeptide-like [Crotalus adamanteus] |
| 121 | 560 | BAN82088.1 | 61.92 | 2 | 2 | 2.5269E5 | 1 | 1 | 1 | N | 58564 | hypothetical protein [Protobothrops flavoviridis] |
| 121 | 561 | ETE61271.1 | 61.92 | 3 | 3 | 2.5269E5 | 1 | 1 | 1 | N | 54055 | Proactivator polypeptide [Ophiophagus hannah] |
| 121 | 767 | XP\_013923763.1 | 61.92 | 3 | 3 | 2.5269E5 | 1 | 1 | 1 | N | 58050 | PREDICTED: prosaposin isoform X2 [Thamnophis sirtalis] |
| 121 | 768 | XP\_013923762.1 | 61.92 | 2 | 2 | 2.5269E5 | 1 | 1 | 1 | N | 58435 | PREDICTED: prosaposin isoform X1 [Thamnophis sirtalis] |
| 121 | 926 | XP\_007441356.1 | 61.92 | 3 | 3 | 2.5269E5 | 1 | 1 | 1 | N | 42934 | prosaposin-like [Python bivittatus] |
| 121 | 927 | XP\_032088229.1 | 61.92 | 3 | 3 | 2.5269E5 | 1 | 1 | 1 | N | 58050 | prosaposin-like isoform X2 [Thamnophis elegans] |
| 121 | 928 | XP\_034291658.1 | 61.92 | 2 | 2 | 2.5269E5 | 1 | 1 | 1 | N | 58438 | prosaposin-like isoform X4 [Pantherophis guttatus] |
| 121 | 929 | XP\_032088228.1 | 61.92 | 2 | 2 | 2.5269E5 | 1 | 1 | 1 | N | 58435 | prosaposin-like isoform X1 [Thamnophis elegans] |
| 121 | 930 | XP\_034291656.1 | 61.92 | 2 | 2 | 2.5269E5 | 1 | 1 | 1 | N | 58696 | prosaposin-like isoform X2 [Pantherophis guttatus] |
| 121 | 931 | XP\_034291657.1 | 61.92 | 2 | 2 | 2.5269E5 | 1 | 1 | 1 | N | 58695 | prosaposin-like isoform X3 [Pantherophis guttatus] |
| 121 | 932 | XP\_034291655.1 | 61.92 | 2 | 2 | 2.5269E5 | 1 | 1 | 1 | N | 58824 | prosaposin-like isoform X1 [Pantherophis guttatus] |
| 138 | 620 | XP\_034265676.1 | 60.31 | 5 | 5 | 3.5037E6 | 1 | 1 | 1 | N | 24564 | peroxiredoxin-6 [Pantherophis guttatus] |
| 138 | 960 | XP\_032082279.1 | 60.31 | 5 | 5 | 3.5037E6 | 1 | 1 | 1 | N | 24580 | peroxiredoxin-6 [Thamnophis elegans] |
| 138 | 961 | JAI09679.1 | 60.31 | 5 | 5 | 3.5037E6 | 1 | 1 | 1 | N | 24589 | Peroxiredoxin-6-like protein [Micrurus fulvius] |
| 138 | 962 | AFJ50899.1 | 60.31 | 5 | 5 | 3.5037E6 | 1 | 1 | 1 | N | 24496 | Peroxiredoxin-6-like [Crotalus adamanteus] |
| 138 | 963 | JAB53717.1 | 60.31 | 5 | 5 | 3.5037E6 | 1 | 1 | 1 | N | 24589 | peroxiredoxin-6-like protein [Micrurus fulvius] |
| 138 | 964 | XP\_026524951.1 | 60.31 | 5 | 5 | 3.5037E6 | 1 | 1 | 1 | N | 24549 | peroxiredoxin-6 [Notechis scutatus] |
| 138 | 965 | JAG67074.1 | 60.31 | 5 | 5 | 3.5037E6 | 1 | 1 | 1 | N | 24547 | Peroxiredoxin-6-like protein [Boiga irregularis] |
| 138 | 966 | LAB58410.1 | 60.31 | 5 | 5 | 3.5037E6 | 1 | 1 | 1 | N | 24562 | hypothetical protein [Micrurus surinamensis] |
| 138 | 967 | JAA96187.1 | 60.31 | 5 | 5 | 3.5037E6 | 1 | 1 | 1 | N | 24496 | Peroxiredoxin-6-like protein [Crotalus horridus] |
| 138 | 968 | JAV49687.1 | 60.31 | 5 | 5 | 3.5037E6 | 1 | 1 | 1 | N | 24496 | Peroxiredoxin-6-like protein [Agkistrodon contortrix contortrix] |
| 138 | 969 | XP\_015687493.1 | 60.31 | 5 | 5 | 3.5037E6 | 1 | 1 | 1 | N | 24526 | peroxiredoxin-6 [Protobothrops mucrosquamatus] |
| 138 | 970 | JAI12121.1 | 60.31 | 5 | 5 | 3.5037E6 | 1 | 1 | 1 | N | 24496 | Peroxiredoxin-6-like protein [Crotalus adamanteus] |
| 138 | 971 | XP\_026559154.1 | 60.31 | 5 | 5 | 3.5037E6 | 1 | 1 | 1 | N | 24519 | peroxiredoxin-6 [Pseudonaja textilis] |
| 153 | 977 | ETE73981.1 | 58.22 | 7 | 7 | 0 | 1 | 1 | 1 | Y | 25402 | 14-3-3 protein sigma, partial [Ophiophagus hannah] |
| 82 | 703 | AAC83997.1 | 56.12 | 12 | 12 | 1.1946E7 | 1 | 1 | 2 | Y | 7981 | alpha-bungarotoxin isoform R15, partial [Bungarus multicinctus] |
| 82 | 775 | AAC83990.1 | 56.12 | 12 | 12 | 1.1946E7 | 1 | 1 | 2 | Y | 8017 | alpha-bungarotoxin isoform R12, partial [Bungarus multicinctus] |
| 82 | 981 | P01385.1 | 56.12 | 12 | 12 | 1.1946E7 | 1 | 1 | 2 | Y | 8135 | RecName: Full=Alpha-elapitoxin-Aa2b; Short=Alpha-EPTX-Aa2b; AltName: Full=Long neurotoxin 1 |
| 82 | 982 | 0512217A | 56.12 | 12 | 12 | 1.1946E7 | 1 | 1 | 2 | Y | 8060 | neurotoxin |
| 82 | 983 | AAC83992.1 | 56.12 | 12 | 12 | 1.1946E7 | 1 | 1 | 2 | Y | 7994 | alpha-bungarotoxin isoform R9, partial [Bungarus multicinctus] |
| 82 | 984 | 1HAJ | 56.12 | 12 | 12 | 1.1946E7 | 1 | 1 | 2 | Y | 7994 | Chain A, Alpha-bungarotoxin |
| 82 | 985 | 1IDH | 56.12 | 12 | 12 | 1.1946E7 | 1 | 1 | 2 | Y | 7994 | Chain A, The Nmr Solution Structure Of The Complex Formed Between Alpha-Bungarotoxin And An 18mer Cognate Peptide |
| 82 | 986 | 2BTX | 56.12 | 12 | 12 | 1.1946E7 | 1 | 1 | 2 | Y | 7994 | Chain A, Solution Nmr Structure Of The Complex Of Alpha-Bungarotoxin With A Library Derived Peptide, Nmr, Minimized Average Structure |
| 82 | 987 | 1IK8 | 56.12 | 12 | 12 | 1.1946E7 | 1 | 1 | 2 | Y | 7994 | Chain A, Nmr Structure Of Alpha-Bungarotoxin |
| 82 | 988 | 1BXP | 56.12 | 12 | 12 | 1.1946E7 | 1 | 1 | 2 | Y | 7994 | Chain A, Solution Nmr Structure Of The Complex Of Alpha-Bungarotoxin With A Library Derived Peptide, 20 Structures |
| 82 | 989 | 1ABT | 56.12 | 12 | 12 | 1.1946E7 | 1 | 1 | 2 | Y | 7994 | Chain A, Nmr Solution Structure Of An Alpha-Bungarotoxin(Slash) Nicotinic Receptor Peptide Complex |
| 82 | 990 | 1IKC | 56.12 | 12 | 12 | 1.1946E7 | 1 | 1 | 2 | Y | 7994 | Chain A, Nmr Structure Of Alpha-Bungarotoxin |
| 82 | 991 | AAC83994.1 | 56.12 | 12 | 12 | 1.1946E7 | 1 | 1 | 2 | Y | 7994 | alpha-bungarotoxin isoform R11, partial [Bungarus multicinctus] |
| 82 | 992 | AAC83991.1 | 56.12 | 12 | 12 | 1.1946E7 | 1 | 1 | 2 | Y | 7994 | alpha-bungarotoxin isoform R8, partial [Bungarus multicinctus] |
| 82 | 993 | 1IDL | 56.12 | 12 | 12 | 1.1946E7 | 1 | 1 | 2 | Y | 7994 | Chain A, The Nmr Solution Structure Of Alpha-Bungarotoxin |
| 82 | 994 | 1HOY | 56.12 | 12 | 12 | 1.1946E7 | 1 | 1 | 2 | Y | 8000 | Chain A, Nmr Structure Of The Complex Between A-Bungarotoxin And A Mimotope Of The Nicotinic Acetilcholine Receptor |
| 82 | 995 | AAC83993.1 | 56.12 | 12 | 12 | 1.1946E7 | 1 | 1 | 2 | Y | 7994 | alpha-bungarotoxin isoform R10, partial [Bungarus multicinctus] |
| 82 | 996 | 720920A | 56.12 | 12 | 12 | 1.1946E7 | 1 | 1 | 2 | Y | 7994 | toxin alpha,bungaro |
| 82 | 997 | 2ABX | 56.12 | 12 | 12 | 1.1946E7 | 1 | 1 | 2 | Y | 7994 | Chain B, The Crystal Structure Of Alpha-Bungarotoxin At 2.5 Angstroms Resolution. Relation To Solution Structure And Binding To Acetylcholine Receptor |
| 82 | 998 | CAM11311.1 | 56.12 | 12 | 12 | 1.1946E7 | 1 | 1 | 2 | Y | 8259 | alpha-bungarotoxin (A31), partial [Bungarus candidus] |
| 82 | 999 | ADN67593.1 | 56.12 | 12 | 12 | 1.1946E7 | 1 | 1 | 2 | Y | 8259 | three-finger toxin precursor, partial [Bungarus multicinctus] |
| 82 | 1000 | CAM11305.1 | 56.12 | 12 | 12 | 1.1946E7 | 1 | 1 | 2 | Y | 8259 | alpha-bungarotoxin (A31), partial [Bungarus candidus] |
| 82 | 1001 | CAM11309.1 | 56.12 | 12 | 12 | 1.1946E7 | 1 | 1 | 2 | Y | 8259 | alpha-bungarotoxin (A31), partial [Bungarus candidus] |
| 82 | 1002 | CAD92407.1 | 56.12 | 12 | 12 | 1.1946E7 | 1 | 1 | 2 | Y | 8259 | alpha-bungarotoxin (A31) precursor, partial [Bungarus candidus] |
| 82 | 1003 | CAM11312.1 | 56.12 | 12 | 12 | 1.1946E7 | 1 | 1 | 2 | Y | 8259 | alpha-bungarotoxin (A31), partial [Bungarus candidus] |
| 82 | 1004 | CAM11307.1 | 56.12 | 12 | 12 | 1.1946E7 | 1 | 1 | 2 | Y | 8259 | alpha-bungarotoxin (A31), partial [Bungarus multicinctus] |
| 82 | 1005 | CAM11310.1 | 56.12 | 12 | 12 | 1.1946E7 | 1 | 1 | 2 | Y | 8259 | alpha-bungarotoxin (A31), partial [Bungarus candidus] |
| 82 | 1006 | CAM11304.1 | 56.12 | 12 | 12 | 1.1946E7 | 1 | 1 | 2 | Y | 8259 | alpha-bungarotoxin (A31), partial [Bungarus candidus] |
| 82 | 1007 | CAM11302.1 | 56.12 | 12 | 12 | 1.1946E7 | 1 | 1 | 2 | Y | 8306 | alpha-delta-bungarotoxin-4, partial [Bungarus caeruleus] |
| 82 | 1008 | D2N116.1 | 56.12 | 12 | 12 | 1.1946E7 | 1 | 1 | 2 | Y | 8306 | RecName: Full=Alpha-delta-bungarotoxin-4; Short=Alpha-delta-Bgt-4; Flags: Precursor |
| 82 | 1009 | CAM11303.1 | 56.12 | 12 | 12 | 1.1946E7 | 1 | 1 | 2 | Y | 8228 | alpha-bungarotoxin (A5), partial [Bungarus candidus] |
| 82 | 1010 | CAM11306.1 | 56.12 | 12 | 12 | 1.1946E7 | 1 | 1 | 2 | Y | 8259 | alpha-bungarotoxin (T7/A8), partial [Bungarus candidus] |
| 82 | 1011 | Q8UW29.1 | 56.12 | 10 | 10 | 1.1946E7 | 1 | 1 | 2 | Y | 10218 | RecName: Full=Long neurotoxin 1; Flags: Precursor |
| 82 | 1012 | AAL54892.1 | 56.12 | 10 | 10 | 1.1946E7 | 1 | 1 | 2 | Y | 10218 | long neurotoxin isoform [Hydrophis hardwickii] |
| 82 | 1013 | A3FM53.1 | 56.12 | 10 | 10 | 1.1946E7 | 1 | 1 | 2 | Y | 10406 | RecName: Full=Long neurotoxin 2; Flags: Precursor |
| 82 | 1014 | ABN54806.1 | 56.12 | 10 | 10 | 1.1946E7 | 1 | 1 | 2 | Y | 10406 | long chain neurotoxin isoform 2 precursor [Hydrophis hardwickii] |
| 82 | 1015 | JAA74770.1 | 56.12 | 10 | 10 | 1.1946E7 | 1 | 1 | 2 | Y | 10255 | 3FTx-Ech-21 [Echiopsis curta] |
| 82 | 1016 | P01384.2 | 56.12 | 10 | 10 | 1.1946E7 | 1 | 1 | 2 | Y | 10289 | RecName: Full=Alpha-elapitoxin-Nss2a; Short=Alpha-EPTX-Nss2a; AltName: Full=Long neurotoxin 1; Short=LNTX-1; AltName: Full=Notechis III-4; Flags: Precursor |
| 82 | 1017 | ABK63537.1 | 56.12 | 10 | 10 | 1.1946E7 | 1 | 1 | 2 | Y | 10289 | LNTX-1 precursor [Notechis scutatus] |
| 82 | 1018 | JAA74654.1 | 56.12 | 10 | 10 | 1.1946E7 | 1 | 1 | 2 | Y | 10392 | 3FTx-Aca-27 [Acanthophis wellsi] |
| 82 | 1019 | AAC83981.1 | 56.12 | 9 | 9 | 1.1946E7 | 1 | 1 | 2 | Y | 10285 | alpha-bungarotoxin precursor [Bungarus multicinctus] |
| 82 | 1020 | AAL30056.1 | 56.12 | 9 | 9 | 1.1946E7 | 1 | 1 | 2 | Y | 10285 | alpha bungaratoxin [Bungarus candidus] |
| 122 | 628 | LAA32179.1 | 55.14 | 3 | 3 | 3.3012E5 | 1 | 1 | 1 | N | 55369 | hypothetical protein, partial [Micrurus lemniscatus carvalhoi] |
| 122 | 629 | XP\_026524224.1 | 55.14 | 3 | 3 | 3.3012E5 | 1 | 1 | 1 | N | 55575 | UTP--glucose-1-phosphate uridylyltransferase isoform X2 [Notechis scutatus] |
| 122 | 630 | XP\_026568672.1 | 55.14 | 3 | 3 | 3.3012E5 | 1 | 1 | 1 | N | 55506 | UTP--glucose-1-phosphate uridylyltransferase isoform X2 [Pseudonaja textilis] |
| 122 | 631 | XP\_026524225.1 | 55.14 | 3 | 3 | 3.3012E5 | 1 | 1 | 1 | N | 55575 | UTP--glucose-1-phosphate uridylyltransferase isoform X2 [Notechis scutatus] |
| 122 | 632 | XP\_026568674.1 | 55.14 | 3 | 3 | 3.3012E5 | 1 | 1 | 1 | N | 55506 | UTP--glucose-1-phosphate uridylyltransferase isoform X2 [Pseudonaja textilis] |
| 122 | 633 | XP\_015667825.1 | 55.14 | 3 | 3 | 3.3012E5 | 1 | 1 | 1 | N | 55573 | UTP--glucose-1-phosphate uridylyltransferase isoform X2 [Protobothrops mucrosquamatus] |
| 122 | 634 | XP\_026524226.1 | 55.14 | 3 | 3 | 3.3012E5 | 1 | 1 | 1 | N | 55575 | UTP--glucose-1-phosphate uridylyltransferase isoform X2 [Notechis scutatus] |
| 122 | 635 | XP\_034288350.1 | 55.14 | 3 | 3 | 3.3012E5 | 1 | 1 | 1 | N | 55563 | UTP--glucose-1-phosphate uridylyltransferase isoform X2 [Pantherophis guttatus] |
| 122 | 636 | XP\_007435816.1 | 55.14 | 3 | 3 | 3.3012E5 | 1 | 1 | 1 | N | 55487 | UTP--glucose-1-phosphate uridylyltransferase isoform X3 [Python bivittatus] |
| 122 | 637 | XP\_026568671.1 | 55.14 | 3 | 3 | 3.3012E5 | 1 | 1 | 1 | N | 55506 | UTP--glucose-1-phosphate uridylyltransferase isoform X2 [Pseudonaja textilis] |
| 122 | 638 | XP\_026568673.1 | 55.14 | 3 | 3 | 3.3012E5 | 1 | 1 | 1 | N | 55506 | UTP--glucose-1-phosphate uridylyltransferase isoform X2 [Pseudonaja textilis] |
| 122 | 639 | XP\_026524223.1 | 55.14 | 3 | 3 | 3.3012E5 | 1 | 1 | 1 | N | 55575 | UTP--glucose-1-phosphate uridylyltransferase isoform X2 [Notechis scutatus] |
| 122 | 640 | ETE70626.1 | 55.14 | 3 | 3 | 3.3012E5 | 1 | 1 | 1 | N | 55501 | UTP--glucose-1-phosphate uridylyltransferase [Ophiophagus hannah] |
| 122 | 641 | LAB55207.1 | 55.14 | 3 | 3 | 3.3012E5 | 1 | 1 | 1 | N | 56720 | hypothetical protein [Micrurus surinamensis] |
| 122 | 642 | LAA32175.1 | 55.14 | 3 | 3 | 3.3012E5 | 1 | 1 | 1 | N | 56750 | hypothetical protein [Micrurus lemniscatus carvalhoi] |
| 122 | 643 | JAV48583.1 | 55.14 | 3 | 3 | 3.3012E5 | 1 | 1 | 1 | N | 56815 | UTP--glucose-1-phosphate uridylyltransferase [Agkistrodon contortrix contortrix] |
| 122 | 644 | XP\_015667816.1 | 55.14 | 3 | 3 | 3.3012E5 | 1 | 1 | 1 | N | 56801 | UTP--glucose-1-phosphate uridylyltransferase isoform X1 [Protobothrops mucrosquamatus] |
| 122 | 645 | XP\_034288348.1 | 55.14 | 3 | 3 | 3.3012E5 | 1 | 1 | 1 | N | 56763 | UTP--glucose-1-phosphate uridylyltransferase isoform X1 [Pantherophis guttatus] |
| 122 | 646 | XP\_007435815.1 | 55.14 | 3 | 3 | 3.3012E5 | 1 | 1 | 1 | N | 56687 | UTP--glucose-1-phosphate uridylyltransferase isoform X2 [Python bivittatus] |
| 122 | 647 | XP\_026524222.1 | 55.14 | 3 | 3 | 3.3012E5 | 1 | 1 | 1 | N | 56775 | UTP--glucose-1-phosphate uridylyltransferase isoform X1 [Notechis scutatus] |
| 122 | 648 | XP\_026568670.1 | 55.14 | 3 | 3 | 3.3012E5 | 1 | 1 | 1 | N | 56706 | UTP--glucose-1-phosphate uridylyltransferase isoform X1 [Pseudonaja textilis] |
| 122 | 649 | LAA32177.1 | 55.14 | 3 | 3 | 3.3012E5 | 1 | 1 | 1 | N | 57127 | hypothetical protein, partial [Micrurus lemniscatus carvalhoi] |
| 122 | 650 | XP\_025027268.1 | 55.14 | 2 | 2 | 3.3012E5 | 1 | 1 | 1 | N | 59921 | UTP--glucose-1-phosphate uridylyltransferase isoform X1 [Python bivittatus] |
| 129 | 1050 | P02010.1 | 54.82 | 21 | 21 | 0 | 1 | 1 | 1 | N | 15437 | RecName: Full=Hemoglobin subunit alpha; AltName: Full=Alpha-globin; AltName: Full=Hemoglobin alpha chain |
| 136 | 569 | XP\_032076462.1 | 52.14 | 5 | 5 | 1.1705E6 | 1 | 1 | 1 | N | 31282 | endonuclease domain-containing 1 protein-like isoform X2 [Thamnophis elegans] |
| 136 | 570 | XP\_032076461.1 | 52.14 | 5 | 5 | 1.1705E6 | 1 | 1 | 1 | N | 34799 | endonuclease domain-containing 1 protein-like isoform X1 [Thamnophis elegans] |
| 136 | 1052 | ETE56723.1 | 52.14 | 9 | 9 | 1.1705E6 | 1 | 1 | 1 | N | 18083 | Endonuclease domain-containing 1 protein [Ophiophagus hannah] |
| 136 | 1053 | XP\_026546198.1 | 52.14 | 5 | 5 | 1.1705E6 | 1 | 1 | 1 | N | 31377 | endonuclease domain-containing 1 protein-like isoform X2 [Notechis scutatus] |
| 136 | 1054 | XP\_026546199.1 | 52.14 | 5 | 5 | 1.1705E6 | 1 | 1 | 1 | N | 31377 | endonuclease domain-containing 1 protein-like isoform X2 [Notechis scutatus] |
| 136 | 1055 | XP\_026546197.1 | 52.14 | 5 | 5 | 1.1705E6 | 1 | 1 | 1 | N | 31377 | endonuclease domain-containing 1 protein-like isoform X2 [Notechis scutatus] |
| 136 | 1056 | JAB54304.1 | 52.14 | 5 | 5 | 1.1705E6 | 1 | 1 | 1 | N | 31577 | endonuclease domain-containing 1 protein [Micrurus fulvius] |
| 136 | 1057 | XP\_026579206.1 | 52.14 | 5 | 5 | 1.1705E6 | 1 | 1 | 1 | N | 31175 | endonuclease domain-containing 1 protein-like [Pseudonaja textilis] |
| 136 | 1058 | XP\_026579205.1 | 52.14 | 5 | 5 | 1.1705E6 | 1 | 1 | 1 | N | 31175 | endonuclease domain-containing 1 protein-like [Pseudonaja textilis] |
| 136 | 1059 | XP\_026579207.1 | 52.14 | 5 | 5 | 1.1705E6 | 1 | 1 | 1 | N | 31175 | endonuclease domain-containing 1 protein-like [Pseudonaja textilis] |
| 136 | 1060 | XP\_026546196.1 | 52.14 | 5 | 5 | 1.1705E6 | 1 | 1 | 1 | N | 33047 | endonuclease domain-containing 1 protein-like isoform X1 [Notechis scutatus] |
| 136 | 1061 | ETE59939.1 | 52.14 | 3 | 3 | 1.1705E6 | 1 | 1 | 1 | N | 51067 | Endonuclease domain-containing 1 protein, partial [Ophiophagus hannah] |
| 102 | 533 | LAA72691.1 | 51.18 | 6 | 6 | 4.5855E5 | 1 | 1 | 1 | N | 47317 | hypothetical protein [Micrurus lemniscatus lemniscatus] |
| 102 | 534 | LAA72685.1 | 51.18 | 3 | 3 | 4.5855E5 | 1 | 1 | 1 | N | 103842 | hypothetical protein [Micrurus lemniscatus lemniscatus] |
| 102 | 535 | ETE73165.1 | 51.18 | 3 | 3 | 4.5855E5 | 1 | 1 | 1 | N | 107545 | Trifunctional purine biosynthetic protein adenosine-3 [Ophiophagus hannah] |
| 102 | 536 | LAA72697.1 | 51.18 | 3 | 3 | 4.5855E5 | 1 | 1 | 1 | N | 107758 | hypothetical protein [Micrurus lemniscatus lemniscatus] |
| 102 | 752 | XP\_026519973.1 | 51.18 | 6 | 6 | 4.5855E5 | 1 | 1 | 1 | N | 47419 | trifunctional purine biosynthetic protein adenosine-3-like [Notechis scutatus] |
| 102 | 753 | XP\_026556356.1 | 51.18 | 3 | 3 | 4.5855E5 | 1 | 1 | 1 | N | 107597 | trifunctional purine biosynthetic protein adenosine-3 [Pseudonaja textilis] |
| 102 | 754 | XP\_026556355.1 | 51.18 | 3 | 3 | 4.5855E5 | 1 | 1 | 1 | N | 107597 | trifunctional purine biosynthetic protein adenosine-3 [Pseudonaja textilis] |
| 102 | 1062 | XP\_034291632.1 | 51.18 | 3 | 3 | 4.5855E5 | 1 | 1 | 1 | N | 107225 | trifunctional purine biosynthetic protein adenosine-3-like [Pantherophis guttatus] |
| 124 | 699 | XP\_013931233.1 | 45.93 | 21 | 21 | 3.3751E7 | 1 | 1 | 1 | Y | 16229 | PREDICTED: hemoglobin subunit beta-2 [Thamnophis sirtalis] |
| 124 | 700 | XP\_032075324.1 | 45.93 | 21 | 21 | 3.3751E7 | 1 | 1 | 1 | Y | 16227 | hemoglobin subunit beta-2 [Thamnophis elegans] |
| 119 | 508 | XP\_026561289.1 | 41.50 | 1 | 1 | 2.615E6 | 1 | 1 | 1 | N | 96775 | ectonucleotide pyrophosphatase/phosphodiesterase family member 3 isoform X5 [Pseudonaja textilis] |
| 141 | 718 | XP\_007429085.1 | 40.86 | 2 | 2 | 1.1454E6 | 1 | 1 | 1 | N | 59718 | cochlin [Python bivittatus] |
| 141 | 1081 | LAA86986.1 | 40.86 | 5 | 5 | 1.1454E6 | 1 | 1 | 1 | N | 28508 | hypothetical protein, partial [Micrurus lemniscatus lemniscatus] |
| 141 | 1082 | LAB57337.1 | 40.86 | 5 | 5 | 1.1454E6 | 1 | 1 | 1 | N | 30451 | hypothetical protein, partial [Micrurus surinamensis] |
| 141 | 1083 | XP\_013923615.1 | 40.86 | 2 | 2 | 1.1454E6 | 1 | 1 | 1 | N | 59675 | PREDICTED: cochlin [Thamnophis sirtalis] |
| 141 | 1084 | XP\_026520312.1 | 40.86 | 2 | 2 | 1.1454E6 | 1 | 1 | 1 | N | 59883 | cochlin [Notechis scutatus] |
| 141 | 1085 | XP\_034273852.1 | 40.86 | 2 | 2 | 1.1454E6 | 1 | 1 | 1 | N | 59540 | cochlin [Pantherophis guttatus] |
| 141 | 1086 | LAB44384.1 | 40.86 | 2 | 2 | 1.1454E6 | 1 | 1 | 1 | N | 59763 | hypothetical protein [Micrurus spixii] |
| 141 | 1087 | XP\_026566518.1 | 40.86 | 2 | 2 | 1.1454E6 | 1 | 1 | 1 | N | 59709 | cochlin [Pseudonaja textilis] |
| 141 | 1088 | XP\_032069561.1 | 40.86 | 2 | 2 | 1.1454E6 | 1 | 1 | 1 | N | 62259 | cochlin [Thamnophis elegans] |
| 145 | 1090 | XP\_026526467.1 | 40.42 | 2 | 2 | 1.7947E7 | 1 | 1 | 1 | N | 86822 | exocyst complex component 6 [Notechis scutatus] |
| 72 | 1097 | ABW24182.1 | 40.08 | 17 | 17 | 1.8079E5 | 1 | 1 | 2 | Y | 16409 | PLA-20 precursor [Austrelaps superbus] |
| 104 | 479 | XP\_013931090.1 | 39.69 | 2 | 2 | 0 | 1 | 1 | 1 | Y | 51798 | PREDICTED: beta-enolase [Thamnophis sirtalis] |
| 104 | 486 | XP\_032091032.1 | 39.69 | 3 | 3 | 0 | 1 | 1 | 1 | Y | 47597 | beta-enolase [Thamnophis elegans] |
| 104 | 676 | JAB54294.1 | 39.69 | 3 | 3 | 0 | 1 | 1 | 1 | Y | 47587 | enolase B [Micrurus fulvius] |
| 104 | 677 | XP\_007441409.1 | 39.69 | 3 | 3 | 0 | 1 | 1 | 1 | Y | 47642 | beta-enolase [Python bivittatus] |
| 104 | 729 | JAG68757.1 | 39.69 | 3 | 3 | 0 | 1 | 1 | 1 | Y | 47546 | Alpha-enolase [Boiga irregularis] |
| 104 | 769 | CBN61477.1 | 39.69 | 3 | 3 | 0 | 1 | 1 | 1 | Y | 47570 | unnamed protein product [Python regius] |
| 104 | 770 | AAD41646.1 | 39.69 | 3 | 3 | 0 | 1 | 1 | 1 | Y | 47570 | alpha enolase [Python regius] |
| 104 | 771 | XP\_026571534.1 | 39.69 | 3 | 3 | 0 | 1 | 1 | 1 | Y | 47553 | alpha-enolase [Pseudonaja textilis] |
| 104 | 1098 | XP\_026582136.1 | 39.69 | 8 | 8 | 0 | 1 | 1 | 1 | Y | 16189 | beta-enolase-like, partial [Pseudonaja textilis] |
| 104 | 1099 | XP\_013923835.1 | 39.69 | 7 | 7 | 0 | 1 | 1 | 1 | Y | 17757 | PREDICTED: gamma-enolase [Thamnophis sirtalis] |
| 104 | 1100 | XP\_013916893.1 | 39.69 | 7 | 7 | 0 | 1 | 1 | 1 | Y | 17752 | PREDICTED: alpha-enolase-like [Thamnophis sirtalis] |
| 104 | 1101 | LAA54352.1 | 39.69 | 4 | 4 | 0 | 1 | 1 | 1 | Y | 30239 | hypothetical protein [Micrurus corallinus] |
| 104 | 1102 | LAA72706.1 | 39.69 | 4 | 4 | 0 | 1 | 1 | 1 | Y | 32447 | hypothetical protein, partial [Micrurus lemniscatus lemniscatus] |
| 104 | 1103 | LAA54354.1 | 39.69 | 3 | 3 | 0 | 1 | 1 | 1 | Y | 39337 | hypothetical protein [Micrurus corallinus] |
| 104 | 1104 | LAA54356.1 | 39.69 | 3 | 3 | 0 | 1 | 1 | 1 | Y | 41086 | hypothetical protein, partial [Micrurus corallinus] |
| 104 | 1105 | LAA78451.1 | 39.69 | 3 | 3 | 0 | 1 | 1 | 1 | Y | 41419 | hypothetical protein [Micrurus lemniscatus lemniscatus] |
| 104 | 1106 | LAA78455.1 | 39.69 | 3 | 3 | 0 | 1 | 1 | 1 | Y | 41419 | hypothetical protein [Micrurus lemniscatus lemniscatus] |
| 104 | 1107 | JAI10301.1 | 39.69 | 3 | 3 | 0 | 1 | 1 | 1 | Y | 47479 | Alpha-enolase [Micrurus fulvius] |
| 104 | 1108 | JAG47510.1 | 39.69 | 3 | 3 | 0 | 1 | 1 | 1 | Y | 47465 | Alpha-enolase [Crotalus horridus] |
| 104 | 1109 | JAG45884.1 | 39.69 | 3 | 3 | 0 | 1 | 1 | 1 | Y | 47491 | Alpha-enolase [Crotalus horridus] |
| 104 | 1110 | XP\_026569147.1 | 39.69 | 3 | 3 | 0 | 1 | 1 | 1 | Y | 47406 | gamma-enolase [Pseudonaja textilis] |
| 104 | 1111 | JAA97809.1 | 39.69 | 3 | 3 | 0 | 1 | 1 | 1 | Y | 47491 | Alpha-enolase [Crotalus horridus] |
| 104 | 1112 | XP\_026542044.1 | 39.69 | 3 | 3 | 0 | 1 | 1 | 1 | Y | 47378 | gamma-enolase [Notechis scutatus] |
| 104 | 1113 | AFJ49385.1 | 39.69 | 3 | 3 | 0 | 1 | 1 | 1 | Y | 47491 | Alpha-enolase [Crotalus adamanteus] |
| 104 | 1114 | XP\_026544503.1 | 39.69 | 3 | 3 | 0 | 1 | 1 | 1 | Y | 47550 | alpha-enolase [Notechis scutatus] |
| 104 | 1115 | JAC96284.1 | 39.69 | 3 | 3 | 0 | 1 | 1 | 1 | Y | 47493 | alpha-enolase [Hypsiglena sp. JMG-2014] |
| 104 | 1116 | JAB54295.1 | 39.69 | 3 | 3 | 0 | 1 | 1 | 1 | Y | 47479 | Alpha-enolase [Micrurus fulvius] |
| 104 | 1117 | JAI14387.1 | 39.69 | 3 | 3 | 0 | 1 | 1 | 1 | Y | 47491 | Alpha-enolase [Crotalus adamanteus] |
| 104 | 1118 | XP\_007420481.1 | 39.69 | 3 | 3 | 0 | 1 | 1 | 1 | Y | 47296 | gamma-enolase [Python bivittatus] |
| 104 | 1119 | JAV51289.1 | 39.69 | 3 | 3 | 0 | 1 | 1 | 1 | Y | 47505 | Alpha-enolase [Agkistrodon contortrix contortrix] |
| 104 | 1120 | ETE72098.1 | 39.69 | 2 | 2 | 0 | 1 | 1 | 1 | Y | 51701 | Gamma-enolase, partial [Ophiophagus hannah] |
| 87 | 1092 | P81236.1 | 39.20 | 36 | 36 | 0 | 1 | 1 | 1 | Y | 12857 | RecName: Full=Basic phospholipase A2 acanthin-1; Short=svPLA2; AltName: Full=Acanthin I; AltName: Full=Phosphatidylcholine 2-acylhydrolase |
| total 346 proteins |  |  |  |  |  |  |  |  |  |  |  |  |
| --- | --- | --- | --- | --- | --- | --- | --- | --- | --- | --- | --- | --- |

  

T\_61

back to list

  

| Protein Coverage
| Supporting Peptides
|

Protein Coverage:

Supporting Peptides:

| Peptide | Uniq | -10lgP | Mass | Length | ppm | m/z | z | RT | Fraction | Scan | Source File | Area BRom | #Feature | #Feature BRom | Start | End | PTM | AScore | Found By |
| --- | --- | --- | --- | --- | --- | --- | --- | --- | --- | --- | --- | --- | --- | --- | --- | --- | --- | --- | --- |
| R.GLSLPVLDGHVTAFLGIPFAEPPVGR.M | Y | 101.40 | 2658.4534 | 26 | 0.7 | 1330.2349 | 2 | 93.83 | 1 | 67305 | 01122020\_RID\_2209\_BuCaKA02.raw | 5.7352E10 | 14 | 14 | 40 | 65 |  |  | PEAKS DB |
| R.AALLGKQLGC(+57.02)HFNNDSELVSC(+57.02)LR.S | Y | 96.22 | 2601.2791 | 23 | 0.3 | 1301.6472 | 2 | 41.43 | 1 | 24779 | 01122020\_RID\_2209\_BuCaKA02.raw | 1.888E8 | 2 | 2 | 268 | 290 | Carbamidomethylation | C10:Carbamidomethylation:1000.00;C21:Carbamidomethylation:1000.00 | PEAKS DB |
| R.AILQSGGPNAPWATVTPAESR.R | N | 95.10 | 2122.0806 | 21 | -0.9 | 1062.0466 | 2 | 52.50 | 1 | 33145 | 01122020\_RID\_2209\_BuCaKA02.raw | 1.3371E9 | 2 | 2 | 245 | 265 |  |  | PEAKS DB |
| R.EALDDIVGDHNVIC(+57.02)PVVQFANDYAKR.N | N | 93.08 | 2957.4341 | 26 | 0.1 | 740.3659 | 4 | 70.15 | 1 | 47677 | 01122020\_RID\_2209\_BuCaKA02.raw | 1.6502E7 | 3 | 3 | 412 | 437 | Carbamidomethylation | C14:Carbamidomethylation:1000.00 | PEAKS DB |
| R.EALDDIVGDHNVIC(+57.02)PVVQFANDYAK.R | N | 88.62 | 2801.3330 | 25 | 1.0 | 1401.6752 | 2 | 77.11 | 1 | 53666 | 01122020\_RID\_2209\_BuCaKA02.raw | 7.8726E8 | 2 | 2 | 412 | 436 | Carbamidomethylation | C14:Carbamidomethylation:1000.00 | PEAKS DB |
| R.LALQWIQNNIHPFGGNPR.A | Y | 87.29 | 2074.0859 | 18 | 4.4 | 692.3723 | 3 | 60.27 | 1 | 39720 | 01122020\_RID\_2209\_BuCaKA02.raw | 2.2787E8 | 1 | 1 | 198 | 215 |  |  | PEAKS DB |
| R.AVTIFGESAGAASVGMHLLSTQSR.T | N | 85.30 | 2389.2061 | 24 | 0.3 | 797.4095 | 3 | 65.27 | 1 | 44229 | 01122020\_RID\_2209\_BuCaKA02.raw | 4.1007E8 | 3 | 3 | 216 | 239 |  |  | PEAKS DB |
| K.NREALDDIVGDHNVIC(+57.02)PVVQFANDYAK.R | N | 82.20 | 3071.4771 | 27 | 0.7 | 768.8771 | 4 | 65.86 | 1 | 44085 | 01122020\_RID\_2209\_BuCaKA02.raw | 2.8252E8 | 1 | 1 | 410 | 436 | Carbamidomethylation | C16:Carbamidomethylation:1000.00 | PEAKS DB |
| R.AQIC(+57.02)AFWNHFLPK.L | N | 81.68 | 1630.8079 | 13 | 0.4 | 816.4115 | 2 | 60.34 | 1 | 39698 | 01122020\_RID\_2209\_BuCaKA02.raw | 3.6259E8 | 1 | 1 | 541 | 553 | Carbamidomethylation | C4:Carbamidomethylation:1000.00 | PEAKS DB |
| R.AGELKVSTQTGSVRGLSLPVLDGHVTAFLGIPFAEPPVGR.M | Y | 77.17 | 4072.2109 | 40 | 4.9 | 815.4535 | 5 | 78.62 | 1 | 54698 | 01122020\_RID\_2209\_BuCaKA02.raw | 1.683E9 | 3 | 3 | 26 | 65 |  |  | PEAKS DB |
| G.LSLPVLDGHVTAFLGIPFAEPPVGR.M | Y | 75.82 | 2601.4319 | 25 | 1.7 | 868.1527 | 3 | 93.27 | 1 | 66854 | 01122020\_RID\_2209\_BuCaKA02.raw | 9.8181E7 | 1 | 1 | 41 | 65 |  |  | PEAKS DB |
| R.AGELKVSTQTGSVR.G | Y | 75.64 | 1431.7681 | 14 | 0.2 | 716.8914 | 2 | 12.05 | 1 | 2024 | 01122020\_RID\_2209\_BuCaKA02.raw | 2.521E6 | 1 | 1 | 26 | 39 |  |  | PEAKS DB |
| R.TLFQRAILQSGGPNAPWATVTPAESR.R | Y | 75.41 | 2767.4404 | 26 | 0.6 | 923.4880 | 3 | 59.02 | 1 | 38537 | 01122020\_RID\_2209\_BuCaKA02.raw | 9.0758E7 | 1 | 1 | 240 | 265 |  |  | PEAKS DB |
| R.AVTIFGESAGAASVGMHLLSTQSRTLFQR.A | Y | 75.28 | 3034.5657 | 29 | 1.4 | 759.6498 | 4 | 72.55 | 1 | 49639 | 01122020\_RID\_2209\_BuCaKA02.raw | 4.0261E7 | 2 | 2 | 216 | 244 |  |  | PEAKS DB |
| M.GLLDQRLALQWIQNNIHPFGGNPRAVTIFGESAGAASVGMHLLSTQSR.T | Y | 74.87 | 5127.6577 | 48 | 1.0 | 1026.5398 | 5 | 84.83 | 1 | 59856 | 01122020\_RID\_2209\_BuCaKA02.raw | 2.0822E7 | 1 | 1 | 192 | 239 |  |  | PEAKS DB |
| L.KVSTQTGSVRGLSLPVLDGHVTAFLGIPFAEPPVGR.M | Y | 71.73 | 3702.0256 | 36 | 5.4 | 926.5187 | 4 | 75.43 | 1 | 52253 | 01122020\_RID\_2209\_BuCaKA02.raw | 5.6822E8 | 4 | 4 | 30 | 65 |  |  | PEAKS DB |
| D.DIVGDHNVIC(+57.02)PVVQFANDYAK.R | N | 70.32 | 2373.1423 | 21 | 1.2 | 792.0557 | 3 | 67.35 | 1 | 45397 | 01122020\_RID\_2209\_BuCaKA02.raw | 3.7367E7 | 1 | 1 | 416 | 436 | Carbamidomethylation | C10:Carbamidomethylation:1000.00 | PEAKS DB |
| K.VSTQTGSVRGLSLPVLDGHVTAFLGIPFAEPPVGR.M | Y | 70.30 | 3573.9307 | 35 | 9.4 | 715.8001 | 5 | 83.23 | 1 | 58537 | 01122020\_RID\_2209\_BuCaKA02.raw | 1.4423E10 | 7 | 7 | 31 | 65 |  |  | PEAKS DB |
| V.SPGRAGELKVSTQTGSVR.G | Y | 70.11 | 1828.9755 | 18 | 0.0 | 610.6658 | 3 | 12.05 | 1 | 2023 | 01122020\_RID\_2209\_BuCaKA02.raw | 1.7435E6 | 1 | 1 | 22 | 39 |  |  | PEAKS DB |
| R.LALQWIQNNIHPFGGNPRAVTIFGESAGAASVGMHLLSTQSR.T | Y | 70.03 | 4445.2812 | 42 | 1.3 | 890.0647 | 5 | 76.25 | 1 | 52778 | 01122020\_RID\_2209\_BuCaKA02.raw | 6.7471E7 | 3 | 3 | 198 | 239 |  |  | PEAKS DB |
| R.SKNPQELIDEEWSVLPYK.S | Y | 69.96 | 2174.0896 | 18 | 0.8 | 725.7044 | 3 | 63.32 | 1 | 42005 | 01122020\_RID\_2209\_BuCaKA02.raw | 2.4894E7 | 2 | 2 | 291 | 308 |  |  | PEAKS DB |
| M.GLLDQRLALQWIQNNIHPFGGNPR.A | Y | 69.87 | 2756.4622 | 24 | 2.9 | 919.8307 | 3 | 77.23 | 1 | 53582 | 01122020\_RID\_2209\_BuCaKA02.raw | 2.4764E8 | 4 | 4 | 192 | 215 |  |  | PEAKS DB |
| R.AILQSGGPNAPWATVTPAESRR.R | N | 67.72 | 2278.1819 | 22 | 1.4 | 760.4023 | 3 | 39.81 | 1 | 23723 | 01122020\_RID\_2209\_BuCaKA02.raw | 7.6056E7 | 1 | 1 | 245 | 266 |  |  | PEAKS DB |
| R.RAALLGKQLGC(+57.02)HFNNDSELVSC(+57.02)LR.S | Y | 65.73 | 2757.3801 | 24 | 2.0 | 690.3537 | 4 | 33.56 | 1 | 18494 | 01122020\_RID\_2209\_BuCaKA02.raw | 1.5928E7 | 1 | 1 | 267 | 290 | Carbamidomethylation | C11:Carbamidomethylation:1000.00;C22:Carbamidomethylation:1000.00 | PEAKS DB |
| F.LGIPFAEPPVGR.M | N | 65.37 | 1251.6975 | 12 | 1.1 | 626.8567 | 2 | 56.11 | 1 | 36154 | 01122020\_RID\_2209\_BuCaKA02.raw | 4.6223E8 | 1 | 1 | 54 | 65 |  |  | PEAKS DB |
| L.SLPVLDGHVTAFLGIPFAEPPVGR.M | Y | 65.32 | 2488.3477 | 24 | 2.0 | 830.4581 | 3 | 86.79 | 1 | 61552 | 01122020\_RID\_2209\_BuCaKA02.raw | 8.1857E7 | 1 | 1 | 42 | 65 |  |  | PEAKS DB |
| V.STQTGSVRGLSLPVLDGHVTAFLGIPFAEPPVGR.M | Y | 65.11 | 3474.8623 | 34 | 6.8 | 869.7288 | 4 | 82.73 | 1 | 58133 | 01122020\_RID\_2209\_BuCaKA02.raw | 0 | 0 | 0 | 32 | 65 |  |  | PEAKS DB |
| R.TGNPTDPADKSGAWPTYTASQPQYVQLNTQPLATQPSLR.A | Y | 63.72 | 4199.0557 | 39 | 1.2 | 1400.6942 | 3 | 59.70 | 1 | 39174 | 01122020\_RID\_2209\_BuCaKA02.raw | 1.0279E9 | 3 | 3 | 502 | 540 |  |  | PEAKS DB |
| R.GLSLPVLDGHVTAFL.G | Y | 63.72 | 1537.8503 | 15 | 1.7 | 769.9337 | 2 | 87.38 | 1 | 62023 | 01122020\_RID\_2209\_BuCaKA02.raw | 1.6202E7 | 1 | 1 | 40 | 54 |  |  | PEAKS DB |
| K.VSTQTGSVRGLSLPVLDGHVTAF.L | Y | 63.61 | 2340.2437 | 23 | 0.9 | 1171.1301 | 2 | 64.35 | 1 | 42978 | 01122020\_RID\_2209\_BuCaKA02.raw | 5.93E8 | 2 | 2 | 31 | 53 |  |  | PEAKS DB |
| L.PVLDGHVTAFLGIPFAEPPVGR.M | Y | 63.60 | 2288.2317 | 22 | 2.5 | 763.7531 | 3 | 79.31 | 1 | 55332 | 01122020\_RID\_2209\_BuCaKA02.raw | 3.4806E6 | 1 | 1 | 44 | 65 |  |  | PEAKS DB |
| R.TLFQRAILQSGGPNAPWATVTPAESRR.R | Y | 62.58 | 2923.5415 | 27 | -0.7 | 731.8922 | 4 | 49.01 | 1 | 30339 | 01122020\_RID\_2209\_BuCaKA02.raw | 0 | 0 | 0 | 240 | 266 |  |  | PEAKS DB |
| S.LPVLDGHVTAFLGIPFAEPPVGR.M | Y | 62.25 | 2401.3157 | 23 | 2.2 | 801.4476 | 3 | 85.81 | 1 | 60666 | 01122020\_RID\_2209\_BuCaKA02.raw | 1.1292E7 | 1 | 1 | 43 | 65 |  |  | PEAKS DB |
| A.FLGIPFAEPPVGR.M | N | 62.09 | 1398.7659 | 13 | 0.0 | 700.3902 | 2 | 73.18 | 1 | 50153 | 01122020\_RID\_2209\_BuCaKA02.raw | 1.8064E7 | 1 | 1 | 53 | 65 |  |  | PEAKS DB |
| K.QLGC(+57.02)HFNNDSELVSC(+57.02)LR.S | Y | 62.05 | 2047.9204 | 17 | 0.7 | 683.6479 | 3 | 34.95 | 1 | 19633 | 01122020\_RID\_2209\_BuCaKA02.raw | 7.1157E7 | 1 | 1 | 274 | 290 | Carbamidomethylation | C4:Carbamidomethylation:1000.00;C15:Carbamidomethylation:1000.00 | PEAKS DB |
| L.ATQPSLRAQIC(+57.02)AFWNHFLPK.L | Y | 60.92 | 2384.2212 | 20 | 2.7 | 795.7498 | 3 | 71.29 | 1 | 48640 | 01122020\_RID\_2209\_BuCaKA02.raw | 2.1393E7 | 2 | 2 | 534 | 553 | Carbamidomethylation | C11:Carbamidomethylation:1000.00 | PEAKS DB |
| R.AVTIFGESAGAASVGMHLLSTQSRTLFQRAILQSGGPNAPWATVTPAESR.R | Y | 60.82 | 5138.6357 | 50 | 0.4 | 1285.6667 | 4 | 83.73 | 1 | 58887 | 01122020\_RID\_2209\_BuCaKA02.raw | 9.1205E7 | 2 | 2 | 216 | 265 |  |  | PEAKS DB |
| R.AQIC(+57.02)AFWNHFLPKL.L | N | 59.24 | 1743.8918 | 14 | 1.7 | 872.9547 | 2 | 72.95 | 1 | 49964 | 01122020\_RID\_2209\_BuCaKA02.raw | 1.5107E8 | 2 | 2 | 541 | 554 | Carbamidomethylation | C4:Carbamidomethylation:1000.00 | PEAKS DB |
| K.SIFRFPFVPVIDG.D | N | 58.74 | 1492.8077 | 13 | 2.3 | 747.4128 | 2 | 91.00 | 1 | 65018 | 01122020\_RID\_2209\_BuCaKA02.raw | 9.7066E6 | 1 | 1 | 309 | 321 |  |  | PEAKS DB |
| V.RGLSLPVLDGHVTAFLGIPFAEPPVGR.M | Y | 58.04 | 2814.5544 | 27 | 2.6 | 704.6477 | 4 | 82.79 | 1 | 58181 | 01122020\_RID\_2209\_BuCaKA02.raw | 0 | 0 | 0 | 39 | 65 |  |  | PEAKS DB |
| R.AGELKVSTQTGSVRGLSLPVLDGHVTAF.L | Y | 57.78 | 2838.5239 | 28 | 0.1 | 710.6383 | 4 | 60.89 | 1 | 40258 | 01122020\_RID\_2209\_BuCaKA02.raw | 9.4408E8 | 5 | 5 | 26 | 53 |  |  | PEAKS DB |
| D.PADKSGAWPTYTASQPQYVQLNTQPLATQPSLR.A | Y | 56.43 | 3613.8164 | 33 | -0.8 | 1205.6118 | 3 | 56.49 | 1 | 36480 | 01122020\_RID\_2209\_BuCaKA02.raw | 4.7617E7 | 1 | 1 | 508 | 540 |  |  | PEAKS DB |
| Q.IC(+57.02)AFWNHFLPK.L | N | 56.19 | 1431.7122 | 11 | 1.5 | 716.8644 | 2 | 55.11 | 1 | 35292 | 01122020\_RID\_2209\_BuCaKA02.raw | 7.8377E6 | 1 | 1 | 543 | 553 | Carbamidomethylation | C2:Carbamidomethylation:1000.00 | PEAKS DB |
| A.TQPSLRAQIC(+57.02)AFWNHFLPK.L | Y | 56.17 | 2313.1841 | 19 | 0.9 | 579.3038 | 4 | 71.36 | 1 | 48657 | 01122020\_RID\_2209\_BuCaKA02.raw | 9.1707E6 | 2 | 2 | 535 | 553 | Carbamidomethylation | C10:Carbamidomethylation:1000.00 | PEAKS DB |
| R.AQIC(+57.02)AFWNHFLPKLLNATVDPPSADR.R | Y | 56.04 | 2980.5017 | 26 | 1.6 | 746.1339 | 4 | 77.71 | 1 | 54026 | 01122020\_RID\_2209\_BuCaKA02.raw | 0 | 0 | 0 | 541 | 566 | Carbamidomethylation | C4:Carbamidomethylation:1000.00 | PEAKS DB |
| R.GLSLPVLDGHVTAF.L | Y | 55.10 | 1424.7664 | 14 | 1.4 | 713.3915 | 2 | 76.68 | 1 | 53109 | 01122020\_RID\_2209\_BuCaKA02.raw | 6.1163E9 | 1 | 1 | 40 | 53 |  |  | PEAKS DB |
| D.GHVTAFLGIPFAEPPVGR.M | Y | 53.85 | 1863.9995 | 18 | 1.0 | 622.3411 | 3 | 65.09 | 1 | 43442 | 01122020\_RID\_2209\_BuCaKA02.raw | 1.6562E7 | 1 | 1 | 48 | 65 |  |  | PEAKS DB |
| R.AQIC(+57.02)AFWNHFLPKLLN.A | Y | 52.39 | 1971.0189 | 16 | 1.1 | 658.0143 | 3 | 78.10 | 1 | 54392 | 01122020\_RID\_2209\_BuCaKA02.raw | 1.1181E8 | 1 | 1 | 541 | 556 | Carbamidomethylation | C4:Carbamidomethylation:1000.00 | PEAKS DB |
| R.AQIC(+57.02)AFWNHFLPKLLNA.T | Y | 52.28 | 2042.0560 | 17 | 0.9 | 1022.0362 | 2 | 80.05 | 1 | 56080 | 01122020\_RID\_2209\_BuCaKA02.raw | 4.8965E8 | 4 | 4 | 541 | 557 | Carbamidomethylation | C4:Carbamidomethylation:1000.00 | PEAKS DB |
| A.QIC(+57.02)AFWNHFLPK.L | N | 49.55 | 1559.7708 | 12 | 1.5 | 780.8938 | 2 | 57.48 | 1 | 37350 | 01122020\_RID\_2209\_BuCaKA02.raw | 2.0913E7 | 1 | 1 | 542 | 553 | Carbamidomethylation | C3:Carbamidomethylation:1000.00 | PEAKS DB |
| R.GLSLPVLDGHVTA.F | Y | 48.82 | 1277.6979 | 13 | 1.7 | 639.8573 | 2 | 62.03 | 1 | 40890 | 01122020\_RID\_2209\_BuCaKA02.raw | 8.2252E7 | 1 | 1 | 40 | 52 |  |  | PEAKS DB |
| R.GLSLPVLDGHVTAFLGIPFAEPPVGRMR.F | Y | 48.68 | 2945.5950 | 28 | 4.5 | 737.4093 | 4 | 85.11 | 1 | 60069 | 01122020\_RID\_2209\_BuCaKA02.raw | 1.6187E7 | 2 | 2 | 40 | 67 |  |  | PEAKS DB |
| L.ALQWIQNNIHPFGGNPR.A | Y | 46.40 | 1961.0020 | 17 | 1.8 | 654.6758 | 3 | 46.23 | 1 | 28435 | 01122020\_RID\_2209\_BuCaKA02.raw | 0 | 0 | 0 | 199 | 215 |  |  | PEAKS DB |
| R.AVTIFGESAGAASVGM(+15.99)HLLSTQSR.T | N | 45.59 | 2405.2009 | 24 | 3.4 | 802.7437 | 3 | 54.96 | 1 | 35227 | 01122020\_RID\_2209\_BuCaKA02.raw | 8.7978E6 | 1 | 1 | 216 | 239 | Oxidation (M) | M16:Oxidation (M):1000.00 | PEAKS DB |
| G.SVRGLSLPVLDGHVTAFLGIPFAEPPVGR.M | Y | 45.26 | 3000.6548 | 29 | 6.1 | 751.1755 | 4 | 83.64 | 1 | 58882 | 01122020\_RID\_2209\_BuCaKA02.raw | 0 | 0 | 0 | 37 | 65 |  |  | PEAKS DB |
| R.AQIC(+57.02)AFWNHFLP.K | N | 44.50 | 1502.7129 | 12 | 0.9 | 752.3644 | 2 | 78.17 | 1 | 54427 | 01122020\_RID\_2209\_BuCaKA02.raw | 5.0431E8 | 1 | 1 | 541 | 552 | Carbamidomethylation | C4:Carbamidomethylation:1000.00 | PEAKS DB |
| K.SGAWPTYTASQPQYVQLNTQPLATQPSLR.A | Y | 43.03 | 3202.6047 | 29 | 0.2 | 1068.5424 | 3 | 65.06 | 1 | 43406 | 01122020\_RID\_2209\_BuCaKA02.raw | 2.1659E8 | 1 | 1 | 512 | 540 |  |  | PEAKS DB |
| R.GLSLPVLDGHVTAFLG.I | Y | 42.79 | 1594.8718 | 16 | 2.7 | 798.4453 | 2 | 85.31 | 1 | 60432 | 01122020\_RID\_2209\_BuCaKA02.raw | 5.2128E8 | 2 | 2 | 40 | 55 |  |  | PEAKS DB |
| L.NTQPLATQPSLRAQIC(+57.02)AFWNHFLPK.L | Y | 42.22 | 2937.5071 | 25 | 4.0 | 735.3870 | 4 | 74.50 | 1 | 51499 | 01122020\_RID\_2209\_BuCaKA02.raw | 2.6172E8 | 1 | 1 | 529 | 553 | Carbamidomethylation | C16:Carbamidomethylation:1000.00 | PEAKS DB |
| R.TGNPTDPADKSGAWPTYTASQPQYVQLNTQPLATQPSLRAQIC(+57.02)AFWNHFLPK.L | Y | 42.13 | 5811.8530 | 52 | 3.1 | 1453.9750 | 4 | 82.20 | 1 | 57744 | 01122020\_RID\_2209\_BuCaKA02.raw | 1.5131E9 | 1 | 1 | 502 | 553 | Carbamidomethylation | C43:Carbamidomethylation:1000.00 | PEAKS DB |
| K.VSTQTGSVRGLSLPVLDGHVTAFLG.I | Y | 41.47 | 2510.3491 | 25 | 1.1 | 837.7913 | 3 | 72.55 | 1 | 49756 | 01122020\_RID\_2209\_BuCaKA02.raw | 2.5098E7 | 1 | 1 | 31 | 55 |  |  | PEAKS DB |
| total 61 peptides |  |  |  |  |  |  |  |  |  |  |  |  |  |  |  |  |  |  |  |
| --- | --- | --- | --- | --- | --- | --- | --- | --- | --- | --- | --- | --- | --- | --- | --- | --- | --- | --- | --- |

T\_55

back to list

  

| Protein Coverage
| Supporting Peptides
|

Protein Coverage:

Supporting Peptides:

| Peptide | Uniq | -10lgP | Mass | Length | ppm | m/z | z | RT | Fraction | Scan | Source File | Area BRom | #Feature | #Feature BRom | Start | End | PTM | AScore | Found By |
| --- | --- | --- | --- | --- | --- | --- | --- | --- | --- | --- | --- | --- | --- | --- | --- | --- | --- | --- | --- |
| R.GLSLPVLDGHVTAFLGIPFAEPPVGR.M | Y | 101.40 | 2658.4534 | 26 | 0.7 | 1330.2349 | 2 | 93.83 | 1 | 67305 | 01122020\_RID\_2209\_BuCaKA02.raw | 5.7352E10 | 14 | 14 | 48 | 73 |  |  | PEAKS DB |
| R.AALLGKQLGC(+57.02)HFNNDSELVSC(+57.02)LR.S | Y | 96.22 | 2601.2791 | 23 | 0.3 | 1301.6472 | 2 | 41.43 | 1 | 24779 | 01122020\_RID\_2209\_BuCaKA02.raw | 1.888E8 | 2 | 2 | 276 | 298 | Carbamidomethylation | C10:Carbamidomethylation:1000.00;C21:Carbamidomethylation:1000.00 | PEAKS DB |
| R.AILQSGGPNAPWATVTPAESR.R | N | 95.10 | 2122.0806 | 21 | -0.9 | 1062.0466 | 2 | 52.50 | 1 | 33145 | 01122020\_RID\_2209\_BuCaKA02.raw | 1.3371E9 | 2 | 2 | 253 | 273 |  |  | PEAKS DB |
| R.EALDDIVGDHNVIC(+57.02)PVVQFANDYAKR.N | N | 93.08 | 2957.4341 | 26 | 0.1 | 740.3659 | 4 | 70.15 | 1 | 47677 | 01122020\_RID\_2209\_BuCaKA02.raw | 1.6502E7 | 3 | 3 | 420 | 445 | Carbamidomethylation | C14:Carbamidomethylation:1000.00 | PEAKS DB |
| R.EALDDIVGDHNVIC(+57.02)PVVQFANDYAK.R | N | 88.62 | 2801.3330 | 25 | 1.0 | 1401.6752 | 2 | 77.11 | 1 | 53666 | 01122020\_RID\_2209\_BuCaKA02.raw | 7.8726E8 | 2 | 2 | 420 | 444 | Carbamidomethylation | C14:Carbamidomethylation:1000.00 | PEAKS DB |
| R.LALQWIQNNIHPFGGNPR.A | Y | 87.29 | 2074.0859 | 18 | 4.4 | 692.3723 | 3 | 60.27 | 1 | 39720 | 01122020\_RID\_2209\_BuCaKA02.raw | 2.2787E8 | 1 | 1 | 206 | 223 |  |  | PEAKS DB |
| R.AVTIFGESAGAASVGMHLLSTQSR.T | N | 85.30 | 2389.2061 | 24 | 0.3 | 797.4095 | 3 | 65.27 | 1 | 44229 | 01122020\_RID\_2209\_BuCaKA02.raw | 4.1007E8 | 3 | 3 | 224 | 247 |  |  | PEAKS DB |
| K.NREALDDIVGDHNVIC(+57.02)PVVQFANDYAK.R | N | 82.20 | 3071.4771 | 27 | 0.7 | 768.8771 | 4 | 65.86 | 1 | 44085 | 01122020\_RID\_2209\_BuCaKA02.raw | 2.8252E8 | 1 | 1 | 418 | 444 | Carbamidomethylation | C16:Carbamidomethylation:1000.00 | PEAKS DB |
| R.AQIC(+57.02)AFWNHFLPK.L | N | 81.68 | 1630.8079 | 13 | 0.4 | 816.4115 | 2 | 60.34 | 1 | 39698 | 01122020\_RID\_2209\_BuCaKA02.raw | 3.6259E8 | 1 | 1 | 549 | 561 | Carbamidomethylation | C4:Carbamidomethylation:1000.00 | PEAKS DB |
| R.AGELKVSTQTGSVRGLSLPVLDGHVTAFLGIPFAEPPVGR.M | Y | 77.17 | 4072.2109 | 40 | 4.9 | 815.4535 | 5 | 78.62 | 1 | 54698 | 01122020\_RID\_2209\_BuCaKA02.raw | 1.683E9 | 3 | 3 | 34 | 73 |  |  | PEAKS DB |
| G.LSLPVLDGHVTAFLGIPFAEPPVGR.M | Y | 75.82 | 2601.4319 | 25 | 1.7 | 868.1527 | 3 | 93.27 | 1 | 66854 | 01122020\_RID\_2209\_BuCaKA02.raw | 9.8181E7 | 1 | 1 | 49 | 73 |  |  | PEAKS DB |
| R.AGELKVSTQTGSVR.G | Y | 75.64 | 1431.7681 | 14 | 0.2 | 716.8914 | 2 | 12.05 | 1 | 2024 | 01122020\_RID\_2209\_BuCaKA02.raw | 2.521E6 | 1 | 1 | 34 | 47 |  |  | PEAKS DB |
| R.TLFQRAILQSGGPNAPWATVTPAESR.R | Y | 75.41 | 2767.4404 | 26 | 0.6 | 923.4880 | 3 | 59.02 | 1 | 38537 | 01122020\_RID\_2209\_BuCaKA02.raw | 9.0758E7 | 1 | 1 | 248 | 273 |  |  | PEAKS DB |
| R.AVTIFGESAGAASVGMHLLSTQSRTLFQR.A | Y | 75.28 | 3034.5657 | 29 | 1.4 | 759.6498 | 4 | 72.55 | 1 | 49639 | 01122020\_RID\_2209\_BuCaKA02.raw | 4.0261E7 | 2 | 2 | 224 | 252 |  |  | PEAKS DB |
| M.GLLDQRLALQWIQNNIHPFGGNPRAVTIFGESAGAASVGMHLLSTQSR.T | Y | 74.87 | 5127.6577 | 48 | 1.0 | 1026.5398 | 5 | 84.83 | 1 | 59856 | 01122020\_RID\_2209\_BuCaKA02.raw | 2.0822E7 | 1 | 1 | 200 | 247 |  |  | PEAKS DB |
| L.KVSTQTGSVRGLSLPVLDGHVTAFLGIPFAEPPVGR.M | Y | 71.73 | 3702.0256 | 36 | 5.4 | 926.5187 | 4 | 75.43 | 1 | 52253 | 01122020\_RID\_2209\_BuCaKA02.raw | 5.6822E8 | 4 | 4 | 38 | 73 |  |  | PEAKS DB |
| D.DIVGDHNVIC(+57.02)PVVQFANDYAK.R | N | 70.32 | 2373.1423 | 21 | 1.2 | 792.0557 | 3 | 67.35 | 1 | 45397 | 01122020\_RID\_2209\_BuCaKA02.raw | 3.7367E7 | 1 | 1 | 424 | 444 | Carbamidomethylation | C10:Carbamidomethylation:1000.00 | PEAKS DB |
| K.VSTQTGSVRGLSLPVLDGHVTAFLGIPFAEPPVGR.M | Y | 70.30 | 3573.9307 | 35 | 9.4 | 715.8001 | 5 | 83.23 | 1 | 58537 | 01122020\_RID\_2209\_BuCaKA02.raw | 1.4423E10 | 7 | 7 | 39 | 73 |  |  | PEAKS DB |
| V.SPGRAGELKVSTQTGSVR.G | Y | 70.11 | 1828.9755 | 18 | 0.0 | 610.6658 | 3 | 12.05 | 1 | 2023 | 01122020\_RID\_2209\_BuCaKA02.raw | 1.7435E6 | 1 | 1 | 30 | 47 |  |  | PEAKS DB |
| R.LALQWIQNNIHPFGGNPRAVTIFGESAGAASVGMHLLSTQSR.T | Y | 70.03 | 4445.2812 | 42 | 1.3 | 890.0647 | 5 | 76.25 | 1 | 52778 | 01122020\_RID\_2209\_BuCaKA02.raw | 6.7471E7 | 3 | 3 | 206 | 247 |  |  | PEAKS DB |
| R.SKNPQELIDEEWSVLPYK.S | Y | 69.96 | 2174.0896 | 18 | 0.8 | 725.7044 | 3 | 63.32 | 1 | 42005 | 01122020\_RID\_2209\_BuCaKA02.raw | 2.4894E7 | 2 | 2 | 299 | 316 |  |  | PEAKS DB |
| M.GLLDQRLALQWIQNNIHPFGGNPR.A | Y | 69.87 | 2756.4622 | 24 | 2.9 | 919.8307 | 3 | 77.23 | 1 | 53582 | 01122020\_RID\_2209\_BuCaKA02.raw | 2.4764E8 | 4 | 4 | 200 | 223 |  |  | PEAKS DB |
| R.AILQSGGPNAPWATVTPAESRR.R | N | 67.72 | 2278.1819 | 22 | 1.4 | 760.4023 | 3 | 39.81 | 1 | 23723 | 01122020\_RID\_2209\_BuCaKA02.raw | 7.6056E7 | 1 | 1 | 253 | 274 |  |  | PEAKS DB |
| R.RAALLGKQLGC(+57.02)HFNNDSELVSC(+57.02)LR.S | Y | 65.73 | 2757.3801 | 24 | 2.0 | 690.3537 | 4 | 33.56 | 1 | 18494 | 01122020\_RID\_2209\_BuCaKA02.raw | 1.5928E7 | 1 | 1 | 275 | 298 | Carbamidomethylation | C11:Carbamidomethylation:1000.00;C22:Carbamidomethylation:1000.00 | PEAKS DB |
| F.LGIPFAEPPVGR.M | N | 65.37 | 1251.6975 | 12 | 1.1 | 626.8567 | 2 | 56.11 | 1 | 36154 | 01122020\_RID\_2209\_BuCaKA02.raw | 4.6223E8 | 1 | 1 | 62 | 73 |  |  | PEAKS DB |
| L.SLPVLDGHVTAFLGIPFAEPPVGR.M | Y | 65.32 | 2488.3477 | 24 | 2.0 | 830.4581 | 3 | 86.79 | 1 | 61552 | 01122020\_RID\_2209\_BuCaKA02.raw | 8.1857E7 | 1 | 1 | 50 | 73 |  |  | PEAKS DB |
| V.STQTGSVRGLSLPVLDGHVTAFLGIPFAEPPVGR.M | Y | 65.11 | 3474.8623 | 34 | 6.8 | 869.7288 | 4 | 82.73 | 1 | 58133 | 01122020\_RID\_2209\_BuCaKA02.raw | 0 | 0 | 0 | 40 | 73 |  |  | PEAKS DB |
| R.TGNPTDPADKSGAWPTYTASQPQYVQLNTQPLATQPSLR.A | Y | 63.72 | 4199.0557 | 39 | 1.2 | 1400.6942 | 3 | 59.70 | 1 | 39174 | 01122020\_RID\_2209\_BuCaKA02.raw | 1.0279E9 | 3 | 3 | 510 | 548 |  |  | PEAKS DB |
| R.GLSLPVLDGHVTAFL.G | Y | 63.72 | 1537.8503 | 15 | 1.7 | 769.9337 | 2 | 87.38 | 1 | 62023 | 01122020\_RID\_2209\_BuCaKA02.raw | 1.6202E7 | 1 | 1 | 48 | 62 |  |  | PEAKS DB |
| K.VSTQTGSVRGLSLPVLDGHVTAF.L | Y | 63.61 | 2340.2437 | 23 | 0.9 | 1171.1301 | 2 | 64.35 | 1 | 42978 | 01122020\_RID\_2209\_BuCaKA02.raw | 5.93E8 | 2 | 2 | 39 | 61 |  |  | PEAKS DB |
| L.PVLDGHVTAFLGIPFAEPPVGR.M | Y | 63.60 | 2288.2317 | 22 | 2.5 | 763.7531 | 3 | 79.31 | 1 | 55332 | 01122020\_RID\_2209\_BuCaKA02.raw | 3.4806E6 | 1 | 1 | 52 | 73 |  |  | PEAKS DB |
| R.TLFQRAILQSGGPNAPWATVTPAESRR.R | Y | 62.58 | 2923.5415 | 27 | -0.7 | 731.8922 | 4 | 49.01 | 1 | 30339 | 01122020\_RID\_2209\_BuCaKA02.raw | 0 | 0 | 0 | 248 | 274 |  |  | PEAKS DB |
| S.LPVLDGHVTAFLGIPFAEPPVGR.M | Y | 62.25 | 2401.3157 | 23 | 2.2 | 801.4476 | 3 | 85.81 | 1 | 60666 | 01122020\_RID\_2209\_BuCaKA02.raw | 1.1292E7 | 1 | 1 | 51 | 73 |  |  | PEAKS DB |
| A.FLGIPFAEPPVGR.M | N | 62.09 | 1398.7659 | 13 | 0.0 | 700.3902 | 2 | 73.18 | 1 | 50153 | 01122020\_RID\_2209\_BuCaKA02.raw | 1.8064E7 | 1 | 1 | 61 | 73 |  |  | PEAKS DB |
| K.QLGC(+57.02)HFNNDSELVSC(+57.02)LR.S | Y | 62.05 | 2047.9204 | 17 | 0.7 | 683.6479 | 3 | 34.95 | 1 | 19633 | 01122020\_RID\_2209\_BuCaKA02.raw | 7.1157E7 | 1 | 1 | 282 | 298 | Carbamidomethylation | C4:Carbamidomethylation:1000.00;C15:Carbamidomethylation:1000.00 | PEAKS DB |
| L.ATQPSLRAQIC(+57.02)AFWNHFLPK.L | Y | 60.92 | 2384.2212 | 20 | 2.7 | 795.7498 | 3 | 71.29 | 1 | 48640 | 01122020\_RID\_2209\_BuCaKA02.raw | 2.1393E7 | 2 | 2 | 542 | 561 | Carbamidomethylation | C11:Carbamidomethylation:1000.00 | PEAKS DB |
| R.AVTIFGESAGAASVGMHLLSTQSRTLFQRAILQSGGPNAPWATVTPAESR.R | Y | 60.82 | 5138.6357 | 50 | 0.4 | 1285.6667 | 4 | 83.73 | 1 | 58887 | 01122020\_RID\_2209\_BuCaKA02.raw | 9.1205E7 | 2 | 2 | 224 | 273 |  |  | PEAKS DB |
| R.AQIC(+57.02)AFWNHFLPKL.L | N | 59.24 | 1743.8918 | 14 | 1.7 | 872.9547 | 2 | 72.95 | 1 | 49964 | 01122020\_RID\_2209\_BuCaKA02.raw | 1.5107E8 | 2 | 2 | 549 | 562 | Carbamidomethylation | C4:Carbamidomethylation:1000.00 | PEAKS DB |
| K.SIFRFPFVPVIDG.D | N | 58.74 | 1492.8077 | 13 | 2.3 | 747.4128 | 2 | 91.00 | 1 | 65018 | 01122020\_RID\_2209\_BuCaKA02.raw | 9.7066E6 | 1 | 1 | 317 | 329 |  |  | PEAKS DB |
| V.RGLSLPVLDGHVTAFLGIPFAEPPVGR.M | Y | 58.04 | 2814.5544 | 27 | 2.6 | 704.6477 | 4 | 82.79 | 1 | 58181 | 01122020\_RID\_2209\_BuCaKA02.raw | 0 | 0 | 0 | 47 | 73 |  |  | PEAKS DB |
| R.AGELKVSTQTGSVRGLSLPVLDGHVTAF.L | Y | 57.78 | 2838.5239 | 28 | 0.1 | 710.6383 | 4 | 60.89 | 1 | 40258 | 01122020\_RID\_2209\_BuCaKA02.raw | 9.4408E8 | 5 | 5 | 34 | 61 |  |  | PEAKS DB |
| D.PADKSGAWPTYTASQPQYVQLNTQPLATQPSLR.A | Y | 56.43 | 3613.8164 | 33 | -0.8 | 1205.6118 | 3 | 56.49 | 1 | 36480 | 01122020\_RID\_2209\_BuCaKA02.raw | 4.7617E7 | 1 | 1 | 516 | 548 |  |  | PEAKS DB |
| Q.IC(+57.02)AFWNHFLPK.L | N | 56.19 | 1431.7122 | 11 | 1.5 | 716.8644 | 2 | 55.11 | 1 | 35292 | 01122020\_RID\_2209\_BuCaKA02.raw | 7.8377E6 | 1 | 1 | 551 | 561 | Carbamidomethylation | C2:Carbamidomethylation:1000.00 | PEAKS DB |
| A.TQPSLRAQIC(+57.02)AFWNHFLPK.L | Y | 56.17 | 2313.1841 | 19 | 0.9 | 579.3038 | 4 | 71.36 | 1 | 48657 | 01122020\_RID\_2209\_BuCaKA02.raw | 9.1707E6 | 2 | 2 | 543 | 561 | Carbamidomethylation | C10:Carbamidomethylation:1000.00 | PEAKS DB |
| R.AQIC(+57.02)AFWNHFLPKLLNATVDPPSADR.R | Y | 56.04 | 2980.5017 | 26 | 1.6 | 746.1339 | 4 | 77.71 | 1 | 54026 | 01122020\_RID\_2209\_BuCaKA02.raw | 0 | 0 | 0 | 549 | 574 | Carbamidomethylation | C4:Carbamidomethylation:1000.00 | PEAKS DB |
| R.GLSLPVLDGHVTAF.L | Y | 55.10 | 1424.7664 | 14 | 1.4 | 713.3915 | 2 | 76.68 | 1 | 53109 | 01122020\_RID\_2209\_BuCaKA02.raw | 6.1163E9 | 1 | 1 | 48 | 61 |  |  | PEAKS DB |
| D.GHVTAFLGIPFAEPPVGR.M | Y | 53.85 | 1863.9995 | 18 | 1.0 | 622.3411 | 3 | 65.09 | 1 | 43442 | 01122020\_RID\_2209\_BuCaKA02.raw | 1.6562E7 | 1 | 1 | 56 | 73 |  |  | PEAKS DB |
| R.AQIC(+57.02)AFWNHFLPKLLN.A | Y | 52.39 | 1971.0189 | 16 | 1.1 | 658.0143 | 3 | 78.10 | 1 | 54392 | 01122020\_RID\_2209\_BuCaKA02.raw | 1.1181E8 | 1 | 1 | 549 | 564 | Carbamidomethylation | C4:Carbamidomethylation:1000.00 | PEAKS DB |
| R.AQIC(+57.02)AFWNHFLPKLLNA.T | Y | 52.28 | 2042.0560 | 17 | 0.9 | 1022.0362 | 2 | 80.05 | 1 | 56080 | 01122020\_RID\_2209\_BuCaKA02.raw | 4.8965E8 | 4 | 4 | 549 | 565 | Carbamidomethylation | C4:Carbamidomethylation:1000.00 | PEAKS DB |
| A.QIC(+57.02)AFWNHFLPK.L | N | 49.55 | 1559.7708 | 12 | 1.5 | 780.8938 | 2 | 57.48 | 1 | 37350 | 01122020\_RID\_2209\_BuCaKA02.raw | 2.0913E7 | 1 | 1 | 550 | 561 | Carbamidomethylation | C3:Carbamidomethylation:1000.00 | PEAKS DB |
| R.GLSLPVLDGHVTA.F | Y | 48.82 | 1277.6979 | 13 | 1.7 | 639.8573 | 2 | 62.03 | 1 | 40890 | 01122020\_RID\_2209\_BuCaKA02.raw | 8.2252E7 | 1 | 1 | 48 | 60 |  |  | PEAKS DB |
| R.GLSLPVLDGHVTAFLGIPFAEPPVGRMR.F | Y | 48.68 | 2945.5950 | 28 | 4.5 | 737.4093 | 4 | 85.11 | 1 | 60069 | 01122020\_RID\_2209\_BuCaKA02.raw | 1.6187E7 | 2 | 2 | 48 | 75 |  |  | PEAKS DB |
| L.ALQWIQNNIHPFGGNPR.A | Y | 46.40 | 1961.0020 | 17 | 1.8 | 654.6758 | 3 | 46.23 | 1 | 28435 | 01122020\_RID\_2209\_BuCaKA02.raw | 0 | 0 | 0 | 207 | 223 |  |  | PEAKS DB |
| R.AVTIFGESAGAASVGM(+15.99)HLLSTQSR.T | N | 45.59 | 2405.2009 | 24 | 3.4 | 802.7437 | 3 | 54.96 | 1 | 35227 | 01122020\_RID\_2209\_BuCaKA02.raw | 8.7978E6 | 1 | 1 | 224 | 247 | Oxidation (M) | M16:Oxidation (M):1000.00 | PEAKS DB |
| G.SVRGLSLPVLDGHVTAFLGIPFAEPPVGR.M | Y | 45.26 | 3000.6548 | 29 | 6.1 | 751.1755 | 4 | 83.64 | 1 | 58882 | 01122020\_RID\_2209\_BuCaKA02.raw | 0 | 0 | 0 | 45 | 73 |  |  | PEAKS DB |
| R.AQIC(+57.02)AFWNHFLP.K | N | 44.50 | 1502.7129 | 12 | 0.9 | 752.3644 | 2 | 78.17 | 1 | 54427 | 01122020\_RID\_2209\_BuCaKA02.raw | 5.0431E8 | 1 | 1 | 549 | 560 | Carbamidomethylation | C4:Carbamidomethylation:1000.00 | PEAKS DB |
| K.SGAWPTYTASQPQYVQLNTQPLATQPSLR.A | Y | 43.03 | 3202.6047 | 29 | 0.2 | 1068.5424 | 3 | 65.06 | 1 | 43406 | 01122020\_RID\_2209\_BuCaKA02.raw | 2.1659E8 | 1 | 1 | 520 | 548 |  |  | PEAKS DB |
| R.GLSLPVLDGHVTAFLG.I | Y | 42.79 | 1594.8718 | 16 | 2.7 | 798.4453 | 2 | 85.31 | 1 | 60432 | 01122020\_RID\_2209\_BuCaKA02.raw | 5.2128E8 | 2 | 2 | 48 | 63 |  |  | PEAKS DB |
| L.NTQPLATQPSLRAQIC(+57.02)AFWNHFLPK.L | Y | 42.22 | 2937.5071 | 25 | 4.0 | 735.3870 | 4 | 74.50 | 1 | 51499 | 01122020\_RID\_2209\_BuCaKA02.raw | 2.6172E8 | 1 | 1 | 537 | 561 | Carbamidomethylation | C16:Carbamidomethylation:1000.00 | PEAKS DB |
| R.TGNPTDPADKSGAWPTYTASQPQYVQLNTQPLATQPSLRAQIC(+57.02)AFWNHFLPK.L | Y | 42.13 | 5811.8530 | 52 | 3.1 | 1453.9750 | 4 | 82.20 | 1 | 57744 | 01122020\_RID\_2209\_BuCaKA02.raw | 1.5131E9 | 1 | 1 | 510 | 561 | Carbamidomethylation | C43:Carbamidomethylation:1000.00 | PEAKS DB |
| K.VSTQTGSVRGLSLPVLDGHVTAFLG.I | Y | 41.47 | 2510.3491 | 25 | 1.1 | 837.7913 | 3 | 72.55 | 1 | 49756 | 01122020\_RID\_2209\_BuCaKA02.raw | 2.5098E7 | 1 | 1 | 39 | 63 |  |  | PEAKS DB |
| total 61 peptides |  |  |  |  |  |  |  |  |  |  |  |  |  |  |  |  |  |  |  |
| --- | --- | --- | --- | --- | --- | --- | --- | --- | --- | --- | --- | --- | --- | --- | --- | --- | --- | --- | --- |

T\_59

back to list

  

| Protein Coverage
| Supporting Peptides
|

Protein Coverage:

Supporting Peptides:

| Peptide | Uniq | -10lgP | Mass | Length | ppm | m/z | z | RT | Fraction | Scan | Source File | Area BRom | #Feature | #Feature BRom | Start | End | PTM | AScore | Found By |
| --- | --- | --- | --- | --- | --- | --- | --- | --- | --- | --- | --- | --- | --- | --- | --- | --- | --- | --- | --- |
| R.GLSLPVLDGHVTAFLGIPFAEPPVGR.M | Y | 101.40 | 2658.4534 | 26 | 0.7 | 1330.2349 | 2 | 93.83 | 1 | 67305 | 01122020\_RID\_2209\_BuCaKA02.raw | 5.7352E10 | 14 | 14 | 48 | 73 |  |  | PEAKS DB |
| R.AALLGKQLGC(+57.02)HFNNDSELVSC(+57.02)LR.S | Y | 96.22 | 2601.2791 | 23 | 0.3 | 1301.6472 | 2 | 41.43 | 1 | 24779 | 01122020\_RID\_2209\_BuCaKA02.raw | 1.888E8 | 2 | 2 | 276 | 298 | Carbamidomethylation | C10:Carbamidomethylation:1000.00;C21:Carbamidomethylation:1000.00 | PEAKS DB |
| R.AILQSGGPNAPWATVTPAESR.R | N | 95.10 | 2122.0806 | 21 | -0.9 | 1062.0466 | 2 | 52.50 | 1 | 33145 | 01122020\_RID\_2209\_BuCaKA02.raw | 1.3371E9 | 2 | 2 | 253 | 273 |  |  | PEAKS DB |
| R.EALDDIVGDHNVIC(+57.02)PVVQFANDYAKR.N | N | 93.08 | 2957.4341 | 26 | 0.1 | 740.3659 | 4 | 70.15 | 1 | 47677 | 01122020\_RID\_2209\_BuCaKA02.raw | 1.6502E7 | 3 | 3 | 420 | 445 | Carbamidomethylation | C14:Carbamidomethylation:1000.00 | PEAKS DB |
| R.EALDDIVGDHNVIC(+57.02)PVVQFANDYAK.R | N | 88.62 | 2801.3330 | 25 | 1.0 | 1401.6752 | 2 | 77.11 | 1 | 53666 | 01122020\_RID\_2209\_BuCaKA02.raw | 7.8726E8 | 2 | 2 | 420 | 444 | Carbamidomethylation | C14:Carbamidomethylation:1000.00 | PEAKS DB |
| R.LALQWIQNNIHPFGGNPR.A | Y | 87.29 | 2074.0859 | 18 | 4.4 | 692.3723 | 3 | 60.27 | 1 | 39720 | 01122020\_RID\_2209\_BuCaKA02.raw | 2.2787E8 | 1 | 1 | 206 | 223 |  |  | PEAKS DB |
| R.AVTIFGESAGAASVGMHLLSTQSR.T | N | 85.30 | 2389.2061 | 24 | 0.3 | 797.4095 | 3 | 65.27 | 1 | 44229 | 01122020\_RID\_2209\_BuCaKA02.raw | 4.1007E8 | 3 | 3 | 224 | 247 |  |  | PEAKS DB |
| K.NREALDDIVGDHNVIC(+57.02)PVVQFANDYAK.R | N | 82.20 | 3071.4771 | 27 | 0.7 | 768.8771 | 4 | 65.86 | 1 | 44085 | 01122020\_RID\_2209\_BuCaKA02.raw | 2.8252E8 | 1 | 1 | 418 | 444 | Carbamidomethylation | C16:Carbamidomethylation:1000.00 | PEAKS DB |
| R.AQIC(+57.02)AFWNHFLPK.L | N | 81.68 | 1630.8079 | 13 | 0.4 | 816.4115 | 2 | 60.34 | 1 | 39698 | 01122020\_RID\_2209\_BuCaKA02.raw | 3.6259E8 | 1 | 1 | 549 | 561 | Carbamidomethylation | C4:Carbamidomethylation:1000.00 | PEAKS DB |
| R.AGELKVSTQTGSVRGLSLPVLDGHVTAFLGIPFAEPPVGR.M | Y | 77.17 | 4072.2109 | 40 | 4.9 | 815.4535 | 5 | 78.62 | 1 | 54698 | 01122020\_RID\_2209\_BuCaKA02.raw | 1.683E9 | 3 | 3 | 34 | 73 |  |  | PEAKS DB |
| G.LSLPVLDGHVTAFLGIPFAEPPVGR.M | Y | 75.82 | 2601.4319 | 25 | 1.7 | 868.1527 | 3 | 93.27 | 1 | 66854 | 01122020\_RID\_2209\_BuCaKA02.raw | 9.8181E7 | 1 | 1 | 49 | 73 |  |  | PEAKS DB |
| R.AGELKVSTQTGSVR.G | Y | 75.64 | 1431.7681 | 14 | 0.2 | 716.8914 | 2 | 12.05 | 1 | 2024 | 01122020\_RID\_2209\_BuCaKA02.raw | 2.521E6 | 1 | 1 | 34 | 47 |  |  | PEAKS DB |
| R.TLFQRAILQSGGPNAPWATVTPAESR.R | Y | 75.41 | 2767.4404 | 26 | 0.6 | 923.4880 | 3 | 59.02 | 1 | 38537 | 01122020\_RID\_2209\_BuCaKA02.raw | 9.0758E7 | 1 | 1 | 248 | 273 |  |  | PEAKS DB |
| R.AVTIFGESAGAASVGMHLLSTQSRTLFQR.A | Y | 75.28 | 3034.5657 | 29 | 1.4 | 759.6498 | 4 | 72.55 | 1 | 49639 | 01122020\_RID\_2209\_BuCaKA02.raw | 4.0261E7 | 2 | 2 | 224 | 252 |  |  | PEAKS DB |
| M.GLLDQRLALQWIQNNIHPFGGNPRAVTIFGESAGAASVGMHLLSTQSR.T | Y | 74.87 | 5127.6577 | 48 | 1.0 | 1026.5398 | 5 | 84.83 | 1 | 59856 | 01122020\_RID\_2209\_BuCaKA02.raw | 2.0822E7 | 1 | 1 | 200 | 247 |  |  | PEAKS DB |
| L.KVSTQTGSVRGLSLPVLDGHVTAFLGIPFAEPPVGR.M | Y | 71.73 | 3702.0256 | 36 | 5.4 | 926.5187 | 4 | 75.43 | 1 | 52253 | 01122020\_RID\_2209\_BuCaKA02.raw | 5.6822E8 | 4 | 4 | 38 | 73 |  |  | PEAKS DB |
| D.DIVGDHNVIC(+57.02)PVVQFANDYAK.R | N | 70.32 | 2373.1423 | 21 | 1.2 | 792.0557 | 3 | 67.35 | 1 | 45397 | 01122020\_RID\_2209\_BuCaKA02.raw | 3.7367E7 | 1 | 1 | 424 | 444 | Carbamidomethylation | C10:Carbamidomethylation:1000.00 | PEAKS DB |
| K.VSTQTGSVRGLSLPVLDGHVTAFLGIPFAEPPVGR.M | Y | 70.30 | 3573.9307 | 35 | 9.4 | 715.8001 | 5 | 83.23 | 1 | 58537 | 01122020\_RID\_2209\_BuCaKA02.raw | 1.4423E10 | 7 | 7 | 39 | 73 |  |  | PEAKS DB |
| V.SPGRAGELKVSTQTGSVR.G | Y | 70.11 | 1828.9755 | 18 | 0.0 | 610.6658 | 3 | 12.05 | 1 | 2023 | 01122020\_RID\_2209\_BuCaKA02.raw | 1.7435E6 | 1 | 1 | 30 | 47 |  |  | PEAKS DB |
| R.LALQWIQNNIHPFGGNPRAVTIFGESAGAASVGMHLLSTQSR.T | Y | 70.03 | 4445.2812 | 42 | 1.3 | 890.0647 | 5 | 76.25 | 1 | 52778 | 01122020\_RID\_2209\_BuCaKA02.raw | 6.7471E7 | 3 | 3 | 206 | 247 |  |  | PEAKS DB |
| R.SKNPQELIDEEWSVLPYK.S | Y | 69.96 | 2174.0896 | 18 | 0.8 | 725.7044 | 3 | 63.32 | 1 | 42005 | 01122020\_RID\_2209\_BuCaKA02.raw | 2.4894E7 | 2 | 2 | 299 | 316 |  |  | PEAKS DB |
| M.GLLDQRLALQWIQNNIHPFGGNPR.A | Y | 69.87 | 2756.4622 | 24 | 2.9 | 919.8307 | 3 | 77.23 | 1 | 53582 | 01122020\_RID\_2209\_BuCaKA02.raw | 2.4764E8 | 4 | 4 | 200 | 223 |  |  | PEAKS DB |
| R.AILQSGGPNAPWATVTPAESRR.R | N | 67.72 | 2278.1819 | 22 | 1.4 | 760.4023 | 3 | 39.81 | 1 | 23723 | 01122020\_RID\_2209\_BuCaKA02.raw | 7.6056E7 | 1 | 1 | 253 | 274 |  |  | PEAKS DB |
| R.RAALLGKQLGC(+57.02)HFNNDSELVSC(+57.02)LR.S | Y | 65.73 | 2757.3801 | 24 | 2.0 | 690.3537 | 4 | 33.56 | 1 | 18494 | 01122020\_RID\_2209\_BuCaKA02.raw | 1.5928E7 | 1 | 1 | 275 | 298 | Carbamidomethylation | C11:Carbamidomethylation:1000.00;C22:Carbamidomethylation:1000.00 | PEAKS DB |
| F.LGIPFAEPPVGR.M | N | 65.37 | 1251.6975 | 12 | 1.1 | 626.8567 | 2 | 56.11 | 1 | 36154 | 01122020\_RID\_2209\_BuCaKA02.raw | 4.6223E8 | 1 | 1 | 62 | 73 |  |  | PEAKS DB |
| L.SLPVLDGHVTAFLGIPFAEPPVGR.M | Y | 65.32 | 2488.3477 | 24 | 2.0 | 830.4581 | 3 | 86.79 | 1 | 61552 | 01122020\_RID\_2209\_BuCaKA02.raw | 8.1857E7 | 1 | 1 | 50 | 73 |  |  | PEAKS DB |
| V.STQTGSVRGLSLPVLDGHVTAFLGIPFAEPPVGR.M | Y | 65.11 | 3474.8623 | 34 | 6.8 | 869.7288 | 4 | 82.73 | 1 | 58133 | 01122020\_RID\_2209\_BuCaKA02.raw | 0 | 0 | 0 | 40 | 73 |  |  | PEAKS DB |
| R.TGNPTDPADKSGAWPTYTASQPQYVQLNTQPLATQPSLR.A | Y | 63.72 | 4199.0557 | 39 | 1.2 | 1400.6942 | 3 | 59.70 | 1 | 39174 | 01122020\_RID\_2209\_BuCaKA02.raw | 1.0279E9 | 3 | 3 | 510 | 548 |  |  | PEAKS DB |
| R.GLSLPVLDGHVTAFL.G | Y | 63.72 | 1537.8503 | 15 | 1.7 | 769.9337 | 2 | 87.38 | 1 | 62023 | 01122020\_RID\_2209\_BuCaKA02.raw | 1.6202E7 | 1 | 1 | 48 | 62 |  |  | PEAKS DB |
| K.VSTQTGSVRGLSLPVLDGHVTAF.L | Y | 63.61 | 2340.2437 | 23 | 0.9 | 1171.1301 | 2 | 64.35 | 1 | 42978 | 01122020\_RID\_2209\_BuCaKA02.raw | 5.93E8 | 2 | 2 | 39 | 61 |  |  | PEAKS DB |
| L.PVLDGHVTAFLGIPFAEPPVGR.M | Y | 63.60 | 2288.2317 | 22 | 2.5 | 763.7531 | 3 | 79.31 | 1 | 55332 | 01122020\_RID\_2209\_BuCaKA02.raw | 3.4806E6 | 1 | 1 | 52 | 73 |  |  | PEAKS DB |
| R.TLFQRAILQSGGPNAPWATVTPAESRR.R | Y | 62.58 | 2923.5415 | 27 | -0.7 | 731.8922 | 4 | 49.01 | 1 | 30339 | 01122020\_RID\_2209\_BuCaKA02.raw | 0 | 0 | 0 | 248 | 274 |  |  | PEAKS DB |
| S.LPVLDGHVTAFLGIPFAEPPVGR.M | Y | 62.25 | 2401.3157 | 23 | 2.2 | 801.4476 | 3 | 85.81 | 1 | 60666 | 01122020\_RID\_2209\_BuCaKA02.raw | 1.1292E7 | 1 | 1 | 51 | 73 |  |  | PEAKS DB |
| A.FLGIPFAEPPVGR.M | N | 62.09 | 1398.7659 | 13 | 0.0 | 700.3902 | 2 | 73.18 | 1 | 50153 | 01122020\_RID\_2209\_BuCaKA02.raw | 1.8064E7 | 1 | 1 | 61 | 73 |  |  | PEAKS DB |
| K.QLGC(+57.02)HFNNDSELVSC(+57.02)LR.S | Y | 62.05 | 2047.9204 | 17 | 0.7 | 683.6479 | 3 | 34.95 | 1 | 19633 | 01122020\_RID\_2209\_BuCaKA02.raw | 7.1157E7 | 1 | 1 | 282 | 298 | Carbamidomethylation | C4:Carbamidomethylation:1000.00;C15:Carbamidomethylation:1000.00 | PEAKS DB |
| L.ATQPSLRAQIC(+57.02)AFWNHFLPK.L | Y | 60.92 | 2384.2212 | 20 | 2.7 | 795.7498 | 3 | 71.29 | 1 | 48640 | 01122020\_RID\_2209\_BuCaKA02.raw | 2.1393E7 | 2 | 2 | 542 | 561 | Carbamidomethylation | C11:Carbamidomethylation:1000.00 | PEAKS DB |
| R.AVTIFGESAGAASVGMHLLSTQSRTLFQRAILQSGGPNAPWATVTPAESR.R | Y | 60.82 | 5138.6357 | 50 | 0.4 | 1285.6667 | 4 | 83.73 | 1 | 58887 | 01122020\_RID\_2209\_BuCaKA02.raw | 9.1205E7 | 2 | 2 | 224 | 273 |  |  | PEAKS DB |
| R.AQIC(+57.02)AFWNHFLPKL.L | N | 59.24 | 1743.8918 | 14 | 1.7 | 872.9547 | 2 | 72.95 | 1 | 49964 | 01122020\_RID\_2209\_BuCaKA02.raw | 1.5107E8 | 2 | 2 | 549 | 562 | Carbamidomethylation | C4:Carbamidomethylation:1000.00 | PEAKS DB |
| K.SIFRFPFVPVIDG.D | N | 58.74 | 1492.8077 | 13 | 2.3 | 747.4128 | 2 | 91.00 | 1 | 65018 | 01122020\_RID\_2209\_BuCaKA02.raw | 9.7066E6 | 1 | 1 | 317 | 329 |  |  | PEAKS DB |
| V.RGLSLPVLDGHVTAFLGIPFAEPPVGR.M | Y | 58.04 | 2814.5544 | 27 | 2.6 | 704.6477 | 4 | 82.79 | 1 | 58181 | 01122020\_RID\_2209\_BuCaKA02.raw | 0 | 0 | 0 | 47 | 73 |  |  | PEAKS DB |
| R.AGELKVSTQTGSVRGLSLPVLDGHVTAF.L | Y | 57.78 | 2838.5239 | 28 | 0.1 | 710.6383 | 4 | 60.89 | 1 | 40258 | 01122020\_RID\_2209\_BuCaKA02.raw | 9.4408E8 | 5 | 5 | 34 | 61 |  |  | PEAKS DB |
| D.PADKSGAWPTYTASQPQYVQLNTQPLATQPSLR.A | Y | 56.43 | 3613.8164 | 33 | -0.8 | 1205.6118 | 3 | 56.49 | 1 | 36480 | 01122020\_RID\_2209\_BuCaKA02.raw | 4.7617E7 | 1 | 1 | 516 | 548 |  |  | PEAKS DB |
| Q.IC(+57.02)AFWNHFLPK.L | N | 56.19 | 1431.7122 | 11 | 1.5 | 716.8644 | 2 | 55.11 | 1 | 35292 | 01122020\_RID\_2209\_BuCaKA02.raw | 7.8377E6 | 1 | 1 | 551 | 561 | Carbamidomethylation | C2:Carbamidomethylation:1000.00 | PEAKS DB |
| A.TQPSLRAQIC(+57.02)AFWNHFLPK.L | Y | 56.17 | 2313.1841 | 19 | 0.9 | 579.3038 | 4 | 71.36 | 1 | 48657 | 01122020\_RID\_2209\_BuCaKA02.raw | 9.1707E6 | 2 | 2 | 543 | 561 | Carbamidomethylation | C10:Carbamidomethylation:1000.00 | PEAKS DB |
| R.AQIC(+57.02)AFWNHFLPKLLNATVDPPSADR.R | Y | 56.04 | 2980.5017 | 26 | 1.6 | 746.1339 | 4 | 77.71 | 1 | 54026 | 01122020\_RID\_2209\_BuCaKA02.raw | 0 | 0 | 0 | 549 | 574 | Carbamidomethylation | C4:Carbamidomethylation:1000.00 | PEAKS DB |
| R.GLSLPVLDGHVTAF.L | Y | 55.10 | 1424.7664 | 14 | 1.4 | 713.3915 | 2 | 76.68 | 1 | 53109 | 01122020\_RID\_2209\_BuCaKA02.raw | 6.1163E9 | 1 | 1 | 48 | 61 |  |  | PEAKS DB |
| D.GHVTAFLGIPFAEPPVGR.M | Y | 53.85 | 1863.9995 | 18 | 1.0 | 622.3411 | 3 | 65.09 | 1 | 43442 | 01122020\_RID\_2209\_BuCaKA02.raw | 1.6562E7 | 1 | 1 | 56 | 73 |  |  | PEAKS DB |
| R.AQIC(+57.02)AFWNHFLPKLLN.A | Y | 52.39 | 1971.0189 | 16 | 1.1 | 658.0143 | 3 | 78.10 | 1 | 54392 | 01122020\_RID\_2209\_BuCaKA02.raw | 1.1181E8 | 1 | 1 | 549 | 564 | Carbamidomethylation | C4:Carbamidomethylation:1000.00 | PEAKS DB |
| R.AQIC(+57.02)AFWNHFLPKLLNA.T | Y | 52.28 | 2042.0560 | 17 | 0.9 | 1022.0362 | 2 | 80.05 | 1 | 56080 | 01122020\_RID\_2209\_BuCaKA02.raw | 4.8965E8 | 4 | 4 | 549 | 565 | Carbamidomethylation | C4:Carbamidomethylation:1000.00 | PEAKS DB |
| A.QIC(+57.02)AFWNHFLPK.L | N | 49.55 | 1559.7708 | 12 | 1.5 | 780.8938 | 2 | 57.48 | 1 | 37350 | 01122020\_RID\_2209\_BuCaKA02.raw | 2.0913E7 | 1 | 1 | 550 | 561 | Carbamidomethylation | C3:Carbamidomethylation:1000.00 | PEAKS DB |
| R.GLSLPVLDGHVTA.F | Y | 48.82 | 1277.6979 | 13 | 1.7 | 639.8573 | 2 | 62.03 | 1 | 40890 | 01122020\_RID\_2209\_BuCaKA02.raw | 8.2252E7 | 1 | 1 | 48 | 60 |  |  | PEAKS DB |
| R.GLSLPVLDGHVTAFLGIPFAEPPVGRMR.F | Y | 48.68 | 2945.5950 | 28 | 4.5 | 737.4093 | 4 | 85.11 | 1 | 60069 | 01122020\_RID\_2209\_BuCaKA02.raw | 1.6187E7 | 2 | 2 | 48 | 75 |  |  | PEAKS DB |
| L.ALQWIQNNIHPFGGNPR.A | Y | 46.40 | 1961.0020 | 17 | 1.8 | 654.6758 | 3 | 46.23 | 1 | 28435 | 01122020\_RID\_2209\_BuCaKA02.raw | 0 | 0 | 0 | 207 | 223 |  |  | PEAKS DB |
| R.AVTIFGESAGAASVGM(+15.99)HLLSTQSR.T | N | 45.59 | 2405.2009 | 24 | 3.4 | 802.7437 | 3 | 54.96 | 1 | 35227 | 01122020\_RID\_2209\_BuCaKA02.raw | 8.7978E6 | 1 | 1 | 224 | 247 | Oxidation (M) | M16:Oxidation (M):1000.00 | PEAKS DB |
| G.SVRGLSLPVLDGHVTAFLGIPFAEPPVGR.M | Y | 45.26 | 3000.6548 | 29 | 6.1 | 751.1755 | 4 | 83.64 | 1 | 58882 | 01122020\_RID\_2209\_BuCaKA02.raw | 0 | 0 | 0 | 45 | 73 |  |  | PEAKS DB |
| R.AQIC(+57.02)AFWNHFLP.K | N | 44.50 | 1502.7129 | 12 | 0.9 | 752.3644 | 2 | 78.17 | 1 | 54427 | 01122020\_RID\_2209\_BuCaKA02.raw | 5.0431E8 | 1 | 1 | 549 | 560 | Carbamidomethylation | C4:Carbamidomethylation:1000.00 | PEAKS DB |
| K.SGAWPTYTASQPQYVQLNTQPLATQPSLR.A | Y | 43.03 | 3202.6047 | 29 | 0.2 | 1068.5424 | 3 | 65.06 | 1 | 43406 | 01122020\_RID\_2209\_BuCaKA02.raw | 2.1659E8 | 1 | 1 | 520 | 548 |  |  | PEAKS DB |
| R.GLSLPVLDGHVTAFLG.I | Y | 42.79 | 1594.8718 | 16 | 2.7 | 798.4453 | 2 | 85.31 | 1 | 60432 | 01122020\_RID\_2209\_BuCaKA02.raw | 5.2128E8 | 2 | 2 | 48 | 63 |  |  | PEAKS DB |
| L.NTQPLATQPSLRAQIC(+57.02)AFWNHFLPK.L | Y | 42.22 | 2937.5071 | 25 | 4.0 | 735.3870 | 4 | 74.50 | 1 | 51499 | 01122020\_RID\_2209\_BuCaKA02.raw | 2.6172E8 | 1 | 1 | 537 | 561 | Carbamidomethylation | C16:Carbamidomethylation:1000.00 | PEAKS DB |
| R.TGNPTDPADKSGAWPTYTASQPQYVQLNTQPLATQPSLRAQIC(+57.02)AFWNHFLPK.L | Y | 42.13 | 5811.8530 | 52 | 3.1 | 1453.9750 | 4 | 82.20 | 1 | 57744 | 01122020\_RID\_2209\_BuCaKA02.raw | 1.5131E9 | 1 | 1 | 510 | 561 | Carbamidomethylation | C43:Carbamidomethylation:1000.00 | PEAKS DB |
| K.VSTQTGSVRGLSLPVLDGHVTAFLG.I | Y | 41.47 | 2510.3491 | 25 | 1.1 | 837.7913 | 3 | 72.55 | 1 | 49756 | 01122020\_RID\_2209\_BuCaKA02.raw | 2.5098E7 | 1 | 1 | 39 | 63 |  |  | PEAKS DB |
| total 61 peptides |  |  |  |  |  |  |  |  |  |  |  |  |  |  |  |  |  |  |  |
| --- | --- | --- | --- | --- | --- | --- | --- | --- | --- | --- | --- | --- | --- | --- | --- | --- | --- | --- | --- |

T\_58

back to list

  

| Protein Coverage
| Supporting Peptides
|

Protein Coverage:

Supporting Peptides:

| Peptide | Uniq | -10lgP | Mass | Length | ppm | m/z | z | RT | Fraction | Scan | Source File | Area BRom | #Feature | #Feature BRom | Start | End | PTM | AScore | Found By |
| --- | --- | --- | --- | --- | --- | --- | --- | --- | --- | --- | --- | --- | --- | --- | --- | --- | --- | --- | --- |
| R.GLSLPVLDGHVTAFLGIPFAEPPVGR.M | Y | 101.40 | 2658.4534 | 26 | 0.7 | 1330.2349 | 2 | 93.83 | 1 | 67305 | 01122020\_RID\_2209\_BuCaKA02.raw | 5.7352E10 | 14 | 14 | 48 | 73 |  |  | PEAKS DB |
| R.AALLGKQLGC(+57.02)HFNNDSELVSC(+57.02)LR.S | Y | 96.22 | 2601.2791 | 23 | 0.3 | 1301.6472 | 2 | 41.43 | 1 | 24779 | 01122020\_RID\_2209\_BuCaKA02.raw | 1.888E8 | 2 | 2 | 276 | 298 | Carbamidomethylation | C10:Carbamidomethylation:1000.00;C21:Carbamidomethylation:1000.00 | PEAKS DB |
| R.AILQSGGPNAPWATVTPAESR.R | N | 95.10 | 2122.0806 | 21 | -0.9 | 1062.0466 | 2 | 52.50 | 1 | 33145 | 01122020\_RID\_2209\_BuCaKA02.raw | 1.3371E9 | 2 | 2 | 253 | 273 |  |  | PEAKS DB |
| R.EALDDIVGDHNVIC(+57.02)PVVQFANDYAKR.N | N | 93.08 | 2957.4341 | 26 | 0.1 | 740.3659 | 4 | 70.15 | 1 | 47677 | 01122020\_RID\_2209\_BuCaKA02.raw | 1.6502E7 | 3 | 3 | 420 | 445 | Carbamidomethylation | C14:Carbamidomethylation:1000.00 | PEAKS DB |
| R.EALDDIVGDHNVIC(+57.02)PVVQFANDYAK.R | N | 88.62 | 2801.3330 | 25 | 1.0 | 1401.6752 | 2 | 77.11 | 1 | 53666 | 01122020\_RID\_2209\_BuCaKA02.raw | 7.8726E8 | 2 | 2 | 420 | 444 | Carbamidomethylation | C14:Carbamidomethylation:1000.00 | PEAKS DB |
| R.LALQWIQNNIHPFGGNPR.A | Y | 87.29 | 2074.0859 | 18 | 4.4 | 692.3723 | 3 | 60.27 | 1 | 39720 | 01122020\_RID\_2209\_BuCaKA02.raw | 2.2787E8 | 1 | 1 | 206 | 223 |  |  | PEAKS DB |
| R.AVTIFGESAGAASVGMHLLSTQSR.T | N | 85.30 | 2389.2061 | 24 | 0.3 | 797.4095 | 3 | 65.27 | 1 | 44229 | 01122020\_RID\_2209\_BuCaKA02.raw | 4.1007E8 | 3 | 3 | 224 | 247 |  |  | PEAKS DB |
| K.NREALDDIVGDHNVIC(+57.02)PVVQFANDYAK.R | N | 82.20 | 3071.4771 | 27 | 0.7 | 768.8771 | 4 | 65.86 | 1 | 44085 | 01122020\_RID\_2209\_BuCaKA02.raw | 2.8252E8 | 1 | 1 | 418 | 444 | Carbamidomethylation | C16:Carbamidomethylation:1000.00 | PEAKS DB |
| R.AQIC(+57.02)AFWNHFLPK.L | N | 81.68 | 1630.8079 | 13 | 0.4 | 816.4115 | 2 | 60.34 | 1 | 39698 | 01122020\_RID\_2209\_BuCaKA02.raw | 3.6259E8 | 1 | 1 | 549 | 561 | Carbamidomethylation | C4:Carbamidomethylation:1000.00 | PEAKS DB |
| R.AGELKVSTQTGSVRGLSLPVLDGHVTAFLGIPFAEPPVGR.M | Y | 77.17 | 4072.2109 | 40 | 4.9 | 815.4535 | 5 | 78.62 | 1 | 54698 | 01122020\_RID\_2209\_BuCaKA02.raw | 1.683E9 | 3 | 3 | 34 | 73 |  |  | PEAKS DB |
| G.LSLPVLDGHVTAFLGIPFAEPPVGR.M | Y | 75.82 | 2601.4319 | 25 | 1.7 | 868.1527 | 3 | 93.27 | 1 | 66854 | 01122020\_RID\_2209\_BuCaKA02.raw | 9.8181E7 | 1 | 1 | 49 | 73 |  |  | PEAKS DB |
| R.AGELKVSTQTGSVR.G | Y | 75.64 | 1431.7681 | 14 | 0.2 | 716.8914 | 2 | 12.05 | 1 | 2024 | 01122020\_RID\_2209\_BuCaKA02.raw | 2.521E6 | 1 | 1 | 34 | 47 |  |  | PEAKS DB |
| R.TLFQRAILQSGGPNAPWATVTPAESR.R | Y | 75.41 | 2767.4404 | 26 | 0.6 | 923.4880 | 3 | 59.02 | 1 | 38537 | 01122020\_RID\_2209\_BuCaKA02.raw | 9.0758E7 | 1 | 1 | 248 | 273 |  |  | PEAKS DB |
| R.AVTIFGESAGAASVGMHLLSTQSRTLFQR.A | Y | 75.28 | 3034.5657 | 29 | 1.4 | 759.6498 | 4 | 72.55 | 1 | 49639 | 01122020\_RID\_2209\_BuCaKA02.raw | 4.0261E7 | 2 | 2 | 224 | 252 |  |  | PEAKS DB |
| M.GLLDQRLALQWIQNNIHPFGGNPRAVTIFGESAGAASVGMHLLSTQSR.T | Y | 74.87 | 5127.6577 | 48 | 1.0 | 1026.5398 | 5 | 84.83 | 1 | 59856 | 01122020\_RID\_2209\_BuCaKA02.raw | 2.0822E7 | 1 | 1 | 200 | 247 |  |  | PEAKS DB |
| L.KVSTQTGSVRGLSLPVLDGHVTAFLGIPFAEPPVGR.M | Y | 71.73 | 3702.0256 | 36 | 5.4 | 926.5187 | 4 | 75.43 | 1 | 52253 | 01122020\_RID\_2209\_BuCaKA02.raw | 5.6822E8 | 4 | 4 | 38 | 73 |  |  | PEAKS DB |
| D.DIVGDHNVIC(+57.02)PVVQFANDYAK.R | N | 70.32 | 2373.1423 | 21 | 1.2 | 792.0557 | 3 | 67.35 | 1 | 45397 | 01122020\_RID\_2209\_BuCaKA02.raw | 3.7367E7 | 1 | 1 | 424 | 444 | Carbamidomethylation | C10:Carbamidomethylation:1000.00 | PEAKS DB |
| K.VSTQTGSVRGLSLPVLDGHVTAFLGIPFAEPPVGR.M | Y | 70.30 | 3573.9307 | 35 | 9.4 | 715.8001 | 5 | 83.23 | 1 | 58537 | 01122020\_RID\_2209\_BuCaKA02.raw | 1.4423E10 | 7 | 7 | 39 | 73 |  |  | PEAKS DB |
| V.SPGRAGELKVSTQTGSVR.G | Y | 70.11 | 1828.9755 | 18 | 0.0 | 610.6658 | 3 | 12.05 | 1 | 2023 | 01122020\_RID\_2209\_BuCaKA02.raw | 1.7435E6 | 1 | 1 | 30 | 47 |  |  | PEAKS DB |
| R.LALQWIQNNIHPFGGNPRAVTIFGESAGAASVGMHLLSTQSR.T | Y | 70.03 | 4445.2812 | 42 | 1.3 | 890.0647 | 5 | 76.25 | 1 | 52778 | 01122020\_RID\_2209\_BuCaKA02.raw | 6.7471E7 | 3 | 3 | 206 | 247 |  |  | PEAKS DB |
| R.SKNPQELIDEEWSVLPYK.S | Y | 69.96 | 2174.0896 | 18 | 0.8 | 725.7044 | 3 | 63.32 | 1 | 42005 | 01122020\_RID\_2209\_BuCaKA02.raw | 2.4894E7 | 2 | 2 | 299 | 316 |  |  | PEAKS DB |
| M.GLLDQRLALQWIQNNIHPFGGNPR.A | Y | 69.87 | 2756.4622 | 24 | 2.9 | 919.8307 | 3 | 77.23 | 1 | 53582 | 01122020\_RID\_2209\_BuCaKA02.raw | 2.4764E8 | 4 | 4 | 200 | 223 |  |  | PEAKS DB |
| R.AILQSGGPNAPWATVTPAESRR.R | N | 67.72 | 2278.1819 | 22 | 1.4 | 760.4023 | 3 | 39.81 | 1 | 23723 | 01122020\_RID\_2209\_BuCaKA02.raw | 7.6056E7 | 1 | 1 | 253 | 274 |  |  | PEAKS DB |
| R.RAALLGKQLGC(+57.02)HFNNDSELVSC(+57.02)LR.S | Y | 65.73 | 2757.3801 | 24 | 2.0 | 690.3537 | 4 | 33.56 | 1 | 18494 | 01122020\_RID\_2209\_BuCaKA02.raw | 1.5928E7 | 1 | 1 | 275 | 298 | Carbamidomethylation | C11:Carbamidomethylation:1000.00;C22:Carbamidomethylation:1000.00 | PEAKS DB |
| F.LGIPFAEPPVGR.M | N | 65.37 | 1251.6975 | 12 | 1.1 | 626.8567 | 2 | 56.11 | 1 | 36154 | 01122020\_RID\_2209\_BuCaKA02.raw | 4.6223E8 | 1 | 1 | 62 | 73 |  |  | PEAKS DB |
| L.SLPVLDGHVTAFLGIPFAEPPVGR.M | Y | 65.32 | 2488.3477 | 24 | 2.0 | 830.4581 | 3 | 86.79 | 1 | 61552 | 01122020\_RID\_2209\_BuCaKA02.raw | 8.1857E7 | 1 | 1 | 50 | 73 |  |  | PEAKS DB |
| V.STQTGSVRGLSLPVLDGHVTAFLGIPFAEPPVGR.M | Y | 65.11 | 3474.8623 | 34 | 6.8 | 869.7288 | 4 | 82.73 | 1 | 58133 | 01122020\_RID\_2209\_BuCaKA02.raw | 0 | 0 | 0 | 40 | 73 |  |  | PEAKS DB |
| R.TGNPTDPADKSGAWPTYTASQPQYVQLNTQPLATQPSLR.A | Y | 63.72 | 4199.0557 | 39 | 1.2 | 1400.6942 | 3 | 59.70 | 1 | 39174 | 01122020\_RID\_2209\_BuCaKA02.raw | 1.0279E9 | 3 | 3 | 510 | 548 |  |  | PEAKS DB |
| R.GLSLPVLDGHVTAFL.G | Y | 63.72 | 1537.8503 | 15 | 1.7 | 769.9337 | 2 | 87.38 | 1 | 62023 | 01122020\_RID\_2209\_BuCaKA02.raw | 1.6202E7 | 1 | 1 | 48 | 62 |  |  | PEAKS DB |
| K.VSTQTGSVRGLSLPVLDGHVTAF.L | Y | 63.61 | 2340.2437 | 23 | 0.9 | 1171.1301 | 2 | 64.35 | 1 | 42978 | 01122020\_RID\_2209\_BuCaKA02.raw | 5.93E8 | 2 | 2 | 39 | 61 |  |  | PEAKS DB |
| L.PVLDGHVTAFLGIPFAEPPVGR.M | Y | 63.60 | 2288.2317 | 22 | 2.5 | 763.7531 | 3 | 79.31 | 1 | 55332 | 01122020\_RID\_2209\_BuCaKA02.raw | 3.4806E6 | 1 | 1 | 52 | 73 |  |  | PEAKS DB |
| R.TLFQRAILQSGGPNAPWATVTPAESRR.R | Y | 62.58 | 2923.5415 | 27 | -0.7 | 731.8922 | 4 | 49.01 | 1 | 30339 | 01122020\_RID\_2209\_BuCaKA02.raw | 0 | 0 | 0 | 248 | 274 |  |  | PEAKS DB |
| S.LPVLDGHVTAFLGIPFAEPPVGR.M | Y | 62.25 | 2401.3157 | 23 | 2.2 | 801.4476 | 3 | 85.81 | 1 | 60666 | 01122020\_RID\_2209\_BuCaKA02.raw | 1.1292E7 | 1 | 1 | 51 | 73 |  |  | PEAKS DB |
| A.FLGIPFAEPPVGR.M | N | 62.09 | 1398.7659 | 13 | 0.0 | 700.3902 | 2 | 73.18 | 1 | 50153 | 01122020\_RID\_2209\_BuCaKA02.raw | 1.8064E7 | 1 | 1 | 61 | 73 |  |  | PEAKS DB |
| K.QLGC(+57.02)HFNNDSELVSC(+57.02)LR.S | Y | 62.05 | 2047.9204 | 17 | 0.7 | 683.6479 | 3 | 34.95 | 1 | 19633 | 01122020\_RID\_2209\_BuCaKA02.raw | 7.1157E7 | 1 | 1 | 282 | 298 | Carbamidomethylation | C4:Carbamidomethylation:1000.00;C15:Carbamidomethylation:1000.00 | PEAKS DB |
| L.ATQPSLRAQIC(+57.02)AFWNHFLPK.L | Y | 60.92 | 2384.2212 | 20 | 2.7 | 795.7498 | 3 | 71.29 | 1 | 48640 | 01122020\_RID\_2209\_BuCaKA02.raw | 2.1393E7 | 2 | 2 | 542 | 561 | Carbamidomethylation | C11:Carbamidomethylation:1000.00 | PEAKS DB |
| R.AVTIFGESAGAASVGMHLLSTQSRTLFQRAILQSGGPNAPWATVTPAESR.R | Y | 60.82 | 5138.6357 | 50 | 0.4 | 1285.6667 | 4 | 83.73 | 1 | 58887 | 01122020\_RID\_2209\_BuCaKA02.raw | 9.1205E7 | 2 | 2 | 224 | 273 |  |  | PEAKS DB |
| R.AQIC(+57.02)AFWNHFLPKL.L | N | 59.24 | 1743.8918 | 14 | 1.7 | 872.9547 | 2 | 72.95 | 1 | 49964 | 01122020\_RID\_2209\_BuCaKA02.raw | 1.5107E8 | 2 | 2 | 549 | 562 | Carbamidomethylation | C4:Carbamidomethylation:1000.00 | PEAKS DB |
| K.SIFRFPFVPVIDG.D | N | 58.74 | 1492.8077 | 13 | 2.3 | 747.4128 | 2 | 91.00 | 1 | 65018 | 01122020\_RID\_2209\_BuCaKA02.raw | 9.7066E6 | 1 | 1 | 317 | 329 |  |  | PEAKS DB |
| V.RGLSLPVLDGHVTAFLGIPFAEPPVGR.M | Y | 58.04 | 2814.5544 | 27 | 2.6 | 704.6477 | 4 | 82.79 | 1 | 58181 | 01122020\_RID\_2209\_BuCaKA02.raw | 0 | 0 | 0 | 47 | 73 |  |  | PEAKS DB |
| R.AGELKVSTQTGSVRGLSLPVLDGHVTAF.L | Y | 57.78 | 2838.5239 | 28 | 0.1 | 710.6383 | 4 | 60.89 | 1 | 40258 | 01122020\_RID\_2209\_BuCaKA02.raw | 9.4408E8 | 5 | 5 | 34 | 61 |  |  | PEAKS DB |
| D.PADKSGAWPTYTASQPQYVQLNTQPLATQPSLR.A | Y | 56.43 | 3613.8164 | 33 | -0.8 | 1205.6118 | 3 | 56.49 | 1 | 36480 | 01122020\_RID\_2209\_BuCaKA02.raw | 4.7617E7 | 1 | 1 | 516 | 548 |  |  | PEAKS DB |
| Q.IC(+57.02)AFWNHFLPK.L | N | 56.19 | 1431.7122 | 11 | 1.5 | 716.8644 | 2 | 55.11 | 1 | 35292 | 01122020\_RID\_2209\_BuCaKA02.raw | 7.8377E6 | 1 | 1 | 551 | 561 | Carbamidomethylation | C2:Carbamidomethylation:1000.00 | PEAKS DB |
| A.TQPSLRAQIC(+57.02)AFWNHFLPK.L | Y | 56.17 | 2313.1841 | 19 | 0.9 | 579.3038 | 4 | 71.36 | 1 | 48657 | 01122020\_RID\_2209\_BuCaKA02.raw | 9.1707E6 | 2 | 2 | 543 | 561 | Carbamidomethylation | C10:Carbamidomethylation:1000.00 | PEAKS DB |
| R.AQIC(+57.02)AFWNHFLPKLLNATVDPPSADR.R | Y | 56.04 | 2980.5017 | 26 | 1.6 | 746.1339 | 4 | 77.71 | 1 | 54026 | 01122020\_RID\_2209\_BuCaKA02.raw | 0 | 0 | 0 | 549 | 574 | Carbamidomethylation | C4:Carbamidomethylation:1000.00 | PEAKS DB |
| R.GLSLPVLDGHVTAF.L | Y | 55.10 | 1424.7664 | 14 | 1.4 | 713.3915 | 2 | 76.68 | 1 | 53109 | 01122020\_RID\_2209\_BuCaKA02.raw | 6.1163E9 | 1 | 1 | 48 | 61 |  |  | PEAKS DB |
| D.GHVTAFLGIPFAEPPVGR.M | Y | 53.85 | 1863.9995 | 18 | 1.0 | 622.3411 | 3 | 65.09 | 1 | 43442 | 01122020\_RID\_2209\_BuCaKA02.raw | 1.6562E7 | 1 | 1 | 56 | 73 |  |  | PEAKS DB |
| R.AQIC(+57.02)AFWNHFLPKLLN.A | Y | 52.39 | 1971.0189 | 16 | 1.1 | 658.0143 | 3 | 78.10 | 1 | 54392 | 01122020\_RID\_2209\_BuCaKA02.raw | 1.1181E8 | 1 | 1 | 549 | 564 | Carbamidomethylation | C4:Carbamidomethylation:1000.00 | PEAKS DB |
| R.AQIC(+57.02)AFWNHFLPKLLNA.T | Y | 52.28 | 2042.0560 | 17 | 0.9 | 1022.0362 | 2 | 80.05 | 1 | 56080 | 01122020\_RID\_2209\_BuCaKA02.raw | 4.8965E8 | 4 | 4 | 549 | 565 | Carbamidomethylation | C4:Carbamidomethylation:1000.00 | PEAKS DB |
| A.QIC(+57.02)AFWNHFLPK.L | N | 49.55 | 1559.7708 | 12 | 1.5 | 780.8938 | 2 | 57.48 | 1 | 37350 | 01122020\_RID\_2209\_BuCaKA02.raw | 2.0913E7 | 1 | 1 | 550 | 561 | Carbamidomethylation | C3:Carbamidomethylation:1000.00 | PEAKS DB |
| R.GLSLPVLDGHVTA.F | Y | 48.82 | 1277.6979 | 13 | 1.7 | 639.8573 | 2 | 62.03 | 1 | 40890 | 01122020\_RID\_2209\_BuCaKA02.raw | 8.2252E7 | 1 | 1 | 48 | 60 |  |  | PEAKS DB |
| R.GLSLPVLDGHVTAFLGIPFAEPPVGRMR.F | Y | 48.68 | 2945.5950 | 28 | 4.5 | 737.4093 | 4 | 85.11 | 1 | 60069 | 01122020\_RID\_2209\_BuCaKA02.raw | 1.6187E7 | 2 | 2 | 48 | 75 |  |  | PEAKS DB |
| L.ALQWIQNNIHPFGGNPR.A | Y | 46.40 | 1961.0020 | 17 | 1.8 | 654.6758 | 3 | 46.23 | 1 | 28435 | 01122020\_RID\_2209\_BuCaKA02.raw | 0 | 0 | 0 | 207 | 223 |  |  | PEAKS DB |
| R.AVTIFGESAGAASVGM(+15.99)HLLSTQSR.T | N | 45.59 | 2405.2009 | 24 | 3.4 | 802.7437 | 3 | 54.96 | 1 | 35227 | 01122020\_RID\_2209\_BuCaKA02.raw | 8.7978E6 | 1 | 1 | 224 | 247 | Oxidation (M) | M16:Oxidation (M):1000.00 | PEAKS DB |
| G.SVRGLSLPVLDGHVTAFLGIPFAEPPVGR.M | Y | 45.26 | 3000.6548 | 29 | 6.1 | 751.1755 | 4 | 83.64 | 1 | 58882 | 01122020\_RID\_2209\_BuCaKA02.raw | 0 | 0 | 0 | 45 | 73 |  |  | PEAKS DB |
| R.AQIC(+57.02)AFWNHFLP.K | N | 44.50 | 1502.7129 | 12 | 0.9 | 752.3644 | 2 | 78.17 | 1 | 54427 | 01122020\_RID\_2209\_BuCaKA02.raw | 5.0431E8 | 1 | 1 | 549 | 560 | Carbamidomethylation | C4:Carbamidomethylation:1000.00 | PEAKS DB |
| K.SGAWPTYTASQPQYVQLNTQPLATQPSLR.A | Y | 43.03 | 3202.6047 | 29 | 0.2 | 1068.5424 | 3 | 65.06 | 1 | 43406 | 01122020\_RID\_2209\_BuCaKA02.raw | 2.1659E8 | 1 | 1 | 520 | 548 |  |  | PEAKS DB |
| R.GLSLPVLDGHVTAFLG.I | Y | 42.79 | 1594.8718 | 16 | 2.7 | 798.4453 | 2 | 85.31 | 1 | 60432 | 01122020\_RID\_2209\_BuCaKA02.raw | 5.2128E8 | 2 | 2 | 48 | 63 |  |  | PEAKS DB |
| L.NTQPLATQPSLRAQIC(+57.02)AFWNHFLPK.L | Y | 42.22 | 2937.5071 | 25 | 4.0 | 735.3870 | 4 | 74.50 | 1 | 51499 | 01122020\_RID\_2209\_BuCaKA02.raw | 2.6172E8 | 1 | 1 | 537 | 561 | Carbamidomethylation | C16:Carbamidomethylation:1000.00 | PEAKS DB |
| R.TGNPTDPADKSGAWPTYTASQPQYVQLNTQPLATQPSLRAQIC(+57.02)AFWNHFLPK.L | Y | 42.13 | 5811.8530 | 52 | 3.1 | 1453.9750 | 4 | 82.20 | 1 | 57744 | 01122020\_RID\_2209\_BuCaKA02.raw | 1.5131E9 | 1 | 1 | 510 | 561 | Carbamidomethylation | C43:Carbamidomethylation:1000.00 | PEAKS DB |
| K.VSTQTGSVRGLSLPVLDGHVTAFLG.I | Y | 41.47 | 2510.3491 | 25 | 1.1 | 837.7913 | 3 | 72.55 | 1 | 49756 | 01122020\_RID\_2209\_BuCaKA02.raw | 2.5098E7 | 1 | 1 | 39 | 63 |  |  | PEAKS DB |
| total 61 peptides |  |  |  |  |  |  |  |  |  |  |  |  |  |  |  |  |  |  |  |
| --- | --- | --- | --- | --- | --- | --- | --- | --- | --- | --- | --- | --- | --- | --- | --- | --- | --- | --- | --- |

T\_60

back to list

  

| Protein Coverage
| Supporting Peptides
|

Protein Coverage:

Supporting Peptides:

| Peptide | Uniq | -10lgP | Mass | Length | ppm | m/z | z | RT | Fraction | Scan | Source File | Area BRom | #Feature | #Feature BRom | Start | End | PTM | AScore | Found By |
| --- | --- | --- | --- | --- | --- | --- | --- | --- | --- | --- | --- | --- | --- | --- | --- | --- | --- | --- | --- |
| R.GLSLPVLDGHVTAFLGIPFAEPPVGR.M | Y | 101.40 | 2658.4534 | 26 | 0.7 | 1330.2349 | 2 | 93.83 | 1 | 67305 | 01122020\_RID\_2209\_BuCaKA02.raw | 5.7352E10 | 14 | 14 | 48 | 73 |  |  | PEAKS DB |
| R.AALLGKQLGC(+57.02)HFNNDSELVSC(+57.02)LR.S | Y | 96.22 | 2601.2791 | 23 | 0.3 | 1301.6472 | 2 | 41.43 | 1 | 24779 | 01122020\_RID\_2209\_BuCaKA02.raw | 1.888E8 | 2 | 2 | 276 | 298 | Carbamidomethylation | C10:Carbamidomethylation:1000.00;C21:Carbamidomethylation:1000.00 | PEAKS DB |
| R.AILQSGGPNAPWATVTPAESR.R | N | 95.10 | 2122.0806 | 21 | -0.9 | 1062.0466 | 2 | 52.50 | 1 | 33145 | 01122020\_RID\_2209\_BuCaKA02.raw | 1.3371E9 | 2 | 2 | 253 | 273 |  |  | PEAKS DB |
| R.EALDDIVGDHNVIC(+57.02)PVVQFANDYAKR.N | N | 93.08 | 2957.4341 | 26 | 0.1 | 740.3659 | 4 | 70.15 | 1 | 47677 | 01122020\_RID\_2209\_BuCaKA02.raw | 1.6502E7 | 3 | 3 | 420 | 445 | Carbamidomethylation | C14:Carbamidomethylation:1000.00 | PEAKS DB |
| R.EALDDIVGDHNVIC(+57.02)PVVQFANDYAK.R | N | 88.62 | 2801.3330 | 25 | 1.0 | 1401.6752 | 2 | 77.11 | 1 | 53666 | 01122020\_RID\_2209\_BuCaKA02.raw | 7.8726E8 | 2 | 2 | 420 | 444 | Carbamidomethylation | C14:Carbamidomethylation:1000.00 | PEAKS DB |
| R.LALQWIQNNIHPFGGNPR.A | Y | 87.29 | 2074.0859 | 18 | 4.4 | 692.3723 | 3 | 60.27 | 1 | 39720 | 01122020\_RID\_2209\_BuCaKA02.raw | 2.2787E8 | 1 | 1 | 206 | 223 |  |  | PEAKS DB |
| R.AVTIFGESAGAASVGMHLLSTQSR.T | N | 85.30 | 2389.2061 | 24 | 0.3 | 797.4095 | 3 | 65.27 | 1 | 44229 | 01122020\_RID\_2209\_BuCaKA02.raw | 4.1007E8 | 3 | 3 | 224 | 247 |  |  | PEAKS DB |
| K.NREALDDIVGDHNVIC(+57.02)PVVQFANDYAK.R | N | 82.20 | 3071.4771 | 27 | 0.7 | 768.8771 | 4 | 65.86 | 1 | 44085 | 01122020\_RID\_2209\_BuCaKA02.raw | 2.8252E8 | 1 | 1 | 418 | 444 | Carbamidomethylation | C16:Carbamidomethylation:1000.00 | PEAKS DB |
| R.AQIC(+57.02)AFWNHFLPK.L | N | 81.68 | 1630.8079 | 13 | 0.4 | 816.4115 | 2 | 60.34 | 1 | 39698 | 01122020\_RID\_2209\_BuCaKA02.raw | 3.6259E8 | 1 | 1 | 549 | 561 | Carbamidomethylation | C4:Carbamidomethylation:1000.00 | PEAKS DB |
| R.AGELKVSTQTGSVRGLSLPVLDGHVTAFLGIPFAEPPVGR.M | Y | 77.17 | 4072.2109 | 40 | 4.9 | 815.4535 | 5 | 78.62 | 1 | 54698 | 01122020\_RID\_2209\_BuCaKA02.raw | 1.683E9 | 3 | 3 | 34 | 73 |  |  | PEAKS DB |
| G.LSLPVLDGHVTAFLGIPFAEPPVGR.M | Y | 75.82 | 2601.4319 | 25 | 1.7 | 868.1527 | 3 | 93.27 | 1 | 66854 | 01122020\_RID\_2209\_BuCaKA02.raw | 9.8181E7 | 1 | 1 | 49 | 73 |  |  | PEAKS DB |
| R.AGELKVSTQTGSVR.G | Y | 75.64 | 1431.7681 | 14 | 0.2 | 716.8914 | 2 | 12.05 | 1 | 2024 | 01122020\_RID\_2209\_BuCaKA02.raw | 2.521E6 | 1 | 1 | 34 | 47 |  |  | PEAKS DB |
| R.TLFQRAILQSGGPNAPWATVTPAESR.R | Y | 75.41 | 2767.4404 | 26 | 0.6 | 923.4880 | 3 | 59.02 | 1 | 38537 | 01122020\_RID\_2209\_BuCaKA02.raw | 9.0758E7 | 1 | 1 | 248 | 273 |  |  | PEAKS DB |
| R.AVTIFGESAGAASVGMHLLSTQSRTLFQR.A | Y | 75.28 | 3034.5657 | 29 | 1.4 | 759.6498 | 4 | 72.55 | 1 | 49639 | 01122020\_RID\_2209\_BuCaKA02.raw | 4.0261E7 | 2 | 2 | 224 | 252 |  |  | PEAKS DB |
| M.GLLDQRLALQWIQNNIHPFGGNPRAVTIFGESAGAASVGMHLLSTQSR.T | Y | 74.87 | 5127.6577 | 48 | 1.0 | 1026.5398 | 5 | 84.83 | 1 | 59856 | 01122020\_RID\_2209\_BuCaKA02.raw | 2.0822E7 | 1 | 1 | 200 | 247 |  |  | PEAKS DB |
| L.KVSTQTGSVRGLSLPVLDGHVTAFLGIPFAEPPVGR.M | Y | 71.73 | 3702.0256 | 36 | 5.4 | 926.5187 | 4 | 75.43 | 1 | 52253 | 01122020\_RID\_2209\_BuCaKA02.raw | 5.6822E8 | 4 | 4 | 38 | 73 |  |  | PEAKS DB |
| D.DIVGDHNVIC(+57.02)PVVQFANDYAK.R | N | 70.32 | 2373.1423 | 21 | 1.2 | 792.0557 | 3 | 67.35 | 1 | 45397 | 01122020\_RID\_2209\_BuCaKA02.raw | 3.7367E7 | 1 | 1 | 424 | 444 | Carbamidomethylation | C10:Carbamidomethylation:1000.00 | PEAKS DB |
| K.VSTQTGSVRGLSLPVLDGHVTAFLGIPFAEPPVGR.M | Y | 70.30 | 3573.9307 | 35 | 9.4 | 715.8001 | 5 | 83.23 | 1 | 58537 | 01122020\_RID\_2209\_BuCaKA02.raw | 1.4423E10 | 7 | 7 | 39 | 73 |  |  | PEAKS DB |
| V.SPGRAGELKVSTQTGSVR.G | Y | 70.11 | 1828.9755 | 18 | 0.0 | 610.6658 | 3 | 12.05 | 1 | 2023 | 01122020\_RID\_2209\_BuCaKA02.raw | 1.7435E6 | 1 | 1 | 30 | 47 |  |  | PEAKS DB |
| R.LALQWIQNNIHPFGGNPRAVTIFGESAGAASVGMHLLSTQSR.T | Y | 70.03 | 4445.2812 | 42 | 1.3 | 890.0647 | 5 | 76.25 | 1 | 52778 | 01122020\_RID\_2209\_BuCaKA02.raw | 6.7471E7 | 3 | 3 | 206 | 247 |  |  | PEAKS DB |
| R.SKNPQELIDEEWSVLPYK.S | Y | 69.96 | 2174.0896 | 18 | 0.8 | 725.7044 | 3 | 63.32 | 1 | 42005 | 01122020\_RID\_2209\_BuCaKA02.raw | 2.4894E7 | 2 | 2 | 299 | 316 |  |  | PEAKS DB |
| M.GLLDQRLALQWIQNNIHPFGGNPR.A | Y | 69.87 | 2756.4622 | 24 | 2.9 | 919.8307 | 3 | 77.23 | 1 | 53582 | 01122020\_RID\_2209\_BuCaKA02.raw | 2.4764E8 | 4 | 4 | 200 | 223 |  |  | PEAKS DB |
| R.AILQSGGPNAPWATVTPAESRR.R | N | 67.72 | 2278.1819 | 22 | 1.4 | 760.4023 | 3 | 39.81 | 1 | 23723 | 01122020\_RID\_2209\_BuCaKA02.raw | 7.6056E7 | 1 | 1 | 253 | 274 |  |  | PEAKS DB |
| R.RAALLGKQLGC(+57.02)HFNNDSELVSC(+57.02)LR.S | Y | 65.73 | 2757.3801 | 24 | 2.0 | 690.3537 | 4 | 33.56 | 1 | 18494 | 01122020\_RID\_2209\_BuCaKA02.raw | 1.5928E7 | 1 | 1 | 275 | 298 | Carbamidomethylation | C11:Carbamidomethylation:1000.00;C22:Carbamidomethylation:1000.00 | PEAKS DB |
| F.LGIPFAEPPVGR.M | N | 65.37 | 1251.6975 | 12 | 1.1 | 626.8567 | 2 | 56.11 | 1 | 36154 | 01122020\_RID\_2209\_BuCaKA02.raw | 4.6223E8 | 1 | 1 | 62 | 73 |  |  | PEAKS DB |
| L.SLPVLDGHVTAFLGIPFAEPPVGR.M | Y | 65.32 | 2488.3477 | 24 | 2.0 | 830.4581 | 3 | 86.79 | 1 | 61552 | 01122020\_RID\_2209\_BuCaKA02.raw | 8.1857E7 | 1 | 1 | 50 | 73 |  |  | PEAKS DB |
| V.STQTGSVRGLSLPVLDGHVTAFLGIPFAEPPVGR.M | Y | 65.11 | 3474.8623 | 34 | 6.8 | 869.7288 | 4 | 82.73 | 1 | 58133 | 01122020\_RID\_2209\_BuCaKA02.raw | 0 | 0 | 0 | 40 | 73 |  |  | PEAKS DB |
| R.TGNPTDPADKSGAWPTYTASQPQYVQLNTQPLATQPSLR.A | Y | 63.72 | 4199.0557 | 39 | 1.2 | 1400.6942 | 3 | 59.70 | 1 | 39174 | 01122020\_RID\_2209\_BuCaKA02.raw | 1.0279E9 | 3 | 3 | 510 | 548 |  |  | PEAKS DB |
| R.GLSLPVLDGHVTAFL.G | Y | 63.72 | 1537.8503 | 15 | 1.7 | 769.9337 | 2 | 87.38 | 1 | 62023 | 01122020\_RID\_2209\_BuCaKA02.raw | 1.6202E7 | 1 | 1 | 48 | 62 |  |  | PEAKS DB |
| K.VSTQTGSVRGLSLPVLDGHVTAF.L | Y | 63.61 | 2340.2437 | 23 | 0.9 | 1171.1301 | 2 | 64.35 | 1 | 42978 | 01122020\_RID\_2209\_BuCaKA02.raw | 5.93E8 | 2 | 2 | 39 | 61 |  |  | PEAKS DB |
| L.PVLDGHVTAFLGIPFAEPPVGR.M | Y | 63.60 | 2288.2317 | 22 | 2.5 | 763.7531 | 3 | 79.31 | 1 | 55332 | 01122020\_RID\_2209\_BuCaKA02.raw | 3.4806E6 | 1 | 1 | 52 | 73 |  |  | PEAKS DB |
| R.TLFQRAILQSGGPNAPWATVTPAESRR.R | Y | 62.58 | 2923.5415 | 27 | -0.7 | 731.8922 | 4 | 49.01 | 1 | 30339 | 01122020\_RID\_2209\_BuCaKA02.raw | 0 | 0 | 0 | 248 | 274 |  |  | PEAKS DB |
| S.LPVLDGHVTAFLGIPFAEPPVGR.M | Y | 62.25 | 2401.3157 | 23 | 2.2 | 801.4476 | 3 | 85.81 | 1 | 60666 | 01122020\_RID\_2209\_BuCaKA02.raw | 1.1292E7 | 1 | 1 | 51 | 73 |  |  | PEAKS DB |
| A.FLGIPFAEPPVGR.M | N | 62.09 | 1398.7659 | 13 | 0.0 | 700.3902 | 2 | 73.18 | 1 | 50153 | 01122020\_RID\_2209\_BuCaKA02.raw | 1.8064E7 | 1 | 1 | 61 | 73 |  |  | PEAKS DB |
| K.QLGC(+57.02)HFNNDSELVSC(+57.02)LR.S | Y | 62.05 | 2047.9204 | 17 | 0.7 | 683.6479 | 3 | 34.95 | 1 | 19633 | 01122020\_RID\_2209\_BuCaKA02.raw | 7.1157E7 | 1 | 1 | 282 | 298 | Carbamidomethylation | C4:Carbamidomethylation:1000.00;C15:Carbamidomethylation:1000.00 | PEAKS DB |
| L.ATQPSLRAQIC(+57.02)AFWNHFLPK.L | Y | 60.92 | 2384.2212 | 20 | 2.7 | 795.7498 | 3 | 71.29 | 1 | 48640 | 01122020\_RID\_2209\_BuCaKA02.raw | 2.1393E7 | 2 | 2 | 542 | 561 | Carbamidomethylation | C11:Carbamidomethylation:1000.00 | PEAKS DB |
| R.AVTIFGESAGAASVGMHLLSTQSRTLFQRAILQSGGPNAPWATVTPAESR.R | Y | 60.82 | 5138.6357 | 50 | 0.4 | 1285.6667 | 4 | 83.73 | 1 | 58887 | 01122020\_RID\_2209\_BuCaKA02.raw | 9.1205E7 | 2 | 2 | 224 | 273 |  |  | PEAKS DB |
| R.AQIC(+57.02)AFWNHFLPKL.L | N | 59.24 | 1743.8918 | 14 | 1.7 | 872.9547 | 2 | 72.95 | 1 | 49964 | 01122020\_RID\_2209\_BuCaKA02.raw | 1.5107E8 | 2 | 2 | 549 | 562 | Carbamidomethylation | C4:Carbamidomethylation:1000.00 | PEAKS DB |
| K.SIFRFPFVPVIDG.D | N | 58.74 | 1492.8077 | 13 | 2.3 | 747.4128 | 2 | 91.00 | 1 | 65018 | 01122020\_RID\_2209\_BuCaKA02.raw | 9.7066E6 | 1 | 1 | 317 | 329 |  |  | PEAKS DB |
| V.RGLSLPVLDGHVTAFLGIPFAEPPVGR.M | Y | 58.04 | 2814.5544 | 27 | 2.6 | 704.6477 | 4 | 82.79 | 1 | 58181 | 01122020\_RID\_2209\_BuCaKA02.raw | 0 | 0 | 0 | 47 | 73 |  |  | PEAKS DB |
| R.AGELKVSTQTGSVRGLSLPVLDGHVTAF.L | Y | 57.78 | 2838.5239 | 28 | 0.1 | 710.6383 | 4 | 60.89 | 1 | 40258 | 01122020\_RID\_2209\_BuCaKA02.raw | 9.4408E8 | 5 | 5 | 34 | 61 |  |  | PEAKS DB |
| D.PADKSGAWPTYTASQPQYVQLNTQPLATQPSLR.A | Y | 56.43 | 3613.8164 | 33 | -0.8 | 1205.6118 | 3 | 56.49 | 1 | 36480 | 01122020\_RID\_2209\_BuCaKA02.raw | 4.7617E7 | 1 | 1 | 516 | 548 |  |  | PEAKS DB |
| Q.IC(+57.02)AFWNHFLPK.L | N | 56.19 | 1431.7122 | 11 | 1.5 | 716.8644 | 2 | 55.11 | 1 | 35292 | 01122020\_RID\_2209\_BuCaKA02.raw | 7.8377E6 | 1 | 1 | 551 | 561 | Carbamidomethylation | C2:Carbamidomethylation:1000.00 | PEAKS DB |
| A.TQPSLRAQIC(+57.02)AFWNHFLPK.L | Y | 56.17 | 2313.1841 | 19 | 0.9 | 579.3038 | 4 | 71.36 | 1 | 48657 | 01122020\_RID\_2209\_BuCaKA02.raw | 9.1707E6 | 2 | 2 | 543 | 561 | Carbamidomethylation | C10:Carbamidomethylation:1000.00 | PEAKS DB |
| R.AQIC(+57.02)AFWNHFLPKLLNATVDPPSADR.R | Y | 56.04 | 2980.5017 | 26 | 1.6 | 746.1339 | 4 | 77.71 | 1 | 54026 | 01122020\_RID\_2209\_BuCaKA02.raw | 0 | 0 | 0 | 549 | 574 | Carbamidomethylation | C4:Carbamidomethylation:1000.00 | PEAKS DB |
| R.GLSLPVLDGHVTAF.L | Y | 55.10 | 1424.7664 | 14 | 1.4 | 713.3915 | 2 | 76.68 | 1 | 53109 | 01122020\_RID\_2209\_BuCaKA02.raw | 6.1163E9 | 1 | 1 | 48 | 61 |  |  | PEAKS DB |
| D.GHVTAFLGIPFAEPPVGR.M | Y | 53.85 | 1863.9995 | 18 | 1.0 | 622.3411 | 3 | 65.09 | 1 | 43442 | 01122020\_RID\_2209\_BuCaKA02.raw | 1.6562E7 | 1 | 1 | 56 | 73 |  |  | PEAKS DB |
| R.AQIC(+57.02)AFWNHFLPKLLN.A | Y | 52.39 | 1971.0189 | 16 | 1.1 | 658.0143 | 3 | 78.10 | 1 | 54392 | 01122020\_RID\_2209\_BuCaKA02.raw | 1.1181E8 | 1 | 1 | 549 | 564 | Carbamidomethylation | C4:Carbamidomethylation:1000.00 | PEAKS DB |
| R.AQIC(+57.02)AFWNHFLPKLLNA.T | Y | 52.28 | 2042.0560 | 17 | 0.9 | 1022.0362 | 2 | 80.05 | 1 | 56080 | 01122020\_RID\_2209\_BuCaKA02.raw | 4.8965E8 | 4 | 4 | 549 | 565 | Carbamidomethylation | C4:Carbamidomethylation:1000.00 | PEAKS DB |
| A.QIC(+57.02)AFWNHFLPK.L | N | 49.55 | 1559.7708 | 12 | 1.5 | 780.8938 | 2 | 57.48 | 1 | 37350 | 01122020\_RID\_2209\_BuCaKA02.raw | 2.0913E7 | 1 | 1 | 550 | 561 | Carbamidomethylation | C3:Carbamidomethylation:1000.00 | PEAKS DB |
| R.GLSLPVLDGHVTA.F | Y | 48.82 | 1277.6979 | 13 | 1.7 | 639.8573 | 2 | 62.03 | 1 | 40890 | 01122020\_RID\_2209\_BuCaKA02.raw | 8.2252E7 | 1 | 1 | 48 | 60 |  |  | PEAKS DB |
| R.GLSLPVLDGHVTAFLGIPFAEPPVGRMR.F | Y | 48.68 | 2945.5950 | 28 | 4.5 | 737.4093 | 4 | 85.11 | 1 | 60069 | 01122020\_RID\_2209\_BuCaKA02.raw | 1.6187E7 | 2 | 2 | 48 | 75 |  |  | PEAKS DB |
| L.ALQWIQNNIHPFGGNPR.A | Y | 46.40 | 1961.0020 | 17 | 1.8 | 654.6758 | 3 | 46.23 | 1 | 28435 | 01122020\_RID\_2209\_BuCaKA02.raw | 0 | 0 | 0 | 207 | 223 |  |  | PEAKS DB |
| R.AVTIFGESAGAASVGM(+15.99)HLLSTQSR.T | N | 45.59 | 2405.2009 | 24 | 3.4 | 802.7437 | 3 | 54.96 | 1 | 35227 | 01122020\_RID\_2209\_BuCaKA02.raw | 8.7978E6 | 1 | 1 | 224 | 247 | Oxidation (M) | M16:Oxidation (M):1000.00 | PEAKS DB |
| G.SVRGLSLPVLDGHVTAFLGIPFAEPPVGR.M | Y | 45.26 | 3000.6548 | 29 | 6.1 | 751.1755 | 4 | 83.64 | 1 | 58882 | 01122020\_RID\_2209\_BuCaKA02.raw | 0 | 0 | 0 | 45 | 73 |  |  | PEAKS DB |
| R.AQIC(+57.02)AFWNHFLP.K | N | 44.50 | 1502.7129 | 12 | 0.9 | 752.3644 | 2 | 78.17 | 1 | 54427 | 01122020\_RID\_2209\_BuCaKA02.raw | 5.0431E8 | 1 | 1 | 549 | 560 | Carbamidomethylation | C4:Carbamidomethylation:1000.00 | PEAKS DB |
| K.SGAWPTYTASQPQYVQLNTQPLATQPSLR.A | Y | 43.03 | 3202.6047 | 29 | 0.2 | 1068.5424 | 3 | 65.06 | 1 | 43406 | 01122020\_RID\_2209\_BuCaKA02.raw | 2.1659E8 | 1 | 1 | 520 | 548 |  |  | PEAKS DB |
| R.GLSLPVLDGHVTAFLG.I | Y | 42.79 | 1594.8718 | 16 | 2.7 | 798.4453 | 2 | 85.31 | 1 | 60432 | 01122020\_RID\_2209\_BuCaKA02.raw | 5.2128E8 | 2 | 2 | 48 | 63 |  |  | PEAKS DB |
| L.NTQPLATQPSLRAQIC(+57.02)AFWNHFLPK.L | Y | 42.22 | 2937.5071 | 25 | 4.0 | 735.3870 | 4 | 74.50 | 1 | 51499 | 01122020\_RID\_2209\_BuCaKA02.raw | 2.6172E8 | 1 | 1 | 537 | 561 | Carbamidomethylation | C16:Carbamidomethylation:1000.00 | PEAKS DB |
| R.TGNPTDPADKSGAWPTYTASQPQYVQLNTQPLATQPSLRAQIC(+57.02)AFWNHFLPK.L | Y | 42.13 | 5811.8530 | 52 | 3.1 | 1453.9750 | 4 | 82.20 | 1 | 57744 | 01122020\_RID\_2209\_BuCaKA02.raw | 1.5131E9 | 1 | 1 | 510 | 561 | Carbamidomethylation | C43:Carbamidomethylation:1000.00 | PEAKS DB |
| K.VSTQTGSVRGLSLPVLDGHVTAFLG.I | Y | 41.47 | 2510.3491 | 25 | 1.1 | 837.7913 | 3 | 72.55 | 1 | 49756 | 01122020\_RID\_2209\_BuCaKA02.raw | 2.5098E7 | 1 | 1 | 39 | 63 |  |  | PEAKS DB |
| total 61 peptides |  |  |  |  |  |  |  |  |  |  |  |  |  |  |  |  |  |  |  |
| --- | --- | --- | --- | --- | --- | --- | --- | --- | --- | --- | --- | --- | --- | --- | --- | --- | --- | --- | --- |

T\_46

back to list

  

| Protein Coverage
| Supporting Peptides
|

Protein Coverage:

Supporting Peptides:

| Peptide | Uniq | -10lgP | Mass | Length | ppm | m/z | z | RT | Fraction | Scan | Source File | Area BRom | #Feature | #Feature BRom | Start | End | PTM | AScore | Found By |
| --- | --- | --- | --- | --- | --- | --- | --- | --- | --- | --- | --- | --- | --- | --- | --- | --- | --- | --- | --- |
| R.IDFKGNTVGLAALGSLC(+57.02)SVKYSVAVIQDYSK.R | Y | 96.62 | 3302.7219 | 31 | 4.3 | 1101.9193 | 3 | 76.99 | 1 | 53318 | 01122020\_RID\_2209\_BuCaKA02.raw | 6.5213E8 | 5 | 5 | 276 | 306 | Carbamidomethylation | C17:Carbamidomethylation:1000.00 | PEAKS DB |
| R.IDFKGNTVGLAALGSLC(+57.02)SVK.Y | Y | 96.48 | 2049.0928 | 20 | 1.0 | 1025.5547 | 2 | 61.93 | 1 | 40849 | 01122020\_RID\_2209\_BuCaKA02.raw | 8.2934E9 | 3 | 3 | 276 | 295 | Carbamidomethylation | C17:Carbamidomethylation:1000.00 | PEAKS DB |
| R.NDNAQLLTRIDFKGNTVGLAALGSLC(+57.02)SVK.Y | Y | 83.77 | 3074.6182 | 29 | 0.6 | 1025.8806 | 3 | 67.59 | 1 | 45617 | 01122020\_RID\_2209\_BuCaKA02.raw | 8.6656E7 | 5 | 5 | 267 | 295 | Carbamidomethylation | C26:Carbamidomethylation:1000.00 | PEAKS DB |
| K.GNTVGLAALGSLC(+57.02)SVK.Y | N | 82.54 | 1545.8185 | 16 | 1.2 | 773.9175 | 2 | 54.52 | 1 | 34666 | 01122020\_RID\_2209\_BuCaKA02.raw | 1.9308E8 | 1 | 1 | 280 | 295 | Carbamidomethylation | C13:Carbamidomethylation:1000.00 | PEAKS DB |
| K.YSVAVIQDYSKR.T | N | 82.32 | 1427.7408 | 12 | 1.0 | 714.8784 | 2 | 25.60 | 1 | 12556 | 01122020\_RID\_2209\_BuCaKA02.raw | 9.1389E7 | 1 | 1 | 296 | 307 |  |  | PEAKS DB |
| K.YSVAVIQDYSK.R | N | 80.30 | 1271.6398 | 11 | 1.5 | 636.8281 | 2 | 35.71 | 1 | 20291 | 01122020\_RID\_2209\_BuCaKA02.raw | 3.0445E7 | 1 | 1 | 296 | 306 |  |  | PEAKS DB |
| R.LNFHIALIGLEIWSKR.D | N | 80.23 | 1909.0938 | 16 | 2.1 | 637.3732 | 3 | 72.46 | 1 | 49569 | 01122020\_RID\_2209\_BuCaKA02.raw | 2.5467E7 | 2 | 2 | 222 | 237 |  |  | PEAKS DB |
| K.GNTVGLAALGSLC(+57.02)SVKYSVAVIQDYSKR.T | N | 80.09 | 2955.5488 | 28 | 0.9 | 739.8951 | 4 | 67.74 | 1 | 45639 | 01122020\_RID\_2209\_BuCaKA02.raw | 5.8811E8 | 2 | 2 | 280 | 307 | Carbamidomethylation | C13:Carbamidomethylation:1000.00 | PEAKS DB |
| L.TRIDFKGNTVGLAALGSLC(+57.02)SVK.Y | Y | 77.54 | 2306.2417 | 22 | 1.3 | 769.7555 | 3 | 50.90 | 1 | 31805 | 01122020\_RID\_2209\_BuCaKA02.raw | 8.9942E7 | 2 | 2 | 274 | 295 | Carbamidomethylation | C19:Carbamidomethylation:1000.00 | PEAKS DB |
| R.VYELVNILNTILR.R | N | 76.43 | 1558.9082 | 13 | 4.2 | 780.4647 | 2 | 100.36 | 1 | 72488 | 01122020\_RID\_2209\_BuCaKA02.raw | 2.5848E8 | 12 | 12 | 208 | 220 |  |  | PEAKS DB |
| R.RVYELVNILNTILR.R | N | 75.99 | 1715.0094 | 14 | -0.8 | 572.6766 | 3 | 82.30 | 1 | 57621 | 01122020\_RID\_2209\_BuCaKA02.raw | 1.0959E8 | 2 | 2 | 207 | 220 |  |  | PEAKS DB |
| L.GSLC(+57.02)SVKYSVAVIQDYSKR.T | N | 75.11 | 2159.1045 | 19 | 2.7 | 720.7107 | 3 | 35.42 | 1 | 20245 | 01122020\_RID\_2209\_BuCaKA02.raw | 4.748E7 | 1 | 1 | 289 | 307 | Carbamidomethylation | C4:Carbamidomethylation:1000.00 | PEAKS DB |
| G.NTVGLAALGSLC(+57.02)SVK.Y | N | 74.25 | 1488.7970 | 15 | 3.1 | 745.4081 | 2 | 54.18 | 1 | 34449 | 01122020\_RID\_2209\_BuCaKA02.raw | 3.2913E6 | 1 | 1 | 281 | 295 | Carbamidomethylation | C12:Carbamidomethylation:1000.00 | PEAKS DB |
| R.DKINVQSDVKATLK.S | N | 74.05 | 1557.8726 | 14 | 2.9 | 520.2996 | 3 | 15.58 | 1 | 4756 | 01122020\_RID\_2209\_BuCaKA02.raw | 1.9437E4 | 3 | 3 | 238 | 251 |  |  | PEAKS DB |
| K.VKKYIELYMAVDNR.M | Y | 73.89 | 1740.9232 | 14 | 1.3 | 581.3158 | 3 | 27.71 | 1 | 14116 | 01122020\_RID\_2209\_BuCaKA02.raw | 7.416E6 | 1 | 1 | 179 | 192 |  |  | PEAKS DB |
| K.GNTVGLAALGSLC(+57.02)SVKYSVAVIQDYSK.R | N | 73.80 | 2799.4475 | 27 | 0.7 | 934.1571 | 3 | 74.40 | 1 | 51257 | 01122020\_RID\_2209\_BuCaKA02.raw | 3.0528E6 | 1 | 1 | 280 | 306 | Carbamidomethylation | C13:Carbamidomethylation:1000.00 | PEAKS DB |
| L.AALGSLC(+57.02)SVKYSVAVIQDYSKR.T | N | 73.79 | 2414.2627 | 22 | 0.8 | 805.7621 | 3 | 48.80 | 1 | 30105 | 01122020\_RID\_2209\_BuCaKA02.raw | 4.4384E7 | 1 | 1 | 286 | 307 | Carbamidomethylation | C7:Carbamidomethylation:1000.00 | PEAKS DB |
| R.RVYELVNILNTILRR.L | N | 73.73 | 1871.1105 | 15 | 2.3 | 624.7122 | 3 | 74.19 | 1 | 50882 | 01122020\_RID\_2209\_BuCaKA02.raw | 1.819E8 | 2 | 2 | 207 | 221 |  |  | PEAKS DB |
| S.VKYSVAVIQDYSK.R | N | 73.13 | 1498.8031 | 13 | 2.1 | 750.4104 | 2 | 27.40 | 1 | 14044 | 01122020\_RID\_2209\_BuCaKA02.raw | 9.3489E6 | 1 | 1 | 294 | 306 |  |  | PEAKS DB |
| K.RRVYELVNILNTILR.R | N | 73.13 | 1871.1105 | 15 | 1.4 | 624.7117 | 3 | 72.32 | 1 | 49628 | 01122020\_RID\_2209\_BuCaKA02.raw | 3.7773E7 | 2 | 2 | 206 | 220 |  |  | PEAKS DB |
| S.VKYSVAVIQDYSKR.T | N | 72.52 | 1654.9042 | 14 | 1.5 | 828.4606 | 2 | 21.08 | 1 | 8895 | 01122020\_RID\_2209\_BuCaKA02.raw | 1.795E6 | 1 | 1 | 294 | 307 |  |  | PEAKS DB |
| G.LAALGSLC(+57.02)SVKYSVAVIQDYSKR.T | N | 71.17 | 2527.3467 | 23 | 1.6 | 632.8450 | 4 | 56.26 | 1 | 36281 | 01122020\_RID\_2209\_BuCaKA02.raw | 4.7681E6 | 1 | 1 | 285 | 307 | Carbamidomethylation | C8:Carbamidomethylation:1000.00 | PEAKS DB |
| R.NGLPC(+57.02)RNNQGYC(+57.02)YNGKC(+57.02)PTLTNQC(+57.02)IALMGPNVK.A | N | 71.08 | 3811.7473 | 33 | 1.5 | 953.9456 | 4 | 37.05 | 1 | 21303 | 01122020\_RID\_2209\_BuCaKA02.raw | 9.2632E8 | 2 | 2 | 471 | 503 | Carbamidomethylation | C5:Carbamidomethylation:1000.00;C12:Carbamidomethylation:1000.00;C17:Carbamidomethylation:1000.00;C24:Carbamidomethylation:1000.00 | PEAKS DB |
| R.VYELVNILNTILRR.L | N | 70.77 | 1715.0094 | 14 | 0.6 | 858.5125 | 2 | 87.67 | 1 | 62336 | 01122020\_RID\_2209\_BuCaKA02.raw | 7.9997E8 | 22 | 22 | 208 | 221 |  |  | PEAKS DB |
| V.YELVNILNTILR.R | N | 68.64 | 1459.8398 | 12 | 5.3 | 730.9310 | 2 | 92.27 | 1 | 66192 | 01122020\_RID\_2209\_BuCaKA02.raw | 1.0989E8 | 3 | 3 | 209 | 220 |  |  | PEAKS DB |
| R.VC(+57.02)NC(+57.02)LILPDDPNYGMVETGTK.C | N | 68.59 | 2395.0857 | 21 | -0.1 | 1198.5500 | 2 | 58.89 | 1 | 38316 | 01122020\_RID\_2209\_BuCaKA02.raw | 4.5386E8 | 2 | 2 | 551 | 571 | Carbamidomethylation | C2:Carbamidomethylation:1000.00;C4:Carbamidomethylation:1000.00 | PEAKS DB |
| L.NFHIALIGLEIWSKR.D | N | 68.46 | 1796.0096 | 15 | 0.6 | 599.6775 | 3 | 64.95 | 1 | 43392 | 01122020\_RID\_2209\_BuCaKA02.raw | 5.4615E6 | 1 | 1 | 223 | 237 |  |  | PEAKS DB |
| R.NNQGYC(+57.02)YNGKC(+57.02)PTLTNQC(+57.02)IALMGPNVK.A | N | 67.23 | 3114.4143 | 27 | 1.1 | 1558.2161 | 2 | 41.61 | 1 | 24894 | 01122020\_RID\_2209\_BuCaKA02.raw | 3.2808E8 | 3 | 3 | 477 | 503 | Carbamidomethylation | C6:Carbamidomethylation:1000.00;C11:Carbamidomethylation:1000.00;C18:Carbamidomethylation:1000.00 | PEAKS DB |
| C.YNGKC(+57.02)PTLTNQC(+57.02)IALMGPNVK.A | N | 65.75 | 2378.1545 | 21 | 0.1 | 1190.0847 | 2 | 36.28 | 1 | 20814 | 01122020\_RID\_2209\_BuCaKA02.raw | 6.173E7 | 1 | 1 | 483 | 503 | Carbamidomethylation | C5:Carbamidomethylation:1000.00;C12:Carbamidomethylation:1000.00 | PEAKS DB |
| I.ALIGLEIWSK.R | N | 61.16 | 1128.6543 | 10 | 0.6 | 565.3348 | 2 | 67.13 | 1 | 45199 | 01122020\_RID\_2209\_BuCaKA02.raw | 2.8404E6 | 1 | 1 | 227 | 236 |  |  | PEAKS DB |
| H.IALIGLEIWSK.R | N | 61.01 | 1241.7383 | 11 | 4.0 | 621.8789 | 2 | 77.42 | 1 | 53834 | 01122020\_RID\_2209\_BuCaKA02.raw | 2.4633E7 | 1 | 1 | 226 | 236 |  |  | PEAKS DB |
| K.INVQSDVKATLK.S | N | 60.79 | 1314.7507 | 12 | 1.0 | 658.3833 | 2 | 17.83 | 1 | 6419 | 01122020\_RID\_2209\_BuCaKA02.raw | 3.7588E6 | 1 | 1 | 240 | 251 |  |  | PEAKS DB |
| R.NNQGYC(+57.02)YNGKC(+57.02)PTLTNQC(+57.02)IALMGPNVKASR.D | N | 60.31 | 3428.5847 | 30 | 0.6 | 858.1539 | 4 | 34.90 | 1 | 19665 | 01122020\_RID\_2209\_BuCaKA02.raw | 2.2249E7 | 1 | 1 | 477 | 506 | Carbamidomethylation | C6:Carbamidomethylation:1000.00;C11:Carbamidomethylation:1000.00;C18:Carbamidomethylation:1000.00 | PEAKS DB |
| H.IALIGLEIWSKR.D | N | 58.71 | 1397.8395 | 12 | 0.8 | 699.9276 | 2 | 62.08 | 1 | 41072 | 01122020\_RID\_2209\_BuCaKA02.raw | 1.3504E8 | 1 | 1 | 226 | 237 |  |  | PEAKS DB |
| R.NGLPC(+57.02)RNNQGYC(+57.02)YNGKC(+57.02)PTLTNQC(+57.02)IALM(+15.99)GPNVK.A | N | 58.17 | 3827.7422 | 33 | 2.4 | 1276.9244 | 3 | 33.05 | 1 | 18101 | 01122020\_RID\_2209\_BuCaKA02.raw | 9.6615E7 | 2 | 2 | 471 | 503 | Carbamidomethylation; Oxidation (M) | C5:Carbamidomethylation:1000.00;C12:Carbamidomethylation:1000.00;C17:Carbamidomethylation:1000.00;C24:Carbamidomethylation:1000.00;M28:Oxidation (M):1000.00 | PEAKS DB |
| R.IDFKGNTVGLAALGSL.C | Y | 58.09 | 1574.8667 | 16 | 2.1 | 788.4423 | 2 | 74.83 | 1 | 51742 | 01122020\_RID\_2209\_BuCaKA02.raw | 5.7233E7 | 1 | 1 | 276 | 291 |  |  | PEAKS DB |
| Y.ELVNILNTILR.R | N | 57.08 | 1296.7765 | 11 | 1.6 | 649.3965 | 2 | 83.53 | 1 | 58885 | 01122020\_RID\_2209\_BuCaKA02.raw | 2.3504E8 | 3 | 3 | 210 | 220 |  |  | PEAKS DB |
| R.DSC(+57.02)FTLNQR.G | N | 56.35 | 1139.5029 | 9 | 0.9 | 570.7593 | 2 | 23.69 | 1 | 10975 | 01122020\_RID\_2209\_BuCaKA02.raw | 6.8626E6 | 1 | 1 | 507 | 515 | Carbamidomethylation | C3:Carbamidomethylation:1000.00 | PEAKS DB |
| R.VYELVNILNTILRRLNFH.I | N | 55.34 | 2226.2637 | 18 | 2.0 | 743.0966 | 3 | 102.90 | 1 | 75118 | 01122020\_RID\_2209\_BuCaKA02.raw | 1.1381E7 | 1 | 1 | 208 | 225 |  |  | PEAKS DB |
| R.RLNFHIALIGLEIWSKR.D | N | 54.39 | 2065.1948 | 17 | 3.5 | 689.4080 | 3 | 63.58 | 1 | 42153 | 01122020\_RID\_2209\_BuCaKA02.raw | 6.407E7 | 1 | 1 | 221 | 237 |  |  | PEAKS DB |
| K.SISDEPHSEFSSC(+57.02)SVQEHQRYLLK.D | N | 51.51 | 2862.3242 | 24 | 0.8 | 716.5889 | 4 | 22.77 | 1 | 10269 | 01122020\_RID\_2209\_BuCaKA02.raw | 4.272E6 | 1 | 1 | 344 | 367 | Carbamidomethylation | C13:Carbamidomethylation:1000.00 | PEAKS DB |
| V.C(+57.02)NC(+57.02)LILPDDPNYGMVETGTK.C | N | 51.10 | 2296.0173 | 20 | 0.1 | 1149.0161 | 2 | 57.12 | 1 | 36890 | 01122020\_RID\_2209\_BuCaKA02.raw | 4.1311E7 | 1 | 1 | 552 | 571 | Carbamidomethylation | C1:Carbamidomethylation:1000.00;C3:Carbamidomethylation:1000.00 | PEAKS DB |
| R.VYELVNILNTIL.R | N | 49.98 | 1402.8071 | 12 | 1.1 | 702.4116 | 2 | 122.78 | 1 | 90483 | 01122020\_RID\_2209\_BuCaKA02.raw | 4.8269E6 | 1 | 1 | 208 | 219 |  |  | PEAKS DB |
| R.IDFKGNTVGLAALGSLC(+57.02).S | Y | 47.99 | 1734.8975 | 17 | 1.1 | 868.4570 | 2 | 73.26 | 1 | 50242 | 01122020\_RID\_2209\_BuCaKA02.raw | 3.7098E7 | 1 | 1 | 276 | 292 | Carbamidomethylation | C17:Carbamidomethylation:1000.00 | PEAKS DB |
| E.LVNILNTILR.R | N | 47.27 | 1167.7339 | 10 | 2.0 | 584.8754 | 2 | 72.87 | 1 | 49866 | 01122020\_RID\_2209\_BuCaKA02.raw | 2.8702E7 | 1 | 1 | 211 | 220 |  |  | PEAKS DB |
| R.IDFKGNTVGLAALGSLC(+57.02)SVKYS.V | Y | 45.97 | 2299.1882 | 22 | 1.1 | 1150.6027 | 2 | 65.95 | 1 | 44235 | 01122020\_RID\_2209\_BuCaKA02.raw | 0 | 0 | 0 | 276 | 297 | Carbamidomethylation | C17:Carbamidomethylation:1000.00 | PEAKS DB |
| R.NNQGYC(+57.02)YNGKC(+57.02)PTLTNQC(+57.02)IAL.M | N | 44.45 | 2488.0933 | 21 | 1.4 | 1245.0557 | 2 | 45.14 | 1 | 27453 | 01122020\_RID\_2209\_BuCaKA02.raw | 1.2974E7 | 1 | 1 | 477 | 497 | Carbamidomethylation | C6:Carbamidomethylation:1000.00;C11:Carbamidomethylation:1000.00;C18:Carbamidomethylation:1000.00 | PEAKS DB |
| R.RVYELVNILNTIL.R | N | 44.30 | 1558.9082 | 13 | 2.4 | 780.4633 | 2 | 95.52 | 1 | 68628 | 01122020\_RID\_2209\_BuCaKA02.raw | 2.1584E6 | 1 | 1 | 207 | 219 |  |  | PEAKS DB |
| V.YELVNILNTILRR.L | N | 40.63 | 1615.9409 | 13 | 1.1 | 808.9786 | 2 | 80.27 | 1 | 56086 | 01122020\_RID\_2209\_BuCaKA02.raw | 4.0753E6 | 1 | 1 | 209 | 221 |  |  | PEAKS DB |
| total 49 peptides |  |  |  |  |  |  |  |  |  |  |  |  |  |  |  |  |  |  |  |
| --- | --- | --- | --- | --- | --- | --- | --- | --- | --- | --- | --- | --- | --- | --- | --- | --- | --- | --- | --- |

T\_35

back to list

  

| Protein Coverage
| Supporting Peptides
|

Protein Coverage:

Supporting Peptides:

| Peptide | Uniq | -10lgP | Mass | Length | ppm | m/z | z | RT | Fraction | Scan | Source File | Area BRom | #Feature | #Feature BRom | Start | End | PTM | AScore | Found By |
| --- | --- | --- | --- | --- | --- | --- | --- | --- | --- | --- | --- | --- | --- | --- | --- | --- | --- | --- | --- |
| K.ISGYILPYKIINVGSEKVGIIGYTTK.E | N | 97.22 | 2825.5942 | 26 | 1.1 | 707.4066 | 4 | 64.17 | 1 | 42708 | 01122020\_RID\_2209\_BuCaKA02.raw | 2.0622E8 | 2 | 2 | 168 | 193 |  |  | PEAKS DB |
| K.ASGNPILLNKSIQEDPAVKAEVSR.M | Y | 90.98 | 2535.3655 | 24 | 2.3 | 634.8501 | 4 | 30.94 | 1 | 16417 | 01122020\_RID\_2209\_BuCaKA02.raw | 3.4664E7 | 2 | 2 | 317 | 340 |  |  | PEAKS DB |
| R.NVLLLDAGDQYQGTVWFNYFKGR.E | Y | 87.10 | 2703.3445 | 23 | 1.7 | 902.1237 | 3 | 80.71 | 1 | 56399 | 01122020\_RID\_2209\_BuCaKA02.raw | 3.7945E7 | 2 | 2 | 91 | 113 |  |  | PEAKS DB |
| K.LTTLGVNKIIALGHSGFKEDC(+57.02)R.I | Y | 84.69 | 2428.2896 | 22 | 0.5 | 608.0800 | 4 | 38.10 | 1 | 22251 | 01122020\_RID\_2209\_BuCaKA02.raw | 5.1088E6 | 1 | 1 | 221 | 242 | Carbamidomethylation | C21:Carbamidomethylation:1000.00 | PEAKS DB |
| R.HGQGSGELLQVSGIKVVYDLSQKPGKR.V | Y | 84.47 | 2879.5618 | 27 | 1.5 | 576.9205 | 5 | 37.42 | 1 | 21629 | 01122020\_RID\_2209\_BuCaKA02.raw | 7.7281E7 | 2 | 2 | 457 | 483 |  |  | PEAKS DB |
| R.MKVQLQSYSSQVIGK.T | Y | 83.18 | 1694.9025 | 15 | 0.6 | 848.4590 | 2 | 27.68 | 1 | 14243 | 01122020\_RID\_2209\_BuCaKA02.raw | 1.2487E8 | 2 | 2 | 341 | 355 |  |  | PEAKS DB |
| K.YLGYLNVIFDDKGKVIK.A | Y | 78.91 | 1984.1033 | 17 | 2.1 | 662.3764 | 3 | 55.49 | 1 | 35690 | 01122020\_RID\_2209\_BuCaKA02.raw | 2.0973E8 | 2 | 2 | 300 | 316 |  |  | PEAKS DB |
| K.ISGYILPYKIINVGSEK.V | N | 78.21 | 1893.0610 | 17 | 2.0 | 632.0289 | 3 | 55.31 | 1 | 35284 | 01122020\_RID\_2209\_BuCaKA02.raw | 1.345E8 | 1 | 1 | 168 | 184 |  |  | PEAKS DB |
| R.ATHRNVLLLDAGDQYQGTVWFNYFKGR.E | Y | 76.85 | 3168.5894 | 27 | 1.4 | 793.1558 | 4 | 61.48 | 1 | 40465 | 01122020\_RID\_2209\_BuCaKA02.raw | 2.5236E8 | 4 | 4 | 87 | 113 |  |  | PEAKS DB |
| K.GPIASKISGYILPYKIINVGSEK.V | Y | 76.23 | 2446.3835 | 23 | 1.9 | 612.6043 | 4 | 55.41 | 1 | 35550 | 01122020\_RID\_2209\_BuCaKA02.raw | 6.6678E7 | 1 | 1 | 162 | 184 |  |  | PEAKS DB |
| R.QVPVVQAYAFGK.Y | N | 74.86 | 1305.7081 | 12 | 1.9 | 653.8625 | 2 | 42.08 | 1 | 25183 | 01122020\_RID\_2209\_BuCaKA02.raw | 1.5608E7 | 1 | 1 | 288 | 299 |  |  | PEAKS DB |
| R.ATHRNVLLLDAGDQYQGTVWFNYFK.G | Y | 74.70 | 2955.4668 | 25 | 3.4 | 739.8765 | 4 | 69.33 | 1 | 46967 | 01122020\_RID\_2209\_BuCaKA02.raw | 7.4329E7 | 3 | 3 | 87 | 111 |  |  | PEAKS DB |
| K.SIQEDPAVKAEVSR.M | Y | 74.61 | 1527.7892 | 14 | 1.6 | 764.9031 | 2 | 12.11 | 1 | 2075 | 01122020\_RID\_2209\_BuCaKA02.raw | 8.4204E5 | 1 | 1 | 327 | 340 |  |  | PEAKS DB |
| R.YDAMALGNHEFDNGLNGLLDPLLKNVKFPILSANIRPK.G | Y | 74.03 | 4207.2251 | 38 | 1.0 | 842.4531 | 5 | 84.37 | 1 | 59405 | 01122020\_RID\_2209\_BuCaKA02.raw | 1.2988E7 | 1 | 1 | 124 | 161 |  |  | PEAKS DB |
| L.GNHEFDNGLNGLLDPLLKNVKFPILSANIRPK.G | Y | 73.58 | 3542.9360 | 32 | 0.9 | 709.5952 | 5 | 77.15 | 1 | 53585 | 01122020\_RID\_2209\_BuCaKA02.raw | 1.9863E7 | 2 | 2 | 130 | 161 |  |  | PEAKS DB |
| G.DSSNHNSGDLDISIVSDYIKR.M | Y | 73.40 | 2334.1086 | 21 | 2.0 | 779.0450 | 3 | 54.19 | 1 | 34699 | 01122020\_RID\_2209\_BuCaKA02.raw | 9.655E7 | 4 | 4 | 529 | 549 |  |  | PEAKS DB |
| Q.VPVVQAYAFGK.Y | N | 71.48 | 1177.6495 | 11 | 1.1 | 589.8327 | 2 | 38.91 | 1 | 22947 | 01122020\_RID\_2209\_BuCaKA02.raw | 6.1249E5 | 1 | 1 | 289 | 299 |  |  | PEAKS DB |
| K.ETPILSNPGPYLEFR.D | Y | 70.08 | 1731.8832 | 15 | 0.1 | 866.9490 | 2 | 65.11 | 1 | 43441 | 01122020\_RID\_2209\_BuCaKA02.raw | 5.5765E7 | 1 | 1 | 194 | 208 |  |  | PEAKS DB |
| K.YLGYLNVIFDDKGK.V | Y | 69.27 | 1643.8558 | 14 | 1.3 | 822.9363 | 2 | 62.23 | 1 | 41088 | 01122020\_RID\_2209\_BuCaKA02.raw | 2.3839E7 | 1 | 1 | 300 | 313 |  |  | PEAKS DB |
| K.VGIIGYTTKETPILSNPGPYLEFR.D | Y | 69.07 | 2664.4163 | 24 | 0.4 | 889.1464 | 3 | 68.11 | 1 | 46095 | 01122020\_RID\_2209\_BuCaKA02.raw | 8.0267E7 | 1 | 1 | 185 | 208 |  |  | PEAKS DB |
| A.GSFKLTILHTNDVHAR.V | Y | 68.98 | 1807.9692 | 16 | 1.6 | 603.6647 | 3 | 19.57 | 1 | 7750 | 01122020\_RID\_2209\_BuCaKA02.raw | 1.1225E7 | 2 | 2 | 40 | 55 |  |  | PEAKS DB |
| E.TPILSNPGPYLEFR.D | Y | 67.29 | 1602.8406 | 14 | 0.8 | 802.4282 | 2 | 61.12 | 1 | 40259 | 01122020\_RID\_2209\_BuCaKA02.raw | 1.503E7 | 1 | 1 | 195 | 208 |  |  | PEAKS DB |
| R.QVPVVQAYAFGKYLGYL.N | N | 67.18 | 1915.0243 | 17 | 0.6 | 958.5200 | 2 | 82.93 | 1 | 58321 | 01122020\_RID\_2209\_BuCaKA02.raw | 1.5413E8 | 2 | 2 | 288 | 304 |  |  | PEAKS DB |
| K.VQLQSYSSQVIGK.T | Y | 63.95 | 1435.7671 | 13 | 1.9 | 718.8922 | 2 | 30.21 | 1 | 15960 | 01122020\_RID\_2209\_BuCaKA02.raw | 6.9334E6 | 1 | 1 | 343 | 355 |  |  | PEAKS DB |
| R.VVSLNVLC(+57.02)TEC(+57.02)RVPTYVPLEMKK.T | Y | 62.65 | 2734.4219 | 23 | 2.4 | 684.6144 | 4 | 54.14 | 1 | 34480 | 01122020\_RID\_2209\_BuCaKA02.raw | 1.9293E7 | 1 | 1 | 484 | 506 | Carbamidomethylation | C8:Carbamidomethylation:1000.00;C11:Carbamidomethylation:1000.00 | PEAKS DB |
| M.ALGNHEFDNGLNGLLDPLLK.N | Y | 62.65 | 2149.1167 | 20 | 1.8 | 717.3808 | 3 | 74.49 | 1 | 51310 | 01122020\_RID\_2209\_BuCaKA02.raw | 1.0742E6 | 1 | 1 | 128 | 147 |  |  | PEAKS DB |
| A.GSFKLTILHTNDVHARVEQTSR.D | Y | 61.69 | 2508.3196 | 22 | 1.1 | 628.0879 | 4 | 19.87 | 1 | 8002 | 01122020\_RID\_2209\_BuCaKA02.raw | 1.2654E7 | 1 | 1 | 40 | 61 |  |  | PEAKS DB |
| L.GNHEFDNGLNGLLDPLLK.N | Y | 61.43 | 1964.9955 | 18 | 3.5 | 656.0081 | 3 | 71.62 | 1 | 48917 | 01122020\_RID\_2209\_BuCaKA02.raw | 0 | 0 | 0 | 130 | 147 |  |  | PEAKS DB |
| F.DNGLNGLLDPLLK.N | Y | 56.77 | 1380.7612 | 13 | 2.0 | 691.3893 | 2 | 83.74 | 1 | 58962 | 01122020\_RID\_2209\_BuCaKA02.raw | 0 | 0 | 0 | 135 | 147 |  |  | PEAKS DB |
| L.KGDSSNHNSGDLDISIVSDYIKR.M | Y | 55.54 | 2519.2251 | 23 | 2.1 | 630.8149 | 4 | 40.63 | 1 | 24203 | 01122020\_RID\_2209\_BuCaKA02.raw | 9.8917E6 | 1 | 1 | 527 | 549 |  |  | PEAKS DB |
| G.DSSNHNSGDLDISIVSDYIK.R | Y | 55.30 | 2178.0076 | 20 | -0.5 | 727.0095 | 3 | 65.83 | 1 | 44064 | 01122020\_RID\_2209\_BuCaKA02.raw | 7.3596E6 | 1 | 1 | 529 | 548 |  |  | PEAKS DB |
| R.QVPVVQAYAFGKYLGY.L | N | 46.91 | 1801.9402 | 16 | 1.5 | 901.9788 | 2 | 99.81 | 1 | 72177 | 01122020\_RID\_2209\_BuCaKA02.raw | 2.7558E5 | 1 | 1 | 288 | 303 |  |  | PEAKS DB |
| K.GVDVVVGGHTNT.F | N | 42.35 | 1153.5728 | 12 | 2.0 | 577.7948 | 2 | 17.43 | 1 | 6387 | 01122020\_RID\_2209\_BuCaKA02.raw | 1.2061E6 | 1 | 1 | 249 | 260 |  |  | PEAKS DB |
| R.NVLLLDAGDQYQGTVWFNYFK.G | Y | 41.75 | 2490.2219 | 21 | 0.9 | 1246.1194 | 2 | 94.14 | 1 | 67510 | 01122020\_RID\_2209\_BuCaKA02.raw | 7.2772E5 | 1 | 1 | 91 | 111 |  |  | PEAKS DB |
| R.QVPVVQAYAFGKYLGYLNVIFDDKGK.V | Y | 40.92 | 2931.5535 | 26 | 0.3 | 733.8959 | 4 | 84.28 | 1 | 59385 | 01122020\_RID\_2209\_BuCaKA02.raw | 1.7157E6 | 1 | 1 | 288 | 313 |  |  | PEAKS DB |
| total 35 peptides |  |  |  |  |  |  |  |  |  |  |  |  |  |  |  |  |  |  |  |
| --- | --- | --- | --- | --- | --- | --- | --- | --- | --- | --- | --- | --- | --- | --- | --- | --- | --- | --- | --- |

T\_114

back to list

  

| Protein Coverage
| Supporting Peptides
|

Protein Coverage:

Supporting Peptides:

| Peptide | Uniq | -10lgP | Mass | Length | ppm | m/z | z | RT | Fraction | Scan | Source File | Area BRom | #Feature | #Feature BRom | Start | End | PTM | AScore | Found By |
| --- | --- | --- | --- | --- | --- | --- | --- | --- | --- | --- | --- | --- | --- | --- | --- | --- | --- | --- | --- |
| K.TYSYTC(+57.02)TKPDLTC(+57.02)TDAAGSC(+57.02)AR.F | Y | 93.21 | 2498.0513 | 22 | 1.8 | 833.6925 | 3 | 22.09 | 1 | 10078 | 01122020\_RID\_2209\_BuCaKA02.raw | 2.0345E8 | 2 | 2 | 49 | 70 | Carbamidomethylation | C6:Carbamidomethylation:1000.00;C13:Carbamidomethylation:1000.00;C20:Carbamidomethylation:1000.00 | PEAKS DB |
| K.APYNIGNIMISSSTTC(+57.02)K | Y | 92.17 | 1855.8809 | 17 | 1.5 | 928.9491 | 2 | 49.78 | 1 | 31251 | 01122020\_RID\_2209\_BuCaKA02.raw | 1.776E9 | 2 | 2 | 86 | 102 | Carbamidomethylation | C16:Carbamidomethylation:1000.00 | PEAKS DB |
| L.KYGC(+57.02)YC(+57.02)GYGGTGTPVDELDR.C | N | 88.44 | 2266.9624 | 20 | -2.4 | 1134.4857 | 2 | 32.64 | 1 | 17782 | 01122020\_RID\_2209\_BuCaKA02.raw | 3.5219E7 | 2 | 2 | 6 | 25 | Carbamidomethylation | C4:Carbamidomethylation:1000.00;C6:Carbamidomethylation:1000.00 | PEAKS DB |
| K.YGC(+57.02)YC(+57.02)GYGGTGTPVDELDR.C | N | 88.01 | 2138.8674 | 19 | 0.4 | 1070.4414 | 2 | 44.33 | 1 | 26645 | 01122020\_RID\_2209\_BuCaKA02.raw | 9.6633E9 | 3 | 3 | 7 | 25 | Carbamidomethylation | C3:Carbamidomethylation:1000.00;C5:Carbamidomethylation:1000.00 | PEAKS DB |
| Y.GC(+57.02)YC(+57.02)GYGGTGTPVDELDR.C | N | 85.01 | 1975.8040 | 18 | 0.4 | 988.9097 | 2 | 38.86 | 1 | 23078 | 01122020\_RID\_2209\_BuCaKA02.raw | 3.3628E9 | 2 | 2 | 8 | 25 | Carbamidomethylation | C2:Carbamidomethylation:1000.00;C4:Carbamidomethylation:1000.00 | PEAKS DB |
| K.APYNIGNIM(+15.99)ISSSTTC(+57.02)K | Y | 82.39 | 1871.8757 | 17 | 0.8 | 936.9459 | 2 | 40.67 | 1 | 24331 | 01122020\_RID\_2209\_BuCaKA02.raw | 1.2934E8 | 1 | 1 | 86 | 102 | Oxidation (M); Carbamidomethylation | M9:Oxidation (M):1000.00;C16:Carbamidomethylation:1000.00 | PEAKS DB |
| Y.C(+57.02)GYGGTGTPVDELDR.C | N | 82.21 | 1595.6886 | 15 | 1.6 | 798.8528 | 2 | 33.04 | 1 | 18122 | 01122020\_RID\_2209\_BuCaKA02.raw | 5.3035E8 | 3 | 3 | 11 | 25 | Carbamidomethylation | C1:Carbamidomethylation:1000.00 | PEAKS DB |
| C.YC(+57.02)GYGGTGTPVDELDR.C | N | 81.92 | 1758.7518 | 16 | 0.3 | 880.3834 | 2 | 37.99 | 1 | 22078 | 01122020\_RID\_2209\_BuCaKA02.raw | 4.9681E7 | 1 | 1 | 10 | 25 | Carbamidomethylation | C2:Carbamidomethylation:1000.00 | PEAKS DB |
| R.FLC(+57.02)DC(+57.02)DRTAALC(+57.02)FAK.A | N | 80.82 | 1846.8164 | 15 | -0.1 | 924.4154 | 2 | 36.40 | 1 | 20993 | 01122020\_RID\_2209\_BuCaKA02.raw | 1.3034E10 | 13 | 13 | 71 | 85 | Carbamidomethylation | C3:Carbamidomethylation:1000.00;C5:Carbamidomethylation:1000.00;C12:Carbamidomethylation:1000.00 | PEAKS DB |
| R.C(+57.02)C(+57.02)YVHDNC(+57.02)YDEAKKIHGC(+57.02)NPTTK.T | N | 80.25 | 2869.2041 | 23 | 1.4 | 574.8489 | 5 | 12.05 | 1 | 2027 | 01122020\_RID\_2209\_BuCaKA02.raw | 0 | 0 | 0 | 26 | 48 | Carbamidomethylation | C1:Carbamidomethylation:1000.00;C2:Carbamidomethylation:1000.00;C8:Carbamidomethylation:1000.00;C18:Carbamidomethylation:1000.00 | PEAKS DB |
| G.C(+57.02)YC(+57.02)GYGGTGTPVDELDR.C | N | 77.93 | 1918.7826 | 17 | 1.1 | 960.3996 | 2 | 38.92 | 1 | 22863 | 01122020\_RID\_2209\_BuCaKA02.raw | 7.0014E7 | 2 | 2 | 9 | 25 | Carbamidomethylation | C1:Carbamidomethylation:1000.00;C3:Carbamidomethylation:1000.00 | PEAKS DB |
| R.TAALC(+57.02)FAKAPYNIGNIMISSSTTC(+57.02)K | Y | 75.65 | 2718.3179 | 25 | 0.5 | 907.1137 | 3 | 60.88 | 1 | 40112 | 01122020\_RID\_2209\_BuCaKA02.raw | 3.1585E8 | 2 | 2 | 78 | 102 | Carbamidomethylation | C5:Carbamidomethylation:1000.00;C24:Carbamidomethylation:1000.00 | PEAKS DB |
| S.C(+57.02)ARFLC(+57.02)DC(+57.02)DRTAALC(+57.02)FAK.A | N | 75.35 | 2233.9854 | 18 | 2.1 | 559.5048 | 4 | 29.81 | 1 | 15728 | 01122020\_RID\_2209\_BuCaKA02.raw | 1.7382E7 | 1 | 1 | 68 | 85 | Carbamidomethylation | C1:Carbamidomethylation:1000.00;C6:Carbamidomethylation:1000.00;C8:Carbamidomethylation:1000.00;C15:Carbamidomethylation:1000.00 | PEAKS DB |
| C.GYGGTGTPVDELDR.C | N | 70.03 | 1435.6580 | 14 | 1.4 | 718.8373 | 2 | 31.57 | 1 | 17094 | 01122020\_RID\_2209\_BuCaKA02.raw | 9.8214E7 | 1 | 1 | 12 | 25 |  |  | PEAKS DB |
| Y.LKYGC(+57.02)YC(+57.02)GYGGTGTPVDELDR.C | N | 69.19 | 2380.0464 | 21 | 0.9 | 794.3568 | 3 | 38.32 | 1 | 22508 | 01122020\_RID\_2209\_BuCaKA02.raw | 1.3586E8 | 1 | 1 | 5 | 25 | Carbamidomethylation | C5:Carbamidomethylation:1000.00;C7:Carbamidomethylation:1000.00 | PEAKS DB |
| G.YGGTGTPVDELDR.C | N | 68.14 | 1378.6365 | 13 | 1.7 | 690.3267 | 2 | 30.08 | 1 | 15917 | 01122020\_RID\_2209\_BuCaKA02.raw | 1.3749E8 | 1 | 1 | 13 | 25 |  |  | PEAKS DB |
| T.TKTYSYTC(+57.02)TKPDLTC(+57.02)TDAAGSC(+57.02)AR.F | Y | 67.48 | 2727.1938 | 24 | 2.2 | 682.8073 | 4 | 14.35 | 1 | 3882 | 01122020\_RID\_2209\_BuCaKA02.raw | 6.801E6 | 2 | 2 | 47 | 70 | Carbamidomethylation | C8:Carbamidomethylation:1000.00;C15:Carbamidomethylation:1000.00;C22:Carbamidomethylation:1000.00 | PEAKS DB |
| K.YGC(+57.02)YC(+57.02)GYGGTGTPVDELDRC(+57.02)C(+57.02)YVHDNC(+57.02)YDEAK.K | N | 66.01 | 3853.4846 | 32 | 0.6 | 1285.5029 | 3 | 41.35 | 1 | 24801 | 01122020\_RID\_2209\_BuCaKA02.raw | 3.1534E9 | 2 | 2 | 7 | 38 | Carbamidomethylation | C3:Carbamidomethylation:1000.00;C5:Carbamidomethylation:1000.00;C20:Carbamidomethylation:1000.00;C21:Carbamidomethylation:1000.00;C27:Carbamidomethylation:1000.00 | PEAKS DB |
| T.YSYTC(+57.02)TKPDLTC(+57.02)TDAAGSC(+57.02)AR.F | Y | 64.81 | 2397.0034 | 21 | 1.9 | 800.0099 | 3 | 20.99 | 1 | 8853 | 01122020\_RID\_2209\_BuCaKA02.raw | 1.9379E6 | 1 | 1 | 50 | 70 | Carbamidomethylation | C5:Carbamidomethylation:1000.00;C12:Carbamidomethylation:1000.00;C19:Carbamidomethylation:1000.00 | PEAKS DB |
| K.YGC(+57.02)YC(+57.02)GYGGTGTPVDELDRC(+57.02)C(+57.02).Y | N | 63.90 | 2458.9287 | 21 | 1.8 | 1230.4739 | 2 | 48.28 | 1 | 29764 | 01122020\_RID\_2209\_BuCaKA02.raw | 8.9471E6 | 1 | 1 | 7 | 27 | Carbamidomethylation | C3:Carbamidomethylation:1000.00;C5:Carbamidomethylation:1000.00;C20:Carbamidomethylation:1000.00;C21:Carbamidomethylation:1000.00 | PEAKS DB |
| R.TAALC(+57.02)FAKAPYNI.G | N | 62.47 | 1438.7278 | 13 | 3.0 | 720.3733 | 2 | 50.01 | 1 | 31149 | 01122020\_RID\_2209\_BuCaKA02.raw | 0 | 0 | 0 | 78 | 90 | Carbamidomethylation | C5:Carbamidomethylation:1000.00 | PEAKS DB |
| F.LC(+57.02)DC(+57.02)DRTAALC(+57.02)FAK.A | N | 61.05 | 1699.7480 | 14 | 2.4 | 567.5913 | 3 | 22.40 | 1 | 9949 | 01122020\_RID\_2209\_BuCaKA02.raw | 3.0159E7 | 2 | 2 | 72 | 85 | Carbamidomethylation | C2:Carbamidomethylation:1000.00;C4:Carbamidomethylation:1000.00;C11:Carbamidomethylation:1000.00 | PEAKS DB |
| A.YLKYGC(+57.02)YC(+57.02)GYGGTGTPVDELDR.C | N | 57.42 | 2543.1096 | 22 | 0.4 | 848.7108 | 3 | 42.36 | 1 | 25537 | 01122020\_RID\_2209\_BuCaKA02.raw | 4.2549E7 | 1 | 1 | 4 | 25 | Carbamidomethylation | C6:Carbamidomethylation:1000.00;C8:Carbamidomethylation:1000.00 | PEAKS DB |
| C.DRTAALC(+57.02)FAK.A | N | 55.24 | 1151.5757 | 10 | 2.7 | 576.7967 | 2 | 15.19 | 1 | 4454 | 01122020\_RID\_2209\_BuCaKA02.raw | 3.7655E5 | 1 | 1 | 76 | 85 | Carbamidomethylation | C7:Carbamidomethylation:1000.00 | PEAKS DB |
| F.AKAPYNIGNIMISSSTTC(+57.02)K | Y | 53.57 | 2055.0129 | 19 | 2.3 | 686.0132 | 3 | 36.75 | 1 | 21219 | 01122020\_RID\_2209\_BuCaKA02.raw | 3.0788E6 | 1 | 1 | 84 | 102 | Carbamidomethylation | C18:Carbamidomethylation:1000.00 | PEAKS DB |
| N.IMISSSTTC(+57.02)K | Y | 53.19 | 1126.5363 | 10 | 1.3 | 564.2761 | 2 | 12.11 | 1 | 2078 | 01122020\_RID\_2209\_BuCaKA02.raw | 1.1258E6 | 1 | 1 | 93 | 102 | Carbamidomethylation | C9:Carbamidomethylation:1000.00 | PEAKS DB |
| P.YNIGNIMISSSTTC(+57.02)K | Y | 51.32 | 1687.7909 | 15 | 1.1 | 844.9036 | 2 | 45.46 | 1 | 27804 | 01122020\_RID\_2209\_BuCaKA02.raw | 1.5758E7 | 1 | 1 | 88 | 102 | Carbamidomethylation | C14:Carbamidomethylation:1000.00 | PEAKS DB |
| K.YGC(+57.02)YC(+57.02)GYGGTGTPVDELDRC(+57.02)C(+57.02)YVHDNC(+57.02)YDEAKK.I | N | 50.58 | 3981.5796 | 33 | 0.5 | 996.4027 | 4 | 34.30 | 1 | 19058 | 01122020\_RID\_2209\_BuCaKA02.raw | 1.957E9 | 2 | 2 | 7 | 39 | Carbamidomethylation | C3:Carbamidomethylation:1000.00;C5:Carbamidomethylation:1000.00;C20:Carbamidomethylation:1000.00;C21:Carbamidomethylation:1000.00;C27:Carbamidomethylation:1000.00 | PEAKS DB |
| K.APYNIGNIMIS.S | N | 48.60 | 1191.5958 | 11 | 0.9 | 596.8057 | 2 | 70.10 | 1 | 47633 | 01122020\_RID\_2209\_BuCaKA02.raw | 3.0202E8 | 1 | 1 | 86 | 96 |  |  | PEAKS DB |
| C.DC(+57.02)DRTAALC(+57.02)FAK.A | N | 47.16 | 1426.6333 | 12 | -2.2 | 714.3224 | 2 | 20.22 | 1 | 8374 | 01122020\_RID\_2209\_BuCaKA02.raw | 1.7388E6 | 1 | 1 | 74 | 85 | Carbamidomethylation | C2:Carbamidomethylation:1000.00;C9:Carbamidomethylation:1000.00 | PEAKS DB |
| R.FLC(+57.02)DC(+57.02)DRTAALC(+57.02)F.A | N | 44.26 | 1647.6843 | 13 | 0.5 | 824.8499 | 2 | 55.77 | 1 | 35810 | 01122020\_RID\_2209\_BuCaKA02.raw | 2.2718E7 | 1 | 1 | 71 | 83 | Carbamidomethylation | C3:Carbamidomethylation:1000.00;C5:Carbamidomethylation:1000.00;C12:Carbamidomethylation:1000.00 | PEAKS DB |
| Y.GGTGTPVDELDR.C | N | 44.26 | 1215.5731 | 12 | 0.2 | 608.7939 | 2 | 24.73 | 1 | 11888 | 01122020\_RID\_2209\_BuCaKA02.raw | 2.8666E5 | 1 | 1 | 14 | 25 |  |  | PEAKS DB |
| V.DELDRC(+57.02)C(+57.02)YVHDNC(+57.02)YDEAK.K | N | 41.02 | 2360.9097 | 18 | 2.3 | 591.2360 | 4 | 16.87 | 1 | 5694 | 01122020\_RID\_2209\_BuCaKA02.raw | 3.8429E6 | 1 | 1 | 21 | 38 | Carbamidomethylation | C6:Carbamidomethylation:1000.00;C7:Carbamidomethylation:1000.00;C13:Carbamidomethylation:1000.00 | PEAKS DB |
| L.KYGC(+57.02)YC(+57.02)GYGGTGTPVDELDRC(+57.02)C(+57.02)YVHDNC(+57.02)YDEAK.K | N | 40.59 | 3981.5796 | 33 | 1.7 | 797.3245 | 5 | 33.51 | 1 | 18445 | 01122020\_RID\_2209\_BuCaKA02.raw | 1.0219E8 | 2 | 2 | 6 | 38 | Carbamidomethylation | C4:Carbamidomethylation:1000.00;C6:Carbamidomethylation:1000.00;C21:Carbamidomethylation:1000.00;C22:Carbamidomethylation:1000.00;C28:Carbamidomethylation:1000.00 | PEAKS DB |
| WIAYLKYGC(+57.02)YC(+57.02)G.Y | N | 40.55 | 1552.6843 | 12 | 3.1 | 777.3518 | 2 | 53.53 | 1 | 33953 | 01122020\_RID\_2209\_BuCaKA02.raw | 2.1406E6 | 1 | 1 | 1 | 12 | Carbamidomethylation | C9:Carbamidomethylation:1000.00;C11:Carbamidomethylation:1000.00 | PEAKS DB |
| K.APYNIGNIMISSSTT.C | Y | 40.41 | 1567.7552 | 15 | 0.5 | 784.8853 | 2 | 68.86 | 1 | 46501 | 01122020\_RID\_2209\_BuCaKA02.raw | 4.1472E8 | 1 | 1 | 86 | 100 |  |  | PEAKS DB |
| D.C(+57.02)DRTAALC(+57.02)FAK.A | N | 39.74 | 1311.6063 | 11 | 1.6 | 656.8115 | 2 | 12.51 | 1 | 2398 | 01122020\_RID\_2209\_BuCaKA02.raw | 5.3941E5 | 1 | 1 | 75 | 85 | Carbamidomethylation | C1:Carbamidomethylation:1000.00;C8:Carbamidomethylation:1000.00 | PEAKS DB |
| total 37 peptides |  |  |  |  |  |  |  |  |  |  |  |  |  |  |  |  |  |  |  |
| --- | --- | --- | --- | --- | --- | --- | --- | --- | --- | --- | --- | --- | --- | --- | --- | --- | --- | --- | --- |

T\_111

back to list

  

| Protein Coverage
| Supporting Peptides
|

Protein Coverage:

Supporting Peptides:

| Peptide | Uniq | -10lgP | Mass | Length | ppm | m/z | z | RT | Fraction | Scan | Source File | Area BRom | #Feature | #Feature BRom | Start | End | PTM | AScore | Found By |
| --- | --- | --- | --- | --- | --- | --- | --- | --- | --- | --- | --- | --- | --- | --- | --- | --- | --- | --- | --- |
| R.SIQFAKQLHPELSEAAIKTLAK.Q | Y | 95.83 | 2422.3584 | 22 | 0.9 | 606.5974 | 4 | 39.52 | 1 | 23337 | 01122020\_RID\_2209\_BuCaKA02.raw | 8.5428E7 | 1 | 1 | 173 | 194 |  |  | PEAKS DB |
| R.DSTALFPSIYLEIVLKSSANALKFVHHR.L | Y | 93.89 | 3155.7131 | 28 | 3.7 | 632.1522 | 5 | 85.50 | 1 | 60508 | 01122020\_RID\_2209\_BuCaKA02.raw | 2.672E8 | 5 | 5 | 262 | 289 |  |  | PEAKS DB |
| K.QLHPELSEAAIKTLAK.Q | Y | 91.26 | 1747.9832 | 16 | 0.9 | 583.6688 | 3 | 29.18 | 1 | 15057 | 01122020\_RID\_2209\_BuCaKA02.raw | 2.4989E7 | 1 | 1 | 179 | 194 |  |  | PEAKS DB |
| K.QLHPELSEAAIKTLAKQEYEK.A | Y | 87.02 | 2425.2852 | 21 | 0.6 | 607.3289 | 4 | 33.30 | 1 | 18158 | 01122020\_RID\_2209\_BuCaKA02.raw | 7.4059E7 | 1 | 1 | 179 | 199 |  |  | PEAKS DB |
| R.MIPLKTFHGLGVIDWENWRPQWDR.N | N | 85.69 | 2993.5122 | 24 | 1.0 | 749.3861 | 4 | 69.60 | 1 | 47315 | 01122020\_RID\_2209\_BuCaKA02.raw | 8.6499E8 | 3 | 3 | 138 | 161 |  |  | PEAKS DB |
| K.TFHGLGVIDWENWRPQWDRNWGSK.N | N | 78.24 | 2983.4265 | 24 | 0.5 | 746.8643 | 4 | 65.21 | 1 | 43525 | 01122020\_RID\_2209\_BuCaKA02.raw | 2.0561E8 | 2 | 2 | 143 | 166 |  |  | PEAKS DB |
| K.HSDSNAFLHLFPDSFK.I | Y | 76.83 | 1860.8794 | 16 | 1.4 | 621.3013 | 3 | 54.96 | 1 | 35108 | 01122020\_RID\_2209\_BuCaKA02.raw | 4.1484E6 | 1 | 1 | 398 | 413 |  |  | PEAKS DB |
| K.TFHGLGVIDWENWRPQWDRNWGSKNVYR.N | N | 76.82 | 3515.7024 | 28 | 1.7 | 586.9587 | 6 | 60.26 | 1 | 39703 | 01122020\_RID\_2209\_BuCaKA02.raw | 1.1791E8 | 4 | 4 | 143 | 170 |  |  | PEAKS DB |
| Y.LEIVLKSSANALKFVHHR.L | Y | 76.61 | 2061.1846 | 18 | 1.5 | 688.0698 | 3 | 24.89 | 1 | 11998 | 01122020\_RID\_2209\_BuCaKA02.raw | 1.1977E7 | 1 | 1 | 272 | 289 |  |  | PEAKS DB |
| F.HGLGVIDWENWRPQWDR.N | N | 74.88 | 2163.0398 | 17 | 1.5 | 722.0216 | 3 | 62.72 | 1 | 41513 | 01122020\_RID\_2209\_BuCaKA02.raw | 1.0693E7 | 1 | 1 | 145 | 161 |  |  | PEAKS DB |
| M.IPLKTFHGLGVIDWENWRPQWDR.N | N | 74.70 | 2862.4717 | 23 | 2.4 | 716.6269 | 4 | 65.54 | 1 | 43835 | 01122020\_RID\_2209\_BuCaKA02.raw | 3.7443E7 | 2 | 2 | 139 | 161 |  |  | PEAKS DB |
| T.FHGLGVIDWENWRPQWDRNWGSKNVYR.N | N | 73.07 | 3414.6548 | 27 | 2.4 | 683.9399 | 5 | 59.74 | 1 | 39259 | 01122020\_RID\_2209\_BuCaKA02.raw | 1.1516E7 | 1 | 1 | 144 | 170 |  |  | PEAKS DB |
| T.FHGLGVIDWENWRPQWDRNWGSK.N | N | 72.71 | 2882.3789 | 23 | 1.0 | 721.6027 | 4 | 64.66 | 1 | 43174 | 01122020\_RID\_2209\_BuCaKA02.raw | 0 | 0 | 0 | 144 | 166 |  |  | PEAKS DB |
| F.HGLGVIDWENWRPQWDRNWGSKNVYR.N | N | 72.19 | 3267.5862 | 26 | 1.2 | 654.5253 | 5 | 55.12 | 1 | 35225 | 01122020\_RID\_2209\_BuCaKA02.raw | 3.0043E7 | 1 | 1 | 145 | 170 |  |  | PEAKS DB |
| K.TFHGLGVIDWENWRPQWDR.N | N | 70.71 | 2411.1560 | 19 | 0.4 | 603.7965 | 4 | 68.13 | 1 | 45893 | 01122020\_RID\_2209\_BuCaKA02.raw | 5.4873E7 | 2 | 2 | 143 | 161 |  |  | PEAKS DB |
| R.DSTALFPSIYLEIVLKSSANALK.F | Y | 69.71 | 2479.3574 | 23 | 2.2 | 827.4615 | 3 | 100.06 | 1 | 72446 | 01122020\_RID\_2209\_BuCaKA02.raw | 3.7541E6 | 1 | 1 | 262 | 284 |  |  | PEAKS DB |
| R.M(+15.99)IPLKTFHGLGVIDWENWRPQWDR.N | N | 68.57 | 3009.5071 | 24 | 0.5 | 753.3844 | 4 | 66.33 | 1 | 44566 | 01122020\_RID\_2209\_BuCaKA02.raw | 1.1028E7 | 1 | 1 | 138 | 161 | Oxidation (M) | M1:Oxidation (M):1000.00 | PEAKS DB |
| F.HGLGVIDWENWRPQWDRNWGSK.N | N | 65.22 | 2735.3105 | 22 | 1.7 | 684.8361 | 4 | 60.91 | 1 | 39964 | 01122020\_RID\_2209\_BuCaKA02.raw | 7.3967E7 | 1 | 1 | 145 | 166 |  |  | PEAKS DB |
| P.SIEISRNDQLLWLWR.D | Y | 63.12 | 1928.0269 | 15 | 1.8 | 643.6841 | 3 | 69.84 | 1 | 47430 | 01122020\_RID\_2209\_BuCaKA02.raw | 7.9751E6 | 1 | 1 | 247 | 261 |  |  | PEAKS DB |
| K.APMYPNEPFLVFWNAPTTQC(+57.02)QMR.Y | Y | 62.84 | 2797.2815 | 23 | -0.7 | 933.4338 | 3 | 85.49 | 1 | 60433 | 01122020\_RID\_2209\_BuCaKA02.raw | 5.5217E6 | 1 | 1 | 46 | 68 | Carbamidomethylation | C20:Carbamidomethylation:1000.00 | PEAKS DB |
| R.NDQLLWLWR.D | N | 61.56 | 1242.6509 | 9 | 1.8 | 622.3339 | 2 | 76.01 | 1 | 52505 | 01122020\_RID\_2209\_BuCaKA02.raw | 1.468E7 | 1 | 1 | 253 | 261 |  |  | PEAKS DB |
| R.DTLLLAENMRPDGYWGYYLYPDC(+57.02)YNYNYK.K | N | 61.22 | 3669.6221 | 29 | 1.5 | 1224.2164 | 3 | 86.29 | 1 | 61045 | 01122020\_RID\_2209\_BuCaKA02.raw | 4.3071E7 | 2 | 2 | 207 | 235 | Carbamidomethylation | C23:Carbamidomethylation:1000.00 | PEAKS DB |
| K.C(+57.02)PSIEISRNDQLLWLWR.D | Y | 60.26 | 2185.1101 | 17 | 0.8 | 729.3779 | 3 | 71.20 | 1 | 48584 | 01122020\_RID\_2209\_BuCaKA02.raw | 7.1936E6 | 1 | 1 | 245 | 261 | Carbamidomethylation | C1:Carbamidomethylation:1000.00 | PEAKS DB |
| R.VASLARQDYALPVFVYARPFYA.Y | Y | 60.21 | 2516.3215 | 22 | 2.3 | 839.7831 | 3 | 75.70 | 1 | 52457 | 01122020\_RID\_2209\_BuCaKA02.raw | 2.2803E7 | 1 | 1 | 296 | 317 |  |  | PEAKS DB |
| R.YKVDLDLK.T | N | 55.02 | 992.5542 | 8 | 1.5 | 497.2851 | 2 | 23.86 | 1 | 11231 | 01122020\_RID\_2209\_BuCaKA02.raw | 0 | 0 | 0 | 69 | 76 |  |  | PEAKS DB |
| R.M(+15.99)IPLKTFHGLGVIDWENWRPQWDRNWGSK.N | N | 55.02 | 3581.7778 | 29 | 1.1 | 717.3636 | 5 | 64.61 | 1 | 43066 | 01122020\_RID\_2209\_BuCaKA02.raw | 2.3034E7 | 1 | 1 | 138 | 166 | Oxidation (M) | M1:Oxidation (M):1000.00 | PEAKS DB |
| R.DSTALFPSIYLEIVLK.S | Y | 54.49 | 1807.9971 | 16 | 1.1 | 905.0068 | 2 | 106.33 | 1 | 77525 | 01122020\_RID\_2209\_BuCaKA02.raw | 4.1484E6 | 1 | 1 | 262 | 277 |  |  | PEAKS DB |
| M.IPLKTFHGLGVIDWENWRPQWDRNWGSK.N | N | 52.42 | 3434.7424 | 28 | 0.1 | 687.9558 | 5 | 63.88 | 1 | 42447 | 01122020\_RID\_2209\_BuCaKA02.raw | 3.5454E7 | 1 | 1 | 139 | 166 |  |  | PEAKS DB |
| K.KPEQYTGKC(+57.02)PSIEISRNDQLLWLWR.D | Y | 50.64 | 3116.5864 | 25 | 0.2 | 624.3247 | 5 | 52.91 | 1 | 33463 | 01122020\_RID\_2209\_BuCaKA02.raw | 0 | 0 | 0 | 237 | 261 | Carbamidomethylation | C9:Carbamidomethylation:1000.00 | PEAKS DB |
| R.MIPLKTFHGLGVIDWENWRPQWDRNWGSK.N | N | 50.45 | 3565.7830 | 29 | 1.2 | 714.1647 | 5 | 67.46 | 1 | 45659 | 01122020\_RID\_2209\_BuCaKA02.raw | 8.4748E8 | 1 | 1 | 138 | 166 |  |  | PEAKS DB |
| G.VIDWENWRPQWDRNWGSK.N | N | 49.40 | 2371.1245 | 18 | 0.7 | 593.7888 | 4 | 57.43 | 1 | 37336 | 01122020\_RID\_2209\_BuCaKA02.raw | 2.1347E6 | 1 | 1 | 149 | 166 |  |  | PEAKS DB |
| R.DSTALFPSIYL.E | N | 44.29 | 1225.6230 | 11 | 1.1 | 613.8195 | 2 | 107.65 | 1 | 78808 | 01122020\_RID\_2209\_BuCaKA02.raw | 3.2385E7 | 1 | 1 | 262 | 272 |  |  | PEAKS DB |
| K.APMYPNEPFLVF.W | N | 42.07 | 1423.6846 | 12 | 0.5 | 712.8499 | 2 | 95.66 | 1 | 68548 | 01122020\_RID\_2209\_BuCaKA02.raw | 1.165E7 | 1 | 1 | 46 | 57 |  |  | PEAKS DB |
| N.DQLLWLWR.D | N | 40.81 | 1128.6080 | 8 | -0.1 | 565.3112 | 2 | 82.89 | 1 | 58160 | 01122020\_RID\_2209\_BuCaKA02.raw | 2.5117E6 | 1 | 1 | 254 | 261 |  |  | PEAKS DB |
| total 34 peptides |  |  |  |  |  |  |  |  |  |  |  |  |  |  |  |  |  |  |  |
| --- | --- | --- | --- | --- | --- | --- | --- | --- | --- | --- | --- | --- | --- | --- | --- | --- | --- | --- | --- |

T\_113

back to list

  

| Protein Coverage
| Supporting Peptides
|

Protein Coverage:

Supporting Peptides:

| Peptide | Uniq | -10lgP | Mass | Length | ppm | m/z | z | RT | Fraction | Scan | Source File | Area BRom | #Feature | #Feature BRom | Start | End | PTM | AScore | Found By |
| --- | --- | --- | --- | --- | --- | --- | --- | --- | --- | --- | --- | --- | --- | --- | --- | --- | --- | --- | --- |
| L.KYGC(+57.02)YC(+57.02)GYGGTGTPVDELDR.C | N | 88.44 | 2266.9624 | 20 | -2.4 | 1134.4857 | 2 | 32.64 | 1 | 17782 | 01122020\_RID\_2209\_BuCaKA02.raw | 3.5219E7 | 2 | 2 | 6 | 25 | Carbamidomethylation | C4:Carbamidomethylation:1000.00;C6:Carbamidomethylation:1000.00 | PEAKS DB |
| K.YGC(+57.02)YC(+57.02)GYGGTGTPVDELDR.C | N | 88.01 | 2138.8674 | 19 | 0.4 | 1070.4414 | 2 | 44.33 | 1 | 26645 | 01122020\_RID\_2209\_BuCaKA02.raw | 9.6633E9 | 3 | 3 | 7 | 25 | Carbamidomethylation | C3:Carbamidomethylation:1000.00;C5:Carbamidomethylation:1000.00 | PEAKS DB |
| Y.GC(+57.02)YC(+57.02)GYGGTGTPVDELDR.C | N | 85.01 | 1975.8040 | 18 | 0.4 | 988.9097 | 2 | 38.86 | 1 | 23078 | 01122020\_RID\_2209\_BuCaKA02.raw | 3.3628E9 | 2 | 2 | 8 | 25 | Carbamidomethylation | C2:Carbamidomethylation:1000.00;C4:Carbamidomethylation:1000.00 | PEAKS DB |
| Y.C(+57.02)GYGGTGTPVDELDR.C | N | 82.21 | 1595.6886 | 15 | 1.6 | 798.8528 | 2 | 33.04 | 1 | 18122 | 01122020\_RID\_2209\_BuCaKA02.raw | 5.3035E8 | 3 | 3 | 11 | 25 | Carbamidomethylation | C1:Carbamidomethylation:1000.00 | PEAKS DB |
| C.YC(+57.02)GYGGTGTPVDELDR.C | N | 81.92 | 1758.7518 | 16 | 0.3 | 880.3834 | 2 | 37.99 | 1 | 22078 | 01122020\_RID\_2209\_BuCaKA02.raw | 4.9681E7 | 1 | 1 | 10 | 25 | Carbamidomethylation | C2:Carbamidomethylation:1000.00 | PEAKS DB |
| R.FLC(+57.02)DC(+57.02)DRTAALC(+57.02)FAK.A | N | 80.82 | 1846.8164 | 15 | -0.1 | 924.4154 | 2 | 36.40 | 1 | 20993 | 01122020\_RID\_2209\_BuCaKA02.raw | 1.3034E10 | 13 | 13 | 71 | 85 | Carbamidomethylation | C3:Carbamidomethylation:1000.00;C5:Carbamidomethylation:1000.00;C12:Carbamidomethylation:1000.00 | PEAKS DB |
| R.C(+57.02)C(+57.02)YVHDNC(+57.02)YDEAKKIHGC(+57.02)NPTTK.T | N | 80.25 | 2869.2041 | 23 | 1.4 | 574.8489 | 5 | 12.05 | 1 | 2027 | 01122020\_RID\_2209\_BuCaKA02.raw | 0 | 0 | 0 | 26 | 48 | Carbamidomethylation | C1:Carbamidomethylation:1000.00;C2:Carbamidomethylation:1000.00;C8:Carbamidomethylation:1000.00;C18:Carbamidomethylation:1000.00 | PEAKS DB |
| G.C(+57.02)YC(+57.02)GYGGTGTPVDELDR.C | N | 77.93 | 1918.7826 | 17 | 1.1 | 960.3996 | 2 | 38.92 | 1 | 22863 | 01122020\_RID\_2209\_BuCaKA02.raw | 7.0014E7 | 2 | 2 | 9 | 25 | Carbamidomethylation | C1:Carbamidomethylation:1000.00;C3:Carbamidomethylation:1000.00 | PEAKS DB |
| K.C(+57.02)ARFLC(+57.02)DC(+57.02)DRTAALC(+57.02)FAK.A | N | 75.35 | 2233.9854 | 18 | 2.1 | 559.5048 | 4 | 29.81 | 1 | 15728 | 01122020\_RID\_2209\_BuCaKA02.raw | 1.7382E7 | 1 | 1 | 68 | 85 | Carbamidomethylation | C1:Carbamidomethylation:1000.00;C6:Carbamidomethylation:1000.00;C8:Carbamidomethylation:1000.00;C15:Carbamidomethylation:1000.00 | PEAKS DB |
| K.APYNIANIMISASTTC(+57.02)Q | Y | 71.09 | 1853.8651 | 17 | 0.2 | 927.9401 | 2 | 90.37 | 1 | 64665 | 01122020\_RID\_2209\_BuCaKA02.raw | 5.2022E10 | 56 | 56 | 86 | 102 | Carbamidomethylation | C16:Carbamidomethylation:1000.00 | PEAKS DB |
| Y.NIANIMISASTTC(+57.02)Q | Y | 70.20 | 1522.7120 | 14 | 0.1 | 762.3634 | 2 | 65.19 | 1 | 45027 | 01122020\_RID\_2209\_BuCaKA02.raw | 1.8917E9 | 20 | 20 | 89 | 102 | Carbamidomethylation | C13:Carbamidomethylation:1000.00 | PEAKS DB |
| C.GYGGTGTPVDELDR.C | N | 70.03 | 1435.6580 | 14 | 1.4 | 718.8373 | 2 | 31.57 | 1 | 17094 | 01122020\_RID\_2209\_BuCaKA02.raw | 9.8214E7 | 1 | 1 | 12 | 25 |  |  | PEAKS DB |
| Y.LKYGC(+57.02)YC(+57.02)GYGGTGTPVDELDR.C | N | 69.19 | 2380.0464 | 21 | 0.9 | 794.3568 | 3 | 38.32 | 1 | 22508 | 01122020\_RID\_2209\_BuCaKA02.raw | 1.3586E8 | 1 | 1 | 5 | 25 | Carbamidomethylation | C5:Carbamidomethylation:1000.00;C7:Carbamidomethylation:1000.00 | PEAKS DB |
| G.YGGTGTPVDELDR.C | N | 68.14 | 1378.6365 | 13 | 1.7 | 690.3267 | 2 | 30.08 | 1 | 15917 | 01122020\_RID\_2209\_BuCaKA02.raw | 1.3749E8 | 1 | 1 | 13 | 25 |  |  | PEAKS DB |
| R.TAALC(+57.02)FAKAPYNIANIMISASTTC(+57.02)Q | Y | 67.03 | 2716.3022 | 25 | 1.9 | 1359.1610 | 2 | 81.59 | 1 | 57212 | 01122020\_RID\_2209\_BuCaKA02.raw | 2.7288E9 | 2 | 2 | 78 | 102 | Carbamidomethylation | C5:Carbamidomethylation:1000.00;C24:Carbamidomethylation:1000.00 | PEAKS DB |
| P.YNIANIMISASTTC(+57.02)Q | Y | 66.30 | 1685.7753 | 15 | 0.3 | 843.8952 | 2 | 76.73 | 1 | 53487 | 01122020\_RID\_2209\_BuCaKA02.raw | 2.8365E9 | 16 | 16 | 88 | 102 | Carbamidomethylation | C14:Carbamidomethylation:1000.00 | PEAKS DB |
| K.YGC(+57.02)YC(+57.02)GYGGTGTPVDELDRC(+57.02)C(+57.02)YVHDNC(+57.02)YDEAK.K | N | 66.01 | 3853.4846 | 32 | 0.6 | 1285.5029 | 3 | 41.35 | 1 | 24801 | 01122020\_RID\_2209\_BuCaKA02.raw | 3.1534E9 | 2 | 2 | 7 | 38 | Carbamidomethylation | C3:Carbamidomethylation:1000.00;C5:Carbamidomethylation:1000.00;C20:Carbamidomethylation:1000.00;C21:Carbamidomethylation:1000.00;C27:Carbamidomethylation:1000.00 | PEAKS DB |
| K.YGC(+57.02)YC(+57.02)GYGGTGTPVDELDRC(+57.02)C(+57.02).Y | N | 63.90 | 2458.9287 | 21 | 1.8 | 1230.4739 | 2 | 48.28 | 1 | 29764 | 01122020\_RID\_2209\_BuCaKA02.raw | 8.9471E6 | 1 | 1 | 7 | 27 | Carbamidomethylation | C3:Carbamidomethylation:1000.00;C5:Carbamidomethylation:1000.00;C20:Carbamidomethylation:1000.00;C21:Carbamidomethylation:1000.00 | PEAKS DB |
| R.TAALC(+57.02)FAKAPYNI.A | N | 62.47 | 1438.7278 | 13 | 3.0 | 720.3733 | 2 | 50.01 | 1 | 31149 | 01122020\_RID\_2209\_BuCaKA02.raw | 0 | 0 | 0 | 78 | 90 | Carbamidomethylation | C5:Carbamidomethylation:1000.00 | PEAKS DB |
| K.APYNIANIM(+15.99)ISASTTC(+57.02)Q | Y | 61.76 | 1869.8601 | 17 | 1.2 | 935.9385 | 2 | 68.64 | 1 | 46719 | 01122020\_RID\_2209\_BuCaKA02.raw | 3.2947E9 | 22 | 22 | 86 | 102 | Oxidation (M); Carbamidomethylation | M9:Oxidation (M):1000.00;C16:Carbamidomethylation:1000.00 | PEAKS DB |
| F.LC(+57.02)DC(+57.02)DRTAALC(+57.02)FAK.A | N | 61.05 | 1699.7480 | 14 | 2.4 | 567.5913 | 3 | 22.40 | 1 | 9949 | 01122020\_RID\_2209\_BuCaKA02.raw | 3.0159E7 | 2 | 2 | 72 | 85 | Carbamidomethylation | C2:Carbamidomethylation:1000.00;C4:Carbamidomethylation:1000.00;C11:Carbamidomethylation:1000.00 | PEAKS DB |
| K.APYNIANIMISAST.T | Y | 60.57 | 1464.7283 | 14 | 0.9 | 733.3721 | 2 | 80.16 | 1 | 55918 | 01122020\_RID\_2209\_BuCaKA02.raw | 8.0527E7 | 1 | 1 | 86 | 99 |  |  | PEAKS DB |
| K.APYNIANIMISASTT.C | Y | 59.17 | 1565.7759 | 15 | 3.2 | 783.8977 | 2 | 83.51 | 1 | 58681 | 01122020\_RID\_2209\_BuCaKA02.raw | 2.8199E10 | 25 | 25 | 86 | 100 |  |  | PEAKS DB |
| K.APYNIANIMISAS.T | Y | 58.01 | 1363.6805 | 13 | 0.7 | 682.8480 | 2 | 78.43 | 1 | 55075 | 01122020\_RID\_2209\_BuCaKA02.raw | 9.8289E9 | 25 | 25 | 86 | 98 |  |  | PEAKS DB |
| A.YLKYGC(+57.02)YC(+57.02)GYGGTGTPVDELDR.C | N | 57.42 | 2543.1096 | 22 | 0.4 | 848.7108 | 3 | 42.36 | 1 | 25537 | 01122020\_RID\_2209\_BuCaKA02.raw | 4.2549E7 | 1 | 1 | 4 | 25 | Carbamidomethylation | C6:Carbamidomethylation:1000.00;C8:Carbamidomethylation:1000.00 | PEAKS DB |
| K.APYNIANIM(+15.99)ISASTT.C | Y | 55.83 | 1581.7709 | 15 | 0.1 | 791.8928 | 2 | 64.90 | 1 | 43275 | 01122020\_RID\_2209\_BuCaKA02.raw | 1.7458E9 | 6 | 6 | 86 | 100 | Oxidation (M) | M9:Oxidation (M):1000.00 | PEAKS DB |
| C.DRTAALC(+57.02)FAK.A | N | 55.24 | 1151.5757 | 10 | 2.7 | 576.7967 | 2 | 15.19 | 1 | 4454 | 01122020\_RID\_2209\_BuCaKA02.raw | 3.7655E5 | 1 | 1 | 76 | 85 | Carbamidomethylation | C7:Carbamidomethylation:1000.00 | PEAKS DB |
| N.IMISASTTC(+57.02)Q | Y | 53.71 | 1110.5050 | 10 | 2.0 | 556.2609 | 2 | 35.69 | 1 | 18491 | 01122020\_RID\_2209\_BuCaKA02.raw | 5.185E8 | 2 | 2 | 93 | 102 | Carbamidomethylation | C9:Carbamidomethylation:1000.00 | PEAKS DB |
| K.APYNIANIMIS.A | Y | 52.39 | 1205.6115 | 11 | 1.6 | 603.8140 | 2 | 74.88 | 1 | 51398 | 01122020\_RID\_2209\_BuCaKA02.raw | 1.5322E9 | 1 | 1 | 86 | 96 |  |  | PEAKS DB |
| N.IM(+15.99)ISASTTC(+57.02)Q | Y | 51.10 | 1126.4999 | 10 | 1.5 | 564.2581 | 2 | 33.67 | 1 | 19510 | 01122020\_RID\_2209\_BuCaKA02.raw | 4.0201E7 | 1 | 1 | 93 | 102 | Oxidation (M); Carbamidomethylation | M2:Oxidation (M):1000.00;C9:Carbamidomethylation:1000.00 | PEAKS DB |
| K.APYNIANIM(+15.99)ISAS.T | Y | 50.91 | 1379.6755 | 13 | 0.1 | 690.8451 | 2 | 63.85 | 1 | 42803 | 01122020\_RID\_2209\_BuCaKA02.raw | 3.1297E8 | 3 | 3 | 86 | 98 | Oxidation (M) | M9:Oxidation (M):1000.00 | PEAKS DB |
| K.YGC(+57.02)YC(+57.02)GYGGTGTPVDELDRC(+57.02)C(+57.02)YVHDNC(+57.02)YDEAKK.I | N | 50.58 | 3981.5796 | 33 | 0.5 | 996.4027 | 4 | 34.30 | 1 | 19058 | 01122020\_RID\_2209\_BuCaKA02.raw | 1.957E9 | 2 | 2 | 7 | 39 | Carbamidomethylation | C3:Carbamidomethylation:1000.00;C5:Carbamidomethylation:1000.00;C20:Carbamidomethylation:1000.00;C21:Carbamidomethylation:1000.00;C27:Carbamidomethylation:1000.00 | PEAKS DB |
| K.APYNIANIMISA.S | Y | 48.93 | 1276.6486 | 12 | 0.6 | 639.3319 | 2 | 79.20 | 1 | 55126 | 01122020\_RID\_2209\_BuCaKA02.raw | 1.3383E9 | 1 | 1 | 86 | 97 |  |  | PEAKS DB |
| R.TAALC(+57.02)FAKAPYNIANIMISASTT.C | Y | 47.57 | 2428.2131 | 23 | -0.7 | 1215.1130 | 2 | 82.32 | 1 | 58215 | 01122020\_RID\_2209\_BuCaKA02.raw | 1.3783E9 | 1 | 1 | 78 | 100 | Carbamidomethylation | C5:Carbamidomethylation:1000.00 | PEAKS DB |
| R.TAALC(+57.02)FAKAPYNIAN.I | Y | 47.27 | 1623.8079 | 15 | 1.2 | 812.9122 | 2 | 46.24 | 1 | 28443 | 01122020\_RID\_2209\_BuCaKA02.raw | 0 | 0 | 0 | 78 | 92 | Carbamidomethylation | C5:Carbamidomethylation:1000.00 | PEAKS DB |
| C.DC(+57.02)DRTAALC(+57.02)FAK.A | N | 47.16 | 1426.6333 | 12 | -2.2 | 714.3224 | 2 | 20.22 | 1 | 8374 | 01122020\_RID\_2209\_BuCaKA02.raw | 1.7388E6 | 1 | 1 | 74 | 85 | Carbamidomethylation | C2:Carbamidomethylation:1000.00;C9:Carbamidomethylation:1000.00 | PEAKS DB |
| R.TAALC(+57.02)FAKAPYNIANIMIS.A | Y | 45.99 | 2068.0486 | 19 | 0.2 | 1035.0317 | 2 | 76.57 | 1 | 52991 | 01122020\_RID\_2209\_BuCaKA02.raw | 8.6302E7 | 1 | 1 | 78 | 96 | Carbamidomethylation | C5:Carbamidomethylation:1000.00 | PEAKS DB |
| R.TAALC(+57.02)FAKAPYNIANIMISA.S | Y | 45.88 | 2139.0857 | 20 | -0.1 | 1070.5500 | 2 | 80.07 | 1 | 55915 | 01122020\_RID\_2209\_BuCaKA02.raw | 1.3369E8 | 1 | 1 | 78 | 97 | Carbamidomethylation | C5:Carbamidomethylation:1000.00 | PEAKS DB |
| R.FLC(+57.02)DC(+57.02)DRTAALC(+57.02)F.A | N | 44.26 | 1647.6843 | 13 | 0.5 | 824.8499 | 2 | 55.77 | 1 | 35810 | 01122020\_RID\_2209\_BuCaKA02.raw | 2.2718E7 | 1 | 1 | 71 | 83 | Carbamidomethylation | C3:Carbamidomethylation:1000.00;C5:Carbamidomethylation:1000.00;C12:Carbamidomethylation:1000.00 | PEAKS DB |
| Y.GGTGTPVDELDR.C | N | 44.26 | 1215.5731 | 12 | 0.2 | 608.7939 | 2 | 24.73 | 1 | 11888 | 01122020\_RID\_2209\_BuCaKA02.raw | 2.8666E5 | 1 | 1 | 14 | 25 |  |  | PEAKS DB |
| K.APYNIANIM(+15.99)IS.A | Y | 43.06 | 1221.6063 | 11 | 0.4 | 611.8107 | 2 | 63.18 | 1 | 41870 | 01122020\_RID\_2209\_BuCaKA02.raw | 7.4486E7 | 1 | 1 | 86 | 96 | Oxidation (M) | M9:Oxidation (M):1000.00 | PEAKS DB |
| V.DELDRC(+57.02)C(+57.02)YVHDNC(+57.02)YDEAK.K | N | 41.02 | 2360.9097 | 18 | 2.3 | 591.2360 | 4 | 16.87 | 1 | 5694 | 01122020\_RID\_2209\_BuCaKA02.raw | 3.8429E6 | 1 | 1 | 21 | 38 | Carbamidomethylation | C6:Carbamidomethylation:1000.00;C7:Carbamidomethylation:1000.00;C13:Carbamidomethylation:1000.00 | PEAKS DB |
| P.YNIANIM(+15.99)ISASTTC(+57.02)Q | Y | 40.64 | 1701.7703 | 15 | 3.6 | 851.8954 | 2 | 78.39 | 1 | 54570 | 01122020\_RID\_2209\_BuCaKA02.raw | 0 | 0 | 0 | 88 | 102 | Oxidation (M); Carbamidomethylation | M7:Oxidation (M):1000.00;C14:Carbamidomethylation:1000.00 | PEAKS DB |
| L.KYGC(+57.02)YC(+57.02)GYGGTGTPVDELDRC(+57.02)C(+57.02)YVHDNC(+57.02)YDEAK.K | N | 40.59 | 3981.5796 | 33 | 1.7 | 797.3245 | 5 | 33.51 | 1 | 18445 | 01122020\_RID\_2209\_BuCaKA02.raw | 1.0219E8 | 2 | 2 | 6 | 38 | Carbamidomethylation | C4:Carbamidomethylation:1000.00;C6:Carbamidomethylation:1000.00;C21:Carbamidomethylation:1000.00;C22:Carbamidomethylation:1000.00;C28:Carbamidomethylation:1000.00 | PEAKS DB |
| WIAYLKYGC(+57.02)YC(+57.02)G.Y | N | 40.55 | 1552.6843 | 12 | 3.1 | 777.3518 | 2 | 53.53 | 1 | 33953 | 01122020\_RID\_2209\_BuCaKA02.raw | 2.1406E6 | 1 | 1 | 1 | 12 | Carbamidomethylation | C9:Carbamidomethylation:1000.00;C11:Carbamidomethylation:1000.00 | PEAKS DB |
| K.APYNIANIM(+15.99)I.S | Y | 40.31 | 1134.5743 | 10 | 3.4 | 568.2964 | 2 | 70.39 | 1 | 47785 | 01122020\_RID\_2209\_BuCaKA02.raw | 3.8935E6 | 1 | 1 | 86 | 95 | Oxidation (M) | M9:Oxidation (M):1000.00 | PEAKS DB |
| K.APYNIANIM(+15.99)ISA.S | Y | 40.13 | 1292.6434 | 12 | 0.7 | 647.3295 | 2 | 66.34 | 1 | 44699 | 01122020\_RID\_2209\_BuCaKA02.raw | 5.0826E7 | 1 | 1 | 86 | 97 | Oxidation (M) | M9:Oxidation (M):1000.00 | PEAKS DB |
| D.C(+57.02)DRTAALC(+57.02)FAK.A | N | 39.74 | 1311.6063 | 11 | 1.6 | 656.8115 | 2 | 12.51 | 1 | 2398 | 01122020\_RID\_2209\_BuCaKA02.raw | 5.3941E5 | 1 | 1 | 75 | 85 | Carbamidomethylation | C1:Carbamidomethylation:1000.00;C8:Carbamidomethylation:1000.00 | PEAKS DB |
| total 48 peptides |  |  |  |  |  |  |  |  |  |  |  |  |  |  |  |  |  |  |  |
| --- | --- | --- | --- | --- | --- | --- | --- | --- | --- | --- | --- | --- | --- | --- | --- | --- | --- | --- | --- |

T\_115

back to list

  

| Protein Coverage
| Supporting Peptides
|

Protein Coverage:

Supporting Peptides:

| Peptide | Uniq | -10lgP | Mass | Length | ppm | m/z | z | RT | Fraction | Scan | Source File | Area BRom | #Feature | #Feature BRom | Start | End | PTM | AScore | Found By |
| --- | --- | --- | --- | --- | --- | --- | --- | --- | --- | --- | --- | --- | --- | --- | --- | --- | --- | --- | --- |
| R.C(+57.02)C(+57.02)YVHDNC(+57.02)YGEAEKIPDC(+57.02)NPK.T | Y | 90.47 | 2628.0500 | 21 | 0.9 | 877.0247 | 3 | 16.44 | 1 | 5430 | 01122020\_RID\_2209\_BuCaKA02.raw | 3.3956E8 | 3 | 3 | 26 | 46 | Carbamidomethylation | C1:Carbamidomethylation:1000.00;C2:Carbamidomethylation:1000.00;C8:Carbamidomethylation:1000.00;C18:Carbamidomethylation:1000.00 | PEAKS DB |
| L.KYGC(+57.02)YC(+57.02)GYGGTGTPVDELDR.C | N | 88.44 | 2266.9624 | 20 | -2.4 | 1134.4857 | 2 | 32.64 | 1 | 17782 | 01122020\_RID\_2209\_BuCaKA02.raw | 3.5219E7 | 2 | 2 | 6 | 25 | Carbamidomethylation | C4:Carbamidomethylation:1000.00;C6:Carbamidomethylation:1000.00 | PEAKS DB |
| K.YGC(+57.02)YC(+57.02)GYGGTGTPVDELDR.C | N | 88.01 | 2138.8674 | 19 | 0.4 | 1070.4414 | 2 | 44.33 | 1 | 26645 | 01122020\_RID\_2209\_BuCaKA02.raw | 9.6633E9 | 3 | 3 | 7 | 25 | Carbamidomethylation | C3:Carbamidomethylation:1000.00;C5:Carbamidomethylation:1000.00 | PEAKS DB |
| Y.GC(+57.02)YC(+57.02)GYGGTGTPVDELDR.C | N | 85.01 | 1975.8040 | 18 | 0.4 | 988.9097 | 2 | 38.86 | 1 | 23078 | 01122020\_RID\_2209\_BuCaKA02.raw | 3.3628E9 | 2 | 2 | 8 | 25 | Carbamidomethylation | C2:Carbamidomethylation:1000.00;C4:Carbamidomethylation:1000.00 | PEAKS DB |
| Y.C(+57.02)GYGGTGTPVDELDR.C | N | 82.21 | 1595.6886 | 15 | 1.6 | 798.8528 | 2 | 33.04 | 1 | 18122 | 01122020\_RID\_2209\_BuCaKA02.raw | 5.3035E8 | 3 | 3 | 11 | 25 | Carbamidomethylation | C1:Carbamidomethylation:1000.00 | PEAKS DB |
| C.YC(+57.02)GYGGTGTPVDELDR.C | N | 81.92 | 1758.7518 | 16 | 0.3 | 880.3834 | 2 | 37.99 | 1 | 22078 | 01122020\_RID\_2209\_BuCaKA02.raw | 4.9681E7 | 1 | 1 | 10 | 25 | Carbamidomethylation | C2:Carbamidomethylation:1000.00 | PEAKS DB |
| R.FLC(+57.02)DC(+57.02)DRTAALC(+57.02)FAK.A | N | 80.82 | 1846.8164 | 15 | -0.1 | 924.4154 | 2 | 36.40 | 1 | 20993 | 01122020\_RID\_2209\_BuCaKA02.raw | 1.3034E10 | 13 | 13 | 71 | 85 | Carbamidomethylation | C3:Carbamidomethylation:1000.00;C5:Carbamidomethylation:1000.00;C12:Carbamidomethylation:1000.00 | PEAKS DB |
| G.C(+57.02)YC(+57.02)GYGGTGTPVDELDR.C | N | 77.93 | 1918.7826 | 17 | 1.1 | 960.3996 | 2 | 38.92 | 1 | 22863 | 01122020\_RID\_2209\_BuCaKA02.raw | 7.0014E7 | 2 | 2 | 9 | 25 | Carbamidomethylation | C1:Carbamidomethylation:1000.00;C3:Carbamidomethylation:1000.00 | PEAKS DB |
| K.C(+57.02)ARFLC(+57.02)DC(+57.02)DRTAALC(+57.02)FAK.A | N | 75.35 | 2233.9854 | 18 | 2.1 | 559.5048 | 4 | 29.81 | 1 | 15728 | 01122020\_RID\_2209\_BuCaKA02.raw | 1.7382E7 | 1 | 1 | 68 | 85 | Carbamidomethylation | C1:Carbamidomethylation:1000.00;C6:Carbamidomethylation:1000.00;C8:Carbamidomethylation:1000.00;C15:Carbamidomethylation:1000.00 | PEAKS DB |
| C.GYGGTGTPVDELDR.C | N | 70.03 | 1435.6580 | 14 | 1.4 | 718.8373 | 2 | 31.57 | 1 | 17094 | 01122020\_RID\_2209\_BuCaKA02.raw | 9.8214E7 | 1 | 1 | 12 | 25 |  |  | PEAKS DB |
| K.APYNIGNIMISASSAC(+57.02)Q | Y | 69.32 | 1795.8232 | 17 | 0.5 | 898.9193 | 2 | 71.54 | 1 | 48744 | 01122020\_RID\_2209\_BuCaKA02.raw | 1.1272E10 | 12 | 12 | 86 | 102 | Carbamidomethylation | C16:Carbamidomethylation:1000.00 | PEAKS DB |
| Y.LKYGC(+57.02)YC(+57.02)GYGGTGTPVDELDR.C | N | 69.19 | 2380.0464 | 21 | 0.9 | 794.3568 | 3 | 38.32 | 1 | 22508 | 01122020\_RID\_2209\_BuCaKA02.raw | 1.3586E8 | 1 | 1 | 5 | 25 | Carbamidomethylation | C5:Carbamidomethylation:1000.00;C7:Carbamidomethylation:1000.00 | PEAKS DB |
| G.YGGTGTPVDELDR.C | N | 68.14 | 1378.6365 | 13 | 1.7 | 690.3267 | 2 | 30.08 | 1 | 15917 | 01122020\_RID\_2209\_BuCaKA02.raw | 1.3749E8 | 1 | 1 | 13 | 25 |  |  | PEAKS DB |
| R.TAALC(+57.02)FAKAPYNIGNIMISASSAC(+57.02)Q | Y | 65.01 | 2658.2605 | 25 | 0.8 | 887.0948 | 3 | 74.14 | 1 | 51081 | 01122020\_RID\_2209\_BuCaKA02.raw | 7.4105E8 | 2 | 2 | 78 | 102 | Carbamidomethylation | C5:Carbamidomethylation:1000.00;C24:Carbamidomethylation:1000.00 | PEAKS DB |
| R.C(+57.02)C(+57.02)YVHDNC(+57.02)YGEAEKIPDC(+57.02)NPKTK.T | Y | 64.36 | 2857.1929 | 23 | 1.1 | 715.3063 | 4 | 12.13 | 1 | 2099 | 01122020\_RID\_2209\_BuCaKA02.raw | 8.1534E5 | 1 | 1 | 26 | 48 | Carbamidomethylation | C1:Carbamidomethylation:1000.00;C2:Carbamidomethylation:1000.00;C8:Carbamidomethylation:1000.00;C18:Carbamidomethylation:1000.00 | PEAKS DB |
| K.YGC(+57.02)YC(+57.02)GYGGTGTPVDELDRC(+57.02)C(+57.02).Y | N | 63.90 | 2458.9287 | 21 | 1.8 | 1230.4739 | 2 | 48.28 | 1 | 29764 | 01122020\_RID\_2209\_BuCaKA02.raw | 8.9471E6 | 1 | 1 | 7 | 27 | Carbamidomethylation | C3:Carbamidomethylation:1000.00;C5:Carbamidomethylation:1000.00;C20:Carbamidomethylation:1000.00;C21:Carbamidomethylation:1000.00 | PEAKS DB |
| Y.NIGNIMISASSAC(+57.02)Q | Y | 63.31 | 1464.6700 | 14 | 1.1 | 733.3431 | 2 | 56.43 | 1 | 36685 | 01122020\_RID\_2209\_BuCaKA02.raw | 2.8731E8 | 3 | 3 | 89 | 102 | Carbamidomethylation | C13:Carbamidomethylation:1000.00 | PEAKS DB |
| R.TAALC(+57.02)FAKAPYNI.G | N | 62.47 | 1438.7278 | 13 | 3.0 | 720.3733 | 2 | 50.01 | 1 | 31149 | 01122020\_RID\_2209\_BuCaKA02.raw | 0 | 0 | 0 | 78 | 90 | Carbamidomethylation | C5:Carbamidomethylation:1000.00 | PEAKS DB |
| F.LC(+57.02)DC(+57.02)DRTAALC(+57.02)FAK.A | N | 61.05 | 1699.7480 | 14 | 2.4 | 567.5913 | 3 | 22.40 | 1 | 9949 | 01122020\_RID\_2209\_BuCaKA02.raw | 3.0159E7 | 2 | 2 | 72 | 85 | Carbamidomethylation | C2:Carbamidomethylation:1000.00;C4:Carbamidomethylation:1000.00;C11:Carbamidomethylation:1000.00 | PEAKS DB |
| K.APYNIGNIM(+15.99)ISASSAC(+57.02)Q | Y | 60.45 | 1811.8182 | 17 | 0.9 | 906.9172 | 2 | 59.67 | 1 | 39709 | 01122020\_RID\_2209\_BuCaKA02.raw | 4.2632E8 | 1 | 1 | 86 | 102 | Oxidation (M); Carbamidomethylation | M9:Oxidation (M):1000.00;C16:Carbamidomethylation:1000.00 | PEAKS DB |
| K.APYNIGNIMISASSA.C | Y | 57.48 | 1507.7340 | 15 | -2.9 | 754.8721 | 2 | 51.18 | 1 | 31949 | 01122020\_RID\_2209\_BuCaKA02.raw | 1.0449E10 | 5 | 5 | 86 | 100 |  |  | PEAKS DB |
| A.YLKYGC(+57.02)YC(+57.02)GYGGTGTPVDELDR.C | N | 57.42 | 2543.1096 | 22 | 0.4 | 848.7108 | 3 | 42.36 | 1 | 25537 | 01122020\_RID\_2209\_BuCaKA02.raw | 4.2549E7 | 1 | 1 | 4 | 25 | Carbamidomethylation | C6:Carbamidomethylation:1000.00;C8:Carbamidomethylation:1000.00 | PEAKS DB |
| K.APYNIGNIMISAS.S | Y | 55.54 | 1349.6649 | 13 | 0.0 | 675.8397 | 2 | 72.27 | 1 | 49682 | 01122020\_RID\_2209\_BuCaKA02.raw | 1.2205E9 | 2 | 2 | 86 | 98 |  |  | PEAKS DB |
| C.DRTAALC(+57.02)FAK.A | N | 55.24 | 1151.5757 | 10 | 2.7 | 576.7967 | 2 | 15.19 | 1 | 4454 | 01122020\_RID\_2209\_BuCaKA02.raw | 3.7655E5 | 1 | 1 | 76 | 85 | Carbamidomethylation | C7:Carbamidomethylation:1000.00 | PEAKS DB |
| R.C(+57.02)C(+57.02)YVHDNC(+57.02)YGEAEKIPDC(+57.02)NPKT.K | Y | 52.77 | 2729.0979 | 22 | 2.1 | 683.2832 | 4 | 17.55 | 1 | 6265 | 01122020\_RID\_2209\_BuCaKA02.raw | 1.0249E6 | 1 | 1 | 26 | 47 | Carbamidomethylation | C1:Carbamidomethylation:1000.00;C2:Carbamidomethylation:1000.00;C8:Carbamidomethylation:1000.00;C18:Carbamidomethylation:1000.00 | PEAKS DB |
| R.FLC(+57.02)DC(+57.02)DRTAALC(+57.02)FAKAPYNIGNIMISASSAC(+57.02)Q | Y | 49.52 | 3624.6292 | 32 | -0.8 | 1209.2161 | 3 | 76.04 | 1 | 52816 | 01122020\_RID\_2209\_BuCaKA02.raw | 1.4982E9 | 2 | 2 | 71 | 102 | Carbamidomethylation | C3:Carbamidomethylation:1000.00;C5:Carbamidomethylation:1000.00;C12:Carbamidomethylation:1000.00;C31:Carbamidomethylation:1000.00 | PEAKS DB |
| K.APYNIGNIM(+15.99)ISAS.S | Y | 48.61 | 1365.6598 | 13 | 1.3 | 683.8381 | 2 | 61.42 | 1 | 40518 | 01122020\_RID\_2209\_BuCaKA02.raw | 0 | 0 | 0 | 86 | 98 | Oxidation (M) | M9:Oxidation (M):1000.00 | PEAKS DB |
| K.APYNIGNIMIS.A | N | 48.60 | 1191.5958 | 11 | 0.9 | 596.8057 | 2 | 70.10 | 1 | 47633 | 01122020\_RID\_2209\_BuCaKA02.raw | 3.0202E8 | 1 | 1 | 86 | 96 |  |  | PEAKS DB |
| D.ELDRC(+57.02)C(+57.02)YVHDNC(+57.02)YGEAEKIPDC(+57.02)NPK.T | Y | 48.14 | 3141.3049 | 25 | 5.7 | 786.3380 | 4 | 19.41 | 1 | 7734 | 01122020\_RID\_2209\_BuCaKA02.raw | 1.9236E6 | 1 | 1 | 22 | 46 | Carbamidomethylation | C5:Carbamidomethylation:1000.00;C6:Carbamidomethylation:1000.00;C12:Carbamidomethylation:1000.00;C22:Carbamidomethylation:1000.00 | PEAKS DB |
| C.DC(+57.02)DRTAALC(+57.02)FAK.A | N | 47.16 | 1426.6333 | 12 | -2.2 | 714.3224 | 2 | 20.22 | 1 | 8374 | 01122020\_RID\_2209\_BuCaKA02.raw | 1.7388E6 | 1 | 1 | 74 | 85 | Carbamidomethylation | C2:Carbamidomethylation:1000.00;C9:Carbamidomethylation:1000.00 | PEAKS DB |
| P.YNIGNIMISASSAC(+57.02)Q | Y | 46.66 | 1627.7334 | 15 | 0.1 | 814.8740 | 2 | 69.24 | 1 | 46840 | 01122020\_RID\_2209\_BuCaKA02.raw | 4.165E8 | 1 | 1 | 88 | 102 | Carbamidomethylation | C14:Carbamidomethylation:1000.00 | PEAKS DB |
| K.APYNIGNIMISASS.A | Y | 46.05 | 1436.6969 | 14 | 0.5 | 719.3561 | 2 | 72.07 | 1 | 49113 | 01122020\_RID\_2209\_BuCaKA02.raw | 3.3983E7 | 1 | 1 | 86 | 99 |  |  | PEAKS DB |
| V.DELDRC(+57.02)C(+57.02)YVHDNC(+57.02)YGEAEKIPDC(+57.02)NPK.T | Y | 44.36 | 3256.3318 | 26 | 1.9 | 815.0917 | 4 | 23.64 | 1 | 11072 | 01122020\_RID\_2209\_BuCaKA02.raw | 8.0747E6 | 1 | 1 | 21 | 46 | Carbamidomethylation | C6:Carbamidomethylation:1000.00;C7:Carbamidomethylation:1000.00;C13:Carbamidomethylation:1000.00;C23:Carbamidomethylation:1000.00 | PEAKS DB |
| R.FLC(+57.02)DC(+57.02)DRTAALC(+57.02)F.A | N | 44.26 | 1647.6843 | 13 | 0.5 | 824.8499 | 2 | 55.77 | 1 | 35810 | 01122020\_RID\_2209\_BuCaKA02.raw | 2.2718E7 | 1 | 1 | 71 | 83 | Carbamidomethylation | C3:Carbamidomethylation:1000.00;C5:Carbamidomethylation:1000.00;C12:Carbamidomethylation:1000.00 | PEAKS DB |
| Y.GGTGTPVDELDR.C | N | 44.26 | 1215.5731 | 12 | 0.2 | 608.7939 | 2 | 24.73 | 1 | 11888 | 01122020\_RID\_2209\_BuCaKA02.raw | 2.8666E5 | 1 | 1 | 14 | 25 |  |  | PEAKS DB |
| K.APYNIGNIM(+15.99)ISASSA.C | Y | 41.40 | 1523.7290 | 15 | 0.9 | 762.8724 | 2 | 60.99 | 1 | 40159 | 01122020\_RID\_2209\_BuCaKA02.raw | 3.4723E8 | 2 | 2 | 86 | 100 | Oxidation (M) | M9:Oxidation (M):1000.00 | PEAKS DB |
| R.TAALC(+57.02)FAKAPYNIGNIMISAS.S | Y | 41.38 | 2212.1021 | 21 | 1.0 | 1107.0594 | 2 | 74.27 | 1 | 51209 | 01122020\_RID\_2209\_BuCaKA02.raw | 1.3388E8 | 1 | 1 | 78 | 98 | Carbamidomethylation | C5:Carbamidomethylation:1000.00 | PEAKS DB |
| K.APYNIGNIMISA.S | Y | 40.97 | 1262.6329 | 12 | 0.8 | 632.3242 | 2 | 74.12 | 1 | 51059 | 01122020\_RID\_2209\_BuCaKA02.raw | 7.7197E8 | 2 | 2 | 86 | 97 |  |  | PEAKS DB |
| WIAYLKYGC(+57.02)YC(+57.02)G.Y | N | 40.55 | 1552.6843 | 12 | 3.1 | 777.3518 | 2 | 53.53 | 1 | 33953 | 01122020\_RID\_2209\_BuCaKA02.raw | 2.1406E6 | 1 | 1 | 1 | 12 | Carbamidomethylation | C9:Carbamidomethylation:1000.00;C11:Carbamidomethylation:1000.00 | PEAKS DB |
| D.C(+57.02)DRTAALC(+57.02)FAK.A | N | 39.74 | 1311.6063 | 11 | 1.6 | 656.8115 | 2 | 12.51 | 1 | 2398 | 01122020\_RID\_2209\_BuCaKA02.raw | 5.3941E5 | 1 | 1 | 75 | 85 | Carbamidomethylation | C1:Carbamidomethylation:1000.00;C8:Carbamidomethylation:1000.00 | PEAKS DB |
| total 40 peptides |  |  |  |  |  |  |  |  |  |  |  |  |  |  |  |  |  |  |  |
| --- | --- | --- | --- | --- | --- | --- | --- | --- | --- | --- | --- | --- | --- | --- | --- | --- | --- | --- | --- |

T\_18

back to list

  

| Protein Coverage
| Supporting Peptides
|

Protein Coverage:

Supporting Peptides:

| Peptide | Uniq | -10lgP | Mass | Length | ppm | m/z | z | RT | Fraction | Scan | Source File | Area BRom | #Feature | #Feature BRom | Start | End | PTM | AScore | Found By |
| --- | --- | --- | --- | --- | --- | --- | --- | --- | --- | --- | --- | --- | --- | --- | --- | --- | --- | --- | --- |
| L.KYGC(+57.02)YC(+57.02)GYGGTGTPVDELDR.C | N | 88.44 | 2266.9624 | 20 | -2.4 | 1134.4857 | 2 | 32.64 | 1 | 17782 | 01122020\_RID\_2209\_BuCaKA02.raw | 3.5219E7 | 2 | 2 | 77 | 96 | Carbamidomethylation | C4:Carbamidomethylation:1000.00;C6:Carbamidomethylation:1000.00 | PEAKS DB |
| K.YGC(+57.02)YC(+57.02)GYGGTGTPVDELDR.C | N | 88.01 | 2138.8674 | 19 | 0.4 | 1070.4414 | 2 | 44.33 | 1 | 26645 | 01122020\_RID\_2209\_BuCaKA02.raw | 9.6633E9 | 3 | 3 | 78 | 96 | Carbamidomethylation | C3:Carbamidomethylation:1000.00;C5:Carbamidomethylation:1000.00 | PEAKS DB |
| K.NMIQC(+57.02)AGTRPWIAYLK.Y | N | 87.82 | 1920.9702 | 16 | 0.2 | 961.4926 | 2 | 50.49 | 1 | 31479 | 01122020\_RID\_2209\_BuCaKA02.raw | 6.2231E10 | 47 | 47 | 62 | 77 | Carbamidomethylation | C5:Carbamidomethylation:1000.00 | PEAKS DB |
| K.TYSYDC(+57.02)SEGKLTC(+57.02)KDTAGTC(+57.02)ER.I | N | 86.11 | 2601.0781 | 22 | 1.5 | 651.2778 | 4 | 12.08 | 1 | 2046 | 01122020\_RID\_2209\_BuCaKA02.raw | 0 | 0 | 0 | 120 | 141 | Carbamidomethylation | C6:Carbamidomethylation:1000.00;C13:Carbamidomethylation:1000.00;C20:Carbamidomethylation:1000.00 | PEAKS DB |
| Y.GC(+57.02)YC(+57.02)GYGGTGTPVDELDR.C | N | 85.01 | 1975.8040 | 18 | 0.4 | 988.9097 | 2 | 38.86 | 1 | 23078 | 01122020\_RID\_2209\_BuCaKA02.raw | 3.3628E9 | 2 | 2 | 79 | 96 | Carbamidomethylation | C2:Carbamidomethylation:1000.00;C4:Carbamidomethylation:1000.00 | PEAKS DB |
| Y.C(+57.02)GYGGTGTPVDELDR.C | N | 82.21 | 1595.6886 | 15 | 1.6 | 798.8528 | 2 | 33.04 | 1 | 18122 | 01122020\_RID\_2209\_BuCaKA02.raw | 5.3035E8 | 3 | 3 | 82 | 96 | Carbamidomethylation | C1:Carbamidomethylation:1000.00 | PEAKS DB |
| C.YC(+57.02)GYGGTGTPVDELDR.C | N | 81.92 | 1758.7518 | 16 | 0.3 | 880.3834 | 2 | 37.99 | 1 | 22078 | 01122020\_RID\_2209\_BuCaKA02.raw | 4.9681E7 | 1 | 1 | 81 | 96 | Carbamidomethylation | C2:Carbamidomethylation:1000.00 | PEAKS DB |
| G.C(+57.02)YC(+57.02)GYGGTGTPVDELDR.C | N | 77.93 | 1918.7826 | 17 | 1.1 | 960.3996 | 2 | 38.92 | 1 | 22863 | 01122020\_RID\_2209\_BuCaKA02.raw | 7.0014E7 | 2 | 2 | 80 | 96 | Carbamidomethylation | C1:Carbamidomethylation:1000.00;C3:Carbamidomethylation:1000.00 | PEAKS DB |
| L.NLYQLKNMIQC(+57.02)AGTRPWIAYLK.Y | Y | 77.59 | 2680.3982 | 22 | 0.9 | 894.4741 | 3 | 71.26 | 1 | 48913 | 01122020\_RID\_2209\_BuCaKA02.raw | 5.746E10 | 63 | 63 | 56 | 77 | Carbamidomethylation | C11:Carbamidomethylation:1000.00 | PEAKS DB |
| R.C(+57.02)C(+57.02)QTHDNC(+57.02)YGEAEKLASC(+57.02)RPYYK.T | N | 77.20 | 2909.1990 | 23 | 0.8 | 728.3076 | 4 | 19.12 | 1 | 7427 | 01122020\_RID\_2209\_BuCaKA02.raw | 4.5345E7 | 2 | 2 | 97 | 119 | Carbamidomethylation | C1:Carbamidomethylation:1000.00;C2:Carbamidomethylation:1000.00;C8:Carbamidomethylation:1000.00;C18:Carbamidomethylation:1000.00 | PEAKS DB |
| P.LNLYQLKNMIQC(+57.02)AGTRPWIAYLK.Y | Y | 75.69 | 2793.4822 | 23 | 1.0 | 932.1689 | 3 | 77.16 | 1 | 53378 | 01122020\_RID\_2209\_BuCaKA02.raw | 2.4262E8 | 5 | 5 | 55 | 77 | Carbamidomethylation | C12:Carbamidomethylation:1000.00 | PEAKS DB |
| K.NM(+15.99)IQC(+57.02)AGTRPWIAYLK.Y | N | 74.51 | 1936.9651 | 16 | 1.2 | 969.4910 | 2 | 42.21 | 1 | 25397 | 01122020\_RID\_2209\_BuCaKA02.raw | 3.1572E9 | 14 | 14 | 62 | 77 | Oxidation (M); Carbamidomethylation | M2:Oxidation (M):1000.00;C5:Carbamidomethylation:1000.00 | PEAKS DB |
| N.MIQC(+57.02)AGTRPWIAYLK.Y | N | 71.95 | 1806.9272 | 15 | 1.1 | 904.4719 | 2 | 44.83 | 1 | 27096 | 01122020\_RID\_2209\_BuCaKA02.raw | 1.4268E9 | 2 | 2 | 63 | 77 | Carbamidomethylation | C4:Carbamidomethylation:1000.00 | PEAKS DB |
| K.NMIQC(+57.02)AGTRPWIAY.L | N | 70.22 | 1679.7913 | 14 | 1.4 | 840.9041 | 2 | 52.63 | 1 | 32999 | 01122020\_RID\_2209\_BuCaKA02.raw | 1.9188E9 | 1 | 1 | 62 | 75 | Carbamidomethylation | C5:Carbamidomethylation:1000.00 | PEAKS DB |
| M.IQC(+57.02)AGTRPWIAYLK.Y | N | 70.19 | 1675.8868 | 14 | 0.6 | 838.9512 | 2 | 39.82 | 1 | 23719 | 01122020\_RID\_2209\_BuCaKA02.raw | 1.3909E9 | 2 | 2 | 64 | 77 | Carbamidomethylation | C3:Carbamidomethylation:1000.00 | PEAKS DB |
| C.GYGGTGTPVDELDR.C | N | 70.03 | 1435.6580 | 14 | 1.4 | 718.8373 | 2 | 31.57 | 1 | 17094 | 01122020\_RID\_2209\_BuCaKA02.raw | 9.8214E7 | 1 | 1 | 83 | 96 |  |  | PEAKS DB |
| L.YQLKNMIQC(+57.02)AGTRPWIAYLK.Y | Y | 69.46 | 2453.2712 | 20 | 1.5 | 818.7656 | 3 | 55.12 | 1 | 35462 | 01122020\_RID\_2209\_BuCaKA02.raw | 6.0347E7 | 1 | 1 | 58 | 77 | Carbamidomethylation | C9:Carbamidomethylation:1000.00 | PEAKS DB |
| Y.LKYGC(+57.02)YC(+57.02)GYGGTGTPVDELDR.C | N | 69.19 | 2380.0464 | 21 | 0.9 | 794.3568 | 3 | 38.32 | 1 | 22508 | 01122020\_RID\_2209\_BuCaKA02.raw | 1.3586E8 | 1 | 1 | 76 | 96 | Carbamidomethylation | C5:Carbamidomethylation:1000.00;C7:Carbamidomethylation:1000.00 | PEAKS DB |
| I.QC(+57.02)AGTRPWIAYLK.Y | N | 68.16 | 1562.8027 | 13 | 0.8 | 782.4093 | 2 | 36.01 | 1 | 20489 | 01122020\_RID\_2209\_BuCaKA02.raw | 3.141E7 | 1 | 1 | 65 | 77 | Carbamidomethylation | C2:Carbamidomethylation:1000.00 | PEAKS DB |
| G.YGGTGTPVDELDR.C | N | 68.14 | 1378.6365 | 13 | 1.7 | 690.3267 | 2 | 30.08 | 1 | 15917 | 01122020\_RID\_2209\_BuCaKA02.raw | 1.3749E8 | 1 | 1 | 84 | 96 |  |  | PEAKS DB |
| L.NLYQLKNM(+15.99)IQC(+57.02)AGTRPWIAYLK.Y | Y | 67.77 | 2696.3931 | 22 | 0.6 | 899.8055 | 3 | 62.34 | 1 | 41258 | 01122020\_RID\_2209\_BuCaKA02.raw | 2.2215E9 | 6 | 6 | 56 | 77 | Oxidation (M); Carbamidomethylation | M8:Oxidation (M):1000.00;C11:Carbamidomethylation:1000.00 | PEAKS DB |
| L.KNMIQC(+57.02)AGTRPWIAYLK.Y | N | 67.22 | 2049.0652 | 17 | 1.1 | 684.0298 | 3 | 37.77 | 1 | 21937 | 01122020\_RID\_2209\_BuCaKA02.raw | 8.2939E7 | 1 | 1 | 61 | 77 | Carbamidomethylation | C6:Carbamidomethylation:1000.00 | PEAKS DB |
| K.NMIQC(+57.02)AGTRPWIAYL.K | N | 65.66 | 1792.8752 | 15 | 0.5 | 897.4453 | 2 | 70.83 | 1 | 48151 | 01122020\_RID\_2209\_BuCaKA02.raw | 1.1174E9 | 2 | 2 | 62 | 76 | Carbamidomethylation | C5:Carbamidomethylation:1000.00 | PEAKS DB |
| K.YGC(+57.02)YC(+57.02)GYGGTGTPVDELDRC(+57.02)C(+57.02).Q | N | 63.90 | 2458.9287 | 21 | 1.8 | 1230.4739 | 2 | 48.28 | 1 | 29764 | 01122020\_RID\_2209\_BuCaKA02.raw | 8.9471E6 | 1 | 1 | 78 | 98 | Carbamidomethylation | C3:Carbamidomethylation:1000.00;C5:Carbamidomethylation:1000.00;C20:Carbamidomethylation:1000.00;C21:Carbamidomethylation:1000.00 | PEAKS DB |
| L.NLYQLKNMIQC(+57.02)AGTRPWIAYLKYGC(+57.02)YC(+57.02)GYGGTGTPVDELDR.C | Y | 57.62 | 4801.2549 | 41 | 2.1 | 961.2603 | 5 | 73.99 | 1 | 51011 | 01122020\_RID\_2209\_BuCaKA02.raw | 5.1197E9 | 2 | 2 | 56 | 96 | Carbamidomethylation | C11:Carbamidomethylation:1000.00;C25:Carbamidomethylation:1000.00;C27:Carbamidomethylation:1000.00 | PEAKS DB |
| A.YLKYGC(+57.02)YC(+57.02)GYGGTGTPVDELDR.C | N | 57.42 | 2543.1096 | 22 | 0.4 | 848.7108 | 3 | 42.36 | 1 | 25537 | 01122020\_RID\_2209\_BuCaKA02.raw | 4.2549E7 | 1 | 1 | 75 | 96 | Carbamidomethylation | C6:Carbamidomethylation:1000.00;C8:Carbamidomethylation:1000.00 | PEAKS DB |
| K.NMIQC(+57.02)AGTRPWIAYLKYGC(+57.02)YC(+57.02)GYGGTGTPVDELDR.C | N | 54.44 | 4041.8269 | 35 | 0.5 | 1011.4645 | 4 | 65.21 | 1 | 43707 | 01122020\_RID\_2209\_BuCaKA02.raw | 4.1718E8 | 1 | 1 | 62 | 96 | Carbamidomethylation | C5:Carbamidomethylation:1000.00;C19:Carbamidomethylation:1000.00;C21:Carbamidomethylation:1000.00 | PEAKS DB |
| K.NMIQC(+57.02)AGTRPWIA.Y | N | 53.98 | 1516.7279 | 13 | 0.1 | 759.3713 | 2 | 43.19 | 1 | 25942 | 01122020\_RID\_2209\_BuCaKA02.raw | 2.5319E7 | 1 | 1 | 62 | 74 | Carbamidomethylation | C5:Carbamidomethylation:1000.00 | PEAKS DB |
| A.GTRPWIAYLK.Y | N | 49.87 | 1203.6764 | 10 | -0.2 | 602.8453 | 2 | 28.40 | 1 | 14933 | 01122020\_RID\_2209\_BuCaKA02.raw | 1.9541E8 | 2 | 2 | 68 | 77 |  |  | PEAKS DB |
| K.NM(+15.99)IQC(+57.02)AGTRPWIAYL.K | N | 48.28 | 1808.8702 | 15 | 0.3 | 905.4426 | 2 | 63.61 | 1 | 42209 | 01122020\_RID\_2209\_BuCaKA02.raw | 4.458E7 | 1 | 1 | 62 | 76 | Oxidation (M); Carbamidomethylation | M2:Oxidation (M):1000.00;C5:Carbamidomethylation:1000.00 | PEAKS DB |
| Q.C(+57.02)AGTRPWIAYLK.Y | N | 45.87 | 1434.7441 | 12 | 1.2 | 718.3802 | 2 | 28.92 | 1 | 14936 | 01122020\_RID\_2209\_BuCaKA02.raw | 0 | 0 | 0 | 66 | 77 | Carbamidomethylation | C1:Carbamidomethylation:1000.00 | PEAKS DB |
| Y.GGTGTPVDELDR.C | N | 44.26 | 1215.5731 | 12 | 0.2 | 608.7939 | 2 | 24.73 | 1 | 11888 | 01122020\_RID\_2209\_BuCaKA02.raw | 2.8666E5 | 1 | 1 | 85 | 96 |  |  | PEAKS DB |
| K.LASC(+57.02)RPYYKTYSYDC(+57.02)SEGKLTC(+57.02)K.D | N | 41.20 | 2849.2822 | 23 | 1.6 | 713.3290 | 4 | 14.97 | 1 | 4367 | 01122020\_RID\_2209\_BuCaKA02.raw | 1.3525E6 | 1 | 1 | 111 | 133 | Carbamidomethylation | C4:Carbamidomethylation:1000.00;C15:Carbamidomethylation:1000.00;C22:Carbamidomethylation:1000.00 | PEAKS DB |
| total 33 peptides |  |  |  |  |  |  |  |  |  |  |  |  |  |  |  |  |  |  |  |
| --- | --- | --- | --- | --- | --- | --- | --- | --- | --- | --- | --- | --- | --- | --- | --- | --- | --- | --- | --- |

T\_24

back to list

  

| Protein Coverage
| Supporting Peptides
|

Protein Coverage:

Supporting Peptides:

| Peptide | Uniq | -10lgP | Mass | Length | ppm | m/z | z | RT | Fraction | Scan | Source File | Area BRom | #Feature | #Feature BRom | Start | End | PTM | AScore | Found By |
| --- | --- | --- | --- | --- | --- | --- | --- | --- | --- | --- | --- | --- | --- | --- | --- | --- | --- | --- | --- |
| K.YGC(+57.02)YC(+57.02)GYGGSGTPVDELDR.C | N | 91.39 | 2124.8516 | 19 | 0.2 | 1063.4332 | 2 | 41.66 | 1 | 24749 | 01122020\_RID\_2209\_BuCaKA02.raw | 2.5458E9 | 1 | 1 | 39 | 57 | Carbamidomethylation | C3:Carbamidomethylation:1000.00;C5:Carbamidomethylation:1000.00 | PEAKS DB |
| K.KIPGC(+57.02)NPIIKIYSYTC(+57.02)TKPNLTC(+57.02)K.D | Y | 88.60 | 2868.4700 | 24 | 0.4 | 574.7015 | 5 | 35.86 | 1 | 20864 | 01122020\_RID\_2209\_BuCaKA02.raw | 1.4414E10 | 4 | 4 | 71 | 94 | Carbamidomethylation | C5:Carbamidomethylation:1000.00;C16:Carbamidomethylation:1000.00;C23:Carbamidomethylation:1000.00 | PEAKS DB |
| R.TWVAYLKYGC(+57.02)YC(+57.02)GYGGSGTPVDELDR.C | Y | 84.57 | 2986.3264 | 26 | 0.8 | 996.4502 | 3 | 62.43 | 1 | 42011 | 01122020\_RID\_2209\_BuCaKA02.raw | 1.6892E9 | 19 | 19 | 32 | 57 | Carbamidomethylation | C10:Carbamidomethylation:1000.00;C12:Carbamidomethylation:1000.00 | PEAKS DB |
| Y.C(+57.02)GYGGSGTPVDELDR.C | N | 83.79 | 1581.6729 | 15 | 0.8 | 791.8444 | 2 | 29.21 | 1 | 14986 | 01122020\_RID\_2209\_BuCaKA02.raw | 1.287E8 | 1 | 1 | 43 | 57 | Carbamidomethylation | C1:Carbamidomethylation:1000.00 | PEAKS DB |
| K.NMIQC(+57.02)AGTRTWVAYLK.Y | N | 82.79 | 1910.9495 | 16 | 1.4 | 956.4833 | 2 | 45.28 | 1 | 27489 | 01122020\_RID\_2209\_BuCaKA02.raw | 2.818E9 | 2 | 2 | 23 | 38 | Carbamidomethylation | C5:Carbamidomethylation:1000.00 | PEAKS DB |
| Y.GC(+57.02)YC(+57.02)GYGGSGTPVDELDR.C | N | 81.97 | 1961.7883 | 18 | 1.3 | 981.9027 | 2 | 36.23 | 1 | 20719 | 01122020\_RID\_2209\_BuCaKA02.raw | 1.0001E9 | 2 | 2 | 40 | 57 | Carbamidomethylation | C2:Carbamidomethylation:1000.00;C4:Carbamidomethylation:1000.00 | PEAKS DB |
| L.NLYQFKNMIQC(+57.02)AGTRTWVAYLK.Y | Y | 75.59 | 2704.3618 | 22 | 0.9 | 902.4620 | 3 | 63.14 | 1 | 41909 | 01122020\_RID\_2209\_BuCaKA02.raw | 1.3687E10 | 5 | 5 | 17 | 38 | Carbamidomethylation | C11:Carbamidomethylation:1000.00 | PEAKS DB |
| L.NLYQFKNMIQC(+57.02)AGTR.T | Y | 75.28 | 1842.8868 | 15 | 1.1 | 615.3036 | 3 | 39.54 | 1 | 23359 | 01122020\_RID\_2209\_BuCaKA02.raw | 2.9212E9 | 4 | 4 | 17 | 31 | Carbamidomethylation | C11:Carbamidomethylation:1000.00 | PEAKS DB |
| C.YC(+57.02)GYGGSGTPVDELDR.C | N | 74.25 | 1744.7362 | 16 | 1.7 | 873.3768 | 2 | 34.92 | 1 | 19611 | 01122020\_RID\_2209\_BuCaKA02.raw | 1.162E7 | 1 | 1 | 42 | 57 | Carbamidomethylation | C2:Carbamidomethylation:1000.00 | PEAKS DB |
| G.C(+57.02)YC(+57.02)GYGGSGTPVDELDR.C | N | 74.02 | 1904.7668 | 17 | 1.3 | 953.3920 | 2 | 36.10 | 1 | 20597 | 01122020\_RID\_2209\_BuCaKA02.raw | 8.7199E6 | 1 | 1 | 41 | 57 | Carbamidomethylation | C1:Carbamidomethylation:1000.00;C3:Carbamidomethylation:1000.00 | PEAKS DB |
| K.IPGC(+57.02)NPIIKIYSYTC(+57.02)TKPNLTC(+57.02)K.D | Y | 72.71 | 2740.3750 | 23 | 1.1 | 686.1018 | 4 | 43.76 | 1 | 26470 | 01122020\_RID\_2209\_BuCaKA02.raw | 7.1863E8 | 1 | 1 | 72 | 94 | Carbamidomethylation | C4:Carbamidomethylation:1000.00;C15:Carbamidomethylation:1000.00;C22:Carbamidomethylation:1000.00 | PEAKS DB |
| C.GYGGSGTPVDELDR.C | N | 70.31 | 1421.6422 | 14 | 2.0 | 711.8298 | 2 | 27.36 | 1 | 13794 | 01122020\_RID\_2209\_BuCaKA02.raw | 2.8856E7 | 1 | 1 | 44 | 57 |  |  | PEAKS DB |
| K.IPGC(+57.02)NPIIKIYSYTC(+57.02)TKPNLTC(+57.02)KDVK.D | Y | 69.32 | 3082.5654 | 26 | 1.0 | 771.6494 | 4 | 38.33 | 1 | 22429 | 01122020\_RID\_2209\_BuCaKA02.raw | 2.5725E9 | 3 | 3 | 72 | 97 | Carbamidomethylation | C4:Carbamidomethylation:1000.00;C15:Carbamidomethylation:1000.00;C22:Carbamidomethylation:1000.00 | PEAKS DB |
| L.KYGC(+57.02)YC(+57.02)GYGGSGTPVDELDR.C | Y | 63.58 | 2252.9465 | 20 | 1.2 | 751.9904 | 3 | 29.85 | 1 | 15544 | 01122020\_RID\_2209\_BuCaKA02.raw | 8.0367E6 | 1 | 1 | 38 | 57 | Carbamidomethylation | C4:Carbamidomethylation:1000.00;C6:Carbamidomethylation:1000.00 | PEAKS DB |
| L.NLYQFKNM(+15.99)IQC(+57.02)AGTR.T | Y | 61.62 | 1858.8818 | 15 | 1.2 | 620.6353 | 3 | 25.20 | 1 | 12451 | 01122020\_RID\_2209\_BuCaKA02.raw | 5.7286E7 | 3 | 3 | 17 | 31 | Oxidation (M); Carbamidomethylation | M8:Oxidation (M):1000.00;C11:Carbamidomethylation:1000.00 | PEAKS DB |
| K.IPGC(+57.02)NPIIKIYS.Y | Y | 60.96 | 1373.7377 | 12 | 1.0 | 687.8768 | 2 | 48.28 | 1 | 29622 | 01122020\_RID\_2209\_BuCaKA02.raw | 4.5465E8 | 1 | 1 | 72 | 83 | Carbamidomethylation | C4:Carbamidomethylation:1000.00 | PEAKS DB |
| G.YGGSGTPVDELDRC(+57.02)C(+57.02)YVHDHC(+57.02)YDK.A | Y | 60.11 | 2902.1746 | 24 | 0.8 | 726.5515 | 4 | 23.42 | 1 | 10827 | 01122020\_RID\_2209\_BuCaKA02.raw | 8.9648E6 | 1 | 1 | 45 | 68 | Carbamidomethylation | C14:Carbamidomethylation:1000.00;C15:Carbamidomethylation:1000.00;C21:Carbamidomethylation:1000.00 | PEAKS DB |
| K.KIPGC(+57.02)NPIIKIYSYTC(+57.02)TKPNL.T | Y | 58.75 | 2479.2966 | 21 | 2.0 | 827.4412 | 3 | 44.40 | 1 | 26904 | 01122020\_RID\_2209\_BuCaKA02.raw | 4.7303E7 | 2 | 2 | 71 | 91 | Carbamidomethylation | C5:Carbamidomethylation:1000.00;C16:Carbamidomethylation:1000.00 | PEAKS DB |
| G.YGGSGTPVDELDRC(+57.02)C(+57.02)YVHDHC(+57.02)YDKAK.K | Y | 56.87 | 3101.3066 | 26 | 1.8 | 776.3353 | 4 | 18.74 | 1 | 7255 | 01122020\_RID\_2209\_BuCaKA02.raw | 5.3908E6 | 1 | 1 | 45 | 70 | Carbamidomethylation | C14:Carbamidomethylation:1000.00;C15:Carbamidomethylation:1000.00;C21:Carbamidomethylation:1000.00 | PEAKS DB |
| K.YGC(+57.02)YC(+57.02)GYGGSGTPVDELDRC(+57.02)C(+57.02)YVHDHC(+57.02)YDK.A | Y | 56.09 | 3662.4053 | 30 | 0.7 | 916.6093 | 4 | 32.67 | 1 | 17727 | 01122020\_RID\_2209\_BuCaKA02.raw | 2.7434E9 | 2 | 2 | 39 | 68 | Carbamidomethylation | C3:Carbamidomethylation:1000.00;C5:Carbamidomethylation:1000.00;C20:Carbamidomethylation:1000.00;C21:Carbamidomethylation:1000.00;C27:Carbamidomethylation:1000.00 | PEAKS DB |
| K.KIPGC(+57.02)NPIIKIYSYTC(+57.02)TKP.N | Y | 53.57 | 2252.1697 | 19 | 1.2 | 564.0504 | 4 | 37.55 | 1 | 21835 | 01122020\_RID\_2209\_BuCaKA02.raw | 2.8126E7 | 1 | 1 | 71 | 89 | Carbamidomethylation | C5:Carbamidomethylation:1000.00;C16:Carbamidomethylation:1000.00 | PEAKS DB |
| N.MIQC(+57.02)AGTRTWVAYLK.Y | N | 51.37 | 1796.9066 | 15 | 0.7 | 599.9766 | 3 | 39.91 | 1 | 23605 | 01122020\_RID\_2209\_BuCaKA02.raw | 2.6769E7 | 1 | 1 | 24 | 38 | Carbamidomethylation | C4:Carbamidomethylation:1000.00 | PEAKS DB |
| K.KIPGC(+57.02)NPIIKIYS.Y | Y | 49.49 | 1501.8326 | 13 | 0.9 | 751.9243 | 2 | 34.62 | 1 | 19408 | 01122020\_RID\_2209\_BuCaKA02.raw | 1.0011E9 | 1 | 1 | 71 | 83 | Carbamidomethylation | C5:Carbamidomethylation:1000.00 | PEAKS DB |
| K.YGC(+57.02)YC(+57.02)GYGGSGTPVDELDRC(+57.02)C(+57.02)YVHDHC(+57.02)YDKAK.K | Y | 47.54 | 3861.5374 | 32 | 0.4 | 966.3920 | 4 | 26.72 | 1 | 13539 | 01122020\_RID\_2209\_BuCaKA02.raw | 1.0723E9 | 3 | 3 | 39 | 70 | Carbamidomethylation | C3:Carbamidomethylation:1000.00;C5:Carbamidomethylation:1000.00;C20:Carbamidomethylation:1000.00;C21:Carbamidomethylation:1000.00;C27:Carbamidomethylation:1000.00 | PEAKS DB |
| K.IPGC(+57.02)NPIIKIYSYT.C | Y | 45.87 | 1637.8486 | 14 | 0.2 | 819.9318 | 2 | 58.87 | 1 | 38326 | 01122020\_RID\_2209\_BuCaKA02.raw | 2.7144E7 | 1 | 1 | 72 | 85 | Carbamidomethylation | C4:Carbamidomethylation:1000.00 | PEAKS DB |
| L.NLYQFKNM(+15.99)IQC(+57.02)AGTRTWVAYLK.Y | Y | 45.66 | 2720.3567 | 22 | 2.0 | 907.7946 | 3 | 53.03 | 1 | 33500 | 01122020\_RID\_2209\_BuCaKA02.raw | 2.8614E8 | 1 | 1 | 17 | 38 | Oxidation (M); Carbamidomethylation | M8:Oxidation (M):1000.00;C11:Carbamidomethylation:1000.00 | PEAKS DB |
| R.TWVAYLKYGC(+57.02)YC(+57.02)GYGGSGTPVDELDRC(+57.02)C(+57.02)YVHDHC(+57.02)YDK.A | Y | 43.28 | 4523.8804 | 37 | 0.8 | 1131.9783 | 4 | 49.89 | 1 | 30947 | 01122020\_RID\_2209\_BuCaKA02.raw | 1.1877E8 | 1 | 1 | 32 | 68 | Carbamidomethylation | C10:Carbamidomethylation:1000.00;C12:Carbamidomethylation:1000.00;C27:Carbamidomethylation:1000.00;C28:Carbamidomethylation:1000.00;C34:Carbamidomethylation:1000.00 | PEAKS DB |
| L.KYGC(+57.02)YC(+57.02)GYGGSGTPVDELDRC(+57.02)C(+57.02)YVHDHC(+57.02)YDK.A | Y | 40.69 | 3790.5002 | 31 | 1.7 | 948.6340 | 4 | 26.20 | 1 | 12963 | 01122020\_RID\_2209\_BuCaKA02.raw | 2.9172E6 | 1 | 1 | 38 | 68 | Carbamidomethylation | C4:Carbamidomethylation:1000.00;C6:Carbamidomethylation:1000.00;C21:Carbamidomethylation:1000.00;C22:Carbamidomethylation:1000.00;C28:Carbamidomethylation:1000.00 | PEAKS DB |
| K.KIPGC(+57.02)NPIIKIYSYT.C | Y | 39.24 | 1765.9436 | 15 | 1.6 | 589.6561 | 3 | 43.73 | 1 | 26294 | 01122020\_RID\_2209\_BuCaKA02.raw | 6.8151E7 | 1 | 1 | 71 | 85 | Carbamidomethylation | C5:Carbamidomethylation:1000.00 | PEAKS DB |
| total 29 peptides |  |  |  |  |  |  |  |  |  |  |  |  |  |  |  |  |  |  |  |
| --- | --- | --- | --- | --- | --- | --- | --- | --- | --- | --- | --- | --- | --- | --- | --- | --- | --- | --- | --- |

Q8QFW4.1

back to list

  

| Protein Coverage
| Supporting Peptides
|

Protein Coverage:

Supporting Peptides:

| Peptide | Uniq | -10lgP | Mass | Length | ppm | m/z | z | RT | Fraction | Scan | Source File | Area BRom | #Feature | #Feature BRom | Start | End | PTM | AScore | Found By |
| --- | --- | --- | --- | --- | --- | --- | --- | --- | --- | --- | --- | --- | --- | --- | --- | --- | --- | --- | --- |
| R.TAALC(+57.02)FGDSEYIEGHKRIDTK.R | Y | 95.89 | 2410.1587 | 21 | 2.1 | 603.5482 | 4 | 24.09 | 1 | 11675 | 01122020\_RID\_2209\_BuCaKA02.raw | 8.885E8 | 9 | 9 | 123 | 143 | Carbamidomethylation | C5:Carbamidomethylation:1000.00 | PEAKS DB |
| R.IVC(+57.02)DC(+57.02)DRTAALC(+57.02)FGDSEYIEGHKR.I | Y | 89.24 | 2871.2737 | 24 | 1.2 | 718.8265 | 4 | 27.03 | 1 | 13592 | 01122020\_RID\_2209\_BuCaKA02.raw | 1.056E9 | 4 | 4 | 116 | 139 | Carbamidomethylation | C3:Carbamidomethylation:1000.00;C5:Carbamidomethylation:1000.00;C12:Carbamidomethylation:1000.00 | PEAKS DB |
| R.TAALC(+57.02)FGDSEYIEGHKR.I | Y | 87.19 | 1952.9050 | 17 | 1.2 | 651.9764 | 3 | 24.20 | 1 | 11332 | 01122020\_RID\_2209\_BuCaKA02.raw | 4.9017E8 | 7 | 7 | 123 | 139 | Carbamidomethylation | C5:Carbamidomethylation:1000.00 | PEAKS DB |
| R.TAALC(+57.02)FGDSEYIEGHK.R | N | 86.36 | 1796.8040 | 16 | 2.0 | 599.9431 | 3 | 35.10 | 1 | 20159 | 01122020\_RID\_2209\_BuCaKA02.raw | 1.9665E8 | 8 | 8 | 123 | 138 | Carbamidomethylation | C5:Carbamidomethylation:1000.00 | PEAKS DB |
| R.RTIIC(+57.02)YGAAGTC(+57.02)AR.I | N | 82.13 | 1568.7551 | 14 | 1.6 | 785.3861 | 2 | 12.20 | 1 | 2149 | 01122020\_RID\_2209\_BuCaKA02.raw | 1.5675E6 | 1 | 1 | 102 | 115 | Carbamidomethylation | C5:Carbamidomethylation:1000.00;C12:Carbamidomethylation:1000.00 | PEAKS DB |
| R.IVC(+57.02)DC(+57.02)DRTAALC(+57.02)FGDSEYIEGHK.R | Y | 80.93 | 2715.1726 | 23 | 0.3 | 906.0651 | 3 | 35.89 | 1 | 20501 | 01122020\_RID\_2209\_BuCaKA02.raw | 1.0104E8 | 3 | 3 | 116 | 138 | Carbamidomethylation | C3:Carbamidomethylation:1000.00;C5:Carbamidomethylation:1000.00;C12:Carbamidomethylation:1000.00 | PEAKS DB |
| K.TWGEYTNYGC(+57.02)YC(+57.02)GAGGSGTPIDALDR.C | Y | 80.49 | 2840.1807 | 26 | 0.5 | 1421.0983 | 2 | 62.62 | 1 | 41742 | 01122020\_RID\_2209\_BuCaKA02.raw | 2.6262E9 | 20 | 20 | 45 | 70 | Carbamidomethylation | C10:Carbamidomethylation:1000.00;C12:Carbamidomethylation:1000.00 | PEAKS DB |
| R.TIIC(+57.02)YGAAGTC(+57.02)AR.I | N | 74.77 | 1412.6541 | 13 | 1.2 | 707.3351 | 2 | 22.55 | 1 | 10267 | 01122020\_RID\_2209\_BuCaKA02.raw | 6.2731E7 | 1 | 1 | 103 | 115 | Carbamidomethylation | C4:Carbamidomethylation:1000.00;C11:Carbamidomethylation:1000.00 | PEAKS DB |
| I.VC(+57.02)DC(+57.02)DRTAALC(+57.02)FGDSEYIEGHKR.I | Y | 68.55 | 2758.1897 | 23 | 1.4 | 690.5557 | 4 | 24.21 | 1 | 11444 | 01122020\_RID\_2209\_BuCaKA02.raw | 1.9985E7 | 1 | 1 | 117 | 139 | Carbamidomethylation | C2:Carbamidomethylation:1000.00;C4:Carbamidomethylation:1000.00;C11:Carbamidomethylation:1000.00 | PEAKS DB |
| L.NLINFMNMIRYTIPC(+57.02)EKTWGEYTNYGC(+57.02)YC(+57.02)GAGGSGTPIDALDR.C | Y | 67.98 | 4978.2280 | 43 | 1.1 | 1245.5657 | 4 | 87.25 | 1 | 61796 | 01122020\_RID\_2209\_BuCaKA02.raw | 1.3046E8 | 1 | 1 | 28 | 70 | Carbamidomethylation | C15:Carbamidomethylation:1000.00;C27:Carbamidomethylation:1000.00;C29:Carbamidomethylation:1000.00 | PEAKS DB |
| L.NLINFMNMIRYTIPC(+57.02)EK.T | Y | 66.20 | 2156.0581 | 17 | 3.1 | 1079.0397 | 2 | 78.89 | 1 | 54989 | 01122020\_RID\_2209\_BuCaKA02.raw | 9.0085E8 | 4 | 4 | 28 | 44 | Carbamidomethylation | C15:Carbamidomethylation:1000.00 | PEAKS DB |
| P.LNLINFMNMIRYTIPC(+57.02)EK.T | Y | 64.25 | 2269.1421 | 18 | 7.9 | 757.3940 | 3 | 87.92 | 1 | 62471 | 01122020\_RID\_2209\_BuCaKA02.raw | 1.9439E7 | 2 | 2 | 27 | 44 | Carbamidomethylation | C16:Carbamidomethylation:1000.00 | PEAKS DB |
| P.LNLINFMNMIR.Y | Y | 62.24 | 1377.7261 | 11 | 4.0 | 689.8730 | 2 | 85.73 | 1 | 60674 | 01122020\_RID\_2209\_BuCaKA02.raw | 6.1255E7 | 2 | 2 | 27 | 37 |  |  | PEAKS DB |
| R.YTIPC(+57.02)EKTWGEYTNYGC(+57.02)YC(+57.02)GAGGSGTPIDALDR.C | Y | 59.82 | 3731.5967 | 33 | 1.0 | 1244.8740 | 3 | 61.76 | 1 | 40700 | 01122020\_RID\_2209\_BuCaKA02.raw | 2.336E8 | 1 | 1 | 38 | 70 | Carbamidomethylation | C5:Carbamidomethylation:1000.00;C17:Carbamidomethylation:1000.00;C19:Carbamidomethylation:1000.00 | PEAKS DB |
| R.IVC(+57.02)DC(+57.02)DRTAALC(+57.02)FG.D | N | 58.90 | 1656.7058 | 14 | 1.9 | 829.3618 | 2 | 40.45 | 1 | 24023 | 01122020\_RID\_2209\_BuCaKA02.raw | 6.5443E7 | 1 | 1 | 116 | 129 | Carbamidomethylation | C3:Carbamidomethylation:1000.00;C5:Carbamidomethylation:1000.00;C12:Carbamidomethylation:1000.00 | PEAKS DB |
| P.LNLINFMNMIRYTIPC(+57.02)EKTWGEYTNYGC(+57.02)YC(+57.02)GAGGSGTPIDALDR.C | Y | 58.41 | 5091.3120 | 44 | 1.1 | 1273.8367 | 4 | 92.87 | 1 | 66524 | 01122020\_RID\_2209\_BuCaKA02.raw | 2.7499E7 | 1 | 1 | 27 | 70 | Carbamidomethylation | C16:Carbamidomethylation:1000.00;C28:Carbamidomethylation:1000.00;C30:Carbamidomethylation:1000.00 | PEAKS DB |
| N.LINFMNMIR.Y | Y | 58.17 | 1150.5991 | 9 | 0.1 | 576.3069 | 2 | 68.89 | 1 | 46751 | 01122020\_RID\_2209\_BuCaKA02.raw | 7.3501E7 | 1 | 1 | 29 | 37 |  |  | PEAKS DB |
| L.NLINFMNMIR.Y | Y | 57.74 | 1264.6421 | 10 | -0.3 | 633.3281 | 2 | 75.88 | 1 | 52490 | 01122020\_RID\_2209\_BuCaKA02.raw | 1.7989E9 | 2 | 2 | 28 | 37 |  |  | PEAKS DB |
| T.AALC(+57.02)FGDSEYIEGHK.R | N | 51.32 | 1695.7562 | 15 | 1.9 | 566.2604 | 3 | 33.81 | 1 | 18718 | 01122020\_RID\_2209\_BuCaKA02.raw | 6.276E6 | 1 | 1 | 124 | 138 | Carbamidomethylation | C4:Carbamidomethylation:1000.00 | PEAKS DB |
| K.TWGEYTNYGC(+57.02)YC(+57.02)GAGGSGTPIDALDRC(+57.02)C(+57.02)YVHDNC(+57.02)YGDAEK.I | Y | 50.01 | 4611.8193 | 40 | 0.0 | 1153.9622 | 4 | 53.58 | 1 | 34082 | 01122020\_RID\_2209\_BuCaKA02.raw | 2.1039E8 | 1 | 1 | 45 | 84 | Carbamidomethylation | C10:Carbamidomethylation:1000.00;C12:Carbamidomethylation:1000.00;C27:Carbamidomethylation:1000.00;C28:Carbamidomethylation:1000.00;C34:Carbamidomethylation:1000.00 | PEAKS DB |
| R.TAALC(+57.02)FGDSEY.I | N | 49.80 | 1232.5020 | 11 | 0.7 | 617.2587 | 2 | 56.34 | 1 | 36497 | 01122020\_RID\_2209\_BuCaKA02.raw | 8.7681E6 | 1 | 1 | 123 | 133 | Carbamidomethylation | C5:Carbamidomethylation:1000.00 | PEAKS DB |
| T.AALC(+57.02)FGDSEYIEGHKR.I | Y | 49.65 | 1851.8573 | 16 | 0.2 | 618.2932 | 3 | 22.81 | 1 | 10318 | 01122020\_RID\_2209\_BuCaKA02.raw | 3.7417E6 | 1 | 1 | 124 | 139 | Carbamidomethylation | C4:Carbamidomethylation:1000.00 | PEAKS DB |
| R.IVC(+57.02)DC(+57.02)DRTAALC(+57.02)FGDSEY.I | N | 47.92 | 2150.8706 | 18 | -0.9 | 1076.4417 | 2 | 50.53 | 1 | 31724 | 01122020\_RID\_2209\_BuCaKA02.raw | 2.4211E8 | 2 | 2 | 116 | 133 | Carbamidomethylation | C3:Carbamidomethylation:1000.00;C5:Carbamidomethylation:1000.00;C12:Carbamidomethylation:1000.00 | PEAKS DB |
| R.TAALC(+57.02)FGDSEYIEGHKRIDT.K | Y | 46.42 | 2282.0637 | 20 | -0.2 | 761.6951 | 3 | 32.62 | 1 | 17758 | 01122020\_RID\_2209\_BuCaKA02.raw | 6.7872E6 | 1 | 1 | 123 | 142 | Carbamidomethylation | C5:Carbamidomethylation:1000.00 | PEAKS DB |
| L.NLINFMNM(+15.99)IRYTIPC(+57.02)EK.T | Y | 41.14 | 2172.0530 | 17 | 1.5 | 725.0260 | 3 | 68.69 | 1 | 46519 | 01122020\_RID\_2209\_BuCaKA02.raw | 5.1906E5 | 1 | 1 | 28 | 44 | Oxidation (M); Carbamidomethylation | M8:Oxidation (M):10.42;C15:Carbamidomethylation:1000.00 | PEAKS DB |
| total 25 peptides |  |  |  |  |  |  |  |  |  |  |  |  |  |  |  |  |  |  |  |
| --- | --- | --- | --- | --- | --- | --- | --- | --- | --- | --- | --- | --- | --- | --- | --- | --- | --- | --- | --- |

AAL87003.1

back to list

  

| Protein Coverage
| Supporting Peptides
|

Protein Coverage:

Supporting Peptides:

| Peptide | Uniq | -10lgP | Mass | Length | ppm | m/z | z | RT | Fraction | Scan | Source File | Area BRom | #Feature | #Feature BRom | Start | End | PTM | AScore | Found By |
| --- | --- | --- | --- | --- | --- | --- | --- | --- | --- | --- | --- | --- | --- | --- | --- | --- | --- | --- | --- |
| R.TAALC(+57.02)FGDSEYIEGHKRIDTK.R | Y | 95.89 | 2410.1587 | 21 | 2.1 | 603.5482 | 4 | 24.09 | 1 | 11675 | 01122020\_RID\_2209\_BuCaKA02.raw | 8.885E8 | 9 | 9 | 123 | 143 | Carbamidomethylation | C5:Carbamidomethylation:1000.00 | PEAKS DB |
| R.IVC(+57.02)DC(+57.02)DRTAALC(+57.02)FGDSEYIEGHKR.I | Y | 89.24 | 2871.2737 | 24 | 1.2 | 718.8265 | 4 | 27.03 | 1 | 13592 | 01122020\_RID\_2209\_BuCaKA02.raw | 1.056E9 | 4 | 4 | 116 | 139 | Carbamidomethylation | C3:Carbamidomethylation:1000.00;C5:Carbamidomethylation:1000.00;C12:Carbamidomethylation:1000.00 | PEAKS DB |
| R.TAALC(+57.02)FGDSEYIEGHKR.I | Y | 87.19 | 1952.9050 | 17 | 1.2 | 651.9764 | 3 | 24.20 | 1 | 11332 | 01122020\_RID\_2209\_BuCaKA02.raw | 4.9017E8 | 7 | 7 | 123 | 139 | Carbamidomethylation | C5:Carbamidomethylation:1000.00 | PEAKS DB |
| R.TAALC(+57.02)FGDSEYIEGHK.R | N | 86.36 | 1796.8040 | 16 | 2.0 | 599.9431 | 3 | 35.10 | 1 | 20159 | 01122020\_RID\_2209\_BuCaKA02.raw | 1.9665E8 | 8 | 8 | 123 | 138 | Carbamidomethylation | C5:Carbamidomethylation:1000.00 | PEAKS DB |
| R.RTIIC(+57.02)YGAAGTC(+57.02)AR.I | N | 82.13 | 1568.7551 | 14 | 1.6 | 785.3861 | 2 | 12.20 | 1 | 2149 | 01122020\_RID\_2209\_BuCaKA02.raw | 1.5675E6 | 1 | 1 | 102 | 115 | Carbamidomethylation | C5:Carbamidomethylation:1000.00;C12:Carbamidomethylation:1000.00 | PEAKS DB |
| R.IVC(+57.02)DC(+57.02)DRTAALC(+57.02)FGDSEYIEGHK.R | Y | 80.93 | 2715.1726 | 23 | 0.3 | 906.0651 | 3 | 35.89 | 1 | 20501 | 01122020\_RID\_2209\_BuCaKA02.raw | 1.0104E8 | 3 | 3 | 116 | 138 | Carbamidomethylation | C3:Carbamidomethylation:1000.00;C5:Carbamidomethylation:1000.00;C12:Carbamidomethylation:1000.00 | PEAKS DB |
| K.TWGEYTNYGC(+57.02)YC(+57.02)GAGGSGTPIDALDR.C | Y | 80.49 | 2840.1807 | 26 | 0.5 | 1421.0983 | 2 | 62.62 | 1 | 41742 | 01122020\_RID\_2209\_BuCaKA02.raw | 2.6262E9 | 20 | 20 | 45 | 70 | Carbamidomethylation | C10:Carbamidomethylation:1000.00;C12:Carbamidomethylation:1000.00 | PEAKS DB |
| R.TIIC(+57.02)YGAAGTC(+57.02)AR.I | N | 74.77 | 1412.6541 | 13 | 1.2 | 707.3351 | 2 | 22.55 | 1 | 10267 | 01122020\_RID\_2209\_BuCaKA02.raw | 6.2731E7 | 1 | 1 | 103 | 115 | Carbamidomethylation | C4:Carbamidomethylation:1000.00;C11:Carbamidomethylation:1000.00 | PEAKS DB |
| I.VC(+57.02)DC(+57.02)DRTAALC(+57.02)FGDSEYIEGHKR.I | Y | 68.55 | 2758.1897 | 23 | 1.4 | 690.5557 | 4 | 24.21 | 1 | 11444 | 01122020\_RID\_2209\_BuCaKA02.raw | 1.9985E7 | 1 | 1 | 117 | 139 | Carbamidomethylation | C2:Carbamidomethylation:1000.00;C4:Carbamidomethylation:1000.00;C11:Carbamidomethylation:1000.00 | PEAKS DB |
| L.NLINFMNMIRYTIPC(+57.02)EKTWGEYTNYGC(+57.02)YC(+57.02)GAGGSGTPIDALDR.C | Y | 67.98 | 4978.2280 | 43 | 1.1 | 1245.5657 | 4 | 87.25 | 1 | 61796 | 01122020\_RID\_2209\_BuCaKA02.raw | 1.3046E8 | 1 | 1 | 28 | 70 | Carbamidomethylation | C15:Carbamidomethylation:1000.00;C27:Carbamidomethylation:1000.00;C29:Carbamidomethylation:1000.00 | PEAKS DB |
| L.NLINFMNMIRYTIPC(+57.02)EK.T | Y | 66.20 | 2156.0581 | 17 | 3.1 | 1079.0397 | 2 | 78.89 | 1 | 54989 | 01122020\_RID\_2209\_BuCaKA02.raw | 9.0085E8 | 4 | 4 | 28 | 44 | Carbamidomethylation | C15:Carbamidomethylation:1000.00 | PEAKS DB |
| P.LNLINFMNMIRYTIPC(+57.02)EK.T | Y | 64.25 | 2269.1421 | 18 | 7.9 | 757.3940 | 3 | 87.92 | 1 | 62471 | 01122020\_RID\_2209\_BuCaKA02.raw | 1.9439E7 | 2 | 2 | 27 | 44 | Carbamidomethylation | C16:Carbamidomethylation:1000.00 | PEAKS DB |
| P.LNLINFMNMIR.Y | Y | 62.24 | 1377.7261 | 11 | 4.0 | 689.8730 | 2 | 85.73 | 1 | 60674 | 01122020\_RID\_2209\_BuCaKA02.raw | 6.1255E7 | 2 | 2 | 27 | 37 |  |  | PEAKS DB |
| R.YTIPC(+57.02)EKTWGEYTNYGC(+57.02)YC(+57.02)GAGGSGTPIDALDR.C | Y | 59.82 | 3731.5967 | 33 | 1.0 | 1244.8740 | 3 | 61.76 | 1 | 40700 | 01122020\_RID\_2209\_BuCaKA02.raw | 2.336E8 | 1 | 1 | 38 | 70 | Carbamidomethylation | C5:Carbamidomethylation:1000.00;C17:Carbamidomethylation:1000.00;C19:Carbamidomethylation:1000.00 | PEAKS DB |
| R.IVC(+57.02)DC(+57.02)DRTAALC(+57.02)FG.D | N | 58.90 | 1656.7058 | 14 | 1.9 | 829.3618 | 2 | 40.45 | 1 | 24023 | 01122020\_RID\_2209\_BuCaKA02.raw | 6.5443E7 | 1 | 1 | 116 | 129 | Carbamidomethylation | C3:Carbamidomethylation:1000.00;C5:Carbamidomethylation:1000.00;C12:Carbamidomethylation:1000.00 | PEAKS DB |
| P.LNLINFMNMIRYTIPC(+57.02)EKTWGEYTNYGC(+57.02)YC(+57.02)GAGGSGTPIDALDR.C | Y | 58.41 | 5091.3120 | 44 | 1.1 | 1273.8367 | 4 | 92.87 | 1 | 66524 | 01122020\_RID\_2209\_BuCaKA02.raw | 2.7499E7 | 1 | 1 | 27 | 70 | Carbamidomethylation | C16:Carbamidomethylation:1000.00;C28:Carbamidomethylation:1000.00;C30:Carbamidomethylation:1000.00 | PEAKS DB |
| N.LINFMNMIR.Y | Y | 58.17 | 1150.5991 | 9 | 0.1 | 576.3069 | 2 | 68.89 | 1 | 46751 | 01122020\_RID\_2209\_BuCaKA02.raw | 7.3501E7 | 1 | 1 | 29 | 37 |  |  | PEAKS DB |
| L.NLINFMNMIR.Y | Y | 57.74 | 1264.6421 | 10 | -0.3 | 633.3281 | 2 | 75.88 | 1 | 52490 | 01122020\_RID\_2209\_BuCaKA02.raw | 1.7989E9 | 2 | 2 | 28 | 37 |  |  | PEAKS DB |
| T.AALC(+57.02)FGDSEYIEGHK.R | N | 51.32 | 1695.7562 | 15 | 1.9 | 566.2604 | 3 | 33.81 | 1 | 18718 | 01122020\_RID\_2209\_BuCaKA02.raw | 6.276E6 | 1 | 1 | 124 | 138 | Carbamidomethylation | C4:Carbamidomethylation:1000.00 | PEAKS DB |
| K.TWGEYTNYGC(+57.02)YC(+57.02)GAGGSGTPIDALDRC(+57.02)C(+57.02)YVHDNC(+57.02)YGDAEK.I | Y | 50.01 | 4611.8193 | 40 | 0.0 | 1153.9622 | 4 | 53.58 | 1 | 34082 | 01122020\_RID\_2209\_BuCaKA02.raw | 2.1039E8 | 1 | 1 | 45 | 84 | Carbamidomethylation | C10:Carbamidomethylation:1000.00;C12:Carbamidomethylation:1000.00;C27:Carbamidomethylation:1000.00;C28:Carbamidomethylation:1000.00;C34:Carbamidomethylation:1000.00 | PEAKS DB |
| R.TAALC(+57.02)FGDSEY.I | N | 49.80 | 1232.5020 | 11 | 0.7 | 617.2587 | 2 | 56.34 | 1 | 36497 | 01122020\_RID\_2209\_BuCaKA02.raw | 8.7681E6 | 1 | 1 | 123 | 133 | Carbamidomethylation | C5:Carbamidomethylation:1000.00 | PEAKS DB |
| T.AALC(+57.02)FGDSEYIEGHKR.I | Y | 49.65 | 1851.8573 | 16 | 0.2 | 618.2932 | 3 | 22.81 | 1 | 10318 | 01122020\_RID\_2209\_BuCaKA02.raw | 3.7417E6 | 1 | 1 | 124 | 139 | Carbamidomethylation | C4:Carbamidomethylation:1000.00 | PEAKS DB |
| R.IVC(+57.02)DC(+57.02)DRTAALC(+57.02)FGDSEY.I | N | 47.92 | 2150.8706 | 18 | -0.9 | 1076.4417 | 2 | 50.53 | 1 | 31724 | 01122020\_RID\_2209\_BuCaKA02.raw | 2.4211E8 | 2 | 2 | 116 | 133 | Carbamidomethylation | C3:Carbamidomethylation:1000.00;C5:Carbamidomethylation:1000.00;C12:Carbamidomethylation:1000.00 | PEAKS DB |
| R.TAALC(+57.02)FGDSEYIEGHKRIDT.K | Y | 46.42 | 2282.0637 | 20 | -0.2 | 761.6951 | 3 | 32.62 | 1 | 17758 | 01122020\_RID\_2209\_BuCaKA02.raw | 6.7872E6 | 1 | 1 | 123 | 142 | Carbamidomethylation | C5:Carbamidomethylation:1000.00 | PEAKS DB |
| L.NLINFMNM(+15.99)IRYTIPC(+57.02)EK.T | Y | 41.14 | 2172.0530 | 17 | 1.5 | 725.0260 | 3 | 68.69 | 1 | 46519 | 01122020\_RID\_2209\_BuCaKA02.raw | 5.1906E5 | 1 | 1 | 28 | 44 | Oxidation (M); Carbamidomethylation | M8:Oxidation (M):10.42;C15:Carbamidomethylation:1000.00 | PEAKS DB |
| total 25 peptides |  |  |  |  |  |  |  |  |  |  |  |  |  |  |  |  |  |  |  |
| --- | --- | --- | --- | --- | --- | --- | --- | --- | --- | --- | --- | --- | --- | --- | --- | --- | --- | --- | --- |

T\_148

back to list

  

| Protein Coverage
| Supporting Peptides
|

Protein Coverage:

Supporting Peptides:

| Peptide | Uniq | -10lgP | Mass | Length | ppm | m/z | z | RT | Fraction | Scan | Source File | Area BRom | #Feature | #Feature BRom | Start | End | PTM | AScore | Found By |
| --- | --- | --- | --- | --- | --- | --- | --- | --- | --- | --- | --- | --- | --- | --- | --- | --- | --- | --- | --- |
| K.C(+57.02)GVSGC(+57.02)HLKITC(+57.02)SAEETFC(+57.02)YK.W | Y | 97.96 | 2506.0750 | 21 | 0.3 | 836.3658 | 3 | 26.68 | 1 | 13245 | 01122020\_RID\_2209\_BuCaKA02.raw | 7.5825E8 | 20 | 20 | 87 | 107 | Carbamidomethylation | C1:Carbamidomethylation:1000.00;C6:Carbamidomethylation:1000.00;C12:Carbamidomethylation:1000.00;C19:Carbamidomethylation:1000.00 | PEAKS DB |
| K.ITC(+57.02)SAEETFC(+57.02)YKWLNKISNER.W | Y | 91.10 | 2648.2363 | 21 | 0.8 | 883.7534 | 3 | 44.56 | 1 | 26919 | 01122020\_RID\_2209\_BuCaKA02.raw | 1.8933E9 | 4 | 4 | 96 | 116 | Carbamidomethylation | C3:Carbamidomethylation:1000.00;C10:Carbamidomethylation:1000.00 | PEAKS DB |
| K.ITC(+57.02)SAEETFC(+57.02)YKWLNK.I | Y | 86.32 | 2048.9336 | 16 | 0.9 | 1025.4750 | 2 | 41.55 | 1 | 24827 | 01122020\_RID\_2209\_BuCaKA02.raw | 1.6905E9 | 5 | 5 | 96 | 111 | Carbamidomethylation | C3:Carbamidomethylation:1000.00;C10:Carbamidomethylation:1000.00 | PEAKS DB |
| K.ITC(+57.02)SAEETFC(+57.02)YK.W | Y | 80.38 | 1507.6323 | 12 | 1.5 | 754.8246 | 2 | 29.61 | 1 | 17051 | 01122020\_RID\_2209\_BuCaKA02.raw | 3.931E9 | 12 | 12 | 96 | 107 | Carbamidomethylation | C3:Carbamidomethylation:1000.00;C10:Carbamidomethylation:1000.00 | PEAKS DB |
| V.SGC(+57.02)HLKITC(+57.02)SAEETFC(+57.02)YK.W | Y | 75.61 | 2189.9543 | 18 | 2.0 | 730.9935 | 3 | 22.02 | 1 | 10045 | 01122020\_RID\_2209\_BuCaKA02.raw | 8.0458E7 | 1 | 1 | 90 | 107 | Carbamidomethylation | C3:Carbamidomethylation:1000.00;C9:Carbamidomethylation:1000.00;C16:Carbamidomethylation:1000.00 | PEAKS DB |
| K.C(+57.02)GVSGC(+57.02)HLKITC(+57.02)SAEETFC(+57.02)YKWLNK.I | Y | 72.94 | 3047.3762 | 25 | 1.9 | 762.8528 | 4 | 37.39 | 1 | 21834 | 01122020\_RID\_2209\_BuCaKA02.raw | 2.6744E8 | 7 | 7 | 87 | 111 | Carbamidomethylation | C1:Carbamidomethylation:1000.00;C6:Carbamidomethylation:1000.00;C12:Carbamidomethylation:1000.00;C19:Carbamidomethylation:1000.00 | PEAKS DB |
| C.SAEETFC(+57.02)YKWLNK.I | Y | 72.89 | 1674.7711 | 13 | 0.9 | 838.3936 | 2 | 36.08 | 1 | 20755 | 01122020\_RID\_2209\_BuCaKA02.raw | 6.994E7 | 2 | 2 | 99 | 111 | Carbamidomethylation | C7:Carbamidomethylation:1000.00 | PEAKS DB |
| I.TC(+57.02)SAEETFC(+57.02)YKWLNK.I | Y | 72.52 | 1935.8495 | 15 | 1.7 | 646.2916 | 3 | 37.85 | 1 | 22124 | 01122020\_RID\_2209\_BuCaKA02.raw | 6.5668E8 | 1 | 1 | 97 | 111 | Carbamidomethylation | C2:Carbamidomethylation:1000.00;C9:Carbamidomethylation:1000.00 | PEAKS DB |
| I.TC(+57.02)SAEETFC(+57.02)YK.W | Y | 68.26 | 1394.5482 | 11 | 1.6 | 698.2825 | 2 | 20.91 | 1 | 8803 | 01122020\_RID\_2209\_BuCaKA02.raw | 5.1144E8 | 3 | 3 | 97 | 107 | Carbamidomethylation | C2:Carbamidomethylation:1000.00;C9:Carbamidomethylation:1000.00 | PEAKS DB |
| L.KITC(+57.02)SAEETFC(+57.02)YK.W | Y | 66.22 | 1635.7273 | 13 | 1.9 | 818.8724 | 2 | 16.85 | 1 | 5761 | 01122020\_RID\_2209\_BuCaKA02.raw | 9.1507E5 | 1 | 1 | 95 | 107 | Carbamidomethylation | C4:Carbamidomethylation:1000.00;C11:Carbamidomethylation:1000.00 | PEAKS DB |
| S.AEETFC(+57.02)YKWLNK.I | Y | 65.79 | 1587.7391 | 12 | 1.1 | 794.8777 | 2 | 34.63 | 1 | 19380 | 01122020\_RID\_2209\_BuCaKA02.raw | 7.9532E6 | 1 | 1 | 100 | 111 | Carbamidomethylation | C6:Carbamidomethylation:1000.00 | PEAKS DB |
| V.SGC(+57.02)HLKITC(+57.02)SAEETFC(+57.02)YKWLNK.I | Y | 63.54 | 2731.2556 | 22 | 1.0 | 683.8218 | 4 | 34.60 | 1 | 19346 | 01122020\_RID\_2209\_BuCaKA02.raw | 2.9994E7 | 2 | 2 | 90 | 111 | Carbamidomethylation | C3:Carbamidomethylation:1000.00;C9:Carbamidomethylation:1000.00;C16:Carbamidomethylation:1000.00 | PEAKS DB |
| K.DTWNVYNKC(+57.02)C(+57.02)TTNLC(+57.02)NT | Y | 62.80 | 2162.8821 | 17 | 0.8 | 1082.4492 | 2 | 40.48 | 1 | 24035 | 01122020\_RID\_2209\_BuCaKA02.raw | 5.4998E7 | 1 | 1 | 128 | 144 | Carbamidomethylation | C9:Carbamidomethylation:1000.00;C10:Carbamidomethylation:1000.00;C15:Carbamidomethylation:1000.00 | PEAKS DB |
| T.C(+57.02)SAEETFC(+57.02)YK.W | Y | 61.68 | 1293.5006 | 10 | 0.2 | 647.7577 | 2 | 19.26 | 1 | 7652 | 01122020\_RID\_2209\_BuCaKA02.raw | 7.591E7 | 1 | 1 | 98 | 107 | Carbamidomethylation | C1:Carbamidomethylation:1000.00;C8:Carbamidomethylation:1000.00 | PEAKS DB |
| K.TC(+57.02)TEKDTWNVYNK.C | Y | 61.64 | 1657.7406 | 13 | 1.3 | 829.8787 | 2 | 12.90 | 1 | 2674 | 01122020\_RID\_2209\_BuCaKA02.raw | 1.3091E5 | 1 | 1 | 123 | 135 | Carbamidomethylation | C2:Carbamidomethylation:1000.00 | PEAKS DB |
| Y.KC(+57.02)GVSGC(+57.02)HLKITC(+57.02)SAEETFC(+57.02)YK.W | Y | 61.19 | 2634.1699 | 22 | 1.0 | 659.5504 | 4 | 19.26 | 1 | 7623 | 01122020\_RID\_2209\_BuCaKA02.raw | 7.3883E6 | 2 | 2 | 86 | 107 | Carbamidomethylation | C2:Carbamidomethylation:1000.00;C7:Carbamidomethylation:1000.00;C13:Carbamidomethylation:1000.00;C20:Carbamidomethylation:1000.00 | PEAKS DB |
| G.VSGC(+57.02)HLKITC(+57.02)SAEETFC(+57.02)YKWLNK.I | Y | 59.80 | 2830.3240 | 23 | 0.8 | 708.5888 | 4 | 35.65 | 1 | 20294 | 01122020\_RID\_2209\_BuCaKA02.raw | 5.1954E6 | 1 | 1 | 89 | 111 | Carbamidomethylation | C4:Carbamidomethylation:1000.00;C10:Carbamidomethylation:1000.00;C17:Carbamidomethylation:1000.00 | PEAKS DB |
| T.C(+57.02)SAEETFC(+57.02)YKWLNK.I | Y | 59.08 | 1834.8018 | 14 | 0.3 | 612.6080 | 3 | 37.84 | 1 | 21964 | 01122020\_RID\_2209\_BuCaKA02.raw | 3.5177E7 | 1 | 1 | 98 | 111 | Carbamidomethylation | C1:Carbamidomethylation:1000.00;C8:Carbamidomethylation:1000.00 | PEAKS DB |
| C.SAEETFC(+57.02)YK.W | Y | 54.77 | 1133.4698 | 9 | 2.3 | 567.7435 | 2 | 17.03 | 1 | 5898 | 01122020\_RID\_2209\_BuCaKA02.raw | 2.3793E6 | 1 | 1 | 99 | 107 | Carbamidomethylation | C7:Carbamidomethylation:1000.00 | PEAKS DB |
| A.EETFC(+57.02)YKWLNK.I | Y | 50.81 | 1516.7020 | 11 | 2.7 | 759.3604 | 2 | 35.18 | 1 | 19885 | 01122020\_RID\_2209\_BuCaKA02.raw | 4.1897E6 | 1 | 1 | 101 | 111 | Carbamidomethylation | C5:Carbamidomethylation:1000.00 | PEAKS DB |
| K.C(+57.02)YKC(+57.02)GVSGC(+57.02)HLKITC(+57.02)SAEETFC(+57.02)YK.W | Y | 49.85 | 2957.2639 | 24 | 0.9 | 740.3239 | 4 | 22.90 | 1 | 10592 | 01122020\_RID\_2209\_BuCaKA02.raw | 5.7982E6 | 1 | 1 | 84 | 107 | Carbamidomethylation | C1:Carbamidomethylation:1000.00;C4:Carbamidomethylation:1000.00;C9:Carbamidomethylation:1000.00;C15:Carbamidomethylation:1000.00;C22:Carbamidomethylation:1000.00 | PEAKS DB |
| K.TC(+57.02)TEKDTWNVYNKC(+57.02)C(+57.02)TTNLC(+57.02)NT | Y | 44.84 | 2782.1455 | 22 | 0.7 | 1392.0811 | 2 | 26.74 | 1 | 13356 | 01122020\_RID\_2209\_BuCaKA02.raw | 4.069E7 | 1 | 1 | 123 | 144 | Carbamidomethylation | C2:Carbamidomethylation:1000.00;C14:Carbamidomethylation:1000.00;C15:Carbamidomethylation:1000.00;C20:Carbamidomethylation:1000.00 | PEAKS DB |
| G.VSGC(+57.02)HLKITC(+57.02)SAEETFC(+57.02)YK.W | Y | 40.93 | 2289.0227 | 19 | 2.2 | 573.2642 | 4 | 23.61 | 1 | 10974 | 01122020\_RID\_2209\_BuCaKA02.raw | 6.558E6 | 1 | 1 | 89 | 107 | Carbamidomethylation | C4:Carbamidomethylation:1000.00;C10:Carbamidomethylation:1000.00;C17:Carbamidomethylation:1000.00 | PEAKS DB |
| total 23 peptides |  |  |  |  |  |  |  |  |  |  |  |  |  |  |  |  |  |  |  |
| --- | --- | --- | --- | --- | --- | --- | --- | --- | --- | --- | --- | --- | --- | --- | --- | --- | --- | --- | --- |

ABG90492.1

back to list

  

| Protein Coverage
| Supporting Peptides
|

Protein Coverage:

Supporting Peptides:

| Peptide | Uniq | -10lgP | Mass | Length | ppm | m/z | z | RT | Fraction | Scan | Source File | Area BRom | #Feature | #Feature BRom | Start | End | PTM | AScore | Found By |
| --- | --- | --- | --- | --- | --- | --- | --- | --- | --- | --- | --- | --- | --- | --- | --- | --- | --- | --- | --- |
| R.IVC(+57.02)DC(+57.02)DRTAALC(+57.02)FGDSEYIER.H | N | 95.72 | 2549.0984 | 21 | 0.6 | 850.7073 | 3 | 45.80 | 1 | 27548 | 01122020\_RID\_2209\_BuCaKA02.raw | 2.6721E9 | 24 | 24 | 87 | 107 | Carbamidomethylation | C3:Carbamidomethylation:1000.00;C5:Carbamidomethylation:1000.00;C12:Carbamidomethylation:1000.00 | PEAKS DB |
| R.TAALC(+57.02)FGDSEYIER.H | N | 82.91 | 1630.7297 | 14 | 1.0 | 816.3729 | 2 | 48.43 | 1 | 32715 | 01122020\_RID\_2209\_BuCaKA02.raw | 4.766E9 | 42 | 42 | 94 | 107 | Carbamidomethylation | C5:Carbamidomethylation:1000.00 | PEAKS DB |
| R.TAALC(+57.02)FGDSEYIERHKNIDTK.R | Y | 79.76 | 2467.1802 | 21 | 2.0 | 617.8035 | 4 | 23.54 | 1 | 10850 | 01122020\_RID\_2209\_BuCaKA02.raw | 2.4407E8 | 3 | 3 | 94 | 114 | Carbamidomethylation | C5:Carbamidomethylation:1000.00 | PEAKS DB |
| R.TAALC(+57.02)FGDSEYIERHK.N | N | 78.30 | 1895.8835 | 16 | 1.0 | 632.9691 | 3 | 24.86 | 1 | 11918 | 01122020\_RID\_2209\_BuCaKA02.raw | 1.8408E7 | 1 | 1 | 94 | 109 | Carbamidomethylation | C5:Carbamidomethylation:1000.00 | PEAKS DB |
| K.RTIIC(+57.02)YGAAGTC(+57.02)GR.I | N | 78.18 | 1554.7395 | 14 | 1.6 | 778.3783 | 2 | 12.18 | 1 | 2117 | 01122020\_RID\_2209\_BuCaKA02.raw | 5.9802E6 | 1 | 1 | 73 | 86 | Carbamidomethylation | C5:Carbamidomethylation:1000.00;C12:Carbamidomethylation:1000.00 | PEAKS DB |
| Y.GC(+57.02)YC(+57.02)GAGGSGTPVDALDR.C | N | 76.28 | 1811.7566 | 18 | 1.3 | 906.8867 | 2 | 29.21 | 1 | 15083 | 01122020\_RID\_2209\_BuCaKA02.raw | 5.769E6 | 1 | 1 | 24 | 41 | Carbamidomethylation | C2:Carbamidomethylation:1000.00;C4:Carbamidomethylation:1000.00 | PEAKS DB |
| N.YGC(+57.02)YC(+57.02)GAGGSGTPVDALDR.C | N | 75.35 | 1974.8199 | 19 | 1.8 | 988.4191 | 2 | 35.54 | 1 | 20103 | 01122020\_RID\_2209\_BuCaKA02.raw | 4.3649E7 | 1 | 1 | 23 | 41 | Carbamidomethylation | C3:Carbamidomethylation:1000.00;C5:Carbamidomethylation:1000.00 | PEAKS DB |
| I.VC(+57.02)DC(+57.02)DRTAALC(+57.02)FGDSEYIER.H | N | 74.30 | 2436.0144 | 20 | 1.1 | 813.0129 | 3 | 44.94 | 1 | 27302 | 01122020\_RID\_2209\_BuCaKA02.raw | 4.4364E7 | 5 | 5 | 88 | 107 | Carbamidomethylation | C2:Carbamidomethylation:1000.00;C4:Carbamidomethylation:1000.00;C11:Carbamidomethylation:1000.00 | PEAKS DB |
| T.AALC(+57.02)FGDSEYIER.H | N | 74.12 | 1529.6820 | 13 | 1.4 | 765.8494 | 2 | 45.78 | 1 | 27956 | 01122020\_RID\_2209\_BuCaKA02.raw | 1.7529E8 | 1 | 1 | 95 | 107 | Carbamidomethylation | C4:Carbamidomethylation:1000.00 | PEAKS DB |
| R.TIIC(+57.02)YGAAGTC(+57.02)GR.I | N | 70.35 | 1398.6384 | 13 | 1.6 | 700.3276 | 2 | 22.35 | 1 | 11385 | 01122020\_RID\_2209\_BuCaKA02.raw | 4.4986E8 | 7 | 7 | 74 | 86 | Carbamidomethylation | C4:Carbamidomethylation:1000.00;C11:Carbamidomethylation:1000.00 | PEAKS DB |
| R.TIIC(+57.02)YGAAGTC(+57.02)GRIVC(+57.02)DC(+57.02)DR.T | N | 70.23 | 2317.0071 | 20 | 0.8 | 1159.5117 | 2 | 29.20 | 1 | 15032 | 01122020\_RID\_2209\_BuCaKA02.raw | 1.3733E8 | 2 | 2 | 74 | 93 | Carbamidomethylation | C4:Carbamidomethylation:1000.00;C11:Carbamidomethylation:1000.00;C16:Carbamidomethylation:1000.00;C18:Carbamidomethylation:1000.00 | PEAKS DB |
| A.ALC(+57.02)FGDSEYIER.H | N | 69.32 | 1458.6449 | 12 | 1.2 | 730.3306 | 2 | 42.66 | 1 | 25737 | 01122020\_RID\_2209\_BuCaKA02.raw | 6.9107E8 | 2 | 2 | 96 | 107 | Carbamidomethylation | C3:Carbamidomethylation:1000.00 | PEAKS DB |
| A.LC(+57.02)FGDSEYIER.H | N | 67.09 | 1387.6078 | 11 | 0.4 | 694.8115 | 2 | 40.28 | 1 | 24144 | 01122020\_RID\_2209\_BuCaKA02.raw | 2.7802E8 | 1 | 1 | 97 | 107 | Carbamidomethylation | C2:Carbamidomethylation:1000.00 | PEAKS DB |
| R.TAALC(+57.02)FGDSEYIERH.K | N | 65.19 | 1767.7886 | 15 | 1.2 | 590.2708 | 3 | 36.47 | 1 | 21210 | 01122020\_RID\_2209\_BuCaKA02.raw | 1.8861E7 | 6 | 6 | 94 | 108 | Carbamidomethylation | C5:Carbamidomethylation:1000.00 | PEAKS DB |
| R.IVC(+57.02)DC(+57.02)DRTAALC(+57.02)FG.D | N | 58.90 | 1656.7058 | 14 | 1.9 | 829.3618 | 2 | 40.45 | 1 | 24023 | 01122020\_RID\_2209\_BuCaKA02.raw | 6.5443E7 | 1 | 1 | 87 | 100 | Carbamidomethylation | C3:Carbamidomethylation:1000.00;C5:Carbamidomethylation:1000.00;C12:Carbamidomethylation:1000.00 | PEAKS DB |
| R.IVC(+57.02)DC(+57.02)DRTAALC(+57.02)FGDSEYIERHK.N | N | 57.48 | 2814.2524 | 23 | 0.0 | 704.5704 | 4 | 28.24 | 1 | 14581 | 01122020\_RID\_2209\_BuCaKA02.raw | 0 | 0 | 0 | 87 | 109 | Carbamidomethylation | C3:Carbamidomethylation:1000.00;C5:Carbamidomethylation:1000.00;C12:Carbamidomethylation:1000.00 | PEAKS DB |
| R.IVC(+57.02)DC(+57.02)DRTAALC(+57.02)FGDSEYIERH.K | N | 56.25 | 2686.1575 | 22 | 0.1 | 896.3932 | 3 | 36.81 | 1 | 21217 | 01122020\_RID\_2209\_BuCaKA02.raw | 1.8955E7 | 3 | 3 | 87 | 108 | Carbamidomethylation | C3:Carbamidomethylation:1000.00;C5:Carbamidomethylation:1000.00;C12:Carbamidomethylation:1000.00 | PEAKS DB |
| R.TAALC(+57.02)FGDSEY.I | N | 49.80 | 1232.5020 | 11 | 0.7 | 617.2587 | 2 | 56.34 | 1 | 36497 | 01122020\_RID\_2209\_BuCaKA02.raw | 8.7681E6 | 1 | 1 | 94 | 104 | Carbamidomethylation | C5:Carbamidomethylation:1000.00 | PEAKS DB |
| K.RTIIC(+57.02)YGAAGTC(+57.02)GRIVC(+57.02)DC(+57.02)DR.T | N | 49.55 | 2473.1084 | 21 | 2.0 | 825.3784 | 3 | 21.10 | 1 | 8964 | 01122020\_RID\_2209\_BuCaKA02.raw | 0 | 0 | 0 | 73 | 93 | Carbamidomethylation | C5:Carbamidomethylation:1000.00;C12:Carbamidomethylation:1000.00;C17:Carbamidomethylation:1000.00;C19:Carbamidomethylation:1000.00 | PEAKS DB |
| D.RTAALC(+57.02)FGDSEYIER.H | N | 48.72 | 1786.8308 | 15 | 1.1 | 596.6182 | 3 | 37.20 | 1 | 21533 | 01122020\_RID\_2209\_BuCaKA02.raw | 3.313E6 | 1 | 1 | 93 | 107 | Carbamidomethylation | C6:Carbamidomethylation:1000.00 | PEAKS DB |
| R.IVC(+57.02)DC(+57.02)DRTAALC(+57.02)FGDSEY.I | N | 47.92 | 2150.8706 | 18 | -0.9 | 1076.4417 | 2 | 50.53 | 1 | 31724 | 01122020\_RID\_2209\_BuCaKA02.raw | 2.4211E8 | 2 | 2 | 87 | 104 | Carbamidomethylation | C3:Carbamidomethylation:1000.00;C5:Carbamidomethylation:1000.00;C12:Carbamidomethylation:1000.00 | PEAKS DB |
| total 21 peptides |  |  |  |  |  |  |  |  |  |  |  |  |  |  |  |  |  |  |  |
| --- | --- | --- | --- | --- | --- | --- | --- | --- | --- | --- | --- | --- | --- | --- | --- | --- | --- | --- | --- |

T\_26

back to list

  

| Protein Coverage
| Supporting Peptides
|

Protein Coverage:

Supporting Peptides:

| Peptide | Uniq | -10lgP | Mass | Length | ppm | m/z | z | RT | Fraction | Scan | Source File | Area BRom | #Feature | #Feature BRom | Start | End | PTM | AScore | Found By |
| --- | --- | --- | --- | --- | --- | --- | --- | --- | --- | --- | --- | --- | --- | --- | --- | --- | --- | --- | --- |
| L.KYGC(+57.02)YC(+57.02)GYGGTGTPVDELDR.C | N | 88.44 | 2266.9624 | 20 | -2.4 | 1134.4857 | 2 | 32.64 | 1 | 17782 | 01122020\_RID\_2209\_BuCaKA02.raw | 3.5219E7 | 2 | 2 | 77 | 96 | Carbamidomethylation | C4:Carbamidomethylation:1000.00;C6:Carbamidomethylation:1000.00 | PEAKS DB |
| K.YGC(+57.02)YC(+57.02)GYGGTGTPVDELDR.C | N | 88.01 | 2138.8674 | 19 | 0.4 | 1070.4414 | 2 | 44.33 | 1 | 26645 | 01122020\_RID\_2209\_BuCaKA02.raw | 9.6633E9 | 3 | 3 | 78 | 96 | Carbamidomethylation | C3:Carbamidomethylation:1000.00;C5:Carbamidomethylation:1000.00 | PEAKS DB |
| Y.GC(+57.02)YC(+57.02)GYGGTGTPVDELDR.C | N | 85.01 | 1975.8040 | 18 | 0.4 | 988.9097 | 2 | 38.86 | 1 | 23078 | 01122020\_RID\_2209\_BuCaKA02.raw | 3.3628E9 | 2 | 2 | 79 | 96 | Carbamidomethylation | C2:Carbamidomethylation:1000.00;C4:Carbamidomethylation:1000.00 | PEAKS DB |
| K.NMIQC(+57.02)AGTRTWVAYLK.Y | N | 82.79 | 1910.9495 | 16 | 1.4 | 956.4833 | 2 | 45.28 | 1 | 27489 | 01122020\_RID\_2209\_BuCaKA02.raw | 2.818E9 | 2 | 2 | 62 | 77 | Carbamidomethylation | C5:Carbamidomethylation:1000.00 | PEAKS DB |
| Y.C(+57.02)GYGGTGTPVDELDR.C | N | 82.21 | 1595.6886 | 15 | 1.6 | 798.8528 | 2 | 33.04 | 1 | 18122 | 01122020\_RID\_2209\_BuCaKA02.raw | 5.3035E8 | 3 | 3 | 82 | 96 | Carbamidomethylation | C1:Carbamidomethylation:1000.00 | PEAKS DB |
| C.YC(+57.02)GYGGTGTPVDELDR.C | N | 81.92 | 1758.7518 | 16 | 0.3 | 880.3834 | 2 | 37.99 | 1 | 22078 | 01122020\_RID\_2209\_BuCaKA02.raw | 4.9681E7 | 1 | 1 | 81 | 96 | Carbamidomethylation | C2:Carbamidomethylation:1000.00 | PEAKS DB |
| G.C(+57.02)YC(+57.02)GYGGTGTPVDELDR.C | N | 77.93 | 1918.7826 | 17 | 1.1 | 960.3996 | 2 | 38.92 | 1 | 22863 | 01122020\_RID\_2209\_BuCaKA02.raw | 7.0014E7 | 2 | 2 | 80 | 96 | Carbamidomethylation | C1:Carbamidomethylation:1000.00;C3:Carbamidomethylation:1000.00 | PEAKS DB |
| L.NLYQLKNMIQC(+57.02)AGTR.T | Y | 73.00 | 1808.9026 | 15 | 0.1 | 905.4586 | 2 | 40.35 | 1 | 23983 | 01122020\_RID\_2209\_BuCaKA02.raw | 1.0949E9 | 2 | 2 | 56 | 70 | Carbamidomethylation | C11:Carbamidomethylation:1000.00 | PEAKS DB |
| C.GYGGTGTPVDELDR.C | N | 70.03 | 1435.6580 | 14 | 1.4 | 718.8373 | 2 | 31.57 | 1 | 17094 | 01122020\_RID\_2209\_BuCaKA02.raw | 9.8214E7 | 1 | 1 | 83 | 96 |  |  | PEAKS DB |
| Y.LKYGC(+57.02)YC(+57.02)GYGGTGTPVDELDR.C | N | 69.19 | 2380.0464 | 21 | 0.9 | 794.3568 | 3 | 38.32 | 1 | 22508 | 01122020\_RID\_2209\_BuCaKA02.raw | 1.3586E8 | 1 | 1 | 76 | 96 | Carbamidomethylation | C5:Carbamidomethylation:1000.00;C7:Carbamidomethylation:1000.00 | PEAKS DB |
| G.YGGTGTPVDELDR.C | N | 68.14 | 1378.6365 | 13 | 1.7 | 690.3267 | 2 | 30.08 | 1 | 15917 | 01122020\_RID\_2209\_BuCaKA02.raw | 1.3749E8 | 1 | 1 | 84 | 96 |  |  | PEAKS DB |
| K.YGC(+57.02)YC(+57.02)GYGGTGTPVDELDRC(+57.02)C(+57.02).Y | N | 63.90 | 2458.9287 | 21 | 1.8 | 1230.4739 | 2 | 48.28 | 1 | 29764 | 01122020\_RID\_2209\_BuCaKA02.raw | 8.9471E6 | 1 | 1 | 78 | 98 | Carbamidomethylation | C3:Carbamidomethylation:1000.00;C5:Carbamidomethylation:1000.00;C20:Carbamidomethylation:1000.00;C21:Carbamidomethylation:1000.00 | PEAKS DB |
| K.YGC(+57.02)YC(+57.02)GYGGTGTPVDELDRC(+57.02)C(+57.02)YVHDNC(+57.02)YGDAEK.I | N | 59.99 | 3910.5061 | 33 | 1.2 | 978.6350 | 4 | 40.50 | 1 | 24038 | 01122020\_RID\_2209\_BuCaKA02.raw | 6.1915E7 | 1 | 1 | 78 | 110 | Carbamidomethylation | C3:Carbamidomethylation:1000.00;C5:Carbamidomethylation:1000.00;C20:Carbamidomethylation:1000.00;C21:Carbamidomethylation:1000.00;C27:Carbamidomethylation:1000.00 | PEAKS DB |
| A.YLKYGC(+57.02)YC(+57.02)GYGGTGTPVDELDR.C | N | 57.42 | 2543.1096 | 22 | 0.4 | 848.7108 | 3 | 42.36 | 1 | 25537 | 01122020\_RID\_2209\_BuCaKA02.raw | 4.2549E7 | 1 | 1 | 75 | 96 | Carbamidomethylation | C6:Carbamidomethylation:1000.00;C8:Carbamidomethylation:1000.00 | PEAKS DB |
| Q.C(+57.02)AGTRTWVAYLKYGC(+57.02)YC(+57.02)GYGGTGTPVDELDR.C | Y | 56.62 | 3545.5803 | 31 | -7.7 | 1182.8583 | 3 | 44.18 | 1 | 26637 | 01122020\_RID\_2209\_BuCaKA02.raw | 3.4648E8 | 1 | 1 | 66 | 96 | Carbamidomethylation | C1:Carbamidomethylation:1000.00;C15:Carbamidomethylation:1000.00;C17:Carbamidomethylation:1000.00 | PEAKS DB |
| N.MIQC(+57.02)AGTRTWVAYLK.Y | N | 51.37 | 1796.9066 | 15 | 0.7 | 599.9766 | 3 | 39.91 | 1 | 23605 | 01122020\_RID\_2209\_BuCaKA02.raw | 2.6769E7 | 1 | 1 | 63 | 77 | Carbamidomethylation | C4:Carbamidomethylation:1000.00 | PEAKS DB |
| L.NLYQLKNM(+15.99)IQC(+57.02)AGTR.T | Y | 50.36 | 1824.8975 | 15 | 1.9 | 913.4578 | 2 | 26.50 | 1 | 13225 | 01122020\_RID\_2209\_BuCaKA02.raw | 1.0085E7 | 3 | 3 | 56 | 70 | Oxidation (M); Carbamidomethylation | M8:Oxidation (M):1000.00;C11:Carbamidomethylation:1000.00 | PEAKS DB |
| Y.GGTGTPVDELDR.C | N | 44.26 | 1215.5731 | 12 | 0.2 | 608.7939 | 2 | 24.73 | 1 | 11888 | 01122020\_RID\_2209\_BuCaKA02.raw | 2.8666E5 | 1 | 1 | 85 | 96 |  |  | PEAKS DB |
| total 18 peptides |  |  |  |  |  |  |  |  |  |  |  |  |  |  |  |  |  |  |  |
| --- | --- | --- | --- | --- | --- | --- | --- | --- | --- | --- | --- | --- | --- | --- | --- | --- | --- | --- | --- |

T\_1

back to list

  

| Protein Coverage
| Supporting Peptides
|

Protein Coverage:

Supporting Peptides:

| Peptide | Uniq | -10lgP | Mass | Length | ppm | m/z | z | RT | Fraction | Scan | Source File | Area BRom | #Feature | #Feature BRom | Start | End | PTM | AScore | Found By |
| --- | --- | --- | --- | --- | --- | --- | --- | --- | --- | --- | --- | --- | --- | --- | --- | --- | --- | --- | --- |
| K.RLGVTILC(+57.02)C(+57.02)TTDNC(+57.02)NR | Y | 91.79 | 1951.9027 | 16 | 0.5 | 976.9591 | 2 | 26.94 | 1 | 13450 | 01122020\_RID\_2209\_BuCaKA02.raw | 8.8567E9 | 19 | 19 | 74 | 89 | Carbamidomethylation | C8:Carbamidomethylation:1000.00;C9:Carbamidomethylation:1000.00;C14:Carbamidomethylation:1000.00 | PEAKS DB |
| R.EC(+57.02)AATC(+57.02)PPKRLGVTILC(+57.02)C(+57.02)TTDNC(+57.02)NR | Y | 85.25 | 2966.3289 | 25 | 0.7 | 989.7843 | 3 | 28.71 | 1 | 14858 | 01122020\_RID\_2209\_BuCaKA02.raw | 5.5167E10 | 15 | 15 | 65 | 89 | Carbamidomethylation | C2:Carbamidomethylation:1000.00;C6:Carbamidomethylation:1000.00;C17:Carbamidomethylation:1000.00;C18:Carbamidomethylation:1000.00;C23:Carbamidomethylation:1000.00 | PEAKS DB |
| R.LGVTILC(+57.02)C(+57.02)TTDNC(+57.02)NR | Y | 84.71 | 1795.8015 | 15 | 0.7 | 898.9087 | 2 | 41.35 | 1 | 24790 | 01122020\_RID\_2209\_BuCaKA02.raw | 3.7267E10 | 18 | 18 | 75 | 89 | Carbamidomethylation | C7:Carbamidomethylation:1000.00;C8:Carbamidomethylation:1000.00;C13:Carbamidomethylation:1000.00 | PEAKS DB |
| L.TC(+57.02)ETC(+57.02)RFYTC(+57.02)PNSETC(+57.02)PDGKNIC(+57.02)VK.L | Y | 82.26 | 3096.2869 | 25 | 1.2 | 775.0800 | 4 | 20.11 | 1 | 8332 | 01122020\_RID\_2209\_BuCaKA02.raw | 1.5911E8 | 2 | 2 | 23 | 47 | Carbamidomethylation | C2:Carbamidomethylation:1000.00;C5:Carbamidomethylation:1000.00;C10:Carbamidomethylation:1000.00;C16:Carbamidomethylation:1000.00;C23:Carbamidomethylation:1000.00 | PEAKS DB |
| R.EC(+57.02)AATC(+57.02)PPKRLGVTIL.C | Y | 72.50 | 1784.9277 | 16 | 1.1 | 893.4721 | 2 | 34.19 | 1 | 19114 | 01122020\_RID\_2209\_BuCaKA02.raw | 2.8125E8 | 1 | 1 | 65 | 80 | Carbamidomethylation | C2:Carbamidomethylation:1000.00;C6:Carbamidomethylation:1000.00 | PEAKS DB |
| E.C(+57.02)AATC(+57.02)PPKRLGVTILC(+57.02)C(+57.02)TTDNC(+57.02)NR | Y | 72.24 | 2837.2864 | 24 | 0.3 | 946.7697 | 3 | 25.27 | 1 | 12190 | 01122020\_RID\_2209\_BuCaKA02.raw | 1.7954E8 | 2 | 2 | 66 | 89 | Carbamidomethylation | C1:Carbamidomethylation:1000.00;C5:Carbamidomethylation:1000.00;C16:Carbamidomethylation:1000.00;C17:Carbamidomethylation:1000.00;C22:Carbamidomethylation:1000.00 | PEAKS DB |
| R.FYTC(+57.02)PNSETC(+57.02)PDGKNIC(+57.02)VK.L | Y | 72.05 | 2288.9863 | 19 | -0.4 | 1145.5000 | 2 | 20.41 | 1 | 8383 | 01122020\_RID\_2209\_BuCaKA02.raw | 1.8912E7 | 1 | 1 | 29 | 47 | Carbamidomethylation | C4:Carbamidomethylation:1000.00;C10:Carbamidomethylation:1000.00;C17:Carbamidomethylation:1000.00 | PEAKS DB |
| L.GVTILC(+57.02)C(+57.02)TTDNC(+57.02)NR | Y | 71.42 | 1682.7175 | 14 | 2.1 | 842.3678 | 2 | 25.97 | 1 | 12849 | 01122020\_RID\_2209\_BuCaKA02.raw | 2.9952E8 | 3 | 3 | 76 | 89 | Carbamidomethylation | C6:Carbamidomethylation:1000.00;C7:Carbamidomethylation:1000.00;C12:Carbamidomethylation:1000.00 | PEAKS DB |
| C.AATC(+57.02)PPKRLGVTILC(+57.02)C(+57.02)TTDNC(+57.02)NR | Y | 70.18 | 2677.2556 | 23 | 1.7 | 893.4274 | 3 | 23.87 | 1 | 11330 | 01122020\_RID\_2209\_BuCaKA02.raw | 1.5716E8 | 2 | 2 | 67 | 89 | Carbamidomethylation | C4:Carbamidomethylation:1000.00;C15:Carbamidomethylation:1000.00;C16:Carbamidomethylation:1000.00;C21:Carbamidomethylation:1000.00 | PEAKS DB |
| R.FYTC(+57.02)PNSETC(+57.02)PDGKNIC(+57.02)VKLSWTVMR.G | Y | 67.12 | 3162.4395 | 26 | 1.5 | 791.6183 | 4 | 48.80 | 1 | 30169 | 01122020\_RID\_2209\_BuCaKA02.raw | 4.5506E7 | 3 | 3 | 29 | 54 | Carbamidomethylation | C4:Carbamidomethylation:1000.00;C10:Carbamidomethylation:1000.00;C17:Carbamidomethylation:1000.00 | PEAKS DB |
| A.ATC(+57.02)PPKRLGVTILC(+57.02)C(+57.02)TTDNC(+57.02)NR | Y | 66.55 | 2606.2185 | 22 | 1.4 | 869.7480 | 3 | 23.11 | 1 | 10513 | 01122020\_RID\_2209\_BuCaKA02.raw | 7.1636E7 | 2 | 2 | 68 | 89 | Carbamidomethylation | C3:Carbamidomethylation:1000.00;C14:Carbamidomethylation:1000.00;C15:Carbamidomethylation:1000.00;C20:Carbamidomethylation:1000.00 | PEAKS DB |
| T.C(+57.02)PPKRLGVTILC(+57.02)C(+57.02)TTDNC(+57.02)NR | Y | 65.38 | 2434.1338 | 20 | -0.3 | 609.5405 | 4 | 20.85 | 1 | 8796 | 01122020\_RID\_2209\_BuCaKA02.raw | 4.0023E6 | 1 | 1 | 70 | 89 | Carbamidomethylation | C1:Carbamidomethylation:1000.00;C12:Carbamidomethylation:1000.00;C13:Carbamidomethylation:1000.00;C18:Carbamidomethylation:1000.00 | PEAKS DB |
| R.EC(+57.02)AATC(+57.02)PPKRLGVTILC(+57.02).C | Y | 61.42 | 1944.9584 | 17 | 0.0 | 973.4865 | 2 | 34.61 | 1 | 19362 | 01122020\_RID\_2209\_BuCaKA02.raw | 1.8188E8 | 2 | 2 | 65 | 81 | Carbamidomethylation | C2:Carbamidomethylation:1000.00;C6:Carbamidomethylation:1000.00;C17:Carbamidomethylation:1000.00 | PEAKS DB |
| T.LTC(+57.02)ETC(+57.02)RFYTC(+57.02)PNSETC(+57.02)PDGKNIC(+57.02)VK.L | Y | 56.88 | 3209.3708 | 26 | 1.0 | 1070.7986 | 3 | 23.46 | 1 | 10881 | 01122020\_RID\_2209\_BuCaKA02.raw | 3.4967E6 | 1 | 1 | 22 | 47 | Carbamidomethylation | C3:Carbamidomethylation:1000.00;C6:Carbamidomethylation:1000.00;C11:Carbamidomethylation:1000.00;C17:Carbamidomethylation:1000.00;C24:Carbamidomethylation:1000.00 | PEAKS DB |
| K.RLGVTILC(+57.02)C(+57.02)TTDNC(+57.02)N.R | Y | 56.15 | 1795.8015 | 15 | 2.2 | 898.9100 | 2 | 40.43 | 1 | 24075 | 01122020\_RID\_2209\_BuCaKA02.raw | 0 | 0 | 0 | 74 | 88 | Carbamidomethylation | C8:Carbamidomethylation:1000.00;C9:Carbamidomethylation:1000.00;C14:Carbamidomethylation:1000.00 | PEAKS DB |
| R.FYTC(+57.02)PNSETC(+57.02)PDGKNIC(+57.02)V.K | Y | 42.06 | 2160.8914 | 18 | 0.8 | 1081.4539 | 2 | 34.70 | 1 | 19646 | 01122020\_RID\_2209\_BuCaKA02.raw | 3.2555E7 | 1 | 1 | 29 | 46 | Carbamidomethylation | C4:Carbamidomethylation:1000.00;C10:Carbamidomethylation:1000.00;C17:Carbamidomethylation:1000.00 | PEAKS DB |
| total 16 peptides |  |  |  |  |  |  |  |  |  |  |  |  |  |  |  |  |  |  |  |
| --- | --- | --- | --- | --- | --- | --- | --- | --- | --- | --- | --- | --- | --- | --- | --- | --- | --- | --- | --- |

Q8QFW3.1

back to list

  

| Protein Coverage
| Supporting Peptides
|

Protein Coverage:

Supporting Peptides:

| Peptide | Uniq | -10lgP | Mass | Length | ppm | m/z | z | RT | Fraction | Scan | Source File | Area BRom | #Feature | #Feature BRom | Start | End | PTM | AScore | Found By |
| --- | --- | --- | --- | --- | --- | --- | --- | --- | --- | --- | --- | --- | --- | --- | --- | --- | --- | --- | --- |
| K.TWGHYADYGC(+57.02)YC(+57.02)GAGGSGTPVDALDR.C | Y | 85.34 | 2805.1548 | 26 | 0.7 | 936.0596 | 3 | 46.88 | 1 | 29158 | 01122020\_RID\_2209\_BuCaKA02.raw | 5.7132E8 | 3 | 3 | 45 | 70 | Carbamidomethylation | C10:Carbamidomethylation:1000.00;C12:Carbamidomethylation:1000.00 | PEAKS DB |
| L.NLINFMEMIRYTIPC(+57.02)DK.T | Y | 84.13 | 2157.0420 | 17 | 2.0 | 1079.5304 | 2 | 84.10 | 1 | 59349 | 01122020\_RID\_2209\_BuCaKA02.raw | 2.5457E8 | 5 | 5 | 28 | 44 | Carbamidomethylation | C15:Carbamidomethylation:1000.00 | PEAKS DB |
| I.RYTIPC(+57.02)DKTWGHYADYGC(+57.02)YC(+57.02)GAGGSGTPVDALDR.C | Y | 78.45 | 3838.6562 | 34 | 0.6 | 960.6719 | 4 | 44.03 | 1 | 26582 | 01122020\_RID\_2209\_BuCaKA02.raw | 1.7456E8 | 3 | 3 | 37 | 70 | Carbamidomethylation | C6:Carbamidomethylation:1000.00;C18:Carbamidomethylation:1000.00;C20:Carbamidomethylation:1000.00 | PEAKS DB |
| K.RTIIC(+57.02)YGAAGTC(+57.02)GR.I | N | 78.18 | 1554.7395 | 14 | 1.6 | 778.3783 | 2 | 12.18 | 1 | 2117 | 01122020\_RID\_2209\_BuCaKA02.raw | 5.9802E6 | 1 | 1 | 102 | 115 | Carbamidomethylation | C5:Carbamidomethylation:1000.00;C12:Carbamidomethylation:1000.00 | PEAKS DB |
| Y.GC(+57.02)YC(+57.02)GAGGSGTPVDALDR.C | N | 76.28 | 1811.7566 | 18 | 1.3 | 906.8867 | 2 | 29.21 | 1 | 15083 | 01122020\_RID\_2209\_BuCaKA02.raw | 5.769E6 | 1 | 1 | 53 | 70 | Carbamidomethylation | C2:Carbamidomethylation:1000.00;C4:Carbamidomethylation:1000.00 | PEAKS DB |
| D.YGC(+57.02)YC(+57.02)GAGGSGTPVDALDR.C | N | 75.35 | 1974.8199 | 19 | 1.8 | 988.4191 | 2 | 35.54 | 1 | 20103 | 01122020\_RID\_2209\_BuCaKA02.raw | 4.3649E7 | 1 | 1 | 52 | 70 | Carbamidomethylation | C3:Carbamidomethylation:1000.00;C5:Carbamidomethylation:1000.00 | PEAKS DB |
| L.NLINFMEMIRYTIPC(+57.02)DKTWGHYADYGC(+57.02)YC(+57.02)GAGGSGTPVDALDR.C | Y | 73.43 | 4944.1860 | 43 | 0.7 | 1237.0547 | 4 | 83.34 | 1 | 58549 | 01122020\_RID\_2209\_BuCaKA02.raw | 1.1437E9 | 2 | 2 | 28 | 70 | Carbamidomethylation | C15:Carbamidomethylation:1000.00;C27:Carbamidomethylation:1000.00;C29:Carbamidomethylation:1000.00 | PEAKS DB |
| M.IRYTIPC(+57.02)DKTWGHYADYGC(+57.02)YC(+57.02)GAGGSGTPVDALDR.C | Y | 71.00 | 3951.7402 | 35 | 0.9 | 988.9432 | 4 | 47.91 | 1 | 29528 | 01122020\_RID\_2209\_BuCaKA02.raw | 5.3528E7 | 1 | 1 | 36 | 70 | Carbamidomethylation | C7:Carbamidomethylation:1000.00;C19:Carbamidomethylation:1000.00;C21:Carbamidomethylation:1000.00 | PEAKS DB |
| R.TIIC(+57.02)YGAAGTC(+57.02)GR.I | N | 70.35 | 1398.6384 | 13 | 1.6 | 700.3276 | 2 | 22.35 | 1 | 11385 | 01122020\_RID\_2209\_BuCaKA02.raw | 4.4986E8 | 7 | 7 | 103 | 115 | Carbamidomethylation | C4:Carbamidomethylation:1000.00;C11:Carbamidomethylation:1000.00 | PEAKS DB |
| R.TIIC(+57.02)YGAAGTC(+57.02)GRIVC(+57.02)DC(+57.02)DR.T | N | 70.23 | 2317.0071 | 20 | 0.8 | 1159.5117 | 2 | 29.20 | 1 | 15032 | 01122020\_RID\_2209\_BuCaKA02.raw | 1.3733E8 | 2 | 2 | 103 | 122 | Carbamidomethylation | C4:Carbamidomethylation:1000.00;C11:Carbamidomethylation:1000.00;C16:Carbamidomethylation:1000.00;C18:Carbamidomethylation:1000.00 | PEAKS DB |
| L.NLINFMEMIR.Y | N | 70.13 | 1279.6417 | 10 | 0.6 | 640.8285 | 2 | 78.90 | 1 | 54806 | 01122020\_RID\_2209\_BuCaKA02.raw | 2.8606E8 | 1 | 1 | 28 | 37 |  |  | PEAKS DB |
| R.YTIPC(+57.02)DKTWGHYADYGC(+57.02)YC(+57.02)GAGGSGTPVDALDR.C | Y | 65.10 | 3682.5552 | 33 | 0.6 | 921.6466 | 4 | 51.29 | 1 | 31798 | 01122020\_RID\_2209\_BuCaKA02.raw | 1.1272E10 | 2 | 2 | 38 | 70 | Carbamidomethylation | C5:Carbamidomethylation:1000.00;C17:Carbamidomethylation:1000.00;C19:Carbamidomethylation:1000.00 | PEAKS DB |
| R.IVC(+57.02)DC(+57.02)DRTAALC(+57.02)FG.D | N | 58.90 | 1656.7058 | 14 | 1.9 | 829.3618 | 2 | 40.45 | 1 | 24023 | 01122020\_RID\_2209\_BuCaKA02.raw | 6.5443E7 | 1 | 1 | 116 | 129 | Carbamidomethylation | C3:Carbamidomethylation:1000.00;C5:Carbamidomethylation:1000.00;C12:Carbamidomethylation:1000.00 | PEAKS DB |
| F.MEMIRYTIPC(+57.02)DKTWGHYADYGC(+57.02)YC(+57.02)GAGGSGTPVDALDR.C | Y | 58.39 | 4342.8638 | 38 | 1.3 | 1086.7246 | 4 | 55.97 | 1 | 36026 | 01122020\_RID\_2209\_BuCaKA02.raw | 1.6697E7 | 1 | 1 | 33 | 70 | Carbamidomethylation | C10:Carbamidomethylation:1000.00;C22:Carbamidomethylation:1000.00;C24:Carbamidomethylation:1000.00 | PEAKS DB |
| N.LINFMEMIR.Y | N | 57.43 | 1165.5988 | 9 | -0.2 | 583.8065 | 2 | 73.46 | 1 | 50593 | 01122020\_RID\_2209\_BuCaKA02.raw | 5.7582E7 | 1 | 1 | 29 | 37 |  |  | PEAKS DB |
| N.LINFM(+15.99)EMIR.Y | N | 56.16 | 1181.5936 | 9 | 0.5 | 591.8044 | 2 | 56.54 | 1 | 36487 | 01122020\_RID\_2209\_BuCaKA02.raw | 3.0018E6 | 1 | 1 | 29 | 37 | Oxidation (M) | M5:Oxidation (M):30.83 | PEAKS DB |
| L.NLINFMEM(+15.99)IR.Y | N | 54.09 | 1295.6366 | 10 | 2.9 | 648.8275 | 2 | 69.38 | 1 | 47058 | 01122020\_RID\_2209\_BuCaKA02.raw | 0 | 0 | 0 | 28 | 37 | Oxidation (M) | M8:Oxidation (M):15.73 | PEAKS DB |
| R.TAALC(+57.02)FGDSEY.I | N | 49.80 | 1232.5020 | 11 | 0.7 | 617.2587 | 2 | 56.34 | 1 | 36497 | 01122020\_RID\_2209\_BuCaKA02.raw | 8.7681E6 | 1 | 1 | 123 | 133 | Carbamidomethylation | C5:Carbamidomethylation:1000.00 | PEAKS DB |
| K.RTIIC(+57.02)YGAAGTC(+57.02)GRIVC(+57.02)DC(+57.02)DR.T | N | 49.55 | 2473.1084 | 21 | 2.0 | 825.3784 | 3 | 21.10 | 1 | 8964 | 01122020\_RID\_2209\_BuCaKA02.raw | 0 | 0 | 0 | 102 | 122 | Carbamidomethylation | C5:Carbamidomethylation:1000.00;C12:Carbamidomethylation:1000.00;C17:Carbamidomethylation:1000.00;C19:Carbamidomethylation:1000.00 | PEAKS DB |
| R.IVC(+57.02)DC(+57.02)DRTAALC(+57.02)FGDSEY.I | N | 47.92 | 2150.8706 | 18 | -0.9 | 1076.4417 | 2 | 50.53 | 1 | 31724 | 01122020\_RID\_2209\_BuCaKA02.raw | 2.4211E8 | 2 | 2 | 116 | 133 | Carbamidomethylation | C3:Carbamidomethylation:1000.00;C5:Carbamidomethylation:1000.00;C12:Carbamidomethylation:1000.00 | PEAKS DB |
| total 20 peptides |  |  |  |  |  |  |  |  |  |  |  |  |  |  |  |  |  |  |  |
| --- | --- | --- | --- | --- | --- | --- | --- | --- | --- | --- | --- | --- | --- | --- | --- | --- | --- | --- | --- |

AAL87004.1

back to list

  

| Protein Coverage
| Supporting Peptides
|

Protein Coverage:

Supporting Peptides:

| Peptide | Uniq | -10lgP | Mass | Length | ppm | m/z | z | RT | Fraction | Scan | Source File | Area BRom | #Feature | #Feature BRom | Start | End | PTM | AScore | Found By |
| --- | --- | --- | --- | --- | --- | --- | --- | --- | --- | --- | --- | --- | --- | --- | --- | --- | --- | --- | --- |
| K.TWGHYADYGC(+57.02)YC(+57.02)GAGGSGTPVDALDR.C | Y | 85.34 | 2805.1548 | 26 | 0.7 | 936.0596 | 3 | 46.88 | 1 | 29158 | 01122020\_RID\_2209\_BuCaKA02.raw | 5.7132E8 | 3 | 3 | 45 | 70 | Carbamidomethylation | C10:Carbamidomethylation:1000.00;C12:Carbamidomethylation:1000.00 | PEAKS DB |
| L.NLINFMEMIRYTIPC(+57.02)DK.T | Y | 84.13 | 2157.0420 | 17 | 2.0 | 1079.5304 | 2 | 84.10 | 1 | 59349 | 01122020\_RID\_2209\_BuCaKA02.raw | 2.5457E8 | 5 | 5 | 28 | 44 | Carbamidomethylation | C15:Carbamidomethylation:1000.00 | PEAKS DB |
| I.RYTIPC(+57.02)DKTWGHYADYGC(+57.02)YC(+57.02)GAGGSGTPVDALDR.C | Y | 78.45 | 3838.6562 | 34 | 0.6 | 960.6719 | 4 | 44.03 | 1 | 26582 | 01122020\_RID\_2209\_BuCaKA02.raw | 1.7456E8 | 3 | 3 | 37 | 70 | Carbamidomethylation | C6:Carbamidomethylation:1000.00;C18:Carbamidomethylation:1000.00;C20:Carbamidomethylation:1000.00 | PEAKS DB |
| K.RTIIC(+57.02)YGAAGTC(+57.02)GR.I | N | 78.18 | 1554.7395 | 14 | 1.6 | 778.3783 | 2 | 12.18 | 1 | 2117 | 01122020\_RID\_2209\_BuCaKA02.raw | 5.9802E6 | 1 | 1 | 102 | 115 | Carbamidomethylation | C5:Carbamidomethylation:1000.00;C12:Carbamidomethylation:1000.00 | PEAKS DB |
| Y.GC(+57.02)YC(+57.02)GAGGSGTPVDALDR.C | N | 76.28 | 1811.7566 | 18 | 1.3 | 906.8867 | 2 | 29.21 | 1 | 15083 | 01122020\_RID\_2209\_BuCaKA02.raw | 5.769E6 | 1 | 1 | 53 | 70 | Carbamidomethylation | C2:Carbamidomethylation:1000.00;C4:Carbamidomethylation:1000.00 | PEAKS DB |
| D.YGC(+57.02)YC(+57.02)GAGGSGTPVDALDR.C | N | 75.35 | 1974.8199 | 19 | 1.8 | 988.4191 | 2 | 35.54 | 1 | 20103 | 01122020\_RID\_2209\_BuCaKA02.raw | 4.3649E7 | 1 | 1 | 52 | 70 | Carbamidomethylation | C3:Carbamidomethylation:1000.00;C5:Carbamidomethylation:1000.00 | PEAKS DB |
| L.NLINFMEMIRYTIPC(+57.02)DKTWGHYADYGC(+57.02)YC(+57.02)GAGGSGTPVDALDR.C | Y | 73.43 | 4944.1860 | 43 | 0.7 | 1237.0547 | 4 | 83.34 | 1 | 58549 | 01122020\_RID\_2209\_BuCaKA02.raw | 1.1437E9 | 2 | 2 | 28 | 70 | Carbamidomethylation | C15:Carbamidomethylation:1000.00;C27:Carbamidomethylation:1000.00;C29:Carbamidomethylation:1000.00 | PEAKS DB |
| M.IRYTIPC(+57.02)DKTWGHYADYGC(+57.02)YC(+57.02)GAGGSGTPVDALDR.C | Y | 71.00 | 3951.7402 | 35 | 0.9 | 988.9432 | 4 | 47.91 | 1 | 29528 | 01122020\_RID\_2209\_BuCaKA02.raw | 5.3528E7 | 1 | 1 | 36 | 70 | Carbamidomethylation | C7:Carbamidomethylation:1000.00;C19:Carbamidomethylation:1000.00;C21:Carbamidomethylation:1000.00 | PEAKS DB |
| R.TIIC(+57.02)YGAAGTC(+57.02)GR.I | N | 70.35 | 1398.6384 | 13 | 1.6 | 700.3276 | 2 | 22.35 | 1 | 11385 | 01122020\_RID\_2209\_BuCaKA02.raw | 4.4986E8 | 7 | 7 | 103 | 115 | Carbamidomethylation | C4:Carbamidomethylation:1000.00;C11:Carbamidomethylation:1000.00 | PEAKS DB |
| R.TIIC(+57.02)YGAAGTC(+57.02)GRIVC(+57.02)DC(+57.02)DR.T | N | 70.23 | 2317.0071 | 20 | 0.8 | 1159.5117 | 2 | 29.20 | 1 | 15032 | 01122020\_RID\_2209\_BuCaKA02.raw | 1.3733E8 | 2 | 2 | 103 | 122 | Carbamidomethylation | C4:Carbamidomethylation:1000.00;C11:Carbamidomethylation:1000.00;C16:Carbamidomethylation:1000.00;C18:Carbamidomethylation:1000.00 | PEAKS DB |
| L.NLINFMEMIR.Y | N | 70.13 | 1279.6417 | 10 | 0.6 | 640.8285 | 2 | 78.90 | 1 | 54806 | 01122020\_RID\_2209\_BuCaKA02.raw | 2.8606E8 | 1 | 1 | 28 | 37 |  |  | PEAKS DB |
| R.YTIPC(+57.02)DKTWGHYADYGC(+57.02)YC(+57.02)GAGGSGTPVDALDR.C | Y | 65.10 | 3682.5552 | 33 | 0.6 | 921.6466 | 4 | 51.29 | 1 | 31798 | 01122020\_RID\_2209\_BuCaKA02.raw | 1.1272E10 | 2 | 2 | 38 | 70 | Carbamidomethylation | C5:Carbamidomethylation:1000.00;C17:Carbamidomethylation:1000.00;C19:Carbamidomethylation:1000.00 | PEAKS DB |
| R.IVC(+57.02)DC(+57.02)DRTAALC(+57.02)FG.D | N | 58.90 | 1656.7058 | 14 | 1.9 | 829.3618 | 2 | 40.45 | 1 | 24023 | 01122020\_RID\_2209\_BuCaKA02.raw | 6.5443E7 | 1 | 1 | 116 | 129 | Carbamidomethylation | C3:Carbamidomethylation:1000.00;C5:Carbamidomethylation:1000.00;C12:Carbamidomethylation:1000.00 | PEAKS DB |
| F.MEMIRYTIPC(+57.02)DKTWGHYADYGC(+57.02)YC(+57.02)GAGGSGTPVDALDR.C | Y | 58.39 | 4342.8638 | 38 | 1.3 | 1086.7246 | 4 | 55.97 | 1 | 36026 | 01122020\_RID\_2209\_BuCaKA02.raw | 1.6697E7 | 1 | 1 | 33 | 70 | Carbamidomethylation | C10:Carbamidomethylation:1000.00;C22:Carbamidomethylation:1000.00;C24:Carbamidomethylation:1000.00 | PEAKS DB |
| N.LINFMEMIR.Y | N | 57.43 | 1165.5988 | 9 | -0.2 | 583.8065 | 2 | 73.46 | 1 | 50593 | 01122020\_RID\_2209\_BuCaKA02.raw | 5.7582E7 | 1 | 1 | 29 | 37 |  |  | PEAKS DB |
| N.LINFM(+15.99)EMIR.Y | N | 56.16 | 1181.5936 | 9 | 0.5 | 591.8044 | 2 | 56.54 | 1 | 36487 | 01122020\_RID\_2209\_BuCaKA02.raw | 3.0018E6 | 1 | 1 | 29 | 37 | Oxidation (M) | M5:Oxidation (M):30.83 | PEAKS DB |
| L.NLINFMEM(+15.99)IR.Y | N | 54.09 | 1295.6366 | 10 | 2.9 | 648.8275 | 2 | 69.38 | 1 | 47058 | 01122020\_RID\_2209\_BuCaKA02.raw | 0 | 0 | 0 | 28 | 37 | Oxidation (M) | M8:Oxidation (M):15.73 | PEAKS DB |
| R.TAALC(+57.02)FGDSEY.I | N | 49.80 | 1232.5020 | 11 | 0.7 | 617.2587 | 2 | 56.34 | 1 | 36497 | 01122020\_RID\_2209\_BuCaKA02.raw | 8.7681E6 | 1 | 1 | 123 | 133 | Carbamidomethylation | C5:Carbamidomethylation:1000.00 | PEAKS DB |
| K.RTIIC(+57.02)YGAAGTC(+57.02)GRIVC(+57.02)DC(+57.02)DR.T | N | 49.55 | 2473.1084 | 21 | 2.0 | 825.3784 | 3 | 21.10 | 1 | 8964 | 01122020\_RID\_2209\_BuCaKA02.raw | 0 | 0 | 0 | 102 | 122 | Carbamidomethylation | C5:Carbamidomethylation:1000.00;C12:Carbamidomethylation:1000.00;C17:Carbamidomethylation:1000.00;C19:Carbamidomethylation:1000.00 | PEAKS DB |
| R.IVC(+57.02)DC(+57.02)DRTAALC(+57.02)FGDSEY.I | N | 47.92 | 2150.8706 | 18 | -0.9 | 1076.4417 | 2 | 50.53 | 1 | 31724 | 01122020\_RID\_2209\_BuCaKA02.raw | 2.4211E8 | 2 | 2 | 116 | 133 | Carbamidomethylation | C3:Carbamidomethylation:1000.00;C5:Carbamidomethylation:1000.00;C12:Carbamidomethylation:1000.00 | PEAKS DB |
| total 20 peptides |  |  |  |  |  |  |  |  |  |  |  |  |  |  |  |  |  |  |  |
| --- | --- | --- | --- | --- | --- | --- | --- | --- | --- | --- | --- | --- | --- | --- | --- | --- | --- | --- | --- |

JAA74736.1

back to list

  

| Protein Coverage
| Supporting Peptides
|

Protein Coverage:

Supporting Peptides:

| Peptide | Uniq | -10lgP | Mass | Length | ppm | m/z | z | RT | Fraction | Scan | Source File | Area BRom | #Feature | #Feature BRom | Start | End | PTM | AScore | Found By |
| --- | --- | --- | --- | --- | --- | --- | --- | --- | --- | --- | --- | --- | --- | --- | --- | --- | --- | --- | --- |
| R.AILQSGGPNAPWATVTPAESR.R | N | 95.10 | 2122.0806 | 21 | -0.9 | 1062.0466 | 2 | 52.50 | 1 | 33145 | 01122020\_RID\_2209\_BuCaKA02.raw | 1.3371E9 | 2 | 2 | 245 | 265 |  |  | PEAKS DB |
| R.EALDDIVGDHNVIC(+57.02)PVVQFANDYAKR.N | N | 93.08 | 2957.4341 | 26 | 0.1 | 740.3659 | 4 | 70.15 | 1 | 47677 | 01122020\_RID\_2209\_BuCaKA02.raw | 1.6502E7 | 3 | 3 | 412 | 437 | Carbamidomethylation | C14:Carbamidomethylation:1000.00 | PEAKS DB |
| R.EALDDIVGDHNVIC(+57.02)PVVQFANDYAK.R | N | 88.62 | 2801.3330 | 25 | 1.0 | 1401.6752 | 2 | 77.11 | 1 | 53666 | 01122020\_RID\_2209\_BuCaKA02.raw | 7.8726E8 | 2 | 2 | 412 | 436 | Carbamidomethylation | C14:Carbamidomethylation:1000.00 | PEAKS DB |
| S.AVTIFGESAGAASVGMHLLSTQSR.A | N | 85.30 | 2389.2061 | 24 | 0.3 | 797.4095 | 3 | 65.27 | 1 | 44229 | 01122020\_RID\_2209\_BuCaKA02.raw | 4.1007E8 | 3 | 3 | 216 | 239 |  |  | PEAKS DB |
| K.NREALDDIVGDHNVIC(+57.02)PVVQFANDYAK.R | N | 82.20 | 3071.4771 | 27 | 0.7 | 768.8771 | 4 | 65.86 | 1 | 44085 | 01122020\_RID\_2209\_BuCaKA02.raw | 2.8252E8 | 1 | 1 | 410 | 436 | Carbamidomethylation | C16:Carbamidomethylation:1000.00 | PEAKS DB |
| D.DIVGDHNVIC(+57.02)PVVQFANDYAK.R | N | 70.32 | 2373.1423 | 21 | 1.2 | 792.0557 | 3 | 67.35 | 1 | 45397 | 01122020\_RID\_2209\_BuCaKA02.raw | 3.7367E7 | 1 | 1 | 416 | 436 | Carbamidomethylation | C10:Carbamidomethylation:1000.00 | PEAKS DB |
| R.AILQSGGPNAPWATVTPAESRR.R | N | 67.72 | 2278.1819 | 22 | 1.4 | 760.4023 | 3 | 39.81 | 1 | 23723 | 01122020\_RID\_2209\_BuCaKA02.raw | 7.6056E7 | 1 | 1 | 245 | 266 |  |  | PEAKS DB |
| F.LGIPFAEPPVGR.M | N | 65.37 | 1251.6975 | 12 | 1.1 | 626.8567 | 2 | 56.11 | 1 | 36154 | 01122020\_RID\_2209\_BuCaKA02.raw | 4.6223E8 | 1 | 1 | 54 | 65 |  |  | PEAKS DB |
| A.FLGIPFAEPPVGR.M | N | 62.09 | 1398.7659 | 13 | 0.0 | 700.3902 | 2 | 73.18 | 1 | 50153 | 01122020\_RID\_2209\_BuCaKA02.raw | 1.8064E7 | 1 | 1 | 53 | 65 |  |  | PEAKS DB |
| K.SIFRFPFVPVIDG.D | N | 58.74 | 1492.8077 | 13 | 2.3 | 747.4128 | 2 | 91.00 | 1 | 65018 | 01122020\_RID\_2209\_BuCaKA02.raw | 9.7066E6 | 1 | 1 | 309 | 321 |  |  | PEAKS DB |
| R.GLSLPVLDGHVSAFLGIPFAEPPVGR.M | Y | 55.48 | 2644.4375 | 26 | -1.8 | 1323.2236 | 2 | 94.08 | 1 | 67445 | 01122020\_RID\_2209\_BuCaKA02.raw | 4.0747E6 | 1 | 1 | 40 | 65 |  |  | PEAKS DB |
| S.AVTIFGESAGAASVGM(+15.99)HLLSTQSR.A | N | 45.59 | 2405.2009 | 24 | 3.4 | 802.7437 | 3 | 54.96 | 1 | 35227 | 01122020\_RID\_2209\_BuCaKA02.raw | 8.7978E6 | 1 | 1 | 216 | 239 | Oxidation (M) | M16:Oxidation (M):1000.00 | PEAKS DB |
| total 12 peptides |  |  |  |  |  |  |  |  |  |  |  |  |  |  |  |  |  |  |  |
| --- | --- | --- | --- | --- | --- | --- | --- | --- | --- | --- | --- | --- | --- | --- | --- | --- | --- | --- | --- |

T\_84

back to list

  

| Protein Coverage
| Supporting Peptides
|

Protein Coverage:

Supporting Peptides:

| Peptide | Uniq | -10lgP | Mass | Length | ppm | m/z | z | RT | Fraction | Scan | Source File | Area BRom | #Feature | #Feature BRom | Start | End | PTM | AScore | Found By |
| --- | --- | --- | --- | --- | --- | --- | --- | --- | --- | --- | --- | --- | --- | --- | --- | --- | --- | --- | --- |
| K.IEPDVDATLKSFAKWR.E | Y | 83.40 | 1874.9890 | 16 | 1.8 | 626.0048 | 3 | 53.89 | 1 | 34389 | 01122020\_RID\_2209\_BuCaKA02.raw | 2.1471E7 | 1 | 1 | 141 | 156 |  |  | PEAKS DB |
| K.IKIEPDVDATLKSFAK.W | Y | 75.86 | 1773.9875 | 16 | 0.8 | 592.3369 | 3 | 43.82 | 1 | 26512 | 01122020\_RID\_2209\_BuCaKA02.raw | 3.6216E8 | 2 | 2 | 139 | 154 |  |  | PEAKS DB |
| K.IEPDVDATLKSFAK.W | Y | 74.96 | 1532.8086 | 14 | 1.1 | 767.4124 | 2 | 44.40 | 1 | 26876 | 01122020\_RID\_2209\_BuCaKA02.raw | 0 | 0 | 0 | 141 | 154 |  |  | PEAKS DB |
| K.VYGLVNHLNMIYK.S | Y | 72.88 | 1562.8279 | 13 | 1.1 | 782.4221 | 2 | 42.26 | 1 | 25359 | 01122020\_RID\_2209\_BuCaKA02.raw | 1.7514E6 | 1 | 1 | 107 | 119 |  |  | PEAKS DB |
| K.YIELYVAVDNR.M | Y | 69.86 | 1353.6929 | 11 | 1.2 | 677.8545 | 2 | 56.57 | 1 | 36531 | 01122020\_RID\_2209\_BuCaKA02.raw | 4.5074E6 | 4 | 4 | 81 | 91 |  |  | PEAKS DB |
| L.IGLEIWSKEDK.I | Y | 67.84 | 1316.6975 | 11 | 0.8 | 659.3566 | 2 | 35.97 | 1 | 20532 | 01122020\_RID\_2209\_BuCaKA02.raw | 1.6806E7 | 1 | 1 | 128 | 138 |  |  | PEAKS DB |
| L.IALIGLEIWSKEDK.I | Y | 67.63 | 1613.9028 | 14 | 2.0 | 807.9603 | 2 | 63.43 | 1 | 42108 | 01122020\_RID\_2209\_BuCaKA02.raw | 5.4921E6 | 1 | 1 | 125 | 138 |  |  | PEAKS DB |
| I.ALIGLEIWSK.E | N | 61.16 | 1128.6543 | 10 | 0.6 | 565.3348 | 2 | 67.13 | 1 | 45199 | 01122020\_RID\_2209\_BuCaKA02.raw | 2.8404E6 | 1 | 1 | 126 | 135 |  |  | PEAKS DB |
| L.IALIGLEIWSK.E | N | 61.01 | 1241.7383 | 11 | 4.0 | 621.8789 | 2 | 77.42 | 1 | 53834 | 01122020\_RID\_2209\_BuCaKA02.raw | 2.4633E7 | 1 | 1 | 125 | 135 |  |  | PEAKS DB |
| R.KVYGLVNHLNMIYKSLNFL.I | Y | 57.51 | 2265.2344 | 19 | 1.8 | 567.3169 | 4 | 71.75 | 1 | 49030 | 01122020\_RID\_2209\_BuCaKA02.raw | 3.4388E7 | 1 | 1 | 106 | 124 |  |  | PEAKS DB |
| K.VYGLVNHLNMIYKSLNFL.I | Y | 57.01 | 2137.1394 | 18 | 1.9 | 713.3884 | 3 | 81.86 | 1 | 57396 | 01122020\_RID\_2209\_BuCaKA02.raw | 3.681E7 | 2 | 2 | 107 | 124 |  |  | PEAKS DB |
| R.KVYGLVNHLNMIYK.S | Y | 55.55 | 1690.9229 | 14 | 1.5 | 564.6490 | 3 | 31.24 | 1 | 16739 | 01122020\_RID\_2209\_BuCaKA02.raw | 5.1752E6 | 1 | 1 | 106 | 119 |  |  | PEAKS DB |
| R.KVYGLVNHLNMIYKSLNF.L | Y | 51.86 | 2152.1504 | 18 | 2.0 | 718.3922 | 3 | 62.40 | 1 | 41280 | 01122020\_RID\_2209\_BuCaKA02.raw | 0 | 0 | 0 | 106 | 123 |  |  | PEAKS DB |
| R.YLQIKKYIEL.Y | Y | 43.34 | 1309.7645 | 10 | 0.4 | 655.8898 | 2 | 42.54 | 1 | 25559 | 01122020\_RID\_2209\_BuCaKA02.raw | 1.6512E7 | 1 | 1 | 75 | 84 |  |  | PEAKS DB |
| L.IALIGLEIWSKEDKIK.I | Y | 42.41 | 1855.0818 | 16 | 1.4 | 619.3687 | 3 | 55.31 | 1 | 35484 | 01122020\_RID\_2209\_BuCaKA02.raw | 6.0952E6 | 1 | 1 | 125 | 140 |  |  | PEAKS DB |
| total 15 peptides |  |  |  |  |  |  |  |  |  |  |  |  |  |  |  |  |  |  |  |
| --- | --- | --- | --- | --- | --- | --- | --- | --- | --- | --- | --- | --- | --- | --- | --- | --- | --- | --- | --- |

ABN72539.1

back to list

  

| Protein Coverage
| Supporting Peptides
|

Protein Coverage:

Supporting Peptides:

| Peptide | Uniq | -10lgP | Mass | Length | ppm | m/z | z | RT | Fraction | Scan | Source File | Area BRom | #Feature | #Feature BRom | Start | End | PTM | AScore | Found By |
| --- | --- | --- | --- | --- | --- | --- | --- | --- | --- | --- | --- | --- | --- | --- | --- | --- | --- | --- | --- |
| R.IYFAGEYTARVHGWLDSTIKSGLTAAR.D | Y | 85.73 | 2982.5352 | 27 | 2.2 | 746.6427 | 4 | 62.50 | 1 | 41757 | 01122020\_RID\_2209\_BuCaKA02.raw | 1.0441E8 | 5 | 5 | 471 | 497 |  |  | PEAKS DB |
| R.VHGWLDSTIKSGLTAAR.D | Y | 85.19 | 1810.9690 | 17 | 1.5 | 604.6645 | 3 | 36.01 | 1 | 20499 | 01122020\_RID\_2209\_BuCaKA02.raw | 1.1031E8 | 3 | 3 | 481 | 497 |  |  | PEAKS DB |
| K.WSLDKYTMGALTSFTPYQFQDYSETVAAPVGR.I | N | 77.95 | 3628.7183 | 32 | -1.3 | 1210.5785 | 3 | 86.56 | 1 | 61367 | 01122020\_RID\_2209\_BuCaKA02.raw | 7.9367E7 | 1 | 1 | 439 | 470 |  |  | PEAKS DB |
| A.DIVINDLSLIHQLPK.N | N | 74.80 | 1716.9774 | 15 | 0.7 | 859.4966 | 2 | 66.71 | 1 | 45159 | 01122020\_RID\_2209\_BuCaKA02.raw | 1.2111E9 | 3 | 3 | 410 | 424 |  |  | PEAKS DB |
| R.IYFAGEYTAR.V | N | 69.43 | 1189.5768 | 10 | 1.3 | 595.7964 | 2 | 33.46 | 1 | 18735 | 01122020\_RID\_2209\_BuCaKA02.raw | 8.9761E7 | 3 | 3 | 471 | 480 |  |  | PEAKS DB |
| R.VAYQTPAKTLSYVTADYVIVC(+57.02)ATSR.A | N | 64.46 | 2776.4106 | 25 | 0.4 | 926.4779 | 3 | 63.60 | 1 | 42220 | 01122020\_RID\_2209\_BuCaKA02.raw | 2.3369E7 | 1 | 1 | 292 | 316 | Carbamidomethylation | C21:Carbamidomethylation:1000.00 | PEAKS DB |
| L.TSFTPYQFQDYSETVAAPVGR.I | N | 61.49 | 2363.1069 | 21 | 0.5 | 1182.5613 | 2 | 62.44 | 1 | 41443 | 01122020\_RID\_2209\_BuCaKA02.raw | 4.6986E7 | 1 | 1 | 450 | 470 |  |  | PEAKS DB |
| F.GLKLNEFFQENENAWYFIR.N | N | 48.64 | 2417.1804 | 19 | 2.0 | 806.7357 | 3 | 77.91 | 1 | 54188 | 01122020\_RID\_2209\_BuCaKA02.raw | 1.6173E6 | 1 | 1 | 125 | 143 |  |  | PEAKS DB |
| K.KWSLDKYTMGALTSFTPYQFQDYSETVAAPVGR.I | N | 45.61 | 3756.8132 | 33 | 5.2 | 940.2155 | 4 | 77.17 | 1 | 53620 | 01122020\_RID\_2209\_BuCaKA02.raw | 3.1936E6 | 1 | 1 | 438 | 470 |  |  | PEAKS DB |
| K.WSLDKYTMGALTSFTPYQF.Q | N | 43.76 | 2255.0608 | 19 | 0.4 | 1128.5381 | 2 | 94.20 | 1 | 67517 | 01122020\_RID\_2209\_BuCaKA02.raw | 1.3712E7 | 1 | 1 | 439 | 457 |  |  | PEAKS DB |
| K.FGLKLNEFFQENENAWYFIRNIR.K | N | 43.46 | 2947.4768 | 23 | 1.5 | 737.8776 | 4 | 82.38 | 1 | 57872 | 01122020\_RID\_2209\_BuCaKA02.raw | 3.2121E6 | 1 | 1 | 124 | 146 |  |  | PEAKS DB |
| K.FGLKLNEFFQENENAWYFIR.N | N | 43.42 | 2564.2488 | 20 | 4.2 | 855.7604 | 3 | 85.11 | 1 | 60048 | 01122020\_RID\_2209\_BuCaKA02.raw | 1.0085E7 | 1 | 1 | 124 | 143 |  |  | PEAKS DB |
| total 12 peptides |  |  |  |  |  |  |  |  |  |  |  |  |  |  |  |  |  |  |  |
| --- | --- | --- | --- | --- | --- | --- | --- | --- | --- | --- | --- | --- | --- | --- | --- | --- | --- | --- | --- |

A8QL51.1

back to list

  

| Protein Coverage
| Supporting Peptides
|

Protein Coverage:

Supporting Peptides:

| Peptide | Uniq | -10lgP | Mass | Length | ppm | m/z | z | RT | Fraction | Scan | Source File | Area BRom | #Feature | #Feature BRom | Start | End | PTM | AScore | Found By |
| --- | --- | --- | --- | --- | --- | --- | --- | --- | --- | --- | --- | --- | --- | --- | --- | --- | --- | --- | --- |
| R.IYFAGEYTARVHGWLDSTIKSGLTAAR.D | Y | 85.73 | 2982.5352 | 27 | 2.2 | 746.6427 | 4 | 62.50 | 1 | 41757 | 01122020\_RID\_2209\_BuCaKA02.raw | 1.0441E8 | 5 | 5 | 471 | 497 |  |  | PEAKS DB |
| R.VHGWLDSTIKSGLTAAR.D | Y | 85.19 | 1810.9690 | 17 | 1.5 | 604.6645 | 3 | 36.01 | 1 | 20499 | 01122020\_RID\_2209\_BuCaKA02.raw | 1.1031E8 | 3 | 3 | 481 | 497 |  |  | PEAKS DB |
| K.WSLDKYTMGALTSFTPYQFQDYSETVAAPVGR.I | N | 77.95 | 3628.7183 | 32 | -1.3 | 1210.5785 | 3 | 86.56 | 1 | 61367 | 01122020\_RID\_2209\_BuCaKA02.raw | 7.9367E7 | 1 | 1 | 439 | 470 |  |  | PEAKS DB |
| A.DIVINDLSLIHQLPK.N | N | 74.80 | 1716.9774 | 15 | 0.7 | 859.4966 | 2 | 66.71 | 1 | 45159 | 01122020\_RID\_2209\_BuCaKA02.raw | 1.2111E9 | 3 | 3 | 410 | 424 |  |  | PEAKS DB |
| R.IYFAGEYTAR.V | N | 69.43 | 1189.5768 | 10 | 1.3 | 595.7964 | 2 | 33.46 | 1 | 18735 | 01122020\_RID\_2209\_BuCaKA02.raw | 8.9761E7 | 3 | 3 | 471 | 480 |  |  | PEAKS DB |
| R.VAYQTPAKTLSYVTADYVIVC(+57.02)ATSR.A | N | 64.46 | 2776.4106 | 25 | 0.4 | 926.4779 | 3 | 63.60 | 1 | 42220 | 01122020\_RID\_2209\_BuCaKA02.raw | 2.3369E7 | 1 | 1 | 292 | 316 | Carbamidomethylation | C21:Carbamidomethylation:1000.00 | PEAKS DB |
| L.TSFTPYQFQDYSETVAAPVGR.I | N | 61.49 | 2363.1069 | 21 | 0.5 | 1182.5613 | 2 | 62.44 | 1 | 41443 | 01122020\_RID\_2209\_BuCaKA02.raw | 4.6986E7 | 1 | 1 | 450 | 470 |  |  | PEAKS DB |
| F.GLKLNEFFQENENAWYFIR.N | N | 48.64 | 2417.1804 | 19 | 2.0 | 806.7357 | 3 | 77.91 | 1 | 54188 | 01122020\_RID\_2209\_BuCaKA02.raw | 1.6173E6 | 1 | 1 | 125 | 143 |  |  | PEAKS DB |
| K.KWSLDKYTMGALTSFTPYQFQDYSETVAAPVGR.I | N | 45.61 | 3756.8132 | 33 | 5.2 | 940.2155 | 4 | 77.17 | 1 | 53620 | 01122020\_RID\_2209\_BuCaKA02.raw | 3.1936E6 | 1 | 1 | 438 | 470 |  |  | PEAKS DB |
| K.WSLDKYTMGALTSFTPYQF.Q | N | 43.76 | 2255.0608 | 19 | 0.4 | 1128.5381 | 2 | 94.20 | 1 | 67517 | 01122020\_RID\_2209\_BuCaKA02.raw | 1.3712E7 | 1 | 1 | 439 | 457 |  |  | PEAKS DB |
| K.FGLKLNEFFQENENAWYFIRNIR.K | N | 43.46 | 2947.4768 | 23 | 1.5 | 737.8776 | 4 | 82.38 | 1 | 57872 | 01122020\_RID\_2209\_BuCaKA02.raw | 3.2121E6 | 1 | 1 | 124 | 146 |  |  | PEAKS DB |
| K.FGLKLNEFFQENENAWYFIR.N | N | 43.42 | 2564.2488 | 20 | 4.2 | 855.7604 | 3 | 85.11 | 1 | 60048 | 01122020\_RID\_2209\_BuCaKA02.raw | 1.0085E7 | 1 | 1 | 124 | 143 |  |  | PEAKS DB |
| total 12 peptides |  |  |  |  |  |  |  |  |  |  |  |  |  |  |  |  |  |  |  |
| --- | --- | --- | --- | --- | --- | --- | --- | --- | --- | --- | --- | --- | --- | --- | --- | --- | --- | --- | --- |

ABU63164.1

back to list

  

| Protein Coverage
| Supporting Peptides
|

Protein Coverage:

Supporting Peptides:

| Peptide | Uniq | -10lgP | Mass | Length | ppm | m/z | z | RT | Fraction | Scan | Source File | Area BRom | #Feature | #Feature BRom | Start | End | PTM | AScore | Found By |
| --- | --- | --- | --- | --- | --- | --- | --- | --- | --- | --- | --- | --- | --- | --- | --- | --- | --- | --- | --- |
| S.YGC(+57.02)YC(+57.02)GYGGSGTPVDELDR.C | N | 91.39 | 2124.8516 | 19 | 0.2 | 1063.4332 | 2 | 41.66 | 1 | 24749 | 01122020\_RID\_2209\_BuCaKA02.raw | 2.5458E9 | 1 | 1 | 50 | 68 | Carbamidomethylation | C3:Carbamidomethylation:1000.00;C5:Carbamidomethylation:1000.00 | PEAKS DB |
| Y.C(+57.02)GYGGSGTPVDELDR.C | N | 83.79 | 1581.6729 | 15 | 0.8 | 791.8444 | 2 | 29.21 | 1 | 14986 | 01122020\_RID\_2209\_BuCaKA02.raw | 1.287E8 | 1 | 1 | 54 | 68 | Carbamidomethylation | C1:Carbamidomethylation:1000.00 | PEAKS DB |
| Y.GC(+57.02)YC(+57.02)GYGGSGTPVDELDR.C | N | 81.97 | 1961.7883 | 18 | 1.3 | 981.9027 | 2 | 36.23 | 1 | 20719 | 01122020\_RID\_2209\_BuCaKA02.raw | 1.0001E9 | 2 | 2 | 51 | 68 | Carbamidomethylation | C2:Carbamidomethylation:1000.00;C4:Carbamidomethylation:1000.00 | PEAKS DB |
| Y.VSYGC(+57.02)YC(+57.02)GYGGSGTPVDELDR.C | Y | 81.93 | 2310.9521 | 21 | -0.2 | 1156.4832 | 2 | 46.14 | 1 | 28319 | 01122020\_RID\_2209\_BuCaKA02.raw | 5.5639E8 | 1 | 1 | 48 | 68 | Carbamidomethylation | C5:Carbamidomethylation:1000.00;C7:Carbamidomethylation:1000.00 | PEAKS DB |
| C.YC(+57.02)GYGGSGTPVDELDR.C | N | 74.25 | 1744.7362 | 16 | 1.7 | 873.3768 | 2 | 34.92 | 1 | 19611 | 01122020\_RID\_2209\_BuCaKA02.raw | 1.162E7 | 1 | 1 | 53 | 68 | Carbamidomethylation | C2:Carbamidomethylation:1000.00 | PEAKS DB |
| G.C(+57.02)YC(+57.02)GYGGSGTPVDELDR.C | N | 74.02 | 1904.7668 | 17 | 1.3 | 953.3920 | 2 | 36.10 | 1 | 20597 | 01122020\_RID\_2209\_BuCaKA02.raw | 8.7199E6 | 1 | 1 | 52 | 68 | Carbamidomethylation | C1:Carbamidomethylation:1000.00;C3:Carbamidomethylation:1000.00 | PEAKS DB |
| C.GYGGSGTPVDELDR.C | N | 70.31 | 1421.6422 | 14 | 2.0 | 711.8298 | 2 | 27.36 | 1 | 13794 | 01122020\_RID\_2209\_BuCaKA02.raw | 2.8856E7 | 1 | 1 | 55 | 68 |  |  | PEAKS DB |
| H.YVSYGC(+57.02)YC(+57.02)GYGGSGTPVDELDR.C | Y | 65.44 | 2474.0154 | 22 | 0.7 | 1238.0159 | 2 | 50.09 | 1 | 31225 | 01122020\_RID\_2209\_BuCaKA02.raw | 6.6427E6 | 2 | 2 | 47 | 68 | Carbamidomethylation | C6:Carbamidomethylation:1000.00;C8:Carbamidomethylation:1000.00 | PEAKS DB |
| total 8 peptides |  |  |  |  |  |  |  |  |  |  |  |  |  |  |  |  |  |  |  |
| --- | --- | --- | --- | --- | --- | --- | --- | --- | --- | --- | --- | --- | --- | --- | --- | --- | --- | --- | --- |

ABU63166.1

back to list

  

| Protein Coverage
| Supporting Peptides
|

Protein Coverage:

Supporting Peptides:

| Peptide | Uniq | -10lgP | Mass | Length | ppm | m/z | z | RT | Fraction | Scan | Source File | Area BRom | #Feature | #Feature BRom | Start | End | PTM | AScore | Found By |
| --- | --- | --- | --- | --- | --- | --- | --- | --- | --- | --- | --- | --- | --- | --- | --- | --- | --- | --- | --- |
| S.YGC(+57.02)YC(+57.02)GYGGSGTPVDELDR.C | N | 91.39 | 2124.8516 | 19 | 0.2 | 1063.4332 | 2 | 41.66 | 1 | 24749 | 01122020\_RID\_2209\_BuCaKA02.raw | 2.5458E9 | 1 | 1 | 50 | 68 | Carbamidomethylation | C3:Carbamidomethylation:1000.00;C5:Carbamidomethylation:1000.00 | PEAKS DB |
| Y.C(+57.02)GYGGSGTPVDELDR.C | N | 83.79 | 1581.6729 | 15 | 0.8 | 791.8444 | 2 | 29.21 | 1 | 14986 | 01122020\_RID\_2209\_BuCaKA02.raw | 1.287E8 | 1 | 1 | 54 | 68 | Carbamidomethylation | C1:Carbamidomethylation:1000.00 | PEAKS DB |
| Y.GC(+57.02)YC(+57.02)GYGGSGTPVDELDR.C | N | 81.97 | 1961.7883 | 18 | 1.3 | 981.9027 | 2 | 36.23 | 1 | 20719 | 01122020\_RID\_2209\_BuCaKA02.raw | 1.0001E9 | 2 | 2 | 51 | 68 | Carbamidomethylation | C2:Carbamidomethylation:1000.00;C4:Carbamidomethylation:1000.00 | PEAKS DB |
| Y.VSYGC(+57.02)YC(+57.02)GYGGSGTPVDELDR.C | Y | 81.93 | 2310.9521 | 21 | -0.2 | 1156.4832 | 2 | 46.14 | 1 | 28319 | 01122020\_RID\_2209\_BuCaKA02.raw | 5.5639E8 | 1 | 1 | 48 | 68 | Carbamidomethylation | C5:Carbamidomethylation:1000.00;C7:Carbamidomethylation:1000.00 | PEAKS DB |
| C.YC(+57.02)GYGGSGTPVDELDR.C | N | 74.25 | 1744.7362 | 16 | 1.7 | 873.3768 | 2 | 34.92 | 1 | 19611 | 01122020\_RID\_2209\_BuCaKA02.raw | 1.162E7 | 1 | 1 | 53 | 68 | Carbamidomethylation | C2:Carbamidomethylation:1000.00 | PEAKS DB |
| G.C(+57.02)YC(+57.02)GYGGSGTPVDELDR.C | N | 74.02 | 1904.7668 | 17 | 1.3 | 953.3920 | 2 | 36.10 | 1 | 20597 | 01122020\_RID\_2209\_BuCaKA02.raw | 8.7199E6 | 1 | 1 | 52 | 68 | Carbamidomethylation | C1:Carbamidomethylation:1000.00;C3:Carbamidomethylation:1000.00 | PEAKS DB |
| C.GYGGSGTPVDELDR.C | N | 70.31 | 1421.6422 | 14 | 2.0 | 711.8298 | 2 | 27.36 | 1 | 13794 | 01122020\_RID\_2209\_BuCaKA02.raw | 2.8856E7 | 1 | 1 | 55 | 68 |  |  | PEAKS DB |
| H.YVSYGC(+57.02)YC(+57.02)GYGGSGTPVDELDR.C | Y | 65.44 | 2474.0154 | 22 | 0.7 | 1238.0159 | 2 | 50.09 | 1 | 31225 | 01122020\_RID\_2209\_BuCaKA02.raw | 6.6427E6 | 2 | 2 | 47 | 68 | Carbamidomethylation | C6:Carbamidomethylation:1000.00;C8:Carbamidomethylation:1000.00 | PEAKS DB |
| total 8 peptides |  |  |  |  |  |  |  |  |  |  |  |  |  |  |  |  |  |  |  |
| --- | --- | --- | --- | --- | --- | --- | --- | --- | --- | --- | --- | --- | --- | --- | --- | --- | --- | --- | --- |

T\_134

back to list

  

| Protein Coverage
| Supporting Peptides
|

Protein Coverage:

Supporting Peptides:

| Peptide | Uniq | -10lgP | Mass | Length | ppm | m/z | z | RT | Fraction | Scan | Source File | Area BRom | #Feature | #Feature BRom | Start | End | PTM | AScore | Found By |
| --- | --- | --- | --- | --- | --- | --- | --- | --- | --- | --- | --- | --- | --- | --- | --- | --- | --- | --- | --- |
| K.WSLDKYTMGALTSFTPYQFQDYSETVAAPVGR.I | N | 77.95 | 3628.7183 | 32 | -1.3 | 1210.5785 | 3 | 86.56 | 1 | 61367 | 01122020\_RID\_2209\_BuCaKA02.raw | 7.9367E7 | 1 | 1 | 335 | 366 |  |  | PEAKS DB |
| A.DIVINDLSLIHQLPK.N | N | 74.80 | 1716.9774 | 15 | 0.7 | 859.4966 | 2 | 66.71 | 1 | 45159 | 01122020\_RID\_2209\_BuCaKA02.raw | 1.2111E9 | 3 | 3 | 306 | 320 |  |  | PEAKS DB |
| K.SATKIFLTC(+57.02)TNKFWEADGIHGGK.S | Y | 73.77 | 2580.2795 | 23 | 0.7 | 646.0776 | 4 | 37.57 | 1 | 21836 | 01122020\_RID\_2209\_BuCaKA02.raw | 2.796E7 | 1 | 1 | 238 | 260 | Carbamidomethylation | C9:Carbamidomethylation:1000.00 | PEAKS DB |
| N.DLSLIHQLPKNEIQDLC(+57.02)YPSLIKK.W | Y | 71.41 | 2864.5469 | 24 | 0.9 | 717.1447 | 4 | 57.89 | 1 | 37618 | 01122020\_RID\_2209\_BuCaKA02.raw | 9.7766E6 | 1 | 1 | 311 | 334 | Carbamidomethylation | C17:Carbamidomethylation:1000.00 | PEAKS DB |
| R.IYFAGEYTAR.L | N | 69.43 | 1189.5768 | 10 | 1.3 | 595.7964 | 2 | 33.46 | 1 | 18735 | 01122020\_RID\_2209\_BuCaKA02.raw | 8.9761E7 | 3 | 3 | 367 | 376 |  |  | PEAKS DB |
| A.DIVINDLSLIHQLPKNEIQDLC(+57.02)YPSLIKK.W | Y | 64.59 | 3418.8533 | 29 | 1.6 | 855.7219 | 4 | 70.46 | 1 | 48074 | 01122020\_RID\_2209\_BuCaKA02.raw | 1.3475E9 | 3 | 3 | 306 | 334 | Carbamidomethylation | C22:Carbamidomethylation:1000.00 | PEAKS DB |
| R.VAYQTPAKTLSYVTADYVIVC(+57.02)ATSR.A | N | 64.46 | 2776.4106 | 25 | 0.4 | 926.4779 | 3 | 63.60 | 1 | 42220 | 01122020\_RID\_2209\_BuCaKA02.raw | 2.3369E7 | 1 | 1 | 188 | 212 | Carbamidomethylation | C21:Carbamidomethylation:1000.00 | PEAKS DB |
| L.TSFTPYQFQDYSETVAAPVGR.I | N | 61.49 | 2363.1069 | 21 | 0.5 | 1182.5613 | 2 | 62.44 | 1 | 41443 | 01122020\_RID\_2209\_BuCaKA02.raw | 4.6986E7 | 1 | 1 | 346 | 366 |  |  | PEAKS DB |
| F.GLKLNEFFQENENAWYFIR.N | N | 48.64 | 2417.1804 | 19 | 2.0 | 806.7357 | 3 | 77.91 | 1 | 54188 | 01122020\_RID\_2209\_BuCaKA02.raw | 1.6173E6 | 1 | 1 | 21 | 39 |  |  | PEAKS DB |
| K.KWSLDKYTMGALTSFTPYQFQDYSETVAAPVGR.I | N | 45.61 | 3756.8132 | 33 | 5.2 | 940.2155 | 4 | 77.17 | 1 | 53620 | 01122020\_RID\_2209\_BuCaKA02.raw | 3.1936E6 | 1 | 1 | 334 | 366 |  |  | PEAKS DB |
| K.WSLDKYTMGALTSFTPYQF.Q | N | 43.76 | 2255.0608 | 19 | 0.4 | 1128.5381 | 2 | 94.20 | 1 | 67517 | 01122020\_RID\_2209\_BuCaKA02.raw | 1.3712E7 | 1 | 1 | 335 | 353 |  |  | PEAKS DB |
| K.FGLKLNEFFQENENAWYFIRNIR.K | N | 43.46 | 2947.4768 | 23 | 1.5 | 737.8776 | 4 | 82.38 | 1 | 57872 | 01122020\_RID\_2209\_BuCaKA02.raw | 3.2121E6 | 1 | 1 | 20 | 42 |  |  | PEAKS DB |
| K.FGLKLNEFFQENENAWYFIR.N | N | 43.42 | 2564.2488 | 20 | 4.2 | 855.7604 | 3 | 85.11 | 1 | 60048 | 01122020\_RID\_2209\_BuCaKA02.raw | 1.0085E7 | 1 | 1 | 20 | 39 |  |  | PEAKS DB |
| total 13 peptides |  |  |  |  |  |  |  |  |  |  |  |  |  |  |  |  |  |  |  |
| --- | --- | --- | --- | --- | --- | --- | --- | --- | --- | --- | --- | --- | --- | --- | --- | --- | --- | --- | --- |

T\_137

back to list

  

| Protein Coverage
| Supporting Peptides
|

Protein Coverage:

Supporting Peptides:

| Peptide | Uniq | -10lgP | Mass | Length | ppm | m/z | z | RT | Fraction | Scan | Source File | Area BRom | #Feature | #Feature BRom | Start | End | PTM | AScore | Found By |
| --- | --- | --- | --- | --- | --- | --- | --- | --- | --- | --- | --- | --- | --- | --- | --- | --- | --- | --- | --- |
| K.WSLDKYTMGALTSFTPYQFQDYSETVAAPVGR.I | N | 77.95 | 3628.7183 | 32 | -1.3 | 1210.5785 | 3 | 86.56 | 1 | 61367 | 01122020\_RID\_2209\_BuCaKA02.raw | 7.9367E7 | 1 | 1 | 335 | 366 |  |  | PEAKS DB |
| A.DIVINDLSLIHQLPK.N | N | 74.80 | 1716.9774 | 15 | 0.7 | 859.4966 | 2 | 66.71 | 1 | 45159 | 01122020\_RID\_2209\_BuCaKA02.raw | 1.2111E9 | 3 | 3 | 306 | 320 |  |  | PEAKS DB |
| K.SATKIFLTC(+57.02)TNKFWEADGIHGGK.S | Y | 73.77 | 2580.2795 | 23 | 0.7 | 646.0776 | 4 | 37.57 | 1 | 21836 | 01122020\_RID\_2209\_BuCaKA02.raw | 2.796E7 | 1 | 1 | 238 | 260 | Carbamidomethylation | C9:Carbamidomethylation:1000.00 | PEAKS DB |
| N.DLSLIHQLPKNEIQDLC(+57.02)YPSLIKK.W | Y | 71.41 | 2864.5469 | 24 | 0.9 | 717.1447 | 4 | 57.89 | 1 | 37618 | 01122020\_RID\_2209\_BuCaKA02.raw | 9.7766E6 | 1 | 1 | 311 | 334 | Carbamidomethylation | C17:Carbamidomethylation:1000.00 | PEAKS DB |
| R.IYFAGEYTAR.L | N | 69.43 | 1189.5768 | 10 | 1.3 | 595.7964 | 2 | 33.46 | 1 | 18735 | 01122020\_RID\_2209\_BuCaKA02.raw | 8.9761E7 | 3 | 3 | 367 | 376 |  |  | PEAKS DB |
| A.DIVINDLSLIHQLPKNEIQDLC(+57.02)YPSLIKK.W | Y | 64.59 | 3418.8533 | 29 | 1.6 | 855.7219 | 4 | 70.46 | 1 | 48074 | 01122020\_RID\_2209\_BuCaKA02.raw | 1.3475E9 | 3 | 3 | 306 | 334 | Carbamidomethylation | C22:Carbamidomethylation:1000.00 | PEAKS DB |
| R.VAYQTPAKTLSYVTADYVIVC(+57.02)ATSR.A | N | 64.46 | 2776.4106 | 25 | 0.4 | 926.4779 | 3 | 63.60 | 1 | 42220 | 01122020\_RID\_2209\_BuCaKA02.raw | 2.3369E7 | 1 | 1 | 188 | 212 | Carbamidomethylation | C21:Carbamidomethylation:1000.00 | PEAKS DB |
| L.TSFTPYQFQDYSETVAAPVGR.I | N | 61.49 | 2363.1069 | 21 | 0.5 | 1182.5613 | 2 | 62.44 | 1 | 41443 | 01122020\_RID\_2209\_BuCaKA02.raw | 4.6986E7 | 1 | 1 | 346 | 366 |  |  | PEAKS DB |
| F.GLKLNEFFQENENAWYFIR.N | N | 48.64 | 2417.1804 | 19 | 2.0 | 806.7357 | 3 | 77.91 | 1 | 54188 | 01122020\_RID\_2209\_BuCaKA02.raw | 1.6173E6 | 1 | 1 | 21 | 39 |  |  | PEAKS DB |
| K.KWSLDKYTMGALTSFTPYQFQDYSETVAAPVGR.I | N | 45.61 | 3756.8132 | 33 | 5.2 | 940.2155 | 4 | 77.17 | 1 | 53620 | 01122020\_RID\_2209\_BuCaKA02.raw | 3.1936E6 | 1 | 1 | 334 | 366 |  |  | PEAKS DB |
| K.WSLDKYTMGALTSFTPYQF.Q | N | 43.76 | 2255.0608 | 19 | 0.4 | 1128.5381 | 2 | 94.20 | 1 | 67517 | 01122020\_RID\_2209\_BuCaKA02.raw | 1.3712E7 | 1 | 1 | 335 | 353 |  |  | PEAKS DB |
| K.FGLKLNEFFQENENAWYFIRNIR.K | N | 43.46 | 2947.4768 | 23 | 1.5 | 737.8776 | 4 | 82.38 | 1 | 57872 | 01122020\_RID\_2209\_BuCaKA02.raw | 3.2121E6 | 1 | 1 | 20 | 42 |  |  | PEAKS DB |
| K.FGLKLNEFFQENENAWYFIR.N | N | 43.42 | 2564.2488 | 20 | 4.2 | 855.7604 | 3 | 85.11 | 1 | 60048 | 01122020\_RID\_2209\_BuCaKA02.raw | 1.0085E7 | 1 | 1 | 20 | 39 |  |  | PEAKS DB |
| total 13 peptides |  |  |  |  |  |  |  |  |  |  |  |  |  |  |  |  |  |  |  |
| --- | --- | --- | --- | --- | --- | --- | --- | --- | --- | --- | --- | --- | --- | --- | --- | --- | --- | --- | --- |

T\_130

back to list

  

| Protein Coverage
| Supporting Peptides
|

Protein Coverage:

Supporting Peptides:

| Peptide | Uniq | -10lgP | Mass | Length | ppm | m/z | z | RT | Fraction | Scan | Source File | Area BRom | #Feature | #Feature BRom | Start | End | PTM | AScore | Found By |
| --- | --- | --- | --- | --- | --- | --- | --- | --- | --- | --- | --- | --- | --- | --- | --- | --- | --- | --- | --- |
| K.WSLDKYTMGALTSFTPYQFQDYSETVAAPVGR.I | N | 77.95 | 3628.7183 | 32 | -1.3 | 1210.5785 | 3 | 86.56 | 1 | 61367 | 01122020\_RID\_2209\_BuCaKA02.raw | 7.9367E7 | 1 | 1 | 335 | 366 |  |  | PEAKS DB |
| A.DIVINDLSLIHQLPK.N | N | 74.80 | 1716.9774 | 15 | 0.7 | 859.4966 | 2 | 66.71 | 1 | 45159 | 01122020\_RID\_2209\_BuCaKA02.raw | 1.2111E9 | 3 | 3 | 306 | 320 |  |  | PEAKS DB |
| K.SATKIFLTC(+57.02)TNKFWEADGIHGGK.S | Y | 73.77 | 2580.2795 | 23 | 0.7 | 646.0776 | 4 | 37.57 | 1 | 21836 | 01122020\_RID\_2209\_BuCaKA02.raw | 2.796E7 | 1 | 1 | 238 | 260 | Carbamidomethylation | C9:Carbamidomethylation:1000.00 | PEAKS DB |
| N.DLSLIHQLPKNEIQDLC(+57.02)YPSLIKK.W | Y | 71.41 | 2864.5469 | 24 | 0.9 | 717.1447 | 4 | 57.89 | 1 | 37618 | 01122020\_RID\_2209\_BuCaKA02.raw | 9.7766E6 | 1 | 1 | 311 | 334 | Carbamidomethylation | C17:Carbamidomethylation:1000.00 | PEAKS DB |
| R.IYFAGEYTAR.L | N | 69.43 | 1189.5768 | 10 | 1.3 | 595.7964 | 2 | 33.46 | 1 | 18735 | 01122020\_RID\_2209\_BuCaKA02.raw | 8.9761E7 | 3 | 3 | 367 | 376 |  |  | PEAKS DB |
| A.DIVINDLSLIHQLPKNEIQDLC(+57.02)YPSLIKK.W | Y | 64.59 | 3418.8533 | 29 | 1.6 | 855.7219 | 4 | 70.46 | 1 | 48074 | 01122020\_RID\_2209\_BuCaKA02.raw | 1.3475E9 | 3 | 3 | 306 | 334 | Carbamidomethylation | C22:Carbamidomethylation:1000.00 | PEAKS DB |
| R.VAYQTPAKTLSYVTADYVIVC(+57.02)ATSR.A | N | 64.46 | 2776.4106 | 25 | 0.4 | 926.4779 | 3 | 63.60 | 1 | 42220 | 01122020\_RID\_2209\_BuCaKA02.raw | 2.3369E7 | 1 | 1 | 188 | 212 | Carbamidomethylation | C21:Carbamidomethylation:1000.00 | PEAKS DB |
| L.TSFTPYQFQDYSETVAAPVGR.I | N | 61.49 | 2363.1069 | 21 | 0.5 | 1182.5613 | 2 | 62.44 | 1 | 41443 | 01122020\_RID\_2209\_BuCaKA02.raw | 4.6986E7 | 1 | 1 | 346 | 366 |  |  | PEAKS DB |
| F.GLKLNEFFQENENAWYFIR.N | N | 48.64 | 2417.1804 | 19 | 2.0 | 806.7357 | 3 | 77.91 | 1 | 54188 | 01122020\_RID\_2209\_BuCaKA02.raw | 1.6173E6 | 1 | 1 | 21 | 39 |  |  | PEAKS DB |
| K.KWSLDKYTMGALTSFTPYQFQDYSETVAAPVGR.I | N | 45.61 | 3756.8132 | 33 | 5.2 | 940.2155 | 4 | 77.17 | 1 | 53620 | 01122020\_RID\_2209\_BuCaKA02.raw | 3.1936E6 | 1 | 1 | 334 | 366 |  |  | PEAKS DB |
| K.WSLDKYTMGALTSFTPYQF.Q | N | 43.76 | 2255.0608 | 19 | 0.4 | 1128.5381 | 2 | 94.20 | 1 | 67517 | 01122020\_RID\_2209\_BuCaKA02.raw | 1.3712E7 | 1 | 1 | 335 | 353 |  |  | PEAKS DB |
| K.FGLKLNEFFQENENAWYFIRNIR.K | N | 43.46 | 2947.4768 | 23 | 1.5 | 737.8776 | 4 | 82.38 | 1 | 57872 | 01122020\_RID\_2209\_BuCaKA02.raw | 3.2121E6 | 1 | 1 | 20 | 42 |  |  | PEAKS DB |
| K.FGLKLNEFFQENENAWYFIR.N | N | 43.42 | 2564.2488 | 20 | 4.2 | 855.7604 | 3 | 85.11 | 1 | 60048 | 01122020\_RID\_2209\_BuCaKA02.raw | 1.0085E7 | 1 | 1 | 20 | 39 |  |  | PEAKS DB |
| total 13 peptides |  |  |  |  |  |  |  |  |  |  |  |  |  |  |  |  |  |  |  |
| --- | --- | --- | --- | --- | --- | --- | --- | --- | --- | --- | --- | --- | --- | --- | --- | --- | --- | --- | --- |

T\_22

back to list

  

| Protein Coverage
| Supporting Peptides
|

Protein Coverage:

Supporting Peptides:

| Peptide | Uniq | -10lgP | Mass | Length | ppm | m/z | z | RT | Fraction | Scan | Source File | Area BRom | #Feature | #Feature BRom | Start | End | PTM | AScore | Found By |
| --- | --- | --- | --- | --- | --- | --- | --- | --- | --- | --- | --- | --- | --- | --- | --- | --- | --- | --- | --- |
| K.TYSYDC(+57.02)SEGKLTC(+57.02)KDTAGTC(+57.02)ER.I | N | 86.11 | 2601.0781 | 22 | 1.5 | 651.2778 | 4 | 12.08 | 1 | 2046 | 01122020\_RID\_2209\_BuCaKA02.raw | 0 | 0 | 0 | 122 | 143 | Carbamidomethylation | C6:Carbamidomethylation:1000.00;C13:Carbamidomethylation:1000.00;C20:Carbamidomethylation:1000.00 | PEAKS DB |
| L.NLIQFSNLIQC(+57.02)ANHNSRPTWHYANYGC(+57.02)YC(+57.02)GK.G | Y | 81.64 | 3785.7039 | 31 | 0.4 | 947.4336 | 4 | 50.56 | 1 | 31551 | 01122020\_RID\_2209\_BuCaKA02.raw | 3.9844E10 | 5 | 5 | 56 | 86 | Carbamidomethylation | C11:Carbamidomethylation:1000.00;C27:Carbamidomethylation:1000.00;C29:Carbamidomethylation:1000.00 | PEAKS DB |
| R.C(+57.02)C(+57.02)QTHDNC(+57.02)YGEAEKLASC(+57.02)RPYYK.T | N | 77.20 | 2909.1990 | 23 | 0.8 | 728.3076 | 4 | 19.12 | 1 | 7427 | 01122020\_RID\_2209\_BuCaKA02.raw | 4.5345E7 | 2 | 2 | 99 | 121 | Carbamidomethylation | C1:Carbamidomethylation:1000.00;C2:Carbamidomethylation:1000.00;C8:Carbamidomethylation:1000.00;C18:Carbamidomethylation:1000.00 | PEAKS DB |
| K.GGGGTPVDDLDRC(+57.02)C(+57.02)QTHDNC(+57.02)YGEAEK.L | Y | 65.25 | 2910.1604 | 26 | 1.4 | 728.5484 | 4 | 16.22 | 1 | 5286 | 01122020\_RID\_2209\_BuCaKA02.raw | 2.7443E6 | 1 | 1 | 87 | 112 | Carbamidomethylation | C13:Carbamidomethylation:1000.00;C14:Carbamidomethylation:1000.00;C20:Carbamidomethylation:1000.00 | PEAKS DB |
| F.SNLIQC(+57.02)ANHNSRPTWHYANYGC(+57.02)YC(+57.02)GK.G | Y | 61.40 | 3170.3657 | 26 | 1.0 | 793.5995 | 4 | 22.40 | 1 | 9966 | 01122020\_RID\_2209\_BuCaKA02.raw | 1.1467E7 | 1 | 1 | 61 | 86 | Carbamidomethylation | C6:Carbamidomethylation:1000.00;C22:Carbamidomethylation:1000.00;C24:Carbamidomethylation:1000.00 | PEAKS DB |
| Q.C(+57.02)ANHNSRPTWHYANYGC(+57.02)YC(+57.02)GKGGGGTPVDDLDR.C | Y | 57.05 | 3754.5847 | 33 | 2.2 | 751.9259 | 5 | 21.02 | 1 | 8863 | 01122020\_RID\_2209\_BuCaKA02.raw | 2.1904E6 | 1 | 1 | 66 | 98 | Carbamidomethylation | C1:Carbamidomethylation:1000.00;C17:Carbamidomethylation:1000.00;C19:Carbamidomethylation:1000.00 | PEAKS DB |
| P.LNLIQFSNLIQC(+57.02)ANHNSRPTWHYANYGC(+57.02)YC(+57.02)GK.G | Y | 56.67 | 3898.7878 | 32 | 0.5 | 780.7653 | 5 | 58.45 | 1 | 37992 | 01122020\_RID\_2209\_BuCaKA02.raw | 9.8639E7 | 1 | 1 | 55 | 86 | Carbamidomethylation | C12:Carbamidomethylation:1000.00;C28:Carbamidomethylation:1000.00;C30:Carbamidomethylation:1000.00 | PEAKS DB |
| K.GGGGTPVDDLDRC(+57.02)C(+57.02)QTHDNC(+57.02)YGEAEKLASC(+57.02)RPYYK.T | Y | 46.28 | 4048.7197 | 35 | 1.1 | 1013.1884 | 4 | 29.89 | 1 | 15633 | 01122020\_RID\_2209\_BuCaKA02.raw | 5.2559E6 | 1 | 1 | 87 | 121 | Carbamidomethylation | C13:Carbamidomethylation:1000.00;C14:Carbamidomethylation:1000.00;C20:Carbamidomethylation:1000.00;C30:Carbamidomethylation:1000.00 | PEAKS DB |
| K.LASC(+57.02)RPYYKTYSYDC(+57.02)SEGKLTC(+57.02)K.D | N | 41.20 | 2849.2822 | 23 | 1.6 | 713.3290 | 4 | 14.97 | 1 | 4367 | 01122020\_RID\_2209\_BuCaKA02.raw | 1.3525E6 | 1 | 1 | 113 | 135 | Carbamidomethylation | C4:Carbamidomethylation:1000.00;C15:Carbamidomethylation:1000.00;C22:Carbamidomethylation:1000.00 | PEAKS DB |
| total 9 peptides |  |  |  |  |  |  |  |  |  |  |  |  |  |  |  |  |  |  |  |
| --- | --- | --- | --- | --- | --- | --- | --- | --- | --- | --- | --- | --- | --- | --- | --- | --- | --- | --- | --- |

T\_23

back to list

  

| Protein Coverage
| Supporting Peptides
|

Protein Coverage:

Supporting Peptides:

| Peptide | Uniq | -10lgP | Mass | Length | ppm | m/z | z | RT | Fraction | Scan | Source File | Area BRom | #Feature | #Feature BRom | Start | End | PTM | AScore | Found By |
| --- | --- | --- | --- | --- | --- | --- | --- | --- | --- | --- | --- | --- | --- | --- | --- | --- | --- | --- | --- |
| K.TYSYDC(+57.02)SEGKLTC(+57.02)KDTAGTC(+57.02)ER.I | N | 86.11 | 2601.0781 | 22 | 1.5 | 651.2778 | 4 | 12.08 | 1 | 2046 | 01122020\_RID\_2209\_BuCaKA02.raw | 0 | 0 | 0 | 122 | 143 | Carbamidomethylation | C6:Carbamidomethylation:1000.00;C13:Carbamidomethylation:1000.00;C20:Carbamidomethylation:1000.00 | PEAKS DB |
| L.NLIQFSNLIQC(+57.02)ANHNSRPTWHYANYGC(+57.02)YC(+57.02)GK.G | Y | 81.64 | 3785.7039 | 31 | 0.4 | 947.4336 | 4 | 50.56 | 1 | 31551 | 01122020\_RID\_2209\_BuCaKA02.raw | 3.9844E10 | 5 | 5 | 56 | 86 | Carbamidomethylation | C11:Carbamidomethylation:1000.00;C27:Carbamidomethylation:1000.00;C29:Carbamidomethylation:1000.00 | PEAKS DB |
| R.C(+57.02)C(+57.02)QTHDNC(+57.02)YGEAEKLASC(+57.02)RPYYK.T | N | 77.20 | 2909.1990 | 23 | 0.8 | 728.3076 | 4 | 19.12 | 1 | 7427 | 01122020\_RID\_2209\_BuCaKA02.raw | 4.5345E7 | 2 | 2 | 99 | 121 | Carbamidomethylation | C1:Carbamidomethylation:1000.00;C2:Carbamidomethylation:1000.00;C8:Carbamidomethylation:1000.00;C18:Carbamidomethylation:1000.00 | PEAKS DB |
| K.GGGGTPVDDLDRC(+57.02)C(+57.02)QTHDNC(+57.02)YGEAEK.L | Y | 65.25 | 2910.1604 | 26 | 1.4 | 728.5484 | 4 | 16.22 | 1 | 5286 | 01122020\_RID\_2209\_BuCaKA02.raw | 2.7443E6 | 1 | 1 | 87 | 112 | Carbamidomethylation | C13:Carbamidomethylation:1000.00;C14:Carbamidomethylation:1000.00;C20:Carbamidomethylation:1000.00 | PEAKS DB |
| F.SNLIQC(+57.02)ANHNSRPTWHYANYGC(+57.02)YC(+57.02)GK.G | Y | 61.40 | 3170.3657 | 26 | 1.0 | 793.5995 | 4 | 22.40 | 1 | 9966 | 01122020\_RID\_2209\_BuCaKA02.raw | 1.1467E7 | 1 | 1 | 61 | 86 | Carbamidomethylation | C6:Carbamidomethylation:1000.00;C22:Carbamidomethylation:1000.00;C24:Carbamidomethylation:1000.00 | PEAKS DB |
| Q.C(+57.02)ANHNSRPTWHYANYGC(+57.02)YC(+57.02)GKGGGGTPVDDLDR.C | Y | 57.05 | 3754.5847 | 33 | 2.2 | 751.9259 | 5 | 21.02 | 1 | 8863 | 01122020\_RID\_2209\_BuCaKA02.raw | 2.1904E6 | 1 | 1 | 66 | 98 | Carbamidomethylation | C1:Carbamidomethylation:1000.00;C17:Carbamidomethylation:1000.00;C19:Carbamidomethylation:1000.00 | PEAKS DB |
| P.LNLIQFSNLIQC(+57.02)ANHNSRPTWHYANYGC(+57.02)YC(+57.02)GK.G | Y | 56.67 | 3898.7878 | 32 | 0.5 | 780.7653 | 5 | 58.45 | 1 | 37992 | 01122020\_RID\_2209\_BuCaKA02.raw | 9.8639E7 | 1 | 1 | 55 | 86 | Carbamidomethylation | C12:Carbamidomethylation:1000.00;C28:Carbamidomethylation:1000.00;C30:Carbamidomethylation:1000.00 | PEAKS DB |
| K.GGGGTPVDDLDRC(+57.02)C(+57.02)QTHDNC(+57.02)YGEAEKLASC(+57.02)RPYYK.T | Y | 46.28 | 4048.7197 | 35 | 1.1 | 1013.1884 | 4 | 29.89 | 1 | 15633 | 01122020\_RID\_2209\_BuCaKA02.raw | 5.2559E6 | 1 | 1 | 87 | 121 | Carbamidomethylation | C13:Carbamidomethylation:1000.00;C14:Carbamidomethylation:1000.00;C20:Carbamidomethylation:1000.00;C30:Carbamidomethylation:1000.00 | PEAKS DB |
| K.LASC(+57.02)RPYYKTYSYDC(+57.02)SEGKLTC(+57.02)K.D | N | 41.20 | 2849.2822 | 23 | 1.6 | 713.3290 | 4 | 14.97 | 1 | 4367 | 01122020\_RID\_2209\_BuCaKA02.raw | 1.3525E6 | 1 | 1 | 113 | 135 | Carbamidomethylation | C4:Carbamidomethylation:1000.00;C15:Carbamidomethylation:1000.00;C22:Carbamidomethylation:1000.00 | PEAKS DB |
| total 9 peptides |  |  |  |  |  |  |  |  |  |  |  |  |  |  |  |  |  |  |  |
| --- | --- | --- | --- | --- | --- | --- | --- | --- | --- | --- | --- | --- | --- | --- | --- | --- | --- | --- | --- |

XP\_026570824.1

back to list

  

| Protein Coverage
| Supporting Peptides
|

Protein Coverage:

Supporting Peptides:

| Peptide | Uniq | -10lgP | Mass | Length | ppm | m/z | z | RT | Fraction | Scan | Source File | Area BRom | #Feature | #Feature BRom | Start | End | PTM | AScore | Found By |
| --- | --- | --- | --- | --- | --- | --- | --- | --- | --- | --- | --- | --- | --- | --- | --- | --- | --- | --- | --- |
| K.KVIDALTEAVNNLDDVAGALSK.L | Y | 92.53 | 2255.2009 | 22 | 1.9 | 752.7423 | 3 | 86.19 | 1 | 61055 | 01122020\_RID\_2209\_BuCaKA02.raw | 3.3221E8 | 5 | 5 | 62 | 83 |  |  | PEAKS DB |
| K.VIDALTEAVNNLDDVAGALSK.L | Y | 88.22 | 2127.1060 | 21 | 0.5 | 1064.5608 | 2 | 94.31 | 1 | 67756 | 01122020\_RID\_2209\_BuCaKA02.raw | 6.027E8 | 6 | 6 | 63 | 83 |  |  | PEAKS DB |
| K.VIDALTEAVNNLDDVAGALSKLSDLHAQKLR.V | Y | 82.13 | 3288.7676 | 31 | 7.2 | 658.7655 | 5 | 87.94 | 1 | 62442 | 01122020\_RID\_2209\_BuCaKA02.raw | 1.4563E6 | 1 | 1 | 63 | 93 |  |  | PEAKS DB |
| K.VIDALTEAVNNLDDVAGALSKLSDLHAQK.L | Y | 80.42 | 3019.5825 | 29 | 1.9 | 755.9044 | 4 | 94.03 | 1 | 67418 | 01122020\_RID\_2209\_BuCaKA02.raw | 3.117E7 | 2 | 2 | 63 | 91 |  |  | PEAKS DB |
| K.KVIDALTEAVNNLDDVAGALSKLSDLHAQK.L | Y | 79.44 | 3147.6775 | 30 | 2.2 | 787.9283 | 4 | 88.58 | 1 | 62958 | 01122020\_RID\_2209\_BuCaKA02.raw | 5.7103E6 | 1 | 1 | 62 | 91 |  |  | PEAKS DB |
| V.IDALTEAVNNLDDVAGALSK.L | Y | 69.06 | 2028.0375 | 20 | 7.2 | 1015.0333 | 2 | 93.27 | 1 | 66932 | 01122020\_RID\_2209\_BuCaKA02.raw | 7.2996E6 | 1 | 1 | 64 | 83 |  |  | PEAKS DB |
| K.NAELYGAETLTRLFSAHPTTK.T | Y | 64.98 | 2319.1858 | 21 | 1.1 | 774.0701 | 3 | 50.12 | 1 | 31206 | 01122020\_RID\_2209\_BuCaKA02.raw | 1.9726E7 | 1 | 1 | 21 | 41 |  |  | PEAKS DB |
| total 7 peptides |  |  |  |  |  |  |  |  |  |  |  |  |  |  |  |  |  |  |  |
| --- | --- | --- | --- | --- | --- | --- | --- | --- | --- | --- | --- | --- | --- | --- | --- | --- | --- | --- | --- |

T\_3

back to list

  

| Protein Coverage
| Supporting Peptides
|

Protein Coverage:

Supporting Peptides:

| Peptide | Uniq | -10lgP | Mass | Length | ppm | m/z | z | RT | Fraction | Scan | Source File | Area BRom | #Feature | #Feature BRom | Start | End | PTM | AScore | Found By |
| --- | --- | --- | --- | --- | --- | --- | --- | --- | --- | --- | --- | --- | --- | --- | --- | --- | --- | --- | --- |
| K.C(+57.02)AATC(+57.02)PSEILSVK.I | Y | 80.44 | 1434.6847 | 13 | 2.2 | 718.3512 | 2 | 33.56 | 1 | 18621 | 01122020\_RID\_2209\_BuCaKA02.raw | 1.5875E8 | 1 | 1 | 66 | 78 | Carbamidomethylation | C1:Carbamidomethylation:1000.00;C5:Carbamidomethylation:1000.00 | PEAKS DB |
| K.TC(+57.02)PFHTC(+57.02)PDSETC(+57.02)PDGKNIC(+57.02)VKLSWLAVR.G | Y | 79.58 | 3447.5833 | 29 | 0.8 | 862.9038 | 4 | 46.60 | 1 | 28650 | 01122020\_RID\_2209\_BuCaKA02.raw | 4.1614E8 | 3 | 3 | 26 | 54 | Carbamidomethylation | C2:Carbamidomethylation:1000.00;C7:Carbamidomethylation:1000.00;C13:Carbamidomethylation:1000.00;C20:Carbamidomethylation:1000.00 | PEAKS DB |
| K.LSWLAVRGDGIKHEIR.R | Y | 66.87 | 1849.0322 | 16 | 0.9 | 617.3519 | 3 | 25.62 | 1 | 12391 | 01122020\_RID\_2209\_BuCaKA02.raw | 1.4394E8 | 1 | 1 | 48 | 63 |  |  | PEAKS DB |
| K.C(+57.02)AATC(+57.02)PSEILSVKIF.C | Y | 65.31 | 1694.8372 | 15 | 1.2 | 848.4269 | 2 | 71.09 | 1 | 48488 | 01122020\_RID\_2209\_BuCaKA02.raw | 9.8971E8 | 2 | 2 | 66 | 80 | Carbamidomethylation | C1:Carbamidomethylation:1000.00;C5:Carbamidomethylation:1000.00 | PEAKS DB |
| R.KC(+57.02)AATC(+57.02)PSEILSVKIF.C | Y | 62.65 | 1822.9321 | 16 | 0.9 | 912.4741 | 2 | 54.81 | 1 | 35125 | 01122020\_RID\_2209\_BuCaKA02.raw | 3.7928E8 | 2 | 2 | 65 | 80 | Carbamidomethylation | C2:Carbamidomethylation:1000.00;C6:Carbamidomethylation:1000.00 | PEAKS DB |
| K.NIC(+57.02)VKLSWLAVR.G | Y | 56.86 | 1457.8176 | 12 | 1.2 | 729.9170 | 2 | 52.74 | 1 | 33325 | 01122020\_RID\_2209\_BuCaKA02.raw | 1.4089E7 | 2 | 2 | 43 | 54 | Carbamidomethylation | C3:Carbamidomethylation:1000.00 | PEAKS DB |
| K.TC(+57.02)PFHTC(+57.02)PDSETC(+57.02)PDGKNIC(+57.02)VK.L | Y | 48.95 | 2622.0972 | 22 | 2.2 | 875.0416 | 3 | 16.13 | 1 | 5233 | 01122020\_RID\_2209\_BuCaKA02.raw | 4.1051E5 | 1 | 1 | 26 | 47 | Carbamidomethylation | C2:Carbamidomethylation:1000.00;C7:Carbamidomethylation:1000.00;C13:Carbamidomethylation:1000.00;C20:Carbamidomethylation:1000.00 | PEAKS DB |
| K.C(+57.02)AATC(+57.02)PSEILSVKI.F | Y | 47.99 | 1547.7687 | 14 | 2.8 | 774.8938 | 2 | 54.68 | 1 | 34941 | 01122020\_RID\_2209\_BuCaKA02.raw | 0 | 0 | 0 | 66 | 79 | Carbamidomethylation | C1:Carbamidomethylation:1000.00;C5:Carbamidomethylation:1000.00 | PEAKS DB |
| R.KC(+57.02)AATC(+57.02)PSEILSVKIFC(+57.02)C(+57.02)TTDNC(+57.02)ND | Y | 45.26 | 2963.2593 | 25 | 0.9 | 988.7612 | 3 | 56.20 | 1 | 36392 | 01122020\_RID\_2209\_BuCaKA02.raw | 2.0137E9 | 2 | 2 | 65 | 89 | Carbamidomethylation | C2:Carbamidomethylation:1000.00;C6:Carbamidomethylation:1000.00;C17:Carbamidomethylation:1000.00;C18:Carbamidomethylation:1000.00;C23:Carbamidomethylation:1000.00 | PEAKS DB |
| total 9 peptides |  |  |  |  |  |  |  |  |  |  |  |  |  |  |  |  |  |  |  |
| --- | --- | --- | --- | --- | --- | --- | --- | --- | --- | --- | --- | --- | --- | --- | --- | --- | --- | --- | --- |

XP\_026548868.1

back to list

  

| Protein Coverage
| Supporting Peptides
|

Protein Coverage:

Supporting Peptides:

| Peptide | Uniq | -10lgP | Mass | Length | ppm | m/z | z | RT | Fraction | Scan | Source File | Area BRom | #Feature | #Feature BRom | Start | End | PTM | AScore | Found By |
| --- | --- | --- | --- | --- | --- | --- | --- | --- | --- | --- | --- | --- | --- | --- | --- | --- | --- | --- | --- |
| R.FFTNFGNLSNAAAIQSNAQVK.A | Y | 74.30 | 2241.1177 | 21 | 1.0 | 748.0472 | 3 | 56.57 | 1 | 36662 | 01122020\_RID\_2209\_BuCaKA02.raw | 1.7745E8 | 2 | 2 | 42 | 62 |  |  | PEAKS DB |
| F.FTNFGNLSNAAAIQSNAQVK.A | Y | 66.04 | 2094.0493 | 20 | 1.4 | 1048.0334 | 2 | 45.00 | 1 | 27292 | 01122020\_RID\_2209\_BuCaKA02.raw | 2.4851E7 | 1 | 1 | 43 | 62 |  |  | PEAKS DB |
| R.LLIVYPWTQR.F | N | 63.20 | 1287.7339 | 10 | 1.2 | 644.8750 | 2 | 65.18 | 1 | 43507 | 01122020\_RID\_2209\_BuCaKA02.raw | 2.7712E7 | 1 | 1 | 32 | 41 |  |  | PEAKS DB |
| F.FTNFGNLSNAAAIQSNAQVKAHGKK.V | Y | 61.51 | 2615.3567 | 25 | -1.8 | 654.8453 | 4 | 20.67 | 1 | 8650 | 01122020\_RID\_2209\_BuCaKA02.raw | 0 | 0 | 0 | 43 | 67 |  |  | PEAKS DB |
| R.FFTNFGNLSNAAAIQSNAQVKAHGKK.V | Y | 61.31 | 2762.4253 | 26 | 2.2 | 691.6151 | 4 | 29.68 | 1 | 15412 | 01122020\_RID\_2209\_BuCaKA02.raw | 5.2857E7 | 1 | 1 | 42 | 67 |  |  | PEAKS DB |
| F.FTNFGNLSNAAAIQSNAQVKAHGK.K | Y | 60.58 | 2487.2617 | 24 | 0.5 | 622.8230 | 4 | 26.88 | 1 | 13510 | 01122020\_RID\_2209\_BuCaKA02.raw | 8.3516E6 | 1 | 1 | 43 | 66 |  |  | PEAKS DB |
| R.FFTNFGNLSNAAAIQSNAQVKAHGK.K | Y | 58.51 | 2634.3303 | 25 | 3.0 | 879.1200 | 3 | 37.01 | 1 | 21359 | 01122020\_RID\_2209\_BuCaKA02.raw | 2.9147E7 | 2 | 2 | 42 | 66 |  |  | PEAKS DB |
| K.LHVDPVNFKLLGDIL.L | N | 58.44 | 1691.9609 | 15 | 1.1 | 846.9886 | 2 | 78.56 | 1 | 54664 | 01122020\_RID\_2209\_BuCaKA02.raw | 1.1278E7 | 2 | 2 | 97 | 111 |  |  | PEAKS DB |
| K.LSELHC(+57.02)DKLHVDPVNFKLLGDILL.T | N | 55.08 | 2787.4993 | 24 | 1.2 | 697.8829 | 4 | 73.38 | 1 | 50333 | 01122020\_RID\_2209\_BuCaKA02.raw | 6.3167E7 | 1 | 1 | 89 | 112 | Carbamidomethylation | C6:Carbamidomethylation:1000.00 | PEAKS DB |
| K.LSELHC(+57.02)DKLHVDPVNFKLLGDILLTVL.A | N | 53.46 | 3100.6995 | 27 | 0.8 | 776.1828 | 4 | 94.78 | 1 | 68135 | 01122020\_RID\_2209\_BuCaKA02.raw | 1.4117E7 | 1 | 1 | 89 | 115 | Carbamidomethylation | C6:Carbamidomethylation:1000.00 | PEAKS DB |
| R.LLIVYPWTQRF.F | N | 51.09 | 1434.8024 | 11 | 1.2 | 718.4093 | 2 | 76.20 | 1 | 52645 | 01122020\_RID\_2209\_BuCaKA02.raw | 7.6335E7 | 1 | 1 | 32 | 42 |  |  | PEAKS DB |
| K.LHVDPVNFKLLGDILL.T | N | 48.69 | 1805.0450 | 16 | 3.5 | 602.6910 | 3 | 85.50 | 1 | 60357 | 01122020\_RID\_2209\_BuCaKA02.raw | 3.6866E6 | 2 | 2 | 97 | 112 |  |  | PEAKS DB |
| K.LSELHC(+57.02)DKLHVDPVNFKLLGDIL.L | N | 40.85 | 2674.4153 | 23 | -0.1 | 1338.2148 | 2 | 67.54 | 1 | 45571 | 01122020\_RID\_2209\_BuCaKA02.raw | 3.3985E6 | 1 | 1 | 89 | 111 | Carbamidomethylation | C6:Carbamidomethylation:1000.00 | PEAKS DB |
| total 13 peptides |  |  |  |  |  |  |  |  |  |  |  |  |  |  |  |  |  |  |  |
| --- | --- | --- | --- | --- | --- | --- | --- | --- | --- | --- | --- | --- | --- | --- | --- | --- | --- | --- | --- |

T\_141

back to list

  

| Protein Coverage
| Supporting Peptides
|

Protein Coverage:

Supporting Peptides:

| Peptide | Uniq | -10lgP | Mass | Length | ppm | m/z | z | RT | Fraction | Scan | Source File | Area BRom | #Feature | #Feature BRom | Start | End | PTM | AScore | Found By |
| --- | --- | --- | --- | --- | --- | --- | --- | --- | --- | --- | --- | --- | --- | --- | --- | --- | --- | --- | --- |
| R.DRPQC(+57.02)ILNKPVITDIVAPAIC(+57.02)GNYFVEVGEEC(+57.02)DC(+57.02)GSPR.D | Y | 84.43 | 4348.0386 | 38 | 0.6 | 1088.0176 | 4 | 76.30 | 1 | 52783 | 01122020\_RID\_2209\_BuCaKA02.raw | 1.838E9 | 4 | 4 | 54 | 91 | Carbamidomethylation | C5:Carbamidomethylation:1000.00;C21:Carbamidomethylation:1000.00;C32:Carbamidomethylation:1000.00;C34:Carbamidomethylation:1000.00 | PEAKS DB |
| Y.FVEVGEEC(+57.02)DC(+57.02)GSPR.D | N | 72.30 | 1639.6606 | 14 | 1.0 | 820.8384 | 2 | 21.06 | 1 | 9089 | 01122020\_RID\_2209\_BuCaKA02.raw | 1.1383E8 | 1 | 1 | 78 | 91 | Carbamidomethylation | C8:Carbamidomethylation:1000.00;C10:Carbamidomethylation:1000.00 | PEAKS DB |
| R.YLLRDRPQC(+57.02)ILNKPVITDIVAPAIC(+57.02)GNYFVEVGEEC(+57.02)DC(+57.02)GSPR.D | Y | 60.01 | 4893.3711 | 42 | 1.5 | 979.6830 | 5 | 71.03 | 1 | 48311 | 01122020\_RID\_2209\_BuCaKA02.raw | 4.4886E8 | 4 | 4 | 50 | 91 | Carbamidomethylation | C9:Carbamidomethylation:1000.00;C25:Carbamidomethylation:1000.00;C36:Carbamidomethylation:1000.00;C38:Carbamidomethylation:1000.00 | PEAKS DB |
| R.DRPQC(+57.02)ILNKPVITDIVAPAIC(+57.02)GNY.F | Y | 59.20 | 2726.3884 | 24 | 0.5 | 909.8039 | 3 | 71.13 | 1 | 48508 | 01122020\_RID\_2209\_BuCaKA02.raw | 2.4764E10 | 3 | 3 | 54 | 77 | Carbamidomethylation | C5:Carbamidomethylation:1000.00;C21:Carbamidomethylation:1000.00 | PEAKS DB |
| R.YLLRDRPQC(+57.02)ILNKPVITDIVAPAIC(+57.02)GNYFVEVGEEC(+57.02)DC(+57.02)GSPRDC(+57.02)R.S | Y | 54.72 | 5324.5298 | 45 | 3.7 | 888.4322 | 6 | 66.13 | 1 | 44381 | 01122020\_RID\_2209\_BuCaKA02.raw | 2.1209E8 | 2 | 2 | 50 | 94 | Carbamidomethylation | C9:Carbamidomethylation:1000.00;C25:Carbamidomethylation:1000.00;C36:Carbamidomethylation:1000.00;C38:Carbamidomethylation:1000.00;C44:Carbamidomethylation:1000.00 | PEAKS DB |
| T.DIVAPAIC(+57.02)GNYFVEVGEEC(+57.02)DC(+57.02)GSPR.D | N | 54.18 | 2813.2095 | 25 | 0.1 | 938.7439 | 3 | 77.95 | 1 | 54032 | 01122020\_RID\_2209\_BuCaKA02.raw | 1.7917E7 | 3 | 3 | 67 | 91 | Carbamidomethylation | C8:Carbamidomethylation:1000.00;C19:Carbamidomethylation:1000.00;C21:Carbamidomethylation:1000.00 | PEAKS DB |
| Y.FVEVGEEC(+57.02)DC(+57.02)GSPRDC(+57.02)R.S | N | 50.32 | 2070.8193 | 17 | 1.2 | 691.2812 | 3 | 12.90 | 1 | 3067 | 01122020\_RID\_2209\_BuCaKA02.raw | 3.9576E6 | 1 | 1 | 78 | 94 | Carbamidomethylation | C8:Carbamidomethylation:1000.00;C10:Carbamidomethylation:1000.00;C16:Carbamidomethylation:1000.00 | PEAKS DB |
| R.DRPQC(+57.02)ILNKPVITDIVAPAIC(+57.02)G.N | Y | 48.98 | 2449.2820 | 22 | 2.4 | 817.4365 | 3 | 66.80 | 1 | 44948 | 01122020\_RID\_2209\_BuCaKA02.raw | 6.3297E7 | 1 | 1 | 54 | 75 | Carbamidomethylation | C5:Carbamidomethylation:1000.00;C21:Carbamidomethylation:1000.00 | PEAKS DB |
| R.YLLRDRPQC(+57.02)ILNKPVITDIVAPAIC(+57.02)GNY.F | Y | 42.83 | 3271.7209 | 28 | 1.2 | 818.9385 | 4 | 65.27 | 1 | 43526 | 01122020\_RID\_2209\_BuCaKA02.raw | 2.736E9 | 1 | 1 | 50 | 77 | Carbamidomethylation | C9:Carbamidomethylation:1000.00;C25:Carbamidomethylation:1000.00 | PEAKS DB |
| R.DRPQC(+57.02)ILNKPVITDIVAPAIC(+57.02)GNYF.V | Y | 40.33 | 2873.4568 | 25 | 0.9 | 958.8270 | 3 | 80.23 | 1 | 56127 | 01122020\_RID\_2209\_BuCaKA02.raw | 1.0367E9 | 1 | 1 | 54 | 78 | Carbamidomethylation | C5:Carbamidomethylation:1000.00;C21:Carbamidomethylation:1000.00 | PEAKS DB |
| R.DRPQC(+57.02)ILNKPVITDIVAPAIC(+57.02)GNYFVEVGEEC(+57.02)DC(+57.02)GSPRDC(+57.02)R.S | Y | 39.70 | 4779.1973 | 41 | 2.1 | 1195.8091 | 4 | 70.29 | 1 | 47545 | 01122020\_RID\_2209\_BuCaKA02.raw | 2.5647E8 | 1 | 1 | 54 | 94 | Carbamidomethylation | C5:Carbamidomethylation:1000.00;C21:Carbamidomethylation:1000.00;C32:Carbamidomethylation:1000.00;C34:Carbamidomethylation:1000.00;C40:Carbamidomethylation:1000.00 | PEAKS DB |
| total 11 peptides |  |  |  |  |  |  |  |  |  |  |  |  |  |  |  |  |  |  |  |
| --- | --- | --- | --- | --- | --- | --- | --- | --- | --- | --- | --- | --- | --- | --- | --- | --- | --- | --- | --- |

T\_140

back to list

  

| Protein Coverage
| Supporting Peptides
|

Protein Coverage:

Supporting Peptides:

| Peptide | Uniq | -10lgP | Mass | Length | ppm | m/z | z | RT | Fraction | Scan | Source File | Area BRom | #Feature | #Feature BRom | Start | End | PTM | AScore | Found By |
| --- | --- | --- | --- | --- | --- | --- | --- | --- | --- | --- | --- | --- | --- | --- | --- | --- | --- | --- | --- |
| R.DRPQC(+57.02)ILNKPVITDIVAPAIC(+57.02)GNYFVEVGEEC(+57.02)DC(+57.02)GSPR.D | Y | 84.43 | 4348.0386 | 38 | 0.6 | 1088.0176 | 4 | 76.30 | 1 | 52783 | 01122020\_RID\_2209\_BuCaKA02.raw | 1.838E9 | 4 | 4 | 54 | 91 | Carbamidomethylation | C5:Carbamidomethylation:1000.00;C21:Carbamidomethylation:1000.00;C32:Carbamidomethylation:1000.00;C34:Carbamidomethylation:1000.00 | PEAKS DB |
| Y.FVEVGEEC(+57.02)DC(+57.02)GSPR.D | N | 72.30 | 1639.6606 | 14 | 1.0 | 820.8384 | 2 | 21.06 | 1 | 9089 | 01122020\_RID\_2209\_BuCaKA02.raw | 1.1383E8 | 1 | 1 | 78 | 91 | Carbamidomethylation | C8:Carbamidomethylation:1000.00;C10:Carbamidomethylation:1000.00 | PEAKS DB |
| R.YLLRDRPQC(+57.02)ILNKPVITDIVAPAIC(+57.02)GNYFVEVGEEC(+57.02)DC(+57.02)GSPR.D | Y | 60.01 | 4893.3711 | 42 | 1.5 | 979.6830 | 5 | 71.03 | 1 | 48311 | 01122020\_RID\_2209\_BuCaKA02.raw | 4.4886E8 | 4 | 4 | 50 | 91 | Carbamidomethylation | C9:Carbamidomethylation:1000.00;C25:Carbamidomethylation:1000.00;C36:Carbamidomethylation:1000.00;C38:Carbamidomethylation:1000.00 | PEAKS DB |
| R.DRPQC(+57.02)ILNKPVITDIVAPAIC(+57.02)GNY.F | Y | 59.20 | 2726.3884 | 24 | 0.5 | 909.8039 | 3 | 71.13 | 1 | 48508 | 01122020\_RID\_2209\_BuCaKA02.raw | 2.4764E10 | 3 | 3 | 54 | 77 | Carbamidomethylation | C5:Carbamidomethylation:1000.00;C21:Carbamidomethylation:1000.00 | PEAKS DB |
| R.YLLRDRPQC(+57.02)ILNKPVITDIVAPAIC(+57.02)GNYFVEVGEEC(+57.02)DC(+57.02)GSPRDC(+57.02)R.S | Y | 54.72 | 5324.5298 | 45 | 3.7 | 888.4322 | 6 | 66.13 | 1 | 44381 | 01122020\_RID\_2209\_BuCaKA02.raw | 2.1209E8 | 2 | 2 | 50 | 94 | Carbamidomethylation | C9:Carbamidomethylation:1000.00;C25:Carbamidomethylation:1000.00;C36:Carbamidomethylation:1000.00;C38:Carbamidomethylation:1000.00;C44:Carbamidomethylation:1000.00 | PEAKS DB |
| T.DIVAPAIC(+57.02)GNYFVEVGEEC(+57.02)DC(+57.02)GSPR.D | N | 54.18 | 2813.2095 | 25 | 0.1 | 938.7439 | 3 | 77.95 | 1 | 54032 | 01122020\_RID\_2209\_BuCaKA02.raw | 1.7917E7 | 3 | 3 | 67 | 91 | Carbamidomethylation | C8:Carbamidomethylation:1000.00;C19:Carbamidomethylation:1000.00;C21:Carbamidomethylation:1000.00 | PEAKS DB |
| Y.FVEVGEEC(+57.02)DC(+57.02)GSPRDC(+57.02)R.S | N | 50.32 | 2070.8193 | 17 | 1.2 | 691.2812 | 3 | 12.90 | 1 | 3067 | 01122020\_RID\_2209\_BuCaKA02.raw | 3.9576E6 | 1 | 1 | 78 | 94 | Carbamidomethylation | C8:Carbamidomethylation:1000.00;C10:Carbamidomethylation:1000.00;C16:Carbamidomethylation:1000.00 | PEAKS DB |
| R.DRPQC(+57.02)ILNKPVITDIVAPAIC(+57.02)G.N | Y | 48.98 | 2449.2820 | 22 | 2.4 | 817.4365 | 3 | 66.80 | 1 | 44948 | 01122020\_RID\_2209\_BuCaKA02.raw | 6.3297E7 | 1 | 1 | 54 | 75 | Carbamidomethylation | C5:Carbamidomethylation:1000.00;C21:Carbamidomethylation:1000.00 | PEAKS DB |
| R.YLLRDRPQC(+57.02)ILNKPVITDIVAPAIC(+57.02)GNY.F | Y | 42.83 | 3271.7209 | 28 | 1.2 | 818.9385 | 4 | 65.27 | 1 | 43526 | 01122020\_RID\_2209\_BuCaKA02.raw | 2.736E9 | 1 | 1 | 50 | 77 | Carbamidomethylation | C9:Carbamidomethylation:1000.00;C25:Carbamidomethylation:1000.00 | PEAKS DB |
| R.DRPQC(+57.02)ILNKPVITDIVAPAIC(+57.02)GNYF.V | Y | 40.33 | 2873.4568 | 25 | 0.9 | 958.8270 | 3 | 80.23 | 1 | 56127 | 01122020\_RID\_2209\_BuCaKA02.raw | 1.0367E9 | 1 | 1 | 54 | 78 | Carbamidomethylation | C5:Carbamidomethylation:1000.00;C21:Carbamidomethylation:1000.00 | PEAKS DB |
| R.DRPQC(+57.02)ILNKPVITDIVAPAIC(+57.02)GNYFVEVGEEC(+57.02)DC(+57.02)GSPRDC(+57.02)R.S | Y | 39.70 | 4779.1973 | 41 | 2.1 | 1195.8091 | 4 | 70.29 | 1 | 47545 | 01122020\_RID\_2209\_BuCaKA02.raw | 2.5647E8 | 1 | 1 | 54 | 94 | Carbamidomethylation | C5:Carbamidomethylation:1000.00;C21:Carbamidomethylation:1000.00;C32:Carbamidomethylation:1000.00;C34:Carbamidomethylation:1000.00;C40:Carbamidomethylation:1000.00 | PEAKS DB |
| total 11 peptides |  |  |  |  |  |  |  |  |  |  |  |  |  |  |  |  |  |  |  |
| --- | --- | --- | --- | --- | --- | --- | --- | --- | --- | --- | --- | --- | --- | --- | --- | --- | --- | --- | --- |

1PO8

back to list

  

| Protein Coverage
| Supporting Peptides
|

Protein Coverage:

Supporting Peptides:

| Peptide | Uniq | -10lgP | Mass | Length | ppm | m/z | z | RT | Fraction | Scan | Source File | Area BRom | #Feature | #Feature BRom | Start | End | PTM | AScore | Found By |
| --- | --- | --- | --- | --- | --- | --- | --- | --- | --- | --- | --- | --- | --- | --- | --- | --- | --- | --- | --- |
| R.FVC(+57.02)DC(+57.02)DRTAAIC(+57.02)FAK.A | Y | 90.11 | 1832.8008 | 15 | 0.5 | 917.4081 | 2 | 29.11 | 1 | 14827 | 01122020\_RID\_2209\_BuCaKA02.raw | 3.5728E10 | 21 | 21 | 87 | 101 | Carbamidomethylation | C3:Carbamidomethylation:1000.00;C5:Carbamidomethylation:1000.00;C12:Carbamidomethylation:1000.00 | PEAKS DB |
| K.C(+57.02)ARFVC(+57.02)DC(+57.02)DRTAAIC(+57.02)FAK.A | Y | 73.89 | 2219.9697 | 18 | 0.9 | 740.9979 | 3 | 24.07 | 1 | 11297 | 01122020\_RID\_2209\_BuCaKA02.raw | 4.6498E7 | 2 | 2 | 84 | 101 | Carbamidomethylation | C1:Carbamidomethylation:1000.00;C6:Carbamidomethylation:1000.00;C8:Carbamidomethylation:1000.00;C15:Carbamidomethylation:1000.00 | PEAKS DB |
| F.VC(+57.02)DC(+57.02)DRTAAIC(+57.02)FAK.A | Y | 62.00 | 1685.7324 | 14 | 0.3 | 843.8738 | 2 | 19.27 | 1 | 7662 | 01122020\_RID\_2209\_BuCaKA02.raw | 9.2148E6 | 1 | 1 | 88 | 101 | Carbamidomethylation | C2:Carbamidomethylation:1000.00;C4:Carbamidomethylation:1000.00;C11:Carbamidomethylation:1000.00 | PEAKS DB |
| R.FVC(+57.02)DC(+57.02)DRTAAIC(+57.02)FAKAPYN.T | Y | 57.69 | 2277.9968 | 19 | 1.0 | 760.3403 | 3 | 39.34 | 1 | 23331 | 01122020\_RID\_2209\_BuCaKA02.raw | 1.219E8 | 1 | 1 | 87 | 105 | Carbamidomethylation | C3:Carbamidomethylation:1000.00;C5:Carbamidomethylation:1000.00;C12:Carbamidomethylation:1000.00 | PEAKS DB |
| A.RFVC(+57.02)DC(+57.02)DRTAAIC(+57.02)FAK.A | Y | 57.32 | 1988.9019 | 16 | 1.4 | 663.9755 | 3 | 21.50 | 1 | 9222 | 01122020\_RID\_2209\_BuCaKA02.raw | 1.2424E7 | 1 | 1 | 86 | 101 | Carbamidomethylation | C4:Carbamidomethylation:1000.00;C6:Carbamidomethylation:1000.00;C13:Carbamidomethylation:1000.00 | PEAKS DB |
| C.DRTAAIC(+57.02)FAK.A | N | 55.24 | 1151.5757 | 10 | 2.7 | 576.7967 | 2 | 15.19 | 1 | 4454 | 01122020\_RID\_2209\_BuCaKA02.raw | 3.7655E5 | 1 | 1 | 92 | 101 | Carbamidomethylation | C7:Carbamidomethylation:1000.00 | PEAKS DB |
| C.DC(+57.02)DRTAAIC(+57.02)FAK.A | N | 47.16 | 1426.6333 | 12 | -2.2 | 714.3224 | 2 | 20.22 | 1 | 8374 | 01122020\_RID\_2209\_BuCaKA02.raw | 1.7388E6 | 1 | 1 | 90 | 101 | Carbamidomethylation | C2:Carbamidomethylation:1000.00;C9:Carbamidomethylation:1000.00 | PEAKS DB |
| R.FVC(+57.02)DC(+57.02)DRTAAIC(+57.02)F.A | N | 44.79 | 1633.6687 | 13 | 0.9 | 817.8423 | 2 | 47.45 | 1 | 29276 | 01122020\_RID\_2209\_BuCaKA02.raw | 0 | 0 | 0 | 87 | 99 | Carbamidomethylation | C3:Carbamidomethylation:1000.00;C5:Carbamidomethylation:1000.00;C12:Carbamidomethylation:1000.00 | PEAKS DB |
| D.C(+57.02)DRTAAIC(+57.02)FAK.A | N | 39.74 | 1311.6063 | 11 | 1.6 | 656.8115 | 2 | 12.51 | 1 | 2398 | 01122020\_RID\_2209\_BuCaKA02.raw | 5.3941E5 | 1 | 1 | 91 | 101 | Carbamidomethylation | C1:Carbamidomethylation:1000.00;C8:Carbamidomethylation:1000.00 | PEAKS DB |
| total 9 peptides |  |  |  |  |  |  |  |  |  |  |  |  |  |  |  |  |  |  |  |
| --- | --- | --- | --- | --- | --- | --- | --- | --- | --- | --- | --- | --- | --- | --- | --- | --- | --- | --- | --- |

1TC8

back to list

  

| Protein Coverage
| Supporting Peptides
|

Protein Coverage:

Supporting Peptides:

| Peptide | Uniq | -10lgP | Mass | Length | ppm | m/z | z | RT | Fraction | Scan | Source File | Area BRom | #Feature | #Feature BRom | Start | End | PTM | AScore | Found By |
| --- | --- | --- | --- | --- | --- | --- | --- | --- | --- | --- | --- | --- | --- | --- | --- | --- | --- | --- | --- |
| R.FVC(+57.02)DC(+57.02)DRTAAIC(+57.02)FAK.A | Y | 90.11 | 1832.8008 | 15 | 0.5 | 917.4081 | 2 | 29.11 | 1 | 14827 | 01122020\_RID\_2209\_BuCaKA02.raw | 3.5728E10 | 21 | 21 | 87 | 101 | Carbamidomethylation | C3:Carbamidomethylation:1000.00;C5:Carbamidomethylation:1000.00;C12:Carbamidomethylation:1000.00 | PEAKS DB |
| K.C(+57.02)ARFVC(+57.02)DC(+57.02)DRTAAIC(+57.02)FAK.A | Y | 73.89 | 2219.9697 | 18 | 0.9 | 740.9979 | 3 | 24.07 | 1 | 11297 | 01122020\_RID\_2209\_BuCaKA02.raw | 4.6498E7 | 2 | 2 | 84 | 101 | Carbamidomethylation | C1:Carbamidomethylation:1000.00;C6:Carbamidomethylation:1000.00;C8:Carbamidomethylation:1000.00;C15:Carbamidomethylation:1000.00 | PEAKS DB |
| F.VC(+57.02)DC(+57.02)DRTAAIC(+57.02)FAK.A | Y | 62.00 | 1685.7324 | 14 | 0.3 | 843.8738 | 2 | 19.27 | 1 | 7662 | 01122020\_RID\_2209\_BuCaKA02.raw | 9.2148E6 | 1 | 1 | 88 | 101 | Carbamidomethylation | C2:Carbamidomethylation:1000.00;C4:Carbamidomethylation:1000.00;C11:Carbamidomethylation:1000.00 | PEAKS DB |
| R.FVC(+57.02)DC(+57.02)DRTAAIC(+57.02)FAKAPYN.T | Y | 57.69 | 2277.9968 | 19 | 1.0 | 760.3403 | 3 | 39.34 | 1 | 23331 | 01122020\_RID\_2209\_BuCaKA02.raw | 1.219E8 | 1 | 1 | 87 | 105 | Carbamidomethylation | C3:Carbamidomethylation:1000.00;C5:Carbamidomethylation:1000.00;C12:Carbamidomethylation:1000.00 | PEAKS DB |
| A.RFVC(+57.02)DC(+57.02)DRTAAIC(+57.02)FAK.A | Y | 57.32 | 1988.9019 | 16 | 1.4 | 663.9755 | 3 | 21.50 | 1 | 9222 | 01122020\_RID\_2209\_BuCaKA02.raw | 1.2424E7 | 1 | 1 | 86 | 101 | Carbamidomethylation | C4:Carbamidomethylation:1000.00;C6:Carbamidomethylation:1000.00;C13:Carbamidomethylation:1000.00 | PEAKS DB |
| C.DRTAAIC(+57.02)FAK.A | N | 55.24 | 1151.5757 | 10 | 2.7 | 576.7967 | 2 | 15.19 | 1 | 4454 | 01122020\_RID\_2209\_BuCaKA02.raw | 3.7655E5 | 1 | 1 | 92 | 101 | Carbamidomethylation | C7:Carbamidomethylation:1000.00 | PEAKS DB |
| C.DC(+57.02)DRTAAIC(+57.02)FAK.A | N | 47.16 | 1426.6333 | 12 | -2.2 | 714.3224 | 2 | 20.22 | 1 | 8374 | 01122020\_RID\_2209\_BuCaKA02.raw | 1.7388E6 | 1 | 1 | 90 | 101 | Carbamidomethylation | C2:Carbamidomethylation:1000.00;C9:Carbamidomethylation:1000.00 | PEAKS DB |
| R.FVC(+57.02)DC(+57.02)DRTAAIC(+57.02)F.A | N | 44.79 | 1633.6687 | 13 | 0.9 | 817.8423 | 2 | 47.45 | 1 | 29276 | 01122020\_RID\_2209\_BuCaKA02.raw | 0 | 0 | 0 | 87 | 99 | Carbamidomethylation | C3:Carbamidomethylation:1000.00;C5:Carbamidomethylation:1000.00;C12:Carbamidomethylation:1000.00 | PEAKS DB |
| D.C(+57.02)DRTAAIC(+57.02)FAK.A | N | 39.74 | 1311.6063 | 11 | 1.6 | 656.8115 | 2 | 12.51 | 1 | 2398 | 01122020\_RID\_2209\_BuCaKA02.raw | 5.3941E5 | 1 | 1 | 91 | 101 | Carbamidomethylation | C1:Carbamidomethylation:1000.00;C8:Carbamidomethylation:1000.00 | PEAKS DB |
| total 9 peptides |  |  |  |  |  |  |  |  |  |  |  |  |  |  |  |  |  |  |  |
| --- | --- | --- | --- | --- | --- | --- | --- | --- | --- | --- | --- | --- | --- | --- | --- | --- | --- | --- | --- |

AAS20530.1

back to list

  

| Protein Coverage
| Supporting Peptides
|

Protein Coverage:

Supporting Peptides:

| Peptide | Uniq | -10lgP | Mass | Length | ppm | m/z | z | RT | Fraction | Scan | Source File | Area BRom | #Feature | #Feature BRom | Start | End | PTM | AScore | Found By |
| --- | --- | --- | --- | --- | --- | --- | --- | --- | --- | --- | --- | --- | --- | --- | --- | --- | --- | --- | --- |
| R.FVC(+57.02)DC(+57.02)DRTAAIC(+57.02)FAK.A | Y | 90.11 | 1832.8008 | 15 | 0.5 | 917.4081 | 2 | 29.11 | 1 | 14827 | 01122020\_RID\_2209\_BuCaKA02.raw | 3.5728E10 | 21 | 21 | 106 | 120 | Carbamidomethylation | C3:Carbamidomethylation:1000.00;C5:Carbamidomethylation:1000.00;C12:Carbamidomethylation:1000.00 | PEAKS DB |
| K.C(+57.02)ARFVC(+57.02)DC(+57.02)DRTAAIC(+57.02)FAK.A | Y | 73.89 | 2219.9697 | 18 | 0.9 | 740.9979 | 3 | 24.07 | 1 | 11297 | 01122020\_RID\_2209\_BuCaKA02.raw | 4.6498E7 | 2 | 2 | 103 | 120 | Carbamidomethylation | C1:Carbamidomethylation:1000.00;C6:Carbamidomethylation:1000.00;C8:Carbamidomethylation:1000.00;C15:Carbamidomethylation:1000.00 | PEAKS DB |
| F.VC(+57.02)DC(+57.02)DRTAAIC(+57.02)FAK.A | Y | 62.00 | 1685.7324 | 14 | 0.3 | 843.8738 | 2 | 19.27 | 1 | 7662 | 01122020\_RID\_2209\_BuCaKA02.raw | 9.2148E6 | 1 | 1 | 107 | 120 | Carbamidomethylation | C2:Carbamidomethylation:1000.00;C4:Carbamidomethylation:1000.00;C11:Carbamidomethylation:1000.00 | PEAKS DB |
| R.FVC(+57.02)DC(+57.02)DRTAAIC(+57.02)FAKAPYN.T | Y | 57.69 | 2277.9968 | 19 | 1.0 | 760.3403 | 3 | 39.34 | 1 | 23331 | 01122020\_RID\_2209\_BuCaKA02.raw | 1.219E8 | 1 | 1 | 106 | 124 | Carbamidomethylation | C3:Carbamidomethylation:1000.00;C5:Carbamidomethylation:1000.00;C12:Carbamidomethylation:1000.00 | PEAKS DB |
| A.RFVC(+57.02)DC(+57.02)DRTAAIC(+57.02)FAK.A | Y | 57.32 | 1988.9019 | 16 | 1.4 | 663.9755 | 3 | 21.50 | 1 | 9222 | 01122020\_RID\_2209\_BuCaKA02.raw | 1.2424E7 | 1 | 1 | 105 | 120 | Carbamidomethylation | C4:Carbamidomethylation:1000.00;C6:Carbamidomethylation:1000.00;C13:Carbamidomethylation:1000.00 | PEAKS DB |
| C.DRTAAIC(+57.02)FAK.A | N | 55.24 | 1151.5757 | 10 | 2.7 | 576.7967 | 2 | 15.19 | 1 | 4454 | 01122020\_RID\_2209\_BuCaKA02.raw | 3.7655E5 | 1 | 1 | 111 | 120 | Carbamidomethylation | C7:Carbamidomethylation:1000.00 | PEAKS DB |
| C.DC(+57.02)DRTAAIC(+57.02)FAK.A | N | 47.16 | 1426.6333 | 12 | -2.2 | 714.3224 | 2 | 20.22 | 1 | 8374 | 01122020\_RID\_2209\_BuCaKA02.raw | 1.7388E6 | 1 | 1 | 109 | 120 | Carbamidomethylation | C2:Carbamidomethylation:1000.00;C9:Carbamidomethylation:1000.00 | PEAKS DB |
| R.FVC(+57.02)DC(+57.02)DRTAAIC(+57.02)F.A | N | 44.79 | 1633.6687 | 13 | 0.9 | 817.8423 | 2 | 47.45 | 1 | 29276 | 01122020\_RID\_2209\_BuCaKA02.raw | 0 | 0 | 0 | 106 | 118 | Carbamidomethylation | C3:Carbamidomethylation:1000.00;C5:Carbamidomethylation:1000.00;C12:Carbamidomethylation:1000.00 | PEAKS DB |
| D.C(+57.02)DRTAAIC(+57.02)FAK.A | N | 39.74 | 1311.6063 | 11 | 1.6 | 656.8115 | 2 | 12.51 | 1 | 2398 | 01122020\_RID\_2209\_BuCaKA02.raw | 5.3941E5 | 1 | 1 | 110 | 120 | Carbamidomethylation | C1:Carbamidomethylation:1000.00;C8:Carbamidomethylation:1000.00 | PEAKS DB |
| total 9 peptides |  |  |  |  |  |  |  |  |  |  |  |  |  |  |  |  |  |  |  |
| --- | --- | --- | --- | --- | --- | --- | --- | --- | --- | --- | --- | --- | --- | --- | --- | --- | --- | --- | --- |

Q6SLM2.1

back to list

  

| Protein Coverage
| Supporting Peptides
|

Protein Coverage:

Supporting Peptides:

| Peptide | Uniq | -10lgP | Mass | Length | ppm | m/z | z | RT | Fraction | Scan | Source File | Area BRom | #Feature | #Feature BRom | Start | End | PTM | AScore | Found By |
| --- | --- | --- | --- | --- | --- | --- | --- | --- | --- | --- | --- | --- | --- | --- | --- | --- | --- | --- | --- |
| R.FVC(+57.02)DC(+57.02)DRTAAIC(+57.02)FAK.A | Y | 90.11 | 1832.8008 | 15 | 0.5 | 917.4081 | 2 | 29.11 | 1 | 14827 | 01122020\_RID\_2209\_BuCaKA02.raw | 3.5728E10 | 21 | 21 | 106 | 120 | Carbamidomethylation | C3:Carbamidomethylation:1000.00;C5:Carbamidomethylation:1000.00;C12:Carbamidomethylation:1000.00 | PEAKS DB |
| K.C(+57.02)ARFVC(+57.02)DC(+57.02)DRTAAIC(+57.02)FAK.A | Y | 73.89 | 2219.9697 | 18 | 0.9 | 740.9979 | 3 | 24.07 | 1 | 11297 | 01122020\_RID\_2209\_BuCaKA02.raw | 4.6498E7 | 2 | 2 | 103 | 120 | Carbamidomethylation | C1:Carbamidomethylation:1000.00;C6:Carbamidomethylation:1000.00;C8:Carbamidomethylation:1000.00;C15:Carbamidomethylation:1000.00 | PEAKS DB |
| F.VC(+57.02)DC(+57.02)DRTAAIC(+57.02)FAK.A | Y | 62.00 | 1685.7324 | 14 | 0.3 | 843.8738 | 2 | 19.27 | 1 | 7662 | 01122020\_RID\_2209\_BuCaKA02.raw | 9.2148E6 | 1 | 1 | 107 | 120 | Carbamidomethylation | C2:Carbamidomethylation:1000.00;C4:Carbamidomethylation:1000.00;C11:Carbamidomethylation:1000.00 | PEAKS DB |
| R.FVC(+57.02)DC(+57.02)DRTAAIC(+57.02)FAKAPYN.T | Y | 57.69 | 2277.9968 | 19 | 1.0 | 760.3403 | 3 | 39.34 | 1 | 23331 | 01122020\_RID\_2209\_BuCaKA02.raw | 1.219E8 | 1 | 1 | 106 | 124 | Carbamidomethylation | C3:Carbamidomethylation:1000.00;C5:Carbamidomethylation:1000.00;C12:Carbamidomethylation:1000.00 | PEAKS DB |
| A.RFVC(+57.02)DC(+57.02)DRTAAIC(+57.02)FAK.A | Y | 57.32 | 1988.9019 | 16 | 1.4 | 663.9755 | 3 | 21.50 | 1 | 9222 | 01122020\_RID\_2209\_BuCaKA02.raw | 1.2424E7 | 1 | 1 | 105 | 120 | Carbamidomethylation | C4:Carbamidomethylation:1000.00;C6:Carbamidomethylation:1000.00;C13:Carbamidomethylation:1000.00 | PEAKS DB |
| C.DRTAAIC(+57.02)FAK.A | N | 55.24 | 1151.5757 | 10 | 2.7 | 576.7967 | 2 | 15.19 | 1 | 4454 | 01122020\_RID\_2209\_BuCaKA02.raw | 3.7655E5 | 1 | 1 | 111 | 120 | Carbamidomethylation | C7:Carbamidomethylation:1000.00 | PEAKS DB |
| C.DC(+57.02)DRTAAIC(+57.02)FAK.A | N | 47.16 | 1426.6333 | 12 | -2.2 | 714.3224 | 2 | 20.22 | 1 | 8374 | 01122020\_RID\_2209\_BuCaKA02.raw | 1.7388E6 | 1 | 1 | 109 | 120 | Carbamidomethylation | C2:Carbamidomethylation:1000.00;C9:Carbamidomethylation:1000.00 | PEAKS DB |
| R.FVC(+57.02)DC(+57.02)DRTAAIC(+57.02)F.A | N | 44.79 | 1633.6687 | 13 | 0.9 | 817.8423 | 2 | 47.45 | 1 | 29276 | 01122020\_RID\_2209\_BuCaKA02.raw | 0 | 0 | 0 | 106 | 118 | Carbamidomethylation | C3:Carbamidomethylation:1000.00;C5:Carbamidomethylation:1000.00;C12:Carbamidomethylation:1000.00 | PEAKS DB |
| D.C(+57.02)DRTAAIC(+57.02)FAK.A | N | 39.74 | 1311.6063 | 11 | 1.6 | 656.8115 | 2 | 12.51 | 1 | 2398 | 01122020\_RID\_2209\_BuCaKA02.raw | 5.3941E5 | 1 | 1 | 110 | 120 | Carbamidomethylation | C1:Carbamidomethylation:1000.00;C8:Carbamidomethylation:1000.00 | PEAKS DB |
| total 9 peptides |  |  |  |  |  |  |  |  |  |  |  |  |  |  |  |  |  |  |  |
| --- | --- | --- | --- | --- | --- | --- | --- | --- | --- | --- | --- | --- | --- | --- | --- | --- | --- | --- | --- |

XP\_026555558.1

back to list

  

| Protein Coverage
| Supporting Peptides
|

Protein Coverage:

Supporting Peptides:

| Peptide | Uniq | -10lgP | Mass | Length | ppm | m/z | z | RT | Fraction | Scan | Source File | Area BRom | #Feature | #Feature BRom | Start | End | PTM | AScore | Found By |
| --- | --- | --- | --- | --- | --- | --- | --- | --- | --- | --- | --- | --- | --- | --- | --- | --- | --- | --- | --- |
| I.VGGSSALPGSHPWLASINVGRNFC(+57.02)AGSLIR.S | Y | 70.14 | 3079.5774 | 30 | 1.8 | 770.9030 | 4 | 58.82 | 1 | 38294 | 01122020\_RID\_2209\_BuCaKA02.raw | 1.5721E8 | 2 | 2 | 305 | 334 | Carbamidomethylation | C24:Carbamidomethylation:1000.00 | PEAKS DB |
| R.YSNVVQEALIPIIPDYK.C | Y | 69.71 | 1961.0509 | 17 | 2.3 | 654.6924 | 3 | 78.09 | 1 | 54253 | 01122020\_RID\_2209\_BuCaKA02.raw | 3.9803E6 | 1 | 1 | 446 | 462 |  |  | PEAKS DB |
| I.VGGSSALPGSHPWLASINVGR.N | Y | 69.67 | 2061.0754 | 21 | 0.1 | 688.0325 | 3 | 46.61 | 1 | 28663 | 01122020\_RID\_2209\_BuCaKA02.raw | 2.7368E7 | 2 | 2 | 305 | 325 |  |  | PEAKS DB |
| V.GGSSALPGSHPWLASINVGR.N | Y | 69.36 | 1962.0071 | 20 | 1.5 | 982.0123 | 2 | 44.48 | 1 | 26921 | 01122020\_RID\_2209\_BuCaKA02.raw | 1.8764E8 | 2 | 2 | 306 | 325 |  |  | PEAKS DB |
| R.YSNVVQEALIPIIPDYKC(+57.02).Q | Y | 56.27 | 2121.0815 | 18 | 0.6 | 1061.5487 | 2 | 80.57 | 1 | 56341 | 01122020\_RID\_2209\_BuCaKA02.raw | 9.3841E6 | 1 | 1 | 446 | 463 | Carbamidomethylation | C18:Carbamidomethylation:1000.00 | PEAKS DB |
| Y.SNVVQEALIPIIPDYK.C | Y | 51.03 | 1797.9875 | 16 | 1.5 | 900.0024 | 2 | 74.60 | 1 | 51396 | 01122020\_RID\_2209\_BuCaKA02.raw | 1.0953E7 | 1 | 1 | 447 | 462 |  |  | PEAKS DB |
| V.GGSSALPGSHPWLASINVGRNFC(+57.02)AGSLIR.S | Y | 49.29 | 2980.5090 | 29 | 0.5 | 994.5107 | 3 | 57.92 | 1 | 37598 | 01122020\_RID\_2209\_BuCaKA02.raw | 3.5364E8 | 2 | 2 | 306 | 334 | Carbamidomethylation | C23:Carbamidomethylation:1000.00 | PEAKS DB |
| G.GSSALPGSHPWLASINVGRNFC(+57.02)AGSLIR.S | Y | 47.12 | 2923.4875 | 28 | 0.7 | 731.8796 | 4 | 57.89 | 1 | 37725 | 01122020\_RID\_2209\_BuCaKA02.raw | 1.8196E6 | 1 | 1 | 307 | 334 | Carbamidomethylation | C22:Carbamidomethylation:1000.00 | PEAKS DB |
| D.EQPWC(+57.02)YFIK.D | Y | 46.80 | 1269.5852 | 9 | 1.3 | 635.8007 | 2 | 63.01 | 1 | 41765 | 01122020\_RID\_2209\_BuCaKA02.raw | 5.3369E6 | 1 | 1 | 240 | 248 | Carbamidomethylation | C5:Carbamidomethylation:1000.00 | PEAKS DB |
| total 9 peptides |  |  |  |  |  |  |  |  |  |  |  |  |  |  |  |  |  |  |  |
| --- | --- | --- | --- | --- | --- | --- | --- | --- | --- | --- | --- | --- | --- | --- | --- | --- | --- | --- | --- |

T\_15

back to list

  

| Protein Coverage
| Supporting Peptides
|

Protein Coverage:

Supporting Peptides:

| Peptide | Uniq | -10lgP | Mass | Length | ppm | m/z | z | RT | Fraction | Scan | Source File | Area BRom | #Feature | #Feature BRom | Start | End | PTM | AScore | Found By |
| --- | --- | --- | --- | --- | --- | --- | --- | --- | --- | --- | --- | --- | --- | --- | --- | --- | --- | --- | --- |
| R.TWIAYVNYGC(+57.02)YC(+57.02)GAGGSGTPVDELDR.C | Y | 80.17 | 2880.2483 | 26 | 1.2 | 1441.1332 | 2 | 81.40 | 1 | 57598 | 01122020\_RID\_2209\_BuCaKA02.raw | 3.0391E10 | 84 | 84 | 44 | 69 | Carbamidomethylation | C10:Carbamidomethylation:1000.00;C12:Carbamidomethylation:1000.00 | PEAKS DB |
| A.YVNYGC(+57.02)YC(+57.02)GAGGSGTPVDELDR.C | Y | 78.56 | 2409.0000 | 22 | 0.9 | 1205.5083 | 2 | 44.29 | 1 | 26760 | 01122020\_RID\_2209\_BuCaKA02.raw | 1.5762E7 | 1 | 1 | 48 | 69 | Carbamidomethylation | C6:Carbamidomethylation:1000.00;C8:Carbamidomethylation:1000.00 | PEAKS DB |
| N.YGC(+57.02)YC(+57.02)GAGGSGTPVDELDR.C | N | 72.32 | 2032.8254 | 19 | 1.0 | 1017.4210 | 2 | 34.88 | 1 | 19734 | 01122020\_RID\_2209\_BuCaKA02.raw | 4.869E7 | 1 | 1 | 51 | 69 | Carbamidomethylation | C3:Carbamidomethylation:1000.00;C5:Carbamidomethylation:1000.00 | PEAKS DB |
| W.IAYVNYGC(+57.02)YC(+57.02)GAGGSGTPVDELDR.C | Y | 64.10 | 2593.1213 | 24 | 1.3 | 1297.5696 | 2 | 53.06 | 1 | 33535 | 01122020\_RID\_2209\_BuCaKA02.raw | 3.38E7 | 1 | 1 | 46 | 69 | Carbamidomethylation | C8:Carbamidomethylation:1000.00;C10:Carbamidomethylation:1000.00 | PEAKS DB |
| K.NMIQC(+57.02)AGTRTWIAYVNYGC(+57.02)YC(+57.02)GAGGSGTPVDELDR.C | Y | 62.46 | 3911.7124 | 35 | 1.3 | 1304.9131 | 3 | 66.88 | 1 | 45267 | 01122020\_RID\_2209\_BuCaKA02.raw | 3.3126E8 | 1 | 1 | 35 | 69 | Carbamidomethylation | C5:Carbamidomethylation:1000.00;C19:Carbamidomethylation:1000.00;C21:Carbamidomethylation:1000.00 | PEAKS DB |
| K.NMIQC(+57.02)AGTRTWIAYVN.Y | Y | 56.33 | 1896.8975 | 16 | 0.3 | 949.4563 | 2 | 55.71 | 1 | 35868 | 01122020\_RID\_2209\_BuCaKA02.raw | 1.9157E8 | 1 | 1 | 35 | 50 | Carbamidomethylation | C5:Carbamidomethylation:1000.00 | PEAKS DB |
| K.NMIQC(+57.02)AGTRTWIAY.V | Y | 46.69 | 1683.7861 | 14 | 0.6 | 842.9009 | 2 | 50.60 | 1 | 31529 | 01122020\_RID\_2209\_BuCaKA02.raw | 1.0506E8 | 1 | 1 | 35 | 48 | Carbamidomethylation | C5:Carbamidomethylation:1000.00 | PEAKS DB |
| total 7 peptides |  |  |  |  |  |  |  |  |  |  |  |  |  |  |  |  |  |  |  |
| --- | --- | --- | --- | --- | --- | --- | --- | --- | --- | --- | --- | --- | --- | --- | --- | --- | --- | --- | --- |

T\_19

back to list

  

| Protein Coverage
| Supporting Peptides
|

Protein Coverage:

Supporting Peptides:

| Peptide | Uniq | -10lgP | Mass | Length | ppm | m/z | z | RT | Fraction | Scan | Source File | Area BRom | #Feature | #Feature BRom | Start | End | PTM | AScore | Found By |
| --- | --- | --- | --- | --- | --- | --- | --- | --- | --- | --- | --- | --- | --- | --- | --- | --- | --- | --- | --- |
| R.TAALC(+57.02)FAEAPYK.R | Y | 76.83 | 1340.6434 | 12 | 1.3 | 671.3298 | 2 | 38.98 | 1 | 22921 | 01122020\_RID\_2209\_BuCaKA02.raw | 1.849E8 | 1 | 1 | 156 | 167 | Carbamidomethylation | C5:Carbamidomethylation:1000.00 | PEAKS DB |
| K.C(+57.02)SSLLNVPYVK.Q | N | 73.15 | 1278.6642 | 11 | 0.7 | 640.3398 | 2 | 45.36 | 1 | 27666 | 01122020\_RID\_2209\_BuCaKA02.raw | 5.5427E7 | 1 | 1 | 116 | 126 | Carbamidomethylation | C1:Carbamidomethylation:1000.00 | PEAKS DB |
| K.EHDDC(+57.02)YAQIKENPKC(+57.02)SSLLNVPYVK.Q | N | 66.45 | 3006.4214 | 25 | 2.6 | 602.2931 | 5 | 34.70 | 1 | 19488 | 01122020\_RID\_2209\_BuCaKA02.raw | 7.9325E6 | 1 | 1 | 102 | 126 | Carbamidomethylation | C5:Carbamidomethylation:1000.00;C15:Carbamidomethylation:1000.00 | PEAKS DB |
| T.AALC(+57.02)FAEAPYK.R | Y | 61.21 | 1239.5957 | 11 | 1.4 | 620.8060 | 2 | 36.73 | 1 | 21078 | 01122020\_RID\_2209\_BuCaKA02.raw | 1.5003E7 | 1 | 1 | 157 | 167 | Carbamidomethylation | C4:Carbamidomethylation:1000.00 | PEAKS DB |
| K.QYSFTC(+57.02)SEGDLTC(+57.02)SADNDEC(+57.02)GAFIC(+57.02)NC(+57.02)DR.T | N | 51.60 | 3451.2793 | 29 | 0.5 | 1151.4343 | 3 | 56.19 | 1 | 36495 | 01122020\_RID\_2209\_BuCaKA02.raw | 1.8306E8 | 1 | 1 | 127 | 155 | Carbamidomethylation | C6:Carbamidomethylation:1000.00;C13:Carbamidomethylation:1000.00;C20:Carbamidomethylation:1000.00;C25:Carbamidomethylation:1000.00;C27:Carbamidomethylation:1000.00 | PEAKS DB |
| K.C(+57.02)SSLLNVPYVKQYSFTC(+57.02)SEGDLTC(+57.02)SADNDEC(+57.02)GAFIC(+57.02)NC(+57.02)DR.T | N | 51.13 | 4711.9326 | 40 | 0.9 | 1571.6528 | 3 | 68.65 | 1 | 46436 | 01122020\_RID\_2209\_BuCaKA02.raw | 7.4401E8 | 2 | 2 | 116 | 155 | Carbamidomethylation | C1:Carbamidomethylation:1000.00;C17:Carbamidomethylation:1000.00;C24:Carbamidomethylation:1000.00;C31:Carbamidomethylation:1000.00;C36:Carbamidomethylation:1000.00;C38:Carbamidomethylation:1000.00 | PEAKS DB |
| R.NFKIDYNTRC(+57.02)Q | Y | 48.95 | 1457.6721 | 11 | 1.1 | 729.8441 | 2 | 17.18 | 1 | 5959 | 01122020\_RID\_2209\_BuCaKA02.raw | 4.6367E6 | 1 | 1 | 170 | 180 | Carbamidomethylation | C10:Carbamidomethylation:1000.00 | PEAKS DB |
| R.NFKIDYNTR.C | Y | 47.78 | 1169.5829 | 9 | 2.1 | 585.7999 | 2 | 16.80 | 1 | 5611 | 01122020\_RID\_2209\_BuCaKA02.raw | 3.6424E7 | 1 | 1 | 170 | 178 |  |  | PEAKS DB |
| total 8 peptides |  |  |  |  |  |  |  |  |  |  |  |  |  |  |  |  |  |  |  |
| --- | --- | --- | --- | --- | --- | --- | --- | --- | --- | --- | --- | --- | --- | --- | --- | --- | --- | --- | --- |

XP\_034286707.1

back to list

  

| Protein Coverage
| Supporting Peptides
|

Protein Coverage:

Supporting Peptides:

| Peptide | Uniq | -10lgP | Mass | Length | ppm | m/z | z | RT | Fraction | Scan | Source File | Area BRom | #Feature | #Feature BRom | Start | End | PTM | AScore | Found By |
| --- | --- | --- | --- | --- | --- | --- | --- | --- | --- | --- | --- | --- | --- | --- | --- | --- | --- | --- | --- |
| R.AQIC(+57.02)AFWNHFLPK.L | N | 81.68 | 1630.8079 | 13 | 0.4 | 816.4115 | 2 | 60.34 | 1 | 39698 | 01122020\_RID\_2209\_BuCaKA02.raw | 3.6259E8 | 1 | 1 | 549 | 561 | Carbamidomethylation | C4:Carbamidomethylation:1000.00 | PEAKS DB |
| F.LGIPFAEPPVGR.M | N | 65.37 | 1251.6975 | 12 | 1.1 | 626.8567 | 2 | 56.11 | 1 | 36154 | 01122020\_RID\_2209\_BuCaKA02.raw | 4.6223E8 | 1 | 1 | 63 | 74 |  |  | PEAKS DB |
| A.FLGIPFAEPPVGR.M | N | 62.09 | 1398.7659 | 13 | 0.0 | 700.3902 | 2 | 73.18 | 1 | 50153 | 01122020\_RID\_2209\_BuCaKA02.raw | 1.8064E7 | 1 | 1 | 62 | 74 |  |  | PEAKS DB |
| R.AQIC(+57.02)AFWNHFLPKL.L | N | 59.24 | 1743.8918 | 14 | 1.7 | 872.9547 | 2 | 72.95 | 1 | 49964 | 01122020\_RID\_2209\_BuCaKA02.raw | 1.5107E8 | 2 | 2 | 549 | 562 | Carbamidomethylation | C4:Carbamidomethylation:1000.00 | PEAKS DB |
| Q.IC(+57.02)AFWNHFLPK.L | N | 56.19 | 1431.7122 | 11 | 1.5 | 716.8644 | 2 | 55.11 | 1 | 35292 | 01122020\_RID\_2209\_BuCaKA02.raw | 7.8377E6 | 1 | 1 | 551 | 561 | Carbamidomethylation | C2:Carbamidomethylation:1000.00 | PEAKS DB |
| A.QIC(+57.02)AFWNHFLPK.L | N | 49.55 | 1559.7708 | 12 | 1.5 | 780.8938 | 2 | 57.48 | 1 | 37350 | 01122020\_RID\_2209\_BuCaKA02.raw | 2.0913E7 | 1 | 1 | 550 | 561 | Carbamidomethylation | C3:Carbamidomethylation:1000.00 | PEAKS DB |
| R.AQIC(+57.02)AFWNHFLP.K | N | 44.50 | 1502.7129 | 12 | 0.9 | 752.3644 | 2 | 78.17 | 1 | 54427 | 01122020\_RID\_2209\_BuCaKA02.raw | 5.0431E8 | 1 | 1 | 549 | 560 | Carbamidomethylation | C4:Carbamidomethylation:1000.00 | PEAKS DB |
| R.GMRM(+15.99)PVLGGHVSAFLGIPFAEPPVGR.M | Y | 44.49 | 2707.4089 | 26 | 9.6 | 1354.7247 | 2 | 93.89 | 1 | 67344 | 01122020\_RID\_2209\_BuCaKA02.raw | 0 | 0 | 0 | 49 | 74 | Oxidation (M) | M4:Oxidation (M):0.00 | PEAKS DB |
| total 8 peptides |  |  |  |  |  |  |  |  |  |  |  |  |  |  |  |  |  |  |  |
| --- | --- | --- | --- | --- | --- | --- | --- | --- | --- | --- | --- | --- | --- | --- | --- | --- | --- | --- | --- |

XP\_034286706.1

back to list

  

| Protein Coverage
| Supporting Peptides
|

Protein Coverage:

Supporting Peptides:

| Peptide | Uniq | -10lgP | Mass | Length | ppm | m/z | z | RT | Fraction | Scan | Source File | Area BRom | #Feature | #Feature BRom | Start | End | PTM | AScore | Found By |
| --- | --- | --- | --- | --- | --- | --- | --- | --- | --- | --- | --- | --- | --- | --- | --- | --- | --- | --- | --- |
| R.AQIC(+57.02)AFWNHFLPK.L | N | 81.68 | 1630.8079 | 13 | 0.4 | 816.4115 | 2 | 60.34 | 1 | 39698 | 01122020\_RID\_2209\_BuCaKA02.raw | 3.6259E8 | 1 | 1 | 548 | 560 | Carbamidomethylation | C4:Carbamidomethylation:1000.00 | PEAKS DB |
| F.LGIPFAEPPVGR.M | N | 65.37 | 1251.6975 | 12 | 1.1 | 626.8567 | 2 | 56.11 | 1 | 36154 | 01122020\_RID\_2209\_BuCaKA02.raw | 4.6223E8 | 1 | 1 | 62 | 73 |  |  | PEAKS DB |
| A.FLGIPFAEPPVGR.M | N | 62.09 | 1398.7659 | 13 | 0.0 | 700.3902 | 2 | 73.18 | 1 | 50153 | 01122020\_RID\_2209\_BuCaKA02.raw | 1.8064E7 | 1 | 1 | 61 | 73 |  |  | PEAKS DB |
| R.AQIC(+57.02)AFWNHFLPKL.L | N | 59.24 | 1743.8918 | 14 | 1.7 | 872.9547 | 2 | 72.95 | 1 | 49964 | 01122020\_RID\_2209\_BuCaKA02.raw | 1.5107E8 | 2 | 2 | 548 | 561 | Carbamidomethylation | C4:Carbamidomethylation:1000.00 | PEAKS DB |
| Q.IC(+57.02)AFWNHFLPK.L | N | 56.19 | 1431.7122 | 11 | 1.5 | 716.8644 | 2 | 55.11 | 1 | 35292 | 01122020\_RID\_2209\_BuCaKA02.raw | 7.8377E6 | 1 | 1 | 550 | 560 | Carbamidomethylation | C2:Carbamidomethylation:1000.00 | PEAKS DB |
| A.QIC(+57.02)AFWNHFLPK.L | N | 49.55 | 1559.7708 | 12 | 1.5 | 780.8938 | 2 | 57.48 | 1 | 37350 | 01122020\_RID\_2209\_BuCaKA02.raw | 2.0913E7 | 1 | 1 | 549 | 560 | Carbamidomethylation | C3:Carbamidomethylation:1000.00 | PEAKS DB |
| R.AQIC(+57.02)AFWNHFLP.K | N | 44.50 | 1502.7129 | 12 | 0.9 | 752.3644 | 2 | 78.17 | 1 | 54427 | 01122020\_RID\_2209\_BuCaKA02.raw | 5.0431E8 | 1 | 1 | 548 | 559 | Carbamidomethylation | C4:Carbamidomethylation:1000.00 | PEAKS DB |
| R.GMRM(+15.99)PVLGGHVSAFLGIPFAEPPVGR.M | Y | 44.49 | 2707.4089 | 26 | 9.6 | 1354.7247 | 2 | 93.89 | 1 | 67344 | 01122020\_RID\_2209\_BuCaKA02.raw | 0 | 0 | 0 | 48 | 73 | Oxidation (M) | M4:Oxidation (M):0.00 | PEAKS DB |
| total 8 peptides |  |  |  |  |  |  |  |  |  |  |  |  |  |  |  |  |  |  |  |
| --- | --- | --- | --- | --- | --- | --- | --- | --- | --- | --- | --- | --- | --- | --- | --- | --- | --- | --- | --- |

XP\_034286705.1

back to list

  

| Protein Coverage
| Supporting Peptides
|

Protein Coverage:

Supporting Peptides:

| Peptide | Uniq | -10lgP | Mass | Length | ppm | m/z | z | RT | Fraction | Scan | Source File | Area BRom | #Feature | #Feature BRom | Start | End | PTM | AScore | Found By |
| --- | --- | --- | --- | --- | --- | --- | --- | --- | --- | --- | --- | --- | --- | --- | --- | --- | --- | --- | --- |
| R.AQIC(+57.02)AFWNHFLPK.L | N | 81.68 | 1630.8079 | 13 | 0.4 | 816.4115 | 2 | 60.34 | 1 | 39698 | 01122020\_RID\_2209\_BuCaKA02.raw | 3.6259E8 | 1 | 1 | 549 | 561 | Carbamidomethylation | C4:Carbamidomethylation:1000.00 | PEAKS DB |
| F.LGIPFAEPPVGR.M | N | 65.37 | 1251.6975 | 12 | 1.1 | 626.8567 | 2 | 56.11 | 1 | 36154 | 01122020\_RID\_2209\_BuCaKA02.raw | 4.6223E8 | 1 | 1 | 63 | 74 |  |  | PEAKS DB |
| A.FLGIPFAEPPVGR.M | N | 62.09 | 1398.7659 | 13 | 0.0 | 700.3902 | 2 | 73.18 | 1 | 50153 | 01122020\_RID\_2209\_BuCaKA02.raw | 1.8064E7 | 1 | 1 | 62 | 74 |  |  | PEAKS DB |
| R.AQIC(+57.02)AFWNHFLPKL.L | N | 59.24 | 1743.8918 | 14 | 1.7 | 872.9547 | 2 | 72.95 | 1 | 49964 | 01122020\_RID\_2209\_BuCaKA02.raw | 1.5107E8 | 2 | 2 | 549 | 562 | Carbamidomethylation | C4:Carbamidomethylation:1000.00 | PEAKS DB |
| Q.IC(+57.02)AFWNHFLPK.L | N | 56.19 | 1431.7122 | 11 | 1.5 | 716.8644 | 2 | 55.11 | 1 | 35292 | 01122020\_RID\_2209\_BuCaKA02.raw | 7.8377E6 | 1 | 1 | 551 | 561 | Carbamidomethylation | C2:Carbamidomethylation:1000.00 | PEAKS DB |
| A.QIC(+57.02)AFWNHFLPK.L | N | 49.55 | 1559.7708 | 12 | 1.5 | 780.8938 | 2 | 57.48 | 1 | 37350 | 01122020\_RID\_2209\_BuCaKA02.raw | 2.0913E7 | 1 | 1 | 550 | 561 | Carbamidomethylation | C3:Carbamidomethylation:1000.00 | PEAKS DB |
| R.AQIC(+57.02)AFWNHFLP.K | N | 44.50 | 1502.7129 | 12 | 0.9 | 752.3644 | 2 | 78.17 | 1 | 54427 | 01122020\_RID\_2209\_BuCaKA02.raw | 5.0431E8 | 1 | 1 | 549 | 560 | Carbamidomethylation | C4:Carbamidomethylation:1000.00 | PEAKS DB |
| R.GMRM(+15.99)PVLGGHVSAFLGIPFAEPPVGR.M | Y | 44.49 | 2707.4089 | 26 | 9.6 | 1354.7247 | 2 | 93.89 | 1 | 67344 | 01122020\_RID\_2209\_BuCaKA02.raw | 0 | 0 | 0 | 49 | 74 | Oxidation (M) | M4:Oxidation (M):0.00 | PEAKS DB |
| total 8 peptides |  |  |  |  |  |  |  |  |  |  |  |  |  |  |  |  |  |  |  |
| --- | --- | --- | --- | --- | --- | --- | --- | --- | --- | --- | --- | --- | --- | --- | --- | --- | --- | --- | --- |

T\_2

back to list

  

| Protein Coverage
| Supporting Peptides
|

Protein Coverage:

Supporting Peptides:

| Peptide | Uniq | -10lgP | Mass | Length | ppm | m/z | z | RT | Fraction | Scan | Source File | Area BRom | #Feature | #Feature BRom | Start | End | PTM | AScore | Found By |
| --- | --- | --- | --- | --- | --- | --- | --- | --- | --- | --- | --- | --- | --- | --- | --- | --- | --- | --- | --- |
| L.DLGYTLTC(+57.02)LIC(+57.02)PEKYC(+57.02)QKVHTC(+57.02)R.N | Y | 81.82 | 2914.3599 | 23 | 2.1 | 583.8805 | 5 | 50.92 | 1 | 31813 | 01122020\_RID\_2209\_BuCaKA02.raw | 3.3951E8 | 2 | 2 | 17 | 39 | Carbamidomethylation | C8:Carbamidomethylation:1000.00;C11:Carbamidomethylation:1000.00;C16:Carbamidomethylation:1000.00;C22:Carbamidomethylation:1000.00 | PEAKS DB |
| L.DLGYTLTC(+57.02)LIC(+57.02)PEKYC(+57.02)QK.V | Y | 78.01 | 2261.0530 | 18 | 1.5 | 754.6927 | 3 | 69.18 | 1 | 46873 | 01122020\_RID\_2209\_BuCaKA02.raw | 5.9501E8 | 2 | 2 | 17 | 34 | Carbamidomethylation | C8:Carbamidomethylation:1000.00;C11:Carbamidomethylation:1000.00;C16:Carbamidomethylation:1000.00 | PEAKS DB |
| L.DLGYTLTC(+57.02)LIC(+57.02)PEK.Y | Y | 71.97 | 1681.8055 | 14 | -0.5 | 841.9097 | 2 | 80.10 | 1 | 56039 | 01122020\_RID\_2209\_BuCaKA02.raw | 3.9167E7 | 4 | 4 | 17 | 30 | Carbamidomethylation | C8:Carbamidomethylation:1000.00;C11:Carbamidomethylation:1000.00 | PEAKS DB |
| L.TC(+57.02)LIC(+57.02)PEKYC(+57.02)QKVHTC(+57.02)R.N | Y | 65.54 | 2252.0322 | 17 | 1.4 | 564.0161 | 4 | 12.15 | 1 | 2111 | 01122020\_RID\_2209\_BuCaKA02.raw | 9.6274E5 | 1 | 1 | 23 | 39 | Carbamidomethylation | C2:Carbamidomethylation:1000.00;C5:Carbamidomethylation:1000.00;C10:Carbamidomethylation:1000.00;C16:Carbamidomethylation:1000.00 | PEAKS DB |
| R.FYDKKLFGWR.A | Y | 60.98 | 1358.7135 | 10 | 2.1 | 680.3655 | 2 | 29.42 | 1 | 15297 | 01122020\_RID\_2209\_BuCaKA02.raw | 1.1379E6 | 1 | 1 | 49 | 58 |  |  | PEAKS DB |
| T.LTC(+57.02)LIC(+57.02)PEKYC(+57.02)QKVHTC(+57.02)R.N | Y | 56.89 | 2365.1162 | 18 | -0.1 | 592.2863 | 4 | 17.42 | 1 | 6172 | 01122020\_RID\_2209\_BuCaKA02.raw | 1.016E6 | 1 | 1 | 22 | 39 | Carbamidomethylation | C3:Carbamidomethylation:1000.00;C6:Carbamidomethylation:1000.00;C11:Carbamidomethylation:1000.00;C17:Carbamidomethylation:1000.00 | PEAKS DB |
| total 6 peptides |  |  |  |  |  |  |  |  |  |  |  |  |  |  |  |  |  |  |  |
| --- | --- | --- | --- | --- | --- | --- | --- | --- | --- | --- | --- | --- | --- | --- | --- | --- | --- | --- | --- |

T\_21

back to list

  

| Protein Coverage
| Supporting Peptides
|

Protein Coverage:

Supporting Peptides:

| Peptide | Uniq | -10lgP | Mass | Length | ppm | m/z | z | RT | Fraction | Scan | Source File | Area BRom | #Feature | #Feature BRom | Start | End | PTM | AScore | Found By |
| --- | --- | --- | --- | --- | --- | --- | --- | --- | --- | --- | --- | --- | --- | --- | --- | --- | --- | --- | --- |
| K.C(+57.02)SSLLNVPYVK.Q | N | 73.15 | 1278.6642 | 11 | 0.7 | 640.3398 | 2 | 45.36 | 1 | 27666 | 01122020\_RID\_2209\_BuCaKA02.raw | 5.5427E7 | 1 | 1 | 116 | 126 | Carbamidomethylation | C1:Carbamidomethylation:1000.00 | PEAKS DB |
| R.TAALC(+57.02)FAKAPYNVENK.E | N | 70.51 | 1795.8927 | 16 | 0.3 | 599.6384 | 3 | 29.16 | 1 | 15058 | 01122020\_RID\_2209\_BuCaKA02.raw | 2.7541E6 | 1 | 1 | 156 | 171 | Carbamidomethylation | C5:Carbamidomethylation:1000.00 | PEAKS DB |
| K.EHDDC(+57.02)YAQIKENPKC(+57.02)SSLLNVPYVK.Q | N | 66.45 | 3006.4214 | 25 | 2.6 | 602.2931 | 5 | 34.70 | 1 | 19488 | 01122020\_RID\_2209\_BuCaKA02.raw | 7.9325E6 | 1 | 1 | 102 | 126 | Carbamidomethylation | C5:Carbamidomethylation:1000.00;C15:Carbamidomethylation:1000.00 | PEAKS DB |
| L.SFYRYTQMIQC(+57.02)TIR.G | Y | 65.79 | 1865.8916 | 14 | 0.9 | 622.9717 | 3 | 43.20 | 1 | 26002 | 01122020\_RID\_2209\_BuCaKA02.raw | 0 | 0 | 0 | 56 | 69 | Carbamidomethylation | C11:Carbamidomethylation:1000.00 | PEAKS DB |
| C.DRTAALC(+57.02)FAK.A | N | 55.24 | 1151.5757 | 10 | 2.7 | 576.7967 | 2 | 15.19 | 1 | 4454 | 01122020\_RID\_2209\_BuCaKA02.raw | 3.7655E5 | 1 | 1 | 154 | 163 | Carbamidomethylation | C7:Carbamidomethylation:1000.00 | PEAKS DB |
| K.QYSFTC(+57.02)SEGDLTC(+57.02)SADNDEC(+57.02)GAFIC(+57.02)NC(+57.02)DR.T | N | 51.60 | 3451.2793 | 29 | 0.5 | 1151.4343 | 3 | 56.19 | 1 | 36495 | 01122020\_RID\_2209\_BuCaKA02.raw | 1.8306E8 | 1 | 1 | 127 | 155 | Carbamidomethylation | C6:Carbamidomethylation:1000.00;C13:Carbamidomethylation:1000.00;C20:Carbamidomethylation:1000.00;C25:Carbamidomethylation:1000.00;C27:Carbamidomethylation:1000.00 | PEAKS DB |
| K.C(+57.02)SSLLNVPYVKQYSFTC(+57.02)SEGDLTC(+57.02)SADNDEC(+57.02)GAFIC(+57.02)NC(+57.02)DR.T | N | 51.13 | 4711.9326 | 40 | 0.9 | 1571.6528 | 3 | 68.65 | 1 | 46436 | 01122020\_RID\_2209\_BuCaKA02.raw | 7.4401E8 | 2 | 2 | 116 | 155 | Carbamidomethylation | C1:Carbamidomethylation:1000.00;C17:Carbamidomethylation:1000.00;C24:Carbamidomethylation:1000.00;C31:Carbamidomethylation:1000.00;C36:Carbamidomethylation:1000.00;C38:Carbamidomethylation:1000.00 | PEAKS DB |
| N.C(+57.02)DRTAALC(+57.02)FAK.A | N | 39.74 | 1311.6063 | 11 | 1.6 | 656.8115 | 2 | 12.51 | 1 | 2398 | 01122020\_RID\_2209\_BuCaKA02.raw | 5.3941E5 | 1 | 1 | 153 | 163 | Carbamidomethylation | C1:Carbamidomethylation:1000.00;C8:Carbamidomethylation:1000.00 | PEAKS DB |
| total 8 peptides |  |  |  |  |  |  |  |  |  |  |  |  |  |  |  |  |  |  |  |
| --- | --- | --- | --- | --- | --- | --- | --- | --- | --- | --- | --- | --- | --- | --- | --- | --- | --- | --- | --- |

LAA65154.1

back to list

  

| Protein Coverage
| Supporting Peptides
|

Protein Coverage:

Supporting Peptides:

| Peptide | Uniq | -10lgP | Mass | Length | ppm | m/z | z | RT | Fraction | Scan | Source File | Area BRom | #Feature | #Feature BRom | Start | End | PTM | AScore | Found By |
| --- | --- | --- | --- | --- | --- | --- | --- | --- | --- | --- | --- | --- | --- | --- | --- | --- | --- | --- | --- |
| K.VFTAFGDAVKNPEAVKDTFAK.L | Y | 83.18 | 2254.1633 | 21 | 1.7 | 564.5491 | 4 | 43.11 | 1 | 25944 | 01122020\_RID\_2209\_BuCaKA02.raw | 0 | 0 | 0 | 36 | 56 |  |  | PEAKS DB |
| K.DTFAKLSELHC(+57.02)DKLHVDPVNFK.L | N | 69.84 | 2612.3057 | 22 | 1.4 | 654.0846 | 4 | 40.63 | 1 | 24218 | 01122020\_RID\_2209\_BuCaKA02.raw | 3.9252E7 | 2 | 2 | 52 | 73 | Carbamidomethylation | C11:Carbamidomethylation:1000.00 | PEAKS DB |
| K.LHVDPVNFKLLGDIL.L | N | 58.44 | 1691.9609 | 15 | 1.1 | 846.9886 | 2 | 78.56 | 1 | 54664 | 01122020\_RID\_2209\_BuCaKA02.raw | 1.1278E7 | 2 | 2 | 65 | 79 |  |  | PEAKS DB |
| K.LSELHC(+57.02)DKLHVDPVNFKLLGDILL.T | N | 55.08 | 2787.4993 | 24 | 1.2 | 697.8829 | 4 | 73.38 | 1 | 50333 | 01122020\_RID\_2209\_BuCaKA02.raw | 6.3167E7 | 1 | 1 | 57 | 80 | Carbamidomethylation | C6:Carbamidomethylation:1000.00 | PEAKS DB |
| K.LSELHC(+57.02)DKLHVDPVNFKLLGDILLTVL.A | N | 53.46 | 3100.6995 | 27 | 0.8 | 776.1828 | 4 | 94.78 | 1 | 68135 | 01122020\_RID\_2209\_BuCaKA02.raw | 1.4117E7 | 1 | 1 | 57 | 83 | Carbamidomethylation | C6:Carbamidomethylation:1000.00 | PEAKS DB |
| K.LLGDILLTVLAAQFGK.E | N | 51.46 | 1670.9971 | 16 | 0.4 | 836.5061 | 2 | 118.03 | 1 | 86948 | 01122020\_RID\_2209\_BuCaKA02.raw | 3.4672E5 | 3 | 3 | 74 | 89 |  |  | PEAKS DB |
| K.LHVDPVNFKLLGDILL.T | N | 48.69 | 1805.0450 | 16 | 3.5 | 602.6910 | 3 | 85.50 | 1 | 60357 | 01122020\_RID\_2209\_BuCaKA02.raw | 3.6866E6 | 2 | 2 | 65 | 80 |  |  | PEAKS DB |
| K.LSELHC(+57.02)DKLHVDPVNFKLLGDIL.L | N | 40.85 | 2674.4153 | 23 | -0.1 | 1338.2148 | 2 | 67.54 | 1 | 45571 | 01122020\_RID\_2209\_BuCaKA02.raw | 3.3985E6 | 1 | 1 | 57 | 79 | Carbamidomethylation | C6:Carbamidomethylation:1000.00 | PEAKS DB |
| total 8 peptides |  |  |  |  |  |  |  |  |  |  |  |  |  |  |  |  |  |  |  |
| --- | --- | --- | --- | --- | --- | --- | --- | --- | --- | --- | --- | --- | --- | --- | --- | --- | --- | --- | --- |

T\_43

back to list

  

| Protein Coverage
| Supporting Peptides
|

Protein Coverage:

Supporting Peptides:

| Peptide | Uniq | -10lgP | Mass | Length | ppm | m/z | z | RT | Fraction | Scan | Source File | Area BRom | #Feature | #Feature BRom | Start | End | PTM | AScore | Found By |
| --- | --- | --- | --- | --- | --- | --- | --- | --- | --- | --- | --- | --- | --- | --- | --- | --- | --- | --- | --- |
| R.AAKDDC(+57.02)DLPEIC(+57.02)TGQSAEC(+57.02)PTDSFQR.N | Y | 78.94 | 2970.2429 | 26 | 1.3 | 1486.1306 | 2 | 37.85 | 1 | 22059 | 01122020\_RID\_2209\_BuCaKA02.raw | 5.3722E8 | 2 | 2 | 197 | 222 | Carbamidomethylation | C6:Carbamidomethylation:1000.00;C12:Carbamidomethylation:1000.00;C19:Carbamidomethylation:1000.00 | PEAKS DB |
| K.ESSYEC(+57.02)FLLNHISQGC(+57.02)GFC(+57.02)R.M | Y | 76.58 | 2463.0405 | 20 | 1.0 | 1232.5288 | 2 | 59.84 | 1 | 39318 | 01122020\_RID\_2209\_BuCaKA02.raw | 2.0922E9 | 2 | 2 | 256 | 275 | Carbamidomethylation | C6:Carbamidomethylation:1000.00;C16:Carbamidomethylation:1000.00;C19:Carbamidomethylation:1000.00 | PEAKS DB |
| E.SSYEC(+57.02)FLLNHISQGC(+57.02)GFC(+57.02)R.M | Y | 67.12 | 2333.9980 | 19 | 0.9 | 779.0073 | 3 | 55.70 | 1 | 35802 | 01122020\_RID\_2209\_BuCaKA02.raw | 8.2656E7 | 2 | 2 | 257 | 275 | Carbamidomethylation | C5:Carbamidomethylation:1000.00;C15:Carbamidomethylation:1000.00;C18:Carbamidomethylation:1000.00 | PEAKS DB |
| K.DDC(+57.02)DLPEIC(+57.02)TGQSAEC(+57.02)PTDSFQR.N | Y | 60.15 | 2700.0737 | 23 | 1.6 | 1351.0463 | 2 | 54.15 | 1 | 34364 | 01122020\_RID\_2209\_BuCaKA02.raw | 7.3423E7 | 2 | 2 | 200 | 222 | Carbamidomethylation | C3:Carbamidomethylation:1000.00;C9:Carbamidomethylation:1000.00;C16:Carbamidomethylation:1000.00 | PEAKS DB |
| K.SVAVVQDYSER.T | N | 52.51 | 1251.6095 | 11 | 1.1 | 626.8127 | 2 | 19.77 | 1 | 7950 | 01122020\_RID\_2209\_BuCaKA02.raw | 1.1175E6 | 1 | 1 | 49 | 59 |  |  | PEAKS DB |
| K.SISDEPHSEFSSC(+57.02)SVQEHQRYLLK.D | N | 51.51 | 2862.3242 | 24 | 0.8 | 716.5889 | 4 | 22.77 | 1 | 10269 | 01122020\_RID\_2209\_BuCaKA02.raw | 4.272E6 | 1 | 1 | 96 | 119 | Carbamidomethylation | C13:Carbamidomethylation:1000.00 | PEAKS DB |
| R.NGLPC(+57.02)QNNQSYC(+57.02)YNGKC(+57.02)PTLTNQC(+57.02)IDHWGPGAK.E | Y | 48.08 | 3851.6660 | 33 | 0.9 | 963.9247 | 4 | 34.93 | 1 | 19835 | 01122020\_RID\_2209\_BuCaKA02.raw | 6.9543E7 | 2 | 2 | 223 | 255 | Carbamidomethylation | C5:Carbamidomethylation:1000.00;C12:Carbamidomethylation:1000.00;C17:Carbamidomethylation:1000.00;C24:Carbamidomethylation:1000.00 | PEAKS DB |
| total 7 peptides |  |  |  |  |  |  |  |  |  |  |  |  |  |  |  |  |  |  |  |
| --- | --- | --- | --- | --- | --- | --- | --- | --- | --- | --- | --- | --- | --- | --- | --- | --- | --- | --- | --- |

T\_142

back to list

  

| Protein Coverage
| Supporting Peptides
|

Protein Coverage:

Supporting Peptides:

| Peptide | Uniq | -10lgP | Mass | Length | ppm | m/z | z | RT | Fraction | Scan | Source File | Area BRom | #Feature | #Feature BRom | Start | End | PTM | AScore | Found By |
| --- | --- | --- | --- | --- | --- | --- | --- | --- | --- | --- | --- | --- | --- | --- | --- | --- | --- | --- | --- |
| K.RDEPLYEFSFC(+57.02)SVQEHRR.Y | Y | 74.77 | 2354.0862 | 18 | 1.4 | 589.5297 | 4 | 31.51 | 1 | 16795 | 01122020\_RID\_2209\_BuCaKA02.raw | 1.3168E8 | 2 | 2 | 32 | 49 | Carbamidomethylation | C11:Carbamidomethylation:1000.00 | PEAKS DB |
| R.DEPLYEFSFC(+57.02)SVQEHRR.Y | Y | 69.34 | 2197.9851 | 17 | 0.8 | 733.6696 | 3 | 44.18 | 1 | 26756 | 01122020\_RID\_2209\_BuCaKA02.raw | 2.3911E8 | 1 | 1 | 33 | 49 | Carbamidomethylation | C10:Carbamidomethylation:1000.00 | PEAKS DB |
| Y.FVEVGEEC(+57.02)DC(+57.02)GSPQDC(+57.02)QSAC(+57.02)C(+57.02)NAATC(+57.02)K.L | Y | 59.03 | 3138.1553 | 27 | 0.3 | 1047.0593 | 3 | 25.62 | 1 | 12335 | 01122020\_RID\_2209\_BuCaKA02.raw | 2.3381E8 | 2 | 2 | 78 | 104 | Carbamidomethylation | C8:Carbamidomethylation:1000.00;C10:Carbamidomethylation:1000.00;C16:Carbamidomethylation:1000.00;C20:Carbamidomethylation:1000.00;C21:Carbamidomethylation:1000.00;C26:Carbamidomethylation:1000.00 | PEAKS DB |
| R.DRPQC(+57.02)ILNKPLSTNIVAPPVC(+57.02)GNYF.V | Y | 46.96 | 2872.4363 | 25 | 0.4 | 958.4865 | 3 | 67.15 | 1 | 45213 | 01122020\_RID\_2209\_BuCaKA02.raw | 1.4843E8 | 1 | 1 | 54 | 78 | Carbamidomethylation | C5:Carbamidomethylation:1000.00;C21:Carbamidomethylation:1000.00 | PEAKS DB |
| Y.FVEVGEEC(+57.02)DC(+57.02)GSPQDC(+57.02)QSAC(+57.02)C(+57.02)NAATC(+57.02)KLK.H | Y | 46.83 | 3379.3342 | 29 | 0.3 | 1127.4524 | 3 | 24.74 | 1 | 11999 | 01122020\_RID\_2209\_BuCaKA02.raw | 3.9844E7 | 1 | 1 | 78 | 106 | Carbamidomethylation | C8:Carbamidomethylation:1000.00;C10:Carbamidomethylation:1000.00;C16:Carbamidomethylation:1000.00;C20:Carbamidomethylation:1000.00;C21:Carbamidomethylation:1000.00;C26:Carbamidomethylation:1000.00 | PEAKS DB |
| R.DEPLYEFSFC(+57.02)SVQEHRRYLLR.D | Y | 46.11 | 2743.3176 | 21 | 0.6 | 686.8371 | 4 | 51.44 | 1 | 32277 | 01122020\_RID\_2209\_BuCaKA02.raw | 0 | 0 | 0 | 33 | 53 | Carbamidomethylation | C10:Carbamidomethylation:1000.00 | PEAKS DB |
| R.DEPLYEFSF.C | Y | 44.00 | 1145.4917 | 9 | 0.1 | 573.7532 | 2 | 96.87 | 1 | 69802 | 01122020\_RID\_2209\_BuCaKA02.raw | 1.4735E7 | 1 | 1 | 33 | 41 |  |  | PEAKS DB |
| R.DRPQC(+57.02)ILNKPLSTNIVAPPVC(+57.02)GNY.F | Y | 40.70 | 2725.3679 | 24 | 2.2 | 682.3508 | 4 | 56.28 | 1 | 36302 | 01122020\_RID\_2209\_BuCaKA02.raw | 5.1758E6 | 1 | 1 | 54 | 77 | Carbamidomethylation | C5:Carbamidomethylation:1000.00;C21:Carbamidomethylation:1000.00 | PEAKS DB |
| total 8 peptides |  |  |  |  |  |  |  |  |  |  |  |  |  |  |  |  |  |  |  |
| --- | --- | --- | --- | --- | --- | --- | --- | --- | --- | --- | --- | --- | --- | --- | --- | --- | --- | --- | --- |

T\_64

back to list

  

| Protein Coverage
| Supporting Peptides
|

Protein Coverage:

Supporting Peptides:

| Peptide | Uniq | -10lgP | Mass | Length | ppm | m/z | z | RT | Fraction | Scan | Source File | Area BRom | #Feature | #Feature BRom | Start | End | PTM | AScore | Found By |
| --- | --- | --- | --- | --- | --- | --- | --- | --- | --- | --- | --- | --- | --- | --- | --- | --- | --- | --- | --- |
| R.SSC(+57.02)RPIETMIDIYQEYPDEVESLFKPSC(+57.02)VLLMK.C | Y | 68.94 | 3976.8970 | 33 | 0.9 | 995.2324 | 4 | 89.72 | 1 | 63987 | 01122020\_RID\_2209\_BuCaKA02.raw | 2.1165E8 | 2 | 2 | 50 | 82 | Carbamidomethylation | C3:Carbamidomethylation:1000.00;C28:Carbamidomethylation:1000.00 | PEAKS DB |
| Q.LDEVIPFMTVFER.S | Y | 66.78 | 1594.8065 | 13 | -0.2 | 798.4104 | 2 | 90.46 | 1 | 64339 | 01122020\_RID\_2209\_BuCaKA02.raw | 6.6157E7 | 2 | 2 | 37 | 49 |  |  | PEAKS DB |
| E.VIPFMTVFER.S | Y | 62.85 | 1237.6528 | 10 | 0.7 | 619.8341 | 2 | 74.75 | 1 | 51579 | 01122020\_RID\_2209\_BuCaKA02.raw | 4.4464E7 | 1 | 1 | 40 | 49 |  |  | PEAKS DB |
| D.EVIPFMTVFER.S | Y | 61.58 | 1366.6954 | 11 | 1.2 | 684.3558 | 2 | 81.04 | 1 | 56653 | 01122020\_RID\_2209\_BuCaKA02.raw | 3.6832E7 | 1 | 1 | 39 | 49 |  |  | PEAKS DB |
| L.DEVIPFMTVFER.S | Y | 60.02 | 1481.7224 | 12 | 0.1 | 741.8686 | 2 | 91.23 | 1 | 65381 | 01122020\_RID\_2209\_BuCaKA02.raw | 6.4416E7 | 2 | 2 | 38 | 49 |  |  | PEAKS DB |
| K.QLDEVIPFMTVFER.S | Y | 59.60 | 1722.8650 | 14 | 2.3 | 862.4418 | 2 | 123.75 | 1 | 91422 | 01122020\_RID\_2209\_BuCaKA02.raw | 2.4991E6 | 2 | 2 | 36 | 49 |  |  | PEAKS DB |
| V.IPFMTVFER.S | Y | 52.77 | 1138.5845 | 9 | 1.3 | 570.3002 | 2 | 64.88 | 1 | 43530 | 01122020\_RID\_2209\_BuCaKA02.raw | 3.5105E7 | 1 | 1 | 41 | 49 |  |  | PEAKS DB |
| E.DGEKQLDEVIPFMTVFER.S | Y | 48.29 | 2152.0510 | 18 | 0.6 | 718.3580 | 3 | 88.46 | 1 | 62759 | 01122020\_RID\_2209\_BuCaKA02.raw | 2.4515E6 | 1 | 1 | 32 | 49 |  |  | PEAKS DB |
| R.SSC(+57.02)RPIETMIDIYQEYPDEVESLFKPSC(+57.02)VLL.M | Y | 41.86 | 3717.7615 | 31 | 2.3 | 1240.2639 | 3 | 98.11 | 1 | 70779 | 01122020\_RID\_2209\_BuCaKA02.raw | 2.0864E7 | 1 | 1 | 50 | 80 | Carbamidomethylation | C3:Carbamidomethylation:1000.00;C28:Carbamidomethylation:1000.00 | PEAKS DB |
| total 9 peptides |  |  |  |  |  |  |  |  |  |  |  |  |  |  |  |  |  |  |  |
| --- | --- | --- | --- | --- | --- | --- | --- | --- | --- | --- | --- | --- | --- | --- | --- | --- | --- | --- | --- |

T\_65

back to list

  

| Protein Coverage
| Supporting Peptides
|

Protein Coverage:

Supporting Peptides:

| Peptide | Uniq | -10lgP | Mass | Length | ppm | m/z | z | RT | Fraction | Scan | Source File | Area BRom | #Feature | #Feature BRom | Start | End | PTM | AScore | Found By |
| --- | --- | --- | --- | --- | --- | --- | --- | --- | --- | --- | --- | --- | --- | --- | --- | --- | --- | --- | --- |
| R.SSC(+57.02)RPIETMIDIYQEYPDEVESLFKPSC(+57.02)VLLMK.C | Y | 68.94 | 3976.8970 | 33 | 0.9 | 995.2324 | 4 | 89.72 | 1 | 63987 | 01122020\_RID\_2209\_BuCaKA02.raw | 2.1165E8 | 2 | 2 | 50 | 82 | Carbamidomethylation | C3:Carbamidomethylation:1000.00;C28:Carbamidomethylation:1000.00 | PEAKS DB |
| Q.LDEVIPFMTVFER.S | Y | 66.78 | 1594.8065 | 13 | -0.2 | 798.4104 | 2 | 90.46 | 1 | 64339 | 01122020\_RID\_2209\_BuCaKA02.raw | 6.6157E7 | 2 | 2 | 37 | 49 |  |  | PEAKS DB |
| E.VIPFMTVFER.S | Y | 62.85 | 1237.6528 | 10 | 0.7 | 619.8341 | 2 | 74.75 | 1 | 51579 | 01122020\_RID\_2209\_BuCaKA02.raw | 4.4464E7 | 1 | 1 | 40 | 49 |  |  | PEAKS DB |
| D.EVIPFMTVFER.S | Y | 61.58 | 1366.6954 | 11 | 1.2 | 684.3558 | 2 | 81.04 | 1 | 56653 | 01122020\_RID\_2209\_BuCaKA02.raw | 3.6832E7 | 1 | 1 | 39 | 49 |  |  | PEAKS DB |
| L.DEVIPFMTVFER.S | Y | 60.02 | 1481.7224 | 12 | 0.1 | 741.8686 | 2 | 91.23 | 1 | 65381 | 01122020\_RID\_2209\_BuCaKA02.raw | 6.4416E7 | 2 | 2 | 38 | 49 |  |  | PEAKS DB |
| K.QLDEVIPFMTVFER.S | Y | 59.60 | 1722.8650 | 14 | 2.3 | 862.4418 | 2 | 123.75 | 1 | 91422 | 01122020\_RID\_2209\_BuCaKA02.raw | 2.4991E6 | 2 | 2 | 36 | 49 |  |  | PEAKS DB |
| V.IPFMTVFER.S | Y | 52.77 | 1138.5845 | 9 | 1.3 | 570.3002 | 2 | 64.88 | 1 | 43530 | 01122020\_RID\_2209\_BuCaKA02.raw | 3.5105E7 | 1 | 1 | 41 | 49 |  |  | PEAKS DB |
| E.DGEKQLDEVIPFMTVFER.S | Y | 48.29 | 2152.0510 | 18 | 0.6 | 718.3580 | 3 | 88.46 | 1 | 62759 | 01122020\_RID\_2209\_BuCaKA02.raw | 2.4515E6 | 1 | 1 | 32 | 49 |  |  | PEAKS DB |
| R.SSC(+57.02)RPIETMIDIYQEYPDEVESLFKPSC(+57.02)VLL.M | Y | 41.86 | 3717.7615 | 31 | 2.3 | 1240.2639 | 3 | 98.11 | 1 | 70779 | 01122020\_RID\_2209\_BuCaKA02.raw | 2.0864E7 | 1 | 1 | 50 | 80 | Carbamidomethylation | C3:Carbamidomethylation:1000.00;C28:Carbamidomethylation:1000.00 | PEAKS DB |
| total 9 peptides |  |  |  |  |  |  |  |  |  |  |  |  |  |  |  |  |  |  |  |
| --- | --- | --- | --- | --- | --- | --- | --- | --- | --- | --- | --- | --- | --- | --- | --- | --- | --- | --- | --- |

T\_68

back to list

  

| Protein Coverage
| Supporting Peptides
|

Protein Coverage:

Supporting Peptides:

| Peptide | Uniq | -10lgP | Mass | Length | ppm | m/z | z | RT | Fraction | Scan | Source File | Area BRom | #Feature | #Feature BRom | Start | End | PTM | AScore | Found By |
| --- | --- | --- | --- | --- | --- | --- | --- | --- | --- | --- | --- | --- | --- | --- | --- | --- | --- | --- | --- |
| R.SSC(+57.02)RPIETMIDIYQEYPDEVESLFKPSC(+57.02)VLLMK.C | Y | 68.94 | 3976.8970 | 33 | 0.9 | 995.2324 | 4 | 89.72 | 1 | 63987 | 01122020\_RID\_2209\_BuCaKA02.raw | 2.1165E8 | 2 | 2 | 50 | 82 | Carbamidomethylation | C3:Carbamidomethylation:1000.00;C28:Carbamidomethylation:1000.00 | PEAKS DB |
| Q.LDEVIPFMTVFER.S | Y | 66.78 | 1594.8065 | 13 | -0.2 | 798.4104 | 2 | 90.46 | 1 | 64339 | 01122020\_RID\_2209\_BuCaKA02.raw | 6.6157E7 | 2 | 2 | 37 | 49 |  |  | PEAKS DB |
| E.VIPFMTVFER.S | Y | 62.85 | 1237.6528 | 10 | 0.7 | 619.8341 | 2 | 74.75 | 1 | 51579 | 01122020\_RID\_2209\_BuCaKA02.raw | 4.4464E7 | 1 | 1 | 40 | 49 |  |  | PEAKS DB |
| D.EVIPFMTVFER.S | Y | 61.58 | 1366.6954 | 11 | 1.2 | 684.3558 | 2 | 81.04 | 1 | 56653 | 01122020\_RID\_2209\_BuCaKA02.raw | 3.6832E7 | 1 | 1 | 39 | 49 |  |  | PEAKS DB |
| L.DEVIPFMTVFER.S | Y | 60.02 | 1481.7224 | 12 | 0.1 | 741.8686 | 2 | 91.23 | 1 | 65381 | 01122020\_RID\_2209\_BuCaKA02.raw | 6.4416E7 | 2 | 2 | 38 | 49 |  |  | PEAKS DB |
| K.QLDEVIPFMTVFER.S | Y | 59.60 | 1722.8650 | 14 | 2.3 | 862.4418 | 2 | 123.75 | 1 | 91422 | 01122020\_RID\_2209\_BuCaKA02.raw | 2.4991E6 | 2 | 2 | 36 | 49 |  |  | PEAKS DB |
| V.IPFMTVFER.S | Y | 52.77 | 1138.5845 | 9 | 1.3 | 570.3002 | 2 | 64.88 | 1 | 43530 | 01122020\_RID\_2209\_BuCaKA02.raw | 3.5105E7 | 1 | 1 | 41 | 49 |  |  | PEAKS DB |
| E.DGEKQLDEVIPFMTVFER.S | Y | 48.29 | 2152.0510 | 18 | 0.6 | 718.3580 | 3 | 88.46 | 1 | 62759 | 01122020\_RID\_2209\_BuCaKA02.raw | 2.4515E6 | 1 | 1 | 32 | 49 |  |  | PEAKS DB |
| R.SSC(+57.02)RPIETMIDIYQEYPDEVESLFKPSC(+57.02)VLL.M | Y | 41.86 | 3717.7615 | 31 | 2.3 | 1240.2639 | 3 | 98.11 | 1 | 70779 | 01122020\_RID\_2209\_BuCaKA02.raw | 2.0864E7 | 1 | 1 | 50 | 80 | Carbamidomethylation | C3:Carbamidomethylation:1000.00;C28:Carbamidomethylation:1000.00 | PEAKS DB |
| total 9 peptides |  |  |  |  |  |  |  |  |  |  |  |  |  |  |  |  |  |  |  |
| --- | --- | --- | --- | --- | --- | --- | --- | --- | --- | --- | --- | --- | --- | --- | --- | --- | --- | --- | --- |

T\_66

back to list

  

| Protein Coverage
| Supporting Peptides
|

Protein Coverage:

Supporting Peptides:

| Peptide | Uniq | -10lgP | Mass | Length | ppm | m/z | z | RT | Fraction | Scan | Source File | Area BRom | #Feature | #Feature BRom | Start | End | PTM | AScore | Found By |
| --- | --- | --- | --- | --- | --- | --- | --- | --- | --- | --- | --- | --- | --- | --- | --- | --- | --- | --- | --- |
| R.SSC(+57.02)RPIETMIDIYQEYPDEVESLFKPSC(+57.02)VLLMK.C | Y | 68.94 | 3976.8970 | 33 | 0.9 | 995.2324 | 4 | 89.72 | 1 | 63987 | 01122020\_RID\_2209\_BuCaKA02.raw | 2.1165E8 | 2 | 2 | 50 | 82 | Carbamidomethylation | C3:Carbamidomethylation:1000.00;C28:Carbamidomethylation:1000.00 | PEAKS DB |
| Q.LDEVIPFMTVFER.S | Y | 66.78 | 1594.8065 | 13 | -0.2 | 798.4104 | 2 | 90.46 | 1 | 64339 | 01122020\_RID\_2209\_BuCaKA02.raw | 6.6157E7 | 2 | 2 | 37 | 49 |  |  | PEAKS DB |
| E.VIPFMTVFER.S | Y | 62.85 | 1237.6528 | 10 | 0.7 | 619.8341 | 2 | 74.75 | 1 | 51579 | 01122020\_RID\_2209\_BuCaKA02.raw | 4.4464E7 | 1 | 1 | 40 | 49 |  |  | PEAKS DB |
| D.EVIPFMTVFER.S | Y | 61.58 | 1366.6954 | 11 | 1.2 | 684.3558 | 2 | 81.04 | 1 | 56653 | 01122020\_RID\_2209\_BuCaKA02.raw | 3.6832E7 | 1 | 1 | 39 | 49 |  |  | PEAKS DB |
| L.DEVIPFMTVFER.S | Y | 60.02 | 1481.7224 | 12 | 0.1 | 741.8686 | 2 | 91.23 | 1 | 65381 | 01122020\_RID\_2209\_BuCaKA02.raw | 6.4416E7 | 2 | 2 | 38 | 49 |  |  | PEAKS DB |
| K.QLDEVIPFMTVFER.S | Y | 59.60 | 1722.8650 | 14 | 2.3 | 862.4418 | 2 | 123.75 | 1 | 91422 | 01122020\_RID\_2209\_BuCaKA02.raw | 2.4991E6 | 2 | 2 | 36 | 49 |  |  | PEAKS DB |
| V.IPFMTVFER.S | Y | 52.77 | 1138.5845 | 9 | 1.3 | 570.3002 | 2 | 64.88 | 1 | 43530 | 01122020\_RID\_2209\_BuCaKA02.raw | 3.5105E7 | 1 | 1 | 41 | 49 |  |  | PEAKS DB |
| E.DGEKQLDEVIPFMTVFER.S | Y | 48.29 | 2152.0510 | 18 | 0.6 | 718.3580 | 3 | 88.46 | 1 | 62759 | 01122020\_RID\_2209\_BuCaKA02.raw | 2.4515E6 | 1 | 1 | 32 | 49 |  |  | PEAKS DB |
| R.SSC(+57.02)RPIETMIDIYQEYPDEVESLFKPSC(+57.02)VLL.M | Y | 41.86 | 3717.7615 | 31 | 2.3 | 1240.2639 | 3 | 98.11 | 1 | 70779 | 01122020\_RID\_2209\_BuCaKA02.raw | 2.0864E7 | 1 | 1 | 50 | 80 | Carbamidomethylation | C3:Carbamidomethylation:1000.00;C28:Carbamidomethylation:1000.00 | PEAKS DB |
| total 9 peptides |  |  |  |  |  |  |  |  |  |  |  |  |  |  |  |  |  |  |  |
| --- | --- | --- | --- | --- | --- | --- | --- | --- | --- | --- | --- | --- | --- | --- | --- | --- | --- | --- | --- |

T\_63

back to list

  

| Protein Coverage
| Supporting Peptides
|

Protein Coverage:

Supporting Peptides:

| Peptide | Uniq | -10lgP | Mass | Length | ppm | m/z | z | RT | Fraction | Scan | Source File | Area BRom | #Feature | #Feature BRom | Start | End | PTM | AScore | Found By |
| --- | --- | --- | --- | --- | --- | --- | --- | --- | --- | --- | --- | --- | --- | --- | --- | --- | --- | --- | --- |
| R.SSC(+57.02)RPIETMIDIYQEYPDEVESLFKPSC(+57.02)VLLMK.C | Y | 68.94 | 3976.8970 | 33 | 0.9 | 995.2324 | 4 | 89.72 | 1 | 63987 | 01122020\_RID\_2209\_BuCaKA02.raw | 2.1165E8 | 2 | 2 | 50 | 82 | Carbamidomethylation | C3:Carbamidomethylation:1000.00;C28:Carbamidomethylation:1000.00 | PEAKS DB |
| Q.LDEVIPFMTVFER.S | Y | 66.78 | 1594.8065 | 13 | -0.2 | 798.4104 | 2 | 90.46 | 1 | 64339 | 01122020\_RID\_2209\_BuCaKA02.raw | 6.6157E7 | 2 | 2 | 37 | 49 |  |  | PEAKS DB |
| E.VIPFMTVFER.S | Y | 62.85 | 1237.6528 | 10 | 0.7 | 619.8341 | 2 | 74.75 | 1 | 51579 | 01122020\_RID\_2209\_BuCaKA02.raw | 4.4464E7 | 1 | 1 | 40 | 49 |  |  | PEAKS DB |
| D.EVIPFMTVFER.S | Y | 61.58 | 1366.6954 | 11 | 1.2 | 684.3558 | 2 | 81.04 | 1 | 56653 | 01122020\_RID\_2209\_BuCaKA02.raw | 3.6832E7 | 1 | 1 | 39 | 49 |  |  | PEAKS DB |
| L.DEVIPFMTVFER.S | Y | 60.02 | 1481.7224 | 12 | 0.1 | 741.8686 | 2 | 91.23 | 1 | 65381 | 01122020\_RID\_2209\_BuCaKA02.raw | 6.4416E7 | 2 | 2 | 38 | 49 |  |  | PEAKS DB |
| K.QLDEVIPFMTVFER.S | Y | 59.60 | 1722.8650 | 14 | 2.3 | 862.4418 | 2 | 123.75 | 1 | 91422 | 01122020\_RID\_2209\_BuCaKA02.raw | 2.4991E6 | 2 | 2 | 36 | 49 |  |  | PEAKS DB |
| V.IPFMTVFER.S | Y | 52.77 | 1138.5845 | 9 | 1.3 | 570.3002 | 2 | 64.88 | 1 | 43530 | 01122020\_RID\_2209\_BuCaKA02.raw | 3.5105E7 | 1 | 1 | 41 | 49 |  |  | PEAKS DB |
| E.DGEKQLDEVIPFMTVFER.S | Y | 48.29 | 2152.0510 | 18 | 0.6 | 718.3580 | 3 | 88.46 | 1 | 62759 | 01122020\_RID\_2209\_BuCaKA02.raw | 2.4515E6 | 1 | 1 | 32 | 49 |  |  | PEAKS DB |
| R.SSC(+57.02)RPIETMIDIYQEYPDEVESLFKPSC(+57.02)VLL.M | Y | 41.86 | 3717.7615 | 31 | 2.3 | 1240.2639 | 3 | 98.11 | 1 | 70779 | 01122020\_RID\_2209\_BuCaKA02.raw | 2.0864E7 | 1 | 1 | 50 | 80 | Carbamidomethylation | C3:Carbamidomethylation:1000.00;C28:Carbamidomethylation:1000.00 | PEAKS DB |
| total 9 peptides |  |  |  |  |  |  |  |  |  |  |  |  |  |  |  |  |  |  |  |
| --- | --- | --- | --- | --- | --- | --- | --- | --- | --- | --- | --- | --- | --- | --- | --- | --- | --- | --- | --- |

T\_67

back to list

  

| Protein Coverage
| Supporting Peptides
|

Protein Coverage:

Supporting Peptides:

| Peptide | Uniq | -10lgP | Mass | Length | ppm | m/z | z | RT | Fraction | Scan | Source File | Area BRom | #Feature | #Feature BRom | Start | End | PTM | AScore | Found By |
| --- | --- | --- | --- | --- | --- | --- | --- | --- | --- | --- | --- | --- | --- | --- | --- | --- | --- | --- | --- |
| R.SSC(+57.02)RPIETMIDIYQEYPDEVESLFKPSC(+57.02)VLLMK.C | Y | 68.94 | 3976.8970 | 33 | 0.9 | 995.2324 | 4 | 89.72 | 1 | 63987 | 01122020\_RID\_2209\_BuCaKA02.raw | 2.1165E8 | 2 | 2 | 50 | 82 | Carbamidomethylation | C3:Carbamidomethylation:1000.00;C28:Carbamidomethylation:1000.00 | PEAKS DB |
| Q.LDEVIPFMTVFER.S | Y | 66.78 | 1594.8065 | 13 | -0.2 | 798.4104 | 2 | 90.46 | 1 | 64339 | 01122020\_RID\_2209\_BuCaKA02.raw | 6.6157E7 | 2 | 2 | 37 | 49 |  |  | PEAKS DB |
| E.VIPFMTVFER.S | Y | 62.85 | 1237.6528 | 10 | 0.7 | 619.8341 | 2 | 74.75 | 1 | 51579 | 01122020\_RID\_2209\_BuCaKA02.raw | 4.4464E7 | 1 | 1 | 40 | 49 |  |  | PEAKS DB |
| D.EVIPFMTVFER.S | Y | 61.58 | 1366.6954 | 11 | 1.2 | 684.3558 | 2 | 81.04 | 1 | 56653 | 01122020\_RID\_2209\_BuCaKA02.raw | 3.6832E7 | 1 | 1 | 39 | 49 |  |  | PEAKS DB |
| L.DEVIPFMTVFER.S | Y | 60.02 | 1481.7224 | 12 | 0.1 | 741.8686 | 2 | 91.23 | 1 | 65381 | 01122020\_RID\_2209\_BuCaKA02.raw | 6.4416E7 | 2 | 2 | 38 | 49 |  |  | PEAKS DB |
| K.QLDEVIPFMTVFER.S | Y | 59.60 | 1722.8650 | 14 | 2.3 | 862.4418 | 2 | 123.75 | 1 | 91422 | 01122020\_RID\_2209\_BuCaKA02.raw | 2.4991E6 | 2 | 2 | 36 | 49 |  |  | PEAKS DB |
| V.IPFMTVFER.S | Y | 52.77 | 1138.5845 | 9 | 1.3 | 570.3002 | 2 | 64.88 | 1 | 43530 | 01122020\_RID\_2209\_BuCaKA02.raw | 3.5105E7 | 1 | 1 | 41 | 49 |  |  | PEAKS DB |
| E.DGEKQLDEVIPFMTVFER.S | Y | 48.29 | 2152.0510 | 18 | 0.6 | 718.3580 | 3 | 88.46 | 1 | 62759 | 01122020\_RID\_2209\_BuCaKA02.raw | 2.4515E6 | 1 | 1 | 32 | 49 |  |  | PEAKS DB |
| R.SSC(+57.02)RPIETMIDIYQEYPDEVESLFKPSC(+57.02)VLL.M | Y | 41.86 | 3717.7615 | 31 | 2.3 | 1240.2639 | 3 | 98.11 | 1 | 70779 | 01122020\_RID\_2209\_BuCaKA02.raw | 2.0864E7 | 1 | 1 | 50 | 80 | Carbamidomethylation | C3:Carbamidomethylation:1000.00;C28:Carbamidomethylation:1000.00 | PEAKS DB |
| total 9 peptides |  |  |  |  |  |  |  |  |  |  |  |  |  |  |  |  |  |  |  |
| --- | --- | --- | --- | --- | --- | --- | --- | --- | --- | --- | --- | --- | --- | --- | --- | --- | --- | --- | --- |

ETE71045.1

back to list

  

| Protein Coverage
| Supporting Peptides
|

Protein Coverage:

Supporting Peptides:

| Peptide | Uniq | -10lgP | Mass | Length | ppm | m/z | z | RT | Fraction | Scan | Source File | Area BRom | #Feature | #Feature BRom | Start | End | PTM | AScore | Found By |
| --- | --- | --- | --- | --- | --- | --- | --- | --- | --- | --- | --- | --- | --- | --- | --- | --- | --- | --- | --- |
| R.GTLSQLSDLHAYNLRVDPVNFK.A | Y | 74.02 | 2486.2917 | 22 | 2.3 | 622.5817 | 4 | 55.60 | 1 | 35705 | 01122020\_RID\_2209\_BuCaKA02.raw | 2.0098E6 | 1 | 1 | 79 | 100 |  |  | PEAKS DB |
| K.TYFSHFNLSPGSKDIIHQGEK.V | Y | 70.86 | 2404.1812 | 21 | 1.6 | 602.0535 | 4 | 29.87 | 1 | 15610 | 01122020\_RID\_2209\_BuCaKA02.raw | 8.4614E6 | 1 | 1 | 42 | 62 |  |  | PEAKS DB |
| K.HLDDIRGTLSQLSDLHAYNLR.V | Y | 67.40 | 2436.2510 | 21 | 2.1 | 610.0713 | 4 | 55.54 | 1 | 35664 | 01122020\_RID\_2209\_BuCaKA02.raw | 0 | 0 | 0 | 73 | 93 |  |  | PEAKS DB |
| K.LGC(+57.02)RLEDIGADALNRL.L | Y | 66.09 | 1784.9203 | 16 | 1.0 | 595.9813 | 3 | 56.45 | 1 | 36444 | 01122020\_RID\_2209\_BuCaKA02.raw | 1.0986E6 | 1 | 1 | 18 | 33 | Carbamidomethylation | C3:Carbamidomethylation:1000.00 | PEAKS DB |
| R.LEDIGADALNRL.L | Y | 58.49 | 1298.6830 | 12 | 2.0 | 650.3500 | 2 | 54.63 | 1 | 34912 | 01122020\_RID\_2209\_BuCaKA02.raw | 4.9377E6 | 1 | 1 | 22 | 33 |  |  | PEAKS DB |
| K.TYFSHFNLSPGSKDIIHQGEKVGK.A | Y | 47.47 | 2688.3660 | 24 | 1.1 | 673.0995 | 4 | 26.80 | 1 | 13460 | 01122020\_RID\_2209\_BuCaKA02.raw | 4.8782E6 | 1 | 1 | 42 | 65 |  |  | PEAKS DB |
| total 6 peptides |  |  |  |  |  |  |  |  |  |  |  |  |  |  |  |  |  |  |  |
| --- | --- | --- | --- | --- | --- | --- | --- | --- | --- | --- | --- | --- | --- | --- | --- | --- | --- | --- | --- |

XP\_026559591.1

back to list

  

| Protein Coverage
| Supporting Peptides
|

Protein Coverage:

Supporting Peptides:

| Peptide | Uniq | -10lgP | Mass | Length | ppm | m/z | z | RT | Fraction | Scan | Source File | Area BRom | #Feature | #Feature BRom | Start | End | PTM | AScore | Found By |
| --- | --- | --- | --- | --- | --- | --- | --- | --- | --- | --- | --- | --- | --- | --- | --- | --- | --- | --- | --- |
| K.TGSAVHAYPSFDPSADVVALNKAITAK.G | Y | 81.71 | 2729.4023 | 27 | -1.2 | 683.3571 | 4 | 56.43 | 1 | 36441 | 01122020\_RID\_2209\_BuCaKA02.raw | 3.0444E6 | 1 | 1 | 27 | 53 |  |  | PEAKS DB |
| K.GVDEAGIIEILTKR.T | Y | 72.18 | 1512.8511 | 14 | 0.5 | 757.4332 | 2 | 62.94 | 1 | 41628 | 01122020\_RID\_2209\_BuCaKA02.raw | 1.9504E7 | 1 | 1 | 54 | 67 |  |  | PEAKS DB |
| K.GVDEAGIIEILTK.R | Y | 69.25 | 1356.7500 | 13 | 0.7 | 679.3828 | 2 | 74.01 | 1 | 50817 | 01122020\_RID\_2209\_BuCaKA02.raw | 9.3039E6 | 1 | 1 | 54 | 66 |  |  | PEAKS DB |
| K.SNLEDVVVAMLKTPAEFDADELR.Y | Y | 52.56 | 2561.2683 | 23 | 3.1 | 854.7660 | 3 | 95.73 | 1 | 68804 | 01122020\_RID\_2209\_BuCaKA02.raw | 5.652E5 | 1 | 1 | 97 | 119 |  |  | PEAKS DB |
| K.YGVSLC(+57.02)QAILDETKGDYEKIL.V | Y | 46.97 | 2414.2039 | 21 | 3.7 | 805.7449 | 3 | 74.27 | 1 | 51066 | 01122020\_RID\_2209\_BuCaKA02.raw | 1.9423E7 | 1 | 1 | 314 | 334 | Carbamidomethylation | C6:Carbamidomethylation:1000.00 | PEAKS DB |
| total 5 peptides |  |  |  |  |  |  |  |  |  |  |  |  |  |  |  |  |  |  |  |
| --- | --- | --- | --- | --- | --- | --- | --- | --- | --- | --- | --- | --- | --- | --- | --- | --- | --- | --- | --- |

XP\_026559592.1

back to list

  

| Protein Coverage
| Supporting Peptides
|

Protein Coverage:

Supporting Peptides:

| Peptide | Uniq | -10lgP | Mass | Length | ppm | m/z | z | RT | Fraction | Scan | Source File | Area BRom | #Feature | #Feature BRom | Start | End | PTM | AScore | Found By |
| --- | --- | --- | --- | --- | --- | --- | --- | --- | --- | --- | --- | --- | --- | --- | --- | --- | --- | --- | --- |
| K.TGSAVHAYPSFDPSADVVALNKAITAK.G | Y | 81.71 | 2729.4023 | 27 | -1.2 | 683.3571 | 4 | 56.43 | 1 | 36441 | 01122020\_RID\_2209\_BuCaKA02.raw | 3.0444E6 | 1 | 1 | 27 | 53 |  |  | PEAKS DB |
| K.GVDEAGIIEILTKR.T | Y | 72.18 | 1512.8511 | 14 | 0.5 | 757.4332 | 2 | 62.94 | 1 | 41628 | 01122020\_RID\_2209\_BuCaKA02.raw | 1.9504E7 | 1 | 1 | 54 | 67 |  |  | PEAKS DB |
| K.GVDEAGIIEILTK.R | Y | 69.25 | 1356.7500 | 13 | 0.7 | 679.3828 | 2 | 74.01 | 1 | 50817 | 01122020\_RID\_2209\_BuCaKA02.raw | 9.3039E6 | 1 | 1 | 54 | 66 |  |  | PEAKS DB |
| K.SNLEDVVVAMLKTPAEFDADELR.Y | Y | 52.56 | 2561.2683 | 23 | 3.1 | 854.7660 | 3 | 95.73 | 1 | 68804 | 01122020\_RID\_2209\_BuCaKA02.raw | 5.652E5 | 1 | 1 | 97 | 119 |  |  | PEAKS DB |
| K.YGVSLC(+57.02)QAILDETKGDYEKIL.V | Y | 46.97 | 2414.2039 | 21 | 3.7 | 805.7449 | 3 | 74.27 | 1 | 51066 | 01122020\_RID\_2209\_BuCaKA02.raw | 1.9423E7 | 1 | 1 | 314 | 334 | Carbamidomethylation | C6:Carbamidomethylation:1000.00 | PEAKS DB |
| total 5 peptides |  |  |  |  |  |  |  |  |  |  |  |  |  |  |  |  |  |  |  |
| --- | --- | --- | --- | --- | --- | --- | --- | --- | --- | --- | --- | --- | --- | --- | --- | --- | --- | --- | --- |

0412250A

back to list

  

| Protein Coverage
| Supporting Peptides
|

Protein Coverage:

Supporting Peptides:

| Peptide | Uniq | -10lgP | Mass | Length | ppm | m/z | z | RT | Fraction | Scan | Source File | Area BRom | #Feature | #Feature BRom | Start | End | PTM | AScore | Found By |
| --- | --- | --- | --- | --- | --- | --- | --- | --- | --- | --- | --- | --- | --- | --- | --- | --- | --- | --- | --- |
| NLINFMEMIR.Y | N | 70.13 | 1279.6417 | 10 | 0.6 | 640.8285 | 2 | 78.90 | 1 | 54806 | 01122020\_RID\_2209\_BuCaKA02.raw | 2.8606E8 | 1 | 1 | 1 | 10 |  |  | PEAKS DB |
| K.RTIIC(+57.02)YGAAGGTC(+57.02)RIVC(+57.02)DC(+57.02)DR.T | Y | 61.54 | 2473.1084 | 21 | 3.3 | 619.2864 | 4 | 21.16 | 1 | 9009 | 01122020\_RID\_2209\_BuCaKA02.raw | 0 | 0 | 0 | 75 | 95 | Carbamidomethylation | C5:Carbamidomethylation:1000.00;C13:Carbamidomethylation:1000.00;C17:Carbamidomethylation:1000.00;C19:Carbamidomethylation:1000.00 | PEAKS DB |
| R.IVC(+57.02)DC(+57.02)DRTAALC(+57.02)FG.Q | N | 58.90 | 1656.7058 | 14 | 1.9 | 829.3618 | 2 | 40.45 | 1 | 24023 | 01122020\_RID\_2209\_BuCaKA02.raw | 6.5443E7 | 1 | 1 | 89 | 102 | Carbamidomethylation | C3:Carbamidomethylation:1000.00;C5:Carbamidomethylation:1000.00;C12:Carbamidomethylation:1000.00 | PEAKS DB |
| N.LINFMEMIR.Y | N | 57.43 | 1165.5988 | 9 | -0.2 | 583.8065 | 2 | 73.46 | 1 | 50593 | 01122020\_RID\_2209\_BuCaKA02.raw | 5.7582E7 | 1 | 1 | 2 | 10 |  |  | PEAKS DB |
| N.LINFM(+15.99)EMIR.Y | N | 56.16 | 1181.5936 | 9 | 0.5 | 591.8044 | 2 | 56.54 | 1 | 36487 | 01122020\_RID\_2209\_BuCaKA02.raw | 3.0018E6 | 1 | 1 | 2 | 10 | Oxidation (M) | M5:Oxidation (M):30.83 | PEAKS DB |
| NLINFMEM(+15.99)IR.Y | N | 54.09 | 1295.6366 | 10 | 2.9 | 648.8275 | 2 | 69.38 | 1 | 47058 | 01122020\_RID\_2209\_BuCaKA02.raw | 0 | 0 | 0 | 1 | 10 | Oxidation (M) | M8:Oxidation (M):15.73 | PEAKS DB |
| total 6 peptides |  |  |  |  |  |  |  |  |  |  |  |  |  |  |  |  |  |  |  |
| --- | --- | --- | --- | --- | --- | --- | --- | --- | --- | --- | --- | --- | --- | --- | --- | --- | --- | --- | --- |

0402253A

back to list

  

| Protein Coverage
| Supporting Peptides
|

Protein Coverage:

Supporting Peptides:

| Peptide | Uniq | -10lgP | Mass | Length | ppm | m/z | z | RT | Fraction | Scan | Source File | Area BRom | #Feature | #Feature BRom | Start | End | PTM | AScore | Found By |
| --- | --- | --- | --- | --- | --- | --- | --- | --- | --- | --- | --- | --- | --- | --- | --- | --- | --- | --- | --- |
| NLINFMEMIR.Y | N | 70.13 | 1279.6417 | 10 | 0.6 | 640.8285 | 2 | 78.90 | 1 | 54806 | 01122020\_RID\_2209\_BuCaKA02.raw | 2.8606E8 | 1 | 1 | 1 | 10 |  |  | PEAKS DB |
| K.RTIIC(+57.02)YGAAGGTC(+57.02)RIVC(+57.02)DC(+57.02)DR.T | Y | 61.54 | 2473.1084 | 21 | 3.3 | 619.2864 | 4 | 21.16 | 1 | 9009 | 01122020\_RID\_2209\_BuCaKA02.raw | 0 | 0 | 0 | 75 | 95 | Carbamidomethylation | C5:Carbamidomethylation:1000.00;C13:Carbamidomethylation:1000.00;C17:Carbamidomethylation:1000.00;C19:Carbamidomethylation:1000.00 | PEAKS DB |
| R.IVC(+57.02)DC(+57.02)DRTAALC(+57.02)FG.Q | N | 58.90 | 1656.7058 | 14 | 1.9 | 829.3618 | 2 | 40.45 | 1 | 24023 | 01122020\_RID\_2209\_BuCaKA02.raw | 6.5443E7 | 1 | 1 | 89 | 102 | Carbamidomethylation | C3:Carbamidomethylation:1000.00;C5:Carbamidomethylation:1000.00;C12:Carbamidomethylation:1000.00 | PEAKS DB |
| N.LINFMEMIR.Y | N | 57.43 | 1165.5988 | 9 | -0.2 | 583.8065 | 2 | 73.46 | 1 | 50593 | 01122020\_RID\_2209\_BuCaKA02.raw | 5.7582E7 | 1 | 1 | 2 | 10 |  |  | PEAKS DB |
| N.LINFM(+15.99)EMIR.Y | N | 56.16 | 1181.5936 | 9 | 0.5 | 591.8044 | 2 | 56.54 | 1 | 36487 | 01122020\_RID\_2209\_BuCaKA02.raw | 3.0018E6 | 1 | 1 | 2 | 10 | Oxidation (M) | M5:Oxidation (M):30.83 | PEAKS DB |
| NLINFMEM(+15.99)IR.Y | N | 54.09 | 1295.6366 | 10 | 2.9 | 648.8275 | 2 | 69.38 | 1 | 47058 | 01122020\_RID\_2209\_BuCaKA02.raw | 0 | 0 | 0 | 1 | 10 | Oxidation (M) | M8:Oxidation (M):15.73 | PEAKS DB |
| total 6 peptides |  |  |  |  |  |  |  |  |  |  |  |  |  |  |  |  |  |  |  |
| --- | --- | --- | --- | --- | --- | --- | --- | --- | --- | --- | --- | --- | --- | --- | --- | --- | --- | --- | --- |

T\_105

back to list

  

| Protein Coverage
| Supporting Peptides
|

Protein Coverage:

Supporting Peptides:

| Peptide | Uniq | -10lgP | Mass | Length | ppm | m/z | z | RT | Fraction | Scan | Source File | Area BRom | #Feature | #Feature BRom | Start | End | PTM | AScore | Found By |
| --- | --- | --- | --- | --- | --- | --- | --- | --- | --- | --- | --- | --- | --- | --- | --- | --- | --- | --- | --- |
| R.TAALC(+57.02)FAKAPYNVENK | N | 70.51 | 1795.8927 | 16 | 0.3 | 599.6384 | 3 | 29.16 | 1 | 15058 | 01122020\_RID\_2209\_BuCaKA02.raw | 2.7541E6 | 1 | 1 | 87 | 102 | Carbamidomethylation | C5:Carbamidomethylation:1000.00 | PEAKS DB |
| R.GTPVDELDRC(+57.02)C(+57.02)QTHDNC(+57.02)YAEAEEHPK.C | Y | 67.95 | 3130.2815 | 26 | 1.4 | 783.5787 | 4 | 17.15 | 1 | 5960 | 01122020\_RID\_2209\_BuCaKA02.raw | 3.8676E6 | 2 | 2 | 21 | 46 | Carbamidomethylation | C10:Carbamidomethylation:1000.00;C11:Carbamidomethylation:1000.00;C17:Carbamidomethylation:1000.00 | PEAKS DB |
| C.DRTAALC(+57.02)FAK.A | N | 55.24 | 1151.5757 | 10 | 2.7 | 576.7967 | 2 | 15.19 | 1 | 4454 | 01122020\_RID\_2209\_BuCaKA02.raw | 3.7655E5 | 1 | 1 | 85 | 94 | Carbamidomethylation | C7:Carbamidomethylation:1000.00 | PEAKS DB |
| N.C(+57.02)DRTAALC(+57.02)FAK.A | N | 39.74 | 1311.6063 | 11 | 1.6 | 656.8115 | 2 | 12.51 | 1 | 2398 | 01122020\_RID\_2209\_BuCaKA02.raw | 5.3941E5 | 1 | 1 | 84 | 94 | Carbamidomethylation | C1:Carbamidomethylation:1000.00;C8:Carbamidomethylation:1000.00 | PEAKS DB |
| total 4 peptides |  |  |  |  |  |  |  |  |  |  |  |  |  |  |  |  |  |  |  |
| --- | --- | --- | --- | --- | --- | --- | --- | --- | --- | --- | --- | --- | --- | --- | --- | --- | --- | --- | --- |

T\_33

back to list

  

| Protein Coverage
| Supporting Peptides
|

Protein Coverage:

Supporting Peptides:

| Peptide | Uniq | -10lgP | Mass | Length | ppm | m/z | z | RT | Fraction | Scan | Source File | Area BRom | #Feature | #Feature BRom | Start | End | PTM | AScore | Found By |
| --- | --- | --- | --- | --- | --- | --- | --- | --- | --- | --- | --- | --- | --- | --- | --- | --- | --- | --- | --- |
| K.YSQANESSKTC(+57.02)PSGQLLC(+57.02)FK.K | Y | 85.25 | 2304.0515 | 20 | 1.9 | 769.0259 | 3 | 26.68 | 1 | 13350 | 01122020\_RID\_2209\_BuCaKA02.raw | 6.6621E7 | 2 | 2 | 44 | 63 | Carbamidomethylation | C11:Carbamidomethylation:1000.00;C18:Carbamidomethylation:1000.00 | PEAKS DB |
| K.TC(+57.02)PSGQLLC(+57.02)FKKWEIGNPSGK.D | Y | 81.95 | 2406.1824 | 21 | 2.0 | 602.5541 | 4 | 41.30 | 1 | 24642 | 01122020\_RID\_2209\_BuCaKA02.raw | 1.172E8 | 3 | 3 | 53 | 73 | Carbamidomethylation | C2:Carbamidomethylation:1000.00;C9:Carbamidomethylation:1000.00 | PEAKS DB |
| total 2 peptides |  |  |  |  |  |  |  |  |  |  |  |  |  |  |  |  |  |  |  |
| --- | --- | --- | --- | --- | --- | --- | --- | --- | --- | --- | --- | --- | --- | --- | --- | --- | --- | --- | --- |

T\_79

back to list

  

| Protein Coverage
| Supporting Peptides
|

Protein Coverage:

Supporting Peptides:

| Peptide | Uniq | -10lgP | Mass | Length | ppm | m/z | z | RT | Fraction | Scan | Source File | Area BRom | #Feature | #Feature BRom | Start | End | PTM | AScore | Found By |
| --- | --- | --- | --- | --- | --- | --- | --- | --- | --- | --- | --- | --- | --- | --- | --- | --- | --- | --- | --- |
| K.C(+57.02)IC(+57.02)EC(+57.02)VESPNKC(+57.02)FLKGER.F | Y | 63.54 | 2285.0061 | 18 | 0.5 | 572.2591 | 4 | 19.42 | 1 | 7724 | 01122020\_RID\_2209\_BuCaKA02.raw | 0 | 0 | 0 | 228 | 245 | Carbamidomethylation | C1:Carbamidomethylation:1000.00;C3:Carbamidomethylation:1000.00;C5:Carbamidomethylation:1000.00;C12:Carbamidomethylation:1000.00 | PEAKS DB |
| K.NKLLPSSC(+57.02)GFNKEFDEEK.C | Y | 62.69 | 2141.0098 | 18 | 2.0 | 714.6786 | 3 | 23.75 | 1 | 11092 | 01122020\_RID\_2209\_BuCaKA02.raw | 2.2348E6 | 1 | 1 | 191 | 208 | Carbamidomethylation | C8:Carbamidomethylation:1000.00 | PEAKS DB |
| R.EVTVDVGKESGATTNIFFKPPC(+57.02)VSVYR.C | Y | 58.76 | 2999.5061 | 27 | 1.4 | 750.8848 | 4 | 55.13 | 1 | 35441 | 01122020\_RID\_2209\_BuCaKA02.raw | 3.7355E7 | 1 | 1 | 4 | 30 | Carbamidomethylation | C22:Carbamidomethylation:1000.00 | PEAKS DB |
| K.KRC(+57.02)DSGFYYSEEVC(+57.02)R.C | Y | 55.86 | 1954.8301 | 15 | 1.9 | 652.6185 | 3 | 13.67 | 1 | 3155 | 01122020\_RID\_2209\_BuCaKA02.raw | 1.6065E6 | 1 | 1 | 263 | 277 | Carbamidomethylation | C3:Carbamidomethylation:1000.00;C14:Carbamidomethylation:1000.00 | PEAKS DB |
| D.SGFYYSEEVC(+57.02)R.C | Y | 45.73 | 1395.5765 | 11 | 1.3 | 698.7964 | 2 | 29.28 | 1 | 15193 | 01122020\_RID\_2209\_BuCaKA02.raw | 1.1668E7 | 1 | 1 | 267 | 277 | Carbamidomethylation | C10:Carbamidomethylation:1000.00 | PEAKS DB |
| total 5 peptides |  |  |  |  |  |  |  |  |  |  |  |  |  |  |  |  |  |  |  |
| --- | --- | --- | --- | --- | --- | --- | --- | --- | --- | --- | --- | --- | --- | --- | --- | --- | --- | --- | --- |

T\_75

back to list

  

| Protein Coverage
| Supporting Peptides
|

Protein Coverage:

Supporting Peptides:

| Peptide | Uniq | -10lgP | Mass | Length | ppm | m/z | z | RT | Fraction | Scan | Source File | Area BRom | #Feature | #Feature BRom | Start | End | PTM | AScore | Found By |
| --- | --- | --- | --- | --- | --- | --- | --- | --- | --- | --- | --- | --- | --- | --- | --- | --- | --- | --- | --- |
| K.C(+57.02)IC(+57.02)EC(+57.02)VESPNKC(+57.02)FLKGER.F | Y | 63.54 | 2285.0061 | 18 | 0.5 | 572.2591 | 4 | 19.42 | 1 | 7724 | 01122020\_RID\_2209\_BuCaKA02.raw | 0 | 0 | 0 | 360 | 377 | Carbamidomethylation | C1:Carbamidomethylation:1000.00;C3:Carbamidomethylation:1000.00;C5:Carbamidomethylation:1000.00;C12:Carbamidomethylation:1000.00 | PEAKS DB |
| K.NKLLPSSC(+57.02)GFNKEFDEEK.C | Y | 62.69 | 2141.0098 | 18 | 2.0 | 714.6786 | 3 | 23.75 | 1 | 11092 | 01122020\_RID\_2209\_BuCaKA02.raw | 2.2348E6 | 1 | 1 | 323 | 340 | Carbamidomethylation | C8:Carbamidomethylation:1000.00 | PEAKS DB |
| R.EVTVDVGKESGATTNIFFKPPC(+57.02)VSVYR.C | Y | 58.76 | 2999.5061 | 27 | 1.4 | 750.8848 | 4 | 55.13 | 1 | 35441 | 01122020\_RID\_2209\_BuCaKA02.raw | 3.7355E7 | 1 | 1 | 136 | 162 | Carbamidomethylation | C22:Carbamidomethylation:1000.00 | PEAKS DB |
| K.KRC(+57.02)DSGFYYSEEVC(+57.02)R.C | Y | 55.86 | 1954.8301 | 15 | 1.9 | 652.6185 | 3 | 13.67 | 1 | 3155 | 01122020\_RID\_2209\_BuCaKA02.raw | 1.6065E6 | 1 | 1 | 395 | 409 | Carbamidomethylation | C3:Carbamidomethylation:1000.00;C14:Carbamidomethylation:1000.00 | PEAKS DB |
| D.SGFYYSEEVC(+57.02)R.C | Y | 45.73 | 1395.5765 | 11 | 1.3 | 698.7964 | 2 | 29.28 | 1 | 15193 | 01122020\_RID\_2209\_BuCaKA02.raw | 1.1668E7 | 1 | 1 | 399 | 409 | Carbamidomethylation | C10:Carbamidomethylation:1000.00 | PEAKS DB |
| total 5 peptides |  |  |  |  |  |  |  |  |  |  |  |  |  |  |  |  |  |  |  |
| --- | --- | --- | --- | --- | --- | --- | --- | --- | --- | --- | --- | --- | --- | --- | --- | --- | --- | --- | --- |

T\_76

back to list

  

| Protein Coverage
| Supporting Peptides
|

Protein Coverage:

Supporting Peptides:

| Peptide | Uniq | -10lgP | Mass | Length | ppm | m/z | z | RT | Fraction | Scan | Source File | Area BRom | #Feature | #Feature BRom | Start | End | PTM | AScore | Found By |
| --- | --- | --- | --- | --- | --- | --- | --- | --- | --- | --- | --- | --- | --- | --- | --- | --- | --- | --- | --- |
| K.C(+57.02)IC(+57.02)EC(+57.02)VESPNKC(+57.02)FLKGER.F | Y | 63.54 | 2285.0061 | 18 | 0.5 | 572.2591 | 4 | 19.42 | 1 | 7724 | 01122020\_RID\_2209\_BuCaKA02.raw | 0 | 0 | 0 | 360 | 377 | Carbamidomethylation | C1:Carbamidomethylation:1000.00;C3:Carbamidomethylation:1000.00;C5:Carbamidomethylation:1000.00;C12:Carbamidomethylation:1000.00 | PEAKS DB |
| K.NKLLPSSC(+57.02)GFNKEFDEEK.C | Y | 62.69 | 2141.0098 | 18 | 2.0 | 714.6786 | 3 | 23.75 | 1 | 11092 | 01122020\_RID\_2209\_BuCaKA02.raw | 2.2348E6 | 1 | 1 | 323 | 340 | Carbamidomethylation | C8:Carbamidomethylation:1000.00 | PEAKS DB |
| R.EVTVDVGKESGATTNIFFKPPC(+57.02)VSVYR.C | Y | 58.76 | 2999.5061 | 27 | 1.4 | 750.8848 | 4 | 55.13 | 1 | 35441 | 01122020\_RID\_2209\_BuCaKA02.raw | 3.7355E7 | 1 | 1 | 136 | 162 | Carbamidomethylation | C22:Carbamidomethylation:1000.00 | PEAKS DB |
| K.KRC(+57.02)DSGFYYSEEVC(+57.02)R.C | Y | 55.86 | 1954.8301 | 15 | 1.9 | 652.6185 | 3 | 13.67 | 1 | 3155 | 01122020\_RID\_2209\_BuCaKA02.raw | 1.6065E6 | 1 | 1 | 395 | 409 | Carbamidomethylation | C3:Carbamidomethylation:1000.00;C14:Carbamidomethylation:1000.00 | PEAKS DB |
| D.SGFYYSEEVC(+57.02)R.C | Y | 45.73 | 1395.5765 | 11 | 1.3 | 698.7964 | 2 | 29.28 | 1 | 15193 | 01122020\_RID\_2209\_BuCaKA02.raw | 1.1668E7 | 1 | 1 | 399 | 409 | Carbamidomethylation | C10:Carbamidomethylation:1000.00 | PEAKS DB |
| total 5 peptides |  |  |  |  |  |  |  |  |  |  |  |  |  |  |  |  |  |  |  |
| --- | --- | --- | --- | --- | --- | --- | --- | --- | --- | --- | --- | --- | --- | --- | --- | --- | --- | --- | --- |

XP\_026542555.1

back to list

  

| Protein Coverage
| Supporting Peptides
|

Protein Coverage:

Supporting Peptides:

| Peptide | Uniq | -10lgP | Mass | Length | ppm | m/z | z | RT | Fraction | Scan | Source File | Area BRom | #Feature | #Feature BRom | Start | End | PTM | AScore | Found By |
| --- | --- | --- | --- | --- | --- | --- | --- | --- | --- | --- | --- | --- | --- | --- | --- | --- | --- | --- | --- |
| L.SLGPPLSGFPLPPASC(+57.02)TPLQLSAVLR.H | Y | 63.45 | 2674.4517 | 26 | 0.8 | 892.4919 | 3 | 92.10 | 1 | 66045 | 01122020\_RID\_2209\_BuCaKA02.raw | 6.0985E7 | 2 | 2 | 57 | 82 | Carbamidomethylation | C16:Carbamidomethylation:1000.00 | PEAKS DB |
| L.GPPLSGFPLPPASC(+57.02)TPLQLSAVLR.H | Y | 62.47 | 2474.3354 | 24 | 0.3 | 825.7860 | 3 | 86.70 | 1 | 61488 | 01122020\_RID\_2209\_BuCaKA02.raw | 1.7738E7 | 2 | 2 | 59 | 82 | Carbamidomethylation | C14:Carbamidomethylation:1000.00 | PEAKS DB |
| R.DPLSLGPPLSGFPLPPASC(+57.02)TPLQLSAVLR.H | Y | 59.13 | 2999.6152 | 29 | 2.8 | 1000.8818 | 3 | 106.13 | 1 | 77439 | 01122020\_RID\_2209\_BuCaKA02.raw | 4.0835E6 | 1 | 1 | 54 | 82 | Carbamidomethylation | C19:Carbamidomethylation:1000.00 | PEAKS DB |
| S.LGPPLSGFPLPPASC(+57.02)TPLQLSAVLR.H | Y | 56.61 | 2587.4194 | 25 | 0.9 | 863.4812 | 3 | 91.75 | 1 | 65524 | 01122020\_RID\_2209\_BuCaKA02.raw | 2.9947E6 | 1 | 1 | 58 | 82 | Carbamidomethylation | C15:Carbamidomethylation:1000.00 | PEAKS DB |
| total 4 peptides |  |  |  |  |  |  |  |  |  |  |  |  |  |  |  |  |  |  |  |
| --- | --- | --- | --- | --- | --- | --- | --- | --- | --- | --- | --- | --- | --- | --- | --- | --- | --- | --- | --- |

XP\_026542553.1

back to list

  

| Protein Coverage
| Supporting Peptides
|

Protein Coverage:

Supporting Peptides:

| Peptide | Uniq | -10lgP | Mass | Length | ppm | m/z | z | RT | Fraction | Scan | Source File | Area BRom | #Feature | #Feature BRom | Start | End | PTM | AScore | Found By |
| --- | --- | --- | --- | --- | --- | --- | --- | --- | --- | --- | --- | --- | --- | --- | --- | --- | --- | --- | --- |
| L.SLGPPLSGFPLPPASC(+57.02)TPLQLSAVLR.H | Y | 63.45 | 2674.4517 | 26 | 0.8 | 892.4919 | 3 | 92.10 | 1 | 66045 | 01122020\_RID\_2209\_BuCaKA02.raw | 6.0985E7 | 2 | 2 | 57 | 82 | Carbamidomethylation | C16:Carbamidomethylation:1000.00 | PEAKS DB |
| L.GPPLSGFPLPPASC(+57.02)TPLQLSAVLR.H | Y | 62.47 | 2474.3354 | 24 | 0.3 | 825.7860 | 3 | 86.70 | 1 | 61488 | 01122020\_RID\_2209\_BuCaKA02.raw | 1.7738E7 | 2 | 2 | 59 | 82 | Carbamidomethylation | C14:Carbamidomethylation:1000.00 | PEAKS DB |
| R.DPLSLGPPLSGFPLPPASC(+57.02)TPLQLSAVLR.H | Y | 59.13 | 2999.6152 | 29 | 2.8 | 1000.8818 | 3 | 106.13 | 1 | 77439 | 01122020\_RID\_2209\_BuCaKA02.raw | 4.0835E6 | 1 | 1 | 54 | 82 | Carbamidomethylation | C19:Carbamidomethylation:1000.00 | PEAKS DB |
| S.LGPPLSGFPLPPASC(+57.02)TPLQLSAVLR.H | Y | 56.61 | 2587.4194 | 25 | 0.9 | 863.4812 | 3 | 91.75 | 1 | 65524 | 01122020\_RID\_2209\_BuCaKA02.raw | 2.9947E6 | 1 | 1 | 58 | 82 | Carbamidomethylation | C15:Carbamidomethylation:1000.00 | PEAKS DB |
| total 4 peptides |  |  |  |  |  |  |  |  |  |  |  |  |  |  |  |  |  |  |  |
| --- | --- | --- | --- | --- | --- | --- | --- | --- | --- | --- | --- | --- | --- | --- | --- | --- | --- | --- | --- |

ETE60521.1

back to list

  

| Protein Coverage
| Supporting Peptides
|

Protein Coverage:

Supporting Peptides:

| Peptide | Uniq | -10lgP | Mass | Length | ppm | m/z | z | RT | Fraction | Scan | Source File | Area BRom | #Feature | #Feature BRom | Start | End | PTM | AScore | Found By |
| --- | --- | --- | --- | --- | --- | --- | --- | --- | --- | --- | --- | --- | --- | --- | --- | --- | --- | --- | --- |
| R.C(+57.02)IESLLAVFQR.Y | Y | 71.56 | 1334.7017 | 11 | 0.3 | 668.3583 | 2 | 75.75 | 1 | 52262 | 01122020\_RID\_2209\_BuCaKA02.raw | 1.3754E7 | 1 | 1 | 69 | 79 | Carbamidomethylation | C1:Carbamidomethylation:1000.00 | PEAKS DB |
| R.YTVGPTETERC(+57.02)IESLLAVFQR.Y | Y | 69.47 | 2468.2368 | 21 | 1.2 | 823.7538 | 3 | 86.96 | 1 | 61629 | 01122020\_RID\_2209\_BuCaKA02.raw | 3.6849E6 | 1 | 1 | 59 | 79 | Carbamidomethylation | C11:Carbamidomethylation:1000.00 | PEAKS DB |
| C.IESLLAVFQR.Y | Y | 53.59 | 1174.6710 | 10 | 1.2 | 588.3435 | 2 | 68.88 | 1 | 46588 | 01122020\_RID\_2209\_BuCaKA02.raw | 4.3068E6 | 1 | 1 | 70 | 79 |  |  | PEAKS DB |
| R.YTVGPTETERC(+57.02)IESLLAVFQRYAGK.E | Y | 40.61 | 2887.4539 | 25 | 6.3 | 722.8753 | 4 | 87.50 | 1 | 62078 | 01122020\_RID\_2209\_BuCaKA02.raw | 0 | 0 | 0 | 59 | 83 | Carbamidomethylation | C11:Carbamidomethylation:1000.00 | PEAKS DB |
| total 4 peptides |  |  |  |  |  |  |  |  |  |  |  |  |  |  |  |  |  |  |  |
| --- | --- | --- | --- | --- | --- | --- | --- | --- | --- | --- | --- | --- | --- | --- | --- | --- | --- | --- | --- |

XP\_026580631.1

back to list

  

| Protein Coverage
| Supporting Peptides
|

Protein Coverage:

Supporting Peptides:

| Peptide | Uniq | -10lgP | Mass | Length | ppm | m/z | z | RT | Fraction | Scan | Source File | Area BRom | #Feature | #Feature BRom | Start | End | PTM | AScore | Found By |
| --- | --- | --- | --- | --- | --- | --- | --- | --- | --- | --- | --- | --- | --- | --- | --- | --- | --- | --- | --- |
| R.C(+57.02)IESLLAVFQR.Y | Y | 71.56 | 1334.7017 | 11 | 0.3 | 668.3583 | 2 | 75.75 | 1 | 52262 | 01122020\_RID\_2209\_BuCaKA02.raw | 1.3754E7 | 1 | 1 | 15 | 25 | Carbamidomethylation | C1:Carbamidomethylation:1000.00 | PEAKS DB |
| R.YTVGPTETERC(+57.02)IESLLAVFQR.Y | Y | 69.47 | 2468.2368 | 21 | 1.2 | 823.7538 | 3 | 86.96 | 1 | 61629 | 01122020\_RID\_2209\_BuCaKA02.raw | 3.6849E6 | 1 | 1 | 5 | 25 | Carbamidomethylation | C11:Carbamidomethylation:1000.00 | PEAKS DB |
| C.IESLLAVFQR.Y | Y | 53.59 | 1174.6710 | 10 | 1.2 | 588.3435 | 2 | 68.88 | 1 | 46588 | 01122020\_RID\_2209\_BuCaKA02.raw | 4.3068E6 | 1 | 1 | 16 | 25 |  |  | PEAKS DB |
| R.YTVGPTETERC(+57.02)IESLLAVFQRYAGK.E | Y | 40.61 | 2887.4539 | 25 | 6.3 | 722.8753 | 4 | 87.50 | 1 | 62078 | 01122020\_RID\_2209\_BuCaKA02.raw | 0 | 0 | 0 | 5 | 29 | Carbamidomethylation | C11:Carbamidomethylation:1000.00 | PEAKS DB |
| total 4 peptides |  |  |  |  |  |  |  |  |  |  |  |  |  |  |  |  |  |  |  |
| --- | --- | --- | --- | --- | --- | --- | --- | --- | --- | --- | --- | --- | --- | --- | --- | --- | --- | --- | --- |

XP\_026580634.1

back to list

  

| Protein Coverage
| Supporting Peptides
|

Protein Coverage:

Supporting Peptides:

| Peptide | Uniq | -10lgP | Mass | Length | ppm | m/z | z | RT | Fraction | Scan | Source File | Area BRom | #Feature | #Feature BRom | Start | End | PTM | AScore | Found By |
| --- | --- | --- | --- | --- | --- | --- | --- | --- | --- | --- | --- | --- | --- | --- | --- | --- | --- | --- | --- |
| R.C(+57.02)IESLLAVFQR.Y | Y | 71.56 | 1334.7017 | 11 | 0.3 | 668.3583 | 2 | 75.75 | 1 | 52262 | 01122020\_RID\_2209\_BuCaKA02.raw | 1.3754E7 | 1 | 1 | 15 | 25 | Carbamidomethylation | C1:Carbamidomethylation:1000.00 | PEAKS DB |
| R.YTVGPTETERC(+57.02)IESLLAVFQR.Y | Y | 69.47 | 2468.2368 | 21 | 1.2 | 823.7538 | 3 | 86.96 | 1 | 61629 | 01122020\_RID\_2209\_BuCaKA02.raw | 3.6849E6 | 1 | 1 | 5 | 25 | Carbamidomethylation | C11:Carbamidomethylation:1000.00 | PEAKS DB |
| C.IESLLAVFQR.Y | Y | 53.59 | 1174.6710 | 10 | 1.2 | 588.3435 | 2 | 68.88 | 1 | 46588 | 01122020\_RID\_2209\_BuCaKA02.raw | 4.3068E6 | 1 | 1 | 16 | 25 |  |  | PEAKS DB |
| R.YTVGPTETERC(+57.02)IESLLAVFQRYAGK.E | Y | 40.61 | 2887.4539 | 25 | 6.3 | 722.8753 | 4 | 87.50 | 1 | 62078 | 01122020\_RID\_2209\_BuCaKA02.raw | 0 | 0 | 0 | 5 | 29 | Carbamidomethylation | C11:Carbamidomethylation:1000.00 | PEAKS DB |
| total 4 peptides |  |  |  |  |  |  |  |  |  |  |  |  |  |  |  |  |  |  |  |
| --- | --- | --- | --- | --- | --- | --- | --- | --- | --- | --- | --- | --- | --- | --- | --- | --- | --- | --- | --- |

JAB53385.1

back to list

  

| Protein Coverage
| Supporting Peptides
|

Protein Coverage:

Supporting Peptides:

| Peptide | Uniq | -10lgP | Mass | Length | ppm | m/z | z | RT | Fraction | Scan | Source File | Area BRom | #Feature | #Feature BRom | Start | End | PTM | AScore | Found By |
| --- | --- | --- | --- | --- | --- | --- | --- | --- | --- | --- | --- | --- | --- | --- | --- | --- | --- | --- | --- |
| R.C(+57.02)IESLLAVFQR.Y | Y | 71.56 | 1334.7017 | 11 | 0.3 | 668.3583 | 2 | 75.75 | 1 | 52262 | 01122020\_RID\_2209\_BuCaKA02.raw | 1.3754E7 | 1 | 1 | 15 | 25 | Carbamidomethylation | C1:Carbamidomethylation:1000.00 | PEAKS DB |
| R.YTVGPTETERC(+57.02)IESLLAVFQR.Y | Y | 69.47 | 2468.2368 | 21 | 1.2 | 823.7538 | 3 | 86.96 | 1 | 61629 | 01122020\_RID\_2209\_BuCaKA02.raw | 3.6849E6 | 1 | 1 | 5 | 25 | Carbamidomethylation | C11:Carbamidomethylation:1000.00 | PEAKS DB |
| C.IESLLAVFQR.Y | Y | 53.59 | 1174.6710 | 10 | 1.2 | 588.3435 | 2 | 68.88 | 1 | 46588 | 01122020\_RID\_2209\_BuCaKA02.raw | 4.3068E6 | 1 | 1 | 16 | 25 |  |  | PEAKS DB |
| R.YTVGPTETERC(+57.02)IESLLAVFQRYAGK.E | Y | 40.61 | 2887.4539 | 25 | 6.3 | 722.8753 | 4 | 87.50 | 1 | 62078 | 01122020\_RID\_2209\_BuCaKA02.raw | 0 | 0 | 0 | 5 | 29 | Carbamidomethylation | C11:Carbamidomethylation:1000.00 | PEAKS DB |
| total 4 peptides |  |  |  |  |  |  |  |  |  |  |  |  |  |  |  |  |  |  |  |
| --- | --- | --- | --- | --- | --- | --- | --- | --- | --- | --- | --- | --- | --- | --- | --- | --- | --- | --- | --- |

XP\_026580630.1

back to list

  

| Protein Coverage
| Supporting Peptides
|

Protein Coverage:

Supporting Peptides:

| Peptide | Uniq | -10lgP | Mass | Length | ppm | m/z | z | RT | Fraction | Scan | Source File | Area BRom | #Feature | #Feature BRom | Start | End | PTM | AScore | Found By |
| --- | --- | --- | --- | --- | --- | --- | --- | --- | --- | --- | --- | --- | --- | --- | --- | --- | --- | --- | --- |
| R.C(+57.02)IESLLAVFQR.Y | Y | 71.56 | 1334.7017 | 11 | 0.3 | 668.3583 | 2 | 75.75 | 1 | 52262 | 01122020\_RID\_2209\_BuCaKA02.raw | 1.3754E7 | 1 | 1 | 15 | 25 | Carbamidomethylation | C1:Carbamidomethylation:1000.00 | PEAKS DB |
| R.YTVGPTETERC(+57.02)IESLLAVFQR.Y | Y | 69.47 | 2468.2368 | 21 | 1.2 | 823.7538 | 3 | 86.96 | 1 | 61629 | 01122020\_RID\_2209\_BuCaKA02.raw | 3.6849E6 | 1 | 1 | 5 | 25 | Carbamidomethylation | C11:Carbamidomethylation:1000.00 | PEAKS DB |
| C.IESLLAVFQR.Y | Y | 53.59 | 1174.6710 | 10 | 1.2 | 588.3435 | 2 | 68.88 | 1 | 46588 | 01122020\_RID\_2209\_BuCaKA02.raw | 4.3068E6 | 1 | 1 | 16 | 25 |  |  | PEAKS DB |
| R.YTVGPTETERC(+57.02)IESLLAVFQRYAGK.E | Y | 40.61 | 2887.4539 | 25 | 6.3 | 722.8753 | 4 | 87.50 | 1 | 62078 | 01122020\_RID\_2209\_BuCaKA02.raw | 0 | 0 | 0 | 5 | 29 | Carbamidomethylation | C11:Carbamidomethylation:1000.00 | PEAKS DB |
| total 4 peptides |  |  |  |  |  |  |  |  |  |  |  |  |  |  |  |  |  |  |  |
| --- | --- | --- | --- | --- | --- | --- | --- | --- | --- | --- | --- | --- | --- | --- | --- | --- | --- | --- | --- |

XP\_026580635.1

back to list

  

| Protein Coverage
| Supporting Peptides
|

Protein Coverage:

Supporting Peptides:

| Peptide | Uniq | -10lgP | Mass | Length | ppm | m/z | z | RT | Fraction | Scan | Source File | Area BRom | #Feature | #Feature BRom | Start | End | PTM | AScore | Found By |
| --- | --- | --- | --- | --- | --- | --- | --- | --- | --- | --- | --- | --- | --- | --- | --- | --- | --- | --- | --- |
| R.C(+57.02)IESLLAVFQR.Y | Y | 71.56 | 1334.7017 | 11 | 0.3 | 668.3583 | 2 | 75.75 | 1 | 52262 | 01122020\_RID\_2209\_BuCaKA02.raw | 1.3754E7 | 1 | 1 | 15 | 25 | Carbamidomethylation | C1:Carbamidomethylation:1000.00 | PEAKS DB |
| R.YTVGPTETERC(+57.02)IESLLAVFQR.Y | Y | 69.47 | 2468.2368 | 21 | 1.2 | 823.7538 | 3 | 86.96 | 1 | 61629 | 01122020\_RID\_2209\_BuCaKA02.raw | 3.6849E6 | 1 | 1 | 5 | 25 | Carbamidomethylation | C11:Carbamidomethylation:1000.00 | PEAKS DB |
| C.IESLLAVFQR.Y | Y | 53.59 | 1174.6710 | 10 | 1.2 | 588.3435 | 2 | 68.88 | 1 | 46588 | 01122020\_RID\_2209\_BuCaKA02.raw | 4.3068E6 | 1 | 1 | 16 | 25 |  |  | PEAKS DB |
| R.YTVGPTETERC(+57.02)IESLLAVFQRYAGK.E | Y | 40.61 | 2887.4539 | 25 | 6.3 | 722.8753 | 4 | 87.50 | 1 | 62078 | 01122020\_RID\_2209\_BuCaKA02.raw | 0 | 0 | 0 | 5 | 29 | Carbamidomethylation | C11:Carbamidomethylation:1000.00 | PEAKS DB |
| total 4 peptides |  |  |  |  |  |  |  |  |  |  |  |  |  |  |  |  |  |  |  |
| --- | --- | --- | --- | --- | --- | --- | --- | --- | --- | --- | --- | --- | --- | --- | --- | --- | --- | --- | --- |

XP\_026555793.1

back to list

  

| Protein Coverage
| Supporting Peptides
|

Protein Coverage:

Supporting Peptides:

| Peptide | Uniq | -10lgP | Mass | Length | ppm | m/z | z | RT | Fraction | Scan | Source File | Area BRom | #Feature | #Feature BRom | Start | End | PTM | AScore | Found By |
| --- | --- | --- | --- | --- | --- | --- | --- | --- | --- | --- | --- | --- | --- | --- | --- | --- | --- | --- | --- |
| R.GTTEHLLEIVLGR.C | Y | 82.40 | 1436.7987 | 13 | 3.0 | 719.4088 | 2 | 62.71 | 1 | 41582 | 01122020\_RID\_2209\_BuCaKA02.raw | 1.9671E8 | 1 | 1 | 56 | 68 |  |  | PEAKS DB |
| K.SSIFGSVEIVNLNPEKVSK.M | Y | 73.26 | 2046.0996 | 19 | -0.7 | 1024.0564 | 2 | 58.26 | 1 | 37931 | 01122020\_RID\_2209\_BuCaKA02.raw | 3.4484E7 | 2 | 2 | 222 | 240 |  |  | PEAKS DB |
| K.MQIWLMHDIGGPQRETC(+57.02)TGHSIAQLR.E | Y | 45.24 | 3034.4688 | 26 | 2.8 | 607.9027 | 5 | 42.43 | 1 | 25469 | 01122020\_RID\_2209\_BuCaKA02.raw | 9.0361E5 | 1 | 1 | 241 | 266 | Carbamidomethylation | C17:Carbamidomethylation:1000.00 | PEAKS DB |
| total 3 peptides |  |  |  |  |  |  |  |  |  |  |  |  |  |  |  |  |  |  |  |
| --- | --- | --- | --- | --- | --- | --- | --- | --- | --- | --- | --- | --- | --- | --- | --- | --- | --- | --- | --- |

XP\_032087784.1

back to list

  

| Protein Coverage
| Supporting Peptides
|

Protein Coverage:

Supporting Peptides:

| Peptide | Uniq | -10lgP | Mass | Length | ppm | m/z | z | RT | Fraction | Scan | Source File | Area BRom | #Feature | #Feature BRom | Start | End | PTM | AScore | Found By |
| --- | --- | --- | --- | --- | --- | --- | --- | --- | --- | --- | --- | --- | --- | --- | --- | --- | --- | --- | --- |
| R.SKPISSPVIIQFGHAETLQPLLSLMGYFKDKVPLNASNYR.H | Y | 72.41 | 4458.3774 | 40 | 5.4 | 744.0742 | 6 | 83.24 | 1 | 58555 | 01122020\_RID\_2209\_BuCaKA02.raw | 1.5375E6 | 1 | 1 | 350 | 389 |  |  | PEAKS DB |
| R.SKPISSPVIIQFGHAETLQPLLSLMGYFK.D | N | 64.44 | 3200.7307 | 29 | 2.9 | 801.1923 | 4 | 88.51 | 1 | 62877 | 01122020\_RID\_2209\_BuCaKA02.raw | 4.2933E6 | 1 | 1 | 350 | 378 |  |  | PEAKS DB |
| R.SKPISSPVIIQFGHAETLQPLLSL.M | N | 41.27 | 2574.4421 | 24 | 2.4 | 859.1567 | 3 | 77.36 | 1 | 53499 | 01122020\_RID\_2209\_BuCaKA02.raw | 1.8791E8 | 1 | 1 | 350 | 373 |  |  | PEAKS DB |
| total 3 peptides |  |  |  |  |  |  |  |  |  |  |  |  |  |  |  |  |  |  |  |
| --- | --- | --- | --- | --- | --- | --- | --- | --- | --- | --- | --- | --- | --- | --- | --- | --- | --- | --- | --- |

XP\_013925207.1

back to list

  

| Protein Coverage
| Supporting Peptides
|

Protein Coverage:

Supporting Peptides:

| Peptide | Uniq | -10lgP | Mass | Length | ppm | m/z | z | RT | Fraction | Scan | Source File | Area BRom | #Feature | #Feature BRom | Start | End | PTM | AScore | Found By |
| --- | --- | --- | --- | --- | --- | --- | --- | --- | --- | --- | --- | --- | --- | --- | --- | --- | --- | --- | --- |
| R.SKPISSPVIIQFGHAETLQPLLSLMGYFKDKVPLNASNYR.H | Y | 72.41 | 4458.3774 | 40 | 5.4 | 744.0742 | 6 | 83.24 | 1 | 58555 | 01122020\_RID\_2209\_BuCaKA02.raw | 1.5375E6 | 1 | 1 | 226 | 265 |  |  | PEAKS DB |
| R.SKPISSPVIIQFGHAETLQPLLSLMGYFK.D | N | 64.44 | 3200.7307 | 29 | 2.9 | 801.1923 | 4 | 88.51 | 1 | 62877 | 01122020\_RID\_2209\_BuCaKA02.raw | 4.2933E6 | 1 | 1 | 226 | 254 |  |  | PEAKS DB |
| R.SKPISSPVIIQFGHAETLQPLLSL.M | N | 41.27 | 2574.4421 | 24 | 2.4 | 859.1567 | 3 | 77.36 | 1 | 53499 | 01122020\_RID\_2209\_BuCaKA02.raw | 1.8791E8 | 1 | 1 | 226 | 249 |  |  | PEAKS DB |
| total 3 peptides |  |  |  |  |  |  |  |  |  |  |  |  |  |  |  |  |  |  |  |
| --- | --- | --- | --- | --- | --- | --- | --- | --- | --- | --- | --- | --- | --- | --- | --- | --- | --- | --- | --- |

XP\_025032622.1

back to list

  

| Protein Coverage
| Supporting Peptides
|

Protein Coverage:

Supporting Peptides:

| Peptide | Uniq | -10lgP | Mass | Length | ppm | m/z | z | RT | Fraction | Scan | Source File | Area BRom | #Feature | #Feature BRom | Start | End | PTM | AScore | Found By |
| --- | --- | --- | --- | --- | --- | --- | --- | --- | --- | --- | --- | --- | --- | --- | --- | --- | --- | --- | --- |
| F.LGIPFAEPPVGR.R | N | 65.37 | 1251.6975 | 12 | 1.1 | 626.8567 | 2 | 56.11 | 1 | 36154 | 01122020\_RID\_2209\_BuCaKA02.raw | 4.6223E8 | 1 | 1 | 57 | 68 |  |  | PEAKS DB |
| A.FLGIPFAEPPVGR.R | N | 62.09 | 1398.7659 | 13 | 0.0 | 700.3902 | 2 | 73.18 | 1 | 50153 | 01122020\_RID\_2209\_BuCaKA02.raw | 1.8064E7 | 1 | 1 | 56 | 68 |  |  | PEAKS DB |
| L.DGHISAFLGIPFAEPPVGR.R | Y | 56.95 | 1979.0264 | 19 | 1.1 | 660.6835 | 3 | 74.66 | 1 | 51410 | 01122020\_RID\_2209\_BuCaKA02.raw | 6.7052E6 | 1 | 1 | 50 | 68 |  |  | PEAKS DB |
| total 3 peptides |  |  |  |  |  |  |  |  |  |  |  |  |  |  |  |  |  |  |  |
| --- | --- | --- | --- | --- | --- | --- | --- | --- | --- | --- | --- | --- | --- | --- | --- | --- | --- | --- | --- |

XP\_025032620.1

back to list

  

| Protein Coverage
| Supporting Peptides
|

Protein Coverage:

Supporting Peptides:

| Peptide | Uniq | -10lgP | Mass | Length | ppm | m/z | z | RT | Fraction | Scan | Source File | Area BRom | #Feature | #Feature BRom | Start | End | PTM | AScore | Found By |
| --- | --- | --- | --- | --- | --- | --- | --- | --- | --- | --- | --- | --- | --- | --- | --- | --- | --- | --- | --- |
| F.LGIPFAEPPVGR.R | N | 65.37 | 1251.6975 | 12 | 1.1 | 626.8567 | 2 | 56.11 | 1 | 36154 | 01122020\_RID\_2209\_BuCaKA02.raw | 4.6223E8 | 1 | 1 | 57 | 68 |  |  | PEAKS DB |
| A.FLGIPFAEPPVGR.R | N | 62.09 | 1398.7659 | 13 | 0.0 | 700.3902 | 2 | 73.18 | 1 | 50153 | 01122020\_RID\_2209\_BuCaKA02.raw | 1.8064E7 | 1 | 1 | 56 | 68 |  |  | PEAKS DB |
| L.DGHISAFLGIPFAEPPVGR.R | Y | 56.95 | 1979.0264 | 19 | 1.1 | 660.6835 | 3 | 74.66 | 1 | 51410 | 01122020\_RID\_2209\_BuCaKA02.raw | 6.7052E6 | 1 | 1 | 50 | 68 |  |  | PEAKS DB |
| total 3 peptides |  |  |  |  |  |  |  |  |  |  |  |  |  |  |  |  |  |  |  |
| --- | --- | --- | --- | --- | --- | --- | --- | --- | --- | --- | --- | --- | --- | --- | --- | --- | --- | --- | --- |

XP\_025032621.1

back to list

  

| Protein Coverage
| Supporting Peptides
|

Protein Coverage:

Supporting Peptides:

| Peptide | Uniq | -10lgP | Mass | Length | ppm | m/z | z | RT | Fraction | Scan | Source File | Area BRom | #Feature | #Feature BRom | Start | End | PTM | AScore | Found By |
| --- | --- | --- | --- | --- | --- | --- | --- | --- | --- | --- | --- | --- | --- | --- | --- | --- | --- | --- | --- |
| F.LGIPFAEPPVGR.R | N | 65.37 | 1251.6975 | 12 | 1.1 | 626.8567 | 2 | 56.11 | 1 | 36154 | 01122020\_RID\_2209\_BuCaKA02.raw | 4.6223E8 | 1 | 1 | 57 | 68 |  |  | PEAKS DB |
| A.FLGIPFAEPPVGR.R | N | 62.09 | 1398.7659 | 13 | 0.0 | 700.3902 | 2 | 73.18 | 1 | 50153 | 01122020\_RID\_2209\_BuCaKA02.raw | 1.8064E7 | 1 | 1 | 56 | 68 |  |  | PEAKS DB |
| L.DGHISAFLGIPFAEPPVGR.R | Y | 56.95 | 1979.0264 | 19 | 1.1 | 660.6835 | 3 | 74.66 | 1 | 51410 | 01122020\_RID\_2209\_BuCaKA02.raw | 6.7052E6 | 1 | 1 | 50 | 68 |  |  | PEAKS DB |
| total 3 peptides |  |  |  |  |  |  |  |  |  |  |  |  |  |  |  |  |  |  |  |
| --- | --- | --- | --- | --- | --- | --- | --- | --- | --- | --- | --- | --- | --- | --- | --- | --- | --- | --- | --- |

JAC94932.1

back to list

  

| Protein Coverage
| Supporting Peptides
|

Protein Coverage:

Supporting Peptides:

| Peptide | Uniq | -10lgP | Mass | Length | ppm | m/z | z | RT | Fraction | Scan | Source File | Area BRom | #Feature | #Feature BRom | Start | End | PTM | AScore | Found By |
| --- | --- | --- | --- | --- | --- | --- | --- | --- | --- | --- | --- | --- | --- | --- | --- | --- | --- | --- | --- |
| F.LGIPFAEPPVGR.L | N | 65.37 | 1251.6975 | 12 | 1.1 | 626.8567 | 2 | 56.11 | 1 | 36154 | 01122020\_RID\_2209\_BuCaKA02.raw | 4.6223E8 | 1 | 1 | 65 | 76 |  |  | PEAKS DB |
| A.FLGIPFAEPPVGR.L | N | 62.09 | 1398.7659 | 13 | 0.0 | 700.3902 | 2 | 73.18 | 1 | 50153 | 01122020\_RID\_2209\_BuCaKA02.raw | 1.8064E7 | 1 | 1 | 64 | 76 |  |  | PEAKS DB |
| L.DGHISAFLGIPFAEPPVGR.L | Y | 56.95 | 1979.0264 | 19 | 1.1 | 660.6835 | 3 | 74.66 | 1 | 51410 | 01122020\_RID\_2209\_BuCaKA02.raw | 6.7052E6 | 1 | 1 | 58 | 76 |  |  | PEAKS DB |
| total 3 peptides |  |  |  |  |  |  |  |  |  |  |  |  |  |  |  |  |  |  |  |
| --- | --- | --- | --- | --- | --- | --- | --- | --- | --- | --- | --- | --- | --- | --- | --- | --- | --- | --- | --- |

XP\_025032619.1

back to list

  

| Protein Coverage
| Supporting Peptides
|

Protein Coverage:

Supporting Peptides:

| Peptide | Uniq | -10lgP | Mass | Length | ppm | m/z | z | RT | Fraction | Scan | Source File | Area BRom | #Feature | #Feature BRom | Start | End | PTM | AScore | Found By |
| --- | --- | --- | --- | --- | --- | --- | --- | --- | --- | --- | --- | --- | --- | --- | --- | --- | --- | --- | --- |
| F.LGIPFAEPPVGR.R | N | 65.37 | 1251.6975 | 12 | 1.1 | 626.8567 | 2 | 56.11 | 1 | 36154 | 01122020\_RID\_2209\_BuCaKA02.raw | 4.6223E8 | 1 | 1 | 97 | 108 |  |  | PEAKS DB |
| A.FLGIPFAEPPVGR.R | N | 62.09 | 1398.7659 | 13 | 0.0 | 700.3902 | 2 | 73.18 | 1 | 50153 | 01122020\_RID\_2209\_BuCaKA02.raw | 1.8064E7 | 1 | 1 | 96 | 108 |  |  | PEAKS DB |
| L.DGHISAFLGIPFAEPPVGR.R | Y | 56.95 | 1979.0264 | 19 | 1.1 | 660.6835 | 3 | 74.66 | 1 | 51410 | 01122020\_RID\_2209\_BuCaKA02.raw | 6.7052E6 | 1 | 1 | 90 | 108 |  |  | PEAKS DB |
| total 3 peptides |  |  |  |  |  |  |  |  |  |  |  |  |  |  |  |  |  |  |  |
| --- | --- | --- | --- | --- | --- | --- | --- | --- | --- | --- | --- | --- | --- | --- | --- | --- | --- | --- | --- |

ETE64512.1

back to list

  

| Protein Coverage
| Supporting Peptides
|

Protein Coverage:

Supporting Peptides:

| Peptide | Uniq | -10lgP | Mass | Length | ppm | m/z | z | RT | Fraction | Scan | Source File | Area BRom | #Feature | #Feature BRom | Start | End | PTM | AScore | Found By |
| --- | --- | --- | --- | --- | --- | --- | --- | --- | --- | --- | --- | --- | --- | --- | --- | --- | --- | --- | --- |
| R.QKLIAQDYKVSYSLAK.S | Y | 74.11 | 1854.0250 | 16 | 1.4 | 619.0165 | 3 | 23.97 | 1 | 11320 | 01122020\_RID\_2209\_BuCaKA02.raw | 3.243E6 | 1 | 1 | 213 | 228 |  |  | PEAKS DB |
| R.LSYLLMC(+57.02)LESAVHRGR.Q | Y | 70.22 | 1903.9761 | 16 | 1.1 | 635.6667 | 3 | 54.54 | 1 | 34825 | 01122020\_RID\_2209\_BuCaKA02.raw | 6.4413E6 | 1 | 1 | 252 | 267 | Carbamidomethylation | C7:Carbamidomethylation:1000.00 | PEAKS DB |
| total 2 peptides |  |  |  |  |  |  |  |  |  |  |  |  |  |  |  |  |  |  |  |
| --- | --- | --- | --- | --- | --- | --- | --- | --- | --- | --- | --- | --- | --- | --- | --- | --- | --- | --- | --- |

XP\_015667558.1

back to list

  

| Protein Coverage
| Supporting Peptides
|

Protein Coverage:

Supporting Peptides:

| Peptide | Uniq | -10lgP | Mass | Length | ppm | m/z | z | RT | Fraction | Scan | Source File | Area BRom | #Feature | #Feature BRom | Start | End | PTM | AScore | Found By |
| --- | --- | --- | --- | --- | --- | --- | --- | --- | --- | --- | --- | --- | --- | --- | --- | --- | --- | --- | --- |
| R.QKLIAQDYKVSYSLAK.S | Y | 74.11 | 1854.0250 | 16 | 1.4 | 619.0165 | 3 | 23.97 | 1 | 11320 | 01122020\_RID\_2209\_BuCaKA02.raw | 3.243E6 | 1 | 1 | 213 | 228 |  |  | PEAKS DB |
| R.LSYLLMC(+57.02)LESAVHRGR.Q | Y | 70.22 | 1903.9761 | 16 | 1.1 | 635.6667 | 3 | 54.54 | 1 | 34825 | 01122020\_RID\_2209\_BuCaKA02.raw | 6.4413E6 | 1 | 1 | 252 | 267 | Carbamidomethylation | C7:Carbamidomethylation:1000.00 | PEAKS DB |
| total 2 peptides |  |  |  |  |  |  |  |  |  |  |  |  |  |  |  |  |  |  |  |
| --- | --- | --- | --- | --- | --- | --- | --- | --- | --- | --- | --- | --- | --- | --- | --- | --- | --- | --- | --- |

XP\_032086218.1

back to list

  

| Protein Coverage
| Supporting Peptides
|

Protein Coverage:

Supporting Peptides:

| Peptide | Uniq | -10lgP | Mass | Length | ppm | m/z | z | RT | Fraction | Scan | Source File | Area BRom | #Feature | #Feature BRom | Start | End | PTM | AScore | Found By |
| --- | --- | --- | --- | --- | --- | --- | --- | --- | --- | --- | --- | --- | --- | --- | --- | --- | --- | --- | --- |
| R.QKLIAQDYKVSYSLAK.S | Y | 74.11 | 1854.0250 | 16 | 1.4 | 619.0165 | 3 | 23.97 | 1 | 11320 | 01122020\_RID\_2209\_BuCaKA02.raw | 3.243E6 | 1 | 1 | 335 | 350 |  |  | PEAKS DB |
| R.LSYLLMC(+57.02)LESAVHRGR.Q | Y | 70.22 | 1903.9761 | 16 | 1.1 | 635.6667 | 3 | 54.54 | 1 | 34825 | 01122020\_RID\_2209\_BuCaKA02.raw | 6.4413E6 | 1 | 1 | 374 | 389 | Carbamidomethylation | C7:Carbamidomethylation:1000.00 | PEAKS DB |
| total 2 peptides |  |  |  |  |  |  |  |  |  |  |  |  |  |  |  |  |  |  |  |
| --- | --- | --- | --- | --- | --- | --- | --- | --- | --- | --- | --- | --- | --- | --- | --- | --- | --- | --- | --- |

XP\_034281438.1

back to list

  

| Protein Coverage
| Supporting Peptides
|

Protein Coverage:

Supporting Peptides:

| Peptide | Uniq | -10lgP | Mass | Length | ppm | m/z | z | RT | Fraction | Scan | Source File | Area BRom | #Feature | #Feature BRom | Start | End | PTM | AScore | Found By |
| --- | --- | --- | --- | --- | --- | --- | --- | --- | --- | --- | --- | --- | --- | --- | --- | --- | --- | --- | --- |
| R.QKLIAQDYKVSYSLAK.S | Y | 74.11 | 1854.0250 | 16 | 1.4 | 619.0165 | 3 | 23.97 | 1 | 11320 | 01122020\_RID\_2209\_BuCaKA02.raw | 3.243E6 | 1 | 1 | 340 | 355 |  |  | PEAKS DB |
| R.LSYLLMC(+57.02)LESAVHRGR.Q | Y | 70.22 | 1903.9761 | 16 | 1.1 | 635.6667 | 3 | 54.54 | 1 | 34825 | 01122020\_RID\_2209\_BuCaKA02.raw | 6.4413E6 | 1 | 1 | 379 | 394 | Carbamidomethylation | C7:Carbamidomethylation:1000.00 | PEAKS DB |
| total 2 peptides |  |  |  |  |  |  |  |  |  |  |  |  |  |  |  |  |  |  |  |
| --- | --- | --- | --- | --- | --- | --- | --- | --- | --- | --- | --- | --- | --- | --- | --- | --- | --- | --- | --- |

LAB62693.1

back to list

  

| Protein Coverage
| Supporting Peptides
|

Protein Coverage:

Supporting Peptides:

| Peptide | Uniq | -10lgP | Mass | Length | ppm | m/z | z | RT | Fraction | Scan | Source File | Area BRom | #Feature | #Feature BRom | Start | End | PTM | AScore | Found By |
| --- | --- | --- | --- | --- | --- | --- | --- | --- | --- | --- | --- | --- | --- | --- | --- | --- | --- | --- | --- |
| R.QKLIAQDYKVSYSLAK.S | Y | 74.11 | 1854.0250 | 16 | 1.4 | 619.0165 | 3 | 23.97 | 1 | 11320 | 01122020\_RID\_2209\_BuCaKA02.raw | 3.243E6 | 1 | 1 | 13 | 28 |  |  | PEAKS DB |
| R.LSYLLMC(+57.02)LESAVHRGR.Q | Y | 70.22 | 1903.9761 | 16 | 1.1 | 635.6667 | 3 | 54.54 | 1 | 34825 | 01122020\_RID\_2209\_BuCaKA02.raw | 6.4413E6 | 1 | 1 | 52 | 67 | Carbamidomethylation | C7:Carbamidomethylation:1000.00 | PEAKS DB |
| total 2 peptides |  |  |  |  |  |  |  |  |  |  |  |  |  |  |  |  |  |  |  |
| --- | --- | --- | --- | --- | --- | --- | --- | --- | --- | --- | --- | --- | --- | --- | --- | --- | --- | --- | --- |

LAB62690.1

back to list

  

| Protein Coverage
| Supporting Peptides
|

Protein Coverage:

Supporting Peptides:

| Peptide | Uniq | -10lgP | Mass | Length | ppm | m/z | z | RT | Fraction | Scan | Source File | Area BRom | #Feature | #Feature BRom | Start | End | PTM | AScore | Found By |
| --- | --- | --- | --- | --- | --- | --- | --- | --- | --- | --- | --- | --- | --- | --- | --- | --- | --- | --- | --- |
| R.QKLIAQDYKVSYSLAK.S | Y | 74.11 | 1854.0250 | 16 | 1.4 | 619.0165 | 3 | 23.97 | 1 | 11320 | 01122020\_RID\_2209\_BuCaKA02.raw | 3.243E6 | 1 | 1 | 240 | 255 |  |  | PEAKS DB |
| R.LSYLLMC(+57.02)LESAVHRGR.Q | Y | 70.22 | 1903.9761 | 16 | 1.1 | 635.6667 | 3 | 54.54 | 1 | 34825 | 01122020\_RID\_2209\_BuCaKA02.raw | 6.4413E6 | 1 | 1 | 279 | 294 | Carbamidomethylation | C7:Carbamidomethylation:1000.00 | PEAKS DB |
| total 2 peptides |  |  |  |  |  |  |  |  |  |  |  |  |  |  |  |  |  |  |  |
| --- | --- | --- | --- | --- | --- | --- | --- | --- | --- | --- | --- | --- | --- | --- | --- | --- | --- | --- | --- |

XP\_026570202.1

back to list

  

| Protein Coverage
| Supporting Peptides
|

Protein Coverage:

Supporting Peptides:

| Peptide | Uniq | -10lgP | Mass | Length | ppm | m/z | z | RT | Fraction | Scan | Source File | Area BRom | #Feature | #Feature BRom | Start | End | PTM | AScore | Found By |
| --- | --- | --- | --- | --- | --- | --- | --- | --- | --- | --- | --- | --- | --- | --- | --- | --- | --- | --- | --- |
| R.QKLIAQDYKVSYSLAK.S | Y | 74.11 | 1854.0250 | 16 | 1.4 | 619.0165 | 3 | 23.97 | 1 | 11320 | 01122020\_RID\_2209\_BuCaKA02.raw | 3.243E6 | 1 | 1 | 345 | 360 |  |  | PEAKS DB |
| R.LSYLLMC(+57.02)LESAVHRGR.Q | Y | 70.22 | 1903.9761 | 16 | 1.1 | 635.6667 | 3 | 54.54 | 1 | 34825 | 01122020\_RID\_2209\_BuCaKA02.raw | 6.4413E6 | 1 | 1 | 384 | 399 | Carbamidomethylation | C7:Carbamidomethylation:1000.00 | PEAKS DB |
| total 2 peptides |  |  |  |  |  |  |  |  |  |  |  |  |  |  |  |  |  |  |  |
| --- | --- | --- | --- | --- | --- | --- | --- | --- | --- | --- | --- | --- | --- | --- | --- | --- | --- | --- | --- |

XP\_026535930.1

back to list

  

| Protein Coverage
| Supporting Peptides
|

Protein Coverage:

Supporting Peptides:

| Peptide | Uniq | -10lgP | Mass | Length | ppm | m/z | z | RT | Fraction | Scan | Source File | Area BRom | #Feature | #Feature BRom | Start | End | PTM | AScore | Found By |
| --- | --- | --- | --- | --- | --- | --- | --- | --- | --- | --- | --- | --- | --- | --- | --- | --- | --- | --- | --- |
| R.QKLIAQDYKVSYSLAK.S | Y | 74.11 | 1854.0250 | 16 | 1.4 | 619.0165 | 3 | 23.97 | 1 | 11320 | 01122020\_RID\_2209\_BuCaKA02.raw | 3.243E6 | 1 | 1 | 341 | 356 |  |  | PEAKS DB |
| R.LSYLLMC(+57.02)LESAVHRGR.Q | Y | 70.22 | 1903.9761 | 16 | 1.1 | 635.6667 | 3 | 54.54 | 1 | 34825 | 01122020\_RID\_2209\_BuCaKA02.raw | 6.4413E6 | 1 | 1 | 380 | 395 | Carbamidomethylation | C7:Carbamidomethylation:1000.00 | PEAKS DB |
| total 2 peptides |  |  |  |  |  |  |  |  |  |  |  |  |  |  |  |  |  |  |  |
| --- | --- | --- | --- | --- | --- | --- | --- | --- | --- | --- | --- | --- | --- | --- | --- | --- | --- | --- | --- |

XP\_025027126.1

back to list

  

| Protein Coverage
| Supporting Peptides
|

Protein Coverage:

Supporting Peptides:

| Peptide | Uniq | -10lgP | Mass | Length | ppm | m/z | z | RT | Fraction | Scan | Source File | Area BRom | #Feature | #Feature BRom | Start | End | PTM | AScore | Found By |
| --- | --- | --- | --- | --- | --- | --- | --- | --- | --- | --- | --- | --- | --- | --- | --- | --- | --- | --- | --- |
| R.QKLIAQDYKVSYSLAK.S | Y | 74.11 | 1854.0250 | 16 | 1.4 | 619.0165 | 3 | 23.97 | 1 | 11320 | 01122020\_RID\_2209\_BuCaKA02.raw | 3.243E6 | 1 | 1 | 137 | 152 |  |  | PEAKS DB |
| R.LSYLLMC(+57.02)LESAVHRGR.Q | Y | 70.22 | 1903.9761 | 16 | 1.1 | 635.6667 | 3 | 54.54 | 1 | 34825 | 01122020\_RID\_2209\_BuCaKA02.raw | 6.4413E6 | 1 | 1 | 176 | 191 | Carbamidomethylation | C7:Carbamidomethylation:1000.00 | PEAKS DB |
| total 2 peptides |  |  |  |  |  |  |  |  |  |  |  |  |  |  |  |  |  |  |  |
| --- | --- | --- | --- | --- | --- | --- | --- | --- | --- | --- | --- | --- | --- | --- | --- | --- | --- | --- | --- |

LAB18302.1

back to list

  

| Protein Coverage
| Supporting Peptides
|

Protein Coverage:

Supporting Peptides:

| Peptide | Uniq | -10lgP | Mass | Length | ppm | m/z | z | RT | Fraction | Scan | Source File | Area BRom | #Feature | #Feature BRom | Start | End | PTM | AScore | Found By |
| --- | --- | --- | --- | --- | --- | --- | --- | --- | --- | --- | --- | --- | --- | --- | --- | --- | --- | --- | --- |
| R.QKLIAQDYKVSYSLAK.S | Y | 74.11 | 1854.0250 | 16 | 1.4 | 619.0165 | 3 | 23.97 | 1 | 11320 | 01122020\_RID\_2209\_BuCaKA02.raw | 3.243E6 | 1 | 1 | 31 | 46 |  |  | PEAKS DB |
| R.LSYLLMC(+57.02)LESAVHRGR.V | Y | 70.22 | 1903.9761 | 16 | 1.1 | 635.6667 | 3 | 54.54 | 1 | 34825 | 01122020\_RID\_2209\_BuCaKA02.raw | 6.4413E6 | 1 | 1 | 70 | 85 | Carbamidomethylation | C7:Carbamidomethylation:1000.00 | PEAKS DB |
| total 2 peptides |  |  |  |  |  |  |  |  |  |  |  |  |  |  |  |  |  |  |  |
| --- | --- | --- | --- | --- | --- | --- | --- | --- | --- | --- | --- | --- | --- | --- | --- | --- | --- | --- | --- |

XP\_015686495.1

back to list

  

| Protein Coverage
| Supporting Peptides
|

Protein Coverage:

Supporting Peptides:

| Peptide | Uniq | -10lgP | Mass | Length | ppm | m/z | z | RT | Fraction | Scan | Source File | Area BRom | #Feature | #Feature BRom | Start | End | PTM | AScore | Found By |
| --- | --- | --- | --- | --- | --- | --- | --- | --- | --- | --- | --- | --- | --- | --- | --- | --- | --- | --- | --- |
| R.QKLIAQDYKVSYSLAK.S | Y | 74.11 | 1854.0250 | 16 | 1.4 | 619.0165 | 3 | 23.97 | 1 | 11320 | 01122020\_RID\_2209\_BuCaKA02.raw | 3.243E6 | 1 | 1 | 101 | 116 |  |  | PEAKS DB |
| R.LSYLLMC(+57.02)LESAVHRGR.V | Y | 70.22 | 1903.9761 | 16 | 1.1 | 635.6667 | 3 | 54.54 | 1 | 34825 | 01122020\_RID\_2209\_BuCaKA02.raw | 6.4413E6 | 1 | 1 | 140 | 155 | Carbamidomethylation | C7:Carbamidomethylation:1000.00 | PEAKS DB |
| total 2 peptides |  |  |  |  |  |  |  |  |  |  |  |  |  |  |  |  |  |  |  |
| --- | --- | --- | --- | --- | --- | --- | --- | --- | --- | --- | --- | --- | --- | --- | --- | --- | --- | --- | --- |

LAB18300.1

back to list

  

| Protein Coverage
| Supporting Peptides
|

Protein Coverage:

Supporting Peptides:

| Peptide | Uniq | -10lgP | Mass | Length | ppm | m/z | z | RT | Fraction | Scan | Source File | Area BRom | #Feature | #Feature BRom | Start | End | PTM | AScore | Found By |
| --- | --- | --- | --- | --- | --- | --- | --- | --- | --- | --- | --- | --- | --- | --- | --- | --- | --- | --- | --- |
| R.QKLIAQDYKVSYSLAK.S | Y | 74.11 | 1854.0250 | 16 | 1.4 | 619.0165 | 3 | 23.97 | 1 | 11320 | 01122020\_RID\_2209\_BuCaKA02.raw | 3.243E6 | 1 | 1 | 31 | 46 |  |  | PEAKS DB |
| R.LSYLLMC(+57.02)LESAVHRGR.Q | Y | 70.22 | 1903.9761 | 16 | 1.1 | 635.6667 | 3 | 54.54 | 1 | 34825 | 01122020\_RID\_2209\_BuCaKA02.raw | 6.4413E6 | 1 | 1 | 70 | 85 | Carbamidomethylation | C7:Carbamidomethylation:1000.00 | PEAKS DB |
| total 2 peptides |  |  |  |  |  |  |  |  |  |  |  |  |  |  |  |  |  |  |  |
| --- | --- | --- | --- | --- | --- | --- | --- | --- | --- | --- | --- | --- | --- | --- | --- | --- | --- | --- | --- |

XP\_026547933.1

back to list

  

| Protein Coverage
| Supporting Peptides
|

Protein Coverage:

Supporting Peptides:

| Peptide | Uniq | -10lgP | Mass | Length | ppm | m/z | z | RT | Fraction | Scan | Source File | Area BRom | #Feature | #Feature BRom | Start | End | PTM | AScore | Found By |
| --- | --- | --- | --- | --- | --- | --- | --- | --- | --- | --- | --- | --- | --- | --- | --- | --- | --- | --- | --- |
| K.DPWWYVDLGR.Q | Y | 74.51 | 1305.6141 | 10 | 1.0 | 653.8150 | 2 | 81.69 | 1 | 57247 | 01122020\_RID\_2209\_BuCaKA02.raw | 3.5002E6 | 1 | 1 | 60 | 69 |  |  | PEAKS DB |
| R.GRPAFQSSTIESQFNSVAR.K | Y | 68.79 | 2081.0291 | 19 | 0.8 | 694.6842 | 3 | 34.62 | 1 | 19412 | 01122020\_RID\_2209\_BuCaKA02.raw | 1.1597E7 | 1 | 1 | 17 | 35 |  |  | PEAKS DB |
| total 2 peptides |  |  |  |  |  |  |  |  |  |  |  |  |  |  |  |  |  |  |  |
| --- | --- | --- | --- | --- | --- | --- | --- | --- | --- | --- | --- | --- | --- | --- | --- | --- | --- | --- | --- |

XP\_026578807.1

back to list

  

| Protein Coverage
| Supporting Peptides
|

Protein Coverage:

Supporting Peptides:

| Peptide | Uniq | -10lgP | Mass | Length | ppm | m/z | z | RT | Fraction | Scan | Source File | Area BRom | #Feature | #Feature BRom | Start | End | PTM | AScore | Found By |
| --- | --- | --- | --- | --- | --- | --- | --- | --- | --- | --- | --- | --- | --- | --- | --- | --- | --- | --- | --- |
| K.DPWWYVDLGR.Q | Y | 74.51 | 1305.6141 | 10 | 1.0 | 653.8150 | 2 | 81.69 | 1 | 57247 | 01122020\_RID\_2209\_BuCaKA02.raw | 3.5002E6 | 1 | 1 | 463 | 472 |  |  | PEAKS DB |
| R.GRPAFQSSTIESQFNSVAR.K | Y | 68.79 | 2081.0291 | 19 | 0.8 | 694.6842 | 3 | 34.62 | 1 | 19412 | 01122020\_RID\_2209\_BuCaKA02.raw | 1.1597E7 | 1 | 1 | 420 | 438 |  |  | PEAKS DB |
| total 2 peptides |  |  |  |  |  |  |  |  |  |  |  |  |  |  |  |  |  |  |  |
| --- | --- | --- | --- | --- | --- | --- | --- | --- | --- | --- | --- | --- | --- | --- | --- | --- | --- | --- | --- |

LAB57810.1

back to list

  

| Protein Coverage
| Supporting Peptides
|

Protein Coverage:

Supporting Peptides:

| Peptide | Uniq | -10lgP | Mass | Length | ppm | m/z | z | RT | Fraction | Scan | Source File | Area BRom | #Feature | #Feature BRom | Start | End | PTM | AScore | Found By |
| --- | --- | --- | --- | --- | --- | --- | --- | --- | --- | --- | --- | --- | --- | --- | --- | --- | --- | --- | --- |
| K.FVLDPLITHTLPFEKINEGFDLLR.S | Y | 78.40 | 2826.5320 | 24 | 2.2 | 707.6418 | 4 | 87.54 | 1 | 62075 | 01122020\_RID\_2209\_BuCaKA02.raw | 1.504E7 | 2 | 2 | 287 | 310 |  |  | PEAKS DB |
| K.KFVLDPLITHTLPFEKINEGFDLLR.S | Y | 55.68 | 2954.6270 | 25 | 0.0 | 739.6640 | 4 | 78.56 | 1 | 54771 | 01122020\_RID\_2209\_BuCaKA02.raw | 1.3431E6 | 1 | 1 | 286 | 310 |  |  | PEAKS DB |
| total 2 peptides |  |  |  |  |  |  |  |  |  |  |  |  |  |  |  |  |  |  |  |
| --- | --- | --- | --- | --- | --- | --- | --- | --- | --- | --- | --- | --- | --- | --- | --- | --- | --- | --- | --- |

P80512.1

back to list

  

| Protein Coverage
| Supporting Peptides
|

Protein Coverage:

Supporting Peptides:

| Peptide | Uniq | -10lgP | Mass | Length | ppm | m/z | z | RT | Fraction | Scan | Source File | Area BRom | #Feature | #Feature BRom | Start | End | PTM | AScore | Found By |
| --- | --- | --- | --- | --- | --- | --- | --- | --- | --- | --- | --- | --- | --- | --- | --- | --- | --- | --- | --- |
| K.FVLDPLITHTLPFEKINEGFDLLR.S | Y | 78.40 | 2826.5320 | 24 | 2.2 | 707.6418 | 4 | 87.54 | 1 | 62075 | 01122020\_RID\_2209\_BuCaKA02.raw | 1.504E7 | 2 | 2 | 341 | 364 |  |  | PEAKS DB |
| K.KFVLDPLITHTLPFEKINEGFDLLR.S | Y | 55.68 | 2954.6270 | 25 | 0.0 | 739.6640 | 4 | 78.56 | 1 | 54771 | 01122020\_RID\_2209\_BuCaKA02.raw | 1.3431E6 | 1 | 1 | 340 | 364 |  |  | PEAKS DB |
| total 2 peptides |  |  |  |  |  |  |  |  |  |  |  |  |  |  |  |  |  |  |  |
| --- | --- | --- | --- | --- | --- | --- | --- | --- | --- | --- | --- | --- | --- | --- | --- | --- | --- | --- | --- |

XP\_026528465.1

back to list

  

| Protein Coverage
| Supporting Peptides
|

Protein Coverage:

Supporting Peptides:

| Peptide | Uniq | -10lgP | Mass | Length | ppm | m/z | z | RT | Fraction | Scan | Source File | Area BRom | #Feature | #Feature BRom | Start | End | PTM | AScore | Found By |
| --- | --- | --- | --- | --- | --- | --- | --- | --- | --- | --- | --- | --- | --- | --- | --- | --- | --- | --- | --- |
| K.FVLDPLITHTLPFEKINEGFDLLR.S | Y | 78.40 | 2826.5320 | 24 | 2.2 | 707.6418 | 4 | 87.54 | 1 | 62075 | 01122020\_RID\_2209\_BuCaKA02.raw | 1.504E7 | 2 | 2 | 342 | 365 |  |  | PEAKS DB |
| K.KFVLDPLITHTLPFEKINEGFDLLR.S | Y | 55.68 | 2954.6270 | 25 | 0.0 | 739.6640 | 4 | 78.56 | 1 | 54771 | 01122020\_RID\_2209\_BuCaKA02.raw | 1.3431E6 | 1 | 1 | 341 | 365 |  |  | PEAKS DB |
| total 2 peptides |  |  |  |  |  |  |  |  |  |  |  |  |  |  |  |  |  |  |  |
| --- | --- | --- | --- | --- | --- | --- | --- | --- | --- | --- | --- | --- | --- | --- | --- | --- | --- | --- | --- |

LAA59635.1

back to list

  

| Protein Coverage
| Supporting Peptides
|

Protein Coverage:

Supporting Peptides:

| Peptide | Uniq | -10lgP | Mass | Length | ppm | m/z | z | RT | Fraction | Scan | Source File | Area BRom | #Feature | #Feature BRom | Start | End | PTM | AScore | Found By |
| --- | --- | --- | --- | --- | --- | --- | --- | --- | --- | --- | --- | --- | --- | --- | --- | --- | --- | --- | --- |
| K.FVLDPLITHTLPFEKINEGFDLLR.S | Y | 78.40 | 2826.5320 | 24 | 2.2 | 707.6418 | 4 | 87.54 | 1 | 62075 | 01122020\_RID\_2209\_BuCaKA02.raw | 1.504E7 | 2 | 2 | 342 | 365 |  |  | PEAKS DB |
| K.KFVLDPLITHTLPFEKINEGFDLLR.S | Y | 55.68 | 2954.6270 | 25 | 0.0 | 739.6640 | 4 | 78.56 | 1 | 54771 | 01122020\_RID\_2209\_BuCaKA02.raw | 1.3431E6 | 1 | 1 | 341 | 365 |  |  | PEAKS DB |
| total 2 peptides |  |  |  |  |  |  |  |  |  |  |  |  |  |  |  |  |  |  |  |
| --- | --- | --- | --- | --- | --- | --- | --- | --- | --- | --- | --- | --- | --- | --- | --- | --- | --- | --- | --- |

XP\_026554078.1

back to list

  

| Protein Coverage
| Supporting Peptides
|

Protein Coverage:

Supporting Peptides:

| Peptide | Uniq | -10lgP | Mass | Length | ppm | m/z | z | RT | Fraction | Scan | Source File | Area BRom | #Feature | #Feature BRom | Start | End | PTM | AScore | Found By |
| --- | --- | --- | --- | --- | --- | --- | --- | --- | --- | --- | --- | --- | --- | --- | --- | --- | --- | --- | --- |
| K.FVLDPLITHTLPFEKINEGFDLLR.S | Y | 78.40 | 2826.5320 | 24 | 2.2 | 707.6418 | 4 | 87.54 | 1 | 62075 | 01122020\_RID\_2209\_BuCaKA02.raw | 1.504E7 | 2 | 2 | 342 | 365 |  |  | PEAKS DB |
| K.KFVLDPLITHTLPFEKINEGFDLLR.S | Y | 55.68 | 2954.6270 | 25 | 0.0 | 739.6640 | 4 | 78.56 | 1 | 54771 | 01122020\_RID\_2209\_BuCaKA02.raw | 1.3431E6 | 1 | 1 | 341 | 365 |  |  | PEAKS DB |
| total 2 peptides |  |  |  |  |  |  |  |  |  |  |  |  |  |  |  |  |  |  |  |
| --- | --- | --- | --- | --- | --- | --- | --- | --- | --- | --- | --- | --- | --- | --- | --- | --- | --- | --- | --- |

T\_81

back to list

  

| Protein Coverage
| Supporting Peptides
|

Protein Coverage:

Supporting Peptides:

| Peptide | Uniq | -10lgP | Mass | Length | ppm | m/z | z | RT | Fraction | Scan | Source File | Area BRom | #Feature | #Feature BRom | Start | End | PTM | AScore | Found By |
| --- | --- | --- | --- | --- | --- | --- | --- | --- | --- | --- | --- | --- | --- | --- | --- | --- | --- | --- | --- |
| R.SIEDGTLYIIEQVPNLVEYSDQTTVLR.K | Y | 77.14 | 3094.5710 | 27 | 0.5 | 1032.5315 | 3 | 103.50 | 1 | 75181 | 01122020\_RID\_2209\_BuCaKA02.raw | 2.7796E7 | 2 | 2 | 373 | 399 |  |  | PEAKS DB |
| R.SIEDGTLYIIEQVPNLVEYSDQTTVLRK.G | Y | 55.51 | 3222.6660 | 28 | 1.4 | 1075.2308 | 3 | 90.50 | 1 | 64666 | 01122020\_RID\_2209\_BuCaKA02.raw | 1.5805E7 | 1 | 1 | 373 | 400 |  |  | PEAKS DB |
| total 2 peptides |  |  |  |  |  |  |  |  |  |  |  |  |  |  |  |  |  |  |  |
| --- | --- | --- | --- | --- | --- | --- | --- | --- | --- | --- | --- | --- | --- | --- | --- | --- | --- | --- | --- |

T\_83

back to list

  

| Protein Coverage
| Supporting Peptides
|

Protein Coverage:

Supporting Peptides:

| Peptide | Uniq | -10lgP | Mass | Length | ppm | m/z | z | RT | Fraction | Scan | Source File | Area BRom | #Feature | #Feature BRom | Start | End | PTM | AScore | Found By |
| --- | --- | --- | --- | --- | --- | --- | --- | --- | --- | --- | --- | --- | --- | --- | --- | --- | --- | --- | --- |
| R.SIEDGTLYIIEQVPNLVEYSDQTTVLR.K | Y | 77.14 | 3094.5710 | 27 | 0.5 | 1032.5315 | 3 | 103.50 | 1 | 75181 | 01122020\_RID\_2209\_BuCaKA02.raw | 2.7796E7 | 2 | 2 | 373 | 399 |  |  | PEAKS DB |
| R.SIEDGTLYIIEQVPNLVEYSDQTTVLRK.G | Y | 55.51 | 3222.6660 | 28 | 1.4 | 1075.2308 | 3 | 90.50 | 1 | 64666 | 01122020\_RID\_2209\_BuCaKA02.raw | 1.5805E7 | 1 | 1 | 373 | 400 |  |  | PEAKS DB |
| total 2 peptides |  |  |  |  |  |  |  |  |  |  |  |  |  |  |  |  |  |  |  |
| --- | --- | --- | --- | --- | --- | --- | --- | --- | --- | --- | --- | --- | --- | --- | --- | --- | --- | --- | --- |

XP\_026545897.1

back to list

  

| Protein Coverage
| Supporting Peptides
|

Protein Coverage:

Supporting Peptides:

| Peptide | Uniq | -10lgP | Mass | Length | ppm | m/z | z | RT | Fraction | Scan | Source File | Area BRom | #Feature | #Feature BRom | Start | End | PTM | AScore | Found By |
| --- | --- | --- | --- | --- | --- | --- | --- | --- | --- | --- | --- | --- | --- | --- | --- | --- | --- | --- | --- |
| R.FFAHFGNLSGPSAIC(+57.02)GNPQVK.A | Y | 67.83 | 2247.0894 | 21 | 0.8 | 750.0377 | 3 | 45.80 | 1 | 28096 | 01122020\_RID\_2209\_BuCaKA02.raw | 7.6599E7 | 1 | 1 | 42 | 62 | Carbamidomethylation | C15:Carbamidomethylation:1000.00 | PEAKS DB |
| F.FAHFGNLSGPSAIC(+57.02)GNPQVK.A | Y | 47.26 | 2100.0210 | 20 | 2.3 | 701.0159 | 3 | 35.51 | 1 | 20138 | 01122020\_RID\_2209\_BuCaKA02.raw | 7.0889E6 | 1 | 1 | 43 | 62 | Carbamidomethylation | C14:Carbamidomethylation:1000.00 | PEAKS DB |
| total 2 peptides |  |  |  |  |  |  |  |  |  |  |  |  |  |  |  |  |  |  |  |
| --- | --- | --- | --- | --- | --- | --- | --- | --- | --- | --- | --- | --- | --- | --- | --- | --- | --- | --- | --- |

JAG67762.1

back to list

  

| Protein Coverage
| Supporting Peptides
|

Protein Coverage:

Supporting Peptides:

| Peptide | Uniq | -10lgP | Mass | Length | ppm | m/z | z | RT | Fraction | Scan | Source File | Area BRom | #Feature | #Feature BRom | Start | End | PTM | AScore | Found By |
| --- | --- | --- | --- | --- | --- | --- | --- | --- | --- | --- | --- | --- | --- | --- | --- | --- | --- | --- | --- |
| R.FFAHFGNLSGPSAIC(+57.02)GNPQVK.A | Y | 67.83 | 2247.0894 | 21 | 0.8 | 750.0377 | 3 | 45.80 | 1 | 28096 | 01122020\_RID\_2209\_BuCaKA02.raw | 7.6599E7 | 1 | 1 | 42 | 62 | Carbamidomethylation | C15:Carbamidomethylation:1000.00 | PEAKS DB |
| F.FAHFGNLSGPSAIC(+57.02)GNPQVK.A | Y | 47.26 | 2100.0210 | 20 | 2.3 | 701.0159 | 3 | 35.51 | 1 | 20138 | 01122020\_RID\_2209\_BuCaKA02.raw | 7.0889E6 | 1 | 1 | 43 | 62 | Carbamidomethylation | C14:Carbamidomethylation:1000.00 | PEAKS DB |
| total 2 peptides |  |  |  |  |  |  |  |  |  |  |  |  |  |  |  |  |  |  |  |
| --- | --- | --- | --- | --- | --- | --- | --- | --- | --- | --- | --- | --- | --- | --- | --- | --- | --- | --- | --- |

JAG67761.1

back to list

  

| Protein Coverage
| Supporting Peptides
|

Protein Coverage:

Supporting Peptides:

| Peptide | Uniq | -10lgP | Mass | Length | ppm | m/z | z | RT | Fraction | Scan | Source File | Area BRom | #Feature | #Feature BRom | Start | End | PTM | AScore | Found By |
| --- | --- | --- | --- | --- | --- | --- | --- | --- | --- | --- | --- | --- | --- | --- | --- | --- | --- | --- | --- |
| R.FFAHFGNLSGPSAIC(+57.02)GNPQVK.A | Y | 67.83 | 2247.0894 | 21 | 0.8 | 750.0377 | 3 | 45.80 | 1 | 28096 | 01122020\_RID\_2209\_BuCaKA02.raw | 7.6599E7 | 1 | 1 | 42 | 62 | Carbamidomethylation | C15:Carbamidomethylation:1000.00 | PEAKS DB |
| F.FAHFGNLSGPSAIC(+57.02)GNPQVK.A | Y | 47.26 | 2100.0210 | 20 | 2.3 | 701.0159 | 3 | 35.51 | 1 | 20138 | 01122020\_RID\_2209\_BuCaKA02.raw | 7.0889E6 | 1 | 1 | 43 | 62 | Carbamidomethylation | C14:Carbamidomethylation:1000.00 | PEAKS DB |
| total 2 peptides |  |  |  |  |  |  |  |  |  |  |  |  |  |  |  |  |  |  |  |
| --- | --- | --- | --- | --- | --- | --- | --- | --- | --- | --- | --- | --- | --- | --- | --- | --- | --- | --- | --- |

ETE73401.1

back to list

  

| Protein Coverage
| Supporting Peptides
|

Protein Coverage:

Supporting Peptides:

| Peptide | Uniq | -10lgP | Mass | Length | ppm | m/z | z | RT | Fraction | Scan | Source File | Area BRom | #Feature | #Feature BRom | Start | End | PTM | AScore | Found By |
| --- | --- | --- | --- | --- | --- | --- | --- | --- | --- | --- | --- | --- | --- | --- | --- | --- | --- | --- | --- |
| K.NGNC(+57.02)EQFC(+57.02)VDSPTTLR.Q | Y | 72.67 | 1896.8094 | 16 | 1.7 | 949.4136 | 2 | 34.34 | 1 | 19139 | 01122020\_RID\_2209\_BuCaKA02.raw | 1.3762E7 | 1 | 1 | 126 | 141 | Carbamidomethylation | C4:Carbamidomethylation:1000.00;C8:Carbamidomethylation:1000.00 | PEAKS DB |
| R.LASDQVSC(+57.02)IAEVDYPC(+57.02)GKIPVLAK.R | Y | 55.92 | 2632.3240 | 24 | 0.8 | 878.4493 | 3 | 55.90 | 1 | 35985 | 01122020\_RID\_2209\_BuCaKA02.raw | 1.2309E6 | 1 | 1 | 151 | 174 | Carbamidomethylation | C8:Carbamidomethylation:1000.00;C16:Carbamidomethylation:1000.00 | PEAKS DB |
| total 2 peptides |  |  |  |  |  |  |  |  |  |  |  |  |  |  |  |  |  |  |  |
| --- | --- | --- | --- | --- | --- | --- | --- | --- | --- | --- | --- | --- | --- | --- | --- | --- | --- | --- | --- |

XP\_026580326.1

back to list

  

| Protein Coverage
| Supporting Peptides
|

Protein Coverage:

Supporting Peptides:

| Peptide | Uniq | -10lgP | Mass | Length | ppm | m/z | z | RT | Fraction | Scan | Source File | Area BRom | #Feature | #Feature BRom | Start | End | PTM | AScore | Found By |
| --- | --- | --- | --- | --- | --- | --- | --- | --- | --- | --- | --- | --- | --- | --- | --- | --- | --- | --- | --- |
| K.NGNC(+57.02)EQFC(+57.02)VDSPTTLR.Q | Y | 72.67 | 1896.8094 | 16 | 1.7 | 949.4136 | 2 | 34.34 | 1 | 19139 | 01122020\_RID\_2209\_BuCaKA02.raw | 1.3762E7 | 1 | 1 | 60 | 75 | Carbamidomethylation | C4:Carbamidomethylation:1000.00;C8:Carbamidomethylation:1000.00 | PEAKS DB |
| R.LASDQVSC(+57.02)IAEVDYPC(+57.02)GKIPVLAK.R | Y | 55.92 | 2632.3240 | 24 | 0.8 | 878.4493 | 3 | 55.90 | 1 | 35985 | 01122020\_RID\_2209\_BuCaKA02.raw | 1.2309E6 | 1 | 1 | 85 | 108 | Carbamidomethylation | C8:Carbamidomethylation:1000.00;C16:Carbamidomethylation:1000.00 | PEAKS DB |
| total 2 peptides |  |  |  |  |  |  |  |  |  |  |  |  |  |  |  |  |  |  |  |
| --- | --- | --- | --- | --- | --- | --- | --- | --- | --- | --- | --- | --- | --- | --- | --- | --- | --- | --- | --- |

JAS05177.1

back to list

  

| Protein Coverage
| Supporting Peptides
|

Protein Coverage:

Supporting Peptides:

| Peptide | Uniq | -10lgP | Mass | Length | ppm | m/z | z | RT | Fraction | Scan | Source File | Area BRom | #Feature | #Feature BRom | Start | End | PTM | AScore | Found By |
| --- | --- | --- | --- | --- | --- | --- | --- | --- | --- | --- | --- | --- | --- | --- | --- | --- | --- | --- | --- |
| K.NGNC(+57.02)EQFC(+57.02)VDSPTTLR.Q | Y | 72.67 | 1896.8094 | 16 | 1.7 | 949.4136 | 2 | 34.34 | 1 | 19139 | 01122020\_RID\_2209\_BuCaKA02.raw | 1.3762E7 | 1 | 1 | 135 | 150 | Carbamidomethylation | C4:Carbamidomethylation:1000.00;C8:Carbamidomethylation:1000.00 | PEAKS DB |
| R.LASDQVSC(+57.02)IAEVDYPC(+57.02)GKIPVLAK.R | Y | 55.92 | 2632.3240 | 24 | 0.8 | 878.4493 | 3 | 55.90 | 1 | 35985 | 01122020\_RID\_2209\_BuCaKA02.raw | 1.2309E6 | 1 | 1 | 160 | 183 | Carbamidomethylation | C8:Carbamidomethylation:1000.00;C16:Carbamidomethylation:1000.00 | PEAKS DB |
| total 2 peptides |  |  |  |  |  |  |  |  |  |  |  |  |  |  |  |  |  |  |  |
| --- | --- | --- | --- | --- | --- | --- | --- | --- | --- | --- | --- | --- | --- | --- | --- | --- | --- | --- | --- |

JAI09074.1

back to list

  

| Protein Coverage
| Supporting Peptides
|

Protein Coverage:

Supporting Peptides:

| Peptide | Uniq | -10lgP | Mass | Length | ppm | m/z | z | RT | Fraction | Scan | Source File | Area BRom | #Feature | #Feature BRom | Start | End | PTM | AScore | Found By |
| --- | --- | --- | --- | --- | --- | --- | --- | --- | --- | --- | --- | --- | --- | --- | --- | --- | --- | --- | --- |
| K.NGNC(+57.02)EQFC(+57.02)VDSPTTLR.Q | Y | 72.67 | 1896.8094 | 16 | 1.7 | 949.4136 | 2 | 34.34 | 1 | 19139 | 01122020\_RID\_2209\_BuCaKA02.raw | 1.3762E7 | 1 | 1 | 135 | 150 | Carbamidomethylation | C4:Carbamidomethylation:1000.00;C8:Carbamidomethylation:1000.00 | PEAKS DB |
| R.LASDQVSC(+57.02)IAEVDYPC(+57.02)GKIPVLAK.R | Y | 55.92 | 2632.3240 | 24 | 0.8 | 878.4493 | 3 | 55.90 | 1 | 35985 | 01122020\_RID\_2209\_BuCaKA02.raw | 1.2309E6 | 1 | 1 | 160 | 183 | Carbamidomethylation | C8:Carbamidomethylation:1000.00;C16:Carbamidomethylation:1000.00 | PEAKS DB |
| total 2 peptides |  |  |  |  |  |  |  |  |  |  |  |  |  |  |  |  |  |  |  |
| --- | --- | --- | --- | --- | --- | --- | --- | --- | --- | --- | --- | --- | --- | --- | --- | --- | --- | --- | --- |

JAS05063.1

back to list

  

| Protein Coverage
| Supporting Peptides
|

Protein Coverage:

Supporting Peptides:

| Peptide | Uniq | -10lgP | Mass | Length | ppm | m/z | z | RT | Fraction | Scan | Source File | Area BRom | #Feature | #Feature BRom | Start | End | PTM | AScore | Found By |
| --- | --- | --- | --- | --- | --- | --- | --- | --- | --- | --- | --- | --- | --- | --- | --- | --- | --- | --- | --- |
| K.NGNC(+57.02)EQFC(+57.02)VDSPTTLR.Q | Y | 72.67 | 1896.8094 | 16 | 1.7 | 949.4136 | 2 | 34.34 | 1 | 19139 | 01122020\_RID\_2209\_BuCaKA02.raw | 1.3762E7 | 1 | 1 | 135 | 150 | Carbamidomethylation | C4:Carbamidomethylation:1000.00;C8:Carbamidomethylation:1000.00 | PEAKS DB |
| R.LASDQVSC(+57.02)IAEVDYPC(+57.02)GKIPVLAK.R | Y | 55.92 | 2632.3240 | 24 | 0.8 | 878.4493 | 3 | 55.90 | 1 | 35985 | 01122020\_RID\_2209\_BuCaKA02.raw | 1.2309E6 | 1 | 1 | 160 | 183 | Carbamidomethylation | C8:Carbamidomethylation:1000.00;C16:Carbamidomethylation:1000.00 | PEAKS DB |
| total 2 peptides |  |  |  |  |  |  |  |  |  |  |  |  |  |  |  |  |  |  |  |
| --- | --- | --- | --- | --- | --- | --- | --- | --- | --- | --- | --- | --- | --- | --- | --- | --- | --- | --- | --- |

JAB54462.1

back to list

  

| Protein Coverage
| Supporting Peptides
|

Protein Coverage:

Supporting Peptides:

| Peptide | Uniq | -10lgP | Mass | Length | ppm | m/z | z | RT | Fraction | Scan | Source File | Area BRom | #Feature | #Feature BRom | Start | End | PTM | AScore | Found By |
| --- | --- | --- | --- | --- | --- | --- | --- | --- | --- | --- | --- | --- | --- | --- | --- | --- | --- | --- | --- |
| K.NGNC(+57.02)EQFC(+57.02)VDSPTTLR.Q | Y | 72.67 | 1896.8094 | 16 | 1.7 | 949.4136 | 2 | 34.34 | 1 | 19139 | 01122020\_RID\_2209\_BuCaKA02.raw | 1.3762E7 | 1 | 1 | 135 | 150 | Carbamidomethylation | C4:Carbamidomethylation:1000.00;C8:Carbamidomethylation:1000.00 | PEAKS DB |
| R.LASDQVSC(+57.02)IAEVDYPC(+57.02)GKIPVLAK.R | Y | 55.92 | 2632.3240 | 24 | 0.8 | 878.4493 | 3 | 55.90 | 1 | 35985 | 01122020\_RID\_2209\_BuCaKA02.raw | 1.2309E6 | 1 | 1 | 160 | 183 | Carbamidomethylation | C8:Carbamidomethylation:1000.00;C16:Carbamidomethylation:1000.00 | PEAKS DB |
| total 2 peptides |  |  |  |  |  |  |  |  |  |  |  |  |  |  |  |  |  |  |  |
| --- | --- | --- | --- | --- | --- | --- | --- | --- | --- | --- | --- | --- | --- | --- | --- | --- | --- | --- | --- |

XP\_026548111.1

back to list

  

| Protein Coverage
| Supporting Peptides
|

Protein Coverage:

Supporting Peptides:

| Peptide | Uniq | -10lgP | Mass | Length | ppm | m/z | z | RT | Fraction | Scan | Source File | Area BRom | #Feature | #Feature BRom | Start | End | PTM | AScore | Found By |
| --- | --- | --- | --- | --- | --- | --- | --- | --- | --- | --- | --- | --- | --- | --- | --- | --- | --- | --- | --- |
| K.NGNC(+57.02)EQFC(+57.02)VDSPTTLR.Q | Y | 72.67 | 1896.8094 | 16 | 1.7 | 949.4136 | 2 | 34.34 | 1 | 19139 | 01122020\_RID\_2209\_BuCaKA02.raw | 1.3762E7 | 1 | 1 | 106 | 121 | Carbamidomethylation | C4:Carbamidomethylation:1000.00;C8:Carbamidomethylation:1000.00 | PEAKS DB |
| R.LASDQVSC(+57.02)IAEVDYPC(+57.02)GKIPVLAK.R | Y | 55.92 | 2632.3240 | 24 | 0.8 | 878.4493 | 3 | 55.90 | 1 | 35985 | 01122020\_RID\_2209\_BuCaKA02.raw | 1.2309E6 | 1 | 1 | 131 | 154 | Carbamidomethylation | C8:Carbamidomethylation:1000.00;C16:Carbamidomethylation:1000.00 | PEAKS DB |
| total 2 peptides |  |  |  |  |  |  |  |  |  |  |  |  |  |  |  |  |  |  |  |
| --- | --- | --- | --- | --- | --- | --- | --- | --- | --- | --- | --- | --- | --- | --- | --- | --- | --- | --- | --- |

AAB20783.1

back to list

  

| Protein Coverage
| Supporting Peptides
|

Protein Coverage:

Supporting Peptides:

| Peptide | Uniq | -10lgP | Mass | Length | ppm | m/z | z | RT | Fraction | Scan | Source File | Area BRom | #Feature | #Feature BRom | Start | End | PTM | AScore | Found By |
| --- | --- | --- | --- | --- | --- | --- | --- | --- | --- | --- | --- | --- | --- | --- | --- | --- | --- | --- | --- |
| D.YGC(+57.02)YC(+57.02)GAGGSGTPVDELDR.C | N | 72.32 | 2032.8254 | 19 | 1.0 | 1017.4210 | 2 | 34.88 | 1 | 19734 | 01122020\_RID\_2209\_BuCaKA02.raw | 4.869E7 | 1 | 1 | 25 | 43 | Carbamidomethylation | C3:Carbamidomethylation:1000.00;C5:Carbamidomethylation:1000.00 | PEAKS DB |
| NLYQFGNMIQC(+57.02)ANHGRRPTLAYADYGC(+57.02)YC(+57.02)GAGGSGTPVDELDR.C | Y | 48.76 | 4824.1323 | 43 | 7.8 | 965.8413 | 5 | 106.07 | 1 | 76568 | 01122020\_RID\_2209\_BuCaKA02.raw | 3.4561E6 | 4 | 4 | 1 | 43 | Carbamidomethylation | C11:Carbamidomethylation:1000.00;C27:Carbamidomethylation:1000.00;C29:Carbamidomethylation:1000.00 | PEAKS DB |
| total 2 peptides |  |  |  |  |  |  |  |  |  |  |  |  |  |  |  |  |  |  |  |
| --- | --- | --- | --- | --- | --- | --- | --- | --- | --- | --- | --- | --- | --- | --- | --- | --- | --- | --- | --- |

P08873.1

back to list

  

| Protein Coverage
| Supporting Peptides
|

Protein Coverage:

Supporting Peptides:

| Peptide | Uniq | -10lgP | Mass | Length | ppm | m/z | z | RT | Fraction | Scan | Source File | Area BRom | #Feature | #Feature BRom | Start | End | PTM | AScore | Found By |
| --- | --- | --- | --- | --- | --- | --- | --- | --- | --- | --- | --- | --- | --- | --- | --- | --- | --- | --- | --- |
| D.YGC(+57.02)YC(+57.02)GAGGSGTPVDELDR.C | N | 72.32 | 2032.8254 | 19 | 1.0 | 1017.4210 | 2 | 34.88 | 1 | 19734 | 01122020\_RID\_2209\_BuCaKA02.raw | 4.869E7 | 1 | 1 | 52 | 70 | Carbamidomethylation | C3:Carbamidomethylation:1000.00;C5:Carbamidomethylation:1000.00 | PEAKS DB |
| L.NLYQFGNMIQC(+57.02)ANHGRRPTLAYADYGC(+57.02)YC(+57.02)GAGGSGTPVDELDR.C | Y | 48.76 | 4824.1323 | 43 | 7.8 | 965.8413 | 5 | 106.07 | 1 | 76568 | 01122020\_RID\_2209\_BuCaKA02.raw | 3.4561E6 | 4 | 4 | 28 | 70 | Carbamidomethylation | C11:Carbamidomethylation:1000.00;C27:Carbamidomethylation:1000.00;C29:Carbamidomethylation:1000.00 | PEAKS DB |
| total 2 peptides |  |  |  |  |  |  |  |  |  |  |  |  |  |  |  |  |  |  |  |
| --- | --- | --- | --- | --- | --- | --- | --- | --- | --- | --- | --- | --- | --- | --- | --- | --- | --- | --- | --- |

CAA31125.1

back to list

  

| Protein Coverage
| Supporting Peptides
|

Protein Coverage:

Supporting Peptides:

| Peptide | Uniq | -10lgP | Mass | Length | ppm | m/z | z | RT | Fraction | Scan | Source File | Area BRom | #Feature | #Feature BRom | Start | End | PTM | AScore | Found By |
| --- | --- | --- | --- | --- | --- | --- | --- | --- | --- | --- | --- | --- | --- | --- | --- | --- | --- | --- | --- |
| D.YGC(+57.02)YC(+57.02)GAGGSGTPVDELDR.C | N | 72.32 | 2032.8254 | 19 | 1.0 | 1017.4210 | 2 | 34.88 | 1 | 19734 | 01122020\_RID\_2209\_BuCaKA02.raw | 4.869E7 | 1 | 1 | 52 | 70 | Carbamidomethylation | C3:Carbamidomethylation:1000.00;C5:Carbamidomethylation:1000.00 | PEAKS DB |
| L.NLYQFGNMIQC(+57.02)ANHGRRPTLAYADYGC(+57.02)YC(+57.02)GAGGSGTPVDELDR.C | Y | 48.76 | 4824.1323 | 43 | 7.8 | 965.8413 | 5 | 106.07 | 1 | 76568 | 01122020\_RID\_2209\_BuCaKA02.raw | 3.4561E6 | 4 | 4 | 28 | 70 | Carbamidomethylation | C11:Carbamidomethylation:1000.00;C27:Carbamidomethylation:1000.00;C29:Carbamidomethylation:1000.00 | PEAKS DB |
| total 2 peptides |  |  |  |  |  |  |  |  |  |  |  |  |  |  |  |  |  |  |  |
| --- | --- | --- | --- | --- | --- | --- | --- | --- | --- | --- | --- | --- | --- | --- | --- | --- | --- | --- | --- |

AFJ49950.1

back to list

  

| Protein Coverage
| Supporting Peptides
|

Protein Coverage:

Supporting Peptides:

| Peptide | Uniq | -10lgP | Mass | Length | ppm | m/z | z | RT | Fraction | Scan | Source File | Area BRom | #Feature | #Feature BRom | Start | End | PTM | AScore | Found By |
| --- | --- | --- | --- | --- | --- | --- | --- | --- | --- | --- | --- | --- | --- | --- | --- | --- | --- | --- | --- |
| K.THINIVVIGHVDSGKSTTTGHLIYK.C | Y | 61.01 | 2689.4551 | 25 | 1.5 | 673.3721 | 4 | 24.93 | 1 | 12000 | 01122020\_RID\_2209\_BuCaKA02.raw | 6.5029E5 | 1 | 1 | 6 | 30 |  |  | PEAKS DB |
| K.DGSASGTTLLEALDSILPPTRPTDKPLRLPLQ.D | Y | 47.78 | 3371.8298 | 32 | 0.9 | 843.9655 | 4 | 92.02 | 1 | 65767 | 01122020\_RID\_2209\_BuCaKA02.raw | 1.7507E7 | 1 | 1 | 220 | 251 |  |  | PEAKS DB |
| total 2 peptides |  |  |  |  |  |  |  |  |  |  |  |  |  |  |  |  |  |  |  |
| --- | --- | --- | --- | --- | --- | --- | --- | --- | --- | --- | --- | --- | --- | --- | --- | --- | --- | --- | --- |

JAA97186.1

back to list

  

| Protein Coverage
| Supporting Peptides
|

Protein Coverage:

Supporting Peptides:

| Peptide | Uniq | -10lgP | Mass | Length | ppm | m/z | z | RT | Fraction | Scan | Source File | Area BRom | #Feature | #Feature BRom | Start | End | PTM | AScore | Found By |
| --- | --- | --- | --- | --- | --- | --- | --- | --- | --- | --- | --- | --- | --- | --- | --- | --- | --- | --- | --- |
| K.THINIVVIGHVDSGKSTTTGHLIYK.C | Y | 61.01 | 2689.4551 | 25 | 1.5 | 673.3721 | 4 | 24.93 | 1 | 12000 | 01122020\_RID\_2209\_BuCaKA02.raw | 6.5029E5 | 1 | 1 | 6 | 30 |  |  | PEAKS DB |
| K.DGSASGTTLLEALDSILPPTRPTDKPLRLPLQ.D | Y | 47.78 | 3371.8298 | 32 | 0.9 | 843.9655 | 4 | 92.02 | 1 | 65767 | 01122020\_RID\_2209\_BuCaKA02.raw | 1.7507E7 | 1 | 1 | 220 | 251 |  |  | PEAKS DB |
| total 2 peptides |  |  |  |  |  |  |  |  |  |  |  |  |  |  |  |  |  |  |  |
| --- | --- | --- | --- | --- | --- | --- | --- | --- | --- | --- | --- | --- | --- | --- | --- | --- | --- | --- | --- |

JAI13488.1

back to list

  

| Protein Coverage
| Supporting Peptides
|

Protein Coverage:

Supporting Peptides:

| Peptide | Uniq | -10lgP | Mass | Length | ppm | m/z | z | RT | Fraction | Scan | Source File | Area BRom | #Feature | #Feature BRom | Start | End | PTM | AScore | Found By |
| --- | --- | --- | --- | --- | --- | --- | --- | --- | --- | --- | --- | --- | --- | --- | --- | --- | --- | --- | --- |
| K.THINIVVIGHVDSGKSTTTGHLIYK.C | Y | 61.01 | 2689.4551 | 25 | 1.5 | 673.3721 | 4 | 24.93 | 1 | 12000 | 01122020\_RID\_2209\_BuCaKA02.raw | 6.5029E5 | 1 | 1 | 6 | 30 |  |  | PEAKS DB |
| K.DGSASGTTLLEALDSILPPTRPTDKPLRLPLQ.D | Y | 47.78 | 3371.8298 | 32 | 0.9 | 843.9655 | 4 | 92.02 | 1 | 65767 | 01122020\_RID\_2209\_BuCaKA02.raw | 1.7507E7 | 1 | 1 | 220 | 251 |  |  | PEAKS DB |
| total 2 peptides |  |  |  |  |  |  |  |  |  |  |  |  |  |  |  |  |  |  |  |
| --- | --- | --- | --- | --- | --- | --- | --- | --- | --- | --- | --- | --- | --- | --- | --- | --- | --- | --- | --- |

JAC95701.1

back to list

  

| Protein Coverage
| Supporting Peptides
|

Protein Coverage:

Supporting Peptides:

| Peptide | Uniq | -10lgP | Mass | Length | ppm | m/z | z | RT | Fraction | Scan | Source File | Area BRom | #Feature | #Feature BRom | Start | End | PTM | AScore | Found By |
| --- | --- | --- | --- | --- | --- | --- | --- | --- | --- | --- | --- | --- | --- | --- | --- | --- | --- | --- | --- |
| K.THINIVVIGHVDSGKSTTTGHLIYK.C | Y | 61.01 | 2689.4551 | 25 | 1.5 | 673.3721 | 4 | 24.93 | 1 | 12000 | 01122020\_RID\_2209\_BuCaKA02.raw | 6.5029E5 | 1 | 1 | 6 | 30 |  |  | PEAKS DB |
| K.DGSASGTTLLEALDSILPPTRPTDKPLRLPLQ.D | Y | 47.78 | 3371.8298 | 32 | 0.9 | 843.9655 | 4 | 92.02 | 1 | 65767 | 01122020\_RID\_2209\_BuCaKA02.raw | 1.7507E7 | 1 | 1 | 220 | 251 |  |  | PEAKS DB |
| total 2 peptides |  |  |  |  |  |  |  |  |  |  |  |  |  |  |  |  |  |  |  |
| --- | --- | --- | --- | --- | --- | --- | --- | --- | --- | --- | --- | --- | --- | --- | --- | --- | --- | --- | --- |

JAC95700.1

back to list

  

| Protein Coverage
| Supporting Peptides
|

Protein Coverage:

Supporting Peptides:

| Peptide | Uniq | -10lgP | Mass | Length | ppm | m/z | z | RT | Fraction | Scan | Source File | Area BRom | #Feature | #Feature BRom | Start | End | PTM | AScore | Found By |
| --- | --- | --- | --- | --- | --- | --- | --- | --- | --- | --- | --- | --- | --- | --- | --- | --- | --- | --- | --- |
| K.THINIVVIGHVDSGKSTTTGHLIYK.C | Y | 61.01 | 2689.4551 | 25 | 1.5 | 673.3721 | 4 | 24.93 | 1 | 12000 | 01122020\_RID\_2209\_BuCaKA02.raw | 6.5029E5 | 1 | 1 | 6 | 30 |  |  | PEAKS DB |
| K.DGSASGTTLLEALDSILPPTRPTDKPLRLPLQ.D | Y | 47.78 | 3371.8298 | 32 | 0.9 | 843.9655 | 4 | 92.02 | 1 | 65767 | 01122020\_RID\_2209\_BuCaKA02.raw | 1.7507E7 | 1 | 1 | 220 | 251 |  |  | PEAKS DB |
| total 2 peptides |  |  |  |  |  |  |  |  |  |  |  |  |  |  |  |  |  |  |  |
| --- | --- | --- | --- | --- | --- | --- | --- | --- | --- | --- | --- | --- | --- | --- | --- | --- | --- | --- | --- |

JAI10049.1

back to list

  

| Protein Coverage
| Supporting Peptides
|

Protein Coverage:

Supporting Peptides:

| Peptide | Uniq | -10lgP | Mass | Length | ppm | m/z | z | RT | Fraction | Scan | Source File | Area BRom | #Feature | #Feature BRom | Start | End | PTM | AScore | Found By |
| --- | --- | --- | --- | --- | --- | --- | --- | --- | --- | --- | --- | --- | --- | --- | --- | --- | --- | --- | --- |
| K.THINIVVIGHVDSGKSTTTGHLIYK.C | Y | 61.01 | 2689.4551 | 25 | 1.5 | 673.3721 | 4 | 24.93 | 1 | 12000 | 01122020\_RID\_2209\_BuCaKA02.raw | 6.5029E5 | 1 | 1 | 6 | 30 |  |  | PEAKS DB |
| K.DGSASGTTLLEALDSILPPTRPTDKPLRLPLQ.D | Y | 47.78 | 3371.8298 | 32 | 0.9 | 843.9655 | 4 | 92.02 | 1 | 65767 | 01122020\_RID\_2209\_BuCaKA02.raw | 1.7507E7 | 1 | 1 | 220 | 251 |  |  | PEAKS DB |
| total 2 peptides |  |  |  |  |  |  |  |  |  |  |  |  |  |  |  |  |  |  |  |
| --- | --- | --- | --- | --- | --- | --- | --- | --- | --- | --- | --- | --- | --- | --- | --- | --- | --- | --- | --- |

JAG46923.1

back to list

  

| Protein Coverage
| Supporting Peptides
|

Protein Coverage:

Supporting Peptides:

| Peptide | Uniq | -10lgP | Mass | Length | ppm | m/z | z | RT | Fraction | Scan | Source File | Area BRom | #Feature | #Feature BRom | Start | End | PTM | AScore | Found By |
| --- | --- | --- | --- | --- | --- | --- | --- | --- | --- | --- | --- | --- | --- | --- | --- | --- | --- | --- | --- |
| K.THINIVVIGHVDSGKSTTTGHLIYK.C | Y | 61.01 | 2689.4551 | 25 | 1.5 | 673.3721 | 4 | 24.93 | 1 | 12000 | 01122020\_RID\_2209\_BuCaKA02.raw | 6.5029E5 | 1 | 1 | 6 | 30 |  |  | PEAKS DB |
| K.DGSASGTTLLEALDSILPPTRPTDKPLRLPLQ.D | Y | 47.78 | 3371.8298 | 32 | 0.9 | 843.9655 | 4 | 92.02 | 1 | 65767 | 01122020\_RID\_2209\_BuCaKA02.raw | 1.7507E7 | 1 | 1 | 220 | 251 |  |  | PEAKS DB |
| total 2 peptides |  |  |  |  |  |  |  |  |  |  |  |  |  |  |  |  |  |  |  |
| --- | --- | --- | --- | --- | --- | --- | --- | --- | --- | --- | --- | --- | --- | --- | --- | --- | --- | --- | --- |

XP\_015672964.1

back to list

  

| Protein Coverage
| Supporting Peptides
|

Protein Coverage:

Supporting Peptides:

| Peptide | Uniq | -10lgP | Mass | Length | ppm | m/z | z | RT | Fraction | Scan | Source File | Area BRom | #Feature | #Feature BRom | Start | End | PTM | AScore | Found By |
| --- | --- | --- | --- | --- | --- | --- | --- | --- | --- | --- | --- | --- | --- | --- | --- | --- | --- | --- | --- |
| K.THINIVVIGHVDSGKSTTTGHLIYK.C | Y | 61.01 | 2689.4551 | 25 | 1.5 | 673.3721 | 4 | 24.93 | 1 | 12000 | 01122020\_RID\_2209\_BuCaKA02.raw | 6.5029E5 | 1 | 1 | 6 | 30 |  |  | PEAKS DB |
| K.DGSASGTTLLEALDSILPPTRPTDKPLRLPLQ.D | Y | 47.78 | 3371.8298 | 32 | 0.9 | 843.9655 | 4 | 92.02 | 1 | 65767 | 01122020\_RID\_2209\_BuCaKA02.raw | 1.7507E7 | 1 | 1 | 220 | 251 |  |  | PEAKS DB |
| total 2 peptides |  |  |  |  |  |  |  |  |  |  |  |  |  |  |  |  |  |  |  |
| --- | --- | --- | --- | --- | --- | --- | --- | --- | --- | --- | --- | --- | --- | --- | --- | --- | --- | --- | --- |

JAG44960.1

back to list

  

| Protein Coverage
| Supporting Peptides
|

Protein Coverage:

Supporting Peptides:

| Peptide | Uniq | -10lgP | Mass | Length | ppm | m/z | z | RT | Fraction | Scan | Source File | Area BRom | #Feature | #Feature BRom | Start | End | PTM | AScore | Found By |
| --- | --- | --- | --- | --- | --- | --- | --- | --- | --- | --- | --- | --- | --- | --- | --- | --- | --- | --- | --- |
| K.THINIVVIGHVDSGKSTTTGHLIYK.C | Y | 61.01 | 2689.4551 | 25 | 1.5 | 673.3721 | 4 | 24.93 | 1 | 12000 | 01122020\_RID\_2209\_BuCaKA02.raw | 6.5029E5 | 1 | 1 | 6 | 30 |  |  | PEAKS DB |
| K.DGSASGTTLLEALDSILPPTRPTDKPLRLPLQ.D | Y | 47.78 | 3371.8298 | 32 | 0.9 | 843.9655 | 4 | 92.02 | 1 | 65767 | 01122020\_RID\_2209\_BuCaKA02.raw | 1.7507E7 | 1 | 1 | 220 | 251 |  |  | PEAKS DB |
| total 2 peptides |  |  |  |  |  |  |  |  |  |  |  |  |  |  |  |  |  |  |  |
| --- | --- | --- | --- | --- | --- | --- | --- | --- | --- | --- | --- | --- | --- | --- | --- | --- | --- | --- | --- |

JAV50678.1

back to list

  

| Protein Coverage
| Supporting Peptides
|

Protein Coverage:

Supporting Peptides:

| Peptide | Uniq | -10lgP | Mass | Length | ppm | m/z | z | RT | Fraction | Scan | Source File | Area BRom | #Feature | #Feature BRom | Start | End | PTM | AScore | Found By |
| --- | --- | --- | --- | --- | --- | --- | --- | --- | --- | --- | --- | --- | --- | --- | --- | --- | --- | --- | --- |
| K.THINIVVIGHVDSGKSTTTGHLIYK.C | Y | 61.01 | 2689.4551 | 25 | 1.5 | 673.3721 | 4 | 24.93 | 1 | 12000 | 01122020\_RID\_2209\_BuCaKA02.raw | 6.5029E5 | 1 | 1 | 6 | 30 |  |  | PEAKS DB |
| K.DGSASGTTLLEALDSILPPTRPTDKPLRLPLQ.D | Y | 47.78 | 3371.8298 | 32 | 0.9 | 843.9655 | 4 | 92.02 | 1 | 65767 | 01122020\_RID\_2209\_BuCaKA02.raw | 1.7507E7 | 1 | 1 | 220 | 251 |  |  | PEAKS DB |
| total 2 peptides |  |  |  |  |  |  |  |  |  |  |  |  |  |  |  |  |  |  |  |
| --- | --- | --- | --- | --- | --- | --- | --- | --- | --- | --- | --- | --- | --- | --- | --- | --- | --- | --- | --- |

JAB54325.1

back to list

  

| Protein Coverage
| Supporting Peptides
|

Protein Coverage:

Supporting Peptides:

| Peptide | Uniq | -10lgP | Mass | Length | ppm | m/z | z | RT | Fraction | Scan | Source File | Area BRom | #Feature | #Feature BRom | Start | End | PTM | AScore | Found By |
| --- | --- | --- | --- | --- | --- | --- | --- | --- | --- | --- | --- | --- | --- | --- | --- | --- | --- | --- | --- |
| K.THINIVVIGHVDSGKSTTTGHLIYK.C | Y | 61.01 | 2689.4551 | 25 | 1.5 | 673.3721 | 4 | 24.93 | 1 | 12000 | 01122020\_RID\_2209\_BuCaKA02.raw | 6.5029E5 | 1 | 1 | 6 | 30 |  |  | PEAKS DB |
| K.DGSASGTTLLEALDSILPPTRPTDKPLRLPLQ.D | Y | 47.78 | 3371.8298 | 32 | 0.9 | 843.9655 | 4 | 92.02 | 1 | 65767 | 01122020\_RID\_2209\_BuCaKA02.raw | 1.7507E7 | 1 | 1 | 220 | 251 |  |  | PEAKS DB |
| total 2 peptides |  |  |  |  |  |  |  |  |  |  |  |  |  |  |  |  |  |  |  |
| --- | --- | --- | --- | --- | --- | --- | --- | --- | --- | --- | --- | --- | --- | --- | --- | --- | --- | --- | --- |

XP\_015682720.1

back to list

  

| Protein Coverage
| Supporting Peptides
|

Protein Coverage:

Supporting Peptides:

| Peptide | Uniq | -10lgP | Mass | Length | ppm | m/z | z | RT | Fraction | Scan | Source File | Area BRom | #Feature | #Feature BRom | Start | End | PTM | AScore | Found By |
| --- | --- | --- | --- | --- | --- | --- | --- | --- | --- | --- | --- | --- | --- | --- | --- | --- | --- | --- | --- |
| K.VLTSFGEAIKHLDNIK.E | Y | 76.91 | 1783.9832 | 16 | 0.4 | 595.6686 | 3 | 43.88 | 1 | 26464 | 01122020\_RID\_2209\_BuCaKA02.raw | 8.7682E6 | 1 | 1 | 68 | 83 |  |  | PEAKS DB |
| total 1 peptides |  |  |  |  |  |  |  |  |  |  |  |  |  |  |  |  |  |  |  |
| --- | --- | --- | --- | --- | --- | --- | --- | --- | --- | --- | --- | --- | --- | --- | --- | --- | --- | --- | --- |

XP\_026542227.1

back to list

  

| Protein Coverage
| Supporting Peptides
|

Protein Coverage:

Supporting Peptides:

| Peptide | Uniq | -10lgP | Mass | Length | ppm | m/z | z | RT | Fraction | Scan | Source File | Area BRom | #Feature | #Feature BRom | Start | End | PTM | AScore | Found By |
| --- | --- | --- | --- | --- | --- | --- | --- | --- | --- | --- | --- | --- | --- | --- | --- | --- | --- | --- | --- |
| K.AGSAVDDFINKLVPLLSR.G | Y | 75.36 | 1914.0574 | 18 | 2.0 | 639.0276 | 3 | 89.85 | 1 | 64053 | 01122020\_RID\_2209\_BuCaKA02.raw | 5.3043E5 | 1 | 1 | 64 | 81 |  |  | PEAKS DB |
| total 1 peptides |  |  |  |  |  |  |  |  |  |  |  |  |  |  |  |  |  |  |  |
| --- | --- | --- | --- | --- | --- | --- | --- | --- | --- | --- | --- | --- | --- | --- | --- | --- | --- | --- | --- |

JAI12109.1

back to list

  

| Protein Coverage
| Supporting Peptides
|

Protein Coverage:

Supporting Peptides:

| Peptide | Uniq | -10lgP | Mass | Length | ppm | m/z | z | RT | Fraction | Scan | Source File | Area BRom | #Feature | #Feature BRom | Start | End | PTM | AScore | Found By |
| --- | --- | --- | --- | --- | --- | --- | --- | --- | --- | --- | --- | --- | --- | --- | --- | --- | --- | --- | --- |
| K.AGSAVDDFINKLVPLLSR.G | Y | 75.36 | 1914.0574 | 18 | 2.0 | 639.0276 | 3 | 89.85 | 1 | 64053 | 01122020\_RID\_2209\_BuCaKA02.raw | 5.3043E5 | 1 | 1 | 77 | 94 |  |  | PEAKS DB |
| total 1 peptides |  |  |  |  |  |  |  |  |  |  |  |  |  |  |  |  |  |  |  |
| --- | --- | --- | --- | --- | --- | --- | --- | --- | --- | --- | --- | --- | --- | --- | --- | --- | --- | --- | --- |

JAG46036.1

back to list

  

| Protein Coverage
| Supporting Peptides
|

Protein Coverage:

Supporting Peptides:

| Peptide | Uniq | -10lgP | Mass | Length | ppm | m/z | z | RT | Fraction | Scan | Source File | Area BRom | #Feature | #Feature BRom | Start | End | PTM | AScore | Found By |
| --- | --- | --- | --- | --- | --- | --- | --- | --- | --- | --- | --- | --- | --- | --- | --- | --- | --- | --- | --- |
| K.AGSAVDDFINKLVPLLSR.G | Y | 75.36 | 1914.0574 | 18 | 2.0 | 639.0276 | 3 | 89.85 | 1 | 64053 | 01122020\_RID\_2209\_BuCaKA02.raw | 5.3043E5 | 1 | 1 | 77 | 94 |  |  | PEAKS DB |
| total 1 peptides |  |  |  |  |  |  |  |  |  |  |  |  |  |  |  |  |  |  |  |
| --- | --- | --- | --- | --- | --- | --- | --- | --- | --- | --- | --- | --- | --- | --- | --- | --- | --- | --- | --- |

XP\_032087395.1

back to list

  

| Protein Coverage
| Supporting Peptides
|

Protein Coverage:

Supporting Peptides:

| Peptide | Uniq | -10lgP | Mass | Length | ppm | m/z | z | RT | Fraction | Scan | Source File | Area BRom | #Feature | #Feature BRom | Start | End | PTM | AScore | Found By |
| --- | --- | --- | --- | --- | --- | --- | --- | --- | --- | --- | --- | --- | --- | --- | --- | --- | --- | --- | --- |
| K.AGSAVDDFINKLVPLLSR.G | Y | 75.36 | 1914.0574 | 18 | 2.0 | 639.0276 | 3 | 89.85 | 1 | 64053 | 01122020\_RID\_2209\_BuCaKA02.raw | 5.3043E5 | 1 | 1 | 77 | 94 |  |  | PEAKS DB |
| total 1 peptides |  |  |  |  |  |  |  |  |  |  |  |  |  |  |  |  |  |  |  |
| --- | --- | --- | --- | --- | --- | --- | --- | --- | --- | --- | --- | --- | --- | --- | --- | --- | --- | --- | --- |

XP\_015676923.1

back to list

  

| Protein Coverage
| Supporting Peptides
|

Protein Coverage:

Supporting Peptides:

| Peptide | Uniq | -10lgP | Mass | Length | ppm | m/z | z | RT | Fraction | Scan | Source File | Area BRom | #Feature | #Feature BRom | Start | End | PTM | AScore | Found By |
| --- | --- | --- | --- | --- | --- | --- | --- | --- | --- | --- | --- | --- | --- | --- | --- | --- | --- | --- | --- |
| K.AGSAVDDFINKLVPLLSR.G | Y | 75.36 | 1914.0574 | 18 | 2.0 | 639.0276 | 3 | 89.85 | 1 | 64053 | 01122020\_RID\_2209\_BuCaKA02.raw | 5.3043E5 | 1 | 1 | 77 | 94 |  |  | PEAKS DB |
| total 1 peptides |  |  |  |  |  |  |  |  |  |  |  |  |  |  |  |  |  |  |  |
| --- | --- | --- | --- | --- | --- | --- | --- | --- | --- | --- | --- | --- | --- | --- | --- | --- | --- | --- | --- |

AFJ50914.1

back to list

  

| Protein Coverage
| Supporting Peptides
|

Protein Coverage:

Supporting Peptides:

| Peptide | Uniq | -10lgP | Mass | Length | ppm | m/z | z | RT | Fraction | Scan | Source File | Area BRom | #Feature | #Feature BRom | Start | End | PTM | AScore | Found By |
| --- | --- | --- | --- | --- | --- | --- | --- | --- | --- | --- | --- | --- | --- | --- | --- | --- | --- | --- | --- |
| K.AGSAVDDFINKLVPLLSR.G | Y | 75.36 | 1914.0574 | 18 | 2.0 | 639.0276 | 3 | 89.85 | 1 | 64053 | 01122020\_RID\_2209\_BuCaKA02.raw | 5.3043E5 | 1 | 1 | 77 | 94 |  |  | PEAKS DB |
| total 1 peptides |  |  |  |  |  |  |  |  |  |  |  |  |  |  |  |  |  |  |  |
| --- | --- | --- | --- | --- | --- | --- | --- | --- | --- | --- | --- | --- | --- | --- | --- | --- | --- | --- | --- |

JAB53691.1

back to list

  

| Protein Coverage
| Supporting Peptides
|

Protein Coverage:

Supporting Peptides:

| Peptide | Uniq | -10lgP | Mass | Length | ppm | m/z | z | RT | Fraction | Scan | Source File | Area BRom | #Feature | #Feature BRom | Start | End | PTM | AScore | Found By |
| --- | --- | --- | --- | --- | --- | --- | --- | --- | --- | --- | --- | --- | --- | --- | --- | --- | --- | --- | --- |
| K.AGSAVDDFINKLVPLLSR.G | Y | 75.36 | 1914.0574 | 18 | 2.0 | 639.0276 | 3 | 89.85 | 1 | 64053 | 01122020\_RID\_2209\_BuCaKA02.raw | 5.3043E5 | 1 | 1 | 77 | 94 |  |  | PEAKS DB |
| total 1 peptides |  |  |  |  |  |  |  |  |  |  |  |  |  |  |  |  |  |  |  |
| --- | --- | --- | --- | --- | --- | --- | --- | --- | --- | --- | --- | --- | --- | --- | --- | --- | --- | --- | --- |

JAV51398.1

back to list

  

| Protein Coverage
| Supporting Peptides
|

Protein Coverage:

Supporting Peptides:

| Peptide | Uniq | -10lgP | Mass | Length | ppm | m/z | z | RT | Fraction | Scan | Source File | Area BRom | #Feature | #Feature BRom | Start | End | PTM | AScore | Found By |
| --- | --- | --- | --- | --- | --- | --- | --- | --- | --- | --- | --- | --- | --- | --- | --- | --- | --- | --- | --- |
| K.AGSAVDDFINKLVPLLSR.G | Y | 75.36 | 1914.0574 | 18 | 2.0 | 639.0276 | 3 | 89.85 | 1 | 64053 | 01122020\_RID\_2209\_BuCaKA02.raw | 5.3043E5 | 1 | 1 | 77 | 94 |  |  | PEAKS DB |
| total 1 peptides |  |  |  |  |  |  |  |  |  |  |  |  |  |  |  |  |  |  |  |
| --- | --- | --- | --- | --- | --- | --- | --- | --- | --- | --- | --- | --- | --- | --- | --- | --- | --- | --- | --- |

XP\_034294750.1

back to list

  

| Protein Coverage
| Supporting Peptides
|

Protein Coverage:

Supporting Peptides:

| Peptide | Uniq | -10lgP | Mass | Length | ppm | m/z | z | RT | Fraction | Scan | Source File | Area BRom | #Feature | #Feature BRom | Start | End | PTM | AScore | Found By |
| --- | --- | --- | --- | --- | --- | --- | --- | --- | --- | --- | --- | --- | --- | --- | --- | --- | --- | --- | --- |
| K.AGSAVDDFINKLVPLLSR.G | Y | 75.36 | 1914.0574 | 18 | 2.0 | 639.0276 | 3 | 89.85 | 1 | 64053 | 01122020\_RID\_2209\_BuCaKA02.raw | 5.3043E5 | 1 | 1 | 77 | 94 |  |  | PEAKS DB |
| total 1 peptides |  |  |  |  |  |  |  |  |  |  |  |  |  |  |  |  |  |  |  |
| --- | --- | --- | --- | --- | --- | --- | --- | --- | --- | --- | --- | --- | --- | --- | --- | --- | --- | --- | --- |

LAA63155.1

back to list

  

| Protein Coverage
| Supporting Peptides
|

Protein Coverage:

Supporting Peptides:

| Peptide | Uniq | -10lgP | Mass | Length | ppm | m/z | z | RT | Fraction | Scan | Source File | Area BRom | #Feature | #Feature BRom | Start | End | PTM | AScore | Found By |
| --- | --- | --- | --- | --- | --- | --- | --- | --- | --- | --- | --- | --- | --- | --- | --- | --- | --- | --- | --- |
| K.AGSAVDDFINKLVPLLSR.G | Y | 75.36 | 1914.0574 | 18 | 2.0 | 639.0276 | 3 | 89.85 | 1 | 64053 | 01122020\_RID\_2209\_BuCaKA02.raw | 5.3043E5 | 1 | 1 | 119 | 136 |  |  | PEAKS DB |
| total 1 peptides |  |  |  |  |  |  |  |  |  |  |  |  |  |  |  |  |  |  |  |
| --- | --- | --- | --- | --- | --- | --- | --- | --- | --- | --- | --- | --- | --- | --- | --- | --- | --- | --- | --- |

JAG68880.1

back to list

  

| Protein Coverage
| Supporting Peptides
|

Protein Coverage:

Supporting Peptides:

| Peptide | Uniq | -10lgP | Mass | Length | ppm | m/z | z | RT | Fraction | Scan | Source File | Area BRom | #Feature | #Feature BRom | Start | End | PTM | AScore | Found By |
| --- | --- | --- | --- | --- | --- | --- | --- | --- | --- | --- | --- | --- | --- | --- | --- | --- | --- | --- | --- |
| K.AGSAVDDFINKLVPLLSR.G | Y | 75.36 | 1914.0574 | 18 | 2.0 | 639.0276 | 3 | 89.85 | 1 | 64053 | 01122020\_RID\_2209\_BuCaKA02.raw | 5.3043E5 | 1 | 1 | 77 | 94 |  |  | PEAKS DB |
| total 1 peptides |  |  |  |  |  |  |  |  |  |  |  |  |  |  |  |  |  |  |  |
| --- | --- | --- | --- | --- | --- | --- | --- | --- | --- | --- | --- | --- | --- | --- | --- | --- | --- | --- | --- |

LAB46151.1

back to list

  

| Protein Coverage
| Supporting Peptides
|

Protein Coverage:

Supporting Peptides:

| Peptide | Uniq | -10lgP | Mass | Length | ppm | m/z | z | RT | Fraction | Scan | Source File | Area BRom | #Feature | #Feature BRom | Start | End | PTM | AScore | Found By |
| --- | --- | --- | --- | --- | --- | --- | --- | --- | --- | --- | --- | --- | --- | --- | --- | --- | --- | --- | --- |
| K.AGSAVDDFINKLVPLLSR.G | Y | 75.36 | 1914.0574 | 18 | 2.0 | 639.0276 | 3 | 89.85 | 1 | 64053 | 01122020\_RID\_2209\_BuCaKA02.raw | 5.3043E5 | 1 | 1 | 112 | 129 |  |  | PEAKS DB |
| total 1 peptides |  |  |  |  |  |  |  |  |  |  |  |  |  |  |  |  |  |  |  |
| --- | --- | --- | --- | --- | --- | --- | --- | --- | --- | --- | --- | --- | --- | --- | --- | --- | --- | --- | --- |

Q9DF52.1

back to list

  

| Protein Coverage
| Supporting Peptides
|

Protein Coverage:

Supporting Peptides:

| Peptide | Uniq | -10lgP | Mass | Length | ppm | m/z | z | RT | Fraction | Scan | Source File | Area BRom | #Feature | #Feature BRom | Start | End | PTM | AScore | Found By |
| --- | --- | --- | --- | --- | --- | --- | --- | --- | --- | --- | --- | --- | --- | --- | --- | --- | --- | --- | --- |
| K.NM(+15.99)IQC(+57.02)AGTRPWTAYVNYGC(+57.02)YC(+57.02)GKGGSGTPVDELDR.C | Y | 49.01 | 3968.7339 | 35 | -0.4 | 993.1903 | 4 | 52.51 | 1 | 32922 | 01122020\_RID\_2209\_BuCaKA02.raw | 1.7513E9 | 1 | 1 | 34 | 68 | Oxidation (M); Carbamidomethylation | M2:Oxidation (M):1000.00;C5:Carbamidomethylation:1000.00;C19:Carbamidomethylation:1000.00;C21:Carbamidomethylation:1000.00 | PEAKS DB |
| R.FLC(+57.02)DC(+57.02)DRTAAIC(+57.02)F.A | N | 44.26 | 1647.6843 | 13 | 0.5 | 824.8499 | 2 | 55.77 | 1 | 35810 | 01122020\_RID\_2209\_BuCaKA02.raw | 2.2718E7 | 1 | 1 | 114 | 126 | Carbamidomethylation | C3:Carbamidomethylation:1000.00;C5:Carbamidomethylation:1000.00;C12:Carbamidomethylation:1000.00 | PEAKS DB |
| total 2 peptides |  |  |  |  |  |  |  |  |  |  |  |  |  |  |  |  |  |  |  |
| --- | --- | --- | --- | --- | --- | --- | --- | --- | --- | --- | --- | --- | --- | --- | --- | --- | --- | --- | --- |

AAG13412.1

back to list

  

| Protein Coverage
| Supporting Peptides
|

Protein Coverage:

Supporting Peptides:

| Peptide | Uniq | -10lgP | Mass | Length | ppm | m/z | z | RT | Fraction | Scan | Source File | Area BRom | #Feature | #Feature BRom | Start | End | PTM | AScore | Found By |
| --- | --- | --- | --- | --- | --- | --- | --- | --- | --- | --- | --- | --- | --- | --- | --- | --- | --- | --- | --- |
| K.NM(+15.99)IQC(+57.02)AGTRPWTAYVNYGC(+57.02)YC(+57.02)GKGGSGTPVDELDR.C | Y | 49.01 | 3968.7339 | 35 | -0.4 | 993.1903 | 4 | 52.51 | 1 | 32922 | 01122020\_RID\_2209\_BuCaKA02.raw | 1.7513E9 | 1 | 1 | 34 | 68 | Oxidation (M); Carbamidomethylation | M2:Oxidation (M):1000.00;C5:Carbamidomethylation:1000.00;C19:Carbamidomethylation:1000.00;C21:Carbamidomethylation:1000.00 | PEAKS DB |
| R.FLC(+57.02)DC(+57.02)DRTAAIC(+57.02)F.A | N | 44.26 | 1647.6843 | 13 | 0.5 | 824.8499 | 2 | 55.77 | 1 | 35810 | 01122020\_RID\_2209\_BuCaKA02.raw | 2.2718E7 | 1 | 1 | 114 | 126 | Carbamidomethylation | C3:Carbamidomethylation:1000.00;C5:Carbamidomethylation:1000.00;C12:Carbamidomethylation:1000.00 | PEAKS DB |
| total 2 peptides |  |  |  |  |  |  |  |  |  |  |  |  |  |  |  |  |  |  |  |
| --- | --- | --- | --- | --- | --- | --- | --- | --- | --- | --- | --- | --- | --- | --- | --- | --- | --- | --- | --- |

P15816.1

back to list

  

| Protein Coverage
| Supporting Peptides
|

Protein Coverage:

Supporting Peptides:

| Peptide | Uniq | -10lgP | Mass | Length | ppm | m/z | z | RT | Fraction | Scan | Source File | Area BRom | #Feature | #Feature BRom | Start | End | PTM | AScore | Found By |
| --- | --- | --- | --- | --- | --- | --- | --- | --- | --- | --- | --- | --- | --- | --- | --- | --- | --- | --- | --- |
| R.GPVIEQGC(+57.02)AATC(+57.02)PEFR.S | Y | 70.87 | 1790.8080 | 16 | 0.7 | 896.4119 | 2 | 30.48 | 1 | 16118 | 01122020\_RID\_2209\_BuCaKA02.raw | 1.7586E6 | 1 | 1 | 56 | 71 | Carbamidomethylation | C8:Carbamidomethylation:1000.00;C12:Carbamidomethylation:1000.00 | PEAKS DB |
| total 1 peptides |  |  |  |  |  |  |  |  |  |  |  |  |  |  |  |  |  |  |  |
| --- | --- | --- | --- | --- | --- | --- | --- | --- | --- | --- | --- | --- | --- | --- | --- | --- | --- | --- | --- |

CAA35774.1

back to list

  

| Protein Coverage
| Supporting Peptides
|

Protein Coverage:

Supporting Peptides:

| Peptide | Uniq | -10lgP | Mass | Length | ppm | m/z | z | RT | Fraction | Scan | Source File | Area BRom | #Feature | #Feature BRom | Start | End | PTM | AScore | Found By |
| --- | --- | --- | --- | --- | --- | --- | --- | --- | --- | --- | --- | --- | --- | --- | --- | --- | --- | --- | --- |
| R.GPVIEQGC(+57.02)AATC(+57.02)PEFR.S | Y | 70.87 | 1790.8080 | 16 | 0.7 | 896.4119 | 2 | 30.48 | 1 | 16118 | 01122020\_RID\_2209\_BuCaKA02.raw | 1.7586E6 | 1 | 1 | 56 | 71 | Carbamidomethylation | C8:Carbamidomethylation:1000.00;C12:Carbamidomethylation:1000.00 | PEAKS DB |
| total 1 peptides |  |  |  |  |  |  |  |  |  |  |  |  |  |  |  |  |  |  |  |
| --- | --- | --- | --- | --- | --- | --- | --- | --- | --- | --- | --- | --- | --- | --- | --- | --- | --- | --- | --- |

O12962.1

back to list

  

| Protein Coverage
| Supporting Peptides
|

Protein Coverage:

Supporting Peptides:

| Peptide | Uniq | -10lgP | Mass | Length | ppm | m/z | z | RT | Fraction | Scan | Source File | Area BRom | #Feature | #Feature BRom | Start | End | PTM | AScore | Found By |
| --- | --- | --- | --- | --- | --- | --- | --- | --- | --- | --- | --- | --- | --- | --- | --- | --- | --- | --- | --- |
| R.GPVIEQGC(+57.02)AATC(+57.02)PEFR.S | Y | 70.87 | 1790.8080 | 16 | 0.7 | 896.4119 | 2 | 30.48 | 1 | 16118 | 01122020\_RID\_2209\_BuCaKA02.raw | 1.7586E6 | 1 | 1 | 56 | 71 | Carbamidomethylation | C8:Carbamidomethylation:1000.00;C12:Carbamidomethylation:1000.00 | PEAKS DB |
| total 1 peptides |  |  |  |  |  |  |  |  |  |  |  |  |  |  |  |  |  |  |  |
| --- | --- | --- | --- | --- | --- | --- | --- | --- | --- | --- | --- | --- | --- | --- | --- | --- | --- | --- | --- |

CAA72940.1

back to list

  

| Protein Coverage
| Supporting Peptides
|

Protein Coverage:

Supporting Peptides:

| Peptide | Uniq | -10lgP | Mass | Length | ppm | m/z | z | RT | Fraction | Scan | Source File | Area BRom | #Feature | #Feature BRom | Start | End | PTM | AScore | Found By |
| --- | --- | --- | --- | --- | --- | --- | --- | --- | --- | --- | --- | --- | --- | --- | --- | --- | --- | --- | --- |
| R.GPVIEQGC(+57.02)AATC(+57.02)PEFR.S | Y | 70.87 | 1790.8080 | 16 | 0.7 | 896.4119 | 2 | 30.48 | 1 | 16118 | 01122020\_RID\_2209\_BuCaKA02.raw | 1.7586E6 | 1 | 1 | 56 | 71 | Carbamidomethylation | C8:Carbamidomethylation:1000.00;C12:Carbamidomethylation:1000.00 | PEAKS DB |
| total 1 peptides |  |  |  |  |  |  |  |  |  |  |  |  |  |  |  |  |  |  |  |
| --- | --- | --- | --- | --- | --- | --- | --- | --- | --- | --- | --- | --- | --- | --- | --- | --- | --- | --- | --- |

ACE73577.1

back to list

  

| Protein Coverage
| Supporting Peptides
|

Protein Coverage:

Supporting Peptides:

| Peptide | Uniq | -10lgP | Mass | Length | ppm | m/z | z | RT | Fraction | Scan | Source File | Area BRom | #Feature | #Feature BRom | Start | End | PTM | AScore | Found By |
| --- | --- | --- | --- | --- | --- | --- | --- | --- | --- | --- | --- | --- | --- | --- | --- | --- | --- | --- | --- |
| R.NMLQMEWNSNAAQNAKR.W | Y | 69.57 | 2004.9258 | 17 | 0.1 | 669.3159 | 3 | 29.96 | 1 | 15691 | 01122020\_RID\_2209\_BuCaKA02.raw | 1.3601E7 | 1 | 1 | 54 | 70 |  |  | PEAKS DB |
| total 1 peptides |  |  |  |  |  |  |  |  |  |  |  |  |  |  |  |  |  |  |  |
| --- | --- | --- | --- | --- | --- | --- | --- | --- | --- | --- | --- | --- | --- | --- | --- | --- | --- | --- | --- |

ACE73578.1

back to list

  

| Protein Coverage
| Supporting Peptides
|

Protein Coverage:

Supporting Peptides:

| Peptide | Uniq | -10lgP | Mass | Length | ppm | m/z | z | RT | Fraction | Scan | Source File | Area BRom | #Feature | #Feature BRom | Start | End | PTM | AScore | Found By |
| --- | --- | --- | --- | --- | --- | --- | --- | --- | --- | --- | --- | --- | --- | --- | --- | --- | --- | --- | --- |
| R.NMLQMEWNSNAAQNAKR.W | Y | 69.57 | 2004.9258 | 17 | 0.1 | 669.3159 | 3 | 29.96 | 1 | 15691 | 01122020\_RID\_2209\_BuCaKA02.raw | 1.3601E7 | 1 | 1 | 54 | 70 |  |  | PEAKS DB |
| total 1 peptides |  |  |  |  |  |  |  |  |  |  |  |  |  |  |  |  |  |  |  |
| --- | --- | --- | --- | --- | --- | --- | --- | --- | --- | --- | --- | --- | --- | --- | --- | --- | --- | --- | --- |

T\_89

back to list

  

| Protein Coverage
| Supporting Peptides
|

Protein Coverage:

Supporting Peptides:

| Peptide | Uniq | -10lgP | Mass | Length | ppm | m/z | z | RT | Fraction | Scan | Source File | Area BRom | #Feature | #Feature BRom | Start | End | PTM | AScore | Found By |
| --- | --- | --- | --- | --- | --- | --- | --- | --- | --- | --- | --- | --- | --- | --- | --- | --- | --- | --- | --- |
| R.NMLQMEWNSNAAQNAKR.W | Y | 69.57 | 2004.9258 | 17 | 0.1 | 669.3159 | 3 | 29.96 | 1 | 15691 | 01122020\_RID\_2209\_BuCaKA02.raw | 1.3601E7 | 1 | 1 | 54 | 70 |  |  | PEAKS DB |
| total 1 peptides |  |  |  |  |  |  |  |  |  |  |  |  |  |  |  |  |  |  |  |
| --- | --- | --- | --- | --- | --- | --- | --- | --- | --- | --- | --- | --- | --- | --- | --- | --- | --- | --- | --- |

T\_93

back to list

  

| Protein Coverage
| Supporting Peptides
|

Protein Coverage:

Supporting Peptides:

| Peptide | Uniq | -10lgP | Mass | Length | ppm | m/z | z | RT | Fraction | Scan | Source File | Area BRom | #Feature | #Feature BRom | Start | End | PTM | AScore | Found By |
| --- | --- | --- | --- | --- | --- | --- | --- | --- | --- | --- | --- | --- | --- | --- | --- | --- | --- | --- | --- |
| R.NMLQMEWNSNAAQNAKR.W | Y | 69.57 | 2004.9258 | 17 | 0.1 | 669.3159 | 3 | 29.96 | 1 | 15691 | 01122020\_RID\_2209\_BuCaKA02.raw | 1.3601E7 | 1 | 1 | 54 | 70 |  |  | PEAKS DB |
| total 1 peptides |  |  |  |  |  |  |  |  |  |  |  |  |  |  |  |  |  |  |  |
| --- | --- | --- | --- | --- | --- | --- | --- | --- | --- | --- | --- | --- | --- | --- | --- | --- | --- | --- | --- |

T\_92

back to list

  

| Protein Coverage
| Supporting Peptides
|

Protein Coverage:

Supporting Peptides:

| Peptide | Uniq | -10lgP | Mass | Length | ppm | m/z | z | RT | Fraction | Scan | Source File | Area BRom | #Feature | #Feature BRom | Start | End | PTM | AScore | Found By |
| --- | --- | --- | --- | --- | --- | --- | --- | --- | --- | --- | --- | --- | --- | --- | --- | --- | --- | --- | --- |
| R.NMLQMEWNSNAAQNAKR.W | Y | 69.57 | 2004.9258 | 17 | 0.1 | 669.3159 | 3 | 29.96 | 1 | 15691 | 01122020\_RID\_2209\_BuCaKA02.raw | 1.3601E7 | 1 | 1 | 54 | 70 |  |  | PEAKS DB |
| total 1 peptides |  |  |  |  |  |  |  |  |  |  |  |  |  |  |  |  |  |  |  |
| --- | --- | --- | --- | --- | --- | --- | --- | --- | --- | --- | --- | --- | --- | --- | --- | --- | --- | --- | --- |

T\_95

back to list

  

| Protein Coverage
| Supporting Peptides
|

Protein Coverage:

Supporting Peptides:

| Peptide | Uniq | -10lgP | Mass | Length | ppm | m/z | z | RT | Fraction | Scan | Source File | Area BRom | #Feature | #Feature BRom | Start | End | PTM | AScore | Found By |
| --- | --- | --- | --- | --- | --- | --- | --- | --- | --- | --- | --- | --- | --- | --- | --- | --- | --- | --- | --- |
| R.NMLQMEWNSNAAQNAKR.W | Y | 69.57 | 2004.9258 | 17 | 0.1 | 669.3159 | 3 | 29.96 | 1 | 15691 | 01122020\_RID\_2209\_BuCaKA02.raw | 1.3601E7 | 1 | 1 | 54 | 70 |  |  | PEAKS DB |
| total 1 peptides |  |  |  |  |  |  |  |  |  |  |  |  |  |  |  |  |  |  |  |
| --- | --- | --- | --- | --- | --- | --- | --- | --- | --- | --- | --- | --- | --- | --- | --- | --- | --- | --- | --- |

T\_91

back to list

  

| Protein Coverage
| Supporting Peptides
|

Protein Coverage:

Supporting Peptides:

| Peptide | Uniq | -10lgP | Mass | Length | ppm | m/z | z | RT | Fraction | Scan | Source File | Area BRom | #Feature | #Feature BRom | Start | End | PTM | AScore | Found By |
| --- | --- | --- | --- | --- | --- | --- | --- | --- | --- | --- | --- | --- | --- | --- | --- | --- | --- | --- | --- |
| R.NMLQMEWNSNAAQNAKR.W | Y | 69.57 | 2004.9258 | 17 | 0.1 | 669.3159 | 3 | 29.96 | 1 | 15691 | 01122020\_RID\_2209\_BuCaKA02.raw | 1.3601E7 | 1 | 1 | 65 | 81 |  |  | PEAKS DB |
| total 1 peptides |  |  |  |  |  |  |  |  |  |  |  |  |  |  |  |  |  |  |  |
| --- | --- | --- | --- | --- | --- | --- | --- | --- | --- | --- | --- | --- | --- | --- | --- | --- | --- | --- | --- |

T\_94

back to list

  

| Protein Coverage
| Supporting Peptides
|

Protein Coverage:

Supporting Peptides:

| Peptide | Uniq | -10lgP | Mass | Length | ppm | m/z | z | RT | Fraction | Scan | Source File | Area BRom | #Feature | #Feature BRom | Start | End | PTM | AScore | Found By |
| --- | --- | --- | --- | --- | --- | --- | --- | --- | --- | --- | --- | --- | --- | --- | --- | --- | --- | --- | --- |
| R.NMLQMEWNSNAAQNAKR.W | Y | 69.57 | 2004.9258 | 17 | 0.1 | 669.3159 | 3 | 29.96 | 1 | 15691 | 01122020\_RID\_2209\_BuCaKA02.raw | 1.3601E7 | 1 | 1 | 96 | 112 |  |  | PEAKS DB |
| total 1 peptides |  |  |  |  |  |  |  |  |  |  |  |  |  |  |  |  |  |  |  |
| --- | --- | --- | --- | --- | --- | --- | --- | --- | --- | --- | --- | --- | --- | --- | --- | --- | --- | --- | --- |

P81993.1

back to list

  

| Protein Coverage
| Supporting Peptides
|

Protein Coverage:

Supporting Peptides:

| Peptide | Uniq | -10lgP | Mass | Length | ppm | m/z | z | RT | Fraction | Scan | Source File | Area BRom | #Feature | #Feature BRom | Start | End | PTM | AScore | Found By |
| --- | --- | --- | --- | --- | --- | --- | --- | --- | --- | --- | --- | --- | --- | --- | --- | --- | --- | --- | --- |
| R.NMLQMEWNSNAAQNAKR.F | Y | 69.57 | 2004.9258 | 17 | 0.1 | 669.3159 | 3 | 29.96 | 1 | 15691 | 01122020\_RID\_2209\_BuCaKA02.raw | 1.3601E7 | 1 | 1 | 29 | 45 |  |  | PEAKS DB |
| total 1 peptides |  |  |  |  |  |  |  |  |  |  |  |  |  |  |  |  |  |  |  |
| --- | --- | --- | --- | --- | --- | --- | --- | --- | --- | --- | --- | --- | --- | --- | --- | --- | --- | --- | --- |

T\_96

back to list

  

| Protein Coverage
| Supporting Peptides
|

Protein Coverage:

Supporting Peptides:

| Peptide | Uniq | -10lgP | Mass | Length | ppm | m/z | z | RT | Fraction | Scan | Source File | Area BRom | #Feature | #Feature BRom | Start | End | PTM | AScore | Found By |
| --- | --- | --- | --- | --- | --- | --- | --- | --- | --- | --- | --- | --- | --- | --- | --- | --- | --- | --- | --- |
| R.NMLQMEWNSNAAQNAKR.W | Y | 69.57 | 2004.9258 | 17 | 0.1 | 669.3159 | 3 | 29.96 | 1 | 15691 | 01122020\_RID\_2209\_BuCaKA02.raw | 1.3601E7 | 1 | 1 | 65 | 81 |  |  | PEAKS DB |
| total 1 peptides |  |  |  |  |  |  |  |  |  |  |  |  |  |  |  |  |  |  |  |
| --- | --- | --- | --- | --- | --- | --- | --- | --- | --- | --- | --- | --- | --- | --- | --- | --- | --- | --- | --- |

ACN93671.1

back to list

  

| Protein Coverage
| Supporting Peptides
|

Protein Coverage:

Supporting Peptides:

| Peptide | Uniq | -10lgP | Mass | Length | ppm | m/z | z | RT | Fraction | Scan | Source File | Area BRom | #Feature | #Feature BRom | Start | End | PTM | AScore | Found By |
| --- | --- | --- | --- | --- | --- | --- | --- | --- | --- | --- | --- | --- | --- | --- | --- | --- | --- | --- | --- |
| R.NMLQMEWNSNAAQNAKR.W | Y | 69.57 | 2004.9258 | 17 | 0.1 | 669.3159 | 3 | 29.96 | 1 | 15691 | 01122020\_RID\_2209\_BuCaKA02.raw | 1.3601E7 | 1 | 1 | 53 | 69 |  |  | PEAKS DB |
| total 1 peptides |  |  |  |  |  |  |  |  |  |  |  |  |  |  |  |  |  |  |  |
| --- | --- | --- | --- | --- | --- | --- | --- | --- | --- | --- | --- | --- | --- | --- | --- | --- | --- | --- | --- |

ACH73168.1

back to list

  

| Protein Coverage
| Supporting Peptides
|

Protein Coverage:

Supporting Peptides:

| Peptide | Uniq | -10lgP | Mass | Length | ppm | m/z | z | RT | Fraction | Scan | Source File | Area BRom | #Feature | #Feature BRom | Start | End | PTM | AScore | Found By |
| --- | --- | --- | --- | --- | --- | --- | --- | --- | --- | --- | --- | --- | --- | --- | --- | --- | --- | --- | --- |
| R.NMLQMEWNSNAAQNAKR.W | Y | 69.57 | 2004.9258 | 17 | 0.1 | 669.3159 | 3 | 29.96 | 1 | 15691 | 01122020\_RID\_2209\_BuCaKA02.raw | 1.3601E7 | 1 | 1 | 54 | 70 |  |  | PEAKS DB |
| total 1 peptides |  |  |  |  |  |  |  |  |  |  |  |  |  |  |  |  |  |  |  |
| --- | --- | --- | --- | --- | --- | --- | --- | --- | --- | --- | --- | --- | --- | --- | --- | --- | --- | --- | --- |

AAP20603.1

back to list

  

| Protein Coverage
| Supporting Peptides
|

Protein Coverage:

Supporting Peptides:

| Peptide | Uniq | -10lgP | Mass | Length | ppm | m/z | z | RT | Fraction | Scan | Source File | Area BRom | #Feature | #Feature BRom | Start | End | PTM | AScore | Found By |
| --- | --- | --- | --- | --- | --- | --- | --- | --- | --- | --- | --- | --- | --- | --- | --- | --- | --- | --- | --- |
| R.NMLQMEWNSNAAQNAKR.W | Y | 69.57 | 2004.9258 | 17 | 0.1 | 669.3159 | 3 | 29.96 | 1 | 15691 | 01122020\_RID\_2209\_BuCaKA02.raw | 1.3601E7 | 1 | 1 | 54 | 70 |  |  | PEAKS DB |
| total 1 peptides |  |  |  |  |  |  |  |  |  |  |  |  |  |  |  |  |  |  |  |
| --- | --- | --- | --- | --- | --- | --- | --- | --- | --- | --- | --- | --- | --- | --- | --- | --- | --- | --- | --- |

Q7ZZN8.1

back to list

  

| Protein Coverage
| Supporting Peptides
|

Protein Coverage:

Supporting Peptides:

| Peptide | Uniq | -10lgP | Mass | Length | ppm | m/z | z | RT | Fraction | Scan | Source File | Area BRom | #Feature | #Feature BRom | Start | End | PTM | AScore | Found By |
| --- | --- | --- | --- | --- | --- | --- | --- | --- | --- | --- | --- | --- | --- | --- | --- | --- | --- | --- | --- |
| R.NMLQMEWNSNAAQNAKR.W | Y | 69.57 | 2004.9258 | 17 | 0.1 | 669.3159 | 3 | 29.96 | 1 | 15691 | 01122020\_RID\_2209\_BuCaKA02.raw | 1.3601E7 | 1 | 1 | 54 | 70 |  |  | PEAKS DB |
| total 1 peptides |  |  |  |  |  |  |  |  |  |  |  |  |  |  |  |  |  |  |  |
| --- | --- | --- | --- | --- | --- | --- | --- | --- | --- | --- | --- | --- | --- | --- | --- | --- | --- | --- | --- |

P84808.2

back to list

  

| Protein Coverage
| Supporting Peptides
|

Protein Coverage:

Supporting Peptides:

| Peptide | Uniq | -10lgP | Mass | Length | ppm | m/z | z | RT | Fraction | Scan | Source File | Area BRom | #Feature | #Feature BRom | Start | End | PTM | AScore | Found By |
| --- | --- | --- | --- | --- | --- | --- | --- | --- | --- | --- | --- | --- | --- | --- | --- | --- | --- | --- | --- |
| R.NMLQMEWNSNAAQNAKR.W | Y | 69.57 | 2004.9258 | 17 | 0.1 | 669.3159 | 3 | 29.96 | 1 | 15691 | 01122020\_RID\_2209\_BuCaKA02.raw | 1.3601E7 | 1 | 1 | 54 | 70 |  |  | PEAKS DB |
| total 1 peptides |  |  |  |  |  |  |  |  |  |  |  |  |  |  |  |  |  |  |  |
| --- | --- | --- | --- | --- | --- | --- | --- | --- | --- | --- | --- | --- | --- | --- | --- | --- | --- | --- | --- |

AHZ08822.1

back to list

  

| Protein Coverage
| Supporting Peptides
|

Protein Coverage:

Supporting Peptides:

| Peptide | Uniq | -10lgP | Mass | Length | ppm | m/z | z | RT | Fraction | Scan | Source File | Area BRom | #Feature | #Feature BRom | Start | End | PTM | AScore | Found By |
| --- | --- | --- | --- | --- | --- | --- | --- | --- | --- | --- | --- | --- | --- | --- | --- | --- | --- | --- | --- |
| R.NMLQMEWNSNAAQNAKR.W | Y | 69.57 | 2004.9258 | 17 | 0.1 | 669.3159 | 3 | 29.96 | 1 | 15691 | 01122020\_RID\_2209\_BuCaKA02.raw | 1.3601E7 | 1 | 1 | 54 | 70 |  |  | PEAKS DB |
| total 1 peptides |  |  |  |  |  |  |  |  |  |  |  |  |  |  |  |  |  |  |  |
| --- | --- | --- | --- | --- | --- | --- | --- | --- | --- | --- | --- | --- | --- | --- | --- | --- | --- | --- | --- |

XP\_032069593.1

back to list

  

| Protein Coverage
| Supporting Peptides
|

Protein Coverage:

Supporting Peptides:

| Peptide | Uniq | -10lgP | Mass | Length | ppm | m/z | z | RT | Fraction | Scan | Source File | Area BRom | #Feature | #Feature BRom | Start | End | PTM | AScore | Found By |
| --- | --- | --- | --- | --- | --- | --- | --- | --- | --- | --- | --- | --- | --- | --- | --- | --- | --- | --- | --- |
| S.NMLQMEWNSNAAQNAKR.W | Y | 69.57 | 2004.9258 | 17 | 0.1 | 669.3159 | 3 | 29.96 | 1 | 15691 | 01122020\_RID\_2209\_BuCaKA02.raw | 1.3601E7 | 1 | 1 | 54 | 70 |  |  | PEAKS DB |
| total 1 peptides |  |  |  |  |  |  |  |  |  |  |  |  |  |  |  |  |  |  |  |
| --- | --- | --- | --- | --- | --- | --- | --- | --- | --- | --- | --- | --- | --- | --- | --- | --- | --- | --- | --- |

JAC94992.1

back to list

  

| Protein Coverage
| Supporting Peptides
|

Protein Coverage:

Supporting Peptides:

| Peptide | Uniq | -10lgP | Mass | Length | ppm | m/z | z | RT | Fraction | Scan | Source File | Area BRom | #Feature | #Feature BRom | Start | End | PTM | AScore | Found By |
| --- | --- | --- | --- | --- | --- | --- | --- | --- | --- | --- | --- | --- | --- | --- | --- | --- | --- | --- | --- |
| R.NMLQMEWNSNAAQNAKR.W | Y | 69.57 | 2004.9258 | 17 | 0.1 | 669.3159 | 3 | 29.96 | 1 | 15691 | 01122020\_RID\_2209\_BuCaKA02.raw | 1.3601E7 | 1 | 1 | 54 | 70 |  |  | PEAKS DB |
| total 1 peptides |  |  |  |  |  |  |  |  |  |  |  |  |  |  |  |  |  |  |  |
| --- | --- | --- | --- | --- | --- | --- | --- | --- | --- | --- | --- | --- | --- | --- | --- | --- | --- | --- | --- |

ETE62137.1

back to list

  

| Protein Coverage
| Supporting Peptides
|

Protein Coverage:

Supporting Peptides:

| Peptide | Uniq | -10lgP | Mass | Length | ppm | m/z | z | RT | Fraction | Scan | Source File | Area BRom | #Feature | #Feature BRom | Start | End | PTM | AScore | Found By |
| --- | --- | --- | --- | --- | --- | --- | --- | --- | --- | --- | --- | --- | --- | --- | --- | --- | --- | --- | --- |
| R.NMLQMEWNSNAAQNAKR.W | Y | 69.57 | 2004.9258 | 17 | 0.1 | 669.3159 | 3 | 29.96 | 1 | 15691 | 01122020\_RID\_2209\_BuCaKA02.raw | 1.3601E7 | 1 | 1 | 60 | 76 |  |  | PEAKS DB |
| total 1 peptides |  |  |  |  |  |  |  |  |  |  |  |  |  |  |  |  |  |  |  |
| --- | --- | --- | --- | --- | --- | --- | --- | --- | --- | --- | --- | --- | --- | --- | --- | --- | --- | --- | --- |

XP\_034292109.1

back to list

  

| Protein Coverage
| Supporting Peptides
|

Protein Coverage:

Supporting Peptides:

| Peptide | Uniq | -10lgP | Mass | Length | ppm | m/z | z | RT | Fraction | Scan | Source File | Area BRom | #Feature | #Feature BRom | Start | End | PTM | AScore | Found By |
| --- | --- | --- | --- | --- | --- | --- | --- | --- | --- | --- | --- | --- | --- | --- | --- | --- | --- | --- | --- |
| R.RKEC(+57.02)PLGSHAC(+57.02)GPC(+57.02)LPQFVEDR.E | Y | 69.56 | 2612.2046 | 22 | 1.5 | 654.0594 | 4 | 24.85 | 1 | 11957 | 01122020\_RID\_2209\_BuCaKA02.raw | 1.5314E7 | 1 | 1 | 54 | 75 | Carbamidomethylation | C4:Carbamidomethylation:1000.00;C11:Carbamidomethylation:1000.00;C14:Carbamidomethylation:1000.00 | PEAKS DB |
| total 1 peptides |  |  |  |  |  |  |  |  |  |  |  |  |  |  |  |  |  |  |  |
| --- | --- | --- | --- | --- | --- | --- | --- | --- | --- | --- | --- | --- | --- | --- | --- | --- | --- | --- | --- |

Q9PRL9.1

back to list

  

| Protein Coverage
| Supporting Peptides
|

Protein Coverage:

Supporting Peptides:

| Peptide | Uniq | -10lgP | Mass | Length | ppm | m/z | z | RT | Fraction | Scan | Source File | Area BRom | #Feature | #Feature BRom | Start | End | PTM | AScore | Found By |
| --- | --- | --- | --- | --- | --- | --- | --- | --- | --- | --- | --- | --- | --- | --- | --- | --- | --- | --- | --- |
| K.NAELYGSETLTR.M | Y | 68.46 | 1352.6572 | 12 | 1.3 | 677.3368 | 2 | 25.94 | 1 | 12572 | 01122020\_RID\_2209\_BuCaKA02.raw | 8.8287E6 | 2 | 2 | 20 | 31 |  |  | PEAKS DB |
| total 1 peptides |  |  |  |  |  |  |  |  |  |  |  |  |  |  |  |  |  |  |  |
| --- | --- | --- | --- | --- | --- | --- | --- | --- | --- | --- | --- | --- | --- | --- | --- | --- | --- | --- | --- |

AAB34003.1

back to list

  

| Protein Coverage
| Supporting Peptides
|

Protein Coverage:

Supporting Peptides:

| Peptide | Uniq | -10lgP | Mass | Length | ppm | m/z | z | RT | Fraction | Scan | Source File | Area BRom | #Feature | #Feature BRom | Start | End | PTM | AScore | Found By |
| --- | --- | --- | --- | --- | --- | --- | --- | --- | --- | --- | --- | --- | --- | --- | --- | --- | --- | --- | --- |
| K.NAELYGSETLTR.M | Y | 68.46 | 1352.6572 | 12 | 1.3 | 677.3368 | 2 | 25.94 | 1 | 12572 | 01122020\_RID\_2209\_BuCaKA02.raw | 8.8287E6 | 2 | 2 | 20 | 31 |  |  | PEAKS DB |
| total 1 peptides |  |  |  |  |  |  |  |  |  |  |  |  |  |  |  |  |  |  |  |
| --- | --- | --- | --- | --- | --- | --- | --- | --- | --- | --- | --- | --- | --- | --- | --- | --- | --- | --- | --- |

XP\_034287104.1

back to list

  

| Protein Coverage
| Supporting Peptides
|

Protein Coverage:

Supporting Peptides:

| Peptide | Uniq | -10lgP | Mass | Length | ppm | m/z | z | RT | Fraction | Scan | Source File | Area BRom | #Feature | #Feature BRom | Start | End | PTM | AScore | Found By |
| --- | --- | --- | --- | --- | --- | --- | --- | --- | --- | --- | --- | --- | --- | --- | --- | --- | --- | --- | --- |
| E.GLGTDETTVIEILTGR.S | Y | 68.09 | 1673.8835 | 16 | 2.0 | 837.9507 | 2 | 84.83 | 1 | 59810 | 01122020\_RID\_2209\_BuCaKA02.raw | 8.9964E5 | 1 | 1 | 3 | 18 |  |  | PEAKS DB |
| total 1 peptides |  |  |  |  |  |  |  |  |  |  |  |  |  |  |  |  |  |  |  |
| --- | --- | --- | --- | --- | --- | --- | --- | --- | --- | --- | --- | --- | --- | --- | --- | --- | --- | --- | --- |

LAB37887.1

back to list

  

| Protein Coverage
| Supporting Peptides
|

Protein Coverage:

Supporting Peptides:

| Peptide | Uniq | -10lgP | Mass | Length | ppm | m/z | z | RT | Fraction | Scan | Source File | Area BRom | #Feature | #Feature BRom | Start | End | PTM | AScore | Found By |
| --- | --- | --- | --- | --- | --- | --- | --- | --- | --- | --- | --- | --- | --- | --- | --- | --- | --- | --- | --- |
| E.GLGTDETTVIEILTGR.S | Y | 68.09 | 1673.8835 | 16 | 2.0 | 837.9507 | 2 | 84.83 | 1 | 59810 | 01122020\_RID\_2209\_BuCaKA02.raw | 8.9964E5 | 1 | 1 | 33 | 48 |  |  | PEAKS DB |
| total 1 peptides |  |  |  |  |  |  |  |  |  |  |  |  |  |  |  |  |  |  |  |
| --- | --- | --- | --- | --- | --- | --- | --- | --- | --- | --- | --- | --- | --- | --- | --- | --- | --- | --- | --- |

JAG68721.1

back to list

  

| Protein Coverage
| Supporting Peptides
|

Protein Coverage:

Supporting Peptides:

| Peptide | Uniq | -10lgP | Mass | Length | ppm | m/z | z | RT | Fraction | Scan | Source File | Area BRom | #Feature | #Feature BRom | Start | End | PTM | AScore | Found By |
| --- | --- | --- | --- | --- | --- | --- | --- | --- | --- | --- | --- | --- | --- | --- | --- | --- | --- | --- | --- |
| E.GLGTDETTVIEILTGR.S | Y | 68.09 | 1673.8835 | 16 | 2.0 | 837.9507 | 2 | 84.83 | 1 | 59810 | 01122020\_RID\_2209\_BuCaKA02.raw | 8.9964E5 | 1 | 1 | 33 | 48 |  |  | PEAKS DB |
| total 1 peptides |  |  |  |  |  |  |  |  |  |  |  |  |  |  |  |  |  |  |  |
| --- | --- | --- | --- | --- | --- | --- | --- | --- | --- | --- | --- | --- | --- | --- | --- | --- | --- | --- | --- |

XP\_026532239.1

back to list

  

| Protein Coverage
| Supporting Peptides
|

Protein Coverage:

Supporting Peptides:

| Peptide | Uniq | -10lgP | Mass | Length | ppm | m/z | z | RT | Fraction | Scan | Source File | Area BRom | #Feature | #Feature BRom | Start | End | PTM | AScore | Found By |
| --- | --- | --- | --- | --- | --- | --- | --- | --- | --- | --- | --- | --- | --- | --- | --- | --- | --- | --- | --- |
| E.GLGTDETTVIEILTGR.S | Y | 68.09 | 1673.8835 | 16 | 2.0 | 837.9507 | 2 | 84.83 | 1 | 59810 | 01122020\_RID\_2209\_BuCaKA02.raw | 8.9964E5 | 1 | 1 | 33 | 48 |  |  | PEAKS DB |
| total 1 peptides |  |  |  |  |  |  |  |  |  |  |  |  |  |  |  |  |  |  |  |
| --- | --- | --- | --- | --- | --- | --- | --- | --- | --- | --- | --- | --- | --- | --- | --- | --- | --- | --- | --- |

XP\_013926160.1

back to list

  

| Protein Coverage
| Supporting Peptides
|

Protein Coverage:

Supporting Peptides:

| Peptide | Uniq | -10lgP | Mass | Length | ppm | m/z | z | RT | Fraction | Scan | Source File | Area BRom | #Feature | #Feature BRom | Start | End | PTM | AScore | Found By |
| --- | --- | --- | --- | --- | --- | --- | --- | --- | --- | --- | --- | --- | --- | --- | --- | --- | --- | --- | --- |
| E.GLGTDETTVIEILTGR.S | Y | 68.09 | 1673.8835 | 16 | 2.0 | 837.9507 | 2 | 84.83 | 1 | 59810 | 01122020\_RID\_2209\_BuCaKA02.raw | 8.9964E5 | 1 | 1 | 33 | 48 |  |  | PEAKS DB |
| total 1 peptides |  |  |  |  |  |  |  |  |  |  |  |  |  |  |  |  |  |  |  |
| --- | --- | --- | --- | --- | --- | --- | --- | --- | --- | --- | --- | --- | --- | --- | --- | --- | --- | --- | --- |

XP\_026574715.1

back to list

  

| Protein Coverage
| Supporting Peptides
|

Protein Coverage:

Supporting Peptides:

| Peptide | Uniq | -10lgP | Mass | Length | ppm | m/z | z | RT | Fraction | Scan | Source File | Area BRom | #Feature | #Feature BRom | Start | End | PTM | AScore | Found By |
| --- | --- | --- | --- | --- | --- | --- | --- | --- | --- | --- | --- | --- | --- | --- | --- | --- | --- | --- | --- |
| E.GLGTDETTVIEILTGR.S | Y | 68.09 | 1673.8835 | 16 | 2.0 | 837.9507 | 2 | 84.83 | 1 | 59810 | 01122020\_RID\_2209\_BuCaKA02.raw | 8.9964E5 | 1 | 1 | 33 | 48 |  |  | PEAKS DB |
| total 1 peptides |  |  |  |  |  |  |  |  |  |  |  |  |  |  |  |  |  |  |  |
| --- | --- | --- | --- | --- | --- | --- | --- | --- | --- | --- | --- | --- | --- | --- | --- | --- | --- | --- | --- |

XP\_032080318.1

back to list

  

| Protein Coverage
| Supporting Peptides
|

Protein Coverage:

Supporting Peptides:

| Peptide | Uniq | -10lgP | Mass | Length | ppm | m/z | z | RT | Fraction | Scan | Source File | Area BRom | #Feature | #Feature BRom | Start | End | PTM | AScore | Found By |
| --- | --- | --- | --- | --- | --- | --- | --- | --- | --- | --- | --- | --- | --- | --- | --- | --- | --- | --- | --- |
| E.GLGTDETTVIEILTGR.S | Y | 68.09 | 1673.8835 | 16 | 2.0 | 837.9507 | 2 | 84.83 | 1 | 59810 | 01122020\_RID\_2209\_BuCaKA02.raw | 8.9964E5 | 1 | 1 | 33 | 48 |  |  | PEAKS DB |
| total 1 peptides |  |  |  |  |  |  |  |  |  |  |  |  |  |  |  |  |  |  |  |
| --- | --- | --- | --- | --- | --- | --- | --- | --- | --- | --- | --- | --- | --- | --- | --- | --- | --- | --- | --- |

JAC96259.1

back to list

  

| Protein Coverage
| Supporting Peptides
|

Protein Coverage:

Supporting Peptides:

| Peptide | Uniq | -10lgP | Mass | Length | ppm | m/z | z | RT | Fraction | Scan | Source File | Area BRom | #Feature | #Feature BRom | Start | End | PTM | AScore | Found By |
| --- | --- | --- | --- | --- | --- | --- | --- | --- | --- | --- | --- | --- | --- | --- | --- | --- | --- | --- | --- |
| E.GLGTDETTVIEILTGR.S | Y | 68.09 | 1673.8835 | 16 | 2.0 | 837.9507 | 2 | 84.83 | 1 | 59810 | 01122020\_RID\_2209\_BuCaKA02.raw | 8.9964E5 | 1 | 1 | 33 | 48 |  |  | PEAKS DB |
| total 1 peptides |  |  |  |  |  |  |  |  |  |  |  |  |  |  |  |  |  |  |  |
| --- | --- | --- | --- | --- | --- | --- | --- | --- | --- | --- | --- | --- | --- | --- | --- | --- | --- | --- | --- |

AAL30054.1

back to list

  

| Protein Coverage
| Supporting Peptides
|

Protein Coverage:

Supporting Peptides:

| Peptide | Uniq | -10lgP | Mass | Length | ppm | m/z | z | RT | Fraction | Scan | Source File | Area BRom | #Feature | #Feature BRom | Start | End | PTM | AScore | Found By |
| --- | --- | --- | --- | --- | --- | --- | --- | --- | --- | --- | --- | --- | --- | --- | --- | --- | --- | --- | --- |
| T.RTC(+57.02)LISPSSTPQTC(+57.02)PQGQDIC(+57.02)FLK.A | Y | 67.87 | 2793.3247 | 24 | -0.6 | 932.1150 | 3 | 42.73 | 1 | 25636 | 01122020\_RID\_2209\_BuCaKA02.raw | 1.3114E8 | 1 | 1 | 22 | 45 | Carbamidomethylation | C3:Carbamidomethylation:1000.00;C14:Carbamidomethylation:1000.00;C21:Carbamidomethylation:1000.00 | PEAKS DB |
| total 1 peptides |  |  |  |  |  |  |  |  |  |  |  |  |  |  |  |  |  |  |  |
| --- | --- | --- | --- | --- | --- | --- | --- | --- | --- | --- | --- | --- | --- | --- | --- | --- | --- | --- | --- |

Q8AY56.1

back to list

  

| Protein Coverage
| Supporting Peptides
|

Protein Coverage:

Supporting Peptides:

| Peptide | Uniq | -10lgP | Mass | Length | ppm | m/z | z | RT | Fraction | Scan | Source File | Area BRom | #Feature | #Feature BRom | Start | End | PTM | AScore | Found By |
| --- | --- | --- | --- | --- | --- | --- | --- | --- | --- | --- | --- | --- | --- | --- | --- | --- | --- | --- | --- |
| T.RTC(+57.02)LISPSSTPQTC(+57.02)PQGQDIC(+57.02)FLK.A | Y | 67.87 | 2793.3247 | 24 | -0.6 | 932.1150 | 3 | 42.73 | 1 | 25636 | 01122020\_RID\_2209\_BuCaKA02.raw | 1.3114E8 | 1 | 1 | 22 | 45 | Carbamidomethylation | C3:Carbamidomethylation:1000.00;C14:Carbamidomethylation:1000.00;C21:Carbamidomethylation:1000.00 | PEAKS DB |
| total 1 peptides |  |  |  |  |  |  |  |  |  |  |  |  |  |  |  |  |  |  |  |
| --- | --- | --- | --- | --- | --- | --- | --- | --- | --- | --- | --- | --- | --- | --- | --- | --- | --- | --- | --- |

LAA33342.1

back to list

  

| Protein Coverage
| Supporting Peptides
|

Protein Coverage:

Supporting Peptides:

| Peptide | Uniq | -10lgP | Mass | Length | ppm | m/z | z | RT | Fraction | Scan | Source File | Area BRom | #Feature | #Feature BRom | Start | End | PTM | AScore | Found By |
| --- | --- | --- | --- | --- | --- | --- | --- | --- | --- | --- | --- | --- | --- | --- | --- | --- | --- | --- | --- |
| R.LGAINTISC(+57.02)KGQVGRYVIVTVPGR.T | Y | 67.24 | 2557.4163 | 24 | -0.8 | 640.3608 | 4 | 39.99 | 1 | 23763 | 01122020\_RID\_2209\_BuCaKA02.raw | 0 | 0 | 0 | 127 | 150 | Carbamidomethylation | C9:Carbamidomethylation:1000.00 | PEAKS DB |
| total 1 peptides |  |  |  |  |  |  |  |  |  |  |  |  |  |  |  |  |  |  |  |
| --- | --- | --- | --- | --- | --- | --- | --- | --- | --- | --- | --- | --- | --- | --- | --- | --- | --- | --- | --- |

LAB47895.1

back to list

  

| Protein Coverage
| Supporting Peptides
|

Protein Coverage:

Supporting Peptides:

| Peptide | Uniq | -10lgP | Mass | Length | ppm | m/z | z | RT | Fraction | Scan | Source File | Area BRom | #Feature | #Feature BRom | Start | End | PTM | AScore | Found By |
| --- | --- | --- | --- | --- | --- | --- | --- | --- | --- | --- | --- | --- | --- | --- | --- | --- | --- | --- | --- |
| R.LGAINTISC(+57.02)KGQVGRYVIVTVPGR.T | Y | 67.24 | 2557.4163 | 24 | -0.8 | 640.3608 | 4 | 39.99 | 1 | 23763 | 01122020\_RID\_2209\_BuCaKA02.raw | 0 | 0 | 0 | 191 | 214 | Carbamidomethylation | C9:Carbamidomethylation:1000.00 | PEAKS DB |
| total 1 peptides |  |  |  |  |  |  |  |  |  |  |  |  |  |  |  |  |  |  |  |
| --- | --- | --- | --- | --- | --- | --- | --- | --- | --- | --- | --- | --- | --- | --- | --- | --- | --- | --- | --- |

T\_74

back to list

  

| Protein Coverage
| Supporting Peptides
|

Protein Coverage:

Supporting Peptides:

| Peptide | Uniq | -10lgP | Mass | Length | ppm | m/z | z | RT | Fraction | Scan | Source File | Area BRom | #Feature | #Feature BRom | Start | End | PTM | AScore | Found By |
| --- | --- | --- | --- | --- | --- | --- | --- | --- | --- | --- | --- | --- | --- | --- | --- | --- | --- | --- | --- |
| R.GAVIEQGC(+57.02)VATC(+57.02)PQFR.S | Y | 66.13 | 1791.8396 | 16 | 2.6 | 896.9294 | 2 | 33.04 | 1 | 18066 | 01122020\_RID\_2209\_BuCaKA02.raw | 1.8424E6 | 1 | 1 | 60 | 75 | Carbamidomethylation | C8:Carbamidomethylation:1000.00;C12:Carbamidomethylation:1000.00 | PEAKS DB |
| total 1 peptides |  |  |  |  |  |  |  |  |  |  |  |  |  |  |  |  |  |  |  |
| --- | --- | --- | --- | --- | --- | --- | --- | --- | --- | --- | --- | --- | --- | --- | --- | --- | --- | --- | --- |

XP\_026528620.1

back to list

  

| Protein Coverage
| Supporting Peptides
|

Protein Coverage:

Supporting Peptides:

| Peptide | Uniq | -10lgP | Mass | Length | ppm | m/z | z | RT | Fraction | Scan | Source File | Area BRom | #Feature | #Feature BRom | Start | End | PTM | AScore | Found By |
| --- | --- | --- | --- | --- | --- | --- | --- | --- | --- | --- | --- | --- | --- | --- | --- | --- | --- | --- | --- |
| K.SILDMLLEATSPK.M | Y | 61.92 | 1416.7534 | 13 | 1.3 | 709.3849 | 2 | 99.16 | 1 | 71510 | 01122020\_RID\_2209\_BuCaKA02.raw | 2.5269E5 | 1 | 1 | 362 | 374 |  |  | PEAKS DB |
| total 1 peptides |  |  |  |  |  |  |  |  |  |  |  |  |  |  |  |  |  |  |  |
| --- | --- | --- | --- | --- | --- | --- | --- | --- | --- | --- | --- | --- | --- | --- | --- | --- | --- | --- | --- |

XP\_026569354.1

back to list

  

| Protein Coverage
| Supporting Peptides
|

Protein Coverage:

Supporting Peptides:

| Peptide | Uniq | -10lgP | Mass | Length | ppm | m/z | z | RT | Fraction | Scan | Source File | Area BRom | #Feature | #Feature BRom | Start | End | PTM | AScore | Found By |
| --- | --- | --- | --- | --- | --- | --- | --- | --- | --- | --- | --- | --- | --- | --- | --- | --- | --- | --- | --- |
| K.SILDMLLEATSPK.M | Y | 61.92 | 1416.7534 | 13 | 1.3 | 709.3849 | 2 | 99.16 | 1 | 71510 | 01122020\_RID\_2209\_BuCaKA02.raw | 2.5269E5 | 1 | 1 | 362 | 374 |  |  | PEAKS DB |
| total 1 peptides |  |  |  |  |  |  |  |  |  |  |  |  |  |  |  |  |  |  |  |
| --- | --- | --- | --- | --- | --- | --- | --- | --- | --- | --- | --- | --- | --- | --- | --- | --- | --- | --- | --- |

XP\_026528619.1

back to list

  

| Protein Coverage
| Supporting Peptides
|

Protein Coverage:

Supporting Peptides:

| Peptide | Uniq | -10lgP | Mass | Length | ppm | m/z | z | RT | Fraction | Scan | Source File | Area BRom | #Feature | #Feature BRom | Start | End | PTM | AScore | Found By |
| --- | --- | --- | --- | --- | --- | --- | --- | --- | --- | --- | --- | --- | --- | --- | --- | --- | --- | --- | --- |
| K.SILDMLLEATSPK.M | Y | 61.92 | 1416.7534 | 13 | 1.3 | 709.3849 | 2 | 99.16 | 1 | 71510 | 01122020\_RID\_2209\_BuCaKA02.raw | 2.5269E5 | 1 | 1 | 365 | 377 |  |  | PEAKS DB |
| total 1 peptides |  |  |  |  |  |  |  |  |  |  |  |  |  |  |  |  |  |  |  |
| --- | --- | --- | --- | --- | --- | --- | --- | --- | --- | --- | --- | --- | --- | --- | --- | --- | --- | --- | --- |

XP\_026569353.1

back to list

  

| Protein Coverage
| Supporting Peptides
|

Protein Coverage:

Supporting Peptides:

| Peptide | Uniq | -10lgP | Mass | Length | ppm | m/z | z | RT | Fraction | Scan | Source File | Area BRom | #Feature | #Feature BRom | Start | End | PTM | AScore | Found By |
| --- | --- | --- | --- | --- | --- | --- | --- | --- | --- | --- | --- | --- | --- | --- | --- | --- | --- | --- | --- |
| K.SILDMLLEATSPK.M | Y | 61.92 | 1416.7534 | 13 | 1.3 | 709.3849 | 2 | 99.16 | 1 | 71510 | 01122020\_RID\_2209\_BuCaKA02.raw | 2.5269E5 | 1 | 1 | 365 | 377 |  |  | PEAKS DB |
| total 1 peptides |  |  |  |  |  |  |  |  |  |  |  |  |  |  |  |  |  |  |  |
| --- | --- | --- | --- | --- | --- | --- | --- | --- | --- | --- | --- | --- | --- | --- | --- | --- | --- | --- | --- |

XP\_015669101.1

back to list

  

| Protein Coverage
| Supporting Peptides
|

Protein Coverage:

Supporting Peptides:

| Peptide | Uniq | -10lgP | Mass | Length | ppm | m/z | z | RT | Fraction | Scan | Source File | Area BRom | #Feature | #Feature BRom | Start | End | PTM | AScore | Found By |
| --- | --- | --- | --- | --- | --- | --- | --- | --- | --- | --- | --- | --- | --- | --- | --- | --- | --- | --- | --- |
| K.SILDMLLEATSPK.M | Y | 61.92 | 1416.7534 | 13 | 1.3 | 709.3849 | 2 | 99.16 | 1 | 71510 | 01122020\_RID\_2209\_BuCaKA02.raw | 2.5269E5 | 1 | 1 | 362 | 374 |  |  | PEAKS DB |
| total 1 peptides |  |  |  |  |  |  |  |  |  |  |  |  |  |  |  |  |  |  |  |
| --- | --- | --- | --- | --- | --- | --- | --- | --- | --- | --- | --- | --- | --- | --- | --- | --- | --- | --- | --- |

AFJ51008.1

back to list

  

| Protein Coverage
| Supporting Peptides
|

Protein Coverage:

Supporting Peptides:

| Peptide | Uniq | -10lgP | Mass | Length | ppm | m/z | z | RT | Fraction | Scan | Source File | Area BRom | #Feature | #Feature BRom | Start | End | PTM | AScore | Found By |
| --- | --- | --- | --- | --- | --- | --- | --- | --- | --- | --- | --- | --- | --- | --- | --- | --- | --- | --- | --- |
| K.SILDMLLEATSPK.M | Y | 61.92 | 1416.7534 | 13 | 1.3 | 709.3849 | 2 | 99.16 | 1 | 71510 | 01122020\_RID\_2209\_BuCaKA02.raw | 2.5269E5 | 1 | 1 | 362 | 374 |  |  | PEAKS DB |
| total 1 peptides |  |  |  |  |  |  |  |  |  |  |  |  |  |  |  |  |  |  |  |
| --- | --- | --- | --- | --- | --- | --- | --- | --- | --- | --- | --- | --- | --- | --- | --- | --- | --- | --- | --- |

BAN82088.1

back to list

  

| Protein Coverage
| Supporting Peptides
|

Protein Coverage:

Supporting Peptides:

| Peptide | Uniq | -10lgP | Mass | Length | ppm | m/z | z | RT | Fraction | Scan | Source File | Area BRom | #Feature | #Feature BRom | Start | End | PTM | AScore | Found By |
| --- | --- | --- | --- | --- | --- | --- | --- | --- | --- | --- | --- | --- | --- | --- | --- | --- | --- | --- | --- |
| K.SILDMLLEATSPK.M | Y | 61.92 | 1416.7534 | 13 | 1.3 | 709.3849 | 2 | 99.16 | 1 | 71510 | 01122020\_RID\_2209\_BuCaKA02.raw | 2.5269E5 | 1 | 1 | 365 | 377 |  |  | PEAKS DB |
| total 1 peptides |  |  |  |  |  |  |  |  |  |  |  |  |  |  |  |  |  |  |  |
| --- | --- | --- | --- | --- | --- | --- | --- | --- | --- | --- | --- | --- | --- | --- | --- | --- | --- | --- | --- |

ETE61271.1

back to list

  

| Protein Coverage
| Supporting Peptides
|

Protein Coverage:

Supporting Peptides:

| Peptide | Uniq | -10lgP | Mass | Length | ppm | m/z | z | RT | Fraction | Scan | Source File | Area BRom | #Feature | #Feature BRom | Start | End | PTM | AScore | Found By |
| --- | --- | --- | --- | --- | --- | --- | --- | --- | --- | --- | --- | --- | --- | --- | --- | --- | --- | --- | --- |
| K.SILDMLLEATSPK.M | Y | 61.92 | 1416.7534 | 13 | 1.3 | 709.3849 | 2 | 99.16 | 1 | 71510 | 01122020\_RID\_2209\_BuCaKA02.raw | 2.5269E5 | 1 | 1 | 366 | 378 |  |  | PEAKS DB |
| total 1 peptides |  |  |  |  |  |  |  |  |  |  |  |  |  |  |  |  |  |  |  |
| --- | --- | --- | --- | --- | --- | --- | --- | --- | --- | --- | --- | --- | --- | --- | --- | --- | --- | --- | --- |

XP\_013923763.1

back to list

  

| Protein Coverage
| Supporting Peptides
|

Protein Coverage:

Supporting Peptides:

| Peptide | Uniq | -10lgP | Mass | Length | ppm | m/z | z | RT | Fraction | Scan | Source File | Area BRom | #Feature | #Feature BRom | Start | End | PTM | AScore | Found By |
| --- | --- | --- | --- | --- | --- | --- | --- | --- | --- | --- | --- | --- | --- | --- | --- | --- | --- | --- | --- |
| K.SILDMLLEATSPK.M | Y | 61.92 | 1416.7534 | 13 | 1.3 | 709.3849 | 2 | 99.16 | 1 | 71510 | 01122020\_RID\_2209\_BuCaKA02.raw | 2.5269E5 | 1 | 1 | 362 | 374 |  |  | PEAKS DB |
| total 1 peptides |  |  |  |  |  |  |  |  |  |  |  |  |  |  |  |  |  |  |  |
| --- | --- | --- | --- | --- | --- | --- | --- | --- | --- | --- | --- | --- | --- | --- | --- | --- | --- | --- | --- |

XP\_013923762.1

back to list

  

| Protein Coverage
| Supporting Peptides
|

Protein Coverage:

Supporting Peptides:

| Peptide | Uniq | -10lgP | Mass | Length | ppm | m/z | z | RT | Fraction | Scan | Source File | Area BRom | #Feature | #Feature BRom | Start | End | PTM | AScore | Found By |
| --- | --- | --- | --- | --- | --- | --- | --- | --- | --- | --- | --- | --- | --- | --- | --- | --- | --- | --- | --- |
| K.SILDMLLEATSPK.M | Y | 61.92 | 1416.7534 | 13 | 1.3 | 709.3849 | 2 | 99.16 | 1 | 71510 | 01122020\_RID\_2209\_BuCaKA02.raw | 2.5269E5 | 1 | 1 | 365 | 377 |  |  | PEAKS DB |
| total 1 peptides |  |  |  |  |  |  |  |  |  |  |  |  |  |  |  |  |  |  |  |
| --- | --- | --- | --- | --- | --- | --- | --- | --- | --- | --- | --- | --- | --- | --- | --- | --- | --- | --- | --- |

XP\_007441356.1

back to list

  

| Protein Coverage
| Supporting Peptides
|

Protein Coverage:

Supporting Peptides:

| Peptide | Uniq | -10lgP | Mass | Length | ppm | m/z | z | RT | Fraction | Scan | Source File | Area BRom | #Feature | #Feature BRom | Start | End | PTM | AScore | Found By |
| --- | --- | --- | --- | --- | --- | --- | --- | --- | --- | --- | --- | --- | --- | --- | --- | --- | --- | --- | --- |
| K.SIIDMLLEATSPK.M | Y | 61.92 | 1416.7534 | 13 | 1.3 | 709.3849 | 2 | 99.16 | 1 | 71510 | 01122020\_RID\_2209\_BuCaKA02.raw | 2.5269E5 | 1 | 1 | 227 | 239 |  |  | PEAKS DB |
| total 1 peptides |  |  |  |  |  |  |  |  |  |  |  |  |  |  |  |  |  |  |  |
| --- | --- | --- | --- | --- | --- | --- | --- | --- | --- | --- | --- | --- | --- | --- | --- | --- | --- | --- | --- |

XP\_032088229.1

back to list

  

| Protein Coverage
| Supporting Peptides
|

Protein Coverage:

Supporting Peptides:

| Peptide | Uniq | -10lgP | Mass | Length | ppm | m/z | z | RT | Fraction | Scan | Source File | Area BRom | #Feature | #Feature BRom | Start | End | PTM | AScore | Found By |
| --- | --- | --- | --- | --- | --- | --- | --- | --- | --- | --- | --- | --- | --- | --- | --- | --- | --- | --- | --- |
| K.SILDMLLEATSPK.M | Y | 61.92 | 1416.7534 | 13 | 1.3 | 709.3849 | 2 | 99.16 | 1 | 71510 | 01122020\_RID\_2209\_BuCaKA02.raw | 2.5269E5 | 1 | 1 | 362 | 374 |  |  | PEAKS DB |
| total 1 peptides |  |  |  |  |  |  |  |  |  |  |  |  |  |  |  |  |  |  |  |
| --- | --- | --- | --- | --- | --- | --- | --- | --- | --- | --- | --- | --- | --- | --- | --- | --- | --- | --- | --- |

XP\_034291658.1

back to list

  

| Protein Coverage
| Supporting Peptides
|

Protein Coverage:

Supporting Peptides:

| Peptide | Uniq | -10lgP | Mass | Length | ppm | m/z | z | RT | Fraction | Scan | Source File | Area BRom | #Feature | #Feature BRom | Start | End | PTM | AScore | Found By |
| --- | --- | --- | --- | --- | --- | --- | --- | --- | --- | --- | --- | --- | --- | --- | --- | --- | --- | --- | --- |
| K.SILDMLLEATSPK.M | Y | 61.92 | 1416.7534 | 13 | 1.3 | 709.3849 | 2 | 99.16 | 1 | 71510 | 01122020\_RID\_2209\_BuCaKA02.raw | 2.5269E5 | 1 | 1 | 365 | 377 |  |  | PEAKS DB |
| total 1 peptides |  |  |  |  |  |  |  |  |  |  |  |  |  |  |  |  |  |  |  |
| --- | --- | --- | --- | --- | --- | --- | --- | --- | --- | --- | --- | --- | --- | --- | --- | --- | --- | --- | --- |

XP\_032088228.1

back to list

  

| Protein Coverage
| Supporting Peptides
|

Protein Coverage:

Supporting Peptides:

| Peptide | Uniq | -10lgP | Mass | Length | ppm | m/z | z | RT | Fraction | Scan | Source File | Area BRom | #Feature | #Feature BRom | Start | End | PTM | AScore | Found By |
| --- | --- | --- | --- | --- | --- | --- | --- | --- | --- | --- | --- | --- | --- | --- | --- | --- | --- | --- | --- |
| K.SILDMLLEATSPK.M | Y | 61.92 | 1416.7534 | 13 | 1.3 | 709.3849 | 2 | 99.16 | 1 | 71510 | 01122020\_RID\_2209\_BuCaKA02.raw | 2.5269E5 | 1 | 1 | 365 | 377 |  |  | PEAKS DB |
| total 1 peptides |  |  |  |  |  |  |  |  |  |  |  |  |  |  |  |  |  |  |  |
| --- | --- | --- | --- | --- | --- | --- | --- | --- | --- | --- | --- | --- | --- | --- | --- | --- | --- | --- | --- |

XP\_034291656.1

back to list

  

| Protein Coverage
| Supporting Peptides
|

Protein Coverage:

Supporting Peptides:

| Peptide | Uniq | -10lgP | Mass | Length | ppm | m/z | z | RT | Fraction | Scan | Source File | Area BRom | #Feature | #Feature BRom | Start | End | PTM | AScore | Found By |
| --- | --- | --- | --- | --- | --- | --- | --- | --- | --- | --- | --- | --- | --- | --- | --- | --- | --- | --- | --- |
| K.SILDMLLEATSPK.M | Y | 61.92 | 1416.7534 | 13 | 1.3 | 709.3849 | 2 | 99.16 | 1 | 71510 | 01122020\_RID\_2209\_BuCaKA02.raw | 2.5269E5 | 1 | 1 | 367 | 379 |  |  | PEAKS DB |
| total 1 peptides |  |  |  |  |  |  |  |  |  |  |  |  |  |  |  |  |  |  |  |
| --- | --- | --- | --- | --- | --- | --- | --- | --- | --- | --- | --- | --- | --- | --- | --- | --- | --- | --- | --- |

XP\_034291657.1

back to list

  

| Protein Coverage
| Supporting Peptides
|

Protein Coverage:

Supporting Peptides:

| Peptide | Uniq | -10lgP | Mass | Length | ppm | m/z | z | RT | Fraction | Scan | Source File | Area BRom | #Feature | #Feature BRom | Start | End | PTM | AScore | Found By |
| --- | --- | --- | --- | --- | --- | --- | --- | --- | --- | --- | --- | --- | --- | --- | --- | --- | --- | --- | --- |
| K.SILDMLLEATSPK.M | Y | 61.92 | 1416.7534 | 13 | 1.3 | 709.3849 | 2 | 99.16 | 1 | 71510 | 01122020\_RID\_2209\_BuCaKA02.raw | 2.5269E5 | 1 | 1 | 368 | 380 |  |  | PEAKS DB |
| total 1 peptides |  |  |  |  |  |  |  |  |  |  |  |  |  |  |  |  |  |  |  |
| --- | --- | --- | --- | --- | --- | --- | --- | --- | --- | --- | --- | --- | --- | --- | --- | --- | --- | --- | --- |

XP\_034291655.1

back to list

  

| Protein Coverage
| Supporting Peptides
|

Protein Coverage:

Supporting Peptides:

| Peptide | Uniq | -10lgP | Mass | Length | ppm | m/z | z | RT | Fraction | Scan | Source File | Area BRom | #Feature | #Feature BRom | Start | End | PTM | AScore | Found By |
| --- | --- | --- | --- | --- | --- | --- | --- | --- | --- | --- | --- | --- | --- | --- | --- | --- | --- | --- | --- |
| K.SILDMLLEATSPK.M | Y | 61.92 | 1416.7534 | 13 | 1.3 | 709.3849 | 2 | 99.16 | 1 | 71510 | 01122020\_RID\_2209\_BuCaKA02.raw | 2.5269E5 | 1 | 1 | 368 | 380 |  |  | PEAKS DB |
| total 1 peptides |  |  |  |  |  |  |  |  |  |  |  |  |  |  |  |  |  |  |  |
| --- | --- | --- | --- | --- | --- | --- | --- | --- | --- | --- | --- | --- | --- | --- | --- | --- | --- | --- | --- |

XP\_034265676.1

back to list

  

| Protein Coverage
| Supporting Peptides
|

Protein Coverage:

Supporting Peptides:

| Peptide | Uniq | -10lgP | Mass | Length | ppm | m/z | z | RT | Fraction | Scan | Source File | Area BRom | #Feature | #Feature BRom | Start | End | PTM | AScore | Found By |
| --- | --- | --- | --- | --- | --- | --- | --- | --- | --- | --- | --- | --- | --- | --- | --- | --- | --- | --- | --- |
| G.DSWGILFSHPR.D | Y | 60.31 | 1313.6516 | 11 | -0.3 | 657.8329 | 2 | 52.38 | 1 | 33030 | 01122020\_RID\_2209\_BuCaKA02.raw | 3.5037E6 | 1 | 1 | 30 | 40 |  |  | PEAKS DB |
| total 1 peptides |  |  |  |  |  |  |  |  |  |  |  |  |  |  |  |  |  |  |  |
| --- | --- | --- | --- | --- | --- | --- | --- | --- | --- | --- | --- | --- | --- | --- | --- | --- | --- | --- | --- |

XP\_032082279.1

back to list

  

| Protein Coverage
| Supporting Peptides
|

Protein Coverage:

Supporting Peptides:

| Peptide | Uniq | -10lgP | Mass | Length | ppm | m/z | z | RT | Fraction | Scan | Source File | Area BRom | #Feature | #Feature BRom | Start | End | PTM | AScore | Found By |
| --- | --- | --- | --- | --- | --- | --- | --- | --- | --- | --- | --- | --- | --- | --- | --- | --- | --- | --- | --- |
| G.DSWGILFSHPR.D | Y | 60.31 | 1313.6516 | 11 | -0.3 | 657.8329 | 2 | 52.38 | 1 | 33030 | 01122020\_RID\_2209\_BuCaKA02.raw | 3.5037E6 | 1 | 1 | 30 | 40 |  |  | PEAKS DB |
| total 1 peptides |  |  |  |  |  |  |  |  |  |  |  |  |  |  |  |  |  |  |  |
| --- | --- | --- | --- | --- | --- | --- | --- | --- | --- | --- | --- | --- | --- | --- | --- | --- | --- | --- | --- |

JAI09679.1

back to list

  

| Protein Coverage
| Supporting Peptides
|

Protein Coverage:

Supporting Peptides:

| Peptide | Uniq | -10lgP | Mass | Length | ppm | m/z | z | RT | Fraction | Scan | Source File | Area BRom | #Feature | #Feature BRom | Start | End | PTM | AScore | Found By |
| --- | --- | --- | --- | --- | --- | --- | --- | --- | --- | --- | --- | --- | --- | --- | --- | --- | --- | --- | --- |
| G.DSWGILFSHPR.D | Y | 60.31 | 1313.6516 | 11 | -0.3 | 657.8329 | 2 | 52.38 | 1 | 33030 | 01122020\_RID\_2209\_BuCaKA02.raw | 3.5037E6 | 1 | 1 | 30 | 40 |  |  | PEAKS DB |
| total 1 peptides |  |  |  |  |  |  |  |  |  |  |  |  |  |  |  |  |  |  |  |
| --- | --- | --- | --- | --- | --- | --- | --- | --- | --- | --- | --- | --- | --- | --- | --- | --- | --- | --- | --- |

AFJ50899.1

back to list

  

| Protein Coverage
| Supporting Peptides
|

Protein Coverage:

Supporting Peptides:

| Peptide | Uniq | -10lgP | Mass | Length | ppm | m/z | z | RT | Fraction | Scan | Source File | Area BRom | #Feature | #Feature BRom | Start | End | PTM | AScore | Found By |
| --- | --- | --- | --- | --- | --- | --- | --- | --- | --- | --- | --- | --- | --- | --- | --- | --- | --- | --- | --- |
| G.DSWGILFSHPR.D | Y | 60.31 | 1313.6516 | 11 | -0.3 | 657.8329 | 2 | 52.38 | 1 | 33030 | 01122020\_RID\_2209\_BuCaKA02.raw | 3.5037E6 | 1 | 1 | 30 | 40 |  |  | PEAKS DB |
| total 1 peptides |  |  |  |  |  |  |  |  |  |  |  |  |  |  |  |  |  |  |  |
| --- | --- | --- | --- | --- | --- | --- | --- | --- | --- | --- | --- | --- | --- | --- | --- | --- | --- | --- | --- |

JAB53717.1

back to list

  

| Protein Coverage
| Supporting Peptides
|

Protein Coverage:

Supporting Peptides:

| Peptide | Uniq | -10lgP | Mass | Length | ppm | m/z | z | RT | Fraction | Scan | Source File | Area BRom | #Feature | #Feature BRom | Start | End | PTM | AScore | Found By |
| --- | --- | --- | --- | --- | --- | --- | --- | --- | --- | --- | --- | --- | --- | --- | --- | --- | --- | --- | --- |
| G.DSWGILFSHPR.D | Y | 60.31 | 1313.6516 | 11 | -0.3 | 657.8329 | 2 | 52.38 | 1 | 33030 | 01122020\_RID\_2209\_BuCaKA02.raw | 3.5037E6 | 1 | 1 | 30 | 40 |  |  | PEAKS DB |
| total 1 peptides |  |  |  |  |  |  |  |  |  |  |  |  |  |  |  |  |  |  |  |
| --- | --- | --- | --- | --- | --- | --- | --- | --- | --- | --- | --- | --- | --- | --- | --- | --- | --- | --- | --- |

XP\_026524951.1

back to list

  

| Protein Coverage
| Supporting Peptides
|

Protein Coverage:

Supporting Peptides:

| Peptide | Uniq | -10lgP | Mass | Length | ppm | m/z | z | RT | Fraction | Scan | Source File | Area BRom | #Feature | #Feature BRom | Start | End | PTM | AScore | Found By |
| --- | --- | --- | --- | --- | --- | --- | --- | --- | --- | --- | --- | --- | --- | --- | --- | --- | --- | --- | --- |
| G.DSWGILFSHPR.D | Y | 60.31 | 1313.6516 | 11 | -0.3 | 657.8329 | 2 | 52.38 | 1 | 33030 | 01122020\_RID\_2209\_BuCaKA02.raw | 3.5037E6 | 1 | 1 | 30 | 40 |  |  | PEAKS DB |
| total 1 peptides |  |  |  |  |  |  |  |  |  |  |  |  |  |  |  |  |  |  |  |
| --- | --- | --- | --- | --- | --- | --- | --- | --- | --- | --- | --- | --- | --- | --- | --- | --- | --- | --- | --- |

JAG67074.1

back to list

  

| Protein Coverage
| Supporting Peptides
|

Protein Coverage:

Supporting Peptides:

| Peptide | Uniq | -10lgP | Mass | Length | ppm | m/z | z | RT | Fraction | Scan | Source File | Area BRom | #Feature | #Feature BRom | Start | End | PTM | AScore | Found By |
| --- | --- | --- | --- | --- | --- | --- | --- | --- | --- | --- | --- | --- | --- | --- | --- | --- | --- | --- | --- |
| G.DSWGILFSHPR.D | Y | 60.31 | 1313.6516 | 11 | -0.3 | 657.8329 | 2 | 52.38 | 1 | 33030 | 01122020\_RID\_2209\_BuCaKA02.raw | 3.5037E6 | 1 | 1 | 30 | 40 |  |  | PEAKS DB |
| total 1 peptides |  |  |  |  |  |  |  |  |  |  |  |  |  |  |  |  |  |  |  |
| --- | --- | --- | --- | --- | --- | --- | --- | --- | --- | --- | --- | --- | --- | --- | --- | --- | --- | --- | --- |

LAB58410.1

back to list

  

| Protein Coverage
| Supporting Peptides
|

Protein Coverage:

Supporting Peptides:

| Peptide | Uniq | -10lgP | Mass | Length | ppm | m/z | z | RT | Fraction | Scan | Source File | Area BRom | #Feature | #Feature BRom | Start | End | PTM | AScore | Found By |
| --- | --- | --- | --- | --- | --- | --- | --- | --- | --- | --- | --- | --- | --- | --- | --- | --- | --- | --- | --- |
| G.DSWGILFSHPR.D | Y | 60.31 | 1313.6516 | 11 | -0.3 | 657.8329 | 2 | 52.38 | 1 | 33030 | 01122020\_RID\_2209\_BuCaKA02.raw | 3.5037E6 | 1 | 1 | 30 | 40 |  |  | PEAKS DB |
| total 1 peptides |  |  |  |  |  |  |  |  |  |  |  |  |  |  |  |  |  |  |  |
| --- | --- | --- | --- | --- | --- | --- | --- | --- | --- | --- | --- | --- | --- | --- | --- | --- | --- | --- | --- |

JAA96187.1

back to list

  

| Protein Coverage
| Supporting Peptides
|

Protein Coverage:

Supporting Peptides:

| Peptide | Uniq | -10lgP | Mass | Length | ppm | m/z | z | RT | Fraction | Scan | Source File | Area BRom | #Feature | #Feature BRom | Start | End | PTM | AScore | Found By |
| --- | --- | --- | --- | --- | --- | --- | --- | --- | --- | --- | --- | --- | --- | --- | --- | --- | --- | --- | --- |
| G.DSWGILFSHPR.D | Y | 60.31 | 1313.6516 | 11 | -0.3 | 657.8329 | 2 | 52.38 | 1 | 33030 | 01122020\_RID\_2209\_BuCaKA02.raw | 3.5037E6 | 1 | 1 | 30 | 40 |  |  | PEAKS DB |
| total 1 peptides |  |  |  |  |  |  |  |  |  |  |  |  |  |  |  |  |  |  |  |
| --- | --- | --- | --- | --- | --- | --- | --- | --- | --- | --- | --- | --- | --- | --- | --- | --- | --- | --- | --- |

JAV49687.1

back to list

  

| Protein Coverage
| Supporting Peptides
|

Protein Coverage:

Supporting Peptides:

| Peptide | Uniq | -10lgP | Mass | Length | ppm | m/z | z | RT | Fraction | Scan | Source File | Area BRom | #Feature | #Feature BRom | Start | End | PTM | AScore | Found By |
| --- | --- | --- | --- | --- | --- | --- | --- | --- | --- | --- | --- | --- | --- | --- | --- | --- | --- | --- | --- |
| G.DSWGILFSHPR.D | Y | 60.31 | 1313.6516 | 11 | -0.3 | 657.8329 | 2 | 52.38 | 1 | 33030 | 01122020\_RID\_2209\_BuCaKA02.raw | 3.5037E6 | 1 | 1 | 30 | 40 |  |  | PEAKS DB |
| total 1 peptides |  |  |  |  |  |  |  |  |  |  |  |  |  |  |  |  |  |  |  |
| --- | --- | --- | --- | --- | --- | --- | --- | --- | --- | --- | --- | --- | --- | --- | --- | --- | --- | --- | --- |

XP\_015687493.1

back to list

  

| Protein Coverage
| Supporting Peptides
|

Protein Coverage:

Supporting Peptides:

| Peptide | Uniq | -10lgP | Mass | Length | ppm | m/z | z | RT | Fraction | Scan | Source File | Area BRom | #Feature | #Feature BRom | Start | End | PTM | AScore | Found By |
| --- | --- | --- | --- | --- | --- | --- | --- | --- | --- | --- | --- | --- | --- | --- | --- | --- | --- | --- | --- |
| G.DSWGILFSHPR.D | Y | 60.31 | 1313.6516 | 11 | -0.3 | 657.8329 | 2 | 52.38 | 1 | 33030 | 01122020\_RID\_2209\_BuCaKA02.raw | 3.5037E6 | 1 | 1 | 30 | 40 |  |  | PEAKS DB |
| total 1 peptides |  |  |  |  |  |  |  |  |  |  |  |  |  |  |  |  |  |  |  |
| --- | --- | --- | --- | --- | --- | --- | --- | --- | --- | --- | --- | --- | --- | --- | --- | --- | --- | --- | --- |

JAI12121.1

back to list

  

| Protein Coverage
| Supporting Peptides
|

Protein Coverage:

Supporting Peptides:

| Peptide | Uniq | -10lgP | Mass | Length | ppm | m/z | z | RT | Fraction | Scan | Source File | Area BRom | #Feature | #Feature BRom | Start | End | PTM | AScore | Found By |
| --- | --- | --- | --- | --- | --- | --- | --- | --- | --- | --- | --- | --- | --- | --- | --- | --- | --- | --- | --- |
| G.DSWGILFSHPR.D | Y | 60.31 | 1313.6516 | 11 | -0.3 | 657.8329 | 2 | 52.38 | 1 | 33030 | 01122020\_RID\_2209\_BuCaKA02.raw | 3.5037E6 | 1 | 1 | 30 | 40 |  |  | PEAKS DB |
| total 1 peptides |  |  |  |  |  |  |  |  |  |  |  |  |  |  |  |  |  |  |  |
| --- | --- | --- | --- | --- | --- | --- | --- | --- | --- | --- | --- | --- | --- | --- | --- | --- | --- | --- | --- |

XP\_026559154.1

back to list

  

| Protein Coverage
| Supporting Peptides
|

Protein Coverage:

Supporting Peptides:

| Peptide | Uniq | -10lgP | Mass | Length | ppm | m/z | z | RT | Fraction | Scan | Source File | Area BRom | #Feature | #Feature BRom | Start | End | PTM | AScore | Found By |
| --- | --- | --- | --- | --- | --- | --- | --- | --- | --- | --- | --- | --- | --- | --- | --- | --- | --- | --- | --- |
| G.DSWGILFSHPR.D | Y | 60.31 | 1313.6516 | 11 | -0.3 | 657.8329 | 2 | 52.38 | 1 | 33030 | 01122020\_RID\_2209\_BuCaKA02.raw | 3.5037E6 | 1 | 1 | 30 | 40 |  |  | PEAKS DB |
| total 1 peptides |  |  |  |  |  |  |  |  |  |  |  |  |  |  |  |  |  |  |  |
| --- | --- | --- | --- | --- | --- | --- | --- | --- | --- | --- | --- | --- | --- | --- | --- | --- | --- | --- | --- |

ETE73981.1

back to list

  

| Protein Coverage
| Supporting Peptides
|

Protein Coverage:

Supporting Peptides:

| Peptide | Uniq | -10lgP | Mass | Length | ppm | m/z | z | RT | Fraction | Scan | Source File | Area BRom | #Feature | #Feature BRom | Start | End | PTM | AScore | Found By |
| --- | --- | --- | --- | --- | --- | --- | --- | --- | --- | --- | --- | --- | --- | --- | --- | --- | --- | --- | --- |
| R.NLLSVAYKNVVGC(+57.02)QR.S | Y | 58.22 | 1719.9091 | 15 | 1.1 | 574.3109 | 3 | 42.31 | 1 | 25383 | 01122020\_RID\_2209\_BuCaKA02.raw | 0 | 0 | 0 | 21 | 35 | Carbamidomethylation | C13:Carbamidomethylation:1000.00 | PEAKS DB |
| total 1 peptides |  |  |  |  |  |  |  |  |  |  |  |  |  |  |  |  |  |  |  |
| --- | --- | --- | --- | --- | --- | --- | --- | --- | --- | --- | --- | --- | --- | --- | --- | --- | --- | --- | --- |

AAC83997.1

back to list

  

| Protein Coverage
| Supporting Peptides
|

Protein Coverage:

Supporting Peptides:

| Peptide | Uniq | -10lgP | Mass | Length | ppm | m/z | z | RT | Fraction | Scan | Source File | Area BRom | #Feature | #Feature BRom | Start | End | PTM | AScore | Found By |
| --- | --- | --- | --- | --- | --- | --- | --- | --- | --- | --- | --- | --- | --- | --- | --- | --- | --- | --- | --- |
| M.WC(+57.02)DAFC(+57.02)SSR.G | Y | 56.12 | 1187.4489 | 9 | 0.5 | 594.7320 | 2 | 25.21 | 1 | 12147 | 01122020\_RID\_2209\_BuCaKA02.raw | 1.1946E7 | 1 | 1 | 28 | 36 | Carbamidomethylation | C2:Carbamidomethylation:1000.00;C6:Carbamidomethylation:1000.00 | PEAKS DB |
| total 1 peptides |  |  |  |  |  |  |  |  |  |  |  |  |  |  |  |  |  |  |  |
| --- | --- | --- | --- | --- | --- | --- | --- | --- | --- | --- | --- | --- | --- | --- | --- | --- | --- | --- | --- |

AAC83990.1

back to list

  

| Protein Coverage
| Supporting Peptides
|

Protein Coverage:

Supporting Peptides:

| Peptide | Uniq | -10lgP | Mass | Length | ppm | m/z | z | RT | Fraction | Scan | Source File | Area BRom | #Feature | #Feature BRom | Start | End | PTM | AScore | Found By |
| --- | --- | --- | --- | --- | --- | --- | --- | --- | --- | --- | --- | --- | --- | --- | --- | --- | --- | --- | --- |
| M.WC(+57.02)DAFC(+57.02)SSR.G | Y | 56.12 | 1187.4489 | 9 | 0.5 | 594.7320 | 2 | 25.21 | 1 | 12147 | 01122020\_RID\_2209\_BuCaKA02.raw | 1.1946E7 | 1 | 1 | 28 | 36 | Carbamidomethylation | C2:Carbamidomethylation:1000.00;C6:Carbamidomethylation:1000.00 | PEAKS DB |
| total 1 peptides |  |  |  |  |  |  |  |  |  |  |  |  |  |  |  |  |  |  |  |
| --- | --- | --- | --- | --- | --- | --- | --- | --- | --- | --- | --- | --- | --- | --- | --- | --- | --- | --- | --- |

P01385.1

back to list

  

| Protein Coverage
| Supporting Peptides
|

Protein Coverage:

Supporting Peptides:

| Peptide | Uniq | -10lgP | Mass | Length | ppm | m/z | z | RT | Fraction | Scan | Source File | Area BRom | #Feature | #Feature BRom | Start | End | PTM | AScore | Found By |
| --- | --- | --- | --- | --- | --- | --- | --- | --- | --- | --- | --- | --- | --- | --- | --- | --- | --- | --- | --- |
| T.WC(+57.02)DAFC(+57.02)SSR.G | Y | 56.12 | 1187.4489 | 9 | 0.5 | 594.7320 | 2 | 25.21 | 1 | 12147 | 01122020\_RID\_2209\_BuCaKA02.raw | 1.1946E7 | 1 | 1 | 25 | 33 | Carbamidomethylation | C2:Carbamidomethylation:1000.00;C6:Carbamidomethylation:1000.00 | PEAKS DB |
| total 1 peptides |  |  |  |  |  |  |  |  |  |  |  |  |  |  |  |  |  |  |  |
| --- | --- | --- | --- | --- | --- | --- | --- | --- | --- | --- | --- | --- | --- | --- | --- | --- | --- | --- | --- |

0512217A

back to list

  

| Protein Coverage
| Supporting Peptides
|

Protein Coverage:

Supporting Peptides:

| Peptide | Uniq | -10lgP | Mass | Length | ppm | m/z | z | RT | Fraction | Scan | Source File | Area BRom | #Feature | #Feature BRom | Start | End | PTM | AScore | Found By |
| --- | --- | --- | --- | --- | --- | --- | --- | --- | --- | --- | --- | --- | --- | --- | --- | --- | --- | --- | --- |
| T.WC(+57.02)DAFC(+57.02)SSR.G | Y | 56.12 | 1187.4489 | 9 | 0.5 | 594.7320 | 2 | 25.21 | 1 | 12147 | 01122020\_RID\_2209\_BuCaKA02.raw | 1.1946E7 | 1 | 1 | 25 | 33 | Carbamidomethylation | C2:Carbamidomethylation:1000.00;C6:Carbamidomethylation:1000.00 | PEAKS DB |
| total 1 peptides |  |  |  |  |  |  |  |  |  |  |  |  |  |  |  |  |  |  |  |
| --- | --- | --- | --- | --- | --- | --- | --- | --- | --- | --- | --- | --- | --- | --- | --- | --- | --- | --- | --- |

AAC83992.1

back to list

  

| Protein Coverage
| Supporting Peptides
|

Protein Coverage:

Supporting Peptides:

| Peptide | Uniq | -10lgP | Mass | Length | ppm | m/z | z | RT | Fraction | Scan | Source File | Area BRom | #Feature | #Feature BRom | Start | End | PTM | AScore | Found By |
| --- | --- | --- | --- | --- | --- | --- | --- | --- | --- | --- | --- | --- | --- | --- | --- | --- | --- | --- | --- |
| M.WC(+57.02)DAFC(+57.02)SSR.G | Y | 56.12 | 1187.4489 | 9 | 0.5 | 594.7320 | 2 | 25.21 | 1 | 12147 | 01122020\_RID\_2209\_BuCaKA02.raw | 1.1946E7 | 1 | 1 | 28 | 36 | Carbamidomethylation | C2:Carbamidomethylation:1000.00;C6:Carbamidomethylation:1000.00 | PEAKS DB |
| total 1 peptides |  |  |  |  |  |  |  |  |  |  |  |  |  |  |  |  |  |  |  |
| --- | --- | --- | --- | --- | --- | --- | --- | --- | --- | --- | --- | --- | --- | --- | --- | --- | --- | --- | --- |

1HAJ

back to list

  

| Protein Coverage
| Supporting Peptides
|

Protein Coverage:

Supporting Peptides:

| Peptide | Uniq | -10lgP | Mass | Length | ppm | m/z | z | RT | Fraction | Scan | Source File | Area BRom | #Feature | #Feature BRom | Start | End | PTM | AScore | Found By |
| --- | --- | --- | --- | --- | --- | --- | --- | --- | --- | --- | --- | --- | --- | --- | --- | --- | --- | --- | --- |
| M.WC(+57.02)DAFC(+57.02)SSR.G | Y | 56.12 | 1187.4489 | 9 | 0.5 | 594.7320 | 2 | 25.21 | 1 | 12147 | 01122020\_RID\_2209\_BuCaKA02.raw | 1.1946E7 | 1 | 1 | 28 | 36 | Carbamidomethylation | C2:Carbamidomethylation:1000.00;C6:Carbamidomethylation:1000.00 | PEAKS DB |
| total 1 peptides |  |  |  |  |  |  |  |  |  |  |  |  |  |  |  |  |  |  |  |
| --- | --- | --- | --- | --- | --- | --- | --- | --- | --- | --- | --- | --- | --- | --- | --- | --- | --- | --- | --- |

1IDH

back to list

  

| Protein Coverage
| Supporting Peptides
|

Protein Coverage:

Supporting Peptides:

| Peptide | Uniq | -10lgP | Mass | Length | ppm | m/z | z | RT | Fraction | Scan | Source File | Area BRom | #Feature | #Feature BRom | Start | End | PTM | AScore | Found By |
| --- | --- | --- | --- | --- | --- | --- | --- | --- | --- | --- | --- | --- | --- | --- | --- | --- | --- | --- | --- |
| M.WC(+57.02)DAFC(+57.02)SSR.G | Y | 56.12 | 1187.4489 | 9 | 0.5 | 594.7320 | 2 | 25.21 | 1 | 12147 | 01122020\_RID\_2209\_BuCaKA02.raw | 1.1946E7 | 1 | 1 | 28 | 36 | Carbamidomethylation | C2:Carbamidomethylation:1000.00;C6:Carbamidomethylation:1000.00 | PEAKS DB |
| total 1 peptides |  |  |  |  |  |  |  |  |  |  |  |  |  |  |  |  |  |  |  |
| --- | --- | --- | --- | --- | --- | --- | --- | --- | --- | --- | --- | --- | --- | --- | --- | --- | --- | --- | --- |

2BTX

back to list

  

| Protein Coverage
| Supporting Peptides
|

Protein Coverage:

Supporting Peptides:

| Peptide | Uniq | -10lgP | Mass | Length | ppm | m/z | z | RT | Fraction | Scan | Source File | Area BRom | #Feature | #Feature BRom | Start | End | PTM | AScore | Found By |
| --- | --- | --- | --- | --- | --- | --- | --- | --- | --- | --- | --- | --- | --- | --- | --- | --- | --- | --- | --- |
| M.WC(+57.02)DAFC(+57.02)SSR.G | Y | 56.12 | 1187.4489 | 9 | 0.5 | 594.7320 | 2 | 25.21 | 1 | 12147 | 01122020\_RID\_2209\_BuCaKA02.raw | 1.1946E7 | 1 | 1 | 28 | 36 | Carbamidomethylation | C2:Carbamidomethylation:1000.00;C6:Carbamidomethylation:1000.00 | PEAKS DB |
| total 1 peptides |  |  |  |  |  |  |  |  |  |  |  |  |  |  |  |  |  |  |  |
| --- | --- | --- | --- | --- | --- | --- | --- | --- | --- | --- | --- | --- | --- | --- | --- | --- | --- | --- | --- |

1IK8

back to list

  

| Protein Coverage
| Supporting Peptides
|

Protein Coverage:

Supporting Peptides:

| Peptide | Uniq | -10lgP | Mass | Length | ppm | m/z | z | RT | Fraction | Scan | Source File | Area BRom | #Feature | #Feature BRom | Start | End | PTM | AScore | Found By |
| --- | --- | --- | --- | --- | --- | --- | --- | --- | --- | --- | --- | --- | --- | --- | --- | --- | --- | --- | --- |
| M.WC(+57.02)DAFC(+57.02)SSR.G | Y | 56.12 | 1187.4489 | 9 | 0.5 | 594.7320 | 2 | 25.21 | 1 | 12147 | 01122020\_RID\_2209\_BuCaKA02.raw | 1.1946E7 | 1 | 1 | 28 | 36 | Carbamidomethylation | C2:Carbamidomethylation:1000.00;C6:Carbamidomethylation:1000.00 | PEAKS DB |
| total 1 peptides |  |  |  |  |  |  |  |  |  |  |  |  |  |  |  |  |  |  |  |
| --- | --- | --- | --- | --- | --- | --- | --- | --- | --- | --- | --- | --- | --- | --- | --- | --- | --- | --- | --- |

1BXP

back to list

  

| Protein Coverage
| Supporting Peptides
|

Protein Coverage:

Supporting Peptides:

| Peptide | Uniq | -10lgP | Mass | Length | ppm | m/z | z | RT | Fraction | Scan | Source File | Area BRom | #Feature | #Feature BRom | Start | End | PTM | AScore | Found By |
| --- | --- | --- | --- | --- | --- | --- | --- | --- | --- | --- | --- | --- | --- | --- | --- | --- | --- | --- | --- |
| M.WC(+57.02)DAFC(+57.02)SSR.G | Y | 56.12 | 1187.4489 | 9 | 0.5 | 594.7320 | 2 | 25.21 | 1 | 12147 | 01122020\_RID\_2209\_BuCaKA02.raw | 1.1946E7 | 1 | 1 | 28 | 36 | Carbamidomethylation | C2:Carbamidomethylation:1000.00;C6:Carbamidomethylation:1000.00 | PEAKS DB |
| total 1 peptides |  |  |  |  |  |  |  |  |  |  |  |  |  |  |  |  |  |  |  |
| --- | --- | --- | --- | --- | --- | --- | --- | --- | --- | --- | --- | --- | --- | --- | --- | --- | --- | --- | --- |

1ABT

back to list

  

| Protein Coverage
| Supporting Peptides
|

Protein Coverage:

Supporting Peptides:

| Peptide | Uniq | -10lgP | Mass | Length | ppm | m/z | z | RT | Fraction | Scan | Source File | Area BRom | #Feature | #Feature BRom | Start | End | PTM | AScore | Found By |
| --- | --- | --- | --- | --- | --- | --- | --- | --- | --- | --- | --- | --- | --- | --- | --- | --- | --- | --- | --- |
| M.WC(+57.02)DAFC(+57.02)SSR.G | Y | 56.12 | 1187.4489 | 9 | 0.5 | 594.7320 | 2 | 25.21 | 1 | 12147 | 01122020\_RID\_2209\_BuCaKA02.raw | 1.1946E7 | 1 | 1 | 28 | 36 | Carbamidomethylation | C2:Carbamidomethylation:1000.00;C6:Carbamidomethylation:1000.00 | PEAKS DB |
| total 1 peptides |  |  |  |  |  |  |  |  |  |  |  |  |  |  |  |  |  |  |  |
| --- | --- | --- | --- | --- | --- | --- | --- | --- | --- | --- | --- | --- | --- | --- | --- | --- | --- | --- | --- |

1IKC

back to list

  

| Protein Coverage
| Supporting Peptides
|

Protein Coverage:

Supporting Peptides:

| Peptide | Uniq | -10lgP | Mass | Length | ppm | m/z | z | RT | Fraction | Scan | Source File | Area BRom | #Feature | #Feature BRom | Start | End | PTM | AScore | Found By |
| --- | --- | --- | --- | --- | --- | --- | --- | --- | --- | --- | --- | --- | --- | --- | --- | --- | --- | --- | --- |
| M.WC(+57.02)DAFC(+57.02)SSR.G | Y | 56.12 | 1187.4489 | 9 | 0.5 | 594.7320 | 2 | 25.21 | 1 | 12147 | 01122020\_RID\_2209\_BuCaKA02.raw | 1.1946E7 | 1 | 1 | 28 | 36 | Carbamidomethylation | C2:Carbamidomethylation:1000.00;C6:Carbamidomethylation:1000.00 | PEAKS DB |
| total 1 peptides |  |  |  |  |  |  |  |  |  |  |  |  |  |  |  |  |  |  |  |
| --- | --- | --- | --- | --- | --- | --- | --- | --- | --- | --- | --- | --- | --- | --- | --- | --- | --- | --- | --- |

AAC83994.1

back to list

  

| Protein Coverage
| Supporting Peptides
|

Protein Coverage:

Supporting Peptides:

| Peptide | Uniq | -10lgP | Mass | Length | ppm | m/z | z | RT | Fraction | Scan | Source File | Area BRom | #Feature | #Feature BRom | Start | End | PTM | AScore | Found By |
| --- | --- | --- | --- | --- | --- | --- | --- | --- | --- | --- | --- | --- | --- | --- | --- | --- | --- | --- | --- |
| M.WC(+57.02)DAFC(+57.02)SSR.G | Y | 56.12 | 1187.4489 | 9 | 0.5 | 594.7320 | 2 | 25.21 | 1 | 12147 | 01122020\_RID\_2209\_BuCaKA02.raw | 1.1946E7 | 1 | 1 | 28 | 36 | Carbamidomethylation | C2:Carbamidomethylation:1000.00;C6:Carbamidomethylation:1000.00 | PEAKS DB |
| total 1 peptides |  |  |  |  |  |  |  |  |  |  |  |  |  |  |  |  |  |  |  |
| --- | --- | --- | --- | --- | --- | --- | --- | --- | --- | --- | --- | --- | --- | --- | --- | --- | --- | --- | --- |

AAC83991.1

back to list

  

| Protein Coverage
| Supporting Peptides
|

Protein Coverage:

Supporting Peptides:

| Peptide | Uniq | -10lgP | Mass | Length | ppm | m/z | z | RT | Fraction | Scan | Source File | Area BRom | #Feature | #Feature BRom | Start | End | PTM | AScore | Found By |
| --- | --- | --- | --- | --- | --- | --- | --- | --- | --- | --- | --- | --- | --- | --- | --- | --- | --- | --- | --- |
| M.WC(+57.02)DAFC(+57.02)SSR.G | Y | 56.12 | 1187.4489 | 9 | 0.5 | 594.7320 | 2 | 25.21 | 1 | 12147 | 01122020\_RID\_2209\_BuCaKA02.raw | 1.1946E7 | 1 | 1 | 28 | 36 | Carbamidomethylation | C2:Carbamidomethylation:1000.00;C6:Carbamidomethylation:1000.00 | PEAKS DB |
| total 1 peptides |  |  |  |  |  |  |  |  |  |  |  |  |  |  |  |  |  |  |  |
| --- | --- | --- | --- | --- | --- | --- | --- | --- | --- | --- | --- | --- | --- | --- | --- | --- | --- | --- | --- |

1IDL

back to list

  

| Protein Coverage
| Supporting Peptides
|

Protein Coverage:

Supporting Peptides:

| Peptide | Uniq | -10lgP | Mass | Length | ppm | m/z | z | RT | Fraction | Scan | Source File | Area BRom | #Feature | #Feature BRom | Start | End | PTM | AScore | Found By |
| --- | --- | --- | --- | --- | --- | --- | --- | --- | --- | --- | --- | --- | --- | --- | --- | --- | --- | --- | --- |
| M.WC(+57.02)DAFC(+57.02)SSR.G | Y | 56.12 | 1187.4489 | 9 | 0.5 | 594.7320 | 2 | 25.21 | 1 | 12147 | 01122020\_RID\_2209\_BuCaKA02.raw | 1.1946E7 | 1 | 1 | 28 | 36 | Carbamidomethylation | C2:Carbamidomethylation:1000.00;C6:Carbamidomethylation:1000.00 | PEAKS DB |
| total 1 peptides |  |  |  |  |  |  |  |  |  |  |  |  |  |  |  |  |  |  |  |
| --- | --- | --- | --- | --- | --- | --- | --- | --- | --- | --- | --- | --- | --- | --- | --- | --- | --- | --- | --- |

1HOY

back to list

  

| Protein Coverage
| Supporting Peptides
|

Protein Coverage:

Supporting Peptides:

| Peptide | Uniq | -10lgP | Mass | Length | ppm | m/z | z | RT | Fraction | Scan | Source File | Area BRom | #Feature | #Feature BRom | Start | End | PTM | AScore | Found By |
| --- | --- | --- | --- | --- | --- | --- | --- | --- | --- | --- | --- | --- | --- | --- | --- | --- | --- | --- | --- |
| M.WC(+57.02)DAFC(+57.02)SSR.G | Y | 56.12 | 1187.4489 | 9 | 0.5 | 594.7320 | 2 | 25.21 | 1 | 12147 | 01122020\_RID\_2209\_BuCaKA02.raw | 1.1946E7 | 1 | 1 | 28 | 36 | Carbamidomethylation | C2:Carbamidomethylation:1000.00;C6:Carbamidomethylation:1000.00 | PEAKS DB |
| total 1 peptides |  |  |  |  |  |  |  |  |  |  |  |  |  |  |  |  |  |  |  |
| --- | --- | --- | --- | --- | --- | --- | --- | --- | --- | --- | --- | --- | --- | --- | --- | --- | --- | --- | --- |

AAC83993.1

back to list

  

| Protein Coverage
| Supporting Peptides
|

Protein Coverage:

Supporting Peptides:

| Peptide | Uniq | -10lgP | Mass | Length | ppm | m/z | z | RT | Fraction | Scan | Source File | Area BRom | #Feature | #Feature BRom | Start | End | PTM | AScore | Found By |
| --- | --- | --- | --- | --- | --- | --- | --- | --- | --- | --- | --- | --- | --- | --- | --- | --- | --- | --- | --- |
| M.WC(+57.02)DAFC(+57.02)SSR.G | Y | 56.12 | 1187.4489 | 9 | 0.5 | 594.7320 | 2 | 25.21 | 1 | 12147 | 01122020\_RID\_2209\_BuCaKA02.raw | 1.1946E7 | 1 | 1 | 28 | 36 | Carbamidomethylation | C2:Carbamidomethylation:1000.00;C6:Carbamidomethylation:1000.00 | PEAKS DB |
| total 1 peptides |  |  |  |  |  |  |  |  |  |  |  |  |  |  |  |  |  |  |  |
| --- | --- | --- | --- | --- | --- | --- | --- | --- | --- | --- | --- | --- | --- | --- | --- | --- | --- | --- | --- |

720920A

back to list

  

| Protein Coverage
| Supporting Peptides
|

Protein Coverage:

Supporting Peptides:

| Peptide | Uniq | -10lgP | Mass | Length | ppm | m/z | z | RT | Fraction | Scan | Source File | Area BRom | #Feature | #Feature BRom | Start | End | PTM | AScore | Found By |
| --- | --- | --- | --- | --- | --- | --- | --- | --- | --- | --- | --- | --- | --- | --- | --- | --- | --- | --- | --- |
| M.WC(+57.02)DAFC(+57.02)SSR.G | Y | 56.12 | 1187.4489 | 9 | 0.5 | 594.7320 | 2 | 25.21 | 1 | 12147 | 01122020\_RID\_2209\_BuCaKA02.raw | 1.1946E7 | 1 | 1 | 28 | 36 | Carbamidomethylation | C2:Carbamidomethylation:1000.00;C6:Carbamidomethylation:1000.00 | PEAKS DB |
| total 1 peptides |  |  |  |  |  |  |  |  |  |  |  |  |  |  |  |  |  |  |  |
| --- | --- | --- | --- | --- | --- | --- | --- | --- | --- | --- | --- | --- | --- | --- | --- | --- | --- | --- | --- |

2ABX

back to list

  

| Protein Coverage
| Supporting Peptides
|

Protein Coverage:

Supporting Peptides:

| Peptide | Uniq | -10lgP | Mass | Length | ppm | m/z | z | RT | Fraction | Scan | Source File | Area BRom | #Feature | #Feature BRom | Start | End | PTM | AScore | Found By |
| --- | --- | --- | --- | --- | --- | --- | --- | --- | --- | --- | --- | --- | --- | --- | --- | --- | --- | --- | --- |
| M.WC(+57.02)DAFC(+57.02)SSR.G | Y | 56.12 | 1187.4489 | 9 | 0.5 | 594.7320 | 2 | 25.21 | 1 | 12147 | 01122020\_RID\_2209\_BuCaKA02.raw | 1.1946E7 | 1 | 1 | 28 | 36 | Carbamidomethylation | C2:Carbamidomethylation:1000.00;C6:Carbamidomethylation:1000.00 | PEAKS DB |
| total 1 peptides |  |  |  |  |  |  |  |  |  |  |  |  |  |  |  |  |  |  |  |
| --- | --- | --- | --- | --- | --- | --- | --- | --- | --- | --- | --- | --- | --- | --- | --- | --- | --- | --- | --- |

CAM11311.1

back to list

  

| Protein Coverage
| Supporting Peptides
|

Protein Coverage:

Supporting Peptides:

| Peptide | Uniq | -10lgP | Mass | Length | ppm | m/z | z | RT | Fraction | Scan | Source File | Area BRom | #Feature | #Feature BRom | Start | End | PTM | AScore | Found By |
| --- | --- | --- | --- | --- | --- | --- | --- | --- | --- | --- | --- | --- | --- | --- | --- | --- | --- | --- | --- |
| M.WC(+57.02)DAFC(+57.02)SSR.G | Y | 56.12 | 1187.4489 | 9 | 0.5 | 594.7320 | 2 | 25.21 | 1 | 12147 | 01122020\_RID\_2209\_BuCaKA02.raw | 1.1946E7 | 1 | 1 | 30 | 38 | Carbamidomethylation | C2:Carbamidomethylation:1000.00;C6:Carbamidomethylation:1000.00 | PEAKS DB |
| total 1 peptides |  |  |  |  |  |  |  |  |  |  |  |  |  |  |  |  |  |  |  |
| --- | --- | --- | --- | --- | --- | --- | --- | --- | --- | --- | --- | --- | --- | --- | --- | --- | --- | --- | --- |

ADN67593.1

back to list

  

| Protein Coverage
| Supporting Peptides
|

Protein Coverage:

Supporting Peptides:

| Peptide | Uniq | -10lgP | Mass | Length | ppm | m/z | z | RT | Fraction | Scan | Source File | Area BRom | #Feature | #Feature BRom | Start | End | PTM | AScore | Found By |
| --- | --- | --- | --- | --- | --- | --- | --- | --- | --- | --- | --- | --- | --- | --- | --- | --- | --- | --- | --- |
| M.WC(+57.02)DAFC(+57.02)SSR.G | Y | 56.12 | 1187.4489 | 9 | 0.5 | 594.7320 | 2 | 25.21 | 1 | 12147 | 01122020\_RID\_2209\_BuCaKA02.raw | 1.1946E7 | 1 | 1 | 30 | 38 | Carbamidomethylation | C2:Carbamidomethylation:1000.00;C6:Carbamidomethylation:1000.00 | PEAKS DB |
| total 1 peptides |  |  |  |  |  |  |  |  |  |  |  |  |  |  |  |  |  |  |  |
| --- | --- | --- | --- | --- | --- | --- | --- | --- | --- | --- | --- | --- | --- | --- | --- | --- | --- | --- | --- |

CAM11305.1

back to list

  

| Protein Coverage
| Supporting Peptides
|

Protein Coverage:

Supporting Peptides:

| Peptide | Uniq | -10lgP | Mass | Length | ppm | m/z | z | RT | Fraction | Scan | Source File | Area BRom | #Feature | #Feature BRom | Start | End | PTM | AScore | Found By |
| --- | --- | --- | --- | --- | --- | --- | --- | --- | --- | --- | --- | --- | --- | --- | --- | --- | --- | --- | --- |
| M.WC(+57.02)DAFC(+57.02)SSR.G | Y | 56.12 | 1187.4489 | 9 | 0.5 | 594.7320 | 2 | 25.21 | 1 | 12147 | 01122020\_RID\_2209\_BuCaKA02.raw | 1.1946E7 | 1 | 1 | 30 | 38 | Carbamidomethylation | C2:Carbamidomethylation:1000.00;C6:Carbamidomethylation:1000.00 | PEAKS DB |
| total 1 peptides |  |  |  |  |  |  |  |  |  |  |  |  |  |  |  |  |  |  |  |
| --- | --- | --- | --- | --- | --- | --- | --- | --- | --- | --- | --- | --- | --- | --- | --- | --- | --- | --- | --- |

CAM11309.1

back to list

  

| Protein Coverage
| Supporting Peptides
|

Protein Coverage:

Supporting Peptides:

| Peptide | Uniq | -10lgP | Mass | Length | ppm | m/z | z | RT | Fraction | Scan | Source File | Area BRom | #Feature | #Feature BRom | Start | End | PTM | AScore | Found By |
| --- | --- | --- | --- | --- | --- | --- | --- | --- | --- | --- | --- | --- | --- | --- | --- | --- | --- | --- | --- |
| M.WC(+57.02)DAFC(+57.02)SSR.G | Y | 56.12 | 1187.4489 | 9 | 0.5 | 594.7320 | 2 | 25.21 | 1 | 12147 | 01122020\_RID\_2209\_BuCaKA02.raw | 1.1946E7 | 1 | 1 | 30 | 38 | Carbamidomethylation | C2:Carbamidomethylation:1000.00;C6:Carbamidomethylation:1000.00 | PEAKS DB |
| total 1 peptides |  |  |  |  |  |  |  |  |  |  |  |  |  |  |  |  |  |  |  |
| --- | --- | --- | --- | --- | --- | --- | --- | --- | --- | --- | --- | --- | --- | --- | --- | --- | --- | --- | --- |

CAD92407.1

back to list

  

| Protein Coverage
| Supporting Peptides
|

Protein Coverage:

Supporting Peptides:

| Peptide | Uniq | -10lgP | Mass | Length | ppm | m/z | z | RT | Fraction | Scan | Source File | Area BRom | #Feature | #Feature BRom | Start | End | PTM | AScore | Found By |
| --- | --- | --- | --- | --- | --- | --- | --- | --- | --- | --- | --- | --- | --- | --- | --- | --- | --- | --- | --- |
| M.WC(+57.02)DAFC(+57.02)SSR.G | Y | 56.12 | 1187.4489 | 9 | 0.5 | 594.7320 | 2 | 25.21 | 1 | 12147 | 01122020\_RID\_2209\_BuCaKA02.raw | 1.1946E7 | 1 | 1 | 30 | 38 | Carbamidomethylation | C2:Carbamidomethylation:1000.00;C6:Carbamidomethylation:1000.00 | PEAKS DB |
| total 1 peptides |  |  |  |  |  |  |  |  |  |  |  |  |  |  |  |  |  |  |  |
| --- | --- | --- | --- | --- | --- | --- | --- | --- | --- | --- | --- | --- | --- | --- | --- | --- | --- | --- | --- |

CAM11312.1

back to list

  

| Protein Coverage
| Supporting Peptides
|

Protein Coverage:

Supporting Peptides:

| Peptide | Uniq | -10lgP | Mass | Length | ppm | m/z | z | RT | Fraction | Scan | Source File | Area BRom | #Feature | #Feature BRom | Start | End | PTM | AScore | Found By |
| --- | --- | --- | --- | --- | --- | --- | --- | --- | --- | --- | --- | --- | --- | --- | --- | --- | --- | --- | --- |
| M.WC(+57.02)DAFC(+57.02)SSR.G | Y | 56.12 | 1187.4489 | 9 | 0.5 | 594.7320 | 2 | 25.21 | 1 | 12147 | 01122020\_RID\_2209\_BuCaKA02.raw | 1.1946E7 | 1 | 1 | 30 | 38 | Carbamidomethylation | C2:Carbamidomethylation:1000.00;C6:Carbamidomethylation:1000.00 | PEAKS DB |
| total 1 peptides |  |  |  |  |  |  |  |  |  |  |  |  |  |  |  |  |  |  |  |
| --- | --- | --- | --- | --- | --- | --- | --- | --- | --- | --- | --- | --- | --- | --- | --- | --- | --- | --- | --- |

CAM11307.1

back to list

  

| Protein Coverage
| Supporting Peptides
|

Protein Coverage:

Supporting Peptides:

| Peptide | Uniq | -10lgP | Mass | Length | ppm | m/z | z | RT | Fraction | Scan | Source File | Area BRom | #Feature | #Feature BRom | Start | End | PTM | AScore | Found By |
| --- | --- | --- | --- | --- | --- | --- | --- | --- | --- | --- | --- | --- | --- | --- | --- | --- | --- | --- | --- |
| M.WC(+57.02)DAFC(+57.02)SSR.G | Y | 56.12 | 1187.4489 | 9 | 0.5 | 594.7320 | 2 | 25.21 | 1 | 12147 | 01122020\_RID\_2209\_BuCaKA02.raw | 1.1946E7 | 1 | 1 | 30 | 38 | Carbamidomethylation | C2:Carbamidomethylation:1000.00;C6:Carbamidomethylation:1000.00 | PEAKS DB |
| total 1 peptides |  |  |  |  |  |  |  |  |  |  |  |  |  |  |  |  |  |  |  |
| --- | --- | --- | --- | --- | --- | --- | --- | --- | --- | --- | --- | --- | --- | --- | --- | --- | --- | --- | --- |

CAM11310.1

back to list

  

| Protein Coverage
| Supporting Peptides
|

Protein Coverage:

Supporting Peptides:

| Peptide | Uniq | -10lgP | Mass | Length | ppm | m/z | z | RT | Fraction | Scan | Source File | Area BRom | #Feature | #Feature BRom | Start | End | PTM | AScore | Found By |
| --- | --- | --- | --- | --- | --- | --- | --- | --- | --- | --- | --- | --- | --- | --- | --- | --- | --- | --- | --- |
| M.WC(+57.02)DAFC(+57.02)SSR.G | Y | 56.12 | 1187.4489 | 9 | 0.5 | 594.7320 | 2 | 25.21 | 1 | 12147 | 01122020\_RID\_2209\_BuCaKA02.raw | 1.1946E7 | 1 | 1 | 30 | 38 | Carbamidomethylation | C2:Carbamidomethylation:1000.00;C6:Carbamidomethylation:1000.00 | PEAKS DB |
| total 1 peptides |  |  |  |  |  |  |  |  |  |  |  |  |  |  |  |  |  |  |  |
| --- | --- | --- | --- | --- | --- | --- | --- | --- | --- | --- | --- | --- | --- | --- | --- | --- | --- | --- | --- |

CAM11304.1

back to list

  

| Protein Coverage
| Supporting Peptides
|

Protein Coverage:

Supporting Peptides:

| Peptide | Uniq | -10lgP | Mass | Length | ppm | m/z | z | RT | Fraction | Scan | Source File | Area BRom | #Feature | #Feature BRom | Start | End | PTM | AScore | Found By |
| --- | --- | --- | --- | --- | --- | --- | --- | --- | --- | --- | --- | --- | --- | --- | --- | --- | --- | --- | --- |
| M.WC(+57.02)DAFC(+57.02)SSR.G | Y | 56.12 | 1187.4489 | 9 | 0.5 | 594.7320 | 2 | 25.21 | 1 | 12147 | 01122020\_RID\_2209\_BuCaKA02.raw | 1.1946E7 | 1 | 1 | 30 | 38 | Carbamidomethylation | C2:Carbamidomethylation:1000.00;C6:Carbamidomethylation:1000.00 | PEAKS DB |
| total 1 peptides |  |  |  |  |  |  |  |  |  |  |  |  |  |  |  |  |  |  |  |
| --- | --- | --- | --- | --- | --- | --- | --- | --- | --- | --- | --- | --- | --- | --- | --- | --- | --- | --- | --- |

CAM11302.1

back to list

  

| Protein Coverage
| Supporting Peptides
|

Protein Coverage:

Supporting Peptides:

| Peptide | Uniq | -10lgP | Mass | Length | ppm | m/z | z | RT | Fraction | Scan | Source File | Area BRom | #Feature | #Feature BRom | Start | End | PTM | AScore | Found By |
| --- | --- | --- | --- | --- | --- | --- | --- | --- | --- | --- | --- | --- | --- | --- | --- | --- | --- | --- | --- |
| M.WC(+57.02)DAFC(+57.02)SSR.G | Y | 56.12 | 1187.4489 | 9 | 0.5 | 594.7320 | 2 | 25.21 | 1 | 12147 | 01122020\_RID\_2209\_BuCaKA02.raw | 1.1946E7 | 1 | 1 | 30 | 38 | Carbamidomethylation | C2:Carbamidomethylation:1000.00;C6:Carbamidomethylation:1000.00 | PEAKS DB |
| total 1 peptides |  |  |  |  |  |  |  |  |  |  |  |  |  |  |  |  |  |  |  |
| --- | --- | --- | --- | --- | --- | --- | --- | --- | --- | --- | --- | --- | --- | --- | --- | --- | --- | --- | --- |

D2N116.1

back to list

  

| Protein Coverage
| Supporting Peptides
|

Protein Coverage:

Supporting Peptides:

| Peptide | Uniq | -10lgP | Mass | Length | ppm | m/z | z | RT | Fraction | Scan | Source File | Area BRom | #Feature | #Feature BRom | Start | End | PTM | AScore | Found By |
| --- | --- | --- | --- | --- | --- | --- | --- | --- | --- | --- | --- | --- | --- | --- | --- | --- | --- | --- | --- |
| M.WC(+57.02)DAFC(+57.02)SSR.G | Y | 56.12 | 1187.4489 | 9 | 0.5 | 594.7320 | 2 | 25.21 | 1 | 12147 | 01122020\_RID\_2209\_BuCaKA02.raw | 1.1946E7 | 1 | 1 | 30 | 38 | Carbamidomethylation | C2:Carbamidomethylation:1000.00;C6:Carbamidomethylation:1000.00 | PEAKS DB |
| total 1 peptides |  |  |  |  |  |  |  |  |  |  |  |  |  |  |  |  |  |  |  |
| --- | --- | --- | --- | --- | --- | --- | --- | --- | --- | --- | --- | --- | --- | --- | --- | --- | --- | --- | --- |

CAM11303.1

back to list

  

| Protein Coverage
| Supporting Peptides
|

Protein Coverage:

Supporting Peptides:

| Peptide | Uniq | -10lgP | Mass | Length | ppm | m/z | z | RT | Fraction | Scan | Source File | Area BRom | #Feature | #Feature BRom | Start | End | PTM | AScore | Found By |
| --- | --- | --- | --- | --- | --- | --- | --- | --- | --- | --- | --- | --- | --- | --- | --- | --- | --- | --- | --- |
| M.WC(+57.02)DAFC(+57.02)SSR.G | Y | 56.12 | 1187.4489 | 9 | 0.5 | 594.7320 | 2 | 25.21 | 1 | 12147 | 01122020\_RID\_2209\_BuCaKA02.raw | 1.1946E7 | 1 | 1 | 30 | 38 | Carbamidomethylation | C2:Carbamidomethylation:1000.00;C6:Carbamidomethylation:1000.00 | PEAKS DB |
| total 1 peptides |  |  |  |  |  |  |  |  |  |  |  |  |  |  |  |  |  |  |  |
| --- | --- | --- | --- | --- | --- | --- | --- | --- | --- | --- | --- | --- | --- | --- | --- | --- | --- | --- | --- |

CAM11306.1

back to list

  

| Protein Coverage
| Supporting Peptides
|

Protein Coverage:

Supporting Peptides:

| Peptide | Uniq | -10lgP | Mass | Length | ppm | m/z | z | RT | Fraction | Scan | Source File | Area BRom | #Feature | #Feature BRom | Start | End | PTM | AScore | Found By |
| --- | --- | --- | --- | --- | --- | --- | --- | --- | --- | --- | --- | --- | --- | --- | --- | --- | --- | --- | --- |
| M.WC(+57.02)DAFC(+57.02)SSR.G | Y | 56.12 | 1187.4489 | 9 | 0.5 | 594.7320 | 2 | 25.21 | 1 | 12147 | 01122020\_RID\_2209\_BuCaKA02.raw | 1.1946E7 | 1 | 1 | 30 | 38 | Carbamidomethylation | C2:Carbamidomethylation:1000.00;C6:Carbamidomethylation:1000.00 | PEAKS DB |
| total 1 peptides |  |  |  |  |  |  |  |  |  |  |  |  |  |  |  |  |  |  |  |
| --- | --- | --- | --- | --- | --- | --- | --- | --- | --- | --- | --- | --- | --- | --- | --- | --- | --- | --- | --- |

Q8UW29.1

back to list

  

| Protein Coverage
| Supporting Peptides
|

Protein Coverage:

Supporting Peptides:

| Peptide | Uniq | -10lgP | Mass | Length | ppm | m/z | z | RT | Fraction | Scan | Source File | Area BRom | #Feature | #Feature BRom | Start | End | PTM | AScore | Found By |
| --- | --- | --- | --- | --- | --- | --- | --- | --- | --- | --- | --- | --- | --- | --- | --- | --- | --- | --- | --- |
| S.WC(+57.02)DAFC(+57.02)SSR.G | Y | 56.12 | 1187.4489 | 9 | 0.5 | 594.7320 | 2 | 25.21 | 1 | 12147 | 01122020\_RID\_2209\_BuCaKA02.raw | 1.1946E7 | 1 | 1 | 46 | 54 | Carbamidomethylation | C2:Carbamidomethylation:1000.00;C6:Carbamidomethylation:1000.00 | PEAKS DB |
| total 1 peptides |  |  |  |  |  |  |  |  |  |  |  |  |  |  |  |  |  |  |  |
| --- | --- | --- | --- | --- | --- | --- | --- | --- | --- | --- | --- | --- | --- | --- | --- | --- | --- | --- | --- |

AAL54892.1

back to list

  

| Protein Coverage
| Supporting Peptides
|

Protein Coverage:

Supporting Peptides:

| Peptide | Uniq | -10lgP | Mass | Length | ppm | m/z | z | RT | Fraction | Scan | Source File | Area BRom | #Feature | #Feature BRom | Start | End | PTM | AScore | Found By |
| --- | --- | --- | --- | --- | --- | --- | --- | --- | --- | --- | --- | --- | --- | --- | --- | --- | --- | --- | --- |
| S.WC(+57.02)DAFC(+57.02)SSR.G | Y | 56.12 | 1187.4489 | 9 | 0.5 | 594.7320 | 2 | 25.21 | 1 | 12147 | 01122020\_RID\_2209\_BuCaKA02.raw | 1.1946E7 | 1 | 1 | 46 | 54 | Carbamidomethylation | C2:Carbamidomethylation:1000.00;C6:Carbamidomethylation:1000.00 | PEAKS DB |
| total 1 peptides |  |  |  |  |  |  |  |  |  |  |  |  |  |  |  |  |  |  |  |
| --- | --- | --- | --- | --- | --- | --- | --- | --- | --- | --- | --- | --- | --- | --- | --- | --- | --- | --- | --- |

A3FM53.1

back to list

  

| Protein Coverage
| Supporting Peptides
|

Protein Coverage:

Supporting Peptides:

| Peptide | Uniq | -10lgP | Mass | Length | ppm | m/z | z | RT | Fraction | Scan | Source File | Area BRom | #Feature | #Feature BRom | Start | End | PTM | AScore | Found By |
| --- | --- | --- | --- | --- | --- | --- | --- | --- | --- | --- | --- | --- | --- | --- | --- | --- | --- | --- | --- |
| S.WC(+57.02)DAFC(+57.02)SSR.G | Y | 56.12 | 1187.4489 | 9 | 0.5 | 594.7320 | 2 | 25.21 | 1 | 12147 | 01122020\_RID\_2209\_BuCaKA02.raw | 1.1946E7 | 1 | 1 | 47 | 55 | Carbamidomethylation | C2:Carbamidomethylation:1000.00;C6:Carbamidomethylation:1000.00 | PEAKS DB |
| total 1 peptides |  |  |  |  |  |  |  |  |  |  |  |  |  |  |  |  |  |  |  |
| --- | --- | --- | --- | --- | --- | --- | --- | --- | --- | --- | --- | --- | --- | --- | --- | --- | --- | --- | --- |

ABN54806.1

back to list

  

| Protein Coverage
| Supporting Peptides
|

Protein Coverage:

Supporting Peptides:

| Peptide | Uniq | -10lgP | Mass | Length | ppm | m/z | z | RT | Fraction | Scan | Source File | Area BRom | #Feature | #Feature BRom | Start | End | PTM | AScore | Found By |
| --- | --- | --- | --- | --- | --- | --- | --- | --- | --- | --- | --- | --- | --- | --- | --- | --- | --- | --- | --- |
| S.WC(+57.02)DAFC(+57.02)SSR.G | Y | 56.12 | 1187.4489 | 9 | 0.5 | 594.7320 | 2 | 25.21 | 1 | 12147 | 01122020\_RID\_2209\_BuCaKA02.raw | 1.1946E7 | 1 | 1 | 47 | 55 | Carbamidomethylation | C2:Carbamidomethylation:1000.00;C6:Carbamidomethylation:1000.00 | PEAKS DB |
| total 1 peptides |  |  |  |  |  |  |  |  |  |  |  |  |  |  |  |  |  |  |  |
| --- | --- | --- | --- | --- | --- | --- | --- | --- | --- | --- | --- | --- | --- | --- | --- | --- | --- | --- | --- |

JAA74770.1

back to list

  

| Protein Coverage
| Supporting Peptides
|

Protein Coverage:

Supporting Peptides:

| Peptide | Uniq | -10lgP | Mass | Length | ppm | m/z | z | RT | Fraction | Scan | Source File | Area BRom | #Feature | #Feature BRom | Start | End | PTM | AScore | Found By |
| --- | --- | --- | --- | --- | --- | --- | --- | --- | --- | --- | --- | --- | --- | --- | --- | --- | --- | --- | --- |
| T.WC(+57.02)DAFC(+57.02)SSR.G | Y | 56.12 | 1187.4489 | 9 | 0.5 | 594.7320 | 2 | 25.21 | 1 | 12147 | 01122020\_RID\_2209\_BuCaKA02.raw | 1.1946E7 | 1 | 1 | 46 | 54 | Carbamidomethylation | C2:Carbamidomethylation:1000.00;C6:Carbamidomethylation:1000.00 | PEAKS DB |
| total 1 peptides |  |  |  |  |  |  |  |  |  |  |  |  |  |  |  |  |  |  |  |
| --- | --- | --- | --- | --- | --- | --- | --- | --- | --- | --- | --- | --- | --- | --- | --- | --- | --- | --- | --- |

P01384.2

back to list

  

| Protein Coverage
| Supporting Peptides
|

Protein Coverage:

Supporting Peptides:

| Peptide | Uniq | -10lgP | Mass | Length | ppm | m/z | z | RT | Fraction | Scan | Source File | Area BRom | #Feature | #Feature BRom | Start | End | PTM | AScore | Found By |
| --- | --- | --- | --- | --- | --- | --- | --- | --- | --- | --- | --- | --- | --- | --- | --- | --- | --- | --- | --- |
| T.WC(+57.02)DAFC(+57.02)SSR.G | Y | 56.12 | 1187.4489 | 9 | 0.5 | 594.7320 | 2 | 25.21 | 1 | 12147 | 01122020\_RID\_2209\_BuCaKA02.raw | 1.1946E7 | 1 | 1 | 46 | 54 | Carbamidomethylation | C2:Carbamidomethylation:1000.00;C6:Carbamidomethylation:1000.00 | PEAKS DB |
| total 1 peptides |  |  |  |  |  |  |  |  |  |  |  |  |  |  |  |  |  |  |  |
| --- | --- | --- | --- | --- | --- | --- | --- | --- | --- | --- | --- | --- | --- | --- | --- | --- | --- | --- | --- |

ABK63537.1

back to list

  

| Protein Coverage
| Supporting Peptides
|

Protein Coverage:

Supporting Peptides:

| Peptide | Uniq | -10lgP | Mass | Length | ppm | m/z | z | RT | Fraction | Scan | Source File | Area BRom | #Feature | #Feature BRom | Start | End | PTM | AScore | Found By |
| --- | --- | --- | --- | --- | --- | --- | --- | --- | --- | --- | --- | --- | --- | --- | --- | --- | --- | --- | --- |
| T.WC(+57.02)DAFC(+57.02)SSR.G | Y | 56.12 | 1187.4489 | 9 | 0.5 | 594.7320 | 2 | 25.21 | 1 | 12147 | 01122020\_RID\_2209\_BuCaKA02.raw | 1.1946E7 | 1 | 1 | 46 | 54 | Carbamidomethylation | C2:Carbamidomethylation:1000.00;C6:Carbamidomethylation:1000.00 | PEAKS DB |
| total 1 peptides |  |  |  |  |  |  |  |  |  |  |  |  |  |  |  |  |  |  |  |
| --- | --- | --- | --- | --- | --- | --- | --- | --- | --- | --- | --- | --- | --- | --- | --- | --- | --- | --- | --- |

JAA74654.1

back to list

  

| Protein Coverage
| Supporting Peptides
|

Protein Coverage:

Supporting Peptides:

| Peptide | Uniq | -10lgP | Mass | Length | ppm | m/z | z | RT | Fraction | Scan | Source File | Area BRom | #Feature | #Feature BRom | Start | End | PTM | AScore | Found By |
| --- | --- | --- | --- | --- | --- | --- | --- | --- | --- | --- | --- | --- | --- | --- | --- | --- | --- | --- | --- |
| T.WC(+57.02)DAFC(+57.02)SSR.G | Y | 56.12 | 1187.4489 | 9 | 0.5 | 594.7320 | 2 | 25.21 | 1 | 12147 | 01122020\_RID\_2209\_BuCaKA02.raw | 1.1946E7 | 1 | 1 | 46 | 54 | Carbamidomethylation | C2:Carbamidomethylation:1000.00;C6:Carbamidomethylation:1000.00 | PEAKS DB |
| total 1 peptides |  |  |  |  |  |  |  |  |  |  |  |  |  |  |  |  |  |  |  |
| --- | --- | --- | --- | --- | --- | --- | --- | --- | --- | --- | --- | --- | --- | --- | --- | --- | --- | --- | --- |

AAC83981.1

back to list

  

| Protein Coverage
| Supporting Peptides
|

Protein Coverage:

Supporting Peptides:

| Peptide | Uniq | -10lgP | Mass | Length | ppm | m/z | z | RT | Fraction | Scan | Source File | Area BRom | #Feature | #Feature BRom | Start | End | PTM | AScore | Found By |
| --- | --- | --- | --- | --- | --- | --- | --- | --- | --- | --- | --- | --- | --- | --- | --- | --- | --- | --- | --- |
| M.WC(+57.02)DAFC(+57.02)SSR.G | Y | 56.12 | 1187.4489 | 9 | 0.5 | 594.7320 | 2 | 25.21 | 1 | 12147 | 01122020\_RID\_2209\_BuCaKA02.raw | 1.1946E7 | 1 | 1 | 49 | 57 | Carbamidomethylation | C2:Carbamidomethylation:1000.00;C6:Carbamidomethylation:1000.00 | PEAKS DB |
| total 1 peptides |  |  |  |  |  |  |  |  |  |  |  |  |  |  |  |  |  |  |  |
| --- | --- | --- | --- | --- | --- | --- | --- | --- | --- | --- | --- | --- | --- | --- | --- | --- | --- | --- | --- |

AAL30056.1

back to list

  

| Protein Coverage
| Supporting Peptides
|

Protein Coverage:

Supporting Peptides:

| Peptide | Uniq | -10lgP | Mass | Length | ppm | m/z | z | RT | Fraction | Scan | Source File | Area BRom | #Feature | #Feature BRom | Start | End | PTM | AScore | Found By |
| --- | --- | --- | --- | --- | --- | --- | --- | --- | --- | --- | --- | --- | --- | --- | --- | --- | --- | --- | --- |
| M.WC(+57.02)DAFC(+57.02)SSR.G | Y | 56.12 | 1187.4489 | 9 | 0.5 | 594.7320 | 2 | 25.21 | 1 | 12147 | 01122020\_RID\_2209\_BuCaKA02.raw | 1.1946E7 | 1 | 1 | 49 | 57 | Carbamidomethylation | C2:Carbamidomethylation:1000.00;C6:Carbamidomethylation:1000.00 | PEAKS DB |
| total 1 peptides |  |  |  |  |  |  |  |  |  |  |  |  |  |  |  |  |  |  |  |
| --- | --- | --- | --- | --- | --- | --- | --- | --- | --- | --- | --- | --- | --- | --- | --- | --- | --- | --- | --- |

LAA32179.1

back to list

  

| Protein Coverage
| Supporting Peptides
|

Protein Coverage:

Supporting Peptides:

| Peptide | Uniq | -10lgP | Mass | Length | ppm | m/z | z | RT | Fraction | Scan | Source File | Area BRom | #Feature | #Feature BRom | Start | End | PTM | AScore | Found By |
| --- | --- | --- | --- | --- | --- | --- | --- | --- | --- | --- | --- | --- | --- | --- | --- | --- | --- | --- | --- |
| R.GIPDSIASVLNKL.V | Y | 55.14 | 1325.7554 | 13 | 1.6 | 663.8860 | 2 | 85.74 | 1 | 60595 | 01122020\_RID\_2209\_BuCaKA02.raw | 3.3012E5 | 1 | 1 | 82 | 94 |  |  | PEAKS DB |
| total 1 peptides |  |  |  |  |  |  |  |  |  |  |  |  |  |  |  |  |  |  |  |
| --- | --- | --- | --- | --- | --- | --- | --- | --- | --- | --- | --- | --- | --- | --- | --- | --- | --- | --- | --- |

XP\_026524224.1

back to list

  

| Protein Coverage
| Supporting Peptides
|

Protein Coverage:

Supporting Peptides:

| Peptide | Uniq | -10lgP | Mass | Length | ppm | m/z | z | RT | Fraction | Scan | Source File | Area BRom | #Feature | #Feature BRom | Start | End | PTM | AScore | Found By |
| --- | --- | --- | --- | --- | --- | --- | --- | --- | --- | --- | --- | --- | --- | --- | --- | --- | --- | --- | --- |
| R.GIPDSIASVLNKL.V | Y | 55.14 | 1325.7554 | 13 | 1.6 | 663.8860 | 2 | 85.74 | 1 | 60595 | 01122020\_RID\_2209\_BuCaKA02.raw | 3.3012E5 | 1 | 1 | 85 | 97 |  |  | PEAKS DB |
| total 1 peptides |  |  |  |  |  |  |  |  |  |  |  |  |  |  |  |  |  |  |  |
| --- | --- | --- | --- | --- | --- | --- | --- | --- | --- | --- | --- | --- | --- | --- | --- | --- | --- | --- | --- |

XP\_026568672.1

back to list

  

| Protein Coverage
| Supporting Peptides
|

Protein Coverage:

Supporting Peptides:

| Peptide | Uniq | -10lgP | Mass | Length | ppm | m/z | z | RT | Fraction | Scan | Source File | Area BRom | #Feature | #Feature BRom | Start | End | PTM | AScore | Found By |
| --- | --- | --- | --- | --- | --- | --- | --- | --- | --- | --- | --- | --- | --- | --- | --- | --- | --- | --- | --- |
| R.GIPDSIASVLNKL.V | Y | 55.14 | 1325.7554 | 13 | 1.6 | 663.8860 | 2 | 85.74 | 1 | 60595 | 01122020\_RID\_2209\_BuCaKA02.raw | 3.3012E5 | 1 | 1 | 85 | 97 |  |  | PEAKS DB |
| total 1 peptides |  |  |  |  |  |  |  |  |  |  |  |  |  |  |  |  |  |  |  |
| --- | --- | --- | --- | --- | --- | --- | --- | --- | --- | --- | --- | --- | --- | --- | --- | --- | --- | --- | --- |

XP\_026524225.1

back to list

  

| Protein Coverage
| Supporting Peptides
|

Protein Coverage:

Supporting Peptides:

| Peptide | Uniq | -10lgP | Mass | Length | ppm | m/z | z | RT | Fraction | Scan | Source File | Area BRom | #Feature | #Feature BRom | Start | End | PTM | AScore | Found By |
| --- | --- | --- | --- | --- | --- | --- | --- | --- | --- | --- | --- | --- | --- | --- | --- | --- | --- | --- | --- |
| R.GIPDSIASVLNKL.V | Y | 55.14 | 1325.7554 | 13 | 1.6 | 663.8860 | 2 | 85.74 | 1 | 60595 | 01122020\_RID\_2209\_BuCaKA02.raw | 3.3012E5 | 1 | 1 | 85 | 97 |  |  | PEAKS DB |
| total 1 peptides |  |  |  |  |  |  |  |  |  |  |  |  |  |  |  |  |  |  |  |
| --- | --- | --- | --- | --- | --- | --- | --- | --- | --- | --- | --- | --- | --- | --- | --- | --- | --- | --- | --- |

XP\_026568674.1

back to list

  

| Protein Coverage
| Supporting Peptides
|

Protein Coverage:

Supporting Peptides:

| Peptide | Uniq | -10lgP | Mass | Length | ppm | m/z | z | RT | Fraction | Scan | Source File | Area BRom | #Feature | #Feature BRom | Start | End | PTM | AScore | Found By |
| --- | --- | --- | --- | --- | --- | --- | --- | --- | --- | --- | --- | --- | --- | --- | --- | --- | --- | --- | --- |
| R.GIPDSIASVLNKL.V | Y | 55.14 | 1325.7554 | 13 | 1.6 | 663.8860 | 2 | 85.74 | 1 | 60595 | 01122020\_RID\_2209\_BuCaKA02.raw | 3.3012E5 | 1 | 1 | 85 | 97 |  |  | PEAKS DB |
| total 1 peptides |  |  |  |  |  |  |  |  |  |  |  |  |  |  |  |  |  |  |  |
| --- | --- | --- | --- | --- | --- | --- | --- | --- | --- | --- | --- | --- | --- | --- | --- | --- | --- | --- | --- |

XP\_015667825.1

back to list

  

| Protein Coverage
| Supporting Peptides
|

Protein Coverage:

Supporting Peptides:

| Peptide | Uniq | -10lgP | Mass | Length | ppm | m/z | z | RT | Fraction | Scan | Source File | Area BRom | #Feature | #Feature BRom | Start | End | PTM | AScore | Found By |
| --- | --- | --- | --- | --- | --- | --- | --- | --- | --- | --- | --- | --- | --- | --- | --- | --- | --- | --- | --- |
| R.GIPDSIASVLNKL.V | Y | 55.14 | 1325.7554 | 13 | 1.6 | 663.8860 | 2 | 85.74 | 1 | 60595 | 01122020\_RID\_2209\_BuCaKA02.raw | 3.3012E5 | 1 | 1 | 85 | 97 |  |  | PEAKS DB |
| total 1 peptides |  |  |  |  |  |  |  |  |  |  |  |  |  |  |  |  |  |  |  |
| --- | --- | --- | --- | --- | --- | --- | --- | --- | --- | --- | --- | --- | --- | --- | --- | --- | --- | --- | --- |

XP\_026524226.1

back to list

  

| Protein Coverage
| Supporting Peptides
|

Protein Coverage:

Supporting Peptides:

| Peptide | Uniq | -10lgP | Mass | Length | ppm | m/z | z | RT | Fraction | Scan | Source File | Area BRom | #Feature | #Feature BRom | Start | End | PTM | AScore | Found By |
| --- | --- | --- | --- | --- | --- | --- | --- | --- | --- | --- | --- | --- | --- | --- | --- | --- | --- | --- | --- |
| R.GIPDSIASVLNKL.V | Y | 55.14 | 1325.7554 | 13 | 1.6 | 663.8860 | 2 | 85.74 | 1 | 60595 | 01122020\_RID\_2209\_BuCaKA02.raw | 3.3012E5 | 1 | 1 | 85 | 97 |  |  | PEAKS DB |
| total 1 peptides |  |  |  |  |  |  |  |  |  |  |  |  |  |  |  |  |  |  |  |
| --- | --- | --- | --- | --- | --- | --- | --- | --- | --- | --- | --- | --- | --- | --- | --- | --- | --- | --- | --- |

XP\_034288350.1

back to list

  

| Protein Coverage
| Supporting Peptides
|

Protein Coverage:

Supporting Peptides:

| Peptide | Uniq | -10lgP | Mass | Length | ppm | m/z | z | RT | Fraction | Scan | Source File | Area BRom | #Feature | #Feature BRom | Start | End | PTM | AScore | Found By |
| --- | --- | --- | --- | --- | --- | --- | --- | --- | --- | --- | --- | --- | --- | --- | --- | --- | --- | --- | --- |
| R.GLPDSIASVLNKL.V | Y | 55.14 | 1325.7554 | 13 | 1.6 | 663.8860 | 2 | 85.74 | 1 | 60595 | 01122020\_RID\_2209\_BuCaKA02.raw | 3.3012E5 | 1 | 1 | 85 | 97 |  |  | PEAKS DB |
| total 1 peptides |  |  |  |  |  |  |  |  |  |  |  |  |  |  |  |  |  |  |  |
| --- | --- | --- | --- | --- | --- | --- | --- | --- | --- | --- | --- | --- | --- | --- | --- | --- | --- | --- | --- |

XP\_007435816.1

back to list

  

| Protein Coverage
| Supporting Peptides
|

Protein Coverage:

Supporting Peptides:

| Peptide | Uniq | -10lgP | Mass | Length | ppm | m/z | z | RT | Fraction | Scan | Source File | Area BRom | #Feature | #Feature BRom | Start | End | PTM | AScore | Found By |
| --- | --- | --- | --- | --- | --- | --- | --- | --- | --- | --- | --- | --- | --- | --- | --- | --- | --- | --- | --- |
| R.GLPDSIASVLNKL.V | Y | 55.14 | 1325.7554 | 13 | 1.6 | 663.8860 | 2 | 85.74 | 1 | 60595 | 01122020\_RID\_2209\_BuCaKA02.raw | 3.3012E5 | 1 | 1 | 85 | 97 |  |  | PEAKS DB |
| total 1 peptides |  |  |  |  |  |  |  |  |  |  |  |  |  |  |  |  |  |  |  |
| --- | --- | --- | --- | --- | --- | --- | --- | --- | --- | --- | --- | --- | --- | --- | --- | --- | --- | --- | --- |

XP\_026568671.1

back to list

  

| Protein Coverage
| Supporting Peptides
|

Protein Coverage:

Supporting Peptides:

| Peptide | Uniq | -10lgP | Mass | Length | ppm | m/z | z | RT | Fraction | Scan | Source File | Area BRom | #Feature | #Feature BRom | Start | End | PTM | AScore | Found By |
| --- | --- | --- | --- | --- | --- | --- | --- | --- | --- | --- | --- | --- | --- | --- | --- | --- | --- | --- | --- |
| R.GIPDSIASVLNKL.V | Y | 55.14 | 1325.7554 | 13 | 1.6 | 663.8860 | 2 | 85.74 | 1 | 60595 | 01122020\_RID\_2209\_BuCaKA02.raw | 3.3012E5 | 1 | 1 | 85 | 97 |  |  | PEAKS DB |
| total 1 peptides |  |  |  |  |  |  |  |  |  |  |  |  |  |  |  |  |  |  |  |
| --- | --- | --- | --- | --- | --- | --- | --- | --- | --- | --- | --- | --- | --- | --- | --- | --- | --- | --- | --- |

XP\_026568673.1

back to list

  

| Protein Coverage
| Supporting Peptides
|

Protein Coverage:

Supporting Peptides:

| Peptide | Uniq | -10lgP | Mass | Length | ppm | m/z | z | RT | Fraction | Scan | Source File | Area BRom | #Feature | #Feature BRom | Start | End | PTM | AScore | Found By |
| --- | --- | --- | --- | --- | --- | --- | --- | --- | --- | --- | --- | --- | --- | --- | --- | --- | --- | --- | --- |
| R.GIPDSIASVLNKL.V | Y | 55.14 | 1325.7554 | 13 | 1.6 | 663.8860 | 2 | 85.74 | 1 | 60595 | 01122020\_RID\_2209\_BuCaKA02.raw | 3.3012E5 | 1 | 1 | 85 | 97 |  |  | PEAKS DB |
| total 1 peptides |  |  |  |  |  |  |  |  |  |  |  |  |  |  |  |  |  |  |  |
| --- | --- | --- | --- | --- | --- | --- | --- | --- | --- | --- | --- | --- | --- | --- | --- | --- | --- | --- | --- |

XP\_026524223.1

back to list

  

| Protein Coverage
| Supporting Peptides
|

Protein Coverage:

Supporting Peptides:

| Peptide | Uniq | -10lgP | Mass | Length | ppm | m/z | z | RT | Fraction | Scan | Source File | Area BRom | #Feature | #Feature BRom | Start | End | PTM | AScore | Found By |
| --- | --- | --- | --- | --- | --- | --- | --- | --- | --- | --- | --- | --- | --- | --- | --- | --- | --- | --- | --- |
| R.GIPDSIASVLNKL.V | Y | 55.14 | 1325.7554 | 13 | 1.6 | 663.8860 | 2 | 85.74 | 1 | 60595 | 01122020\_RID\_2209\_BuCaKA02.raw | 3.3012E5 | 1 | 1 | 85 | 97 |  |  | PEAKS DB |
| total 1 peptides |  |  |  |  |  |  |  |  |  |  |  |  |  |  |  |  |  |  |  |
| --- | --- | --- | --- | --- | --- | --- | --- | --- | --- | --- | --- | --- | --- | --- | --- | --- | --- | --- | --- |

ETE70626.1

back to list

  

| Protein Coverage
| Supporting Peptides
|

Protein Coverage:

Supporting Peptides:

| Peptide | Uniq | -10lgP | Mass | Length | ppm | m/z | z | RT | Fraction | Scan | Source File | Area BRom | #Feature | #Feature BRom | Start | End | PTM | AScore | Found By |
| --- | --- | --- | --- | --- | --- | --- | --- | --- | --- | --- | --- | --- | --- | --- | --- | --- | --- | --- | --- |
| R.GIPDSIASVLNKL.V | Y | 55.14 | 1325.7554 | 13 | 1.6 | 663.8860 | 2 | 85.74 | 1 | 60595 | 01122020\_RID\_2209\_BuCaKA02.raw | 3.3012E5 | 1 | 1 | 96 | 108 |  |  | PEAKS DB |
| total 1 peptides |  |  |  |  |  |  |  |  |  |  |  |  |  |  |  |  |  |  |  |
| --- | --- | --- | --- | --- | --- | --- | --- | --- | --- | --- | --- | --- | --- | --- | --- | --- | --- | --- | --- |

LAB55207.1

back to list

  

| Protein Coverage
| Supporting Peptides
|

Protein Coverage:

Supporting Peptides:

| Peptide | Uniq | -10lgP | Mass | Length | ppm | m/z | z | RT | Fraction | Scan | Source File | Area BRom | #Feature | #Feature BRom | Start | End | PTM | AScore | Found By |
| --- | --- | --- | --- | --- | --- | --- | --- | --- | --- | --- | --- | --- | --- | --- | --- | --- | --- | --- | --- |
| R.GIPDSIASVLNKL.V | Y | 55.14 | 1325.7554 | 13 | 1.6 | 663.8860 | 2 | 85.74 | 1 | 60595 | 01122020\_RID\_2209\_BuCaKA02.raw | 3.3012E5 | 1 | 1 | 95 | 107 |  |  | PEAKS DB |
| total 1 peptides |  |  |  |  |  |  |  |  |  |  |  |  |  |  |  |  |  |  |  |
| --- | --- | --- | --- | --- | --- | --- | --- | --- | --- | --- | --- | --- | --- | --- | --- | --- | --- | --- | --- |

LAA32175.1

back to list

  

| Protein Coverage
| Supporting Peptides
|

Protein Coverage:

Supporting Peptides:

| Peptide | Uniq | -10lgP | Mass | Length | ppm | m/z | z | RT | Fraction | Scan | Source File | Area BRom | #Feature | #Feature BRom | Start | End | PTM | AScore | Found By |
| --- | --- | --- | --- | --- | --- | --- | --- | --- | --- | --- | --- | --- | --- | --- | --- | --- | --- | --- | --- |
| R.GIPDSIASVLNKL.V | Y | 55.14 | 1325.7554 | 13 | 1.6 | 663.8860 | 2 | 85.74 | 1 | 60595 | 01122020\_RID\_2209\_BuCaKA02.raw | 3.3012E5 | 1 | 1 | 95 | 107 |  |  | PEAKS DB |
| total 1 peptides |  |  |  |  |  |  |  |  |  |  |  |  |  |  |  |  |  |  |  |
| --- | --- | --- | --- | --- | --- | --- | --- | --- | --- | --- | --- | --- | --- | --- | --- | --- | --- | --- | --- |

JAV48583.1

back to list

  

| Protein Coverage
| Supporting Peptides
|

Protein Coverage:

Supporting Peptides:

| Peptide | Uniq | -10lgP | Mass | Length | ppm | m/z | z | RT | Fraction | Scan | Source File | Area BRom | #Feature | #Feature BRom | Start | End | PTM | AScore | Found By |
| --- | --- | --- | --- | --- | --- | --- | --- | --- | --- | --- | --- | --- | --- | --- | --- | --- | --- | --- | --- |
| R.GIPDSIASVLNKL.V | Y | 55.14 | 1325.7554 | 13 | 1.6 | 663.8860 | 2 | 85.74 | 1 | 60595 | 01122020\_RID\_2209\_BuCaKA02.raw | 3.3012E5 | 1 | 1 | 96 | 108 |  |  | PEAKS DB |
| total 1 peptides |  |  |  |  |  |  |  |  |  |  |  |  |  |  |  |  |  |  |  |
| --- | --- | --- | --- | --- | --- | --- | --- | --- | --- | --- | --- | --- | --- | --- | --- | --- | --- | --- | --- |

XP\_015667816.1

back to list

  

| Protein Coverage
| Supporting Peptides
|

Protein Coverage:

Supporting Peptides:

| Peptide | Uniq | -10lgP | Mass | Length | ppm | m/z | z | RT | Fraction | Scan | Source File | Area BRom | #Feature | #Feature BRom | Start | End | PTM | AScore | Found By |
| --- | --- | --- | --- | --- | --- | --- | --- | --- | --- | --- | --- | --- | --- | --- | --- | --- | --- | --- | --- |
| R.GIPDSIASVLNKL.V | Y | 55.14 | 1325.7554 | 13 | 1.6 | 663.8860 | 2 | 85.74 | 1 | 60595 | 01122020\_RID\_2209\_BuCaKA02.raw | 3.3012E5 | 1 | 1 | 96 | 108 |  |  | PEAKS DB |
| total 1 peptides |  |  |  |  |  |  |  |  |  |  |  |  |  |  |  |  |  |  |  |
| --- | --- | --- | --- | --- | --- | --- | --- | --- | --- | --- | --- | --- | --- | --- | --- | --- | --- | --- | --- |

XP\_034288348.1

back to list

  

| Protein Coverage
| Supporting Peptides
|

Protein Coverage:

Supporting Peptides:

| Peptide | Uniq | -10lgP | Mass | Length | ppm | m/z | z | RT | Fraction | Scan | Source File | Area BRom | #Feature | #Feature BRom | Start | End | PTM | AScore | Found By |
| --- | --- | --- | --- | --- | --- | --- | --- | --- | --- | --- | --- | --- | --- | --- | --- | --- | --- | --- | --- |
| R.GLPDSIASVLNKL.V | Y | 55.14 | 1325.7554 | 13 | 1.6 | 663.8860 | 2 | 85.74 | 1 | 60595 | 01122020\_RID\_2209\_BuCaKA02.raw | 3.3012E5 | 1 | 1 | 96 | 108 |  |  | PEAKS DB |
| total 1 peptides |  |  |  |  |  |  |  |  |  |  |  |  |  |  |  |  |  |  |  |
| --- | --- | --- | --- | --- | --- | --- | --- | --- | --- | --- | --- | --- | --- | --- | --- | --- | --- | --- | --- |

XP\_007435815.1

back to list

  

| Protein Coverage
| Supporting Peptides
|

Protein Coverage:

Supporting Peptides:

| Peptide | Uniq | -10lgP | Mass | Length | ppm | m/z | z | RT | Fraction | Scan | Source File | Area BRom | #Feature | #Feature BRom | Start | End | PTM | AScore | Found By |
| --- | --- | --- | --- | --- | --- | --- | --- | --- | --- | --- | --- | --- | --- | --- | --- | --- | --- | --- | --- |
| R.GLPDSIASVLNKL.V | Y | 55.14 | 1325.7554 | 13 | 1.6 | 663.8860 | 2 | 85.74 | 1 | 60595 | 01122020\_RID\_2209\_BuCaKA02.raw | 3.3012E5 | 1 | 1 | 96 | 108 |  |  | PEAKS DB |
| total 1 peptides |  |  |  |  |  |  |  |  |  |  |  |  |  |  |  |  |  |  |  |
| --- | --- | --- | --- | --- | --- | --- | --- | --- | --- | --- | --- | --- | --- | --- | --- | --- | --- | --- | --- |

XP\_026524222.1

back to list

  

| Protein Coverage
| Supporting Peptides
|

Protein Coverage:

Supporting Peptides:

| Peptide | Uniq | -10lgP | Mass | Length | ppm | m/z | z | RT | Fraction | Scan | Source File | Area BRom | #Feature | #Feature BRom | Start | End | PTM | AScore | Found By |
| --- | --- | --- | --- | --- | --- | --- | --- | --- | --- | --- | --- | --- | --- | --- | --- | --- | --- | --- | --- |
| R.GIPDSIASVLNKL.V | Y | 55.14 | 1325.7554 | 13 | 1.6 | 663.8860 | 2 | 85.74 | 1 | 60595 | 01122020\_RID\_2209\_BuCaKA02.raw | 3.3012E5 | 1 | 1 | 96 | 108 |  |  | PEAKS DB |
| total 1 peptides |  |  |  |  |  |  |  |  |  |  |  |  |  |  |  |  |  |  |  |
| --- | --- | --- | --- | --- | --- | --- | --- | --- | --- | --- | --- | --- | --- | --- | --- | --- | --- | --- | --- |

XP\_026568670.1

back to list

  

| Protein Coverage
| Supporting Peptides
|

Protein Coverage:

Supporting Peptides:

| Peptide | Uniq | -10lgP | Mass | Length | ppm | m/z | z | RT | Fraction | Scan | Source File | Area BRom | #Feature | #Feature BRom | Start | End | PTM | AScore | Found By |
| --- | --- | --- | --- | --- | --- | --- | --- | --- | --- | --- | --- | --- | --- | --- | --- | --- | --- | --- | --- |
| R.GIPDSIASVLNKL.V | Y | 55.14 | 1325.7554 | 13 | 1.6 | 663.8860 | 2 | 85.74 | 1 | 60595 | 01122020\_RID\_2209\_BuCaKA02.raw | 3.3012E5 | 1 | 1 | 96 | 108 |  |  | PEAKS DB |
| total 1 peptides |  |  |  |  |  |  |  |  |  |  |  |  |  |  |  |  |  |  |  |
| --- | --- | --- | --- | --- | --- | --- | --- | --- | --- | --- | --- | --- | --- | --- | --- | --- | --- | --- | --- |

LAA32177.1

back to list

  

| Protein Coverage
| Supporting Peptides
|

Protein Coverage:

Supporting Peptides:

| Peptide | Uniq | -10lgP | Mass | Length | ppm | m/z | z | RT | Fraction | Scan | Source File | Area BRom | #Feature | #Feature BRom | Start | End | PTM | AScore | Found By |
| --- | --- | --- | --- | --- | --- | --- | --- | --- | --- | --- | --- | --- | --- | --- | --- | --- | --- | --- | --- |
| R.GIPDSIASVLNKL.V | Y | 55.14 | 1325.7554 | 13 | 1.6 | 663.8860 | 2 | 85.74 | 1 | 60595 | 01122020\_RID\_2209\_BuCaKA02.raw | 3.3012E5 | 1 | 1 | 97 | 109 |  |  | PEAKS DB |
| total 1 peptides |  |  |  |  |  |  |  |  |  |  |  |  |  |  |  |  |  |  |  |
| --- | --- | --- | --- | --- | --- | --- | --- | --- | --- | --- | --- | --- | --- | --- | --- | --- | --- | --- | --- |

XP\_025027268.1

back to list

  

| Protein Coverage
| Supporting Peptides
|

Protein Coverage:

Supporting Peptides:

| Peptide | Uniq | -10lgP | Mass | Length | ppm | m/z | z | RT | Fraction | Scan | Source File | Area BRom | #Feature | #Feature BRom | Start | End | PTM | AScore | Found By |
| --- | --- | --- | --- | --- | --- | --- | --- | --- | --- | --- | --- | --- | --- | --- | --- | --- | --- | --- | --- |
| R.GLPDSIASVLNKL.V | Y | 55.14 | 1325.7554 | 13 | 1.6 | 663.8860 | 2 | 85.74 | 1 | 60595 | 01122020\_RID\_2209\_BuCaKA02.raw | 3.3012E5 | 1 | 1 | 124 | 136 |  |  | PEAKS DB |
| total 1 peptides |  |  |  |  |  |  |  |  |  |  |  |  |  |  |  |  |  |  |  |
| --- | --- | --- | --- | --- | --- | --- | --- | --- | --- | --- | --- | --- | --- | --- | --- | --- | --- | --- | --- |

P02010.1

back to list

  

| Protein Coverage
| Supporting Peptides
|

Protein Coverage:

Supporting Peptides:

| Peptide | Uniq | -10lgP | Mass | Length | ppm | m/z | z | RT | Fraction | Scan | Source File | Area BRom | #Feature | #Feature BRom | Start | End | PTM | AScore | Found By |
| --- | --- | --- | --- | --- | --- | --- | --- | --- | --- | --- | --- | --- | --- | --- | --- | --- | --- | --- | --- |
| K.VIDALDNAVEGLDDAVATLSKLSDLHAQK.L | Y | 54.82 | 3020.5664 | 29 | 9.3 | 756.1559 | 4 | 94.00 | 1 | 67406 | 01122020\_RID\_2209\_BuCaKA02.raw | 0 | 0 | 0 | 62 | 90 |  |  | PEAKS DB |
| total 1 peptides |  |  |  |  |  |  |  |  |  |  |  |  |  |  |  |  |  |  |  |
| --- | --- | --- | --- | --- | --- | --- | --- | --- | --- | --- | --- | --- | --- | --- | --- | --- | --- | --- | --- |

XP\_032076462.1

back to list

  

| Protein Coverage
| Supporting Peptides
|

Protein Coverage:

Supporting Peptides:

| Peptide | Uniq | -10lgP | Mass | Length | ppm | m/z | z | RT | Fraction | Scan | Source File | Area BRom | #Feature | #Feature BRom | Start | End | PTM | AScore | Found By |
| --- | --- | --- | --- | --- | --- | --- | --- | --- | --- | --- | --- | --- | --- | --- | --- | --- | --- | --- | --- |
| K.SSTFTLTNIVPQFIK.L | Y | 52.14 | 1694.9243 | 15 | 0.3 | 848.4697 | 2 | 79.49 | 1 | 55463 | 01122020\_RID\_2209\_BuCaKA02.raw | 1.1705E6 | 1 | 1 | 154 | 168 |  |  | PEAKS DB |
| total 1 peptides |  |  |  |  |  |  |  |  |  |  |  |  |  |  |  |  |  |  |  |
| --- | --- | --- | --- | --- | --- | --- | --- | --- | --- | --- | --- | --- | --- | --- | --- | --- | --- | --- | --- |

XP\_032076461.1

back to list

  

| Protein Coverage
| Supporting Peptides
|

Protein Coverage:

Supporting Peptides:

| Peptide | Uniq | -10lgP | Mass | Length | ppm | m/z | z | RT | Fraction | Scan | Source File | Area BRom | #Feature | #Feature BRom | Start | End | PTM | AScore | Found By |
| --- | --- | --- | --- | --- | --- | --- | --- | --- | --- | --- | --- | --- | --- | --- | --- | --- | --- | --- | --- |
| K.SSTFTLTNIVPQFIK.L | Y | 52.14 | 1694.9243 | 15 | 0.3 | 848.4697 | 2 | 79.49 | 1 | 55463 | 01122020\_RID\_2209\_BuCaKA02.raw | 1.1705E6 | 1 | 1 | 183 | 197 |  |  | PEAKS DB |
| total 1 peptides |  |  |  |  |  |  |  |  |  |  |  |  |  |  |  |  |  |  |  |
| --- | --- | --- | --- | --- | --- | --- | --- | --- | --- | --- | --- | --- | --- | --- | --- | --- | --- | --- | --- |

ETE56723.1

back to list

  

| Protein Coverage
| Supporting Peptides
|

Protein Coverage:

Supporting Peptides:

| Peptide | Uniq | -10lgP | Mass | Length | ppm | m/z | z | RT | Fraction | Scan | Source File | Area BRom | #Feature | #Feature BRom | Start | End | PTM | AScore | Found By |
| --- | --- | --- | --- | --- | --- | --- | --- | --- | --- | --- | --- | --- | --- | --- | --- | --- | --- | --- | --- |
| K.SSTFTLTNIVPQFIK.L | Y | 52.14 | 1694.9243 | 15 | 0.3 | 848.4697 | 2 | 79.49 | 1 | 55463 | 01122020\_RID\_2209\_BuCaKA02.raw | 1.1705E6 | 1 | 1 | 51 | 65 |  |  | PEAKS DB |
| total 1 peptides |  |  |  |  |  |  |  |  |  |  |  |  |  |  |  |  |  |  |  |
| --- | --- | --- | --- | --- | --- | --- | --- | --- | --- | --- | --- | --- | --- | --- | --- | --- | --- | --- | --- |

XP\_026546198.1

back to list

  

| Protein Coverage
| Supporting Peptides
|

Protein Coverage:

Supporting Peptides:

| Peptide | Uniq | -10lgP | Mass | Length | ppm | m/z | z | RT | Fraction | Scan | Source File | Area BRom | #Feature | #Feature BRom | Start | End | PTM | AScore | Found By |
| --- | --- | --- | --- | --- | --- | --- | --- | --- | --- | --- | --- | --- | --- | --- | --- | --- | --- | --- | --- |
| K.SSTFTLTNIVPQFIK.L | Y | 52.14 | 1694.9243 | 15 | 0.3 | 848.4697 | 2 | 79.49 | 1 | 55463 | 01122020\_RID\_2209\_BuCaKA02.raw | 1.1705E6 | 1 | 1 | 154 | 168 |  |  | PEAKS DB |
| total 1 peptides |  |  |  |  |  |  |  |  |  |  |  |  |  |  |  |  |  |  |  |
| --- | --- | --- | --- | --- | --- | --- | --- | --- | --- | --- | --- | --- | --- | --- | --- | --- | --- | --- | --- |

XP\_026546199.1

back to list

  

| Protein Coverage
| Supporting Peptides
|

Protein Coverage:

Supporting Peptides:

| Peptide | Uniq | -10lgP | Mass | Length | ppm | m/z | z | RT | Fraction | Scan | Source File | Area BRom | #Feature | #Feature BRom | Start | End | PTM | AScore | Found By |
| --- | --- | --- | --- | --- | --- | --- | --- | --- | --- | --- | --- | --- | --- | --- | --- | --- | --- | --- | --- |
| K.SSTFTLTNIVPQFIK.L | Y | 52.14 | 1694.9243 | 15 | 0.3 | 848.4697 | 2 | 79.49 | 1 | 55463 | 01122020\_RID\_2209\_BuCaKA02.raw | 1.1705E6 | 1 | 1 | 154 | 168 |  |  | PEAKS DB |
| total 1 peptides |  |  |  |  |  |  |  |  |  |  |  |  |  |  |  |  |  |  |  |
| --- | --- | --- | --- | --- | --- | --- | --- | --- | --- | --- | --- | --- | --- | --- | --- | --- | --- | --- | --- |

XP\_026546197.1

back to list

  

| Protein Coverage
| Supporting Peptides
|

Protein Coverage:

Supporting Peptides:

| Peptide | Uniq | -10lgP | Mass | Length | ppm | m/z | z | RT | Fraction | Scan | Source File | Area BRom | #Feature | #Feature BRom | Start | End | PTM | AScore | Found By |
| --- | --- | --- | --- | --- | --- | --- | --- | --- | --- | --- | --- | --- | --- | --- | --- | --- | --- | --- | --- |
| K.SSTFTLTNIVPQFIK.L | Y | 52.14 | 1694.9243 | 15 | 0.3 | 848.4697 | 2 | 79.49 | 1 | 55463 | 01122020\_RID\_2209\_BuCaKA02.raw | 1.1705E6 | 1 | 1 | 154 | 168 |  |  | PEAKS DB |
| total 1 peptides |  |  |  |  |  |  |  |  |  |  |  |  |  |  |  |  |  |  |  |
| --- | --- | --- | --- | --- | --- | --- | --- | --- | --- | --- | --- | --- | --- | --- | --- | --- | --- | --- | --- |

JAB54304.1

back to list

  

| Protein Coverage
| Supporting Peptides
|

Protein Coverage:

Supporting Peptides:

| Peptide | Uniq | -10lgP | Mass | Length | ppm | m/z | z | RT | Fraction | Scan | Source File | Area BRom | #Feature | #Feature BRom | Start | End | PTM | AScore | Found By |
| --- | --- | --- | --- | --- | --- | --- | --- | --- | --- | --- | --- | --- | --- | --- | --- | --- | --- | --- | --- |
| K.SSTFTLTNIVPQFIK.L | Y | 52.14 | 1694.9243 | 15 | 0.3 | 848.4697 | 2 | 79.49 | 1 | 55463 | 01122020\_RID\_2209\_BuCaKA02.raw | 1.1705E6 | 1 | 1 | 154 | 168 |  |  | PEAKS DB |
| total 1 peptides |  |  |  |  |  |  |  |  |  |  |  |  |  |  |  |  |  |  |  |
| --- | --- | --- | --- | --- | --- | --- | --- | --- | --- | --- | --- | --- | --- | --- | --- | --- | --- | --- | --- |

XP\_026579206.1

back to list

  

| Protein Coverage
| Supporting Peptides
|

Protein Coverage:

Supporting Peptides:

| Peptide | Uniq | -10lgP | Mass | Length | ppm | m/z | z | RT | Fraction | Scan | Source File | Area BRom | #Feature | #Feature BRom | Start | End | PTM | AScore | Found By |
| --- | --- | --- | --- | --- | --- | --- | --- | --- | --- | --- | --- | --- | --- | --- | --- | --- | --- | --- | --- |
| K.SSTFTLTNIVPQFIK.L | Y | 52.14 | 1694.9243 | 15 | 0.3 | 848.4697 | 2 | 79.49 | 1 | 55463 | 01122020\_RID\_2209\_BuCaKA02.raw | 1.1705E6 | 1 | 1 | 154 | 168 |  |  | PEAKS DB |
| total 1 peptides |  |  |  |  |  |  |  |  |  |  |  |  |  |  |  |  |  |  |  |
| --- | --- | --- | --- | --- | --- | --- | --- | --- | --- | --- | --- | --- | --- | --- | --- | --- | --- | --- | --- |

XP\_026579205.1

back to list

  

| Protein Coverage
| Supporting Peptides
|

Protein Coverage:

Supporting Peptides:

| Peptide | Uniq | -10lgP | Mass | Length | ppm | m/z | z | RT | Fraction | Scan | Source File | Area BRom | #Feature | #Feature BRom | Start | End | PTM | AScore | Found By |
| --- | --- | --- | --- | --- | --- | --- | --- | --- | --- | --- | --- | --- | --- | --- | --- | --- | --- | --- | --- |
| K.SSTFTLTNIVPQFIK.L | Y | 52.14 | 1694.9243 | 15 | 0.3 | 848.4697 | 2 | 79.49 | 1 | 55463 | 01122020\_RID\_2209\_BuCaKA02.raw | 1.1705E6 | 1 | 1 | 154 | 168 |  |  | PEAKS DB |
| total 1 peptides |  |  |  |  |  |  |  |  |  |  |  |  |  |  |  |  |  |  |  |
| --- | --- | --- | --- | --- | --- | --- | --- | --- | --- | --- | --- | --- | --- | --- | --- | --- | --- | --- | --- |

XP\_026579207.1

back to list

  

| Protein Coverage
| Supporting Peptides
|

Protein Coverage:

Supporting Peptides:

| Peptide | Uniq | -10lgP | Mass | Length | ppm | m/z | z | RT | Fraction | Scan | Source File | Area BRom | #Feature | #Feature BRom | Start | End | PTM | AScore | Found By |
| --- | --- | --- | --- | --- | --- | --- | --- | --- | --- | --- | --- | --- | --- | --- | --- | --- | --- | --- | --- |
| K.SSTFTLTNIVPQFIK.L | Y | 52.14 | 1694.9243 | 15 | 0.3 | 848.4697 | 2 | 79.49 | 1 | 55463 | 01122020\_RID\_2209\_BuCaKA02.raw | 1.1705E6 | 1 | 1 | 154 | 168 |  |  | PEAKS DB |
| total 1 peptides |  |  |  |  |  |  |  |  |  |  |  |  |  |  |  |  |  |  |  |
| --- | --- | --- | --- | --- | --- | --- | --- | --- | --- | --- | --- | --- | --- | --- | --- | --- | --- | --- | --- |

XP\_026546196.1

back to list

  

| Protein Coverage
| Supporting Peptides
|

Protein Coverage:

Supporting Peptides:

| Peptide | Uniq | -10lgP | Mass | Length | ppm | m/z | z | RT | Fraction | Scan | Source File | Area BRom | #Feature | #Feature BRom | Start | End | PTM | AScore | Found By |
| --- | --- | --- | --- | --- | --- | --- | --- | --- | --- | --- | --- | --- | --- | --- | --- | --- | --- | --- | --- |
| K.SSTFTLTNIVPQFIK.L | Y | 52.14 | 1694.9243 | 15 | 0.3 | 848.4697 | 2 | 79.49 | 1 | 55463 | 01122020\_RID\_2209\_BuCaKA02.raw | 1.1705E6 | 1 | 1 | 168 | 182 |  |  | PEAKS DB |
| total 1 peptides |  |  |  |  |  |  |  |  |  |  |  |  |  |  |  |  |  |  |  |
| --- | --- | --- | --- | --- | --- | --- | --- | --- | --- | --- | --- | --- | --- | --- | --- | --- | --- | --- | --- |

ETE59939.1

back to list

  

| Protein Coverage
| Supporting Peptides
|

Protein Coverage:

Supporting Peptides:

| Peptide | Uniq | -10lgP | Mass | Length | ppm | m/z | z | RT | Fraction | Scan | Source File | Area BRom | #Feature | #Feature BRom | Start | End | PTM | AScore | Found By |
| --- | --- | --- | --- | --- | --- | --- | --- | --- | --- | --- | --- | --- | --- | --- | --- | --- | --- | --- | --- |
| K.SSTFTLTNIVPQFIK.L | Y | 52.14 | 1694.9243 | 15 | 0.3 | 848.4697 | 2 | 79.49 | 1 | 55463 | 01122020\_RID\_2209\_BuCaKA02.raw | 1.1705E6 | 1 | 1 | 134 | 148 |  |  | PEAKS DB |
| total 1 peptides |  |  |  |  |  |  |  |  |  |  |  |  |  |  |  |  |  |  |  |
| --- | --- | --- | --- | --- | --- | --- | --- | --- | --- | --- | --- | --- | --- | --- | --- | --- | --- | --- | --- |

LAA72691.1

back to list

  

| Protein Coverage
| Supporting Peptides
|

Protein Coverage:

Supporting Peptides:

| Peptide | Uniq | -10lgP | Mass | Length | ppm | m/z | z | RT | Fraction | Scan | Source File | Area BRom | #Feature | #Feature BRom | Start | End | PTM | AScore | Found By |
| --- | --- | --- | --- | --- | --- | --- | --- | --- | --- | --- | --- | --- | --- | --- | --- | --- | --- | --- | --- |
| K.IGLVVIGPEVPLAAGIVDDLAAAGVR.C | Y | 51.18 | 2484.4314 | 26 | 0.6 | 829.1516 | 3 | 107.41 | 1 | 78285 | 01122020\_RID\_2209\_BuCaKA02.raw | 4.5855E5 | 1 | 1 | 67 | 92 |  |  | PEAKS DB |
| total 1 peptides |  |  |  |  |  |  |  |  |  |  |  |  |  |  |  |  |  |  |  |
| --- | --- | --- | --- | --- | --- | --- | --- | --- | --- | --- | --- | --- | --- | --- | --- | --- | --- | --- | --- |

LAA72685.1

back to list

  

| Protein Coverage
| Supporting Peptides
|

Protein Coverage:

Supporting Peptides:

| Peptide | Uniq | -10lgP | Mass | Length | ppm | m/z | z | RT | Fraction | Scan | Source File | Area BRom | #Feature | #Feature BRom | Start | End | PTM | AScore | Found By |
| --- | --- | --- | --- | --- | --- | --- | --- | --- | --- | --- | --- | --- | --- | --- | --- | --- | --- | --- | --- |
| K.IGLVVIGPEVPLAAGIVDDLAAAGVR.C | Y | 51.18 | 2484.4314 | 26 | 0.6 | 829.1516 | 3 | 107.41 | 1 | 78285 | 01122020\_RID\_2209\_BuCaKA02.raw | 4.5855E5 | 1 | 1 | 67 | 92 |  |  | PEAKS DB |
| total 1 peptides |  |  |  |  |  |  |  |  |  |  |  |  |  |  |  |  |  |  |  |
| --- | --- | --- | --- | --- | --- | --- | --- | --- | --- | --- | --- | --- | --- | --- | --- | --- | --- | --- | --- |

ETE73165.1

back to list

  

| Protein Coverage
| Supporting Peptides
|

Protein Coverage:

Supporting Peptides:

| Peptide | Uniq | -10lgP | Mass | Length | ppm | m/z | z | RT | Fraction | Scan | Source File | Area BRom | #Feature | #Feature BRom | Start | End | PTM | AScore | Found By |
| --- | --- | --- | --- | --- | --- | --- | --- | --- | --- | --- | --- | --- | --- | --- | --- | --- | --- | --- | --- |
| K.IGLVVIGPEVPLAAGIVDDLAAAGVR.C | Y | 51.18 | 2484.4314 | 26 | 0.6 | 829.1516 | 3 | 107.41 | 1 | 78285 | 01122020\_RID\_2209\_BuCaKA02.raw | 4.5855E5 | 1 | 1 | 67 | 92 |  |  | PEAKS DB |
| total 1 peptides |  |  |  |  |  |  |  |  |  |  |  |  |  |  |  |  |  |  |  |
| --- | --- | --- | --- | --- | --- | --- | --- | --- | --- | --- | --- | --- | --- | --- | --- | --- | --- | --- | --- |

LAA72697.1

back to list

  

| Protein Coverage
| Supporting Peptides
|

Protein Coverage:

Supporting Peptides:

| Peptide | Uniq | -10lgP | Mass | Length | ppm | m/z | z | RT | Fraction | Scan | Source File | Area BRom | #Feature | #Feature BRom | Start | End | PTM | AScore | Found By |
| --- | --- | --- | --- | --- | --- | --- | --- | --- | --- | --- | --- | --- | --- | --- | --- | --- | --- | --- | --- |
| K.IGLVVIGPEVPLAAGIVDDLAAAGVR.C | Y | 51.18 | 2484.4314 | 26 | 0.6 | 829.1516 | 3 | 107.41 | 1 | 78285 | 01122020\_RID\_2209\_BuCaKA02.raw | 4.5855E5 | 1 | 1 | 67 | 92 |  |  | PEAKS DB |
| total 1 peptides |  |  |  |  |  |  |  |  |  |  |  |  |  |  |  |  |  |  |  |
| --- | --- | --- | --- | --- | --- | --- | --- | --- | --- | --- | --- | --- | --- | --- | --- | --- | --- | --- | --- |

XP\_026519973.1

back to list

  

| Protein Coverage
| Supporting Peptides
|

Protein Coverage:

Supporting Peptides:

| Peptide | Uniq | -10lgP | Mass | Length | ppm | m/z | z | RT | Fraction | Scan | Source File | Area BRom | #Feature | #Feature BRom | Start | End | PTM | AScore | Found By |
| --- | --- | --- | --- | --- | --- | --- | --- | --- | --- | --- | --- | --- | --- | --- | --- | --- | --- | --- | --- |
| K.IGLVVIGPEVPLAAGIVDDLAAAGVR.C | Y | 51.18 | 2484.4314 | 26 | 0.6 | 829.1516 | 3 | 107.41 | 1 | 78285 | 01122020\_RID\_2209\_BuCaKA02.raw | 4.5855E5 | 1 | 1 | 67 | 92 |  |  | PEAKS DB |
| total 1 peptides |  |  |  |  |  |  |  |  |  |  |  |  |  |  |  |  |  |  |  |
| --- | --- | --- | --- | --- | --- | --- | --- | --- | --- | --- | --- | --- | --- | --- | --- | --- | --- | --- | --- |

XP\_026556356.1

back to list

  

| Protein Coverage
| Supporting Peptides
|

Protein Coverage:

Supporting Peptides:

| Peptide | Uniq | -10lgP | Mass | Length | ppm | m/z | z | RT | Fraction | Scan | Source File | Area BRom | #Feature | #Feature BRom | Start | End | PTM | AScore | Found By |
| --- | --- | --- | --- | --- | --- | --- | --- | --- | --- | --- | --- | --- | --- | --- | --- | --- | --- | --- | --- |
| K.IGLVVIGPEVPLAAGIVDDLAAAGVR.C | Y | 51.18 | 2484.4314 | 26 | 0.6 | 829.1516 | 3 | 107.41 | 1 | 78285 | 01122020\_RID\_2209\_BuCaKA02.raw | 4.5855E5 | 1 | 1 | 67 | 92 |  |  | PEAKS DB |
| total 1 peptides |  |  |  |  |  |  |  |  |  |  |  |  |  |  |  |  |  |  |  |
| --- | --- | --- | --- | --- | --- | --- | --- | --- | --- | --- | --- | --- | --- | --- | --- | --- | --- | --- | --- |

XP\_026556355.1

back to list

  

| Protein Coverage
| Supporting Peptides
|

Protein Coverage:

Supporting Peptides:

| Peptide | Uniq | -10lgP | Mass | Length | ppm | m/z | z | RT | Fraction | Scan | Source File | Area BRom | #Feature | #Feature BRom | Start | End | PTM | AScore | Found By |
| --- | --- | --- | --- | --- | --- | --- | --- | --- | --- | --- | --- | --- | --- | --- | --- | --- | --- | --- | --- |
| K.IGLVVIGPEVPLAAGIVDDLAAAGVR.C | Y | 51.18 | 2484.4314 | 26 | 0.6 | 829.1516 | 3 | 107.41 | 1 | 78285 | 01122020\_RID\_2209\_BuCaKA02.raw | 4.5855E5 | 1 | 1 | 67 | 92 |  |  | PEAKS DB |
| total 1 peptides |  |  |  |  |  |  |  |  |  |  |  |  |  |  |  |  |  |  |  |
| --- | --- | --- | --- | --- | --- | --- | --- | --- | --- | --- | --- | --- | --- | --- | --- | --- | --- | --- | --- |

XP\_034291632.1

back to list

  

| Protein Coverage
| Supporting Peptides
|

Protein Coverage:

Supporting Peptides:

| Peptide | Uniq | -10lgP | Mass | Length | ppm | m/z | z | RT | Fraction | Scan | Source File | Area BRom | #Feature | #Feature BRom | Start | End | PTM | AScore | Found By |
| --- | --- | --- | --- | --- | --- | --- | --- | --- | --- | --- | --- | --- | --- | --- | --- | --- | --- | --- | --- |
| K.IGLVVIGPEVPLAAGIVDDLAAAGVR.C | Y | 51.18 | 2484.4314 | 26 | 0.6 | 829.1516 | 3 | 107.41 | 1 | 78285 | 01122020\_RID\_2209\_BuCaKA02.raw | 4.5855E5 | 1 | 1 | 67 | 92 |  |  | PEAKS DB |
| total 1 peptides |  |  |  |  |  |  |  |  |  |  |  |  |  |  |  |  |  |  |  |
| --- | --- | --- | --- | --- | --- | --- | --- | --- | --- | --- | --- | --- | --- | --- | --- | --- | --- | --- | --- |

XP\_013931233.1

back to list

  

| Protein Coverage
| Supporting Peptides
|

Protein Coverage:

Supporting Peptides:

| Peptide | Uniq | -10lgP | Mass | Length | ppm | m/z | z | RT | Fraction | Scan | Source File | Area BRom | #Feature | #Feature BRom | Start | End | PTM | AScore | Found By |
| --- | --- | --- | --- | --- | --- | --- | --- | --- | --- | --- | --- | --- | --- | --- | --- | --- | --- | --- | --- |
| K.LLVVYPWTQRFFAHFGNLSGASAIC(+57.02)GNPQVK.A | Y | 45.93 | 3476.7815 | 31 | 3.4 | 696.3659 | 5 | 59.19 | 1 | 38711 | 01122020\_RID\_2209\_BuCaKA02.raw | 3.3751E7 | 1 | 1 | 32 | 62 | Carbamidomethylation | C25:Carbamidomethylation:1000.00 | PEAKS DB |
| total 1 peptides |  |  |  |  |  |  |  |  |  |  |  |  |  |  |  |  |  |  |  |
| --- | --- | --- | --- | --- | --- | --- | --- | --- | --- | --- | --- | --- | --- | --- | --- | --- | --- | --- | --- |

XP\_032075324.1

back to list

  

| Protein Coverage
| Supporting Peptides
|

Protein Coverage:

Supporting Peptides:

| Peptide | Uniq | -10lgP | Mass | Length | ppm | m/z | z | RT | Fraction | Scan | Source File | Area BRom | #Feature | #Feature BRom | Start | End | PTM | AScore | Found By |
| --- | --- | --- | --- | --- | --- | --- | --- | --- | --- | --- | --- | --- | --- | --- | --- | --- | --- | --- | --- |
| K.LLVVYPWTQRFFAHFGNLSGASAIC(+57.02)GNPQVK.A | Y | 45.93 | 3476.7815 | 31 | 3.4 | 696.3659 | 5 | 59.19 | 1 | 38711 | 01122020\_RID\_2209\_BuCaKA02.raw | 3.3751E7 | 1 | 1 | 32 | 62 | Carbamidomethylation | C25:Carbamidomethylation:1000.00 | PEAKS DB |
| total 1 peptides |  |  |  |  |  |  |  |  |  |  |  |  |  |  |  |  |  |  |  |
| --- | --- | --- | --- | --- | --- | --- | --- | --- | --- | --- | --- | --- | --- | --- | --- | --- | --- | --- | --- |

XP\_026561289.1

back to list

  

| Protein Coverage
| Supporting Peptides
|

Protein Coverage:

Supporting Peptides:

| Peptide | Uniq | -10lgP | Mass | Length | ppm | m/z | z | RT | Fraction | Scan | Source File | Area BRom | #Feature | #Feature BRom | Start | End | PTM | AScore | Found By |
| --- | --- | --- | --- | --- | --- | --- | --- | --- | --- | --- | --- | --- | --- | --- | --- | --- | --- | --- | --- |
| R.VRDVELLTGLDF.Y | Y | 41.50 | 1375.7347 | 12 | 2.1 | 688.8761 | 2 | 74.92 | 1 | 51748 | 01122020\_RID\_2209\_BuCaKA02.raw | 2.615E6 | 1 | 1 | 816 | 827 |  |  | PEAKS DB |
| total 1 peptides |  |  |  |  |  |  |  |  |  |  |  |  |  |  |  |  |  |  |  |
| --- | --- | --- | --- | --- | --- | --- | --- | --- | --- | --- | --- | --- | --- | --- | --- | --- | --- | --- | --- |

XP\_007429085.1

back to list

  

| Protein Coverage
| Supporting Peptides
|

Protein Coverage:

Supporting Peptides:

| Peptide | Uniq | -10lgP | Mass | Length | ppm | m/z | z | RT | Fraction | Scan | Source File | Area BRom | #Feature | #Feature BRom | Start | End | PTM | AScore | Found By |
| --- | --- | --- | --- | --- | --- | --- | --- | --- | --- | --- | --- | --- | --- | --- | --- | --- | --- | --- | --- |
| E.FTGLEQIVPNIVR.A | Y | 40.86 | 1484.8351 | 13 | 4.1 | 743.4279 | 2 | 69.07 | 1 | 46817 | 01122020\_RID\_2209\_BuCaKA02.raw | 1.1454E6 | 1 | 1 | 523 | 535 |  |  | PEAKS DB |
| total 1 peptides |  |  |  |  |  |  |  |  |  |  |  |  |  |  |  |  |  |  |  |
| --- | --- | --- | --- | --- | --- | --- | --- | --- | --- | --- | --- | --- | --- | --- | --- | --- | --- | --- | --- |

LAA86986.1

back to list

  

| Protein Coverage
| Supporting Peptides
|

Protein Coverage:

Supporting Peptides:

| Peptide | Uniq | -10lgP | Mass | Length | ppm | m/z | z | RT | Fraction | Scan | Source File | Area BRom | #Feature | #Feature BRom | Start | End | PTM | AScore | Found By |
| --- | --- | --- | --- | --- | --- | --- | --- | --- | --- | --- | --- | --- | --- | --- | --- | --- | --- | --- | --- |
| E.FTGLEQIVPNIVR.A | Y | 40.86 | 1484.8351 | 13 | 4.1 | 743.4279 | 2 | 69.07 | 1 | 46817 | 01122020\_RID\_2209\_BuCaKA02.raw | 1.1454E6 | 1 | 1 | 234 | 246 |  |  | PEAKS DB |
| total 1 peptides |  |  |  |  |  |  |  |  |  |  |  |  |  |  |  |  |  |  |  |
| --- | --- | --- | --- | --- | --- | --- | --- | --- | --- | --- | --- | --- | --- | --- | --- | --- | --- | --- | --- |

LAB57337.1

back to list

  

| Protein Coverage
| Supporting Peptides
|

Protein Coverage:

Supporting Peptides:

| Peptide | Uniq | -10lgP | Mass | Length | ppm | m/z | z | RT | Fraction | Scan | Source File | Area BRom | #Feature | #Feature BRom | Start | End | PTM | AScore | Found By |
| --- | --- | --- | --- | --- | --- | --- | --- | --- | --- | --- | --- | --- | --- | --- | --- | --- | --- | --- | --- |
| E.FTGLEQIVPNIVR.A | Y | 40.86 | 1484.8351 | 13 | 4.1 | 743.4279 | 2 | 69.07 | 1 | 46817 | 01122020\_RID\_2209\_BuCaKA02.raw | 1.1454E6 | 1 | 1 | 252 | 264 |  |  | PEAKS DB |
| total 1 peptides |  |  |  |  |  |  |  |  |  |  |  |  |  |  |  |  |  |  |  |
| --- | --- | --- | --- | --- | --- | --- | --- | --- | --- | --- | --- | --- | --- | --- | --- | --- | --- | --- | --- |

XP\_013923615.1

back to list

  

| Protein Coverage
| Supporting Peptides
|

Protein Coverage:

Supporting Peptides:

| Peptide | Uniq | -10lgP | Mass | Length | ppm | m/z | z | RT | Fraction | Scan | Source File | Area BRom | #Feature | #Feature BRom | Start | End | PTM | AScore | Found By |
| --- | --- | --- | --- | --- | --- | --- | --- | --- | --- | --- | --- | --- | --- | --- | --- | --- | --- | --- | --- |
| E.FTGLEQIVPNIVR.A | Y | 40.86 | 1484.8351 | 13 | 4.1 | 743.4279 | 2 | 69.07 | 1 | 46817 | 01122020\_RID\_2209\_BuCaKA02.raw | 1.1454E6 | 1 | 1 | 523 | 535 |  |  | PEAKS DB |
| total 1 peptides |  |  |  |  |  |  |  |  |  |  |  |  |  |  |  |  |  |  |  |
| --- | --- | --- | --- | --- | --- | --- | --- | --- | --- | --- | --- | --- | --- | --- | --- | --- | --- | --- | --- |

XP\_026520312.1

back to list

  

| Protein Coverage
| Supporting Peptides
|

Protein Coverage:

Supporting Peptides:

| Peptide | Uniq | -10lgP | Mass | Length | ppm | m/z | z | RT | Fraction | Scan | Source File | Area BRom | #Feature | #Feature BRom | Start | End | PTM | AScore | Found By |
| --- | --- | --- | --- | --- | --- | --- | --- | --- | --- | --- | --- | --- | --- | --- | --- | --- | --- | --- | --- |
| E.FTGLEQIVPNIVR.A | Y | 40.86 | 1484.8351 | 13 | 4.1 | 743.4279 | 2 | 69.07 | 1 | 46817 | 01122020\_RID\_2209\_BuCaKA02.raw | 1.1454E6 | 1 | 1 | 523 | 535 |  |  | PEAKS DB |
| total 1 peptides |  |  |  |  |  |  |  |  |  |  |  |  |  |  |  |  |  |  |  |
| --- | --- | --- | --- | --- | --- | --- | --- | --- | --- | --- | --- | --- | --- | --- | --- | --- | --- | --- | --- |

XP\_034273852.1

back to list

  

| Protein Coverage
| Supporting Peptides
|

Protein Coverage:

Supporting Peptides:

| Peptide | Uniq | -10lgP | Mass | Length | ppm | m/z | z | RT | Fraction | Scan | Source File | Area BRom | #Feature | #Feature BRom | Start | End | PTM | AScore | Found By |
| --- | --- | --- | --- | --- | --- | --- | --- | --- | --- | --- | --- | --- | --- | --- | --- | --- | --- | --- | --- |
| E.FTGLEQIVPNIVR.A | Y | 40.86 | 1484.8351 | 13 | 4.1 | 743.4279 | 2 | 69.07 | 1 | 46817 | 01122020\_RID\_2209\_BuCaKA02.raw | 1.1454E6 | 1 | 1 | 523 | 535 |  |  | PEAKS DB |
| total 1 peptides |  |  |  |  |  |  |  |  |  |  |  |  |  |  |  |  |  |  |  |
| --- | --- | --- | --- | --- | --- | --- | --- | --- | --- | --- | --- | --- | --- | --- | --- | --- | --- | --- | --- |

LAB44384.1

back to list

  

| Protein Coverage
| Supporting Peptides
|

Protein Coverage:

Supporting Peptides:

| Peptide | Uniq | -10lgP | Mass | Length | ppm | m/z | z | RT | Fraction | Scan | Source File | Area BRom | #Feature | #Feature BRom | Start | End | PTM | AScore | Found By |
| --- | --- | --- | --- | --- | --- | --- | --- | --- | --- | --- | --- | --- | --- | --- | --- | --- | --- | --- | --- |
| E.FTGLEQIVPNIVR.A | Y | 40.86 | 1484.8351 | 13 | 4.1 | 743.4279 | 2 | 69.07 | 1 | 46817 | 01122020\_RID\_2209\_BuCaKA02.raw | 1.1454E6 | 1 | 1 | 523 | 535 |  |  | PEAKS DB |
| total 1 peptides |  |  |  |  |  |  |  |  |  |  |  |  |  |  |  |  |  |  |  |
| --- | --- | --- | --- | --- | --- | --- | --- | --- | --- | --- | --- | --- | --- | --- | --- | --- | --- | --- | --- |

XP\_026566518.1

back to list

  

| Protein Coverage
| Supporting Peptides
|

Protein Coverage:

Supporting Peptides:

| Peptide | Uniq | -10lgP | Mass | Length | ppm | m/z | z | RT | Fraction | Scan | Source File | Area BRom | #Feature | #Feature BRom | Start | End | PTM | AScore | Found By |
| --- | --- | --- | --- | --- | --- | --- | --- | --- | --- | --- | --- | --- | --- | --- | --- | --- | --- | --- | --- |
| E.FTGLEQIVPNIVR.A | Y | 40.86 | 1484.8351 | 13 | 4.1 | 743.4279 | 2 | 69.07 | 1 | 46817 | 01122020\_RID\_2209\_BuCaKA02.raw | 1.1454E6 | 1 | 1 | 523 | 535 |  |  | PEAKS DB |
| total 1 peptides |  |  |  |  |  |  |  |  |  |  |  |  |  |  |  |  |  |  |  |
| --- | --- | --- | --- | --- | --- | --- | --- | --- | --- | --- | --- | --- | --- | --- | --- | --- | --- | --- | --- |

XP\_032069561.1

back to list

  

| Protein Coverage
| Supporting Peptides
|

Protein Coverage:

Supporting Peptides:

| Peptide | Uniq | -10lgP | Mass | Length | ppm | m/z | z | RT | Fraction | Scan | Source File | Area BRom | #Feature | #Feature BRom | Start | End | PTM | AScore | Found By |
| --- | --- | --- | --- | --- | --- | --- | --- | --- | --- | --- | --- | --- | --- | --- | --- | --- | --- | --- | --- |
| E.FTGLEQIVPNIVR.A | Y | 40.86 | 1484.8351 | 13 | 4.1 | 743.4279 | 2 | 69.07 | 1 | 46817 | 01122020\_RID\_2209\_BuCaKA02.raw | 1.1454E6 | 1 | 1 | 542 | 554 |  |  | PEAKS DB |
| total 1 peptides |  |  |  |  |  |  |  |  |  |  |  |  |  |  |  |  |  |  |  |
| --- | --- | --- | --- | --- | --- | --- | --- | --- | --- | --- | --- | --- | --- | --- | --- | --- | --- | --- | --- |

XP\_026526467.1

back to list

  

| Protein Coverage
| Supporting Peptides
|

Protein Coverage:

Supporting Peptides:

| Peptide | Uniq | -10lgP | Mass | Length | ppm | m/z | z | RT | Fraction | Scan | Source File | Area BRom | #Feature | #Feature BRom | Start | End | PTM | AScore | Found By |
| --- | --- | --- | --- | --- | --- | --- | --- | --- | --- | --- | --- | --- | --- | --- | --- | --- | --- | --- | --- |
| K.NVHISSNGIAEAR.N | Y | 40.42 | 1366.6953 | 13 | -2.6 | 684.3531 | 2 | 72.27 | 1 | 49381 | 01122020\_RID\_2209\_BuCaKA02.raw | 1.7947E7 | 1 | 1 | 237 | 249 |  |  | PEAKS DB |
| total 1 peptides |  |  |  |  |  |  |  |  |  |  |  |  |  |  |  |  |  |  |  |
| --- | --- | --- | --- | --- | --- | --- | --- | --- | --- | --- | --- | --- | --- | --- | --- | --- | --- | --- | --- |

ABW24182.1

back to list

  

| Protein Coverage
| Supporting Peptides
|

Protein Coverage:

Supporting Peptides:

| Peptide | Uniq | -10lgP | Mass | Length | ppm | m/z | z | RT | Fraction | Scan | Source File | Area BRom | #Feature | #Feature BRom | Start | End | PTM | AScore | Found By |
| --- | --- | --- | --- | --- | --- | --- | --- | --- | --- | --- | --- | --- | --- | --- | --- | --- | --- | --- | --- |
| P.TKHYMDYSC(+57.02)YC(+57.02)GKGGSGTPVDELDR.C | Y | 40.08 | 2895.2261 | 25 | 0.9 | 966.0835 | 3 | 85.70 | 1 | 60609 | 01122020\_RID\_2209\_BuCaKA02.raw | 1.8079E5 | 2 | 2 | 46 | 70 | Carbamidomethylation | C9:Carbamidomethylation:1000.00;C11:Carbamidomethylation:1000.00 | PEAKS DB |
| total 1 peptides |  |  |  |  |  |  |  |  |  |  |  |  |  |  |  |  |  |  |  |
| --- | --- | --- | --- | --- | --- | --- | --- | --- | --- | --- | --- | --- | --- | --- | --- | --- | --- | --- | --- |

XP\_013931090.1

back to list

  

| Protein Coverage
| Supporting Peptides
|

Protein Coverage:

Supporting Peptides:

| Peptide | Uniq | -10lgP | Mass | Length | ppm | m/z | z | RT | Fraction | Scan | Source File | Area BRom | #Feature | #Feature BRom | Start | End | PTM | AScore | Found By |
| --- | --- | --- | --- | --- | --- | --- | --- | --- | --- | --- | --- | --- | --- | --- | --- | --- | --- | --- | --- |
| D.LVVGLC(+57.02)TGQIK.T | Y | 39.69 | 1186.6743 | 11 | 1.1 | 594.3451 | 2 | 36.12 | 1 | 20687 | 01122020\_RID\_2209\_BuCaKA02.raw | 0 | 0 | 0 | 427 | 437 | Carbamidomethylation | C6:Carbamidomethylation:1000.00 | PEAKS DB |
| total 1 peptides |  |  |  |  |  |  |  |  |  |  |  |  |  |  |  |  |  |  |  |
| --- | --- | --- | --- | --- | --- | --- | --- | --- | --- | --- | --- | --- | --- | --- | --- | --- | --- | --- | --- |

XP\_032091032.1

back to list

  

| Protein Coverage
| Supporting Peptides
|

Protein Coverage:

Supporting Peptides:

| Peptide | Uniq | -10lgP | Mass | Length | ppm | m/z | z | RT | Fraction | Scan | Source File | Area BRom | #Feature | #Feature BRom | Start | End | PTM | AScore | Found By |
| --- | --- | --- | --- | --- | --- | --- | --- | --- | --- | --- | --- | --- | --- | --- | --- | --- | --- | --- | --- |
| D.LVVGLC(+57.02)TGQIK.T | Y | 39.69 | 1186.6743 | 11 | 1.1 | 594.3451 | 2 | 36.12 | 1 | 20687 | 01122020\_RID\_2209\_BuCaKA02.raw | 0 | 0 | 0 | 384 | 394 | Carbamidomethylation | C6:Carbamidomethylation:1000.00 | PEAKS DB |
| total 1 peptides |  |  |  |  |  |  |  |  |  |  |  |  |  |  |  |  |  |  |  |
| --- | --- | --- | --- | --- | --- | --- | --- | --- | --- | --- | --- | --- | --- | --- | --- | --- | --- | --- | --- |

JAB54294.1

back to list

  

| Protein Coverage
| Supporting Peptides
|

Protein Coverage:

Supporting Peptides:

| Peptide | Uniq | -10lgP | Mass | Length | ppm | m/z | z | RT | Fraction | Scan | Source File | Area BRom | #Feature | #Feature BRom | Start | End | PTM | AScore | Found By |
| --- | --- | --- | --- | --- | --- | --- | --- | --- | --- | --- | --- | --- | --- | --- | --- | --- | --- | --- | --- |
| D.LVVGLC(+57.02)TGQIK.T | Y | 39.69 | 1186.6743 | 11 | 1.1 | 594.3451 | 2 | 36.12 | 1 | 20687 | 01122020\_RID\_2209\_BuCaKA02.raw | 0 | 0 | 0 | 384 | 394 | Carbamidomethylation | C6:Carbamidomethylation:1000.00 | PEAKS DB |
| total 1 peptides |  |  |  |  |  |  |  |  |  |  |  |  |  |  |  |  |  |  |  |
| --- | --- | --- | --- | --- | --- | --- | --- | --- | --- | --- | --- | --- | --- | --- | --- | --- | --- | --- | --- |

XP\_007441409.1

back to list

  

| Protein Coverage
| Supporting Peptides
|

Protein Coverage:

Supporting Peptides:

| Peptide | Uniq | -10lgP | Mass | Length | ppm | m/z | z | RT | Fraction | Scan | Source File | Area BRom | #Feature | #Feature BRom | Start | End | PTM | AScore | Found By |
| --- | --- | --- | --- | --- | --- | --- | --- | --- | --- | --- | --- | --- | --- | --- | --- | --- | --- | --- | --- |
| D.LVVGLC(+57.02)TGQIK.T | Y | 39.69 | 1186.6743 | 11 | 1.1 | 594.3451 | 2 | 36.12 | 1 | 20687 | 01122020\_RID\_2209\_BuCaKA02.raw | 0 | 0 | 0 | 384 | 394 | Carbamidomethylation | C6:Carbamidomethylation:1000.00 | PEAKS DB |
| total 1 peptides |  |  |  |  |  |  |  |  |  |  |  |  |  |  |  |  |  |  |  |
| --- | --- | --- | --- | --- | --- | --- | --- | --- | --- | --- | --- | --- | --- | --- | --- | --- | --- | --- | --- |

JAG68757.1

back to list

  

| Protein Coverage
| Supporting Peptides
|

Protein Coverage:

Supporting Peptides:

| Peptide | Uniq | -10lgP | Mass | Length | ppm | m/z | z | RT | Fraction | Scan | Source File | Area BRom | #Feature | #Feature BRom | Start | End | PTM | AScore | Found By |
| --- | --- | --- | --- | --- | --- | --- | --- | --- | --- | --- | --- | --- | --- | --- | --- | --- | --- | --- | --- |
| D.LVVGLC(+57.02)TGQIK.T | Y | 39.69 | 1186.6743 | 11 | 1.1 | 594.3451 | 2 | 36.12 | 1 | 20687 | 01122020\_RID\_2209\_BuCaKA02.raw | 0 | 0 | 0 | 384 | 394 | Carbamidomethylation | C6:Carbamidomethylation:1000.00 | PEAKS DB |
| total 1 peptides |  |  |  |  |  |  |  |  |  |  |  |  |  |  |  |  |  |  |  |
| --- | --- | --- | --- | --- | --- | --- | --- | --- | --- | --- | --- | --- | --- | --- | --- | --- | --- | --- | --- |

CBN61477.1

back to list

  

| Protein Coverage
| Supporting Peptides
|

Protein Coverage:

Supporting Peptides:

| Peptide | Uniq | -10lgP | Mass | Length | ppm | m/z | z | RT | Fraction | Scan | Source File | Area BRom | #Feature | #Feature BRom | Start | End | PTM | AScore | Found By |
| --- | --- | --- | --- | --- | --- | --- | --- | --- | --- | --- | --- | --- | --- | --- | --- | --- | --- | --- | --- |
| D.LVVGLC(+57.02)TGQIK.T | Y | 39.69 | 1186.6743 | 11 | 1.1 | 594.3451 | 2 | 36.12 | 1 | 20687 | 01122020\_RID\_2209\_BuCaKA02.raw | 0 | 0 | 0 | 384 | 394 | Carbamidomethylation | C6:Carbamidomethylation:1000.00 | PEAKS DB |
| total 1 peptides |  |  |  |  |  |  |  |  |  |  |  |  |  |  |  |  |  |  |  |
| --- | --- | --- | --- | --- | --- | --- | --- | --- | --- | --- | --- | --- | --- | --- | --- | --- | --- | --- | --- |

AAD41646.1

back to list

  

| Protein Coverage
| Supporting Peptides
|

Protein Coverage:

Supporting Peptides:

| Peptide | Uniq | -10lgP | Mass | Length | ppm | m/z | z | RT | Fraction | Scan | Source File | Area BRom | #Feature | #Feature BRom | Start | End | PTM | AScore | Found By |
| --- | --- | --- | --- | --- | --- | --- | --- | --- | --- | --- | --- | --- | --- | --- | --- | --- | --- | --- | --- |
| D.LVVGLC(+57.02)TGQIK.T | Y | 39.69 | 1186.6743 | 11 | 1.1 | 594.3451 | 2 | 36.12 | 1 | 20687 | 01122020\_RID\_2209\_BuCaKA02.raw | 0 | 0 | 0 | 384 | 394 | Carbamidomethylation | C6:Carbamidomethylation:1000.00 | PEAKS DB |
| total 1 peptides |  |  |  |  |  |  |  |  |  |  |  |  |  |  |  |  |  |  |  |
| --- | --- | --- | --- | --- | --- | --- | --- | --- | --- | --- | --- | --- | --- | --- | --- | --- | --- | --- | --- |

XP\_026571534.1

back to list

  

| Protein Coverage
| Supporting Peptides
|

Protein Coverage:

Supporting Peptides:

| Peptide | Uniq | -10lgP | Mass | Length | ppm | m/z | z | RT | Fraction | Scan | Source File | Area BRom | #Feature | #Feature BRom | Start | End | PTM | AScore | Found By |
| --- | --- | --- | --- | --- | --- | --- | --- | --- | --- | --- | --- | --- | --- | --- | --- | --- | --- | --- | --- |
| D.LVVGLC(+57.02)TGQIK.T | Y | 39.69 | 1186.6743 | 11 | 1.1 | 594.3451 | 2 | 36.12 | 1 | 20687 | 01122020\_RID\_2209\_BuCaKA02.raw | 0 | 0 | 0 | 384 | 394 | Carbamidomethylation | C6:Carbamidomethylation:1000.00 | PEAKS DB |
| total 1 peptides |  |  |  |  |  |  |  |  |  |  |  |  |  |  |  |  |  |  |  |
| --- | --- | --- | --- | --- | --- | --- | --- | --- | --- | --- | --- | --- | --- | --- | --- | --- | --- | --- | --- |

XP\_026582136.1

back to list

  

| Protein Coverage
| Supporting Peptides
|

Protein Coverage:

Supporting Peptides:

| Peptide | Uniq | -10lgP | Mass | Length | ppm | m/z | z | RT | Fraction | Scan | Source File | Area BRom | #Feature | #Feature BRom | Start | End | PTM | AScore | Found By |
| --- | --- | --- | --- | --- | --- | --- | --- | --- | --- | --- | --- | --- | --- | --- | --- | --- | --- | --- | --- |
| D.LVVGLC(+57.02)TGQIK.T | Y | 39.69 | 1186.6743 | 11 | 1.1 | 594.3451 | 2 | 36.12 | 1 | 20687 | 01122020\_RID\_2209\_BuCaKA02.raw | 0 | 0 | 0 | 95 | 105 | Carbamidomethylation | C6:Carbamidomethylation:1000.00 | PEAKS DB |
| total 1 peptides |  |  |  |  |  |  |  |  |  |  |  |  |  |  |  |  |  |  |  |
| --- | --- | --- | --- | --- | --- | --- | --- | --- | --- | --- | --- | --- | --- | --- | --- | --- | --- | --- | --- |

XP\_013923835.1

back to list

  

| Protein Coverage
| Supporting Peptides
|

Protein Coverage:

Supporting Peptides:

| Peptide | Uniq | -10lgP | Mass | Length | ppm | m/z | z | RT | Fraction | Scan | Source File | Area BRom | #Feature | #Feature BRom | Start | End | PTM | AScore | Found By |
| --- | --- | --- | --- | --- | --- | --- | --- | --- | --- | --- | --- | --- | --- | --- | --- | --- | --- | --- | --- |
| D.LVVGLC(+57.02)TGQIK.T | Y | 39.69 | 1186.6743 | 11 | 1.1 | 594.3451 | 2 | 36.12 | 1 | 20687 | 01122020\_RID\_2209\_BuCaKA02.raw | 0 | 0 | 0 | 109 | 119 | Carbamidomethylation | C6:Carbamidomethylation:1000.00 | PEAKS DB |
| total 1 peptides |  |  |  |  |  |  |  |  |  |  |  |  |  |  |  |  |  |  |  |
| --- | --- | --- | --- | --- | --- | --- | --- | --- | --- | --- | --- | --- | --- | --- | --- | --- | --- | --- | --- |

XP\_013916893.1

back to list

  

| Protein Coverage
| Supporting Peptides
|

Protein Coverage:

Supporting Peptides:

| Peptide | Uniq | -10lgP | Mass | Length | ppm | m/z | z | RT | Fraction | Scan | Source File | Area BRom | #Feature | #Feature BRom | Start | End | PTM | AScore | Found By |
| --- | --- | --- | --- | --- | --- | --- | --- | --- | --- | --- | --- | --- | --- | --- | --- | --- | --- | --- | --- |
| D.LVVGLC(+57.02)TGQIK.T | Y | 39.69 | 1186.6743 | 11 | 1.1 | 594.3451 | 2 | 36.12 | 1 | 20687 | 01122020\_RID\_2209\_BuCaKA02.raw | 0 | 0 | 0 | 136 | 146 | Carbamidomethylation | C6:Carbamidomethylation:1000.00 | PEAKS DB |
| total 1 peptides |  |  |  |  |  |  |  |  |  |  |  |  |  |  |  |  |  |  |  |
| --- | --- | --- | --- | --- | --- | --- | --- | --- | --- | --- | --- | --- | --- | --- | --- | --- | --- | --- | --- |

LAA54352.1

back to list

  

| Protein Coverage
| Supporting Peptides
|

Protein Coverage:

Supporting Peptides:

| Peptide | Uniq | -10lgP | Mass | Length | ppm | m/z | z | RT | Fraction | Scan | Source File | Area BRom | #Feature | #Feature BRom | Start | End | PTM | AScore | Found By |
| --- | --- | --- | --- | --- | --- | --- | --- | --- | --- | --- | --- | --- | --- | --- | --- | --- | --- | --- | --- |
| D.LVVGLC(+57.02)TGQIK.T | Y | 39.69 | 1186.6743 | 11 | 1.1 | 594.3451 | 2 | 36.12 | 1 | 20687 | 01122020\_RID\_2209\_BuCaKA02.raw | 0 | 0 | 0 | 220 | 230 | Carbamidomethylation | C6:Carbamidomethylation:1000.00 | PEAKS DB |
| total 1 peptides |  |  |  |  |  |  |  |  |  |  |  |  |  |  |  |  |  |  |  |
| --- | --- | --- | --- | --- | --- | --- | --- | --- | --- | --- | --- | --- | --- | --- | --- | --- | --- | --- | --- |

LAA72706.1

back to list

  

| Protein Coverage
| Supporting Peptides
|

Protein Coverage:

Supporting Peptides:

| Peptide | Uniq | -10lgP | Mass | Length | ppm | m/z | z | RT | Fraction | Scan | Source File | Area BRom | #Feature | #Feature BRom | Start | End | PTM | AScore | Found By |
| --- | --- | --- | --- | --- | --- | --- | --- | --- | --- | --- | --- | --- | --- | --- | --- | --- | --- | --- | --- |
| D.LVVGLC(+57.02)TGQIK.T | Y | 39.69 | 1186.6743 | 11 | 1.1 | 594.3451 | 2 | 36.12 | 1 | 20687 | 01122020\_RID\_2209\_BuCaKA02.raw | 0 | 0 | 0 | 244 | 254 | Carbamidomethylation | C6:Carbamidomethylation:1000.00 | PEAKS DB |
| total 1 peptides |  |  |  |  |  |  |  |  |  |  |  |  |  |  |  |  |  |  |  |
| --- | --- | --- | --- | --- | --- | --- | --- | --- | --- | --- | --- | --- | --- | --- | --- | --- | --- | --- | --- |

LAA54354.1

back to list

  

| Protein Coverage
| Supporting Peptides
|

Protein Coverage:

Supporting Peptides:

| Peptide | Uniq | -10lgP | Mass | Length | ppm | m/z | z | RT | Fraction | Scan | Source File | Area BRom | #Feature | #Feature BRom | Start | End | PTM | AScore | Found By |
| --- | --- | --- | --- | --- | --- | --- | --- | --- | --- | --- | --- | --- | --- | --- | --- | --- | --- | --- | --- |
| D.LVVGLC(+57.02)TGQIK.T | Y | 39.69 | 1186.6743 | 11 | 1.1 | 594.3451 | 2 | 36.12 | 1 | 20687 | 01122020\_RID\_2209\_BuCaKA02.raw | 0 | 0 | 0 | 306 | 316 | Carbamidomethylation | C6:Carbamidomethylation:1000.00 | PEAKS DB |
| total 1 peptides |  |  |  |  |  |  |  |  |  |  |  |  |  |  |  |  |  |  |  |
| --- | --- | --- | --- | --- | --- | --- | --- | --- | --- | --- | --- | --- | --- | --- | --- | --- | --- | --- | --- |

LAA54356.1

back to list

  

| Protein Coverage
| Supporting Peptides
|

Protein Coverage:

Supporting Peptides:

| Peptide | Uniq | -10lgP | Mass | Length | ppm | m/z | z | RT | Fraction | Scan | Source File | Area BRom | #Feature | #Feature BRom | Start | End | PTM | AScore | Found By |
| --- | --- | --- | --- | --- | --- | --- | --- | --- | --- | --- | --- | --- | --- | --- | --- | --- | --- | --- | --- |
| D.LVVGLC(+57.02)TGQIK.T | Y | 39.69 | 1186.6743 | 11 | 1.1 | 594.3451 | 2 | 36.12 | 1 | 20687 | 01122020\_RID\_2209\_BuCaKA02.raw | 0 | 0 | 0 | 324 | 334 | Carbamidomethylation | C6:Carbamidomethylation:1000.00 | PEAKS DB |
| total 1 peptides |  |  |  |  |  |  |  |  |  |  |  |  |  |  |  |  |  |  |  |
| --- | --- | --- | --- | --- | --- | --- | --- | --- | --- | --- | --- | --- | --- | --- | --- | --- | --- | --- | --- |

LAA78451.1

back to list

  

| Protein Coverage
| Supporting Peptides
|

Protein Coverage:

Supporting Peptides:

| Peptide | Uniq | -10lgP | Mass | Length | ppm | m/z | z | RT | Fraction | Scan | Source File | Area BRom | #Feature | #Feature BRom | Start | End | PTM | AScore | Found By |
| --- | --- | --- | --- | --- | --- | --- | --- | --- | --- | --- | --- | --- | --- | --- | --- | --- | --- | --- | --- |
| D.LVVGLC(+57.02)TGQIK.T | Y | 39.69 | 1186.6743 | 11 | 1.1 | 594.3451 | 2 | 36.12 | 1 | 20687 | 01122020\_RID\_2209\_BuCaKA02.raw | 0 | 0 | 0 | 327 | 337 | Carbamidomethylation | C6:Carbamidomethylation:1000.00 | PEAKS DB |
| total 1 peptides |  |  |  |  |  |  |  |  |  |  |  |  |  |  |  |  |  |  |  |
| --- | --- | --- | --- | --- | --- | --- | --- | --- | --- | --- | --- | --- | --- | --- | --- | --- | --- | --- | --- |

LAA78455.1

back to list

  

| Protein Coverage
| Supporting Peptides
|

Protein Coverage:

Supporting Peptides:

| Peptide | Uniq | -10lgP | Mass | Length | ppm | m/z | z | RT | Fraction | Scan | Source File | Area BRom | #Feature | #Feature BRom | Start | End | PTM | AScore | Found By |
| --- | --- | --- | --- | --- | --- | --- | --- | --- | --- | --- | --- | --- | --- | --- | --- | --- | --- | --- | --- |
| D.LVVGLC(+57.02)TGQIK.T | Y | 39.69 | 1186.6743 | 11 | 1.1 | 594.3451 | 2 | 36.12 | 1 | 20687 | 01122020\_RID\_2209\_BuCaKA02.raw | 0 | 0 | 0 | 327 | 337 | Carbamidomethylation | C6:Carbamidomethylation:1000.00 | PEAKS DB |
| total 1 peptides |  |  |  |  |  |  |  |  |  |  |  |  |  |  |  |  |  |  |  |
| --- | --- | --- | --- | --- | --- | --- | --- | --- | --- | --- | --- | --- | --- | --- | --- | --- | --- | --- | --- |

JAI10301.1

back to list

  

| Protein Coverage
| Supporting Peptides
|

Protein Coverage:

Supporting Peptides:

| Peptide | Uniq | -10lgP | Mass | Length | ppm | m/z | z | RT | Fraction | Scan | Source File | Area BRom | #Feature | #Feature BRom | Start | End | PTM | AScore | Found By |
| --- | --- | --- | --- | --- | --- | --- | --- | --- | --- | --- | --- | --- | --- | --- | --- | --- | --- | --- | --- |
| D.LVVGLC(+57.02)TGQIK.T | Y | 39.69 | 1186.6743 | 11 | 1.1 | 594.3451 | 2 | 36.12 | 1 | 20687 | 01122020\_RID\_2209\_BuCaKA02.raw | 0 | 0 | 0 | 384 | 394 | Carbamidomethylation | C6:Carbamidomethylation:1000.00 | PEAKS DB |
| total 1 peptides |  |  |  |  |  |  |  |  |  |  |  |  |  |  |  |  |  |  |  |
| --- | --- | --- | --- | --- | --- | --- | --- | --- | --- | --- | --- | --- | --- | --- | --- | --- | --- | --- | --- |

JAG47510.1

back to list

  

| Protein Coverage
| Supporting Peptides
|

Protein Coverage:

Supporting Peptides:

| Peptide | Uniq | -10lgP | Mass | Length | ppm | m/z | z | RT | Fraction | Scan | Source File | Area BRom | #Feature | #Feature BRom | Start | End | PTM | AScore | Found By |
| --- | --- | --- | --- | --- | --- | --- | --- | --- | --- | --- | --- | --- | --- | --- | --- | --- | --- | --- | --- |
| D.LVVGLC(+57.02)TGQIK.T | Y | 39.69 | 1186.6743 | 11 | 1.1 | 594.3451 | 2 | 36.12 | 1 | 20687 | 01122020\_RID\_2209\_BuCaKA02.raw | 0 | 0 | 0 | 384 | 394 | Carbamidomethylation | C6:Carbamidomethylation:1000.00 | PEAKS DB |
| total 1 peptides |  |  |  |  |  |  |  |  |  |  |  |  |  |  |  |  |  |  |  |
| --- | --- | --- | --- | --- | --- | --- | --- | --- | --- | --- | --- | --- | --- | --- | --- | --- | --- | --- | --- |

JAG45884.1

back to list

  

| Protein Coverage
| Supporting Peptides
|

Protein Coverage:

Supporting Peptides:

| Peptide | Uniq | -10lgP | Mass | Length | ppm | m/z | z | RT | Fraction | Scan | Source File | Area BRom | #Feature | #Feature BRom | Start | End | PTM | AScore | Found By |
| --- | --- | --- | --- | --- | --- | --- | --- | --- | --- | --- | --- | --- | --- | --- | --- | --- | --- | --- | --- |
| D.LVVGLC(+57.02)TGQIK.T | Y | 39.69 | 1186.6743 | 11 | 1.1 | 594.3451 | 2 | 36.12 | 1 | 20687 | 01122020\_RID\_2209\_BuCaKA02.raw | 0 | 0 | 0 | 384 | 394 | Carbamidomethylation | C6:Carbamidomethylation:1000.00 | PEAKS DB |
| total 1 peptides |  |  |  |  |  |  |  |  |  |  |  |  |  |  |  |  |  |  |  |
| --- | --- | --- | --- | --- | --- | --- | --- | --- | --- | --- | --- | --- | --- | --- | --- | --- | --- | --- | --- |

XP\_026569147.1

back to list

  

| Protein Coverage
| Supporting Peptides
|

Protein Coverage:

Supporting Peptides:

| Peptide | Uniq | -10lgP | Mass | Length | ppm | m/z | z | RT | Fraction | Scan | Source File | Area BRom | #Feature | #Feature BRom | Start | End | PTM | AScore | Found By |
| --- | --- | --- | --- | --- | --- | --- | --- | --- | --- | --- | --- | --- | --- | --- | --- | --- | --- | --- | --- |
| D.LVVGLC(+57.02)TGQIK.T | Y | 39.69 | 1186.6743 | 11 | 1.1 | 594.3451 | 2 | 36.12 | 1 | 20687 | 01122020\_RID\_2209\_BuCaKA02.raw | 0 | 0 | 0 | 384 | 394 | Carbamidomethylation | C6:Carbamidomethylation:1000.00 | PEAKS DB |
| total 1 peptides |  |  |  |  |  |  |  |  |  |  |  |  |  |  |  |  |  |  |  |
| --- | --- | --- | --- | --- | --- | --- | --- | --- | --- | --- | --- | --- | --- | --- | --- | --- | --- | --- | --- |

JAA97809.1

back to list

  

| Protein Coverage
| Supporting Peptides
|

Protein Coverage:

Supporting Peptides:

| Peptide | Uniq | -10lgP | Mass | Length | ppm | m/z | z | RT | Fraction | Scan | Source File | Area BRom | #Feature | #Feature BRom | Start | End | PTM | AScore | Found By |
| --- | --- | --- | --- | --- | --- | --- | --- | --- | --- | --- | --- | --- | --- | --- | --- | --- | --- | --- | --- |
| D.LVVGLC(+57.02)TGQIK.T | Y | 39.69 | 1186.6743 | 11 | 1.1 | 594.3451 | 2 | 36.12 | 1 | 20687 | 01122020\_RID\_2209\_BuCaKA02.raw | 0 | 0 | 0 | 384 | 394 | Carbamidomethylation | C6:Carbamidomethylation:1000.00 | PEAKS DB |
| total 1 peptides |  |  |  |  |  |  |  |  |  |  |  |  |  |  |  |  |  |  |  |
| --- | --- | --- | --- | --- | --- | --- | --- | --- | --- | --- | --- | --- | --- | --- | --- | --- | --- | --- | --- |

XP\_026542044.1

back to list

  

| Protein Coverage
| Supporting Peptides
|

Protein Coverage:

Supporting Peptides:

| Peptide | Uniq | -10lgP | Mass | Length | ppm | m/z | z | RT | Fraction | Scan | Source File | Area BRom | #Feature | #Feature BRom | Start | End | PTM | AScore | Found By |
| --- | --- | --- | --- | --- | --- | --- | --- | --- | --- | --- | --- | --- | --- | --- | --- | --- | --- | --- | --- |
| D.LVVGLC(+57.02)TGQIK.T | Y | 39.69 | 1186.6743 | 11 | 1.1 | 594.3451 | 2 | 36.12 | 1 | 20687 | 01122020\_RID\_2209\_BuCaKA02.raw | 0 | 0 | 0 | 384 | 394 | Carbamidomethylation | C6:Carbamidomethylation:1000.00 | PEAKS DB |
| total 1 peptides |  |  |  |  |  |  |  |  |  |  |  |  |  |  |  |  |  |  |  |
| --- | --- | --- | --- | --- | --- | --- | --- | --- | --- | --- | --- | --- | --- | --- | --- | --- | --- | --- | --- |

AFJ49385.1

back to list

  

| Protein Coverage
| Supporting Peptides
|

Protein Coverage:

Supporting Peptides:

| Peptide | Uniq | -10lgP | Mass | Length | ppm | m/z | z | RT | Fraction | Scan | Source File | Area BRom | #Feature | #Feature BRom | Start | End | PTM | AScore | Found By |
| --- | --- | --- | --- | --- | --- | --- | --- | --- | --- | --- | --- | --- | --- | --- | --- | --- | --- | --- | --- |
| D.LVVGLC(+57.02)TGQIK.T | Y | 39.69 | 1186.6743 | 11 | 1.1 | 594.3451 | 2 | 36.12 | 1 | 20687 | 01122020\_RID\_2209\_BuCaKA02.raw | 0 | 0 | 0 | 384 | 394 | Carbamidomethylation | C6:Carbamidomethylation:1000.00 | PEAKS DB |
| total 1 peptides |  |  |  |  |  |  |  |  |  |  |  |  |  |  |  |  |  |  |  |
| --- | --- | --- | --- | --- | --- | --- | --- | --- | --- | --- | --- | --- | --- | --- | --- | --- | --- | --- | --- |

XP\_026544503.1

back to list

  

| Protein Coverage
| Supporting Peptides
|

Protein Coverage:

Supporting Peptides:

| Peptide | Uniq | -10lgP | Mass | Length | ppm | m/z | z | RT | Fraction | Scan | Source File | Area BRom | #Feature | #Feature BRom | Start | End | PTM | AScore | Found By |
| --- | --- | --- | --- | --- | --- | --- | --- | --- | --- | --- | --- | --- | --- | --- | --- | --- | --- | --- | --- |
| D.LVVGLC(+57.02)TGQIK.T | Y | 39.69 | 1186.6743 | 11 | 1.1 | 594.3451 | 2 | 36.12 | 1 | 20687 | 01122020\_RID\_2209\_BuCaKA02.raw | 0 | 0 | 0 | 384 | 394 | Carbamidomethylation | C6:Carbamidomethylation:1000.00 | PEAKS DB |
| total 1 peptides |  |  |  |  |  |  |  |  |  |  |  |  |  |  |  |  |  |  |  |
| --- | --- | --- | --- | --- | --- | --- | --- | --- | --- | --- | --- | --- | --- | --- | --- | --- | --- | --- | --- |

JAC96284.1

back to list

  

| Protein Coverage
| Supporting Peptides
|

Protein Coverage:

Supporting Peptides:

| Peptide | Uniq | -10lgP | Mass | Length | ppm | m/z | z | RT | Fraction | Scan | Source File | Area BRom | #Feature | #Feature BRom | Start | End | PTM | AScore | Found By |
| --- | --- | --- | --- | --- | --- | --- | --- | --- | --- | --- | --- | --- | --- | --- | --- | --- | --- | --- | --- |
| D.LVVGLC(+57.02)TGQIK.T | Y | 39.69 | 1186.6743 | 11 | 1.1 | 594.3451 | 2 | 36.12 | 1 | 20687 | 01122020\_RID\_2209\_BuCaKA02.raw | 0 | 0 | 0 | 384 | 394 | Carbamidomethylation | C6:Carbamidomethylation:1000.00 | PEAKS DB |
| total 1 peptides |  |  |  |  |  |  |  |  |  |  |  |  |  |  |  |  |  |  |  |
| --- | --- | --- | --- | --- | --- | --- | --- | --- | --- | --- | --- | --- | --- | --- | --- | --- | --- | --- | --- |

JAB54295.1

back to list

  

| Protein Coverage
| Supporting Peptides
|

Protein Coverage:

Supporting Peptides:

| Peptide | Uniq | -10lgP | Mass | Length | ppm | m/z | z | RT | Fraction | Scan | Source File | Area BRom | #Feature | #Feature BRom | Start | End | PTM | AScore | Found By |
| --- | --- | --- | --- | --- | --- | --- | --- | --- | --- | --- | --- | --- | --- | --- | --- | --- | --- | --- | --- |
| D.LVVGLC(+57.02)TGQIK.T | Y | 39.69 | 1186.6743 | 11 | 1.1 | 594.3451 | 2 | 36.12 | 1 | 20687 | 01122020\_RID\_2209\_BuCaKA02.raw | 0 | 0 | 0 | 384 | 394 | Carbamidomethylation | C6:Carbamidomethylation:1000.00 | PEAKS DB |
| total 1 peptides |  |  |  |  |  |  |  |  |  |  |  |  |  |  |  |  |  |  |  |
| --- | --- | --- | --- | --- | --- | --- | --- | --- | --- | --- | --- | --- | --- | --- | --- | --- | --- | --- | --- |

JAI14387.1

back to list

  

| Protein Coverage
| Supporting Peptides
|

Protein Coverage:

Supporting Peptides:

| Peptide | Uniq | -10lgP | Mass | Length | ppm | m/z | z | RT | Fraction | Scan | Source File | Area BRom | #Feature | #Feature BRom | Start | End | PTM | AScore | Found By |
| --- | --- | --- | --- | --- | --- | --- | --- | --- | --- | --- | --- | --- | --- | --- | --- | --- | --- | --- | --- |
| D.LVVGLC(+57.02)TGQIK.T | Y | 39.69 | 1186.6743 | 11 | 1.1 | 594.3451 | 2 | 36.12 | 1 | 20687 | 01122020\_RID\_2209\_BuCaKA02.raw | 0 | 0 | 0 | 384 | 394 | Carbamidomethylation | C6:Carbamidomethylation:1000.00 | PEAKS DB |
| total 1 peptides |  |  |  |  |  |  |  |  |  |  |  |  |  |  |  |  |  |  |  |
| --- | --- | --- | --- | --- | --- | --- | --- | --- | --- | --- | --- | --- | --- | --- | --- | --- | --- | --- | --- |

XP\_007420481.1

back to list

  

| Protein Coverage
| Supporting Peptides
|

Protein Coverage:

Supporting Peptides:

| Peptide | Uniq | -10lgP | Mass | Length | ppm | m/z | z | RT | Fraction | Scan | Source File | Area BRom | #Feature | #Feature BRom | Start | End | PTM | AScore | Found By |
| --- | --- | --- | --- | --- | --- | --- | --- | --- | --- | --- | --- | --- | --- | --- | --- | --- | --- | --- | --- |
| D.LVVGLC(+57.02)TGQIK.T | Y | 39.69 | 1186.6743 | 11 | 1.1 | 594.3451 | 2 | 36.12 | 1 | 20687 | 01122020\_RID\_2209\_BuCaKA02.raw | 0 | 0 | 0 | 384 | 394 | Carbamidomethylation | C6:Carbamidomethylation:1000.00 | PEAKS DB |
| total 1 peptides |  |  |  |  |  |  |  |  |  |  |  |  |  |  |  |  |  |  |  |
| --- | --- | --- | --- | --- | --- | --- | --- | --- | --- | --- | --- | --- | --- | --- | --- | --- | --- | --- | --- |

JAV51289.1

back to list

  

| Protein Coverage
| Supporting Peptides
|

Protein Coverage:

Supporting Peptides:

| Peptide | Uniq | -10lgP | Mass | Length | ppm | m/z | z | RT | Fraction | Scan | Source File | Area BRom | #Feature | #Feature BRom | Start | End | PTM | AScore | Found By |
| --- | --- | --- | --- | --- | --- | --- | --- | --- | --- | --- | --- | --- | --- | --- | --- | --- | --- | --- | --- |
| D.LVVGLC(+57.02)TGQIK.T | Y | 39.69 | 1186.6743 | 11 | 1.1 | 594.3451 | 2 | 36.12 | 1 | 20687 | 01122020\_RID\_2209\_BuCaKA02.raw | 0 | 0 | 0 | 384 | 394 | Carbamidomethylation | C6:Carbamidomethylation:1000.00 | PEAKS DB |
| total 1 peptides |  |  |  |  |  |  |  |  |  |  |  |  |  |  |  |  |  |  |  |
| --- | --- | --- | --- | --- | --- | --- | --- | --- | --- | --- | --- | --- | --- | --- | --- | --- | --- | --- | --- |

ETE72098.1

back to list

  

| Protein Coverage
| Supporting Peptides
|

Protein Coverage:

Supporting Peptides:

| Peptide | Uniq | -10lgP | Mass | Length | ppm | m/z | z | RT | Fraction | Scan | Source File | Area BRom | #Feature | #Feature BRom | Start | End | PTM | AScore | Found By |
| --- | --- | --- | --- | --- | --- | --- | --- | --- | --- | --- | --- | --- | --- | --- | --- | --- | --- | --- | --- |
| D.LVVGLC(+57.02)TGQIK.T | Y | 39.69 | 1186.6743 | 11 | 1.1 | 594.3451 | 2 | 36.12 | 1 | 20687 | 01122020\_RID\_2209\_BuCaKA02.raw | 0 | 0 | 0 | 422 | 432 | Carbamidomethylation | C6:Carbamidomethylation:1000.00 | PEAKS DB |
| total 1 peptides |  |  |  |  |  |  |  |  |  |  |  |  |  |  |  |  |  |  |  |
| --- | --- | --- | --- | --- | --- | --- | --- | --- | --- | --- | --- | --- | --- | --- | --- | --- | --- | --- | --- |

P81236.1

back to list

  

| Protein Coverage
| Supporting Peptides
|

Protein Coverage:

Supporting Peptides:

| Peptide | Uniq | -10lgP | Mass | Length | ppm | m/z | z | RT | Fraction | Scan | Source File | Area BRom | #Feature | #Feature BRom | Start | End | PTM | AScore | Found By |
| --- | --- | --- | --- | --- | --- | --- | --- | --- | --- | --- | --- | --- | --- | --- | --- | --- | --- | --- | --- |
| DLFQFGGMIGC(+57.02)ANKGARSWLSYVNYGC(+57.02)YC(+57.02)GWGGSGTPVDELDR.C | Y | 39.20 | 4820.1304 | 43 | 8.4 | 1206.0500 | 4 | 61.66 | 1 | 40689 | 01122020\_RID\_2209\_BuCaKA02.raw | 0 | 0 | 0 | 1 | 43 | Carbamidomethylation | C11:Carbamidomethylation:1000.00;C27:Carbamidomethylation:1000.00;C29:Carbamidomethylation:1000.00 | PEAKS DB |
| total 1 peptides |  |  |  |  |  |  |  |  |  |  |  |  |  |  |  |  |  |  |  |
| --- | --- | --- | --- | --- | --- | --- | --- | --- | --- | --- | --- | --- | --- | --- | --- | --- | --- | --- | --- |

Peptide List

  
  

---

Prepared with PEAKS ™ (bioinfor.com)
