# Supplementary material for: A Wolf in Another Wolf’s Clothing: Post-Genomic Regulation Dictates Venom Profiles of Medically-Important Cryptic Kraits in India
Source: Toxins (Basel). 2021 Jan 19;13(1):69. doi: 10.3390/toxins13010069 (PMC7832344; doi:10.3390/toxins13010069)
Supplement: Supplementary file 1 [file toxins-13-00069-s001.zip › File S3.zip!/B. sindanus/B. sindanus.html]

proteins


Summary

  

# 1. Notes

# 2. Result Statistics

**Figure 1.**
False discovery rate (FDR) curve. X axis is the number of peptide-spectrum matches (PSM) being kept. Y axis is the corresponding FDR.

  


  

**Figure 2.**
PSM score distribution.  **(a)**
Distribution of PEAKS peptide score;  **(b)**
Scatterplot of PEAKS peptide score versus precursor mass error.

**(a)**

**(b)**

**Table 1.**
Statistics of data.


|  |  |  |  |  |  |  |  |  |  |  |  |
| --- | --- | --- | --- | --- | --- | --- | --- | --- | --- | --- | --- |
|  | #Scans | | #Features | Identified | | | #Peptides | #Sequences | #Proteins^\*^ | | |
|  | MS1 | MS2 |  | #PSMs | #Scans | #Features |  |  | Groups | All | Top |
| Total | 12271 | 64809 | 95736 | 1581 | 1560 | 11993 | 534 | 506 | 72 | 418 | 247 |
| BuCaMH03\_B17 | 12271 | 64809 | 95736 | 1581 | 1560 | 11993 | 534 | 506 | 72 | 418 | 247 |

^\* proteins with significant peptides are used in counts.^

**Figure 3.**
Sample overlap for Proteins and Peptides (up to 8 samples).  **(a)**
All Proteins;  **(b)**
Top Proteins;  **(c)**
Peptides;

**(a)**

**Not applicable to only one sample**

**(b)**

**Not applicable to only one sample**

**(c)**

**Not applicable to only one sample**

**Figure 4.**
Distribution of peptide feature detection.  **(a)**
Feature m/z distribution;  **(b)**
Feature RT distribution.

**(a)**

**(b)**

**Figure 5.**
Distribution of identified peptide features.  **(a)**
Feature abundance distribution;  **(b)**
 *De novo*
sequencing validation.

**(a)**

**(b)**

**Table 2.**
Result filtration parameters.

|  |  |
| --- | --- |
| Peptide -10lgP | ≥53 |
| PTM Ascore | ≥0 |
| Protein -10lgP | ≥20 |
| Proteins unique peptides | ≥1 |
| De novo score(%) | ≥50% |


**Table 3.**
Statistics of filtered result.

|  |  |
| --- | --- |
| FDR (Peptide-Spectrum Matches) | 0.1% |
| FDR (Peptide Sequences) | 0.2% |
| FDR (Protein Group) | 1.4% |
| De Novo Only Spectra | 17288 |

**Table 4.**
PTM profile.

| Name | ∆Mass | Position | #PSM | -10lgP | Abundance | AScore || Carbamidomethyl | 57.02 | C | 941 | 122.66 | 7.29E8 | 1000.00 |
| Oxidation | 15.99 | M | 121 | 97.35 | 1.42E7 | 91.37 |

# 3. Experiment Control

**Figure 6.**
Precursor mass error of peptide-spectrum matches (PSM) in filtered result.  **(a)**
Distribution of precursor mass error in ppm;  **(b)**
Scatterplot of precursor m/z versus precursor mass error in ppm.

**(a)**

**(b)**

**Table 5.**
Number of identified peptides in each sample by the number of missed cleavages.

|  |  |  |  |  |  |
| --- | --- | --- | --- | --- | --- |
| Missed Cleavages | 0 | 1 | 2 | 3 | 4+ |
| BuCaMH03\_B17 | 456 | 71 | 7 | 0 | 0 |

# 4. Other Information

**Table 6.**
Search parameters.

Search Engine Name: PEAKS  
Parent Mass Error Tolerance: 10.0 ppm  
Fragment Mass Error Tolerance: 0.6 Da  
Precursor Mass Search Type: monoisotopic  
Enzyme: Trypsin  
Max Missed Cleavages: 2  
Digest Mode: Semispecific  
Fixed Modifications:   
  Carbamidomethylation: 57.02  
Variable Modifications:   
  Oxidation (M): 15.99  
Max Variable PTM Per Peptide: 3  
Database: NCBI\_Serpentes\_BuCaMH03  
Taxon: All  
Contaminant Database: cRAP\_contaminants  
Searched Entry: 410252  
FDR Estimation: Enabled  
Merge Options: no merge  
Precursor Options: corrected  
Charge Options: no correction  
Filter Charge: 2 - 8  
Process: true  
Associate chimera: yes

**Table 7.**
Instrument parameters.

Fractions: BuCaMH03.raw  
Ion Source: ESI(nano-spray)  
Fragmentation Mode: CID, CAD(y and b ions)  
MS Scan Mode: FT-ICR/Orbitrap  
MS/MS Scan Mode: FT-ICR/Orbitrap

  

Protein List

  

Protein Accession Contains:

Protein Description Contains:

Protein Sample Area >=

Protein PTM Contains:

| Protein Group | Protein ID | Accession | -10lgP | Coverage (%) | Coverage (%) BuCaMH03\_B17 | Area BuCaMH03\_B17 | #Peptides | #Unique | #Spec BuCaMH03\_B17 | PTM | Avg. Mass | Description |
| --- | --- | --- | --- | --- | --- | --- | --- | --- | --- | --- | --- | --- |
| 10 | 1 | T\_169 | 452.57 | 64 | 64 | 1.082E8 | 52 | 6 | 117 | Y | 64765 | T\_169 |
| 10 | 2 | T\_163 | 452.57 | 60 | 60 | 1.082E8 | 52 | 6 | 117 | Y | 69045 | T\_163 |
| 10 | 10 | T\_161 | 452.57 | 55 | 55 | 1.082E8 | 52 | 6 | 117 | Y | 74793 | T\_161 |
| 10 | 11 | T\_167 | 452.57 | 54 | 54 | 1.082E8 | 52 | 6 | 117 | Y | 77947 | T\_167 |
| 11 | 3 | T\_170 | 448.54 | 59 | 59 | 1.3777E8 | 49 | 3 | 114 | Y | 67886 | T\_170 |
| 11 | 5 | T\_168 | 448.54 | 59 | 59 | 1.3777E8 | 49 | 3 | 114 | Y | 68170 | T\_168 |
| 11 | 7 | T\_171 | 448.54 | 59 | 59 | 1.3777E8 | 49 | 3 | 114 | Y | 68170 | T\_171 |
| 11 | 8 | T\_166 | 448.54 | 60 | 60 | 1.3777E8 | 49 | 3 | 114 | Y | 67340 | T\_166 |
| 5 | 32 | T\_48 | 429.30 | 69 | 69 | 3.9741E9 | 34 | 10 | 154 | Y | 26263 | T\_48 |
| 5 | 33 | T\_44 | 429.30 | 58 | 58 | 3.9741E9 | 34 | 10 | 154 | Y | 31189 | T\_44 |
| 4 | 138 | T\_42 | 421.05 | 69 | 69 | 1.2985E10 | 38 | 37 | 178 | Y | 17261 | T\_42 |
| 15 | 109 | ACE73578.1 | 377.32 | 44 | 44 | 3.6678E7 | 19 | 1 | 106 | Y | 26443 | cysteine-rich seceretory protein Bc-CRPa [Bungarus candidus] |
| 8 | 270 | T\_127 | 361.32 | 51 | 51 | 9.6016E9 | 24 | 10 | 117 | Y | 15485 | T\_127 |
| 3 | 232 | T\_32 | 343.33 | 54 | 54 | 6.0057E8 | 21 | 3 | 179 | Y | 14900 | T\_32 |
| 7 | 153 | T\_39 | 338.21 | 73 | 73 | 7.6555E9 | 24 | 23 | 91 | Y | 16664 | T\_39 |
| 27 | 13 | T\_130 | 336.02 | 41 | 41 | 3.506E6 | 23 | 2 | 31 | Y | 62867 | T\_130 |
| 27 | 14 | T\_131 | 336.02 | 41 | 41 | 3.506E6 | 23 | 2 | 31 | Y | 62867 | T\_131 |
| 28 | 15 | T\_128 | 330.74 | 41 | 41 | 9.3511E5 | 22 | 1 | 30 | Y | 62831 | T\_128 |
| 28 | 16 | T\_129 | 330.74 | 41 | 41 | 9.3511E5 | 22 | 1 | 30 | Y | 62831 | T\_129 |
| 14 | 26 | T\_6 | 311.20 | 22 | 22 | 1.915E7 | 21 | 2 | 99 | Y | 68837 | T\_6 |
| 23 | 290 | T\_33 | 310.13 | 87 | 87 | 1.0325E9 | 14 | 7 | 35 | Y | 9799 | T\_33 |
| 24 | 28 | ABN72537.1 | 304.60 | 32 | 32 | 1.2597E8 | 21 | 3 | 26 | Y | 68988 | metalloproteinase [Bungarus multicinctus] |
| 24 | 29 | A8QL49.1 | 304.60 | 32 | 32 | 1.2597E8 | 21 | 3 | 26 | Y | 68988 | RecName: Full=Zinc metalloproteinase-disintegrin-like BmMP; AltName: Full=Snake venom metalloproteinase; Short=SVMP; Flags: Precursor |
| 33 | 125 | T\_11 | 301.36 | 27 | 27 | 7.5173E6 | 12 | 2 | 19 | Y | 31495 | T\_11 |
| 19 | 686 | T\_81 | 300.23 | 42 | 42 | 2.4212E10 | 19 | 14 | 59 | Y | 11656 | T\_81 |
| 26 | 412 | P15816.1 | 297.69 | 43 | 43 | 1.6077E9 | 10 | 1 | 29 | Y | 9517 | RecName: Full=Kappa-2-bungarotoxin; AltName: Full=Kappa-neurotoxin CB1; Flags: Precursor |
| 26 | 413 | CAA35774.1 | 297.69 | 43 | 43 | 1.6077E9 | 10 | 1 | 29 | Y | 9517 | unnamed protein product [Bungarus multicinctus] |
| 29 | 36 | T\_111 | 287.68 | 26 | 26 | 1.1285E7 | 16 | 5 | 25 | Y | 54560 | T\_111 |
| 32 | 21 | T\_144 | 281.36 | 30 | 30 | 8.5108E6 | 13 | 3 | 14 | Y | 64593 | T\_144 |
| 32 | 22 | T\_146 | 281.36 | 30 | 30 | 8.5108E6 | 13 | 3 | 14 | Y | 64593 | T\_146 |
| 32 | 23 | T\_147 | 281.36 | 30 | 30 | 8.5108E6 | 13 | 3 | 14 | Y | 64593 | T\_147 |
| 32 | 24 | T\_143 | 281.36 | 30 | 30 | 8.5108E6 | 13 | 3 | 14 | Y | 64593 | T\_143 |
| 9 | 261 | 0402253A | 256.44 | 38 | 38 | 0 | 10 | 1 | 116 | Y | 13561 | toxin RCM A,beta1 bungaro |
| 9 | 309 | 0412250A | 256.44 | 38 | 38 | 0 | 10 | 1 | 116 | Y | 13489 | toxin A,beta1 bungaro |
| 40 | 301 | T\_12 | 255.57 | 31 | 31 | 9.7094E7 | 7 | 6 | 12 | Y | 21438 | T\_12 |
| 48 | 66 | XP\_026559083.1 | 248.31 | 7 | 7 | 4.7046E3 | 5 | 1 | 7 | N | 88597 | sulfhydryl oxidase 1 [Pseudonaja textilis] |
| 35 | 118 | T\_16 | 227.52 | 24 | 24 | 5.7608E7 | 7 | 1 | 12 | Y | 25774 | T\_16 |
| 22 | 1663 | CAP74383.1 | 224.26 | 20 | 20 | 2.8949E9 | 4 | 3 | 43 | Y | 9227 | protease inhibitor-like protein 3 precursor [Bungarus multicinctus] |
| 22 | 1664 | B4ESA4.1 | 224.26 | 20 | 20 | 2.8949E9 | 4 | 3 | 43 | Y | 9227 | RecName: Full=Kunitz-type serine protease inhibitor PILP-3; AltName: Full=Protease inhibitor-like protein 3; Flags: Precursor |
| 66 | 74 | XP\_026571757.1 | 223.00 | 24 | 24 | 2.3295E7 | 5 | 5 | 7 | Y | 37548 | cathepsin B [Pseudonaja textilis] |
| 43 | 472 | XP\_026555793.1 | 221.30 | 14 | 14 | 5.4434E7 | 8 | 5 | 14 | Y | 34167 | ADP-ribosyl cyclase/cyclic ADP-ribose hydrolase 1-like [Pseudonaja textilis] |
| 38 | 451 | AAL30054.1 | 219.26 | 57 | 57 | 4.4559E8 | 8 | 6 | 17 | Y | 9580 | kappa 1a bungaratoxin [Bungarus candidus] |
| 38 | 452 | Q8AY56.1 | 219.26 | 57 | 57 | 4.4559E8 | 8 | 6 | 17 | Y | 9580 | RecName: Full=Kappa 1a-bungarotoxin; Flags: Precursor |
| 59 | 111 | ETE59846.1 | 213.65 | 15 | 15 | 8.1876E6 | 9 | 5 | 11 | Y | 72383 | Alpha-fetoprotein, partial [Ophiophagus hannah] |
| 61 | 60 | XP\_034296605.1 | 209.17 | 9 | 9 | 1.3683E6 | 6 | 1 | 6 | N | 106081 | endoplasmic reticulum aminopeptidase 1 isoform X1 [Pantherophis guttatus] |
| 61 | 61 | XP\_034296606.1 | 209.17 | 9 | 9 | 1.3683E6 | 6 | 1 | 6 | N | 106081 | endoplasmic reticulum aminopeptidase 1 isoform X1 [Pantherophis guttatus] |
| 61 | 62 | XP\_034296607.1 | 209.17 | 10 | 10 | 1.3683E6 | 6 | 1 | 6 | N | 98369 | endoplasmic reticulum aminopeptidase 1 isoform X2 [Pantherophis guttatus] |
| 67 | 64 | XP\_026570202.1 | 207.56 | 7 | 7 | 2.7819E6 | 6 | 6 | 6 | Y | 133145 | Golgi apparatus protein 1 [Pseudonaja textilis] |
| 67 | 65 | XP\_026535930.1 | 207.56 | 7 | 7 | 2.7819E6 | 6 | 6 | 6 | Y | 132789 | Golgi apparatus protein 1 [Notechis scutatus] |
| 41 | 586 | T\_22 | 203.81 | 56 | 56 | 5.4547E8 | 4 | 4 | 12 | Y | 10626 | T\_22 |
| 37 | 557 | Q9YGI8.1 | 200.58 | 35 | 35 | 1.6622E9 | 5 | 5 | 12 | Y | 9519 | RecName: Full=Short neurotoxin homolog NTL4; Flags: Precursor |
| 37 | 558 | CAA06887.1 | 200.58 | 35 | 35 | 1.6622E9 | 5 | 5 | 12 | Y | 9519 | neurotoxin-like protein [Bungarus multicinctus multicinctus] |
| 74 | 82 | XP\_026521896.1 | 199.72 | 6 | 6 | 7.8843E5 | 4 | 1 | 4 | Y | 96900 | ectonucleotide pyrophosphatase/phosphodiesterase family member 3 isoform X2 [Notechis scutatus] |
| 74 | 83 | XP\_026521895.1 | 199.72 | 6 | 6 | 7.8843E5 | 4 | 1 | 4 | Y | 100962 | ectonucleotide pyrophosphatase/phosphodiesterase family member 3 isoform X1 [Notechis scutatus] |
| 44 | 1252 | T\_176 | 197.44 | 38 | 38 | 7.226E8 | 4 | 4 | 10 | Y | 8627 | T\_176 |
| 84 | 80 | T\_83 | 192.46 | 16 | 16 | 4.1885E6 | 6 | 6 | 6 | N | 64069 | T\_83 |
| 77 | 194 | XP\_034277309.1 | 182.52 | 10 | 10 | 2.5945E6 | 6 | 1 | 7 | Y | 54304 | hyaluronidase [Pantherophis guttatus] |
| 76 | 395 | XP\_034264771.1 | 175.61 | 15 | 15 | 1.4301E7 | 5 | 2 | 8 | Y | 34352 | ADP-ribosyl cyclase/cyclic ADP-ribose hydrolase 1-like isoform X1 [Pantherophis guttatus] |
| 36 | 328 | T\_158 | 174.15 | 33 | 33 | 3.9784E8 | 6 | 6 | 6 | Y | 22464 | T\_158 |
| 36 | 329 | T\_153 | 174.15 | 33 | 33 | 3.9784E8 | 6 | 6 | 6 | Y | 22464 | T\_153 |
| 36 | 330 | T\_157 | 174.15 | 30 | 30 | 3.9784E8 | 6 | 6 | 6 | Y | 24614 | T\_157 |
| 36 | 331 | T\_151 | 174.15 | 30 | 30 | 3.9784E8 | 6 | 6 | 6 | Y | 25392 | T\_151 |
| 36 | 332 | T\_152 | 174.15 | 30 | 30 | 3.9784E8 | 6 | 6 | 6 | Y | 25392 | T\_152 |
| 47 | 1253 | T\_175 | 167.03 | 30 | 30 | 2.4148E8 | 2 | 2 | 14 | Y | 8565 | T\_175 |
| 82 | 221 | AHJ80886.1 | 165.14 | 5 | 5 | 2.6873E6 | 2 | 1 | 2 | Y | 45031 | 5'-nucleotidase, partial [Macrovipera lebetina] |
| 80 | 703 | T\_132 | 164.12 | 31 | 31 | 7.7006E6 | 2 | 2 | 3 | Y | 9276 | T\_132 |
| 73 | 1064 | Q9W729.1 | 163.69 | 16 | 16 | 3.3205E8 | 2 | 2 | 7 | Y | 9657 | RecName: Full=Kappa-6-bungarotoxin; Short=K-6 bungarotoxin; Flags: Precursor |
| 73 | 1065 | CAB46659.1 | 163.69 | 16 | 16 | 3.3205E8 | 2 | 2 | 7 | Y | 9657 | k-6 bungarotoxin [Bungarus multicinctus] |
| 90 | 198 | T\_102 | 161.71 | 26 | 26 | 1.9147E6 | 4 | 2 | 4 | Y | 31778 | T\_102 |
| 31 | 1608 | ETE56933.1 | 159.08 | 9 | 9 | 5.9738E8 | 3 | 3 | 19 | Y | 15656 | hypothetical protein L345\_17355, partial [Ophiophagus hannah] |
| 31 | 1667 | 1IJC | 159.08 | 19 | 19 | 5.9738E8 | 3 | 3 | 19 | Y | 7285 | Chain A, Solution Structure Of Bucandin, A Neurotoxin From The Venom Of The Malayan Krait |
| 31 | 1668 | 1F94 | 159.08 | 19 | 19 | 5.9738E8 | 3 | 3 | 19 | Y | 7285 | Chain A, Bucandin |
| 31 | 1669 | P81782.1 | 159.08 | 19 | 19 | 5.9738E8 | 3 | 3 | 19 | Y | 7285 | RecName: Full=Bucandin |
| 92 | 313 | ABN72545.1 | 158.73 | 16 | 16 | 7.7512E5 | 3 | 1 | 3 | Y | 31010 | putative serine protease, partial [Bungarus multicinctus] |
| 92 | 314 | A8QL57.1 | 158.73 | 16 | 16 | 7.7512E5 | 3 | 1 | 3 | Y | 31010 | RecName: Full=Snake venom serine protease BmSP; Short=SVSP; Flags: Precursor |
| 87 | 267 | XP\_026555558.1 | 158.55 | 17 | 17 | 2.2263E6 | 5 | 3 | 6 | Y | 61349 | hepatocyte growth factor activator [Pseudonaja textilis] |
| 53 | 587 | T\_54 | 157.51 | 30 | 30 | 1.863E8 | 3 | 3 | 6 | Y | 9748 | T\_54 |
| 78 | 1670 | Q8AY42.2 | 157.20 | 17 | 17 | 4.697E8 | 3 | 2 | 10 | Y | 8919 | RecName: Full=Kunitz-type serine protease inhibitor B; AltName: Full=Kunitz inhibitor B; Flags: Precursor |
| 78 | 1671 | Q8AY43.2 | 157.20 | 17 | 17 | 4.697E8 | 3 | 2 | 10 | Y | 9077 | RecName: Full=Kunitz-type serine protease inhibitor A; AltName: Full=Kunitz inhibitor A; Flags: Precursor |
| 78 | 1672 | AAL30069.1 | 157.20 | 16 | 16 | 4.697E8 | 3 | 2 | 10 | Y | 9391 | Kunitz inhibitor b, partial [Bungarus candidus] |
| 78 | 1673 | AAL30068.1 | 157.20 | 16 | 16 | 4.697E8 | 3 | 2 | 10 | Y | 9606 | Kunitz inhibitor a, partial [Bungarus candidus] |
| 109 | 426 | XP\_026544110.1 | 150.69 | 13 | 13 | 6.3406E6 | 3 | 1 | 3 | Y | 50196 | hepatocyte growth factor activator [Notechis scutatus] |
| 102 | 945 | P41331.1 | 142.59 | 27 | 27 | 6.0532E5 | 3 | 1 | 4 | N | 15437 | RecName: Full=Hemoglobin subunit alpha; AltName: Full=Alpha-globin; AltName: Full=Hemoglobin alpha chain |
| 96 | 213 | ETE59912.1 | 139.66 | 7 | 7 | 1.9025E6 | 3 | 3 | 3 | N | 67365 | 78 kDa glucose-regulated protein [Ophiophagus hannah] |
| 96 | 200 | XP\_034262421.1 | 139.66 | 6 | 6 | 1.9025E6 | 3 | 3 | 3 | N | 68299 | endoplasmic reticulum chaperone BiP, partial [Pantherophis guttatus] |
| 96 | 201 | JAG67806.1 | 139.66 | 6 | 6 | 1.9025E6 | 3 | 3 | 3 | N | 71941 | 78 kDa glucose-regulated protein [Boiga irregularis] |
| 96 | 214 | XP\_025022112.1 | 139.66 | 6 | 6 | 1.9025E6 | 3 | 3 | 3 | N | 71864 | endoplasmic reticulum chaperone BiP [Python bivittatus] |
| 96 | 215 | JAB54174.1 | 139.66 | 6 | 6 | 1.9025E6 | 3 | 3 | 3 | N | 72106 | glucose-regulated protein [Micrurus fulvius] |
| 96 | 216 | XP\_026568326.1 | 139.66 | 6 | 6 | 1.9025E6 | 3 | 3 | 3 | N | 72187 | endoplasmic reticulum chaperone BiP [Pseudonaja textilis] |
| 96 | 217 | XP\_026542482.1 | 139.66 | 6 | 6 | 1.9025E6 | 3 | 3 | 3 | N | 72286 | endoplasmic reticulum chaperone BiP [Notechis scutatus] |
| 96 | 204 | JAV50419.1 | 139.66 | 6 | 6 | 1.9025E6 | 3 | 3 | 3 | N | 72067 | 78 kDa glucose-regulated protein [Agkistrodon contortrix contortrix] |
| 96 | 205 | JAG44540.1 | 139.66 | 6 | 6 | 1.9025E6 | 3 | 3 | 3 | N | 72105 | 78 kDa glucose-regulated protein precursor [Crotalus horridus] |
| 96 | 206 | JAI13107.1 | 139.66 | 6 | 6 | 1.9025E6 | 3 | 3 | 3 | N | 72105 | 78 kDa glucose-regulated protein [Crotalus adamanteus] |
| 96 | 207 | XP\_015681610.1 | 139.66 | 6 | 6 | 1.9025E6 | 3 | 3 | 3 | N | 72155 | endoplasmic reticulum chaperone BiP [Protobothrops mucrosquamatus] |
| 96 | 208 | AFJ50327.1 | 139.66 | 6 | 6 | 1.9025E6 | 3 | 3 | 3 | N | 72105 | Heat shock protein 5 [Crotalus adamanteus] |
| 96 | 209 | JAG46692.1 | 139.66 | 6 | 6 | 1.9025E6 | 3 | 3 | 3 | N | 72105 | 78 kDa glucose-regulated protein [Crotalus horridus] |
| 96 | 210 | JAA96909.1 | 139.66 | 6 | 6 | 1.9025E6 | 3 | 3 | 3 | N | 72105 | 78 kDa glucose-regulated protein [Crotalus horridus] |
| 96 | 211 | AFJ50239.1 | 139.66 | 6 | 6 | 1.9025E6 | 3 | 3 | 3 | N | 75480 | 78 kDa glucose-regulated protein precursor [Crotalus adamanteus] |
| 98 | 342 | ABH05181.1 | 128.06 | 10 | 10 | 1.5378E6 | 1 | 1 | 1 | N | 18269 | C-type lectin 2 [Bungarus multicinctus] |
| 98 | 343 | A1XXJ9.1 | 128.06 | 10 | 10 | 1.5378E6 | 1 | 1 | 1 | N | 18269 | RecName: Full=C-type lectin BML-2; Short=CTL; Flags: Precursor |
| 112 | 800 | ABU68503.1 | 125.75 | 16 | 16 | 8.6156E5 | 3 | 1 | 3 | Y | 16620 | PLA2(IB)-Tri1 [Trimorphodon biscutatus] |
| 122 | 427 | T\_84 | 118.47 | 15 | 15 | 1.6227E6 | 2 | 2 | 2 | N | 24381 | T\_84 |
| 111 | 1724 | CAM11302.1 | 117.13 | 17 | 17 | 4.2251E7 | 2 | 2 | 2 | Y | 8306 | alpha-delta-bungarotoxin-4, partial [Bungarus caeruleus] |
| 111 | 1725 | D2N116.1 | 117.13 | 17 | 17 | 4.2251E7 | 2 | 2 | 2 | Y | 8306 | RecName: Full=Alpha-delta-bungarotoxin-4; Short=Alpha-delta-Bgt-4; Flags: Precursor |
| 121 | 1677 | ACY68722.1 | 108.97 | 8 | 8 | 9.8406E5 | 1 | 1 | 1 | Y | 20018 | CRISP isoform 1 [Parasuta nigriceps] |
| 99 | 417 | AAB25729.1 | 104.44 | 3 | 3 | 7.1214E6 | 1 | 1 | 1 | N | 27514 | nerve growth factor precursor [Bungarus multicinctus] |
| 99 | 418 | P34128.1 | 104.44 | 3 | 3 | 7.1214E6 | 1 | 1 | 1 | N | 27514 | RecName: Full=Venom nerve growth factor; Short=v-NGF; Short=vNGF; Flags: Precursor |
| 114 | 974 | B4ESA3.1 | 104.39 | 23 | 23 | 6.4804E7 | 2 | 1 | 4 | Y | 9158 | RecName: Full=Kunitz-type serine protease inhibitor PILP-2; AltName: Full=Protease inhibitor-like protein 2; Flags: Precursor |
| 114 | 975 | Q8AY41.2 | 104.39 | 23 | 23 | 6.4804E7 | 2 | 1 | 4 | Y | 9130 | RecName: Full=Kunitz-type serine protease inhibitor C; AltName: Full=Kunitz inhibitor C; Flags: Precursor |
| 114 | 976 | CAP74382.1 | 104.39 | 23 | 23 | 6.4804E7 | 2 | 1 | 4 | Y | 9158 | protease inhibitor-like protein 2 precursor [Bungarus multicinctus] |
| 114 | 977 | AAL30070.1 | 104.39 | 22 | 22 | 6.4804E7 | 2 | 1 | 4 | Y | 9659 | Kunitz inhibitor c, partial [Bungarus candidus] |
| 46 | 1409 | JAA74768.1 | 97.53 | 9 | 9 | 9.3226E6 | 1 | 1 | 1 | Y | 11631 | 3FTx-Ech-35 [Echiopsis curta] |
| 46 | 1410 | JAA74767.1 | 97.53 | 9 | 9 | 9.3226E6 | 1 | 1 | 1 | Y | 11631 | 3FTx-Ech-37 [Echiopsis curta] |
| 46 | 1772 | P01387.1 | 97.53 | 14 | 14 | 9.3226E6 | 1 | 1 | 1 | Y | 8106 | RecName: Full=Long neurotoxin 1; AltName: Full=Neurotoxin A |
| 46 | 1774 | Q53B58.1 | 97.53 | 11 | 11 | 9.3226E6 | 1 | 1 | 1 | Y | 10210 | RecName: Full=Long neurotoxin OH-55; AltName: Full=CM-11; Flags: Precursor |
| 46 | 1775 | AAT97250.1 | 97.53 | 11 | 11 | 9.3226E6 | 1 | 1 | 1 | Y | 10210 | long chain neurotoxin precursor [Ophiophagus hannah] |
| 83 | 1000 | BAC77656.1 | 96.75 | 14 | 14 | 1.0036E8 | 1 | 1 | 4 | Y | 15875 | phospholipase A2II precursor [Bungarus flaviceps] |
| 83 | 1001 | ADF50035.1 | 96.75 | 14 | 14 | 1.0036E8 | 1 | 1 | 4 | Y | 15845 | phospholipase a2 isoform 1 [Bungarus flaviceps] |
| 83 | 1002 | Q7T2Q4.1 | 96.75 | 14 | 14 | 1.0036E8 | 1 | 1 | 4 | Y | 15875 | RecName: Full=Acidic phospholipase A2 2; Short=svPLA2; AltName: Full=PA2-II; AltName: Full=Phosphatidylcholine 2-acylhydrolase; Flags: Precursor |
| 113 | 679 | ACY68711.1 | 87.44 | 12 | 12 | 2.4189E6 | 1 | 1 | 1 | Y | 15455 | phospholipase A2 isoform 2 [Parasuta nigriceps] |
| 164 | 1142 | JAC94986.1 | 82.18 | 14 | 14 | 2.3001E6 | 1 | 1 | 1 | Y | 16723 | Epididymal secretory protein type E1 [Opheodrys aestivus] |
| 133 | 918 | JAA97769.1 | 81.97 | 5 | 5 | 6.5848E5 | 1 | 1 | 1 | N | 38523 | Annexin A2-like protein [Crotalus horridus] |
| 133 | 1051 | JAI10286.1 | 81.97 | 5 | 5 | 6.5848E5 | 1 | 1 | 1 | N | 38532 | Annexin A2-like protein [Micrurus fulvius] |
| 133 | 919 | JAG47471.1 | 81.97 | 5 | 5 | 6.5848E5 | 1 | 1 | 1 | N | 38523 | Annexin A2-like protein [Crotalus horridus] |
| 133 | 1052 | JAB54690.1 | 81.97 | 5 | 5 | 6.5848E5 | 1 | 1 | 1 | N | 38562 | annexin A2 [Micrurus fulvius] |
| 133 | 920 | JAG45828.1 | 81.97 | 5 | 5 | 6.5848E5 | 1 | 1 | 1 | N | 38523 | Annexin A2-like protein [Crotalus horridus] |
| 133 | 921 | JAV51253.1 | 81.97 | 5 | 5 | 6.5848E5 | 1 | 1 | 1 | N | 38542 | Annexin A2-like protein [Agkistrodon contortrix contortrix] |
| 133 | 922 | XP\_007439873.1 | 81.97 | 5 | 5 | 6.5848E5 | 1 | 1 | 1 | N | 38569 | annexin A2 [Python bivittatus] |
| 133 | 923 | LAB40267.1 | 81.97 | 5 | 5 | 6.5848E5 | 1 | 1 | 1 | N | 39471 | hypothetical protein, partial [Micrurus spixii] |
| 133 | 1676 | ETE59687.1 | 81.97 | 7 | 7 | 6.5848E5 | 1 | 1 | 1 | N | 27908 | Annexin A2 [Ophiophagus hannah] |
| 125 | 616 | ETE61271.1 | 80.26 | 3 | 3 | 1.2447E7 | 1 | 1 | 2 | N | 54055 | Proactivator polypeptide [Ophiophagus hannah] |
| 125 | 620 | AFJ51008.1 | 80.26 | 3 | 3 | 1.2447E7 | 1 | 1 | 2 | N | 58013 | Proactivator polypeptide-like [Crotalus adamanteus] |
| 125 | 628 | XP\_032088229.1 | 80.26 | 3 | 3 | 1.2447E7 | 1 | 1 | 2 | N | 58050 | prosaposin-like isoform X2 [Thamnophis elegans] |
| 125 | 624 | XP\_013923763.1 | 80.26 | 3 | 3 | 1.2447E7 | 1 | 1 | 2 | N | 58050 | PREDICTED: prosaposin isoform X2 [Thamnophis sirtalis] |
| 125 | 625 | XP\_013923762.1 | 80.26 | 2 | 2 | 1.2447E7 | 1 | 1 | 2 | N | 58435 | PREDICTED: prosaposin isoform X1 [Thamnophis sirtalis] |
| 125 | 632 | XP\_032088228.1 | 80.26 | 2 | 2 | 1.2447E7 | 1 | 1 | 2 | N | 58435 | prosaposin-like isoform X1 [Thamnophis elegans] |
| 125 | 629 | XP\_015669101.1 | 80.26 | 3 | 3 | 1.2447E7 | 1 | 1 | 2 | N | 57981 | prosaposin [Protobothrops mucrosquamatus] |
| 125 | 630 | BAN82088.1 | 80.26 | 2 | 2 | 1.2447E7 | 1 | 1 | 2 | N | 58564 | hypothetical protein [Protobothrops flavoviridis] |
| 125 | 631 | XP\_034291658.1 | 80.26 | 2 | 2 | 1.2447E7 | 1 | 1 | 2 | N | 58438 | prosaposin-like isoform X4 [Pantherophis guttatus] |
| 125 | 633 | XP\_034291656.1 | 80.26 | 2 | 2 | 1.2447E7 | 1 | 1 | 2 | N | 58696 | prosaposin-like isoform X2 [Pantherophis guttatus] |
| 125 | 634 | XP\_034291657.1 | 80.26 | 2 | 2 | 1.2447E7 | 1 | 1 | 2 | N | 58695 | prosaposin-like isoform X3 [Pantherophis guttatus] |
| 125 | 635 | XP\_034291655.1 | 80.26 | 2 | 2 | 1.2447E7 | 1 | 1 | 2 | N | 58824 | prosaposin-like isoform X1 [Pantherophis guttatus] |
| 125 | 626 | XP\_026569354.1 | 80.26 | 3 | 3 | 1.2447E7 | 1 | 1 | 2 | N | 58289 | prosaposin isoform X2 [Pseudonaja textilis] |
| 125 | 627 | XP\_026569353.1 | 80.26 | 2 | 2 | 1.2447E7 | 1 | 1 | 2 | N | 58674 | prosaposin isoform X1 [Pseudonaja textilis] |
| 125 | 621 | XP\_026528620.1 | 80.26 | 3 | 3 | 1.2447E7 | 1 | 1 | 2 | N | 58373 | prosaposin-like isoform X2 [Notechis scutatus] |
| 125 | 622 | XP\_026528619.1 | 80.26 | 2 | 2 | 1.2447E7 | 1 | 1 | 2 | N | 58759 | prosaposin-like isoform X1 [Notechis scutatus] |
| 125 | 1050 | XP\_007441356.1 | 80.26 | 3 | 3 | 1.2447E7 | 1 | 1 | 2 | N | 42934 | prosaposin-like [Python bivittatus] |
| 116 | 1016 | 1MR6 | 77.20 | 25 | 25 | 1.6747E6 | 1 | 1 | 1 | Y | 7535 | Chain A, neurotoxin |
| 116 | 1017 | CAA06885.1 | 77.20 | 19 | 19 | 1.6747E6 | 1 | 1 | 1 | Y | 9826 | neurotoxin-like protein [Bungarus multicinctus multicinctus] |
| 116 | 1018 | Q9YGJ0.1 | 77.20 | 19 | 19 | 1.6747E6 | 1 | 1 | 1 | Y | 9826 | RecName: Full=Gamma-bungarotoxin; AltName: Full=Long neurotoxin homolog NTL2I; Flags: Precursor |
| 116 | 1019 | AAD41806.1 | 77.20 | 19 | 19 | 1.6747E6 | 1 | 1 | 1 | Y | 9826 | neurotoxin precursor [Bungarus multicinctus] |
| 116 | 1020 | CAA72941.1 | 77.20 | 19 | 19 | 1.6747E6 | 1 | 1 | 1 | Y | 9756 | neurotoxin [Bungarus multicinctus] |
| 116 | 1021 | CAD01082.1 | 77.20 | 19 | 19 | 1.6747E6 | 1 | 1 | 1 | Y | 9826 | gamma-bungarotoxin [Bungarus multicinctus] |
| 116 | 1022 | O12963.1 | 77.20 | 19 | 19 | 1.6747E6 | 1 | 1 | 1 | Y | 9756 | RecName: Full=Long neurotoxin homolog TA-bm16; AltName: Full=Delta-protein; Flags: Precursor |
| 131 | 730 | LAB19298.1 | 74.21 | 4 | 4 | 1.0705E6 | 1 | 1 | 1 | N | 52913 | hypothetical protein, partial [Micrurus spixii] |
| 131 | 731 | LAB19293.1 | 74.21 | 4 | 4 | 1.0705E6 | 1 | 1 | 1 | N | 52927 | hypothetical protein, partial [Micrurus spixii] |
| 131 | 732 | LAB19285.1 | 74.21 | 3 | 3 | 1.0705E6 | 1 | 1 | 1 | N | 54068 | hypothetical protein, partial [Micrurus spixii] |
| 131 | 733 | LAB19295.1 | 74.21 | 3 | 3 | 1.0705E6 | 1 | 1 | 1 | N | 54082 | hypothetical protein, partial [Micrurus spixii] |
| 131 | 729 | LAB19282.1 | 74.21 | 3 | 3 | 1.0705E6 | 1 | 1 | 1 | N | 58575 | hypothetical protein, partial [Micrurus spixii] |
| 131 | 734 | LAB19291.1 | 74.21 | 3 | 3 | 1.0705E6 | 1 | 1 | 1 | N | 60939 | hypothetical protein, partial [Micrurus spixii] |
| 131 | 735 | LAB19280.1 | 74.21 | 3 | 3 | 1.0705E6 | 1 | 1 | 1 | N | 61644 | hypothetical protein, partial [Micrurus spixii] |
| 156 | 1370 | XP\_026576367.1 | 64.67 | 10 | 10 | 2.2177E5 | 1 | 1 | 1 | N | 13998 | histone H2B 1/2/3/4/6-like [Pseudonaja textilis] |
| 156 | 1736 | XP\_026577747.1 | 64.67 | 16 | 16 | 2.2177E5 | 1 | 1 | 1 | N | 9164 | histone H2B 8-like [Pseudonaja textilis] |
| 156 | 1737 | XP\_015675791.1 | 64.67 | 17 | 17 | 2.2177E5 | 1 | 1 | 1 | N | 8685 | histone H2B-like [Protobothrops mucrosquamatus] |
| 156 | 1738 | XP\_015672174.1 | 64.67 | 10 | 10 | 2.2177E5 | 1 | 1 | 1 | N | 13996 | histone H2B 8-like [Protobothrops mucrosquamatus] |
| 156 | 1739 | XP\_026539722.1 | 64.67 | 10 | 10 | 2.2177E5 | 1 | 1 | 1 | N | 13952 | histone H2B 1/2/3/4/6 [Notechis scutatus] |
| 156 | 1740 | LAA79020.1 | 64.67 | 10 | 10 | 2.2177E5 | 1 | 1 | 1 | N | 13980 | hypothetical protein [Micrurus lemniscatus lemniscatus] |
| 156 | 1741 | XP\_026569519.1 | 64.67 | 10 | 10 | 2.2177E5 | 1 | 1 | 1 | N | 13980 | histone H2B 5 [Pseudonaja textilis] |
| 156 | 1742 | XP\_026576364.1 | 64.67 | 10 | 10 | 2.2177E5 | 1 | 1 | 1 | N | 13980 | histone H2B 1/2/3/4/6-like [Pseudonaja textilis] |
| 156 | 1743 | XP\_015678744.1 | 64.67 | 10 | 10 | 2.2177E5 | 1 | 1 | 1 | N | 13980 | histone H2B 5 [Protobothrops mucrosquamatus] |
| 156 | 1744 | XP\_026545427.1 | 64.67 | 10 | 10 | 2.2177E5 | 1 | 1 | 1 | N | 14050 | histone H2B 8-like [Notechis scutatus] |
| 156 | 1745 | XP\_026529646.1 | 64.67 | 10 | 10 | 2.2177E5 | 1 | 1 | 1 | N | 13980 | histone H2B 5 [Notechis scutatus] |
| 156 | 1746 | XP\_013925621.1 | 64.67 | 10 | 10 | 2.2177E5 | 1 | 1 | 1 | N | 14036 | PREDICTED: histone H2B 5 [Thamnophis sirtalis] |
| 156 | 1747 | XP\_026529644.1 | 64.67 | 10 | 10 | 2.2177E5 | 1 | 1 | 1 | N | 13980 | histone H2B 5 [Notechis scutatus] |
| 156 | 1748 | XP\_025022125.1 | 64.67 | 10 | 10 | 2.2177E5 | 1 | 1 | 1 | N | 13980 | histone H2B 5 [Python bivittatus] |
| 156 | 1749 | XP\_026576334.1 | 64.67 | 10 | 10 | 2.2177E5 | 1 | 1 | 1 | N | 14008 | histone H2B 1/2/3/4/6-like [Pseudonaja textilis] |
| 156 | 1750 | XP\_026576366.1 | 64.67 | 10 | 10 | 2.2177E5 | 1 | 1 | 1 | N | 13952 | histone H2B 1/2/3/4/6 [Pseudonaja textilis] |
| 156 | 1751 | XP\_015672062.1 | 64.67 | 10 | 10 | 2.2177E5 | 1 | 1 | 1 | N | 14036 | histone H2B 8-like [Protobothrops mucrosquamatus] |
| 156 | 1752 | XP\_026576365.1 | 64.67 | 10 | 10 | 2.2177E5 | 1 | 1 | 1 | N | 13994 | histone H2B 1/2/3/4/6-like [Pseudonaja textilis] |
| 156 | 1753 | XP\_026536969.1 | 64.67 | 10 | 10 | 2.2177E5 | 1 | 1 | 1 | N | 13944 | histone H2B 8-like [Notechis scutatus] |
| 156 | 1754 | XP\_007429385.1 | 64.67 | 10 | 10 | 2.2177E5 | 1 | 1 | 1 | N | 13994 | histone H2B 1/2/3/4/6-like [Python bivittatus] |
| 156 | 1755 | XP\_026545426.1 | 64.67 | 10 | 10 | 2.2177E5 | 1 | 1 | 1 | N | 14050 | histone H2B 8-like [Notechis scutatus] |
| 156 | 1756 | XP\_015681932.1 | 64.67 | 10 | 10 | 2.2177E5 | 1 | 1 | 1 | N | 14050 | histone H2B 8-like [Protobothrops mucrosquamatus] |
| 156 | 1757 | XP\_026547833.1 | 64.67 | 10 | 10 | 2.2177E5 | 1 | 1 | 1 | N | 14008 | histone H2B 1/2/3/4/6-like [Notechis scutatus] |
| 156 | 1758 | XP\_026539701.1 | 64.67 | 10 | 10 | 2.2177E5 | 1 | 1 | 1 | N | 14008 | histone H2B 1/2/3/4/6-like [Notechis scutatus] |
| 156 | 1759 | XP\_026580816.1 | 64.67 | 10 | 10 | 2.2177E5 | 1 | 1 | 1 | N | 14050 | histone H2B 8-like [Pseudonaja textilis] |
| 156 | 1760 | XP\_015681933.1 | 64.67 | 10 | 10 | 2.2177E5 | 1 | 1 | 1 | N | 14050 | histone H2B 8-like [Protobothrops mucrosquamatus] |
| 156 | 1761 | XP\_026548170.1 | 64.67 | 10 | 10 | 2.2177E5 | 1 | 1 | 1 | N | 13994 | histone H2B 1/2/3/4/6-like [Notechis scutatus] |
| 156 | 1762 | XP\_026569640.1 | 64.67 | 10 | 10 | 2.2177E5 | 1 | 1 | 1 | N | 13980 | histone H2B 5 [Pseudonaja textilis] |
| 156 | 1763 | ETE56555.1 | 64.67 | 10 | 10 | 2.2177E5 | 1 | 1 | 1 | N | 14008 | Histone H2B 1/2/3/4/6, partial [Ophiophagus hannah] |
| 156 | 1764 | XP\_015678747.1 | 64.67 | 10 | 10 | 2.2177E5 | 1 | 1 | 1 | N | 13994 | histone H2B 8 [Protobothrops mucrosquamatus] |
| 137 | 1734 | XP\_007434930.1 | 62.11 | 3 | 3 | 3.3371E5 | 1 | 1 | 2 | Y | 38422 | alpha-2-HS-glycoprotein [Python bivittatus] |
| 145 | 641 | XP\_034281839.1 | 59.37 | 2 | 2 | 3.2482E5 | 1 | 1 | 1 | N | 53585 | multiple inositol polyphosphate phosphatase 1 [Pantherophis guttatus] |
| 145 | 845 | XP\_013925207.1 | 59.37 | 3 | 3 | 3.2482E5 | 1 | 1 | 1 | N | 39926 | PREDICTED: multiple inositol polyphosphate phosphatase 1 [Thamnophis sirtalis] |
| 145 | 801 | XP\_026542555.1 | 59.37 | 2 | 2 | 3.2482E5 | 1 | 1 | 1 | N | 53135 | multiple inositol polyphosphate phosphatase 1 isoform X2 [Notechis scutatus] |
| 145 | 803 | XP\_026575692.1 | 59.37 | 2 | 2 | 3.2482E5 | 1 | 1 | 1 | N | 53443 | multiple inositol polyphosphate phosphatase 1 isoform X2 [Pseudonaja textilis] |
| 145 | 802 | XP\_026542553.1 | 59.37 | 2 | 2 | 3.2482E5 | 1 | 1 | 1 | N | 53549 | multiple inositol polyphosphate phosphatase 1 isoform X1 [Notechis scutatus] |
| 145 | 770 | XP\_029139434.1 | 59.37 | 2 | 2 | 3.2482E5 | 1 | 1 | 1 | N | 53980 | multiple inositol polyphosphate phosphatase 1 [Protobothrops mucrosquamatus] |
| 145 | 804 | XP\_026575691.1 | 59.37 | 2 | 2 | 3.2482E5 | 1 | 1 | 1 | N | 53858 | multiple inositol polyphosphate phosphatase 1 isoform X1 [Pseudonaja textilis] |
| 145 | 1080 | XP\_032087784.1 | 59.37 | 2 | 2 | 3.2482E5 | 1 | 1 | 1 | N | 54267 | multiple inositol polyphosphate phosphatase 1 [Thamnophis elegans] |
| 145 | 1388 | XP\_015746961.1 | 59.37 | 3 | 3 | 3.2482E5 | 1 | 1 | 1 | N | 37973 | multiple inositol polyphosphate phosphatase 1 [Python bivittatus] |
| 152 | 1232 | JAB52761.1 | 55.04 | 6 | 6 | 4.7538E5 | 1 | 1 | 1 | Y | 15817 | phospholipase A2 8 [Micrurus fulvius] |
| 152 | 1233 | JAS04986.1 | 55.04 | 5 | 5 | 4.7538E5 | 1 | 1 | 1 | Y | 15923 | Phospholipase A2 8b [Micrurus fulvius] |
| 152 | 1234 | JAS04987.1 | 55.04 | 5 | 5 | 4.7538E5 | 1 | 1 | 1 | Y | 15913 | Phospholipase A2 8a [Micrurus fulvius] |
| 152 | 1235 | JAS04982.1 | 55.04 | 5 | 5 | 4.7538E5 | 1 | 1 | 1 | Y | 15924 | Phospholipase A2 8f [Micrurus fulvius] |
| 152 | 1236 | JAS04985.1 | 55.04 | 5 | 5 | 4.7538E5 | 1 | 1 | 1 | Y | 15938 | Phospholipase A2 8c [Micrurus fulvius] |
| 152 | 1237 | JAI08996.1 | 55.04 | 5 | 5 | 4.7538E5 | 1 | 1 | 1 | Y | 15938 | Phospholipase A2 8c [Micrurus fulvius] |
| 152 | 1238 | JAI08997.1 | 55.04 | 5 | 5 | 4.7538E5 | 1 | 1 | 1 | Y | 15923 | Phospholipase A2 8b [Micrurus fulvius] |
| 152 | 1239 | JAI08998.1 | 55.04 | 5 | 5 | 4.7538E5 | 1 | 1 | 1 | Y | 15913 | Phospholipase A2 8a [Micrurus fulvius] |
| 152 | 1240 | JAI08993.1 | 55.04 | 5 | 5 | 4.7538E5 | 1 | 1 | 1 | Y | 15924 | Phospholipase A2 8f [Micrurus fulvius] |
| 152 | 1241 | JAB52775.1 | 55.04 | 5 | 5 | 4.7538E5 | 1 | 1 | 1 | Y | 15923 | phospholipase A2 3b [Micrurus fulvius] |
| 152 | 1242 | JAB52776.1 | 55.04 | 5 | 5 | 4.7538E5 | 1 | 1 | 1 | Y | 15913 | phospholipase A2 3a [Micrurus fulvius] |
| 152 | 1083 | JAS05103.1 | 55.04 | 5 | 5 | 4.7538E5 | 1 | 1 | 1 | Y | 15807 | Phospholipase A2 8i [Micrurus tener] |
| 152 | 1335 | JAS05108.1 | 55.04 | 5 | 5 | 4.7538E5 | 1 | 1 | 1 | Y | 15724 | Phospholipase A2 8d [Micrurus tener] |
| 152 | 1336 | JAS05109.1 | 55.04 | 5 | 5 | 4.7538E5 | 1 | 1 | 1 | Y | 15865 | Phospholipase A2 8c [Micrurus tener] |
| 152 | 1807 | JAS05107.1 | 55.04 | 5 | 5 | 4.7538E5 | 1 | 1 | 1 | Y | 15708 | Phospholipase A2 8e [Micrurus tener] |
| 152 | 1337 | JAS05106.1 | 55.04 | 5 | 5 | 4.7538E5 | 1 | 1 | 1 | Y | 15767 | Phospholipase A2 8f [Micrurus tener] |
| 152 | 1338 | JAS05111.1 | 55.04 | 5 | 5 | 4.7538E5 | 1 | 1 | 1 | Y | 15766 | Phospholipase A2 8a [Micrurus tener] |
| 152 | 1339 | JAS05101.1 | 55.04 | 5 | 5 | 4.7538E5 | 1 | 1 | 1 | Y | 15739 | Phospholipase A2 8k [Micrurus tener] |
| 152 | 1340 | JAS05102.1 | 55.04 | 5 | 5 | 4.7538E5 | 1 | 1 | 1 | Y | 15781 | Phospholipase A2 8j [Micrurus tener] |
| 152 | 1341 | JAS05104.1 | 55.04 | 5 | 5 | 4.7538E5 | 1 | 1 | 1 | Y | 15781 | Phospholipase A2 8h [Micrurus tener] |
| 152 | 1342 | JAS05105.1 | 55.04 | 5 | 5 | 4.7538E5 | 1 | 1 | 1 | Y | 15952 | Phospholipase A2 8g [Micrurus tener] |
| 152 | 1343 | JAS05110.1 | 55.04 | 5 | 5 | 4.7538E5 | 1 | 1 | 1 | Y | 15766 | Phospholipase A2 8b [Micrurus tener] |
| 144 | 751 | ACR78492.1 | 54.81 | 22 | 22 | 1.474E7 | 1 | 1 | 1 | Y | 9611 | putative long chain neurotoxin 193R [Drysdalia coronoides] |
| 144 | 752 | ACR78484.1 | 54.81 | 22 | 22 | 1.474E7 | 1 | 1 | 1 | Y | 9611 | putative long chain neurotoxin 20 [Drysdalia coronoides] |
| 144 | 753 | F8J2F2.1 | 54.81 | 22 | 22 | 1.474E7 | 1 | 1 | 1 | Y | 9611 | RecName: Full=Long neurotoxin 20; Short=LNTX-20; AltName: Full=Long neurotoxin 193R; Flags: Precursor |
| 144 | 754 | ACR78483.1 | 54.81 | 22 | 22 | 1.474E7 | 1 | 1 | 1 | Y | 9664 | putative long chain neurotoxin 291 [Drysdalia coronoides] |
| 144 | 755 | ACR78488.1 | 54.81 | 22 | 22 | 1.474E7 | 1 | 1 | 1 | Y | 9664 | putative long chain neurotoxin 472 [Drysdalia coronoides] |
| 144 | 758 | JAA74738.1 | 54.81 | 20 | 20 | 1.474E7 | 1 | 1 | 1 | Y | 10113 | 3FTx-Den-9 [Denisonia devisi] |
| 144 | 838 | ACR78486.1 | 54.81 | 22 | 22 | 1.474E7 | 1 | 1 | 1 | Y | 9526 | putative long chain neurotoxin 31 [Drysdalia coronoides] |
| 144 | 839 | F8J2E6.1 | 54.81 | 22 | 22 | 1.474E7 | 1 | 1 | 1 | Y | 9526 | RecName: Full=Long neurotoxin 31; Short=LNTX-31; Flags: Precursor |
| 144 | 840 | ACR78491.1 | 54.81 | 22 | 22 | 1.474E7 | 1 | 1 | 1 | Y | 9500 | putative long chain neurotoxin 178R [Drysdalia coronoides] |
| 144 | 1129 | JAA74652.1 | 54.81 | 22 | 22 | 1.474E7 | 1 | 1 | 1 | Y | 9635 | 3FTx-Aca-53 [Acanthophis wellsi] |
| 144 | 1130 | JAA74651.1 | 54.81 | 22 | 22 | 1.474E7 | 1 | 1 | 1 | Y | 9635 | 3FTx-Aca-54 [Acanthophis wellsi] |
| 144 | 1128 | JAA74655.1 | 54.81 | 22 | 22 | 1.474E7 | 1 | 1 | 1 | Y | 9574 | 3FTx-Aca-18 [Acanthophis wellsi] |
| 144 | 1767 | ABK63538.1 | 54.81 | 20 | 20 | 1.474E7 | 1 | 1 | 1 | Y | 10137 | LNTX-1 precursor [Tropidechis carinatus] |
| 144 | 1768 | A8HDK4.1 | 54.81 | 20 | 20 | 1.474E7 | 1 | 1 | 1 | Y | 10137 | RecName: Full=Long neurotoxin 1; Short=LNTX-1; Flags: Precursor |
| 144 | 1769 | ABX58163.1 | 54.81 | 22 | 22 | 1.474E7 | 1 | 1 | 1 | Y | 9588 | putative long neurotoxin [Austrelaps labialis] |
| 144 | 1506 | ABX58162.1 | 54.81 | 22 | 22 | 1.474E7 | 1 | 1 | 1 | Y | 9567 | putative long neurotoxin [Austrelaps labialis] |
| 144 | 1507 | ABX58156.1 | 54.81 | 22 | 22 | 1.474E7 | 1 | 1 | 1 | Y | 9594 | putative long neurotoxin [Austrelaps labialis] |
| 144 | 1508 | ABX58159.1 | 54.81 | 22 | 22 | 1.474E7 | 1 | 1 | 1 | Y | 9567 | putative long neurotoxin [Austrelaps labialis] |
| 144 | 1509 | ABX58160.1 | 54.81 | 22 | 22 | 1.474E7 | 1 | 1 | 1 | Y | 9650 | putative long neurotoxin [Austrelaps labialis] |
| 144 | 1510 | ABX58161.1 | 54.81 | 22 | 22 | 1.474E7 | 1 | 1 | 1 | Y | 9622 | putative long neurotoxin [Austrelaps labialis] |
| 144 | 1511 | ABX58158.1 | 54.81 | 22 | 22 | 1.474E7 | 1 | 1 | 1 | Y | 9566 | putative long neurotoxin [Austrelaps labialis] |
| 157 | 1059 | P01397.2 | 54.53 | 17 | 17 | 2.0197E6 | 1 | 1 | 1 | Y | 7948 | RecName: Full=Alpha-elapitoxin-Dpp2c; Short=Alpha-EPTX-Dpp2c; AltName: Full=Long neurotoxin 2; AltName: Full=Neurotoxin delta; AltName: Full=Toxin TN2 |
| 157 | 1058 | P25667.1 | 54.53 | 17 | 17 | 2.0197E6 | 1 | 1 | 1 | Y | 7939 | RecName: Full=Alpha-elapitoxin-Dpp2b; Short=Alpha-EPTX-Dpp2b; AltName: Full=Long neurotoxin 3; AltName: Full=Toxin VN2 |
| total 246 proteins |  |  |  |  |  |  |  |  |  |  |  |  |
| --- | --- | --- | --- | --- | --- | --- | --- | --- | --- | --- | --- | --- |

  

T\_169

back to list

  

| Protein Coverage
| Supporting Peptides
|

Protein Coverage:

Supporting Peptides:

| Peptide | Uniq | -10lgP | Mass | Length | ppm | m/z | z | RT | Fraction | Scan | Source File | Area BuCaMH03\_B17 | #Feature | #Feature BuCaMH03\_B17 | Start | End | PTM | AScore | Found By |
| --- | --- | --- | --- | --- | --- | --- | --- | --- | --- | --- | --- | --- | --- | --- | --- | --- | --- | --- | --- |
| R.AVTIFGESAGAASVGMHLLSTQSR.T | N | 110.40 | 2389.2061 | 24 | 0.0 | 797.4093 | 3 | 70.91 | 1 | 45278 | BuCaMH03.raw | 2.6603E8 | 4 | 4 | 224 | 247 |  |  | PEAKS DB |
| R.FLTYTQNVILVSLSYR.V | N | 110.00 | 1916.0408 | 16 | 0.4 | 959.0281 | 2 | 83.45 | 1 | 55322 | BuCaMH03.raw | 3.0247E7 | 3 | 3 | 165 | 180 |  |  | PEAKS DB |
| R.FPFVPVIDGDFFPDTPEAMLSSGNFK.E | N | 109.19 | 2873.3621 | 26 | 0.6 | 1437.6892 | 2 | 98.27 | 1 | 67043 | BuCaMH03.raw | 4.4491E8 | 2 | 2 | 321 | 346 |  |  | PEAKS DB |
| K.DEGSYFLIYGLPGFSK.D | N | 106.68 | 1791.8718 | 16 | 0.8 | 896.9439 | 2 | 93.19 | 1 | 62858 | BuCaMH03.raw | 2.4463E8 | 2 | 2 | 357 | 372 |  |  | PEAKS DB |
| R.AILQSGGPNAPWATVTPAESR.R | N | 105.98 | 2122.0806 | 21 | 0.3 | 1062.0479 | 2 | 59.69 | 1 | 35420 | BuCaMH03.raw | 9.8415E7 | 2 | 2 | 253 | 273 |  |  | PEAKS DB |
| K.NPQELIDEEWSVLPYK.S | N | 102.10 | 1958.9625 | 16 | 0.4 | 980.4890 | 2 | 78.54 | 1 | 51369 | BuCaMH03.raw | 6.749E7 | 2 | 2 | 301 | 316 |  |  | PEAKS DB |
| R.SKNPQELIDEEWSVLPYK.S | N | 100.91 | 2174.0896 | 18 | 2.3 | 725.7054 | 3 | 68.47 | 1 | 43161 | BuCaMH03.raw | 1.243E8 | 2 | 2 | 299 | 316 |  |  | PEAKS DB |
| M.SVPHANDIATESVVLQYTDWQDQDNR.E | Y | 97.78 | 3000.3850 | 26 | -0.1 | 1001.1355 | 3 | 64.89 | 1 | 39962 | BuCaMH03.raw | 5.6416E6 | 1 | 1 | 390 | 415 |  |  | PEAKS DB |
| R.LALQWIQNNIHPFGGNPR.A | N | 96.68 | 2074.0859 | 18 | 0.3 | 692.3694 | 3 | 67.05 | 1 | 41669 | BuCaMH03.raw | 1.057E7 | 2 | 2 | 206 | 223 |  |  | PEAKS DB |
| Q.SGGPNAPWATVTPAESR.R | N | 95.90 | 1696.8169 | 17 | -0.7 | 849.4152 | 2 | 46.64 | 1 | 24656 | BuCaMH03.raw | 4.0143E6 | 1 | 1 | 257 | 273 |  |  | PEAKS DB |
| R.EALDDIVGDHNVIC(+57.02)PVVQFANDYAK.R | N | 93.85 | 2801.3330 | 25 | -0.2 | 934.7847 | 3 | 78.96 | 1 | 51733 | BuCaMH03.raw | 2.9516E8 | 2 | 2 | 420 | 444 | Carbamidomethylation | C14:Carbamidomethylation:1000.00 | PEAKS DB |
| R.VGAFGFLGLPGSPEAPGNMGLLDQR.L | N | 92.98 | 2499.2581 | 25 | 1.1 | 834.0942 | 3 | 90.41 | 1 | 60987 | BuCaMH03.raw | 3.7724E8 | 2 | 2 | 181 | 205 |  |  | PEAKS DB |
| R.GLSLPVLDGHVTAFLGIPFAEPPVGR.M | N | 86.95 | 2658.4534 | 26 | 1.3 | 887.1595 | 3 | 96.29 | 1 | 65362 | BuCaMH03.raw | 2.0859E9 | 4 | 4 | 48 | 73 |  |  | PEAKS DB |
| A.LQWIQNNIHPFGGNPR.A | N | 86.76 | 1889.9648 | 16 | 0.8 | 945.9904 | 2 | 52.84 | 1 | 29897 | BuCaMH03.raw | 2.3373E7 | 2 | 2 | 208 | 223 |  |  | PEAKS DB |
| R.MSVPHANDIATESVVLQYTDWQDQDNR.E | Y | 85.54 | 3131.4253 | 27 | 1.1 | 1044.8169 | 3 | 69.31 | 1 | 43770 | BuCaMH03.raw | 8.1462E7 | 2 | 2 | 389 | 415 |  |  | PEAKS DB |
| L.ALQWIQNNIHPFGGNPR.A | N | 84.31 | 1961.0020 | 17 | 0.2 | 981.5084 | 2 | 56.08 | 1 | 32631 | BuCaMH03.raw | 7.657E7 | 2 | 2 | 207 | 223 |  |  | PEAKS DB |
| K.SGAWPTYTASQPQYVQLNTQPLATQPSLR.A | N | 79.74 | 3202.6047 | 29 | 0.8 | 1068.5431 | 3 | 70.50 | 1 | 44551 | BuCaMH03.raw | 4.9159E7 | 1 | 1 | 520 | 548 |  |  | PEAKS DB |
| K.SIFRFPFVPVIDGDFFPDTPEAMLSSGNFK.E | N | 79.19 | 3376.6477 | 30 | -0.6 | 1126.5558 | 3 | 98.25 | 1 | 66983 | BuCaMH03.raw | 1.9571E6 | 1 | 1 | 317 | 346 |  |  | PEAKS DB |
| R.FPFVPVIDGDFFPDTPEAM(+15.99)LSSGNFK.E | N | 79.18 | 2889.3569 | 26 | 0.0 | 964.1262 | 3 | 98.20 | 1 | 66887 | BuCaMH03.raw | 1.6186E7 | 1 | 1 | 321 | 346 | Oxidation (M) | M19:Oxidation (M):1000.00 | PEAKS DB |
| K.NREALDDIVGDHNVIC(+57.02)PVVQFANDYAK.R | N | 77.79 | 3071.4771 | 27 | 0.4 | 768.8768 | 4 | 70.23 | 1 | 44298 | BuCaMH03.raw | 9.8218E6 | 2 | 2 | 418 | 444 | Carbamidomethylation | C16:Carbamidomethylation:1000.00 | PEAKS DB |
| K.ETQVLLGVVKDEGSYFLIYGLPGFSK.D | N | 77.49 | 2858.5105 | 26 | 0.5 | 953.8446 | 3 | 94.22 | 1 | 63592 | BuCaMH03.raw | 1.1881E7 | 1 | 1 | 347 | 372 |  |  | PEAKS DB |
| K.VYAYLFDHR.A | N | 76.38 | 1182.5822 | 9 | 0.3 | 592.2985 | 2 | 41.43 | 1 | 20432 | BuCaMH03.raw | 2.3244E7 | 2 | 2 | 449 | 457 |  |  | PEAKS DB |
| R.AQIC(+57.02)AFWNHFLPK.L | N | 76.29 | 1630.8079 | 13 | 0.1 | 544.6099 | 3 | 67.38 | 1 | 41864 | BuCaMH03.raw | 6.214E6 | 1 | 1 | 549 | 561 | Carbamidomethylation | C4:Carbamidomethylation:1000.00 | PEAKS DB |
| A.VTIFGESAGAASVGMHLLSTQSR.T | N | 75.90 | 2318.1689 | 23 | 1.1 | 773.7311 | 3 | 69.24 | 1 | 43488 | BuCaMH03.raw | 1.4432E6 | 1 | 1 | 225 | 247 |  |  | PEAKS DB |
| I.C(+57.02)AFWNHFLPK.L | N | 74.57 | 1318.6281 | 10 | -0.5 | 660.3210 | 2 | 56.23 | 1 | 32768 | BuCaMH03.raw | 7.6723E6 | 1 | 1 | 552 | 561 | Carbamidomethylation | C1:Carbamidomethylation:1000.00 | PEAKS DB |
| Q.WIQNNIHPFGGNPR.A | N | 73.92 | 1648.8223 | 14 | 0.4 | 825.4187 | 2 | 39.98 | 1 | 19293 | BuCaMH03.raw | 1.286E7 | 2 | 2 | 210 | 223 |  |  | PEAKS DB |
| P.FVPVIDGDFFPDTPEAMLSSGNFK.E | N | 73.61 | 2629.2410 | 24 | 0.1 | 1315.6279 | 2 | 94.45 | 1 | 64055 | BuCaMH03.raw | 2.0905E7 | 1 | 1 | 323 | 346 |  |  | PEAKS DB |
| V.GAFGFLGLPGSPEAPGNMGLLDQR.L | N | 72.34 | 2400.1895 | 24 | 0.4 | 1201.1025 | 2 | 88.38 | 1 | 58926 | BuCaMH03.raw | 3.179E7 | 2 | 2 | 182 | 205 |  |  | PEAKS DB |
| R.MSVPHANDIATESVVLQYTDWQDQDNREK.N | Y | 71.07 | 3388.5630 | 29 | 0.2 | 848.1482 | 4 | 62.82 | 1 | 38255 | BuCaMH03.raw | 5.174E6 | 1 | 1 | 389 | 417 |  |  | PEAKS DB |
| G.LPGSPEAPGNMGLLDQR.L | N | 70.60 | 1750.8672 | 17 | 0.7 | 876.4415 | 2 | 54.97 | 1 | 31655 | BuCaMH03.raw | 4.4619E6 | 1 | 1 | 189 | 205 |  |  | PEAKS DB |
| R.FLRPEPVKPWPHVLDATSYK.P | N | 69.65 | 2379.2739 | 20 | 0.8 | 595.8262 | 4 | 48.92 | 1 | 26568 | BuCaMH03.raw | 6.2045E6 | 1 | 1 | 76 | 95 |  |  | PEAKS DB |
| Q.IC(+57.02)AFWNHFLPK.L | N | 69.60 | 1431.7122 | 11 | 0.3 | 478.2448 | 3 | 63.50 | 1 | 38737 | BuCaMH03.raw | 2.159E6 | 1 | 1 | 551 | 561 | Carbamidomethylation | C2:Carbamidomethylation:1000.00 | PEAKS DB |
| L.NATVDPPSADR.R | Y | 69.09 | 1141.5364 | 11 | -0.3 | 571.7753 | 2 | 16.18 | 1 | 3477 | BuCaMH03.raw | 1.4697E6 | 1 | 1 | 564 | 574 |  |  | PEAKS DB |
| A.QIC(+57.02)AFWNHFLPK.L | N | 68.21 | 1559.7708 | 12 | 0.3 | 520.9310 | 3 | 65.09 | 1 | 40072 | BuCaMH03.raw | 1.1741E7 | 2 | 2 | 550 | 561 | Carbamidomethylation | C3:Carbamidomethylation:1000.00 | PEAKS DB |
| K.ETQVLLGVVK.D | N | 66.91 | 1084.6492 | 10 | -0.4 | 543.3317 | 2 | 48.73 | 1 | 26723 | BuCaMH03.raw | 1.4354E8 | 1 | 1 | 347 | 356 |  |  | PEAKS DB |
| A.ILQSGGPNAPWATVTPAESR.R | N | 64.65 | 2051.0435 | 20 | 0.5 | 1026.5295 | 2 | 55.18 | 1 | 31833 | BuCaMH03.raw | 2.893E6 | 1 | 1 | 254 | 273 |  |  | PEAKS DB |
| L.SLPVLDGHVTAFLGIPFAEPPVGR.M | N | 64.20 | 2488.3477 | 24 | 1.4 | 830.4576 | 3 | 89.15 | 1 | 59573 | BuCaMH03.raw | 2.1629E7 | 1 | 1 | 50 | 73 |  |  | PEAKS DB |
| R.AVTIFGESAGAASVGMHLLSTQSRTLFQR.A | N | 62.77 | 3034.5657 | 29 | 0.9 | 759.6494 | 4 | 77.95 | 1 | 50683 | BuCaMH03.raw | 8.7118E5 | 1 | 1 | 224 | 252 |  |  | PEAKS DB |
| G.LSLPVLDGHVTAFLGIPFAEPPVGR.M | N | 61.54 | 2601.4319 | 25 | 0.0 | 868.1512 | 3 | 94.83 | 1 | 64548 | BuCaMH03.raw | 9.5169E6 | 2 | 2 | 49 | 73 |  |  | PEAKS DB |
| F.LTYTQNVILVSLSYR.V | N | 56.26 | 1768.9723 | 15 | 0.1 | 885.4935 | 2 | 75.21 | 1 | 48520 | BuCaMH03.raw | 1.4443E6 | 1 | 1 | 166 | 180 |  |  | PEAKS DB |
| P.QELIDEEWSVLPYK.S | N | 56.12 | 1747.8668 | 14 | 0.9 | 874.9415 | 2 | 73.88 | 1 | 47373 | BuCaMH03.raw | 3.1518E6 | 1 | 1 | 303 | 316 |  |  | PEAKS DB |
| L.QWIQNNIHPFGGNPR.A | N | 55.84 | 1776.8809 | 15 | -0.4 | 593.3007 | 3 | 43.44 | 1 | 22097 | BuCaMH03.raw | 2.5227E7 | 1 | 1 | 209 | 223 |  |  | PEAKS DB |
| R.AVTIFGESAGAASVGM(+15.99)HLLSTQSR.T | N | 55.79 | 2405.2009 | 24 | 0.0 | 802.7409 | 3 | 70.98 | 1 | 44980 | BuCaMH03.raw | 5.9259E6 | 1 | 1 | 224 | 247 | Oxidation (M) | M16:Oxidation (M):1000.00 | PEAKS DB |
| R.ADFLEGVR.M | N | 55.33 | 905.4606 | 8 | 0.5 | 453.7378 | 2 | 47.32 | 1 | 25289 | BuCaMH03.raw | 6.9015E7 | 2 | 2 | 381 | 388 |  |  | PEAKS DB |
| E.GSYFLIYGLPGFSK.D | N | 55.01 | 1547.8024 | 14 | 0.7 | 774.9090 | 2 | 85.55 | 1 | 56608 | BuCaMH03.raw | 1.0538E6 | 1 | 1 | 359 | 372 |  |  | PEAKS DB |
| D.GDFFPDTPEAMLSSGNFK.E | N | 54.97 | 1958.8719 | 18 | 0.7 | 980.4440 | 2 | 77.20 | 1 | 50118 | BuCaMH03.raw | 9.7457E5 | 1 | 1 | 329 | 346 |  |  | PEAKS DB |
| R.FPFVPVIDGDFFPDTPEAM.L | N | 54.90 | 2139.9863 | 19 | 0.5 | 1071.0010 | 2 | 98.34 | 1 | 67085 | BuCaMH03.raw | 5.9713E6 | 1 | 1 | 321 | 339 |  |  | PEAKS DB |
| R.FLRPEPVKPWPH.V | N | 54.64 | 1501.8193 | 12 | 0.7 | 501.6141 | 3 | 36.30 | 1 | 16415 | BuCaMH03.raw | 7.868E6 | 1 | 1 | 76 | 87 |  |  | PEAKS DB |
| L.LNATVDPPSADR.R | Y | 54.61 | 1254.6204 | 12 | 0.9 | 628.3180 | 2 | 26.78 | 1 | 9665 | BuCaMH03.raw | 7.2628E6 | 1 | 1 | 563 | 574 |  |  | PEAKS DB |
| N.PQELIDEEWSVLPYK.S | N | 54.23 | 1844.9196 | 15 | 1.3 | 923.4683 | 2 | 74.37 | 1 | 47795 | BuCaMH03.raw | 4.9513E5 | 1 | 1 | 302 | 316 |  |  | PEAKS DB |
| K.QLGC(+57.02)HFNNDSELVSC(+57.02)LR.S | N | 54.19 | 2047.9204 | 17 | 0.2 | 683.6476 | 3 | 45.75 | 1 | 23845 | BuCaMH03.raw | 8.5586E6 | 1 | 1 | 282 | 298 | Carbamidomethylation | C4:Carbamidomethylation:1000.00;C15:Carbamidomethylation:1000.00 | PEAKS DB |
| F.GFLGLPGSPEAPGNMGLLDQR.L | N | 54.11 | 2125.0625 | 21 | 0.3 | 1063.5388 | 2 | 79.70 | 1 | 52045 | BuCaMH03.raw | 1.5382E6 | 1 | 1 | 185 | 205 |  |  | PEAKS DB |
| K.LLNATVDPPSADR.R | Y | 53.44 | 1367.7045 | 13 | 0.5 | 684.8599 | 2 | 37.10 | 1 | 17092 | BuCaMH03.raw | 7.186E6 | 1 | 1 | 562 | 574 |  |  | PEAKS DB |
| R.LALQWIQNNIHPF.G | N | 53.30 | 1592.8463 | 13 | 0.4 | 797.4308 | 2 | 79.77 | 1 | 52223 | BuCaMH03.raw | 3.4534E5 | 1 | 1 | 206 | 218 |  |  | PEAKS DB |
| total 54 peptides |  |  |  |  |  |  |  |  |  |  |  |  |  |  |  |  |  |  |  |
| --- | --- | --- | --- | --- | --- | --- | --- | --- | --- | --- | --- | --- | --- | --- | --- | --- | --- | --- | --- |

T\_163

back to list

  

| Protein Coverage
| Supporting Peptides
|

Protein Coverage:

Supporting Peptides:

| Peptide | Uniq | -10lgP | Mass | Length | ppm | m/z | z | RT | Fraction | Scan | Source File | Area BuCaMH03\_B17 | #Feature | #Feature BuCaMH03\_B17 | Start | End | PTM | AScore | Found By |
| --- | --- | --- | --- | --- | --- | --- | --- | --- | --- | --- | --- | --- | --- | --- | --- | --- | --- | --- | --- |
| R.AVTIFGESAGAASVGMHLLSTQSR.T | N | 110.40 | 2389.2061 | 24 | 0.0 | 797.4093 | 3 | 70.91 | 1 | 45278 | BuCaMH03.raw | 2.6603E8 | 4 | 4 | 264 | 287 |  |  | PEAKS DB |
| R.FLTYTQNVILVSLSYR.V | N | 110.00 | 1916.0408 | 16 | 0.4 | 959.0281 | 2 | 83.45 | 1 | 55322 | BuCaMH03.raw | 3.0247E7 | 3 | 3 | 205 | 220 |  |  | PEAKS DB |
| R.FPFVPVIDGDFFPDTPEAMLSSGNFK.E | N | 109.19 | 2873.3621 | 26 | 0.6 | 1437.6892 | 2 | 98.27 | 1 | 67043 | BuCaMH03.raw | 4.4491E8 | 2 | 2 | 361 | 386 |  |  | PEAKS DB |
| K.DEGSYFLIYGLPGFSK.D | N | 106.68 | 1791.8718 | 16 | 0.8 | 896.9439 | 2 | 93.19 | 1 | 62858 | BuCaMH03.raw | 2.4463E8 | 2 | 2 | 397 | 412 |  |  | PEAKS DB |
| R.AILQSGGPNAPWATVTPAESR.R | N | 105.98 | 2122.0806 | 21 | 0.3 | 1062.0479 | 2 | 59.69 | 1 | 35420 | BuCaMH03.raw | 9.8415E7 | 2 | 2 | 293 | 313 |  |  | PEAKS DB |
| K.NPQELIDEEWSVLPYK.S | N | 102.10 | 1958.9625 | 16 | 0.4 | 980.4890 | 2 | 78.54 | 1 | 51369 | BuCaMH03.raw | 6.749E7 | 2 | 2 | 341 | 356 |  |  | PEAKS DB |
| R.SKNPQELIDEEWSVLPYK.S | N | 100.91 | 2174.0896 | 18 | 2.3 | 725.7054 | 3 | 68.47 | 1 | 43161 | BuCaMH03.raw | 1.243E8 | 2 | 2 | 339 | 356 |  |  | PEAKS DB |
| M.SVPHANDIATESVVLQYTDWQDQDNR.E | Y | 97.78 | 3000.3850 | 26 | -0.1 | 1001.1355 | 3 | 64.89 | 1 | 39962 | BuCaMH03.raw | 5.6416E6 | 1 | 1 | 430 | 455 |  |  | PEAKS DB |
| R.LALQWIQNNIHPFGGNPR.A | N | 96.68 | 2074.0859 | 18 | 0.3 | 692.3694 | 3 | 67.05 | 1 | 41669 | BuCaMH03.raw | 1.057E7 | 2 | 2 | 246 | 263 |  |  | PEAKS DB |
| Q.SGGPNAPWATVTPAESR.R | N | 95.90 | 1696.8169 | 17 | -0.7 | 849.4152 | 2 | 46.64 | 1 | 24656 | BuCaMH03.raw | 4.0143E6 | 1 | 1 | 297 | 313 |  |  | PEAKS DB |
| R.EALDDIVGDHNVIC(+57.02)PVVQFANDYAK.R | N | 93.85 | 2801.3330 | 25 | -0.2 | 934.7847 | 3 | 78.96 | 1 | 51733 | BuCaMH03.raw | 2.9516E8 | 2 | 2 | 460 | 484 | Carbamidomethylation | C14:Carbamidomethylation:1000.00 | PEAKS DB |
| R.VGAFGFLGLPGSPEAPGNMGLLDQR.L | N | 92.98 | 2499.2581 | 25 | 1.1 | 834.0942 | 3 | 90.41 | 1 | 60987 | BuCaMH03.raw | 3.7724E8 | 2 | 2 | 221 | 245 |  |  | PEAKS DB |
| R.GLSLPVLDGHVTAFLGIPFAEPPVGR.M | N | 86.95 | 2658.4534 | 26 | 1.3 | 887.1595 | 3 | 96.29 | 1 | 65362 | BuCaMH03.raw | 2.0859E9 | 4 | 4 | 88 | 113 |  |  | PEAKS DB |
| A.LQWIQNNIHPFGGNPR.A | N | 86.76 | 1889.9648 | 16 | 0.8 | 945.9904 | 2 | 52.84 | 1 | 29897 | BuCaMH03.raw | 2.3373E7 | 2 | 2 | 248 | 263 |  |  | PEAKS DB |
| R.MSVPHANDIATESVVLQYTDWQDQDNR.E | Y | 85.54 | 3131.4253 | 27 | 1.1 | 1044.8169 | 3 | 69.31 | 1 | 43770 | BuCaMH03.raw | 8.1462E7 | 2 | 2 | 429 | 455 |  |  | PEAKS DB |
| L.ALQWIQNNIHPFGGNPR.A | N | 84.31 | 1961.0020 | 17 | 0.2 | 981.5084 | 2 | 56.08 | 1 | 32631 | BuCaMH03.raw | 7.657E7 | 2 | 2 | 247 | 263 |  |  | PEAKS DB |
| K.SGAWPTYTASQPQYVQLNTQPLATQPSLR.A | N | 79.74 | 3202.6047 | 29 | 0.8 | 1068.5431 | 3 | 70.50 | 1 | 44551 | BuCaMH03.raw | 4.9159E7 | 1 | 1 | 560 | 588 |  |  | PEAKS DB |
| K.SIFRFPFVPVIDGDFFPDTPEAMLSSGNFK.E | N | 79.19 | 3376.6477 | 30 | -0.6 | 1126.5558 | 3 | 98.25 | 1 | 66983 | BuCaMH03.raw | 1.9571E6 | 1 | 1 | 357 | 386 |  |  | PEAKS DB |
| R.FPFVPVIDGDFFPDTPEAM(+15.99)LSSGNFK.E | N | 79.18 | 2889.3569 | 26 | 0.0 | 964.1262 | 3 | 98.20 | 1 | 66887 | BuCaMH03.raw | 1.6186E7 | 1 | 1 | 361 | 386 | Oxidation (M) | M19:Oxidation (M):1000.00 | PEAKS DB |
| K.NREALDDIVGDHNVIC(+57.02)PVVQFANDYAK.R | N | 77.79 | 3071.4771 | 27 | 0.4 | 768.8768 | 4 | 70.23 | 1 | 44298 | BuCaMH03.raw | 9.8218E6 | 2 | 2 | 458 | 484 | Carbamidomethylation | C16:Carbamidomethylation:1000.00 | PEAKS DB |
| K.ETQVLLGVVKDEGSYFLIYGLPGFSK.D | N | 77.49 | 2858.5105 | 26 | 0.5 | 953.8446 | 3 | 94.22 | 1 | 63592 | BuCaMH03.raw | 1.1881E7 | 1 | 1 | 387 | 412 |  |  | PEAKS DB |
| K.VYAYLFDHR.A | N | 76.38 | 1182.5822 | 9 | 0.3 | 592.2985 | 2 | 41.43 | 1 | 20432 | BuCaMH03.raw | 2.3244E7 | 2 | 2 | 489 | 497 |  |  | PEAKS DB |
| R.AQIC(+57.02)AFWNHFLPK.L | N | 76.29 | 1630.8079 | 13 | 0.1 | 544.6099 | 3 | 67.38 | 1 | 41864 | BuCaMH03.raw | 6.214E6 | 1 | 1 | 589 | 601 | Carbamidomethylation | C4:Carbamidomethylation:1000.00 | PEAKS DB |
| A.VTIFGESAGAASVGMHLLSTQSR.T | N | 75.90 | 2318.1689 | 23 | 1.1 | 773.7311 | 3 | 69.24 | 1 | 43488 | BuCaMH03.raw | 1.4432E6 | 1 | 1 | 265 | 287 |  |  | PEAKS DB |
| I.C(+57.02)AFWNHFLPK.L | N | 74.57 | 1318.6281 | 10 | -0.5 | 660.3210 | 2 | 56.23 | 1 | 32768 | BuCaMH03.raw | 7.6723E6 | 1 | 1 | 592 | 601 | Carbamidomethylation | C1:Carbamidomethylation:1000.00 | PEAKS DB |
| Q.WIQNNIHPFGGNPR.A | N | 73.92 | 1648.8223 | 14 | 0.4 | 825.4187 | 2 | 39.98 | 1 | 19293 | BuCaMH03.raw | 1.286E7 | 2 | 2 | 250 | 263 |  |  | PEAKS DB |
| P.FVPVIDGDFFPDTPEAMLSSGNFK.E | N | 73.61 | 2629.2410 | 24 | 0.1 | 1315.6279 | 2 | 94.45 | 1 | 64055 | BuCaMH03.raw | 2.0905E7 | 1 | 1 | 363 | 386 |  |  | PEAKS DB |
| V.GAFGFLGLPGSPEAPGNMGLLDQR.L | N | 72.34 | 2400.1895 | 24 | 0.4 | 1201.1025 | 2 | 88.38 | 1 | 58926 | BuCaMH03.raw | 3.179E7 | 2 | 2 | 222 | 245 |  |  | PEAKS DB |
| R.MSVPHANDIATESVVLQYTDWQDQDNREK.N | Y | 71.07 | 3388.5630 | 29 | 0.2 | 848.1482 | 4 | 62.82 | 1 | 38255 | BuCaMH03.raw | 5.174E6 | 1 | 1 | 429 | 457 |  |  | PEAKS DB |
| G.LPGSPEAPGNMGLLDQR.L | N | 70.60 | 1750.8672 | 17 | 0.7 | 876.4415 | 2 | 54.97 | 1 | 31655 | BuCaMH03.raw | 4.4619E6 | 1 | 1 | 229 | 245 |  |  | PEAKS DB |
| R.FLRPEPVKPWPHVLDATSYK.P | N | 69.65 | 2379.2739 | 20 | 0.8 | 595.8262 | 4 | 48.92 | 1 | 26568 | BuCaMH03.raw | 6.2045E6 | 1 | 1 | 116 | 135 |  |  | PEAKS DB |
| Q.IC(+57.02)AFWNHFLPK.L | N | 69.60 | 1431.7122 | 11 | 0.3 | 478.2448 | 3 | 63.50 | 1 | 38737 | BuCaMH03.raw | 2.159E6 | 1 | 1 | 591 | 601 | Carbamidomethylation | C2:Carbamidomethylation:1000.00 | PEAKS DB |
| L.NATVDPPSADR.R | Y | 69.09 | 1141.5364 | 11 | -0.3 | 571.7753 | 2 | 16.18 | 1 | 3477 | BuCaMH03.raw | 1.4697E6 | 1 | 1 | 604 | 614 |  |  | PEAKS DB |
| A.QIC(+57.02)AFWNHFLPK.L | N | 68.21 | 1559.7708 | 12 | 0.3 | 520.9310 | 3 | 65.09 | 1 | 40072 | BuCaMH03.raw | 1.1741E7 | 2 | 2 | 590 | 601 | Carbamidomethylation | C3:Carbamidomethylation:1000.00 | PEAKS DB |
| K.ETQVLLGVVK.D | N | 66.91 | 1084.6492 | 10 | -0.4 | 543.3317 | 2 | 48.73 | 1 | 26723 | BuCaMH03.raw | 1.4354E8 | 1 | 1 | 387 | 396 |  |  | PEAKS DB |
| A.ILQSGGPNAPWATVTPAESR.R | N | 64.65 | 2051.0435 | 20 | 0.5 | 1026.5295 | 2 | 55.18 | 1 | 31833 | BuCaMH03.raw | 2.893E6 | 1 | 1 | 294 | 313 |  |  | PEAKS DB |
| L.SLPVLDGHVTAFLGIPFAEPPVGR.M | N | 64.20 | 2488.3477 | 24 | 1.4 | 830.4576 | 3 | 89.15 | 1 | 59573 | BuCaMH03.raw | 2.1629E7 | 1 | 1 | 90 | 113 |  |  | PEAKS DB |
| R.AVTIFGESAGAASVGMHLLSTQSRTLFQR.A | N | 62.77 | 3034.5657 | 29 | 0.9 | 759.6494 | 4 | 77.95 | 1 | 50683 | BuCaMH03.raw | 8.7118E5 | 1 | 1 | 264 | 292 |  |  | PEAKS DB |
| G.LSLPVLDGHVTAFLGIPFAEPPVGR.M | N | 61.54 | 2601.4319 | 25 | 0.0 | 868.1512 | 3 | 94.83 | 1 | 64548 | BuCaMH03.raw | 9.5169E6 | 2 | 2 | 89 | 113 |  |  | PEAKS DB |
| F.LTYTQNVILVSLSYR.V | N | 56.26 | 1768.9723 | 15 | 0.1 | 885.4935 | 2 | 75.21 | 1 | 48520 | BuCaMH03.raw | 1.4443E6 | 1 | 1 | 206 | 220 |  |  | PEAKS DB |
| P.QELIDEEWSVLPYK.S | N | 56.12 | 1747.8668 | 14 | 0.9 | 874.9415 | 2 | 73.88 | 1 | 47373 | BuCaMH03.raw | 3.1518E6 | 1 | 1 | 343 | 356 |  |  | PEAKS DB |
| L.QWIQNNIHPFGGNPR.A | N | 55.84 | 1776.8809 | 15 | -0.4 | 593.3007 | 3 | 43.44 | 1 | 22097 | BuCaMH03.raw | 2.5227E7 | 1 | 1 | 249 | 263 |  |  | PEAKS DB |
| R.AVTIFGESAGAASVGM(+15.99)HLLSTQSR.T | N | 55.79 | 2405.2009 | 24 | 0.0 | 802.7409 | 3 | 70.98 | 1 | 44980 | BuCaMH03.raw | 5.9259E6 | 1 | 1 | 264 | 287 | Oxidation (M) | M16:Oxidation (M):1000.00 | PEAKS DB |
| R.ADFLEGVR.M | N | 55.33 | 905.4606 | 8 | 0.5 | 453.7378 | 2 | 47.32 | 1 | 25289 | BuCaMH03.raw | 6.9015E7 | 2 | 2 | 421 | 428 |  |  | PEAKS DB |
| E.GSYFLIYGLPGFSK.D | N | 55.01 | 1547.8024 | 14 | 0.7 | 774.9090 | 2 | 85.55 | 1 | 56608 | BuCaMH03.raw | 1.0538E6 | 1 | 1 | 399 | 412 |  |  | PEAKS DB |
| D.GDFFPDTPEAMLSSGNFK.E | N | 54.97 | 1958.8719 | 18 | 0.7 | 980.4440 | 2 | 77.20 | 1 | 50118 | BuCaMH03.raw | 9.7457E5 | 1 | 1 | 369 | 386 |  |  | PEAKS DB |
| R.FPFVPVIDGDFFPDTPEAM.L | N | 54.90 | 2139.9863 | 19 | 0.5 | 1071.0010 | 2 | 98.34 | 1 | 67085 | BuCaMH03.raw | 5.9713E6 | 1 | 1 | 361 | 379 |  |  | PEAKS DB |
| R.FLRPEPVKPWPH.V | N | 54.64 | 1501.8193 | 12 | 0.7 | 501.6141 | 3 | 36.30 | 1 | 16415 | BuCaMH03.raw | 7.868E6 | 1 | 1 | 116 | 127 |  |  | PEAKS DB |
| L.LNATVDPPSADR.R | Y | 54.61 | 1254.6204 | 12 | 0.9 | 628.3180 | 2 | 26.78 | 1 | 9665 | BuCaMH03.raw | 7.2628E6 | 1 | 1 | 603 | 614 |  |  | PEAKS DB |
| N.PQELIDEEWSVLPYK.S | N | 54.23 | 1844.9196 | 15 | 1.3 | 923.4683 | 2 | 74.37 | 1 | 47795 | BuCaMH03.raw | 4.9513E5 | 1 | 1 | 342 | 356 |  |  | PEAKS DB |
| K.QLGC(+57.02)HFNNDSELVSC(+57.02)LR.S | N | 54.19 | 2047.9204 | 17 | 0.2 | 683.6476 | 3 | 45.75 | 1 | 23845 | BuCaMH03.raw | 8.5586E6 | 1 | 1 | 322 | 338 | Carbamidomethylation | C4:Carbamidomethylation:1000.00;C15:Carbamidomethylation:1000.00 | PEAKS DB |
| F.GFLGLPGSPEAPGNMGLLDQR.L | N | 54.11 | 2125.0625 | 21 | 0.3 | 1063.5388 | 2 | 79.70 | 1 | 52045 | BuCaMH03.raw | 1.5382E6 | 1 | 1 | 225 | 245 |  |  | PEAKS DB |
| K.LLNATVDPPSADR.R | Y | 53.44 | 1367.7045 | 13 | 0.5 | 684.8599 | 2 | 37.10 | 1 | 17092 | BuCaMH03.raw | 7.186E6 | 1 | 1 | 602 | 614 |  |  | PEAKS DB |
| R.LALQWIQNNIHPF.G | N | 53.30 | 1592.8463 | 13 | 0.4 | 797.4308 | 2 | 79.77 | 1 | 52223 | BuCaMH03.raw | 3.4534E5 | 1 | 1 | 246 | 258 |  |  | PEAKS DB |
| total 54 peptides |  |  |  |  |  |  |  |  |  |  |  |  |  |  |  |  |  |  |  |
| --- | --- | --- | --- | --- | --- | --- | --- | --- | --- | --- | --- | --- | --- | --- | --- | --- | --- | --- | --- |

T\_161

back to list

  

| Protein Coverage
| Supporting Peptides
|

Protein Coverage:

Supporting Peptides:

| Peptide | Uniq | -10lgP | Mass | Length | ppm | m/z | z | RT | Fraction | Scan | Source File | Area BuCaMH03\_B17 | #Feature | #Feature BuCaMH03\_B17 | Start | End | PTM | AScore | Found By |
| --- | --- | --- | --- | --- | --- | --- | --- | --- | --- | --- | --- | --- | --- | --- | --- | --- | --- | --- | --- |
| R.AVTIFGESAGAASVGMHLLSTQSR.T | N | 110.40 | 2389.2061 | 24 | 0.0 | 797.4093 | 3 | 70.91 | 1 | 45278 | BuCaMH03.raw | 2.6603E8 | 4 | 4 | 320 | 343 |  |  | PEAKS DB |
| R.FLTYTQNVILVSLSYR.V | N | 110.00 | 1916.0408 | 16 | 0.4 | 959.0281 | 2 | 83.45 | 1 | 55322 | BuCaMH03.raw | 3.0247E7 | 3 | 3 | 261 | 276 |  |  | PEAKS DB |
| R.FPFVPVIDGDFFPDTPEAMLSSGNFK.E | N | 109.19 | 2873.3621 | 26 | 0.6 | 1437.6892 | 2 | 98.27 | 1 | 67043 | BuCaMH03.raw | 4.4491E8 | 2 | 2 | 417 | 442 |  |  | PEAKS DB |
| K.DEGSYFLIYGLPGFSK.D | N | 106.68 | 1791.8718 | 16 | 0.8 | 896.9439 | 2 | 93.19 | 1 | 62858 | BuCaMH03.raw | 2.4463E8 | 2 | 2 | 453 | 468 |  |  | PEAKS DB |
| R.AILQSGGPNAPWATVTPAESR.R | N | 105.98 | 2122.0806 | 21 | 0.3 | 1062.0479 | 2 | 59.69 | 1 | 35420 | BuCaMH03.raw | 9.8415E7 | 2 | 2 | 349 | 369 |  |  | PEAKS DB |
| K.NPQELIDEEWSVLPYK.S | N | 102.10 | 1958.9625 | 16 | 0.4 | 980.4890 | 2 | 78.54 | 1 | 51369 | BuCaMH03.raw | 6.749E7 | 2 | 2 | 397 | 412 |  |  | PEAKS DB |
| R.SKNPQELIDEEWSVLPYK.S | N | 100.91 | 2174.0896 | 18 | 2.3 | 725.7054 | 3 | 68.47 | 1 | 43161 | BuCaMH03.raw | 1.243E8 | 2 | 2 | 395 | 412 |  |  | PEAKS DB |
| M.SVPHANDIATESVVLQYTDWQDQDNR.E | Y | 97.78 | 3000.3850 | 26 | -0.1 | 1001.1355 | 3 | 64.89 | 1 | 39962 | BuCaMH03.raw | 5.6416E6 | 1 | 1 | 486 | 511 |  |  | PEAKS DB |
| R.LALQWIQNNIHPFGGNPR.A | N | 96.68 | 2074.0859 | 18 | 0.3 | 692.3694 | 3 | 67.05 | 1 | 41669 | BuCaMH03.raw | 1.057E7 | 2 | 2 | 302 | 319 |  |  | PEAKS DB |
| Q.SGGPNAPWATVTPAESR.R | N | 95.90 | 1696.8169 | 17 | -0.7 | 849.4152 | 2 | 46.64 | 1 | 24656 | BuCaMH03.raw | 4.0143E6 | 1 | 1 | 353 | 369 |  |  | PEAKS DB |
| R.EALDDIVGDHNVIC(+57.02)PVVQFANDYAK.R | N | 93.85 | 2801.3330 | 25 | -0.2 | 934.7847 | 3 | 78.96 | 1 | 51733 | BuCaMH03.raw | 2.9516E8 | 2 | 2 | 516 | 540 | Carbamidomethylation | C14:Carbamidomethylation:1000.00 | PEAKS DB |
| R.VGAFGFLGLPGSPEAPGNMGLLDQR.L | N | 92.98 | 2499.2581 | 25 | 1.1 | 834.0942 | 3 | 90.41 | 1 | 60987 | BuCaMH03.raw | 3.7724E8 | 2 | 2 | 277 | 301 |  |  | PEAKS DB |
| R.GLSLPVLDGHVTAFLGIPFAEPPVGR.M | N | 86.95 | 2658.4534 | 26 | 1.3 | 887.1595 | 3 | 96.29 | 1 | 65362 | BuCaMH03.raw | 2.0859E9 | 4 | 4 | 144 | 169 |  |  | PEAKS DB |
| A.LQWIQNNIHPFGGNPR.A | N | 86.76 | 1889.9648 | 16 | 0.8 | 945.9904 | 2 | 52.84 | 1 | 29897 | BuCaMH03.raw | 2.3373E7 | 2 | 2 | 304 | 319 |  |  | PEAKS DB |
| R.MSVPHANDIATESVVLQYTDWQDQDNR.E | Y | 85.54 | 3131.4253 | 27 | 1.1 | 1044.8169 | 3 | 69.31 | 1 | 43770 | BuCaMH03.raw | 8.1462E7 | 2 | 2 | 485 | 511 |  |  | PEAKS DB |
| L.ALQWIQNNIHPFGGNPR.A | N | 84.31 | 1961.0020 | 17 | 0.2 | 981.5084 | 2 | 56.08 | 1 | 32631 | BuCaMH03.raw | 7.657E7 | 2 | 2 | 303 | 319 |  |  | PEAKS DB |
| K.SGAWPTYTASQPQYVQLNTQPLATQPSLR.A | N | 79.74 | 3202.6047 | 29 | 0.8 | 1068.5431 | 3 | 70.50 | 1 | 44551 | BuCaMH03.raw | 4.9159E7 | 1 | 1 | 616 | 644 |  |  | PEAKS DB |
| K.SIFRFPFVPVIDGDFFPDTPEAMLSSGNFK.E | N | 79.19 | 3376.6477 | 30 | -0.6 | 1126.5558 | 3 | 98.25 | 1 | 66983 | BuCaMH03.raw | 1.9571E6 | 1 | 1 | 413 | 442 |  |  | PEAKS DB |
| R.FPFVPVIDGDFFPDTPEAM(+15.99)LSSGNFK.E | N | 79.18 | 2889.3569 | 26 | 0.0 | 964.1262 | 3 | 98.20 | 1 | 66887 | BuCaMH03.raw | 1.6186E7 | 1 | 1 | 417 | 442 | Oxidation (M) | M19:Oxidation (M):1000.00 | PEAKS DB |
| K.NREALDDIVGDHNVIC(+57.02)PVVQFANDYAK.R | N | 77.79 | 3071.4771 | 27 | 0.4 | 768.8768 | 4 | 70.23 | 1 | 44298 | BuCaMH03.raw | 9.8218E6 | 2 | 2 | 514 | 540 | Carbamidomethylation | C16:Carbamidomethylation:1000.00 | PEAKS DB |
| K.ETQVLLGVVKDEGSYFLIYGLPGFSK.D | N | 77.49 | 2858.5105 | 26 | 0.5 | 953.8446 | 3 | 94.22 | 1 | 63592 | BuCaMH03.raw | 1.1881E7 | 1 | 1 | 443 | 468 |  |  | PEAKS DB |
| K.VYAYLFDHR.A | N | 76.38 | 1182.5822 | 9 | 0.3 | 592.2985 | 2 | 41.43 | 1 | 20432 | BuCaMH03.raw | 2.3244E7 | 2 | 2 | 545 | 553 |  |  | PEAKS DB |
| R.AQIC(+57.02)AFWNHFLPK.L | N | 76.29 | 1630.8079 | 13 | 0.1 | 544.6099 | 3 | 67.38 | 1 | 41864 | BuCaMH03.raw | 6.214E6 | 1 | 1 | 645 | 657 | Carbamidomethylation | C4:Carbamidomethylation:1000.00 | PEAKS DB |
| A.VTIFGESAGAASVGMHLLSTQSR.T | N | 75.90 | 2318.1689 | 23 | 1.1 | 773.7311 | 3 | 69.24 | 1 | 43488 | BuCaMH03.raw | 1.4432E6 | 1 | 1 | 321 | 343 |  |  | PEAKS DB |
| I.C(+57.02)AFWNHFLPK.L | N | 74.57 | 1318.6281 | 10 | -0.5 | 660.3210 | 2 | 56.23 | 1 | 32768 | BuCaMH03.raw | 7.6723E6 | 1 | 1 | 648 | 657 | Carbamidomethylation | C1:Carbamidomethylation:1000.00 | PEAKS DB |
| Q.WIQNNIHPFGGNPR.A | N | 73.92 | 1648.8223 | 14 | 0.4 | 825.4187 | 2 | 39.98 | 1 | 19293 | BuCaMH03.raw | 1.286E7 | 2 | 2 | 306 | 319 |  |  | PEAKS DB |
| P.FVPVIDGDFFPDTPEAMLSSGNFK.E | N | 73.61 | 2629.2410 | 24 | 0.1 | 1315.6279 | 2 | 94.45 | 1 | 64055 | BuCaMH03.raw | 2.0905E7 | 1 | 1 | 419 | 442 |  |  | PEAKS DB |
| V.GAFGFLGLPGSPEAPGNMGLLDQR.L | N | 72.34 | 2400.1895 | 24 | 0.4 | 1201.1025 | 2 | 88.38 | 1 | 58926 | BuCaMH03.raw | 3.179E7 | 2 | 2 | 278 | 301 |  |  | PEAKS DB |
| R.MSVPHANDIATESVVLQYTDWQDQDNREK.N | Y | 71.07 | 3388.5630 | 29 | 0.2 | 848.1482 | 4 | 62.82 | 1 | 38255 | BuCaMH03.raw | 5.174E6 | 1 | 1 | 485 | 513 |  |  | PEAKS DB |
| G.LPGSPEAPGNMGLLDQR.L | N | 70.60 | 1750.8672 | 17 | 0.7 | 876.4415 | 2 | 54.97 | 1 | 31655 | BuCaMH03.raw | 4.4619E6 | 1 | 1 | 285 | 301 |  |  | PEAKS DB |
| R.FLRPEPVKPWPHVLDATSYK.P | N | 69.65 | 2379.2739 | 20 | 0.8 | 595.8262 | 4 | 48.92 | 1 | 26568 | BuCaMH03.raw | 6.2045E6 | 1 | 1 | 172 | 191 |  |  | PEAKS DB |
| Q.IC(+57.02)AFWNHFLPK.L | N | 69.60 | 1431.7122 | 11 | 0.3 | 478.2448 | 3 | 63.50 | 1 | 38737 | BuCaMH03.raw | 2.159E6 | 1 | 1 | 647 | 657 | Carbamidomethylation | C2:Carbamidomethylation:1000.00 | PEAKS DB |
| L.NATVDPPSADR.R | Y | 69.09 | 1141.5364 | 11 | -0.3 | 571.7753 | 2 | 16.18 | 1 | 3477 | BuCaMH03.raw | 1.4697E6 | 1 | 1 | 660 | 670 |  |  | PEAKS DB |
| A.QIC(+57.02)AFWNHFLPK.L | N | 68.21 | 1559.7708 | 12 | 0.3 | 520.9310 | 3 | 65.09 | 1 | 40072 | BuCaMH03.raw | 1.1741E7 | 2 | 2 | 646 | 657 | Carbamidomethylation | C3:Carbamidomethylation:1000.00 | PEAKS DB |
| K.ETQVLLGVVK.D | N | 66.91 | 1084.6492 | 10 | -0.4 | 543.3317 | 2 | 48.73 | 1 | 26723 | BuCaMH03.raw | 1.4354E8 | 1 | 1 | 443 | 452 |  |  | PEAKS DB |
| A.ILQSGGPNAPWATVTPAESR.R | N | 64.65 | 2051.0435 | 20 | 0.5 | 1026.5295 | 2 | 55.18 | 1 | 31833 | BuCaMH03.raw | 2.893E6 | 1 | 1 | 350 | 369 |  |  | PEAKS DB |
| L.SLPVLDGHVTAFLGIPFAEPPVGR.M | N | 64.20 | 2488.3477 | 24 | 1.4 | 830.4576 | 3 | 89.15 | 1 | 59573 | BuCaMH03.raw | 2.1629E7 | 1 | 1 | 146 | 169 |  |  | PEAKS DB |
| R.AVTIFGESAGAASVGMHLLSTQSRTLFQR.A | N | 62.77 | 3034.5657 | 29 | 0.9 | 759.6494 | 4 | 77.95 | 1 | 50683 | BuCaMH03.raw | 8.7118E5 | 1 | 1 | 320 | 348 |  |  | PEAKS DB |
| G.LSLPVLDGHVTAFLGIPFAEPPVGR.M | N | 61.54 | 2601.4319 | 25 | 0.0 | 868.1512 | 3 | 94.83 | 1 | 64548 | BuCaMH03.raw | 9.5169E6 | 2 | 2 | 145 | 169 |  |  | PEAKS DB |
| F.LTYTQNVILVSLSYR.V | N | 56.26 | 1768.9723 | 15 | 0.1 | 885.4935 | 2 | 75.21 | 1 | 48520 | BuCaMH03.raw | 1.4443E6 | 1 | 1 | 262 | 276 |  |  | PEAKS DB |
| P.QELIDEEWSVLPYK.S | N | 56.12 | 1747.8668 | 14 | 0.9 | 874.9415 | 2 | 73.88 | 1 | 47373 | BuCaMH03.raw | 3.1518E6 | 1 | 1 | 399 | 412 |  |  | PEAKS DB |
| L.QWIQNNIHPFGGNPR.A | N | 55.84 | 1776.8809 | 15 | -0.4 | 593.3007 | 3 | 43.44 | 1 | 22097 | BuCaMH03.raw | 2.5227E7 | 1 | 1 | 305 | 319 |  |  | PEAKS DB |
| R.AVTIFGESAGAASVGM(+15.99)HLLSTQSR.T | N | 55.79 | 2405.2009 | 24 | 0.0 | 802.7409 | 3 | 70.98 | 1 | 44980 | BuCaMH03.raw | 5.9259E6 | 1 | 1 | 320 | 343 | Oxidation (M) | M16:Oxidation (M):1000.00 | PEAKS DB |
| R.ADFLEGVR.M | N | 55.33 | 905.4606 | 8 | 0.5 | 453.7378 | 2 | 47.32 | 1 | 25289 | BuCaMH03.raw | 6.9015E7 | 2 | 2 | 477 | 484 |  |  | PEAKS DB |
| E.GSYFLIYGLPGFSK.D | N | 55.01 | 1547.8024 | 14 | 0.7 | 774.9090 | 2 | 85.55 | 1 | 56608 | BuCaMH03.raw | 1.0538E6 | 1 | 1 | 455 | 468 |  |  | PEAKS DB |
| D.GDFFPDTPEAMLSSGNFK.E | N | 54.97 | 1958.8719 | 18 | 0.7 | 980.4440 | 2 | 77.20 | 1 | 50118 | BuCaMH03.raw | 9.7457E5 | 1 | 1 | 425 | 442 |  |  | PEAKS DB |
| R.FPFVPVIDGDFFPDTPEAM.L | N | 54.90 | 2139.9863 | 19 | 0.5 | 1071.0010 | 2 | 98.34 | 1 | 67085 | BuCaMH03.raw | 5.9713E6 | 1 | 1 | 417 | 435 |  |  | PEAKS DB |
| R.FLRPEPVKPWPH.V | N | 54.64 | 1501.8193 | 12 | 0.7 | 501.6141 | 3 | 36.30 | 1 | 16415 | BuCaMH03.raw | 7.868E6 | 1 | 1 | 172 | 183 |  |  | PEAKS DB |
| L.LNATVDPPSADR.R | Y | 54.61 | 1254.6204 | 12 | 0.9 | 628.3180 | 2 | 26.78 | 1 | 9665 | BuCaMH03.raw | 7.2628E6 | 1 | 1 | 659 | 670 |  |  | PEAKS DB |
| N.PQELIDEEWSVLPYK.S | N | 54.23 | 1844.9196 | 15 | 1.3 | 923.4683 | 2 | 74.37 | 1 | 47795 | BuCaMH03.raw | 4.9513E5 | 1 | 1 | 398 | 412 |  |  | PEAKS DB |
| K.QLGC(+57.02)HFNNDSELVSC(+57.02)LR.S | N | 54.19 | 2047.9204 | 17 | 0.2 | 683.6476 | 3 | 45.75 | 1 | 23845 | BuCaMH03.raw | 8.5586E6 | 1 | 1 | 378 | 394 | Carbamidomethylation | C4:Carbamidomethylation:1000.00;C15:Carbamidomethylation:1000.00 | PEAKS DB |
| F.GFLGLPGSPEAPGNMGLLDQR.L | N | 54.11 | 2125.0625 | 21 | 0.3 | 1063.5388 | 2 | 79.70 | 1 | 52045 | BuCaMH03.raw | 1.5382E6 | 1 | 1 | 281 | 301 |  |  | PEAKS DB |
| K.LLNATVDPPSADR.R | Y | 53.44 | 1367.7045 | 13 | 0.5 | 684.8599 | 2 | 37.10 | 1 | 17092 | BuCaMH03.raw | 7.186E6 | 1 | 1 | 658 | 670 |  |  | PEAKS DB |
| R.LALQWIQNNIHPF.G | N | 53.30 | 1592.8463 | 13 | 0.4 | 797.4308 | 2 | 79.77 | 1 | 52223 | BuCaMH03.raw | 3.4534E5 | 1 | 1 | 302 | 314 |  |  | PEAKS DB |
| total 54 peptides |  |  |  |  |  |  |  |  |  |  |  |  |  |  |  |  |  |  |  |
| --- | --- | --- | --- | --- | --- | --- | --- | --- | --- | --- | --- | --- | --- | --- | --- | --- | --- | --- | --- |

T\_167

back to list

  

| Protein Coverage
| Supporting Peptides
|

Protein Coverage:

Supporting Peptides:

| Peptide | Uniq | -10lgP | Mass | Length | ppm | m/z | z | RT | Fraction | Scan | Source File | Area BuCaMH03\_B17 | #Feature | #Feature BuCaMH03\_B17 | Start | End | PTM | AScore | Found By |
| --- | --- | --- | --- | --- | --- | --- | --- | --- | --- | --- | --- | --- | --- | --- | --- | --- | --- | --- | --- |
| R.AVTIFGESAGAASVGMHLLSTQSR.T | N | 110.40 | 2389.2061 | 24 | 0.0 | 797.4093 | 3 | 70.91 | 1 | 45278 | BuCaMH03.raw | 2.6603E8 | 4 | 4 | 342 | 365 |  |  | PEAKS DB |
| R.FLTYTQNVILVSLSYR.V | N | 110.00 | 1916.0408 | 16 | 0.4 | 959.0281 | 2 | 83.45 | 1 | 55322 | BuCaMH03.raw | 3.0247E7 | 3 | 3 | 283 | 298 |  |  | PEAKS DB |
| R.FPFVPVIDGDFFPDTPEAMLSSGNFK.E | N | 109.19 | 2873.3621 | 26 | 0.6 | 1437.6892 | 2 | 98.27 | 1 | 67043 | BuCaMH03.raw | 4.4491E8 | 2 | 2 | 439 | 464 |  |  | PEAKS DB |
| K.DEGSYFLIYGLPGFSK.D | N | 106.68 | 1791.8718 | 16 | 0.8 | 896.9439 | 2 | 93.19 | 1 | 62858 | BuCaMH03.raw | 2.4463E8 | 2 | 2 | 475 | 490 |  |  | PEAKS DB |
| R.AILQSGGPNAPWATVTPAESR.R | N | 105.98 | 2122.0806 | 21 | 0.3 | 1062.0479 | 2 | 59.69 | 1 | 35420 | BuCaMH03.raw | 9.8415E7 | 2 | 2 | 371 | 391 |  |  | PEAKS DB |
| K.NPQELIDEEWSVLPYK.S | N | 102.10 | 1958.9625 | 16 | 0.4 | 980.4890 | 2 | 78.54 | 1 | 51369 | BuCaMH03.raw | 6.749E7 | 2 | 2 | 419 | 434 |  |  | PEAKS DB |
| R.SKNPQELIDEEWSVLPYK.S | N | 100.91 | 2174.0896 | 18 | 2.3 | 725.7054 | 3 | 68.47 | 1 | 43161 | BuCaMH03.raw | 1.243E8 | 2 | 2 | 417 | 434 |  |  | PEAKS DB |
| M.SVPHANDIATESVVLQYTDWQDQDNR.E | Y | 97.78 | 3000.3850 | 26 | -0.1 | 1001.1355 | 3 | 64.89 | 1 | 39962 | BuCaMH03.raw | 5.6416E6 | 1 | 1 | 508 | 533 |  |  | PEAKS DB |
| R.LALQWIQNNIHPFGGNPR.A | N | 96.68 | 2074.0859 | 18 | 0.3 | 692.3694 | 3 | 67.05 | 1 | 41669 | BuCaMH03.raw | 1.057E7 | 2 | 2 | 324 | 341 |  |  | PEAKS DB |
| Q.SGGPNAPWATVTPAESR.R | N | 95.90 | 1696.8169 | 17 | -0.7 | 849.4152 | 2 | 46.64 | 1 | 24656 | BuCaMH03.raw | 4.0143E6 | 1 | 1 | 375 | 391 |  |  | PEAKS DB |
| R.EALDDIVGDHNVIC(+57.02)PVVQFANDYAK.R | N | 93.85 | 2801.3330 | 25 | -0.2 | 934.7847 | 3 | 78.96 | 1 | 51733 | BuCaMH03.raw | 2.9516E8 | 2 | 2 | 538 | 562 | Carbamidomethylation | C14:Carbamidomethylation:1000.00 | PEAKS DB |
| R.VGAFGFLGLPGSPEAPGNMGLLDQR.L | N | 92.98 | 2499.2581 | 25 | 1.1 | 834.0942 | 3 | 90.41 | 1 | 60987 | BuCaMH03.raw | 3.7724E8 | 2 | 2 | 299 | 323 |  |  | PEAKS DB |
| R.GLSLPVLDGHVTAFLGIPFAEPPVGR.M | N | 86.95 | 2658.4534 | 26 | 1.3 | 887.1595 | 3 | 96.29 | 1 | 65362 | BuCaMH03.raw | 2.0859E9 | 4 | 4 | 166 | 191 |  |  | PEAKS DB |
| A.LQWIQNNIHPFGGNPR.A | N | 86.76 | 1889.9648 | 16 | 0.8 | 945.9904 | 2 | 52.84 | 1 | 29897 | BuCaMH03.raw | 2.3373E7 | 2 | 2 | 326 | 341 |  |  | PEAKS DB |
| R.MSVPHANDIATESVVLQYTDWQDQDNR.E | Y | 85.54 | 3131.4253 | 27 | 1.1 | 1044.8169 | 3 | 69.31 | 1 | 43770 | BuCaMH03.raw | 8.1462E7 | 2 | 2 | 507 | 533 |  |  | PEAKS DB |
| L.ALQWIQNNIHPFGGNPR.A | N | 84.31 | 1961.0020 | 17 | 0.2 | 981.5084 | 2 | 56.08 | 1 | 32631 | BuCaMH03.raw | 7.657E7 | 2 | 2 | 325 | 341 |  |  | PEAKS DB |
| K.SGAWPTYTASQPQYVQLNTQPLATQPSLR.A | N | 79.74 | 3202.6047 | 29 | 0.8 | 1068.5431 | 3 | 70.50 | 1 | 44551 | BuCaMH03.raw | 4.9159E7 | 1 | 1 | 638 | 666 |  |  | PEAKS DB |
| K.SIFRFPFVPVIDGDFFPDTPEAMLSSGNFK.E | N | 79.19 | 3376.6477 | 30 | -0.6 | 1126.5558 | 3 | 98.25 | 1 | 66983 | BuCaMH03.raw | 1.9571E6 | 1 | 1 | 435 | 464 |  |  | PEAKS DB |
| R.FPFVPVIDGDFFPDTPEAM(+15.99)LSSGNFK.E | N | 79.18 | 2889.3569 | 26 | 0.0 | 964.1262 | 3 | 98.20 | 1 | 66887 | BuCaMH03.raw | 1.6186E7 | 1 | 1 | 439 | 464 | Oxidation (M) | M19:Oxidation (M):1000.00 | PEAKS DB |
| K.NREALDDIVGDHNVIC(+57.02)PVVQFANDYAK.R | N | 77.79 | 3071.4771 | 27 | 0.4 | 768.8768 | 4 | 70.23 | 1 | 44298 | BuCaMH03.raw | 9.8218E6 | 2 | 2 | 536 | 562 | Carbamidomethylation | C16:Carbamidomethylation:1000.00 | PEAKS DB |
| K.ETQVLLGVVKDEGSYFLIYGLPGFSK.D | N | 77.49 | 2858.5105 | 26 | 0.5 | 953.8446 | 3 | 94.22 | 1 | 63592 | BuCaMH03.raw | 1.1881E7 | 1 | 1 | 465 | 490 |  |  | PEAKS DB |
| K.VYAYLFDHR.A | N | 76.38 | 1182.5822 | 9 | 0.3 | 592.2985 | 2 | 41.43 | 1 | 20432 | BuCaMH03.raw | 2.3244E7 | 2 | 2 | 567 | 575 |  |  | PEAKS DB |
| R.AQIC(+57.02)AFWNHFLPK.L | N | 76.29 | 1630.8079 | 13 | 0.1 | 544.6099 | 3 | 67.38 | 1 | 41864 | BuCaMH03.raw | 6.214E6 | 1 | 1 | 667 | 679 | Carbamidomethylation | C4:Carbamidomethylation:1000.00 | PEAKS DB |
| A.VTIFGESAGAASVGMHLLSTQSR.T | N | 75.90 | 2318.1689 | 23 | 1.1 | 773.7311 | 3 | 69.24 | 1 | 43488 | BuCaMH03.raw | 1.4432E6 | 1 | 1 | 343 | 365 |  |  | PEAKS DB |
| I.C(+57.02)AFWNHFLPK.L | N | 74.57 | 1318.6281 | 10 | -0.5 | 660.3210 | 2 | 56.23 | 1 | 32768 | BuCaMH03.raw | 7.6723E6 | 1 | 1 | 670 | 679 | Carbamidomethylation | C1:Carbamidomethylation:1000.00 | PEAKS DB |
| Q.WIQNNIHPFGGNPR.A | N | 73.92 | 1648.8223 | 14 | 0.4 | 825.4187 | 2 | 39.98 | 1 | 19293 | BuCaMH03.raw | 1.286E7 | 2 | 2 | 328 | 341 |  |  | PEAKS DB |
| P.FVPVIDGDFFPDTPEAMLSSGNFK.E | N | 73.61 | 2629.2410 | 24 | 0.1 | 1315.6279 | 2 | 94.45 | 1 | 64055 | BuCaMH03.raw | 2.0905E7 | 1 | 1 | 441 | 464 |  |  | PEAKS DB |
| V.GAFGFLGLPGSPEAPGNMGLLDQR.L | N | 72.34 | 2400.1895 | 24 | 0.4 | 1201.1025 | 2 | 88.38 | 1 | 58926 | BuCaMH03.raw | 3.179E7 | 2 | 2 | 300 | 323 |  |  | PEAKS DB |
| R.MSVPHANDIATESVVLQYTDWQDQDNREK.N | Y | 71.07 | 3388.5630 | 29 | 0.2 | 848.1482 | 4 | 62.82 | 1 | 38255 | BuCaMH03.raw | 5.174E6 | 1 | 1 | 507 | 535 |  |  | PEAKS DB |
| G.LPGSPEAPGNMGLLDQR.L | N | 70.60 | 1750.8672 | 17 | 0.7 | 876.4415 | 2 | 54.97 | 1 | 31655 | BuCaMH03.raw | 4.4619E6 | 1 | 1 | 307 | 323 |  |  | PEAKS DB |
| R.FLRPEPVKPWPHVLDATSYK.P | N | 69.65 | 2379.2739 | 20 | 0.8 | 595.8262 | 4 | 48.92 | 1 | 26568 | BuCaMH03.raw | 6.2045E6 | 1 | 1 | 194 | 213 |  |  | PEAKS DB |
| Q.IC(+57.02)AFWNHFLPK.L | N | 69.60 | 1431.7122 | 11 | 0.3 | 478.2448 | 3 | 63.50 | 1 | 38737 | BuCaMH03.raw | 2.159E6 | 1 | 1 | 669 | 679 | Carbamidomethylation | C2:Carbamidomethylation:1000.00 | PEAKS DB |
| L.NATVDPPSADR.R | Y | 69.09 | 1141.5364 | 11 | -0.3 | 571.7753 | 2 | 16.18 | 1 | 3477 | BuCaMH03.raw | 1.4697E6 | 1 | 1 | 682 | 692 |  |  | PEAKS DB |
| A.QIC(+57.02)AFWNHFLPK.L | N | 68.21 | 1559.7708 | 12 | 0.3 | 520.9310 | 3 | 65.09 | 1 | 40072 | BuCaMH03.raw | 1.1741E7 | 2 | 2 | 668 | 679 | Carbamidomethylation | C3:Carbamidomethylation:1000.00 | PEAKS DB |
| K.ETQVLLGVVK.D | N | 66.91 | 1084.6492 | 10 | -0.4 | 543.3317 | 2 | 48.73 | 1 | 26723 | BuCaMH03.raw | 1.4354E8 | 1 | 1 | 465 | 474 |  |  | PEAKS DB |
| A.ILQSGGPNAPWATVTPAESR.R | N | 64.65 | 2051.0435 | 20 | 0.5 | 1026.5295 | 2 | 55.18 | 1 | 31833 | BuCaMH03.raw | 2.893E6 | 1 | 1 | 372 | 391 |  |  | PEAKS DB |
| L.SLPVLDGHVTAFLGIPFAEPPVGR.M | N | 64.20 | 2488.3477 | 24 | 1.4 | 830.4576 | 3 | 89.15 | 1 | 59573 | BuCaMH03.raw | 2.1629E7 | 1 | 1 | 168 | 191 |  |  | PEAKS DB |
| R.AVTIFGESAGAASVGMHLLSTQSRTLFQR.A | N | 62.77 | 3034.5657 | 29 | 0.9 | 759.6494 | 4 | 77.95 | 1 | 50683 | BuCaMH03.raw | 8.7118E5 | 1 | 1 | 342 | 370 |  |  | PEAKS DB |
| G.LSLPVLDGHVTAFLGIPFAEPPVGR.M | N | 61.54 | 2601.4319 | 25 | 0.0 | 868.1512 | 3 | 94.83 | 1 | 64548 | BuCaMH03.raw | 9.5169E6 | 2 | 2 | 167 | 191 |  |  | PEAKS DB |
| F.LTYTQNVILVSLSYR.V | N | 56.26 | 1768.9723 | 15 | 0.1 | 885.4935 | 2 | 75.21 | 1 | 48520 | BuCaMH03.raw | 1.4443E6 | 1 | 1 | 284 | 298 |  |  | PEAKS DB |
| P.QELIDEEWSVLPYK.S | N | 56.12 | 1747.8668 | 14 | 0.9 | 874.9415 | 2 | 73.88 | 1 | 47373 | BuCaMH03.raw | 3.1518E6 | 1 | 1 | 421 | 434 |  |  | PEAKS DB |
| L.QWIQNNIHPFGGNPR.A | N | 55.84 | 1776.8809 | 15 | -0.4 | 593.3007 | 3 | 43.44 | 1 | 22097 | BuCaMH03.raw | 2.5227E7 | 1 | 1 | 327 | 341 |  |  | PEAKS DB |
| R.AVTIFGESAGAASVGM(+15.99)HLLSTQSR.T | N | 55.79 | 2405.2009 | 24 | 0.0 | 802.7409 | 3 | 70.98 | 1 | 44980 | BuCaMH03.raw | 5.9259E6 | 1 | 1 | 342 | 365 | Oxidation (M) | M16:Oxidation (M):1000.00 | PEAKS DB |
| R.ADFLEGVR.M | N | 55.33 | 905.4606 | 8 | 0.5 | 453.7378 | 2 | 47.32 | 1 | 25289 | BuCaMH03.raw | 6.9015E7 | 2 | 2 | 499 | 506 |  |  | PEAKS DB |
| E.GSYFLIYGLPGFSK.D | N | 55.01 | 1547.8024 | 14 | 0.7 | 774.9090 | 2 | 85.55 | 1 | 56608 | BuCaMH03.raw | 1.0538E6 | 1 | 1 | 477 | 490 |  |  | PEAKS DB |
| D.GDFFPDTPEAMLSSGNFK.E | N | 54.97 | 1958.8719 | 18 | 0.7 | 980.4440 | 2 | 77.20 | 1 | 50118 | BuCaMH03.raw | 9.7457E5 | 1 | 1 | 447 | 464 |  |  | PEAKS DB |
| R.FPFVPVIDGDFFPDTPEAM.L | N | 54.90 | 2139.9863 | 19 | 0.5 | 1071.0010 | 2 | 98.34 | 1 | 67085 | BuCaMH03.raw | 5.9713E6 | 1 | 1 | 439 | 457 |  |  | PEAKS DB |
| R.FLRPEPVKPWPH.V | N | 54.64 | 1501.8193 | 12 | 0.7 | 501.6141 | 3 | 36.30 | 1 | 16415 | BuCaMH03.raw | 7.868E6 | 1 | 1 | 194 | 205 |  |  | PEAKS DB |
| L.LNATVDPPSADR.R | Y | 54.61 | 1254.6204 | 12 | 0.9 | 628.3180 | 2 | 26.78 | 1 | 9665 | BuCaMH03.raw | 7.2628E6 | 1 | 1 | 681 | 692 |  |  | PEAKS DB |
| N.PQELIDEEWSVLPYK.S | N | 54.23 | 1844.9196 | 15 | 1.3 | 923.4683 | 2 | 74.37 | 1 | 47795 | BuCaMH03.raw | 4.9513E5 | 1 | 1 | 420 | 434 |  |  | PEAKS DB |
| K.QLGC(+57.02)HFNNDSELVSC(+57.02)LR.S | N | 54.19 | 2047.9204 | 17 | 0.2 | 683.6476 | 3 | 45.75 | 1 | 23845 | BuCaMH03.raw | 8.5586E6 | 1 | 1 | 400 | 416 | Carbamidomethylation | C4:Carbamidomethylation:1000.00;C15:Carbamidomethylation:1000.00 | PEAKS DB |
| F.GFLGLPGSPEAPGNMGLLDQR.L | N | 54.11 | 2125.0625 | 21 | 0.3 | 1063.5388 | 2 | 79.70 | 1 | 52045 | BuCaMH03.raw | 1.5382E6 | 1 | 1 | 303 | 323 |  |  | PEAKS DB |
| K.LLNATVDPPSADR.R | Y | 53.44 | 1367.7045 | 13 | 0.5 | 684.8599 | 2 | 37.10 | 1 | 17092 | BuCaMH03.raw | 7.186E6 | 1 | 1 | 680 | 692 |  |  | PEAKS DB |
| R.LALQWIQNNIHPF.G | N | 53.30 | 1592.8463 | 13 | 0.4 | 797.4308 | 2 | 79.77 | 1 | 52223 | BuCaMH03.raw | 3.4534E5 | 1 | 1 | 324 | 336 |  |  | PEAKS DB |
| total 54 peptides |  |  |  |  |  |  |  |  |  |  |  |  |  |  |  |  |  |  |  |
| --- | --- | --- | --- | --- | --- | --- | --- | --- | --- | --- | --- | --- | --- | --- | --- | --- | --- | --- | --- |

T\_170

back to list

  

| Protein Coverage
| Supporting Peptides
|

Protein Coverage:

Supporting Peptides:

| Peptide | Uniq | -10lgP | Mass | Length | ppm | m/z | z | RT | Fraction | Scan | Source File | Area BuCaMH03\_B17 | #Feature | #Feature BuCaMH03\_B17 | Start | End | PTM | AScore | Found By |
| --- | --- | --- | --- | --- | --- | --- | --- | --- | --- | --- | --- | --- | --- | --- | --- | --- | --- | --- | --- |
| R.AVTIFGESAGAASVGMHLLSTQSR.T | N | 110.40 | 2389.2061 | 24 | 0.0 | 797.4093 | 3 | 70.91 | 1 | 45278 | BuCaMH03.raw | 2.6603E8 | 4 | 4 | 222 | 245 |  |  | PEAKS DB |
| R.FLTYTQNVILVSLSYR.V | N | 110.00 | 1916.0408 | 16 | 0.4 | 959.0281 | 2 | 83.45 | 1 | 55322 | BuCaMH03.raw | 3.0247E7 | 3 | 3 | 163 | 178 |  |  | PEAKS DB |
| R.FPFVPVIDGDFFPDTPEAMLSSGNFK.E | N | 109.19 | 2873.3621 | 26 | 0.6 | 1437.6892 | 2 | 98.27 | 1 | 67043 | BuCaMH03.raw | 4.4491E8 | 2 | 2 | 319 | 344 |  |  | PEAKS DB |
| K.DEGSYFLIYGLPGFSK.D | N | 106.68 | 1791.8718 | 16 | 0.8 | 896.9439 | 2 | 93.19 | 1 | 62858 | BuCaMH03.raw | 2.4463E8 | 2 | 2 | 355 | 370 |  |  | PEAKS DB |
| R.AILQSGGPNAPWATVTPAESR.R | N | 105.98 | 2122.0806 | 21 | 0.3 | 1062.0479 | 2 | 59.69 | 1 | 35420 | BuCaMH03.raw | 9.8415E7 | 2 | 2 | 251 | 271 |  |  | PEAKS DB |
| K.NPQELIDEEWSVLPYK.S | N | 102.10 | 1958.9625 | 16 | 0.4 | 980.4890 | 2 | 78.54 | 1 | 51369 | BuCaMH03.raw | 6.749E7 | 2 | 2 | 299 | 314 |  |  | PEAKS DB |
| R.SKNPQELIDEEWSVLPYK.S | N | 100.91 | 2174.0896 | 18 | 2.3 | 725.7054 | 3 | 68.47 | 1 | 43161 | BuCaMH03.raw | 1.243E8 | 2 | 2 | 297 | 314 |  |  | PEAKS DB |
| R.LALQWIQNNIHPFGGNPR.A | N | 96.68 | 2074.0859 | 18 | 0.3 | 692.3694 | 3 | 67.05 | 1 | 41669 | BuCaMH03.raw | 1.057E7 | 2 | 2 | 204 | 221 |  |  | PEAKS DB |
| Q.SGGPNAPWATVTPAESR.R | N | 95.90 | 1696.8169 | 17 | -0.7 | 849.4152 | 2 | 46.64 | 1 | 24656 | BuCaMH03.raw | 4.0143E6 | 1 | 1 | 255 | 271 |  |  | PEAKS DB |
| R.MSVPHANDIATEAVVLQYTDWQDQDNR.E | Y | 94.52 | 3115.4304 | 27 | 0.3 | 1039.4844 | 3 | 72.50 | 1 | 46334 | BuCaMH03.raw | 1.2758E8 | 2 | 2 | 387 | 413 |  |  | PEAKS DB |
| R.EALDDIVGDHNVIC(+57.02)PVVQFANDYAK.R | N | 93.85 | 2801.3330 | 25 | -0.2 | 934.7847 | 3 | 78.96 | 1 | 51733 | BuCaMH03.raw | 2.9516E8 | 2 | 2 | 418 | 442 | Carbamidomethylation | C14:Carbamidomethylation:1000.00 | PEAKS DB |
| R.VGAFGFLGLPGSPEAPGNMGLLDQR.L | N | 92.98 | 2499.2581 | 25 | 1.1 | 834.0942 | 3 | 90.41 | 1 | 60987 | BuCaMH03.raw | 3.7724E8 | 2 | 2 | 179 | 203 |  |  | PEAKS DB |
| R.GLSLPVLDGHVTAFLGIPFAEPPVGR.M | N | 86.95 | 2658.4534 | 26 | 1.3 | 887.1595 | 3 | 96.29 | 1 | 65362 | BuCaMH03.raw | 2.0859E9 | 4 | 4 | 46 | 71 |  |  | PEAKS DB |
| A.LQWIQNNIHPFGGNPR.A | N | 86.76 | 1889.9648 | 16 | 0.8 | 945.9904 | 2 | 52.84 | 1 | 29897 | BuCaMH03.raw | 2.3373E7 | 2 | 2 | 206 | 221 |  |  | PEAKS DB |
| L.ALQWIQNNIHPFGGNPR.A | N | 84.31 | 1961.0020 | 17 | 0.2 | 981.5084 | 2 | 56.08 | 1 | 32631 | BuCaMH03.raw | 7.657E7 | 2 | 2 | 205 | 221 |  |  | PEAKS DB |
| K.SGAWPTYTASQPQYVQLNTQPLATQPSLR.A | N | 79.74 | 3202.6047 | 29 | 0.8 | 1068.5431 | 3 | 70.50 | 1 | 44551 | BuCaMH03.raw | 4.9159E7 | 1 | 1 | 518 | 546 |  |  | PEAKS DB |
| K.SIFRFPFVPVIDGDFFPDTPEAMLSSGNFK.E | N | 79.19 | 3376.6477 | 30 | -0.6 | 1126.5558 | 3 | 98.25 | 1 | 66983 | BuCaMH03.raw | 1.9571E6 | 1 | 1 | 315 | 344 |  |  | PEAKS DB |
| R.FPFVPVIDGDFFPDTPEAM(+15.99)LSSGNFK.E | N | 79.18 | 2889.3569 | 26 | 0.0 | 964.1262 | 3 | 98.20 | 1 | 66887 | BuCaMH03.raw | 1.6186E7 | 1 | 1 | 319 | 344 | Oxidation (M) | M19:Oxidation (M):1000.00 | PEAKS DB |
| K.NREALDDIVGDHNVIC(+57.02)PVVQFANDYAK.R | N | 77.79 | 3071.4771 | 27 | 0.4 | 768.8768 | 4 | 70.23 | 1 | 44298 | BuCaMH03.raw | 9.8218E6 | 2 | 2 | 416 | 442 | Carbamidomethylation | C16:Carbamidomethylation:1000.00 | PEAKS DB |
| K.ETQVLLGVVKDEGSYFLIYGLPGFSK.D | N | 77.49 | 2858.5105 | 26 | 0.5 | 953.8446 | 3 | 94.22 | 1 | 63592 | BuCaMH03.raw | 1.1881E7 | 1 | 1 | 345 | 370 |  |  | PEAKS DB |
| K.VYAYLFDHR.A | N | 76.38 | 1182.5822 | 9 | 0.3 | 592.2985 | 2 | 41.43 | 1 | 20432 | BuCaMH03.raw | 2.3244E7 | 2 | 2 | 447 | 455 |  |  | PEAKS DB |
| R.AQIC(+57.02)AFWNHFLPK.L | N | 76.29 | 1630.8079 | 13 | 0.1 | 544.6099 | 3 | 67.38 | 1 | 41864 | BuCaMH03.raw | 6.214E6 | 1 | 1 | 547 | 559 | Carbamidomethylation | C4:Carbamidomethylation:1000.00 | PEAKS DB |
| A.VTIFGESAGAASVGMHLLSTQSR.T | N | 75.90 | 2318.1689 | 23 | 1.1 | 773.7311 | 3 | 69.24 | 1 | 43488 | BuCaMH03.raw | 1.4432E6 | 1 | 1 | 223 | 245 |  |  | PEAKS DB |
| I.C(+57.02)AFWNHFLPK.L | N | 74.57 | 1318.6281 | 10 | -0.5 | 660.3210 | 2 | 56.23 | 1 | 32768 | BuCaMH03.raw | 7.6723E6 | 1 | 1 | 550 | 559 | Carbamidomethylation | C1:Carbamidomethylation:1000.00 | PEAKS DB |
| Q.WIQNNIHPFGGNPR.A | N | 73.92 | 1648.8223 | 14 | 0.4 | 825.4187 | 2 | 39.98 | 1 | 19293 | BuCaMH03.raw | 1.286E7 | 2 | 2 | 208 | 221 |  |  | PEAKS DB |
| P.FVPVIDGDFFPDTPEAMLSSGNFK.E | N | 73.61 | 2629.2410 | 24 | 0.1 | 1315.6279 | 2 | 94.45 | 1 | 64055 | BuCaMH03.raw | 2.0905E7 | 1 | 1 | 321 | 344 |  |  | PEAKS DB |
| V.GAFGFLGLPGSPEAPGNMGLLDQR.L | N | 72.34 | 2400.1895 | 24 | 0.4 | 1201.1025 | 2 | 88.38 | 1 | 58926 | BuCaMH03.raw | 3.179E7 | 2 | 2 | 180 | 203 |  |  | PEAKS DB |
| H.ANDIATEAVVLQYTDWQDQDNR.E | Y | 72.06 | 2564.1780 | 22 | 1.6 | 855.7346 | 3 | 75.14 | 1 | 48499 | BuCaMH03.raw | 5.1842E5 | 1 | 1 | 392 | 413 |  |  | PEAKS DB |
| G.LPGSPEAPGNMGLLDQR.L | N | 70.60 | 1750.8672 | 17 | 0.7 | 876.4415 | 2 | 54.97 | 1 | 31655 | BuCaMH03.raw | 4.4619E6 | 1 | 1 | 187 | 203 |  |  | PEAKS DB |
| R.FLRPEPVKPWPHVLDATSYK.P | N | 69.65 | 2379.2739 | 20 | 0.8 | 595.8262 | 4 | 48.92 | 1 | 26568 | BuCaMH03.raw | 6.2045E6 | 1 | 1 | 74 | 93 |  |  | PEAKS DB |
| Q.IC(+57.02)AFWNHFLPK.L | N | 69.60 | 1431.7122 | 11 | 0.3 | 478.2448 | 3 | 63.50 | 1 | 38737 | BuCaMH03.raw | 2.159E6 | 1 | 1 | 549 | 559 | Carbamidomethylation | C2:Carbamidomethylation:1000.00 | PEAKS DB |
| A.QIC(+57.02)AFWNHFLPK.L | N | 68.21 | 1559.7708 | 12 | 0.3 | 520.9310 | 3 | 65.09 | 1 | 40072 | BuCaMH03.raw | 1.1741E7 | 2 | 2 | 548 | 559 | Carbamidomethylation | C3:Carbamidomethylation:1000.00 | PEAKS DB |
| K.ETQVLLGVVK.D | N | 66.91 | 1084.6492 | 10 | -0.4 | 543.3317 | 2 | 48.73 | 1 | 26723 | BuCaMH03.raw | 1.4354E8 | 1 | 1 | 345 | 354 |  |  | PEAKS DB |
| A.ILQSGGPNAPWATVTPAESR.R | N | 64.65 | 2051.0435 | 20 | 0.5 | 1026.5295 | 2 | 55.18 | 1 | 31833 | BuCaMH03.raw | 2.893E6 | 1 | 1 | 252 | 271 |  |  | PEAKS DB |
| L.SLPVLDGHVTAFLGIPFAEPPVGR.M | N | 64.20 | 2488.3477 | 24 | 1.4 | 830.4576 | 3 | 89.15 | 1 | 59573 | BuCaMH03.raw | 2.1629E7 | 1 | 1 | 48 | 71 |  |  | PEAKS DB |
| R.AVTIFGESAGAASVGMHLLSTQSRTLFQR.A | N | 62.77 | 3034.5657 | 29 | 0.9 | 759.6494 | 4 | 77.95 | 1 | 50683 | BuCaMH03.raw | 8.7118E5 | 1 | 1 | 222 | 250 |  |  | PEAKS DB |
| G.LSLPVLDGHVTAFLGIPFAEPPVGR.M | N | 61.54 | 2601.4319 | 25 | 0.0 | 868.1512 | 3 | 94.83 | 1 | 64548 | BuCaMH03.raw | 9.5169E6 | 2 | 2 | 47 | 71 |  |  | PEAKS DB |
| M.SVPHANDIATEAVVLQYTDWQDQDNR.E | Y | 57.10 | 2984.3899 | 26 | 0.2 | 995.8041 | 3 | 68.45 | 1 | 42800 | BuCaMH03.raw | 9.6717E6 | 1 | 1 | 388 | 413 |  |  | PEAKS DB |
| F.LTYTQNVILVSLSYR.V | N | 56.26 | 1768.9723 | 15 | 0.1 | 885.4935 | 2 | 75.21 | 1 | 48520 | BuCaMH03.raw | 1.4443E6 | 1 | 1 | 164 | 178 |  |  | PEAKS DB |
| P.QELIDEEWSVLPYK.S | N | 56.12 | 1747.8668 | 14 | 0.9 | 874.9415 | 2 | 73.88 | 1 | 47373 | BuCaMH03.raw | 3.1518E6 | 1 | 1 | 301 | 314 |  |  | PEAKS DB |
| L.QWIQNNIHPFGGNPR.A | N | 55.84 | 1776.8809 | 15 | -0.4 | 593.3007 | 3 | 43.44 | 1 | 22097 | BuCaMH03.raw | 2.5227E7 | 1 | 1 | 207 | 221 |  |  | PEAKS DB |
| R.AVTIFGESAGAASVGM(+15.99)HLLSTQSR.T | N | 55.79 | 2405.2009 | 24 | 0.0 | 802.7409 | 3 | 70.98 | 1 | 44980 | BuCaMH03.raw | 5.9259E6 | 1 | 1 | 222 | 245 | Oxidation (M) | M16:Oxidation (M):1000.00 | PEAKS DB |
| R.ADFLEGVR.M | N | 55.33 | 905.4606 | 8 | 0.5 | 453.7378 | 2 | 47.32 | 1 | 25289 | BuCaMH03.raw | 6.9015E7 | 2 | 2 | 379 | 386 |  |  | PEAKS DB |
| E.GSYFLIYGLPGFSK.D | N | 55.01 | 1547.8024 | 14 | 0.7 | 774.9090 | 2 | 85.55 | 1 | 56608 | BuCaMH03.raw | 1.0538E6 | 1 | 1 | 357 | 370 |  |  | PEAKS DB |
| D.GDFFPDTPEAMLSSGNFK.E | N | 54.97 | 1958.8719 | 18 | 0.7 | 980.4440 | 2 | 77.20 | 1 | 50118 | BuCaMH03.raw | 9.7457E5 | 1 | 1 | 327 | 344 |  |  | PEAKS DB |
| R.FPFVPVIDGDFFPDTPEAM.L | N | 54.90 | 2139.9863 | 19 | 0.5 | 1071.0010 | 2 | 98.34 | 1 | 67085 | BuCaMH03.raw | 5.9713E6 | 1 | 1 | 319 | 337 |  |  | PEAKS DB |
| R.FLRPEPVKPWPH.V | N | 54.64 | 1501.8193 | 12 | 0.7 | 501.6141 | 3 | 36.30 | 1 | 16415 | BuCaMH03.raw | 7.868E6 | 1 | 1 | 74 | 85 |  |  | PEAKS DB |
| N.PQELIDEEWSVLPYK.S | N | 54.23 | 1844.9196 | 15 | 1.3 | 923.4683 | 2 | 74.37 | 1 | 47795 | BuCaMH03.raw | 4.9513E5 | 1 | 1 | 300 | 314 |  |  | PEAKS DB |
| K.QLGC(+57.02)HFNNDSELVSC(+57.02)LR.S | N | 54.19 | 2047.9204 | 17 | 0.2 | 683.6476 | 3 | 45.75 | 1 | 23845 | BuCaMH03.raw | 8.5586E6 | 1 | 1 | 280 | 296 | Carbamidomethylation | C4:Carbamidomethylation:1000.00;C15:Carbamidomethylation:1000.00 | PEAKS DB |
| F.GFLGLPGSPEAPGNMGLLDQR.L | N | 54.11 | 2125.0625 | 21 | 0.3 | 1063.5388 | 2 | 79.70 | 1 | 52045 | BuCaMH03.raw | 1.5382E6 | 1 | 1 | 183 | 203 |  |  | PEAKS DB |
| R.LALQWIQNNIHPF.G | N | 53.30 | 1592.8463 | 13 | 0.4 | 797.4308 | 2 | 79.77 | 1 | 52223 | BuCaMH03.raw | 3.4534E5 | 1 | 1 | 204 | 216 |  |  | PEAKS DB |
| total 51 peptides |  |  |  |  |  |  |  |  |  |  |  |  |  |  |  |  |  |  |  |
| --- | --- | --- | --- | --- | --- | --- | --- | --- | --- | --- | --- | --- | --- | --- | --- | --- | --- | --- | --- |

T\_168

back to list

  

| Protein Coverage
| Supporting Peptides
|

Protein Coverage:

Supporting Peptides:

| Peptide | Uniq | -10lgP | Mass | Length | ppm | m/z | z | RT | Fraction | Scan | Source File | Area BuCaMH03\_B17 | #Feature | #Feature BuCaMH03\_B17 | Start | End | PTM | AScore | Found By |
| --- | --- | --- | --- | --- | --- | --- | --- | --- | --- | --- | --- | --- | --- | --- | --- | --- | --- | --- | --- |
| R.AVTIFGESAGAASVGMHLLSTQSR.T | N | 110.40 | 2389.2061 | 24 | 0.0 | 797.4093 | 3 | 70.91 | 1 | 45278 | BuCaMH03.raw | 2.6603E8 | 4 | 4 | 224 | 247 |  |  | PEAKS DB |
| R.FLTYTQNVILVSLSYR.V | N | 110.00 | 1916.0408 | 16 | 0.4 | 959.0281 | 2 | 83.45 | 1 | 55322 | BuCaMH03.raw | 3.0247E7 | 3 | 3 | 165 | 180 |  |  | PEAKS DB |
| R.FPFVPVIDGDFFPDTPEAMLSSGNFK.E | N | 109.19 | 2873.3621 | 26 | 0.6 | 1437.6892 | 2 | 98.27 | 1 | 67043 | BuCaMH03.raw | 4.4491E8 | 2 | 2 | 321 | 346 |  |  | PEAKS DB |
| K.DEGSYFLIYGLPGFSK.D | N | 106.68 | 1791.8718 | 16 | 0.8 | 896.9439 | 2 | 93.19 | 1 | 62858 | BuCaMH03.raw | 2.4463E8 | 2 | 2 | 357 | 372 |  |  | PEAKS DB |
| R.AILQSGGPNAPWATVTPAESR.R | N | 105.98 | 2122.0806 | 21 | 0.3 | 1062.0479 | 2 | 59.69 | 1 | 35420 | BuCaMH03.raw | 9.8415E7 | 2 | 2 | 253 | 273 |  |  | PEAKS DB |
| K.NPQELIDEEWSVLPYK.S | N | 102.10 | 1958.9625 | 16 | 0.4 | 980.4890 | 2 | 78.54 | 1 | 51369 | BuCaMH03.raw | 6.749E7 | 2 | 2 | 301 | 316 |  |  | PEAKS DB |
| R.SKNPQELIDEEWSVLPYK.S | N | 100.91 | 2174.0896 | 18 | 2.3 | 725.7054 | 3 | 68.47 | 1 | 43161 | BuCaMH03.raw | 1.243E8 | 2 | 2 | 299 | 316 |  |  | PEAKS DB |
| R.LALQWIQNNIHPFGGNPR.A | N | 96.68 | 2074.0859 | 18 | 0.3 | 692.3694 | 3 | 67.05 | 1 | 41669 | BuCaMH03.raw | 1.057E7 | 2 | 2 | 206 | 223 |  |  | PEAKS DB |
| Q.SGGPNAPWATVTPAESR.R | N | 95.90 | 1696.8169 | 17 | -0.7 | 849.4152 | 2 | 46.64 | 1 | 24656 | BuCaMH03.raw | 4.0143E6 | 1 | 1 | 257 | 273 |  |  | PEAKS DB |
| R.MSVPHANDIATEAVVLQYTDWQDQDNR.E | Y | 94.52 | 3115.4304 | 27 | 0.3 | 1039.4844 | 3 | 72.50 | 1 | 46334 | BuCaMH03.raw | 1.2758E8 | 2 | 2 | 389 | 415 |  |  | PEAKS DB |
| R.EALDDIVGDHNVIC(+57.02)PVVQFANDYAK.R | N | 93.85 | 2801.3330 | 25 | -0.2 | 934.7847 | 3 | 78.96 | 1 | 51733 | BuCaMH03.raw | 2.9516E8 | 2 | 2 | 420 | 444 | Carbamidomethylation | C14:Carbamidomethylation:1000.00 | PEAKS DB |
| R.VGAFGFLGLPGSPEAPGNMGLLDQR.L | N | 92.98 | 2499.2581 | 25 | 1.1 | 834.0942 | 3 | 90.41 | 1 | 60987 | BuCaMH03.raw | 3.7724E8 | 2 | 2 | 181 | 205 |  |  | PEAKS DB |
| R.GLSLPVLDGHVTAFLGIPFAEPPVGR.M | N | 86.95 | 2658.4534 | 26 | 1.3 | 887.1595 | 3 | 96.29 | 1 | 65362 | BuCaMH03.raw | 2.0859E9 | 4 | 4 | 48 | 73 |  |  | PEAKS DB |
| A.LQWIQNNIHPFGGNPR.A | N | 86.76 | 1889.9648 | 16 | 0.8 | 945.9904 | 2 | 52.84 | 1 | 29897 | BuCaMH03.raw | 2.3373E7 | 2 | 2 | 208 | 223 |  |  | PEAKS DB |
| L.ALQWIQNNIHPFGGNPR.A | N | 84.31 | 1961.0020 | 17 | 0.2 | 981.5084 | 2 | 56.08 | 1 | 32631 | BuCaMH03.raw | 7.657E7 | 2 | 2 | 207 | 223 |  |  | PEAKS DB |
| K.SGAWPTYTASQPQYVQLNTQPLATQPSLR.A | N | 79.74 | 3202.6047 | 29 | 0.8 | 1068.5431 | 3 | 70.50 | 1 | 44551 | BuCaMH03.raw | 4.9159E7 | 1 | 1 | 520 | 548 |  |  | PEAKS DB |
| K.SIFRFPFVPVIDGDFFPDTPEAMLSSGNFK.E | N | 79.19 | 3376.6477 | 30 | -0.6 | 1126.5558 | 3 | 98.25 | 1 | 66983 | BuCaMH03.raw | 1.9571E6 | 1 | 1 | 317 | 346 |  |  | PEAKS DB |
| R.FPFVPVIDGDFFPDTPEAM(+15.99)LSSGNFK.E | N | 79.18 | 2889.3569 | 26 | 0.0 | 964.1262 | 3 | 98.20 | 1 | 66887 | BuCaMH03.raw | 1.6186E7 | 1 | 1 | 321 | 346 | Oxidation (M) | M19:Oxidation (M):1000.00 | PEAKS DB |
| K.NREALDDIVGDHNVIC(+57.02)PVVQFANDYAK.R | N | 77.79 | 3071.4771 | 27 | 0.4 | 768.8768 | 4 | 70.23 | 1 | 44298 | BuCaMH03.raw | 9.8218E6 | 2 | 2 | 418 | 444 | Carbamidomethylation | C16:Carbamidomethylation:1000.00 | PEAKS DB |
| K.ETQVLLGVVKDEGSYFLIYGLPGFSK.D | N | 77.49 | 2858.5105 | 26 | 0.5 | 953.8446 | 3 | 94.22 | 1 | 63592 | BuCaMH03.raw | 1.1881E7 | 1 | 1 | 347 | 372 |  |  | PEAKS DB |
| K.VYAYLFDHR.A | N | 76.38 | 1182.5822 | 9 | 0.3 | 592.2985 | 2 | 41.43 | 1 | 20432 | BuCaMH03.raw | 2.3244E7 | 2 | 2 | 449 | 457 |  |  | PEAKS DB |
| R.AQIC(+57.02)AFWNHFLPK.L | N | 76.29 | 1630.8079 | 13 | 0.1 | 544.6099 | 3 | 67.38 | 1 | 41864 | BuCaMH03.raw | 6.214E6 | 1 | 1 | 549 | 561 | Carbamidomethylation | C4:Carbamidomethylation:1000.00 | PEAKS DB |
| A.VTIFGESAGAASVGMHLLSTQSR.T | N | 75.90 | 2318.1689 | 23 | 1.1 | 773.7311 | 3 | 69.24 | 1 | 43488 | BuCaMH03.raw | 1.4432E6 | 1 | 1 | 225 | 247 |  |  | PEAKS DB |
| I.C(+57.02)AFWNHFLPK.L | N | 74.57 | 1318.6281 | 10 | -0.5 | 660.3210 | 2 | 56.23 | 1 | 32768 | BuCaMH03.raw | 7.6723E6 | 1 | 1 | 552 | 561 | Carbamidomethylation | C1:Carbamidomethylation:1000.00 | PEAKS DB |
| Q.WIQNNIHPFGGNPR.A | N | 73.92 | 1648.8223 | 14 | 0.4 | 825.4187 | 2 | 39.98 | 1 | 19293 | BuCaMH03.raw | 1.286E7 | 2 | 2 | 210 | 223 |  |  | PEAKS DB |
| P.FVPVIDGDFFPDTPEAMLSSGNFK.E | N | 73.61 | 2629.2410 | 24 | 0.1 | 1315.6279 | 2 | 94.45 | 1 | 64055 | BuCaMH03.raw | 2.0905E7 | 1 | 1 | 323 | 346 |  |  | PEAKS DB |
| V.GAFGFLGLPGSPEAPGNMGLLDQR.L | N | 72.34 | 2400.1895 | 24 | 0.4 | 1201.1025 | 2 | 88.38 | 1 | 58926 | BuCaMH03.raw | 3.179E7 | 2 | 2 | 182 | 205 |  |  | PEAKS DB |
| H.ANDIATEAVVLQYTDWQDQDNR.E | Y | 72.06 | 2564.1780 | 22 | 1.6 | 855.7346 | 3 | 75.14 | 1 | 48499 | BuCaMH03.raw | 5.1842E5 | 1 | 1 | 394 | 415 |  |  | PEAKS DB |
| G.LPGSPEAPGNMGLLDQR.L | N | 70.60 | 1750.8672 | 17 | 0.7 | 876.4415 | 2 | 54.97 | 1 | 31655 | BuCaMH03.raw | 4.4619E6 | 1 | 1 | 189 | 205 |  |  | PEAKS DB |
| R.FLRPEPVKPWPHVLDATSYK.P | N | 69.65 | 2379.2739 | 20 | 0.8 | 595.8262 | 4 | 48.92 | 1 | 26568 | BuCaMH03.raw | 6.2045E6 | 1 | 1 | 76 | 95 |  |  | PEAKS DB |
| Q.IC(+57.02)AFWNHFLPK.L | N | 69.60 | 1431.7122 | 11 | 0.3 | 478.2448 | 3 | 63.50 | 1 | 38737 | BuCaMH03.raw | 2.159E6 | 1 | 1 | 551 | 561 | Carbamidomethylation | C2:Carbamidomethylation:1000.00 | PEAKS DB |
| A.QIC(+57.02)AFWNHFLPK.L | N | 68.21 | 1559.7708 | 12 | 0.3 | 520.9310 | 3 | 65.09 | 1 | 40072 | BuCaMH03.raw | 1.1741E7 | 2 | 2 | 550 | 561 | Carbamidomethylation | C3:Carbamidomethylation:1000.00 | PEAKS DB |
| K.ETQVLLGVVK.D | N | 66.91 | 1084.6492 | 10 | -0.4 | 543.3317 | 2 | 48.73 | 1 | 26723 | BuCaMH03.raw | 1.4354E8 | 1 | 1 | 347 | 356 |  |  | PEAKS DB |
| A.ILQSGGPNAPWATVTPAESR.R | N | 64.65 | 2051.0435 | 20 | 0.5 | 1026.5295 | 2 | 55.18 | 1 | 31833 | BuCaMH03.raw | 2.893E6 | 1 | 1 | 254 | 273 |  |  | PEAKS DB |
| L.SLPVLDGHVTAFLGIPFAEPPVGR.M | N | 64.20 | 2488.3477 | 24 | 1.4 | 830.4576 | 3 | 89.15 | 1 | 59573 | BuCaMH03.raw | 2.1629E7 | 1 | 1 | 50 | 73 |  |  | PEAKS DB |
| R.AVTIFGESAGAASVGMHLLSTQSRTLFQR.A | N | 62.77 | 3034.5657 | 29 | 0.9 | 759.6494 | 4 | 77.95 | 1 | 50683 | BuCaMH03.raw | 8.7118E5 | 1 | 1 | 224 | 252 |  |  | PEAKS DB |
| G.LSLPVLDGHVTAFLGIPFAEPPVGR.M | N | 61.54 | 2601.4319 | 25 | 0.0 | 868.1512 | 3 | 94.83 | 1 | 64548 | BuCaMH03.raw | 9.5169E6 | 2 | 2 | 49 | 73 |  |  | PEAKS DB |
| M.SVPHANDIATEAVVLQYTDWQDQDNR.E | Y | 57.10 | 2984.3899 | 26 | 0.2 | 995.8041 | 3 | 68.45 | 1 | 42800 | BuCaMH03.raw | 9.6717E6 | 1 | 1 | 390 | 415 |  |  | PEAKS DB |
| F.LTYTQNVILVSLSYR.V | N | 56.26 | 1768.9723 | 15 | 0.1 | 885.4935 | 2 | 75.21 | 1 | 48520 | BuCaMH03.raw | 1.4443E6 | 1 | 1 | 166 | 180 |  |  | PEAKS DB |
| P.QELIDEEWSVLPYK.S | N | 56.12 | 1747.8668 | 14 | 0.9 | 874.9415 | 2 | 73.88 | 1 | 47373 | BuCaMH03.raw | 3.1518E6 | 1 | 1 | 303 | 316 |  |  | PEAKS DB |
| L.QWIQNNIHPFGGNPR.A | N | 55.84 | 1776.8809 | 15 | -0.4 | 593.3007 | 3 | 43.44 | 1 | 22097 | BuCaMH03.raw | 2.5227E7 | 1 | 1 | 209 | 223 |  |  | PEAKS DB |
| R.AVTIFGESAGAASVGM(+15.99)HLLSTQSR.T | N | 55.79 | 2405.2009 | 24 | 0.0 | 802.7409 | 3 | 70.98 | 1 | 44980 | BuCaMH03.raw | 5.9259E6 | 1 | 1 | 224 | 247 | Oxidation (M) | M16:Oxidation (M):1000.00 | PEAKS DB |
| R.ADFLEGVR.M | N | 55.33 | 905.4606 | 8 | 0.5 | 453.7378 | 2 | 47.32 | 1 | 25289 | BuCaMH03.raw | 6.9015E7 | 2 | 2 | 381 | 388 |  |  | PEAKS DB |
| E.GSYFLIYGLPGFSK.D | N | 55.01 | 1547.8024 | 14 | 0.7 | 774.9090 | 2 | 85.55 | 1 | 56608 | BuCaMH03.raw | 1.0538E6 | 1 | 1 | 359 | 372 |  |  | PEAKS DB |
| D.GDFFPDTPEAMLSSGNFK.E | N | 54.97 | 1958.8719 | 18 | 0.7 | 980.4440 | 2 | 77.20 | 1 | 50118 | BuCaMH03.raw | 9.7457E5 | 1 | 1 | 329 | 346 |  |  | PEAKS DB |
| R.FPFVPVIDGDFFPDTPEAM.L | N | 54.90 | 2139.9863 | 19 | 0.5 | 1071.0010 | 2 | 98.34 | 1 | 67085 | BuCaMH03.raw | 5.9713E6 | 1 | 1 | 321 | 339 |  |  | PEAKS DB |
| R.FLRPEPVKPWPH.V | N | 54.64 | 1501.8193 | 12 | 0.7 | 501.6141 | 3 | 36.30 | 1 | 16415 | BuCaMH03.raw | 7.868E6 | 1 | 1 | 76 | 87 |  |  | PEAKS DB |
| N.PQELIDEEWSVLPYK.S | N | 54.23 | 1844.9196 | 15 | 1.3 | 923.4683 | 2 | 74.37 | 1 | 47795 | BuCaMH03.raw | 4.9513E5 | 1 | 1 | 302 | 316 |  |  | PEAKS DB |
| K.QLGC(+57.02)HFNNDSELVSC(+57.02)LR.S | N | 54.19 | 2047.9204 | 17 | 0.2 | 683.6476 | 3 | 45.75 | 1 | 23845 | BuCaMH03.raw | 8.5586E6 | 1 | 1 | 282 | 298 | Carbamidomethylation | C4:Carbamidomethylation:1000.00;C15:Carbamidomethylation:1000.00 | PEAKS DB |
| F.GFLGLPGSPEAPGNMGLLDQR.L | N | 54.11 | 2125.0625 | 21 | 0.3 | 1063.5388 | 2 | 79.70 | 1 | 52045 | BuCaMH03.raw | 1.5382E6 | 1 | 1 | 185 | 205 |  |  | PEAKS DB |
| R.LALQWIQNNIHPF.G | N | 53.30 | 1592.8463 | 13 | 0.4 | 797.4308 | 2 | 79.77 | 1 | 52223 | BuCaMH03.raw | 3.4534E5 | 1 | 1 | 206 | 218 |  |  | PEAKS DB |
| total 51 peptides |  |  |  |  |  |  |  |  |  |  |  |  |  |  |  |  |  |  |  |
| --- | --- | --- | --- | --- | --- | --- | --- | --- | --- | --- | --- | --- | --- | --- | --- | --- | --- | --- | --- |

T\_171

back to list

  

| Protein Coverage
| Supporting Peptides
|

Protein Coverage:

Supporting Peptides:

| Peptide | Uniq | -10lgP | Mass | Length | ppm | m/z | z | RT | Fraction | Scan | Source File | Area BuCaMH03\_B17 | #Feature | #Feature BuCaMH03\_B17 | Start | End | PTM | AScore | Found By |
| --- | --- | --- | --- | --- | --- | --- | --- | --- | --- | --- | --- | --- | --- | --- | --- | --- | --- | --- | --- |
| R.AVTIFGESAGAASVGMHLLSTQSR.T | N | 110.40 | 2389.2061 | 24 | 0.0 | 797.4093 | 3 | 70.91 | 1 | 45278 | BuCaMH03.raw | 2.6603E8 | 4 | 4 | 224 | 247 |  |  | PEAKS DB |
| R.FLTYTQNVILVSLSYR.V | N | 110.00 | 1916.0408 | 16 | 0.4 | 959.0281 | 2 | 83.45 | 1 | 55322 | BuCaMH03.raw | 3.0247E7 | 3 | 3 | 165 | 180 |  |  | PEAKS DB |
| R.FPFVPVIDGDFFPDTPEAMLSSGNFK.E | N | 109.19 | 2873.3621 | 26 | 0.6 | 1437.6892 | 2 | 98.27 | 1 | 67043 | BuCaMH03.raw | 4.4491E8 | 2 | 2 | 321 | 346 |  |  | PEAKS DB |
| K.DEGSYFLIYGLPGFSK.D | N | 106.68 | 1791.8718 | 16 | 0.8 | 896.9439 | 2 | 93.19 | 1 | 62858 | BuCaMH03.raw | 2.4463E8 | 2 | 2 | 357 | 372 |  |  | PEAKS DB |
| R.AILQSGGPNAPWATVTPAESR.R | N | 105.98 | 2122.0806 | 21 | 0.3 | 1062.0479 | 2 | 59.69 | 1 | 35420 | BuCaMH03.raw | 9.8415E7 | 2 | 2 | 253 | 273 |  |  | PEAKS DB |
| K.NPQELIDEEWSVLPYK.S | N | 102.10 | 1958.9625 | 16 | 0.4 | 980.4890 | 2 | 78.54 | 1 | 51369 | BuCaMH03.raw | 6.749E7 | 2 | 2 | 301 | 316 |  |  | PEAKS DB |
| R.SKNPQELIDEEWSVLPYK.S | N | 100.91 | 2174.0896 | 18 | 2.3 | 725.7054 | 3 | 68.47 | 1 | 43161 | BuCaMH03.raw | 1.243E8 | 2 | 2 | 299 | 316 |  |  | PEAKS DB |
| R.LALQWIQNNIHPFGGNPR.A | N | 96.68 | 2074.0859 | 18 | 0.3 | 692.3694 | 3 | 67.05 | 1 | 41669 | BuCaMH03.raw | 1.057E7 | 2 | 2 | 206 | 223 |  |  | PEAKS DB |
| Q.SGGPNAPWATVTPAESR.R | N | 95.90 | 1696.8169 | 17 | -0.7 | 849.4152 | 2 | 46.64 | 1 | 24656 | BuCaMH03.raw | 4.0143E6 | 1 | 1 | 257 | 273 |  |  | PEAKS DB |
| R.MSVPHANDIATEAVVLQYTDWQDQDNR.E | Y | 94.52 | 3115.4304 | 27 | 0.3 | 1039.4844 | 3 | 72.50 | 1 | 46334 | BuCaMH03.raw | 1.2758E8 | 2 | 2 | 389 | 415 |  |  | PEAKS DB |
| R.EALDDIVGDHNVIC(+57.02)PVVQFANDYAK.R | N | 93.85 | 2801.3330 | 25 | -0.2 | 934.7847 | 3 | 78.96 | 1 | 51733 | BuCaMH03.raw | 2.9516E8 | 2 | 2 | 420 | 444 | Carbamidomethylation | C14:Carbamidomethylation:1000.00 | PEAKS DB |
| R.VGAFGFLGLPGSPEAPGNMGLLDQR.L | N | 92.98 | 2499.2581 | 25 | 1.1 | 834.0942 | 3 | 90.41 | 1 | 60987 | BuCaMH03.raw | 3.7724E8 | 2 | 2 | 181 | 205 |  |  | PEAKS DB |
| R.GLSLPVLDGHVTAFLGIPFAEPPVGR.M | N | 86.95 | 2658.4534 | 26 | 1.3 | 887.1595 | 3 | 96.29 | 1 | 65362 | BuCaMH03.raw | 2.0859E9 | 4 | 4 | 48 | 73 |  |  | PEAKS DB |
| A.LQWIQNNIHPFGGNPR.A | N | 86.76 | 1889.9648 | 16 | 0.8 | 945.9904 | 2 | 52.84 | 1 | 29897 | BuCaMH03.raw | 2.3373E7 | 2 | 2 | 208 | 223 |  |  | PEAKS DB |
| L.ALQWIQNNIHPFGGNPR.A | N | 84.31 | 1961.0020 | 17 | 0.2 | 981.5084 | 2 | 56.08 | 1 | 32631 | BuCaMH03.raw | 7.657E7 | 2 | 2 | 207 | 223 |  |  | PEAKS DB |
| K.SGAWPTYTASQPQYVQLNTQPLATQPSLR.A | N | 79.74 | 3202.6047 | 29 | 0.8 | 1068.5431 | 3 | 70.50 | 1 | 44551 | BuCaMH03.raw | 4.9159E7 | 1 | 1 | 520 | 548 |  |  | PEAKS DB |
| K.SIFRFPFVPVIDGDFFPDTPEAMLSSGNFK.E | N | 79.19 | 3376.6477 | 30 | -0.6 | 1126.5558 | 3 | 98.25 | 1 | 66983 | BuCaMH03.raw | 1.9571E6 | 1 | 1 | 317 | 346 |  |  | PEAKS DB |
| R.FPFVPVIDGDFFPDTPEAM(+15.99)LSSGNFK.E | N | 79.18 | 2889.3569 | 26 | 0.0 | 964.1262 | 3 | 98.20 | 1 | 66887 | BuCaMH03.raw | 1.6186E7 | 1 | 1 | 321 | 346 | Oxidation (M) | M19:Oxidation (M):1000.00 | PEAKS DB |
| K.NREALDDIVGDHNVIC(+57.02)PVVQFANDYAK.R | N | 77.79 | 3071.4771 | 27 | 0.4 | 768.8768 | 4 | 70.23 | 1 | 44298 | BuCaMH03.raw | 9.8218E6 | 2 | 2 | 418 | 444 | Carbamidomethylation | C16:Carbamidomethylation:1000.00 | PEAKS DB |
| K.ETQVLLGVVKDEGSYFLIYGLPGFSK.D | N | 77.49 | 2858.5105 | 26 | 0.5 | 953.8446 | 3 | 94.22 | 1 | 63592 | BuCaMH03.raw | 1.1881E7 | 1 | 1 | 347 | 372 |  |  | PEAKS DB |
| K.VYAYLFDHR.A | N | 76.38 | 1182.5822 | 9 | 0.3 | 592.2985 | 2 | 41.43 | 1 | 20432 | BuCaMH03.raw | 2.3244E7 | 2 | 2 | 449 | 457 |  |  | PEAKS DB |
| R.AQIC(+57.02)AFWNHFLPK.L | N | 76.29 | 1630.8079 | 13 | 0.1 | 544.6099 | 3 | 67.38 | 1 | 41864 | BuCaMH03.raw | 6.214E6 | 1 | 1 | 549 | 561 | Carbamidomethylation | C4:Carbamidomethylation:1000.00 | PEAKS DB |
| A.VTIFGESAGAASVGMHLLSTQSR.T | N | 75.90 | 2318.1689 | 23 | 1.1 | 773.7311 | 3 | 69.24 | 1 | 43488 | BuCaMH03.raw | 1.4432E6 | 1 | 1 | 225 | 247 |  |  | PEAKS DB |
| I.C(+57.02)AFWNHFLPK.L | N | 74.57 | 1318.6281 | 10 | -0.5 | 660.3210 | 2 | 56.23 | 1 | 32768 | BuCaMH03.raw | 7.6723E6 | 1 | 1 | 552 | 561 | Carbamidomethylation | C1:Carbamidomethylation:1000.00 | PEAKS DB |
| Q.WIQNNIHPFGGNPR.A | N | 73.92 | 1648.8223 | 14 | 0.4 | 825.4187 | 2 | 39.98 | 1 | 19293 | BuCaMH03.raw | 1.286E7 | 2 | 2 | 210 | 223 |  |  | PEAKS DB |
| P.FVPVIDGDFFPDTPEAMLSSGNFK.E | N | 73.61 | 2629.2410 | 24 | 0.1 | 1315.6279 | 2 | 94.45 | 1 | 64055 | BuCaMH03.raw | 2.0905E7 | 1 | 1 | 323 | 346 |  |  | PEAKS DB |
| V.GAFGFLGLPGSPEAPGNMGLLDQR.L | N | 72.34 | 2400.1895 | 24 | 0.4 | 1201.1025 | 2 | 88.38 | 1 | 58926 | BuCaMH03.raw | 3.179E7 | 2 | 2 | 182 | 205 |  |  | PEAKS DB |
| H.ANDIATEAVVLQYTDWQDQDNR.E | Y | 72.06 | 2564.1780 | 22 | 1.6 | 855.7346 | 3 | 75.14 | 1 | 48499 | BuCaMH03.raw | 5.1842E5 | 1 | 1 | 394 | 415 |  |  | PEAKS DB |
| G.LPGSPEAPGNMGLLDQR.L | N | 70.60 | 1750.8672 | 17 | 0.7 | 876.4415 | 2 | 54.97 | 1 | 31655 | BuCaMH03.raw | 4.4619E6 | 1 | 1 | 189 | 205 |  |  | PEAKS DB |
| R.FLRPEPVKPWPHVLDATSYK.P | N | 69.65 | 2379.2739 | 20 | 0.8 | 595.8262 | 4 | 48.92 | 1 | 26568 | BuCaMH03.raw | 6.2045E6 | 1 | 1 | 76 | 95 |  |  | PEAKS DB |
| Q.IC(+57.02)AFWNHFLPK.L | N | 69.60 | 1431.7122 | 11 | 0.3 | 478.2448 | 3 | 63.50 | 1 | 38737 | BuCaMH03.raw | 2.159E6 | 1 | 1 | 551 | 561 | Carbamidomethylation | C2:Carbamidomethylation:1000.00 | PEAKS DB |
| A.QIC(+57.02)AFWNHFLPK.L | N | 68.21 | 1559.7708 | 12 | 0.3 | 520.9310 | 3 | 65.09 | 1 | 40072 | BuCaMH03.raw | 1.1741E7 | 2 | 2 | 550 | 561 | Carbamidomethylation | C3:Carbamidomethylation:1000.00 | PEAKS DB |
| K.ETQVLLGVVK.D | N | 66.91 | 1084.6492 | 10 | -0.4 | 543.3317 | 2 | 48.73 | 1 | 26723 | BuCaMH03.raw | 1.4354E8 | 1 | 1 | 347 | 356 |  |  | PEAKS DB |
| A.ILQSGGPNAPWATVTPAESR.R | N | 64.65 | 2051.0435 | 20 | 0.5 | 1026.5295 | 2 | 55.18 | 1 | 31833 | BuCaMH03.raw | 2.893E6 | 1 | 1 | 254 | 273 |  |  | PEAKS DB |
| L.SLPVLDGHVTAFLGIPFAEPPVGR.M | N | 64.20 | 2488.3477 | 24 | 1.4 | 830.4576 | 3 | 89.15 | 1 | 59573 | BuCaMH03.raw | 2.1629E7 | 1 | 1 | 50 | 73 |  |  | PEAKS DB |
| R.AVTIFGESAGAASVGMHLLSTQSRTLFQR.A | N | 62.77 | 3034.5657 | 29 | 0.9 | 759.6494 | 4 | 77.95 | 1 | 50683 | BuCaMH03.raw | 8.7118E5 | 1 | 1 | 224 | 252 |  |  | PEAKS DB |
| G.LSLPVLDGHVTAFLGIPFAEPPVGR.M | N | 61.54 | 2601.4319 | 25 | 0.0 | 868.1512 | 3 | 94.83 | 1 | 64548 | BuCaMH03.raw | 9.5169E6 | 2 | 2 | 49 | 73 |  |  | PEAKS DB |
| M.SVPHANDIATEAVVLQYTDWQDQDNR.E | Y | 57.10 | 2984.3899 | 26 | 0.2 | 995.8041 | 3 | 68.45 | 1 | 42800 | BuCaMH03.raw | 9.6717E6 | 1 | 1 | 390 | 415 |  |  | PEAKS DB |
| F.LTYTQNVILVSLSYR.V | N | 56.26 | 1768.9723 | 15 | 0.1 | 885.4935 | 2 | 75.21 | 1 | 48520 | BuCaMH03.raw | 1.4443E6 | 1 | 1 | 166 | 180 |  |  | PEAKS DB |
| P.QELIDEEWSVLPYK.S | N | 56.12 | 1747.8668 | 14 | 0.9 | 874.9415 | 2 | 73.88 | 1 | 47373 | BuCaMH03.raw | 3.1518E6 | 1 | 1 | 303 | 316 |  |  | PEAKS DB |
| L.QWIQNNIHPFGGNPR.A | N | 55.84 | 1776.8809 | 15 | -0.4 | 593.3007 | 3 | 43.44 | 1 | 22097 | BuCaMH03.raw | 2.5227E7 | 1 | 1 | 209 | 223 |  |  | PEAKS DB |
| R.AVTIFGESAGAASVGM(+15.99)HLLSTQSR.T | N | 55.79 | 2405.2009 | 24 | 0.0 | 802.7409 | 3 | 70.98 | 1 | 44980 | BuCaMH03.raw | 5.9259E6 | 1 | 1 | 224 | 247 | Oxidation (M) | M16:Oxidation (M):1000.00 | PEAKS DB |
| R.ADFLEGVR.M | N | 55.33 | 905.4606 | 8 | 0.5 | 453.7378 | 2 | 47.32 | 1 | 25289 | BuCaMH03.raw | 6.9015E7 | 2 | 2 | 381 | 388 |  |  | PEAKS DB |
| E.GSYFLIYGLPGFSK.D | N | 55.01 | 1547.8024 | 14 | 0.7 | 774.9090 | 2 | 85.55 | 1 | 56608 | BuCaMH03.raw | 1.0538E6 | 1 | 1 | 359 | 372 |  |  | PEAKS DB |
| D.GDFFPDTPEAMLSSGNFK.E | N | 54.97 | 1958.8719 | 18 | 0.7 | 980.4440 | 2 | 77.20 | 1 | 50118 | BuCaMH03.raw | 9.7457E5 | 1 | 1 | 329 | 346 |  |  | PEAKS DB |
| R.FPFVPVIDGDFFPDTPEAM.L | N | 54.90 | 2139.9863 | 19 | 0.5 | 1071.0010 | 2 | 98.34 | 1 | 67085 | BuCaMH03.raw | 5.9713E6 | 1 | 1 | 321 | 339 |  |  | PEAKS DB |
| R.FLRPEPVKPWPH.V | N | 54.64 | 1501.8193 | 12 | 0.7 | 501.6141 | 3 | 36.30 | 1 | 16415 | BuCaMH03.raw | 7.868E6 | 1 | 1 | 76 | 87 |  |  | PEAKS DB |
| N.PQELIDEEWSVLPYK.S | N | 54.23 | 1844.9196 | 15 | 1.3 | 923.4683 | 2 | 74.37 | 1 | 47795 | BuCaMH03.raw | 4.9513E5 | 1 | 1 | 302 | 316 |  |  | PEAKS DB |
| K.QLGC(+57.02)HFNNDSELVSC(+57.02)LR.S | N | 54.19 | 2047.9204 | 17 | 0.2 | 683.6476 | 3 | 45.75 | 1 | 23845 | BuCaMH03.raw | 8.5586E6 | 1 | 1 | 282 | 298 | Carbamidomethylation | C4:Carbamidomethylation:1000.00;C15:Carbamidomethylation:1000.00 | PEAKS DB |
| F.GFLGLPGSPEAPGNMGLLDQR.L | N | 54.11 | 2125.0625 | 21 | 0.3 | 1063.5388 | 2 | 79.70 | 1 | 52045 | BuCaMH03.raw | 1.5382E6 | 1 | 1 | 185 | 205 |  |  | PEAKS DB |
| R.LALQWIQNNIHPF.G | N | 53.30 | 1592.8463 | 13 | 0.4 | 797.4308 | 2 | 79.77 | 1 | 52223 | BuCaMH03.raw | 3.4534E5 | 1 | 1 | 206 | 218 |  |  | PEAKS DB |
| total 51 peptides |  |  |  |  |  |  |  |  |  |  |  |  |  |  |  |  |  |  |  |
| --- | --- | --- | --- | --- | --- | --- | --- | --- | --- | --- | --- | --- | --- | --- | --- | --- | --- | --- | --- |

T\_166

back to list

  

| Protein Coverage
| Supporting Peptides
|

Protein Coverage:

Supporting Peptides:

| Peptide | Uniq | -10lgP | Mass | Length | ppm | m/z | z | RT | Fraction | Scan | Source File | Area BuCaMH03\_B17 | #Feature | #Feature BuCaMH03\_B17 | Start | End | PTM | AScore | Found By |
| --- | --- | --- | --- | --- | --- | --- | --- | --- | --- | --- | --- | --- | --- | --- | --- | --- | --- | --- | --- |
| R.AVTIFGESAGAASVGMHLLSTQSR.T | N | 110.40 | 2389.2061 | 24 | 0.0 | 797.4093 | 3 | 70.91 | 1 | 45278 | BuCaMH03.raw | 2.6603E8 | 4 | 4 | 216 | 239 |  |  | PEAKS DB |
| R.FLTYTQNVILVSLSYR.V | N | 110.00 | 1916.0408 | 16 | 0.4 | 959.0281 | 2 | 83.45 | 1 | 55322 | BuCaMH03.raw | 3.0247E7 | 3 | 3 | 157 | 172 |  |  | PEAKS DB |
| R.FPFVPVIDGDFFPDTPEAMLSSGNFK.E | N | 109.19 | 2873.3621 | 26 | 0.6 | 1437.6892 | 2 | 98.27 | 1 | 67043 | BuCaMH03.raw | 4.4491E8 | 2 | 2 | 313 | 338 |  |  | PEAKS DB |
| K.DEGSYFLIYGLPGFSK.D | N | 106.68 | 1791.8718 | 16 | 0.8 | 896.9439 | 2 | 93.19 | 1 | 62858 | BuCaMH03.raw | 2.4463E8 | 2 | 2 | 349 | 364 |  |  | PEAKS DB |
| R.AILQSGGPNAPWATVTPAESR.R | N | 105.98 | 2122.0806 | 21 | 0.3 | 1062.0479 | 2 | 59.69 | 1 | 35420 | BuCaMH03.raw | 9.8415E7 | 2 | 2 | 245 | 265 |  |  | PEAKS DB |
| K.NPQELIDEEWSVLPYK.S | N | 102.10 | 1958.9625 | 16 | 0.4 | 980.4890 | 2 | 78.54 | 1 | 51369 | BuCaMH03.raw | 6.749E7 | 2 | 2 | 293 | 308 |  |  | PEAKS DB |
| R.SKNPQELIDEEWSVLPYK.S | N | 100.91 | 2174.0896 | 18 | 2.3 | 725.7054 | 3 | 68.47 | 1 | 43161 | BuCaMH03.raw | 1.243E8 | 2 | 2 | 291 | 308 |  |  | PEAKS DB |
| R.LALQWIQNNIHPFGGNPR.A | N | 96.68 | 2074.0859 | 18 | 0.3 | 692.3694 | 3 | 67.05 | 1 | 41669 | BuCaMH03.raw | 1.057E7 | 2 | 2 | 198 | 215 |  |  | PEAKS DB |
| Q.SGGPNAPWATVTPAESR.R | N | 95.90 | 1696.8169 | 17 | -0.7 | 849.4152 | 2 | 46.64 | 1 | 24656 | BuCaMH03.raw | 4.0143E6 | 1 | 1 | 249 | 265 |  |  | PEAKS DB |
| R.MSVPHANDIATEAVVLQYTDWQDQDNR.E | Y | 94.52 | 3115.4304 | 27 | 0.3 | 1039.4844 | 3 | 72.50 | 1 | 46334 | BuCaMH03.raw | 1.2758E8 | 2 | 2 | 381 | 407 |  |  | PEAKS DB |
| R.EALDDIVGDHNVIC(+57.02)PVVQFANDYAK.R | N | 93.85 | 2801.3330 | 25 | -0.2 | 934.7847 | 3 | 78.96 | 1 | 51733 | BuCaMH03.raw | 2.9516E8 | 2 | 2 | 412 | 436 | Carbamidomethylation | C14:Carbamidomethylation:1000.00 | PEAKS DB |
| R.VGAFGFLGLPGSPEAPGNMGLLDQR.L | N | 92.98 | 2499.2581 | 25 | 1.1 | 834.0942 | 3 | 90.41 | 1 | 60987 | BuCaMH03.raw | 3.7724E8 | 2 | 2 | 173 | 197 |  |  | PEAKS DB |
| R.GLSLPVLDGHVTAFLGIPFAEPPVGR.M | N | 86.95 | 2658.4534 | 26 | 1.3 | 887.1595 | 3 | 96.29 | 1 | 65362 | BuCaMH03.raw | 2.0859E9 | 4 | 4 | 40 | 65 |  |  | PEAKS DB |
| A.LQWIQNNIHPFGGNPR.A | N | 86.76 | 1889.9648 | 16 | 0.8 | 945.9904 | 2 | 52.84 | 1 | 29897 | BuCaMH03.raw | 2.3373E7 | 2 | 2 | 200 | 215 |  |  | PEAKS DB |
| L.ALQWIQNNIHPFGGNPR.A | N | 84.31 | 1961.0020 | 17 | 0.2 | 981.5084 | 2 | 56.08 | 1 | 32631 | BuCaMH03.raw | 7.657E7 | 2 | 2 | 199 | 215 |  |  | PEAKS DB |
| K.SGAWPTYTASQPQYVQLNTQPLATQPSLR.A | N | 79.74 | 3202.6047 | 29 | 0.8 | 1068.5431 | 3 | 70.50 | 1 | 44551 | BuCaMH03.raw | 4.9159E7 | 1 | 1 | 512 | 540 |  |  | PEAKS DB |
| K.SIFRFPFVPVIDGDFFPDTPEAMLSSGNFK.E | N | 79.19 | 3376.6477 | 30 | -0.6 | 1126.5558 | 3 | 98.25 | 1 | 66983 | BuCaMH03.raw | 1.9571E6 | 1 | 1 | 309 | 338 |  |  | PEAKS DB |
| R.FPFVPVIDGDFFPDTPEAM(+15.99)LSSGNFK.E | N | 79.18 | 2889.3569 | 26 | 0.0 | 964.1262 | 3 | 98.20 | 1 | 66887 | BuCaMH03.raw | 1.6186E7 | 1 | 1 | 313 | 338 | Oxidation (M) | M19:Oxidation (M):1000.00 | PEAKS DB |
| K.NREALDDIVGDHNVIC(+57.02)PVVQFANDYAK.R | N | 77.79 | 3071.4771 | 27 | 0.4 | 768.8768 | 4 | 70.23 | 1 | 44298 | BuCaMH03.raw | 9.8218E6 | 2 | 2 | 410 | 436 | Carbamidomethylation | C16:Carbamidomethylation:1000.00 | PEAKS DB |
| K.ETQVLLGVVKDEGSYFLIYGLPGFSK.D | N | 77.49 | 2858.5105 | 26 | 0.5 | 953.8446 | 3 | 94.22 | 1 | 63592 | BuCaMH03.raw | 1.1881E7 | 1 | 1 | 339 | 364 |  |  | PEAKS DB |
| K.VYAYLFDHR.A | N | 76.38 | 1182.5822 | 9 | 0.3 | 592.2985 | 2 | 41.43 | 1 | 20432 | BuCaMH03.raw | 2.3244E7 | 2 | 2 | 441 | 449 |  |  | PEAKS DB |
| R.AQIC(+57.02)AFWNHFLPK.L | N | 76.29 | 1630.8079 | 13 | 0.1 | 544.6099 | 3 | 67.38 | 1 | 41864 | BuCaMH03.raw | 6.214E6 | 1 | 1 | 541 | 553 | Carbamidomethylation | C4:Carbamidomethylation:1000.00 | PEAKS DB |
| A.VTIFGESAGAASVGMHLLSTQSR.T | N | 75.90 | 2318.1689 | 23 | 1.1 | 773.7311 | 3 | 69.24 | 1 | 43488 | BuCaMH03.raw | 1.4432E6 | 1 | 1 | 217 | 239 |  |  | PEAKS DB |
| I.C(+57.02)AFWNHFLPK.L | N | 74.57 | 1318.6281 | 10 | -0.5 | 660.3210 | 2 | 56.23 | 1 | 32768 | BuCaMH03.raw | 7.6723E6 | 1 | 1 | 544 | 553 | Carbamidomethylation | C1:Carbamidomethylation:1000.00 | PEAKS DB |
| Q.WIQNNIHPFGGNPR.A | N | 73.92 | 1648.8223 | 14 | 0.4 | 825.4187 | 2 | 39.98 | 1 | 19293 | BuCaMH03.raw | 1.286E7 | 2 | 2 | 202 | 215 |  |  | PEAKS DB |
| P.FVPVIDGDFFPDTPEAMLSSGNFK.E | N | 73.61 | 2629.2410 | 24 | 0.1 | 1315.6279 | 2 | 94.45 | 1 | 64055 | BuCaMH03.raw | 2.0905E7 | 1 | 1 | 315 | 338 |  |  | PEAKS DB |
| V.GAFGFLGLPGSPEAPGNMGLLDQR.L | N | 72.34 | 2400.1895 | 24 | 0.4 | 1201.1025 | 2 | 88.38 | 1 | 58926 | BuCaMH03.raw | 3.179E7 | 2 | 2 | 174 | 197 |  |  | PEAKS DB |
| H.ANDIATEAVVLQYTDWQDQDNR.E | Y | 72.06 | 2564.1780 | 22 | 1.6 | 855.7346 | 3 | 75.14 | 1 | 48499 | BuCaMH03.raw | 5.1842E5 | 1 | 1 | 386 | 407 |  |  | PEAKS DB |
| G.LPGSPEAPGNMGLLDQR.L | N | 70.60 | 1750.8672 | 17 | 0.7 | 876.4415 | 2 | 54.97 | 1 | 31655 | BuCaMH03.raw | 4.4619E6 | 1 | 1 | 181 | 197 |  |  | PEAKS DB |
| R.FLRPEPVKPWPHVLDATSYK.P | N | 69.65 | 2379.2739 | 20 | 0.8 | 595.8262 | 4 | 48.92 | 1 | 26568 | BuCaMH03.raw | 6.2045E6 | 1 | 1 | 68 | 87 |  |  | PEAKS DB |
| Q.IC(+57.02)AFWNHFLPK.L | N | 69.60 | 1431.7122 | 11 | 0.3 | 478.2448 | 3 | 63.50 | 1 | 38737 | BuCaMH03.raw | 2.159E6 | 1 | 1 | 543 | 553 | Carbamidomethylation | C2:Carbamidomethylation:1000.00 | PEAKS DB |
| A.QIC(+57.02)AFWNHFLPK.L | N | 68.21 | 1559.7708 | 12 | 0.3 | 520.9310 | 3 | 65.09 | 1 | 40072 | BuCaMH03.raw | 1.1741E7 | 2 | 2 | 542 | 553 | Carbamidomethylation | C3:Carbamidomethylation:1000.00 | PEAKS DB |
| K.ETQVLLGVVK.D | N | 66.91 | 1084.6492 | 10 | -0.4 | 543.3317 | 2 | 48.73 | 1 | 26723 | BuCaMH03.raw | 1.4354E8 | 1 | 1 | 339 | 348 |  |  | PEAKS DB |
| A.ILQSGGPNAPWATVTPAESR.R | N | 64.65 | 2051.0435 | 20 | 0.5 | 1026.5295 | 2 | 55.18 | 1 | 31833 | BuCaMH03.raw | 2.893E6 | 1 | 1 | 246 | 265 |  |  | PEAKS DB |
| L.SLPVLDGHVTAFLGIPFAEPPVGR.M | N | 64.20 | 2488.3477 | 24 | 1.4 | 830.4576 | 3 | 89.15 | 1 | 59573 | BuCaMH03.raw | 2.1629E7 | 1 | 1 | 42 | 65 |  |  | PEAKS DB |
| R.AVTIFGESAGAASVGMHLLSTQSRTLFQR.A | N | 62.77 | 3034.5657 | 29 | 0.9 | 759.6494 | 4 | 77.95 | 1 | 50683 | BuCaMH03.raw | 8.7118E5 | 1 | 1 | 216 | 244 |  |  | PEAKS DB |
| G.LSLPVLDGHVTAFLGIPFAEPPVGR.M | N | 61.54 | 2601.4319 | 25 | 0.0 | 868.1512 | 3 | 94.83 | 1 | 64548 | BuCaMH03.raw | 9.5169E6 | 2 | 2 | 41 | 65 |  |  | PEAKS DB |
| M.SVPHANDIATEAVVLQYTDWQDQDNR.E | Y | 57.10 | 2984.3899 | 26 | 0.2 | 995.8041 | 3 | 68.45 | 1 | 42800 | BuCaMH03.raw | 9.6717E6 | 1 | 1 | 382 | 407 |  |  | PEAKS DB |
| F.LTYTQNVILVSLSYR.V | N | 56.26 | 1768.9723 | 15 | 0.1 | 885.4935 | 2 | 75.21 | 1 | 48520 | BuCaMH03.raw | 1.4443E6 | 1 | 1 | 158 | 172 |  |  | PEAKS DB |
| P.QELIDEEWSVLPYK.S | N | 56.12 | 1747.8668 | 14 | 0.9 | 874.9415 | 2 | 73.88 | 1 | 47373 | BuCaMH03.raw | 3.1518E6 | 1 | 1 | 295 | 308 |  |  | PEAKS DB |
| L.QWIQNNIHPFGGNPR.A | N | 55.84 | 1776.8809 | 15 | -0.4 | 593.3007 | 3 | 43.44 | 1 | 22097 | BuCaMH03.raw | 2.5227E7 | 1 | 1 | 201 | 215 |  |  | PEAKS DB |
| R.AVTIFGESAGAASVGM(+15.99)HLLSTQSR.T | N | 55.79 | 2405.2009 | 24 | 0.0 | 802.7409 | 3 | 70.98 | 1 | 44980 | BuCaMH03.raw | 5.9259E6 | 1 | 1 | 216 | 239 | Oxidation (M) | M16:Oxidation (M):1000.00 | PEAKS DB |
| R.ADFLEGVR.M | N | 55.33 | 905.4606 | 8 | 0.5 | 453.7378 | 2 | 47.32 | 1 | 25289 | BuCaMH03.raw | 6.9015E7 | 2 | 2 | 373 | 380 |  |  | PEAKS DB |
| E.GSYFLIYGLPGFSK.D | N | 55.01 | 1547.8024 | 14 | 0.7 | 774.9090 | 2 | 85.55 | 1 | 56608 | BuCaMH03.raw | 1.0538E6 | 1 | 1 | 351 | 364 |  |  | PEAKS DB |
| D.GDFFPDTPEAMLSSGNFK.E | N | 54.97 | 1958.8719 | 18 | 0.7 | 980.4440 | 2 | 77.20 | 1 | 50118 | BuCaMH03.raw | 9.7457E5 | 1 | 1 | 321 | 338 |  |  | PEAKS DB |
| R.FPFVPVIDGDFFPDTPEAM.L | N | 54.90 | 2139.9863 | 19 | 0.5 | 1071.0010 | 2 | 98.34 | 1 | 67085 | BuCaMH03.raw | 5.9713E6 | 1 | 1 | 313 | 331 |  |  | PEAKS DB |
| R.FLRPEPVKPWPH.V | N | 54.64 | 1501.8193 | 12 | 0.7 | 501.6141 | 3 | 36.30 | 1 | 16415 | BuCaMH03.raw | 7.868E6 | 1 | 1 | 68 | 79 |  |  | PEAKS DB |
| N.PQELIDEEWSVLPYK.S | N | 54.23 | 1844.9196 | 15 | 1.3 | 923.4683 | 2 | 74.37 | 1 | 47795 | BuCaMH03.raw | 4.9513E5 | 1 | 1 | 294 | 308 |  |  | PEAKS DB |
| K.QLGC(+57.02)HFNNDSELVSC(+57.02)LR.S | N | 54.19 | 2047.9204 | 17 | 0.2 | 683.6476 | 3 | 45.75 | 1 | 23845 | BuCaMH03.raw | 8.5586E6 | 1 | 1 | 274 | 290 | Carbamidomethylation | C4:Carbamidomethylation:1000.00;C15:Carbamidomethylation:1000.00 | PEAKS DB |
| F.GFLGLPGSPEAPGNMGLLDQR.L | N | 54.11 | 2125.0625 | 21 | 0.3 | 1063.5388 | 2 | 79.70 | 1 | 52045 | BuCaMH03.raw | 1.5382E6 | 1 | 1 | 177 | 197 |  |  | PEAKS DB |
| R.LALQWIQNNIHPF.G | N | 53.30 | 1592.8463 | 13 | 0.4 | 797.4308 | 2 | 79.77 | 1 | 52223 | BuCaMH03.raw | 3.4534E5 | 1 | 1 | 198 | 210 |  |  | PEAKS DB |
| total 51 peptides |  |  |  |  |  |  |  |  |  |  |  |  |  |  |  |  |  |  |  |
| --- | --- | --- | --- | --- | --- | --- | --- | --- | --- | --- | --- | --- | --- | --- | --- | --- | --- | --- | --- |

T\_48

back to list

  

| Protein Coverage
| Supporting Peptides
|

Protein Coverage:

Supporting Peptides:

| Peptide | Uniq | -10lgP | Mass | Length | ppm | m/z | z | RT | Fraction | Scan | Source File | Area BuCaMH03\_B17 | #Feature | #Feature BuCaMH03\_B17 | Start | End | PTM | AScore | Found By |
| --- | --- | --- | --- | --- | --- | --- | --- | --- | --- | --- | --- | --- | --- | --- | --- | --- | --- | --- | --- |
| K.SGPPC(+57.02)GDC(+57.02)PSAC(+57.02)VNGLC(+57.02)TNPC(+57.02)K.Y | N | 121.75 | 2406.9482 | 22 | -0.5 | 1204.4808 | 2 | 38.36 | 1 | 18143 | BuCaMH03.raw | 4.9343E8 | 3 | 3 | 178 | 199 | Carbamidomethylation | C5:Carbamidomethylation:1000.00;C8:Carbamidomethylation:1000.00;C12:Carbamidomethylation:1000.00;C17:Carbamidomethylation:1000.00;C21:Carbamidomethylation:1000.00 | PEAKS DB |
| K.FSC(+57.02)GENLFMSSQPYAWSK.V | N | 111.14 | 2137.9238 | 18 | 0.0 | 1069.9692 | 2 | 69.16 | 1 | 43364 | BuCaMH03.raw | 4.5041E8 | 3 | 3 | 88 | 105 | Carbamidomethylation | C3:Carbamidomethylation:1000.00 | PEAKS DB |
| K.NFVYGVGANPPDSVIGHYTQIVWYK.S | Y | 106.01 | 2823.4021 | 25 | 0.4 | 942.1417 | 3 | 76.37 | 1 | 49615 | BuCaMH03.raw | 2.5384E9 | 3 | 3 | 116 | 140 |  |  | PEAKS DB |
| R.NMLQMEWNSNAAQNAK.R | N | 104.88 | 1848.8247 | 16 | 0.4 | 925.4200 | 2 | 52.32 | 1 | 29479 | BuCaMH03.raw | 2.3872E9 | 6 | 6 | 52 | 67 |  |  | PEAKS DB |
| R.NMLQM(+15.99)EWNSNAAQNAK.R | N | 97.35 | 1864.8196 | 16 | -0.5 | 933.4166 | 2 | 41.32 | 1 | 20903 | BuCaMH03.raw | 2.4898E8 | 4 | 4 | 52 | 67 | Oxidation (M) | M5:Oxidation (M):91.37 | PEAKS DB |
| K.NFVYGVGANPPDSVIGHYTQIVWYKSQLLGC(+57.02)AVTR.C | Y | 94.72 | 3908.9670 | 35 | 2.7 | 978.2516 | 4 | 82.15 | 1 | 54324 | BuCaMH03.raw | 3.4868E8 | 2 | 2 | 116 | 150 | Carbamidomethylation | C31:Carbamidomethylation:1000.00 | PEAKS DB |
| F.SC(+57.02)GENLFMSSQPYAWSK.V | N | 93.20 | 1990.8553 | 17 | -0.1 | 996.4349 | 2 | 63.34 | 1 | 38585 | BuCaMH03.raw | 2.72E8 | 1 | 1 | 89 | 105 | Carbamidomethylation | C2:Carbamidomethylation:1000.00 | PEAKS DB |
| R.NM(+15.99)LQMEWNSNAAQNAK.R | N | 90.98 | 1864.8196 | 16 | 3.6 | 933.4204 | 2 | 40.80 | 1 | 20445 | BuCaMH03.raw | 2.3955E8 | 4 | 4 | 52 | 67 | Oxidation (M) | M2:Oxidation (M):92.25 | PEAKS DB |
| K.FSC(+57.02)GENLFM(+15.99)SSQPYAWSK.V | N | 90.78 | 2153.9187 | 18 | -3.0 | 1077.9634 | 2 | 69.20 | 1 | 43521 | BuCaMH03.raw | 1.7168E7 | 5 | 5 | 88 | 105 | Carbamidomethylation; Oxidation (M) | C3:Carbamidomethylation:1000.00;M9:Oxidation (M):1000.00 | PEAKS DB |
| K.YIYVC(+57.02)QYC(+57.02)PAGNIR.G | N | 90.66 | 1775.8124 | 14 | 4.0 | 888.9171 | 2 | 52.86 | 1 | 30346 | BuCaMH03.raw | 1.8742E9 | 6 | 6 | 156 | 169 | Carbamidomethylation | C5:Carbamidomethylation:1000.00;C8:Carbamidomethylation:1000.00 | PEAKS DB |
| N.FVYGVGANPPDSVIGHYTQIVWYK.S | Y | 86.70 | 2709.3591 | 24 | 0.4 | 904.1273 | 3 | 75.27 | 1 | 48539 | BuCaMH03.raw | 6.0887E6 | 1 | 1 | 117 | 140 |  |  | PEAKS DB |
| K.SQLLGC(+57.02)AVTRC(+57.02)SSSKYIYVC(+57.02)QYC(+57.02)PAGNIR.G | Y | 83.02 | 3410.5991 | 29 | -1.8 | 1137.8716 | 3 | 50.08 | 1 | 27603 | BuCaMH03.raw | 2.3175E7 | 2 | 2 | 141 | 169 | Carbamidomethylation | C6:Carbamidomethylation:1000.00;C11:Carbamidomethylation:1000.00;C20:Carbamidomethylation:1000.00;C23:Carbamidomethylation:1000.00 | PEAKS DB |
| R.C(+57.02)TFAHSPPHLR.T | N | 82.82 | 1321.6350 | 11 | 0.4 | 661.8251 | 2 | 17.45 | 1 | 4128 | BuCaMH03.raw | 5.1391E8 | 3 | 3 | 73 | 83 | Carbamidomethylation | C1:Carbamidomethylation:1000.00 | PEAKS DB |
| K.VIQSWYDENKNFVYGVGANPPDSVIGHYTQIVWYK.S | Y | 80.33 | 4085.9951 | 35 | 0.6 | 818.2068 | 5 | 78.35 | 1 | 51012 | BuCaMH03.raw | 3.1771E7 | 3 | 3 | 106 | 140 |  |  | PEAKS DB |
| M.EWNSNAAQNAK.R | N | 80.28 | 1231.5581 | 11 | -0.7 | 616.7859 | 2 | 12.85 | 1 | 2052 | BuCaMH03.raw | 6.4438E5 | 1 | 1 | 57 | 67 |  |  | PEAKS DB |
| N.MLQMEWNSNAAQNAK.R | N | 79.43 | 1734.7817 | 15 | 0.8 | 868.3989 | 2 | 46.49 | 1 | 24445 | BuCaMH03.raw | 5.4821E7 | 1 | 1 | 53 | 67 |  |  | PEAKS DB |
| F.SC(+57.02)GENLFM(+15.99)SSQPYAWSK.V | N | 78.92 | 2006.8502 | 17 | -1.7 | 1004.4307 | 2 | 63.36 | 1 | 38717 | BuCaMH03.raw | 0 | 0 | 0 | 89 | 105 | Carbamidomethylation; Oxidation (M) | C2:Carbamidomethylation:1000.00;M8:Oxidation (M):1000.00 | PEAKS DB |
| C.TFAHSPPHLR.T | N | 78.16 | 1161.6042 | 10 | 0.4 | 388.2088 | 3 | 12.74 | 1 | 1951 | BuCaMH03.raw | 1.9167E8 | 3 | 3 | 74 | 83 |  |  | PEAKS DB |
| K.VIQSWYDENK.N | N | 77.34 | 1280.6036 | 10 | 0.6 | 641.3095 | 2 | 37.09 | 1 | 17259 | BuCaMH03.raw | 5.4482E8 | 1 | 1 | 106 | 115 |  |  | PEAKS DB |
| D.SVIGHYTQIVWYK.S | N | 76.45 | 1592.8351 | 13 | 1.1 | 531.9529 | 3 | 53.77 | 1 | 30679 | BuCaMH03.raw | 1.5322E6 | 1 | 1 | 128 | 140 |  |  | PEAKS DB |
| K.NFVYGVGANPPDSVIGH.Y | Y | 75.23 | 1741.8423 | 17 | 1.3 | 871.9296 | 2 | 59.44 | 1 | 35413 | BuCaMH03.raw | 3.7341E6 | 1 | 1 | 116 | 132 |  |  | PEAKS DB |
| K.VIQSWYDENKNFVYGVGANPPDSVIGHYTQIVWYKSQLLGC(+57.02)AVTR.C | Y | 74.74 | 5171.5601 | 45 | -0.2 | 1035.3191 | 5 | 83.12 | 1 | 54986 | BuCaMH03.raw | 9.9727E7 | 1 | 1 | 106 | 150 | Carbamidomethylation | C41:Carbamidomethylation:1000.00 | PEAKS DB |
| Y.IYVC(+57.02)QYC(+57.02)PAGNIR.G | N | 69.26 | 1612.7490 | 13 | 0.7 | 807.3823 | 2 | 42.95 | 1 | 22016 | BuCaMH03.raw | 1.4932E8 | 1 | 1 | 157 | 169 | Carbamidomethylation | C4:Carbamidomethylation:1000.00;C7:Carbamidomethylation:1000.00 | PEAKS DB |
| P.DSVIGHYTQIVWYK.S | Y | 68.88 | 1707.8621 | 14 | 1.3 | 570.2953 | 3 | 63.67 | 1 | 38955 | BuCaMH03.raw | 7.1495E5 | 1 | 1 | 127 | 140 |  |  | PEAKS DB |
| C.GENLFMSSQPYAWSK.V | N | 67.08 | 1743.7926 | 15 | -0.7 | 872.9030 | 2 | 62.86 | 1 | 38254 | BuCaMH03.raw | 6.1843E6 | 1 | 1 | 91 | 105 |  |  | PEAKS DB |
| K.YNDDYTNC(+57.02)K.S | N | 66.61 | 1191.4502 | 9 | 0.0 | 596.7324 | 2 | 13.11 | 1 | 2718 | BuCaMH03.raw | 1.4739E8 | 2 | 2 | 200 | 208 | Carbamidomethylation | C8:Carbamidomethylation:1000.00 | PEAKS DB |
| K.SQLLGC(+57.02)AVTR.C | Y | 66.59 | 1103.5757 | 10 | 0.0 | 552.7951 | 2 | 33.38 | 1 | 14240 | BuCaMH03.raw | 7.8985E8 | 2 | 2 | 141 | 150 | Carbamidomethylation | C6:Carbamidomethylation:1000.00 | PEAKS DB |
| Y.KSGPPC(+57.02)GDC(+57.02)PSAC(+57.02)VNGLC(+57.02)TNPC(+57.02)K.Y | N | 66.32 | 2535.0432 | 23 | 0.9 | 846.0225 | 3 | 30.53 | 1 | 12230 | BuCaMH03.raw | 3.4129E5 | 1 | 1 | 177 | 199 | Carbamidomethylation | C6:Carbamidomethylation:1000.00;C9:Carbamidomethylation:1000.00;C13:Carbamidomethylation:1000.00;C18:Carbamidomethylation:1000.00;C22:Carbamidomethylation:1000.00 | PEAKS DB |
| K.C(+57.02)AASC(+57.02)FC(+57.02)R.T | N | 63.47 | 1030.3783 | 8 | -0.5 | 516.1962 | 2 | 15.60 | 1 | 3145 | BuCaMH03.raw | 2.3446E8 | 1 | 1 | 225 | 232 | Carbamidomethylation | C1:Carbamidomethylation:1000.00;C5:Carbamidomethylation:1000.00;C7:Carbamidomethylation:1000.00 | PEAKS DB |
| N.MLQM(+15.99)EWNSNAAQNAK.R | N | 62.98 | 1750.7767 | 15 | 1.1 | 876.3966 | 2 | 46.48 | 1 | 24489 | BuCaMH03.raw | 7.4598E5 | 1 | 1 | 53 | 67 | Oxidation (M) | M4:Oxidation (M):9.05 | PEAKS DB |
| S.C(+57.02)GENLFMSSQPYAWSK.V | N | 62.04 | 1903.8232 | 16 | 0.6 | 952.9195 | 2 | 64.03 | 1 | 39173 | BuCaMH03.raw | 3.3027E7 | 1 | 1 | 90 | 105 | Carbamidomethylation | C1:Carbamidomethylation:1000.00 | PEAKS DB |
| A.DRC(+57.02)TFAHSPPHLR.T | N | 59.95 | 1592.7629 | 13 | 0.0 | 399.1980 | 4 | 17.37 | 1 | 4025 | BuCaMH03.raw | 1.6408E6 | 1 | 1 | 71 | 83 | Carbamidomethylation | C3:Carbamidomethylation:1000.00 | PEAKS DB |
| I.YVC(+57.02)QYC(+57.02)PAGNIR.G | N | 58.43 | 1499.6649 | 12 | 0.3 | 750.8400 | 2 | 32.81 | 1 | 13775 | BuCaMH03.raw | 1.3464E8 | 1 | 1 | 158 | 169 | Carbamidomethylation | C3:Carbamidomethylation:1000.00;C6:Carbamidomethylation:1000.00 | PEAKS DB |
| S.QLLGC(+57.02)AVTR.C | Y | 57.22 | 1016.5437 | 9 | -0.2 | 509.2790 | 2 | 30.69 | 1 | 12644 | BuCaMH03.raw | 1.32E8 | 1 | 1 | 142 | 150 | Carbamidomethylation | C5:Carbamidomethylation:1000.00 | PEAKS DB |
| R.C(+57.02)SSSKYIYVC(+57.02)QYC(+57.02)PAGNIR.G | N | 56.75 | 2325.0339 | 19 | -0.5 | 776.0182 | 3 | 41.99 | 1 | 20895 | BuCaMH03.raw | 1.169E6 | 1 | 1 | 151 | 169 | Carbamidomethylation | C1:Carbamidomethylation:1000.00;C10:Carbamidomethylation:1000.00;C13:Carbamidomethylation:1000.00 | PEAKS DB |
| E.NLFMSSQPYAWSK.V | N | 56.27 | 1557.7285 | 13 | -0.7 | 779.8710 | 2 | 60.42 | 1 | 36234 | BuCaMH03.raw | 3.801E5 | 1 | 1 | 93 | 105 |  |  | PEAKS DB |
| I.QSWYDENK.N | N | 54.93 | 1068.4512 | 8 | -0.1 | 535.2328 | 2 | 24.52 | 1 | 8368 | BuCaMH03.raw | 2.7802E7 | 1 | 1 | 108 | 115 |  |  | PEAKS DB |
| K.C(+57.02)AASC(+57.02)FC(+57.02)RTEII | N | 53.43 | 1486.6367 | 12 | 0.9 | 744.3263 | 2 | 46.59 | 1 | 24640 | BuCaMH03.raw | 2.8965E6 | 1 | 1 | 225 | 236 | Carbamidomethylation | C1:Carbamidomethylation:1000.00;C5:Carbamidomethylation:1000.00;C7:Carbamidomethylation:1000.00 | PEAKS DB |
| R.GSIATPYK.S | N | 53.13 | 835.4440 | 8 | -0.5 | 418.7290 | 2 | 19.69 | 1 | 5275 | BuCaMH03.raw | 1.8197E8 | 1 | 1 | 170 | 177 |  |  | PEAKS DB |
| R.NM(+15.99)LQM(+15.99)EWNSNAAQNAK.R | N | 53.13 | 1880.8146 | 16 | 1.2 | 941.4156 | 2 | 46.89 | 1 | 24806 | BuCaMH03.raw | 3.9953E5 | 1 | 1 | 52 | 67 | Oxidation (M) | M2:Oxidation (M):1000.00;M5:Oxidation (M):1000.00 | PEAKS DB |
| total 40 peptides |  |  |  |  |  |  |  |  |  |  |  |  |  |  |  |  |  |  |  |
| --- | --- | --- | --- | --- | --- | --- | --- | --- | --- | --- | --- | --- | --- | --- | --- | --- | --- | --- | --- |

T\_44

back to list

  

| Protein Coverage
| Supporting Peptides
|

Protein Coverage:

Supporting Peptides:

| Peptide | Uniq | -10lgP | Mass | Length | ppm | m/z | z | RT | Fraction | Scan | Source File | Area BuCaMH03\_B17 | #Feature | #Feature BuCaMH03\_B17 | Start | End | PTM | AScore | Found By |
| --- | --- | --- | --- | --- | --- | --- | --- | --- | --- | --- | --- | --- | --- | --- | --- | --- | --- | --- | --- |
| K.SGPPC(+57.02)GDC(+57.02)PSAC(+57.02)VNGLC(+57.02)TNPC(+57.02)K.Y | N | 121.75 | 2406.9482 | 22 | -0.5 | 1204.4808 | 2 | 38.36 | 1 | 18143 | BuCaMH03.raw | 4.9343E8 | 3 | 3 | 220 | 241 | Carbamidomethylation | C5:Carbamidomethylation:1000.00;C8:Carbamidomethylation:1000.00;C12:Carbamidomethylation:1000.00;C17:Carbamidomethylation:1000.00;C21:Carbamidomethylation:1000.00 | PEAKS DB |
| K.FSC(+57.02)GENLFMSSQPYAWSK.V | N | 111.14 | 2137.9238 | 18 | 0.0 | 1069.9692 | 2 | 69.16 | 1 | 43364 | BuCaMH03.raw | 4.5041E8 | 3 | 3 | 130 | 147 | Carbamidomethylation | C3:Carbamidomethylation:1000.00 | PEAKS DB |
| K.NFVYGVGANPPDSVIGHYTQIVWYK.S | Y | 106.01 | 2823.4021 | 25 | 0.4 | 942.1417 | 3 | 76.37 | 1 | 49615 | BuCaMH03.raw | 2.5384E9 | 3 | 3 | 158 | 182 |  |  | PEAKS DB |
| R.NMLQMEWNSNAAQNAK.R | N | 104.88 | 1848.8247 | 16 | 0.4 | 925.4200 | 2 | 52.32 | 1 | 29479 | BuCaMH03.raw | 2.3872E9 | 6 | 6 | 94 | 109 |  |  | PEAKS DB |
| R.NMLQM(+15.99)EWNSNAAQNAK.R | N | 97.35 | 1864.8196 | 16 | -0.5 | 933.4166 | 2 | 41.32 | 1 | 20903 | BuCaMH03.raw | 2.4898E8 | 4 | 4 | 94 | 109 | Oxidation (M) | M5:Oxidation (M):91.37 | PEAKS DB |
| K.NFVYGVGANPPDSVIGHYTQIVWYKSQLLGC(+57.02)AVTR.C | Y | 94.72 | 3908.9670 | 35 | 2.7 | 978.2516 | 4 | 82.15 | 1 | 54324 | BuCaMH03.raw | 3.4868E8 | 2 | 2 | 158 | 192 | Carbamidomethylation | C31:Carbamidomethylation:1000.00 | PEAKS DB |
| F.SC(+57.02)GENLFMSSQPYAWSK.V | N | 93.20 | 1990.8553 | 17 | -0.1 | 996.4349 | 2 | 63.34 | 1 | 38585 | BuCaMH03.raw | 2.72E8 | 1 | 1 | 131 | 147 | Carbamidomethylation | C2:Carbamidomethylation:1000.00 | PEAKS DB |
| R.NM(+15.99)LQMEWNSNAAQNAK.R | N | 90.98 | 1864.8196 | 16 | 3.6 | 933.4204 | 2 | 40.80 | 1 | 20445 | BuCaMH03.raw | 2.3955E8 | 4 | 4 | 94 | 109 | Oxidation (M) | M2:Oxidation (M):92.25 | PEAKS DB |
| K.FSC(+57.02)GENLFM(+15.99)SSQPYAWSK.V | N | 90.78 | 2153.9187 | 18 | -3.0 | 1077.9634 | 2 | 69.20 | 1 | 43521 | BuCaMH03.raw | 1.7168E7 | 5 | 5 | 130 | 147 | Carbamidomethylation; Oxidation (M) | C3:Carbamidomethylation:1000.00;M9:Oxidation (M):1000.00 | PEAKS DB |
| K.YIYVC(+57.02)QYC(+57.02)PAGNIR.G | N | 90.66 | 1775.8124 | 14 | 4.0 | 888.9171 | 2 | 52.86 | 1 | 30346 | BuCaMH03.raw | 1.8742E9 | 6 | 6 | 198 | 211 | Carbamidomethylation | C5:Carbamidomethylation:1000.00;C8:Carbamidomethylation:1000.00 | PEAKS DB |
| N.FVYGVGANPPDSVIGHYTQIVWYK.S | Y | 86.70 | 2709.3591 | 24 | 0.4 | 904.1273 | 3 | 75.27 | 1 | 48539 | BuCaMH03.raw | 6.0887E6 | 1 | 1 | 159 | 182 |  |  | PEAKS DB |
| K.SQLLGC(+57.02)AVTRC(+57.02)SSSKYIYVC(+57.02)QYC(+57.02)PAGNIR.G | Y | 83.02 | 3410.5991 | 29 | -1.8 | 1137.8716 | 3 | 50.08 | 1 | 27603 | BuCaMH03.raw | 2.3175E7 | 2 | 2 | 183 | 211 | Carbamidomethylation | C6:Carbamidomethylation:1000.00;C11:Carbamidomethylation:1000.00;C20:Carbamidomethylation:1000.00;C23:Carbamidomethylation:1000.00 | PEAKS DB |
| R.C(+57.02)TFAHSPPHLR.T | N | 82.82 | 1321.6350 | 11 | 0.4 | 661.8251 | 2 | 17.45 | 1 | 4128 | BuCaMH03.raw | 5.1391E8 | 3 | 3 | 115 | 125 | Carbamidomethylation | C1:Carbamidomethylation:1000.00 | PEAKS DB |
| K.VIQSWYDENKNFVYGVGANPPDSVIGHYTQIVWYK.S | Y | 80.33 | 4085.9951 | 35 | 0.6 | 818.2068 | 5 | 78.35 | 1 | 51012 | BuCaMH03.raw | 3.1771E7 | 3 | 3 | 148 | 182 |  |  | PEAKS DB |
| M.EWNSNAAQNAK.R | N | 80.28 | 1231.5581 | 11 | -0.7 | 616.7859 | 2 | 12.85 | 1 | 2052 | BuCaMH03.raw | 6.4438E5 | 1 | 1 | 99 | 109 |  |  | PEAKS DB |
| N.MLQMEWNSNAAQNAK.R | N | 79.43 | 1734.7817 | 15 | 0.8 | 868.3989 | 2 | 46.49 | 1 | 24445 | BuCaMH03.raw | 5.4821E7 | 1 | 1 | 95 | 109 |  |  | PEAKS DB |
| F.SC(+57.02)GENLFM(+15.99)SSQPYAWSK.V | N | 78.92 | 2006.8502 | 17 | -1.7 | 1004.4307 | 2 | 63.36 | 1 | 38717 | BuCaMH03.raw | 0 | 0 | 0 | 131 | 147 | Carbamidomethylation; Oxidation (M) | C2:Carbamidomethylation:1000.00;M8:Oxidation (M):1000.00 | PEAKS DB |
| C.TFAHSPPHLR.T | N | 78.16 | 1161.6042 | 10 | 0.4 | 388.2088 | 3 | 12.74 | 1 | 1951 | BuCaMH03.raw | 1.9167E8 | 3 | 3 | 116 | 125 |  |  | PEAKS DB |
| K.VIQSWYDENK.N | N | 77.34 | 1280.6036 | 10 | 0.6 | 641.3095 | 2 | 37.09 | 1 | 17259 | BuCaMH03.raw | 5.4482E8 | 1 | 1 | 148 | 157 |  |  | PEAKS DB |
| D.SVIGHYTQIVWYK.S | N | 76.45 | 1592.8351 | 13 | 1.1 | 531.9529 | 3 | 53.77 | 1 | 30679 | BuCaMH03.raw | 1.5322E6 | 1 | 1 | 170 | 182 |  |  | PEAKS DB |
| K.NFVYGVGANPPDSVIGH.Y | Y | 75.23 | 1741.8423 | 17 | 1.3 | 871.9296 | 2 | 59.44 | 1 | 35413 | BuCaMH03.raw | 3.7341E6 | 1 | 1 | 158 | 174 |  |  | PEAKS DB |
| K.VIQSWYDENKNFVYGVGANPPDSVIGHYTQIVWYKSQLLGC(+57.02)AVTR.C | Y | 74.74 | 5171.5601 | 45 | -0.2 | 1035.3191 | 5 | 83.12 | 1 | 54986 | BuCaMH03.raw | 9.9727E7 | 1 | 1 | 148 | 192 | Carbamidomethylation | C41:Carbamidomethylation:1000.00 | PEAKS DB |
| Y.IYVC(+57.02)QYC(+57.02)PAGNIR.G | N | 69.26 | 1612.7490 | 13 | 0.7 | 807.3823 | 2 | 42.95 | 1 | 22016 | BuCaMH03.raw | 1.4932E8 | 1 | 1 | 199 | 211 | Carbamidomethylation | C4:Carbamidomethylation:1000.00;C7:Carbamidomethylation:1000.00 | PEAKS DB |
| P.DSVIGHYTQIVWYK.S | Y | 68.88 | 1707.8621 | 14 | 1.3 | 570.2953 | 3 | 63.67 | 1 | 38955 | BuCaMH03.raw | 7.1495E5 | 1 | 1 | 169 | 182 |  |  | PEAKS DB |
| C.GENLFMSSQPYAWSK.V | N | 67.08 | 1743.7926 | 15 | -0.7 | 872.9030 | 2 | 62.86 | 1 | 38254 | BuCaMH03.raw | 6.1843E6 | 1 | 1 | 133 | 147 |  |  | PEAKS DB |
| K.YNDDYTNC(+57.02)K.S | N | 66.61 | 1191.4502 | 9 | 0.0 | 596.7324 | 2 | 13.11 | 1 | 2718 | BuCaMH03.raw | 1.4739E8 | 2 | 2 | 242 | 250 | Carbamidomethylation | C8:Carbamidomethylation:1000.00 | PEAKS DB |
| K.SQLLGC(+57.02)AVTR.C | Y | 66.59 | 1103.5757 | 10 | 0.0 | 552.7951 | 2 | 33.38 | 1 | 14240 | BuCaMH03.raw | 7.8985E8 | 2 | 2 | 183 | 192 | Carbamidomethylation | C6:Carbamidomethylation:1000.00 | PEAKS DB |
| Y.KSGPPC(+57.02)GDC(+57.02)PSAC(+57.02)VNGLC(+57.02)TNPC(+57.02)K.Y | N | 66.32 | 2535.0432 | 23 | 0.9 | 846.0225 | 3 | 30.53 | 1 | 12230 | BuCaMH03.raw | 3.4129E5 | 1 | 1 | 219 | 241 | Carbamidomethylation | C6:Carbamidomethylation:1000.00;C9:Carbamidomethylation:1000.00;C13:Carbamidomethylation:1000.00;C18:Carbamidomethylation:1000.00;C22:Carbamidomethylation:1000.00 | PEAKS DB |
| K.C(+57.02)AASC(+57.02)FC(+57.02)R.T | N | 63.47 | 1030.3783 | 8 | -0.5 | 516.1962 | 2 | 15.60 | 1 | 3145 | BuCaMH03.raw | 2.3446E8 | 1 | 1 | 267 | 274 | Carbamidomethylation | C1:Carbamidomethylation:1000.00;C5:Carbamidomethylation:1000.00;C7:Carbamidomethylation:1000.00 | PEAKS DB |
| N.MLQM(+15.99)EWNSNAAQNAK.R | N | 62.98 | 1750.7767 | 15 | 1.1 | 876.3966 | 2 | 46.48 | 1 | 24489 | BuCaMH03.raw | 7.4598E5 | 1 | 1 | 95 | 109 | Oxidation (M) | M4:Oxidation (M):9.05 | PEAKS DB |
| S.C(+57.02)GENLFMSSQPYAWSK.V | N | 62.04 | 1903.8232 | 16 | 0.6 | 952.9195 | 2 | 64.03 | 1 | 39173 | BuCaMH03.raw | 3.3027E7 | 1 | 1 | 132 | 147 | Carbamidomethylation | C1:Carbamidomethylation:1000.00 | PEAKS DB |
| A.DRC(+57.02)TFAHSPPHLR.T | N | 59.95 | 1592.7629 | 13 | 0.0 | 399.1980 | 4 | 17.37 | 1 | 4025 | BuCaMH03.raw | 1.6408E6 | 1 | 1 | 113 | 125 | Carbamidomethylation | C3:Carbamidomethylation:1000.00 | PEAKS DB |
| I.YVC(+57.02)QYC(+57.02)PAGNIR.G | N | 58.43 | 1499.6649 | 12 | 0.3 | 750.8400 | 2 | 32.81 | 1 | 13775 | BuCaMH03.raw | 1.3464E8 | 1 | 1 | 200 | 211 | Carbamidomethylation | C3:Carbamidomethylation:1000.00;C6:Carbamidomethylation:1000.00 | PEAKS DB |
| S.QLLGC(+57.02)AVTR.C | Y | 57.22 | 1016.5437 | 9 | -0.2 | 509.2790 | 2 | 30.69 | 1 | 12644 | BuCaMH03.raw | 1.32E8 | 1 | 1 | 184 | 192 | Carbamidomethylation | C5:Carbamidomethylation:1000.00 | PEAKS DB |
| R.C(+57.02)SSSKYIYVC(+57.02)QYC(+57.02)PAGNIR.G | N | 56.75 | 2325.0339 | 19 | -0.5 | 776.0182 | 3 | 41.99 | 1 | 20895 | BuCaMH03.raw | 1.169E6 | 1 | 1 | 193 | 211 | Carbamidomethylation | C1:Carbamidomethylation:1000.00;C10:Carbamidomethylation:1000.00;C13:Carbamidomethylation:1000.00 | PEAKS DB |
| E.NLFMSSQPYAWSK.V | N | 56.27 | 1557.7285 | 13 | -0.7 | 779.8710 | 2 | 60.42 | 1 | 36234 | BuCaMH03.raw | 3.801E5 | 1 | 1 | 135 | 147 |  |  | PEAKS DB |
| I.QSWYDENK.N | N | 54.93 | 1068.4512 | 8 | -0.1 | 535.2328 | 2 | 24.52 | 1 | 8368 | BuCaMH03.raw | 2.7802E7 | 1 | 1 | 150 | 157 |  |  | PEAKS DB |
| K.C(+57.02)AASC(+57.02)FC(+57.02)RTEII | N | 53.43 | 1486.6367 | 12 | 0.9 | 744.3263 | 2 | 46.59 | 1 | 24640 | BuCaMH03.raw | 2.8965E6 | 1 | 1 | 267 | 278 | Carbamidomethylation | C1:Carbamidomethylation:1000.00;C5:Carbamidomethylation:1000.00;C7:Carbamidomethylation:1000.00 | PEAKS DB |
| R.GSIATPYK.S | N | 53.13 | 835.4440 | 8 | -0.5 | 418.7290 | 2 | 19.69 | 1 | 5275 | BuCaMH03.raw | 1.8197E8 | 1 | 1 | 212 | 219 |  |  | PEAKS DB |
| R.NM(+15.99)LQM(+15.99)EWNSNAAQNAK.R | N | 53.13 | 1880.8146 | 16 | 1.2 | 941.4156 | 2 | 46.89 | 1 | 24806 | BuCaMH03.raw | 3.9953E5 | 1 | 1 | 94 | 109 | Oxidation (M) | M2:Oxidation (M):1000.00;M5:Oxidation (M):1000.00 | PEAKS DB |
| total 40 peptides |  |  |  |  |  |  |  |  |  |  |  |  |  |  |  |  |  |  |  |
| --- | --- | --- | --- | --- | --- | --- | --- | --- | --- | --- | --- | --- | --- | --- | --- | --- | --- | --- | --- |

T\_42

back to list

  

| Protein Coverage
| Supporting Peptides
|

Protein Coverage:

Supporting Peptides:

| Peptide | Uniq | -10lgP | Mass | Length | ppm | m/z | z | RT | Fraction | Scan | Source File | Area BuCaMH03\_B17 | #Feature | #Feature BuCaMH03\_B17 | Start | End | PTM | AScore | Found By |
| --- | --- | --- | --- | --- | --- | --- | --- | --- | --- | --- | --- | --- | --- | --- | --- | --- | --- | --- | --- |
| R.RPTWHYMDYGC(+57.02)YC(+57.02)GYGGSGTPIDDLDR.C | Y | 122.66 | 3211.3223 | 27 | -0.1 | 1071.4479 | 3 | 56.78 | 1 | 33454 | BuCaMH03.raw | 1.8052E9 | 9 | 9 | 51 | 77 | Carbamidomethylation | C11:Carbamidomethylation:1000.00;C13:Carbamidomethylation:1000.00 | PEAKS DB |
| K.VHDDC(+57.02)YGAVLK.R | Y | 100.01 | 1275.5918 | 11 | -0.3 | 638.8030 | 2 | 24.91 | 1 | 8530 | BuCaMH03.raw | 5.296E8 | 3 | 3 | 81 | 91 | Carbamidomethylation | C5:Carbamidomethylation:1000.00 | PEAKS DB |
| R.TPYINANYNINPR.R | Y | 98.04 | 1548.7684 | 13 | 0.3 | 775.3917 | 2 | 44.77 | 1 | 23425 | BuCaMH03.raw | 1.339E9 | 3 | 3 | 137 | 149 |  |  | PEAKS DB |
| H.YMDYGC(+57.02)YC(+57.02)GYGGSGTPIDDLDR.C | Y | 96.67 | 2533.9824 | 22 | 1.3 | 1268.0001 | 2 | 63.10 | 1 | 38558 | BuCaMH03.raw | 2.7198E8 | 2 | 2 | 56 | 77 | Carbamidomethylation | C6:Carbamidomethylation:1000.00;C8:Carbamidomethylation:1000.00 | PEAKS DB |
| K.C(+57.02)NPYFTTYDYYC(+57.02)GADGPYC(+57.02)R.N | Y | 96.58 | 2541.9663 | 20 | 2.3 | 1271.9934 | 2 | 61.92 | 1 | 37752 | BuCaMH03.raw | 1.7996E9 | 3 | 3 | 94 | 113 | Carbamidomethylation | C1:Carbamidomethylation:1000.00;C12:Carbamidomethylation:1000.00;C19:Carbamidomethylation:1000.00 | PEAKS DB |
| R.RRPTWHYMDYGC(+57.02)YC(+57.02)GYGGSGTPIDDLDR.C | Y | 89.31 | 3367.4233 | 28 | 0.5 | 674.4923 | 5 | 52.46 | 1 | 29426 | BuCaMH03.raw | 1.5194E9 | 3 | 3 | 50 | 77 | Carbamidomethylation | C12:Carbamidomethylation:1000.00;C14:Carbamidomethylation:1000.00 | PEAKS DB |
| K.VHDDC(+57.02)YGAVLKR.K | Y | 86.68 | 1431.6929 | 12 | 0.6 | 478.2385 | 3 | 18.32 | 1 | 5355 | BuCaMH03.raw | 1.325E8 | 4 | 4 | 81 | 92 | Carbamidomethylation | C5:Carbamidomethylation:1000.00 | PEAKS DB |
| T.WHYMDYGC(+57.02)YC(+57.02)GYGGSGTPIDDLDR.C | Y | 86.29 | 2857.1206 | 24 | 0.2 | 953.3810 | 3 | 62.86 | 1 | 38208 | BuCaMH03.raw | 2.4087E7 | 1 | 1 | 54 | 77 | Carbamidomethylation | C8:Carbamidomethylation:1000.00;C10:Carbamidomethylation:1000.00 | PEAKS DB |
| R.KC(+57.02)NPYFTTYDYYC(+57.02)GADGPYC(+57.02)R.N | Y | 85.96 | 2670.0613 | 21 | -0.7 | 891.0271 | 3 | 51.56 | 1 | 29044 | BuCaMH03.raw | 7.2782E7 | 2 | 2 | 93 | 113 | Carbamidomethylation | C2:Carbamidomethylation:1000.00;C13:Carbamidomethylation:1000.00;C20:Carbamidomethylation:1000.00 | PEAKS DB |
| P.TWHYMDYGC(+57.02)YC(+57.02)GYGGSGTPIDDLDR.C | Y | 84.02 | 2958.1685 | 25 | -0.6 | 987.0628 | 3 | 63.50 | 1 | 39184 | BuCaMH03.raw | 3.0856E8 | 2 | 2 | 53 | 77 | Carbamidomethylation | C9:Carbamidomethylation:1000.00;C11:Carbamidomethylation:1000.00 | PEAKS DB |
| D.YGC(+57.02)YC(+57.02)GYGGSGTPIDDLDR.C | Y | 83.91 | 2124.8516 | 19 | 0.3 | 1063.4333 | 2 | 53.31 | 1 | 30243 | BuCaMH03.raw | 1.1347E7 | 1 | 1 | 59 | 77 | Carbamidomethylation | C3:Carbamidomethylation:1000.00;C5:Carbamidomethylation:1000.00 | PEAKS DB |
| L.NLIQFSYLIQC(+57.02)ANHR.R | Y | 83.84 | 1875.9414 | 15 | -0.1 | 938.9779 | 2 | 66.59 | 1 | 41694 | BuCaMH03.raw | 2.8781E9 | 11 | 11 | 35 | 49 | Carbamidomethylation | C11:Carbamidomethylation:1000.00 | PEAKS DB |
| C.NPYFTTYDYYC(+57.02)GADGPYC(+57.02)R.N | Y | 83.40 | 2381.9358 | 19 | -0.7 | 1191.9744 | 2 | 61.00 | 1 | 36962 | BuCaMH03.raw | 3.2482E8 | 2 | 2 | 95 | 113 | Carbamidomethylation | C11:Carbamidomethylation:1000.00;C18:Carbamidomethylation:1000.00 | PEAKS DB |
| M.DYGC(+57.02)YC(+57.02)GYGGSGTPIDDLDR.C | Y | 83.36 | 2239.8787 | 20 | 0.4 | 1120.9470 | 2 | 59.09 | 1 | 35006 | BuCaMH03.raw | 1.049E7 | 1 | 1 | 58 | 77 | Carbamidomethylation | C4:Carbamidomethylation:1000.00;C6:Carbamidomethylation:1000.00 | PEAKS DB |
| R.PTWHYMDYGC(+57.02)YC(+57.02)GYGGSGTPIDDLDR.C | Y | 83.00 | 3055.2212 | 26 | -1.0 | 1019.4133 | 3 | 63.84 | 1 | 39516 | BuCaMH03.raw | 1.0892E8 | 2 | 2 | 52 | 77 | Carbamidomethylation | C10:Carbamidomethylation:1000.00;C12:Carbamidomethylation:1000.00 | PEAKS DB |
| P.YINANYNINPR.R | Y | 81.77 | 1350.6680 | 11 | 0.6 | 676.3417 | 2 | 35.58 | 1 | 15920 | BuCaMH03.raw | 5.2139E8 | 1 | 1 | 139 | 149 |  |  | PEAKS DB |
| V.HDDC(+57.02)YGAVLKR.K | Y | 81.74 | 1332.6244 | 11 | -0.4 | 445.2152 | 3 | 17.97 | 1 | 4312 | BuCaMH03.raw | 3.012E7 | 2 | 2 | 82 | 92 | Carbamidomethylation | C4:Carbamidomethylation:1000.00 | PEAKS DB |
| P.YFTTYDYYC(+57.02)GADGPYC(+57.02)R.N | Y | 80.93 | 2170.8401 | 17 | -2.5 | 1086.4246 | 2 | 56.36 | 1 | 32762 | BuCaMH03.raw | 1.3663E8 | 1 | 1 | 97 | 113 | Carbamidomethylation | C9:Carbamidomethylation:1000.00;C16:Carbamidomethylation:1000.00 | PEAKS DB |
| R.RPTWHYM(+15.99)DYGC(+57.02)YC(+57.02)GYGGSGTPIDDLDR.C | Y | 80.32 | 3227.3171 | 27 | 0.1 | 1076.7798 | 3 | 52.72 | 1 | 29904 | BuCaMH03.raw | 6.0388E7 | 5 | 5 | 51 | 77 | Oxidation (M); Carbamidomethylation | M7:Oxidation (M):1000.00;C11:Carbamidomethylation:1000.00;C13:Carbamidomethylation:1000.00 | PEAKS DB |
| N.LIQFSYLIQC(+57.02)ANHR.R | Y | 80.06 | 1761.8984 | 14 | 0.5 | 881.9570 | 2 | 62.52 | 1 | 37958 | BuCaMH03.raw | 2.3579E7 | 2 | 2 | 36 | 49 | Carbamidomethylation | C10:Carbamidomethylation:1000.00 | PEAKS DB |
| P.LNLIQFSYLIQC(+57.02)ANHR.R | Y | 79.98 | 1989.0254 | 16 | 1.7 | 664.0168 | 3 | 77.20 | 1 | 50404 | BuCaMH03.raw | 1.3534E8 | 2 | 2 | 34 | 49 | Carbamidomethylation | C12:Carbamidomethylation:1000.00 | PEAKS DB |
| R.AVC(+57.02)DC(+57.02)DVKAALC(+57.02)FAR.T | Y | 79.37 | 1754.7902 | 15 | 0.5 | 878.4028 | 2 | 46.95 | 1 | 24960 | BuCaMH03.raw | 2.3702E6 | 2 | 2 | 122 | 136 | Carbamidomethylation | C3:Carbamidomethylation:1000.00;C5:Carbamidomethylation:1000.00;C12:Carbamidomethylation:1000.00 | PEAKS DB |
| Y.FTTYDYYC(+57.02)GADGPYC(+57.02)R.N | Y | 78.56 | 2007.7767 | 16 | 1.9 | 1004.8975 | 2 | 50.42 | 1 | 28170 | BuCaMH03.raw | 2.492E8 | 3 | 3 | 98 | 113 | Carbamidomethylation | C8:Carbamidomethylation:1000.00;C15:Carbamidomethylation:1000.00 | PEAKS DB |
| R.RRPTWHYM(+15.99)DYGC(+57.02)YC(+57.02)GYGGSGTPIDDLDR.C | Y | 78.49 | 3383.4182 | 28 | -1.3 | 846.8607 | 4 | 52.41 | 1 | 29985 | BuCaMH03.raw | 3.2508E7 | 3 | 3 | 50 | 77 | Oxidation (M); Carbamidomethylation | M8:Oxidation (M):1000.00;C12:Carbamidomethylation:1000.00;C14:Carbamidomethylation:1000.00 | PEAKS DB |
| V.HDDC(+57.02)YGAVLK.R | Y | 77.02 | 1176.5233 | 10 | 0.1 | 589.2690 | 2 | 22.89 | 1 | 7411 | BuCaMH03.raw | 2.7654E8 | 2 | 2 | 82 | 91 | Carbamidomethylation | C4:Carbamidomethylation:1000.00 | PEAKS DB |
| L.IQFSYLIQC(+57.02)ANHR.R | Y | 76.97 | 1648.8143 | 13 | 1.2 | 825.4154 | 2 | 53.58 | 1 | 30470 | BuCaMH03.raw | 1.7614E7 | 2 | 2 | 37 | 49 | Carbamidomethylation | C9:Carbamidomethylation:1000.00 | PEAKS DB |
| N.PYFTTYDYYC(+57.02)GADGPYC(+57.02)R.N | Y | 73.27 | 2267.8928 | 18 | -0.5 | 1134.9531 | 2 | 60.38 | 1 | 36085 | BuCaMH03.raw | 1.0805E7 | 1 | 1 | 96 | 113 | Carbamidomethylation | C10:Carbamidomethylation:1000.00;C17:Carbamidomethylation:1000.00 | PEAKS DB |
| Y.INANYNINPR.R | Y | 70.91 | 1187.6047 | 10 | 0.2 | 594.8098 | 2 | 28.98 | 1 | 11434 | BuCaMH03.raw | 1.004E8 | 1 | 1 | 140 | 149 |  |  | PEAKS DB |
| Y.MDYGC(+57.02)YC(+57.02)GYGGSGTPIDDLDR.C | Y | 69.34 | 2370.9192 | 21 | -0.6 | 1186.4662 | 2 | 60.14 | 1 | 35844 | BuCaMH03.raw | 1.2299E7 | 1 | 1 | 57 | 77 | Carbamidomethylation | C5:Carbamidomethylation:1000.00;C7:Carbamidomethylation:1000.00 | PEAKS DB |
| H.DDC(+57.02)YGAVLKR.K | Y | 68.46 | 1195.5656 | 10 | 0.3 | 598.7902 | 2 | 27.06 | 1 | 10045 | BuCaMH03.raw | 5.0135E6 | 1 | 1 | 83 | 92 | Carbamidomethylation | C3:Carbamidomethylation:1000.00 | PEAKS DB |
| F.TTYDYYC(+57.02)GADGPYC(+57.02)R.N | Y | 68.25 | 1860.7083 | 15 | 0.3 | 931.3617 | 2 | 41.22 | 1 | 20201 | BuCaMH03.raw | 4.1122E7 | 1 | 1 | 99 | 113 | Carbamidomethylation | C7:Carbamidomethylation:1000.00;C14:Carbamidomethylation:1000.00 | PEAKS DB |
| D.YYC(+57.02)GADGPYC(+57.02)R.N | Y | 67.84 | 1380.5227 | 11 | 0.8 | 691.2692 | 2 | 25.98 | 1 | 9110 | BuCaMH03.raw | 2.2258E7 | 1 | 1 | 103 | 113 | Carbamidomethylation | C3:Carbamidomethylation:1000.00;C10:Carbamidomethylation:1000.00 | PEAKS DB |
| H.YM(+15.99)DYGC(+57.02)YC(+57.02)GYGGSGTPIDDLDR.C | Y | 67.64 | 2549.9773 | 22 | -0.1 | 1275.9958 | 2 | 58.36 | 1 | 34419 | BuCaMH03.raw | 6.7922E6 | 2 | 2 | 56 | 77 | Oxidation (M); Carbamidomethylation | M2:Oxidation (M):1000.00;C6:Carbamidomethylation:1000.00;C8:Carbamidomethylation:1000.00 | PEAKS DB |
| W.HYMDYGC(+57.02)YC(+57.02)GYGGSGTPIDDLDR.C | Y | 66.91 | 2671.0413 | 23 | 0.5 | 891.3548 | 3 | 54.97 | 1 | 31590 | BuCaMH03.raw | 3.6351E7 | 1 | 1 | 55 | 77 | Carbamidomethylation | C7:Carbamidomethylation:1000.00;C9:Carbamidomethylation:1000.00 | PEAKS DB |
| L.NLIQFSYLIQC(+57.02)ANHRR.R | Y | 66.64 | 2032.0425 | 16 | -0.3 | 509.0178 | 4 | 57.41 | 1 | 33555 | BuCaMH03.raw | 2.8392E7 | 1 | 1 | 35 | 50 | Carbamidomethylation | C11:Carbamidomethylation:1000.00 | PEAKS DB |
| T.TYDYYC(+57.02)GADGPYC(+57.02)R.N | Y | 65.81 | 1759.6606 | 14 | -0.1 | 880.8375 | 2 | 40.08 | 1 | 19315 | BuCaMH03.raw | 3.1492E7 | 1 | 1 | 100 | 113 | Carbamidomethylation | C6:Carbamidomethylation:1000.00;C13:Carbamidomethylation:1000.00 | PEAKS DB |
| T.YDYYC(+57.02)GADGPYC(+57.02)R.N | Y | 65.39 | 1658.6129 | 13 | -0.5 | 830.3134 | 2 | 39.00 | 1 | 18639 | BuCaMH03.raw | 2.0689E7 | 1 | 1 | 101 | 113 | Carbamidomethylation | C5:Carbamidomethylation:1000.00;C12:Carbamidomethylation:1000.00 | PEAKS DB |
| K.AALC(+57.02)FAR.T | N | 64.40 | 807.4061 | 7 | 0.3 | 404.7105 | 2 | 30.53 | 1 | 12209 | BuCaMH03.raw | 2.029E7 | 1 | 1 | 130 | 136 | Carbamidomethylation | C4:Carbamidomethylation:1000.00 | PEAKS DB |
| G.YGGSGTPIDDLDR.C | Y | 57.06 | 1364.6207 | 13 | 0.9 | 683.3182 | 2 | 41.20 | 1 | 20295 | BuCaMH03.raw | 1.215E6 | 1 | 1 | 65 | 77 |  |  | PEAKS DB |
| H.DDC(+57.02)YGAVLK.R | Y | 56.12 | 1039.4645 | 9 | 0.7 | 520.7399 | 2 | 35.85 | 1 | 16119 | BuCaMH03.raw | 1.8094E7 | 1 | 1 | 83 | 91 | Carbamidomethylation | C3:Carbamidomethylation:1000.00 | PEAKS DB |
| I.NANYNINPR.R | Y | 54.99 | 1074.5206 | 9 | 0.7 | 538.2679 | 2 | 20.02 | 1 | 5503 | BuCaMH03.raw | 2.7619E7 | 1 | 1 | 141 | 149 |  |  | PEAKS DB |
| total 41 peptides |  |  |  |  |  |  |  |  |  |  |  |  |  |  |  |  |  |  |  |
| --- | --- | --- | --- | --- | --- | --- | --- | --- | --- | --- | --- | --- | --- | --- | --- | --- | --- | --- | --- |

ACE73578.1

back to list

  

| Protein Coverage
| Supporting Peptides
|

Protein Coverage:

Supporting Peptides:

| Peptide | Uniq | -10lgP | Mass | Length | ppm | m/z | z | RT | Fraction | Scan | Source File | Area BuCaMH03\_B17 | #Feature | #Feature BuCaMH03\_B17 | Start | End | PTM | AScore | Found By |
| --- | --- | --- | --- | --- | --- | --- | --- | --- | --- | --- | --- | --- | --- | --- | --- | --- | --- | --- | --- |
| K.SGPPC(+57.02)GDC(+57.02)PSAC(+57.02)VNGLC(+57.02)TNPC(+57.02)K.Y | N | 121.75 | 2406.9482 | 22 | -0.5 | 1204.4808 | 2 | 38.36 | 1 | 18143 | BuCaMH03.raw | 4.9343E8 | 3 | 3 | 180 | 201 | Carbamidomethylation | C5:Carbamidomethylation:1000.00;C8:Carbamidomethylation:1000.00;C12:Carbamidomethylation:1000.00;C17:Carbamidomethylation:1000.00;C21:Carbamidomethylation:1000.00 | PEAKS DB |
| I.FSC(+57.02)GENLFMSSQPYAWSK.V | N | 111.14 | 2137.9238 | 18 | 0.0 | 1069.9692 | 2 | 69.16 | 1 | 43364 | BuCaMH03.raw | 4.5041E8 | 3 | 3 | 90 | 107 | Carbamidomethylation | C3:Carbamidomethylation:1000.00 | PEAKS DB |
| G.IFSC(+57.02)GENLFMSSQPYAWSK.V | Y | 106.85 | 2251.0078 | 19 | -0.1 | 1126.5111 | 2 | 75.24 | 1 | 48893 | BuCaMH03.raw | 3.6678E7 | 4 | 4 | 89 | 107 | Carbamidomethylation | C4:Carbamidomethylation:1000.00 | PEAKS DB |
| R.NMLQMEWNSNAAQNAK.R | N | 104.88 | 1848.8247 | 16 | 0.4 | 925.4200 | 2 | 52.32 | 1 | 29479 | BuCaMH03.raw | 2.3872E9 | 6 | 6 | 54 | 69 |  |  | PEAKS DB |
| R.NMLQM(+15.99)EWNSNAAQNAK.R | N | 97.35 | 1864.8196 | 16 | -0.5 | 933.4166 | 2 | 41.32 | 1 | 20903 | BuCaMH03.raw | 2.4898E8 | 4 | 4 | 54 | 69 | Oxidation (M) | M5:Oxidation (M):91.37 | PEAKS DB |
| F.SC(+57.02)GENLFMSSQPYAWSK.V | N | 93.20 | 1990.8553 | 17 | -0.1 | 996.4349 | 2 | 63.34 | 1 | 38585 | BuCaMH03.raw | 2.72E8 | 1 | 1 | 91 | 107 | Carbamidomethylation | C2:Carbamidomethylation:1000.00 | PEAKS DB |
| R.NM(+15.99)LQMEWNSNAAQNAK.R | N | 90.98 | 1864.8196 | 16 | 3.6 | 933.4204 | 2 | 40.80 | 1 | 20445 | BuCaMH03.raw | 2.3955E8 | 4 | 4 | 54 | 69 | Oxidation (M) | M2:Oxidation (M):92.25 | PEAKS DB |
| I.FSC(+57.02)GENLFM(+15.99)SSQPYAWSK.V | N | 90.78 | 2153.9187 | 18 | -3.0 | 1077.9634 | 2 | 69.20 | 1 | 43521 | BuCaMH03.raw | 1.7168E7 | 5 | 5 | 90 | 107 | Carbamidomethylation; Oxidation (M) | C3:Carbamidomethylation:1000.00;M9:Oxidation (M):1000.00 | PEAKS DB |
| R.C(+57.02)TFAHSPPHLR.T | N | 82.82 | 1321.6350 | 11 | 0.4 | 661.8251 | 2 | 17.45 | 1 | 4128 | BuCaMH03.raw | 5.1391E8 | 3 | 3 | 75 | 85 | Carbamidomethylation | C1:Carbamidomethylation:1000.00 | PEAKS DB |
| M.EWNSNAAQNAK.R | N | 80.28 | 1231.5581 | 11 | -0.7 | 616.7859 | 2 | 12.85 | 1 | 2052 | BuCaMH03.raw | 6.4438E5 | 1 | 1 | 59 | 69 |  |  | PEAKS DB |
| N.MLQMEWNSNAAQNAK.R | N | 79.43 | 1734.7817 | 15 | 0.8 | 868.3989 | 2 | 46.49 | 1 | 24445 | BuCaMH03.raw | 5.4821E7 | 1 | 1 | 55 | 69 |  |  | PEAKS DB |
| F.SC(+57.02)GENLFM(+15.99)SSQPYAWSK.V | N | 78.92 | 2006.8502 | 17 | -1.7 | 1004.4307 | 2 | 63.36 | 1 | 38717 | BuCaMH03.raw | 0 | 0 | 0 | 91 | 107 | Carbamidomethylation; Oxidation (M) | C2:Carbamidomethylation:1000.00;M8:Oxidation (M):1000.00 | PEAKS DB |
| C.TFAHSPPHLR.T | N | 78.16 | 1161.6042 | 10 | 0.4 | 388.2088 | 3 | 12.74 | 1 | 1951 | BuCaMH03.raw | 1.9167E8 | 3 | 3 | 76 | 85 |  |  | PEAKS DB |
| K.VIQSWYDENK.K | N | 77.34 | 1280.6036 | 10 | 0.6 | 641.3095 | 2 | 37.09 | 1 | 17259 | BuCaMH03.raw | 5.4482E8 | 1 | 1 | 108 | 117 |  |  | PEAKS DB |
| G.SVIGHYTQIVWYK.S | N | 76.45 | 1592.8351 | 13 | 1.1 | 531.9529 | 3 | 53.77 | 1 | 30679 | BuCaMH03.raw | 1.5322E6 | 1 | 1 | 130 | 142 |  |  | PEAKS DB |
| C.GENLFMSSQPYAWSK.V | N | 67.08 | 1743.7926 | 15 | -0.7 | 872.9030 | 2 | 62.86 | 1 | 38254 | BuCaMH03.raw | 6.1843E6 | 1 | 1 | 93 | 107 |  |  | PEAKS DB |
| K.YNDDYTNC(+57.02)K.S | N | 66.61 | 1191.4502 | 9 | 0.0 | 596.7324 | 2 | 13.11 | 1 | 2718 | BuCaMH03.raw | 1.4739E8 | 2 | 2 | 202 | 210 | Carbamidomethylation | C8:Carbamidomethylation:1000.00 | PEAKS DB |
| Y.KSGPPC(+57.02)GDC(+57.02)PSAC(+57.02)VNGLC(+57.02)TNPC(+57.02)K.Y | N | 66.32 | 2535.0432 | 23 | 0.9 | 846.0225 | 3 | 30.53 | 1 | 12230 | BuCaMH03.raw | 3.4129E5 | 1 | 1 | 179 | 201 | Carbamidomethylation | C6:Carbamidomethylation:1000.00;C9:Carbamidomethylation:1000.00;C13:Carbamidomethylation:1000.00;C18:Carbamidomethylation:1000.00;C22:Carbamidomethylation:1000.00 | PEAKS DB |
| N.MLQM(+15.99)EWNSNAAQNAK.R | N | 62.98 | 1750.7767 | 15 | 1.1 | 876.3966 | 2 | 46.48 | 1 | 24489 | BuCaMH03.raw | 7.4598E5 | 1 | 1 | 55 | 69 | Oxidation (M) | M4:Oxidation (M):9.05 | PEAKS DB |
| S.C(+57.02)GENLFMSSQPYAWSK.V | N | 62.04 | 1903.8232 | 16 | 0.6 | 952.9195 | 2 | 64.03 | 1 | 39173 | BuCaMH03.raw | 3.3027E7 | 1 | 1 | 92 | 107 | Carbamidomethylation | C1:Carbamidomethylation:1000.00 | PEAKS DB |
| A.DRC(+57.02)TFAHSPPHLR.T | N | 59.95 | 1592.7629 | 13 | 0.0 | 399.1980 | 4 | 17.37 | 1 | 4025 | BuCaMH03.raw | 1.6408E6 | 1 | 1 | 73 | 85 | Carbamidomethylation | C3:Carbamidomethylation:1000.00 | PEAKS DB |
| E.NLFMSSQPYAWSK.V | N | 56.27 | 1557.7285 | 13 | -0.7 | 779.8710 | 2 | 60.42 | 1 | 36234 | BuCaMH03.raw | 3.801E5 | 1 | 1 | 95 | 107 |  |  | PEAKS DB |
| I.QSWYDENK.K | N | 54.93 | 1068.4512 | 8 | -0.1 | 535.2328 | 2 | 24.52 | 1 | 8368 | BuCaMH03.raw | 2.7802E7 | 1 | 1 | 110 | 117 |  |  | PEAKS DB |
| K.VIQSWYDENKK.F | N | 53.89 | 1408.6986 | 11 | 0.7 | 705.3571 | 2 | 25.45 | 1 | 8817 | BuCaMH03.raw | 4.9114E5 | 1 | 1 | 108 | 118 |  |  | PEAKS DB |
| R.NM(+15.99)LQM(+15.99)EWNSNAAQNAK.R | N | 53.13 | 1880.8146 | 16 | 1.2 | 941.4156 | 2 | 46.89 | 1 | 24806 | BuCaMH03.raw | 3.9953E5 | 1 | 1 | 54 | 69 | Oxidation (M) | M2:Oxidation (M):1000.00;M5:Oxidation (M):1000.00 | PEAKS DB |
| total 25 peptides |  |  |  |  |  |  |  |  |  |  |  |  |  |  |  |  |  |  |  |
| --- | --- | --- | --- | --- | --- | --- | --- | --- | --- | --- | --- | --- | --- | --- | --- | --- | --- | --- | --- |

T\_127

back to list

  

| Protein Coverage
| Supporting Peptides
|

Protein Coverage:

Supporting Peptides:

| Peptide | Uniq | -10lgP | Mass | Length | ppm | m/z | z | RT | Fraction | Scan | Source File | Area BuCaMH03\_B17 | #Feature | #Feature BuCaMH03\_B17 | Start | End | PTM | AScore | Found By |
| --- | --- | --- | --- | --- | --- | --- | --- | --- | --- | --- | --- | --- | --- | --- | --- | --- | --- | --- | --- |
| K.TWGHYVDYGC(+57.02)YC(+57.02)GAGGSGTPVDALDR.C | Y | 116.25 | 2833.1860 | 26 | -0.1 | 1417.6002 | 2 | 56.08 | 1 | 32896 | BuCaMH03.raw | 4.0017E9 | 7 | 7 | 53 | 78 | Carbamidomethylation | C10:Carbamidomethylation:1000.00;C12:Carbamidomethylation:1000.00 | PEAKS DB |
| R.RTIIC(+57.02)YGAAGTC(+57.02)AR.I | N | 98.88 | 1568.7551 | 14 | 0.3 | 785.3851 | 2 | 24.68 | 1 | 8363 | BuCaMH03.raw | 2.3225E7 | 2 | 2 | 110 | 123 | Carbamidomethylation | C5:Carbamidomethylation:1000.00;C12:Carbamidomethylation:1000.00 | PEAKS DB |
| G.HYVDYGC(+57.02)YC(+57.02)GAGGSGTPVDALDR.C | Y | 98.37 | 2489.0376 | 23 | -0.5 | 830.6861 | 3 | 46.53 | 1 | 24631 | BuCaMH03.raw | 1.0074E7 | 2 | 2 | 56 | 78 | Carbamidomethylation | C7:Carbamidomethylation:1000.00;C9:Carbamidomethylation:1000.00 | PEAKS DB |
| R.C(+57.02)C(+57.02)YVHDNC(+57.02)YGDAEK.I | N | 90.97 | 1789.6494 | 14 | -0.2 | 895.8318 | 2 | 17.70 | 1 | 4370 | BuCaMH03.raw | 2.9791E8 | 4 | 4 | 79 | 92 | Carbamidomethylation | C1:Carbamidomethylation:1000.00;C2:Carbamidomethylation:1000.00;C8:Carbamidomethylation:1000.00 | PEAKS DB |
| R.TIIC(+57.02)YGAAGTC(+57.02)AR.I | N | 87.88 | 1412.6541 | 13 | 0.2 | 707.3345 | 2 | 33.70 | 1 | 14805 | BuCaMH03.raw | 2.1013E9 | 2 | 2 | 111 | 123 | Carbamidomethylation | C4:Carbamidomethylation:1000.00;C11:Carbamidomethylation:1000.00 | PEAKS DB |
| H.YVDYGC(+57.02)YC(+57.02)GAGGSGTPVDALDR.C | Y | 83.29 | 2351.9788 | 22 | -0.2 | 1176.9965 | 2 | 54.31 | 1 | 31046 | BuCaMH03.raw | 3.6621E7 | 2 | 2 | 57 | 78 | Carbamidomethylation | C6:Carbamidomethylation:1000.00;C8:Carbamidomethylation:1000.00 | PEAKS DB |
| Y.GC(+57.02)YC(+57.02)GAGGSGTPVDALDR.C | N | 81.29 | 1811.7566 | 18 | 1.0 | 906.8865 | 2 | 39.40 | 1 | 18973 | BuCaMH03.raw | 1.6098E6 | 1 | 1 | 61 | 78 | Carbamidomethylation | C2:Carbamidomethylation:1000.00;C4:Carbamidomethylation:1000.00 | PEAKS DB |
| C.YC(+57.02)GAGGSGTPVDALDR.C | N | 81.27 | 1594.7046 | 16 | -0.2 | 798.3594 | 2 | 37.56 | 1 | 17571 | BuCaMH03.raw | 3.935E6 | 1 | 1 | 63 | 78 | Carbamidomethylation | C2:Carbamidomethylation:1000.00 | PEAKS DB |
| T.IIC(+57.02)YGAAGTC(+57.02)AR.I | N | 80.96 | 1311.6063 | 12 | -0.3 | 656.8102 | 2 | 27.05 | 1 | 10166 | BuCaMH03.raw | 1.2342E8 | 1 | 1 | 112 | 123 | Carbamidomethylation | C3:Carbamidomethylation:1000.00;C10:Carbamidomethylation:1000.00 | PEAKS DB |
| H.LNLYQFMQMIR.Y | Y | 76.26 | 1455.7367 | 11 | 1.0 | 728.8763 | 2 | 91.16 | 1 | 61535 | BuCaMH03.raw | 1.3252E8 | 2 | 2 | 35 | 45 |  |  | PEAKS DB |
| G.C(+57.02)YC(+57.02)GAGGSGTPVDALDR.C | N | 74.59 | 1754.7352 | 17 | -0.1 | 878.3748 | 2 | 39.18 | 1 | 18847 | BuCaMH03.raw | 0 | 0 | 0 | 62 | 78 | Carbamidomethylation | C1:Carbamidomethylation:1000.00;C3:Carbamidomethylation:1000.00 | PEAKS DB |
| V.DYGC(+57.02)YC(+57.02)GAGGSGTPVDALDR.C | N | 74.49 | 2089.8469 | 20 | 0.0 | 1045.9308 | 2 | 50.93 | 1 | 28639 | BuCaMH03.raw | 5.2431E7 | 2 | 2 | 59 | 78 | Carbamidomethylation | C4:Carbamidomethylation:1000.00;C6:Carbamidomethylation:1000.00 | PEAKS DB |
| C.C(+57.02)YVHDNC(+57.02)YGDAEK.I | N | 73.17 | 1629.6188 | 13 | -0.3 | 544.2134 | 3 | 14.86 | 1 | 2919 | BuCaMH03.raw | 1.0574E7 | 2 | 2 | 80 | 92 | Carbamidomethylation | C1:Carbamidomethylation:1000.00;C7:Carbamidomethylation:1000.00 | PEAKS DB |
| L.NLYQFMQMIR.Y | Y | 71.53 | 1342.6526 | 10 | 0.8 | 672.3341 | 2 | 79.49 | 1 | 51920 | BuCaMH03.raw | 5.2816E9 | 4 | 4 | 36 | 45 |  |  | PEAKS DB |
| R.YTIPC(+57.02)DKTWGHYVDYGC(+57.02)YC(+57.02)GAGGSGTPVDALDR.C | Y | 71.26 | 3710.5864 | 33 | 0.3 | 928.6542 | 4 | 60.20 | 1 | 35965 | BuCaMH03.raw | 2.0275E7 | 2 | 2 | 46 | 78 | Carbamidomethylation | C5:Carbamidomethylation:1000.00;C17:Carbamidomethylation:1000.00;C19:Carbamidomethylation:1000.00 | PEAKS DB |
| D.YGC(+57.02)YC(+57.02)GAGGSGTPVDALDR.C | N | 70.85 | 1974.8199 | 19 | 0.3 | 988.4175 | 2 | 44.69 | 1 | 23010 | BuCaMH03.raw | 1.2369E7 | 1 | 1 | 60 | 78 | Carbamidomethylation | C3:Carbamidomethylation:1000.00;C5:Carbamidomethylation:1000.00 | PEAKS DB |
| C.DKTWGHYVDYGC(+57.02)YC(+57.02)GAGGSGTPVDALDR.C | Y | 69.96 | 3076.3079 | 28 | 0.6 | 770.0847 | 4 | 53.88 | 1 | 30769 | BuCaMH03.raw | 7.7241E6 | 2 | 2 | 51 | 78 | Carbamidomethylation | C12:Carbamidomethylation:1000.00;C14:Carbamidomethylation:1000.00 | PEAKS DB |
| C.YVHDNC(+57.02)YGDAEK.I | N | 69.88 | 1469.5881 | 12 | 0.7 | 735.8019 | 2 | 12.57 | 1 | 1878 | BuCaMH03.raw | 7.7179E6 | 2 | 2 | 81 | 92 | Carbamidomethylation | C6:Carbamidomethylation:1000.00 | PEAKS DB |
| T.WGHYVDYGC(+57.02)YC(+57.02)GAGGSGTPVDALDR.C | Y | 68.98 | 2732.1384 | 25 | 0.5 | 911.7206 | 3 | 54.52 | 1 | 31246 | BuCaMH03.raw | 1.6963E7 | 1 | 1 | 54 | 78 | Carbamidomethylation | C9:Carbamidomethylation:1000.00;C11:Carbamidomethylation:1000.00 | PEAKS DB |
| I.IC(+57.02)YGAAGTC(+57.02)AR.I | N | 65.84 | 1198.5223 | 11 | 0.0 | 600.2684 | 2 | 19.82 | 1 | 5420 | BuCaMH03.raw | 1.7918E7 | 1 | 1 | 113 | 123 | Carbamidomethylation | C2:Carbamidomethylation:1000.00;C9:Carbamidomethylation:1000.00 | PEAKS DB |
| L.NLYQFMQM(+15.99)IR.Y | Y | 62.65 | 1358.6475 | 10 | 0.6 | 680.3314 | 2 | 67.92 | 1 | 42635 | BuCaMH03.raw | 9.2925E6 | 1 | 1 | 36 | 45 | Oxidation (M) | M8:Oxidation (M):45.01 | PEAKS DB |
| H.LNLYQFMQM(+15.99)IR.Y | Y | 58.29 | 1471.7316 | 11 | 0.8 | 736.8737 | 2 | 81.39 | 1 | 53949 | BuCaMH03.raw | 2.4076E6 | 2 | 2 | 35 | 45 | Oxidation (M) | M9:Oxidation (M):25.66 | PEAKS DB |
| Y.C(+57.02)GAGGSGTPVDALDR.C | N | 58.21 | 1431.6412 | 15 | 0.0 | 716.8279 | 2 | 32.56 | 1 | 13642 | BuCaMH03.raw | 3.4042E7 | 1 | 1 | 64 | 78 | Carbamidomethylation | C1:Carbamidomethylation:1000.00 | PEAKS DB |
| N.LYQFMQMIR.Y | Y | 58.01 | 1228.6096 | 9 | 0.1 | 615.3121 | 2 | 66.33 | 1 | 41479 | BuCaMH03.raw | 3.6425E7 | 1 | 1 | 37 | 45 |  |  | PEAKS DB |
| L.NLYQFMQMIRYTIPC(+57.02)DK.T | Y | 57.25 | 2220.0530 | 17 | 2.1 | 741.0265 | 3 | 84.76 | 1 | 55874 | BuCaMH03.raw | 4.5928E7 | 1 | 1 | 36 | 52 | Carbamidomethylation | C15:Carbamidomethylation:1000.00 | PEAKS DB |
| R.YTIPC(+57.02)DK.T | N | 54.13 | 895.4109 | 7 | -1.2 | 448.7122 | 2 | 23.30 | 1 | 7704 | BuCaMH03.raw | 4.3871E8 | 1 | 1 | 46 | 52 | Carbamidomethylation | C5:Carbamidomethylation:1000.00 | PEAKS DB |
| total 26 peptides |  |  |  |  |  |  |  |  |  |  |  |  |  |  |  |  |  |  |  |
| --- | --- | --- | --- | --- | --- | --- | --- | --- | --- | --- | --- | --- | --- | --- | --- | --- | --- | --- | --- |

T\_32

back to list

  

| Protein Coverage
| Supporting Peptides
|

Protein Coverage:

Supporting Peptides:

| Peptide | Uniq | -10lgP | Mass | Length | ppm | m/z | z | RT | Fraction | Scan | Source File | Area BuCaMH03\_B17 | #Feature | #Feature BuCaMH03\_B17 | Start | End | PTM | AScore | Found By |
| --- | --- | --- | --- | --- | --- | --- | --- | --- | --- | --- | --- | --- | --- | --- | --- | --- | --- | --- | --- |
| K.TWGEYADYGC(+57.02)YC(+57.02)GAGGSGTPVDALDR.C | N | 107.03 | 2797.1384 | 26 | -0.2 | 1399.5762 | 2 | 64.67 | 1 | 41686 | BuCaMH03.raw | 1.9214E9 | 9 | 9 | 54 | 79 | Carbamidomethylation | C10:Carbamidomethylation:1000.00;C12:Carbamidomethylation:1000.00 | PEAKS DB |
| K.RTIIC(+57.02)YGAAGTC(+57.02)AR.I | N | 98.88 | 1568.7551 | 14 | 0.3 | 785.3851 | 2 | 24.68 | 1 | 8363 | BuCaMH03.raw | 2.3225E7 | 2 | 2 | 111 | 124 | Carbamidomethylation | C5:Carbamidomethylation:1000.00;C12:Carbamidomethylation:1000.00 | PEAKS DB |
| R.C(+57.02)C(+57.02)YVHDNC(+57.02)YADAEKK.H | Y | 92.10 | 1931.7600 | 15 | -0.2 | 966.8871 | 2 | 12.70 | 1 | 1944 | BuCaMH03.raw | 2.6495E8 | 4 | 4 | 80 | 94 | Carbamidomethylation | C1:Carbamidomethylation:1000.00;C2:Carbamidomethylation:1000.00;C8:Carbamidomethylation:1000.00 | PEAKS DB |
| R.TIIC(+57.02)YGAAGTC(+57.02)AR.I | N | 87.88 | 1412.6541 | 13 | 0.2 | 707.3345 | 2 | 33.70 | 1 | 14805 | BuCaMH03.raw | 2.1013E9 | 2 | 2 | 112 | 124 | Carbamidomethylation | C4:Carbamidomethylation:1000.00;C11:Carbamidomethylation:1000.00 | PEAKS DB |
| R.C(+57.02)C(+57.02)YVHDNC(+57.02)YADAEK.K | Y | 85.72 | 1803.6650 | 14 | 0.3 | 902.8401 | 2 | 19.62 | 1 | 5276 | BuCaMH03.raw | 3.3243E8 | 4 | 4 | 80 | 93 | Carbamidomethylation | C1:Carbamidomethylation:1000.00;C2:Carbamidomethylation:1000.00;C8:Carbamidomethylation:1000.00 | PEAKS DB |
| Y.GC(+57.02)YC(+57.02)GAGGSGTPVDALDR.C | N | 81.29 | 1811.7566 | 18 | 1.0 | 906.8865 | 2 | 39.40 | 1 | 18973 | BuCaMH03.raw | 1.6098E6 | 1 | 1 | 62 | 79 | Carbamidomethylation | C2:Carbamidomethylation:1000.00;C4:Carbamidomethylation:1000.00 | PEAKS DB |
| C.YC(+57.02)GAGGSGTPVDALDR.C | N | 81.27 | 1594.7046 | 16 | -0.2 | 798.3594 | 2 | 37.56 | 1 | 17571 | BuCaMH03.raw | 3.935E6 | 1 | 1 | 64 | 79 | Carbamidomethylation | C2:Carbamidomethylation:1000.00 | PEAKS DB |
| T.IIC(+57.02)YGAAGTC(+57.02)AR.I | N | 80.96 | 1311.6063 | 12 | -0.3 | 656.8102 | 2 | 27.05 | 1 | 10166 | BuCaMH03.raw | 1.2342E8 | 1 | 1 | 113 | 124 | Carbamidomethylation | C3:Carbamidomethylation:1000.00;C10:Carbamidomethylation:1000.00 | PEAKS DB |
| L.NLINFMEM(+15.99)IR.Y | N | 78.21 | 1295.6366 | 10 | -0.2 | 648.8254 | 2 | 78.80 | 1 | 54312 | BuCaMH03.raw | 4.9087E8 | 4 | 4 | 37 | 46 | Oxidation (M) | M8:Oxidation (M):52.04 | PEAKS DB |
| L.NLINFM(+15.99)EMIR.Y | N | 74.92 | 1295.6366 | 10 | 0.0 | 648.8256 | 2 | 67.65 | 1 | 42306 | BuCaMH03.raw | 3.5844E8 | 3 | 3 | 37 | 46 | Oxidation (M) | M6:Oxidation (M):32.28 | PEAKS DB |
| G.C(+57.02)YC(+57.02)GAGGSGTPVDALDR.C | N | 74.59 | 1754.7352 | 17 | -0.1 | 878.3748 | 2 | 39.18 | 1 | 18847 | BuCaMH03.raw | 0 | 0 | 0 | 63 | 79 | Carbamidomethylation | C1:Carbamidomethylation:1000.00;C3:Carbamidomethylation:1000.00 | PEAKS DB |
| A.DYGC(+57.02)YC(+57.02)GAGGSGTPVDALDR.C | N | 74.49 | 2089.8469 | 20 | 0.0 | 1045.9308 | 2 | 50.93 | 1 | 28639 | BuCaMH03.raw | 5.2431E7 | 2 | 2 | 60 | 79 | Carbamidomethylation | C4:Carbamidomethylation:1000.00;C6:Carbamidomethylation:1000.00 | PEAKS DB |
| L.NLINFM(+15.99)EM(+15.99)IR.Y | N | 74.44 | 1311.6315 | 10 | 0.7 | 656.8235 | 2 | 83.18 | 1 | 54954 | BuCaMH03.raw | 8.4715E6 | 1 | 1 | 37 | 46 | Oxidation (M) | M6:Oxidation (M):1000.00;M8:Oxidation (M):1000.00 | PEAKS DB |
| L.NLINFMEMIR.Y | N | 73.01 | 1279.6417 | 10 | 0.6 | 640.8285 | 2 | 88.88 | 1 | 59453 | BuCaMH03.raw | 1.4319E10 | 1 | 1 | 37 | 46 |  |  | PEAKS DB |
| D.YGC(+57.02)YC(+57.02)GAGGSGTPVDALDR.C | N | 70.85 | 1974.8199 | 19 | 0.3 | 988.4175 | 2 | 44.69 | 1 | 23010 | BuCaMH03.raw | 1.2369E7 | 1 | 1 | 61 | 79 | Carbamidomethylation | C3:Carbamidomethylation:1000.00;C5:Carbamidomethylation:1000.00 | PEAKS DB |
| W.GEYADYGC(+57.02)YC(+57.02)GAGGSGTPVDALDR.C | N | 68.56 | 2510.0115 | 24 | 1.4 | 1256.0148 | 2 | 53.68 | 1 | 30613 | BuCaMH03.raw | 6.51E5 | 1 | 1 | 56 | 79 | Carbamidomethylation | C8:Carbamidomethylation:1000.00;C10:Carbamidomethylation:1000.00 | PEAKS DB |
| P.LNLINFMEM(+15.99)IR.Y | N | 67.56 | 1408.7207 | 11 | 0.2 | 705.3678 | 2 | 89.30 | 1 | 63361 | BuCaMH03.raw | 3.3651E7 | 3 | 3 | 36 | 46 | Oxidation (M) | M9:Oxidation (M):36.05 | PEAKS DB |
| G.EYADYGC(+57.02)YC(+57.02)GAGGSGTPVDALDR.C | N | 67.26 | 2452.9900 | 23 | 2.3 | 1227.5051 | 2 | 53.86 | 1 | 30821 | BuCaMH03.raw | 0 | 0 | 0 | 57 | 79 | Carbamidomethylation | C7:Carbamidomethylation:1000.00;C9:Carbamidomethylation:1000.00 | PEAKS DB |
| I.IC(+57.02)YGAAGTC(+57.02)AR.I | N | 65.84 | 1198.5223 | 11 | 0.0 | 600.2684 | 2 | 19.82 | 1 | 5420 | BuCaMH03.raw | 1.7918E7 | 1 | 1 | 114 | 124 | Carbamidomethylation | C2:Carbamidomethylation:1000.00;C9:Carbamidomethylation:1000.00 | PEAKS DB |
| P.LNLINFMEMIR.Y | N | 65.28 | 1392.7257 | 11 | -0.1 | 697.3701 | 2 | 93.64 | 1 | 63504 | BuCaMH03.raw | 7.6606E8 | 2 | 2 | 36 | 46 |  |  | PEAKS DB |
| L.NLINFMEMIRYTIPC(+57.02)EK.T | N | 61.55 | 2171.0576 | 17 | 0.4 | 724.6934 | 3 | 87.62 | 1 | 58648 | BuCaMH03.raw | 5.837E7 | 2 | 2 | 37 | 53 | Carbamidomethylation | C15:Carbamidomethylation:1000.00 | PEAKS DB |
| Y.C(+57.02)GAGGSGTPVDALDR.C | N | 58.21 | 1431.6412 | 15 | 0.0 | 716.8279 | 2 | 32.56 | 1 | 13642 | BuCaMH03.raw | 3.4042E7 | 1 | 1 | 65 | 79 | Carbamidomethylation | C1:Carbamidomethylation:1000.00 | PEAKS DB |
| P.LNLINFM(+15.99)EMIR.Y | N | 57.80 | 1408.7207 | 11 | 0.2 | 705.3678 | 2 | 89.30 | 1 | 63002 | BuCaMH03.raw | 1.8584E7 | 1 | 1 | 36 | 46 | Oxidation (M) | M7:Oxidation (M):18.53 | PEAKS DB |
| N.LINFM(+15.99)EMIR.Y | N | 56.49 | 1181.5936 | 9 | 0.4 | 591.8043 | 2 | 64.15 | 1 | 39326 | BuCaMH03.raw | 1.3572E6 | 1 | 1 | 38 | 46 | Oxidation (M) | M5:Oxidation (M):36.05 | PEAKS DB |
| N.LINFMEMIR.Y | N | 56.00 | 1165.5988 | 9 | 0.3 | 583.8068 | 2 | 78.38 | 1 | 51198 | BuCaMH03.raw | 3.6549E8 | 1 | 1 | 38 | 46 |  |  | PEAKS DB |
| C.YVHDNC(+57.02)YADAEK.K | Y | 55.73 | 1483.6038 | 12 | 0.4 | 495.5421 | 3 | 12.59 | 1 | 1893 | BuCaMH03.raw | 3.1811E6 | 2 | 2 | 82 | 93 | Carbamidomethylation | C6:Carbamidomethylation:1000.00 | PEAKS DB |
| N.LINFMEM(+15.99)IR.Y | N | 55.71 | 1181.5936 | 9 | -0.6 | 591.8037 | 2 | 78.37 | 1 | 50945 | BuCaMH03.raw | 5.8354E6 | 2 | 2 | 38 | 46 | Oxidation (M) | M7:Oxidation (M):36.05 | PEAKS DB |
| R.YTIPC(+57.02)EK.T | N | 54.55 | 909.4266 | 7 | 0.4 | 455.7207 | 2 | 24.68 | 1 | 8545 | BuCaMH03.raw | 6.5047E8 | 1 | 1 | 47 | 53 | Carbamidomethylation | C5:Carbamidomethylation:1000.00 | PEAKS DB |
| total 28 peptides |  |  |  |  |  |  |  |  |  |  |  |  |  |  |  |  |  |  |  |
| --- | --- | --- | --- | --- | --- | --- | --- | --- | --- | --- | --- | --- | --- | --- | --- | --- | --- | --- | --- |

T\_39

back to list

  

| Protein Coverage
| Supporting Peptides
|

Protein Coverage:

Supporting Peptides:

| Peptide | Uniq | -10lgP | Mass | Length | ppm | m/z | z | RT | Fraction | Scan | Source File | Area BuCaMH03\_B17 | #Feature | #Feature BuCaMH03\_B17 | Start | End | PTM | AScore | Found By |
| --- | --- | --- | --- | --- | --- | --- | --- | --- | --- | --- | --- | --- | --- | --- | --- | --- | --- | --- | --- |
| R.TAALC(+57.02)FASAPYNEKNFDVVLETHC(+57.02)H | Y | 105.87 | 2893.3164 | 25 | 0.6 | 724.3368 | 4 | 60.61 | 1 | 36301 | BuCaMH03.raw | 2.5498E7 | 2 | 2 | 130 | 154 | Carbamidomethylation | C5:Carbamidomethylation:1000.00;C24:Carbamidomethylation:1000.00 | PEAKS DB |
| P.LDELDTC(+57.02)C(+57.02)QTHANC(+57.02)YAEAR.K | Y | 88.26 | 2325.9412 | 19 | 0.3 | 1163.9781 | 2 | 30.55 | 1 | 12281 | BuCaMH03.raw | 1.3893E6 | 1 | 1 | 72 | 90 | Carbamidomethylation | C7:Carbamidomethylation:1000.00;C8:Carbamidomethylation:1000.00;C14:Carbamidomethylation:1000.00 | PEAKS DB |
| R.TAALC(+57.02)FASAPYNEK.N | Y | 85.72 | 1541.7184 | 14 | 0.1 | 771.8665 | 2 | 47.16 | 1 | 25162 | BuCaMH03.raw | 2.244E9 | 6 | 6 | 130 | 143 | Carbamidomethylation | C5:Carbamidomethylation:1000.00 | PEAKS DB |
| K.NFDVVLETHC(+57.02)H | Y | 84.01 | 1369.6085 | 11 | 0.5 | 685.8119 | 2 | 39.84 | 1 | 19506 | BuCaMH03.raw | 1.8057E9 | 18 | 18 | 144 | 154 | Carbamidomethylation | C10:Carbamidomethylation:1000.00 | PEAKS DB |
| C.DIGNTGTPLDELDTC(+57.02)C(+57.02)QTHANC(+57.02)YAEAR.K | Y | 75.50 | 3081.2861 | 27 | -1.5 | 1028.1011 | 3 | 53.91 | 1 | 30888 | BuCaMH03.raw | 2.1865E6 | 1 | 1 | 64 | 90 | Carbamidomethylation | C15:Carbamidomethylation:1000.00;C16:Carbamidomethylation:1000.00;C22:Carbamidomethylation:1000.00 | PEAKS DB |
| R.KLPEC(+57.02)APYYETYSYTC(+57.02)SGGTVTC(+57.02)SADNEGC(+57.02)AASVC(+57.02)NC(+57.02)DR.T | Y | 74.93 | 4472.7695 | 39 | -0.4 | 1491.9298 | 3 | 51.87 | 1 | 28996 | BuCaMH03.raw | 1.0858E9 | 3 | 3 | 91 | 129 | Carbamidomethylation | C5:Carbamidomethylation:1000.00;C16:Carbamidomethylation:1000.00;C23:Carbamidomethylation:1000.00;C30:Carbamidomethylation:1000.00;C35:Carbamidomethylation:1000.00;C37:Carbamidomethylation:1000.00 | PEAKS DB |
| K.SMVQC(+57.02)TSTRPWLDYADYGC(+57.02)NC(+57.02)DIGNTGTPLDELDTC(+57.02)C(+57.02)QTHANC(+57.02)YAEAR.K | Y | 72.34 | 5646.3042 | 48 | 0.1 | 1412.5835 | 4 | 67.68 | 1 | 42292 | BuCaMH03.raw | 0 | 0 | 0 | 43 | 90 | Carbamidomethylation | C5:Carbamidomethylation:1000.00;C19:Carbamidomethylation:1000.00;C21:Carbamidomethylation:1000.00;C36:Carbamidomethylation:1000.00;C37:Carbamidomethylation:1000.00;C43:Carbamidomethylation:1000.00 | PEAKS DB |
| C.SADNEGC(+57.02)AASVC(+57.02)NC(+57.02)DR.T | Y | 72.28 | 1784.6512 | 16 | 0.0 | 893.3329 | 2 | 15.58 | 1 | 3272 | BuCaMH03.raw | 5.675E6 | 1 | 1 | 114 | 129 | Carbamidomethylation | C7:Carbamidomethylation:1000.00;C12:Carbamidomethylation:1000.00;C14:Carbamidomethylation:1000.00 | PEAKS DB |
| K.SMVQC(+57.02)TSTRPWLD.Y | Y | 69.81 | 1579.7123 | 13 | 0.4 | 790.8638 | 2 | 49.04 | 1 | 26694 | BuCaMH03.raw | 1.8661E6 | 1 | 1 | 43 | 55 | Carbamidomethylation | C5:Carbamidomethylation:1000.00 | PEAKS DB |
| N.C(+57.02)DIGNTGTPLDELDTC(+57.02)C(+57.02)QTHANC(+57.02)YAEAR.K | Y | 68.95 | 3241.3169 | 28 | 0.3 | 1081.4465 | 3 | 51.87 | 1 | 28984 | BuCaMH03.raw | 3.2874E6 | 1 | 1 | 63 | 90 | Carbamidomethylation | C1:Carbamidomethylation:1000.00;C16:Carbamidomethylation:1000.00;C17:Carbamidomethylation:1000.00;C23:Carbamidomethylation:1000.00 | PEAKS DB |
| R.KLPEC(+57.02)APYYETY.S | Y | 68.44 | 1532.6857 | 12 | 0.5 | 767.3505 | 2 | 46.50 | 1 | 24408 | BuCaMH03.raw | 3.0376E8 | 1 | 1 | 91 | 102 | Carbamidomethylation | C5:Carbamidomethylation:1000.00 | PEAKS DB |
| N.FDVVLETHC(+57.02)H | Y | 67.04 | 1255.5656 | 10 | -0.2 | 628.7899 | 2 | 36.64 | 1 | 17140 | BuCaMH03.raw | 1.4532E9 | 3 | 3 | 145 | 154 | Carbamidomethylation | C9:Carbamidomethylation:1000.00 | PEAKS DB |
| K.SMVQC(+57.02)TSTRP.W | Y | 64.72 | 1165.5220 | 10 | 0.5 | 583.7686 | 2 | 16.91 | 1 | 3933 | BuCaMH03.raw | 1.1226E7 | 1 | 1 | 43 | 52 | Carbamidomethylation | C5:Carbamidomethylation:1000.00 | PEAKS DB |
| L.DELDTC(+57.02)C(+57.02)QTHANC(+57.02)YAEAR.K | Y | 64.29 | 2212.8572 | 18 | -0.3 | 1107.4355 | 2 | 25.81 | 1 | 9384 | BuCaMH03.raw | 1.3446E8 | 2 | 2 | 73 | 90 | Carbamidomethylation | C6:Carbamidomethylation:1000.00;C7:Carbamidomethylation:1000.00;C13:Carbamidomethylation:1000.00 | PEAKS DB |
| R.TAALC(+57.02)FASAPYN.E | N | 63.97 | 1284.5808 | 12 | 0.9 | 643.2983 | 2 | 61.35 | 1 | 36882 | BuCaMH03.raw | 4.7091E7 | 1 | 1 | 130 | 141 | Carbamidomethylation | C5:Carbamidomethylation:1000.00 | PEAKS DB |
| A.DYGC(+57.02)NC(+57.02)DIGNTGTPLDELDTC(+57.02)C(+57.02)QTHANC(+57.02)YAEAR.K | Y | 63.34 | 3850.5022 | 33 | -2.4 | 1284.5049 | 3 | 56.35 | 1 | 32763 | BuCaMH03.raw | 1.7314E8 | 1 | 1 | 58 | 90 | Carbamidomethylation | C4:Carbamidomethylation:1000.00;C6:Carbamidomethylation:1000.00;C21:Carbamidomethylation:1000.00;C22:Carbamidomethylation:1000.00;C28:Carbamidomethylation:1000.00 | PEAKS DB |
| K.SMVQC(+57.02)TSTRPWLDYA.D | Y | 61.83 | 1813.8127 | 15 | 0.9 | 907.9145 | 2 | 59.67 | 1 | 35458 | BuCaMH03.raw | 1.4356E7 | 1 | 1 | 43 | 57 | Carbamidomethylation | C5:Carbamidomethylation:1000.00 | PEAKS DB |
| R.KLPEC(+57.02)APYYETYSYTC(+57.02).S | Y | 59.07 | 2043.8594 | 16 | 0.6 | 1022.9376 | 2 | 53.06 | 1 | 30006 | BuCaMH03.raw | 1.4876E7 | 1 | 1 | 91 | 106 | Carbamidomethylation | C5:Carbamidomethylation:1000.00;C16:Carbamidomethylation:1000.00 | PEAKS DB |
| F.DVVLETHC(+57.02)H | Y | 58.95 | 1108.4972 | 9 | 0.2 | 555.2560 | 2 | 18.98 | 1 | 5154 | BuCaMH03.raw | 9.5924E7 | 1 | 1 | 146 | 154 | Carbamidomethylation | C8:Carbamidomethylation:1000.00 | PEAKS DB |
| K.SM(+15.99)VQC(+57.02)TSTRP.W | Y | 57.70 | 1181.5168 | 10 | 0.1 | 591.7657 | 2 | 16.16 | 1 | 4018 | BuCaMH03.raw | 2.6305E6 | 1 | 1 | 43 | 52 | Oxidation (M); Carbamidomethylation | M2:Oxidation (M):1000.00;C5:Carbamidomethylation:1000.00 | PEAKS DB |
| D.RTAALC(+57.02)FASAPYNEK.N | Y | 57.60 | 1697.8195 | 15 | -0.1 | 566.9470 | 3 | 35.73 | 1 | 16103 | BuCaMH03.raw | 3.6281E5 | 1 | 1 | 129 | 143 | Carbamidomethylation | C6:Carbamidomethylation:1000.00 | PEAKS DB |
| A.LC(+57.02)FASAPYNEK.N | Y | 55.94 | 1298.5964 | 11 | -0.7 | 650.3051 | 2 | 39.43 | 1 | 18904 | BuCaMH03.raw | 5.8058E7 | 1 | 1 | 133 | 143 | Carbamidomethylation | C2:Carbamidomethylation:1000.00 | PEAKS DB |
| T.AALC(+57.02)FASAPYNEK.N | Y | 55.31 | 1440.6707 | 13 | 0.4 | 721.3429 | 2 | 44.56 | 1 | 23283 | BuCaMH03.raw | 4.4605E7 | 1 | 1 | 131 | 143 | Carbamidomethylation | C4:Carbamidomethylation:1000.00 | PEAKS DB |
| R.PWLDYADYGC(+57.02)NC(+57.02)DIGNTGTPLDELDTC(+57.02)C(+57.02)QTHANC(+57.02)YAEAR.K | Y | 54.88 | 4595.8457 | 39 | -0.2 | 1532.9556 | 3 | 72.17 | 1 | 46214 | BuCaMH03.raw | 1.6965E8 | 1 | 1 | 52 | 90 | Carbamidomethylation | C10:Carbamidomethylation:1000.00;C12:Carbamidomethylation:1000.00;C27:Carbamidomethylation:1000.00;C28:Carbamidomethylation:1000.00;C34:Carbamidomethylation:1000.00 | PEAKS DB |
| K.NFDVVLETH.C | Y | 53.00 | 1072.5189 | 9 | -0.5 | 537.2665 | 2 | 51.60 | 1 | 28907 | BuCaMH03.raw | 3.8982E6 | 1 | 1 | 144 | 152 |  |  | PEAKS DB |
| total 25 peptides |  |  |  |  |  |  |  |  |  |  |  |  |  |  |  |  |  |  |  |
| --- | --- | --- | --- | --- | --- | --- | --- | --- | --- | --- | --- | --- | --- | --- | --- | --- | --- | --- | --- |

T\_130

back to list

  

| Protein Coverage
| Supporting Peptides
|

Protein Coverage:

Supporting Peptides:

| Peptide | Uniq | -10lgP | Mass | Length | ppm | m/z | z | RT | Fraction | Scan | Source File | Area BuCaMH03\_B17 | #Feature | #Feature BuCaMH03\_B17 | Start | End | PTM | AScore | Found By |
| --- | --- | --- | --- | --- | --- | --- | --- | --- | --- | --- | --- | --- | --- | --- | --- | --- | --- | --- | --- |
| K.HVVVVGAGMAGLSAAYVLSQAGHR.V | N | 97.43 | 2349.2375 | 24 | 0.6 | 784.0869 | 3 | 63.13 | 1 | 38339 | BuCaMH03.raw | 1.3603E7 | 2 | 2 | 90 | 113 |  |  | PEAKS DB |
| K.SMHQAIAEIVHLNAQVTK.I | N | 92.86 | 1989.0465 | 18 | 1.1 | 664.0235 | 3 | 50.27 | 1 | 27750 | BuCaMH03.raw | 6.3424E6 | 3 | 3 | 301 | 318 |  |  | PEAKS DB |
| R.EADYEEFLEIAR.N | N | 91.88 | 1483.6830 | 12 | 0.7 | 742.8493 | 2 | 71.75 | 1 | 45615 | BuCaMH03.raw | 5.1224E6 | 1 | 1 | 68 | 79 |  |  | PEAKS DB |
| Y.VLADDSDFFQALDIK.T | N | 88.26 | 1695.8354 | 15 | -0.1 | 848.9249 | 2 | 81.35 | 1 | 53415 | BuCaMH03.raw | 5.554E6 | 1 | 1 | 428 | 442 |  |  | PEAKS DB |
| E.ADYEEFLEIAR.N | N | 84.13 | 1354.6404 | 11 | 0.7 | 678.3279 | 2 | 68.97 | 1 | 43275 | BuCaMH03.raw | 7.4529E6 | 1 | 1 | 69 | 79 |  |  | PEAKS DB |
| K.TSADIVINDLSLIHQLPK.N | N | 82.55 | 1976.0942 | 18 | 0.9 | 659.7059 | 3 | 72.99 | 1 | 46603 | BuCaMH03.raw | 7.9369E6 | 2 | 2 | 443 | 460 |  |  | PEAKS DB |
| T.SFTPYQFQDYSETFAAPVGR.I | Y | 79.76 | 2310.0593 | 20 | -1.5 | 1156.0352 | 2 | 73.49 | 1 | 47054 | BuCaMH03.raw | 1.8E6 | 1 | 1 | 487 | 506 |  |  | PEAKS DB |
| K.LNEFFQENENAWYFIR.N | N | 75.72 | 2118.9800 | 16 | 0.4 | 1060.4977 | 2 | 82.64 | 1 | 54427 | BuCaMH03.raw | 3.5944E6 | 1 | 1 | 164 | 179 |  |  | PEAKS DB |
| L.NEFFQENENAWYFIR.N | N | 69.66 | 2005.8959 | 15 | 0.5 | 1003.9557 | 2 | 79.06 | 1 | 51465 | BuCaMH03.raw | 5.1838E6 | 1 | 1 | 165 | 179 |  |  | PEAKS DB |
| K.RFDEIIDGFDQLPK.S | N | 69.25 | 1691.8518 | 14 | -0.2 | 564.9578 | 3 | 66.71 | 1 | 41409 | BuCaMH03.raw | 2.6804E6 | 2 | 2 | 287 | 300 |  |  | PEAKS DB |
| K.EGWYVNMGPMR.L | N | 68.49 | 1338.5850 | 11 | 0.8 | 670.3003 | 2 | 58.96 | 1 | 34889 | BuCaMH03.raw | 1.2121E7 | 1 | 1 | 135 | 145 |  |  | PEAKS DB |
| K.NEIQDLC(+57.02)YPSLIK.K | N | 68.00 | 1591.7915 | 13 | 0.4 | 796.9034 | 2 | 62.11 | 1 | 37547 | BuCaMH03.raw | 7.0247E6 | 1 | 1 | 461 | 473 | Carbamidomethylation | C7:Carbamidomethylation:1000.00 | PEAKS DB |
| D.YEEFLEIAR.N | N | 66.92 | 1168.5764 | 9 | 2.1 | 585.2967 | 2 | 56.08 | 1 | 32599 | BuCaMH03.raw | 1.8928E6 | 1 | 1 | 71 | 79 |  |  | PEAKS DB |
| R.SPLEEC(+57.02)FR.E | N | 65.33 | 1036.4647 | 8 | 0.1 | 519.2397 | 2 | 34.43 | 1 | 15058 | BuCaMH03.raw | 2.9904E6 | 1 | 1 | 60 | 67 | Carbamidomethylation | C6:Carbamidomethylation:1000.00 | PEAKS DB |
| N.EFFQENENAWYFIR.N | N | 65.02 | 1891.8529 | 14 | -1.7 | 946.9321 | 2 | 79.09 | 1 | 51607 | BuCaMH03.raw | 9.4924E5 | 1 | 1 | 166 | 179 |  |  | PEAKS DB |
| R.ISFEPPLPPK.K | N | 64.81 | 1123.6277 | 10 | 0.4 | 562.8214 | 2 | 55.18 | 1 | 32023 | BuCaMH03.raw | 2.0577E6 | 1 | 1 | 357 | 366 |  |  | PEAKS DB |
| R.VHGWLDSTIK.S | N | 62.75 | 1154.6084 | 10 | -0.1 | 578.3114 | 2 | 36.99 | 1 | 17052 | BuCaMH03.raw | 2.2229E6 | 2 | 2 | 517 | 526 |  |  | PEAKS DB |
| A.DYEEFLEIAR.N | N | 61.24 | 1283.6033 | 10 | 1.2 | 642.8097 | 2 | 71.33 | 1 | 45329 | BuCaMH03.raw | 4.864E5 | 1 | 1 | 70 | 79 |  |  | PEAKS DB |
| R.FDEIIDGFDQLPK.S | N | 61.23 | 1535.7507 | 13 | 0.7 | 768.8832 | 2 | 72.44 | 1 | 46158 | BuCaMH03.raw | 1.0313E7 | 1 | 1 | 288 | 300 |  |  | PEAKS DB |
| K.TLSYVTADYVIVC(+57.02)ATSR.A | N | 60.60 | 1917.9506 | 17 | 0.5 | 959.9830 | 2 | 69.81 | 1 | 44066 | BuCaMH03.raw | 2.4641E6 | 2 | 2 | 336 | 352 | Carbamidomethylation | C13:Carbamidomethylation:1000.00 | PEAKS DB |
| F.QENENAWYFIR.N | N | 60.27 | 1468.6735 | 11 | 0.6 | 735.3445 | 2 | 60.74 | 1 | 36464 | BuCaMH03.raw | 5.201E5 | 1 | 1 | 169 | 179 |  |  | PEAKS DB |
| K.WSLDKYTMGALTSFTPYQFQDYSETFAAPVGR.I | Y | 59.97 | 3676.7183 | 32 | -0.6 | 1226.5793 | 3 | 91.66 | 1 | 61646 | BuCaMH03.raw | 1.7061E6 | 1 | 1 | 475 | 506 |  |  | PEAKS DB |
| K.FWEADGIHGGK.S | N | 53.59 | 1215.5673 | 11 | 0.0 | 406.1964 | 3 | 29.75 | 1 | 11639 | BuCaMH03.raw | 3.2139E6 | 1 | 1 | 390 | 400 |  |  | PEAKS DB |
| total 23 peptides |  |  |  |  |  |  |  |  |  |  |  |  |  |  |  |  |  |  |  |
| --- | --- | --- | --- | --- | --- | --- | --- | --- | --- | --- | --- | --- | --- | --- | --- | --- | --- | --- | --- |

T\_131

back to list

  

| Protein Coverage
| Supporting Peptides
|

Protein Coverage:

Supporting Peptides:

| Peptide | Uniq | -10lgP | Mass | Length | ppm | m/z | z | RT | Fraction | Scan | Source File | Area BuCaMH03\_B17 | #Feature | #Feature BuCaMH03\_B17 | Start | End | PTM | AScore | Found By |
| --- | --- | --- | --- | --- | --- | --- | --- | --- | --- | --- | --- | --- | --- | --- | --- | --- | --- | --- | --- |
| K.HVVVVGAGMAGLSAAYVLSQAGHR.V | N | 97.43 | 2349.2375 | 24 | 0.6 | 784.0869 | 3 | 63.13 | 1 | 38339 | BuCaMH03.raw | 1.3603E7 | 2 | 2 | 90 | 113 |  |  | PEAKS DB |
| K.SMHQAIAEIVHLNAQVTK.I | N | 92.86 | 1989.0465 | 18 | 1.1 | 664.0235 | 3 | 50.27 | 1 | 27750 | BuCaMH03.raw | 6.3424E6 | 3 | 3 | 301 | 318 |  |  | PEAKS DB |
| R.EADYEEFLEIAR.N | N | 91.88 | 1483.6830 | 12 | 0.7 | 742.8493 | 2 | 71.75 | 1 | 45615 | BuCaMH03.raw | 5.1224E6 | 1 | 1 | 68 | 79 |  |  | PEAKS DB |
| Y.VLADDSDFFQALDIK.T | N | 88.26 | 1695.8354 | 15 | -0.1 | 848.9249 | 2 | 81.35 | 1 | 53415 | BuCaMH03.raw | 5.554E6 | 1 | 1 | 428 | 442 |  |  | PEAKS DB |
| E.ADYEEFLEIAR.N | N | 84.13 | 1354.6404 | 11 | 0.7 | 678.3279 | 2 | 68.97 | 1 | 43275 | BuCaMH03.raw | 7.4529E6 | 1 | 1 | 69 | 79 |  |  | PEAKS DB |
| K.TSADIVINDLSLIHQLPK.N | N | 82.55 | 1976.0942 | 18 | 0.9 | 659.7059 | 3 | 72.99 | 1 | 46603 | BuCaMH03.raw | 7.9369E6 | 2 | 2 | 443 | 460 |  |  | PEAKS DB |
| T.SFTPYQFQDYSETFAAPVGR.I | Y | 79.76 | 2310.0593 | 20 | -1.5 | 1156.0352 | 2 | 73.49 | 1 | 47054 | BuCaMH03.raw | 1.8E6 | 1 | 1 | 487 | 506 |  |  | PEAKS DB |
| K.LNEFFQENENAWYFIR.N | N | 75.72 | 2118.9800 | 16 | 0.4 | 1060.4977 | 2 | 82.64 | 1 | 54427 | BuCaMH03.raw | 3.5944E6 | 1 | 1 | 164 | 179 |  |  | PEAKS DB |
| L.NEFFQENENAWYFIR.N | N | 69.66 | 2005.8959 | 15 | 0.5 | 1003.9557 | 2 | 79.06 | 1 | 51465 | BuCaMH03.raw | 5.1838E6 | 1 | 1 | 165 | 179 |  |  | PEAKS DB |
| K.RFDEIIDGFDQLPK.S | N | 69.25 | 1691.8518 | 14 | -0.2 | 564.9578 | 3 | 66.71 | 1 | 41409 | BuCaMH03.raw | 2.6804E6 | 2 | 2 | 287 | 300 |  |  | PEAKS DB |
| K.EGWYVNMGPMR.L | N | 68.49 | 1338.5850 | 11 | 0.8 | 670.3003 | 2 | 58.96 | 1 | 34889 | BuCaMH03.raw | 1.2121E7 | 1 | 1 | 135 | 145 |  |  | PEAKS DB |
| K.NEIQDLC(+57.02)YPSLIK.K | N | 68.00 | 1591.7915 | 13 | 0.4 | 796.9034 | 2 | 62.11 | 1 | 37547 | BuCaMH03.raw | 7.0247E6 | 1 | 1 | 461 | 473 | Carbamidomethylation | C7:Carbamidomethylation:1000.00 | PEAKS DB |
| D.YEEFLEIAR.N | N | 66.92 | 1168.5764 | 9 | 2.1 | 585.2967 | 2 | 56.08 | 1 | 32599 | BuCaMH03.raw | 1.8928E6 | 1 | 1 | 71 | 79 |  |  | PEAKS DB |
| R.SPLEEC(+57.02)FR.E | N | 65.33 | 1036.4647 | 8 | 0.1 | 519.2397 | 2 | 34.43 | 1 | 15058 | BuCaMH03.raw | 2.9904E6 | 1 | 1 | 60 | 67 | Carbamidomethylation | C6:Carbamidomethylation:1000.00 | PEAKS DB |
| N.EFFQENENAWYFIR.N | N | 65.02 | 1891.8529 | 14 | -1.7 | 946.9321 | 2 | 79.09 | 1 | 51607 | BuCaMH03.raw | 9.4924E5 | 1 | 1 | 166 | 179 |  |  | PEAKS DB |
| R.ISFEPPLPPK.K | N | 64.81 | 1123.6277 | 10 | 0.4 | 562.8214 | 2 | 55.18 | 1 | 32023 | BuCaMH03.raw | 2.0577E6 | 1 | 1 | 357 | 366 |  |  | PEAKS DB |
| R.VHGWLDSTIK.S | N | 62.75 | 1154.6084 | 10 | -0.1 | 578.3114 | 2 | 36.99 | 1 | 17052 | BuCaMH03.raw | 2.2229E6 | 2 | 2 | 517 | 526 |  |  | PEAKS DB |
| A.DYEEFLEIAR.N | N | 61.24 | 1283.6033 | 10 | 1.2 | 642.8097 | 2 | 71.33 | 1 | 45329 | BuCaMH03.raw | 4.864E5 | 1 | 1 | 70 | 79 |  |  | PEAKS DB |
| R.FDEIIDGFDQLPK.S | N | 61.23 | 1535.7507 | 13 | 0.7 | 768.8832 | 2 | 72.44 | 1 | 46158 | BuCaMH03.raw | 1.0313E7 | 1 | 1 | 288 | 300 |  |  | PEAKS DB |
| K.TLSYVTADYVIVC(+57.02)ATSR.A | N | 60.60 | 1917.9506 | 17 | 0.5 | 959.9830 | 2 | 69.81 | 1 | 44066 | BuCaMH03.raw | 2.4641E6 | 2 | 2 | 336 | 352 | Carbamidomethylation | C13:Carbamidomethylation:1000.00 | PEAKS DB |
| F.QENENAWYFIR.N | N | 60.27 | 1468.6735 | 11 | 0.6 | 735.3445 | 2 | 60.74 | 1 | 36464 | BuCaMH03.raw | 5.201E5 | 1 | 1 | 169 | 179 |  |  | PEAKS DB |
| K.WSLDKYTMGALTSFTPYQFQDYSETFAAPVGR.I | Y | 59.97 | 3676.7183 | 32 | -0.6 | 1226.5793 | 3 | 91.66 | 1 | 61646 | BuCaMH03.raw | 1.7061E6 | 1 | 1 | 475 | 506 |  |  | PEAKS DB |
| K.FWEADGIHGGK.S | N | 53.59 | 1215.5673 | 11 | 0.0 | 406.1964 | 3 | 29.75 | 1 | 11639 | BuCaMH03.raw | 3.2139E6 | 1 | 1 | 390 | 400 |  |  | PEAKS DB |
| total 23 peptides |  |  |  |  |  |  |  |  |  |  |  |  |  |  |  |  |  |  |  |
| --- | --- | --- | --- | --- | --- | --- | --- | --- | --- | --- | --- | --- | --- | --- | --- | --- | --- | --- | --- |

T\_128

back to list

  

| Protein Coverage
| Supporting Peptides
|

Protein Coverage:

Supporting Peptides:

| Peptide | Uniq | -10lgP | Mass | Length | ppm | m/z | z | RT | Fraction | Scan | Source File | Area BuCaMH03\_B17 | #Feature | #Feature BuCaMH03\_B17 | Start | End | PTM | AScore | Found By |
| --- | --- | --- | --- | --- | --- | --- | --- | --- | --- | --- | --- | --- | --- | --- | --- | --- | --- | --- | --- |
| K.HVVVVGAGMAGLSAAYVLSQAGHR.V | N | 97.43 | 2349.2375 | 24 | 0.6 | 784.0869 | 3 | 63.13 | 1 | 38339 | BuCaMH03.raw | 1.3603E7 | 2 | 2 | 90 | 113 |  |  | PEAKS DB |
| K.SMHQAIAEIVHLNAQVTK.I | N | 92.86 | 1989.0465 | 18 | 1.1 | 664.0235 | 3 | 50.27 | 1 | 27750 | BuCaMH03.raw | 6.3424E6 | 3 | 3 | 301 | 318 |  |  | PEAKS DB |
| R.EADYEEFLEIAR.N | N | 91.88 | 1483.6830 | 12 | 0.7 | 742.8493 | 2 | 71.75 | 1 | 45615 | BuCaMH03.raw | 5.1224E6 | 1 | 1 | 68 | 79 |  |  | PEAKS DB |
| Y.VLADDSDFFQALDIK.T | N | 88.26 | 1695.8354 | 15 | -0.1 | 848.9249 | 2 | 81.35 | 1 | 53415 | BuCaMH03.raw | 5.554E6 | 1 | 1 | 428 | 442 |  |  | PEAKS DB |
| E.ADYEEFLEIAR.N | N | 84.13 | 1354.6404 | 11 | 0.7 | 678.3279 | 2 | 68.97 | 1 | 43275 | BuCaMH03.raw | 7.4529E6 | 1 | 1 | 69 | 79 |  |  | PEAKS DB |
| K.TSADIVINDLSLIHQLPK.N | N | 82.55 | 1976.0942 | 18 | 0.9 | 659.7059 | 3 | 72.99 | 1 | 46603 | BuCaMH03.raw | 7.9369E6 | 2 | 2 | 443 | 460 |  |  | PEAKS DB |
| K.LNEFFQENENAWYFIR.N | N | 75.72 | 2118.9800 | 16 | 0.4 | 1060.4977 | 2 | 82.64 | 1 | 54427 | BuCaMH03.raw | 3.5944E6 | 1 | 1 | 164 | 179 |  |  | PEAKS DB |
| L.NEFFQENENAWYFIR.N | N | 69.66 | 2005.8959 | 15 | 0.5 | 1003.9557 | 2 | 79.06 | 1 | 51465 | BuCaMH03.raw | 5.1838E6 | 1 | 1 | 165 | 179 |  |  | PEAKS DB |
| K.RFDEIIDGFDQLPK.S | N | 69.25 | 1691.8518 | 14 | -0.2 | 564.9578 | 3 | 66.71 | 1 | 41409 | BuCaMH03.raw | 2.6804E6 | 2 | 2 | 287 | 300 |  |  | PEAKS DB |
| K.EGWYVNMGPMR.L | N | 68.49 | 1338.5850 | 11 | 0.8 | 670.3003 | 2 | 58.96 | 1 | 34889 | BuCaMH03.raw | 1.2121E7 | 1 | 1 | 135 | 145 |  |  | PEAKS DB |
| K.NEIQDLC(+57.02)YPSLIK.K | N | 68.00 | 1591.7915 | 13 | 0.4 | 796.9034 | 2 | 62.11 | 1 | 37547 | BuCaMH03.raw | 7.0247E6 | 1 | 1 | 461 | 473 | Carbamidomethylation | C7:Carbamidomethylation:1000.00 | PEAKS DB |
| D.YEEFLEIAR.N | N | 66.92 | 1168.5764 | 9 | 2.1 | 585.2967 | 2 | 56.08 | 1 | 32599 | BuCaMH03.raw | 1.8928E6 | 1 | 1 | 71 | 79 |  |  | PEAKS DB |
| R.SPLEEC(+57.02)FR.E | N | 65.33 | 1036.4647 | 8 | 0.1 | 519.2397 | 2 | 34.43 | 1 | 15058 | BuCaMH03.raw | 2.9904E6 | 1 | 1 | 60 | 67 | Carbamidomethylation | C6:Carbamidomethylation:1000.00 | PEAKS DB |
| N.EFFQENENAWYFIR.N | N | 65.02 | 1891.8529 | 14 | -1.7 | 946.9321 | 2 | 79.09 | 1 | 51607 | BuCaMH03.raw | 9.4924E5 | 1 | 1 | 166 | 179 |  |  | PEAKS DB |
| R.ISFEPPLPPK.K | N | 64.81 | 1123.6277 | 10 | 0.4 | 562.8214 | 2 | 55.18 | 1 | 32023 | BuCaMH03.raw | 2.0577E6 | 1 | 1 | 357 | 366 |  |  | PEAKS DB |
| R.VHGWLDSTIK.S | N | 62.75 | 1154.6084 | 10 | -0.1 | 578.3114 | 2 | 36.99 | 1 | 17052 | BuCaMH03.raw | 2.2229E6 | 2 | 2 | 517 | 526 |  |  | PEAKS DB |
| K.YTMGALTSFTPYQFQDYIETVAAPVGR.V | Y | 61.65 | 3025.4531 | 27 | 0.4 | 1513.7345 | 2 | 98.03 | 1 | 66720 | BuCaMH03.raw | 9.3511E5 | 1 | 1 | 480 | 506 |  |  | PEAKS DB |
| A.DYEEFLEIAR.N | N | 61.24 | 1283.6033 | 10 | 1.2 | 642.8097 | 2 | 71.33 | 1 | 45329 | BuCaMH03.raw | 4.864E5 | 1 | 1 | 70 | 79 |  |  | PEAKS DB |
| R.FDEIIDGFDQLPK.S | N | 61.23 | 1535.7507 | 13 | 0.7 | 768.8832 | 2 | 72.44 | 1 | 46158 | BuCaMH03.raw | 1.0313E7 | 1 | 1 | 288 | 300 |  |  | PEAKS DB |
| K.TLSYVTADYVIVC(+57.02)ATSR.A | N | 60.60 | 1917.9506 | 17 | 0.5 | 959.9830 | 2 | 69.81 | 1 | 44066 | BuCaMH03.raw | 2.4641E6 | 2 | 2 | 336 | 352 | Carbamidomethylation | C13:Carbamidomethylation:1000.00 | PEAKS DB |
| F.QENENAWYFIR.N | N | 60.27 | 1468.6735 | 11 | 0.6 | 735.3445 | 2 | 60.74 | 1 | 36464 | BuCaMH03.raw | 5.201E5 | 1 | 1 | 169 | 179 |  |  | PEAKS DB |
| K.FWEADGIHGGK.S | N | 53.59 | 1215.5673 | 11 | 0.0 | 406.1964 | 3 | 29.75 | 1 | 11639 | BuCaMH03.raw | 3.2139E6 | 1 | 1 | 390 | 400 |  |  | PEAKS DB |
| total 22 peptides |  |  |  |  |  |  |  |  |  |  |  |  |  |  |  |  |  |  |  |
| --- | --- | --- | --- | --- | --- | --- | --- | --- | --- | --- | --- | --- | --- | --- | --- | --- | --- | --- | --- |

T\_129

back to list

  

| Protein Coverage
| Supporting Peptides
|

Protein Coverage:

Supporting Peptides:

| Peptide | Uniq | -10lgP | Mass | Length | ppm | m/z | z | RT | Fraction | Scan | Source File | Area BuCaMH03\_B17 | #Feature | #Feature BuCaMH03\_B17 | Start | End | PTM | AScore | Found By |
| --- | --- | --- | --- | --- | --- | --- | --- | --- | --- | --- | --- | --- | --- | --- | --- | --- | --- | --- | --- |
| K.HVVVVGAGMAGLSAAYVLSQAGHR.V | N | 97.43 | 2349.2375 | 24 | 0.6 | 784.0869 | 3 | 63.13 | 1 | 38339 | BuCaMH03.raw | 1.3603E7 | 2 | 2 | 90 | 113 |  |  | PEAKS DB |
| K.SMHQAIAEIVHLNAQVTK.I | N | 92.86 | 1989.0465 | 18 | 1.1 | 664.0235 | 3 | 50.27 | 1 | 27750 | BuCaMH03.raw | 6.3424E6 | 3 | 3 | 301 | 318 |  |  | PEAKS DB |
| R.EADYEEFLEIAR.N | N | 91.88 | 1483.6830 | 12 | 0.7 | 742.8493 | 2 | 71.75 | 1 | 45615 | BuCaMH03.raw | 5.1224E6 | 1 | 1 | 68 | 79 |  |  | PEAKS DB |
| Y.VLADDSDFFQALDIK.T | N | 88.26 | 1695.8354 | 15 | -0.1 | 848.9249 | 2 | 81.35 | 1 | 53415 | BuCaMH03.raw | 5.554E6 | 1 | 1 | 428 | 442 |  |  | PEAKS DB |
| E.ADYEEFLEIAR.N | N | 84.13 | 1354.6404 | 11 | 0.7 | 678.3279 | 2 | 68.97 | 1 | 43275 | BuCaMH03.raw | 7.4529E6 | 1 | 1 | 69 | 79 |  |  | PEAKS DB |
| K.TSADIVINDLSLIHQLPK.N | N | 82.55 | 1976.0942 | 18 | 0.9 | 659.7059 | 3 | 72.99 | 1 | 46603 | BuCaMH03.raw | 7.9369E6 | 2 | 2 | 443 | 460 |  |  | PEAKS DB |
| K.LNEFFQENENAWYFIR.N | N | 75.72 | 2118.9800 | 16 | 0.4 | 1060.4977 | 2 | 82.64 | 1 | 54427 | BuCaMH03.raw | 3.5944E6 | 1 | 1 | 164 | 179 |  |  | PEAKS DB |
| L.NEFFQENENAWYFIR.N | N | 69.66 | 2005.8959 | 15 | 0.5 | 1003.9557 | 2 | 79.06 | 1 | 51465 | BuCaMH03.raw | 5.1838E6 | 1 | 1 | 165 | 179 |  |  | PEAKS DB |
| K.RFDEIIDGFDQLPK.S | N | 69.25 | 1691.8518 | 14 | -0.2 | 564.9578 | 3 | 66.71 | 1 | 41409 | BuCaMH03.raw | 2.6804E6 | 2 | 2 | 287 | 300 |  |  | PEAKS DB |
| K.EGWYVNMGPMR.L | N | 68.49 | 1338.5850 | 11 | 0.8 | 670.3003 | 2 | 58.96 | 1 | 34889 | BuCaMH03.raw | 1.2121E7 | 1 | 1 | 135 | 145 |  |  | PEAKS DB |
| K.NEIQDLC(+57.02)YPSLIK.K | N | 68.00 | 1591.7915 | 13 | 0.4 | 796.9034 | 2 | 62.11 | 1 | 37547 | BuCaMH03.raw | 7.0247E6 | 1 | 1 | 461 | 473 | Carbamidomethylation | C7:Carbamidomethylation:1000.00 | PEAKS DB |
| D.YEEFLEIAR.N | N | 66.92 | 1168.5764 | 9 | 2.1 | 585.2967 | 2 | 56.08 | 1 | 32599 | BuCaMH03.raw | 1.8928E6 | 1 | 1 | 71 | 79 |  |  | PEAKS DB |
| R.SPLEEC(+57.02)FR.E | N | 65.33 | 1036.4647 | 8 | 0.1 | 519.2397 | 2 | 34.43 | 1 | 15058 | BuCaMH03.raw | 2.9904E6 | 1 | 1 | 60 | 67 | Carbamidomethylation | C6:Carbamidomethylation:1000.00 | PEAKS DB |
| N.EFFQENENAWYFIR.N | N | 65.02 | 1891.8529 | 14 | -1.7 | 946.9321 | 2 | 79.09 | 1 | 51607 | BuCaMH03.raw | 9.4924E5 | 1 | 1 | 166 | 179 |  |  | PEAKS DB |
| R.ISFEPPLPPK.K | N | 64.81 | 1123.6277 | 10 | 0.4 | 562.8214 | 2 | 55.18 | 1 | 32023 | BuCaMH03.raw | 2.0577E6 | 1 | 1 | 357 | 366 |  |  | PEAKS DB |
| R.VHGWLDSTIK.S | N | 62.75 | 1154.6084 | 10 | -0.1 | 578.3114 | 2 | 36.99 | 1 | 17052 | BuCaMH03.raw | 2.2229E6 | 2 | 2 | 517 | 526 |  |  | PEAKS DB |
| K.YTMGALTSFTPYQFQDYIETVAAPVGR.V | Y | 61.65 | 3025.4531 | 27 | 0.4 | 1513.7345 | 2 | 98.03 | 1 | 66720 | BuCaMH03.raw | 9.3511E5 | 1 | 1 | 480 | 506 |  |  | PEAKS DB |
| A.DYEEFLEIAR.N | N | 61.24 | 1283.6033 | 10 | 1.2 | 642.8097 | 2 | 71.33 | 1 | 45329 | BuCaMH03.raw | 4.864E5 | 1 | 1 | 70 | 79 |  |  | PEAKS DB |
| R.FDEIIDGFDQLPK.S | N | 61.23 | 1535.7507 | 13 | 0.7 | 768.8832 | 2 | 72.44 | 1 | 46158 | BuCaMH03.raw | 1.0313E7 | 1 | 1 | 288 | 300 |  |  | PEAKS DB |
| K.TLSYVTADYVIVC(+57.02)ATSR.A | N | 60.60 | 1917.9506 | 17 | 0.5 | 959.9830 | 2 | 69.81 | 1 | 44066 | BuCaMH03.raw | 2.4641E6 | 2 | 2 | 336 | 352 | Carbamidomethylation | C13:Carbamidomethylation:1000.00 | PEAKS DB |
| F.QENENAWYFIR.N | N | 60.27 | 1468.6735 | 11 | 0.6 | 735.3445 | 2 | 60.74 | 1 | 36464 | BuCaMH03.raw | 5.201E5 | 1 | 1 | 169 | 179 |  |  | PEAKS DB |
| K.FWEADGIHGGK.S | N | 53.59 | 1215.5673 | 11 | 0.0 | 406.1964 | 3 | 29.75 | 1 | 11639 | BuCaMH03.raw | 3.2139E6 | 1 | 1 | 390 | 400 |  |  | PEAKS DB |
| total 22 peptides |  |  |  |  |  |  |  |  |  |  |  |  |  |  |  |  |  |  |  |
| --- | --- | --- | --- | --- | --- | --- | --- | --- | --- | --- | --- | --- | --- | --- | --- | --- | --- | --- | --- |

T\_6

back to list

  

| Protein Coverage
| Supporting Peptides
|

Protein Coverage:

Supporting Peptides:

| Peptide | Uniq | -10lgP | Mass | Length | ppm | m/z | z | RT | Fraction | Scan | Source File | Area BuCaMH03\_B17 | #Feature | #Feature BuCaMH03\_B17 | Start | End | PTM | AScore | Found By |
| --- | --- | --- | --- | --- | --- | --- | --- | --- | --- | --- | --- | --- | --- | --- | --- | --- | --- | --- | --- |
| K.VYGLVNHLNMIYK.T | Y | 95.55 | 1562.8279 | 13 | 0.2 | 782.4214 | 2 | 53.03 | 1 | 29989 | BuCaMH03.raw | 1.7353E7 | 2 | 2 | 232 | 244 |  |  | PEAKS DB |
| K.YIELYMAVDNR.M | N | 86.42 | 1385.6649 | 11 | 0.7 | 693.8402 | 2 | 60.14 | 1 | 37661 | BuCaMH03.raw | 2.1755E8 | 18 | 18 | 206 | 216 |  |  | PEAKS DB |
| C.LMYESISDEPHSEFSSC(+57.02)SVQEHQR.Y | N | 78.99 | 2881.2283 | 24 | -0.2 | 961.4165 | 3 | 39.09 | 1 | 18706 | BuCaMH03.raw | 1.8302E7 | 2 | 2 | 364 | 387 | Carbamidomethylation | C17:Carbamidomethylation:1000.00 | PEAKS DB |
| L.MYESISDEPHSEFSSC(+57.02)SVQEHQR.Y | N | 75.75 | 2768.1443 | 23 | -0.6 | 693.0429 | 4 | 32.28 | 1 | 13474 | BuCaMH03.raw | 2.6278E7 | 2 | 2 | 365 | 387 | Carbamidomethylation | C16:Carbamidomethylation:1000.00 | PEAKS DB |
| K.KSVAVVQDYSK.R | N | 75.23 | 1222.6558 | 11 | -0.2 | 612.3350 | 2 | 15.59 | 1 | 3167 | BuCaMH03.raw | 4.4932E6 | 2 | 2 | 320 | 330 |  |  | PEAKS DB |
| K.YIELYM(+15.99)AVDNR.M | N | 72.96 | 1401.6598 | 11 | -0.6 | 701.8367 | 2 | 49.91 | 1 | 27539 | BuCaMH03.raw | 2.5137E6 | 2 | 2 | 206 | 216 | Oxidation (M) | M6:Oxidation (M):1000.00 | PEAKS DB |
| R.NSC(+57.02)FTLNQR.G | N | 71.24 | 1138.5189 | 9 | -0.7 | 570.2664 | 2 | 27.05 | 1 | 9901 | BuCaMH03.raw | 2.443E7 | 1 | 1 | 531 | 539 | Carbamidomethylation | C3:Carbamidomethylation:1000.00 | PEAKS DB |
| K.C(+57.02)PTLTNQC(+57.02)IALMGPNVK.A | N | 70.69 | 1915.9318 | 17 | 0.6 | 639.6516 | 3 | 55.90 | 1 | 32502 | BuCaMH03.raw | 1.0403E6 | 1 | 1 | 511 | 527 | Carbamidomethylation | C1:Carbamidomethylation:1000.00;C8:Carbamidomethylation:1000.00 | PEAKS DB |
| V.C(+57.02)NC(+57.02)LILPDDPNYGMVETGTK.C | N | 67.70 | 2296.0173 | 20 | -0.4 | 1149.0155 | 2 | 62.88 | 1 | 38333 | BuCaMH03.raw | 1.54E6 | 1 | 1 | 576 | 595 | Carbamidomethylation | C1:Carbamidomethylation:1000.00;C3:Carbamidomethylation:1000.00 | PEAKS DB |
| Y.IELYMAVDNR.M | N | 65.94 | 1222.6016 | 10 | 0.3 | 612.3082 | 2 | 51.87 | 1 | 29293 | BuCaMH03.raw | 6.9266E7 | 1 | 1 | 207 | 216 |  |  | PEAKS DB |
| P.TLTNQC(+57.02)IALMGPNVK.A | N | 64.79 | 1658.8484 | 15 | 1.0 | 830.4323 | 2 | 51.45 | 1 | 28756 | BuCaMH03.raw | 1.0501E6 | 1 | 1 | 513 | 527 | Carbamidomethylation | C6:Carbamidomethylation:1000.00 | PEAKS DB |
| V.YGLVNHLNMIYK.T | Y | 62.22 | 1463.7595 | 12 | 0.0 | 732.8870 | 2 | 48.33 | 1 | 26042 | BuCaMH03.raw | 1.7972E6 | 1 | 1 | 233 | 244 |  |  | PEAKS DB |
| M.YESISDEPHSEFSSC(+57.02)SVQEHQR.Y | N | 60.65 | 2637.1038 | 22 | 0.9 | 660.2838 | 4 | 28.14 | 1 | 10623 | BuCaMH03.raw | 8.8375E5 | 1 | 1 | 366 | 387 | Carbamidomethylation | C15:Carbamidomethylation:1000.00 | PEAKS DB |
| K.SVAVVQDYSK.R | N | 60.55 | 1094.5608 | 10 | -0.5 | 548.2874 | 2 | 26.31 | 1 | 9703 | BuCaMH03.raw | 3.8359E7 | 1 | 1 | 321 | 330 |  |  | PEAKS DB |
| Y.IELYM(+15.99)AVDNR.M | N | 59.55 | 1238.5966 | 10 | 0.8 | 620.3060 | 2 | 51.87 | 1 | 29062 | BuCaMH03.raw | 1.1103E6 | 1 | 1 | 207 | 216 | Oxidation (M) | M5:Oxidation (M):1000.00 | PEAKS DB |
| R.ETVLLPHKR.N | N | 59.06 | 1091.6451 | 9 | 0.9 | 546.8303 | 2 | 12.59 | 1 | 1892 | BuCaMH03.raw | 1.9337E6 | 1 | 1 | 282 | 290 |  |  | PEAKS DB |
| K.IEPDVDATLK.S | N | 58.89 | 1099.5760 | 10 | 0.0 | 550.7953 | 2 | 36.77 | 1 | 17181 | BuCaMH03.raw | 6.4067E7 | 1 | 1 | 266 | 275 |  |  | PEAKS DB |
| R.VC(+57.02)NC(+57.02)LILPDDPNYGMVETGTK.C | N | 58.49 | 2395.0857 | 21 | 0.6 | 799.3696 | 3 | 64.16 | 1 | 39325 | BuCaMH03.raw | 4.0264E6 | 1 | 1 | 575 | 595 | Carbamidomethylation | C2:Carbamidomethylation:1000.00;C4:Carbamidomethylation:1000.00 | PEAKS DB |
| R.ETVLLPHK.R | N | 58.18 | 935.5440 | 8 | 0.4 | 468.7795 | 2 | 22.25 | 1 | 6754 | BuCaMH03.raw | 9.3762E6 | 1 | 1 | 282 | 289 |  |  | PEAKS DB |
| K.DDC(+57.02)DLPER.C | N | 56.58 | 1018.4025 | 8 | 0.5 | 510.2088 | 2 | 20.35 | 1 | 5679 | BuCaMH03.raw | 2.5284E6 | 1 | 1 | 472 | 479 | Carbamidomethylation | C3:Carbamidomethylation:1000.00 | PEAKS DB |
| I.ELYMAVDNR.M | N | 55.87 | 1109.5176 | 9 | 0.7 | 555.7665 | 2 | 36.61 | 1 | 16797 | BuCaMH03.raw | 7.7591E6 | 1 | 1 | 208 | 216 |  |  | PEAKS DB |
| K.KYIELYMAVDNR.M | N | 54.08 | 1513.7599 | 12 | 0.1 | 505.5940 | 3 | 47.31 | 1 | 25389 | BuCaMH03.raw | 4.4357E5 | 1 | 1 | 205 | 216 |  |  | PEAKS DB |
| S.C(+57.02)FTLNQR.G | N | 53.69 | 937.4440 | 7 | -0.2 | 469.7292 | 2 | 25.79 | 1 | 9047 | BuCaMH03.raw | 1.2157E6 | 1 | 1 | 533 | 539 | Carbamidomethylation | C1:Carbamidomethylation:1000.00 | PEAKS DB |
| total 23 peptides |  |  |  |  |  |  |  |  |  |  |  |  |  |  |  |  |  |  |  |
| --- | --- | --- | --- | --- | --- | --- | --- | --- | --- | --- | --- | --- | --- | --- | --- | --- | --- | --- | --- |

T\_33

back to list

  

| Protein Coverage
| Supporting Peptides
|

Protein Coverage:

Supporting Peptides:

| Peptide | Uniq | -10lgP | Mass | Length | ppm | m/z | z | RT | Fraction | Scan | Source File | Area BuCaMH03\_B17 | #Feature | #Feature BuCaMH03\_B17 | Start | End | PTM | AScore | Found By |
| --- | --- | --- | --- | --- | --- | --- | --- | --- | --- | --- | --- | --- | --- | --- | --- | --- | --- | --- | --- |
| R.VAAIC(+57.02)FAGAPYNNSNFMVASK.I | Y | 99.75 | 2231.0503 | 21 | -0.3 | 1116.5321 | 2 | 66.90 | 1 | 41542 | BuCaMH03.raw | 6.6548E8 | 3 | 3 | 67 | 87 | Carbamidomethylation | C5:Carbamidomethylation:1000.00 | PEAKS DB |
| A.AIC(+57.02)FAGAPYNNSNFMVASK.I | Y | 95.58 | 2060.9448 | 19 | 0.5 | 1031.4802 | 2 | 62.66 | 1 | 38110 | BuCaMH03.raw | 1.5008E8 | 2 | 2 | 69 | 87 | Carbamidomethylation | C3:Carbamidomethylation:1000.00 | PEAKS DB |
| K.TYSYDC(+57.02)SEGKLTC(+57.02)KDTAGTC(+57.02)AR.I | Y | 84.63 | 2543.0728 | 22 | 0.0 | 636.7755 | 4 | 22.48 | 1 | 6906 | BuCaMH03.raw | 3.3749E6 | 1 | 1 | 38 | 59 | Carbamidomethylation | C6:Carbamidomethylation:1000.00;C13:Carbamidomethylation:1000.00;C20:Carbamidomethylation:1000.00 | PEAKS DB |
| V.AAIC(+57.02)FAGAPYNNSNFMVASK.I | Y | 84.58 | 2131.9819 | 20 | 0.3 | 1066.9985 | 2 | 63.68 | 1 | 39029 | BuCaMH03.raw | 1.0774E8 | 2 | 2 | 68 | 87 | Carbamidomethylation | C4:Carbamidomethylation:1000.00 | PEAKS DB |
| R.VAAIC(+57.02)FAGAPYNNSNFMVASKIKC(+57.02)E | Y | 82.10 | 2761.3025 | 25 | 1.2 | 921.4426 | 3 | 62.54 | 1 | 37961 | BuCaMH03.raw | 3.9735E6 | 1 | 1 | 67 | 91 | Carbamidomethylation | C5:Carbamidomethylation:1000.00;C24:Carbamidomethylation:1000.00 | PEAKS DB |
| K.GGGGTPVDDLDR.C | N | 78.80 | 1157.5312 | 12 | -0.3 | 579.7727 | 2 | 27.28 | 1 | 10243 | BuCaMH03.raw | 3.2115E9 | 2 | 2 | 3 | 14 |  |  | PEAKS DB |
| G.GGGTPVDDLDR.C | N | 76.90 | 1100.5098 | 11 | 0.1 | 551.2622 | 2 | 27.24 | 1 | 10065 | BuCaMH03.raw | 3.4495E7 | 1 | 1 | 4 | 14 |  |  | PEAKS DB |
| A.GAPYNNSNFMVASK.I | Y | 73.09 | 1498.6875 | 14 | 0.0 | 750.3510 | 2 | 38.75 | 1 | 18464 | BuCaMH03.raw | 3.7326E7 | 1 | 1 | 74 | 87 |  |  | PEAKS DB |
| F.AGAPYNNSNFM(+15.99)VASK.I | Y | 71.28 | 1585.7195 | 15 | 0.7 | 793.8676 | 2 | 40.01 | 1 | 19307 | BuCaMH03.raw | 3.1461E6 | 2 | 2 | 73 | 87 | Oxidation (M) | M11:Oxidation (M):1000.00 | PEAKS DB |
| F.AGAPYNNSNFMVASK.I | Y | 71.02 | 1569.7246 | 15 | -0.2 | 785.8694 | 2 | 39.97 | 1 | 19594 | BuCaMH03.raw | 5.5867E7 | 1 | 1 | 73 | 87 |  |  | PEAKS DB |
| A.GAPYNNSNFM(+15.99)VASK.I | Y | 66.24 | 1514.6824 | 14 | -0.4 | 758.3481 | 2 | 38.75 | 1 | 18546 | BuCaMH03.raw | 1.9449E6 | 1 | 1 | 74 | 87 | Oxidation (M) | M10:Oxidation (M):1000.00 | PEAKS DB |
| K.TYSYDC(+57.02)SEGKLTC(+57.02)K.D | N | 65.46 | 1710.7229 | 14 | 0.1 | 571.2483 | 3 | 23.16 | 1 | 7349 | BuCaMH03.raw | 2.7246E6 | 1 | 1 | 38 | 51 | Carbamidomethylation | C6:Carbamidomethylation:1000.00;C13:Carbamidomethylation:1000.00 | PEAKS DB |
| K.TYSYDC(+57.02)SEGK.L | N | 61.86 | 1208.4656 | 10 | 0.1 | 605.2401 | 2 | 15.61 | 1 | 4034 | BuCaMH03.raw | 6.0668E8 | 1 | 1 | 38 | 47 | Carbamidomethylation | C6:Carbamidomethylation:1000.00 | PEAKS DB |
| K.LASC(+57.02)RPYYK.T | N | 58.27 | 1156.5698 | 9 | -0.1 | 579.2921 | 2 | 12.61 | 1 | 1896 | BuCaMH03.raw | 4.5113E6 | 1 | 1 | 29 | 37 | Carbamidomethylation | C4:Carbamidomethylation:1000.00 | PEAKS DB |
| R.VAAIC(+57.02)FAGAPYNNSNFM(+15.99)VASK.I | Y | 55.07 | 2247.0452 | 21 | 0.2 | 750.0225 | 3 | 66.90 | 1 | 41828 | BuCaMH03.raw | 3.5394E6 | 1 | 1 | 67 | 87 | Carbamidomethylation; Oxidation (M) | C5:Carbamidomethylation:1000.00;M17:Oxidation (M):1000.00 | PEAKS DB |
| G.GGTPVDDLDR.C | N | 54.58 | 1043.4883 | 10 | -0.6 | 522.7511 | 2 | 27.32 | 1 | 10090 | BuCaMH03.raw | 4.8161E6 | 1 | 1 | 5 | 14 |  |  | PEAKS DB |
| K.VHDDC(+57.02)YGEAEKLASC(+57.02)RPYYK.T | N | 53.00 | 2460.0837 | 20 | 0.9 | 616.0287 | 4 | 34.87 | 1 | 15300 | BuCaMH03.raw | 4.6326E6 | 1 | 1 | 18 | 37 | Carbamidomethylation | C5:Carbamidomethylation:1000.00;C15:Carbamidomethylation:1000.00 | PEAKS DB |
| total 17 peptides |  |  |  |  |  |  |  |  |  |  |  |  |  |  |  |  |  |  |  |
| --- | --- | --- | --- | --- | --- | --- | --- | --- | --- | --- | --- | --- | --- | --- | --- | --- | --- | --- | --- |

ABN72537.1

back to list

  

| Protein Coverage
| Supporting Peptides
|

Protein Coverage:

Supporting Peptides:

| Peptide | Uniq | -10lgP | Mass | Length | ppm | m/z | z | RT | Fraction | Scan | Source File | Area BuCaMH03\_B17 | #Feature | #Feature BuCaMH03\_B17 | Start | End | PTM | AScore | Found By |
| --- | --- | --- | --- | --- | --- | --- | --- | --- | --- | --- | --- | --- | --- | --- | --- | --- | --- | --- | --- |
| R.DRPQC(+57.02)ILNKPLITDIVAPAIC(+57.02)GNYFVEVGEEC(+57.02)DC(+57.02)GSPR.D | Y | 97.02 | 4362.0542 | 38 | -2.6 | 1091.5179 | 4 | 83.53 | 1 | 55065 | BuCaMH03.raw | 1.1448E8 | 2 | 2 | 392 | 429 | Carbamidomethylation | C5:Carbamidomethylation:1000.00;C21:Carbamidomethylation:1000.00;C32:Carbamidomethylation:1000.00;C34:Carbamidomethylation:1000.00 | PEAKS DB |
| R.IDFNGNTVGLAALGSLC(+57.02)SVK.Y | N | 84.40 | 2035.0408 | 20 | 0.6 | 1018.5283 | 2 | 79.20 | 1 | 51554 | BuCaMH03.raw | 1.4604E7 | 1 | 1 | 300 | 319 | Carbamidomethylation | C17:Carbamidomethylation:1000.00 | PEAKS DB |
| R.VYELVNILNTILR.R | N | 80.71 | 1558.9082 | 13 | 1.0 | 780.4622 | 2 | 98.30 | 1 | 67045 | BuCaMH03.raw | 2.3113E7 | 1 | 1 | 232 | 244 |  |  | PEAKS DB |
| K.YSVAVIQDYSK.R | N | 71.25 | 1271.6398 | 11 | -0.2 | 636.8270 | 2 | 45.55 | 1 | 23713 | BuCaMH03.raw | 1.1049E7 | 1 | 1 | 320 | 330 |  |  | PEAKS DB |
| K.C(+57.02)PTLTNQC(+57.02)IALMGPNVK.V | N | 70.69 | 1915.9318 | 17 | 0.6 | 639.6516 | 3 | 55.90 | 1 | 32502 | BuCaMH03.raw | 1.0403E6 | 1 | 1 | 511 | 527 | Carbamidomethylation | C1:Carbamidomethylation:1000.00;C8:Carbamidomethylation:1000.00 | PEAKS DB |
| R.DSC(+57.02)FTLNQR.G | N | 68.01 | 1139.5029 | 9 | -0.1 | 570.7587 | 2 | 38.10 | 1 | 18031 | BuCaMH03.raw | 1.3816E6 | 1 | 1 | 531 | 539 | Carbamidomethylation | C3:Carbamidomethylation:1000.00 | PEAKS DB |
| V.C(+57.02)NC(+57.02)LILPDDPNYGMVETGTK.C | N | 67.70 | 2296.0173 | 20 | -0.4 | 1149.0155 | 2 | 62.88 | 1 | 38333 | BuCaMH03.raw | 1.54E6 | 1 | 1 | 576 | 595 | Carbamidomethylation | C1:Carbamidomethylation:1000.00;C3:Carbamidomethylation:1000.00 | PEAKS DB |
| P.TLTNQC(+57.02)IALMGPNVK.V | N | 64.79 | 1658.8484 | 15 | 1.0 | 830.4323 | 2 | 51.45 | 1 | 28756 | BuCaMH03.raw | 1.0501E6 | 1 | 1 | 513 | 527 | Carbamidomethylation | C6:Carbamidomethylation:1000.00 | PEAKS DB |
| R.TSMVASTMAHEMGHNLGINHDR.A | N | 64.46 | 2408.0784 | 22 | 0.4 | 603.0271 | 4 | 38.14 | 1 | 17887 | BuCaMH03.raw | 8.2173E6 | 1 | 1 | 332 | 353 |  |  | PEAKS DB |
| Y.FVEVGEEC(+57.02)DC(+57.02)GSPR.D | N | 62.34 | 1639.6606 | 14 | 0.5 | 820.8380 | 2 | 31.45 | 1 | 12962 | BuCaMH03.raw | 8.184E5 | 1 | 1 | 416 | 429 | Carbamidomethylation | C8:Carbamidomethylation:1000.00;C10:Carbamidomethylation:1000.00 | PEAKS DB |
| T.DIVAPAIC(+57.02)GNYFVEVGEEC(+57.02)DC(+57.02)GSPR.D | Y | 61.70 | 2813.2095 | 25 | 1.2 | 938.7449 | 3 | 76.88 | 1 | 49781 | BuCaMH03.raw | 1.0195E7 | 1 | 1 | 405 | 429 | Carbamidomethylation | C8:Carbamidomethylation:1000.00;C19:Carbamidomethylation:1000.00;C21:Carbamidomethylation:1000.00 | PEAKS DB |
| R.RVYELVNILNTILR.R | N | 61.38 | 1715.0094 | 14 | 0.9 | 572.6776 | 3 | 87.25 | 1 | 57914 | BuCaMH03.raw | 9.6658E6 | 1 | 1 | 231 | 244 |  |  | PEAKS DB |
| R.DKINVQSDVK.A | N | 59.76 | 1144.6088 | 10 | 0.4 | 573.3119 | 2 | 13.87 | 1 | 2502 | BuCaMH03.raw | 4.1024E6 | 1 | 1 | 262 | 271 |  |  | PEAKS DB |
| G.VC(+57.02)NC(+57.02)LILPDDPNYGMVETGTK.C | N | 58.49 | 2395.0857 | 21 | 0.6 | 799.3696 | 3 | 64.16 | 1 | 39325 | BuCaMH03.raw | 4.0264E6 | 1 | 1 | 575 | 595 | Carbamidomethylation | C2:Carbamidomethylation:1000.00;C4:Carbamidomethylation:1000.00 | PEAKS DB |
| K.YSVAVIQDYSKR.T | N | 57.88 | 1427.7408 | 12 | -0.5 | 714.8773 | 2 | 37.41 | 1 | 17503 | BuCaMH03.raw | 4.5095E5 | 1 | 1 | 320 | 331 |  |  | PEAKS DB |
| K.DDC(+57.02)DLPER.C | N | 56.58 | 1018.4025 | 8 | 0.5 | 510.2088 | 2 | 20.35 | 1 | 5679 | BuCaMH03.raw | 2.5284E6 | 1 | 1 | 472 | 479 | Carbamidomethylation | C3:Carbamidomethylation:1000.00 | PEAKS DB |
| R.NDNAQLLTR.I | N | 55.52 | 1043.5360 | 9 | 0.1 | 522.7753 | 2 | 23.63 | 1 | 7605 | BuCaMH03.raw | 8.9599E6 | 1 | 1 | 291 | 299 |  |  | PEAKS DB |
| L.NFHIALIGLEIWSK.R | N | 53.97 | 1639.9086 | 14 | 0.2 | 547.6436 | 3 | 80.89 | 1 | 53058 | BuCaMH03.raw | 1.4388E5 | 1 | 1 | 247 | 260 |  |  | PEAKS DB |
| K.INVQSDVK.A | N | 53.76 | 901.4869 | 8 | 0.2 | 451.7508 | 2 | 17.18 | 1 | 3871 | BuCaMH03.raw | 3.0691E6 | 1 | 1 | 264 | 271 |  |  | PEAKS DB |
| S.C(+57.02)FTLNQR.G | N | 53.69 | 937.4440 | 7 | -0.2 | 469.7292 | 2 | 25.79 | 1 | 9047 | BuCaMH03.raw | 1.2157E6 | 1 | 1 | 533 | 539 | Carbamidomethylation | C1:Carbamidomethylation:1000.00 | PEAKS DB |
| N.KPLITDIVAPAIC(+57.02)GNYFVEVGEEC(+57.02)DC(+57.02)GSPR.D | Y | 53.23 | 3365.5730 | 30 | -0.6 | 1122.8643 | 3 | 78.25 | 1 | 50839 | BuCaMH03.raw | 1.302E6 | 1 | 1 | 400 | 429 | Carbamidomethylation | C13:Carbamidomethylation:1000.00;C24:Carbamidomethylation:1000.00;C26:Carbamidomethylation:1000.00 | PEAKS DB |
| total 21 peptides |  |  |  |  |  |  |  |  |  |  |  |  |  |  |  |  |  |  |  |
| --- | --- | --- | --- | --- | --- | --- | --- | --- | --- | --- | --- | --- | --- | --- | --- | --- | --- | --- | --- |

A8QL49.1

back to list

  

| Protein Coverage
| Supporting Peptides
|

Protein Coverage:

Supporting Peptides:

| Peptide | Uniq | -10lgP | Mass | Length | ppm | m/z | z | RT | Fraction | Scan | Source File | Area BuCaMH03\_B17 | #Feature | #Feature BuCaMH03\_B17 | Start | End | PTM | AScore | Found By |
| --- | --- | --- | --- | --- | --- | --- | --- | --- | --- | --- | --- | --- | --- | --- | --- | --- | --- | --- | --- |
| R.DRPQC(+57.02)ILNKPLITDIVAPAIC(+57.02)GNYFVEVGEEC(+57.02)DC(+57.02)GSPR.D | Y | 97.02 | 4362.0542 | 38 | -2.6 | 1091.5179 | 4 | 83.53 | 1 | 55065 | BuCaMH03.raw | 1.1448E8 | 2 | 2 | 392 | 429 | Carbamidomethylation | C5:Carbamidomethylation:1000.00;C21:Carbamidomethylation:1000.00;C32:Carbamidomethylation:1000.00;C34:Carbamidomethylation:1000.00 | PEAKS DB |
| R.IDFNGNTVGLAALGSLC(+57.02)SVK.Y | N | 84.40 | 2035.0408 | 20 | 0.6 | 1018.5283 | 2 | 79.20 | 1 | 51554 | BuCaMH03.raw | 1.4604E7 | 1 | 1 | 300 | 319 | Carbamidomethylation | C17:Carbamidomethylation:1000.00 | PEAKS DB |
| R.VYELVNILNTILR.R | N | 80.71 | 1558.9082 | 13 | 1.0 | 780.4622 | 2 | 98.30 | 1 | 67045 | BuCaMH03.raw | 2.3113E7 | 1 | 1 | 232 | 244 |  |  | PEAKS DB |
| K.YSVAVIQDYSK.R | N | 71.25 | 1271.6398 | 11 | -0.2 | 636.8270 | 2 | 45.55 | 1 | 23713 | BuCaMH03.raw | 1.1049E7 | 1 | 1 | 320 | 330 |  |  | PEAKS DB |
| K.C(+57.02)PTLTNQC(+57.02)IALMGPNVK.V | N | 70.69 | 1915.9318 | 17 | 0.6 | 639.6516 | 3 | 55.90 | 1 | 32502 | BuCaMH03.raw | 1.0403E6 | 1 | 1 | 511 | 527 | Carbamidomethylation | C1:Carbamidomethylation:1000.00;C8:Carbamidomethylation:1000.00 | PEAKS DB |
| R.DSC(+57.02)FTLNQR.G | N | 68.01 | 1139.5029 | 9 | -0.1 | 570.7587 | 2 | 38.10 | 1 | 18031 | BuCaMH03.raw | 1.3816E6 | 1 | 1 | 531 | 539 | Carbamidomethylation | C3:Carbamidomethylation:1000.00 | PEAKS DB |
| V.C(+57.02)NC(+57.02)LILPDDPNYGMVETGTK.C | N | 67.70 | 2296.0173 | 20 | -0.4 | 1149.0155 | 2 | 62.88 | 1 | 38333 | BuCaMH03.raw | 1.54E6 | 1 | 1 | 576 | 595 | Carbamidomethylation | C1:Carbamidomethylation:1000.00;C3:Carbamidomethylation:1000.00 | PEAKS DB |
| P.TLTNQC(+57.02)IALMGPNVK.V | N | 64.79 | 1658.8484 | 15 | 1.0 | 830.4323 | 2 | 51.45 | 1 | 28756 | BuCaMH03.raw | 1.0501E6 | 1 | 1 | 513 | 527 | Carbamidomethylation | C6:Carbamidomethylation:1000.00 | PEAKS DB |
| R.TSMVASTMAHEMGHNLGINHDR.A | N | 64.46 | 2408.0784 | 22 | 0.4 | 603.0271 | 4 | 38.14 | 1 | 17887 | BuCaMH03.raw | 8.2173E6 | 1 | 1 | 332 | 353 |  |  | PEAKS DB |
| Y.FVEVGEEC(+57.02)DC(+57.02)GSPR.D | N | 62.34 | 1639.6606 | 14 | 0.5 | 820.8380 | 2 | 31.45 | 1 | 12962 | BuCaMH03.raw | 8.184E5 | 1 | 1 | 416 | 429 | Carbamidomethylation | C8:Carbamidomethylation:1000.00;C10:Carbamidomethylation:1000.00 | PEAKS DB |
| T.DIVAPAIC(+57.02)GNYFVEVGEEC(+57.02)DC(+57.02)GSPR.D | Y | 61.70 | 2813.2095 | 25 | 1.2 | 938.7449 | 3 | 76.88 | 1 | 49781 | BuCaMH03.raw | 1.0195E7 | 1 | 1 | 405 | 429 | Carbamidomethylation | C8:Carbamidomethylation:1000.00;C19:Carbamidomethylation:1000.00;C21:Carbamidomethylation:1000.00 | PEAKS DB |
| R.RVYELVNILNTILR.R | N | 61.38 | 1715.0094 | 14 | 0.9 | 572.6776 | 3 | 87.25 | 1 | 57914 | BuCaMH03.raw | 9.6658E6 | 1 | 1 | 231 | 244 |  |  | PEAKS DB |
| R.DKINVQSDVK.A | N | 59.76 | 1144.6088 | 10 | 0.4 | 573.3119 | 2 | 13.87 | 1 | 2502 | BuCaMH03.raw | 4.1024E6 | 1 | 1 | 262 | 271 |  |  | PEAKS DB |
| G.VC(+57.02)NC(+57.02)LILPDDPNYGMVETGTK.C | N | 58.49 | 2395.0857 | 21 | 0.6 | 799.3696 | 3 | 64.16 | 1 | 39325 | BuCaMH03.raw | 4.0264E6 | 1 | 1 | 575 | 595 | Carbamidomethylation | C2:Carbamidomethylation:1000.00;C4:Carbamidomethylation:1000.00 | PEAKS DB |
| K.YSVAVIQDYSKR.T | N | 57.88 | 1427.7408 | 12 | -0.5 | 714.8773 | 2 | 37.41 | 1 | 17503 | BuCaMH03.raw | 4.5095E5 | 1 | 1 | 320 | 331 |  |  | PEAKS DB |
| K.DDC(+57.02)DLPER.C | N | 56.58 | 1018.4025 | 8 | 0.5 | 510.2088 | 2 | 20.35 | 1 | 5679 | BuCaMH03.raw | 2.5284E6 | 1 | 1 | 472 | 479 | Carbamidomethylation | C3:Carbamidomethylation:1000.00 | PEAKS DB |
| R.NDNAQLLTR.I | N | 55.52 | 1043.5360 | 9 | 0.1 | 522.7753 | 2 | 23.63 | 1 | 7605 | BuCaMH03.raw | 8.9599E6 | 1 | 1 | 291 | 299 |  |  | PEAKS DB |
| L.NFHIALIGLEIWSK.R | N | 53.97 | 1639.9086 | 14 | 0.2 | 547.6436 | 3 | 80.89 | 1 | 53058 | BuCaMH03.raw | 1.4388E5 | 1 | 1 | 247 | 260 |  |  | PEAKS DB |
| K.INVQSDVK.A | N | 53.76 | 901.4869 | 8 | 0.2 | 451.7508 | 2 | 17.18 | 1 | 3871 | BuCaMH03.raw | 3.0691E6 | 1 | 1 | 264 | 271 |  |  | PEAKS DB |
| S.C(+57.02)FTLNQR.G | N | 53.69 | 937.4440 | 7 | -0.2 | 469.7292 | 2 | 25.79 | 1 | 9047 | BuCaMH03.raw | 1.2157E6 | 1 | 1 | 533 | 539 | Carbamidomethylation | C1:Carbamidomethylation:1000.00 | PEAKS DB |
| N.KPLITDIVAPAIC(+57.02)GNYFVEVGEEC(+57.02)DC(+57.02)GSPR.D | Y | 53.23 | 3365.5730 | 30 | -0.6 | 1122.8643 | 3 | 78.25 | 1 | 50839 | BuCaMH03.raw | 1.302E6 | 1 | 1 | 400 | 429 | Carbamidomethylation | C13:Carbamidomethylation:1000.00;C24:Carbamidomethylation:1000.00;C26:Carbamidomethylation:1000.00 | PEAKS DB |
| total 21 peptides |  |  |  |  |  |  |  |  |  |  |  |  |  |  |  |  |  |  |  |
| --- | --- | --- | --- | --- | --- | --- | --- | --- | --- | --- | --- | --- | --- | --- | --- | --- | --- | --- | --- |

T\_11

back to list

  

| Protein Coverage
| Supporting Peptides
|

Protein Coverage:

Supporting Peptides:

| Peptide | Uniq | -10lgP | Mass | Length | ppm | m/z | z | RT | Fraction | Scan | Source File | Area BuCaMH03\_B17 | #Feature | #Feature BuCaMH03\_B17 | Start | End | PTM | AScore | Found By |
| --- | --- | --- | --- | --- | --- | --- | --- | --- | --- | --- | --- | --- | --- | --- | --- | --- | --- | --- | --- |
| K.VSPDAC(+57.02)FSLNHISQGC(+57.02)GFC(+57.02)R.M | N | 97.28 | 2310.9932 | 20 | -1.5 | 1156.5021 | 2 | 51.24 | 1 | 28545 | BuCaMH03.raw | 9.4127E7 | 2 | 2 | 202 | 221 | Carbamidomethylation | C6:Carbamidomethylation:1000.00;C16:Carbamidomethylation:1000.00;C19:Carbamidomethylation:1000.00 | PEAKS DB |
| K.C(+57.02)PTLTNQC(+57.02)IALWGPGAK.V | N | 96.95 | 1885.9178 | 17 | 1.0 | 943.9672 | 2 | 61.17 | 1 | 36745 | BuCaMH03.raw | 3.4505E7 | 2 | 2 | 185 | 201 | Carbamidomethylation | C1:Carbamidomethylation:1000.00;C8:Carbamidomethylation:1000.00 | PEAKS DB |
| I.VLDDPDNGMVEPGTK.C | N | 94.02 | 1585.7294 | 15 | 0.8 | 793.8726 | 2 | 36.89 | 1 | 17056 | BuCaMH03.raw | 3.3063E6 | 1 | 1 | 254 | 268 |  |  | PEAKS DB |
| K.RDEPLYEFSFC(+57.02)SVQEHR.R | Y | 91.56 | 2197.9851 | 17 | 0.6 | 733.6694 | 3 | 51.10 | 1 | 28374 | BuCaMH03.raw | 6.3865E6 | 1 | 1 | 44 | 60 | Carbamidomethylation | C11:Carbamidomethylation:1000.00 | PEAKS DB |
| K.IVLDDPDNGMVEPGTK.C | N | 86.01 | 1698.8135 | 16 | 0.1 | 850.4141 | 2 | 47.31 | 1 | 25449 | BuCaMH03.raw | 5.6917E7 | 1 | 1 | 253 | 268 |  |  | PEAKS DB |
| V.SPDAC(+57.02)FSLNHISQGC(+57.02)GFC(+57.02)R.M | N | 80.77 | 2211.9248 | 19 | -0.3 | 1106.9694 | 2 | 49.02 | 1 | 26738 | BuCaMH03.raw | 1.0185E7 | 2 | 2 | 203 | 221 | Carbamidomethylation | C5:Carbamidomethylation:1000.00;C15:Carbamidomethylation:1000.00;C18:Carbamidomethylation:1000.00 | PEAKS DB |
| K.IVLDDPDNGM(+15.99)VEPGTK.C | N | 78.87 | 1714.8083 | 16 | 0.3 | 858.4117 | 2 | 47.32 | 1 | 25299 | BuCaMH03.raw | 3.3217E6 | 2 | 2 | 253 | 268 | Oxidation (M) | M10:Oxidation (M):1000.00 | PEAKS DB |
| R.DEPLYEFSFC(+57.02)SVQEHR.R | Y | 73.36 | 2041.8839 | 16 | 0.8 | 681.6358 | 3 | 63.30 | 1 | 38612 | BuCaMH03.raw | 1.1308E6 | 1 | 1 | 45 | 60 | Carbamidomethylation | C10:Carbamidomethylation:1000.00 | PEAKS DB |
| P.DAC(+57.02)FSLNHISQGC(+57.02)GFC(+57.02)R.M | N | 71.16 | 2027.8400 | 17 | 1.5 | 676.9550 | 3 | 50.33 | 1 | 27777 | BuCaMH03.raw | 3.3793E6 | 1 | 1 | 205 | 221 | Carbamidomethylation | C3:Carbamidomethylation:1000.00;C13:Carbamidomethylation:1000.00;C16:Carbamidomethylation:1000.00 | PEAKS DB |
| K.VSPDAC(+57.02)FSLNHISQGC(+57.02)GF.C | N | 71.10 | 1994.8615 | 18 | 0.0 | 998.4380 | 2 | 63.68 | 1 | 38988 | BuCaMH03.raw | 0 | 0 | 0 | 202 | 219 | Carbamidomethylation | C6:Carbamidomethylation:1000.00;C16:Carbamidomethylation:1000.00 | PEAKS DB |
| F.SLNHISQGC(+57.02)GFC(+57.02)R.M | N | 69.13 | 1534.6769 | 13 | 0.0 | 512.5662 | 3 | 23.17 | 1 | 7446 | BuCaMH03.raw | 6.8367E5 | 1 | 1 | 209 | 221 | Carbamidomethylation | C9:Carbamidomethylation:1000.00;C12:Carbamidomethylation:1000.00 | PEAKS DB |
| P.TLTNQC(+57.02)IALWGPGAK.V | N | 62.29 | 1628.8345 | 15 | 0.1 | 815.4246 | 2 | 57.13 | 1 | 33455 | BuCaMH03.raw | 7.9432E6 | 1 | 1 | 187 | 201 | Carbamidomethylation | C6:Carbamidomethylation:1000.00 | PEAKS DB |
| K.DDC(+57.02)DLPER.C | N | 56.58 | 1018.4025 | 8 | 0.5 | 510.2088 | 2 | 20.35 | 1 | 5679 | BuCaMH03.raw | 2.5284E6 | 1 | 1 | 146 | 153 | Carbamidomethylation | C3:Carbamidomethylation:1000.00 | PEAKS DB |
| total 13 peptides |  |  |  |  |  |  |  |  |  |  |  |  |  |  |  |  |  |  |  |
| --- | --- | --- | --- | --- | --- | --- | --- | --- | --- | --- | --- | --- | --- | --- | --- | --- | --- | --- | --- |

T\_81

back to list

  

| Protein Coverage
| Supporting Peptides
|

Protein Coverage:

Supporting Peptides:

| Peptide | Uniq | -10lgP | Mass | Length | ppm | m/z | z | RT | Fraction | Scan | Source File | Area BuCaMH03\_B17 | #Feature | #Feature BuCaMH03\_B17 | Start | End | PTM | AScore | Found By |
| --- | --- | --- | --- | --- | --- | --- | --- | --- | --- | --- | --- | --- | --- | --- | --- | --- | --- | --- | --- |
| R.LNLIQFSNMIQC(+57.02)ANHNTRPTWHYANYGC(+57.02)YC(+57.02)GK.G | Y | 91.32 | 3930.7600 | 32 | 0.0 | 787.1593 | 5 | 63.87 | 1 | 39030 | BuCaMH03.raw | 4.7504E8 | 3 | 3 | 44 | 75 | Carbamidomethylation | C12:Carbamidomethylation:1000.00;C28:Carbamidomethylation:1000.00;C30:Carbamidomethylation:1000.00 | PEAKS DB |
| R.PTWHYANYGC(+57.02)YC(+57.02)GK.G | Y | 84.41 | 1775.7184 | 14 | 0.8 | 592.9139 | 3 | 35.11 | 1 | 15457 | BuCaMH03.raw | 3.4783E8 | 3 | 3 | 62 | 75 | Carbamidomethylation | C10:Carbamidomethylation:1000.00;C12:Carbamidomethylation:1000.00 | PEAKS DB |
| W.HYANYGC(+57.02)YC(+57.02)GK.G | Y | 81.27 | 1391.5387 | 11 | 0.3 | 696.7769 | 2 | 14.14 | 1 | 2644 | BuCaMH03.raw | 2.1221E7 | 4 | 4 | 65 | 75 | Carbamidomethylation | C7:Carbamidomethylation:1000.00;C9:Carbamidomethylation:1000.00 | PEAKS DB |
| K.GGGGTPVDDLDR.C | N | 78.80 | 1157.5312 | 12 | -0.3 | 579.7727 | 2 | 27.28 | 1 | 10243 | BuCaMH03.raw | 3.2115E9 | 2 | 2 | 76 | 87 |  |  | PEAKS DB |
| L.NLIQFSNMIQC(+57.02)ANHNTRPTWHYANYGC(+57.02)YC(+57.02)GK.G | Y | 77.92 | 3817.6758 | 31 | 0.0 | 955.4262 | 4 | 117.13 | 1 | 75083 | BuCaMH03.raw | 2.3113E10 | 4 | 4 | 45 | 75 | Carbamidomethylation | C11:Carbamidomethylation:1000.00;C27:Carbamidomethylation:1000.00;C29:Carbamidomethylation:1000.00 | PEAKS DB |
| G.GGGTPVDDLDR.C | N | 76.90 | 1100.5098 | 11 | 0.1 | 551.2622 | 2 | 27.24 | 1 | 10065 | BuCaMH03.raw | 3.4495E7 | 1 | 1 | 77 | 87 |  |  | PEAKS DB |
| C.ANHNTRPTWHYANYGC(+57.02)YC(+57.02)GK.G | Y | 75.53 | 2469.0491 | 20 | 0.4 | 618.2698 | 4 | 25.24 | 1 | 8842 | BuCaMH03.raw | 1.5919E7 | 1 | 1 | 56 | 75 | Carbamidomethylation | C16:Carbamidomethylation:1000.00;C18:Carbamidomethylation:1000.00 | PEAKS DB |
| F.SNMIQC(+57.02)ANHNTRPTWHYANYGC(+57.02)YC(+57.02)GK.G | Y | 73.54 | 3202.3379 | 26 | -0.1 | 801.5917 | 4 | 33.99 | 1 | 14982 | BuCaMH03.raw | 1.0099E8 | 1 | 1 | 50 | 75 | Carbamidomethylation | C6:Carbamidomethylation:1000.00;C22:Carbamidomethylation:1000.00;C24:Carbamidomethylation:1000.00 | PEAKS DB |
| P.TWHYANYGC(+57.02)YC(+57.02)GK.G | Y | 69.81 | 1678.6656 | 13 | 0.0 | 840.3401 | 2 | 32.26 | 1 | 13415 | BuCaMH03.raw | 3.4421E7 | 2 | 2 | 63 | 75 | Carbamidomethylation | C9:Carbamidomethylation:1000.00;C11:Carbamidomethylation:1000.00 | PEAKS DB |
| N.HNTRPTWHYANYGC(+57.02)YC(+57.02)GK.G | Y | 66.71 | 2283.9690 | 18 | 0.8 | 762.3309 | 3 | 27.71 | 1 | 10295 | BuCaMH03.raw | 1.7431E7 | 4 | 4 | 58 | 75 | Carbamidomethylation | C14:Carbamidomethylation:1000.00;C16:Carbamidomethylation:1000.00 | PEAKS DB |
| T.RPTWHYANYGC(+57.02)YC(+57.02)GK.G | Y | 61.38 | 1931.8196 | 15 | 0.4 | 483.9623 | 4 | 28.24 | 1 | 10651 | BuCaMH03.raw | 1.7436E6 | 1 | 1 | 61 | 75 | Carbamidomethylation | C11:Carbamidomethylation:1000.00;C13:Carbamidomethylation:1000.00 | PEAKS DB |
| T.WHYANYGC(+57.02)YC(+57.02)GK.G | Y | 60.87 | 1577.6180 | 12 | 0.3 | 789.8165 | 2 | 30.69 | 1 | 12350 | BuCaMH03.raw | 4.2298E6 | 1 | 1 | 64 | 75 | Carbamidomethylation | C8:Carbamidomethylation:1000.00;C10:Carbamidomethylation:1000.00 | PEAKS DB |
| R.LNLIQFSNMIQC(+57.02)ANHNTRPTWHYANYGC(+57.02)YC(+57.02)GKGGGGTPVDDLDR.C | Y | 60.53 | 5070.2808 | 44 | 0.3 | 1015.0638 | 5 | 64.33 | 1 | 39517 | BuCaMH03.raw | 2.074E7 | 1 | 1 | 44 | 87 | Carbamidomethylation | C12:Carbamidomethylation:1000.00;C28:Carbamidomethylation:1000.00;C30:Carbamidomethylation:1000.00 | PEAKS DB |
| A.NHNTRPTWHYANYGC(+57.02)YC(+57.02)GK.G | Y | 59.99 | 2398.0120 | 19 | 0.6 | 800.3451 | 3 | 26.35 | 1 | 9602 | BuCaMH03.raw | 3.1191E6 | 1 | 1 | 57 | 75 | Carbamidomethylation | C15:Carbamidomethylation:1000.00;C17:Carbamidomethylation:1000.00 | PEAKS DB |
| H.YANYGC(+57.02)YC(+57.02)GK.G | N | 57.76 | 1254.4797 | 10 | 1.1 | 628.2479 | 2 | 22.28 | 1 | 7058 | BuCaMH03.raw | 3.6803E7 | 1 | 1 | 66 | 75 | Carbamidomethylation | C6:Carbamidomethylation:1000.00;C8:Carbamidomethylation:1000.00 | PEAKS DB |
| R.LNLIQFSNMIQC(+57.02)ANHNTR.P | Y | 57.52 | 2173.0520 | 18 | 0.9 | 725.3586 | 3 | 66.28 | 1 | 41049 | BuCaMH03.raw | 1.6904E6 | 1 | 1 | 44 | 61 | Carbamidomethylation | C12:Carbamidomethylation:1000.00 | PEAKS DB |
| Y.ANYGC(+57.02)YC(+57.02)GK.G | N | 54.65 | 1091.4165 | 9 | 0.5 | 546.7158 | 2 | 14.01 | 1 | 2519 | BuCaMH03.raw | 4.3578E6 | 1 | 1 | 67 | 75 | Carbamidomethylation | C5:Carbamidomethylation:1000.00;C7:Carbamidomethylation:1000.00 | PEAKS DB |
| G.GGTPVDDLDR.C | N | 54.58 | 1043.4883 | 10 | -0.6 | 522.7511 | 2 | 27.32 | 1 | 10090 | BuCaMH03.raw | 4.8161E6 | 1 | 1 | 78 | 87 |  |  | PEAKS DB |
| N.MIQC(+57.02)ANHNTRPTWHYANYGC(+57.02)YC(+57.02)GK.G | Y | 53.64 | 3001.2629 | 24 | -0.3 | 751.3228 | 4 | 30.26 | 1 | 12339 | BuCaMH03.raw | 5.4667E7 | 1 | 1 | 52 | 75 | Carbamidomethylation | C4:Carbamidomethylation:1000.00;C20:Carbamidomethylation:1000.00;C22:Carbamidomethylation:1000.00 | PEAKS DB |
| total 19 peptides |  |  |  |  |  |  |  |  |  |  |  |  |  |  |  |  |  |  |  |
| --- | --- | --- | --- | --- | --- | --- | --- | --- | --- | --- | --- | --- | --- | --- | --- | --- | --- | --- | --- |

P15816.1

back to list

  

| Protein Coverage
| Supporting Peptides
|

Protein Coverage:

Supporting Peptides:

| Peptide | Uniq | -10lgP | Mass | Length | ppm | m/z | z | RT | Fraction | Scan | Source File | Area BuCaMH03\_B17 | #Feature | #Feature BuCaMH03\_B17 | Start | End | PTM | AScore | Found By |
| --- | --- | --- | --- | --- | --- | --- | --- | --- | --- | --- | --- | --- | --- | --- | --- | --- | --- | --- | --- |
| R.GPVIEQGC(+57.02)AATC(+57.02)PEFR.S | N | 122.18 | 1790.8080 | 16 | -0.3 | 896.4110 | 2 | 41.38 | 1 | 20692 | BuCaMH03.raw | 1.6329E9 | 1 | 1 | 56 | 71 | Carbamidomethylation | C8:Carbamidomethylation:1000.00;C12:Carbamidomethylation:1000.00 | PEAKS DB |
| G.PVIEQGC(+57.02)AATC(+57.02)PEFR.S | N | 93.92 | 1733.7865 | 15 | 0.1 | 867.9006 | 2 | 40.50 | 1 | 19840 | BuCaMH03.raw | 1.1911E6 | 1 | 1 | 57 | 71 | Carbamidomethylation | C7:Carbamidomethylation:1000.00;C11:Carbamidomethylation:1000.00 | PEAKS DB |
| P.VIEQGC(+57.02)AATC(+57.02)PEFR.S | N | 88.61 | 1636.7338 | 14 | 0.1 | 819.3742 | 2 | 34.87 | 1 | 15468 | BuCaMH03.raw | 2.1479E9 | 2 | 2 | 58 | 71 | Carbamidomethylation | C6:Carbamidomethylation:1000.00;C10:Carbamidomethylation:1000.00 | PEAKS DB |
| R.SLLC(+57.02)C(+57.02)TTDNC(+57.02)NH | N | 82.48 | 1493.5698 | 12 | 0.1 | 747.7922 | 2 | 27.93 | 1 | 10590 | BuCaMH03.raw | 1.9154E9 | 2 | 2 | 76 | 87 | Carbamidomethylation | C4:Carbamidomethylation:1000.00;C5:Carbamidomethylation:1000.00;C10:Carbamidomethylation:1000.00 | PEAKS DB |
| V.IEQGC(+57.02)AATC(+57.02)PEFR.S | N | 73.44 | 1537.6653 | 13 | 0.2 | 769.8401 | 2 | 30.10 | 1 | 12233 | BuCaMH03.raw | 5.1231E7 | 1 | 1 | 59 | 71 | Carbamidomethylation | C5:Carbamidomethylation:1000.00;C9:Carbamidomethylation:1000.00 | PEAKS DB |
| I.EQGC(+57.02)AATC(+57.02)PEFR.S | N | 67.86 | 1424.5813 | 12 | 0.6 | 713.2983 | 2 | 25.26 | 1 | 8777 | BuCaMH03.raw | 7.8103E6 | 1 | 1 | 60 | 71 | Carbamidomethylation | C4:Carbamidomethylation:1000.00;C8:Carbamidomethylation:1000.00 | PEAKS DB |
| V.SC(+57.02)EQFC(+57.02)PIR.G | Y | 56.95 | 1195.5114 | 9 | 0.1 | 598.7630 | 2 | 31.99 | 1 | 13537 | BuCaMH03.raw | 1.6077E9 | 1 | 1 | 47 | 55 | Carbamidomethylation | C2:Carbamidomethylation:1000.00;C6:Carbamidomethylation:1000.00 | PEAKS DB |
| Q.GC(+57.02)AATC(+57.02)PEFR.S | N | 56.90 | 1167.4801 | 10 | 1.2 | 584.7480 | 2 | 22.89 | 1 | 7277 | BuCaMH03.raw | 5.054E5 | 1 | 1 | 62 | 71 | Carbamidomethylation | C2:Carbamidomethylation:1000.00;C6:Carbamidomethylation:1000.00 | PEAKS DB |
| F.C(+57.02)PIRGPVIEQGC(+57.02)AATC(+57.02)PEFR.S | N | 54.87 | 2317.0764 | 20 | 2.5 | 773.3680 | 3 | 59.71 | 1 | 35597 | BuCaMH03.raw | 1.0593E6 | 1 | 1 | 52 | 71 | Carbamidomethylation | C1:Carbamidomethylation:1000.00;C12:Carbamidomethylation:1000.00;C16:Carbamidomethylation:1000.00 | PEAKS DB |
| S.LLC(+57.02)C(+57.02)TTDNC(+57.02)NH | N | 54.64 | 1406.5377 | 11 | 0.0 | 704.2761 | 2 | 19.18 | 1 | 5296 | BuCaMH03.raw | 2.3152E7 | 1 | 1 | 77 | 87 | Carbamidomethylation | C3:Carbamidomethylation:1000.00;C4:Carbamidomethylation:1000.00;C9:Carbamidomethylation:1000.00 | PEAKS DB |
| total 10 peptides |  |  |  |  |  |  |  |  |  |  |  |  |  |  |  |  |  |  |  |
| --- | --- | --- | --- | --- | --- | --- | --- | --- | --- | --- | --- | --- | --- | --- | --- | --- | --- | --- | --- |

CAA35774.1

back to list

  

| Protein Coverage
| Supporting Peptides
|

Protein Coverage:

Supporting Peptides:

| Peptide | Uniq | -10lgP | Mass | Length | ppm | m/z | z | RT | Fraction | Scan | Source File | Area BuCaMH03\_B17 | #Feature | #Feature BuCaMH03\_B17 | Start | End | PTM | AScore | Found By |
| --- | --- | --- | --- | --- | --- | --- | --- | --- | --- | --- | --- | --- | --- | --- | --- | --- | --- | --- | --- |
| R.GPVIEQGC(+57.02)AATC(+57.02)PEFR.S | N | 122.18 | 1790.8080 | 16 | -0.3 | 896.4110 | 2 | 41.38 | 1 | 20692 | BuCaMH03.raw | 1.6329E9 | 1 | 1 | 56 | 71 | Carbamidomethylation | C8:Carbamidomethylation:1000.00;C12:Carbamidomethylation:1000.00 | PEAKS DB |
| G.PVIEQGC(+57.02)AATC(+57.02)PEFR.S | N | 93.92 | 1733.7865 | 15 | 0.1 | 867.9006 | 2 | 40.50 | 1 | 19840 | BuCaMH03.raw | 1.1911E6 | 1 | 1 | 57 | 71 | Carbamidomethylation | C7:Carbamidomethylation:1000.00;C11:Carbamidomethylation:1000.00 | PEAKS DB |
| P.VIEQGC(+57.02)AATC(+57.02)PEFR.S | N | 88.61 | 1636.7338 | 14 | 0.1 | 819.3742 | 2 | 34.87 | 1 | 15468 | BuCaMH03.raw | 2.1479E9 | 2 | 2 | 58 | 71 | Carbamidomethylation | C6:Carbamidomethylation:1000.00;C10:Carbamidomethylation:1000.00 | PEAKS DB |
| R.SLLC(+57.02)C(+57.02)TTDNC(+57.02)NH | N | 82.48 | 1493.5698 | 12 | 0.1 | 747.7922 | 2 | 27.93 | 1 | 10590 | BuCaMH03.raw | 1.9154E9 | 2 | 2 | 76 | 87 | Carbamidomethylation | C4:Carbamidomethylation:1000.00;C5:Carbamidomethylation:1000.00;C10:Carbamidomethylation:1000.00 | PEAKS DB |
| V.IEQGC(+57.02)AATC(+57.02)PEFR.S | N | 73.44 | 1537.6653 | 13 | 0.2 | 769.8401 | 2 | 30.10 | 1 | 12233 | BuCaMH03.raw | 5.1231E7 | 1 | 1 | 59 | 71 | Carbamidomethylation | C5:Carbamidomethylation:1000.00;C9:Carbamidomethylation:1000.00 | PEAKS DB |
| I.EQGC(+57.02)AATC(+57.02)PEFR.S | N | 67.86 | 1424.5813 | 12 | 0.6 | 713.2983 | 2 | 25.26 | 1 | 8777 | BuCaMH03.raw | 7.8103E6 | 1 | 1 | 60 | 71 | Carbamidomethylation | C4:Carbamidomethylation:1000.00;C8:Carbamidomethylation:1000.00 | PEAKS DB |
| V.SC(+57.02)EQFC(+57.02)PIR.G | Y | 56.95 | 1195.5114 | 9 | 0.1 | 598.7630 | 2 | 31.99 | 1 | 13537 | BuCaMH03.raw | 1.6077E9 | 1 | 1 | 47 | 55 | Carbamidomethylation | C2:Carbamidomethylation:1000.00;C6:Carbamidomethylation:1000.00 | PEAKS DB |
| Q.GC(+57.02)AATC(+57.02)PEFR.S | N | 56.90 | 1167.4801 | 10 | 1.2 | 584.7480 | 2 | 22.89 | 1 | 7277 | BuCaMH03.raw | 5.054E5 | 1 | 1 | 62 | 71 | Carbamidomethylation | C2:Carbamidomethylation:1000.00;C6:Carbamidomethylation:1000.00 | PEAKS DB |
| F.C(+57.02)PIRGPVIEQGC(+57.02)AATC(+57.02)PEFR.S | N | 54.87 | 2317.0764 | 20 | 2.5 | 773.3680 | 3 | 59.71 | 1 | 35597 | BuCaMH03.raw | 1.0593E6 | 1 | 1 | 52 | 71 | Carbamidomethylation | C1:Carbamidomethylation:1000.00;C12:Carbamidomethylation:1000.00;C16:Carbamidomethylation:1000.00 | PEAKS DB |
| S.LLC(+57.02)C(+57.02)TTDNC(+57.02)NH | N | 54.64 | 1406.5377 | 11 | 0.0 | 704.2761 | 2 | 19.18 | 1 | 5296 | BuCaMH03.raw | 2.3152E7 | 1 | 1 | 77 | 87 | Carbamidomethylation | C3:Carbamidomethylation:1000.00;C4:Carbamidomethylation:1000.00;C9:Carbamidomethylation:1000.00 | PEAKS DB |
| total 10 peptides |  |  |  |  |  |  |  |  |  |  |  |  |  |  |  |  |  |  |  |
| --- | --- | --- | --- | --- | --- | --- | --- | --- | --- | --- | --- | --- | --- | --- | --- | --- | --- | --- | --- |

T\_111

back to list

  

| Protein Coverage
| Supporting Peptides
|

Protein Coverage:

Supporting Peptides:

| Peptide | Uniq | -10lgP | Mass | Length | ppm | m/z | z | RT | Fraction | Scan | Source File | Area BuCaMH03\_B17 | #Feature | #Feature BuCaMH03\_B17 | Start | End | PTM | AScore | Found By |
| --- | --- | --- | --- | --- | --- | --- | --- | --- | --- | --- | --- | --- | --- | --- | --- | --- | --- | --- | --- |
| K.HSDSNAFLHLFPDSFR.I | N | 95.96 | 1888.8856 | 16 | 0.0 | 473.2287 | 4 | 62.79 | 1 | 38132 | BuCaMH03.raw | 1.8811E7 | 2 | 2 | 398 | 413 |  |  | PEAKS DB |
| K.TFHGLGVIDWENWRPQWDR.N | N | 82.43 | 2411.1560 | 19 | 0.2 | 804.7261 | 3 | 72.59 | 1 | 46304 | BuCaMH03.raw | 4.6736E7 | 2 | 2 | 143 | 161 |  |  | PEAKS DB |
| R.KHSDSNAFLHLFPDSFR.I | N | 79.67 | 2016.9806 | 17 | -0.3 | 505.2523 | 4 | 55.38 | 1 | 31919 | BuCaMH03.raw | 9.1159E6 | 2 | 2 | 397 | 413 |  |  | PEAKS DB |
| S.DSNAFLHLFPDSFR.I | N | 77.76 | 1664.7947 | 14 | -0.3 | 555.9387 | 3 | 76.68 | 1 | 49658 | BuCaMH03.raw | 9.9155E5 | 1 | 1 | 400 | 413 |  |  | PEAKS DB |
| R.PGGYWGYYLYPDC(+57.02)YNYNYK.K | N | 76.63 | 2455.0256 | 19 | 0.5 | 1228.5208 | 2 | 75.00 | 1 | 48496 | BuCaMH03.raw | 1.505E7 | 1 | 1 | 217 | 235 | Carbamidomethylation | C13:Carbamidomethylation:1000.00 | PEAKS DB |
| K.TFHGLGVIDWENWR.P | N | 73.74 | 1728.8372 | 14 | 0.7 | 577.2867 | 3 | 70.27 | 1 | 44786 | BuCaMH03.raw | 1.9216E7 | 2 | 2 | 143 | 156 |  |  | PEAKS DB |
| T.FHGLGVIDWENWRPQWDR.N | N | 72.06 | 2310.1082 | 18 | 0.7 | 578.5347 | 4 | 72.04 | 1 | 45863 | BuCaMH03.raw | 8.7408E5 | 1 | 1 | 144 | 161 |  |  | PEAKS DB |
| R.NDQLLWLWR.D | N | 63.60 | 1242.6509 | 9 | 0.8 | 622.3332 | 2 | 79.40 | 1 | 51700 | BuCaMH03.raw | 5.3353E7 | 1 | 1 | 253 | 261 |  |  | PEAKS DB |
| K.C(+57.02)PNIEISR.N | N | 60.84 | 987.4808 | 8 | 0.7 | 494.7480 | 2 | 26.63 | 1 | 9851 | BuCaMH03.raw | 7.2279E6 | 1 | 1 | 245 | 252 | Carbamidomethylation | C1:Carbamidomethylation:1000.00 | PEAKS DB |
| K.QLHPQLSEAAIK.R | Y | 60.71 | 1333.7354 | 12 | 0.4 | 667.8752 | 2 | 28.11 | 1 | 10546 | BuCaMH03.raw | 2.4195E6 | 1 | 1 | 179 | 190 |  |  | PEAKS DB |
| H.SDSNAFLHLFPDSFR.I | N | 59.37 | 1751.8267 | 15 | 0.6 | 584.9498 | 3 | 72.39 | 1 | 46085 | BuCaMH03.raw | 4.6921E6 | 1 | 1 | 399 | 413 |  |  | PEAKS DB |
| K.GLYC(+57.02)EEHYK.K | Y | 58.66 | 1197.5125 | 9 | 1.8 | 599.7646 | 2 | 15.62 | 1 | 3255 | BuCaMH03.raw | 0 | 0 | 0 | 453 | 461 | Carbamidomethylation | C4:Carbamidomethylation:1000.00 | PEAKS DB |
| K.IC(+57.02)SHYLC(+57.02)K.R | Y | 56.16 | 1079.4893 | 8 | 0.4 | 540.7521 | 2 | 12.67 | 1 | 1954 | BuCaMH03.raw | 4.476E5 | 1 | 1 | 382 | 389 | Carbamidomethylation | C2:Carbamidomethylation:1000.00;C7:Carbamidomethylation:1000.00 | PEAKS DB |
| K.QLHPQLSEAAIKR.L | Y | 54.60 | 1489.8364 | 13 | 0.5 | 497.6197 | 3 | 21.94 | 1 | 6625 | BuCaMH03.raw | 1.7007E6 | 1 | 1 | 179 | 191 |  |  | PEAKS DB |
| F.HGLGVIDWENWRPQWDR.N | N | 54.37 | 2163.0398 | 17 | 1.0 | 541.7678 | 4 | 67.50 | 1 | 42023 | BuCaMH03.raw | 1.0462E6 | 1 | 1 | 145 | 161 |  |  | PEAKS DB |
| P.MYPNEPFLVFWNAPTTQC(+57.02)QMR.Y | Y | 53.83 | 2629.1917 | 21 | 0.9 | 1315.6042 | 2 | 88.26 | 1 | 58806 | BuCaMH03.raw | 6.7175E6 | 1 | 1 | 48 | 68 | Carbamidomethylation | C18:Carbamidomethylation:1000.00 | PEAKS DB |
| total 16 peptides |  |  |  |  |  |  |  |  |  |  |  |  |  |  |  |  |  |  |  |
| --- | --- | --- | --- | --- | --- | --- | --- | --- | --- | --- | --- | --- | --- | --- | --- | --- | --- | --- | --- |

T\_144

back to list

  

| Protein Coverage
| Supporting Peptides
|

Protein Coverage:

Supporting Peptides:

| Peptide | Uniq | -10lgP | Mass | Length | ppm | m/z | z | RT | Fraction | Scan | Source File | Area BuCaMH03\_B17 | #Feature | #Feature BuCaMH03\_B17 | Start | End | PTM | AScore | Found By |
| --- | --- | --- | --- | --- | --- | --- | --- | --- | --- | --- | --- | --- | --- | --- | --- | --- | --- | --- | --- |
| K.VLLPSFLAAGGDGYYMLR.G | Y | 88.95 | 1942.0022 | 18 | 0.4 | 972.0088 | 2 | 88.51 | 1 | 59043 | BuCaMH03.raw | 7.3409E6 | 2 | 2 | 510 | 527 |  |  | PEAKS DB |
| K.YLGYLNVIFDDK.G | N | 79.95 | 1458.7394 | 12 | 0.2 | 730.3771 | 2 | 80.21 | 1 | 52452 | BuCaMH03.raw | 3.1502E6 | 1 | 1 | 300 | 311 |  |  | PEAKS DB |
| R.HGQGSGELLQVSGIK.V | N | 77.18 | 1508.7947 | 15 | -0.2 | 755.4045 | 2 | 35.50 | 1 | 15903 | BuCaMH03.raw | 9.0487E5 | 1 | 1 | 457 | 471 |  |  | PEAKS DB |
| R.NVLLLDAGDQYQGTVWFNYFK.G | N | 77.09 | 2490.2219 | 21 | 0.7 | 1246.1191 | 2 | 98.05 | 1 | 66828 | BuCaMH03.raw | 1.3741E5 | 1 | 1 | 91 | 111 |  |  | PEAKS DB |
| R.VPTYVPLEMEK.T | N | 71.52 | 1304.6686 | 11 | 0.5 | 653.3419 | 2 | 52.81 | 1 | 29825 | BuCaMH03.raw | 1.5662E6 | 1 | 1 | 496 | 506 |  |  | PEAKS DB |
| R.FHEC(+57.02)NLGNLIC(+57.02)DAVVYNNLR.H | N | 70.15 | 2420.1365 | 20 | 0.4 | 807.7197 | 3 | 72.44 | 1 | 46256 | BuCaMH03.raw | 6.1847E6 | 1 | 1 | 369 | 388 | Carbamidomethylation | C4:Carbamidomethylation:1000.00;C11:Carbamidomethylation:1000.00 | PEAKS DB |
| R.GDSSNHNSGDLDISIVSDYIK.R | Y | 69.90 | 2235.0291 | 21 | -0.3 | 1118.5215 | 2 | 65.18 | 1 | 40220 | BuCaMH03.raw | 4.3844E5 | 1 | 1 | 528 | 548 |  |  | PEAKS DB |
| R.EVVQFMNSLR.Y | N | 58.75 | 1221.6176 | 10 | 0.6 | 611.8164 | 2 | 55.10 | 1 | 31782 | BuCaMH03.raw | 3.5633E6 | 1 | 1 | 114 | 123 |  |  | PEAKS DB |
| R.YDAMALGNHEFDNGLNGLLDPLLK.N | N | 58.04 | 2629.2847 | 24 | 1.2 | 877.4366 | 3 | 86.36 | 1 | 57223 | BuCaMH03.raw | 1.9298E7 | 1 | 1 | 124 | 147 |  |  | PEAKS DB |
| K.ISGYILPYK.I | N | 57.37 | 1052.5906 | 9 | 0.8 | 527.3030 | 2 | 49.87 | 1 | 27392 | BuCaMH03.raw | 3.3176E5 | 1 | 1 | 168 | 176 |  |  | PEAKS DB |
| V.LLPSFLAAGGDGYYMLR.G | Y | 55.79 | 1842.9338 | 17 | 0.3 | 922.4744 | 2 | 82.64 | 1 | 54460 | BuCaMH03.raw | 7.3139E5 | 1 | 1 | 511 | 527 |  |  | PEAKS DB |
| K.VVYDLSQKPGK.R | N | 54.90 | 1232.6764 | 11 | 0.7 | 411.8997 | 3 | 21.73 | 1 | 6594 | BuCaMH03.raw | 0 | 0 | 0 | 472 | 482 |  |  | PEAKS DB |
| K.YLGYLNVIFDDKGK.V | N | 53.57 | 1643.8558 | 14 | 1.1 | 548.9598 | 3 | 67.37 | 1 | 41965 | BuCaMH03.raw | 3.7401E5 | 1 | 1 | 300 | 313 |  |  | PEAKS DB |
| total 13 peptides |  |  |  |  |  |  |  |  |  |  |  |  |  |  |  |  |  |  |  |
| --- | --- | --- | --- | --- | --- | --- | --- | --- | --- | --- | --- | --- | --- | --- | --- | --- | --- | --- | --- |

T\_146

back to list

  

| Protein Coverage
| Supporting Peptides
|

Protein Coverage:

Supporting Peptides:

| Peptide | Uniq | -10lgP | Mass | Length | ppm | m/z | z | RT | Fraction | Scan | Source File | Area BuCaMH03\_B17 | #Feature | #Feature BuCaMH03\_B17 | Start | End | PTM | AScore | Found By |
| --- | --- | --- | --- | --- | --- | --- | --- | --- | --- | --- | --- | --- | --- | --- | --- | --- | --- | --- | --- |
| K.VLLPSFLAAGGDGYYMLR.G | Y | 88.95 | 1942.0022 | 18 | 0.4 | 972.0088 | 2 | 88.51 | 1 | 59043 | BuCaMH03.raw | 7.3409E6 | 2 | 2 | 510 | 527 |  |  | PEAKS DB |
| K.YLGYLNVIFDDK.G | N | 79.95 | 1458.7394 | 12 | 0.2 | 730.3771 | 2 | 80.21 | 1 | 52452 | BuCaMH03.raw | 3.1502E6 | 1 | 1 | 300 | 311 |  |  | PEAKS DB |
| R.HGQGSGELLQVSGIK.V | N | 77.18 | 1508.7947 | 15 | -0.2 | 755.4045 | 2 | 35.50 | 1 | 15903 | BuCaMH03.raw | 9.0487E5 | 1 | 1 | 457 | 471 |  |  | PEAKS DB |
| R.NVLLLDAGDQYQGTVWFNYFK.G | N | 77.09 | 2490.2219 | 21 | 0.7 | 1246.1191 | 2 | 98.05 | 1 | 66828 | BuCaMH03.raw | 1.3741E5 | 1 | 1 | 91 | 111 |  |  | PEAKS DB |
| R.VPTYVPLEMEK.T | N | 71.52 | 1304.6686 | 11 | 0.5 | 653.3419 | 2 | 52.81 | 1 | 29825 | BuCaMH03.raw | 1.5662E6 | 1 | 1 | 496 | 506 |  |  | PEAKS DB |
| R.FHEC(+57.02)NLGNLIC(+57.02)DAVVYNNLR.H | N | 70.15 | 2420.1365 | 20 | 0.4 | 807.7197 | 3 | 72.44 | 1 | 46256 | BuCaMH03.raw | 6.1847E6 | 1 | 1 | 369 | 388 | Carbamidomethylation | C4:Carbamidomethylation:1000.00;C11:Carbamidomethylation:1000.00 | PEAKS DB |
| R.GDSSNHNSGDLDISIVSDYIK.R | Y | 69.90 | 2235.0291 | 21 | -0.3 | 1118.5215 | 2 | 65.18 | 1 | 40220 | BuCaMH03.raw | 4.3844E5 | 1 | 1 | 528 | 548 |  |  | PEAKS DB |
| R.EVVQFMNSLR.Y | N | 58.75 | 1221.6176 | 10 | 0.6 | 611.8164 | 2 | 55.10 | 1 | 31782 | BuCaMH03.raw | 3.5633E6 | 1 | 1 | 114 | 123 |  |  | PEAKS DB |
| R.YDAMALGNHEFDNGLNGLLDPLLK.N | N | 58.04 | 2629.2847 | 24 | 1.2 | 877.4366 | 3 | 86.36 | 1 | 57223 | BuCaMH03.raw | 1.9298E7 | 1 | 1 | 124 | 147 |  |  | PEAKS DB |
| K.ISGYILPYK.I | N | 57.37 | 1052.5906 | 9 | 0.8 | 527.3030 | 2 | 49.87 | 1 | 27392 | BuCaMH03.raw | 3.3176E5 | 1 | 1 | 168 | 176 |  |  | PEAKS DB |
| V.LLPSFLAAGGDGYYMLR.G | Y | 55.79 | 1842.9338 | 17 | 0.3 | 922.4744 | 2 | 82.64 | 1 | 54460 | BuCaMH03.raw | 7.3139E5 | 1 | 1 | 511 | 527 |  |  | PEAKS DB |
| K.VVYDLSQKPGK.R | N | 54.90 | 1232.6764 | 11 | 0.7 | 411.8997 | 3 | 21.73 | 1 | 6594 | BuCaMH03.raw | 0 | 0 | 0 | 472 | 482 |  |  | PEAKS DB |
| K.YLGYLNVIFDDKGK.V | N | 53.57 | 1643.8558 | 14 | 1.1 | 548.9598 | 3 | 67.37 | 1 | 41965 | BuCaMH03.raw | 3.7401E5 | 1 | 1 | 300 | 313 |  |  | PEAKS DB |
| total 13 peptides |  |  |  |  |  |  |  |  |  |  |  |  |  |  |  |  |  |  |  |
| --- | --- | --- | --- | --- | --- | --- | --- | --- | --- | --- | --- | --- | --- | --- | --- | --- | --- | --- | --- |

T\_147

back to list

  

| Protein Coverage
| Supporting Peptides
|

Protein Coverage:

Supporting Peptides:

| Peptide | Uniq | -10lgP | Mass | Length | ppm | m/z | z | RT | Fraction | Scan | Source File | Area BuCaMH03\_B17 | #Feature | #Feature BuCaMH03\_B17 | Start | End | PTM | AScore | Found By |
| --- | --- | --- | --- | --- | --- | --- | --- | --- | --- | --- | --- | --- | --- | --- | --- | --- | --- | --- | --- |
| K.VLLPSFLAAGGDGYYMLR.G | Y | 88.95 | 1942.0022 | 18 | 0.4 | 972.0088 | 2 | 88.51 | 1 | 59043 | BuCaMH03.raw | 7.3409E6 | 2 | 2 | 510 | 527 |  |  | PEAKS DB |
| K.YLGYLNVIFDDK.G | N | 79.95 | 1458.7394 | 12 | 0.2 | 730.3771 | 2 | 80.21 | 1 | 52452 | BuCaMH03.raw | 3.1502E6 | 1 | 1 | 300 | 311 |  |  | PEAKS DB |
| R.HGQGSGELLQVSGIK.V | N | 77.18 | 1508.7947 | 15 | -0.2 | 755.4045 | 2 | 35.50 | 1 | 15903 | BuCaMH03.raw | 9.0487E5 | 1 | 1 | 457 | 471 |  |  | PEAKS DB |
| R.NVLLLDAGDQYQGTVWFNYFK.G | N | 77.09 | 2490.2219 | 21 | 0.7 | 1246.1191 | 2 | 98.05 | 1 | 66828 | BuCaMH03.raw | 1.3741E5 | 1 | 1 | 91 | 111 |  |  | PEAKS DB |
| R.VPTYVPLEMEK.T | N | 71.52 | 1304.6686 | 11 | 0.5 | 653.3419 | 2 | 52.81 | 1 | 29825 | BuCaMH03.raw | 1.5662E6 | 1 | 1 | 496 | 506 |  |  | PEAKS DB |
| R.FHEC(+57.02)NLGNLIC(+57.02)DAVVYNNLR.H | N | 70.15 | 2420.1365 | 20 | 0.4 | 807.7197 | 3 | 72.44 | 1 | 46256 | BuCaMH03.raw | 6.1847E6 | 1 | 1 | 369 | 388 | Carbamidomethylation | C4:Carbamidomethylation:1000.00;C11:Carbamidomethylation:1000.00 | PEAKS DB |
| R.GDSSNHNSGDLDISIVSDYIK.R | Y | 69.90 | 2235.0291 | 21 | -0.3 | 1118.5215 | 2 | 65.18 | 1 | 40220 | BuCaMH03.raw | 4.3844E5 | 1 | 1 | 528 | 548 |  |  | PEAKS DB |
| R.EVVQFMNSLR.Y | N | 58.75 | 1221.6176 | 10 | 0.6 | 611.8164 | 2 | 55.10 | 1 | 31782 | BuCaMH03.raw | 3.5633E6 | 1 | 1 | 114 | 123 |  |  | PEAKS DB |
| R.YDAMALGNHEFDNGLNGLLDPLLK.N | N | 58.04 | 2629.2847 | 24 | 1.2 | 877.4366 | 3 | 86.36 | 1 | 57223 | BuCaMH03.raw | 1.9298E7 | 1 | 1 | 124 | 147 |  |  | PEAKS DB |
| K.ISGYILPYK.I | N | 57.37 | 1052.5906 | 9 | 0.8 | 527.3030 | 2 | 49.87 | 1 | 27392 | BuCaMH03.raw | 3.3176E5 | 1 | 1 | 168 | 176 |  |  | PEAKS DB |
| V.LLPSFLAAGGDGYYMLR.G | Y | 55.79 | 1842.9338 | 17 | 0.3 | 922.4744 | 2 | 82.64 | 1 | 54460 | BuCaMH03.raw | 7.3139E5 | 1 | 1 | 511 | 527 |  |  | PEAKS DB |
| K.VVYDLSQKPGK.R | N | 54.90 | 1232.6764 | 11 | 0.7 | 411.8997 | 3 | 21.73 | 1 | 6594 | BuCaMH03.raw | 0 | 0 | 0 | 472 | 482 |  |  | PEAKS DB |
| K.YLGYLNVIFDDKGK.V | N | 53.57 | 1643.8558 | 14 | 1.1 | 548.9598 | 3 | 67.37 | 1 | 41965 | BuCaMH03.raw | 3.7401E5 | 1 | 1 | 300 | 313 |  |  | PEAKS DB |
| total 13 peptides |  |  |  |  |  |  |  |  |  |  |  |  |  |  |  |  |  |  |  |
| --- | --- | --- | --- | --- | --- | --- | --- | --- | --- | --- | --- | --- | --- | --- | --- | --- | --- | --- | --- |

T\_143

back to list

  

| Protein Coverage
| Supporting Peptides
|

Protein Coverage:

Supporting Peptides:

| Peptide | Uniq | -10lgP | Mass | Length | ppm | m/z | z | RT | Fraction | Scan | Source File | Area BuCaMH03\_B17 | #Feature | #Feature BuCaMH03\_B17 | Start | End | PTM | AScore | Found By |
| --- | --- | --- | --- | --- | --- | --- | --- | --- | --- | --- | --- | --- | --- | --- | --- | --- | --- | --- | --- |
| K.VLLPSFLAAGGDGYYMLR.G | Y | 88.95 | 1942.0022 | 18 | 0.4 | 972.0088 | 2 | 88.51 | 1 | 59043 | BuCaMH03.raw | 7.3409E6 | 2 | 2 | 510 | 527 |  |  | PEAKS DB |
| K.YLGYLNVIFDDK.G | N | 79.95 | 1458.7394 | 12 | 0.2 | 730.3771 | 2 | 80.21 | 1 | 52452 | BuCaMH03.raw | 3.1502E6 | 1 | 1 | 300 | 311 |  |  | PEAKS DB |
| R.HGQGSGELLQVSGIK.V | N | 77.18 | 1508.7947 | 15 | -0.2 | 755.4045 | 2 | 35.50 | 1 | 15903 | BuCaMH03.raw | 9.0487E5 | 1 | 1 | 457 | 471 |  |  | PEAKS DB |
| R.NVLLLDAGDQYQGTVWFNYFK.G | N | 77.09 | 2490.2219 | 21 | 0.7 | 1246.1191 | 2 | 98.05 | 1 | 66828 | BuCaMH03.raw | 1.3741E5 | 1 | 1 | 91 | 111 |  |  | PEAKS DB |
| R.VPTYVPLEMEK.T | N | 71.52 | 1304.6686 | 11 | 0.5 | 653.3419 | 2 | 52.81 | 1 | 29825 | BuCaMH03.raw | 1.5662E6 | 1 | 1 | 496 | 506 |  |  | PEAKS DB |
| R.FHEC(+57.02)NLGNLIC(+57.02)DAVVYNNLR.H | N | 70.15 | 2420.1365 | 20 | 0.4 | 807.7197 | 3 | 72.44 | 1 | 46256 | BuCaMH03.raw | 6.1847E6 | 1 | 1 | 369 | 388 | Carbamidomethylation | C4:Carbamidomethylation:1000.00;C11:Carbamidomethylation:1000.00 | PEAKS DB |
| R.GDSSNHNSGDLDISIVSDYIK.R | Y | 69.90 | 2235.0291 | 21 | -0.3 | 1118.5215 | 2 | 65.18 | 1 | 40220 | BuCaMH03.raw | 4.3844E5 | 1 | 1 | 528 | 548 |  |  | PEAKS DB |
| R.EVVQFMNSLR.Y | N | 58.75 | 1221.6176 | 10 | 0.6 | 611.8164 | 2 | 55.10 | 1 | 31782 | BuCaMH03.raw | 3.5633E6 | 1 | 1 | 114 | 123 |  |  | PEAKS DB |
| R.YDAMALGNHEFDNGLNGLLDPLLK.N | N | 58.04 | 2629.2847 | 24 | 1.2 | 877.4366 | 3 | 86.36 | 1 | 57223 | BuCaMH03.raw | 1.9298E7 | 1 | 1 | 124 | 147 |  |  | PEAKS DB |
| K.ISGYILPYK.I | N | 57.37 | 1052.5906 | 9 | 0.8 | 527.3030 | 2 | 49.87 | 1 | 27392 | BuCaMH03.raw | 3.3176E5 | 1 | 1 | 168 | 176 |  |  | PEAKS DB |
| V.LLPSFLAAGGDGYYMLR.G | Y | 55.79 | 1842.9338 | 17 | 0.3 | 922.4744 | 2 | 82.64 | 1 | 54460 | BuCaMH03.raw | 7.3139E5 | 1 | 1 | 511 | 527 |  |  | PEAKS DB |
| K.VVYDLSQKPGK.R | N | 54.90 | 1232.6764 | 11 | 0.7 | 411.8997 | 3 | 21.73 | 1 | 6594 | BuCaMH03.raw | 0 | 0 | 0 | 472 | 482 |  |  | PEAKS DB |
| K.YLGYLNVIFDDKGK.V | N | 53.57 | 1643.8558 | 14 | 1.1 | 548.9598 | 3 | 67.37 | 1 | 41965 | BuCaMH03.raw | 3.7401E5 | 1 | 1 | 300 | 313 |  |  | PEAKS DB |
| total 13 peptides |  |  |  |  |  |  |  |  |  |  |  |  |  |  |  |  |  |  |  |
| --- | --- | --- | --- | --- | --- | --- | --- | --- | --- | --- | --- | --- | --- | --- | --- | --- | --- | --- | --- |

0402253A

back to list

  

| Protein Coverage
| Supporting Peptides
|

Protein Coverage:

Supporting Peptides:

| Peptide | Uniq | -10lgP | Mass | Length | ppm | m/z | z | RT | Fraction | Scan | Source File | Area BuCaMH03\_B17 | #Feature | #Feature BuCaMH03\_B17 | Start | End | PTM | AScore | Found By |
| --- | --- | --- | --- | --- | --- | --- | --- | --- | --- | --- | --- | --- | --- | --- | --- | --- | --- | --- | --- |
| R.C(+57.02)C(+57.02)YVHDNC(+57.02)YGDAEKK.H | N | 96.62 | 1917.7444 | 15 | -0.1 | 959.8794 | 2 | 12.59 | 1 | 1877 | BuCaMH03.raw | 1.3627E8 | 3 | 3 | 44 | 58 | Carbamidomethylation | C1:Carbamidomethylation:1000.00;C2:Carbamidomethylation:1000.00;C8:Carbamidomethylation:1000.00 | PEAKS DB |
| R.C(+57.02)C(+57.02)YVHDNC(+57.02)YGDAEK.K | N | 90.97 | 1789.6494 | 14 | -0.2 | 895.8318 | 2 | 17.70 | 1 | 4370 | BuCaMH03.raw | 2.9791E8 | 4 | 4 | 44 | 57 | Carbamidomethylation | C1:Carbamidomethylation:1000.00;C2:Carbamidomethylation:1000.00;C8:Carbamidomethylation:1000.00 | PEAKS DB |
| NLINFMEM(+15.99)IR.Y | N | 78.21 | 1295.6366 | 10 | -0.2 | 648.8254 | 2 | 78.80 | 1 | 54312 | BuCaMH03.raw | 4.9087E8 | 4 | 4 | 1 | 10 | Oxidation (M) | M8:Oxidation (M):52.04 | PEAKS DB |
| NLINFM(+15.99)EMIR.Y | N | 74.92 | 1295.6366 | 10 | 0.0 | 648.8256 | 2 | 67.65 | 1 | 42306 | BuCaMH03.raw | 3.5844E8 | 3 | 3 | 1 | 10 | Oxidation (M) | M6:Oxidation (M):32.28 | PEAKS DB |
| NLINFM(+15.99)EM(+15.99)IR.Y | N | 74.44 | 1311.6315 | 10 | 0.7 | 656.8235 | 2 | 83.18 | 1 | 54954 | BuCaMH03.raw | 8.4715E6 | 1 | 1 | 1 | 10 | Oxidation (M) | M6:Oxidation (M):1000.00;M8:Oxidation (M):1000.00 | PEAKS DB |
| C.C(+57.02)YVHDNC(+57.02)YGDAEK.K | N | 73.17 | 1629.6188 | 13 | -0.3 | 544.2134 | 3 | 14.86 | 1 | 2919 | BuCaMH03.raw | 1.0574E7 | 2 | 2 | 45 | 57 | Carbamidomethylation | C1:Carbamidomethylation:1000.00;C7:Carbamidomethylation:1000.00 | PEAKS DB |
| NLINFMEMIR.Y | N | 73.01 | 1279.6417 | 10 | 0.6 | 640.8285 | 2 | 88.88 | 1 | 59453 | BuCaMH03.raw | 1.4319E10 | 1 | 1 | 1 | 10 |  |  | PEAKS DB |
| C.YVHDNC(+57.02)YGDAEK.K | N | 69.88 | 1469.5881 | 12 | 0.7 | 735.8019 | 2 | 12.57 | 1 | 1878 | BuCaMH03.raw | 7.7179E6 | 2 | 2 | 46 | 57 | Carbamidomethylation | C6:Carbamidomethylation:1000.00 | PEAKS DB |
| NLINFMEMIRYTIPC(+57.02)EK.T | N | 61.55 | 2171.0576 | 17 | 0.4 | 724.6934 | 3 | 87.62 | 1 | 58648 | BuCaMH03.raw | 5.837E7 | 2 | 2 | 1 | 17 | Carbamidomethylation | C15:Carbamidomethylation:1000.00 | PEAKS DB |
| R.TIIC(+57.02)YGAAGGTC(+57.02)R.I | Y | 60.68 | 1398.6384 | 13 | -0.2 | 700.3264 | 2 | 37.66 | 1 | 17679 | BuCaMH03.raw | 0 | 0 | 0 | 76 | 88 | Carbamidomethylation | C4:Carbamidomethylation:1000.00;C12:Carbamidomethylation:1000.00 | PEAKS DB |
| C.C(+57.02)YVHDNC(+57.02)YGDAEKK.H | N | 60.63 | 1757.7137 | 14 | -0.5 | 586.9116 | 3 | 12.57 | 1 | 1871 | BuCaMH03.raw | 2.5293E6 | 1 | 1 | 45 | 58 | Carbamidomethylation | C1:Carbamidomethylation:1000.00;C7:Carbamidomethylation:1000.00 | PEAKS DB |
| N.LINFM(+15.99)EMIR.Y | N | 56.49 | 1181.5936 | 9 | 0.4 | 591.8043 | 2 | 64.15 | 1 | 39326 | BuCaMH03.raw | 1.3572E6 | 1 | 1 | 2 | 10 | Oxidation (M) | M5:Oxidation (M):36.05 | PEAKS DB |
| N.LINFMEMIR.Y | N | 56.00 | 1165.5988 | 9 | 0.3 | 583.8068 | 2 | 78.38 | 1 | 51198 | BuCaMH03.raw | 3.6549E8 | 1 | 1 | 2 | 10 |  |  | PEAKS DB |
| N.LINFMEM(+15.99)IR.Y | N | 55.71 | 1181.5936 | 9 | -0.6 | 591.8037 | 2 | 78.37 | 1 | 50945 | BuCaMH03.raw | 5.8354E6 | 2 | 2 | 2 | 10 | Oxidation (M) | M7:Oxidation (M):36.05 | PEAKS DB |
| R.YTIPC(+57.02)EK.T | N | 54.55 | 909.4266 | 7 | 0.4 | 455.7207 | 2 | 24.68 | 1 | 8545 | BuCaMH03.raw | 6.5047E8 | 1 | 1 | 11 | 17 | Carbamidomethylation | C5:Carbamidomethylation:1000.00 | PEAKS DB |
| total 15 peptides |  |  |  |  |  |  |  |  |  |  |  |  |  |  |  |  |  |  |  |
| --- | --- | --- | --- | --- | --- | --- | --- | --- | --- | --- | --- | --- | --- | --- | --- | --- | --- | --- | --- |

0412250A

back to list

  

| Protein Coverage
| Supporting Peptides
|

Protein Coverage:

Supporting Peptides:

| Peptide | Uniq | -10lgP | Mass | Length | ppm | m/z | z | RT | Fraction | Scan | Source File | Area BuCaMH03\_B17 | #Feature | #Feature BuCaMH03\_B17 | Start | End | PTM | AScore | Found By |
| --- | --- | --- | --- | --- | --- | --- | --- | --- | --- | --- | --- | --- | --- | --- | --- | --- | --- | --- | --- |
| R.C(+57.02)C(+57.02)YVHDNC(+57.02)YGDAEKK.H | N | 96.62 | 1917.7444 | 15 | -0.1 | 959.8794 | 2 | 12.59 | 1 | 1877 | BuCaMH03.raw | 1.3627E8 | 3 | 3 | 44 | 58 | Carbamidomethylation | C1:Carbamidomethylation:1000.00;C2:Carbamidomethylation:1000.00;C8:Carbamidomethylation:1000.00 | PEAKS DB |
| R.C(+57.02)C(+57.02)YVHDNC(+57.02)YGDAEK.K | N | 90.97 | 1789.6494 | 14 | -0.2 | 895.8318 | 2 | 17.70 | 1 | 4370 | BuCaMH03.raw | 2.9791E8 | 4 | 4 | 44 | 57 | Carbamidomethylation | C1:Carbamidomethylation:1000.00;C2:Carbamidomethylation:1000.00;C8:Carbamidomethylation:1000.00 | PEAKS DB |
| NLINFMEM(+15.99)IR.Y | N | 78.21 | 1295.6366 | 10 | -0.2 | 648.8254 | 2 | 78.80 | 1 | 54312 | BuCaMH03.raw | 4.9087E8 | 4 | 4 | 1 | 10 | Oxidation (M) | M8:Oxidation (M):52.04 | PEAKS DB |
| NLINFM(+15.99)EMIR.Y | N | 74.92 | 1295.6366 | 10 | 0.0 | 648.8256 | 2 | 67.65 | 1 | 42306 | BuCaMH03.raw | 3.5844E8 | 3 | 3 | 1 | 10 | Oxidation (M) | M6:Oxidation (M):32.28 | PEAKS DB |
| NLINFM(+15.99)EM(+15.99)IR.Y | N | 74.44 | 1311.6315 | 10 | 0.7 | 656.8235 | 2 | 83.18 | 1 | 54954 | BuCaMH03.raw | 8.4715E6 | 1 | 1 | 1 | 10 | Oxidation (M) | M6:Oxidation (M):1000.00;M8:Oxidation (M):1000.00 | PEAKS DB |
| C.C(+57.02)YVHDNC(+57.02)YGDAEK.K | N | 73.17 | 1629.6188 | 13 | -0.3 | 544.2134 | 3 | 14.86 | 1 | 2919 | BuCaMH03.raw | 1.0574E7 | 2 | 2 | 45 | 57 | Carbamidomethylation | C1:Carbamidomethylation:1000.00;C7:Carbamidomethylation:1000.00 | PEAKS DB |
| NLINFMEMIR.Y | N | 73.01 | 1279.6417 | 10 | 0.6 | 640.8285 | 2 | 88.88 | 1 | 59453 | BuCaMH03.raw | 1.4319E10 | 1 | 1 | 1 | 10 |  |  | PEAKS DB |
| C.YVHDNC(+57.02)YGDAEK.K | N | 69.88 | 1469.5881 | 12 | 0.7 | 735.8019 | 2 | 12.57 | 1 | 1878 | BuCaMH03.raw | 7.7179E6 | 2 | 2 | 46 | 57 | Carbamidomethylation | C6:Carbamidomethylation:1000.00 | PEAKS DB |
| NLINFMEMIRYTIPC(+57.02)EK.T | N | 61.55 | 2171.0576 | 17 | 0.4 | 724.6934 | 3 | 87.62 | 1 | 58648 | BuCaMH03.raw | 5.837E7 | 2 | 2 | 1 | 17 | Carbamidomethylation | C15:Carbamidomethylation:1000.00 | PEAKS DB |
| R.TIIC(+57.02)YGAAGGTC(+57.02)R.I | Y | 60.68 | 1398.6384 | 13 | -0.2 | 700.3264 | 2 | 37.66 | 1 | 17679 | BuCaMH03.raw | 0 | 0 | 0 | 76 | 88 | Carbamidomethylation | C4:Carbamidomethylation:1000.00;C12:Carbamidomethylation:1000.00 | PEAKS DB |
| C.C(+57.02)YVHDNC(+57.02)YGDAEKK.H | N | 60.63 | 1757.7137 | 14 | -0.5 | 586.9116 | 3 | 12.57 | 1 | 1871 | BuCaMH03.raw | 2.5293E6 | 1 | 1 | 45 | 58 | Carbamidomethylation | C1:Carbamidomethylation:1000.00;C7:Carbamidomethylation:1000.00 | PEAKS DB |
| N.LINFM(+15.99)EMIR.Y | N | 56.49 | 1181.5936 | 9 | 0.4 | 591.8043 | 2 | 64.15 | 1 | 39326 | BuCaMH03.raw | 1.3572E6 | 1 | 1 | 2 | 10 | Oxidation (M) | M5:Oxidation (M):36.05 | PEAKS DB |
| N.LINFMEMIR.Y | N | 56.00 | 1165.5988 | 9 | 0.3 | 583.8068 | 2 | 78.38 | 1 | 51198 | BuCaMH03.raw | 3.6549E8 | 1 | 1 | 2 | 10 |  |  | PEAKS DB |
| N.LINFMEM(+15.99)IR.Y | N | 55.71 | 1181.5936 | 9 | -0.6 | 591.8037 | 2 | 78.37 | 1 | 50945 | BuCaMH03.raw | 5.8354E6 | 2 | 2 | 2 | 10 | Oxidation (M) | M7:Oxidation (M):36.05 | PEAKS DB |
| R.YTIPC(+57.02)EK.T | N | 54.55 | 909.4266 | 7 | 0.4 | 455.7207 | 2 | 24.68 | 1 | 8545 | BuCaMH03.raw | 6.5047E8 | 1 | 1 | 11 | 17 | Carbamidomethylation | C5:Carbamidomethylation:1000.00 | PEAKS DB |
| total 15 peptides |  |  |  |  |  |  |  |  |  |  |  |  |  |  |  |  |  |  |  |
| --- | --- | --- | --- | --- | --- | --- | --- | --- | --- | --- | --- | --- | --- | --- | --- | --- | --- | --- | --- |

T\_12

back to list

  

| Protein Coverage
| Supporting Peptides
|

Protein Coverage:

Supporting Peptides:

| Peptide | Uniq | -10lgP | Mass | Length | ppm | m/z | z | RT | Fraction | Scan | Source File | Area BuCaMH03\_B17 | #Feature | #Feature BuCaMH03\_B17 | Start | End | PTM | AScore | Found By |
| --- | --- | --- | --- | --- | --- | --- | --- | --- | --- | --- | --- | --- | --- | --- | --- | --- | --- | --- | --- |
| I.VLGDPDNGMVEPGTK.C | Y | 90.53 | 1527.7239 | 15 | 0.2 | 764.8694 | 2 | 37.98 | 1 | 17950 | BuCaMH03.raw | 3.0399E6 | 1 | 1 | 164 | 178 |  |  | PEAKS DB |
| K.C(+57.02)PTLTNQC(+57.02)IDLWGPGAK.E | Y | 86.02 | 1929.9077 | 17 | -0.8 | 965.9603 | 2 | 83.33 | 1 | 55002 | BuCaMH03.raw | 2.3497E7 | 3 | 3 | 95 | 111 | Carbamidomethylation | C1:Carbamidomethylation:1000.00;C8:Carbamidomethylation:1000.00 | PEAKS DB |
| K.ESPDTC(+57.02)FLLNHISQGC(+57.02)GFC(+57.02)R.M | Y | 85.77 | 2397.0300 | 20 | 1.2 | 800.0182 | 3 | 60.59 | 1 | 36289 | BuCaMH03.raw | 4.5929E7 | 1 | 1 | 112 | 131 | Carbamidomethylation | C6:Carbamidomethylation:1000.00;C16:Carbamidomethylation:1000.00;C19:Carbamidomethylation:1000.00 | PEAKS DB |
| K.IVLGDPDNGMVEPGTK.C | Y | 82.84 | 1640.8080 | 16 | 0.3 | 821.4115 | 2 | 47.44 | 1 | 25271 | BuCaMH03.raw | 2.0532E7 | 1 | 1 | 163 | 178 |  |  | PEAKS DB |
| K.IVLGDPDNGM(+15.99)VEPGTK.C | Y | 81.83 | 1656.8029 | 16 | 0.6 | 829.4092 | 2 | 47.47 | 1 | 25344 | BuCaMH03.raw | 1.1917E6 | 1 | 1 | 163 | 178 | Oxidation (M) | M10:Oxidation (M):1000.00 | PEAKS DB |
| P.DTC(+57.02)FLLNHISQGC(+57.02)GFC(+57.02)R.M | Y | 76.61 | 2083.9026 | 17 | 0.1 | 695.6415 | 3 | 59.22 | 1 | 35196 | BuCaMH03.raw | 5.8212E5 | 1 | 1 | 115 | 131 | Carbamidomethylation | C3:Carbamidomethylation:1000.00;C13:Carbamidomethylation:1000.00;C16:Carbamidomethylation:1000.00 | PEAKS DB |
| E.SPDTC(+57.02)FLLNHISQGC(+57.02)GFC(+57.02)R.M | Y | 68.48 | 2267.9873 | 19 | -0.1 | 757.0030 | 3 | 58.69 | 1 | 34768 | BuCaMH03.raw | 2.3221E6 | 1 | 1 | 113 | 131 | Carbamidomethylation | C5:Carbamidomethylation:1000.00;C15:Carbamidomethylation:1000.00;C18:Carbamidomethylation:1000.00 | PEAKS DB |
| K.DDC(+57.02)DLPER.C | N | 56.58 | 1018.4025 | 8 | 0.5 | 510.2088 | 2 | 20.35 | 1 | 5679 | BuCaMH03.raw | 2.5284E6 | 1 | 1 | 56 | 63 | Carbamidomethylation | C3:Carbamidomethylation:1000.00 | PEAKS DB |
| total 8 peptides |  |  |  |  |  |  |  |  |  |  |  |  |  |  |  |  |  |  |  |
| --- | --- | --- | --- | --- | --- | --- | --- | --- | --- | --- | --- | --- | --- | --- | --- | --- | --- | --- | --- |

XP\_026559083.1

back to list

  

| Protein Coverage
| Supporting Peptides
|

Protein Coverage:

Supporting Peptides:

| Peptide | Uniq | -10lgP | Mass | Length | ppm | m/z | z | RT | Fraction | Scan | Source File | Area BuCaMH03\_B17 | #Feature | #Feature BuCaMH03\_B17 | Start | End | PTM | AScore | Found By |
| --- | --- | --- | --- | --- | --- | --- | --- | --- | --- | --- | --- | --- | --- | --- | --- | --- | --- | --- | --- |
| R.EVALDMSQFENIAVR.R | N | 103.31 | 1720.8453 | 15 | 0.1 | 861.4301 | 2 | 73.22 | 1 | 46870 | BuCaMH03.raw | 8.4278E5 | 1 | 1 | 207 | 221 |  |  | PEAKS DB |
| R.LSALKDFVSVLVQHFAGR.L | N | 99.00 | 1986.1050 | 18 | 0.5 | 663.0426 | 3 | 87.38 | 1 | 58049 | BuCaMH03.raw | 6.5502E6 | 2 | 2 | 333 | 350 |  |  | PEAKS DB |
| I.YMADIESAVLYSLR.I | N | 60.87 | 1629.8073 | 14 | 0.2 | 815.9111 | 2 | 81.59 | 1 | 53679 | BuCaMH03.raw | 5.1598E5 | 1 | 1 | 306 | 319 |  |  | PEAKS DB |
| K.IYMADIESAVLYSLR.I | N | 59.73 | 1742.8912 | 15 | 0.4 | 872.4532 | 2 | 90.36 | 1 | 60545 | BuCaMH03.raw | 7.4102E5 | 1 | 1 | 305 | 319 |  |  | PEAKS DB |
| K.AFAPLWK.G | Y | 54.26 | 831.4643 | 7 | -0.5 | 416.7392 | 2 | 52.75 | 1 | 29875 | BuCaMH03.raw | 4.7046E3 | 1 | 1 | 79 | 85 |  |  | PEAKS DB |
| total 5 peptides |  |  |  |  |  |  |  |  |  |  |  |  |  |  |  |  |  |  |  |
| --- | --- | --- | --- | --- | --- | --- | --- | --- | --- | --- | --- | --- | --- | --- | --- | --- | --- | --- | --- |

T\_16

back to list

  

| Protein Coverage
| Supporting Peptides
|

Protein Coverage:

Supporting Peptides:

| Peptide | Uniq | -10lgP | Mass | Length | ppm | m/z | z | RT | Fraction | Scan | Source File | Area BuCaMH03\_B17 | #Feature | #Feature BuCaMH03\_B17 | Start | End | PTM | AScore | Found By |
| --- | --- | --- | --- | --- | --- | --- | --- | --- | --- | --- | --- | --- | --- | --- | --- | --- | --- | --- | --- |
| K.DRPPC(+57.02)ILNKPLSK.N | Y | 83.20 | 1536.8446 | 13 | -1.0 | 385.2180 | 4 | 25.81 | 1 | 9117 | BuCaMH03.raw | 5.7608E7 | 2 | 2 | 108 | 120 | Carbamidomethylation | C5:Carbamidomethylation:1000.00 | PEAKS DB |
| C.LMYESISDEPHSEFSSC(+57.02)SVQEHQR.Y | N | 78.99 | 2881.2283 | 24 | -0.2 | 961.4165 | 3 | 39.09 | 1 | 18706 | BuCaMH03.raw | 1.8302E7 | 2 | 2 | 80 | 103 | Carbamidomethylation | C17:Carbamidomethylation:1000.00 | PEAKS DB |
| L.MYESISDEPHSEFSSC(+57.02)SVQEHQR.Y | N | 75.75 | 2768.1443 | 23 | -0.6 | 693.0429 | 4 | 32.28 | 1 | 13474 | BuCaMH03.raw | 2.6278E7 | 2 | 2 | 81 | 103 | Carbamidomethylation | C16:Carbamidomethylation:1000.00 | PEAKS DB |
| K.KSVAVVQDYSK.R | N | 75.23 | 1222.6558 | 11 | -0.2 | 612.3350 | 2 | 15.59 | 1 | 3167 | BuCaMH03.raw | 4.4932E6 | 2 | 2 | 36 | 46 |  |  | PEAKS DB |
| M.YESISDEPHSEFSSC(+57.02)SVQEHQR.Y | N | 60.65 | 2637.1038 | 22 | 0.9 | 660.2838 | 4 | 28.14 | 1 | 10623 | BuCaMH03.raw | 8.8375E5 | 1 | 1 | 82 | 103 | Carbamidomethylation | C15:Carbamidomethylation:1000.00 | PEAKS DB |
| K.SVAVVQDYSK.R | N | 60.55 | 1094.5608 | 10 | -0.5 | 548.2874 | 2 | 26.31 | 1 | 9703 | BuCaMH03.raw | 3.8359E7 | 1 | 1 | 37 | 46 |  |  | PEAKS DB |
| K.DDC(+57.02)DLPER.C | N | 56.58 | 1018.4025 | 8 | 0.5 | 510.2088 | 2 | 20.35 | 1 | 5679 | BuCaMH03.raw | 2.5284E6 | 1 | 1 | 188 | 195 | Carbamidomethylation | C3:Carbamidomethylation:1000.00 | PEAKS DB |
| total 7 peptides |  |  |  |  |  |  |  |  |  |  |  |  |  |  |  |  |  |  |  |
| --- | --- | --- | --- | --- | --- | --- | --- | --- | --- | --- | --- | --- | --- | --- | --- | --- | --- | --- | --- |

CAP74383.1

back to list

  

| Protein Coverage
| Supporting Peptides
|

Protein Coverage:

Supporting Peptides:

| Peptide | Uniq | -10lgP | Mass | Length | ppm | m/z | z | RT | Fraction | Scan | Source File | Area BuCaMH03\_B17 | #Feature | #Feature BuCaMH03\_B17 | Start | End | PTM | AScore | Found By |
| --- | --- | --- | --- | --- | --- | --- | --- | --- | --- | --- | --- | --- | --- | --- | --- | --- | --- | --- | --- |
| K.C(+57.02)IEFSYGGC(+57.02)GGNANNFK.S | Y | 109.55 | 1893.7773 | 17 | 0.5 | 947.8964 | 2 | 48.13 | 1 | 25971 | BuCaMH03.raw | 2.7802E9 | 12 | 12 | 56 | 72 | Carbamidomethylation | C1:Carbamidomethylation:1000.00;C9:Carbamidomethylation:1000.00 | PEAKS DB |
| F.SYGGC(+57.02)GGNANNFK.S | Y | 87.30 | 1344.5516 | 13 | 0.5 | 673.2834 | 2 | 16.40 | 1 | 3550 | BuCaMH03.raw | 2.5827E7 | 1 | 1 | 60 | 72 | Carbamidomethylation | C5:Carbamidomethylation:1000.00 | PEAKS DB |
| C.IEFSYGGC(+57.02)GGNANNFK.S | Y | 83.14 | 1733.7467 | 16 | 0.1 | 867.8807 | 2 | 45.39 | 1 | 23967 | BuCaMH03.raw | 8.889E7 | 2 | 2 | 57 | 72 | Carbamidomethylation | C8:Carbamidomethylation:1000.00 | PEAKS DB |
| S.YGGC(+57.02)GGNANNFK.S | N | 75.97 | 1257.5197 | 12 | 0.4 | 629.7673 | 2 | 13.72 | 1 | 2615 | BuCaMH03.raw | 8.8737E6 | 1 | 1 | 61 | 72 | Carbamidomethylation | C4:Carbamidomethylation:1000.00 | PEAKS DB |
| total 4 peptides |  |  |  |  |  |  |  |  |  |  |  |  |  |  |  |  |  |  |  |
| --- | --- | --- | --- | --- | --- | --- | --- | --- | --- | --- | --- | --- | --- | --- | --- | --- | --- | --- | --- |

B4ESA4.1

back to list

  

| Protein Coverage
| Supporting Peptides
|

Protein Coverage:

Supporting Peptides:

| Peptide | Uniq | -10lgP | Mass | Length | ppm | m/z | z | RT | Fraction | Scan | Source File | Area BuCaMH03\_B17 | #Feature | #Feature BuCaMH03\_B17 | Start | End | PTM | AScore | Found By |
| --- | --- | --- | --- | --- | --- | --- | --- | --- | --- | --- | --- | --- | --- | --- | --- | --- | --- | --- | --- |
| K.C(+57.02)IEFSYGGC(+57.02)GGNANNFK.S | Y | 109.55 | 1893.7773 | 17 | 0.5 | 947.8964 | 2 | 48.13 | 1 | 25971 | BuCaMH03.raw | 2.7802E9 | 12 | 12 | 56 | 72 | Carbamidomethylation | C1:Carbamidomethylation:1000.00;C9:Carbamidomethylation:1000.00 | PEAKS DB |
| F.SYGGC(+57.02)GGNANNFK.S | Y | 87.30 | 1344.5516 | 13 | 0.5 | 673.2834 | 2 | 16.40 | 1 | 3550 | BuCaMH03.raw | 2.5827E7 | 1 | 1 | 60 | 72 | Carbamidomethylation | C5:Carbamidomethylation:1000.00 | PEAKS DB |
| C.IEFSYGGC(+57.02)GGNANNFK.S | Y | 83.14 | 1733.7467 | 16 | 0.1 | 867.8807 | 2 | 45.39 | 1 | 23967 | BuCaMH03.raw | 8.889E7 | 2 | 2 | 57 | 72 | Carbamidomethylation | C8:Carbamidomethylation:1000.00 | PEAKS DB |
| S.YGGC(+57.02)GGNANNFK.S | N | 75.97 | 1257.5197 | 12 | 0.4 | 629.7673 | 2 | 13.72 | 1 | 2615 | BuCaMH03.raw | 8.8737E6 | 1 | 1 | 61 | 72 | Carbamidomethylation | C4:Carbamidomethylation:1000.00 | PEAKS DB |
| total 4 peptides |  |  |  |  |  |  |  |  |  |  |  |  |  |  |  |  |  |  |  |
| --- | --- | --- | --- | --- | --- | --- | --- | --- | --- | --- | --- | --- | --- | --- | --- | --- | --- | --- | --- |

XP\_026571757.1

back to list

  

| Protein Coverage
| Supporting Peptides
|

Protein Coverage:

Supporting Peptides:

| Peptide | Uniq | -10lgP | Mass | Length | ppm | m/z | z | RT | Fraction | Scan | Source File | Area BuCaMH03\_B17 | #Feature | #Feature BuCaMH03\_B17 | Start | End | PTM | AScore | Found By |
| --- | --- | --- | --- | --- | --- | --- | --- | --- | --- | --- | --- | --- | --- | --- | --- | --- | --- | --- | --- |
| K.NGPVEGAFTVYSDFLMYK.S | Y | 96.86 | 2036.9553 | 18 | 0.2 | 1019.4852 | 2 | 87.81 | 1 | 58471 | BuCaMH03.raw | 5.2409E6 | 1 | 1 | 247 | 264 |  |  | PEAKS DB |
| R.FEFAGDLVLPQNFDSR.Q | Y | 88.64 | 1853.8948 | 16 | 0.5 | 927.9551 | 2 | 79.13 | 1 | 51518 | BuCaMH03.raw | 1.5339E7 | 1 | 1 | 72 | 87 |  |  | PEAKS DB |
| K.GLVSGGLYDSHVGC(+57.02)RPY.S | Y | 64.27 | 1835.8624 | 17 | 0.2 | 612.9615 | 3 | 43.97 | 1 | 22620 | BuCaMH03.raw | 8.6384E5 | 1 | 1 | 166 | 182 | Carbamidomethylation | C14:Carbamidomethylation:1000.00 | PEAKS DB |
| R.DQGSC(+57.02)GSC(+57.02)WAFGAVEAMSDR.V | Y | 59.94 | 2189.8564 | 20 | 0.3 | 1095.9358 | 2 | 73.83 | 1 | 47278 | BuCaMH03.raw | 1.5931E6 | 1 | 1 | 101 | 120 | Carbamidomethylation | C5:Carbamidomethylation:1000.00;C8:Carbamidomethylation:1000.00 | PEAKS DB |
| L.C(+57.02)GTFLHGPK.L | Y | 56.81 | 1015.4909 | 9 | 0.3 | 508.7529 | 2 | 17.26 | 1 | 3956 | BuCaMH03.raw | 2.5885E5 | 1 | 1 | 59 | 67 | Carbamidomethylation | C1:Carbamidomethylation:1000.00 | PEAKS DB |
| total 5 peptides |  |  |  |  |  |  |  |  |  |  |  |  |  |  |  |  |  |  |  |
| --- | --- | --- | --- | --- | --- | --- | --- | --- | --- | --- | --- | --- | --- | --- | --- | --- | --- | --- | --- |

XP\_026555793.1

back to list

  

| Protein Coverage
| Supporting Peptides
|

Protein Coverage:

Supporting Peptides:

| Peptide | Uniq | -10lgP | Mass | Length | ppm | m/z | z | RT | Fraction | Scan | Source File | Area BuCaMH03\_B17 | #Feature | #Feature BuCaMH03\_B17 | Start | End | PTM | AScore | Found By |
| --- | --- | --- | --- | --- | --- | --- | --- | --- | --- | --- | --- | --- | --- | --- | --- | --- | --- | --- | --- |
| K.MQIWLMHDIGGPQR.E | Y | 87.05 | 1680.8229 | 14 | 0.2 | 841.4189 | 2 | 59.44 | 1 | 35311 | BuCaMH03.raw | 2.1166E7 | 2 | 2 | 241 | 254 |  |  | PEAKS DB |
| K.SSIFGSVEIVNLNPEK.V | N | 79.12 | 1731.9043 | 16 | 2.2 | 866.9613 | 2 | 72.81 | 1 | 46448 | BuCaMH03.raw | 4.2647E7 | 1 | 1 | 222 | 237 |  |  | PEAKS DB |
| M.QIWLMHDIGGPQR.E | Y | 76.99 | 1549.7823 | 13 | 0.1 | 775.8986 | 2 | 51.22 | 1 | 28527 | BuCaMH03.raw | 1.7276E7 | 2 | 2 | 242 | 254 |  |  | PEAKS DB |
| R.KSSIFGSVEIVNLNPEK.V | N | 70.79 | 1859.9993 | 17 | 1.1 | 931.0079 | 2 | 59.81 | 1 | 35636 | BuCaMH03.raw | 2.1478E6 | 1 | 1 | 221 | 237 |  |  | PEAKS DB |
| R.ETC(+57.02)TGHSIAQLR.E | Y | 61.00 | 1371.6565 | 12 | 0.9 | 458.2265 | 3 | 18.72 | 1 | 4728 | BuCaMH03.raw | 1.1618E7 | 2 | 2 | 255 | 266 | Carbamidomethylation | C3:Carbamidomethylation:1000.00 | PEAKS DB |
| Q.IWLMHDIGGPQR.E | Y | 59.67 | 1421.7238 | 12 | -0.7 | 474.9149 | 3 | 49.30 | 1 | 26852 | BuCaMH03.raw | 2.4159E6 | 1 | 1 | 243 | 254 |  |  | PEAKS DB |
| E.TC(+57.02)TGHSIAQLR.E | Y | 56.92 | 1242.6139 | 11 | -0.6 | 415.2117 | 3 | 14.86 | 1 | 3039 | BuCaMH03.raw | 1.9581E6 | 1 | 1 | 256 | 266 | Carbamidomethylation | C2:Carbamidomethylation:1000.00 | PEAKS DB |
| T.C(+57.02)TGHSIAQLR.E | N | 53.80 | 1141.5662 | 10 | -0.1 | 381.5293 | 3 | 12.93 | 1 | 2105 | BuCaMH03.raw | 6.8899E4 | 1 | 1 | 257 | 266 | Carbamidomethylation | C1:Carbamidomethylation:1000.00 | PEAKS DB |
| total 8 peptides |  |  |  |  |  |  |  |  |  |  |  |  |  |  |  |  |  |  |  |
| --- | --- | --- | --- | --- | --- | --- | --- | --- | --- | --- | --- | --- | --- | --- | --- | --- | --- | --- | --- |

AAL30054.1

back to list

  

| Protein Coverage
| Supporting Peptides
|

Protein Coverage:

Supporting Peptides:

| Peptide | Uniq | -10lgP | Mass | Length | ppm | m/z | z | RT | Fraction | Scan | Source File | Area BuCaMH03\_B17 | #Feature | #Feature BuCaMH03\_B17 | Start | End | PTM | AScore | Found By |
| --- | --- | --- | --- | --- | --- | --- | --- | --- | --- | --- | --- | --- | --- | --- | --- | --- | --- | --- | --- |
| P.VIEQGC(+57.02)VATC(+57.02)PQFR.S | Y | 82.95 | 1663.7810 | 14 | 0.4 | 832.8981 | 2 | 37.98 | 1 | 18196 | BuCaMH03.raw | 8.891E7 | 1 | 1 | 58 | 71 | Carbamidomethylation | C6:Carbamidomethylation:1000.00;C10:Carbamidomethylation:1000.00 | PEAKS DB |
| R.SLLC(+57.02)C(+57.02)TTDNC(+57.02)NH | N | 82.48 | 1493.5698 | 12 | 0.1 | 747.7922 | 2 | 27.93 | 1 | 10590 | BuCaMH03.raw | 1.9154E9 | 2 | 2 | 76 | 87 | Carbamidomethylation | C4:Carbamidomethylation:1000.00;C5:Carbamidomethylation:1000.00;C10:Carbamidomethylation:1000.00 | PEAKS DB |
| R.TC(+57.02)LISPSSTPQTC(+57.02)PQGQDIC(+57.02)FLK.A | Y | 74.64 | 2637.2236 | 23 | 0.3 | 1319.6195 | 2 | 62.25 | 1 | 37664 | BuCaMH03.raw | 1.5001E8 | 2 | 2 | 23 | 45 | Carbamidomethylation | C2:Carbamidomethylation:1000.00;C13:Carbamidomethylation:1000.00;C20:Carbamidomethylation:1000.00 | PEAKS DB |
| V.IEQGC(+57.02)VATC(+57.02)PQFR.S | Y | 74.26 | 1564.7126 | 13 | -0.6 | 783.3632 | 2 | 34.05 | 1 | 14772 | BuCaMH03.raw | 1.3902E7 | 1 | 1 | 59 | 71 | Carbamidomethylation | C5:Carbamidomethylation:1000.00;C9:Carbamidomethylation:1000.00 | PEAKS DB |
| T.RTC(+57.02)LISPSSTPQTC(+57.02)PQGQDIC(+57.02)FLK.A | Y | 60.08 | 2793.3247 | 24 | 0.1 | 932.1156 | 3 | 52.60 | 1 | 29613 | BuCaMH03.raw | 1.7261E8 | 1 | 1 | 22 | 45 | Carbamidomethylation | C3:Carbamidomethylation:1000.00;C14:Carbamidomethylation:1000.00;C21:Carbamidomethylation:1000.00 | PEAKS DB |
| L.ISPSSTPQTC(+57.02)PQGQDIC(+57.02)FLK.A | Y | 58.97 | 2263.0613 | 20 | -0.5 | 1132.5374 | 2 | 52.19 | 1 | 29233 | BuCaMH03.raw | 1.7471E7 | 2 | 2 | 26 | 45 | Carbamidomethylation | C10:Carbamidomethylation:1000.00;C17:Carbamidomethylation:1000.00 | PEAKS DB |
| C.LISPSSTPQTC(+57.02)PQGQDIC(+57.02)FLK.A | Y | 54.83 | 2376.1453 | 21 | 0.1 | 793.0558 | 3 | 58.09 | 1 | 34215 | BuCaMH03.raw | 2.684E6 | 1 | 1 | 25 | 45 | Carbamidomethylation | C11:Carbamidomethylation:1000.00;C18:Carbamidomethylation:1000.00 | PEAKS DB |
| S.LLC(+57.02)C(+57.02)TTDNC(+57.02)NH | N | 54.64 | 1406.5377 | 11 | 0.0 | 704.2761 | 2 | 19.18 | 1 | 5296 | BuCaMH03.raw | 2.3152E7 | 1 | 1 | 77 | 87 | Carbamidomethylation | C3:Carbamidomethylation:1000.00;C4:Carbamidomethylation:1000.00;C9:Carbamidomethylation:1000.00 | PEAKS DB |
| total 8 peptides |  |  |  |  |  |  |  |  |  |  |  |  |  |  |  |  |  |  |  |
| --- | --- | --- | --- | --- | --- | --- | --- | --- | --- | --- | --- | --- | --- | --- | --- | --- | --- | --- | --- |

Q8AY56.1

back to list

  

| Protein Coverage
| Supporting Peptides
|

Protein Coverage:

Supporting Peptides:

| Peptide | Uniq | -10lgP | Mass | Length | ppm | m/z | z | RT | Fraction | Scan | Source File | Area BuCaMH03\_B17 | #Feature | #Feature BuCaMH03\_B17 | Start | End | PTM | AScore | Found By |
| --- | --- | --- | --- | --- | --- | --- | --- | --- | --- | --- | --- | --- | --- | --- | --- | --- | --- | --- | --- |
| P.VIEQGC(+57.02)VATC(+57.02)PQFR.S | Y | 82.95 | 1663.7810 | 14 | 0.4 | 832.8981 | 2 | 37.98 | 1 | 18196 | BuCaMH03.raw | 8.891E7 | 1 | 1 | 58 | 71 | Carbamidomethylation | C6:Carbamidomethylation:1000.00;C10:Carbamidomethylation:1000.00 | PEAKS DB |
| R.SLLC(+57.02)C(+57.02)TTDNC(+57.02)NH | N | 82.48 | 1493.5698 | 12 | 0.1 | 747.7922 | 2 | 27.93 | 1 | 10590 | BuCaMH03.raw | 1.9154E9 | 2 | 2 | 76 | 87 | Carbamidomethylation | C4:Carbamidomethylation:1000.00;C5:Carbamidomethylation:1000.00;C10:Carbamidomethylation:1000.00 | PEAKS DB |
| R.TC(+57.02)LISPSSTPQTC(+57.02)PQGQDIC(+57.02)FLK.A | Y | 74.64 | 2637.2236 | 23 | 0.3 | 1319.6195 | 2 | 62.25 | 1 | 37664 | BuCaMH03.raw | 1.5001E8 | 2 | 2 | 23 | 45 | Carbamidomethylation | C2:Carbamidomethylation:1000.00;C13:Carbamidomethylation:1000.00;C20:Carbamidomethylation:1000.00 | PEAKS DB |
| V.IEQGC(+57.02)VATC(+57.02)PQFR.S | Y | 74.26 | 1564.7126 | 13 | -0.6 | 783.3632 | 2 | 34.05 | 1 | 14772 | BuCaMH03.raw | 1.3902E7 | 1 | 1 | 59 | 71 | Carbamidomethylation | C5:Carbamidomethylation:1000.00;C9:Carbamidomethylation:1000.00 | PEAKS DB |
| T.RTC(+57.02)LISPSSTPQTC(+57.02)PQGQDIC(+57.02)FLK.A | Y | 60.08 | 2793.3247 | 24 | 0.1 | 932.1156 | 3 | 52.60 | 1 | 29613 | BuCaMH03.raw | 1.7261E8 | 1 | 1 | 22 | 45 | Carbamidomethylation | C3:Carbamidomethylation:1000.00;C14:Carbamidomethylation:1000.00;C21:Carbamidomethylation:1000.00 | PEAKS DB |
| L.ISPSSTPQTC(+57.02)PQGQDIC(+57.02)FLK.A | Y | 58.97 | 2263.0613 | 20 | -0.5 | 1132.5374 | 2 | 52.19 | 1 | 29233 | BuCaMH03.raw | 1.7471E7 | 2 | 2 | 26 | 45 | Carbamidomethylation | C10:Carbamidomethylation:1000.00;C17:Carbamidomethylation:1000.00 | PEAKS DB |
| C.LISPSSTPQTC(+57.02)PQGQDIC(+57.02)FLK.A | Y | 54.83 | 2376.1453 | 21 | 0.1 | 793.0558 | 3 | 58.09 | 1 | 34215 | BuCaMH03.raw | 2.684E6 | 1 | 1 | 25 | 45 | Carbamidomethylation | C11:Carbamidomethylation:1000.00;C18:Carbamidomethylation:1000.00 | PEAKS DB |
| S.LLC(+57.02)C(+57.02)TTDNC(+57.02)NH | N | 54.64 | 1406.5377 | 11 | 0.0 | 704.2761 | 2 | 19.18 | 1 | 5296 | BuCaMH03.raw | 2.3152E7 | 1 | 1 | 77 | 87 | Carbamidomethylation | C3:Carbamidomethylation:1000.00;C4:Carbamidomethylation:1000.00;C9:Carbamidomethylation:1000.00 | PEAKS DB |
| total 8 peptides |  |  |  |  |  |  |  |  |  |  |  |  |  |  |  |  |  |  |  |
| --- | --- | --- | --- | --- | --- | --- | --- | --- | --- | --- | --- | --- | --- | --- | --- | --- | --- | --- | --- |

ETE59846.1

back to list

  

| Protein Coverage
| Supporting Peptides
|

Protein Coverage:

Supporting Peptides:

| Peptide | Uniq | -10lgP | Mass | Length | ppm | m/z | z | RT | Fraction | Scan | Source File | Area BuCaMH03\_B17 | #Feature | #Feature BuCaMH03\_B17 | Start | End | PTM | AScore | Found By |
| --- | --- | --- | --- | --- | --- | --- | --- | --- | --- | --- | --- | --- | --- | --- | --- | --- | --- | --- | --- |
| K.FVNANQETISHIVK.V | N | 82.82 | 1598.8417 | 14 | 0.4 | 800.4284 | 2 | 36.65 | 1 | 16914 | BuCaMH03.raw | 2.325E6 | 1 | 1 | 222 | 235 |  |  | PEAKS DB |
| K.LIDLELLHKYEELLEK.C | Y | 77.85 | 1997.1084 | 16 | -0.2 | 666.7100 | 3 | 79.40 | 1 | 51813 | BuCaMH03.raw | 1.9868E6 | 1 | 1 | 343 | 358 |  |  | PEAKS DB |
| L.IDLELLHKYEELLEK.C | Y | 65.17 | 1884.0244 | 15 | 0.8 | 629.0159 | 3 | 74.42 | 1 | 47774 | BuCaMH03.raw | 4.1076E6 | 1 | 1 | 344 | 358 |  |  | PEAKS DB |
| K.ESFLFTLTR.N | N | 64.62 | 1112.5865 | 9 | -0.5 | 557.3003 | 2 | 68.54 | 1 | 42869 | BuCaMH03.raw | 3.8022E6 | 1 | 1 | 327 | 335 |  |  | PEAKS DB |
| R.DDYKESFLFTLTR.N | N | 56.79 | 1633.7987 | 13 | 0.3 | 817.9069 | 2 | 68.43 | 1 | 42961 | BuCaMH03.raw | 1.9548E6 | 2 | 2 | 323 | 335 |  |  | PEAKS DB |
| K.DAISSNVGHC(+57.02)C(+57.02)EK.P | Y | 56.12 | 1475.6133 | 13 | 0.2 | 738.8141 | 2 | 12.77 | 1 | 2024 | BuCaMH03.raw | 0 | 0 | 0 | 268 | 280 | Carbamidomethylation | C10:Carbamidomethylation:1000.00;C11:Carbamidomethylation:1000.00 | PEAKS DB |
| K.GDSVEVLVDR.A | Y | 55.99 | 1087.5509 | 10 | -0.2 | 544.7826 | 2 | 40.00 | 1 | 19317 | BuCaMH03.raw | 7.8771E5 | 1 | 1 | 247 | 256 |  |  | PEAKS DB |
| K.YGINDC(+57.02)C(+57.02)AK.A | N | 55.32 | 1099.4426 | 9 | 1.3 | 550.7293 | 2 | 18.90 | 1 | 4831 | BuCaMH03.raw | 6.6309E5 | 1 | 1 | 112 | 120 | Carbamidomethylation | C6:Carbamidomethylation:1000.00;C7:Carbamidomethylation:1000.00 | PEAKS DB |
| K.C(+57.02)C(+57.02)NLDSNHQVSC(+57.02)ALENTDK.V | Y | 55.15 | 2263.9255 | 19 | 0.7 | 755.6497 | 3 | 29.16 | 1 | 11233 | BuCaMH03.raw | 1.3055E6 | 1 | 1 | 436 | 454 | Carbamidomethylation | C1:Carbamidomethylation:1000.00;C2:Carbamidomethylation:1000.00;C12:Carbamidomethylation:1000.00 | PEAKS DB |
| total 9 peptides |  |  |  |  |  |  |  |  |  |  |  |  |  |  |  |  |  |  |  |
| --- | --- | --- | --- | --- | --- | --- | --- | --- | --- | --- | --- | --- | --- | --- | --- | --- | --- | --- | --- |

XP\_034296605.1

back to list

  

| Protein Coverage
| Supporting Peptides
|

Protein Coverage:

Supporting Peptides:

| Peptide | Uniq | -10lgP | Mass | Length | ppm | m/z | z | RT | Fraction | Scan | Source File | Area BuCaMH03\_B17 | #Feature | #Feature BuCaMH03\_B17 | Start | End | PTM | AScore | Found By |
| --- | --- | --- | --- | --- | --- | --- | --- | --- | --- | --- | --- | --- | --- | --- | --- | --- | --- | --- | --- |
| K.LQWLMDQALQGDIIK.T | N | 76.36 | 1770.9338 | 15 | 0.5 | 886.4746 | 2 | 80.09 | 1 | 52433 | BuCaMH03.raw | 3.2396E5 | 1 | 1 | 799 | 813 |  |  | PEAKS DB |
| R.NPYGYQLAWK.F | N | 70.15 | 1238.6084 | 10 | -0.7 | 620.3110 | 2 | 51.24 | 1 | 28651 | BuCaMH03.raw | 8.0714E5 | 1 | 1 | 827 | 836 |  |  | PEAKS DB |
| K.ALDLTLYLK.H | N | 65.37 | 1048.6168 | 9 | -0.1 | 525.3156 | 2 | 69.67 | 1 | 43911 | BuCaMH03.raw | 1.2666E6 | 1 | 1 | 642 | 650 |  |  | PEAKS DB |
| A.SLINNVFQLVSAGK.L | Y | 65.06 | 1488.8300 | 14 | 0.8 | 745.4229 | 2 | 84.21 | 1 | 55425 | BuCaMH03.raw | 1.3683E6 | 1 | 1 | 623 | 636 |  |  | PEAKS DB |
| K.QDLAAIPDFQSGAMENWGLTTYR.E | N | 64.54 | 2583.2063 | 23 | 1.3 | 862.0771 | 3 | 83.98 | 1 | 55332 | BuCaMH03.raw | 9.1463E5 | 1 | 1 | 291 | 313 |  |  | PEAKS DB |
| R.VLASTQFEPTAAR.M | N | 61.32 | 1389.7252 | 13 | 0.6 | 695.8703 | 2 | 38.08 | 1 | 17978 | BuCaMH03.raw | 5.7735E5 | 1 | 1 | 161 | 173 |  |  | PEAKS DB |
| total 6 peptides |  |  |  |  |  |  |  |  |  |  |  |  |  |  |  |  |  |  |  |
| --- | --- | --- | --- | --- | --- | --- | --- | --- | --- | --- | --- | --- | --- | --- | --- | --- | --- | --- | --- |

XP\_034296606.1

back to list

  

| Protein Coverage
| Supporting Peptides
|

Protein Coverage:

Supporting Peptides:

| Peptide | Uniq | -10lgP | Mass | Length | ppm | m/z | z | RT | Fraction | Scan | Source File | Area BuCaMH03\_B17 | #Feature | #Feature BuCaMH03\_B17 | Start | End | PTM | AScore | Found By |
| --- | --- | --- | --- | --- | --- | --- | --- | --- | --- | --- | --- | --- | --- | --- | --- | --- | --- | --- | --- |
| K.LQWLMDQALQGDIIK.T | N | 76.36 | 1770.9338 | 15 | 0.5 | 886.4746 | 2 | 80.09 | 1 | 52433 | BuCaMH03.raw | 3.2396E5 | 1 | 1 | 799 | 813 |  |  | PEAKS DB |
| R.NPYGYQLAWK.F | N | 70.15 | 1238.6084 | 10 | -0.7 | 620.3110 | 2 | 51.24 | 1 | 28651 | BuCaMH03.raw | 8.0714E5 | 1 | 1 | 827 | 836 |  |  | PEAKS DB |
| K.ALDLTLYLK.H | N | 65.37 | 1048.6168 | 9 | -0.1 | 525.3156 | 2 | 69.67 | 1 | 43911 | BuCaMH03.raw | 1.2666E6 | 1 | 1 | 642 | 650 |  |  | PEAKS DB |
| A.SLINNVFQLVSAGK.L | Y | 65.06 | 1488.8300 | 14 | 0.8 | 745.4229 | 2 | 84.21 | 1 | 55425 | BuCaMH03.raw | 1.3683E6 | 1 | 1 | 623 | 636 |  |  | PEAKS DB |
| K.QDLAAIPDFQSGAMENWGLTTYR.E | N | 64.54 | 2583.2063 | 23 | 1.3 | 862.0771 | 3 | 83.98 | 1 | 55332 | BuCaMH03.raw | 9.1463E5 | 1 | 1 | 291 | 313 |  |  | PEAKS DB |
| R.VLASTQFEPTAAR.M | N | 61.32 | 1389.7252 | 13 | 0.6 | 695.8703 | 2 | 38.08 | 1 | 17978 | BuCaMH03.raw | 5.7735E5 | 1 | 1 | 161 | 173 |  |  | PEAKS DB |
| total 6 peptides |  |  |  |  |  |  |  |  |  |  |  |  |  |  |  |  |  |  |  |
| --- | --- | --- | --- | --- | --- | --- | --- | --- | --- | --- | --- | --- | --- | --- | --- | --- | --- | --- | --- |

XP\_034296607.1

back to list

  

| Protein Coverage
| Supporting Peptides
|

Protein Coverage:

Supporting Peptides:

| Peptide | Uniq | -10lgP | Mass | Length | ppm | m/z | z | RT | Fraction | Scan | Source File | Area BuCaMH03\_B17 | #Feature | #Feature BuCaMH03\_B17 | Start | End | PTM | AScore | Found By |
| --- | --- | --- | --- | --- | --- | --- | --- | --- | --- | --- | --- | --- | --- | --- | --- | --- | --- | --- | --- |
| K.LQWLMDQALQGDIIK.T | N | 76.36 | 1770.9338 | 15 | 0.5 | 886.4746 | 2 | 80.09 | 1 | 52433 | BuCaMH03.raw | 3.2396E5 | 1 | 1 | 799 | 813 |  |  | PEAKS DB |
| R.NPYGYQLAWK.F | N | 70.15 | 1238.6084 | 10 | -0.7 | 620.3110 | 2 | 51.24 | 1 | 28651 | BuCaMH03.raw | 8.0714E5 | 1 | 1 | 827 | 836 |  |  | PEAKS DB |
| K.ALDLTLYLK.H | N | 65.37 | 1048.6168 | 9 | -0.1 | 525.3156 | 2 | 69.67 | 1 | 43911 | BuCaMH03.raw | 1.2666E6 | 1 | 1 | 642 | 650 |  |  | PEAKS DB |
| A.SLINNVFQLVSAGK.L | Y | 65.06 | 1488.8300 | 14 | 0.8 | 745.4229 | 2 | 84.21 | 1 | 55425 | BuCaMH03.raw | 1.3683E6 | 1 | 1 | 623 | 636 |  |  | PEAKS DB |
| K.QDLAAIPDFQSGAMENWGLTTYR.E | N | 64.54 | 2583.2063 | 23 | 1.3 | 862.0771 | 3 | 83.98 | 1 | 55332 | BuCaMH03.raw | 9.1463E5 | 1 | 1 | 291 | 313 |  |  | PEAKS DB |
| R.VLASTQFEPTAAR.M | N | 61.32 | 1389.7252 | 13 | 0.6 | 695.8703 | 2 | 38.08 | 1 | 17978 | BuCaMH03.raw | 5.7735E5 | 1 | 1 | 161 | 173 |  |  | PEAKS DB |
| total 6 peptides |  |  |  |  |  |  |  |  |  |  |  |  |  |  |  |  |  |  |  |
| --- | --- | --- | --- | --- | --- | --- | --- | --- | --- | --- | --- | --- | --- | --- | --- | --- | --- | --- | --- |

XP\_026570202.1

back to list

  

| Protein Coverage
| Supporting Peptides
|

Protein Coverage:

Supporting Peptides:

| Peptide | Uniq | -10lgP | Mass | Length | ppm | m/z | z | RT | Fraction | Scan | Source File | Area BuCaMH03\_B17 | #Feature | #Feature BuCaMH03\_B17 | Start | End | PTM | AScore | Found By |
| --- | --- | --- | --- | --- | --- | --- | --- | --- | --- | --- | --- | --- | --- | --- | --- | --- | --- | --- | --- |
| K.HSWANNLAVLEC(+57.02)LQDVR.E | Y | 82.21 | 2023.9897 | 17 | 0.6 | 675.6710 | 3 | 67.82 | 1 | 42379 | BuCaMH03.raw | 5.9885E5 | 1 | 1 | 115 | 131 | Carbamidomethylation | C12:Carbamidomethylation:1000.00 | PEAKS DB |
| R.QVSSEC(+57.02)QGEMLDYR.R | Y | 70.47 | 1700.7134 | 14 | -0.6 | 851.3635 | 2 | 36.48 | 1 | 16709 | BuCaMH03.raw | 2.4546E5 | 1 | 1 | 400 | 413 | Carbamidomethylation | C6:Carbamidomethylation:1000.00 | PEAKS DB |
| L.SYLLMC(+57.02)LESAVHR.G | Y | 63.89 | 1577.7694 | 13 | -0.3 | 526.9302 | 3 | 63.71 | 1 | 38985 | BuCaMH03.raw | 1.047E5 | 1 | 1 | 385 | 397 | Carbamidomethylation | C6:Carbamidomethylation:1000.00 | PEAKS DB |
| R.MLMEDFSLSPEIILGC(+57.02)R.T | Y | 62.19 | 2009.9624 | 17 | -0.7 | 1005.9877 | 2 | 91.61 | 1 | 61584 | BuCaMH03.raw | 1.1397E6 | 1 | 1 | 415 | 431 | Carbamidomethylation | C16:Carbamidomethylation:1000.00 | PEAKS DB |
| R.LLELQYFISR.D | Y | 54.07 | 1280.7129 | 10 | 0.6 | 641.3641 | 2 | 75.24 | 1 | 48507 | BuCaMH03.raw | 2.4604E5 | 1 | 1 | 532 | 541 |  |  | PEAKS DB |
| K.MTAIIFSDYR.L | Y | 53.61 | 1215.5958 | 10 | 0.2 | 608.8053 | 2 | 58.74 | 1 | 34721 | BuCaMH03.raw | 4.4716E5 | 1 | 1 | 209 | 218 |  |  | PEAKS DB |
| total 6 peptides |  |  |  |  |  |  |  |  |  |  |  |  |  |  |  |  |  |  |  |
| --- | --- | --- | --- | --- | --- | --- | --- | --- | --- | --- | --- | --- | --- | --- | --- | --- | --- | --- | --- |

XP\_026535930.1

back to list

  

| Protein Coverage
| Supporting Peptides
|

Protein Coverage:

Supporting Peptides:

| Peptide | Uniq | -10lgP | Mass | Length | ppm | m/z | z | RT | Fraction | Scan | Source File | Area BuCaMH03\_B17 | #Feature | #Feature BuCaMH03\_B17 | Start | End | PTM | AScore | Found By |
| --- | --- | --- | --- | --- | --- | --- | --- | --- | --- | --- | --- | --- | --- | --- | --- | --- | --- | --- | --- |
| K.HSWANNLAVLEC(+57.02)LQDVR.E | Y | 82.21 | 2023.9897 | 17 | 0.6 | 675.6710 | 3 | 67.82 | 1 | 42379 | BuCaMH03.raw | 5.9885E5 | 1 | 1 | 111 | 127 | Carbamidomethylation | C12:Carbamidomethylation:1000.00 | PEAKS DB |
| R.QVSSEC(+57.02)QGEMLDYR.R | Y | 70.47 | 1700.7134 | 14 | -0.6 | 851.3635 | 2 | 36.48 | 1 | 16709 | BuCaMH03.raw | 2.4546E5 | 1 | 1 | 396 | 409 | Carbamidomethylation | C6:Carbamidomethylation:1000.00 | PEAKS DB |
| L.SYLLMC(+57.02)LESAVHR.G | Y | 63.89 | 1577.7694 | 13 | -0.3 | 526.9302 | 3 | 63.71 | 1 | 38985 | BuCaMH03.raw | 1.047E5 | 1 | 1 | 381 | 393 | Carbamidomethylation | C6:Carbamidomethylation:1000.00 | PEAKS DB |
| R.MLMEDFSLSPEIILGC(+57.02)R.T | Y | 62.19 | 2009.9624 | 17 | -0.7 | 1005.9877 | 2 | 91.61 | 1 | 61584 | BuCaMH03.raw | 1.1397E6 | 1 | 1 | 411 | 427 | Carbamidomethylation | C16:Carbamidomethylation:1000.00 | PEAKS DB |
| R.LLELQYFISR.D | Y | 54.07 | 1280.7129 | 10 | 0.6 | 641.3641 | 2 | 75.24 | 1 | 48507 | BuCaMH03.raw | 2.4604E5 | 1 | 1 | 528 | 537 |  |  | PEAKS DB |
| K.MTAIIFSDYR.L | Y | 53.61 | 1215.5958 | 10 | 0.2 | 608.8053 | 2 | 58.74 | 1 | 34721 | BuCaMH03.raw | 4.4716E5 | 1 | 1 | 205 | 214 |  |  | PEAKS DB |
| total 6 peptides |  |  |  |  |  |  |  |  |  |  |  |  |  |  |  |  |  |  |  |
| --- | --- | --- | --- | --- | --- | --- | --- | --- | --- | --- | --- | --- | --- | --- | --- | --- | --- | --- | --- |

T\_22

back to list

  

| Protein Coverage
| Supporting Peptides
|

Protein Coverage:

Supporting Peptides:

| Peptide | Uniq | -10lgP | Mass | Length | ppm | m/z | z | RT | Fraction | Scan | Source File | Area BuCaMH03\_B17 | #Feature | #Feature BuCaMH03\_B17 | Start | End | PTM | AScore | Found By |
| --- | --- | --- | --- | --- | --- | --- | --- | --- | --- | --- | --- | --- | --- | --- | --- | --- | --- | --- | --- |
| K.TC(+57.02)PFHTC(+57.02)PNSETC(+57.02)PDGK.N | Y | 105.52 | 2006.7921 | 17 | -0.4 | 1004.4030 | 2 | 19.73 | 1 | 5324 | BuCaMH03.raw | 3.4203E8 | 4 | 4 | 34 | 50 | Carbamidomethylation | C2:Carbamidomethylation:1000.00;C7:Carbamidomethylation:1000.00;C13:Carbamidomethylation:1000.00 | PEAKS DB |
| R.KC(+57.02)AATC(+57.02)PSDILSVTILC(+57.02)C(+57.02)TTDNC(+57.02)ND | Y | 66.84 | 2888.2119 | 25 | 0.2 | 1445.1135 | 2 | 74.22 | 1 | 48000 | BuCaMH03.raw | 3.6043E7 | 1 | 1 | 73 | 97 | Carbamidomethylation | C2:Carbamidomethylation:1000.00;C6:Carbamidomethylation:1000.00;C17:Carbamidomethylation:1000.00;C18:Carbamidomethylation:1000.00;C23:Carbamidomethylation:1000.00 | PEAKS DB |
| K.C(+57.02)AATC(+57.02)PSDILSVTILC(+57.02)C(+57.02)TTDNC(+57.02)ND | Y | 64.36 | 2760.1169 | 24 | 0.5 | 1381.0664 | 2 | 87.75 | 1 | 58749 | BuCaMH03.raw | 1.6429E8 | 2 | 2 | 74 | 97 | Carbamidomethylation | C1:Carbamidomethylation:1000.00;C5:Carbamidomethylation:1000.00;C16:Carbamidomethylation:1000.00;C17:Carbamidomethylation:1000.00;C22:Carbamidomethylation:1000.00 | PEAKS DB |
| K.TC(+57.02)PFHTC(+57.02)PNSETC(+57.02)PDGKNIC(+57.02)VKLSWLAVR.G | Y | 54.42 | 3446.5991 | 29 | -0.9 | 862.6562 | 4 | 53.70 | 1 | 30664 | BuCaMH03.raw | 3.1014E6 | 2 | 2 | 34 | 62 | Carbamidomethylation | C2:Carbamidomethylation:1000.00;C7:Carbamidomethylation:1000.00;C13:Carbamidomethylation:1000.00;C20:Carbamidomethylation:1000.00 | PEAKS DB |
| total 4 peptides |  |  |  |  |  |  |  |  |  |  |  |  |  |  |  |  |  |  |  |
| --- | --- | --- | --- | --- | --- | --- | --- | --- | --- | --- | --- | --- | --- | --- | --- | --- | --- | --- | --- |

Q9YGI8.1

back to list

  

| Protein Coverage
| Supporting Peptides
|

Protein Coverage:

Supporting Peptides:

| Peptide | Uniq | -10lgP | Mass | Length | ppm | m/z | z | RT | Fraction | Scan | Source File | Area BuCaMH03\_B17 | #Feature | #Feature BuCaMH03\_B17 | Start | End | PTM | AScore | Found By |
| --- | --- | --- | --- | --- | --- | --- | --- | --- | --- | --- | --- | --- | --- | --- | --- | --- | --- | --- | --- |
| K.TC(+57.02)PSGQLLC(+57.02)LK.K | Y | 82.21 | 1275.6315 | 11 | 0.4 | 638.8232 | 2 | 43.47 | 1 | 22037 | BuCaMH03.raw | 5.9298E8 | 1 | 1 | 37 | 47 | Carbamidomethylation | C2:Carbamidomethylation:1000.00;C9:Carbamidomethylation:1000.00 | PEAKS DB |
| K.KNEIIQC(+57.02)C(+57.02)AK.D | Y | 73.30 | 1262.6111 | 10 | -0.9 | 632.3123 | 2 | 12.57 | 1 | 1868 | BuCaMH03.raw | 1.5666E7 | 1 | 1 | 72 | 81 | Carbamidomethylation | C7:Carbamidomethylation:1000.00;C8:Carbamidomethylation:1000.00 | PEAKS DB |
| T.C(+57.02)PSGQLLC(+57.02)LK.K | Y | 70.51 | 1174.5839 | 10 | 1.0 | 588.2998 | 2 | 41.74 | 1 | 20969 | BuCaMH03.raw | 5.3383E7 | 1 | 1 | 38 | 47 | Carbamidomethylation | C1:Carbamidomethylation:1000.00;C8:Carbamidomethylation:1000.00 | PEAKS DB |
| K.WEIGNPSGK.E | Y | 66.05 | 986.4821 | 9 | -0.5 | 494.2481 | 2 | 34.15 | 1 | 15030 | BuCaMH03.raw | 9.4483E8 | 1 | 1 | 49 | 57 |  |  | PEAKS DB |
| K.NEIIQC(+57.02)C(+57.02)AK.D | Y | 58.17 | 1134.5161 | 9 | 0.2 | 568.2654 | 2 | 19.18 | 1 | 5077 | BuCaMH03.raw | 5.5334E7 | 1 | 1 | 73 | 81 | Carbamidomethylation | C6:Carbamidomethylation:1000.00;C7:Carbamidomethylation:1000.00 | PEAKS DB |
| total 5 peptides |  |  |  |  |  |  |  |  |  |  |  |  |  |  |  |  |  |  |  |
| --- | --- | --- | --- | --- | --- | --- | --- | --- | --- | --- | --- | --- | --- | --- | --- | --- | --- | --- | --- |

CAA06887.1

back to list

  

| Protein Coverage
| Supporting Peptides
|

Protein Coverage:

Supporting Peptides:

| Peptide | Uniq | -10lgP | Mass | Length | ppm | m/z | z | RT | Fraction | Scan | Source File | Area BuCaMH03\_B17 | #Feature | #Feature BuCaMH03\_B17 | Start | End | PTM | AScore | Found By |
| --- | --- | --- | --- | --- | --- | --- | --- | --- | --- | --- | --- | --- | --- | --- | --- | --- | --- | --- | --- |
| K.TC(+57.02)PSGQLLC(+57.02)LK.K | Y | 82.21 | 1275.6315 | 11 | 0.4 | 638.8232 | 2 | 43.47 | 1 | 22037 | BuCaMH03.raw | 5.9298E8 | 1 | 1 | 37 | 47 | Carbamidomethylation | C2:Carbamidomethylation:1000.00;C9:Carbamidomethylation:1000.00 | PEAKS DB |
| K.KNEIIQC(+57.02)C(+57.02)AK.D | Y | 73.30 | 1262.6111 | 10 | -0.9 | 632.3123 | 2 | 12.57 | 1 | 1868 | BuCaMH03.raw | 1.5666E7 | 1 | 1 | 72 | 81 | Carbamidomethylation | C7:Carbamidomethylation:1000.00;C8:Carbamidomethylation:1000.00 | PEAKS DB |
| T.C(+57.02)PSGQLLC(+57.02)LK.K | Y | 70.51 | 1174.5839 | 10 | 1.0 | 588.2998 | 2 | 41.74 | 1 | 20969 | BuCaMH03.raw | 5.3383E7 | 1 | 1 | 38 | 47 | Carbamidomethylation | C1:Carbamidomethylation:1000.00;C8:Carbamidomethylation:1000.00 | PEAKS DB |
| K.WEIGNPSGK.E | Y | 66.05 | 986.4821 | 9 | -0.5 | 494.2481 | 2 | 34.15 | 1 | 15030 | BuCaMH03.raw | 9.4483E8 | 1 | 1 | 49 | 57 |  |  | PEAKS DB |
| K.NEIIQC(+57.02)C(+57.02)AK.D | Y | 58.17 | 1134.5161 | 9 | 0.2 | 568.2654 | 2 | 19.18 | 1 | 5077 | BuCaMH03.raw | 5.5334E7 | 1 | 1 | 73 | 81 | Carbamidomethylation | C6:Carbamidomethylation:1000.00;C7:Carbamidomethylation:1000.00 | PEAKS DB |
| total 5 peptides |  |  |  |  |  |  |  |  |  |  |  |  |  |  |  |  |  |  |  |
| --- | --- | --- | --- | --- | --- | --- | --- | --- | --- | --- | --- | --- | --- | --- | --- | --- | --- | --- | --- |

XP\_026521896.1

back to list

  

| Protein Coverage
| Supporting Peptides
|

Protein Coverage:

Supporting Peptides:

| Peptide | Uniq | -10lgP | Mass | Length | ppm | m/z | z | RT | Fraction | Scan | Source File | Area BuCaMH03\_B17 | #Feature | #Feature BuCaMH03\_B17 | Start | End | PTM | AScore | Found By |
| --- | --- | --- | --- | --- | --- | --- | --- | --- | --- | --- | --- | --- | --- | --- | --- | --- | --- | --- | --- |
| R.NLHNC(+57.02)VNLILLADHGMEAISC(+57.02)NR.L | N | 85.12 | 2663.2729 | 23 | -0.1 | 666.8254 | 4 | 67.62 | 1 | 42179 | BuCaMH03.raw | 1.0977E6 | 1 | 1 | 340 | 362 | Carbamidomethylation | C5:Carbamidomethylation:1000.00;C21:Carbamidomethylation:1000.00 | PEAKS DB |
| K.FGPVSGQIIK.S | Y | 64.29 | 1044.5967 | 10 | 0.1 | 523.3057 | 2 | 43.11 | 1 | 21815 | BuCaMH03.raw | 7.8843E5 | 1 | 1 | 311 | 320 |  |  | PEAKS DB |
| K.SPDNLWVEER.M | N | 64.17 | 1243.5833 | 10 | -0.3 | 622.7987 | 2 | 43.64 | 1 | 22314 | BuCaMH03.raw | 5.2781E5 | 1 | 1 | 799 | 808 |  |  | PEAKS DB |
| K.DFYTFDSEAIVK.N | N | 54.12 | 1433.6714 | 12 | 1.4 | 717.8440 | 2 | 67.17 | 1 | 41791 | BuCaMH03.raw | 7.0826E5 | 1 | 1 | 393 | 404 |  |  | PEAKS DB |
| total 4 peptides |  |  |  |  |  |  |  |  |  |  |  |  |  |  |  |  |  |  |  |
| --- | --- | --- | --- | --- | --- | --- | --- | --- | --- | --- | --- | --- | --- | --- | --- | --- | --- | --- | --- |

XP\_026521895.1

back to list

  

| Protein Coverage
| Supporting Peptides
|

Protein Coverage:

Supporting Peptides:

| Peptide | Uniq | -10lgP | Mass | Length | ppm | m/z | z | RT | Fraction | Scan | Source File | Area BuCaMH03\_B17 | #Feature | #Feature BuCaMH03\_B17 | Start | End | PTM | AScore | Found By |
| --- | --- | --- | --- | --- | --- | --- | --- | --- | --- | --- | --- | --- | --- | --- | --- | --- | --- | --- | --- |
| R.NLHNC(+57.02)VNLILLADHGMEAISC(+57.02)NR.L | N | 85.12 | 2663.2729 | 23 | -0.1 | 666.8254 | 4 | 67.62 | 1 | 42179 | BuCaMH03.raw | 1.0977E6 | 1 | 1 | 376 | 398 | Carbamidomethylation | C5:Carbamidomethylation:1000.00;C21:Carbamidomethylation:1000.00 | PEAKS DB |
| K.FGPVSGQIIK.S | Y | 64.29 | 1044.5967 | 10 | 0.1 | 523.3057 | 2 | 43.11 | 1 | 21815 | BuCaMH03.raw | 7.8843E5 | 1 | 1 | 347 | 356 |  |  | PEAKS DB |
| K.SPDNLWVEER.M | N | 64.17 | 1243.5833 | 10 | -0.3 | 622.7987 | 2 | 43.64 | 1 | 22314 | BuCaMH03.raw | 5.2781E5 | 1 | 1 | 835 | 844 |  |  | PEAKS DB |
| K.DFYTFDSEAIVK.N | N | 54.12 | 1433.6714 | 12 | 1.4 | 717.8440 | 2 | 67.17 | 1 | 41791 | BuCaMH03.raw | 7.0826E5 | 1 | 1 | 429 | 440 |  |  | PEAKS DB |
| total 4 peptides |  |  |  |  |  |  |  |  |  |  |  |  |  |  |  |  |  |  |  |
| --- | --- | --- | --- | --- | --- | --- | --- | --- | --- | --- | --- | --- | --- | --- | --- | --- | --- | --- | --- |

T\_176

back to list

  

| Protein Coverage
| Supporting Peptides
|

Protein Coverage:

Supporting Peptides:

| Peptide | Uniq | -10lgP | Mass | Length | ppm | m/z | z | RT | Fraction | Scan | Source File | Area BuCaMH03\_B17 | #Feature | #Feature BuCaMH03\_B17 | Start | End | PTM | AScore | Found By |
| --- | --- | --- | --- | --- | --- | --- | --- | --- | --- | --- | --- | --- | --- | --- | --- | --- | --- | --- | --- |
| R.DTYVTC(+57.02)EEGYVC(+57.02)YTYYSVKPPNR.F | Y | 103.99 | 2863.2468 | 23 | 0.4 | 955.4233 | 3 | 58.62 | 1 | 34612 | BuCaMH03.raw | 6.9416E8 | 4 | 4 | 30 | 52 | Carbamidomethylation | C6:Carbamidomethylation:1000.00;C12:Carbamidomethylation:1000.00 | PEAKS DB |
| T.IIC(+57.02)HSRDTYVTC(+57.02)EEGYVC(+57.02)YTYYSVKPPNR.F | Y | 75.28 | 3629.6377 | 29 | 0.9 | 908.4175 | 4 | 46.60 | 1 | 24863 | BuCaMH03.raw | 2.4476E7 | 1 | 1 | 24 | 52 | Carbamidomethylation | C3:Carbamidomethylation:1000.00;C12:Carbamidomethylation:1000.00;C18:Carbamidomethylation:1000.00 | PEAKS DB |
| Y.TYYSVKPPNR.F | Y | 61.37 | 1223.6299 | 10 | -0.1 | 408.8839 | 3 | 20.05 | 1 | 5479 | BuCaMH03.raw | 3.3234E6 | 1 | 1 | 43 | 52 |  |  | PEAKS DB |
| I.C(+57.02)HSRDTYVTC(+57.02)EEGYVC(+57.02)YTYYSVKPPNR.F | Y | 55.70 | 3403.4695 | 27 | -0.4 | 681.7009 | 5 | 45.65 | 1 | 23777 | BuCaMH03.raw | 6.361E5 | 1 | 1 | 26 | 52 | Carbamidomethylation | C1:Carbamidomethylation:1000.00;C10:Carbamidomethylation:1000.00;C16:Carbamidomethylation:1000.00 | PEAKS DB |
| total 4 peptides |  |  |  |  |  |  |  |  |  |  |  |  |  |  |  |  |  |  |  |
| --- | --- | --- | --- | --- | --- | --- | --- | --- | --- | --- | --- | --- | --- | --- | --- | --- | --- | --- | --- |

T\_83

back to list

  

| Protein Coverage
| Supporting Peptides
|

Protein Coverage:

Supporting Peptides:

| Peptide | Uniq | -10lgP | Mass | Length | ppm | m/z | z | RT | Fraction | Scan | Source File | Area BuCaMH03\_B17 | #Feature | #Feature BuCaMH03\_B17 | Start | End | PTM | AScore | Found By |
| --- | --- | --- | --- | --- | --- | --- | --- | --- | --- | --- | --- | --- | --- | --- | --- | --- | --- | --- | --- |
| K.HQGLPESYNFDFVTMKPVL | Y | 82.29 | 2221.0876 | 19 | 0.6 | 741.3703 | 3 | 74.84 | 1 | 48178 | BuCaMH03.raw | 1.3326E6 | 1 | 1 | 535 | 553 |  |  | PEAKS DB |
| K.YGLDFSYEMAPR.A | Y | 81.73 | 1447.6442 | 12 | 0.0 | 724.8293 | 2 | 63.81 | 1 | 39095 | BuCaMH03.raw | 9.0867E5 | 1 | 1 | 427 | 438 |  |  | PEAKS DB |
| K.QVVPESLFAWER.V | Y | 65.87 | 1459.7460 | 12 | 0.8 | 730.8809 | 2 | 71.71 | 1 | 45618 | BuCaMH03.raw | 5.1221E5 | 1 | 1 | 319 | 330 |  |  | PEAKS DB |
| A.DLHYATVYWLEAEK.S | Y | 60.70 | 1736.8409 | 14 | 0.6 | 579.9546 | 3 | 65.47 | 1 | 40425 | BuCaMH03.raw | 3.8121E5 | 1 | 1 | 37 | 50 |  |  | PEAKS DB |
| R.NAGYIIAQLDGLYMGNLEWAK.R | Y | 56.62 | 2339.1619 | 21 | 0.1 | 780.7280 | 3 | 95.03 | 1 | 64340 | BuCaMH03.raw | 3.2773E5 | 1 | 1 | 159 | 179 |  |  | PEAKS DB |
| K.TWAQIFEK.Q | Y | 53.01 | 1021.5233 | 8 | 0.5 | 511.7692 | 2 | 55.61 | 1 | 32227 | BuCaMH03.raw | 7.2599E5 | 1 | 1 | 343 | 350 |  |  | PEAKS DB |
| total 6 peptides |  |  |  |  |  |  |  |  |  |  |  |  |  |  |  |  |  |  |  |
| --- | --- | --- | --- | --- | --- | --- | --- | --- | --- | --- | --- | --- | --- | --- | --- | --- | --- | --- | --- |

XP\_034277309.1

back to list

  

| Protein Coverage
| Supporting Peptides
|

Protein Coverage:

Supporting Peptides:

| Peptide | Uniq | -10lgP | Mass | Length | ppm | m/z | z | RT | Fraction | Scan | Source File | Area BuCaMH03\_B17 | #Feature | #Feature BuCaMH03\_B17 | Start | End | PTM | AScore | Found By |
| --- | --- | --- | --- | --- | --- | --- | --- | --- | --- | --- | --- | --- | --- | --- | --- | --- | --- | --- | --- |
| K.FHGLGVIDWENWR.P | Y | 78.08 | 1627.7896 | 13 | 0.2 | 543.6039 | 3 | 69.32 | 1 | 43607 | BuCaMH03.raw | 2.5945E6 | 1 | 1 | 142 | 154 |  |  | PEAKS DB |
| K.FHGLGVIDWENWRPQWDR.N | N | 72.06 | 2310.1082 | 18 | 0.7 | 578.5347 | 4 | 72.04 | 1 | 45863 | BuCaMH03.raw | 8.7408E5 | 1 | 1 | 142 | 159 |  |  | PEAKS DB |
| R.NDQLLWLWR.D | N | 63.60 | 1242.6509 | 9 | 0.8 | 622.3332 | 2 | 79.40 | 1 | 51700 | BuCaMH03.raw | 5.3353E7 | 1 | 1 | 251 | 259 |  |  | PEAKS DB |
| K.C(+57.02)PNIEISR.N | N | 60.84 | 987.4808 | 8 | 0.7 | 494.7480 | 2 | 26.63 | 1 | 9851 | BuCaMH03.raw | 7.2279E6 | 1 | 1 | 243 | 250 | Carbamidomethylation | C1:Carbamidomethylation:1000.00 | PEAKS DB |
| F.HGLGVIDWENWRPQWDR.N | N | 54.37 | 2163.0398 | 17 | 1.0 | 541.7678 | 4 | 67.50 | 1 | 42023 | BuCaMH03.raw | 1.0462E6 | 1 | 1 | 143 | 159 |  |  | PEAKS DB |
| K.DYALPVFVYAR.P | N | 53.97 | 1312.6815 | 11 | 0.9 | 657.3486 | 2 | 77.76 | 1 | 50512 | BuCaMH03.raw | 5.5419E6 | 1 | 1 | 301 | 311 |  |  | PEAKS DB |
| total 6 peptides |  |  |  |  |  |  |  |  |  |  |  |  |  |  |  |  |  |  |  |
| --- | --- | --- | --- | --- | --- | --- | --- | --- | --- | --- | --- | --- | --- | --- | --- | --- | --- | --- | --- |

XP\_034264771.1

back to list

  

| Protein Coverage
| Supporting Peptides
|

Protein Coverage:

Supporting Peptides:

| Peptide | Uniq | -10lgP | Mass | Length | ppm | m/z | z | RT | Fraction | Scan | Source File | Area BuCaMH03\_B17 | #Feature | #Feature BuCaMH03\_B17 | Start | End | PTM | AScore | Found By |
| --- | --- | --- | --- | --- | --- | --- | --- | --- | --- | --- | --- | --- | --- | --- | --- | --- | --- | --- | --- |
| K.SSIFGSVEIVNLNPEK.V | N | 79.12 | 1731.9043 | 16 | 2.2 | 866.9613 | 2 | 72.81 | 1 | 46448 | BuCaMH03.raw | 4.2647E7 | 1 | 1 | 222 | 237 |  |  | PEAKS DB |
| R.KSSIFGSVEIVNLNPEK.V | N | 70.79 | 1859.9993 | 17 | 1.1 | 931.0079 | 2 | 59.81 | 1 | 35636 | BuCaMH03.raw | 2.1478E6 | 1 | 1 | 221 | 237 |  |  | PEAKS DB |
| K.LFEQAFLYK.D | Y | 64.24 | 1157.6121 | 9 | 0.0 | 579.8133 | 2 | 60.60 | 1 | 36224 | BuCaMH03.raw | 1.3908E7 | 1 | 1 | 91 | 99 |  |  | PEAKS DB |
| R.PAQLLQC(+57.02)TR.N | Y | 53.90 | 1085.5652 | 9 | -0.5 | 543.7896 | 2 | 25.13 | 1 | 8617 | BuCaMH03.raw | 3.9358E5 | 1 | 1 | 284 | 292 | Carbamidomethylation | C7:Carbamidomethylation:1000.00 | PEAKS DB |
| S.C(+57.02)TGHSIAQLR.E | N | 53.80 | 1141.5662 | 10 | -0.1 | 381.5293 | 3 | 12.93 | 1 | 2105 | BuCaMH03.raw | 6.8899E4 | 1 | 1 | 257 | 266 | Carbamidomethylation | C1:Carbamidomethylation:1000.00 | PEAKS DB |
| total 5 peptides |  |  |  |  |  |  |  |  |  |  |  |  |  |  |  |  |  |  |  |
| --- | --- | --- | --- | --- | --- | --- | --- | --- | --- | --- | --- | --- | --- | --- | --- | --- | --- | --- | --- |

T\_158

back to list

  

| Protein Coverage
| Supporting Peptides
|

Protein Coverage:

Supporting Peptides:

| Peptide | Uniq | -10lgP | Mass | Length | ppm | m/z | z | RT | Fraction | Scan | Source File | Area BuCaMH03\_B17 | #Feature | #Feature BuCaMH03\_B17 | Start | End | PTM | AScore | Found By |
| --- | --- | --- | --- | --- | --- | --- | --- | --- | --- | --- | --- | --- | --- | --- | --- | --- | --- | --- | --- |
| L.DEVIPFMTVFER.S | Y | 62.24 | 1481.7224 | 12 | 0.6 | 741.8689 | 2 | 90.82 | 1 | 60896 | BuCaMH03.raw | 1.9077E7 | 1 | 1 | 38 | 49 |  |  | PEAKS DB |
| Q.LDEVIPFMTVFER.S | Y | 60.82 | 1594.8065 | 13 | 0.7 | 798.4111 | 2 | 91.54 | 1 | 61776 | BuCaMH03.raw | 3.1721E8 | 1 | 1 | 37 | 49 |  |  | PEAKS DB |
| R.SSC(+57.02)RPIETMIDIYQEYPDEVEYIFKPSC(+57.02)VLLMK.C | Y | 58.94 | 4052.9282 | 33 | 0.5 | 1014.2398 | 4 | 90.87 | 1 | 61330 | BuCaMH03.raw | 5.2638E7 | 1 | 1 | 50 | 82 | Carbamidomethylation | C3:Carbamidomethylation:1000.00;C28:Carbamidomethylation:1000.00 | PEAKS DB |
| R.HFQSQHIHPMSFQQH.S | Y | 58.54 | 1887.8588 | 15 | 0.4 | 472.9721 | 4 | 22.46 | 1 | 7191 | BuCaMH03.raw | 7.9421E6 | 1 | 1 | 111 | 125 |  |  | PEAKS DB |
| K.QLDEVIPFMTVFER.S | Y | 56.59 | 1722.8650 | 14 | 0.3 | 862.4401 | 2 | 98.36 | 1 | 67114 | BuCaMH03.raw | 9.7411E5 | 1 | 1 | 36 | 49 |  |  | PEAKS DB |
| R.HFQSQHIHPMSFQQHSK.C | Y | 53.49 | 2102.9856 | 17 | -0.1 | 421.6043 | 5 | 21.44 | 1 | 6424 | BuCaMH03.raw | 0 | 0 | 0 | 111 | 127 |  |  | PEAKS DB |
| total 6 peptides |  |  |  |  |  |  |  |  |  |  |  |  |  |  |  |  |  |  |  |
| --- | --- | --- | --- | --- | --- | --- | --- | --- | --- | --- | --- | --- | --- | --- | --- | --- | --- | --- | --- |

T\_153

back to list

  

| Protein Coverage
| Supporting Peptides
|

Protein Coverage:

Supporting Peptides:

| Peptide | Uniq | -10lgP | Mass | Length | ppm | m/z | z | RT | Fraction | Scan | Source File | Area BuCaMH03\_B17 | #Feature | #Feature BuCaMH03\_B17 | Start | End | PTM | AScore | Found By |
| --- | --- | --- | --- | --- | --- | --- | --- | --- | --- | --- | --- | --- | --- | --- | --- | --- | --- | --- | --- |
| L.DEVIPFMTVFER.S | Y | 62.24 | 1481.7224 | 12 | 0.6 | 741.8689 | 2 | 90.82 | 1 | 60896 | BuCaMH03.raw | 1.9077E7 | 1 | 1 | 38 | 49 |  |  | PEAKS DB |
| Q.LDEVIPFMTVFER.S | Y | 60.82 | 1594.8065 | 13 | 0.7 | 798.4111 | 2 | 91.54 | 1 | 61776 | BuCaMH03.raw | 3.1721E8 | 1 | 1 | 37 | 49 |  |  | PEAKS DB |
| R.SSC(+57.02)RPIETMIDIYQEYPDEVEYIFKPSC(+57.02)VLLMK.C | Y | 58.94 | 4052.9282 | 33 | 0.5 | 1014.2398 | 4 | 90.87 | 1 | 61330 | BuCaMH03.raw | 5.2638E7 | 1 | 1 | 50 | 82 | Carbamidomethylation | C3:Carbamidomethylation:1000.00;C28:Carbamidomethylation:1000.00 | PEAKS DB |
| R.HFQSQHIHPMSFQQH.S | Y | 58.54 | 1887.8588 | 15 | 0.4 | 472.9721 | 4 | 22.46 | 1 | 7191 | BuCaMH03.raw | 7.9421E6 | 1 | 1 | 111 | 125 |  |  | PEAKS DB |
| K.QLDEVIPFMTVFER.S | Y | 56.59 | 1722.8650 | 14 | 0.3 | 862.4401 | 2 | 98.36 | 1 | 67114 | BuCaMH03.raw | 9.7411E5 | 1 | 1 | 36 | 49 |  |  | PEAKS DB |
| R.HFQSQHIHPMSFQQHSK.C | Y | 53.49 | 2102.9856 | 17 | -0.1 | 421.6043 | 5 | 21.44 | 1 | 6424 | BuCaMH03.raw | 0 | 0 | 0 | 111 | 127 |  |  | PEAKS DB |
| total 6 peptides |  |  |  |  |  |  |  |  |  |  |  |  |  |  |  |  |  |  |  |
| --- | --- | --- | --- | --- | --- | --- | --- | --- | --- | --- | --- | --- | --- | --- | --- | --- | --- | --- | --- |

T\_157

back to list

  

| Protein Coverage
| Supporting Peptides
|

Protein Coverage:

Supporting Peptides:

| Peptide | Uniq | -10lgP | Mass | Length | ppm | m/z | z | RT | Fraction | Scan | Source File | Area BuCaMH03\_B17 | #Feature | #Feature BuCaMH03\_B17 | Start | End | PTM | AScore | Found By |
| --- | --- | --- | --- | --- | --- | --- | --- | --- | --- | --- | --- | --- | --- | --- | --- | --- | --- | --- | --- |
| L.DEVIPFMTVFER.S | Y | 62.24 | 1481.7224 | 12 | 0.6 | 741.8689 | 2 | 90.82 | 1 | 60896 | BuCaMH03.raw | 1.9077E7 | 1 | 1 | 38 | 49 |  |  | PEAKS DB |
| Q.LDEVIPFMTVFER.S | Y | 60.82 | 1594.8065 | 13 | 0.7 | 798.4111 | 2 | 91.54 | 1 | 61776 | BuCaMH03.raw | 3.1721E8 | 1 | 1 | 37 | 49 |  |  | PEAKS DB |
| R.SSC(+57.02)RPIETMIDIYQEYPDEVEYIFKPSC(+57.02)VLLMK.C | Y | 58.94 | 4052.9282 | 33 | 0.5 | 1014.2398 | 4 | 90.87 | 1 | 61330 | BuCaMH03.raw | 5.2638E7 | 1 | 1 | 50 | 82 | Carbamidomethylation | C3:Carbamidomethylation:1000.00;C28:Carbamidomethylation:1000.00 | PEAKS DB |
| R.HFQSQHIHPMSFQQH.S | Y | 58.54 | 1887.8588 | 15 | 0.4 | 472.9721 | 4 | 22.46 | 1 | 7191 | BuCaMH03.raw | 7.9421E6 | 1 | 1 | 111 | 125 |  |  | PEAKS DB |
| K.QLDEVIPFMTVFER.S | Y | 56.59 | 1722.8650 | 14 | 0.3 | 862.4401 | 2 | 98.36 | 1 | 67114 | BuCaMH03.raw | 9.7411E5 | 1 | 1 | 36 | 49 |  |  | PEAKS DB |
| R.HFQSQHIHPMSFQQHSK.C | Y | 53.49 | 2102.9856 | 17 | -0.1 | 421.6043 | 5 | 21.44 | 1 | 6424 | BuCaMH03.raw | 0 | 0 | 0 | 111 | 127 |  |  | PEAKS DB |
| total 6 peptides |  |  |  |  |  |  |  |  |  |  |  |  |  |  |  |  |  |  |  |
| --- | --- | --- | --- | --- | --- | --- | --- | --- | --- | --- | --- | --- | --- | --- | --- | --- | --- | --- | --- |

T\_151

back to list

  

| Protein Coverage
| Supporting Peptides
|

Protein Coverage:

Supporting Peptides:

| Peptide | Uniq | -10lgP | Mass | Length | ppm | m/z | z | RT | Fraction | Scan | Source File | Area BuCaMH03\_B17 | #Feature | #Feature BuCaMH03\_B17 | Start | End | PTM | AScore | Found By |
| --- | --- | --- | --- | --- | --- | --- | --- | --- | --- | --- | --- | --- | --- | --- | --- | --- | --- | --- | --- |
| L.DEVIPFMTVFER.S | Y | 62.24 | 1481.7224 | 12 | 0.6 | 741.8689 | 2 | 90.82 | 1 | 60896 | BuCaMH03.raw | 1.9077E7 | 1 | 1 | 38 | 49 |  |  | PEAKS DB |
| Q.LDEVIPFMTVFER.S | Y | 60.82 | 1594.8065 | 13 | 0.7 | 798.4111 | 2 | 91.54 | 1 | 61776 | BuCaMH03.raw | 3.1721E8 | 1 | 1 | 37 | 49 |  |  | PEAKS DB |
| R.SSC(+57.02)RPIETMIDIYQEYPDEVEYIFKPSC(+57.02)VLLMK.C | Y | 58.94 | 4052.9282 | 33 | 0.5 | 1014.2398 | 4 | 90.87 | 1 | 61330 | BuCaMH03.raw | 5.2638E7 | 1 | 1 | 50 | 82 | Carbamidomethylation | C3:Carbamidomethylation:1000.00;C28:Carbamidomethylation:1000.00 | PEAKS DB |
| R.HFQSQHIHPMSFQQH.S | Y | 58.54 | 1887.8588 | 15 | 0.4 | 472.9721 | 4 | 22.46 | 1 | 7191 | BuCaMH03.raw | 7.9421E6 | 1 | 1 | 111 | 125 |  |  | PEAKS DB |
| K.QLDEVIPFMTVFER.S | Y | 56.59 | 1722.8650 | 14 | 0.3 | 862.4401 | 2 | 98.36 | 1 | 67114 | BuCaMH03.raw | 9.7411E5 | 1 | 1 | 36 | 49 |  |  | PEAKS DB |
| R.HFQSQHIHPMSFQQHSK.C | Y | 53.49 | 2102.9856 | 17 | -0.1 | 421.6043 | 5 | 21.44 | 1 | 6424 | BuCaMH03.raw | 0 | 0 | 0 | 111 | 127 |  |  | PEAKS DB |
| total 6 peptides |  |  |  |  |  |  |  |  |  |  |  |  |  |  |  |  |  |  |  |
| --- | --- | --- | --- | --- | --- | --- | --- | --- | --- | --- | --- | --- | --- | --- | --- | --- | --- | --- | --- |

T\_152

back to list

  

| Protein Coverage
| Supporting Peptides
|

Protein Coverage:

Supporting Peptides:

| Peptide | Uniq | -10lgP | Mass | Length | ppm | m/z | z | RT | Fraction | Scan | Source File | Area BuCaMH03\_B17 | #Feature | #Feature BuCaMH03\_B17 | Start | End | PTM | AScore | Found By |
| --- | --- | --- | --- | --- | --- | --- | --- | --- | --- | --- | --- | --- | --- | --- | --- | --- | --- | --- | --- |
| L.DEVIPFMTVFER.S | Y | 62.24 | 1481.7224 | 12 | 0.6 | 741.8689 | 2 | 90.82 | 1 | 60896 | BuCaMH03.raw | 1.9077E7 | 1 | 1 | 38 | 49 |  |  | PEAKS DB |
| Q.LDEVIPFMTVFER.S | Y | 60.82 | 1594.8065 | 13 | 0.7 | 798.4111 | 2 | 91.54 | 1 | 61776 | BuCaMH03.raw | 3.1721E8 | 1 | 1 | 37 | 49 |  |  | PEAKS DB |
| R.SSC(+57.02)RPIETMIDIYQEYPDEVEYIFKPSC(+57.02)VLLMK.C | Y | 58.94 | 4052.9282 | 33 | 0.5 | 1014.2398 | 4 | 90.87 | 1 | 61330 | BuCaMH03.raw | 5.2638E7 | 1 | 1 | 50 | 82 | Carbamidomethylation | C3:Carbamidomethylation:1000.00;C28:Carbamidomethylation:1000.00 | PEAKS DB |
| R.HFQSQHIHPMSFQQH.S | Y | 58.54 | 1887.8588 | 15 | 0.4 | 472.9721 | 4 | 22.46 | 1 | 7191 | BuCaMH03.raw | 7.9421E6 | 1 | 1 | 111 | 125 |  |  | PEAKS DB |
| K.QLDEVIPFMTVFER.S | Y | 56.59 | 1722.8650 | 14 | 0.3 | 862.4401 | 2 | 98.36 | 1 | 67114 | BuCaMH03.raw | 9.7411E5 | 1 | 1 | 36 | 49 |  |  | PEAKS DB |
| R.HFQSQHIHPMSFQQHSK.C | Y | 53.49 | 2102.9856 | 17 | -0.1 | 421.6043 | 5 | 21.44 | 1 | 6424 | BuCaMH03.raw | 0 | 0 | 0 | 111 | 127 |  |  | PEAKS DB |
| total 6 peptides |  |  |  |  |  |  |  |  |  |  |  |  |  |  |  |  |  |  |  |
| --- | --- | --- | --- | --- | --- | --- | --- | --- | --- | --- | --- | --- | --- | --- | --- | --- | --- | --- | --- |

T\_175

back to list

  

| Protein Coverage
| Supporting Peptides
|

Protein Coverage:

Supporting Peptides:

| Peptide | Uniq | -10lgP | Mass | Length | ppm | m/z | z | RT | Fraction | Scan | Source File | Area BuCaMH03\_B17 | #Feature | #Feature BuCaMH03\_B17 | Start | End | PTM | AScore | Found By |
| --- | --- | --- | --- | --- | --- | --- | --- | --- | --- | --- | --- | --- | --- | --- | --- | --- | --- | --- | --- |
| R.DTYQTC(+57.02)EEGHVC(+57.02)YSFVTIEPPNR.F | Y | 118.75 | 2801.2061 | 23 | 0.4 | 934.7430 | 3 | 57.43 | 1 | 33838 | BuCaMH03.raw | 2.3921E8 | 3 | 3 | 30 | 52 | Carbamidomethylation | C6:Carbamidomethylation:1000.00;C12:Carbamidomethylation:1000.00 | PEAKS DB |
| Y.SFVTIEPPNR.F | Y | 63.76 | 1158.6033 | 10 | 0.9 | 580.3094 | 2 | 43.97 | 1 | 22414 | BuCaMH03.raw | 2.2714E6 | 1 | 1 | 43 | 52 |  |  | PEAKS DB |
| total 2 peptides |  |  |  |  |  |  |  |  |  |  |  |  |  |  |  |  |  |  |  |
| --- | --- | --- | --- | --- | --- | --- | --- | --- | --- | --- | --- | --- | --- | --- | --- | --- | --- | --- | --- |

AHJ80886.1

back to list

  

| Protein Coverage
| Supporting Peptides
|

Protein Coverage:

Supporting Peptides:

| Peptide | Uniq | -10lgP | Mass | Length | ppm | m/z | z | RT | Fraction | Scan | Source File | Area BuCaMH03\_B17 | #Feature | #Feature BuCaMH03\_B17 | Start | End | PTM | AScore | Found By |
| --- | --- | --- | --- | --- | --- | --- | --- | --- | --- | --- | --- | --- | --- | --- | --- | --- | --- | --- | --- |
| R.VPTYVPLEMEK.T | N | 71.52 | 1304.6686 | 11 | 0.5 | 653.3419 | 2 | 52.81 | 1 | 29825 | BuCaMH03.raw | 1.5662E6 | 1 | 1 | 316 | 326 |  |  | PEAKS DB |
| R.VVSLNVLC(+57.02)TK.C | Y | 69.31 | 1131.6322 | 10 | -0.5 | 566.8231 | 2 | 49.07 | 1 | 26714 | BuCaMH03.raw | 2.6873E6 | 1 | 1 | 304 | 313 | Carbamidomethylation | C8:Carbamidomethylation:1000.00 | PEAKS DB |
| total 2 peptides |  |  |  |  |  |  |  |  |  |  |  |  |  |  |  |  |  |  |  |
| --- | --- | --- | --- | --- | --- | --- | --- | --- | --- | --- | --- | --- | --- | --- | --- | --- | --- | --- | --- |

T\_132

back to list

  

| Protein Coverage
| Supporting Peptides
|

Protein Coverage:

Supporting Peptides:

| Peptide | Uniq | -10lgP | Mass | Length | ppm | m/z | z | RT | Fraction | Scan | Source File | Area BuCaMH03\_B17 | #Feature | #Feature BuCaMH03\_B17 | Start | End | PTM | AScore | Found By |
| --- | --- | --- | --- | --- | --- | --- | --- | --- | --- | --- | --- | --- | --- | --- | --- | --- | --- | --- | --- |
| R.RGC(+57.02)AATC(+57.02)PESSHMVNVEC(+57.02)C(+57.02)STDK.C | Y | 92.69 | 2655.0605 | 23 | 0.1 | 664.7725 | 4 | 20.35 | 1 | 5945 | BuCaMH03.raw | 5.5361E6 | 1 | 1 | 59 | 81 | Carbamidomethylation | C3:Carbamidomethylation:1000.00;C7:Carbamidomethylation:1000.00;C18:Carbamidomethylation:1000.00;C19:Carbamidomethylation:1000.00 | PEAKS DB |
| R.RGC(+57.02)AATC(+57.02)PESSHMVNVEC(+57.02)C(+57.02)STDKC(+57.02)NQ | Y | 72.98 | 3057.1926 | 26 | -0.2 | 765.3053 | 4 | 21.07 | 1 | 6155 | BuCaMH03.raw | 2.1644E6 | 1 | 1 | 59 | 84 | Carbamidomethylation | C3:Carbamidomethylation:1000.00;C7:Carbamidomethylation:1000.00;C18:Carbamidomethylation:1000.00;C19:Carbamidomethylation:1000.00;C24:Carbamidomethylation:1000.00 | PEAKS DB |
| total 2 peptides |  |  |  |  |  |  |  |  |  |  |  |  |  |  |  |  |  |  |  |
| --- | --- | --- | --- | --- | --- | --- | --- | --- | --- | --- | --- | --- | --- | --- | --- | --- | --- | --- | --- |

Q9W729.1

back to list

  

| Protein Coverage
| Supporting Peptides
|

Protein Coverage:

Supporting Peptides:

| Peptide | Uniq | -10lgP | Mass | Length | ppm | m/z | z | RT | Fraction | Scan | Source File | Area BuCaMH03\_B17 | #Feature | #Feature BuCaMH03\_B17 | Start | End | PTM | AScore | Found By |
| --- | --- | --- | --- | --- | --- | --- | --- | --- | --- | --- | --- | --- | --- | --- | --- | --- | --- | --- | --- |
| A.VIEQGC(+57.02)VATC(+57.02)PEFR.S | Y | 88.05 | 1664.7650 | 14 | 0.5 | 833.3902 | 2 | 40.11 | 1 | 19491 | BuCaMH03.raw | 3.2121E8 | 3 | 3 | 58 | 71 | Carbamidomethylation | C6:Carbamidomethylation:1000.00;C10:Carbamidomethylation:1000.00 | PEAKS DB |
| V.IEQGC(+57.02)VATC(+57.02)PEFR.S | Y | 66.77 | 1565.6967 | 13 | 0.4 | 783.8559 | 2 | 35.92 | 1 | 16214 | BuCaMH03.raw | 1.0834E7 | 1 | 1 | 59 | 71 | Carbamidomethylation | C5:Carbamidomethylation:1000.00;C9:Carbamidomethylation:1000.00 | PEAKS DB |
| total 2 peptides |  |  |  |  |  |  |  |  |  |  |  |  |  |  |  |  |  |  |  |
| --- | --- | --- | --- | --- | --- | --- | --- | --- | --- | --- | --- | --- | --- | --- | --- | --- | --- | --- | --- |

CAB46659.1

back to list

  

| Protein Coverage
| Supporting Peptides
|

Protein Coverage:

Supporting Peptides:

| Peptide | Uniq | -10lgP | Mass | Length | ppm | m/z | z | RT | Fraction | Scan | Source File | Area BuCaMH03\_B17 | #Feature | #Feature BuCaMH03\_B17 | Start | End | PTM | AScore | Found By |
| --- | --- | --- | --- | --- | --- | --- | --- | --- | --- | --- | --- | --- | --- | --- | --- | --- | --- | --- | --- |
| A.VIEQGC(+57.02)VATC(+57.02)PEFR.S | Y | 88.05 | 1664.7650 | 14 | 0.5 | 833.3902 | 2 | 40.11 | 1 | 19491 | BuCaMH03.raw | 3.2121E8 | 3 | 3 | 58 | 71 | Carbamidomethylation | C6:Carbamidomethylation:1000.00;C10:Carbamidomethylation:1000.00 | PEAKS DB |
| V.IEQGC(+57.02)VATC(+57.02)PEFR.S | Y | 66.77 | 1565.6967 | 13 | 0.4 | 783.8559 | 2 | 35.92 | 1 | 16214 | BuCaMH03.raw | 1.0834E7 | 1 | 1 | 59 | 71 | Carbamidomethylation | C5:Carbamidomethylation:1000.00;C9:Carbamidomethylation:1000.00 | PEAKS DB |
| total 2 peptides |  |  |  |  |  |  |  |  |  |  |  |  |  |  |  |  |  |  |  |
| --- | --- | --- | --- | --- | --- | --- | --- | --- | --- | --- | --- | --- | --- | --- | --- | --- | --- | --- | --- |

T\_102

back to list

  

| Protein Coverage
| Supporting Peptides
|

Protein Coverage:

Supporting Peptides:

| Peptide | Uniq | -10lgP | Mass | Length | ppm | m/z | z | RT | Fraction | Scan | Source File | Area BuCaMH03\_B17 | #Feature | #Feature BuCaMH03\_B17 | Start | End | PTM | AScore | Found By |
| --- | --- | --- | --- | --- | --- | --- | --- | --- | --- | --- | --- | --- | --- | --- | --- | --- | --- | --- | --- |
| K.VFDYIDWIK.G | N | 72.95 | 1197.6069 | 9 | 0.6 | 599.8111 | 2 | 77.99 | 1 | 50633 | BuCaMH03.raw | 2.2549E6 | 1 | 1 | 270 | 278 |  |  | PEAKS DB |
| K.GDSGGPLIC(+57.02)NGQIQGIVSWGR.F | Y | 66.04 | 2170.0588 | 21 | 0.0 | 1086.0367 | 2 | 71.90 | 1 | 45805 | BuCaMH03.raw | 8.7812E5 | 1 | 1 | 235 | 255 | Carbamidomethylation | C9:Carbamidomethylation:1000.00 | PEAKS DB |
| K.HIEPLSLPSRPPSMGSVC(+57.02)TVMGWGTITSPK.V | Y | 58.55 | 3221.6035 | 30 | 0.7 | 806.4088 | 4 | 70.11 | 1 | 44237 | BuCaMH03.raw | 1.0365E6 | 1 | 1 | 159 | 188 | Carbamidomethylation | C18:Carbamidomethylation:1000.00 | PEAKS DB |
| R.FPC(+57.02)AQLLEPGVYTK.V | N | 57.74 | 1621.8174 | 14 | 0.6 | 811.9164 | 2 | 62.52 | 1 | 37982 | BuCaMH03.raw | 1.0252E6 | 1 | 1 | 256 | 269 | Carbamidomethylation | C3:Carbamidomethylation:1000.00 | PEAKS DB |
| total 4 peptides |  |  |  |  |  |  |  |  |  |  |  |  |  |  |  |  |  |  |  |
| --- | --- | --- | --- | --- | --- | --- | --- | --- | --- | --- | --- | --- | --- | --- | --- | --- | --- | --- | --- |

ETE56933.1

back to list

  

| Protein Coverage
| Supporting Peptides
|

Protein Coverage:

Supporting Peptides:

| Peptide | Uniq | -10lgP | Mass | Length | ppm | m/z | z | RT | Fraction | Scan | Source File | Area BuCaMH03\_B17 | #Feature | #Feature BuCaMH03\_B17 | Start | End | PTM | AScore | Found By |
| --- | --- | --- | --- | --- | --- | --- | --- | --- | --- | --- | --- | --- | --- | --- | --- | --- | --- | --- | --- |
| K.ITC(+57.02)SAEETFC(+57.02)YK.W | Y | 94.26 | 1507.6323 | 12 | 0.1 | 754.8235 | 2 | 36.59 | 1 | 17633 | BuCaMH03.raw | 3.3402E8 | 10 | 10 | 90 | 101 | Carbamidomethylation | C3:Carbamidomethylation:1000.00;C10:Carbamidomethylation:1000.00 | PEAKS DB |
| T.C(+57.02)SAEETFC(+57.02)YK.W | Y | 63.39 | 1293.5006 | 10 | 0.2 | 647.7577 | 2 | 29.89 | 1 | 12055 | BuCaMH03.raw | 5.743E7 | 1 | 1 | 92 | 101 | Carbamidomethylation | C1:Carbamidomethylation:1000.00;C8:Carbamidomethylation:1000.00 | PEAKS DB |
| I.TC(+57.02)SAEETFC(+57.02)YK.W | Y | 62.90 | 1394.5482 | 11 | 0.4 | 698.2817 | 2 | 30.77 | 1 | 12810 | BuCaMH03.raw | 2.0593E8 | 1 | 1 | 91 | 101 | Carbamidomethylation | C2:Carbamidomethylation:1000.00;C9:Carbamidomethylation:1000.00 | PEAKS DB |
| total 3 peptides |  |  |  |  |  |  |  |  |  |  |  |  |  |  |  |  |  |  |  |
| --- | --- | --- | --- | --- | --- | --- | --- | --- | --- | --- | --- | --- | --- | --- | --- | --- | --- | --- | --- |

1IJC

back to list

  

| Protein Coverage
| Supporting Peptides
|

Protein Coverage:

Supporting Peptides:

| Peptide | Uniq | -10lgP | Mass | Length | ppm | m/z | z | RT | Fraction | Scan | Source File | Area BuCaMH03\_B17 | #Feature | #Feature BuCaMH03\_B17 | Start | End | PTM | AScore | Found By |
| --- | --- | --- | --- | --- | --- | --- | --- | --- | --- | --- | --- | --- | --- | --- | --- | --- | --- | --- | --- |
| K.ITC(+57.02)SAEETFC(+57.02)YK.W | Y | 94.26 | 1507.6323 | 12 | 0.1 | 754.8235 | 2 | 36.59 | 1 | 17633 | BuCaMH03.raw | 3.3402E8 | 10 | 10 | 15 | 26 | Carbamidomethylation | C3:Carbamidomethylation:1000.00;C10:Carbamidomethylation:1000.00 | PEAKS DB |
| T.C(+57.02)SAEETFC(+57.02)YK.W | Y | 63.39 | 1293.5006 | 10 | 0.2 | 647.7577 | 2 | 29.89 | 1 | 12055 | BuCaMH03.raw | 5.743E7 | 1 | 1 | 17 | 26 | Carbamidomethylation | C1:Carbamidomethylation:1000.00;C8:Carbamidomethylation:1000.00 | PEAKS DB |
| I.TC(+57.02)SAEETFC(+57.02)YK.W | Y | 62.90 | 1394.5482 | 11 | 0.4 | 698.2817 | 2 | 30.77 | 1 | 12810 | BuCaMH03.raw | 2.0593E8 | 1 | 1 | 16 | 26 | Carbamidomethylation | C2:Carbamidomethylation:1000.00;C9:Carbamidomethylation:1000.00 | PEAKS DB |
| total 3 peptides |  |  |  |  |  |  |  |  |  |  |  |  |  |  |  |  |  |  |  |
| --- | --- | --- | --- | --- | --- | --- | --- | --- | --- | --- | --- | --- | --- | --- | --- | --- | --- | --- | --- |

1F94

back to list

  

| Protein Coverage
| Supporting Peptides
|

Protein Coverage:

Supporting Peptides:

| Peptide | Uniq | -10lgP | Mass | Length | ppm | m/z | z | RT | Fraction | Scan | Source File | Area BuCaMH03\_B17 | #Feature | #Feature BuCaMH03\_B17 | Start | End | PTM | AScore | Found By |
| --- | --- | --- | --- | --- | --- | --- | --- | --- | --- | --- | --- | --- | --- | --- | --- | --- | --- | --- | --- |
| K.ITC(+57.02)SAEETFC(+57.02)YK.W | Y | 94.26 | 1507.6323 | 12 | 0.1 | 754.8235 | 2 | 36.59 | 1 | 17633 | BuCaMH03.raw | 3.3402E8 | 10 | 10 | 15 | 26 | Carbamidomethylation | C3:Carbamidomethylation:1000.00;C10:Carbamidomethylation:1000.00 | PEAKS DB |
| T.C(+57.02)SAEETFC(+57.02)YK.W | Y | 63.39 | 1293.5006 | 10 | 0.2 | 647.7577 | 2 | 29.89 | 1 | 12055 | BuCaMH03.raw | 5.743E7 | 1 | 1 | 17 | 26 | Carbamidomethylation | C1:Carbamidomethylation:1000.00;C8:Carbamidomethylation:1000.00 | PEAKS DB |
| I.TC(+57.02)SAEETFC(+57.02)YK.W | Y | 62.90 | 1394.5482 | 11 | 0.4 | 698.2817 | 2 | 30.77 | 1 | 12810 | BuCaMH03.raw | 2.0593E8 | 1 | 1 | 16 | 26 | Carbamidomethylation | C2:Carbamidomethylation:1000.00;C9:Carbamidomethylation:1000.00 | PEAKS DB |
| total 3 peptides |  |  |  |  |  |  |  |  |  |  |  |  |  |  |  |  |  |  |  |
| --- | --- | --- | --- | --- | --- | --- | --- | --- | --- | --- | --- | --- | --- | --- | --- | --- | --- | --- | --- |

P81782.1

back to list

  

| Protein Coverage
| Supporting Peptides
|

Protein Coverage:

Supporting Peptides:

| Peptide | Uniq | -10lgP | Mass | Length | ppm | m/z | z | RT | Fraction | Scan | Source File | Area BuCaMH03\_B17 | #Feature | #Feature BuCaMH03\_B17 | Start | End | PTM | AScore | Found By |
| --- | --- | --- | --- | --- | --- | --- | --- | --- | --- | --- | --- | --- | --- | --- | --- | --- | --- | --- | --- |
| K.ITC(+57.02)SAEETFC(+57.02)YK.W | Y | 94.26 | 1507.6323 | 12 | 0.1 | 754.8235 | 2 | 36.59 | 1 | 17633 | BuCaMH03.raw | 3.3402E8 | 10 | 10 | 15 | 26 | Carbamidomethylation | C3:Carbamidomethylation:1000.00;C10:Carbamidomethylation:1000.00 | PEAKS DB |
| T.C(+57.02)SAEETFC(+57.02)YK.W | Y | 63.39 | 1293.5006 | 10 | 0.2 | 647.7577 | 2 | 29.89 | 1 | 12055 | BuCaMH03.raw | 5.743E7 | 1 | 1 | 17 | 26 | Carbamidomethylation | C1:Carbamidomethylation:1000.00;C8:Carbamidomethylation:1000.00 | PEAKS DB |
| I.TC(+57.02)SAEETFC(+57.02)YK.W | Y | 62.90 | 1394.5482 | 11 | 0.4 | 698.2817 | 2 | 30.77 | 1 | 12810 | BuCaMH03.raw | 2.0593E8 | 1 | 1 | 16 | 26 | Carbamidomethylation | C2:Carbamidomethylation:1000.00;C9:Carbamidomethylation:1000.00 | PEAKS DB |
| total 3 peptides |  |  |  |  |  |  |  |  |  |  |  |  |  |  |  |  |  |  |  |
| --- | --- | --- | --- | --- | --- | --- | --- | --- | --- | --- | --- | --- | --- | --- | --- | --- | --- | --- | --- |

ABN72545.1

back to list

  

| Protein Coverage
| Supporting Peptides
|

Protein Coverage:

Supporting Peptides:

| Peptide | Uniq | -10lgP | Mass | Length | ppm | m/z | z | RT | Fraction | Scan | Source File | Area BuCaMH03\_B17 | #Feature | #Feature BuCaMH03\_B17 | Start | End | PTM | AScore | Found By |
| --- | --- | --- | --- | --- | --- | --- | --- | --- | --- | --- | --- | --- | --- | --- | --- | --- | --- | --- | --- |
| K.VFDYIDWIK.G | N | 72.95 | 1197.6069 | 9 | 0.6 | 599.8111 | 2 | 77.99 | 1 | 50633 | BuCaMH03.raw | 2.2549E6 | 1 | 1 | 268 | 276 |  |  | PEAKS DB |
| K.GDSGGPLIC(+57.02)DGQIQGIVSWGR.F | Y | 68.41 | 2171.0430 | 21 | 0.3 | 1086.5291 | 2 | 74.18 | 1 | 47633 | BuCaMH03.raw | 7.7512E5 | 1 | 1 | 233 | 253 | Carbamidomethylation | C9:Carbamidomethylation:1000.00 | PEAKS DB |
| R.FPC(+57.02)AQLLEPGVYTK.V | N | 57.74 | 1621.8174 | 14 | 0.6 | 811.9164 | 2 | 62.52 | 1 | 37982 | BuCaMH03.raw | 1.0252E6 | 1 | 1 | 254 | 267 | Carbamidomethylation | C3:Carbamidomethylation:1000.00 | PEAKS DB |
| total 3 peptides |  |  |  |  |  |  |  |  |  |  |  |  |  |  |  |  |  |  |  |
| --- | --- | --- | --- | --- | --- | --- | --- | --- | --- | --- | --- | --- | --- | --- | --- | --- | --- | --- | --- |

A8QL57.1

back to list

  

| Protein Coverage
| Supporting Peptides
|

Protein Coverage:

Supporting Peptides:

| Peptide | Uniq | -10lgP | Mass | Length | ppm | m/z | z | RT | Fraction | Scan | Source File | Area BuCaMH03\_B17 | #Feature | #Feature BuCaMH03\_B17 | Start | End | PTM | AScore | Found By |
| --- | --- | --- | --- | --- | --- | --- | --- | --- | --- | --- | --- | --- | --- | --- | --- | --- | --- | --- | --- |
| K.VFDYIDWIK.G | N | 72.95 | 1197.6069 | 9 | 0.6 | 599.8111 | 2 | 77.99 | 1 | 50633 | BuCaMH03.raw | 2.2549E6 | 1 | 1 | 268 | 276 |  |  | PEAKS DB |
| K.GDSGGPLIC(+57.02)DGQIQGIVSWGR.F | Y | 68.41 | 2171.0430 | 21 | 0.3 | 1086.5291 | 2 | 74.18 | 1 | 47633 | BuCaMH03.raw | 7.7512E5 | 1 | 1 | 233 | 253 | Carbamidomethylation | C9:Carbamidomethylation:1000.00 | PEAKS DB |
| R.FPC(+57.02)AQLLEPGVYTK.V | N | 57.74 | 1621.8174 | 14 | 0.6 | 811.9164 | 2 | 62.52 | 1 | 37982 | BuCaMH03.raw | 1.0252E6 | 1 | 1 | 254 | 267 | Carbamidomethylation | C3:Carbamidomethylation:1000.00 | PEAKS DB |
| total 3 peptides |  |  |  |  |  |  |  |  |  |  |  |  |  |  |  |  |  |  |  |
| --- | --- | --- | --- | --- | --- | --- | --- | --- | --- | --- | --- | --- | --- | --- | --- | --- | --- | --- | --- |

XP\_026555558.1

back to list

  

| Protein Coverage
| Supporting Peptides
|

Protein Coverage:

Supporting Peptides:

| Peptide | Uniq | -10lgP | Mass | Length | ppm | m/z | z | RT | Fraction | Scan | Source File | Area BuCaMH03\_B17 | #Feature | #Feature BuCaMH03\_B17 | Start | End | PTM | AScore | Found By |
| --- | --- | --- | --- | --- | --- | --- | --- | --- | --- | --- | --- | --- | --- | --- | --- | --- | --- | --- | --- |
| K.C(+57.02)QNADVYGAEISENMFC(+57.02)AGYLDGR.S | Y | 75.99 | 2739.1362 | 24 | 1.5 | 914.0540 | 3 | 74.89 | 1 | 48289 | BuCaMH03.raw | 7.903E5 | 1 | 1 | 463 | 486 | Carbamidomethylation | C1:Carbamidomethylation:1000.00;C17:Carbamidomethylation:1000.00 | PEAKS DB |
| R.SDAC(+57.02)QGDSGGPLAC(+57.02)ER.D | Y | 69.39 | 1678.6675 | 16 | 0.2 | 840.3412 | 2 | 22.91 | 1 | 7194 | BuCaMH03.raw | 1.0147E6 | 1 | 1 | 487 | 502 | Carbamidomethylation | C4:Carbamidomethylation:1000.00;C14:Carbamidomethylation:1000.00 | PEAKS DB |
| R.SPDEDEQPWC(+57.02)YFIK.D | N | 68.05 | 1812.7665 | 14 | 0.4 | 907.3909 | 2 | 74.89 | 1 | 48131 | BuCaMH03.raw | 2.3067E6 | 1 | 1 | 235 | 248 | Carbamidomethylation | C10:Carbamidomethylation:1000.00 | PEAKS DB |
| R.IVGGSSALPGSHPWLASINVGR.N | N | 58.18 | 2174.1597 | 22 | 0.1 | 725.7272 | 3 | 59.37 | 1 | 35332 | BuCaMH03.raw | 7.2346E5 | 1 | 1 | 304 | 325 |  |  | PEAKS DB |
| R.YSNVVQEALIPIIPDYK.C | Y | 53.12 | 1961.0509 | 17 | 0.3 | 654.6911 | 3 | 81.74 | 1 | 53784 | BuCaMH03.raw | 4.2127E5 | 1 | 1 | 446 | 462 |  |  | PEAKS DB |
| total 5 peptides |  |  |  |  |  |  |  |  |  |  |  |  |  |  |  |  |  |  |  |
| --- | --- | --- | --- | --- | --- | --- | --- | --- | --- | --- | --- | --- | --- | --- | --- | --- | --- | --- | --- |

T\_54

back to list

  

| Protein Coverage
| Supporting Peptides
|

Protein Coverage:

Supporting Peptides:

| Peptide | Uniq | -10lgP | Mass | Length | ppm | m/z | z | RT | Fraction | Scan | Source File | Area BuCaMH03\_B17 | #Feature | #Feature BuCaMH03\_B17 | Start | End | PTM | AScore | Found By |
| --- | --- | --- | --- | --- | --- | --- | --- | --- | --- | --- | --- | --- | --- | --- | --- | --- | --- | --- | --- |
| I.C(+57.02)LTC(+57.02)PDKYC(+57.02)QTVHTC(+57.02)R.N | Y | 68.74 | 2097.8853 | 16 | 0.1 | 525.4786 | 4 | 20.10 | 1 | 5810 | BuCaMH03.raw | 6.404E7 | 2 | 2 | 24 | 39 | Carbamidomethylation | C1:Carbamidomethylation:1000.00;C4:Carbamidomethylation:1000.00;C9:Carbamidomethylation:1000.00;C15:Carbamidomethylation:1000.00 | PEAKS DB |
| R.FYDKNQLGWR.A | Y | 60.99 | 1325.6516 | 10 | -0.4 | 442.8910 | 3 | 34.92 | 1 | 15387 | BuCaMH03.raw | 3.8137E6 | 2 | 2 | 49 | 58 |  |  | PEAKS DB |
| F.YDKNQLGWR.A | Y | 58.79 | 1178.5833 | 9 | -0.4 | 590.2986 | 2 | 25.14 | 1 | 8823 | BuCaMH03.raw | 1.1845E8 | 2 | 2 | 50 | 58 |  |  | PEAKS DB |
| total 3 peptides |  |  |  |  |  |  |  |  |  |  |  |  |  |  |  |  |  |  |  |
| --- | --- | --- | --- | --- | --- | --- | --- | --- | --- | --- | --- | --- | --- | --- | --- | --- | --- | --- | --- |

Q8AY42.2

back to list

  

| Protein Coverage
| Supporting Peptides
|

Protein Coverage:

Supporting Peptides:

| Peptide | Uniq | -10lgP | Mass | Length | ppm | m/z | z | RT | Fraction | Scan | Source File | Area BuCaMH03\_B17 | #Feature | #Feature BuCaMH03\_B17 | Start | End | PTM | AScore | Found By |
| --- | --- | --- | --- | --- | --- | --- | --- | --- | --- | --- | --- | --- | --- | --- | --- | --- | --- | --- | --- |
| E.FTYGGC(+57.02)GGNANNFK.S | Y | 88.26 | 1505.6357 | 14 | -2.1 | 753.8235 | 2 | 31.75 | 1 | 13356 | BuCaMH03.raw | 4.3845E8 | 2 | 2 | 59 | 72 | Carbamidomethylation | C6:Carbamidomethylation:1000.00 | PEAKS DB |
| F.TYGGC(+57.02)GGNANNFK.S | Y | 87.23 | 1358.5674 | 13 | 0.3 | 680.2911 | 2 | 16.91 | 1 | 3953 | BuCaMH03.raw | 3.1257E7 | 1 | 1 | 60 | 72 | Carbamidomethylation | C5:Carbamidomethylation:1000.00 | PEAKS DB |
| T.YGGC(+57.02)GGNANNFK.S | N | 75.97 | 1257.5197 | 12 | 0.4 | 629.7673 | 2 | 13.72 | 1 | 2615 | BuCaMH03.raw | 8.8737E6 | 1 | 1 | 61 | 72 | Carbamidomethylation | C4:Carbamidomethylation:1000.00 | PEAKS DB |
| total 3 peptides |  |  |  |  |  |  |  |  |  |  |  |  |  |  |  |  |  |  |  |
| --- | --- | --- | --- | --- | --- | --- | --- | --- | --- | --- | --- | --- | --- | --- | --- | --- | --- | --- | --- |

Q8AY43.2

back to list

  

| Protein Coverage
| Supporting Peptides
|

Protein Coverage:

Supporting Peptides:

| Peptide | Uniq | -10lgP | Mass | Length | ppm | m/z | z | RT | Fraction | Scan | Source File | Area BuCaMH03\_B17 | #Feature | #Feature BuCaMH03\_B17 | Start | End | PTM | AScore | Found By |
| --- | --- | --- | --- | --- | --- | --- | --- | --- | --- | --- | --- | --- | --- | --- | --- | --- | --- | --- | --- |
| E.FTYGGC(+57.02)GGNANNFK.S | Y | 88.26 | 1505.6357 | 14 | -2.1 | 753.8235 | 2 | 31.75 | 1 | 13356 | BuCaMH03.raw | 4.3845E8 | 2 | 2 | 59 | 72 | Carbamidomethylation | C6:Carbamidomethylation:1000.00 | PEAKS DB |
| F.TYGGC(+57.02)GGNANNFK.S | Y | 87.23 | 1358.5674 | 13 | 0.3 | 680.2911 | 2 | 16.91 | 1 | 3953 | BuCaMH03.raw | 3.1257E7 | 1 | 1 | 60 | 72 | Carbamidomethylation | C5:Carbamidomethylation:1000.00 | PEAKS DB |
| T.YGGC(+57.02)GGNANNFK.S | N | 75.97 | 1257.5197 | 12 | 0.4 | 629.7673 | 2 | 13.72 | 1 | 2615 | BuCaMH03.raw | 8.8737E6 | 1 | 1 | 61 | 72 | Carbamidomethylation | C4:Carbamidomethylation:1000.00 | PEAKS DB |
| total 3 peptides |  |  |  |  |  |  |  |  |  |  |  |  |  |  |  |  |  |  |  |
| --- | --- | --- | --- | --- | --- | --- | --- | --- | --- | --- | --- | --- | --- | --- | --- | --- | --- | --- | --- |

AAL30069.1

back to list

  

| Protein Coverage
| Supporting Peptides
|

Protein Coverage:

Supporting Peptides:

| Peptide | Uniq | -10lgP | Mass | Length | ppm | m/z | z | RT | Fraction | Scan | Source File | Area BuCaMH03\_B17 | #Feature | #Feature BuCaMH03\_B17 | Start | End | PTM | AScore | Found By |
| --- | --- | --- | --- | --- | --- | --- | --- | --- | --- | --- | --- | --- | --- | --- | --- | --- | --- | --- | --- |
| E.FTYGGC(+57.02)GGNANNFK.S | Y | 88.26 | 1505.6357 | 14 | -2.1 | 753.8235 | 2 | 31.75 | 1 | 13356 | BuCaMH03.raw | 4.3845E8 | 2 | 2 | 64 | 77 | Carbamidomethylation | C6:Carbamidomethylation:1000.00 | PEAKS DB |
| F.TYGGC(+57.02)GGNANNFK.S | Y | 87.23 | 1358.5674 | 13 | 0.3 | 680.2911 | 2 | 16.91 | 1 | 3953 | BuCaMH03.raw | 3.1257E7 | 1 | 1 | 65 | 77 | Carbamidomethylation | C5:Carbamidomethylation:1000.00 | PEAKS DB |
| T.YGGC(+57.02)GGNANNFK.S | N | 75.97 | 1257.5197 | 12 | 0.4 | 629.7673 | 2 | 13.72 | 1 | 2615 | BuCaMH03.raw | 8.8737E6 | 1 | 1 | 66 | 77 | Carbamidomethylation | C4:Carbamidomethylation:1000.00 | PEAKS DB |
| total 3 peptides |  |  |  |  |  |  |  |  |  |  |  |  |  |  |  |  |  |  |  |
| --- | --- | --- | --- | --- | --- | --- | --- | --- | --- | --- | --- | --- | --- | --- | --- | --- | --- | --- | --- |

AAL30068.1

back to list

  

| Protein Coverage
| Supporting Peptides
|

Protein Coverage:

Supporting Peptides:

| Peptide | Uniq | -10lgP | Mass | Length | ppm | m/z | z | RT | Fraction | Scan | Source File | Area BuCaMH03\_B17 | #Feature | #Feature BuCaMH03\_B17 | Start | End | PTM | AScore | Found By |
| --- | --- | --- | --- | --- | --- | --- | --- | --- | --- | --- | --- | --- | --- | --- | --- | --- | --- | --- | --- |
| E.FTYGGC(+57.02)GGNANNFK.S | Y | 88.26 | 1505.6357 | 14 | -2.1 | 753.8235 | 2 | 31.75 | 1 | 13356 | BuCaMH03.raw | 4.3845E8 | 2 | 2 | 64 | 77 | Carbamidomethylation | C6:Carbamidomethylation:1000.00 | PEAKS DB |
| F.TYGGC(+57.02)GGNANNFK.S | Y | 87.23 | 1358.5674 | 13 | 0.3 | 680.2911 | 2 | 16.91 | 1 | 3953 | BuCaMH03.raw | 3.1257E7 | 1 | 1 | 65 | 77 | Carbamidomethylation | C5:Carbamidomethylation:1000.00 | PEAKS DB |
| T.YGGC(+57.02)GGNANNFK.S | N | 75.97 | 1257.5197 | 12 | 0.4 | 629.7673 | 2 | 13.72 | 1 | 2615 | BuCaMH03.raw | 8.8737E6 | 1 | 1 | 66 | 77 | Carbamidomethylation | C4:Carbamidomethylation:1000.00 | PEAKS DB |
| total 3 peptides |  |  |  |  |  |  |  |  |  |  |  |  |  |  |  |  |  |  |  |
| --- | --- | --- | --- | --- | --- | --- | --- | --- | --- | --- | --- | --- | --- | --- | --- | --- | --- | --- | --- |

XP\_026544110.1

back to list

  

| Protein Coverage
| Supporting Peptides
|

Protein Coverage:

Supporting Peptides:

| Peptide | Uniq | -10lgP | Mass | Length | ppm | m/z | z | RT | Fraction | Scan | Source File | Area BuCaMH03\_B17 | #Feature | #Feature BuCaMH03\_B17 | Start | End | PTM | AScore | Found By |
| --- | --- | --- | --- | --- | --- | --- | --- | --- | --- | --- | --- | --- | --- | --- | --- | --- | --- | --- | --- |
| K.YIMHPDYSVFNPTENDIVLIK.L | Y | 97.27 | 2507.2407 | 21 | 0.8 | 836.7549 | 3 | 72.89 | 1 | 46619 | BuCaMH03.raw | 6.3406E6 | 1 | 1 | 378 | 398 |  |  | PEAKS DB |
| R.SPDEDEQPWC(+57.02)YFIK.D | N | 68.05 | 1812.7665 | 14 | 0.4 | 907.3909 | 2 | 74.89 | 1 | 48131 | BuCaMH03.raw | 2.3067E6 | 1 | 1 | 235 | 248 | Carbamidomethylation | C10:Carbamidomethylation:1000.00 | PEAKS DB |
| R.IVGGSSALPGSHPWLASINVGR.N | N | 58.18 | 2174.1597 | 22 | 0.1 | 725.7272 | 3 | 59.37 | 1 | 35332 | BuCaMH03.raw | 7.2346E5 | 1 | 1 | 304 | 325 |  |  | PEAKS DB |
| total 3 peptides |  |  |  |  |  |  |  |  |  |  |  |  |  |  |  |  |  |  |  |
| --- | --- | --- | --- | --- | --- | --- | --- | --- | --- | --- | --- | --- | --- | --- | --- | --- | --- | --- | --- |

P41331.1

back to list

  

| Protein Coverage
| Supporting Peptides
|

Protein Coverage:

Supporting Peptides:

| Peptide | Uniq | -10lgP | Mass | Length | ppm | m/z | z | RT | Fraction | Scan | Source File | Area BuCaMH03\_B17 | #Feature | #Feature BuCaMH03\_B17 | Start | End | PTM | AScore | Found By |
| --- | --- | --- | --- | --- | --- | --- | --- | --- | --- | --- | --- | --- | --- | --- | --- | --- | --- | --- | --- |
| K.VIDALTEAVNNLDDVAGALSK.L | N | 92.44 | 2127.1060 | 21 | 0.4 | 710.0428 | 3 | 94.16 | 1 | 63594 | BuCaMH03.raw | 4.2072E6 | 2 | 2 | 62 | 82 |  |  | PEAKS DB |
| K.KVIDALTEAVNNLDDVAGALSK.L | N | 61.22 | 2255.2009 | 22 | -0.2 | 1128.6075 | 2 | 87.59 | 1 | 58386 | BuCaMH03.raw | 3.6124E5 | 1 | 1 | 61 | 82 |  |  | PEAKS DB |
| K.TYFPHFDLSPGSNDLK.V | Y | 58.62 | 1836.8682 | 16 | -0.4 | 613.2964 | 3 | 59.18 | 1 | 35091 | BuCaMH03.raw | 6.0532E5 | 1 | 1 | 41 | 56 |  |  | PEAKS DB |
| total 3 peptides |  |  |  |  |  |  |  |  |  |  |  |  |  |  |  |  |  |  |  |
| --- | --- | --- | --- | --- | --- | --- | --- | --- | --- | --- | --- | --- | --- | --- | --- | --- | --- | --- | --- |

ETE59912.1

back to list

  

| Protein Coverage
| Supporting Peptides
|

Protein Coverage:

Supporting Peptides:

| Peptide | Uniq | -10lgP | Mass | Length | ppm | m/z | z | RT | Fraction | Scan | Source File | Area BuCaMH03\_B17 | #Feature | #Feature BuCaMH03\_B17 | Start | End | PTM | AScore | Found By |
| --- | --- | --- | --- | --- | --- | --- | --- | --- | --- | --- | --- | --- | --- | --- | --- | --- | --- | --- | --- |
| R.IEIESFFEGEDFSETLTR.A | Y | 65.88 | 2147.9897 | 18 | 0.5 | 1075.0027 | 2 | 85.86 | 1 | 56881 | BuCaMH03.raw | 3.5489E5 | 1 | 1 | 307 | 324 |  |  | PEAKS DB |
| K.FEELNMDLFR.S | Y | 63.72 | 1312.6122 | 10 | 0.3 | 657.3135 | 2 | 71.75 | 1 | 45607 | BuCaMH03.raw | 1.0935E6 | 1 | 1 | 327 | 336 |  |  | PEAKS DB |
| K.DAGTIAGLNVMR.I | Y | 60.58 | 1216.6234 | 12 | 0.1 | 609.3190 | 2 | 54.94 | 1 | 31734 | BuCaMH03.raw | 4.5409E5 | 1 | 1 | 186 | 197 |  |  | PEAKS DB |
| total 3 peptides |  |  |  |  |  |  |  |  |  |  |  |  |  |  |  |  |  |  |  |
| --- | --- | --- | --- | --- | --- | --- | --- | --- | --- | --- | --- | --- | --- | --- | --- | --- | --- | --- | --- |

XP\_034262421.1

back to list

  

| Protein Coverage
| Supporting Peptides
|

Protein Coverage:

Supporting Peptides:

| Peptide | Uniq | -10lgP | Mass | Length | ppm | m/z | z | RT | Fraction | Scan | Source File | Area BuCaMH03\_B17 | #Feature | #Feature BuCaMH03\_B17 | Start | End | PTM | AScore | Found By |
| --- | --- | --- | --- | --- | --- | --- | --- | --- | --- | --- | --- | --- | --- | --- | --- | --- | --- | --- | --- |
| R.IEIESFFEGEDFSETLTR.A | Y | 65.88 | 2147.9897 | 18 | 0.5 | 1075.0027 | 2 | 85.86 | 1 | 56881 | BuCaMH03.raw | 3.5489E5 | 1 | 1 | 272 | 289 |  |  | PEAKS DB |
| K.FEELNMDLFR.S | Y | 63.72 | 1312.6122 | 10 | 0.3 | 657.3135 | 2 | 71.75 | 1 | 45607 | BuCaMH03.raw | 1.0935E6 | 1 | 1 | 292 | 301 |  |  | PEAKS DB |
| K.DAGTIAGLNVMR.I | Y | 60.58 | 1216.6234 | 12 | 0.1 | 609.3190 | 2 | 54.94 | 1 | 31734 | BuCaMH03.raw | 4.5409E5 | 1 | 1 | 151 | 162 |  |  | PEAKS DB |
| total 3 peptides |  |  |  |  |  |  |  |  |  |  |  |  |  |  |  |  |  |  |  |
| --- | --- | --- | --- | --- | --- | --- | --- | --- | --- | --- | --- | --- | --- | --- | --- | --- | --- | --- | --- |

JAG67806.1

back to list

  

| Protein Coverage
| Supporting Peptides
|

Protein Coverage:

Supporting Peptides:

| Peptide | Uniq | -10lgP | Mass | Length | ppm | m/z | z | RT | Fraction | Scan | Source File | Area BuCaMH03\_B17 | #Feature | #Feature BuCaMH03\_B17 | Start | End | PTM | AScore | Found By |
| --- | --- | --- | --- | --- | --- | --- | --- | --- | --- | --- | --- | --- | --- | --- | --- | --- | --- | --- | --- |
| R.IEIESFFEGEDFSETLTR.A | Y | 65.88 | 2147.9897 | 18 | 0.5 | 1075.0027 | 2 | 85.86 | 1 | 56881 | BuCaMH03.raw | 3.5489E5 | 1 | 1 | 306 | 323 |  |  | PEAKS DB |
| K.FEELNMDLFR.S | Y | 63.72 | 1312.6122 | 10 | 0.3 | 657.3135 | 2 | 71.75 | 1 | 45607 | BuCaMH03.raw | 1.0935E6 | 1 | 1 | 326 | 335 |  |  | PEAKS DB |
| K.DAGTIAGLNVMR.I | Y | 60.58 | 1216.6234 | 12 | 0.1 | 609.3190 | 2 | 54.94 | 1 | 31734 | BuCaMH03.raw | 4.5409E5 | 1 | 1 | 185 | 196 |  |  | PEAKS DB |
| total 3 peptides |  |  |  |  |  |  |  |  |  |  |  |  |  |  |  |  |  |  |  |
| --- | --- | --- | --- | --- | --- | --- | --- | --- | --- | --- | --- | --- | --- | --- | --- | --- | --- | --- | --- |

XP\_025022112.1

back to list

  

| Protein Coverage
| Supporting Peptides
|

Protein Coverage:

Supporting Peptides:

| Peptide | Uniq | -10lgP | Mass | Length | ppm | m/z | z | RT | Fraction | Scan | Source File | Area BuCaMH03\_B17 | #Feature | #Feature BuCaMH03\_B17 | Start | End | PTM | AScore | Found By |
| --- | --- | --- | --- | --- | --- | --- | --- | --- | --- | --- | --- | --- | --- | --- | --- | --- | --- | --- | --- |
| R.IEIESFFEGEDFSETLTR.A | Y | 65.88 | 2147.9897 | 18 | 0.5 | 1075.0027 | 2 | 85.86 | 1 | 56881 | BuCaMH03.raw | 3.5489E5 | 1 | 1 | 305 | 322 |  |  | PEAKS DB |
| K.FEELNMDLFR.S | Y | 63.72 | 1312.6122 | 10 | 0.3 | 657.3135 | 2 | 71.75 | 1 | 45607 | BuCaMH03.raw | 1.0935E6 | 1 | 1 | 325 | 334 |  |  | PEAKS DB |
| K.DAGTIAGLNVMR.I | Y | 60.58 | 1216.6234 | 12 | 0.1 | 609.3190 | 2 | 54.94 | 1 | 31734 | BuCaMH03.raw | 4.5409E5 | 1 | 1 | 184 | 195 |  |  | PEAKS DB |
| total 3 peptides |  |  |  |  |  |  |  |  |  |  |  |  |  |  |  |  |  |  |  |
| --- | --- | --- | --- | --- | --- | --- | --- | --- | --- | --- | --- | --- | --- | --- | --- | --- | --- | --- | --- |

JAB54174.1

back to list

  

| Protein Coverage
| Supporting Peptides
|

Protein Coverage:

Supporting Peptides:

| Peptide | Uniq | -10lgP | Mass | Length | ppm | m/z | z | RT | Fraction | Scan | Source File | Area BuCaMH03\_B17 | #Feature | #Feature BuCaMH03\_B17 | Start | End | PTM | AScore | Found By |
| --- | --- | --- | --- | --- | --- | --- | --- | --- | --- | --- | --- | --- | --- | --- | --- | --- | --- | --- | --- |
| R.IEIESFFEGEDFSETLTR.A | Y | 65.88 | 2147.9897 | 18 | 0.5 | 1075.0027 | 2 | 85.86 | 1 | 56881 | BuCaMH03.raw | 3.5489E5 | 1 | 1 | 307 | 324 |  |  | PEAKS DB |
| K.FEELNMDLFR.S | Y | 63.72 | 1312.6122 | 10 | 0.3 | 657.3135 | 2 | 71.75 | 1 | 45607 | BuCaMH03.raw | 1.0935E6 | 1 | 1 | 327 | 336 |  |  | PEAKS DB |
| K.DAGTIAGLNVMR.I | Y | 60.58 | 1216.6234 | 12 | 0.1 | 609.3190 | 2 | 54.94 | 1 | 31734 | BuCaMH03.raw | 4.5409E5 | 1 | 1 | 186 | 197 |  |  | PEAKS DB |
| total 3 peptides |  |  |  |  |  |  |  |  |  |  |  |  |  |  |  |  |  |  |  |
| --- | --- | --- | --- | --- | --- | --- | --- | --- | --- | --- | --- | --- | --- | --- | --- | --- | --- | --- | --- |

XP\_026568326.1

back to list

  

| Protein Coverage
| Supporting Peptides
|

Protein Coverage:

Supporting Peptides:

| Peptide | Uniq | -10lgP | Mass | Length | ppm | m/z | z | RT | Fraction | Scan | Source File | Area BuCaMH03\_B17 | #Feature | #Feature BuCaMH03\_B17 | Start | End | PTM | AScore | Found By |
| --- | --- | --- | --- | --- | --- | --- | --- | --- | --- | --- | --- | --- | --- | --- | --- | --- | --- | --- | --- |
| R.IEIESFFEGEDFSETLTR.A | Y | 65.88 | 2147.9897 | 18 | 0.5 | 1075.0027 | 2 | 85.86 | 1 | 56881 | BuCaMH03.raw | 3.5489E5 | 1 | 1 | 307 | 324 |  |  | PEAKS DB |
| K.FEELNMDLFR.S | Y | 63.72 | 1312.6122 | 10 | 0.3 | 657.3135 | 2 | 71.75 | 1 | 45607 | BuCaMH03.raw | 1.0935E6 | 1 | 1 | 327 | 336 |  |  | PEAKS DB |
| K.DAGTIAGLNVMR.I | Y | 60.58 | 1216.6234 | 12 | 0.1 | 609.3190 | 2 | 54.94 | 1 | 31734 | BuCaMH03.raw | 4.5409E5 | 1 | 1 | 186 | 197 |  |  | PEAKS DB |
| total 3 peptides |  |  |  |  |  |  |  |  |  |  |  |  |  |  |  |  |  |  |  |
| --- | --- | --- | --- | --- | --- | --- | --- | --- | --- | --- | --- | --- | --- | --- | --- | --- | --- | --- | --- |

XP\_026542482.1

back to list

  

| Protein Coverage
| Supporting Peptides
|

Protein Coverage:

Supporting Peptides:

| Peptide | Uniq | -10lgP | Mass | Length | ppm | m/z | z | RT | Fraction | Scan | Source File | Area BuCaMH03\_B17 | #Feature | #Feature BuCaMH03\_B17 | Start | End | PTM | AScore | Found By |
| --- | --- | --- | --- | --- | --- | --- | --- | --- | --- | --- | --- | --- | --- | --- | --- | --- | --- | --- | --- |
| R.IEIESFFEGEDFSETLTR.A | Y | 65.88 | 2147.9897 | 18 | 0.5 | 1075.0027 | 2 | 85.86 | 1 | 56881 | BuCaMH03.raw | 3.5489E5 | 1 | 1 | 307 | 324 |  |  | PEAKS DB |
| K.FEELNMDLFR.S | Y | 63.72 | 1312.6122 | 10 | 0.3 | 657.3135 | 2 | 71.75 | 1 | 45607 | BuCaMH03.raw | 1.0935E6 | 1 | 1 | 327 | 336 |  |  | PEAKS DB |
| K.DAGTIAGLNVMR.I | Y | 60.58 | 1216.6234 | 12 | 0.1 | 609.3190 | 2 | 54.94 | 1 | 31734 | BuCaMH03.raw | 4.5409E5 | 1 | 1 | 186 | 197 |  |  | PEAKS DB |
| total 3 peptides |  |  |  |  |  |  |  |  |  |  |  |  |  |  |  |  |  |  |  |
| --- | --- | --- | --- | --- | --- | --- | --- | --- | --- | --- | --- | --- | --- | --- | --- | --- | --- | --- | --- |

JAV50419.1

back to list

  

| Protein Coverage
| Supporting Peptides
|

Protein Coverage:

Supporting Peptides:

| Peptide | Uniq | -10lgP | Mass | Length | ppm | m/z | z | RT | Fraction | Scan | Source File | Area BuCaMH03\_B17 | #Feature | #Feature BuCaMH03\_B17 | Start | End | PTM | AScore | Found By |
| --- | --- | --- | --- | --- | --- | --- | --- | --- | --- | --- | --- | --- | --- | --- | --- | --- | --- | --- | --- |
| R.IEIESFFEGEDFSETLTR.A | Y | 65.88 | 2147.9897 | 18 | 0.5 | 1075.0027 | 2 | 85.86 | 1 | 56881 | BuCaMH03.raw | 3.5489E5 | 1 | 1 | 306 | 323 |  |  | PEAKS DB |
| K.FEELNMDLFR.S | Y | 63.72 | 1312.6122 | 10 | 0.3 | 657.3135 | 2 | 71.75 | 1 | 45607 | BuCaMH03.raw | 1.0935E6 | 1 | 1 | 326 | 335 |  |  | PEAKS DB |
| K.DAGTIAGLNVMR.I | Y | 60.58 | 1216.6234 | 12 | 0.1 | 609.3190 | 2 | 54.94 | 1 | 31734 | BuCaMH03.raw | 4.5409E5 | 1 | 1 | 185 | 196 |  |  | PEAKS DB |
| total 3 peptides |  |  |  |  |  |  |  |  |  |  |  |  |  |  |  |  |  |  |  |
| --- | --- | --- | --- | --- | --- | --- | --- | --- | --- | --- | --- | --- | --- | --- | --- | --- | --- | --- | --- |

JAG44540.1

back to list

  

| Protein Coverage
| Supporting Peptides
|

Protein Coverage:

Supporting Peptides:

| Peptide | Uniq | -10lgP | Mass | Length | ppm | m/z | z | RT | Fraction | Scan | Source File | Area BuCaMH03\_B17 | #Feature | #Feature BuCaMH03\_B17 | Start | End | PTM | AScore | Found By |
| --- | --- | --- | --- | --- | --- | --- | --- | --- | --- | --- | --- | --- | --- | --- | --- | --- | --- | --- | --- |
| R.IEIESFFEGEDFSETLTR.A | Y | 65.88 | 2147.9897 | 18 | 0.5 | 1075.0027 | 2 | 85.86 | 1 | 56881 | BuCaMH03.raw | 3.5489E5 | 1 | 1 | 306 | 323 |  |  | PEAKS DB |
| K.FEELNMDLFR.S | Y | 63.72 | 1312.6122 | 10 | 0.3 | 657.3135 | 2 | 71.75 | 1 | 45607 | BuCaMH03.raw | 1.0935E6 | 1 | 1 | 326 | 335 |  |  | PEAKS DB |
| K.DAGTIAGLNVMR.I | Y | 60.58 | 1216.6234 | 12 | 0.1 | 609.3190 | 2 | 54.94 | 1 | 31734 | BuCaMH03.raw | 4.5409E5 | 1 | 1 | 185 | 196 |  |  | PEAKS DB |
| total 3 peptides |  |  |  |  |  |  |  |  |  |  |  |  |  |  |  |  |  |  |  |
| --- | --- | --- | --- | --- | --- | --- | --- | --- | --- | --- | --- | --- | --- | --- | --- | --- | --- | --- | --- |

JAI13107.1

back to list

  

| Protein Coverage
| Supporting Peptides
|

Protein Coverage:

Supporting Peptides:

| Peptide | Uniq | -10lgP | Mass | Length | ppm | m/z | z | RT | Fraction | Scan | Source File | Area BuCaMH03\_B17 | #Feature | #Feature BuCaMH03\_B17 | Start | End | PTM | AScore | Found By |
| --- | --- | --- | --- | --- | --- | --- | --- | --- | --- | --- | --- | --- | --- | --- | --- | --- | --- | --- | --- |
| R.IEIESFFEGEDFSETLTR.A | Y | 65.88 | 2147.9897 | 18 | 0.5 | 1075.0027 | 2 | 85.86 | 1 | 56881 | BuCaMH03.raw | 3.5489E5 | 1 | 1 | 306 | 323 |  |  | PEAKS DB |
| K.FEELNMDLFR.S | Y | 63.72 | 1312.6122 | 10 | 0.3 | 657.3135 | 2 | 71.75 | 1 | 45607 | BuCaMH03.raw | 1.0935E6 | 1 | 1 | 326 | 335 |  |  | PEAKS DB |
| K.DAGTIAGLNVMR.I | Y | 60.58 | 1216.6234 | 12 | 0.1 | 609.3190 | 2 | 54.94 | 1 | 31734 | BuCaMH03.raw | 4.5409E5 | 1 | 1 | 185 | 196 |  |  | PEAKS DB |
| total 3 peptides |  |  |  |  |  |  |  |  |  |  |  |  |  |  |  |  |  |  |  |
| --- | --- | --- | --- | --- | --- | --- | --- | --- | --- | --- | --- | --- | --- | --- | --- | --- | --- | --- | --- |

XP\_015681610.1

back to list

  

| Protein Coverage
| Supporting Peptides
|

Protein Coverage:

Supporting Peptides:

| Peptide | Uniq | -10lgP | Mass | Length | ppm | m/z | z | RT | Fraction | Scan | Source File | Area BuCaMH03\_B17 | #Feature | #Feature BuCaMH03\_B17 | Start | End | PTM | AScore | Found By |
| --- | --- | --- | --- | --- | --- | --- | --- | --- | --- | --- | --- | --- | --- | --- | --- | --- | --- | --- | --- |
| R.IEIESFFEGEDFSETLTR.A | Y | 65.88 | 2147.9897 | 18 | 0.5 | 1075.0027 | 2 | 85.86 | 1 | 56881 | BuCaMH03.raw | 3.5489E5 | 1 | 1 | 306 | 323 |  |  | PEAKS DB |
| K.FEELNMDLFR.S | Y | 63.72 | 1312.6122 | 10 | 0.3 | 657.3135 | 2 | 71.75 | 1 | 45607 | BuCaMH03.raw | 1.0935E6 | 1 | 1 | 326 | 335 |  |  | PEAKS DB |
| K.DAGTIAGLNVMR.I | Y | 60.58 | 1216.6234 | 12 | 0.1 | 609.3190 | 2 | 54.94 | 1 | 31734 | BuCaMH03.raw | 4.5409E5 | 1 | 1 | 185 | 196 |  |  | PEAKS DB |
| total 3 peptides |  |  |  |  |  |  |  |  |  |  |  |  |  |  |  |  |  |  |  |
| --- | --- | --- | --- | --- | --- | --- | --- | --- | --- | --- | --- | --- | --- | --- | --- | --- | --- | --- | --- |

AFJ50327.1

back to list

  

| Protein Coverage
| Supporting Peptides
|

Protein Coverage:

Supporting Peptides:

| Peptide | Uniq | -10lgP | Mass | Length | ppm | m/z | z | RT | Fraction | Scan | Source File | Area BuCaMH03\_B17 | #Feature | #Feature BuCaMH03\_B17 | Start | End | PTM | AScore | Found By |
| --- | --- | --- | --- | --- | --- | --- | --- | --- | --- | --- | --- | --- | --- | --- | --- | --- | --- | --- | --- |
| R.IEIESFFEGEDFSETLTR.A | Y | 65.88 | 2147.9897 | 18 | 0.5 | 1075.0027 | 2 | 85.86 | 1 | 56881 | BuCaMH03.raw | 3.5489E5 | 1 | 1 | 306 | 323 |  |  | PEAKS DB |
| K.FEELNMDLFR.S | Y | 63.72 | 1312.6122 | 10 | 0.3 | 657.3135 | 2 | 71.75 | 1 | 45607 | BuCaMH03.raw | 1.0935E6 | 1 | 1 | 326 | 335 |  |  | PEAKS DB |
| K.DAGTIAGLNVMR.I | Y | 60.58 | 1216.6234 | 12 | 0.1 | 609.3190 | 2 | 54.94 | 1 | 31734 | BuCaMH03.raw | 4.5409E5 | 1 | 1 | 185 | 196 |  |  | PEAKS DB |
| total 3 peptides |  |  |  |  |  |  |  |  |  |  |  |  |  |  |  |  |  |  |  |
| --- | --- | --- | --- | --- | --- | --- | --- | --- | --- | --- | --- | --- | --- | --- | --- | --- | --- | --- | --- |

JAG46692.1

back to list

  

| Protein Coverage
| Supporting Peptides
|

Protein Coverage:

Supporting Peptides:

| Peptide | Uniq | -10lgP | Mass | Length | ppm | m/z | z | RT | Fraction | Scan | Source File | Area BuCaMH03\_B17 | #Feature | #Feature BuCaMH03\_B17 | Start | End | PTM | AScore | Found By |
| --- | --- | --- | --- | --- | --- | --- | --- | --- | --- | --- | --- | --- | --- | --- | --- | --- | --- | --- | --- |
| R.IEIESFFEGEDFSETLTR.A | Y | 65.88 | 2147.9897 | 18 | 0.5 | 1075.0027 | 2 | 85.86 | 1 | 56881 | BuCaMH03.raw | 3.5489E5 | 1 | 1 | 306 | 323 |  |  | PEAKS DB |
| K.FEELNMDLFR.S | Y | 63.72 | 1312.6122 | 10 | 0.3 | 657.3135 | 2 | 71.75 | 1 | 45607 | BuCaMH03.raw | 1.0935E6 | 1 | 1 | 326 | 335 |  |  | PEAKS DB |
| K.DAGTIAGLNVMR.I | Y | 60.58 | 1216.6234 | 12 | 0.1 | 609.3190 | 2 | 54.94 | 1 | 31734 | BuCaMH03.raw | 4.5409E5 | 1 | 1 | 185 | 196 |  |  | PEAKS DB |
| total 3 peptides |  |  |  |  |  |  |  |  |  |  |  |  |  |  |  |  |  |  |  |
| --- | --- | --- | --- | --- | --- | --- | --- | --- | --- | --- | --- | --- | --- | --- | --- | --- | --- | --- | --- |

JAA96909.1

back to list

  

| Protein Coverage
| Supporting Peptides
|

Protein Coverage:

Supporting Peptides:

| Peptide | Uniq | -10lgP | Mass | Length | ppm | m/z | z | RT | Fraction | Scan | Source File | Area BuCaMH03\_B17 | #Feature | #Feature BuCaMH03\_B17 | Start | End | PTM | AScore | Found By |
| --- | --- | --- | --- | --- | --- | --- | --- | --- | --- | --- | --- | --- | --- | --- | --- | --- | --- | --- | --- |
| R.IEIESFFEGEDFSETLTR.A | Y | 65.88 | 2147.9897 | 18 | 0.5 | 1075.0027 | 2 | 85.86 | 1 | 56881 | BuCaMH03.raw | 3.5489E5 | 1 | 1 | 306 | 323 |  |  | PEAKS DB |
| K.FEELNMDLFR.S | Y | 63.72 | 1312.6122 | 10 | 0.3 | 657.3135 | 2 | 71.75 | 1 | 45607 | BuCaMH03.raw | 1.0935E6 | 1 | 1 | 326 | 335 |  |  | PEAKS DB |
| K.DAGTIAGLNVMR.I | Y | 60.58 | 1216.6234 | 12 | 0.1 | 609.3190 | 2 | 54.94 | 1 | 31734 | BuCaMH03.raw | 4.5409E5 | 1 | 1 | 185 | 196 |  |  | PEAKS DB |
| total 3 peptides |  |  |  |  |  |  |  |  |  |  |  |  |  |  |  |  |  |  |  |
| --- | --- | --- | --- | --- | --- | --- | --- | --- | --- | --- | --- | --- | --- | --- | --- | --- | --- | --- | --- |

AFJ50239.1

back to list

  

| Protein Coverage
| Supporting Peptides
|

Protein Coverage:

Supporting Peptides:

| Peptide | Uniq | -10lgP | Mass | Length | ppm | m/z | z | RT | Fraction | Scan | Source File | Area BuCaMH03\_B17 | #Feature | #Feature BuCaMH03\_B17 | Start | End | PTM | AScore | Found By |
| --- | --- | --- | --- | --- | --- | --- | --- | --- | --- | --- | --- | --- | --- | --- | --- | --- | --- | --- | --- |
| R.IEIESFFEGEDFSETLTR.A | Y | 65.88 | 2147.9897 | 18 | 0.5 | 1075.0027 | 2 | 85.86 | 1 | 56881 | BuCaMH03.raw | 3.5489E5 | 1 | 1 | 306 | 323 |  |  | PEAKS DB |
| K.FEELNMDLFR.S | Y | 63.72 | 1312.6122 | 10 | 0.3 | 657.3135 | 2 | 71.75 | 1 | 45607 | BuCaMH03.raw | 1.0935E6 | 1 | 1 | 326 | 335 |  |  | PEAKS DB |
| K.DAGTIAGLNVMR.I | Y | 60.58 | 1216.6234 | 12 | 0.1 | 609.3190 | 2 | 54.94 | 1 | 31734 | BuCaMH03.raw | 4.5409E5 | 1 | 1 | 185 | 196 |  |  | PEAKS DB |
| total 3 peptides |  |  |  |  |  |  |  |  |  |  |  |  |  |  |  |  |  |  |  |
| --- | --- | --- | --- | --- | --- | --- | --- | --- | --- | --- | --- | --- | --- | --- | --- | --- | --- | --- | --- |

ABH05181.1

back to list

  

| Protein Coverage
| Supporting Peptides
|

Protein Coverage:

Supporting Peptides:

| Peptide | Uniq | -10lgP | Mass | Length | ppm | m/z | z | RT | Fraction | Scan | Source File | Area BuCaMH03\_B17 | #Feature | #Feature BuCaMH03\_B17 | Start | End | PTM | AScore | Found By |
| --- | --- | --- | --- | --- | --- | --- | --- | --- | --- | --- | --- | --- | --- | --- | --- | --- | --- | --- | --- |
| R.DADSADLAEYISDYLK.V | Y | 64.89 | 1787.8101 | 16 | 0.7 | 894.9130 | 2 | 92.01 | 1 | 61834 | BuCaMH03.raw | 1.5378E6 | 1 | 1 | 69 | 84 |  |  | PEAKS DB |
| total 1 peptides |  |  |  |  |  |  |  |  |  |  |  |  |  |  |  |  |  |  |  |
| --- | --- | --- | --- | --- | --- | --- | --- | --- | --- | --- | --- | --- | --- | --- | --- | --- | --- | --- | --- |

A1XXJ9.1

back to list

  

| Protein Coverage
| Supporting Peptides
|

Protein Coverage:

Supporting Peptides:

| Peptide | Uniq | -10lgP | Mass | Length | ppm | m/z | z | RT | Fraction | Scan | Source File | Area BuCaMH03\_B17 | #Feature | #Feature BuCaMH03\_B17 | Start | End | PTM | AScore | Found By |
| --- | --- | --- | --- | --- | --- | --- | --- | --- | --- | --- | --- | --- | --- | --- | --- | --- | --- | --- | --- |
| R.DADSADLAEYISDYLK.V | Y | 64.89 | 1787.8101 | 16 | 0.7 | 894.9130 | 2 | 92.01 | 1 | 61834 | BuCaMH03.raw | 1.5378E6 | 1 | 1 | 69 | 84 |  |  | PEAKS DB |
| total 1 peptides |  |  |  |  |  |  |  |  |  |  |  |  |  |  |  |  |  |  |  |
| --- | --- | --- | --- | --- | --- | --- | --- | --- | --- | --- | --- | --- | --- | --- | --- | --- | --- | --- | --- |

ABU68503.1

back to list

  

| Protein Coverage
| Supporting Peptides
|

Protein Coverage:

Supporting Peptides:

| Peptide | Uniq | -10lgP | Mass | Length | ppm | m/z | z | RT | Fraction | Scan | Source File | Area BuCaMH03\_B17 | #Feature | #Feature BuCaMH03\_B17 | Start | End | PTM | AScore | Found By |
| --- | --- | --- | --- | --- | --- | --- | --- | --- | --- | --- | --- | --- | --- | --- | --- | --- | --- | --- | --- |
| V.AAIC(+57.02)FAR.A | N | 64.40 | 807.4061 | 7 | 0.3 | 404.7105 | 2 | 30.53 | 1 | 12209 | BuCaMH03.raw | 2.029E7 | 1 | 1 | 129 | 135 | Carbamidomethylation | C4:Carbamidomethylation:1000.00 | PEAKS DB |
| R.VAAIC(+57.02)FAR.A | Y | 62.11 | 906.4745 | 8 | 2.3 | 454.2456 | 2 | 38.73 | 1 | 18474 | BuCaMH03.raw | 8.6156E5 | 1 | 1 | 128 | 135 | Carbamidomethylation | C5:Carbamidomethylation:1000.00 | PEAKS DB |
| C.YC(+57.02)GYGGSGTPVDELDR.C | N | 61.33 | 1744.7362 | 16 | 0.7 | 873.3760 | 2 | 48.17 | 1 | 25865 | BuCaMH03.raw | 3.7552E6 | 1 | 1 | 55 | 70 | Carbamidomethylation | C2:Carbamidomethylation:1000.00 | PEAKS DB |
| total 3 peptides |  |  |  |  |  |  |  |  |  |  |  |  |  |  |  |  |  |  |  |
| --- | --- | --- | --- | --- | --- | --- | --- | --- | --- | --- | --- | --- | --- | --- | --- | --- | --- | --- | --- |

T\_84

back to list

  

| Protein Coverage
| Supporting Peptides
|

Protein Coverage:

Supporting Peptides:

| Peptide | Uniq | -10lgP | Mass | Length | ppm | m/z | z | RT | Fraction | Scan | Source File | Area BuCaMH03\_B17 | #Feature | #Feature BuCaMH03\_B17 | Start | End | PTM | AScore | Found By |
| --- | --- | --- | --- | --- | --- | --- | --- | --- | --- | --- | --- | --- | --- | --- | --- | --- | --- | --- | --- |
| K.ADVTFDSNTAFESLVVSPDKK.T | Y | 68.94 | 2269.1113 | 21 | 1.1 | 757.3785 | 3 | 63.88 | 1 | 39147 | BuCaMH03.raw | 6.4817E5 | 1 | 1 | 57 | 77 |  |  | PEAKS DB |
| K.IVVFLDYNEEK.V | Y | 64.72 | 1367.6973 | 11 | 1.0 | 684.8566 | 2 | 59.94 | 1 | 35768 | BuCaMH03.raw | 9.7449E5 | 1 | 1 | 172 | 182 |  |  | PEAKS DB |
| total 2 peptides |  |  |  |  |  |  |  |  |  |  |  |  |  |  |  |  |  |  |  |
| --- | --- | --- | --- | --- | --- | --- | --- | --- | --- | --- | --- | --- | --- | --- | --- | --- | --- | --- | --- |

CAM11302.1

back to list

  

| Protein Coverage
| Supporting Peptides
|

Protein Coverage:

Supporting Peptides:

| Peptide | Uniq | -10lgP | Mass | Length | ppm | m/z | z | RT | Fraction | Scan | Source File | Area BuCaMH03\_B17 | #Feature | #Feature BuCaMH03\_B17 | Start | End | PTM | AScore | Found By |
| --- | --- | --- | --- | --- | --- | --- | --- | --- | --- | --- | --- | --- | --- | --- | --- | --- | --- | --- | --- |
| T.C(+57.02)PSGENLC(+57.02)YTK.M | Y | 68.81 | 1327.5537 | 11 | 0.1 | 664.7842 | 2 | 21.67 | 1 | 6555 | BuCaMH03.raw | 3.4853E6 | 1 | 1 | 18 | 28 | Carbamidomethylation | C1:Carbamidomethylation:1000.00;C8:Carbamidomethylation:1000.00 | PEAKS DB |
| T.VTC(+57.02)PSGENLC(+57.02)YTK.M | Y | 64.79 | 1527.6698 | 13 | 0.0 | 764.8422 | 2 | 28.21 | 1 | 10563 | BuCaMH03.raw | 3.8765E7 | 1 | 1 | 16 | 28 | Carbamidomethylation | C3:Carbamidomethylation:1000.00;C10:Carbamidomethylation:1000.00 | PEAKS DB |
| total 2 peptides |  |  |  |  |  |  |  |  |  |  |  |  |  |  |  |  |  |  |  |
| --- | --- | --- | --- | --- | --- | --- | --- | --- | --- | --- | --- | --- | --- | --- | --- | --- | --- | --- | --- |

D2N116.1

back to list

  

| Protein Coverage
| Supporting Peptides
|

Protein Coverage:

Supporting Peptides:

| Peptide | Uniq | -10lgP | Mass | Length | ppm | m/z | z | RT | Fraction | Scan | Source File | Area BuCaMH03\_B17 | #Feature | #Feature BuCaMH03\_B17 | Start | End | PTM | AScore | Found By |
| --- | --- | --- | --- | --- | --- | --- | --- | --- | --- | --- | --- | --- | --- | --- | --- | --- | --- | --- | --- |
| T.C(+57.02)PSGENLC(+57.02)YTK.M | Y | 68.81 | 1327.5537 | 11 | 0.1 | 664.7842 | 2 | 21.67 | 1 | 6555 | BuCaMH03.raw | 3.4853E6 | 1 | 1 | 18 | 28 | Carbamidomethylation | C1:Carbamidomethylation:1000.00;C8:Carbamidomethylation:1000.00 | PEAKS DB |
| T.VTC(+57.02)PSGENLC(+57.02)YTK.M | Y | 64.79 | 1527.6698 | 13 | 0.0 | 764.8422 | 2 | 28.21 | 1 | 10563 | BuCaMH03.raw | 3.8765E7 | 1 | 1 | 16 | 28 | Carbamidomethylation | C3:Carbamidomethylation:1000.00;C10:Carbamidomethylation:1000.00 | PEAKS DB |
| total 2 peptides |  |  |  |  |  |  |  |  |  |  |  |  |  |  |  |  |  |  |  |
| --- | --- | --- | --- | --- | --- | --- | --- | --- | --- | --- | --- | --- | --- | --- | --- | --- | --- | --- | --- |

ACY68722.1

back to list

  

| Protein Coverage
| Supporting Peptides
|

Protein Coverage:

Supporting Peptides:

| Peptide | Uniq | -10lgP | Mass | Length | ppm | m/z | z | RT | Fraction | Scan | Source File | Area BuCaMH03\_B17 | #Feature | #Feature BuCaMH03\_B17 | Start | End | PTM | AScore | Found By |
| --- | --- | --- | --- | --- | --- | --- | --- | --- | --- | --- | --- | --- | --- | --- | --- | --- | --- | --- | --- |
| K.YLYVC(+57.02)QYC(+57.02)PAGNIKR.L | Y | 70.72 | 1903.9072 | 15 | -0.3 | 635.6428 | 3 | 38.93 | 1 | 18668 | BuCaMH03.raw | 9.8406E5 | 1 | 1 | 158 | 172 | Carbamidomethylation | C5:Carbamidomethylation:1000.00;C8:Carbamidomethylation:1000.00 | PEAKS DB |
| total 1 peptides |  |  |  |  |  |  |  |  |  |  |  |  |  |  |  |  |  |  |  |
| --- | --- | --- | --- | --- | --- | --- | --- | --- | --- | --- | --- | --- | --- | --- | --- | --- | --- | --- | --- |

AAB25729.1

back to list

  

| Protein Coverage
| Supporting Peptides
|

Protein Coverage:

Supporting Peptides:

| Peptide | Uniq | -10lgP | Mass | Length | ppm | m/z | z | RT | Fraction | Scan | Source File | Area BuCaMH03\_B17 | #Feature | #Feature BuCaMH03\_B17 | Start | End | PTM | AScore | Found By |
| --- | --- | --- | --- | --- | --- | --- | --- | --- | --- | --- | --- | --- | --- | --- | --- | --- | --- | --- | --- |
| K.QYFFETK.C | Y | 53.56 | 961.4545 | 7 | 0.9 | 481.7349 | 2 | 41.53 | 1 | 20492 | BuCaMH03.raw | 7.1214E6 | 1 | 1 | 175 | 181 |  |  | PEAKS DB |
| total 1 peptides |  |  |  |  |  |  |  |  |  |  |  |  |  |  |  |  |  |  |  |
| --- | --- | --- | --- | --- | --- | --- | --- | --- | --- | --- | --- | --- | --- | --- | --- | --- | --- | --- | --- |

P34128.1

back to list

  

| Protein Coverage
| Supporting Peptides
|

Protein Coverage:

Supporting Peptides:

| Peptide | Uniq | -10lgP | Mass | Length | ppm | m/z | z | RT | Fraction | Scan | Source File | Area BuCaMH03\_B17 | #Feature | #Feature BuCaMH03\_B17 | Start | End | PTM | AScore | Found By |
| --- | --- | --- | --- | --- | --- | --- | --- | --- | --- | --- | --- | --- | --- | --- | --- | --- | --- | --- | --- |
| K.QYFFETK.C | Y | 53.56 | 961.4545 | 7 | 0.9 | 481.7349 | 2 | 41.53 | 1 | 20492 | BuCaMH03.raw | 7.1214E6 | 1 | 1 | 175 | 181 |  |  | PEAKS DB |
| total 1 peptides |  |  |  |  |  |  |  |  |  |  |  |  |  |  |  |  |  |  |  |
| --- | --- | --- | --- | --- | --- | --- | --- | --- | --- | --- | --- | --- | --- | --- | --- | --- | --- | --- | --- |

B4ESA3.1

back to list

  

| Protein Coverage
| Supporting Peptides
|

Protein Coverage:

Supporting Peptides:

| Peptide | Uniq | -10lgP | Mass | Length | ppm | m/z | z | RT | Fraction | Scan | Source File | Area BuCaMH03\_B17 | #Feature | #Feature BuCaMH03\_B17 | Start | End | PTM | AScore | Found By |
| --- | --- | --- | --- | --- | --- | --- | --- | --- | --- | --- | --- | --- | --- | --- | --- | --- | --- | --- | --- |
| P.YGGC(+57.02)GGNANNFK.T | N | 75.97 | 1257.5197 | 12 | 0.4 | 629.7673 | 2 | 13.72 | 1 | 2615 | BuCaMH03.raw | 8.8737E6 | 1 | 1 | 61 | 72 | Carbamidomethylation | C4:Carbamidomethylation:1000.00 | PEAKS DB |
| R.AFYYNSR.P | Y | 56.83 | 919.4188 | 7 | -1.0 | 460.7162 | 2 | 24.64 | 1 | 8266 | BuCaMH03.raw | 6.4804E7 | 1 | 1 | 46 | 52 |  |  | PEAKS DB |
| total 2 peptides |  |  |  |  |  |  |  |  |  |  |  |  |  |  |  |  |  |  |  |
| --- | --- | --- | --- | --- | --- | --- | --- | --- | --- | --- | --- | --- | --- | --- | --- | --- | --- | --- | --- |

Q8AY41.2

back to list

  

| Protein Coverage
| Supporting Peptides
|

Protein Coverage:

Supporting Peptides:

| Peptide | Uniq | -10lgP | Mass | Length | ppm | m/z | z | RT | Fraction | Scan | Source File | Area BuCaMH03\_B17 | #Feature | #Feature BuCaMH03\_B17 | Start | End | PTM | AScore | Found By |
| --- | --- | --- | --- | --- | --- | --- | --- | --- | --- | --- | --- | --- | --- | --- | --- | --- | --- | --- | --- |
| P.YGGC(+57.02)GGNANNFK.T | N | 75.97 | 1257.5197 | 12 | 0.4 | 629.7673 | 2 | 13.72 | 1 | 2615 | BuCaMH03.raw | 8.8737E6 | 1 | 1 | 61 | 72 | Carbamidomethylation | C4:Carbamidomethylation:1000.00 | PEAKS DB |
| R.AFYYNSR.L | Y | 56.83 | 919.4188 | 7 | -1.0 | 460.7162 | 2 | 24.64 | 1 | 8266 | BuCaMH03.raw | 6.4804E7 | 1 | 1 | 46 | 52 |  |  | PEAKS DB |
| total 2 peptides |  |  |  |  |  |  |  |  |  |  |  |  |  |  |  |  |  |  |  |
| --- | --- | --- | --- | --- | --- | --- | --- | --- | --- | --- | --- | --- | --- | --- | --- | --- | --- | --- | --- |

CAP74382.1

back to list

  

| Protein Coverage
| Supporting Peptides
|

Protein Coverage:

Supporting Peptides:

| Peptide | Uniq | -10lgP | Mass | Length | ppm | m/z | z | RT | Fraction | Scan | Source File | Area BuCaMH03\_B17 | #Feature | #Feature BuCaMH03\_B17 | Start | End | PTM | AScore | Found By |
| --- | --- | --- | --- | --- | --- | --- | --- | --- | --- | --- | --- | --- | --- | --- | --- | --- | --- | --- | --- |
| P.YGGC(+57.02)GGNANNFK.T | N | 75.97 | 1257.5197 | 12 | 0.4 | 629.7673 | 2 | 13.72 | 1 | 2615 | BuCaMH03.raw | 8.8737E6 | 1 | 1 | 61 | 72 | Carbamidomethylation | C4:Carbamidomethylation:1000.00 | PEAKS DB |
| R.AFYYNSR.P | Y | 56.83 | 919.4188 | 7 | -1.0 | 460.7162 | 2 | 24.64 | 1 | 8266 | BuCaMH03.raw | 6.4804E7 | 1 | 1 | 46 | 52 |  |  | PEAKS DB |
| total 2 peptides |  |  |  |  |  |  |  |  |  |  |  |  |  |  |  |  |  |  |  |
| --- | --- | --- | --- | --- | --- | --- | --- | --- | --- | --- | --- | --- | --- | --- | --- | --- | --- | --- | --- |

AAL30070.1

back to list

  

| Protein Coverage
| Supporting Peptides
|

Protein Coverage:

Supporting Peptides:

| Peptide | Uniq | -10lgP | Mass | Length | ppm | m/z | z | RT | Fraction | Scan | Source File | Area BuCaMH03\_B17 | #Feature | #Feature BuCaMH03\_B17 | Start | End | PTM | AScore | Found By |
| --- | --- | --- | --- | --- | --- | --- | --- | --- | --- | --- | --- | --- | --- | --- | --- | --- | --- | --- | --- |
| P.YGGC(+57.02)GGNANNFK.T | N | 75.97 | 1257.5197 | 12 | 0.4 | 629.7673 | 2 | 13.72 | 1 | 2615 | BuCaMH03.raw | 8.8737E6 | 1 | 1 | 66 | 77 | Carbamidomethylation | C4:Carbamidomethylation:1000.00 | PEAKS DB |
| R.AFYYNSR.L | Y | 56.83 | 919.4188 | 7 | -1.0 | 460.7162 | 2 | 24.64 | 1 | 8266 | BuCaMH03.raw | 6.4804E7 | 1 | 1 | 51 | 57 |  |  | PEAKS DB |
| total 2 peptides |  |  |  |  |  |  |  |  |  |  |  |  |  |  |  |  |  |  |  |
| --- | --- | --- | --- | --- | --- | --- | --- | --- | --- | --- | --- | --- | --- | --- | --- | --- | --- | --- | --- |

JAA74768.1

back to list

  

| Protein Coverage
| Supporting Peptides
|

Protein Coverage:

Supporting Peptides:

| Peptide | Uniq | -10lgP | Mass | Length | ppm | m/z | z | RT | Fraction | Scan | Source File | Area BuCaMH03\_B17 | #Feature | #Feature BuCaMH03\_B17 | Start | End | PTM | AScore | Found By |
| --- | --- | --- | --- | --- | --- | --- | --- | --- | --- | --- | --- | --- | --- | --- | --- | --- | --- | --- | --- |
| K.TWC(+57.02)DAWC(+57.02)TSR.G | Y | 62.45 | 1341.5231 | 10 | 0.5 | 671.7692 | 2 | 45.01 | 1 | 23310 | BuCaMH03.raw | 9.3226E6 | 1 | 1 | 46 | 55 | Carbamidomethylation | C3:Carbamidomethylation:1000.00;C7:Carbamidomethylation:1000.00 | PEAKS DB |
| total 1 peptides |  |  |  |  |  |  |  |  |  |  |  |  |  |  |  |  |  |  |  |
| --- | --- | --- | --- | --- | --- | --- | --- | --- | --- | --- | --- | --- | --- | --- | --- | --- | --- | --- | --- |

JAA74767.1

back to list

  

| Protein Coverage
| Supporting Peptides
|

Protein Coverage:

Supporting Peptides:

| Peptide | Uniq | -10lgP | Mass | Length | ppm | m/z | z | RT | Fraction | Scan | Source File | Area BuCaMH03\_B17 | #Feature | #Feature BuCaMH03\_B17 | Start | End | PTM | AScore | Found By |
| --- | --- | --- | --- | --- | --- | --- | --- | --- | --- | --- | --- | --- | --- | --- | --- | --- | --- | --- | --- |
| K.TWC(+57.02)DAWC(+57.02)TSR.G | Y | 62.45 | 1341.5231 | 10 | 0.5 | 671.7692 | 2 | 45.01 | 1 | 23310 | BuCaMH03.raw | 9.3226E6 | 1 | 1 | 46 | 55 | Carbamidomethylation | C3:Carbamidomethylation:1000.00;C7:Carbamidomethylation:1000.00 | PEAKS DB |
| total 1 peptides |  |  |  |  |  |  |  |  |  |  |  |  |  |  |  |  |  |  |  |
| --- | --- | --- | --- | --- | --- | --- | --- | --- | --- | --- | --- | --- | --- | --- | --- | --- | --- | --- | --- |

P01387.1

back to list

  

| Protein Coverage
| Supporting Peptides
|

Protein Coverage:

Supporting Peptides:

| Peptide | Uniq | -10lgP | Mass | Length | ppm | m/z | z | RT | Fraction | Scan | Source File | Area BuCaMH03\_B17 | #Feature | #Feature BuCaMH03\_B17 | Start | End | PTM | AScore | Found By |
| --- | --- | --- | --- | --- | --- | --- | --- | --- | --- | --- | --- | --- | --- | --- | --- | --- | --- | --- | --- |
| E.TWC(+57.02)DAWC(+57.02)TSR.G | Y | 62.45 | 1341.5231 | 10 | 0.5 | 671.7692 | 2 | 45.01 | 1 | 23310 | BuCaMH03.raw | 9.3226E6 | 1 | 1 | 25 | 34 | Carbamidomethylation | C3:Carbamidomethylation:1000.00;C7:Carbamidomethylation:1000.00 | PEAKS DB |
| total 1 peptides |  |  |  |  |  |  |  |  |  |  |  |  |  |  |  |  |  |  |  |
| --- | --- | --- | --- | --- | --- | --- | --- | --- | --- | --- | --- | --- | --- | --- | --- | --- | --- | --- | --- |

Q53B58.1

back to list

  

| Protein Coverage
| Supporting Peptides
|

Protein Coverage:

Supporting Peptides:

| Peptide | Uniq | -10lgP | Mass | Length | ppm | m/z | z | RT | Fraction | Scan | Source File | Area BuCaMH03\_B17 | #Feature | #Feature BuCaMH03\_B17 | Start | End | PTM | AScore | Found By |
| --- | --- | --- | --- | --- | --- | --- | --- | --- | --- | --- | --- | --- | --- | --- | --- | --- | --- | --- | --- |
| E.TWC(+57.02)DAWC(+57.02)TSR.G | Y | 62.45 | 1341.5231 | 10 | 0.5 | 671.7692 | 2 | 45.01 | 1 | 23310 | BuCaMH03.raw | 9.3226E6 | 1 | 1 | 46 | 55 | Carbamidomethylation | C3:Carbamidomethylation:1000.00;C7:Carbamidomethylation:1000.00 | PEAKS DB |
| total 1 peptides |  |  |  |  |  |  |  |  |  |  |  |  |  |  |  |  |  |  |  |
| --- | --- | --- | --- | --- | --- | --- | --- | --- | --- | --- | --- | --- | --- | --- | --- | --- | --- | --- | --- |

AAT97250.1

back to list

  

| Protein Coverage
| Supporting Peptides
|

Protein Coverage:

Supporting Peptides:

| Peptide | Uniq | -10lgP | Mass | Length | ppm | m/z | z | RT | Fraction | Scan | Source File | Area BuCaMH03\_B17 | #Feature | #Feature BuCaMH03\_B17 | Start | End | PTM | AScore | Found By |
| --- | --- | --- | --- | --- | --- | --- | --- | --- | --- | --- | --- | --- | --- | --- | --- | --- | --- | --- | --- |
| E.TWC(+57.02)DAWC(+57.02)TSR.G | Y | 62.45 | 1341.5231 | 10 | 0.5 | 671.7692 | 2 | 45.01 | 1 | 23310 | BuCaMH03.raw | 9.3226E6 | 1 | 1 | 46 | 55 | Carbamidomethylation | C3:Carbamidomethylation:1000.00;C7:Carbamidomethylation:1000.00 | PEAKS DB |
| total 1 peptides |  |  |  |  |  |  |  |  |  |  |  |  |  |  |  |  |  |  |  |
| --- | --- | --- | --- | --- | --- | --- | --- | --- | --- | --- | --- | --- | --- | --- | --- | --- | --- | --- | --- |

BAC77656.1

back to list

  

| Protein Coverage
| Supporting Peptides
|

Protein Coverage:

Supporting Peptides:

| Peptide | Uniq | -10lgP | Mass | Length | ppm | m/z | z | RT | Fraction | Scan | Source File | Area BuCaMH03\_B17 | #Feature | #Feature BuCaMH03\_B17 | Start | End | PTM | AScore | Found By |
| --- | --- | --- | --- | --- | --- | --- | --- | --- | --- | --- | --- | --- | --- | --- | --- | --- | --- | --- | --- |
| R.VAAIC(+57.02)FAGAPYNDKNFM(+15.99)INT.E | Y | 59.60 | 2232.0344 | 20 | -0.7 | 1117.0237 | 2 | 68.70 | 1 | 44155 | BuCaMH03.raw | 1.0036E8 | 2 | 2 | 123 | 142 | Carbamidomethylation; Oxidation (M) | C5:Carbamidomethylation:1000.00;M17:Oxidation (M):1000.00 | PEAKS DB |
| total 1 peptides |  |  |  |  |  |  |  |  |  |  |  |  |  |  |  |  |  |  |  |
| --- | --- | --- | --- | --- | --- | --- | --- | --- | --- | --- | --- | --- | --- | --- | --- | --- | --- | --- | --- |

ADF50035.1

back to list

  

| Protein Coverage
| Supporting Peptides
|

Protein Coverage:

Supporting Peptides:

| Peptide | Uniq | -10lgP | Mass | Length | ppm | m/z | z | RT | Fraction | Scan | Source File | Area BuCaMH03\_B17 | #Feature | #Feature BuCaMH03\_B17 | Start | End | PTM | AScore | Found By |
| --- | --- | --- | --- | --- | --- | --- | --- | --- | --- | --- | --- | --- | --- | --- | --- | --- | --- | --- | --- |
| R.VAAIC(+57.02)FAGAPYNDKNFM(+15.99)INT.E | Y | 59.60 | 2232.0344 | 20 | -0.7 | 1117.0237 | 2 | 68.70 | 1 | 44155 | BuCaMH03.raw | 1.0036E8 | 2 | 2 | 123 | 142 | Carbamidomethylation; Oxidation (M) | C5:Carbamidomethylation:1000.00;M17:Oxidation (M):1000.00 | PEAKS DB |
| total 1 peptides |  |  |  |  |  |  |  |  |  |  |  |  |  |  |  |  |  |  |  |
| --- | --- | --- | --- | --- | --- | --- | --- | --- | --- | --- | --- | --- | --- | --- | --- | --- | --- | --- | --- |

Q7T2Q4.1

back to list

  

| Protein Coverage
| Supporting Peptides
|

Protein Coverage:

Supporting Peptides:

| Peptide | Uniq | -10lgP | Mass | Length | ppm | m/z | z | RT | Fraction | Scan | Source File | Area BuCaMH03\_B17 | #Feature | #Feature BuCaMH03\_B17 | Start | End | PTM | AScore | Found By |
| --- | --- | --- | --- | --- | --- | --- | --- | --- | --- | --- | --- | --- | --- | --- | --- | --- | --- | --- | --- |
| R.VAAIC(+57.02)FAGAPYNDKNFM(+15.99)INT.E | Y | 59.60 | 2232.0344 | 20 | -0.7 | 1117.0237 | 2 | 68.70 | 1 | 44155 | BuCaMH03.raw | 1.0036E8 | 2 | 2 | 123 | 142 | Carbamidomethylation; Oxidation (M) | C5:Carbamidomethylation:1000.00;M17:Oxidation (M):1000.00 | PEAKS DB |
| total 1 peptides |  |  |  |  |  |  |  |  |  |  |  |  |  |  |  |  |  |  |  |
| --- | --- | --- | --- | --- | --- | --- | --- | --- | --- | --- | --- | --- | --- | --- | --- | --- | --- | --- | --- |

ACY68711.1

back to list

  

| Protein Coverage
| Supporting Peptides
|

Protein Coverage:

Supporting Peptides:

| Peptide | Uniq | -10lgP | Mass | Length | ppm | m/z | z | RT | Fraction | Scan | Source File | Area BuCaMH03\_B17 | #Feature | #Feature BuCaMH03\_B17 | Start | End | PTM | AScore | Found By |
| --- | --- | --- | --- | --- | --- | --- | --- | --- | --- | --- | --- | --- | --- | --- | --- | --- | --- | --- | --- |
| L.NLIQFSYLIQC(+57.02)ANHGR.R | Y | 61.51 | 1932.9628 | 16 | 0.3 | 645.3284 | 3 | 66.34 | 1 | 41154 | BuCaMH03.raw | 2.4189E6 | 1 | 1 | 28 | 43 | Carbamidomethylation | C11:Carbamidomethylation:1000.00 | PEAKS DB |
| total 1 peptides |  |  |  |  |  |  |  |  |  |  |  |  |  |  |  |  |  |  |  |
| --- | --- | --- | --- | --- | --- | --- | --- | --- | --- | --- | --- | --- | --- | --- | --- | --- | --- | --- | --- |

JAC94986.1

back to list

  

| Protein Coverage
| Supporting Peptides
|

Protein Coverage:

Supporting Peptides:

| Peptide | Uniq | -10lgP | Mass | Length | ppm | m/z | z | RT | Fraction | Scan | Source File | Area BuCaMH03\_B17 | #Feature | #Feature BuCaMH03\_B17 | Start | End | PTM | AScore | Found By |
| --- | --- | --- | --- | --- | --- | --- | --- | --- | --- | --- | --- | --- | --- | --- | --- | --- | --- | --- | --- |
| K.DGSILEVNVTPC(+57.02)PTLPC(+57.02)ILHK.G | Y | 82.18 | 2362.2024 | 21 | 0.9 | 788.4088 | 3 | 74.40 | 1 | 47822 | BuCaMH03.raw | 2.3001E6 | 1 | 1 | 35 | 55 | Carbamidomethylation | C12:Carbamidomethylation:1000.00;C17:Carbamidomethylation:1000.00 | PEAKS DB |
| total 1 peptides |  |  |  |  |  |  |  |  |  |  |  |  |  |  |  |  |  |  |  |
| --- | --- | --- | --- | --- | --- | --- | --- | --- | --- | --- | --- | --- | --- | --- | --- | --- | --- | --- | --- |

JAA97769.1

back to list

  

| Protein Coverage
| Supporting Peptides
|

Protein Coverage:

Supporting Peptides:

| Peptide | Uniq | -10lgP | Mass | Length | ppm | m/z | z | RT | Fraction | Scan | Source File | Area BuCaMH03\_B17 | #Feature | #Feature BuCaMH03\_B17 | Start | End | PTM | AScore | Found By |
| --- | --- | --- | --- | --- | --- | --- | --- | --- | --- | --- | --- | --- | --- | --- | --- | --- | --- | --- | --- |
| K.SALSGHLEAVILGLLK.T | Y | 81.97 | 1619.9609 | 16 | 0.2 | 540.9944 | 3 | 86.74 | 1 | 57625 | BuCaMH03.raw | 6.5848E5 | 1 | 1 | 89 | 104 |  |  | PEAKS DB |
| total 1 peptides |  |  |  |  |  |  |  |  |  |  |  |  |  |  |  |  |  |  |  |
| --- | --- | --- | --- | --- | --- | --- | --- | --- | --- | --- | --- | --- | --- | --- | --- | --- | --- | --- | --- |

JAI10286.1

back to list

  

| Protein Coverage
| Supporting Peptides
|

Protein Coverage:

Supporting Peptides:

| Peptide | Uniq | -10lgP | Mass | Length | ppm | m/z | z | RT | Fraction | Scan | Source File | Area BuCaMH03\_B17 | #Feature | #Feature BuCaMH03\_B17 | Start | End | PTM | AScore | Found By |
| --- | --- | --- | --- | --- | --- | --- | --- | --- | --- | --- | --- | --- | --- | --- | --- | --- | --- | --- | --- |
| K.SALSGHLEAVILGLLK.T | Y | 81.97 | 1619.9609 | 16 | 0.2 | 540.9944 | 3 | 86.74 | 1 | 57625 | BuCaMH03.raw | 6.5848E5 | 1 | 1 | 89 | 104 |  |  | PEAKS DB |
| total 1 peptides |  |  |  |  |  |  |  |  |  |  |  |  |  |  |  |  |  |  |  |
| --- | --- | --- | --- | --- | --- | --- | --- | --- | --- | --- | --- | --- | --- | --- | --- | --- | --- | --- | --- |

JAG47471.1

back to list

  

| Protein Coverage
| Supporting Peptides
|

Protein Coverage:

Supporting Peptides:

| Peptide | Uniq | -10lgP | Mass | Length | ppm | m/z | z | RT | Fraction | Scan | Source File | Area BuCaMH03\_B17 | #Feature | #Feature BuCaMH03\_B17 | Start | End | PTM | AScore | Found By |
| --- | --- | --- | --- | --- | --- | --- | --- | --- | --- | --- | --- | --- | --- | --- | --- | --- | --- | --- | --- |
| K.SALSGHLEAVILGLLK.T | Y | 81.97 | 1619.9609 | 16 | 0.2 | 540.9944 | 3 | 86.74 | 1 | 57625 | BuCaMH03.raw | 6.5848E5 | 1 | 1 | 89 | 104 |  |  | PEAKS DB |
| total 1 peptides |  |  |  |  |  |  |  |  |  |  |  |  |  |  |  |  |  |  |  |
| --- | --- | --- | --- | --- | --- | --- | --- | --- | --- | --- | --- | --- | --- | --- | --- | --- | --- | --- | --- |

JAB54690.1

back to list

  

| Protein Coverage
| Supporting Peptides
|

Protein Coverage:

Supporting Peptides:

| Peptide | Uniq | -10lgP | Mass | Length | ppm | m/z | z | RT | Fraction | Scan | Source File | Area BuCaMH03\_B17 | #Feature | #Feature BuCaMH03\_B17 | Start | End | PTM | AScore | Found By |
| --- | --- | --- | --- | --- | --- | --- | --- | --- | --- | --- | --- | --- | --- | --- | --- | --- | --- | --- | --- |
| K.SALSGHLEAVILGLLK.T | Y | 81.97 | 1619.9609 | 16 | 0.2 | 540.9944 | 3 | 86.74 | 1 | 57625 | BuCaMH03.raw | 6.5848E5 | 1 | 1 | 89 | 104 |  |  | PEAKS DB |
| total 1 peptides |  |  |  |  |  |  |  |  |  |  |  |  |  |  |  |  |  |  |  |
| --- | --- | --- | --- | --- | --- | --- | --- | --- | --- | --- | --- | --- | --- | --- | --- | --- | --- | --- | --- |

JAG45828.1

back to list

  

| Protein Coverage
| Supporting Peptides
|

Protein Coverage:

Supporting Peptides:

| Peptide | Uniq | -10lgP | Mass | Length | ppm | m/z | z | RT | Fraction | Scan | Source File | Area BuCaMH03\_B17 | #Feature | #Feature BuCaMH03\_B17 | Start | End | PTM | AScore | Found By |
| --- | --- | --- | --- | --- | --- | --- | --- | --- | --- | --- | --- | --- | --- | --- | --- | --- | --- | --- | --- |
| K.SALSGHLEAVILGLLK.T | Y | 81.97 | 1619.9609 | 16 | 0.2 | 540.9944 | 3 | 86.74 | 1 | 57625 | BuCaMH03.raw | 6.5848E5 | 1 | 1 | 89 | 104 |  |  | PEAKS DB |
| total 1 peptides |  |  |  |  |  |  |  |  |  |  |  |  |  |  |  |  |  |  |  |
| --- | --- | --- | --- | --- | --- | --- | --- | --- | --- | --- | --- | --- | --- | --- | --- | --- | --- | --- | --- |

JAV51253.1

back to list

  

| Protein Coverage
| Supporting Peptides
|

Protein Coverage:

Supporting Peptides:

| Peptide | Uniq | -10lgP | Mass | Length | ppm | m/z | z | RT | Fraction | Scan | Source File | Area BuCaMH03\_B17 | #Feature | #Feature BuCaMH03\_B17 | Start | End | PTM | AScore | Found By |
| --- | --- | --- | --- | --- | --- | --- | --- | --- | --- | --- | --- | --- | --- | --- | --- | --- | --- | --- | --- |
| K.SALSGHLEAVILGLLK.T | Y | 81.97 | 1619.9609 | 16 | 0.2 | 540.9944 | 3 | 86.74 | 1 | 57625 | BuCaMH03.raw | 6.5848E5 | 1 | 1 | 89 | 104 |  |  | PEAKS DB |
| total 1 peptides |  |  |  |  |  |  |  |  |  |  |  |  |  |  |  |  |  |  |  |
| --- | --- | --- | --- | --- | --- | --- | --- | --- | --- | --- | --- | --- | --- | --- | --- | --- | --- | --- | --- |

XP\_007439873.1

back to list

  

| Protein Coverage
| Supporting Peptides
|

Protein Coverage:

Supporting Peptides:

| Peptide | Uniq | -10lgP | Mass | Length | ppm | m/z | z | RT | Fraction | Scan | Source File | Area BuCaMH03\_B17 | #Feature | #Feature BuCaMH03\_B17 | Start | End | PTM | AScore | Found By |
| --- | --- | --- | --- | --- | --- | --- | --- | --- | --- | --- | --- | --- | --- | --- | --- | --- | --- | --- | --- |
| K.SALSGHLEAVILGLLK.T | Y | 81.97 | 1619.9609 | 16 | 0.2 | 540.9944 | 3 | 86.74 | 1 | 57625 | BuCaMH03.raw | 6.5848E5 | 1 | 1 | 90 | 105 |  |  | PEAKS DB |
| total 1 peptides |  |  |  |  |  |  |  |  |  |  |  |  |  |  |  |  |  |  |  |
| --- | --- | --- | --- | --- | --- | --- | --- | --- | --- | --- | --- | --- | --- | --- | --- | --- | --- | --- | --- |

LAB40267.1

back to list

  

| Protein Coverage
| Supporting Peptides
|

Protein Coverage:

Supporting Peptides:

| Peptide | Uniq | -10lgP | Mass | Length | ppm | m/z | z | RT | Fraction | Scan | Source File | Area BuCaMH03\_B17 | #Feature | #Feature BuCaMH03\_B17 | Start | End | PTM | AScore | Found By |
| --- | --- | --- | --- | --- | --- | --- | --- | --- | --- | --- | --- | --- | --- | --- | --- | --- | --- | --- | --- |
| K.SALSGHLEAVILGLLK.T | Y | 81.97 | 1619.9609 | 16 | 0.2 | 540.9944 | 3 | 86.74 | 1 | 57625 | BuCaMH03.raw | 6.5848E5 | 1 | 1 | 97 | 112 |  |  | PEAKS DB |
| total 1 peptides |  |  |  |  |  |  |  |  |  |  |  |  |  |  |  |  |  |  |  |
| --- | --- | --- | --- | --- | --- | --- | --- | --- | --- | --- | --- | --- | --- | --- | --- | --- | --- | --- | --- |

ETE59687.1

back to list

  

| Protein Coverage
| Supporting Peptides
|

Protein Coverage:

Supporting Peptides:

| Peptide | Uniq | -10lgP | Mass | Length | ppm | m/z | z | RT | Fraction | Scan | Source File | Area BuCaMH03\_B17 | #Feature | #Feature BuCaMH03\_B17 | Start | End | PTM | AScore | Found By |
| --- | --- | --- | --- | --- | --- | --- | --- | --- | --- | --- | --- | --- | --- | --- | --- | --- | --- | --- | --- |
| K.SALSGHLEAVILGLLK.T | Y | 81.97 | 1619.9609 | 16 | 0.2 | 540.9944 | 3 | 86.74 | 1 | 57625 | BuCaMH03.raw | 6.5848E5 | 1 | 1 | 34 | 49 |  |  | PEAKS DB |
| total 1 peptides |  |  |  |  |  |  |  |  |  |  |  |  |  |  |  |  |  |  |  |
| --- | --- | --- | --- | --- | --- | --- | --- | --- | --- | --- | --- | --- | --- | --- | --- | --- | --- | --- | --- |

ETE61271.1

back to list

  

| Protein Coverage
| Supporting Peptides
|

Protein Coverage:

Supporting Peptides:

| Peptide | Uniq | -10lgP | Mass | Length | ppm | m/z | z | RT | Fraction | Scan | Source File | Area BuCaMH03\_B17 | #Feature | #Feature BuCaMH03\_B17 | Start | End | PTM | AScore | Found By |
| --- | --- | --- | --- | --- | --- | --- | --- | --- | --- | --- | --- | --- | --- | --- | --- | --- | --- | --- | --- |
| K.SILDMLLEATSPK.M | Y | 80.26 | 1416.7534 | 13 | 0.8 | 709.3845 | 2 | 97.63 | 1 | 66659 | BuCaMH03.raw | 1.2447E7 | 1 | 1 | 366 | 378 |  |  | PEAKS DB |
| total 1 peptides |  |  |  |  |  |  |  |  |  |  |  |  |  |  |  |  |  |  |  |
| --- | --- | --- | --- | --- | --- | --- | --- | --- | --- | --- | --- | --- | --- | --- | --- | --- | --- | --- | --- |

AFJ51008.1

back to list

  

| Protein Coverage
| Supporting Peptides
|

Protein Coverage:

Supporting Peptides:

| Peptide | Uniq | -10lgP | Mass | Length | ppm | m/z | z | RT | Fraction | Scan | Source File | Area BuCaMH03\_B17 | #Feature | #Feature BuCaMH03\_B17 | Start | End | PTM | AScore | Found By |
| --- | --- | --- | --- | --- | --- | --- | --- | --- | --- | --- | --- | --- | --- | --- | --- | --- | --- | --- | --- |
| K.SILDMLLEATSPK.M | Y | 80.26 | 1416.7534 | 13 | 0.8 | 709.3845 | 2 | 97.63 | 1 | 66659 | BuCaMH03.raw | 1.2447E7 | 1 | 1 | 362 | 374 |  |  | PEAKS DB |
| total 1 peptides |  |  |  |  |  |  |  |  |  |  |  |  |  |  |  |  |  |  |  |
| --- | --- | --- | --- | --- | --- | --- | --- | --- | --- | --- | --- | --- | --- | --- | --- | --- | --- | --- | --- |

XP\_032088229.1

back to list

  

| Protein Coverage
| Supporting Peptides
|

Protein Coverage:

Supporting Peptides:

| Peptide | Uniq | -10lgP | Mass | Length | ppm | m/z | z | RT | Fraction | Scan | Source File | Area BuCaMH03\_B17 | #Feature | #Feature BuCaMH03\_B17 | Start | End | PTM | AScore | Found By |
| --- | --- | --- | --- | --- | --- | --- | --- | --- | --- | --- | --- | --- | --- | --- | --- | --- | --- | --- | --- |
| K.SILDMLLEATSPK.M | Y | 80.26 | 1416.7534 | 13 | 0.8 | 709.3845 | 2 | 97.63 | 1 | 66659 | BuCaMH03.raw | 1.2447E7 | 1 | 1 | 362 | 374 |  |  | PEAKS DB |
| total 1 peptides |  |  |  |  |  |  |  |  |  |  |  |  |  |  |  |  |  |  |  |
| --- | --- | --- | --- | --- | --- | --- | --- | --- | --- | --- | --- | --- | --- | --- | --- | --- | --- | --- | --- |

XP\_013923763.1

back to list

  

| Protein Coverage
| Supporting Peptides
|

Protein Coverage:

Supporting Peptides:

| Peptide | Uniq | -10lgP | Mass | Length | ppm | m/z | z | RT | Fraction | Scan | Source File | Area BuCaMH03\_B17 | #Feature | #Feature BuCaMH03\_B17 | Start | End | PTM | AScore | Found By |
| --- | --- | --- | --- | --- | --- | --- | --- | --- | --- | --- | --- | --- | --- | --- | --- | --- | --- | --- | --- |
| K.SILDMLLEATSPK.M | Y | 80.26 | 1416.7534 | 13 | 0.8 | 709.3845 | 2 | 97.63 | 1 | 66659 | BuCaMH03.raw | 1.2447E7 | 1 | 1 | 362 | 374 |  |  | PEAKS DB |
| total 1 peptides |  |  |  |  |  |  |  |  |  |  |  |  |  |  |  |  |  |  |  |
| --- | --- | --- | --- | --- | --- | --- | --- | --- | --- | --- | --- | --- | --- | --- | --- | --- | --- | --- | --- |

XP\_013923762.1

back to list

  

| Protein Coverage
| Supporting Peptides
|

Protein Coverage:

Supporting Peptides:

| Peptide | Uniq | -10lgP | Mass | Length | ppm | m/z | z | RT | Fraction | Scan | Source File | Area BuCaMH03\_B17 | #Feature | #Feature BuCaMH03\_B17 | Start | End | PTM | AScore | Found By |
| --- | --- | --- | --- | --- | --- | --- | --- | --- | --- | --- | --- | --- | --- | --- | --- | --- | --- | --- | --- |
| K.SILDMLLEATSPK.M | Y | 80.26 | 1416.7534 | 13 | 0.8 | 709.3845 | 2 | 97.63 | 1 | 66659 | BuCaMH03.raw | 1.2447E7 | 1 | 1 | 365 | 377 |  |  | PEAKS DB |
| total 1 peptides |  |  |  |  |  |  |  |  |  |  |  |  |  |  |  |  |  |  |  |
| --- | --- | --- | --- | --- | --- | --- | --- | --- | --- | --- | --- | --- | --- | --- | --- | --- | --- | --- | --- |

XP\_032088228.1

back to list

  

| Protein Coverage
| Supporting Peptides
|

Protein Coverage:

Supporting Peptides:

| Peptide | Uniq | -10lgP | Mass | Length | ppm | m/z | z | RT | Fraction | Scan | Source File | Area BuCaMH03\_B17 | #Feature | #Feature BuCaMH03\_B17 | Start | End | PTM | AScore | Found By |
| --- | --- | --- | --- | --- | --- | --- | --- | --- | --- | --- | --- | --- | --- | --- | --- | --- | --- | --- | --- |
| K.SILDMLLEATSPK.M | Y | 80.26 | 1416.7534 | 13 | 0.8 | 709.3845 | 2 | 97.63 | 1 | 66659 | BuCaMH03.raw | 1.2447E7 | 1 | 1 | 365 | 377 |  |  | PEAKS DB |
| total 1 peptides |  |  |  |  |  |  |  |  |  |  |  |  |  |  |  |  |  |  |  |
| --- | --- | --- | --- | --- | --- | --- | --- | --- | --- | --- | --- | --- | --- | --- | --- | --- | --- | --- | --- |

XP\_015669101.1

back to list

  

| Protein Coverage
| Supporting Peptides
|

Protein Coverage:

Supporting Peptides:

| Peptide | Uniq | -10lgP | Mass | Length | ppm | m/z | z | RT | Fraction | Scan | Source File | Area BuCaMH03\_B17 | #Feature | #Feature BuCaMH03\_B17 | Start | End | PTM | AScore | Found By |
| --- | --- | --- | --- | --- | --- | --- | --- | --- | --- | --- | --- | --- | --- | --- | --- | --- | --- | --- | --- |
| K.SILDMLLEATSPK.M | Y | 80.26 | 1416.7534 | 13 | 0.8 | 709.3845 | 2 | 97.63 | 1 | 66659 | BuCaMH03.raw | 1.2447E7 | 1 | 1 | 362 | 374 |  |  | PEAKS DB |
| total 1 peptides |  |  |  |  |  |  |  |  |  |  |  |  |  |  |  |  |  |  |  |
| --- | --- | --- | --- | --- | --- | --- | --- | --- | --- | --- | --- | --- | --- | --- | --- | --- | --- | --- | --- |

BAN82088.1

back to list

  

| Protein Coverage
| Supporting Peptides
|

Protein Coverage:

Supporting Peptides:

| Peptide | Uniq | -10lgP | Mass | Length | ppm | m/z | z | RT | Fraction | Scan | Source File | Area BuCaMH03\_B17 | #Feature | #Feature BuCaMH03\_B17 | Start | End | PTM | AScore | Found By |
| --- | --- | --- | --- | --- | --- | --- | --- | --- | --- | --- | --- | --- | --- | --- | --- | --- | --- | --- | --- |
| K.SILDMLLEATSPK.M | Y | 80.26 | 1416.7534 | 13 | 0.8 | 709.3845 | 2 | 97.63 | 1 | 66659 | BuCaMH03.raw | 1.2447E7 | 1 | 1 | 365 | 377 |  |  | PEAKS DB |
| total 1 peptides |  |  |  |  |  |  |  |  |  |  |  |  |  |  |  |  |  |  |  |
| --- | --- | --- | --- | --- | --- | --- | --- | --- | --- | --- | --- | --- | --- | --- | --- | --- | --- | --- | --- |

XP\_034291658.1

back to list

  

| Protein Coverage
| Supporting Peptides
|

Protein Coverage:

Supporting Peptides:

| Peptide | Uniq | -10lgP | Mass | Length | ppm | m/z | z | RT | Fraction | Scan | Source File | Area BuCaMH03\_B17 | #Feature | #Feature BuCaMH03\_B17 | Start | End | PTM | AScore | Found By |
| --- | --- | --- | --- | --- | --- | --- | --- | --- | --- | --- | --- | --- | --- | --- | --- | --- | --- | --- | --- |
| K.SILDMLLEATSPK.M | Y | 80.26 | 1416.7534 | 13 | 0.8 | 709.3845 | 2 | 97.63 | 1 | 66659 | BuCaMH03.raw | 1.2447E7 | 1 | 1 | 365 | 377 |  |  | PEAKS DB |
| total 1 peptides |  |  |  |  |  |  |  |  |  |  |  |  |  |  |  |  |  |  |  |
| --- | --- | --- | --- | --- | --- | --- | --- | --- | --- | --- | --- | --- | --- | --- | --- | --- | --- | --- | --- |

XP\_034291656.1

back to list

  

| Protein Coverage
| Supporting Peptides
|

Protein Coverage:

Supporting Peptides:

| Peptide | Uniq | -10lgP | Mass | Length | ppm | m/z | z | RT | Fraction | Scan | Source File | Area BuCaMH03\_B17 | #Feature | #Feature BuCaMH03\_B17 | Start | End | PTM | AScore | Found By |
| --- | --- | --- | --- | --- | --- | --- | --- | --- | --- | --- | --- | --- | --- | --- | --- | --- | --- | --- | --- |
| K.SILDMLLEATSPK.M | Y | 80.26 | 1416.7534 | 13 | 0.8 | 709.3845 | 2 | 97.63 | 1 | 66659 | BuCaMH03.raw | 1.2447E7 | 1 | 1 | 367 | 379 |  |  | PEAKS DB |
| total 1 peptides |  |  |  |  |  |  |  |  |  |  |  |  |  |  |  |  |  |  |  |
| --- | --- | --- | --- | --- | --- | --- | --- | --- | --- | --- | --- | --- | --- | --- | --- | --- | --- | --- | --- |

XP\_034291657.1

back to list

  

| Protein Coverage
| Supporting Peptides
|

Protein Coverage:

Supporting Peptides:

| Peptide | Uniq | -10lgP | Mass | Length | ppm | m/z | z | RT | Fraction | Scan | Source File | Area BuCaMH03\_B17 | #Feature | #Feature BuCaMH03\_B17 | Start | End | PTM | AScore | Found By |
| --- | --- | --- | --- | --- | --- | --- | --- | --- | --- | --- | --- | --- | --- | --- | --- | --- | --- | --- | --- |
| K.SILDMLLEATSPK.M | Y | 80.26 | 1416.7534 | 13 | 0.8 | 709.3845 | 2 | 97.63 | 1 | 66659 | BuCaMH03.raw | 1.2447E7 | 1 | 1 | 368 | 380 |  |  | PEAKS DB |
| total 1 peptides |  |  |  |  |  |  |  |  |  |  |  |  |  |  |  |  |  |  |  |
| --- | --- | --- | --- | --- | --- | --- | --- | --- | --- | --- | --- | --- | --- | --- | --- | --- | --- | --- | --- |

XP\_034291655.1

back to list

  

| Protein Coverage
| Supporting Peptides
|

Protein Coverage:

Supporting Peptides:

| Peptide | Uniq | -10lgP | Mass | Length | ppm | m/z | z | RT | Fraction | Scan | Source File | Area BuCaMH03\_B17 | #Feature | #Feature BuCaMH03\_B17 | Start | End | PTM | AScore | Found By |
| --- | --- | --- | --- | --- | --- | --- | --- | --- | --- | --- | --- | --- | --- | --- | --- | --- | --- | --- | --- |
| K.SILDMLLEATSPK.M | Y | 80.26 | 1416.7534 | 13 | 0.8 | 709.3845 | 2 | 97.63 | 1 | 66659 | BuCaMH03.raw | 1.2447E7 | 1 | 1 | 368 | 380 |  |  | PEAKS DB |
| total 1 peptides |  |  |  |  |  |  |  |  |  |  |  |  |  |  |  |  |  |  |  |
| --- | --- | --- | --- | --- | --- | --- | --- | --- | --- | --- | --- | --- | --- | --- | --- | --- | --- | --- | --- |

XP\_026569354.1

back to list

  

| Protein Coverage
| Supporting Peptides
|

Protein Coverage:

Supporting Peptides:

| Peptide | Uniq | -10lgP | Mass | Length | ppm | m/z | z | RT | Fraction | Scan | Source File | Area BuCaMH03\_B17 | #Feature | #Feature BuCaMH03\_B17 | Start | End | PTM | AScore | Found By |
| --- | --- | --- | --- | --- | --- | --- | --- | --- | --- | --- | --- | --- | --- | --- | --- | --- | --- | --- | --- |
| K.SILDMLLEATSPK.M | Y | 80.26 | 1416.7534 | 13 | 0.8 | 709.3845 | 2 | 97.63 | 1 | 66659 | BuCaMH03.raw | 1.2447E7 | 1 | 1 | 362 | 374 |  |  | PEAKS DB |
| total 1 peptides |  |  |  |  |  |  |  |  |  |  |  |  |  |  |  |  |  |  |  |
| --- | --- | --- | --- | --- | --- | --- | --- | --- | --- | --- | --- | --- | --- | --- | --- | --- | --- | --- | --- |

XP\_026569353.1

back to list

  

| Protein Coverage
| Supporting Peptides
|

Protein Coverage:

Supporting Peptides:

| Peptide | Uniq | -10lgP | Mass | Length | ppm | m/z | z | RT | Fraction | Scan | Source File | Area BuCaMH03\_B17 | #Feature | #Feature BuCaMH03\_B17 | Start | End | PTM | AScore | Found By |
| --- | --- | --- | --- | --- | --- | --- | --- | --- | --- | --- | --- | --- | --- | --- | --- | --- | --- | --- | --- |
| K.SILDMLLEATSPK.M | Y | 80.26 | 1416.7534 | 13 | 0.8 | 709.3845 | 2 | 97.63 | 1 | 66659 | BuCaMH03.raw | 1.2447E7 | 1 | 1 | 365 | 377 |  |  | PEAKS DB |
| total 1 peptides |  |  |  |  |  |  |  |  |  |  |  |  |  |  |  |  |  |  |  |
| --- | --- | --- | --- | --- | --- | --- | --- | --- | --- | --- | --- | --- | --- | --- | --- | --- | --- | --- | --- |

XP\_026528620.1

back to list

  

| Protein Coverage
| Supporting Peptides
|

Protein Coverage:

Supporting Peptides:

| Peptide | Uniq | -10lgP | Mass | Length | ppm | m/z | z | RT | Fraction | Scan | Source File | Area BuCaMH03\_B17 | #Feature | #Feature BuCaMH03\_B17 | Start | End | PTM | AScore | Found By |
| --- | --- | --- | --- | --- | --- | --- | --- | --- | --- | --- | --- | --- | --- | --- | --- | --- | --- | --- | --- |
| K.SILDMLLEATSPK.M | Y | 80.26 | 1416.7534 | 13 | 0.8 | 709.3845 | 2 | 97.63 | 1 | 66659 | BuCaMH03.raw | 1.2447E7 | 1 | 1 | 362 | 374 |  |  | PEAKS DB |
| total 1 peptides |  |  |  |  |  |  |  |  |  |  |  |  |  |  |  |  |  |  |  |
| --- | --- | --- | --- | --- | --- | --- | --- | --- | --- | --- | --- | --- | --- | --- | --- | --- | --- | --- | --- |

XP\_026528619.1

back to list

  

| Protein Coverage
| Supporting Peptides
|

Protein Coverage:

Supporting Peptides:

| Peptide | Uniq | -10lgP | Mass | Length | ppm | m/z | z | RT | Fraction | Scan | Source File | Area BuCaMH03\_B17 | #Feature | #Feature BuCaMH03\_B17 | Start | End | PTM | AScore | Found By |
| --- | --- | --- | --- | --- | --- | --- | --- | --- | --- | --- | --- | --- | --- | --- | --- | --- | --- | --- | --- |
| K.SILDMLLEATSPK.M | Y | 80.26 | 1416.7534 | 13 | 0.8 | 709.3845 | 2 | 97.63 | 1 | 66659 | BuCaMH03.raw | 1.2447E7 | 1 | 1 | 365 | 377 |  |  | PEAKS DB |
| total 1 peptides |  |  |  |  |  |  |  |  |  |  |  |  |  |  |  |  |  |  |  |
| --- | --- | --- | --- | --- | --- | --- | --- | --- | --- | --- | --- | --- | --- | --- | --- | --- | --- | --- | --- |

XP\_007441356.1

back to list

  

| Protein Coverage
| Supporting Peptides
|

Protein Coverage:

Supporting Peptides:

| Peptide | Uniq | -10lgP | Mass | Length | ppm | m/z | z | RT | Fraction | Scan | Source File | Area BuCaMH03\_B17 | #Feature | #Feature BuCaMH03\_B17 | Start | End | PTM | AScore | Found By |
| --- | --- | --- | --- | --- | --- | --- | --- | --- | --- | --- | --- | --- | --- | --- | --- | --- | --- | --- | --- |
| K.SIIDMLLEATSPK.M | Y | 80.26 | 1416.7534 | 13 | 0.8 | 709.3845 | 2 | 97.63 | 1 | 66659 | BuCaMH03.raw | 1.2447E7 | 1 | 1 | 227 | 239 |  |  | PEAKS DB |
| total 1 peptides |  |  |  |  |  |  |  |  |  |  |  |  |  |  |  |  |  |  |  |
| --- | --- | --- | --- | --- | --- | --- | --- | --- | --- | --- | --- | --- | --- | --- | --- | --- | --- | --- | --- |

1MR6

back to list

  

| Protein Coverage
| Supporting Peptides
|

Protein Coverage:

Supporting Peptides:

| Peptide | Uniq | -10lgP | Mass | Length | ppm | m/z | z | RT | Fraction | Scan | Source File | Area BuCaMH03\_B17 | #Feature | #Feature BuCaMH03\_B17 | Start | End | PTM | AScore | Found By |
| --- | --- | --- | --- | --- | --- | --- | --- | --- | --- | --- | --- | --- | --- | --- | --- | --- | --- | --- | --- |
| K.TC(+57.02)SFYTC(+57.02)PNSETC(+57.02)PDGK.N | Y | 57.17 | 2022.7758 | 17 | 4.4 | 675.2688 | 3 | 19.76 | 1 | 5449 | BuCaMH03.raw | 1.6747E6 | 1 | 1 | 5 | 21 | Carbamidomethylation | C2:Carbamidomethylation:1000.00;C7:Carbamidomethylation:1000.00;C13:Carbamidomethylation:1000.00 | PEAKS DB |
| total 1 peptides |  |  |  |  |  |  |  |  |  |  |  |  |  |  |  |  |  |  |  |
| --- | --- | --- | --- | --- | --- | --- | --- | --- | --- | --- | --- | --- | --- | --- | --- | --- | --- | --- | --- |

CAA06885.1

back to list

  

| Protein Coverage
| Supporting Peptides
|

Protein Coverage:

Supporting Peptides:

| Peptide | Uniq | -10lgP | Mass | Length | ppm | m/z | z | RT | Fraction | Scan | Source File | Area BuCaMH03\_B17 | #Feature | #Feature BuCaMH03\_B17 | Start | End | PTM | AScore | Found By |
| --- | --- | --- | --- | --- | --- | --- | --- | --- | --- | --- | --- | --- | --- | --- | --- | --- | --- | --- | --- |
| K.TC(+57.02)SFYTC(+57.02)PNSETC(+57.02)PDGK.N | Y | 57.17 | 2022.7758 | 17 | 4.4 | 675.2688 | 3 | 19.76 | 1 | 5449 | BuCaMH03.raw | 1.6747E6 | 1 | 1 | 26 | 42 | Carbamidomethylation | C2:Carbamidomethylation:1000.00;C7:Carbamidomethylation:1000.00;C13:Carbamidomethylation:1000.00 | PEAKS DB |
| total 1 peptides |  |  |  |  |  |  |  |  |  |  |  |  |  |  |  |  |  |  |  |
| --- | --- | --- | --- | --- | --- | --- | --- | --- | --- | --- | --- | --- | --- | --- | --- | --- | --- | --- | --- |

Q9YGJ0.1

back to list

  

| Protein Coverage
| Supporting Peptides
|

Protein Coverage:

Supporting Peptides:

| Peptide | Uniq | -10lgP | Mass | Length | ppm | m/z | z | RT | Fraction | Scan | Source File | Area BuCaMH03\_B17 | #Feature | #Feature BuCaMH03\_B17 | Start | End | PTM | AScore | Found By |
| --- | --- | --- | --- | --- | --- | --- | --- | --- | --- | --- | --- | --- | --- | --- | --- | --- | --- | --- | --- |
| K.TC(+57.02)SFYTC(+57.02)PNSETC(+57.02)PDGK.N | Y | 57.17 | 2022.7758 | 17 | 4.4 | 675.2688 | 3 | 19.76 | 1 | 5449 | BuCaMH03.raw | 1.6747E6 | 1 | 1 | 26 | 42 | Carbamidomethylation | C2:Carbamidomethylation:1000.00;C7:Carbamidomethylation:1000.00;C13:Carbamidomethylation:1000.00 | PEAKS DB |
| total 1 peptides |  |  |  |  |  |  |  |  |  |  |  |  |  |  |  |  |  |  |  |
| --- | --- | --- | --- | --- | --- | --- | --- | --- | --- | --- | --- | --- | --- | --- | --- | --- | --- | --- | --- |

AAD41806.1

back to list

  

| Protein Coverage
| Supporting Peptides
|

Protein Coverage:

Supporting Peptides:

| Peptide | Uniq | -10lgP | Mass | Length | ppm | m/z | z | RT | Fraction | Scan | Source File | Area BuCaMH03\_B17 | #Feature | #Feature BuCaMH03\_B17 | Start | End | PTM | AScore | Found By |
| --- | --- | --- | --- | --- | --- | --- | --- | --- | --- | --- | --- | --- | --- | --- | --- | --- | --- | --- | --- |
| K.TC(+57.02)SFYTC(+57.02)PNSETC(+57.02)PDGK.N | Y | 57.17 | 2022.7758 | 17 | 4.4 | 675.2688 | 3 | 19.76 | 1 | 5449 | BuCaMH03.raw | 1.6747E6 | 1 | 1 | 26 | 42 | Carbamidomethylation | C2:Carbamidomethylation:1000.00;C7:Carbamidomethylation:1000.00;C13:Carbamidomethylation:1000.00 | PEAKS DB |
| total 1 peptides |  |  |  |  |  |  |  |  |  |  |  |  |  |  |  |  |  |  |  |
| --- | --- | --- | --- | --- | --- | --- | --- | --- | --- | --- | --- | --- | --- | --- | --- | --- | --- | --- | --- |

CAA72941.1

back to list

  

| Protein Coverage
| Supporting Peptides
|

Protein Coverage:

Supporting Peptides:

| Peptide | Uniq | -10lgP | Mass | Length | ppm | m/z | z | RT | Fraction | Scan | Source File | Area BuCaMH03\_B17 | #Feature | #Feature BuCaMH03\_B17 | Start | End | PTM | AScore | Found By |
| --- | --- | --- | --- | --- | --- | --- | --- | --- | --- | --- | --- | --- | --- | --- | --- | --- | --- | --- | --- |
| K.TC(+57.02)SFYTC(+57.02)PNSETC(+57.02)PDGK.N | Y | 57.17 | 2022.7758 | 17 | 4.4 | 675.2688 | 3 | 19.76 | 1 | 5449 | BuCaMH03.raw | 1.6747E6 | 1 | 1 | 26 | 42 | Carbamidomethylation | C2:Carbamidomethylation:1000.00;C7:Carbamidomethylation:1000.00;C13:Carbamidomethylation:1000.00 | PEAKS DB |
| total 1 peptides |  |  |  |  |  |  |  |  |  |  |  |  |  |  |  |  |  |  |  |
| --- | --- | --- | --- | --- | --- | --- | --- | --- | --- | --- | --- | --- | --- | --- | --- | --- | --- | --- | --- |

CAD01082.1

back to list

  

| Protein Coverage
| Supporting Peptides
|

Protein Coverage:

Supporting Peptides:

| Peptide | Uniq | -10lgP | Mass | Length | ppm | m/z | z | RT | Fraction | Scan | Source File | Area BuCaMH03\_B17 | #Feature | #Feature BuCaMH03\_B17 | Start | End | PTM | AScore | Found By |
| --- | --- | --- | --- | --- | --- | --- | --- | --- | --- | --- | --- | --- | --- | --- | --- | --- | --- | --- | --- |
| K.TC(+57.02)SFYTC(+57.02)PNSETC(+57.02)PDGK.N | Y | 57.17 | 2022.7758 | 17 | 4.4 | 675.2688 | 3 | 19.76 | 1 | 5449 | BuCaMH03.raw | 1.6747E6 | 1 | 1 | 26 | 42 | Carbamidomethylation | C2:Carbamidomethylation:1000.00;C7:Carbamidomethylation:1000.00;C13:Carbamidomethylation:1000.00 | PEAKS DB |
| total 1 peptides |  |  |  |  |  |  |  |  |  |  |  |  |  |  |  |  |  |  |  |
| --- | --- | --- | --- | --- | --- | --- | --- | --- | --- | --- | --- | --- | --- | --- | --- | --- | --- | --- | --- |

O12963.1

back to list

  

| Protein Coverage
| Supporting Peptides
|

Protein Coverage:

Supporting Peptides:

| Peptide | Uniq | -10lgP | Mass | Length | ppm | m/z | z | RT | Fraction | Scan | Source File | Area BuCaMH03\_B17 | #Feature | #Feature BuCaMH03\_B17 | Start | End | PTM | AScore | Found By |
| --- | --- | --- | --- | --- | --- | --- | --- | --- | --- | --- | --- | --- | --- | --- | --- | --- | --- | --- | --- |
| K.TC(+57.02)SFYTC(+57.02)PNSETC(+57.02)PDGK.N | Y | 57.17 | 2022.7758 | 17 | 4.4 | 675.2688 | 3 | 19.76 | 1 | 5449 | BuCaMH03.raw | 1.6747E6 | 1 | 1 | 26 | 42 | Carbamidomethylation | C2:Carbamidomethylation:1000.00;C7:Carbamidomethylation:1000.00;C13:Carbamidomethylation:1000.00 | PEAKS DB |
| total 1 peptides |  |  |  |  |  |  |  |  |  |  |  |  |  |  |  |  |  |  |  |
| --- | --- | --- | --- | --- | --- | --- | --- | --- | --- | --- | --- | --- | --- | --- | --- | --- | --- | --- | --- |

LAB19298.1

back to list

  

| Protein Coverage
| Supporting Peptides
|

Protein Coverage:

Supporting Peptides:

| Peptide | Uniq | -10lgP | Mass | Length | ppm | m/z | z | RT | Fraction | Scan | Source File | Area BuCaMH03\_B17 | #Feature | #Feature BuCaMH03\_B17 | Start | End | PTM | AScore | Found By |
| --- | --- | --- | --- | --- | --- | --- | --- | --- | --- | --- | --- | --- | --- | --- | --- | --- | --- | --- | --- |
| R.WLQNEQPVSESAYFTSK.A | Y | 54.48 | 2012.9479 | 17 | 0.8 | 1007.4821 | 2 | 55.17 | 1 | 31835 | BuCaMH03.raw | 1.0705E6 | 1 | 1 | 388 | 404 |  |  | PEAKS DB |
| total 1 peptides |  |  |  |  |  |  |  |  |  |  |  |  |  |  |  |  |  |  |  |
| --- | --- | --- | --- | --- | --- | --- | --- | --- | --- | --- | --- | --- | --- | --- | --- | --- | --- | --- | --- |

LAB19293.1

back to list

  

| Protein Coverage
| Supporting Peptides
|

Protein Coverage:

Supporting Peptides:

| Peptide | Uniq | -10lgP | Mass | Length | ppm | m/z | z | RT | Fraction | Scan | Source File | Area BuCaMH03\_B17 | #Feature | #Feature BuCaMH03\_B17 | Start | End | PTM | AScore | Found By |
| --- | --- | --- | --- | --- | --- | --- | --- | --- | --- | --- | --- | --- | --- | --- | --- | --- | --- | --- | --- |
| R.WLQNEQPVSESAYFTSK.A | Y | 54.48 | 2012.9479 | 17 | 0.8 | 1007.4821 | 2 | 55.17 | 1 | 31835 | BuCaMH03.raw | 1.0705E6 | 1 | 1 | 388 | 404 |  |  | PEAKS DB |
| total 1 peptides |  |  |  |  |  |  |  |  |  |  |  |  |  |  |  |  |  |  |  |
| --- | --- | --- | --- | --- | --- | --- | --- | --- | --- | --- | --- | --- | --- | --- | --- | --- | --- | --- | --- |

LAB19285.1

back to list

  

| Protein Coverage
| Supporting Peptides
|

Protein Coverage:

Supporting Peptides:

| Peptide | Uniq | -10lgP | Mass | Length | ppm | m/z | z | RT | Fraction | Scan | Source File | Area BuCaMH03\_B17 | #Feature | #Feature BuCaMH03\_B17 | Start | End | PTM | AScore | Found By |
| --- | --- | --- | --- | --- | --- | --- | --- | --- | --- | --- | --- | --- | --- | --- | --- | --- | --- | --- | --- |
| R.WLQNEQPVSESAYFTSK.A | Y | 54.48 | 2012.9479 | 17 | 0.8 | 1007.4821 | 2 | 55.17 | 1 | 31835 | BuCaMH03.raw | 1.0705E6 | 1 | 1 | 398 | 414 |  |  | PEAKS DB |
| total 1 peptides |  |  |  |  |  |  |  |  |  |  |  |  |  |  |  |  |  |  |  |
| --- | --- | --- | --- | --- | --- | --- | --- | --- | --- | --- | --- | --- | --- | --- | --- | --- | --- | --- | --- |

LAB19295.1

back to list

  

| Protein Coverage
| Supporting Peptides
|

Protein Coverage:

Supporting Peptides:

| Peptide | Uniq | -10lgP | Mass | Length | ppm | m/z | z | RT | Fraction | Scan | Source File | Area BuCaMH03\_B17 | #Feature | #Feature BuCaMH03\_B17 | Start | End | PTM | AScore | Found By |
| --- | --- | --- | --- | --- | --- | --- | --- | --- | --- | --- | --- | --- | --- | --- | --- | --- | --- | --- | --- |
| R.WLQNEQPVSESAYFTSK.A | Y | 54.48 | 2012.9479 | 17 | 0.8 | 1007.4821 | 2 | 55.17 | 1 | 31835 | BuCaMH03.raw | 1.0705E6 | 1 | 1 | 398 | 414 |  |  | PEAKS DB |
| total 1 peptides |  |  |  |  |  |  |  |  |  |  |  |  |  |  |  |  |  |  |  |
| --- | --- | --- | --- | --- | --- | --- | --- | --- | --- | --- | --- | --- | --- | --- | --- | --- | --- | --- | --- |

LAB19282.1

back to list

  

| Protein Coverage
| Supporting Peptides
|

Protein Coverage:

Supporting Peptides:

| Peptide | Uniq | -10lgP | Mass | Length | ppm | m/z | z | RT | Fraction | Scan | Source File | Area BuCaMH03\_B17 | #Feature | #Feature BuCaMH03\_B17 | Start | End | PTM | AScore | Found By |
| --- | --- | --- | --- | --- | --- | --- | --- | --- | --- | --- | --- | --- | --- | --- | --- | --- | --- | --- | --- |
| R.WLQNEQPVSESAYFTSK.A | Y | 54.48 | 2012.9479 | 17 | 0.8 | 1007.4821 | 2 | 55.17 | 1 | 31835 | BuCaMH03.raw | 1.0705E6 | 1 | 1 | 439 | 455 |  |  | PEAKS DB |
| total 1 peptides |  |  |  |  |  |  |  |  |  |  |  |  |  |  |  |  |  |  |  |
| --- | --- | --- | --- | --- | --- | --- | --- | --- | --- | --- | --- | --- | --- | --- | --- | --- | --- | --- | --- |

LAB19291.1

back to list

  

| Protein Coverage
| Supporting Peptides
|

Protein Coverage:

Supporting Peptides:

| Peptide | Uniq | -10lgP | Mass | Length | ppm | m/z | z | RT | Fraction | Scan | Source File | Area BuCaMH03\_B17 | #Feature | #Feature BuCaMH03\_B17 | Start | End | PTM | AScore | Found By |
| --- | --- | --- | --- | --- | --- | --- | --- | --- | --- | --- | --- | --- | --- | --- | --- | --- | --- | --- | --- |
| R.WLQNEQPVSESAYFTSK.A | Y | 54.48 | 2012.9479 | 17 | 0.8 | 1007.4821 | 2 | 55.17 | 1 | 31835 | BuCaMH03.raw | 1.0705E6 | 1 | 1 | 458 | 474 |  |  | PEAKS DB |
| total 1 peptides |  |  |  |  |  |  |  |  |  |  |  |  |  |  |  |  |  |  |  |
| --- | --- | --- | --- | --- | --- | --- | --- | --- | --- | --- | --- | --- | --- | --- | --- | --- | --- | --- | --- |

LAB19280.1

back to list

  

| Protein Coverage
| Supporting Peptides
|

Protein Coverage:

Supporting Peptides:

| Peptide | Uniq | -10lgP | Mass | Length | ppm | m/z | z | RT | Fraction | Scan | Source File | Area BuCaMH03\_B17 | #Feature | #Feature BuCaMH03\_B17 | Start | End | PTM | AScore | Found By |
| --- | --- | --- | --- | --- | --- | --- | --- | --- | --- | --- | --- | --- | --- | --- | --- | --- | --- | --- | --- |
| R.WLQNEQPVSESAYFTSK.A | Y | 54.48 | 2012.9479 | 17 | 0.8 | 1007.4821 | 2 | 55.17 | 1 | 31835 | BuCaMH03.raw | 1.0705E6 | 1 | 1 | 465 | 481 |  |  | PEAKS DB |
| total 1 peptides |  |  |  |  |  |  |  |  |  |  |  |  |  |  |  |  |  |  |  |
| --- | --- | --- | --- | --- | --- | --- | --- | --- | --- | --- | --- | --- | --- | --- | --- | --- | --- | --- | --- |

XP\_026576367.1

back to list

  

| Protein Coverage
| Supporting Peptides
|

Protein Coverage:

Supporting Peptides:

| Peptide | Uniq | -10lgP | Mass | Length | ppm | m/z | z | RT | Fraction | Scan | Source File | Area BuCaMH03\_B17 | #Feature | #Feature BuCaMH03\_B17 | Start | End | PTM | AScore | Found By |
| --- | --- | --- | --- | --- | --- | --- | --- | --- | --- | --- | --- | --- | --- | --- | --- | --- | --- | --- | --- |
| M.SIMNSFVNDIFER.I | Y | 64.67 | 1570.7450 | 13 | 0.4 | 786.3801 | 2 | 81.33 | 1 | 53458 | BuCaMH03.raw | 2.2177E5 | 1 | 1 | 61 | 73 |  |  | PEAKS DB |
| total 1 peptides |  |  |  |  |  |  |  |  |  |  |  |  |  |  |  |  |  |  |  |
| --- | --- | --- | --- | --- | --- | --- | --- | --- | --- | --- | --- | --- | --- | --- | --- | --- | --- | --- | --- |

XP\_026577747.1

back to list

  

| Protein Coverage
| Supporting Peptides
|

Protein Coverage:

Supporting Peptides:

| Peptide | Uniq | -10lgP | Mass | Length | ppm | m/z | z | RT | Fraction | Scan | Source File | Area BuCaMH03\_B17 | #Feature | #Feature BuCaMH03\_B17 | Start | End | PTM | AScore | Found By |
| --- | --- | --- | --- | --- | --- | --- | --- | --- | --- | --- | --- | --- | --- | --- | --- | --- | --- | --- | --- |
| M.SIMNSFVNDIFER.I | Y | 64.67 | 1570.7450 | 13 | 0.4 | 786.3801 | 2 | 81.33 | 1 | 53458 | BuCaMH03.raw | 2.2177E5 | 1 | 1 | 61 | 73 |  |  | PEAKS DB |
| total 1 peptides |  |  |  |  |  |  |  |  |  |  |  |  |  |  |  |  |  |  |  |
| --- | --- | --- | --- | --- | --- | --- | --- | --- | --- | --- | --- | --- | --- | --- | --- | --- | --- | --- | --- |

XP\_015675791.1

back to list

  

| Protein Coverage
| Supporting Peptides
|

Protein Coverage:

Supporting Peptides:

| Peptide | Uniq | -10lgP | Mass | Length | ppm | m/z | z | RT | Fraction | Scan | Source File | Area BuCaMH03\_B17 | #Feature | #Feature BuCaMH03\_B17 | Start | End | PTM | AScore | Found By |
| --- | --- | --- | --- | --- | --- | --- | --- | --- | --- | --- | --- | --- | --- | --- | --- | --- | --- | --- | --- |
| V.SIMNSFVNDIFER.I | Y | 64.67 | 1570.7450 | 13 | 0.4 | 786.3801 | 2 | 81.33 | 1 | 53458 | BuCaMH03.raw | 2.2177E5 | 1 | 1 | 13 | 25 |  |  | PEAKS DB |
| total 1 peptides |  |  |  |  |  |  |  |  |  |  |  |  |  |  |  |  |  |  |  |
| --- | --- | --- | --- | --- | --- | --- | --- | --- | --- | --- | --- | --- | --- | --- | --- | --- | --- | --- | --- |

XP\_015672174.1

back to list

  

| Protein Coverage
| Supporting Peptides
|

Protein Coverage:

Supporting Peptides:

| Peptide | Uniq | -10lgP | Mass | Length | ppm | m/z | z | RT | Fraction | Scan | Source File | Area BuCaMH03\_B17 | #Feature | #Feature BuCaMH03\_B17 | Start | End | PTM | AScore | Found By |
| --- | --- | --- | --- | --- | --- | --- | --- | --- | --- | --- | --- | --- | --- | --- | --- | --- | --- | --- | --- |
| M.SIMNSFVNDIFER.I | Y | 64.67 | 1570.7450 | 13 | 0.4 | 786.3801 | 2 | 81.33 | 1 | 53458 | BuCaMH03.raw | 2.2177E5 | 1 | 1 | 61 | 73 |  |  | PEAKS DB |
| total 1 peptides |  |  |  |  |  |  |  |  |  |  |  |  |  |  |  |  |  |  |  |
| --- | --- | --- | --- | --- | --- | --- | --- | --- | --- | --- | --- | --- | --- | --- | --- | --- | --- | --- | --- |

XP\_026539722.1

back to list

  

| Protein Coverage
| Supporting Peptides
|

Protein Coverage:

Supporting Peptides:

| Peptide | Uniq | -10lgP | Mass | Length | ppm | m/z | z | RT | Fraction | Scan | Source File | Area BuCaMH03\_B17 | #Feature | #Feature BuCaMH03\_B17 | Start | End | PTM | AScore | Found By |
| --- | --- | --- | --- | --- | --- | --- | --- | --- | --- | --- | --- | --- | --- | --- | --- | --- | --- | --- | --- |
| M.SIMNSFVNDIFER.I | Y | 64.67 | 1570.7450 | 13 | 0.4 | 786.3801 | 2 | 81.33 | 1 | 53458 | BuCaMH03.raw | 2.2177E5 | 1 | 1 | 61 | 73 |  |  | PEAKS DB |
| total 1 peptides |  |  |  |  |  |  |  |  |  |  |  |  |  |  |  |  |  |  |  |
| --- | --- | --- | --- | --- | --- | --- | --- | --- | --- | --- | --- | --- | --- | --- | --- | --- | --- | --- | --- |

LAA79020.1

back to list

  

| Protein Coverage
| Supporting Peptides
|

Protein Coverage:

Supporting Peptides:

| Peptide | Uniq | -10lgP | Mass | Length | ppm | m/z | z | RT | Fraction | Scan | Source File | Area BuCaMH03\_B17 | #Feature | #Feature BuCaMH03\_B17 | Start | End | PTM | AScore | Found By |
| --- | --- | --- | --- | --- | --- | --- | --- | --- | --- | --- | --- | --- | --- | --- | --- | --- | --- | --- | --- |
| M.SIMNSFVNDIFER.I | Y | 64.67 | 1570.7450 | 13 | 0.4 | 786.3801 | 2 | 81.33 | 1 | 53458 | BuCaMH03.raw | 2.2177E5 | 1 | 1 | 61 | 73 |  |  | PEAKS DB |
| total 1 peptides |  |  |  |  |  |  |  |  |  |  |  |  |  |  |  |  |  |  |  |
| --- | --- | --- | --- | --- | --- | --- | --- | --- | --- | --- | --- | --- | --- | --- | --- | --- | --- | --- | --- |

XP\_026569519.1

back to list

  

| Protein Coverage
| Supporting Peptides
|

Protein Coverage:

Supporting Peptides:

| Peptide | Uniq | -10lgP | Mass | Length | ppm | m/z | z | RT | Fraction | Scan | Source File | Area BuCaMH03\_B17 | #Feature | #Feature BuCaMH03\_B17 | Start | End | PTM | AScore | Found By |
| --- | --- | --- | --- | --- | --- | --- | --- | --- | --- | --- | --- | --- | --- | --- | --- | --- | --- | --- | --- |
| M.SIMNSFVNDIFER.I | Y | 64.67 | 1570.7450 | 13 | 0.4 | 786.3801 | 2 | 81.33 | 1 | 53458 | BuCaMH03.raw | 2.2177E5 | 1 | 1 | 61 | 73 |  |  | PEAKS DB |
| total 1 peptides |  |  |  |  |  |  |  |  |  |  |  |  |  |  |  |  |  |  |  |
| --- | --- | --- | --- | --- | --- | --- | --- | --- | --- | --- | --- | --- | --- | --- | --- | --- | --- | --- | --- |

XP\_026576364.1

back to list

  

| Protein Coverage
| Supporting Peptides
|

Protein Coverage:

Supporting Peptides:

| Peptide | Uniq | -10lgP | Mass | Length | ppm | m/z | z | RT | Fraction | Scan | Source File | Area BuCaMH03\_B17 | #Feature | #Feature BuCaMH03\_B17 | Start | End | PTM | AScore | Found By |
| --- | --- | --- | --- | --- | --- | --- | --- | --- | --- | --- | --- | --- | --- | --- | --- | --- | --- | --- | --- |
| M.SIMNSFVNDIFER.I | Y | 64.67 | 1570.7450 | 13 | 0.4 | 786.3801 | 2 | 81.33 | 1 | 53458 | BuCaMH03.raw | 2.2177E5 | 1 | 1 | 61 | 73 |  |  | PEAKS DB |
| total 1 peptides |  |  |  |  |  |  |  |  |  |  |  |  |  |  |  |  |  |  |  |
| --- | --- | --- | --- | --- | --- | --- | --- | --- | --- | --- | --- | --- | --- | --- | --- | --- | --- | --- | --- |

XP\_015678744.1

back to list

  

| Protein Coverage
| Supporting Peptides
|

Protein Coverage:

Supporting Peptides:

| Peptide | Uniq | -10lgP | Mass | Length | ppm | m/z | z | RT | Fraction | Scan | Source File | Area BuCaMH03\_B17 | #Feature | #Feature BuCaMH03\_B17 | Start | End | PTM | AScore | Found By |
| --- | --- | --- | --- | --- | --- | --- | --- | --- | --- | --- | --- | --- | --- | --- | --- | --- | --- | --- | --- |
| M.SIMNSFVNDIFER.I | Y | 64.67 | 1570.7450 | 13 | 0.4 | 786.3801 | 2 | 81.33 | 1 | 53458 | BuCaMH03.raw | 2.2177E5 | 1 | 1 | 61 | 73 |  |  | PEAKS DB |
| total 1 peptides |  |  |  |  |  |  |  |  |  |  |  |  |  |  |  |  |  |  |  |
| --- | --- | --- | --- | --- | --- | --- | --- | --- | --- | --- | --- | --- | --- | --- | --- | --- | --- | --- | --- |

XP\_026545427.1

back to list

  

| Protein Coverage
| Supporting Peptides
|

Protein Coverage:

Supporting Peptides:

| Peptide | Uniq | -10lgP | Mass | Length | ppm | m/z | z | RT | Fraction | Scan | Source File | Area BuCaMH03\_B17 | #Feature | #Feature BuCaMH03\_B17 | Start | End | PTM | AScore | Found By |
| --- | --- | --- | --- | --- | --- | --- | --- | --- | --- | --- | --- | --- | --- | --- | --- | --- | --- | --- | --- |
| M.SIMNSFVNDIFER.I | Y | 64.67 | 1570.7450 | 13 | 0.4 | 786.3801 | 2 | 81.33 | 1 | 53458 | BuCaMH03.raw | 2.2177E5 | 1 | 1 | 61 | 73 |  |  | PEAKS DB |
| total 1 peptides |  |  |  |  |  |  |  |  |  |  |  |  |  |  |  |  |  |  |  |
| --- | --- | --- | --- | --- | --- | --- | --- | --- | --- | --- | --- | --- | --- | --- | --- | --- | --- | --- | --- |

XP\_026529646.1

back to list

  

| Protein Coverage
| Supporting Peptides
|

Protein Coverage:

Supporting Peptides:

| Peptide | Uniq | -10lgP | Mass | Length | ppm | m/z | z | RT | Fraction | Scan | Source File | Area BuCaMH03\_B17 | #Feature | #Feature BuCaMH03\_B17 | Start | End | PTM | AScore | Found By |
| --- | --- | --- | --- | --- | --- | --- | --- | --- | --- | --- | --- | --- | --- | --- | --- | --- | --- | --- | --- |
| M.SIMNSFVNDIFER.I | Y | 64.67 | 1570.7450 | 13 | 0.4 | 786.3801 | 2 | 81.33 | 1 | 53458 | BuCaMH03.raw | 2.2177E5 | 1 | 1 | 61 | 73 |  |  | PEAKS DB |
| total 1 peptides |  |  |  |  |  |  |  |  |  |  |  |  |  |  |  |  |  |  |  |
| --- | --- | --- | --- | --- | --- | --- | --- | --- | --- | --- | --- | --- | --- | --- | --- | --- | --- | --- | --- |

XP\_013925621.1

back to list

  

| Protein Coverage
| Supporting Peptides
|

Protein Coverage:

Supporting Peptides:

| Peptide | Uniq | -10lgP | Mass | Length | ppm | m/z | z | RT | Fraction | Scan | Source File | Area BuCaMH03\_B17 | #Feature | #Feature BuCaMH03\_B17 | Start | End | PTM | AScore | Found By |
| --- | --- | --- | --- | --- | --- | --- | --- | --- | --- | --- | --- | --- | --- | --- | --- | --- | --- | --- | --- |
| M.SIMNSFVNDIFER.I | Y | 64.67 | 1570.7450 | 13 | 0.4 | 786.3801 | 2 | 81.33 | 1 | 53458 | BuCaMH03.raw | 2.2177E5 | 1 | 1 | 61 | 73 |  |  | PEAKS DB |
| total 1 peptides |  |  |  |  |  |  |  |  |  |  |  |  |  |  |  |  |  |  |  |
| --- | --- | --- | --- | --- | --- | --- | --- | --- | --- | --- | --- | --- | --- | --- | --- | --- | --- | --- | --- |

XP\_026529644.1

back to list

  

| Protein Coverage
| Supporting Peptides
|

Protein Coverage:

Supporting Peptides:

| Peptide | Uniq | -10lgP | Mass | Length | ppm | m/z | z | RT | Fraction | Scan | Source File | Area BuCaMH03\_B17 | #Feature | #Feature BuCaMH03\_B17 | Start | End | PTM | AScore | Found By |
| --- | --- | --- | --- | --- | --- | --- | --- | --- | --- | --- | --- | --- | --- | --- | --- | --- | --- | --- | --- |
| M.SIMNSFVNDIFER.I | Y | 64.67 | 1570.7450 | 13 | 0.4 | 786.3801 | 2 | 81.33 | 1 | 53458 | BuCaMH03.raw | 2.2177E5 | 1 | 1 | 61 | 73 |  |  | PEAKS DB |
| total 1 peptides |  |  |  |  |  |  |  |  |  |  |  |  |  |  |  |  |  |  |  |
| --- | --- | --- | --- | --- | --- | --- | --- | --- | --- | --- | --- | --- | --- | --- | --- | --- | --- | --- | --- |

XP\_025022125.1

back to list

  

| Protein Coverage
| Supporting Peptides
|

Protein Coverage:

Supporting Peptides:

| Peptide | Uniq | -10lgP | Mass | Length | ppm | m/z | z | RT | Fraction | Scan | Source File | Area BuCaMH03\_B17 | #Feature | #Feature BuCaMH03\_B17 | Start | End | PTM | AScore | Found By |
| --- | --- | --- | --- | --- | --- | --- | --- | --- | --- | --- | --- | --- | --- | --- | --- | --- | --- | --- | --- |
| M.SIMNSFVNDIFER.I | Y | 64.67 | 1570.7450 | 13 | 0.4 | 786.3801 | 2 | 81.33 | 1 | 53458 | BuCaMH03.raw | 2.2177E5 | 1 | 1 | 61 | 73 |  |  | PEAKS DB |
| total 1 peptides |  |  |  |  |  |  |  |  |  |  |  |  |  |  |  |  |  |  |  |
| --- | --- | --- | --- | --- | --- | --- | --- | --- | --- | --- | --- | --- | --- | --- | --- | --- | --- | --- | --- |

XP\_026576334.1

back to list

  

| Protein Coverage
| Supporting Peptides
|

Protein Coverage:

Supporting Peptides:

| Peptide | Uniq | -10lgP | Mass | Length | ppm | m/z | z | RT | Fraction | Scan | Source File | Area BuCaMH03\_B17 | #Feature | #Feature BuCaMH03\_B17 | Start | End | PTM | AScore | Found By |
| --- | --- | --- | --- | --- | --- | --- | --- | --- | --- | --- | --- | --- | --- | --- | --- | --- | --- | --- | --- |
| M.SIMNSFVNDIFER.I | Y | 64.67 | 1570.7450 | 13 | 0.4 | 786.3801 | 2 | 81.33 | 1 | 53458 | BuCaMH03.raw | 2.2177E5 | 1 | 1 | 61 | 73 |  |  | PEAKS DB |
| total 1 peptides |  |  |  |  |  |  |  |  |  |  |  |  |  |  |  |  |  |  |  |
| --- | --- | --- | --- | --- | --- | --- | --- | --- | --- | --- | --- | --- | --- | --- | --- | --- | --- | --- | --- |

XP\_026576366.1

back to list

  

| Protein Coverage
| Supporting Peptides
|

Protein Coverage:

Supporting Peptides:

| Peptide | Uniq | -10lgP | Mass | Length | ppm | m/z | z | RT | Fraction | Scan | Source File | Area BuCaMH03\_B17 | #Feature | #Feature BuCaMH03\_B17 | Start | End | PTM | AScore | Found By |
| --- | --- | --- | --- | --- | --- | --- | --- | --- | --- | --- | --- | --- | --- | --- | --- | --- | --- | --- | --- |
| M.SIMNSFVNDIFER.I | Y | 64.67 | 1570.7450 | 13 | 0.4 | 786.3801 | 2 | 81.33 | 1 | 53458 | BuCaMH03.raw | 2.2177E5 | 1 | 1 | 61 | 73 |  |  | PEAKS DB |
| total 1 peptides |  |  |  |  |  |  |  |  |  |  |  |  |  |  |  |  |  |  |  |
| --- | --- | --- | --- | --- | --- | --- | --- | --- | --- | --- | --- | --- | --- | --- | --- | --- | --- | --- | --- |

XP\_015672062.1

back to list

  

| Protein Coverage
| Supporting Peptides
|

Protein Coverage:

Supporting Peptides:

| Peptide | Uniq | -10lgP | Mass | Length | ppm | m/z | z | RT | Fraction | Scan | Source File | Area BuCaMH03\_B17 | #Feature | #Feature BuCaMH03\_B17 | Start | End | PTM | AScore | Found By |
| --- | --- | --- | --- | --- | --- | --- | --- | --- | --- | --- | --- | --- | --- | --- | --- | --- | --- | --- | --- |
| M.SIMNSFVNDIFER.I | Y | 64.67 | 1570.7450 | 13 | 0.4 | 786.3801 | 2 | 81.33 | 1 | 53458 | BuCaMH03.raw | 2.2177E5 | 1 | 1 | 61 | 73 |  |  | PEAKS DB |
| total 1 peptides |  |  |  |  |  |  |  |  |  |  |  |  |  |  |  |  |  |  |  |
| --- | --- | --- | --- | --- | --- | --- | --- | --- | --- | --- | --- | --- | --- | --- | --- | --- | --- | --- | --- |

XP\_026576365.1

back to list

  

| Protein Coverage
| Supporting Peptides
|

Protein Coverage:

Supporting Peptides:

| Peptide | Uniq | -10lgP | Mass | Length | ppm | m/z | z | RT | Fraction | Scan | Source File | Area BuCaMH03\_B17 | #Feature | #Feature BuCaMH03\_B17 | Start | End | PTM | AScore | Found By |
| --- | --- | --- | --- | --- | --- | --- | --- | --- | --- | --- | --- | --- | --- | --- | --- | --- | --- | --- | --- |
| M.SIMNSFVNDIFER.I | Y | 64.67 | 1570.7450 | 13 | 0.4 | 786.3801 | 2 | 81.33 | 1 | 53458 | BuCaMH03.raw | 2.2177E5 | 1 | 1 | 61 | 73 |  |  | PEAKS DB |
| total 1 peptides |  |  |  |  |  |  |  |  |  |  |  |  |  |  |  |  |  |  |  |
| --- | --- | --- | --- | --- | --- | --- | --- | --- | --- | --- | --- | --- | --- | --- | --- | --- | --- | --- | --- |

XP\_026536969.1

back to list

  

| Protein Coverage
| Supporting Peptides
|

Protein Coverage:

Supporting Peptides:

| Peptide | Uniq | -10lgP | Mass | Length | ppm | m/z | z | RT | Fraction | Scan | Source File | Area BuCaMH03\_B17 | #Feature | #Feature BuCaMH03\_B17 | Start | End | PTM | AScore | Found By |
| --- | --- | --- | --- | --- | --- | --- | --- | --- | --- | --- | --- | --- | --- | --- | --- | --- | --- | --- | --- |
| M.SIMNSFVNDIFER.I | Y | 64.67 | 1570.7450 | 13 | 0.4 | 786.3801 | 2 | 81.33 | 1 | 53458 | BuCaMH03.raw | 2.2177E5 | 1 | 1 | 61 | 73 |  |  | PEAKS DB |
| total 1 peptides |  |  |  |  |  |  |  |  |  |  |  |  |  |  |  |  |  |  |  |
| --- | --- | --- | --- | --- | --- | --- | --- | --- | --- | --- | --- | --- | --- | --- | --- | --- | --- | --- | --- |

XP\_007429385.1

back to list

  

| Protein Coverage
| Supporting Peptides
|

Protein Coverage:

Supporting Peptides:

| Peptide | Uniq | -10lgP | Mass | Length | ppm | m/z | z | RT | Fraction | Scan | Source File | Area BuCaMH03\_B17 | #Feature | #Feature BuCaMH03\_B17 | Start | End | PTM | AScore | Found By |
| --- | --- | --- | --- | --- | --- | --- | --- | --- | --- | --- | --- | --- | --- | --- | --- | --- | --- | --- | --- |
| M.SIMNSFVNDIFER.I | Y | 64.67 | 1570.7450 | 13 | 0.4 | 786.3801 | 2 | 81.33 | 1 | 53458 | BuCaMH03.raw | 2.2177E5 | 1 | 1 | 61 | 73 |  |  | PEAKS DB |
| total 1 peptides |  |  |  |  |  |  |  |  |  |  |  |  |  |  |  |  |  |  |  |
| --- | --- | --- | --- | --- | --- | --- | --- | --- | --- | --- | --- | --- | --- | --- | --- | --- | --- | --- | --- |

XP\_026545426.1

back to list

  

| Protein Coverage
| Supporting Peptides
|

Protein Coverage:

Supporting Peptides:

| Peptide | Uniq | -10lgP | Mass | Length | ppm | m/z | z | RT | Fraction | Scan | Source File | Area BuCaMH03\_B17 | #Feature | #Feature BuCaMH03\_B17 | Start | End | PTM | AScore | Found By |
| --- | --- | --- | --- | --- | --- | --- | --- | --- | --- | --- | --- | --- | --- | --- | --- | --- | --- | --- | --- |
| M.SIMNSFVNDIFER.I | Y | 64.67 | 1570.7450 | 13 | 0.4 | 786.3801 | 2 | 81.33 | 1 | 53458 | BuCaMH03.raw | 2.2177E5 | 1 | 1 | 61 | 73 |  |  | PEAKS DB |
| total 1 peptides |  |  |  |  |  |  |  |  |  |  |  |  |  |  |  |  |  |  |  |
| --- | --- | --- | --- | --- | --- | --- | --- | --- | --- | --- | --- | --- | --- | --- | --- | --- | --- | --- | --- |

XP\_015681932.1

back to list

  

| Protein Coverage
| Supporting Peptides
|

Protein Coverage:

Supporting Peptides:

| Peptide | Uniq | -10lgP | Mass | Length | ppm | m/z | z | RT | Fraction | Scan | Source File | Area BuCaMH03\_B17 | #Feature | #Feature BuCaMH03\_B17 | Start | End | PTM | AScore | Found By |
| --- | --- | --- | --- | --- | --- | --- | --- | --- | --- | --- | --- | --- | --- | --- | --- | --- | --- | --- | --- |
| M.SIMNSFVNDIFER.I | Y | 64.67 | 1570.7450 | 13 | 0.4 | 786.3801 | 2 | 81.33 | 1 | 53458 | BuCaMH03.raw | 2.2177E5 | 1 | 1 | 61 | 73 |  |  | PEAKS DB |
| total 1 peptides |  |  |  |  |  |  |  |  |  |  |  |  |  |  |  |  |  |  |  |
| --- | --- | --- | --- | --- | --- | --- | --- | --- | --- | --- | --- | --- | --- | --- | --- | --- | --- | --- | --- |

XP\_026547833.1

back to list

  

| Protein Coverage
| Supporting Peptides
|

Protein Coverage:

Supporting Peptides:

| Peptide | Uniq | -10lgP | Mass | Length | ppm | m/z | z | RT | Fraction | Scan | Source File | Area BuCaMH03\_B17 | #Feature | #Feature BuCaMH03\_B17 | Start | End | PTM | AScore | Found By |
| --- | --- | --- | --- | --- | --- | --- | --- | --- | --- | --- | --- | --- | --- | --- | --- | --- | --- | --- | --- |
| M.SIMNSFVNDIFER.I | Y | 64.67 | 1570.7450 | 13 | 0.4 | 786.3801 | 2 | 81.33 | 1 | 53458 | BuCaMH03.raw | 2.2177E5 | 1 | 1 | 61 | 73 |  |  | PEAKS DB |
| total 1 peptides |  |  |  |  |  |  |  |  |  |  |  |  |  |  |  |  |  |  |  |
| --- | --- | --- | --- | --- | --- | --- | --- | --- | --- | --- | --- | --- | --- | --- | --- | --- | --- | --- | --- |

XP\_026539701.1

back to list

  

| Protein Coverage
| Supporting Peptides
|

Protein Coverage:

Supporting Peptides:

| Peptide | Uniq | -10lgP | Mass | Length | ppm | m/z | z | RT | Fraction | Scan | Source File | Area BuCaMH03\_B17 | #Feature | #Feature BuCaMH03\_B17 | Start | End | PTM | AScore | Found By |
| --- | --- | --- | --- | --- | --- | --- | --- | --- | --- | --- | --- | --- | --- | --- | --- | --- | --- | --- | --- |
| M.SIMNSFVNDIFER.I | Y | 64.67 | 1570.7450 | 13 | 0.4 | 786.3801 | 2 | 81.33 | 1 | 53458 | BuCaMH03.raw | 2.2177E5 | 1 | 1 | 61 | 73 |  |  | PEAKS DB |
| total 1 peptides |  |  |  |  |  |  |  |  |  |  |  |  |  |  |  |  |  |  |  |
| --- | --- | --- | --- | --- | --- | --- | --- | --- | --- | --- | --- | --- | --- | --- | --- | --- | --- | --- | --- |

XP\_026580816.1

back to list

  

| Protein Coverage
| Supporting Peptides
|

Protein Coverage:

Supporting Peptides:

| Peptide | Uniq | -10lgP | Mass | Length | ppm | m/z | z | RT | Fraction | Scan | Source File | Area BuCaMH03\_B17 | #Feature | #Feature BuCaMH03\_B17 | Start | End | PTM | AScore | Found By |
| --- | --- | --- | --- | --- | --- | --- | --- | --- | --- | --- | --- | --- | --- | --- | --- | --- | --- | --- | --- |
| M.SIMNSFVNDIFER.I | Y | 64.67 | 1570.7450 | 13 | 0.4 | 786.3801 | 2 | 81.33 | 1 | 53458 | BuCaMH03.raw | 2.2177E5 | 1 | 1 | 61 | 73 |  |  | PEAKS DB |
| total 1 peptides |  |  |  |  |  |  |  |  |  |  |  |  |  |  |  |  |  |  |  |
| --- | --- | --- | --- | --- | --- | --- | --- | --- | --- | --- | --- | --- | --- | --- | --- | --- | --- | --- | --- |

XP\_015681933.1

back to list

  

| Protein Coverage
| Supporting Peptides
|

Protein Coverage:

Supporting Peptides:

| Peptide | Uniq | -10lgP | Mass | Length | ppm | m/z | z | RT | Fraction | Scan | Source File | Area BuCaMH03\_B17 | #Feature | #Feature BuCaMH03\_B17 | Start | End | PTM | AScore | Found By |
| --- | --- | --- | --- | --- | --- | --- | --- | --- | --- | --- | --- | --- | --- | --- | --- | --- | --- | --- | --- |
| M.SIMNSFVNDIFER.I | Y | 64.67 | 1570.7450 | 13 | 0.4 | 786.3801 | 2 | 81.33 | 1 | 53458 | BuCaMH03.raw | 2.2177E5 | 1 | 1 | 61 | 73 |  |  | PEAKS DB |
| total 1 peptides |  |  |  |  |  |  |  |  |  |  |  |  |  |  |  |  |  |  |  |
| --- | --- | --- | --- | --- | --- | --- | --- | --- | --- | --- | --- | --- | --- | --- | --- | --- | --- | --- | --- |

XP\_026548170.1

back to list

  

| Protein Coverage
| Supporting Peptides
|

Protein Coverage:

Supporting Peptides:

| Peptide | Uniq | -10lgP | Mass | Length | ppm | m/z | z | RT | Fraction | Scan | Source File | Area BuCaMH03\_B17 | #Feature | #Feature BuCaMH03\_B17 | Start | End | PTM | AScore | Found By |
| --- | --- | --- | --- | --- | --- | --- | --- | --- | --- | --- | --- | --- | --- | --- | --- | --- | --- | --- | --- |
| M.SIMNSFVNDIFER.I | Y | 64.67 | 1570.7450 | 13 | 0.4 | 786.3801 | 2 | 81.33 | 1 | 53458 | BuCaMH03.raw | 2.2177E5 | 1 | 1 | 61 | 73 |  |  | PEAKS DB |
| total 1 peptides |  |  |  |  |  |  |  |  |  |  |  |  |  |  |  |  |  |  |  |
| --- | --- | --- | --- | --- | --- | --- | --- | --- | --- | --- | --- | --- | --- | --- | --- | --- | --- | --- | --- |

XP\_026569640.1

back to list

  

| Protein Coverage
| Supporting Peptides
|

Protein Coverage:

Supporting Peptides:

| Peptide | Uniq | -10lgP | Mass | Length | ppm | m/z | z | RT | Fraction | Scan | Source File | Area BuCaMH03\_B17 | #Feature | #Feature BuCaMH03\_B17 | Start | End | PTM | AScore | Found By |
| --- | --- | --- | --- | --- | --- | --- | --- | --- | --- | --- | --- | --- | --- | --- | --- | --- | --- | --- | --- |
| M.SIMNSFVNDIFER.I | Y | 64.67 | 1570.7450 | 13 | 0.4 | 786.3801 | 2 | 81.33 | 1 | 53458 | BuCaMH03.raw | 2.2177E5 | 1 | 1 | 61 | 73 |  |  | PEAKS DB |
| total 1 peptides |  |  |  |  |  |  |  |  |  |  |  |  |  |  |  |  |  |  |  |
| --- | --- | --- | --- | --- | --- | --- | --- | --- | --- | --- | --- | --- | --- | --- | --- | --- | --- | --- | --- |

ETE56555.1

back to list

  

| Protein Coverage
| Supporting Peptides
|

Protein Coverage:

Supporting Peptides:

| Peptide | Uniq | -10lgP | Mass | Length | ppm | m/z | z | RT | Fraction | Scan | Source File | Area BuCaMH03\_B17 | #Feature | #Feature BuCaMH03\_B17 | Start | End | PTM | AScore | Found By |
| --- | --- | --- | --- | --- | --- | --- | --- | --- | --- | --- | --- | --- | --- | --- | --- | --- | --- | --- | --- |
| M.SIMNSFVNDIFER.I | Y | 64.67 | 1570.7450 | 13 | 0.4 | 786.3801 | 2 | 81.33 | 1 | 53458 | BuCaMH03.raw | 2.2177E5 | 1 | 1 | 61 | 73 |  |  | PEAKS DB |
| total 1 peptides |  |  |  |  |  |  |  |  |  |  |  |  |  |  |  |  |  |  |  |
| --- | --- | --- | --- | --- | --- | --- | --- | --- | --- | --- | --- | --- | --- | --- | --- | --- | --- | --- | --- |

XP\_015678747.1

back to list

  

| Protein Coverage
| Supporting Peptides
|

Protein Coverage:

Supporting Peptides:

| Peptide | Uniq | -10lgP | Mass | Length | ppm | m/z | z | RT | Fraction | Scan | Source File | Area BuCaMH03\_B17 | #Feature | #Feature BuCaMH03\_B17 | Start | End | PTM | AScore | Found By |
| --- | --- | --- | --- | --- | --- | --- | --- | --- | --- | --- | --- | --- | --- | --- | --- | --- | --- | --- | --- |
| M.SIMNSFVNDIFER.I | Y | 64.67 | 1570.7450 | 13 | 0.4 | 786.3801 | 2 | 81.33 | 1 | 53458 | BuCaMH03.raw | 2.2177E5 | 1 | 1 | 61 | 73 |  |  | PEAKS DB |
| total 1 peptides |  |  |  |  |  |  |  |  |  |  |  |  |  |  |  |  |  |  |  |
| --- | --- | --- | --- | --- | --- | --- | --- | --- | --- | --- | --- | --- | --- | --- | --- | --- | --- | --- | --- |

XP\_007434930.1

back to list

  

| Protein Coverage
| Supporting Peptides
|

Protein Coverage:

Supporting Peptides:

| Peptide | Uniq | -10lgP | Mass | Length | ppm | m/z | z | RT | Fraction | Scan | Source File | Area BuCaMH03\_B17 | #Feature | #Feature BuCaMH03\_B17 | Start | End | PTM | AScore | Found By |
| --- | --- | --- | --- | --- | --- | --- | --- | --- | --- | --- | --- | --- | --- | --- | --- | --- | --- | --- | --- |
| K.AAADAHVGFC(+57.02)K.A | Y | 62.11 | 1145.5287 | 11 | 0.4 | 573.7719 | 2 | 12.81 | 1 | 2030 | BuCaMH03.raw | 3.3371E5 | 2 | 2 | 220 | 230 | Carbamidomethylation | C10:Carbamidomethylation:1000.00 | PEAKS DB |
| total 1 peptides |  |  |  |  |  |  |  |  |  |  |  |  |  |  |  |  |  |  |  |
| --- | --- | --- | --- | --- | --- | --- | --- | --- | --- | --- | --- | --- | --- | --- | --- | --- | --- | --- | --- |

XP\_034281839.1

back to list

  

| Protein Coverage
| Supporting Peptides
|

Protein Coverage:

Supporting Peptides:

| Peptide | Uniq | -10lgP | Mass | Length | ppm | m/z | z | RT | Fraction | Scan | Source File | Area BuCaMH03\_B17 | #Feature | #Feature BuCaMH03\_B17 | Start | End | PTM | AScore | Found By |
| --- | --- | --- | --- | --- | --- | --- | --- | --- | --- | --- | --- | --- | --- | --- | --- | --- | --- | --- | --- |
| R.FPSLLAPGR.R | Y | 59.37 | 956.5443 | 9 | -0.5 | 479.2792 | 2 | 53.64 | 1 | 30634 | BuCaMH03.raw | 3.2482E5 | 1 | 1 | 157 | 165 |  |  | PEAKS DB |
| total 1 peptides |  |  |  |  |  |  |  |  |  |  |  |  |  |  |  |  |  |  |  |
| --- | --- | --- | --- | --- | --- | --- | --- | --- | --- | --- | --- | --- | --- | --- | --- | --- | --- | --- | --- |

XP\_013925207.1

back to list

  

| Protein Coverage
| Supporting Peptides
|

Protein Coverage:

Supporting Peptides:

| Peptide | Uniq | -10lgP | Mass | Length | ppm | m/z | z | RT | Fraction | Scan | Source File | Area BuCaMH03\_B17 | #Feature | #Feature BuCaMH03\_B17 | Start | End | PTM | AScore | Found By |
| --- | --- | --- | --- | --- | --- | --- | --- | --- | --- | --- | --- | --- | --- | --- | --- | --- | --- | --- | --- |
| R.FPSLLAPGR.R | Y | 59.37 | 956.5443 | 9 | -0.5 | 479.2792 | 2 | 53.64 | 1 | 30634 | BuCaMH03.raw | 3.2482E5 | 1 | 1 | 155 | 163 |  |  | PEAKS DB |
| total 1 peptides |  |  |  |  |  |  |  |  |  |  |  |  |  |  |  |  |  |  |  |
| --- | --- | --- | --- | --- | --- | --- | --- | --- | --- | --- | --- | --- | --- | --- | --- | --- | --- | --- | --- |

XP\_026542555.1

back to list

  

| Protein Coverage
| Supporting Peptides
|

Protein Coverage:

Supporting Peptides:

| Peptide | Uniq | -10lgP | Mass | Length | ppm | m/z | z | RT | Fraction | Scan | Source File | Area BuCaMH03\_B17 | #Feature | #Feature BuCaMH03\_B17 | Start | End | PTM | AScore | Found By |
| --- | --- | --- | --- | --- | --- | --- | --- | --- | --- | --- | --- | --- | --- | --- | --- | --- | --- | --- | --- |
| R.FPSLLAPGR.R | Y | 59.37 | 956.5443 | 9 | -0.5 | 479.2792 | 2 | 53.64 | 1 | 30634 | BuCaMH03.raw | 3.2482E5 | 1 | 1 | 152 | 160 |  |  | PEAKS DB |
| total 1 peptides |  |  |  |  |  |  |  |  |  |  |  |  |  |  |  |  |  |  |  |
| --- | --- | --- | --- | --- | --- | --- | --- | --- | --- | --- | --- | --- | --- | --- | --- | --- | --- | --- | --- |

XP\_026575692.1

back to list

  

| Protein Coverage
| Supporting Peptides
|

Protein Coverage:

Supporting Peptides:

| Peptide | Uniq | -10lgP | Mass | Length | ppm | m/z | z | RT | Fraction | Scan | Source File | Area BuCaMH03\_B17 | #Feature | #Feature BuCaMH03\_B17 | Start | End | PTM | AScore | Found By |
| --- | --- | --- | --- | --- | --- | --- | --- | --- | --- | --- | --- | --- | --- | --- | --- | --- | --- | --- | --- |
| R.FPSLLAPGR.R | Y | 59.37 | 956.5443 | 9 | -0.5 | 479.2792 | 2 | 53.64 | 1 | 30634 | BuCaMH03.raw | 3.2482E5 | 1 | 1 | 155 | 163 |  |  | PEAKS DB |
| total 1 peptides |  |  |  |  |  |  |  |  |  |  |  |  |  |  |  |  |  |  |  |
| --- | --- | --- | --- | --- | --- | --- | --- | --- | --- | --- | --- | --- | --- | --- | --- | --- | --- | --- | --- |

XP\_026542553.1

back to list

  

| Protein Coverage
| Supporting Peptides
|

Protein Coverage:

Supporting Peptides:

| Peptide | Uniq | -10lgP | Mass | Length | ppm | m/z | z | RT | Fraction | Scan | Source File | Area BuCaMH03\_B17 | #Feature | #Feature BuCaMH03\_B17 | Start | End | PTM | AScore | Found By |
| --- | --- | --- | --- | --- | --- | --- | --- | --- | --- | --- | --- | --- | --- | --- | --- | --- | --- | --- | --- |
| R.FPSLLAPGR.R | Y | 59.37 | 956.5443 | 9 | -0.5 | 479.2792 | 2 | 53.64 | 1 | 30634 | BuCaMH03.raw | 3.2482E5 | 1 | 1 | 152 | 160 |  |  | PEAKS DB |
| total 1 peptides |  |  |  |  |  |  |  |  |  |  |  |  |  |  |  |  |  |  |  |
| --- | --- | --- | --- | --- | --- | --- | --- | --- | --- | --- | --- | --- | --- | --- | --- | --- | --- | --- | --- |

XP\_029139434.1

back to list

  

| Protein Coverage
| Supporting Peptides
|

Protein Coverage:

Supporting Peptides:

| Peptide | Uniq | -10lgP | Mass | Length | ppm | m/z | z | RT | Fraction | Scan | Source File | Area BuCaMH03\_B17 | #Feature | #Feature BuCaMH03\_B17 | Start | End | PTM | AScore | Found By |
| --- | --- | --- | --- | --- | --- | --- | --- | --- | --- | --- | --- | --- | --- | --- | --- | --- | --- | --- | --- |
| R.FPSLLAPGR.R | Y | 59.37 | 956.5443 | 9 | -0.5 | 479.2792 | 2 | 53.64 | 1 | 30634 | BuCaMH03.raw | 3.2482E5 | 1 | 1 | 154 | 162 |  |  | PEAKS DB |
| total 1 peptides |  |  |  |  |  |  |  |  |  |  |  |  |  |  |  |  |  |  |  |
| --- | --- | --- | --- | --- | --- | --- | --- | --- | --- | --- | --- | --- | --- | --- | --- | --- | --- | --- | --- |

XP\_026575691.1

back to list

  

| Protein Coverage
| Supporting Peptides
|

Protein Coverage:

Supporting Peptides:

| Peptide | Uniq | -10lgP | Mass | Length | ppm | m/z | z | RT | Fraction | Scan | Source File | Area BuCaMH03\_B17 | #Feature | #Feature BuCaMH03\_B17 | Start | End | PTM | AScore | Found By |
| --- | --- | --- | --- | --- | --- | --- | --- | --- | --- | --- | --- | --- | --- | --- | --- | --- | --- | --- | --- |
| R.FPSLLAPGR.R | Y | 59.37 | 956.5443 | 9 | -0.5 | 479.2792 | 2 | 53.64 | 1 | 30634 | BuCaMH03.raw | 3.2482E5 | 1 | 1 | 155 | 163 |  |  | PEAKS DB |
| total 1 peptides |  |  |  |  |  |  |  |  |  |  |  |  |  |  |  |  |  |  |  |
| --- | --- | --- | --- | --- | --- | --- | --- | --- | --- | --- | --- | --- | --- | --- | --- | --- | --- | --- | --- |

XP\_032087784.1

back to list

  

| Protein Coverage
| Supporting Peptides
|

Protein Coverage:

Supporting Peptides:

| Peptide | Uniq | -10lgP | Mass | Length | ppm | m/z | z | RT | Fraction | Scan | Source File | Area BuCaMH03\_B17 | #Feature | #Feature BuCaMH03\_B17 | Start | End | PTM | AScore | Found By |
| --- | --- | --- | --- | --- | --- | --- | --- | --- | --- | --- | --- | --- | --- | --- | --- | --- | --- | --- | --- |
| R.FPSLLAPGR.R | Y | 59.37 | 956.5443 | 9 | -0.5 | 479.2792 | 2 | 53.64 | 1 | 30634 | BuCaMH03.raw | 3.2482E5 | 1 | 1 | 160 | 168 |  |  | PEAKS DB |
| total 1 peptides |  |  |  |  |  |  |  |  |  |  |  |  |  |  |  |  |  |  |  |
| --- | --- | --- | --- | --- | --- | --- | --- | --- | --- | --- | --- | --- | --- | --- | --- | --- | --- | --- | --- |

XP\_015746961.1

back to list

  

| Protein Coverage
| Supporting Peptides
|

Protein Coverage:

Supporting Peptides:

| Peptide | Uniq | -10lgP | Mass | Length | ppm | m/z | z | RT | Fraction | Scan | Source File | Area BuCaMH03\_B17 | #Feature | #Feature BuCaMH03\_B17 | Start | End | PTM | AScore | Found By |
| --- | --- | --- | --- | --- | --- | --- | --- | --- | --- | --- | --- | --- | --- | --- | --- | --- | --- | --- | --- |
| R.FPSLLAPGR.R | Y | 59.37 | 956.5443 | 9 | -0.5 | 479.2792 | 2 | 53.64 | 1 | 30634 | BuCaMH03.raw | 3.2482E5 | 1 | 1 | 12 | 20 |  |  | PEAKS DB |
| total 1 peptides |  |  |  |  |  |  |  |  |  |  |  |  |  |  |  |  |  |  |  |
| --- | --- | --- | --- | --- | --- | --- | --- | --- | --- | --- | --- | --- | --- | --- | --- | --- | --- | --- | --- |

JAB52761.1

back to list

  

| Protein Coverage
| Supporting Peptides
|

Protein Coverage:

Supporting Peptides:

| Peptide | Uniq | -10lgP | Mass | Length | ppm | m/z | z | RT | Fraction | Scan | Source File | Area BuCaMH03\_B17 | #Feature | #Feature BuCaMH03\_B17 | Start | End | PTM | AScore | Found By |
| --- | --- | --- | --- | --- | --- | --- | --- | --- | --- | --- | --- | --- | --- | --- | --- | --- | --- | --- | --- |
| R.TAALC(+57.02)FGK.A | Y | 55.04 | 866.4320 | 8 | -0.2 | 434.2232 | 2 | 30.55 | 1 | 12370 | BuCaMH03.raw | 4.7538E5 | 1 | 1 | 121 | 128 | Carbamidomethylation | C5:Carbamidomethylation:1000.00 | PEAKS DB |
| total 1 peptides |  |  |  |  |  |  |  |  |  |  |  |  |  |  |  |  |  |  |  |
| --- | --- | --- | --- | --- | --- | --- | --- | --- | --- | --- | --- | --- | --- | --- | --- | --- | --- | --- | --- |

JAS04986.1

back to list

  

| Protein Coverage
| Supporting Peptides
|

Protein Coverage:

Supporting Peptides:

| Peptide | Uniq | -10lgP | Mass | Length | ppm | m/z | z | RT | Fraction | Scan | Source File | Area BuCaMH03\_B17 | #Feature | #Feature BuCaMH03\_B17 | Start | End | PTM | AScore | Found By |
| --- | --- | --- | --- | --- | --- | --- | --- | --- | --- | --- | --- | --- | --- | --- | --- | --- | --- | --- | --- |
| R.TAALC(+57.02)FGK.A | Y | 55.04 | 866.4320 | 8 | -0.2 | 434.2232 | 2 | 30.55 | 1 | 12370 | BuCaMH03.raw | 4.7538E5 | 1 | 1 | 123 | 130 | Carbamidomethylation | C5:Carbamidomethylation:1000.00 | PEAKS DB |
| total 1 peptides |  |  |  |  |  |  |  |  |  |  |  |  |  |  |  |  |  |  |  |
| --- | --- | --- | --- | --- | --- | --- | --- | --- | --- | --- | --- | --- | --- | --- | --- | --- | --- | --- | --- |

JAS04987.1

back to list

  

| Protein Coverage
| Supporting Peptides
|

Protein Coverage:

Supporting Peptides:

| Peptide | Uniq | -10lgP | Mass | Length | ppm | m/z | z | RT | Fraction | Scan | Source File | Area BuCaMH03\_B17 | #Feature | #Feature BuCaMH03\_B17 | Start | End | PTM | AScore | Found By |
| --- | --- | --- | --- | --- | --- | --- | --- | --- | --- | --- | --- | --- | --- | --- | --- | --- | --- | --- | --- |
| R.TAALC(+57.02)FGK.A | Y | 55.04 | 866.4320 | 8 | -0.2 | 434.2232 | 2 | 30.55 | 1 | 12370 | BuCaMH03.raw | 4.7538E5 | 1 | 1 | 123 | 130 | Carbamidomethylation | C5:Carbamidomethylation:1000.00 | PEAKS DB |
| total 1 peptides |  |  |  |  |  |  |  |  |  |  |  |  |  |  |  |  |  |  |  |
| --- | --- | --- | --- | --- | --- | --- | --- | --- | --- | --- | --- | --- | --- | --- | --- | --- | --- | --- | --- |

JAS04982.1

back to list

  

| Protein Coverage
| Supporting Peptides
|

Protein Coverage:

Supporting Peptides:

| Peptide | Uniq | -10lgP | Mass | Length | ppm | m/z | z | RT | Fraction | Scan | Source File | Area BuCaMH03\_B17 | #Feature | #Feature BuCaMH03\_B17 | Start | End | PTM | AScore | Found By |
| --- | --- | --- | --- | --- | --- | --- | --- | --- | --- | --- | --- | --- | --- | --- | --- | --- | --- | --- | --- |
| R.TAALC(+57.02)FGK.A | Y | 55.04 | 866.4320 | 8 | -0.2 | 434.2232 | 2 | 30.55 | 1 | 12370 | BuCaMH03.raw | 4.7538E5 | 1 | 1 | 123 | 130 | Carbamidomethylation | C5:Carbamidomethylation:1000.00 | PEAKS DB |
| total 1 peptides |  |  |  |  |  |  |  |  |  |  |  |  |  |  |  |  |  |  |  |
| --- | --- | --- | --- | --- | --- | --- | --- | --- | --- | --- | --- | --- | --- | --- | --- | --- | --- | --- | --- |

JAS04985.1

back to list

  

| Protein Coverage
| Supporting Peptides
|

Protein Coverage:

Supporting Peptides:

| Peptide | Uniq | -10lgP | Mass | Length | ppm | m/z | z | RT | Fraction | Scan | Source File | Area BuCaMH03\_B17 | #Feature | #Feature BuCaMH03\_B17 | Start | End | PTM | AScore | Found By |
| --- | --- | --- | --- | --- | --- | --- | --- | --- | --- | --- | --- | --- | --- | --- | --- | --- | --- | --- | --- |
| R.TAALC(+57.02)FGK.A | Y | 55.04 | 866.4320 | 8 | -0.2 | 434.2232 | 2 | 30.55 | 1 | 12370 | BuCaMH03.raw | 4.7538E5 | 1 | 1 | 123 | 130 | Carbamidomethylation | C5:Carbamidomethylation:1000.00 | PEAKS DB |
| total 1 peptides |  |  |  |  |  |  |  |  |  |  |  |  |  |  |  |  |  |  |  |
| --- | --- | --- | --- | --- | --- | --- | --- | --- | --- | --- | --- | --- | --- | --- | --- | --- | --- | --- | --- |

JAI08996.1

back to list

  

| Protein Coverage
| Supporting Peptides
|

Protein Coverage:

Supporting Peptides:

| Peptide | Uniq | -10lgP | Mass | Length | ppm | m/z | z | RT | Fraction | Scan | Source File | Area BuCaMH03\_B17 | #Feature | #Feature BuCaMH03\_B17 | Start | End | PTM | AScore | Found By |
| --- | --- | --- | --- | --- | --- | --- | --- | --- | --- | --- | --- | --- | --- | --- | --- | --- | --- | --- | --- |
| R.TAALC(+57.02)FGK.A | Y | 55.04 | 866.4320 | 8 | -0.2 | 434.2232 | 2 | 30.55 | 1 | 12370 | BuCaMH03.raw | 4.7538E5 | 1 | 1 | 123 | 130 | Carbamidomethylation | C5:Carbamidomethylation:1000.00 | PEAKS DB |
| total 1 peptides |  |  |  |  |  |  |  |  |  |  |  |  |  |  |  |  |  |  |  |
| --- | --- | --- | --- | --- | --- | --- | --- | --- | --- | --- | --- | --- | --- | --- | --- | --- | --- | --- | --- |

JAI08997.1

back to list

  

| Protein Coverage
| Supporting Peptides
|

Protein Coverage:

Supporting Peptides:

| Peptide | Uniq | -10lgP | Mass | Length | ppm | m/z | z | RT | Fraction | Scan | Source File | Area BuCaMH03\_B17 | #Feature | #Feature BuCaMH03\_B17 | Start | End | PTM | AScore | Found By |
| --- | --- | --- | --- | --- | --- | --- | --- | --- | --- | --- | --- | --- | --- | --- | --- | --- | --- | --- | --- |
| R.TAALC(+57.02)FGK.A | Y | 55.04 | 866.4320 | 8 | -0.2 | 434.2232 | 2 | 30.55 | 1 | 12370 | BuCaMH03.raw | 4.7538E5 | 1 | 1 | 123 | 130 | Carbamidomethylation | C5:Carbamidomethylation:1000.00 | PEAKS DB |
| total 1 peptides |  |  |  |  |  |  |  |  |  |  |  |  |  |  |  |  |  |  |  |
| --- | --- | --- | --- | --- | --- | --- | --- | --- | --- | --- | --- | --- | --- | --- | --- | --- | --- | --- | --- |

JAI08998.1

back to list

  

| Protein Coverage
| Supporting Peptides
|

Protein Coverage:

Supporting Peptides:

| Peptide | Uniq | -10lgP | Mass | Length | ppm | m/z | z | RT | Fraction | Scan | Source File | Area BuCaMH03\_B17 | #Feature | #Feature BuCaMH03\_B17 | Start | End | PTM | AScore | Found By |
| --- | --- | --- | --- | --- | --- | --- | --- | --- | --- | --- | --- | --- | --- | --- | --- | --- | --- | --- | --- |
| R.TAALC(+57.02)FGK.A | Y | 55.04 | 866.4320 | 8 | -0.2 | 434.2232 | 2 | 30.55 | 1 | 12370 | BuCaMH03.raw | 4.7538E5 | 1 | 1 | 123 | 130 | Carbamidomethylation | C5:Carbamidomethylation:1000.00 | PEAKS DB |
| total 1 peptides |  |  |  |  |  |  |  |  |  |  |  |  |  |  |  |  |  |  |  |
| --- | --- | --- | --- | --- | --- | --- | --- | --- | --- | --- | --- | --- | --- | --- | --- | --- | --- | --- | --- |

JAI08993.1

back to list

  

| Protein Coverage
| Supporting Peptides
|

Protein Coverage:

Supporting Peptides:

| Peptide | Uniq | -10lgP | Mass | Length | ppm | m/z | z | RT | Fraction | Scan | Source File | Area BuCaMH03\_B17 | #Feature | #Feature BuCaMH03\_B17 | Start | End | PTM | AScore | Found By |
| --- | --- | --- | --- | --- | --- | --- | --- | --- | --- | --- | --- | --- | --- | --- | --- | --- | --- | --- | --- |
| R.TAALC(+57.02)FGK.A | Y | 55.04 | 866.4320 | 8 | -0.2 | 434.2232 | 2 | 30.55 | 1 | 12370 | BuCaMH03.raw | 4.7538E5 | 1 | 1 | 123 | 130 | Carbamidomethylation | C5:Carbamidomethylation:1000.00 | PEAKS DB |
| total 1 peptides |  |  |  |  |  |  |  |  |  |  |  |  |  |  |  |  |  |  |  |
| --- | --- | --- | --- | --- | --- | --- | --- | --- | --- | --- | --- | --- | --- | --- | --- | --- | --- | --- | --- |

JAB52775.1

back to list

  

| Protein Coverage
| Supporting Peptides
|

Protein Coverage:

Supporting Peptides:

| Peptide | Uniq | -10lgP | Mass | Length | ppm | m/z | z | RT | Fraction | Scan | Source File | Area BuCaMH03\_B17 | #Feature | #Feature BuCaMH03\_B17 | Start | End | PTM | AScore | Found By |
| --- | --- | --- | --- | --- | --- | --- | --- | --- | --- | --- | --- | --- | --- | --- | --- | --- | --- | --- | --- |
| R.TAALC(+57.02)FGK.A | Y | 55.04 | 866.4320 | 8 | -0.2 | 434.2232 | 2 | 30.55 | 1 | 12370 | BuCaMH03.raw | 4.7538E5 | 1 | 1 | 123 | 130 | Carbamidomethylation | C5:Carbamidomethylation:1000.00 | PEAKS DB |
| total 1 peptides |  |  |  |  |  |  |  |  |  |  |  |  |  |  |  |  |  |  |  |
| --- | --- | --- | --- | --- | --- | --- | --- | --- | --- | --- | --- | --- | --- | --- | --- | --- | --- | --- | --- |

JAB52776.1

back to list

  

| Protein Coverage
| Supporting Peptides
|

Protein Coverage:

Supporting Peptides:

| Peptide | Uniq | -10lgP | Mass | Length | ppm | m/z | z | RT | Fraction | Scan | Source File | Area BuCaMH03\_B17 | #Feature | #Feature BuCaMH03\_B17 | Start | End | PTM | AScore | Found By |
| --- | --- | --- | --- | --- | --- | --- | --- | --- | --- | --- | --- | --- | --- | --- | --- | --- | --- | --- | --- |
| R.TAALC(+57.02)FGK.A | Y | 55.04 | 866.4320 | 8 | -0.2 | 434.2232 | 2 | 30.55 | 1 | 12370 | BuCaMH03.raw | 4.7538E5 | 1 | 1 | 123 | 130 | Carbamidomethylation | C5:Carbamidomethylation:1000.00 | PEAKS DB |
| total 1 peptides |  |  |  |  |  |  |  |  |  |  |  |  |  |  |  |  |  |  |  |
| --- | --- | --- | --- | --- | --- | --- | --- | --- | --- | --- | --- | --- | --- | --- | --- | --- | --- | --- | --- |

JAS05103.1

back to list

  

| Protein Coverage
| Supporting Peptides
|

Protein Coverage:

Supporting Peptides:

| Peptide | Uniq | -10lgP | Mass | Length | ppm | m/z | z | RT | Fraction | Scan | Source File | Area BuCaMH03\_B17 | #Feature | #Feature BuCaMH03\_B17 | Start | End | PTM | AScore | Found By |
| --- | --- | --- | --- | --- | --- | --- | --- | --- | --- | --- | --- | --- | --- | --- | --- | --- | --- | --- | --- |
| R.TAALC(+57.02)FGK.A | Y | 55.04 | 866.4320 | 8 | -0.2 | 434.2232 | 2 | 30.55 | 1 | 12370 | BuCaMH03.raw | 4.7538E5 | 1 | 1 | 123 | 130 | Carbamidomethylation | C5:Carbamidomethylation:1000.00 | PEAKS DB |
| total 1 peptides |  |  |  |  |  |  |  |  |  |  |  |  |  |  |  |  |  |  |  |
| --- | --- | --- | --- | --- | --- | --- | --- | --- | --- | --- | --- | --- | --- | --- | --- | --- | --- | --- | --- |

JAS05108.1

back to list

  

| Protein Coverage
| Supporting Peptides
|

Protein Coverage:

Supporting Peptides:

| Peptide | Uniq | -10lgP | Mass | Length | ppm | m/z | z | RT | Fraction | Scan | Source File | Area BuCaMH03\_B17 | #Feature | #Feature BuCaMH03\_B17 | Start | End | PTM | AScore | Found By |
| --- | --- | --- | --- | --- | --- | --- | --- | --- | --- | --- | --- | --- | --- | --- | --- | --- | --- | --- | --- |
| R.TAALC(+57.02)FGK.A | Y | 55.04 | 866.4320 | 8 | -0.2 | 434.2232 | 2 | 30.55 | 1 | 12370 | BuCaMH03.raw | 4.7538E5 | 1 | 1 | 123 | 130 | Carbamidomethylation | C5:Carbamidomethylation:1000.00 | PEAKS DB |
| total 1 peptides |  |  |  |  |  |  |  |  |  |  |  |  |  |  |  |  |  |  |  |
| --- | --- | --- | --- | --- | --- | --- | --- | --- | --- | --- | --- | --- | --- | --- | --- | --- | --- | --- | --- |

JAS05109.1

back to list

  

| Protein Coverage
| Supporting Peptides
|

Protein Coverage:

Supporting Peptides:

| Peptide | Uniq | -10lgP | Mass | Length | ppm | m/z | z | RT | Fraction | Scan | Source File | Area BuCaMH03\_B17 | #Feature | #Feature BuCaMH03\_B17 | Start | End | PTM | AScore | Found By |
| --- | --- | --- | --- | --- | --- | --- | --- | --- | --- | --- | --- | --- | --- | --- | --- | --- | --- | --- | --- |
| R.TAALC(+57.02)FGK.A | Y | 55.04 | 866.4320 | 8 | -0.2 | 434.2232 | 2 | 30.55 | 1 | 12370 | BuCaMH03.raw | 4.7538E5 | 1 | 1 | 123 | 130 | Carbamidomethylation | C5:Carbamidomethylation:1000.00 | PEAKS DB |
| total 1 peptides |  |  |  |  |  |  |  |  |  |  |  |  |  |  |  |  |  |  |  |
| --- | --- | --- | --- | --- | --- | --- | --- | --- | --- | --- | --- | --- | --- | --- | --- | --- | --- | --- | --- |

JAS05107.1

back to list

  

| Protein Coverage
| Supporting Peptides
|

Protein Coverage:

Supporting Peptides:

| Peptide | Uniq | -10lgP | Mass | Length | ppm | m/z | z | RT | Fraction | Scan | Source File | Area BuCaMH03\_B17 | #Feature | #Feature BuCaMH03\_B17 | Start | End | PTM | AScore | Found By |
| --- | --- | --- | --- | --- | --- | --- | --- | --- | --- | --- | --- | --- | --- | --- | --- | --- | --- | --- | --- |
| R.TAALC(+57.02)FGK.A | Y | 55.04 | 866.4320 | 8 | -0.2 | 434.2232 | 2 | 30.55 | 1 | 12370 | BuCaMH03.raw | 4.7538E5 | 1 | 1 | 123 | 130 | Carbamidomethylation | C5:Carbamidomethylation:1000.00 | PEAKS DB |
| total 1 peptides |  |  |  |  |  |  |  |  |  |  |  |  |  |  |  |  |  |  |  |
| --- | --- | --- | --- | --- | --- | --- | --- | --- | --- | --- | --- | --- | --- | --- | --- | --- | --- | --- | --- |

JAS05106.1

back to list

  

| Protein Coverage
| Supporting Peptides
|

Protein Coverage:

Supporting Peptides:

| Peptide | Uniq | -10lgP | Mass | Length | ppm | m/z | z | RT | Fraction | Scan | Source File | Area BuCaMH03\_B17 | #Feature | #Feature BuCaMH03\_B17 | Start | End | PTM | AScore | Found By |
| --- | --- | --- | --- | --- | --- | --- | --- | --- | --- | --- | --- | --- | --- | --- | --- | --- | --- | --- | --- |
| R.TAALC(+57.02)FGK.A | Y | 55.04 | 866.4320 | 8 | -0.2 | 434.2232 | 2 | 30.55 | 1 | 12370 | BuCaMH03.raw | 4.7538E5 | 1 | 1 | 123 | 130 | Carbamidomethylation | C5:Carbamidomethylation:1000.00 | PEAKS DB |
| total 1 peptides |  |  |  |  |  |  |  |  |  |  |  |  |  |  |  |  |  |  |  |
| --- | --- | --- | --- | --- | --- | --- | --- | --- | --- | --- | --- | --- | --- | --- | --- | --- | --- | --- | --- |

JAS05111.1

back to list

  

| Protein Coverage
| Supporting Peptides
|

Protein Coverage:

Supporting Peptides:

| Peptide | Uniq | -10lgP | Mass | Length | ppm | m/z | z | RT | Fraction | Scan | Source File | Area BuCaMH03\_B17 | #Feature | #Feature BuCaMH03\_B17 | Start | End | PTM | AScore | Found By |
| --- | --- | --- | --- | --- | --- | --- | --- | --- | --- | --- | --- | --- | --- | --- | --- | --- | --- | --- | --- |
| R.TAALC(+57.02)FGK.A | Y | 55.04 | 866.4320 | 8 | -0.2 | 434.2232 | 2 | 30.55 | 1 | 12370 | BuCaMH03.raw | 4.7538E5 | 1 | 1 | 123 | 130 | Carbamidomethylation | C5:Carbamidomethylation:1000.00 | PEAKS DB |
| total 1 peptides |  |  |  |  |  |  |  |  |  |  |  |  |  |  |  |  |  |  |  |
| --- | --- | --- | --- | --- | --- | --- | --- | --- | --- | --- | --- | --- | --- | --- | --- | --- | --- | --- | --- |

JAS05101.1

back to list

  

| Protein Coverage
| Supporting Peptides
|

Protein Coverage:

Supporting Peptides:

| Peptide | Uniq | -10lgP | Mass | Length | ppm | m/z | z | RT | Fraction | Scan | Source File | Area BuCaMH03\_B17 | #Feature | #Feature BuCaMH03\_B17 | Start | End | PTM | AScore | Found By |
| --- | --- | --- | --- | --- | --- | --- | --- | --- | --- | --- | --- | --- | --- | --- | --- | --- | --- | --- | --- |
| R.TAALC(+57.02)FGK.A | Y | 55.04 | 866.4320 | 8 | -0.2 | 434.2232 | 2 | 30.55 | 1 | 12370 | BuCaMH03.raw | 4.7538E5 | 1 | 1 | 123 | 130 | Carbamidomethylation | C5:Carbamidomethylation:1000.00 | PEAKS DB |
| total 1 peptides |  |  |  |  |  |  |  |  |  |  |  |  |  |  |  |  |  |  |  |
| --- | --- | --- | --- | --- | --- | --- | --- | --- | --- | --- | --- | --- | --- | --- | --- | --- | --- | --- | --- |

JAS05102.1

back to list

  

| Protein Coverage
| Supporting Peptides
|

Protein Coverage:

Supporting Peptides:

| Peptide | Uniq | -10lgP | Mass | Length | ppm | m/z | z | RT | Fraction | Scan | Source File | Area BuCaMH03\_B17 | #Feature | #Feature BuCaMH03\_B17 | Start | End | PTM | AScore | Found By |
| --- | --- | --- | --- | --- | --- | --- | --- | --- | --- | --- | --- | --- | --- | --- | --- | --- | --- | --- | --- |
| R.TAALC(+57.02)FGK.A | Y | 55.04 | 866.4320 | 8 | -0.2 | 434.2232 | 2 | 30.55 | 1 | 12370 | BuCaMH03.raw | 4.7538E5 | 1 | 1 | 123 | 130 | Carbamidomethylation | C5:Carbamidomethylation:1000.00 | PEAKS DB |
| total 1 peptides |  |  |  |  |  |  |  |  |  |  |  |  |  |  |  |  |  |  |  |
| --- | --- | --- | --- | --- | --- | --- | --- | --- | --- | --- | --- | --- | --- | --- | --- | --- | --- | --- | --- |

JAS05104.1

back to list

  

| Protein Coverage
| Supporting Peptides
|

Protein Coverage:

Supporting Peptides:

| Peptide | Uniq | -10lgP | Mass | Length | ppm | m/z | z | RT | Fraction | Scan | Source File | Area BuCaMH03\_B17 | #Feature | #Feature BuCaMH03\_B17 | Start | End | PTM | AScore | Found By |
| --- | --- | --- | --- | --- | --- | --- | --- | --- | --- | --- | --- | --- | --- | --- | --- | --- | --- | --- | --- |
| R.TAALC(+57.02)FGK.A | Y | 55.04 | 866.4320 | 8 | -0.2 | 434.2232 | 2 | 30.55 | 1 | 12370 | BuCaMH03.raw | 4.7538E5 | 1 | 1 | 123 | 130 | Carbamidomethylation | C5:Carbamidomethylation:1000.00 | PEAKS DB |
| total 1 peptides |  |  |  |  |  |  |  |  |  |  |  |  |  |  |  |  |  |  |  |
| --- | --- | --- | --- | --- | --- | --- | --- | --- | --- | --- | --- | --- | --- | --- | --- | --- | --- | --- | --- |

JAS05105.1

back to list

  

| Protein Coverage
| Supporting Peptides
|

Protein Coverage:

Supporting Peptides:

| Peptide | Uniq | -10lgP | Mass | Length | ppm | m/z | z | RT | Fraction | Scan | Source File | Area BuCaMH03\_B17 | #Feature | #Feature BuCaMH03\_B17 | Start | End | PTM | AScore | Found By |
| --- | --- | --- | --- | --- | --- | --- | --- | --- | --- | --- | --- | --- | --- | --- | --- | --- | --- | --- | --- |
| R.TAALC(+57.02)FGK.A | Y | 55.04 | 866.4320 | 8 | -0.2 | 434.2232 | 2 | 30.55 | 1 | 12370 | BuCaMH03.raw | 4.7538E5 | 1 | 1 | 123 | 130 | Carbamidomethylation | C5:Carbamidomethylation:1000.00 | PEAKS DB |
| total 1 peptides |  |  |  |  |  |  |  |  |  |  |  |  |  |  |  |  |  |  |  |
| --- | --- | --- | --- | --- | --- | --- | --- | --- | --- | --- | --- | --- | --- | --- | --- | --- | --- | --- | --- |

JAS05110.1

back to list

  

| Protein Coverage
| Supporting Peptides
|

Protein Coverage:

Supporting Peptides:

| Peptide | Uniq | -10lgP | Mass | Length | ppm | m/z | z | RT | Fraction | Scan | Source File | Area BuCaMH03\_B17 | #Feature | #Feature BuCaMH03\_B17 | Start | End | PTM | AScore | Found By |
| --- | --- | --- | --- | --- | --- | --- | --- | --- | --- | --- | --- | --- | --- | --- | --- | --- | --- | --- | --- |
| R.TAALC(+57.02)FGK.A | Y | 55.04 | 866.4320 | 8 | -0.2 | 434.2232 | 2 | 30.55 | 1 | 12370 | BuCaMH03.raw | 4.7538E5 | 1 | 1 | 123 | 130 | Carbamidomethylation | C5:Carbamidomethylation:1000.00 | PEAKS DB |
| total 1 peptides |  |  |  |  |  |  |  |  |  |  |  |  |  |  |  |  |  |  |  |
| --- | --- | --- | --- | --- | --- | --- | --- | --- | --- | --- | --- | --- | --- | --- | --- | --- | --- | --- | --- |

ACR78492.1

back to list

  

| Protein Coverage
| Supporting Peptides
|

Protein Coverage:

Supporting Peptides:

| Peptide | Uniq | -10lgP | Mass | Length | ppm | m/z | z | RT | Fraction | Scan | Source File | Area BuCaMH03\_B17 | #Feature | #Feature BuCaMH03\_B17 | Start | End | PTM | AScore | Found By |
| --- | --- | --- | --- | --- | --- | --- | --- | --- | --- | --- | --- | --- | --- | --- | --- | --- | --- | --- | --- |
| K.VIELGC(+57.02)AATC(+57.02)PPAEPKKDI.T | Y | 54.81 | 2068.0332 | 19 | 5.8 | 518.0186 | 4 | 24.32 | 1 | 8213 | BuCaMH03.raw | 1.474E7 | 1 | 1 | 58 | 76 | Carbamidomethylation | C6:Carbamidomethylation:1000.00;C10:Carbamidomethylation:1000.00 | PEAKS DB |
| total 1 peptides |  |  |  |  |  |  |  |  |  |  |  |  |  |  |  |  |  |  |  |
| --- | --- | --- | --- | --- | --- | --- | --- | --- | --- | --- | --- | --- | --- | --- | --- | --- | --- | --- | --- |

ACR78484.1

back to list

  

| Protein Coverage
| Supporting Peptides
|

Protein Coverage:

Supporting Peptides:

| Peptide | Uniq | -10lgP | Mass | Length | ppm | m/z | z | RT | Fraction | Scan | Source File | Area BuCaMH03\_B17 | #Feature | #Feature BuCaMH03\_B17 | Start | End | PTM | AScore | Found By |
| --- | --- | --- | --- | --- | --- | --- | --- | --- | --- | --- | --- | --- | --- | --- | --- | --- | --- | --- | --- |
| K.VIELGC(+57.02)AATC(+57.02)PPAEPKKDI.T | Y | 54.81 | 2068.0332 | 19 | 5.8 | 518.0186 | 4 | 24.32 | 1 | 8213 | BuCaMH03.raw | 1.474E7 | 1 | 1 | 58 | 76 | Carbamidomethylation | C6:Carbamidomethylation:1000.00;C10:Carbamidomethylation:1000.00 | PEAKS DB |
| total 1 peptides |  |  |  |  |  |  |  |  |  |  |  |  |  |  |  |  |  |  |  |
| --- | --- | --- | --- | --- | --- | --- | --- | --- | --- | --- | --- | --- | --- | --- | --- | --- | --- | --- | --- |

F8J2F2.1

back to list

  

| Protein Coverage
| Supporting Peptides
|

Protein Coverage:

Supporting Peptides:

| Peptide | Uniq | -10lgP | Mass | Length | ppm | m/z | z | RT | Fraction | Scan | Source File | Area BuCaMH03\_B17 | #Feature | #Feature BuCaMH03\_B17 | Start | End | PTM | AScore | Found By |
| --- | --- | --- | --- | --- | --- | --- | --- | --- | --- | --- | --- | --- | --- | --- | --- | --- | --- | --- | --- |
| K.VIELGC(+57.02)AATC(+57.02)PPAEPKKDI.T | Y | 54.81 | 2068.0332 | 19 | 5.8 | 518.0186 | 4 | 24.32 | 1 | 8213 | BuCaMH03.raw | 1.474E7 | 1 | 1 | 58 | 76 | Carbamidomethylation | C6:Carbamidomethylation:1000.00;C10:Carbamidomethylation:1000.00 | PEAKS DB |
| total 1 peptides |  |  |  |  |  |  |  |  |  |  |  |  |  |  |  |  |  |  |  |
| --- | --- | --- | --- | --- | --- | --- | --- | --- | --- | --- | --- | --- | --- | --- | --- | --- | --- | --- | --- |

ACR78483.1

back to list

  

| Protein Coverage
| Supporting Peptides
|

Protein Coverage:

Supporting Peptides:

| Peptide | Uniq | -10lgP | Mass | Length | ppm | m/z | z | RT | Fraction | Scan | Source File | Area BuCaMH03\_B17 | #Feature | #Feature BuCaMH03\_B17 | Start | End | PTM | AScore | Found By |
| --- | --- | --- | --- | --- | --- | --- | --- | --- | --- | --- | --- | --- | --- | --- | --- | --- | --- | --- | --- |
| K.VIELGC(+57.02)AATC(+57.02)PPAEPKKDI.T | Y | 54.81 | 2068.0332 | 19 | 5.8 | 518.0186 | 4 | 24.32 | 1 | 8213 | BuCaMH03.raw | 1.474E7 | 1 | 1 | 58 | 76 | Carbamidomethylation | C6:Carbamidomethylation:1000.00;C10:Carbamidomethylation:1000.00 | PEAKS DB |
| total 1 peptides |  |  |  |  |  |  |  |  |  |  |  |  |  |  |  |  |  |  |  |
| --- | --- | --- | --- | --- | --- | --- | --- | --- | --- | --- | --- | --- | --- | --- | --- | --- | --- | --- | --- |

ACR78488.1

back to list

  

| Protein Coverage
| Supporting Peptides
|

Protein Coverage:

Supporting Peptides:

| Peptide | Uniq | -10lgP | Mass | Length | ppm | m/z | z | RT | Fraction | Scan | Source File | Area BuCaMH03\_B17 | #Feature | #Feature BuCaMH03\_B17 | Start | End | PTM | AScore | Found By |
| --- | --- | --- | --- | --- | --- | --- | --- | --- | --- | --- | --- | --- | --- | --- | --- | --- | --- | --- | --- |
| K.VIELGC(+57.02)AATC(+57.02)PPAEPKKDI.T | Y | 54.81 | 2068.0332 | 19 | 5.8 | 518.0186 | 4 | 24.32 | 1 | 8213 | BuCaMH03.raw | 1.474E7 | 1 | 1 | 58 | 76 | Carbamidomethylation | C6:Carbamidomethylation:1000.00;C10:Carbamidomethylation:1000.00 | PEAKS DB |
| total 1 peptides |  |  |  |  |  |  |  |  |  |  |  |  |  |  |  |  |  |  |  |
| --- | --- | --- | --- | --- | --- | --- | --- | --- | --- | --- | --- | --- | --- | --- | --- | --- | --- | --- | --- |

JAA74738.1

back to list

  

| Protein Coverage
| Supporting Peptides
|

Protein Coverage:

Supporting Peptides:

| Peptide | Uniq | -10lgP | Mass | Length | ppm | m/z | z | RT | Fraction | Scan | Source File | Area BuCaMH03\_B17 | #Feature | #Feature BuCaMH03\_B17 | Start | End | PTM | AScore | Found By |
| --- | --- | --- | --- | --- | --- | --- | --- | --- | --- | --- | --- | --- | --- | --- | --- | --- | --- | --- | --- |
| K.VIELGC(+57.02)AATC(+57.02)PPAEPKKDI.T | Y | 54.81 | 2068.0332 | 19 | 5.8 | 518.0186 | 4 | 24.32 | 1 | 8213 | BuCaMH03.raw | 1.474E7 | 1 | 1 | 58 | 76 | Carbamidomethylation | C6:Carbamidomethylation:1000.00;C10:Carbamidomethylation:1000.00 | PEAKS DB |
| total 1 peptides |  |  |  |  |  |  |  |  |  |  |  |  |  |  |  |  |  |  |  |
| --- | --- | --- | --- | --- | --- | --- | --- | --- | --- | --- | --- | --- | --- | --- | --- | --- | --- | --- | --- |

ACR78486.1

back to list

  

| Protein Coverage
| Supporting Peptides
|

Protein Coverage:

Supporting Peptides:

| Peptide | Uniq | -10lgP | Mass | Length | ppm | m/z | z | RT | Fraction | Scan | Source File | Area BuCaMH03\_B17 | #Feature | #Feature BuCaMH03\_B17 | Start | End | PTM | AScore | Found By |
| --- | --- | --- | --- | --- | --- | --- | --- | --- | --- | --- | --- | --- | --- | --- | --- | --- | --- | --- | --- |
| K.VIELGC(+57.02)AATC(+57.02)PPAEPKKDI.T | Y | 54.81 | 2068.0332 | 19 | 5.8 | 518.0186 | 4 | 24.32 | 1 | 8213 | BuCaMH03.raw | 1.474E7 | 1 | 1 | 58 | 76 | Carbamidomethylation | C6:Carbamidomethylation:1000.00;C10:Carbamidomethylation:1000.00 | PEAKS DB |
| total 1 peptides |  |  |  |  |  |  |  |  |  |  |  |  |  |  |  |  |  |  |  |
| --- | --- | --- | --- | --- | --- | --- | --- | --- | --- | --- | --- | --- | --- | --- | --- | --- | --- | --- | --- |

F8J2E6.1

back to list

  

| Protein Coverage
| Supporting Peptides
|

Protein Coverage:

Supporting Peptides:

| Peptide | Uniq | -10lgP | Mass | Length | ppm | m/z | z | RT | Fraction | Scan | Source File | Area BuCaMH03\_B17 | #Feature | #Feature BuCaMH03\_B17 | Start | End | PTM | AScore | Found By |
| --- | --- | --- | --- | --- | --- | --- | --- | --- | --- | --- | --- | --- | --- | --- | --- | --- | --- | --- | --- |
| K.VIELGC(+57.02)AATC(+57.02)PPAEPKKDI.T | Y | 54.81 | 2068.0332 | 19 | 5.8 | 518.0186 | 4 | 24.32 | 1 | 8213 | BuCaMH03.raw | 1.474E7 | 1 | 1 | 58 | 76 | Carbamidomethylation | C6:Carbamidomethylation:1000.00;C10:Carbamidomethylation:1000.00 | PEAKS DB |
| total 1 peptides |  |  |  |  |  |  |  |  |  |  |  |  |  |  |  |  |  |  |  |
| --- | --- | --- | --- | --- | --- | --- | --- | --- | --- | --- | --- | --- | --- | --- | --- | --- | --- | --- | --- |

ACR78491.1

back to list

  

| Protein Coverage
| Supporting Peptides
|

Protein Coverage:

Supporting Peptides:

| Peptide | Uniq | -10lgP | Mass | Length | ppm | m/z | z | RT | Fraction | Scan | Source File | Area BuCaMH03\_B17 | #Feature | #Feature BuCaMH03\_B17 | Start | End | PTM | AScore | Found By |
| --- | --- | --- | --- | --- | --- | --- | --- | --- | --- | --- | --- | --- | --- | --- | --- | --- | --- | --- | --- |
| K.VIELGC(+57.02)AATC(+57.02)PPAEPKKDI.T | Y | 54.81 | 2068.0332 | 19 | 5.8 | 518.0186 | 4 | 24.32 | 1 | 8213 | BuCaMH03.raw | 1.474E7 | 1 | 1 | 58 | 76 | Carbamidomethylation | C6:Carbamidomethylation:1000.00;C10:Carbamidomethylation:1000.00 | PEAKS DB |
| total 1 peptides |  |  |  |  |  |  |  |  |  |  |  |  |  |  |  |  |  |  |  |
| --- | --- | --- | --- | --- | --- | --- | --- | --- | --- | --- | --- | --- | --- | --- | --- | --- | --- | --- | --- |

JAA74652.1

back to list

  

| Protein Coverage
| Supporting Peptides
|

Protein Coverage:

Supporting Peptides:

| Peptide | Uniq | -10lgP | Mass | Length | ppm | m/z | z | RT | Fraction | Scan | Source File | Area BuCaMH03\_B17 | #Feature | #Feature BuCaMH03\_B17 | Start | End | PTM | AScore | Found By |
| --- | --- | --- | --- | --- | --- | --- | --- | --- | --- | --- | --- | --- | --- | --- | --- | --- | --- | --- | --- |
| K.VIELGC(+57.02)AATC(+57.02)PPAEPKKDI.K | Y | 54.81 | 2068.0332 | 19 | 5.8 | 518.0186 | 4 | 24.32 | 1 | 8213 | BuCaMH03.raw | 1.474E7 | 1 | 1 | 58 | 76 | Carbamidomethylation | C6:Carbamidomethylation:1000.00;C10:Carbamidomethylation:1000.00 | PEAKS DB |
| total 1 peptides |  |  |  |  |  |  |  |  |  |  |  |  |  |  |  |  |  |  |  |
| --- | --- | --- | --- | --- | --- | --- | --- | --- | --- | --- | --- | --- | --- | --- | --- | --- | --- | --- | --- |

JAA74651.1

back to list

  

| Protein Coverage
| Supporting Peptides
|

Protein Coverage:

Supporting Peptides:

| Peptide | Uniq | -10lgP | Mass | Length | ppm | m/z | z | RT | Fraction | Scan | Source File | Area BuCaMH03\_B17 | #Feature | #Feature BuCaMH03\_B17 | Start | End | PTM | AScore | Found By |
| --- | --- | --- | --- | --- | --- | --- | --- | --- | --- | --- | --- | --- | --- | --- | --- | --- | --- | --- | --- |
| K.VIELGC(+57.02)AATC(+57.02)PPAEPKKDI.K | Y | 54.81 | 2068.0332 | 19 | 5.8 | 518.0186 | 4 | 24.32 | 1 | 8213 | BuCaMH03.raw | 1.474E7 | 1 | 1 | 58 | 76 | Carbamidomethylation | C6:Carbamidomethylation:1000.00;C10:Carbamidomethylation:1000.00 | PEAKS DB |
| total 1 peptides |  |  |  |  |  |  |  |  |  |  |  |  |  |  |  |  |  |  |  |
| --- | --- | --- | --- | --- | --- | --- | --- | --- | --- | --- | --- | --- | --- | --- | --- | --- | --- | --- | --- |

JAA74655.1

back to list

  

| Protein Coverage
| Supporting Peptides
|

Protein Coverage:

Supporting Peptides:

| Peptide | Uniq | -10lgP | Mass | Length | ppm | m/z | z | RT | Fraction | Scan | Source File | Area BuCaMH03\_B17 | #Feature | #Feature BuCaMH03\_B17 | Start | End | PTM | AScore | Found By |
| --- | --- | --- | --- | --- | --- | --- | --- | --- | --- | --- | --- | --- | --- | --- | --- | --- | --- | --- | --- |
| K.VIELGC(+57.02)AATC(+57.02)PPAEPKKDI.K | Y | 54.81 | 2068.0332 | 19 | 5.8 | 518.0186 | 4 | 24.32 | 1 | 8213 | BuCaMH03.raw | 1.474E7 | 1 | 1 | 58 | 76 | Carbamidomethylation | C6:Carbamidomethylation:1000.00;C10:Carbamidomethylation:1000.00 | PEAKS DB |
| total 1 peptides |  |  |  |  |  |  |  |  |  |  |  |  |  |  |  |  |  |  |  |
| --- | --- | --- | --- | --- | --- | --- | --- | --- | --- | --- | --- | --- | --- | --- | --- | --- | --- | --- | --- |

ABK63538.1

back to list

  

| Protein Coverage
| Supporting Peptides
|

Protein Coverage:

Supporting Peptides:

| Peptide | Uniq | -10lgP | Mass | Length | ppm | m/z | z | RT | Fraction | Scan | Source File | Area BuCaMH03\_B17 | #Feature | #Feature BuCaMH03\_B17 | Start | End | PTM | AScore | Found By |
| --- | --- | --- | --- | --- | --- | --- | --- | --- | --- | --- | --- | --- | --- | --- | --- | --- | --- | --- | --- |
| R.VIELGC(+57.02)AATC(+57.02)PPAEPKKDI.S | Y | 54.81 | 2068.0332 | 19 | 5.8 | 518.0186 | 4 | 24.32 | 1 | 8213 | BuCaMH03.raw | 1.474E7 | 1 | 1 | 58 | 76 | Carbamidomethylation | C6:Carbamidomethylation:1000.00;C10:Carbamidomethylation:1000.00 | PEAKS DB |
| total 1 peptides |  |  |  |  |  |  |  |  |  |  |  |  |  |  |  |  |  |  |  |
| --- | --- | --- | --- | --- | --- | --- | --- | --- | --- | --- | --- | --- | --- | --- | --- | --- | --- | --- | --- |

A8HDK4.1

back to list

  

| Protein Coverage
| Supporting Peptides
|

Protein Coverage:

Supporting Peptides:

| Peptide | Uniq | -10lgP | Mass | Length | ppm | m/z | z | RT | Fraction | Scan | Source File | Area BuCaMH03\_B17 | #Feature | #Feature BuCaMH03\_B17 | Start | End | PTM | AScore | Found By |
| --- | --- | --- | --- | --- | --- | --- | --- | --- | --- | --- | --- | --- | --- | --- | --- | --- | --- | --- | --- |
| R.VIELGC(+57.02)AATC(+57.02)PPAEPKKDI.S | Y | 54.81 | 2068.0332 | 19 | 5.8 | 518.0186 | 4 | 24.32 | 1 | 8213 | BuCaMH03.raw | 1.474E7 | 1 | 1 | 58 | 76 | Carbamidomethylation | C6:Carbamidomethylation:1000.00;C10:Carbamidomethylation:1000.00 | PEAKS DB |
| total 1 peptides |  |  |  |  |  |  |  |  |  |  |  |  |  |  |  |  |  |  |  |
| --- | --- | --- | --- | --- | --- | --- | --- | --- | --- | --- | --- | --- | --- | --- | --- | --- | --- | --- | --- |

ABX58163.1

back to list

  

| Protein Coverage
| Supporting Peptides
|

Protein Coverage:

Supporting Peptides:

| Peptide | Uniq | -10lgP | Mass | Length | ppm | m/z | z | RT | Fraction | Scan | Source File | Area BuCaMH03\_B17 | #Feature | #Feature BuCaMH03\_B17 | Start | End | PTM | AScore | Found By |
| --- | --- | --- | --- | --- | --- | --- | --- | --- | --- | --- | --- | --- | --- | --- | --- | --- | --- | --- | --- |
| K.VIELGC(+57.02)AATC(+57.02)PPAEPKKDI.T | Y | 54.81 | 2068.0332 | 19 | 5.8 | 518.0186 | 4 | 24.32 | 1 | 8213 | BuCaMH03.raw | 1.474E7 | 1 | 1 | 58 | 76 | Carbamidomethylation | C6:Carbamidomethylation:1000.00;C10:Carbamidomethylation:1000.00 | PEAKS DB |
| total 1 peptides |  |  |  |  |  |  |  |  |  |  |  |  |  |  |  |  |  |  |  |
| --- | --- | --- | --- | --- | --- | --- | --- | --- | --- | --- | --- | --- | --- | --- | --- | --- | --- | --- | --- |

ABX58162.1

back to list

  

| Protein Coverage
| Supporting Peptides
|

Protein Coverage:

Supporting Peptides:

| Peptide | Uniq | -10lgP | Mass | Length | ppm | m/z | z | RT | Fraction | Scan | Source File | Area BuCaMH03\_B17 | #Feature | #Feature BuCaMH03\_B17 | Start | End | PTM | AScore | Found By |
| --- | --- | --- | --- | --- | --- | --- | --- | --- | --- | --- | --- | --- | --- | --- | --- | --- | --- | --- | --- |
| K.VIELGC(+57.02)AATC(+57.02)PPAEPKKDI.T | Y | 54.81 | 2068.0332 | 19 | 5.8 | 518.0186 | 4 | 24.32 | 1 | 8213 | BuCaMH03.raw | 1.474E7 | 1 | 1 | 58 | 76 | Carbamidomethylation | C6:Carbamidomethylation:1000.00;C10:Carbamidomethylation:1000.00 | PEAKS DB |
| total 1 peptides |  |  |  |  |  |  |  |  |  |  |  |  |  |  |  |  |  |  |  |
| --- | --- | --- | --- | --- | --- | --- | --- | --- | --- | --- | --- | --- | --- | --- | --- | --- | --- | --- | --- |

ABX58156.1

back to list

  

| Protein Coverage
| Supporting Peptides
|

Protein Coverage:

Supporting Peptides:

| Peptide | Uniq | -10lgP | Mass | Length | ppm | m/z | z | RT | Fraction | Scan | Source File | Area BuCaMH03\_B17 | #Feature | #Feature BuCaMH03\_B17 | Start | End | PTM | AScore | Found By |
| --- | --- | --- | --- | --- | --- | --- | --- | --- | --- | --- | --- | --- | --- | --- | --- | --- | --- | --- | --- |
| K.VIELGC(+57.02)AATC(+57.02)PPAEPKKDI.T | Y | 54.81 | 2068.0332 | 19 | 5.8 | 518.0186 | 4 | 24.32 | 1 | 8213 | BuCaMH03.raw | 1.474E7 | 1 | 1 | 58 | 76 | Carbamidomethylation | C6:Carbamidomethylation:1000.00;C10:Carbamidomethylation:1000.00 | PEAKS DB |
| total 1 peptides |  |  |  |  |  |  |  |  |  |  |  |  |  |  |  |  |  |  |  |
| --- | --- | --- | --- | --- | --- | --- | --- | --- | --- | --- | --- | --- | --- | --- | --- | --- | --- | --- | --- |

ABX58159.1

back to list

  

| Protein Coverage
| Supporting Peptides
|

Protein Coverage:

Supporting Peptides:

| Peptide | Uniq | -10lgP | Mass | Length | ppm | m/z | z | RT | Fraction | Scan | Source File | Area BuCaMH03\_B17 | #Feature | #Feature BuCaMH03\_B17 | Start | End | PTM | AScore | Found By |
| --- | --- | --- | --- | --- | --- | --- | --- | --- | --- | --- | --- | --- | --- | --- | --- | --- | --- | --- | --- |
| K.VIELGC(+57.02)AATC(+57.02)PPAEPKKDI.T | Y | 54.81 | 2068.0332 | 19 | 5.8 | 518.0186 | 4 | 24.32 | 1 | 8213 | BuCaMH03.raw | 1.474E7 | 1 | 1 | 58 | 76 | Carbamidomethylation | C6:Carbamidomethylation:1000.00;C10:Carbamidomethylation:1000.00 | PEAKS DB |
| total 1 peptides |  |  |  |  |  |  |  |  |  |  |  |  |  |  |  |  |  |  |  |
| --- | --- | --- | --- | --- | --- | --- | --- | --- | --- | --- | --- | --- | --- | --- | --- | --- | --- | --- | --- |

ABX58160.1

back to list

  

| Protein Coverage
| Supporting Peptides
|

Protein Coverage:

Supporting Peptides:

| Peptide | Uniq | -10lgP | Mass | Length | ppm | m/z | z | RT | Fraction | Scan | Source File | Area BuCaMH03\_B17 | #Feature | #Feature BuCaMH03\_B17 | Start | End | PTM | AScore | Found By |
| --- | --- | --- | --- | --- | --- | --- | --- | --- | --- | --- | --- | --- | --- | --- | --- | --- | --- | --- | --- |
| K.VIELGC(+57.02)AATC(+57.02)PPAEPKKDI.T | Y | 54.81 | 2068.0332 | 19 | 5.8 | 518.0186 | 4 | 24.32 | 1 | 8213 | BuCaMH03.raw | 1.474E7 | 1 | 1 | 58 | 76 | Carbamidomethylation | C6:Carbamidomethylation:1000.00;C10:Carbamidomethylation:1000.00 | PEAKS DB |
| total 1 peptides |  |  |  |  |  |  |  |  |  |  |  |  |  |  |  |  |  |  |  |
| --- | --- | --- | --- | --- | --- | --- | --- | --- | --- | --- | --- | --- | --- | --- | --- | --- | --- | --- | --- |

ABX58161.1

back to list

  

| Protein Coverage
| Supporting Peptides
|

Protein Coverage:

Supporting Peptides:

| Peptide | Uniq | -10lgP | Mass | Length | ppm | m/z | z | RT | Fraction | Scan | Source File | Area BuCaMH03\_B17 | #Feature | #Feature BuCaMH03\_B17 | Start | End | PTM | AScore | Found By |
| --- | --- | --- | --- | --- | --- | --- | --- | --- | --- | --- | --- | --- | --- | --- | --- | --- | --- | --- | --- |
| K.VIELGC(+57.02)AATC(+57.02)PPAEPKKDI.T | Y | 54.81 | 2068.0332 | 19 | 5.8 | 518.0186 | 4 | 24.32 | 1 | 8213 | BuCaMH03.raw | 1.474E7 | 1 | 1 | 58 | 76 | Carbamidomethylation | C6:Carbamidomethylation:1000.00;C10:Carbamidomethylation:1000.00 | PEAKS DB |
| total 1 peptides |  |  |  |  |  |  |  |  |  |  |  |  |  |  |  |  |  |  |  |
| --- | --- | --- | --- | --- | --- | --- | --- | --- | --- | --- | --- | --- | --- | --- | --- | --- | --- | --- | --- |

ABX58158.1

back to list

  

| Protein Coverage
| Supporting Peptides
|

Protein Coverage:

Supporting Peptides:

| Peptide | Uniq | -10lgP | Mass | Length | ppm | m/z | z | RT | Fraction | Scan | Source File | Area BuCaMH03\_B17 | #Feature | #Feature BuCaMH03\_B17 | Start | End | PTM | AScore | Found By |
| --- | --- | --- | --- | --- | --- | --- | --- | --- | --- | --- | --- | --- | --- | --- | --- | --- | --- | --- | --- |
| K.VIELGC(+57.02)AATC(+57.02)PPAEPKKDI.T | Y | 54.81 | 2068.0332 | 19 | 5.8 | 518.0186 | 4 | 24.32 | 1 | 8213 | BuCaMH03.raw | 1.474E7 | 1 | 1 | 58 | 76 | Carbamidomethylation | C6:Carbamidomethylation:1000.00;C10:Carbamidomethylation:1000.00 | PEAKS DB |
| total 1 peptides |  |  |  |  |  |  |  |  |  |  |  |  |  |  |  |  |  |  |  |
| --- | --- | --- | --- | --- | --- | --- | --- | --- | --- | --- | --- | --- | --- | --- | --- | --- | --- | --- | --- |

P01397.2

back to list

  

| Protein Coverage
| Supporting Peptides
|

Protein Coverage:

Supporting Peptides:

| Peptide | Uniq | -10lgP | Mass | Length | ppm | m/z | z | RT | Fraction | Scan | Source File | Area BuCaMH03\_B17 | #Feature | #Feature BuCaMH03\_B17 | Start | End | PTM | AScore | Found By |
| --- | --- | --- | --- | --- | --- | --- | --- | --- | --- | --- | --- | --- | --- | --- | --- | --- | --- | --- | --- |
| K.IVELGC(+57.02)AATC(+57.02)PK.V | Y | 54.53 | 1317.6421 | 12 | 0.9 | 659.8289 | 2 | 35.23 | 1 | 15670 | BuCaMH03.raw | 2.0197E6 | 1 | 1 | 37 | 48 | Carbamidomethylation | C6:Carbamidomethylation:1000.00;C10:Carbamidomethylation:1000.00 | PEAKS DB |
| total 1 peptides |  |  |  |  |  |  |  |  |  |  |  |  |  |  |  |  |  |  |  |
| --- | --- | --- | --- | --- | --- | --- | --- | --- | --- | --- | --- | --- | --- | --- | --- | --- | --- | --- | --- |

P25667.1

back to list

  

| Protein Coverage
| Supporting Peptides
|

Protein Coverage:

Supporting Peptides:

| Peptide | Uniq | -10lgP | Mass | Length | ppm | m/z | z | RT | Fraction | Scan | Source File | Area BuCaMH03\_B17 | #Feature | #Feature BuCaMH03\_B17 | Start | End | PTM | AScore | Found By |
| --- | --- | --- | --- | --- | --- | --- | --- | --- | --- | --- | --- | --- | --- | --- | --- | --- | --- | --- | --- |
| K.IVELGC(+57.02)AATC(+57.02)PK.V | Y | 54.53 | 1317.6421 | 12 | 0.9 | 659.8289 | 2 | 35.23 | 1 | 15670 | BuCaMH03.raw | 2.0197E6 | 1 | 1 | 37 | 48 | Carbamidomethylation | C6:Carbamidomethylation:1000.00;C10:Carbamidomethylation:1000.00 | PEAKS DB |
| total 1 peptides |  |  |  |  |  |  |  |  |  |  |  |  |  |  |  |  |  |  |  |
| --- | --- | --- | --- | --- | --- | --- | --- | --- | --- | --- | --- | --- | --- | --- | --- | --- | --- | --- | --- |

Peptide List

  
  

---

Prepared with PEAKS ™ (bioinfor.com)
